# European Population Substructure: Clustering of Northern and Southern Populations 

**DOI:** 10.1371/journal.pgen.0020143

**Published:** 2006-09-15

**Authors:** Michael F Seldin, Russell Shigeta, Pablo Villoslada, Carlo Selmi, Jaakko Tuomilehto, Gabriel Silva, John W Belmont, Lars Klareskog, Peter K Gregersen

**Affiliations:** 1 Rowe Program in Human Genetics, Departments of Biological Chemistry and Medicine, University of California Davis, Davis, California, United States of America; 2 Center for Applied Medical Research, University of Navarra, Pamplona, Spain; 3 Department of Medicine, Surgery, and Dentistry, San Paolo School of Medicine, University of Milan, Milan, Italy; 4 Department of Medicine, University of California Davis, Davis, California, United States of America; 5 Department of Epidemiology and Health Promotion, National Public Health Institute, Helsinki, Finland; 6 South Ostrobothnia Central Hospital, Seinajoki, Finland; 7 Obras Sociales del Hermano Pedro, Antigua, Guatemala; 8 Department of Molecular and Human Genetics, Baylor College of Medicine, Houston, Texas, United States of America; 9 Karolinska University Hospital, Stockholm, Sweden; 10 The Robert S. Boas Center for Genomics and Human Genetics, Feinstein Institute for Medical Research, North Shore Long Island Jewish Health System, Manhasset, New York, United States of America; University of Chicago, United States of America

## Abstract

Using a genome-wide single nucleotide polymorphism (SNP) panel, we observed population structure in a diverse group of Europeans and European Americans. Under a variety of conditions and tests, there is a consistent and reproducible distinction between “northern” and “southern” European population groups: most individual participants with southern European ancestry (Italian, Spanish, Portuguese, and Greek) have >85% membership in the “southern” population; and most northern, western, eastern, and central Europeans have >90% in the “northern” population group. Ashkenazi Jewish as well as Sephardic Jewish origin also showed >85% membership in the “southern” population, consistent with a later Mediterranean origin of these ethnic groups. Based on this work, we have developed a core set of informative SNP markers that can control for this partition in European population structure in a variety of clinical and genetic studies.

## Synopsis

Two unrelated persons in the human population have hundreds of thousands of base pair differences between them in DNA sequence. Previous studies have shown that a small proportion of these sequence differences correlate with a person's continental ancestry: broadly, Asia, Africa Oceana, America, or continental Europe. In the current study, DNA differences within a particular continental group, Europe, were examined. Overall, the analysis of sequence variation allowed the authors to distinguish individuals with northern European ancestry (Swedish, English, Irish, German, and Ukrainian) from individuals with southern European ancestry (Italian, Spanish, Portuguese, and Greek). Interestingly, Ashkenazi Jewish individuals tend to group together with individuals from southern European countries. This study is important because it provides a method of taking into account these differences when searching for genetic variations that are associated with particular human traits, such as disease susceptibility, response to drug treatment, or side effects from therapy. Specifically, these methods may allow scientists to uncover disease-associated genetic variations that might be hidden unless differences related to European ancestry are considered.

## Introduction

The recent development of methodologies for defining population structure has provided the ability to identify the major ethnic contributions in individual participants in diverse populations [1–7]. These statistical approaches utilize non-hierarchical clustering algorithms in which Markov chain Monte Carlo methods are used to infer ancestry, based solely on genotyping information. Furthermore, related algorithms provide methods for controlling for population stratification in genetic studies [8–10]. These methods are important in assessing the results of case-control and other non–family-based association tests. In addition, defining population structure is potentially useful both in clinical outcome studies and in the examination of pharmacologic response and toxicity.

Previous studies of human population structure have primarily considered different continental populations or admixed populations between two or more different continental populations [3–7]. However, some of these studies have also suggested that sub-continental differences in population structure can be discerned [4,11]. The examination of population differences within Europe using mitochondrial [12–15] or Y chromosome [16–18] haplogroups has been particularly useful in tracing part of the routes of migration and populating of Europe, but these haplogroups do not provide strong inferences on population genetic structure. Autosomal studies using small numbers of classical genetic markers (nuclear protein polymorphisms) have suggested broad genetic gradients across Europe, leading to the proposal of demic diffusion models [19–22]. These include a principal component analysis of small numbers of classic genetic markers that suggested three clines accounting for a proportion of the genetic variation in the continent [22]. Together with subsequent studies including a recent analysis of microsatellite data, these studies have provided additional support for a large Neolithic component of the European genome and a strong element of demic diffusion originating from the Near East [23,24]. However, it must be noted that the issue of Paleolithic versus Neolithic origin of Europeans is still controversial, and other recent studies examining ancient mitochondrial DNA have suggested virtually no Neolithic contribution to European populations [25].

In this report we expand on the autosomal DNA observations by examining a large number of single nucleotide polymorphisms (SNP) genotypes, using statistical methods to directly examine population genetic structure. The results show clear evidence of large differences in population structure between southern and northern European populations. In addition, we present data that extend recent studies suggesting that population structure can create false-positive association tests in European Americans [26]. Moreover, the current results suggest practical applications of defining this population's genetic substructure in genetic studies.

## Results

### Allele Frequency Differences and F_st_ between Different European Populations Are Small

A total of 1,094 participants were genotyped with more than 5,700 SNPs distributed over the entire genome. After excluding participants with > 10% estimated non-European ancestry (see Methods), 928 participants were selected for further analysis. The allele frequency differences and F_st_ values were determined for the following subgroups with European heritage: 162 western European Americans (see Methods for description of populations), 41 central European Americans, 27 eastern European Americans, 86 Italian participants, 74 Spanish participants, and 90 Swedish participants (Table 1). Although the F_st_ values are small (mean intra-European group F_st_ = 0.0029), the distance between the Italian and Spanish participants (F_st_ = 0.0021) was smaller and showed no overlap with the 95% confidence intervals between either of these groups and the other groups of European populations including those containing western European, central European, eastern European, and Swedish participants (mean F_st_ = 0.0042). As a comparison, the F_st_ between each of the European participant groups and Amerindian (Mayan) participants was >0.12 (0.12–0.13).

**Table 1:**
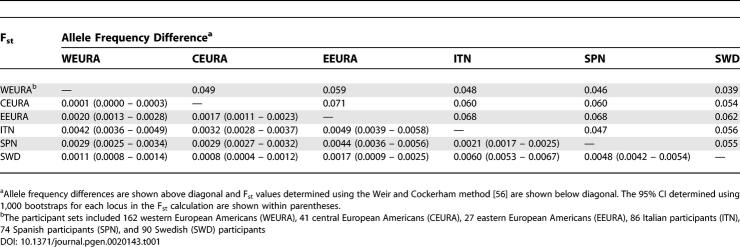
Allele Frequency Differences and F_st_ Values between Selected Participants of European Ancestry

### Evidence for Major Difference in Population Structure of “Northern” and “Southern” Europe

**Figure 1:**
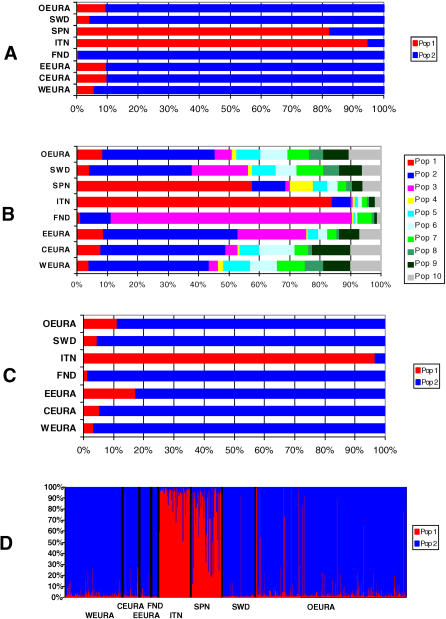
Analysis of Population Structure in Participants of European Ancestry Analysis was performed without any prior population assignment using STRUCTURE [29] (see Methods). The European ancestry groups, western European American (WEURA), central European American (CEURA), eastern European American (EEURA), Finland (FND), Italy (ITN), Spain (SPN), Sweden (SWD), and other European American (OEURA) are indicated by color code. The latter group consisted of individuals with mixed European ancestry from several regions and additional smaller groups (see Methods). The average contribution of each color-coded cluster is indicated by the proportion of the horizontal bars in (A), (B), and (C), whereas in (D), the proportion of each cluster (ordinate) is shown for each individual. (A) Analyses were performed with 2,657 SNPs under the condition of two population (Pop) groups (k = 2). (B) An analysis is shown for k = 10. (C) The results of an analysis using only a selected subset of 400 SNPs is shown for k = 2. These SNPs were selected for potential informativeness for the north–south division in European population structure (see text). None of the participant samples used for selecting this SNP panel (including the Spanish samples) were included in this analysis assessing these same markers. Panel (D) Depicts individual participant results under same conditions as (A).

For the analysis of population genetic structure, we first examined the 928 participants of European ancestry using a set of 2,657 SNPs in which the interval between each SNP was a minimum of 500 kilobases (kb) (Figure 1). Since strong linkage disequilibrium (LD) is rarely observed at chromosomal distances greater than 50 kb in European populations [27,28], this criterion served to reduce or eliminate LD between markers (see Methods). Using the program STRUCTURE [29], the participants were examined under different assumptions of the number of population groups (clusters), ranging from one to ten (k = 1, k = 2…k = 10) without any pre-assignment of population affiliation. The estimation of log_e_ probability of the data using the F model modestly but significantly favored the assumption of k = 2 (k = 1, −3,174,551; k = 2, −3,172,646; k = 3 = −3,173,661; k = 4, −3,173,350; k = 5–10, all < −3,173,500, using mean from five replicates for each measurement). More impressively, the analyses all showed consistent clustering of the Italian and Spanish participant sets from the participant sets of other European ancestry including those of western, central, eastern, and Scandinavian European ancestry (Figure 1A, for k = 2, and Figure 1B, for k = 10). (Hereafter these clusters are referred to as “northern” and “southern” populations). The only additional clear separation of the majority of individuals in any of the self-identified populations was for the Finnish participants that grouped separately from the other “northern” groups when k was greater than 7. When the results for each individual participant are examined (Figure 1D), most of the members of each self-reported ancestry group showed similar results; however, some individuals differed markedly relative to other members of their self-assigned regional ancestry group.

**Figure 2:**
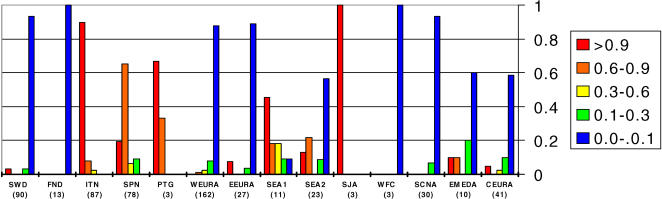
Distribution of “Southern” Population Components among Participants with Various Self-Identified Ethnic or Regional European Origins For each self-identified group, the fraction of individual participants in each group with the color-coded frequency “southern” contribution is shown. For southern European American I (SEA1), only southern European grandparents were identified. For southern European American II (SEA2), grandparents were self-reported as being of both southern European decent and western, central, or eastern European decent. For the eastern Mediterranean American (EMEDA) group, four of ten were of mixed-European decent with one or more grandparents of western, central, or eastern European decent. CEURA, central European American; EEURA, eastern European American; FND, Finland; ITN, Italy; PTG, Portugal; SCNA, Scandinavian; SJA, Sephardic Jewish American; SPN, Spain; SWD, Sweden; WEURA, western European American; WFC, White French Canadian.

Grouping of individuals with different north–south contributions from the k = 2 analysis further illustrates this division of individual participants from different European population sets and some of the variability observed (Figure 2). Italy (84 of 86 individuals), Spain (66 of 74), Portugal (3 of 3), and Sephardic Jewish Americans (3 of 3) had majority contributions from the “southern” population group as defined by this population structure analysis. In addition, a large fraction of southern European Americans (7 of 11) without other reported European heritage had majority “southern” contribution. Those Americans with self-identified mixed “southern” and “northern” heritage showed a substantial but less impressive “southern” population component (8 of 23 with majority “southern”). Those American participants with mixed eastern Mediterranean–reported heritage also had two of ten individuals with a majority “southern” population component. All other groups showed only a few isolated participants with more than a limited “southern” population component. Trends in both the Italian and Spanish participants were also consistent with this north–south pattern: ten of 32 participants from northern Italy had greater than a 10% “northern” component compared with two of 28 from southern Italy; and 23 of 43 from northern Spain had greater than a 10% “northern” contribution compared to five of 19 from southern Spain.

### Examination of Self-Identified Groups

We also investigated the correspondence of the self-assigned groups with those based on genotypes using an application of the Mountain-Rannala algorithm [30]. Similar to the previous analyses, using a leave-one-out cross-validation assignment of population affinity there was a north–south separation (Table 2). There was only a partial ability to separate the different “northern” populations between primarily western European and Swedish groupings. Similarly, there was only partial distinction on an individual level between the Spanish and Italian groups.

**Table 2:**
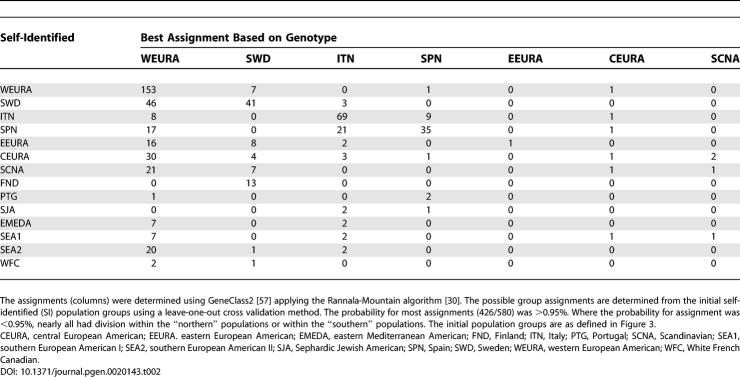
Assignment of Regional European Population Membership for Individual Participants

**Figure 3:**
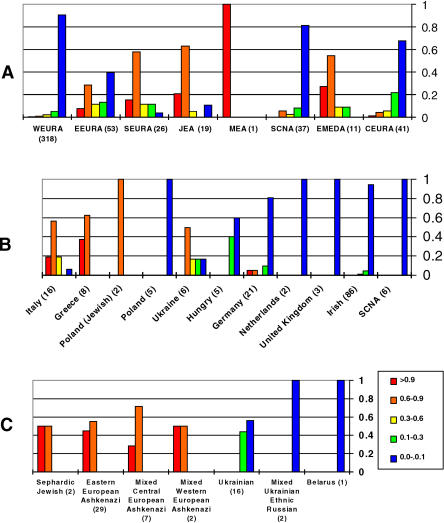
Population Genetic Structure Analysis of the New York City Self-Identified European Americans and a Selected Group of Participants of Jewish and Eastern European Descent Analysis was performed using 749 SNPs informative for European substructure using STRUCTURE. These summary results show the percentage of individual participants in each group with the color-coded percentage “southern” contribution. (A) and (B) show results from participants recruited in New York City as part of the New York Cancer Project. For (A), the individuals were grouped by regional location based on available grandparental data from one or more grandparents. (B) A subset of the same participants as in (A), those with four grandparents from the same country of origin, is shown. (C) shows the results of a different set of participants chosen or recruited specifically on the basis of additional ethnic information (Jewish and eastern European descent). Each of the Ukrainian participants did not have known Jewish ancestry. Similarly, the two participants of mixed Ukrainian and Russian ethnic ancestry (two grandparents Ukrainian, and two grandparents without known Jewish ancestry. For the Ashkenazi Jewish participants, the eastern European group had varying countries of origin, including Ukrainian, Polish, Lithuanian, Russian, and Romanian. For the mixed central and western European Ashkenazi, the participants included those with one to three grandparents from Germany, Austria, or Hungry (central), or England or Belgium (west), and other grandparents from eastern European countries. Each of these Ashkenazi Jewish participants self-reported four grandparents of Ashkenazi Jewish ancestry. CEURA, central European American; EEURA, eastern European American; EMEDA, eastern Mediterranean American; JEA, Jewish ancestry; MEA, Mediterranean European American; SCNA, Scandinavian; SEURA, southern European American; WEURA, western European American.

### Identification of Informative Marker Sets

As a measure of informativeness (i.e., the ability to distinguish populations based on genotypes), the informativeness for assignment (I_n_) [31] was used to select smaller SNP marker sets that might be useful in assessing European population structure. We utilized a small subset of the original participants in order to select these markers, and their performance was then tested on the remaining dataset. Thus, the most informative 400 markers (included in Table S1), selected using 74 participants from Spain and 74 participants from western Europe, showed similar distinction of “southern” and “northern” population groups in Italian and SWD samples as did the original marker set (Figure 1C) (*r^2^* correlation coefficient = 0.77, *p* < 10^−5^). Nearly identical results for these types of analyses were also obtained using other measurements of informativeness including Fishers information content, and informativeness for ancestry coefficient [31,32] (unpublished data). A smaller dataset (top 200 by I_n_) had lower correlation (*r^2^* = 0.62, *p* <10^−5^) and larger dataset (top 800 by I_n_) had a higher correlation (*r^2^* = 0.85, *p* < 10^−5^). Similar marker sets and STRUCTURE results (*r^2^* > 0.7 for sets of 400 and 800 markers) were also obtained if marker sets were chosen based on different sample sets from the “southern” and “northern” groups, e.g., Italian instead of Spanish (unpublished data). In contrast, random sets of 400 markers showed poor separation of the northern and southern populations (*r^2^* values for all ten random sets of 400 were less than 0.1, *p* > 0.01).

### Further Validation in Additional Sample Sets Using Markers Informative for European Ancestry

To further examine and confirm the suggested differences in European substructure, additional studies were performed using a large sample set collected as part of the New York Cancer Project [33]. Information on the origin of all four grandparents was available for 506 of these participants. This sample set was genotyped using the 768 most informative SNPs (indicated in Table S1) that were selected using the I_n_ criteria as defined above. Analysis of the population genetic structure in this dataset also favored the two population group model (log_e_ probability k = 2 > k = 1, k = 3, k = 4…k = 10) and showed markedly different clustering in most of the northern compared to southern European populations (Figure 3A and 3B). In particular, most of the participants with four grandparents with origin in the same European country showed clear membership in the corresponding “northern” or “southern” clusters: German, 22 participants, mean 0.86 “northern”; Irish, 86 participants, mean 0.97 “northern”;Scandinavian, six participants, mean 0.98 “northern”; Italian, 16 participants, mean 0.75 “southern”; and Greek, 7 participants, mean 0.83 “southern” (Figure 3B).

Interestingly, those participants who indicated Jewish ancestry in the New York City participant set had a majority of “southern” cluster membership (19 participants, mean 0.73 “southern”) (Figure 3A). Also, the eastern European ancestry appeared to have substantially higher “southern” contribution in this New York City participant set than that seen in the initial dataset (predominantly rheumatoid arthritis [RA] participants from disparate US locations). When four-grandparent data is examined, this relationship was still unclear since those participants with four grandparents from the Ukraine (without reported Jewish ancestry) showed disparate membership in the “northern” and “southern” clusters, and some of these participants had >80% south membership (Figure 3B). This was in contrast to the “northern”-only cluster membership of each of the five Polish participants without reported Jewish ancestry. This raised the question of whether there might be differences among different Slavic populations (eastern versus western), reflecting different historic migrations or an incomplete reporting of Jewish ancestry. These results also suggested the value in further examining Jewish ancestry and country of origin information in participants with four-grandparent ethnicity information.

### Participants with Ashkenazi Jewish Heritage Group with Southern European Populations

To further clarify the eastern European and Jewish relationships, we examined a final set of participants for which clear information on both Jewish ancestry and eastern European ancestry was available. As shown in Figure 3C, each of 38 participants with four grandparents of Ashkenazi Jewish European ancestry showed >60% “southern” group membership (mean = 0.86 ± 0.08 [standard deviation] “southern”). In contrast, each of 19 pairs of non-Ashkenazi eastern European (west Slavic membership) including 16 with exclusively Ukrainian non-Jewish ancestry showed more than 65% “northern” group membership (mean = 0.88 ± 0.11 “northern”). Consistent with our initial observations (Figure 2), the two Sephardic Jewish participants also showed “southern” cluster membership (Figure 3C).

### Factor Analysis

**Figure 4:**
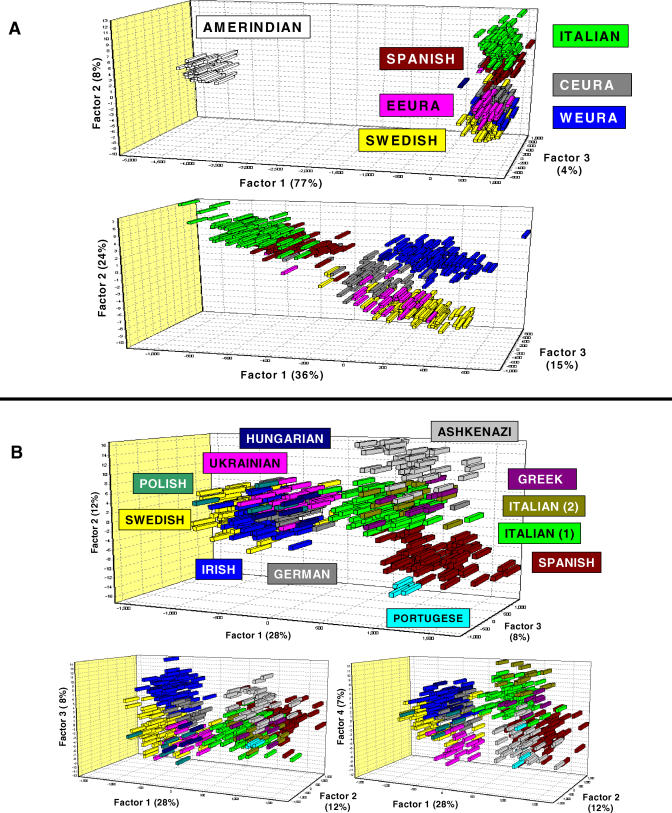
Factor Correspondence Analysis Comparing Different Individuals from European Ancestry Groups with an Amerindian Ethnic Group The individual participants are represented by rectangular shapes distributed by the strength of their separation in three dimensions: along the first factor (abscissa), second factor (ordinate), and third factor (depth). This factor analysis is based on vectors fitted to the individual allele frequencies of each SNP. The percentage of inertia for each factor is provided on each of the axes and correspond to the eigenvalue vectors. (A) The analysis utilized the set of 2,657 random SNPs. In the upper panel, the European ancestry groups, Italy (ITN), Spain (SPN), eastern European American (EEURA), western European American (WEURA), Sweden (SWD), and Amerindian (AMI) are indicated by color code, and show that the ITN and SPN participants are mostly distinct from the other European populations examined. The eigenvalue vectors for factors 1–3 were 0.0234, 0.0024, and 0.0019, respectively. In the bottom panel, the same groups are shown without the AMI participants. The eigenvalue vectors for factors 1–3 were 0.0033, 0.0022, and 0.0014, respectively. (B) The analysis was performed using 749 SNPs chosen for European substructure information. The country of origin is shown by the color coding indicated in the upper panel. Except for the Spanish, Italian (1), and Swedish groups, the participants in (B) do not overlap with those in (A), and were European Americans self-reported as having four grandparents with the same country of origin. The Italian (2) group were Italian Americans. The bottom right of (B) shows a different three-dimensional view (factor 3 as ordinate), and the bottom left of (B) shows factor 4. The vector eigenvalues for factors 1–4 were 0.0059, 0.0026, 0.0017, and 0.0016, respectively.

To further explore the genetic relationship among the populations of European descent, a factor analysis of correspondence was applied using the Genetix software package [34] (see Methods). There was little overlap between the southern European–derived participants (Italian and Spanish) with that of the northern European participants (western European, central European, eastern European, and Swedish) when the initial population samples, genotyped with the unselected panel of 2,657 SNP, were examined (Figure 4A). Similar results were observed with or without inclusion of an out-group (Amerindian). In addition, this analysis was performed for the participant sample set having four grandparents with the same country of origin that were genotyped using the SNP panel selected for European substructure information. The first factor accounting for the largest inertia component (analogous to variance) showed a similar north–south distinction among the different participant groups (Figure 4B). This analysis also showed additional putative relationships among the various European populations that can be discerned by additional factors.

### Structured Association Testing

**Figure 5:**
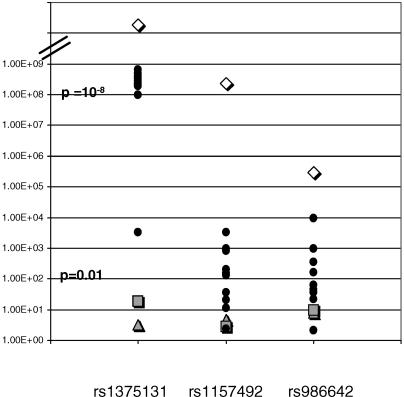
Structured Association Testing Using Unselected SNPs and SNPs Selected for European Structure Information The model examined the real genotypes of Italian participants compared with western and northern European participants, for three different SNPs indicated on the abscissa. The log of 1 divided by probability is shown on the ordinate with the nominal *p*-values (association test without controlling for population structure) indicated by the open diamonds, and the results of the structured association with the entire set of 2,657 SNPs depicted with the gray triangles. The results of the structured association tests are shown for ten random sets of 400 SNPs (filled circles) and for the set of 400 SNPs chosen for north–south population structure (see text) (gray squares).

In order to provide some insight into the potential effect of this European population structure, we examined whether different sets of SNPs could control for population stratification in association tests. We examined three different unlinked loci that had differences in allele frequencies in Italian participants compared with those of western European, and Scandinavian heritage. When 92 Italian participants were used as cases and 255 “northern” Europeans as controls, the three selected loci (rs1375131, rs115749, and rs986642) showed the following odds ratios (OR): 5.4 (95% confidence interval [CI] = 4.0–7.4, *p* = 5 × 10^−28^); 2.9 (95% CI = 2.0–4.1, *p* = 4 × 10^−9^); and 2.5 (95%CI = 1.7–3.8, *p* = 3 × 10^−6^) (Figure 5). When a structured association test (see Methods) was performed using the genotyping results of the entire set of 2,657 SNPs for these participants, no association was observed (*p-*values > 0.05).

In order to further explore the requirements for controlling for European population stratification, several sets of genotyping results using 400 SNPs were examined. These included ten random sets, and the 400 SNPs selected for I_n_ using a subset of Spanish and western European participants. Although the set of SNPs selected for north–south European structure information adequately controlled for association for all three modeled susceptibility alleles (nominal *p*-values above 0.05), the random sets of 400 markers showed substantial variation, and for the most significant model, *p*-values < 10^−8^ were observed (Figure 5). Thus, these data suggest that 400 markers can only adequately control for stratification if selected for informativeness.

We also examined the effect of controlling for population structure in analysis of putative RA susceptibility loci. As part of the North American Rheumatoid Arthritis Consortium (NARAC), SNPs from candidate chromosomal regions were examined using NARAC probands and New York Cancer Project samples as controls. Evidence for association was examined using (1) standard χ^2^ methods; (2) applying population structure information using STRUCTURE [29] and STRAT [8]; and (3) examining only the subset of “northern” participants determined from our STRUCTURE analysis. For the STRUCTURE analysis, we used the 768 informative SNP set described above. As illustrated in Table 3, some candidate SNPs survive our controlling for population stratification by this method (e.g., rs2476601 and rs1291490), whereas others lose significance (e.g., rs10838316 and rs2288774). In the case of rs2476601, the SNP (a variant for PTPN22) has been shown by multiple studies to be associated with RA susceptibility [35–38]. Of interest is finding a SNP that is more significant in a subset of the participants as is shown for the “northern” subset for rs1291490. This latter situation is observed only in a couple of SNPs (out of over 2,000 candidates). This suggests that these SNPs are in LD with a susceptibility gene that has a larger genetic effect in one subpopulation (in this case, “northern”) as might be expected in a complex genetic disease when a more homogeneous population is examined. Of course, these results remain to be confirmed in a separate dataset.

**Table 3:**
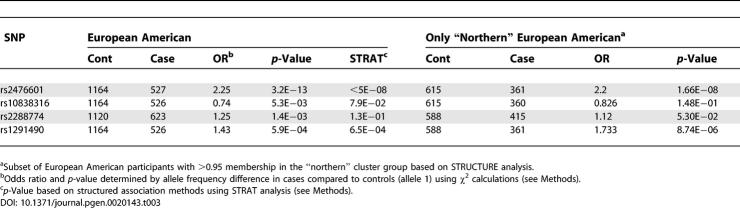
RA Candidate SNP Analysis and European Substructure

Finally, to further examine the effects of population stratification, we used the SNP (rs1375131) that is within the large genomic region that shows positive selection in northern Europeans for lactose tolerance within European populations [39]. Homozygosity of the minor allele is thus a good surrogate for the lactose intolerance phenotype in Europeans, allowing a model to test for the effect of population stratification when studying the genetics of a disease phenotype. First, using the Swedish, Italian, Spanish, and four-grandparent–defined European American genotypes, we examined whether unlinked SNPs showed evidence for association with the putative lactose intolerance phenotype. Of 749 SNPs (selected from the initial 5,000 SNPs), 17 unlinked SNPs and two linked SNPs showed positive allelic association (*p* < 0.001) (Table 4). The strongest allelic association for unlinked SNPs was found for rs905290 (*p* = 1.1 × 10^−7^) and rs233722 (*p* = 6.4 × 10^−6^) on different chromosomes. The association was similar to that of the linked SNPs rs891821 (*p* = 1.2 × 10^−6^) and rs113906 (*p* = 4.2 × 10^−6^) that are located 1.6-Mb proximal and distal to the lactase gene, respectively. When structured association testing was performed (using the structure information provided by the selected informative SNP panel), each of these SNPs was either no longer associated with the surrogate phenotype or showed only marginal association (*p* > 0.01). For the rs1375131, the *p-*value remained significant (*p* < 5 × 10^−8^). One of the linked SNPs (rs891821) had a suggestive association (*p* < 0.005). Next we considered the same model, excluding those individuals with southern European countries of origin. The Ashkenazi population, known to have a high frequency of lactose intolerance, was not excluded since each had four grandparents originating from western European, central European, or eastern European countries. Similar to those results obtained without exclusion of participants of southern European birth or country of origin, we also observed false-positive associations in this participant set unless structured association methods were applied (Table 4). In this latter example, a very high percentage of the putative lactose intolerance phenotype was, as expected, associated with Ashkenazi ethnicity [82.5% (33/40) compared with 16.7% (38/227 in the non-Ashkenazi participants from northern European countries]. When the Ashkenazi participants were exclude from this sample only the rs1375131 (*p* = 1 × 10^−41^) showed nominal association at the *p* < 0.001 level. Thus these data further illustrate the importance of matching or controlling for such ethnic differences in association tests.

**Table 4:**
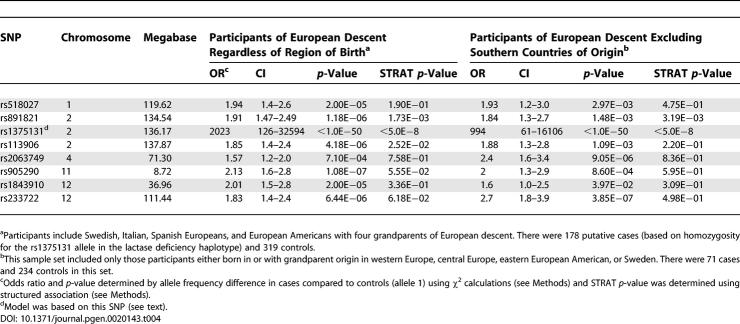
Analysis of Association for a Model of Lactase Deficiency in Participants of European Descent

## Discussion

Using a variety of approaches and algorithms, we have demonstrated that a major aspect of European population genetic structure follows a north–south distribution. Despite the use of over 5,000 SNPs in the initial dataset, the STRUCTURE analyses showed only a modest ability to distinguish other differences in European populations. The Finnish participants were a notable exception in that 11 of 12 individuals showed predominant affiliation with a unique population group (cluster) when the number of groups (k) set in the STRUCTURE analysis was greater than 7. There were some differences in the population group distribution among the different self-identified participants (e.g., see Figure 1B), but it is unclear whether the proportions of these groups have any correspondence to differences in contributions (admixture) of founding populations. A leave-one-out cross-validation study using a different algorithm similarly showed a limited ability to distinguish within the “northern” or “southern” population groups.

Factor analysis of correspondence also showed that the largest component (Factor 1) also aligned with this north and south clustering. This analysis also suggested that individual population groups could be at least partially distinguished when additional smaller factors (lower eigenvalues) were considered. These studies suggest the possibility that additional population structure may be discernable within Europe when larger SNP sets and additional “ethnic” or historical population subsets are examined.

The current study has potential limitations in participant selection including the inclusion of large numbers of RA probands that might bias allele frequencies and the lack of a comprehensive sampling strategy. However, the clear clustering of participants of northern compared to southern European ancestry was consistently observed in this diverse set of participants, including a wide distribution of European Americans and participants from Italy, Spain, and Sweden. In addition, this population genetic structure was observed in ten random sets of 25 individuals selected from the different large population groups (western European Americans, Swedish, central European Americans, European Americans, Italian, and Spanish) providing further evidence that these results cannot be attributed to sample selection bias (unpublished data). The patterns of ancestry in those American participants of multiple diverse European origin also strongly support the current results as does the ability to identify a much smaller set of SNPs that distinguish between the “northern” and “southern” European populations (using a subset of Spanish and western European participants). Finally, the reproduction of these results using a panel of the most informative markers in additional sample sets provides additional support for our findings of a north–south European distinction.

What is the importance of the current observations? First, the potential for false-positive results in association studies based on unrecognized population stratification is of substantial concern for any candidate gene study using a case control design. The potential for false-positive associations in studies of European Americans have recently been emphasized [26]. The use of either structured association tests or genomic control strategies has been suggested by several investigators [8,10,40–42]. In the current study we selected three loci that show allele frequency differences in Italians compared with western, eastern, and central European populations. These three selected loci effectively function as surrogates for test alleles in a case control analysis in which we examined whether these differences could be correctly controlled for by structured association testing. Both the entire set of 2,657 SNPs and a set of 400 SNPs enriched for the north–south informativeness controlled each of the loci. In contrast, 400 randomly selected SNPs showed substantial variation in the ability to account for the European population structure in this study. These results suggest potential problems when limited numbers of SNPs are used to control for European population stratification unless a set of more informative SNPs is utilized.

Second, genetic heterogeneity may be an important factor in decreasing the power of genetic studies. Performing separate analyses on European participants stratified by population genetic structure is worthy of exploration. Although allele frequency differences are generally small between these European populations (Table S1), a comparison of Italian and western European participants showed that 10.0% of SNPs had an allele frequency difference >10%, and 1.9% of the SNPs had an allele frequency difference > 15%. Such differences may be important when examining non-Mendelian traits where low and modest relative risks are the general expectation.

A third issue is the explicit consideration of whether ancestry differences are associated with differences in phenotypic expression. Although controversial [43,44], some have advocated considering the importance of the ethnicity defined by DNA typing in clinical studies [45,46]. Ethnic or regional geographic differences in disease frequency have been noted for both Mendelian diseases and more complex genetic disease. A north/south gradient in the incidence of autoimmune diseases has been noted for several continents, and there is some evidence for increased incidence of multiple sclerosis, type 1 diabetes, and Crohn's disease in northern European compared with southern European countries [47]. Do differences in European population structure underlie phenotypic differences with respect to disease, response to therapy, or adverse reaction to particular environmental agents? The answer is unknown, but this study suggests that the ability to discern European population structure may enable testing such possibilities.

The identification of a subset of SNPs informative for European substructure also raises the question of whether these informative SNPs may also be in LD with physiologically important functions that were subject to selection events. Therefore, we compared the location of these SNPs with those identified by recent studies examining signals for positive selection using the HapMap data [48]. Although the most informative SNP was in fact closely associated with a known positive selection event within European populations (rs1375131within 600 kb of the lactase gene), overall we did not find support for the overrepresentation of the most informative SNPs in the chromosomal positions recently shown as having signals for positive selection in the HapMap European participants (no difference in SNP frequency in the 100-kb regions flanking the 250 strongest selection signals comparing the most informative SNPs and random SNP sets). However, it is possible that signals may be present in either particular subgroups of European participants (e.g. “southern” Europeans not included within the CEPH [Utah residents with ancestry from northern and western Europe; CEU] samples). Ongoing studies will examine this possibility as well as the distribution of European substructure “informative” SNPs when these are chosen from much larger initial genome-wide SNP screens.

The finding in the current study that individuals of Ashkenazi Jewish descent are predominantly “southern” European further suggests the later migration of this ethnic group from the Mediterranean region. Regardless of the European country of origin, each of those participants with four grandparents of Ashkenazi Jewish heritage showed this predominant “southern” cluster membership. This finding suggests the importance of ascertaining this aspect of ethnic origin in the design of association studies in European populations. As an example of this potential issue, we showed that inclusion of Ashkenazi samples with other participants of northern European origin (based on country of grandparental birth) did in fact cause a type 2 error when population stratification was not considered.

It is interesting to speculate how the ability to distinguish northern and southern European populations relates to ancient as well as more modern differences in migration and admixture patterns. Archeological and skeletal evidence as well as studies of mitochondrial and Y chromosome haplogroups have provided evidence of upper Paleolithic, Neolithic, and more recent settlement and migrations as contributing to the origin of current European populations [12–18,22,49–52]. Phylogenetic analyses of Y haplotypic groups are interpreted to support both separate migrations from the Middle East 4,000 to 7,000 y ago as well as a more recent “Greek” expansion into Italy and the Iberian peninsula occurring closer to 2,500 y ago [16,18]. The earlier migrations would be consistent with waves spreading agricultural techniques from the Middle East and are supported by some mitochondrial DNA studies [13]. However, there is little consensus concerning the association of any of these migrations with agricultural techniques or trading routes [50,51], or for that matter with the spread of Indo-European languages [22,51,53]. Some studies of specific mitochondrial and Y haplogroups [53] are consistent with the demic diffusion hypothesis suggested by Cavali-Sforza et al. [22], and the work of Sokal et al. [54] and others have provided evidence of different patterns of repopulation from glacial refuges or have suggested a later influence from North Africa in both Italy and Spain [14,15,18]. As recently discussed by Barbujani and Chikhi, the origin(s) of modern European ancestors remains a controversial issue [55]. Other major population events, including the multiple epidemics during the Middle Ages, may also have resulted in genetic bottlenecks contributing to current differences in European population structure.

Regardless of the historical explanations for the north–south genetic differences we have described, our results emphasize the importance of considering population structure in both genetic and epidemiological studies in European populations. Future examination of population structure using larger numbers of SNPs in additional population samples may enable a better definition of the differences between European population groups, and similar studies may provide analogous information in other continental populations.

## Materials and Methods

### Statistical analyses.

F_st_ was determined using Genetix software [34] that applies the Weir and Cockerham [56] algorithm, and δ was calculated by determining the absolute value of the allele frequency difference between two populations. The 95% confidence limits for F_st_ were determined by permutation testing (set at 50,000). The measures of informativeness of each SNP (I_n_, I_a_, ORCA, and FIC) were determined using the algorithms previously described [31,32]. (We thank Dr. Noah Rosenberg for providing the Perl script used for I_n_, I_a_, and ORCA). LD was examined using the Genetix software [34]. For the set of 2,657 SNPs, there was no evidence for LD among adjacent markers in each self-identified ethnic set (*r^2^* < 0.2).

Population structure was examined using STRUCTURE v2.1 [1,29]. Each STRUCTURE analysis was performed without any prior population assignment and was performed at least five times with similar results (see Results) using more than 10,000 replicates and burn-in cycles under the admixture model applying the infer α option with a separate α estimated for each population under the F model (where α is the Dirichlet parameter for degree of admixture). Most runs were performed under the λ = 1 option where λ parameterizes the allele frequency prior and is based on the Dirichlet distribution of allele frequencies. When λ = 1 a uniform prior distribution of allele frequencies over all loci is used. Runs using the infer λ option or setting λ = 1 showed similar results for a limited number of selected analyses. The leave-one-out cross-validation analysis of ethnic group affiliation was performed using the GeneClass 2 software [57,58] applying the Rannala and Mountain algorithm [30]. Structured association was performed using the STRAT software [8] that performs association tests with and without population structure information that is provided by a prior analysis with STRUCTURE.

Factor analysis of correspondence was performed using the Genetix software [34] that utilizes a strategy similar to that discussed and described by Guinand [59]. This methodology can be considered a special case of principal component analysis in which the correspondence analysis is applied to contingency tables to develop a correspondence matrix. The groups of individuals are viewed as a group of positions in hyperspace that has as many dimensions as alleles for each different loci. The algorithm determines the independent directions in hyperspace where the size of the vector is proportional to the inertia (number of individuals in a point of the hyperspace) multiplied by the square of the distance to the center of the co-ordinates. The objective of the method is to decompose the overall inertia to a small number of dimensions in which the deviations from expected values can be represented within given constraints. A description of the mathematical principles is available (http://www.unesco.org/webworld/idams/advguide/Chapt6_5.htm) [60].

Hardy-Weinberg equilibrium was examined using an exact test implemented in the FINETTI software that can be accessed interactively at the Internet address: http://ihg.gsf.de/cgi-bin/hw/hwa1.pl. This program was also used for χ^2^ statistic calculations for the association tests.

### Populations studied: First sample set.

European Americans of different regional European origins (681 participants), East Asian Americans (13), African Americans (22), South Asian Americans (48), Amerindians (48), and Swedish (92), Finnish (13), Italian (91), Portuguese (3), southern France (1), and Spanish participants (82) were included in this study. None of the individuals were first-degree relatives of other participants in the study. These populations were based on self-identified ethnic affiliation. The European Americans, African Americans, and East Asian Americans were recruited from across the United States, and the majority of the participants, including all of the European Americans, were RA probands identified as part of the North American Rheumatoid Arthritis Consortium (NARAC) as previously described [61]. The South Asian American participants were recruited from Houston, Texas, and Amerindian participants were self-identified as Mayan (Kachiquel language group) and were recruited in Chimaltenango, Guatemala, as previously described [11]. The Italian participants were normal healthy volunteers recruited from throughout Italy: 38 from northern Italy, 23 from central Italy, and 30 from southern Italy. The Swedish and Finnish participants were healthy normal controls collected in these countries. The other participants recruited in southwestern Europe included 86 from Spain, three from Portugal, and one from southern France. Of the Spanish participants, there were 43 from northern Spain, 12 from central Spain, and 19 from southern Spain. Of these participants from Spain and Portugal, 61 were probands for a multiple sclerosis study. Blood cell samples were obtained from all individuals, according to protocols and informed-consent procedures approved by institutional review boards, and were labeled with an anonymous code number linked only to demographic information.

For the European Americans, grandparental information was available for the majority of the participants. These included the following self-identifier classifications of grandparents: western European (United Kingdom, northern France, Holland, Belgium, and Switzerland), eastern European (Russia, Poland, Romania, Ukraine, Lithuania, Latvia, Estonia, and Czech Republic), central European (Germany, Austria, and Hungary), southern European (Spain, Portugal, Italy, and southern France), Scandinavian (Denmark, Norway, Sweden, and Finland), and eastern Mediterranean (Greece, Turkey, Croatia, Bosnia, Yugoslavia, and Albania), Sephardic Jewish American, and White French Canadian. All participants with any reported mixed-continental origins (e.g., African) were excluded.

### Populations studied: Second sample set.

This sample set included 1,164 self-identified European American participants that were recruited as part of the New York Cancer Project, a prospective longitudinal study [33]. For a substantial portion of this set, European country of origin was available as was a record of the four-grandparental country of origin. Jewish ancestry was indicated for a subset of these participants, but specific information for each grandparent was not available for this aspect of the study.

### Populations studied: Third sample set.

This sample set included 40 participants of Jewish ancestry who are part of a larger Ashkenazi Jewish control population recruited by Dr. Ann E. Pulver (Johns Hopkins University School of Medicine). Both the country of origin and the Jewish ethnic information for each grandparent were available for each participant. The participants included 38 individuals with four grandparents identified as Ashkenazi Jewish and two with Sephardic Jewish grandparents. In addition, 19 non-Jewish participants of eastern European ancestry were specifically recruited for the current study, based on self-reported information on the origin and ethnicity of all four grandparents.

### Exclusion of individuals with evidence of non-European ancestry.

To simplify the analysis of European population structure, initial studies were used to identify individuals in the first sample set that were likely to have substantial non-European admixture. STRUCTURE [29] analyses were performed using four different sets of 150 SNPs selected for informativeness (I_n_) for European ancestry when compared with African, East Asian (Chinese, Japanese, and Filipino), South Asian (Indian subcontinent), and Amerindian (see Table S1, for specific SNPs utilized). This set of SNPs provides world-wide information with respect to continental population admixture. For self-identified European Americans (681), this initial study using the STRUCTURE algorithms identified 11 participants with evidence of >10% non-European ancestry. Similarly, two of 92 Swedish, four of 82 Spanish, and five of 91 Italian participants were excluded from the additional studies.

For the second sample set (New York Cancer Project), a similar exclusion was performed using a set of 140 world-wide ancestry informative markers [11].

(It should be noted that without exclusion of individuals with evidence of continental admixture, the analyses of population substructure showed inconsistent results in these participant sets).

### Genotyping.

The initial genotyping was performed using the Illumina Linkage IV Panel using the Illumina bead array method as previously described for the Linkage III panel (Illumina, San Diego, California, United States) [62]. For each European ethnic group, very few SNPs showed deviation from Hardy-Weinberg expectations (<4% at *p* = 0.05 and <1% at *p* = 0.01). A second SNP typing set of 768 SNPs chosen for informativeness (see Results) from the initial genotyping results was used for typing sample set 2 and 3 using the same methods. The results for 19 of the 768 SNPs were not included in the analyses based on evaluation of the quality of the typing results.

## Supporting Information

Table S1 SNP Frequencies and Information (796 KB PDF)

## Abbreviations

CI: confidence interval
kb: kilobases
LD: linkage disequilibrium
OR: odds ratio
RA: rheumatoid arthritis
SNP: single nucleotide polymorphism
