# Supplementary material for: European Population Substructure: Clustering of Northern and Southern Populations
Source: PLoS Genet. 2006 Sep 15;2(9):e143. doi: 10.1371/journal.pgen.0020143 (PMC1564423; doi:10.1371/journal.pgen.0020143)
Supplement: Supplementary file 1 [file pgen.0020143.st001.pdf]

Supplemental Table 1. SNP Freqencies and Information.

						Frequency of Allele A in each ethnic group[b]											informativeness for assignment (In)[c]							
SNP	Chr	Mb[a]	Sequence 5' to 3'	Allele A	Allele B	AFA	SWD	WEURA	CEURA	EEURA	EAS	AMI	SAS	FIN	ITN	SPN	WEURA/AFA	WEUR/AMI	WEURA/SAS	WEUR/EAS	WEUR/SPN	2657 SNP Set[d]	400 SNPset (InWEUR/SPN)[e]	768 SNPset (InWEUR/SPN)[f]
rs884080	1	1.90	GAGGGCCTGCCCTCCTGGCAGGGGCCCTTGTGTGCGTCCTGAGCTCACC[A/G]TGTTGGAGGTGGCTGGGCAGTGGCGGGCAGAGCCCCATGCTGTCCTCTGCCTTTG	A	G	0.14	0.52	0.52	0.49	0.32	0.06	0.40	0.32	0.46	0.59	0.60	0.109	0.008	0.011	0.162	0.0032	1		
rs2017143	1	2.09	CAGGTGTCAAGTCCTGACAGAGAGGAGGCAGGAGGGCCACCTCGGGTCTCGAGCTCCCTGCCTAGAGA[A/G]CCTCAACATAACAGAGGAGCTGCATCTCTAGCCTCTGC	A	G	0.69	0.41	0.48	0.41	0.54	0.64	0.61	0.44	0.50	0.39	0.39	0.014	0.001	0.010	0.005	0.0057			1
rs2840531	1	2.16	GACTCTCAGCCCGGCTCAGGCTGTTTCTGGCTTGACGATGATGTGAGTGTCCAGCC[A/G]TAAATTCTCCAGCACAAAAGATATTTCCCTTGGGAAACCTAGAGGAACGTGCTTT	A	G	0.05	0.18	0.18	0.13	0.15	0.32	0.59	0.20	0.21	0.13	0.16	0.014	0.088	0.000	0.014	0.0004			
rs2477703	1	2.30	GGTGGAAACATTCGCTCACACCTGCCTCAGCGTCTCTCATGTGTTGTTTTAAACTC[A/G]TTAAGTTCTGAATCCTTTTTCCCACCAGTGGTGACTACGTAATAACTGCGTTTGT	A	G	0.93	0.76	0.75	0.59	0.71	1.00	0.84	0.82	0.79	0.78	0.70	0.031	0.006	0.001	0.096	0.0022			
rs734999	1	2.38	TTAACTAATGCGTCTTGTTGAGTCGGAGTCACATAAGTCTCCTGTTCTCTGTT[A/G]TGCTTTTCTTTCCCACGGGTATTTCAGTGGCTGTTTGCACCGTTTCTGTTGCCCTGTCTC	A	G	0.74	0.48	0.45	0.45	0.48	0.36	0.56	0.69	0.46	0.41	0.48	0.044	0.006	0.046	0.006	0.0005			
rs2377041	1	2.50	ATGCTCAAATATGTTGAGCATTACCAGAAAATGATCAACAAAGTCACACCGAATC[A/G]TGAGCTTCTGAGGAAGGAGGGGACCCCGGGTGCCCAGAGCACAGGCACTCAGGG	A	G	0.24	0.47	0.48	0.44	0.45	0.30	0.46	0.24	0.39	0.55	0.49	0.040	0.001	0.047	0.019	0.0001	1		
rs2606418	1	2.65	CTAAGAAGTTCTACAAACCCCAAACAGGTCTATGAAAGTTAGGGCTGTGAATATG[A/G]GTGGCCTACAGAAAGATGGAGGGAGTAATCATCAGGGTGAGGACCATCTCCTAC	A	G	0.48	0.66	0.71	0.68	0.63	0.50	0.41	0.63	0.68	0.73	0.69	0.022	0.048	0.006	0.025	0.0002			
rs2027262	1	2.71	CAGGGCATCTGAGTGGAAGATACCACAAAGGCAACTGCTAGTCTCAGACTTC[A/G]CTCCCAGCTTGAATGATGGAGGCCTTGGAGAGTCTCTGGGGAGAAGTGGAGGCTGG	A	G	0.95	0.87	0.86	0.88	0.95	0.95	0.65	0.98	0.96	0.89	0.85	0.005	0.069	0.010	0.002	0.0010			
rs2981881	1	2.79	AAAATTGATTTGTCAATAACCAGAGACTTGTTGTCCAAAGGCATCTCAAGGTGTGAGA[A/G]AAAAAAGAAGAGTAAAAATGGAAACAATATGGTACTGGCAGACACAACCAAGACC	A	G	0.69	0.74	0.66	0.59	0.80	0.91	0.99	0.94	0.79	0.70	0.63	0.001	0.134	0.051	0.047	0.0006			
rs7534363	1	3.01	AAGGATTTTATCAACTGATCAACTTCCATTCGAATAACAAGTTTCAGCCAACATC[A/G]TTACCTGCATGTGGGAGGCTGTGGACGAGTGGGCAAACAGATGGGAAATGGTCAC	A	G	0.57	1.00	1.00	1.00	1.00	1.00	0.98	1.00	1.00	0.98	0.98	0.178	0.009	0.000	0.000	0.0061			1
rs2065525	1	3.09	AACACCGCCTCAAGGGATTCGTTCTCTCTGTTTTAGGTCCATAGGCTGAATTAAAG[C/G]CACAAACCCAGAGGGAACTCGAAATGCACATTTAAGCAAATGCAACACCAAAATG	C	G	0.45	0.52	0.52	0.60	0.54	1.00	0.82	0.76	0.68	0.47	0.47	0.003	0.053	0.056	0.202	0.0028	1		
rs2821039	1	3.19	TCCACAGCCCTCCACGGCCCTCAAAGGAAGGGCAGTACCAGGAGCTCTGGGCCC[A/G]TGAAGTGTCCTTCTCAGTGGTCACCAAAGGCCAGGGTCCCCTCTGGCTGGGACT	A	G	0.53	0.10	0.11	0.11	0.10	0.18	0.32	0.07	0.07	0.09	0.07	0.113	0.050	0.000	0.010	0.0008			
rs878063	1	3.26	CAATTTGGTTCAGCCAGCCGGCGGTTTTGGATGCGCCACTTACCATGAAACTTC[A/G]TGTAATGAGTGTGACGTTGTCACCTCTGGGAGCTCATGGGGGTGTCTTTGTGCCAA	A	G	0.43	0.85	0.91	0.93	0.88	0.85	0.97	0.79	0.89	0.88	0.88	0.150	0.003	0.021	0.007	0.0018			
rs1870509	1	4.24	CTGGAGGAGTCTGCCTTTCTTTTTCTTTTATTTTTATTGTGGCAAGAACACAAC[A/G]TGAGGTCTACCCTCTTAACAGAAGCAGATCGCTGGGACTTACATATTGCGTAACTGGA	A	G	0.33	0.46	0.51	0.56	0.50	0.18	0.09	0.41	0.25	0.41	0.44	0.032	0.171	0.020	0.088	0.0040	1		1
rs2035453	1	4.38	ATTGGCTGAGATTGGCATTGGGATGGAGT[A/G]GTGAAGCTAAGAAATTCGTTATCCGTTTGTCCAGTGCTGCTGGACTTTTCACTAAGTGAAGAGGTAAATGCTGAGTCTCCC	A	G	0.45	0.33	0.35	0.27	0.36	0.08	0.16	0.59	0.29	0.30	0.34	0.002	0.047	0.019	0.044	0.0004			
rs557477	1	5.02	GAGAGTGTGCCCTCTGCTGGCCCACTGTGACTTTGGCACATAGGATGGAAGA[A/C]GTGGAGCTGGTGCCCAGCACTCAGGGTTAGTGGAGCCACTCCAAGCCTGCACATGTC	A	C	0.31	0.64	0.69	0.62	0.63	0.36	0.38	0.58	0.71	0.46	0.56	0.078	0.039	0.011	0.049	0.0088	1	1	1[g]
rs912991	1	5.03	AGGATCGGGGTCCCCACACACAGGTGAACGACATGAGTCAGCCAGACTCACA[A/G]CATTTCCACGTGAGGAGACATAGACCTTTGTTGTGCAGAGTCCCTGGGTTGGAGCT	A	G	0.57	0.53	0.59	0.66	0.63	0.17	0.66	0.43	0.43	0.55	0.52	0.000	0.008	0.008	0.074	0.0020			
rs1570964	1	5.05	GGGCATCCCTTACCTTGTGCCAGATGCGTGCTTGCAAATAATTTTCATGGTTA[A/G]CATGTTGGTTCATTAATTCCCAAACTAGACTGGATCTTGTAAAATCTCAGGGAAACTTG	A	G	0.14	0.40	0.43	0.50	0.50	0.27	0.45	0.38	0.43	0.45	0.38	0.037	0.007	0.000	0.004	0.0007			
rs729206	1	5.07	ATTACTGGCTGCTGTGATTGCGATGGGGAGACAATTACTCAGTTTGTTTCAC[A/G]TAAAAACAGGAAGAATATGCTGCCATCAAAGCGAGGTCTAGCCCCACTCTCCTGGGTA	A	G	0.38	0.53	0.62	0.71	0.66	0.27	0.65	0.46	0.46	0.58	0.53	0.022	0.002	0.010	0.048	0.0054			
rs531099	1	5.77	ACATTATTTCCTGGGAGATCCGGCAGCTCTTCACTCTTCCGAATCAGGCTGTGGGAAAC[A/G]TTTGTGTGCATCTCGCTTTATTTCTCTCTTGAACTATTAGGATAAAGTCACA	A	G	0.57	0.52	0.53	0.56	0.50	0.50	0.41	0.54	0.54	0.53	0.53	0.002	0.006	0.009	0.000	0.0000	1		
rs709209	1	5.99	CCGTGGATGAGCAGTCAGAGAGTCTACAGAACACGCACGACGACAGCAGGAACA[A/G]CGCGGCCTCAGCCAGGTAAAGCAAGTCTCTCCACTGGAGAGTGTGCACGCGAT	A	G	0.21	0.70	0.64	0.71	0.57	0.82	0.94	0.58	0.71	0.65	0.67	0.080	0.083	0.005	0.032	0.0001			
rs200440	1	6.42	TTAATGGGCACATTTCACAACACCAGGGGAAACATGGGGCTGCATGTCCAGA[G/T]GGTATCAGATAGCACAGTGACTTCACTGTGTGCCTTCCCTCTGTGGTTGCCATCAGG	A	G	0.52	0.45	0.44	0.50	0.45	0.59	0.16	0.34	0.50	0.43	0.52	0.007	0.042	0.003	0.021	0.0035	1		
rs707455	1	7.53	GTACCACAAACTGTGACTTACACCACATGCATTGCCTTACATTTGATCATTCTC[A/G]TCATTAAGGCAGGTTCGTAATTGTCAGTGTAGAGAAATACTCTTACAGCAGACAGCAGC	A	G	0.73	0.46	0.41	0.37	0.30	0.73	0.81	0.42	0.54	0.46	0.52	0.062	0.101	0.005	0.073	0.0049	1		1
rs697674	1	7.55	TACAGTTAGCGTGCTGCAATCAGATCCAGATGTATCCTGTCCTCTGCCTTGTTTA[C/G]AAGTGACACTTTGAGACACAGGGCAAGCTGAGCAGCCTTTCCATATGTGGAAAACT	C	G	0.79	0.81	0.84	0.89	0.89	1.00	0.99	0.98	0.68	0.91	0.80	0.003	0.028	0.015	0.045	0.0015			
rs8627	1	8.12	GCATCCATTTTTCCAAAGAAGAGTCGAGTCCTCTGAGGTCTGTGCTTGAAA[A/G]CCGTCCGAAGGCATTCTTAGTTAGCTTTGCTTTTCTCCCCATATCCCAAGGCGAAGCGCT	A	G	0.31	0.46	0.49	0.51	0.52	0.75	0.79	0.40	0.43	0.47	0.60	0.030	0.040	0.005	0.025	0.0021	1		
rs1473420	1	8.33	TAGGGTCTTACTAAGGGGTATAAAACATAGCC[A/C]AGAGATACTTTGTTGACAAAAACTGTACATTCTGTTATTGTTATTCTTGCAATTTATGATTCCTCTTCTCTATTTGCATA	A	C	0.62	0.38	0.37	0.29	0.45	0.18	0.25	0.57	0.29	0.49	0.49	0.013	0.020	0.006	0.039	0.0068		1	1
rs737261	1	8.97	GTGATTTATGGTGGGATCTAAAATTTCTTTGTAAAAAGATACCCTTTTGCTTTG[C/G]GAGGGATCACAAGGAGCTCAGCCCTCGTGGCCCAGGACCACCTGGGCAGATGCCA	C	G	0.29	0.45	0.44	0.38	0.38	0.18	0.18	0.43	0.23	0.48	0.53	0.004	0.032	0.000	0.026	0.0079	1	1	1
rs916380	1	9.05	CTCTCTGTGCGCCTCTTTTCTGGAAAGCAGGGTTTATTATTCAGTTCACAGA[A/G]GAGGAACAAGCTCACAGTGGTGAAACTGCCCTGTGCCACTCTCTCAGCAGCAGGGTC	A	G	0.55	0.57	0.54	0.57	0.54	0.64	0.65	0.58	0.46	0.60	0.55	0.001	0.140	0.000	0.003	0.0000			
rs630075	1	9.08	TTCTCATTTGGAGATTTGAAGAAAGGTCATAATGAAAGGTTGCCTTGCATTT[A/G]CATTCTACTTCTAAGGGGGGGCAGCAATGCCAAAAGCAAGCTCCTTACAACTATAATTGTCC	A	G	0.50	0.36	0.27	0.28	0.23	0.68	0.68	0.36	0.50	0.28	0.34	0.034	0.094	0.002	0.097	0.0033			
rs6698575	1	9.34	TTCTGGGTCCTTTCTTACCAAAGGGTGATGCTGCTGAATGACCAGTGTCTTTTTCT[A/G]TGTTGGATTTGCCTAAATATAGCGGACATAGCAAATAGTCATTGAGAATGCTAGGC	A	G	0.62	0.79	0.83	0.82	0.77	0.73	0.74	0.74	0.75	0.88	0.80	0.032	0.010	0.006	0.012	0.0007			
rs6677649	1	9.46	AAGGGAACAAGGGCTCCAATGAGTGGGAGCAGAGGCTCATTGGTTCCTGCCTT[A/G]TGTTTTGGGATTTAACAAGAGGATTGGCCCTTTTAGTAGCAAGTAGAGGAGCAGC	A	G	0.78	0.88	0.92	0.93	0.95	0.73	0.61	0.68	0.82	0.89	0.94	0.041	0.088	0.075	0.059	0.0005			
rs2119508	1	9.56	TAAAAACAGGGTAAGTAACGCAGGCACTTGCAGGCTGGTGGAGGAACACGTTTTC[A/G]GAACAGAGCCCGAGCTTTCTCTTAGGTTTATACCTTGTCCAGAGAATTAAATACAC	A	G	0.02	0.04	0.10	0.12	0.11	0.00	0.51	0.16	0.07	0.07	0.10	0.006	0.129	0.010	0.031	0.0001			
rs2379107	1	9.62	TGATGCCTTCGGAGGGTCTGGCTCACTGTGGTTTTAAATAGCCATTCCTCTTTGC[A/G]GCAGCTTCTCCTTCACCCAAACCCAACACCTCTTCCAAGAAGTATGGCCGGGCCCA	A	G	0.69	0.75	0.78	0.80	0.87	0.78	0.64	0.58	0.79	0.83	0.81	0.015	0.039	0.039	0.242	0.0000			
rs7528979	1	9.80	CCGCTTGTTCCCTGAGCAGCCTCCTTTGGCCGCCACTGCTGCTGCTACAGGGTT[A/G]TGTGTTCGCCAGCCCCACTTATTACCCTCTGCTTCCCCAGGTTACCCAGCTAGGCAGCGCC	A	G	0.69	0.18	0.14	0.16	0.14	0.41	0.02	0.40	0.25	0.17	0.19	0.208	0.004	0.062	0.091	0.0040	1		1
rs2295294	1	9.90	AAAATAAAAGCTCTGTATCTGGAGCTCATCTTTGATACTTCCTCTGGATTTGTTT[A/T]CTGAACACTTACCCTTCCGAGCTCTAAGTAATGCTGGCAGATTGGCTAACCACACAG	A	T	0.64	0.33	0.34	0.37	0.48	0.45	0.51	0.46	0.39	0.41	0.40	0.053	0.021	0.007	0.015	0.0014			
rs6541085	1	10.00	AGGAGCAAGTGAGAAGGTCCTGGAAGAATTAAGGATTTAAAAAGAACAAGAC[A/G]AAGGACACAGTGGGGAAAGGGAAAGTTGGAGCAAAAACACAGGGCAGAAAAAAC	A	G	0.36	0.56	0.52	0.51	0.50	0.73	0.56	0.57	0.50	0.52	0.50	0.009	0.001	0.003	0.031	0.0000			
rs912962	1	10.06	TTCTGAAAAAGTACCTCCTACATGGCTGCCTTACTGATGCCCAGGTTCCTCTTTTA[A/C]TCTTACATCTGTTTTTGCTCTTTGCCTACCTTTGCTGGTTGTTGTTGTTCTTCATCTTTCTC	A	C	0.33	0.56	0.53	0.51	0.50	0.73	0.56	0.57	0.50	0.52	0.50	0.013	0.001	0.003	0.031	0.0001			
rs649101	1	10.20	AGCTCCATGCACCTGTACCTCAGGTGTATCTTGCAAAATCGACCATCAACTTT[A/G]TCATTCTATCACCAACAATAGCGTCACAAAGACTACAATGAGTGAACGACCCGTGACC	A	G	0.36	0.55	0.46	0.50	0.55	0.64	0.28	0.49	0.50	0.47	0.55	0.005	0.034	0.001	0.010	0.0034			
rs2506887	1	10.29	GGAAGGGCATCAGATATAGGGCATAAAGACCCTCCTTTCATCTTCCCATTATT[A/G]GTAGGACACAGTGACTCTTGGCCAAGATGGCTCTGGTGCTCAGTAGGCCTTTGTGG	A	G	0.81	0.84	0.83	0.87	0.95	0.82	0.88	0.78	0.86	0.79	0.85	0.002	0.000	0.006	0.001	0.0001			
rs481453	1	10.38	GCTTCTGAGCGAAGCTGATGGGCTGATGTGAGAAGCTAGAGACACTGCTGTAT[A/G]GAGACATGGCTCCAACCCTGATGAGCCTGGAGGCGACCTCAATGGGCTGGCCCCG	A	G	0.64	0.86	0.85	0.88	0.91	0.95	0.98	0.88	0.86	0.82	0.84	0.032	0.031	0.007	0.019	0.0001			
rs488834	1	10.48	GTCTTCCCAGTACTGTGCCTCTCCCCGGCCTTGGAGAATCCTTCCTAACTAC[A/G]TCAGCTACCAGGTCCCAGATGTGCTGGCCACTCACTCATATGGGCCACTCACAAA	A	G	0.43	0.73	0.77	0.87	0.77	0.68	0.40	0.72	0.64	0.78	0.77	0.090	0.098	0.003	0.011	0.0005	1		
rs7555879	1	10.60	AATCCTCAGAGGGGAGAAACCCAACAAGCTATTTTCATTTGGAAAACAGAAAA[A/G]GAAACGACCCATGGGTGAGACAGGGGCAATGCTGGGTGGGGGCCCTATCCTTAG	A	G	0.21	0.05	0.08	0.02	0.00	0.05	0.28	0.06	0.11	0.03	0.01	0.022	0.044	0.000	0.002	0.0168		1	1
rs718206	1	11.00	ATAGTTTTTCTTCCTGTCACAGCTGTATCTAAGGCTGTTCTGCCTGTGCAGAAA[A/T]GTCTGTTGCTCATTGAGTATCTAGCAGCTACTTCATCTGTCGTGGAGGTCTGGGTT	A	T	0.67	0.23	0.27	0.28	0.29	0.00	0.00	0.16	0.36	0.26	0.24	0.127	0.065	0.003	0.065	0.0008	1		
rs2179393	1	11.12	TGACAGGCGTGAGCACCTGCACTTGGCCGGGAAGGATTCTTTATCTTTGACTA[C/G]GAGTGCTTGATGAAACACCGGGGCTGGGGAAGGGGTGGGAGGATGAGAAGTGGAC	C	G	0.10	0.49	0.54	0.57	0.48	0.14	0.02	0.18	0.61	0.43	0.53	0.138	0.246	0.114	0.122	0.0000			
rs3010218	1	11.21	AACTCCTCCCTAGGGAAAGAAGTCTCCTCTGTCAGGCCCACAGCAGAGAGTGATCC[A/T]GCTGCTGCTGAAGATCTTTCTAGAACAAATCAGACCCCACCACTCCCCTCCAATC	A	T	0.10	0.08	0.08	0.06	0.04	0.00	0.00	0.07	0.04	0.03	0.08	0.000	0.043	0.000	0.043	0.0011			
rs4846012	1	11.27	CAGTCAGATATTTGTTGAGACAAAGAAGGAAAAGGAGTCTCCGATGAACTTGGGTG[A/C]GGTATTCAATAGAACTTTCCCCTGGACAAATGAATTAACAGCATTCACAGCAACC	A	C	0.93	0.33	0.37	0.40	0.30	0.77	0.36	0.78	0.32	0.42	0.39	0.187	0.001	0.091	0.086	0.0003			
rs2817594	1	11.33	GGGAGTGCCCTCCTGTTGAGATGAGTCTGGCTCTTTGGCTCTCTGCGATGTAG[A/C]ATAGGGGGAGTAACAGGAAAATCTAGGGCTGAGATGAAGGCGGGGCTAGGGGGTT	A	C	0.55	0.99	0.99	0.99	0.98	1.00	1.00	0.98	1.00	0.98	0.95	0.140	0.013	0.002	0.013	0.0043			1
rs6685318	1	11.41	ATATGCAGTGTTCTCACAGTAGGGACTATAGAGATAATAAAGCTTGGCAAGATCA[A/T]CTTGGCCATTGGGGATCTTTCCTACATGAGGAGAACGGGGGTAACACAGAGTGAG	A	T	0.17	0.11	0.09	0.16	0.11	0.18	0.17	0.13	0.07	0.11	0.07	0.009	0.008	0.007	0.017	0.0021			
rs6671723	1	11.68	ATGTTCGTCTTTAAAATTATTTCCCCTTTTTCCTTCAACATATCTTTCTCGGAAC[C/G]GAGATTGCTGTCCCAGAATTGTCTGAAGAAAAAGGCTGAAGTCAATAGCTCTTTTGG	C	G	0.67	0.61	0.64	0.61	0.68	0.23	0.76	0.53	0.50	0.73	0.64	0.000	0.009	0.000	0.083	0.0002	1		
rs3818157	1	11.74	GACTCTTGACATAGGGGTGCTGGCATGGGTATCTGCAGGCAGGTAGGAGTAGAG[A/G]ATTGAGAAAACCTGAGATGGAGTTTGGTCCCTGAGCTGCTGTTGCCACCGTGTGC	A	G	0.50	0.54	0.45	0.49	0.54	0.77	0.31	0.64	0.46	0.47	0.51	0.002	0.008	0.009	0.057	0.0023			
rs4644506	1	11.82	GTAGTAAACCCTTTAAATCTAGCTGGGCTCATTGTCTCCTTAGCATCTGAATCTAT[C/G]AGAATGTGGCAAGCCGAACTAATTGCTTGAAAAATTATTATTATTTGAGACTGAAT	C	G	0.55	0.74	0.74	0.70	0.64	0.36	0.84	0.45	0.71	0.68	0.70	0.020	0.100	0.076	0.083	0.0007			
rs235256	1	12.01	ATGGCTTTTAATGAACCTGTGGAACTTGAAGAATGCTTCTCCGCAGGGTAGAA[A/C]AATGCTTTTTCTTTTTGTAACTGGTGTAGTATGGTACCCAATGCTTATGGATCCAAAGC	A	C	0.57	0.10	0.09	0.04	0.09	0.23	0.34	0.14	0.14	0.08	0.08	0.142	0.053	0.005	0.019	0.0000			
rs761162	1	13.73	GTGGCTACACAGTTAATGGCGAGAGAGGAGTCTCATTTACCAATTCCCATTTTAGAA[A/C]ATTGCCTGGTTATCTCATATTACTTACCACTCTTAATGATAATAATAATAATAATA	A	C	0.15	0.48	0.55	0.48	0.48	0.27	0.03	0.45	0.50	0.47	0.48	0.076	0.164	0.004	0.025	0.0027	1		
rs1416633	1	13.85	TGGTGGACTATAACAGAATTCAAATCTTCACCATATTAGCTATCTATTGCTGTATAA[A/G]AAATTACATCCAAACAGAGCAGCTTAAAACAATAACAGATCCTTATCTCACAATGT	A	G	0.05	0.22	0.25	0.27	0.23	0.05	0.02	0.32	0.25	0.24	0.25	0.041	0.061	0.003	0.045	0.0001			
rs2794609	1	14.03	CATCATCTCGGCCCTGACAACTCTACTTTTGAAAACCATGTTTGATTTTTATTGATTT[C/G]AGACTGGAACCCGAAATCAATACGAAACTGAATTTTTGTGCCTAAAATGGTTTGCA	C	G	0.88	0.90	0.87	0.88	0.91	0.68	0.50	0.91	0.86	0.92	0.93	0.000	0.084	0.001	0.032	0.0039			
rs7531416	1	14.11	TATTCTGGGTTCTGATGAGATGTGATCTGAGTGTGGTTCAGTGATTTATTGAAGAAT[A/G]GTGGCTTTCCGTGTTTACTCAGCTATCTGCCCTAAAACACTTGCTCAGCAGCATGACGACA	A	G	0.64	0.48	0.44	0.39	0.35	0.27	0.08	0.15	0.50	0.51	0.45	0.009	0.137	0.069	0.031	0.0000			
rs3927648	1	14.23	GGTGGTAGTTCCCTTCAATATCTCCAGCCAACCACGTATCTTGAACACTTATGTTC[A/G]TGCCACTGCCCCTAGGATGTAGATTATATGTTGAGTCACATGTTGTGATAAAGG	A	G	0.33	0.46	0.47	0.45	0.66	0.55	0.14	0.53	0.43	0.48	0.49	0.007	0.077	0.000	0.002	0.0000			
rs1883661	1	14.37	TAATATAATTCGTGTGTTTGACCTACGC[A/G]TCTTCTTTGAGACAGGCTGTAACTCTCAAATTCGAGCAACTGAGTTTTAATTACGACCTTTATTGTTTCCTTGCACTTCTGGAGC	A	G	0.60	0.48	0.44	0.46	0.48	0.36	0.79	0.39	0.50	0.49	0.47	0.014	0.080	0.002	0.001	0.0001	1		
rs4357518	1	14.44	GGACACTTGGCTAGGACTTGGAGGTTCTTTCCCTAACCAGCCTGTGACCAATCGA[A/C]GTAGGCTGTGTACATTTCAGAGAGGGAGATCCATGTGGGCTCCAGCCACGGGGA	A	C	0.35	0.54	0.50	0.55	0.52	0.05	0.02	0.23	0.54	0.53	0.53	0.009	0.169	0.034	0.129	0.0000			
rs3845596	1	14.55	CACTAAGTTCTTCATATATATTCGGGATGTGCTCAGCTTTGCAAACCAGGACACAG[A/G]ACCAATTTCACCAGTTATTATCACTGGCACAATTCATGGGCCTTATTAGCTATCG	A	G	0.29	0.56	0.55	0.62	0.61	0.86	0.85	0.63	0.61	0.54	0.59	0.031	0.039	0.001	0.049	0.0019			
rs6429691	1	14.62	CCCACTATGGCGTGCCACCAGCCTTGGCAGAAGGATAAAGTTCAGTCCATCACA[C/G]TGGACCACAACACAGGGTGGAGGGCAGAGGGTGCCCAGGAGCACAGGTTGAGACTC	C	G	0.62	0.73	0.76	0.78	0.82	0.64	0.79	0.63	0.85	0.79	0.70	0.005	0.005	0.000	0.001	0.0000			
rs2871890	1	14.71	GGTTAAGTAGAATTGCCCTTGGTTCCATGGAGTATGCTGGTAATTGGCACAGC[A/G]CTGACCAGAATAGTTCTCAAACATCGACAGGCCCTGATCAGGCCACTACTGGCATCTGCCC	A	G	0.45	0.36	0.32	0.40	0.32	0.32	0.31	0.28	0.18	0.43	0.38	0.003	0.005	0.009	0.003	0.0007			
rs7515649	1	14.80	CTCCAGGAGCCATGAATCAAACAAAACACAGGAAAAAGCCTTTCTCCTGAACTTATC[A/C]CGCTTCTGCTGAACTTCCTTCTGGAGCCACAGTGCCTACCTTCAGGAGTTTTTTCTGA	A	C	0.35	0.24	0.20	0.22	0.13	0.14	0.03	0.16	0.25	0.22	0.18	0.010	0.039	0.002	0.006	0.0000			
rs488595	1	15.05	GATACATTCTTTGCCCGAGACACTCACACATGCCTGCTCTTCCAAGGACCCT[A/G]CCTCTAAGAGGCTGCCTTCCAGATTAGGCTCTTGTCTTGCTTTCTCTCCCAAACTATTGT	A	G	0.24	0.41	0.47	0.52	0.54	0.14	0.53	0.55	0.43	0.37	0.40	0.040	0.000	0.001	0.085	0.0008	1		
rs1007150	1	16.00	CCTTGGCATGGAAACTCTACCTGATCATTTTTGGTGTTGGTCTCAAGGAT[A/G]GGTTACTGGGACTCAGGTCCTTAAGTTTAATGTGCCATGTAAATGATAATAGTAATATGCTT	A	G	0.48	0.74	0.75	0.80	0.83	0.64	0.33	0.63	0.75	0.82	0.75	0.044	0.131	0.030	0.021	0.0000	1		
rs761423	1	16.68	CACCCGCATCATTATCTGGCCACCTAGCCACCCACCTGATGTTTATTCAACAC[A/G]TATTATCCACCAGGCCCACTTCCAGGACACCCTGAGCTCCCCTCCTTGCTACTTTTCA	A	G	0.64	0.62	0.55	0.55	0.48	0.77	0.49	0.54	0.71	0.58	0.54	0.004	0.005	0.002	0.017	0.0000	1		
rs1005753	1	16.83	GGAGGGGTCAGTGCTGGGGGCAGGGAGCATGCAGGACTCTGAGGTGGGA[A/C]CTGTGCTGACACCGGATGAGTCCCATCTCCTCTGCCTCAGGCTGAACTCACACGG	A	C	0.33	0.64	0.59	0.63	0.70	0.82	0.80	0.64	0.71	0.53	0.56	0.024	0.041	0.001	0.043	0.0005			
rs3766306	1	17.35	AAGAAAAAAAATACTGGCAAAATAAAAGAAACAAATTATTCAAGCCCCAGGGGAAC[A/C]CAGGCATTCAAATAAGATTTCAAATGACTCTTCTTCCATTAAAACTTTTGCTGCAGT	A	C	0.07	0.16	0.15	0.20	0.18	0.14	0.00	0.14	0.18	0.07	0.16	0.006	0.138	0.015	0.046	0.0000	1		
rs1578215	1	17.50	TGCCCAAGCCTGTCCTGAGTGGTGTCTTCTGACTTCTTGCACTGTCGAGGGGAC[A/G]ATAACCACACAAATGACAACAGATGCCGGCCTGGGAATGGAACAGCATTTCCTAG	A	G	0.10	0.13	0.08	0.11	0.11	0.14	0.36	0.00	0.18	0.06	0.08	0.001	0.039	0.049	0.000	0.0005			
rs477558	1	17.60	GGAGAGGGAACAGGGATCTTAGGGCCGTGGAAGTATGACATTTGTCCCTTTGCC[A/G]CTTCTATAGCGATAATTGGCACACAGGAGCAAGCTTCCAGGGGGAACCAACAGGG	A	G	0.45	0.55	0.56	0.63	0.48	0.73	0.98	0.58	0.36	0.55	0.53	0.006	0.163	0.001	0.017	0.0009			
rs223201	1	18.06	CCCAGCATCTCATTCCTTCCCGGTACTGCACCCCACTTGCCCCTTCATTACAC[A/G]CCCATGGTTAGAATCACTTCTTTGCAGAATGTGGAATGATGTATTCATCTACATTTTG	A	G	0.33	0.17	0.17	0.22	0.16	0.05	0.04	0.13	0.19	0.11	0.15	0.010	0.048	0.006	0.034	0.0019	1		
rs872355	1	18.25	GCCTGGGGCTCTAGAGCTGGGCTTCAGGAAGAATGTGGTCAGGGCCATCAGG[A/G]TGGAACAAGGTAGCTCAAGACCTTGGAAAGACACCCTGAGGGCACAATCGTCCTTG	A	G	0.23	0.30	0.33	0.41	0.33	0.18	0.29	0.21	0.43	0.43	0.33	0.003	0.001	0.003	0.007	0.0000			
rs766325	1	18.39	GGCGTCCCCTCAAACCCACCAGCAAGGAGCTCAGAGTTG[A/G]TGAGTTCAGAGCTGTATCAAGTCTTGACGGGCTCCCTCCCTCCCCATTTCCCACCTCTTTGGGCCCCCAGA	A	G	0.50	0.49	0.52	0.52	0.57	0.18	0.42	0.48	0.64	0.48	0.56	0.003	0.000	0.001	0.039	0.0020			
rs485874	1	18.51	CTGAAGTTCCATGCTTTTAAGAGCTGGGACCTTGGGAGGATGATTCAAACCCTC[A/G]ATTCTCCTCCCTGGGAACTTTTTACCACCTTTACCTATTTATCAAAATCATATTCAT	A	G	0.33	0.48	0.43	0.54	0.37	0.59	0.61	0.38	0.46	0.34	0.39	0.001	0.023	0.000	0.021	0.0000			
rs942182	1	18.95	GCAAACCAGCAGCTACCTAGCACAAAGAAGCCAAGCACAATGCTTAAGAATAT[A/G]CCACTGTAGGAGAACTTAAGGAAACTCTAGACTCTGCTACAGAGGCAGCAGACAGC	A	G	0.10	0.15	0.18	0.18	0.11	0.00	0.17	0.01	0.11	0.15	0.17	0.012	0.001	0.072	0.072	0.0005	1		
rs214348	1	19.15	GTCTCAAGCTGAGCCCTTCATTTCCGGCTAGCGCTTTGTAGGAGGTTGCTCC[A/G]AAAGACCAACTGGAGCCTCGGTCTGCCAGGCTCAGGAGGGATCAGGGTGTAGGGAG	A	G	0.19	0.38	0.36	0.34	0.36	0.64	0.52	0.27	0.43	0.38	0.32	0.037	0.003	0.015	0.018	0.0030			
rs214346	1	19.15	CAAGGTTCTAAACCCACCCAGCAACTTGCTATGGGGACATCTAAGGTAGAAGGC[A/G]GCGTGGGTTCAGGGGAGGCAGTACCTTCCAGTCCAAGTCCAAGTTTGCCTAGA	A	G	0.45	0.43	0.41	0.34	0.39	0.64	0.52	0.27	0.43	0.42	0.36	0.000	0.001	0.020	0.013	0.0028			
rs1266438	1	19.57	CACAGCTCCCGTCCAACTTCTTCCCACCAGCTCCTCTCTGCCAGGGCTTCCCT[A/C]TTATTGCATAAGTGGCTTCTTCTGTTCTCTAAACTGGGGAGACGCTTTTATGCAGTGA	A	C	0.69	0.48	0.49	0.56	0.46	0.86	0.49	0.56	0.57	0.42	0.44	0.025	0.001	0.009	0.100	0.0010	1		
rs951805	1	20.53	GCTTTGGGCAACACTTCACCACAGCCATCTGCCAGCCTTGAGTCCAGTGACAAGA[A/G]GGAACAAGATGGTTTACAAGTCACCCTCATTCTCTGCAAATCCAGTTTATTAAACT	A	G	0.17	0.34	0.38	0.38	0.55	0.00	0.24	0.34	0.25	0.36	0.37	0.022	0.006	0.000	0.138	0.0002	1		
rs213006	1	21.15	GGAAGGAGGCTCTGAAAACGTTAATAACGTTTCCCTCTTCCCAAAATGGACATA[A/C]ATCTGTCAATTCAGCTAAATACAGAATCACATCAAAGAGTTTCCAATGGTTAATATC	A	C	0.62	0.65	0.65	0.67	0.77	0.73	0.68	0.61	0.69	0.65	0.67	0.001	0.008	0.002	0.017	0.0015	1		
rs861212	1	21.40	TGTTTATGTGTCATCTCAACTTCCCTTCCTTATCTCCTCCACACTGGGAGTTT[A/C]CAGGGGCCTGAGTTCCAGGAATTTTTGCTGACAAAGCTGATCAATGCTGAATATGCCTG	A	C	0.23	0.47	0.53	0.56	0.50	0.82	0.74	0.59	0.43	0.43	0.43	0.036	0.036	0.012	0.071	0.0041			1[g]
rs4654786	1	21.98	TCGAGTGGGTGAAAGAATGAGGATTCCACCTCGTAGCACTGAGCAGTCGCAGTGG[A/G]CCAGTGTGACTTAGAGAAAGCACTGTGGCAATTTGTGAAGGAAGAACTGGAGGG	A	G	0.43	0.36	0.34	0.35	0.29	0.45	0.54	0.49	0.32	0.38	0.32	0.004	0.021	0.022	0.009	0.0000	1		
rs2473237	1	22.06	TCCCTGTGAGTGGCATGGAGAATGTAAAAGTCAGGGCTGGAGACAGGCAG[A/G]AGTGGTCAGTGACCTGGTTTTAGGGTTTGCTACCCCACATCCCCCTCATCTCCCT	A	G	0.08	0.21	0.22	0.20	0.18	0.27	0.25	0.24	0.29	0.20	0.18	0.023	0.001	0.000	0.001	0.0019			
rs6426747	1	22.11	GTCAAGTACCTCCCCACAGTCTCACAGGAAAAGGGTGGGGCTGGGATTTGGA[A/T]CCTGGTTTCTCTGATTCTAGGAGCTGAAGTCTTGCAGGGATCCAGGCAGCTCCTGG	A	T	0.19	0.20	0.18	0.28	0.23	0.00	0.00	0.05	0.29	0.26	0.33	0.001	0.062	0.008	0.062	0.0156		1	1[g]
rs116119	1	22.69	CCTTTAATAGAATTATGCCTTTTGCCATGTTACTTTCAACACTGCCCAACAAC[A/G]TATGTCTGTCTTTGCATTATGGATCCTGCTGGGAACTCACTGAACCTGTTTTTCA	A	G	0.31	0.52	0.51	0.46	0.46	0.27	0.68	0.42	0.46	0.48	0.48	0.015	0.021	0.001	0.028	0.0002	1		
rs1555024	1	23.26	CACCAAAGACATAATGTGTGGAGGTTGCCTCTGAAGAGGTAGCATCTGGAAA[A/G]TTGCCAGAAACCAGCACCACTTCTCCTTTCTGCCTTGCTGTGCGGTGTGGGGCTTG	A	G	0.29	0.66	0.68	0.65	0.73	0.45	0.78	0.49	0.75	0.64	0.69	0.072	0.005	0.026	0.025	0.0001	1		
rs874515	1	23.35	AGATGAGAGATTCAGGACCATCAGGACAGACAACCATATTGCTCCATTCCCCA[C/G]CCCACATACACACCTGACTCTCTTCATCAGGGCCAGGGGCCACCTGGTCTTACAGTGG	C	G	0.88	0.74	0.67	0.76	0.63	0.73	0.78	0.82	0.64	0.63	0.63	0.038	0.012	0.020	0.002	0.0011			
rs4237	1	23.58	CTAGGGCGCTGTGGAAATTGGGTCTTGGGCTGGGTGGCATCTGGCAGTCATGG[A/G]TAACACTTGCTTTTCCAGTTAATGTGGCCATGTGATTCCAAGTGTCATGTTGCTTTG	A	G	0.65	0.54	0.58	0.67	0.46	0.59	0.70	0.75	0.68	0.66	0.54	0.009	0.021	0.029	0.004	0.0001			
rs196395	1	24.18	ACTAGAATCCTAATCTCTAAAGGAGATGATCAGATTTTGCACGGAAGCTAGA[A/C]GAATCAACCTGGAACAGATCACTCCTGCCCTACCTGTCCAGGCGGCGGACAGAAGCAG	A	C	0.21	0.49	0.54	0.53	0.45	0.27	0.68	0.51	0.46	0.58	0.51	0.068	0.010	0.000	0.037	0.0004	1		
rs742230	1	24.60	ACAGCAATGACCTGGTTTTCCTCCCTTCCTTTTCTGGTTCACACCTTCAGCCCT[A/G]TTTGTTAAGAGCTCTGTGGTGTACTGGGTGCATGTCTTTCATGGAAAGCCATCTTCC	A	G	0.45	0.46	0.57	0.63	0.50	0.68	0.60	0.60	0.50	0.47	0.62	0.006	0.001	0.003	0.009	0.0009			
rs718391	1	25.73	GGGGGCTTTGAAAGAATGGAAGAGAGCCCCAAGGAACCAAGGTCCTGGGGATTGA[C/G]AGGCATGAAAATATGTCCCTCAACACCACCCACCCTCTATTTCCAAGGCCCCTCTC	C	G	0.52	0.54	0.45	0.48	0.48	0.27	0.14	0.29	0.32	0.42	0.46	0.004	0.064	0.020	0.022	0.0003	1		
rs1466286	1	26.48	AGGTGAGTGTGAGCTTATCAAGTGGAGACAGAGATTGACACCACACAGACAC[C/G]CAGGGGATTCAGCAGAGGAGAGAGAGCAGCGGGGCCCAAGCAGAGC	C	G	0.24	0.31	0.27	0.20	0.24	0.86	0.45	0.52	0.18	0.29	0.26	0.003	0.014	0.029	0.168	0.0001	1		
rs6565	1	27.82	TAATAAATGATACCGTTCTCATTCATAGAGAAGCTCAGGTGTCATTTTTATTCT[A/G]TGGTTTTAAAATGAAATAATCTTGGTAGTCACTTACAACTTTGACGACTTTAACCACCT	A	G	0.40	0.59	0.65	0.68	0.55	0.50	0.38	0.71	0.75	0.59	0.65	0.021	0.021	0.006	0.003	0.0001	1		
rs6564	1	27.82	ATTTCACCACCAGCCTTTTGGGGCCCAAGGTCCTAAGGTTGTGTTCTTGAAGCCAA[A/G]TTTCCCACATTAATCCATGTTAACCACATGTGGATTAAACCCCTGTTTACATATACA	A	G	0.60	0.41	0.35	0.32	0.45	0.50	0.63	0.29	0.25	0.41	0.35	0.019	0.020	0.006	0.003	0.0001			
rs8559	1	28.17	CTTTCACTAGTTGAGGAGTAGAAGAGGATGACCAGCTAGCCTCCATGGAATTGG[A/G]ACTCCTATTCCTTGCTTAGACATTACGGGTTATGCTTTGAGATCTCTTTGGGGTGA	A	G	0.43	0.58	0.62	0.63	0.55	0.91	0.78	0.74	0.61	0.66	0.74	0.010	0.024	0.004	0.068	0.0086		1	1
rs204057	1	28.96	ATTTTTAAAATGAGCTATATCACCAACCCCAAATAGTGTTAAATGTACTTTTATACTGCAACCATAGCATCA[A/C]CCTAGGGAAAAAAGGAGTTTCATAACTCTAAAGGAAGG	A	C	0.50	0.16	0.22	0.26	0.20	0.86	0.61	0.49	0.36	0.24	0.23	0.063	0.160	0.050	0.294	0.0003	1		
rs2007350	1	29.33	ACAAAGAGCCCCCCTGGTCCCCAGCGATCTCTCCAGGACTCTCTGAAGGCTC[A/C]TGATTAACAACTCCTCAAGGCCAGGCCCAGCCAGATTCCAAAACTCAGGCCTGG	A	C	0.68	0.33	0.42	0.35	0.43	0.68	0.69	0.50	0.32	0.45	0.45	0.026	0.027	0.003	0.020	0.0000			
rs1886651	1	29.39	GATCATGGAGGCAGCATGTTGACACAGTTGGGACAAAGAACCC[A/G]GATAATGTGGATGTGGAACATCAGTGGAGCAGCAGCGTTTGCGAGAATCACCTGATTTTCAGT	A	G	0.26	0.52	0.52	0.41	0.44	0.41	0.16	0.43	0.46	0.54	0.49	0.063	0.116	0.033	0.016	0.0038			
rs722497	1	29.67	GCCATCCAGCCCCCACCACAGGCCCCGGATAACCAACAGGATGAGGACTGA[A/G]TCAGAAACGCGGGAGGCAAGGAATGATGTATTAGCCCTCAGCGGAGCAC	A	G	0.67	0.33	0.33	0.39	0.41	0.27	0.06	0.27	0.46	0.37	0.44	0.016	0.126	0.042	0.031	0.0007	1		
rs895786	1	29.81	ACTCTAGTTTCTAATCCTTTTTGCAATTTGAAAAGTGGTTTATTTGGTCCCTGCA[A/G]TGATGGAGTACACTATTAGCTCTGCTTTGCTTTCCCCTATCTTAACGTGGAGGATGT	A	G	0.19	0.26	0.31	0.27	0.18	0.45	0.03	0.31	0.32	0.43	0.32	0.003	0.059	0.003	0.018	0.0006			
rs1015099	1	31.00	GGTCCTCATTTATACCATTTATTAGATACATTTGTTCTTTGATTCAGCAAAT[A/G]TTTGATCCCCAGGCACTGTGCTAGGTTCTGAGGTCAAAATTAGGATAATTACTCTTCTGAC	A	G	0.36	0.17	0.17	0.16	0.11	0.50	0.06	0.33	0.21	0.15	0.13	0.047	0.002	0.062	0.107	0.0003	1		
rs1039630	1	31.38	CAAAGGTCTCCAGATGCTTTCCTTACAGATGTCCAGGGTCTTCTGTGAGTG[A/G]TATGTGGGCAGTTTGTGTGTGGAGGGGGGGGTGTTTAGGTATATGTGCACAG	A	G	0.60	0.64	0.60	0.62	0.50	0.45	0.51	0.27	0.61	0.58	0.58	0.000	0.001	0.050	0.007	0.0001			
rs1320580	1	31.48	AACAGGATCTTCTCCCAGCTCTGCAGCAGTTGTCTCCTAATGTGATGGGTT[C/G]GTGTCCGGGCCACACTGCTTTGTTTGAAATATCTTGACTGTTTCTCCCGGCCAGTGAACGTC	C	G	0.76	0.55	0.57	0.57	0.43	0.32	0.27	0.43	0.29	0.64	0.59	0.027	0.044	0.010	0.025	0.0002			
rs726344	1	32.84	CAGAGGTAAAAGGCCTGTGGAAGTGTCCAGCCCAAGAAGCTGAACCTCTTCT[A/G]AGGGAGGAGCGAAGGCAAGGACTCTATACCCTCTATAAGGCAGCTAACAATATGCTG	A	G	0.24	0.12	0.11	0.09	0.14	0.00	0.41	0.07	0.11	0.10	0.08	0.033	0.103	0.002	0.018	0.0006	1		
rs760594	1	32.96	TTTGGCGTGGCATGACCTTGAACCCAAGATACTGGCCATGCCAAGTATTTTGTAAAGTTTGG[A/G]TCCTGTAAAAGACACCAAGTTAGCTGTGGAAGGCCCCTACCC	A	G	0.71	0.71	0.74	0.83	0.75	0.64	0.27	0.64	0.84	0.69	0.74	0.014	0.105	0.018	0.003	0.0007			
rs536766	1	33.94	GATCCTGAGATGGGCCTTATCTGACTTTCAAGCTCATGCTCAAACACTTGCC[A/G]TATTCTCCTCTGCAGTGTGCTTTGTGTCGAGCCCTGTGCTGAGATGAGTAACTCTCAGCC	A	G	0.50	0.57	0.57	0.46	0.58	0.36	0.68	0.39	0.64	0.58	0.56	0.001	0.007	0.010	0.016	0.0004	1		
rs899378	1	34.02	GGTAATTGTTATGAGAAAAATAAAGCAGAGCAACTGGCAGAGTATGACAAGGC[A/G]CAATCAATGGAACCTTTTTGGGAAGTGATACCTCACTTGTTTTCACATGTCAGTCCCT	A	G	0.50	0.58	0.58	0.45	0.64	0.09	0.29	0.32	0.68	0.60	0.62	0.001	0.030	0.008	0.114	0.0019			
rs491603	1	35.96	GACCTCCAGTTTCTACAGATTCTGACAATAAAGAGTCACGTATGCAACATTTTTGC[A/G]GTCTTTCCTTCTTTGGTTCTAAAAGACTAATTAAAGGACTAACTTAAGAGATTTTTG	A	G	0.50	0.22	0.15	0.13	0.18	0.68	0.82	0.32	0.29	0.24	0.16	0.078	0.239	0.027	0.146	0.0004	1		
rs1331331	1	37.23	CCGAGCTGGGGAATCAGTCACCCTTCCTTGGTGAGGCAAACATTCAGGGAAA[A/G]GTGAAGGAGGAGGTCCTGGGAAGGGGAAGTGGCAGGAAGTAAAACAAATGGGAGC	A	G	0.31	0.29	0.33	0.37	0.36	0.05	0.03	0.46	0.27	0.38	0.38	0.003	0.121	0.002	0.100	0.0006	1		
rs6619	1	37.46	TGGCTGCCAGAGGAACACAGTAAATAACTCCCA[A/G]GTGTCTTGTTGGAAATTAATCATGTGGAAATTATCACTTCTTTGGTGCAATAATGCCTTCCAGTTGGGCCTTCAGCTG	A	G	0.71	0.53	0.55	0.56	0.68	0.64	0.22	0.71	0.61	0.61	0.69	0.015	0.072	0.010	0.003	0.0084		1	1
rs589927	1	37.60	TACTCTTGGTAGAAGTAAAAATTGATACTGCTTTTTTGGAGGCTGGCATAAGC[A/G]AGGATATTTCTGCAGCATAAGTTAATACCTTCCAAGGAAACCTAGTTGACCAAATAGGA	A	G	0.52	0.52	0.44	0.48	0.48	0.77	0.59	0.50	0.54	0.46	0.45	0.015	0.029	0.019	0.105	0.0018			
rs649593	1	37.64	TGCTTCATCACTCCTCTCGCCTTTGTGCTCAGGCCTTTTCTGAGATTTGGCTGAGAAGCCAGCT	A	G	0.24	0.35	0.33	0.38	0.39	0.68	0.39	0.40	0.18	0.43	0.38	0.008	0.000	0.006	0.064	0.0033			
rs999918	1	38.89	TAAGTGGCTCCTGGCTGGGAAGTAGAGTAGGTTTCTCTAAGGAAAATGACACCTGCTCACCACCC[C/G]CCCCACTATACCACATTGTTGTTAGGCTATCATATAATGATAAGGT	C	G	0.21	0.39	0.46	0.39	0.43	0.05	0.02	0.14	0.39	0.34	0.44	0.028	0.144	0.070	0.121	0.0005	1		
[illegible]																								
rs580928	1	76.13	ACATTTGTTGAGGGTATTTTTCCCGCTATGCCCAGGGCTAGGCACTGGACAT[A/T]CCGAGACATACTGTGTCCAGAAGCTCAACAATTTAGCTGAGGAGATGGATGATGCAGC	A	T	0.52	0.34	0.38	0.46	0.48	0.00	0.08	0.24	0.21	0.38	0.40	0.015	0.053	0.008	0.140	0.0021	1		
rs1389790	1	77.51	CAAGATTTTCTGTGGTGTGCTGTGATTGAATTTTCACATGTCTACTAATCTTGGGA[A/G]TTGGATTATAGACATTGTACATATGTTGCTGAGGGTCAGGATTTTGTTTTCTTTCCT	A	G	0.71	0.29	0.30	0.33	0.29	0.32	0.01	0.65	0.46	0.41	0.38	0.102	0.089	0.068	0.001	0.0090	1	1	1
rs720887	1	78.19	GAAAGAAGAGAATGTGCCCTAAGAAAGTTTGGGAAACAATTCTACTACAATGAAGA[A/C]CATTGTCTCATTTGATAGAGAGTCCTGAGAGCTGCTATCTACATAAATAACCCCAC	A	C	0.36	0.50	0.58	0.55	0.59	0.68	0.35	0.53	0.75	0.49	0.55	0.019	0.016	0.000	0.015	0.0001	1		
rs1489396	1	78.82	TAAATTATTTATAACAAGTAGAGTGAACATTCTTAATGTGTTTGGACAATGTAAT[A/G]TATATGTTAAAAATCTGACTCAAGTTTTAATTTTTAATTTCACTATGGCACTCATGGTT	A	G	0.48	0.65	0.64	0.65	0.70	0.77	0.78	0.52	0.61	0.67	0.62	0.005	0.027	0.000	0.020	0.0000	1		
rs870855	1	79.43	TGGCCTTCTTACCATCTAAAGATAGCCTTTCATGCTATTGGACAAATCACTTAA[A/C]CTCTTTAGATCTTGGACTCCTCATCCTTAAAAGGAGTCCTAAGTAAATCTATTATCTCA	A	C	0.36	0.57	0.59	0.59	0.46	0.55	0.48	0.48	0.64	0.55	0.62	0.017	0.003	0.013	0.000	0.0002	1		
rs1687835	1	80.20	AGTTATCATGGAGGCTTTGTAAAGGTTAGGAGGAAATCTTTCCTAGGATACA[C/G]CTTGTGCATTCCTTCCTGGAGATGGGGTAAGGACATAGTTACAATACCAGTGGATGGG	C	G	0.67	0.55	0.52	0.46	0.50	0.73	0.53	0.68	0.68	0.65	0.59	0.014	0.000	0.014	0.020	0.0085	1	1	1
rs1536570	1	81.13	CTTTAGTCTTCAGTTCATTTTCTTTCCATATTCCAGCTCATATGCAAAAATATC[A/G]ATGGCAAGAATTCTATATAGCTATACTGCCATATTTAGATTTCAAAATCTTGTCAATTCTA	A	G	0.69	0.44	0.47	0.39	0.59	0.95	0.69	0.50	0.64	0.47	0.41	0.030	0.021	0.003	0.153	0.0005	1		
rs654654	1	81.79	AGTATGAGTATATGGGTCAGTCTTGTGACTTTGGGCAAAGTCATTTTACTTCTCT[A/G]GGTTTTAATTAAAACCTTTTCTGAAGAATTAAAGCCATTTCAAAGTCTTATAAATTTTC	A	G	0.57	0.49	0.56	0.52	0.48	0.27	0.01	0.43	0.68	0.68	0.59	0.001	0.202	0.003	0.034	0.0012	1		
rs400382	1	81.79	GAGTGAAAAAGTTGGACTATCAGTGACAACTTATCTTTTAGGTTTACTTTTAACACT[A/G]TTTTCATCTAAGTTAAGATGAAATTGAAATAAGCTTTCTGGATGCTTCTAAATTTCA	A	G	0.40	0.51	0.44	0.48	0.52	0.73	0.99	0.57	0.32	0.32	0.41	0.001	0.217	0.005	0.041	0.0007			
rs1492259	1	81.81	GAATACTTGTATGAAAATTCCTGAGGGGAGTTGGGATATAATTTGACCATGAGGGT[A/G]TACTCTCTATAAGTAGATGATAAAGTGAATTCCTTCTATGTGGGAAGGAAGGACAT	A	G	0.81	0.70	0.70	0.65	0.71	0.86	1.00	0.85	0.61	0.65	0.70	0.020	0.140	0.033	0.032	0.0006			
rs785605	1	82.15	AATTGGAATGTCTTTTTAGAACGGTATGATCTCCCTCTTTCTGAGAAAGGTG[A/G]GAATATTCAGACGTACATCGTAATATGGCCTCTTTGATCAGAGTCAACGGGTCTAGGGG	A	G	0.55	0.59	0.64	0.59	0.70	0.77	0.43	0.55	0.57	0.66	0.72	0.000	0.006	0.000	0.031	0.0071		1	1
rs1887251	1	82.38	ACTCAGTGCTGTATATTTGGAAAAGGGAGGAAGGCCAGAGGCTCCCACACAGAAACATCT[A/G]TGTTGGTGCCCATTTTCTCAGATCTATTTGGAGTAATTTTTTTCATACTCT	A	G	0.19	0.31	0.39	0.43	0.32	0.59	0.38	0.39	0.43	0.32	0.33	0.011	0.004	0.005	0.036	0.0025	1		
rs437749	1	82.68	CTCACTTCTTTTGGACTTCCTAAAGTGACCGCCATGTAAGATATTTGTGTACT[A/G]TAAAGATTCAGTGGGTGCCTATTTCTCCCATCCCCTCTTTGTTTTGGTTGATACTCT	A	G	0.50	0.35	0.37	0.45	0.38	0.05	0.08	0.41	0.32	0.49	0.49	0.002	0.082	0.000	0.123	0.0037			
rs1871924	1	83.82	TCCAGCAGTTCCCTTTAAGCTCCTTGATCTGGGTTTACACACCTTCTGTGGCCGCTTAAATATCATGTTTTGGAAACGTAATCAGGACATAC[A/T]GCTGTAGAGATGTAGTC	A	T	0.65	0.37	0.38	0.50	0.39	0.73	0.68	0.63	0.61	0.49	0.37	0.046	0.072	0.043	0.084	0.0005	1		
rs315537	1	84.24	TTAGATTCCACTGAAAAGTGGCCAGTTTGTGACCTCACCTTTAAATCAATTCA[A/T]TCAATCTCCTCTGCAGATGTCTGATCAGAGGTTCAAATGTGGGGGCGGCGGGGAGTAC	A	T	0.18	0.37	0.34	0.45	0.39	0.14	0.21	0.29	0.50	0.39	0.38	0.024	0.009	0.002	0.031	0.0015			
rs912073	1	86.15	TCACAATACGAATTCCGGGCAGTAGACTCTGAAATTGAACTCCTTTCTCTTTAA[A/G]TTGTTTTTATTGTCTTCAGCCAGATAGCGGTTGAAAGGAAATGGTAACCAACAAAT	A	G	0.05	0.36	0.28	0.24	0.29	0.15	0.78	0.14	0.29	0.24	0.30	0.037	0.160	0.003	0.005	0.0012	1		
rs2075630	1	86.38	ACCAAGGAGCAATATTCCATATTTACTTGGAGACTGTTTAGCCACATGAAACAGA[A/G]TAGGAGGTTGAAAGGGGCTGGCAGGCATAAGGTCTCAGAACCTTGCAGTGTCTTA	A	G	0.60	0.28	0.41	0.41	0.30	0.23	0.01	0.27	0.25	0.40	0.42	0.042	0.116	0.001	0.009	0.0011			
rs305404	1	87.70	AAGCATGAGCAATTCAATAAACTAGAATGAGACTAATAGTTATAGATATGCCAAA[A/C]TCCAACCTCACATATACTGTTCTTGGTGGATTAAAGGCATAAATGTGGAAAGAAAGA	A	C	0.12	0.40	0.40	0.40	0.46	0.09	0.33	0.43	0.50	0.46	0.47	0.041	0.000	0.003	0.047	0.0038	1		
rs1413533	1	87.81	GTGGAAGATTAAAAAAAAAAAAAAAAGGAAAACCAAAATATCACAAAAGGTAAAAC[A/G]CTCGACACACTAAATGCCCTTCAATGTTAACACTGGCATTGTGGAATAACCTACCAG	A	G	0.14	0.47	0.49	0.43	0.52	0.40	0.42	0.32	0.39	0.51	0.53	0.080	0.004	0.004	0.003	0.0003			
rs1334334	1	87.85	AGGGAGAGGGCAATGGGAATAGTTTAGGTTATAATACCACAGAGCTTACTGTTCTT[A/G]CTGTGATCTAACAGTTGTTCTTCAATAAAGACTGCTAGAGTTTTTGCAAGCCTTGA	A	G	0.12	0.18	0.19	0.26	0.25	0.36	0.43	0.27	0.36	0.20	0.22	0.002	0.048	0.010	0.031	0.0003			
rs1334339	1	87.88	CAAGTCAGACCGAAAGTAACTTTAGGAGTGGGCTAATAATAAGAAATGGAATCTCAT[A/G]AAAGACTCTAAAAATTCTCTAATGGAGAAAGATGACTATTGATTTGAGTCTGTGT	A	G	0.67	0.42	0.40	0.49	0.36	0.23	0.20	0.52	0.36	0.43	0.43	0.024	0.037	0.005	0.025	0.0005			
rs1413527	1	87.90	CTCAATCACTACTTTTTTTTAGTTTTGAAAGTATTGCGATAATACTGCAAATATGCC[A/G]AATGTATGAAAAAAATACTAAGCTGCTTTCAAAGAAAACATAAAATGTCATCATTTA	A	G	0.25	0.46	0.50	0.49	0.43	0.55	0.47	0.39	0.50	0.49	0.47	0.031	0.001	0.008	0.001	0.0015			
rs1380416	1	89.42	TATGGGCTGTTTGAATTGGAAGAAATCTGTTCTATTATTAAGAACATGGAAGAA[A/G]TCTATTATTAAGTACAATCAAGAAGCAAAAAGAACTGCCACTGAGTACCCCTTTTA	A	G	0.38	0.41	0.49	0.44	0.52	0.14	0.53	0.57	0.57	0.50	0.51	0.006	0.004	0.011	0.058	0.0005	1		
rs922104	1	89.51	TTTTTCCCCCAAATCTCCCCAGGAGGAGAAAGCGTACAAGATAGACAATAAACT[A/G]TCTGAGAAAAGTGCCTGTCTTTTAAGTCTTCTTGGAAGTTGCACTAGACTTCTAGGAT	A	G	0.50	0.46	0.56	0.48	0.57	0.14	0.52	0.57	0.61	0.55	0.56	0.002	0.000	0.000	0.085	0.0004			
rs1215512	1	89.55	CTCAAAATGGGCTATACGGTATCTTTTTTTCTCAGTCAACATGGGCTGATGCATA[A/C]CAATTCACCCTTAATGGTCCTGAAATATATTCACTGTAGAGAAATGAAGATCAAAAAC	A	C	0.67	0.38	0.43	0.39	0.39	0.77	0.51	0.34	0.38	0.43	0.45	0.027	0.000	0.001	0.057	0.0001			
rs1215500	1	89.56	AGCTGAATCTAACACCAGTCTTAATTAACCTACAACAAGACAAAAATTTAGGTC[C/G]GGGCTTACCTCCTAGTCAGCAGATGGGAAAATTCCTAGAGAGTCTCTGGGAAAAAG	C	G	0.40	0.53	0.50	0.50	0.54	0.23	0.17	0.56	0.61	0.50	0.49	0.003	0.044	0.003	0.031	0.0000			
rs1343710	1	89.76	TAATATTTGCTAAGGTGTTATGTCTTAGCTGAAAAAACTGCTGTAACAA[A/G]TGTTTGCTGAATAAATGCCATAAATATATTCAATATAAAAAACTATATCTTCAAAGTAAATGAA	A	G	0.95	0.77	0.76	0.68	0.66	1.00	1.00	0.89	0.64	0.74	0.74	0.074	0.132	0.001	0.132	0.0001			
rs1344059	1	89.84	CATCACTTTTAGGCTTAAAAAAATCCCTATTTGACTAAAGAAAATGAGCGGAAC[A/G]ATTCCTCTTTATAAAATGCTAAACAGAACAAGACATCTGGTACTGTACACACTGTAGCC	A	G	0.60	0.57	0.53	0.59	0.61	0.82	1.00	0.67	0.61	0.54	0.58	0.002	0.176	0.011	0.036	0.0002			
rs954145	1	89.97	TTGTGTGAGGAAGGAGAAAGGAACAGTCAGAAGGAAAATTGGAAATAGACAG[A/C]TGGCAGATTTTCTTTGTGTGCTTGTTTTCTAAGTATGAGGAATAAAGCTCCTTGTGG	A	C	0.48	0.54	0.51	0.63	0.48	0.95	0.90	0.53	0.57	0.50	0.60	0.001	0.079	0.000	0.129	0.0085	1	1	1
rs1526480	1	90.68	ATCTGGTATAAGATACAGATAGTTCCTATG[A/G]GCTAGACTTGGTCAGATCTCCACAGAGAAAAGGGAGCTTGCTGGTAGTATTGCCCTTTGGGACTGCACAGATGACAAACAC	A	G	0.64	0.55	0.66	0.67	0.50	0.45	0.33	0.50	0.39	0.65	0.60	0.000	0.039	0.002	0.011	0.0019	1		
rs698274	1	90.71	TGAATCTGTTCCACTCTCTAGCCTGAGTTTTTCTAAGCTGAATAGATGTCTGCT[A/G]GAATCTCCTGAGCACTCACACATATGCAAACTAGAATGCAAATCTAGACATTTGAGA	A	G	0.67	0.55	0.53	0.50	0.46	0.60	0.43	0.64	0.54	0.57	0.53	0.007	0.013	0.000	0.000	0.0001			
rs1017956	1	91.79	TAAGCATATGACTCCCAAGGGTCCTTCCTTCTAATAAGGAGTCCTGGTCATCATT[A/T]TCTCTTGCCAGTATCTCCAAATCCACCTTTGCCCAACAGCCTATTCTCAGCCCAGCA	A	T	0.40	0.43	0.44	0.51	0.50	0.73	0.40	0.40	0.54	0.40	0.39	0.000	0.000	0.001	0.056	0.0006	1		
rs912794	1	92.35	CACCTTGGCAGGAAGCGGTTTATCAGATTAACAACCTGCCTGCTCCCACAGCAGC[A/G]ACCAGTGCTCTAAGGAAACAGAATTGGGCTGGGCTCAGCCTTCTGTGGGGGCCA	A	G	0.40	0.42	0.40	0.39	0.32	0.91	0.63	0.67	0.32	0.49	0.53	0.001	0.026	0.026	0.162	0.0086	1	1	1
rs797668	1	93.17	TGTGACAGATGACCTTTAAGTAAGCAAGCCAATGCATGTGCAGTTGAGCTAA[C/G]GATTGAGCGGCAATAAATGAGCTTGCAAATCTTTCTAATTTACTTACTCCCTAGTCTGG	C	G	0.79	0.34	0.37	0.40	0.32	0.41	0.60	0.30	0.39	0.37	0.35	0.105	0.035	0.001	0.003	0.0003	1		
rs743117	1	93.91	AGCAAGAGTCTCATCTCTCCCAAATTAGACAATGCTATGGAAGTGATCATTTTGC[A/G]TCATAATTTTAGAACATGATGTCAGAGCAAGAGGGCAAGAAACAAGTCTTGGGCTT	A	G	0.57	0.70	0.71	0.68	0.71	1.00	0.56	0.78	0.54	0.76	0.62	0.006	0.005	0.009	0.134	0.0036	1		
rs952499	1	94.03	AATTCACATTAGAGAAGAGATGAAAACACTTTGAACAAAAAGCATTTGCTCTTAT[A/G]AAAGCAGCCTCTTTTGAGAGGCCCTTTGCTCCCCATTTCTCCTTCTCAGAC	A	G	0.58	0.49	0.57	0.50	0.48	0.36	0.55	0.59	0.50	0.53	0.58	0.000	0.002	0.001	0.026	0.0000			
rs16946	1	94.40	TCTTAAAAGGGCTTCTTTTTTTAATGCAGAAAGAGGGGAAAAA[A/G]GAGCGAGCTGTGGTGGACAAGGTGTTTTTCTCAAGGCTCATCAGATTCTGAAAATCATGGTCCCTA	A	G	0.05	0.49	0.51	0.54	0.46	0.27	0.26	0.26	0.12	0.57	0.47	0.186	0.047	0.052	0.044	0.0018			
rs1360699	1	94.77	TGCTCAAAACCCAATCACCAATCATTTTGTGCAATGTATAAGAATGAAACAAAGG[A/G]GAATCCAAATGTTAAAAGATTTCATTGCCCTGAAGAAAAGAAATGACTTCCCAAGCT	A	G	0.63	0.41	0.46	0.39	0.60	0.32	0.48	0.54	0.54	0.44	0.44	0.014	0.001	0.002	0.007	0.0005	1		
rs1573065	1	95.79	TTCTGGTTGTCTTCCAATGCCTTTCCCAAGCCAAACCCTAGTAAGGCAATCGATG[A/C]AATCAGCAGAGTTCAGTCTCCCAGGCCACTGAGCAGAGAACAGAGAATATGTGAG	A	C	0.12	0.21	0.22	0.33	0.29	0.05	0.07	0.22	0.21	0.23	0.24	0.003	0.007	0.002	0.019	0.0040	1		1
rs1974203	1	97.75	CCTGATCCCAGTCTTGAGGATGCCCTGAGCCTGTAGCCCTGGTGTGTAAAAAG[A/G]ACGAGGGGTTACAACCTTCTCTGGAATATGCTAGCTAAATAAAACGTTAAATAATAC	A	G	0.67	0.72	0.79	0.73	0.82	1.00	0.98	0.82	0.64	0.82	0.80	0.010	0.031	0.004	0.060	0.0000	1		
rs963852	1	98.09	CTTTATAATGGTCTTATAAACAGTCTGGGATGTATCT[C/G]TGGTCCAAATGTTACCTTTACCTCAAATGTGGAGAATAGCATGCTTTAGAGATGGGACATAAAAATCTATCTCTGT	C	G	0.93	0.74	0.66	0.82	0.75	0.91	1.00	0.82	0.82	0.83	0.80	0.049	0.096	0.002	0.023	0.0100		1	1
rs834984	1	99.59	AACTTCCTGCCACTCCCTATATGGTAACTTAGATTCCAGACTTGCTGTTCTAA[A/G]TAATGTCCCACATTTTTTAGAATTTGTTCACATCAAGCCCTTGACTTGAATGCCCTTTCT	A	G	0.71	0.65	0.62	0.62	0.57	0.77	0.81	0.65	0.75	0.60	0.66	0.000	0.009	0.001	0.005	0.0004	1		
rs1981193	1	99.61	TGCTCACATTTACTAAATAGACATCATCCTCATACATATTATTACAACTCTTCCA[A/G]TCATGCTGGATCACTGTTGAGATTTTCTGTTTAGATCAGTATTTGCAAGGACAAGTGT	A	G	0.57	0.35	0.35	0.35	0.45	0.41	0.75	0.42	0.50	0.41	0.34	0.032	0.125	0.007	0.007	0.0002			
rs508020	1	100.42	TCCTTCCTTATACCAGATTCATTCTTCATGATGACTAAAGAACACATTCACAA[A/G]TGACAAATGAGGAGAACCCAACCTGGGCCTCCATTCTGCCCTTAGGCTCCAGCCCATCT	A	G	0.57	0.57	0.52	0.60	0.48	0.59	0.82	0.56	0.54	0.56	0.52	0.001	0.049	0.000	0.001	0.0008	1		
rs486567	1	101.09	AAGTGAGGAGGGAAAAAACCCTTACAAACCTGGTGCTTTTTTTCTCCTTTGGAA[A/G]TGAAAAGCAACAGGCTATACTCTGTGAAAGAAAGGAAATGGTGCTGACGAGCTTTTC	A	G	0.13	0.28	0.29	0.29	0.38	0.23	0.02	0.32	0.57	0.28	0.27	0.033	0.088	0.000	0.005	0.0022	1		
rs910633	1	101.21	CATTGTAAAGGAATGCTTCAGTACCTGAAATGTAAAGTGCATGAATGCTTACCAA[A/G]ATTTGTCGTATTGTTATACTATTGCCTTATGTTTGTACGGTGCTTTCCAGTTCTGAAA	A	G	0.48	0.50	0.46	0.51	0.48	0.55	0.78	0.34	0.46	0.43	0.44	0.001	0.066	0.003	0.007	0.0002			
rs1445225	1	101.75	ACAACTGACACATAAGTCAATAACTCCAGCAATATTGGATGCCTACTGCTTGACAAGC[A/G]CTAGGCCAGCTGCTTTGCATGTGCTATTTCAAATCTATGCAAGAACTCTGGGTT	A	G	0.31	0.51	0.52	0.46	0.36	0.68	0.36	0.55	0.79	0.51	0.57	0.036	0.030	0.001	0.006	0.0006	1		
rs716581	1	101.80	TTACCTACATGGCTTATTATCAGCAGACTGGCCTAAGAGCAGGAATAATCATC[A/G]TAGCTTTGTAGTCCCCTAATTCTATAAGTTGGAAAAATAGTTAGGGAACTTTTTAAAATA	A	G	0.50	0.49	0.59	0.55	0.57	0.40	0.27	0.26	0.46	0.48	0.57	0.005	0.052	0.051	0.016	0.0000			
rs1517432	1	102.56	TAAAAACATGGTGGTATAGACTGGTAGTGTTCAGCTAAAACTTAATAACCTATAG[A/G]CAGGGGTGCTGACCTCTAGGATGGAGATATATGAGCTGAATCAATAGCTAATATATG	A	G	0.10	0.60	0.53	0.55	0.55	0.23	0.86	0.52	0.82	0.55	0.54	0.114	0.095	0.002	0.031	0.0000	1		
rs977155	1	102.58	GTGACTCGGTTTCATTCACTAAAGAGTGAGCAGAA[A/G]TTATGTATGCAATCTTCTAATCTAGCCCTTAAAAAAGTGAACAAAACTAAAGCCTACCATACTCTTGTTGTTTTCATC	A	G	0.71	0.40	0.46	0.45	0.45	0.73	0.11	0.45	0.18	0.45	0.47	0.037	0.094	0.001	0.028	0.0004			
rs2065188	1	103.95	TCAGATATGGGGATCAGGTAGATCTTGACAGGCTTAATAGAAAAACTGATAGTT[C/G]AGTCAAGTTTGGAAGGATATATAGGAATTCCATATTAGGGAGCAAGGAAGATGTATG	C	G	0.24	0.63	0.45	0.43	0.52	0.18	0.05	0.52	0.57	0.45	0.49	0.025	0.130	0.000	0.050	0.0006	1		
rs1909043	1	104.19	AAAAATTGTAATAAAAAAGTAAGGTGTCCCATTGCATCAATATTACTCAGAAAA[A/G]ACTGAGCAGAATTCAATTTGTGTATGTGTGTATGTGTGTCATATAACAGAGGCGAATTT	A	G	0.57	0.42	0.55	0.53	0.55	0.60	0.08	0.49	0.39	0.48	0.43	0.001	0.111	0.001	0.005	0.0051			1
rs2048839	1	105.30	TTTGAAATAACACAAGTAAACATAA[A/T]ATCCACTGGAATACTATTTTACTACTAGGAATCAGCCACAATTGAAATACTGGAATTGGGAACATTTGGGGATTAAAGAATCCACAGA	A	T	0.69	0.49	0.56	0.51	0.48	0.50	0.43	0.68	0.50	0.54	0.56	0.005	0.018	0.002	0.008	0.0000	1		
rs987243	1	105.31	GAGAATGTGAATATAATTATTTCTCTATGAGCTTAGGTAATGAATCAG[A/C]TGGTGAAATTAGACGCACATGAATCTGAGATCTTAACCAGAATCAAGCTTTATGAATTATTTCAA	A	C	0.53	0.49	0.57	0.51	0.48	0.50	0.44	0.68	0.50	0.54	0.55	0.002	0.015	0.002	0.008	0.0000			

rs1330225	1	106.19	ATCTATTTATTTATTTATTTATTTTCGACGTTCTCCACTAGAATACAAGTTATGCA[A/G]ACAGGAAGTTTGTTGTTTGTTGTTTGAGCTTGTTGTTTAACATTATATTCCAGAGTCT	A	G	0.38	0.72	0.71	0.78	0.63	0.95	0.93	0.74	0.85	0.66	0.58	0.054	0.042	0.000	0.060	0.0090	1	1	1
rs1888181	1	106.21	GAGCCTTTGGTATGTATCATTTTATAAGCTACATCTATGTAAAAGCAATTGAGATT[A/G]TCTTCTCTTGCTTGTAAATCTGAGAACATTGACTCATAGATTATATTAGTGTTCTATT	A	G	0.71	0.57	0.55	0.52	0.54	0.64	0.81	0.52	0.36	0.49	0.58	0.027	0.065	0.000	0.011	0.0008			
rs2031504	1	106.41	TTAGTTATTATAATAAAACAAAAATGGAAATGTGATTGTTTAAAGAAAAAGTAC[A/C]TGATTTACACGACAGTTCATAGCCAGCATTTGTAAGGAAAACAAATTAGATGTCAATGA	A	C	0.62	0.42	0.39	0.41	0.54	0.05	0.07	0.32	0.43	0.48	0.52	0.031	0.062	0.003	0.087	0.0040			
rs1020812	1	107.84	CTGAAGGAACATTCTCAAAGTTTTTTGGAGTTCAGACTCTAAGGGGAGGCAGGA[A/G]AAGATGAGGGGTATTCTAGGAGGAGACAGTCTAGGGCTGGCTGGATGTGGCCTG	A	G	0.63	0.69	0.79	0.74	0.74	0.95	0.96	0.61	0.86	0.76	0.84	0.019	0.038	0.029	0.033	0.0012	1		
rs338491	1	108.79	AGAACCCCAGAAAGCATTTTGAGGTACAGATCACAGTTATGTTTGTATTGGCTTT[C/G]ATGTAGGAAGACAGGAAAGTAAGGCCGGGCATGGGGAAGTGGAAGGAGAGTACT	C	G	0.48	0.74	0.72	0.67	0.64	0.91	0.82	0.78	0.75	0.65	0.72	0.047	0.011	0.005	0.033	0.0000	1		
rs12030	1	108.82	TTCTAATCTATGATTCACTTAACTTCCTACTTTATAAACACTAGGTAAAGTAATCTC[A/G]CAAGCTGCCAAAGAACACATCCCAAGCAGTAATCACAGACGATGAAGCACCTTACA	A	G	0.93	0.74	0.76	0.74	0.79	0.59	0.52	0.77	0.92	0.82	0.77	0.037	0.028	0.000	0.014	0.0006			
rs1887551	1	109.62	AAGCTGCAAAATAACACGGTTACATTATTGTTATGATATGATCCCATTTGTATAT[A/G]TTTTTTACAAAGCACATATAGTGACTTGTAGCTATACGTGCTTGTCTATATACATAGAA	A	G	0.93	0.78	0.76	0.80	0.73	0.77	0.97	0.78	0.75	0.82	0.76	0.022	0.036	0.001	0.000	0.0001	1		
rs924181	1	109.98	GTGTGTTGGGAATTTGGGACCCCTTCTTCACCCTTTTCCCCATGTACAAACT[A/G]GAGGAATGTCCTTGCTGCTCTCTAAATATTAGAGGGATACTGCTTTCCTCAAAGACTTT	A	G	0.26	0.50	0.46	0.46	0.38	0.20	0.00	0.44	0.36	0.49	0.40	0.019	0.188	0.001	0.036	0.0012			
rs575208	1	111.51	CCTTCAAAATGCAGATAAAAGATGCCATTCCTAGTGCTCAGAACAATAGTTGG[A/G]AAATAGTAGATGCTCAATAAACATTTATTGAATGAAAGAAAAAAGGGGTTGACCTTGGC	A	G	0.50	0.59	0.59	0.49	0.54	0.41	0.24	0.54	0.46	0.55	0.63	0.006	0.052	0.001	0.012	0.0000	1		
rs1246194	1	111.63	TGGATGATGTTGAAGTTAAATTCCCCGGTGATTTTTAACAGAGACTCTTTTTAA[A/G]CCAGGTCCTTCCCATCAGCTATACGTCATTGATTATTTGAATAAAAATGCTGGTGTTCT	A	G	0.40	0.28	0.26	0.22	0.34	0.23	0.08	0.20	0.25	0.25	0.24	0.018	0.015	0.000	0.000	0.0013			
rs1036959	1	111.75	GTCCAAAGACCTAACTCTGAATTTTAGTCCTGCTACAACCCTTAGTGTTGTTAC[A/G]AATAAGAATTCTAAAGTCAGAGCCCTAGGTTTTGTTCCTGGCCCTGCCACCTACTAG	A	G	0.07	0.14	0.13	0.07	0.11	0.23	0.40	0.23	0.14	0.17	0.10	0.003	0.031	0.013	0.007	0.0006			
rs1808973	1	111.79	CCACCTGGGGTTGTGCTGGTGTACCTCGAACAAGGGCATCAGAGGCAAGTGGTG[A/G]CATTCTGCTCAACAAATCATCTGCCCTGCAGGAGGAGGGGTGCCCTGGGGCTGC	A	G	0.60	0.47	0.53	0.40	0.65	0.41	0.45	0.49	0.68	0.47	0.52	0.009	0.004	0.001	0.004	0.0001			
rs930548	1	111.81	TCAACAAACATTCCTGGCCTGCTGACCATGTGTTCAGGCCCTTTAGGGTGATGAA[C/G]AGCTTGCCAGGTGTGGTCACTATGCACAGAAAATGAACCAGATGAACTGGATGAC	C	G	0.40	0.51	0.45	0.48	0.30	0.23	0.18	0.46	0.18	0.49	0.39	0.002	0.044	0.000	0.027	0.0046			1
rs954679	1	112.55	ACCCACTCCATTATTTCACCTTGCCCTGTTTTGT[A/G]AAGATGGGCTGGGGTAGCCCCAACCTGGGGTGGGCAGTGTTGATGGAGGGCAATCACTACTGGGGTGAAAGCCAG	A	G	0.48	0.41	0.39	0.40	0.45	0.64	0.55	0.33	0.54	0.33	0.30	0.006	0.013	0.002	0.038	0.0038	1		
rs1935745	1	113.19	CAATAATGGATTTGATATGAAGGCATTTGCCTAGATGCATACGCAGAAAATACA[A/G]TAGTTCTTGTAATATTCCCTTTGGAAGGAACATATGGGTTAACCAATGATGTTAGCAT	A	G	0.62	0.51	0.44	0.45	0.52	0.18	0.49	0.67	0.32	0.48	0.56	0.018	0.001	0.031	0.026	0.0090	1	1	1[g]
rs537478	1	113.98	GGGCATAAATCTTGTTTTGTTCAAAGAGAGAAAGCTTAGTGGGCTCTGCTTAC[A/G]CCCCAGGAAGGGTCTGGGGGACACGCCATTCTCATAGCTTGTGCTGACTTTGACCAG	A	G	0.50	0.51	0.55	0.52	0.46	0.50	0.51	0.63	0.71	0.49	0.60	0.000	0.006	0.009	0.001	0.0021	1		
rs360642	1	114.72	CCCCCATGCACATGATATTTCTATCCATTTGGGACAACCTTAAGGCAGCTTGAA[C/G]GCTTGGGGTGTCCTGAACTACGCAGGTGATTTGAACCTGGGCTGCAAATCTGCCTG	C	G	0.83	0.36	0.42	0.34	0.34	0.50	0.53	0.66	0.46	0.43	0.47	0.072	0.002	0.016	0.000	0.0001	1		
rs360636	1	114.72	CCTCCTCCAACCGAACCAACTCAGATTGAGAAGTACAGTACTAGAAAGCATTACAAC[A/G]AACTTGTAATGAAAGTCAATGTTTAGAACATAACAAGAGGAATACATAAGGCACA	A	G	0.90	0.59	0.55	0.51	0.52	0.50	0.57	0.72	0.54	0.56	0.63	0.062	0.001	0.003	0.007	0.0002			
rs2057127	1	115.11	CACTTGGTGTAGGGATCGGCCACAATGTGAA[C/G]CACACATCATTCAAGACATACACCCCCCTTTCCCCCCTTTATGTGAAAAGACAATCATGCTCAAGTTCTTCCATGATTAT	C	G	0.52	0.60	0.64	0.61	0.64	0.36	0.55	0.50	0.68	0.62	0.68	0.005	0.000	0.015	0.025	0.0011			
rs975351	1	116.18	ATCTACTTTGTTATTGCATTGCAGGAAACTCAGGACTTATTAGAGGGTCACCC[A/G]TGAGTTTGGCTAGTTCACTGTAATAGCATAAGATACTTCTAAGTTTAATTGCTTGAGAG	A	G	0.93	0.61	0.62	0.60	0.57	0.27	0.30	0.70	0.57	0.52	0.46	0.066	0.050	0.004	0.069	0.0087	1	1	1[g]
rs1555793	1	116.90	GACCTTAAACATGTTTGAATAATCAGTGCCTTTTGTGAAGAAAAACAGACATATCT[A/G]CAACTAATATAAGGATTCACTAGGCTGGGTTTAAATTCAAAGTCCAGAAAGATTAAT	A	G	0.64	0.45	0.33	0.35	0.45	0.59	0.76	0.49	0.50	0.30	0.34	0.050	0.099	0.025	0.040	0.0005	1		
rs998532	1	116.96	AAAGAAGGCTGATAAGAAGCAAAGAGAAAAGGGAGATCAGCTTTTCGATCAAAAG[A/G]AAGAACAGAAGCCTGAAATGATGGAGAAAGACCTCTCATCTGGCCTGGTACCAAA	A	G	0.93	0.45	0.42	0.32	0.54	0.23	0.31	0.44	0.64	0.40	0.37	0.157	0.010	0.005	0.031	0.0004			
rs544173	1	117.65	TATCTCCAGTTTCCAGAAAAGCTAAATGCCTGGGGAAGGACTGAGTTTCTTG[A/G]GGGCAGGAGCTGTGTTTCCTATCACAAGGCAGCCAGGGGGACAGTGCATTCAAAGGG	A	G	0.24	0.64	0.61	0.65	0.57	0.18	0.39	0.67	0.54	0.48	0.56	0.068	0.026	0.005	0.107	0.0004	1		
rs1229133	1	118.34	AGTATCTTCAAACATACTTTCTGGTGCCCAAATGAGAGAGTTTTTGCCAATAT[A/G]CCAATGTAAGTTCTGAGCTGTAGATATGCATAATTTCAGTTTATTATGTATTGACGTATG	A	G	0.26	0.63	0.65	0.68	0.68	0.00	0.03	0.53	0.71	0.61	0.62	0.064	0.234	0.004	0.301	0.0000	1		
rs1625797	1	118.76	ATCTTTCTTAATATTGTATTACAAATGCAAACTAAATTTCAGACCCTATTCTAA[A/G]GATTAGAAACTAAATTAGGGACATTTGAATGAATGAGAAACATATACATCATTTTGTTCC	A	G	0.10	0.47	0.52	0.52	0.63	0.05	0.24	0.53	0.61	0.61	0.55	0.128	0.056	0.001	0.187	0.0003			
rs984222	1	118.85	CTCTGTGCCCCATACTCTGTTCATATTCTGTCGAGACACCCATC[C/G]CCCTGTGTTTCACTTGTCTGATTACATATCTGTCTTCTTAAGAGCAGAGAATGGTGTTTATTCATCTTT	C	G	0.64	0.32	0.39	0.35	0.36	0.55	0.34	0.52	0.36	0.49	0.48	0.050	0.000	0.013	0.013	0.0099		1	1
rs947127	1	119.29	TTAAGAGATTGCTTCTGAGATTTCATATGCATCTCTTCAGTTTACTGTGGGTATTC[A/C]TTTTTCCATGTTTCTGAGCAGTAGAGAACAGCCTTTTGGCATTATCTTGATAACATT	A	C	0.13	0.33	0.31	0.35	0.32	0.00	0.03	0.28	0.42	0.35	0.42	0.045	0.109	0.004	0.164	0.0054	1		
rs947129	1	119.32	CAATTATAAAAATCCCAGGTCACTCAAAATTAGACACCATAGAAAGTCAGGTAACCA[A/G]CTGGCTGACACTACAGCTAAAAGAGCAGCATCCAAGCCACCAGCCCCAGTACAA	A	G	0.74	0.70	0.73	0.67	0.71	0.59	0.94	0.57	0.64	0.65	0.62	0.004	0.056	0.007	0.003	0.0064			
rs1341009	1	119.35	CAAAAAAAAAATTATAATAAGGGAGACTAGTGTTCCTACATTGGAATGCAGATTGCC[A/G]TTGTGAGCCCATCATTTCTTGACAATTTATGCATTAGCATAAAAAAGAGAATAGA	A	G	0.10	0.45	0.41	0.35	0.48	0.36	0.67	0.20	0.50	0.39	0.40	0.056	0.035	0.030	0.000	0.0000			
rs1856884	1	119.35	AATTGACCTCTCTAGGACTATAGCACAAGGGGTGTCATTATCCTAAAGTAAC[A/C]CAGAAATTTAATAAGCTGGCCTAAAAGAGGGATTTCCTTTTCCCAACATGAGGGGCTGT	A	C	0.21	0.53	0.58	0.62	0.52	0.36	0.27	0.57	0.50	0.55	0.55	0.100	0.063	0.005	0.045	0.0018			
rs1361529	1	119.35	AATTTTGCAACGTGATCACTAACAAGCAGGAAAGCATGAAAAAAAACTGATTTGCA[A/G]GATTCTGACTGGGTGTTTGGAGGGGTGGACATTATCGTACTTATGTTTTCTCTGAT	A	G	0.90	0.55	0.60	0.65	0.52	0.64	0.33	0.80	0.58	0.61	0.60	0.051	0.041	0.025	0.000	0.0000			
[illegible]																								

rs1378726	2	33.73	TAAGTCCCCAATTCACAAGGACCTCATGAATCCATCCTGATCATTTCTGTCTCC[A/G]GGGCCCCTATGCATAGTAATAATAACAAATGCGATAATAACAGTAACAT	A	G	0.14	0.42	0.40	0.43	0.45	0.32	0.48	0.23	0.39	0.41	0.51	0.046	0.003	0.010	0.004	0.0063	1	1	1
rs1990838	2	34.40	CTATCATAAAAAGAACGTCCACCTAAGGTTGAAATAAAGTAGAGGGAAGCACTGT[A/G]AGAGATAGTGAGCACATGGCTAAGCTCTGGTGACATTATTTGAACCACTCTATCTC	A	G	0.81	0.55	0.49	0.54	0.61	0.55	0.68	0.44	0.46	0.51	0.48	0.054	0.005	0.010	0.000	0.0001	1		
rs1026301	2	35.19	AAATCCCTCCGATGAGCTTATTTTTCAGGTTCCATAATACTACTTTGAGA[A/C]AGTAACATTTTGAAAAAATATTTTGGTTATTTTGAAAAGTACTTAGCTTTAGTAAAATA	A	C	0.86	0.42	0.38	0.40	0.46	0.73	0.45	0.63	0.32	0.39	0.35	0.114	0.001	0.033	0.052	0.0025	1		
rs305179	2	36.34	TGGGCTCTGACTATTGCCTAGGCATTTTTGTCTACCCATTATTTCTTCACACA[C/G]TGGCTCCTTTTTATTTTCAGTATTTTTATAAAGTTGCCTCCAGCCAAGTACACTGTGTAC	C	G	0.45	0.69	0.74	0.72	0.82	0.50	0.64	0.42	0.64	0.74	0.75	0.035	0.000	0.044	0.001	0.0001	1		
rs1167465	2	36.37	TATTTTTATTCTTTAATCACTGAAGAAACTCTAATGCATGAGTGGGTCTTC[A/G]CAAAGCTGCTTCTCATTCAGAGTGTTTGTGAAAGTACATCTTTGAACCCACCTAAATG	A	G	0.69	0.54	0.66	0.62	0.73	0.73	0.64	0.56	0.64	0.59	0.62	0.001	0.000	0.004	0.004	0.0014			
rs1403858	2	36.69	ACAGCTCACAGAACTCCAGCCTTTTAGAGGAGCACAACTTACA[A/G]TATTGGGTGAGCTGAGACTGAAGTGGCTTACTAAATCCACATAGGTCAAGACGTGCCTGACATCCAG	A	G	0.19	0.25	0.25	0.27	0.25	0.00	0.36	0.44	0.29	0.22	0.25	0.005	0.005	0.010	0.109	0.0002			
rs884215	2	36.72	TGCAAACACGCACGTCAGATTTCTTCTGACCACTTCCGTACATGTCAGCATTTA[A/G]CAAGGCAGTAATTGTCAGTCCTCTCTAAGCCCTTTTTGAAAGCAATAAGGGAGGCTTT	A	G	0.83	0.61	0.58	0.55	0.48	0.73	0.73	0.50	0.64	0.60	0.57	0.051	0.012	0.000	0.015	0.0002			
rs1504	2	37.04	ATAAATGGGATC[A/C]TGTACGTTCTCTGCATCTCACATTTCTCATTTAACAATGTAGGATGAGAATTTCTCTAAGTCTATCATAGCTCTTATTTCCTTTTTATTATTACAATAAACAT	A	C	0.50	0.58	0.60	0.50	0.50	0.36	0.56	0.38	0.64	0.62	0.58	0.004	0.000	0.021	0.025	0.0000	1		
rs960902	2	37.70	GTTCATTCTTTTCTCTCCTTCATTTCTCCCTTGATCTTTCTTTACAGTGT[A/G]CTGCCTCTCTAGGCATTTTCTCTTCAAATTCTCATGCACTTGATTGGCCTTCAG	A	G	0.55	0.55	0.50	0.48	0.61	0.45	0.64	0.42	0.57	0.44	0.53	0.000	0.008	0.021	0.003	0.0004	1		
rs1446297	2	38.18	TTTCAGCCACATTTGGAGATCTACTCCATCACACACTGTGGGATTGGGGCCCAAT[A/G]TCTGTGGTTTAATGAGCCTCCAATGATTCTGATGTATGCTAAGGTTTGACACCCAC	A	G	0.45	0.66	0.66	0.68	0.73	0.18	0.39	0.65	0.61	0.64	0.63	0.015	0.029	0.000	0.114	0.0002			
rs163077	2	38.26	ACACAATTTCTGGTCCTGAAGTCCCCTGCCTGAGCAAAGCTGAGAAAA[A/G]GTGGACTCTAAACAGAATTCTGGGGCATTACTTTATTCCTTCCAAGGCTGGG	A	G	0.05	0.29	0.27	0.29	0.16	0.64	0.56	0.25	0.25	0.22	0.26	0.041	0.034	0.000	0.065	0.0001			
rs170815	2	38.39	TTAAACATTTGTGACTTTAACATAACCAAAACGGATATTGGTTAATGTCTAACTGCT[A/G]TTCTTTTTAAAGCGTATATTAATGTATCAAGTATATATTTTACTAGGACACTCTGTAC	A	G	0.24	0.41	0.43	0.33	0.32	0.23	0.06	0.27	0.46	0.34	0.41	0.032	0.131	0.034	0.041	0.0014	1		
rs440176	2	38.84	TCCCACTAATTCTGATGGCTAGAGGGGACTCACAGACCAACTTCTTCAAGGCCT[C/G]GTGTCTGGGGATACAGCTGCCACCTGGCTTACCCCAATCCAGCAGGGAAACTGG	C	G	0.35	0.61	0.59	0.59	0.64	0.14	0.05	0.51	0.73	0.57	0.62	0.042	0.231	0.009	0.140	0.0004			
rs1530853	2	39.00	ACACAGATCAATGCTTGGGTTTTTAATTTTATCCTGGAGAAGCTGATCAAGTTC[A/G]CAACGAAGCTCCTTTACCAGTTCAGCCACCTAAAATGAAAGCTACAATATAAGCAGTG	A	G	0.33	0.67	0.64	0.71	0.61	0.64	0.54	0.71	0.54	0.71	0.63	0.045	0.002	0.013	0.001	0.0000			
rs10219	2	39.06	CTAATGTACAGATTCCACAACTCATTCTGGCTGATTCTCTGAGCAGCATTCTGA[A/G]TAAAGTCTTCTACATCCAATCCCAACAAATCCAACAAAGGATCTCAAGGCCAGGA	A	G	0.86	0.75	0.71	0.73	0.71	0.73	0.51	0.78	0.61	0.78	0.76	0.017	0.015	0.007	0.001	0.0020			
rs929642	2	40.33	TAAAATAAAACCTTATGTTGGGGAAAATTTATGTCATTTTAAATTGCAACACTTTT[A/C]GACTTCTGAATTCCATAGAAAGAACTTGATATATAAAACTGGTTACCCTGGTTGTTC	A	C	0.83	0.61	0.49	0.54	0.63	0.91	0.76	0.61	0.61	0.50	0.49	0.084	0.049	0.006	0.119	0.0002	1		
rs764464	2	41.03	TTTTGGTGACGGCATCTCAGAAAACTTGAATGTCTACTGTAGATTTGGCTTG[A/C]CCCTAATAGGGCCCAGAATTTGGAGTAAAAATGGGAGATCAGGAAGAACTCAAGATGC	A	C	0.67	0.42	0.37	0.20	0.30	0.50	0.78	0.28	0.36	0.31	0.31	0.046	0.098	0.002	0.013	0.0022	1		
rs1560260	2	41.81	CAAATATATATTACATCCTACCACATAGCATAAAAAAATGAAGTCCACTGATCTGGATAGAGAGGAGCA[A/G]TTATTACCTTGTTCCCTCTAAACAAATGTTACTCTTTATTATAT	A	G	0.31	0.55	0.51	0.37	0.43	0.50	0.44	0.59	0.25	0.39	0.52	0.011	0.001	0.019	0.003	0.0002	1		
rs749651	2	41.94	GTGGTTCTTACCTCCTTCTTTGTCCAGAGAGAGAGGACACTTATTCTTCTTTAAG[A/C]ACCTTATTAACTTTCTCAACATTTTTTTGTCACTAAGGAGTCACTCTACACCCCTGCTAC	A	C	0.69	0.57	0.51	0.61	0.52	0.41	0.41	0.47	0.86	0.59	0.53	0.012	0.004	0.000	0.004	0.0001			
rs726548	2	42.03	TGGCCATTTCTGATAGGATGCCCAGAGAGATGCAGCTGTC[A/C]TCAGAGAGCTGCCATGTCAGGGCCTCTGGGTTAGGAAATGCAGTGAGAATGAGGAGCAGGACAAGAG	A	C	0.48	0.42	0.46	0.46	0.48	0.25	0.65	0.56	0.43	0.34	0.38	0.001	0.014	0.009	0.031	0.0038			
rs1496328	2	42.65	TACCCAATTGTTTGCTCTTGTGCTCAGTGAACAGTACTCAGTTTTTCAGCA[A/G]TTGTCAGTGGTGGCATTGGTGAGAGTGCCATTTGGAAACATGAGGAATTTTGGCAGGCTT	A	G	0.29	0.35	0.36	0.33	0.36	0.05	0.78	0.30	0.23	0.36	0.35	0.000	0.138	0.000	0.059	0.0003	1		
rs1390610	2	42.69	TGTGCCCTTCCAATGCCTAGCAGATTTATTTTGACTCTTCCTGGATTTTTGTC[A/C]TTTCTTCCACCCTGAATAAAGCCTCATTCTTGCTCCCTACTGCTCATCCCCTC	A	C	0.75	0.78	0.81	0.76	0.63	0.91	0.96	0.71	0.82	0.74	0.76	0.003	0.026	0.001	0.011	0.0044			1
rs1016607	2	42.95	TCAGAGCTACACTTTCCTTCTCAGAGGTCACCTTCTATGGGTCACTGAGAATG[A/G]GGGAAAGGGGAACAGAGGTCAGGTTAATTTAAAAGATCGTTTACCTGCAGGTAAA	A	G	0.69	0.61	0.61	0.61	0.74	0.82	0.72	0.75	0.75	0.70	0.59	0.004	0.007	0.004	0.029	0.0000			
rs896986	2	44.33	CTATTAAGTTTCAAGAGCCAACTGCTGGTAGAACAATTACCTAGTCATTCAAAGGT[A/G]TATCTGAATTGCATTTTCTAAGTAGGAGATGTACATCGCTTATAATTCCAAATAG	A	G	0.55	0.46	0.46	0.46	0.66	0.59	0.23	0.47	0.36	0.43	0.49	0.003	0.024	0.000	0.009	0.0002	1		
rs1437747	2	44.84	TTACGTGAAAACTCTTCTAGTGAGAACCCAAGGCAAAAACTGTGTGTTTCTTCT[A/G]TAATACTTCCTCAGAGTATCTATTGTCTTTCAAATGGGGCCAAGGCTTCTCAAGGGAA	A	G	0.38	0.57	0.57	0.52	0.55	0.18	0.36	0.55	0.58	0.54	0.41	0.029	0.029	0.000	0.091	0.0118	1	1	1
rs343955	2	44.92	CCACATGTGAGCCAAATATTTCTGCTATTAAATAGCACTTCAAAGCTTGACATGA[A/G]TGTTTGTAACAGCTGAATGTCTCTTGTGACTTCATTGCAAAGAAGATGTATAAGCTG	A	G	0.88	0.62	0.60	0.60	0.66	0.68	0.57	0.83	0.82	0.65	0.59	0.047	0.000	0.026	0.003	0.0004			
rs83995	2	45.14	AGCCCCTAGGCCAGGCATGGGTATTTATTCTTAGGTATGTTCTTTAAGAAG[A/C]TGTAATCAGCATCTTGAGCCGGGCTCCCTTTGTGAGGCTTCTCTTACTATGGAAGTG	A	C	0.88	0.61	0.57	0.52	0.54	1.00	0.99	0.93	0.81	0.61	0.58	0.063	0.188	0.104	0.188	0.0000			
rs921115	2	45.22	TGGAGAAAAGCCAGCGTCTATCCCCCCAAGAATTCCTGTGATCAGACCGTCT[C/G]GCAACCTAGTGCACCTCTGGGTTGTGTGTCTGTCTCCGAGGTCTCGCTCTTCGCGGAC	C	G	0.60	0.46	0.45	0.44	0.52	0.64	0.65	0.50	0.46	0.44	0.45	0.007	0.006	0.000	0.010	0.0003			
rs1447107	2	45.25	CATCAGTCTCCACTAGGAAGATCACAAGGAGGAAAAAGAAGATAGACTGAGCG[A/G]TGAACTTAATACCCAGCCAATTCAGAGGTGAGGAAGCCAGGCCCTCAGGATGC	A	G	0.81	0.50	0.52	0.56	0.41	0.41	0.68	0.73	0.58	0.55	0.52	0.045	0.014	0.008	0.000	0.0000			
rs1014454	2	45.59	TTAGAGGGTTGGCAAAAAATGCTGTAACTGCAGGTATCCTGTACTGGATTTTTC[A/G]CATCCATCTTCATTTTTCTGTCATAGTGTCGATTGTTCCATTATCCCAGTTGCATTGTA	A	G	0.71	0.53	0.48	0.49	0.48	0.77	0.60	0.65	0.64	0.55	0.46	0.011	0.006	0.014	0.041	0.0001	1		
rs748573	2	45.74	CTGCAACTTAACTCCAGTTTAACTTTTTGCTCCTTTGGTAATTAGAATTTGCA[C/G]CTTTGGATGACTTGCCCCCTAATATGTGTAAGTATCATGATTCGTAAGGTATTTTAAGGG	C	G	0.69	0.75	0.75	0.73	0.77	0.86	0.61	0.60	0.64	0.78	0.85	0.001	0.010	0.021	0.012	0.0087		1	1[9]
rs1020445	2	46.35	ATATCAGAGATGTTCACTGTGAACCACTCCAATAGGTTGCCTTTCCTGAAAGTTGTTTGGGATGTTTTGCTC[A/G]GCATGAAGTGTTTGTTTGCCTTATTCCTGCCCAAACTGAC	A	G	0.80	0.71	0.69	0.79	0.75	0.82	0.97	0.78	0.68	0.64	0.65	0.009	0.081	0.009	0.015	0.0001	1		
rs1000532	2	46.49	TGCAAGCGACGTCTCAGAAGCCCCATTGTTCATGGAGGAAATTAACTCAAATTATT[A/G]TTATACACACCCCACATAATCAATTGCATTTAAGCAGGTGTCCATATACAAAGGAC	A	G	0.70	0.49	0.58	0.44	0.61	0.45	0.84	0.61	0.57	0.49	0.51	0.004	0.028	0.000	0.015	0.0037			
rs1868092	2	46.59	TGAAGTCGTTTGGATTCCACACTGTTCTGTATATTCACATAGTGCACTTTG[A/G]GATGGAGGTCCCATTAGGAAAACAGCGCCAGTTTCACGTTGAAATGTCCAAAGCGC	A	G	0.14	0.45	0.45	0.46	0.48	0.05	0.06	0.31	0.32	0.43	0.41	0.065	0.103	0.008	0.118	0.0017			
rs1530625	2	46.63	GAGTATTCTGATACTCAGCAACACATTACAGCAACTTCATCCTCCCCTTTCTTAGGACAAAAATTTCATAGAAGACCCAAATGACAACATGCCTCT	A	G	0.31	0.48	0.49	0.50	0.43	0.73	0.72	0.39	0.68	0.39	0.47	0.035	0.009	0.025	0.011	0.0008			
rs1051317	2	47.10	TTCTGGGAGATACAATTCAAGTTGAGATTTGTGGGGGGACACAACCAAAC[A/C]ATATCAGCATCCTTTCAAGAATATTAGATAATTGGAGCTGAGTACTCAGG	A	C	0.17	0.30	0.31	0.28	0.14	0.18	0.01	0.25	0.29	0.17	0.23	0.024	0.120	0.010	0.020	0.0046	1		1
rs922903	2	48.32	GCTTCTAGGAGGAGCCTAGCTTTTAGCTTCACTACACCCAACAGTGAGGGGCA[A/G]TGACGGGTAGTCTCAAAGAAGGCAGAAGCAGCAGAGGCAGCATCCCTGGGTCCCTA	A	G	0.79	0.49	0.45	0.54	0.54	0.95	0.42	0.80	0.46	0.61	0.60	0.072	0.000	0.108	0.188	0.0113	1	1	1
rs1509562	2	48.47	TTATTCCTATTCATAACATGTGTCCTTATCTTCAAAGATAATCCACTATTGAA[A/G]TATTAGGGATTTGGGAGTTGCGGATAAAAGAAGTGAATTAAATTCATTCATTCAACAGTA	A	G	0.29	0.33	0.30	0.28	0.43	0.05	0.02	0.41	0.21	0.34	0.33	0.001	0.099	0.003	0.066	0.0000			
rs1504188	2	49.25	GTCCCGCTTACTCAAAGCGAAAAGGAGAGGCTGATGATTCTACTGGCTCCTG[A/G]TGAAAAATGAAAGGCCAGTGTTAGACTGGTTAGATCTCTGAATGGGAAGAGGGGATTT	A	G	0.21	0.51	0.48	0.54	0.48	0.68	0.42	0.51	0.50	0.47	0.56	0.045	0.001	0.000	0.023	0.0056	1		1
rs1394207	2	49.26	GTATTTCAGTTCAGCCACTTCTCTCTTGCCTGGGCGGCAGGATACTATAACA[A/G]CTCAGCCTTGGAATATGCAGCTGTTCAGCTTGGCCAGGGCATTGACTTCCCAGGGGCA	A	G	0.21	0.41	0.39	0.40	0.25	0.68	0.41	0.48	0.32	0.41	0.46	0.020	0.002	0.003	0.052	0.0039			
rs960038	2	49.31	AGGACATATGAGAGCCATGTTCTATATGTTGGAGAGAATGGCAGCCCCAGA[C/G]CCCTTCTTTGACAGACAAAGGGCCATTGTATAACCAGGCATTACTTTATTCCTTCCCAGT	C	G	0.33	0.66	0.65	0.60	0.74	0.14	0.11	0.46	0.57	0.55	0.57	0.050	0.195	0.024	0.166	0.0029			
rs1520446	2	50.58	CAAATTTCATAAGTACGTAGTGACAGTGAAATATATGAGCAAATGGCAGAA[A/C]ACAAACTGGACTGGTGAGAACAAGGTAGGAACTGAGATCTGAGGTTATTGTCTGCCATTA	A	C	0.40	0.26	0.20	0.20	0.25	0.68	0.29	0.11	0.32	0.24	0.20	0.021	0.002	0.016	0.094	0.0002	1		
rs1995584	2	51.24	ATGCTCTGTGTTTATTGCTGCAATCAGACCCTGTGCTTTAACATTAGGAAATCA[A/G]TGTAAACACCTACTTTCTCAATTAACATTAAGGTAAAGTGGAAGCTTTGAGATACCTAT	A	G	0.21	0.49	0.40	0.50	0.43	0.59	0.43	0.41	0.39	0.48	0.50	0.023	0.001	0.000	0.021	0.0047	1		1
rs1876088	2	51.32	GGCATATACTCTGCCATGATTTCCAATTAAGTCAAGTAAGTCATGGGATTTATA[A/C]TGCTATCCAAAGAAAGGAATCATTTTCCATATTCCATTGAAAGTCTGTTTTTCT	A	C	0.67	0.63	0.66	0.62	0.64	0.55	0.63	0.43	0.61	0.59	0.69	0.002	0.000	0.033	0.006	0.0016			
rs1017418	2	52.02	ATGTGTGTGTAACTATGTCTACTCTTTTTCTGAACTGCTTCTTATCTCATCA[C/G]TGTAAAGAGGTAAAGAGTTAGAAGCCCCATGCATACAACCTTGCATTCTCTGGGCTT	C	G	0.64	0.43	0.46	0.41	0.39	0.75	0.93	0.47	0.50	0.41	0.44	0.012	0.105	0.001	0.031	0.0002	1		
rs1483869	2	52.88	AAACCTTGCTTTAACTTGTATAGCCCACGAATTCATTTATTTCAGTTGCAGTTATTTCA[A/G]CAAGTGACTCTAGCCTGTTTCCTGTATTTTCTGAATGAGTT	A	G	0.88	0.61	0.65	0.65	0.61	0.27	0.53	0.52	0.57	0.73	0.67	0.044	0.002	0.000	0.060	0.0002	1		
rs2009142	2	53.92	ATCAAACATGGATAATATTTCTGATAACTGCTGAAAAGATAAATCCAAATTATGC[A/C]ACTAATGATTCAGGCTAAGATTTATGAAAATTATCAGTAGATATGACATATATG	A	C	0.26	0.23	0.23	0.18	0.21	0.09	0.21	0.26	0.21	0.27	0.30	0.001	0.000	0.001	0.017	0.0022	1		
rs805423	2	54.10	CAACCAGATATTGCTTAGCCCAGAAAAAATTAAGGAAGGAATTAAACGCCAACA[A/G]GAAAAGAATGCCGATGCCCTAAGGTAATTGATGAATGGTGTACTTCAATAGATGACAT	A	G	0.57	0.54	0.52	0.46	0.55	0.73	0.47	0.68	0.57	0.45	0.44	0.004	0.000	0.012	0.028	0.0006			
rs805308	2	54.17	CATAGATCAAGTACATTCCTAAAGACCAAAGTGTAAATGCAGTTGAGAGTTA[C/G]TGGGACTATCTTGCTGATCAGAAATGTGCACCCGTAATCTGGACATCAGAGGTGTGTTT	C	G	0.62	0.55	0.52	0.50	0.55	0.73	0.47	0.69	0.61	0.47	0.46	0.010	0.000	0.017	0.031	0.0001			
rs843635	2	54.52	ACAGAAAAAAAAAGCTTCAGAAATATACACAGGGGTTCTTTTGAGTCTTTCCTGC[A/G]TATTAGCCATGCGTATGTAGACTGAAATTCCACAAAGTTGTGCAAAGAATGCCTGG	A	G	0.48	0.46	0.48	0.49	0.45	0.73	0.56	0.71	0.36	0.53	0.46	0.000	0.005	0.027	0.034	0.0006	1		
rs2033411	2	55.05	GAAAATTCCACCAGTACAGGGGCATTTGGAATTCCAACAGCTGGACCAACAAAGA[A/T]ACTAGAAATAAGCTCAAATACCCAATAATAAGACAGTGATGAAGTAAATTATGATG	A	T	0.43	0.38	0.43	0.34	0.39	0.45	0.77	0.58	0.46	0.48	0.39	0.000	0.062	0.002	0.000	0.0003	1		
rs1961245	2	55.07	AAGTTTTCAAAGACATTTTATGAAGCAATTAAAGATTTTACAAGAATGTCAAGAT[A/G]TCGATATTTTTGGAACCTGATGAAAGGCATCTCACCCAGATGTTTCTGTTGTACTTT	A	G	0.62	0.30	0.38	0.41	0.39	0.45	0.52	0.53	0.32	0.31	0.26	0.029	0.005	0.007	0.002	0.0102		1	1
rs727878	2	56.09	AACTTTCTATTCATCCCACTCTTCTAACAGAGCCATTTAATCCTATCCAGAAAGAT[A/G]AGTCCATTTTCAGACTCTCCTTTGATCCTACAGATTAGAGGACAGAGAAGTCACC	A	G	0.57	0.39	0.31	0.38	0.41	0.77	0.34	0.40	0.25	0.38	0.34	0.032	0.000	0.003	0.105	0.0003	1		
rs1430197	2	56.11	TTTCACAAGGGAAATCTAACATCATCATTGAATAAAAATTCTTCCCTAACCC[A/G]GGTTGTGACAGACTTGGTCTGCAAGGTGGGCAGTGAGTTGGACCCCATGAGTTGTTCC	A	G	0.40	0.29	0.35	0.45	0.41	0.82	0.38	0.40	0.32	0.40	0.38	0.002	0.000	0.000	0.124	0.0020			
rs1432567	2	56.31	TACATCTATTTACACCTCACATTATACTGGAATTACTTTATTCATTTGCTTGC[A/G]TACCTGCTTCTTGTCTACCTTTGCCCACTAGTATGTAATGACTATGAGTCCAGAGACTGTC	A	G	0.19	0.38	0.43	0.45	0.36	0.22	0.69	0.33	0.46	0.30	0.41	0.042	0.026	0.008	0.036	0.0007			
rs1030092	2	56.59	GTTCATAGTGAGGTTTTTTTCCCTCTTCTTTCATTTGGTATGTTTAGACTT[A/G]CCAGCCTTTTGTCTAGCTGTCAGGAAGTCGAGGGCCAAGTTAGAAGTTCAGTCACAGAAA	A	G	0.64	0.51	0.59	0.60	0.63	0.41	0.17	0.55	0.54	0.59	0.72	0.002	0.090	0.001	0.014	0.0098	1	1	1
rs879808	2	56.68	ACTTAAAGAATTCCTCTGTTGGGCTGCATATTAGTAAAAAGATCATTAGCTTGC[A/C]GGAAAGAGATAGCTACTAAAGCTAGCATGAGGGAAAATGACATTTAATAAAATTTA	A	C	0.76	0.53	0.49	0.50	0.46	0.64	0.80	0.53	0.61	0.46	0.35	0.035	0.055	0.000	0.011	0.0095			
rs1017267	2	56.76	AGAATGTATTTACATTAGACCTGCCCCCAAAATTGGTCTTTAATAAAATTATCTG[A/G]ACTTTGATTAAAGTTCTTTGAGCAATGACAGCCCATTTCTTTGCCTAAAATTGAAAAC	A	G	0.71	0.43	0.49	0.48	0.54	0.36	0.48	0.45	0.43	0.50	0.58	0.020	0.000	0.000	0.010	0.0029			
rs715271	2	57.11	GGAATAGGTGAAACAGAGAGGAGGAACTCATATTAGTTCTTCAGGTTGTCCC[A/G]AGCCAGTTTAAAACAAACACAATAATTTGTGAATAAGGTCTGCTTTGCTCCCTTCAGAT	A	G	0.45	0.68	0.69	0.61	0.67	0.41	0.30	0.67	0.61	0.64	0.68	0.043	0.111	0.002	0.061	0.0019	1		
rs1568452	2	57.99	AGTCTAGAGAAACAAGGATAGTGCCTCTCATCTTTGTGTTTCATCTTCTGCCATA[A/G]CATTGCCAGGAGTTCCATTTGGAGAGAGCAGAAACACGATTTGGGGTAGTGTT	A	G	0.12	0.36	0.37	0.38	0.43	0.41	0.29	0.47	0.32	0.48	0.41	0.035	0.000	0.023	0.004	0.0013	1		
rs980481	2	58.65	GATGTTAAAGTTGTGCCTTTTATTTTAATAACAGACTAAATCATTGCTCTGTACTG[A/C]AATTATAAGAGCAAAACTTTGTACTACTTGTACATTCCAGTTTTTGAAATATATGATT	A	C	0.81	0.51	0.52	0.59	0.46	0.45	0.80	0.67	0.46	0.47	0.45	0.024	0.043	0.003	0.010	0.0058	1		1
rs764974	2	58.74	GAACCTCGGCTGTATTCTGCTCAGGCAGTATGATTCTATTGAAATTTCAAGTCTCTCTGGGGATTTTTTCCAATCA[A/G]CCAAACTCATGATATGAATTTTTGATGGCAAAATTA	A	G	0.25	0.59	0.59	0.49	0.48	0.32	0.65	0.54	0.54	0.57	0.56	0.061	0.006	0.000	0.041	0.0014			
rs1012545	2	58.80	GTTGGAATACAGGTGGACTCCTGAACCTGGGGCAGCCCGGGGACTAAGACAAG[C/G]CCTGGCTAAATCAATCAGAGTTTTTCCCCATCAAGAAAATTAGAAGCAAGAACAGAC	C	G	0.14	0.46	0.48	0.35	0.34	0.23	0.73	0.39	0.46	0.42	0.47	0.055	0.001	0.001	0.032	0.0002			
rs1014585	2	58.92	TCTCTGGGTGACTACTGACCAGTTCTTCTTAGCTCTTAATGCCCATCAACTTA[C/G]TGTCCTTCAGCTCTGAGATCCAGGGAGCCACTTGTAGAGTG	C	G	0.31	0.39	0.45	0.39	0.38	0.20	0.25	0.43	0.50	0.44	0.49	0.014	0.034	0.009	0.051	0.0001			
rs887941	2	59.66	AACTATCACCCCTACCCTACAGGTTATTTTTCTTCCTCCTCAATAATGAGT[A/G]GCCTAACCCACCCATTCATTTGACAAATAGCTGTTGGGCATGGTACACATGGGTGATT	A	G	0.20	0.42	0.40	0.45	0.35	0.36	0.69	0.43	0.29	0.45	0.41	0.028	0.048	0.000	0.003	0.0000	1		
rs359265	2	60.43	ATACAAAGTTTAGAGTTTGTAGGACAAGGCCCTGAGTGAAGAGAAGTGGAATAC[A/G]AGACTGATCCGATAGGAGCCATCAAAACAAGCAGGACAGTCCCTCCATGGACAGCC	A	G	0.90	0.61	0.67	0.61	0.66	0.77	0.91	0.56	0.64	0.54	0.52	0.049	0.086	0.001	0.017	0.0135	1	1	1
rs1512226	2	60.53	AAAGCTTTTGCTCCCGTTCGGTGGTGTTCGTCTCTTGTGAGTTTGGGTGT[A/G]GTTAGCTGCCTTCTGCCACTTCCCATCAAGGCCTTCACTTTTGTAAAGCTGTCACTTCTCC	A	G	0.71	0.53	0.55	0.66	0.52	0.41	0.44	0.74	0.50	0.54	0.53	0.020	0.006	0.028	0.012	0.0000			
rs1177261	2	61.31	AGGTGCAACTAAATGTGTGACTCATTTCAGATGAAATGTGATCTGAGACGTGTA[C/G]CTGAGATGATTTGGAGTGATACTCAATGAACCATTAAATAACATTCATCAATGTATG	C	G	0.83	0.58	0.57	0.59	0.55	0.32	0.33	0.26	0.46	0.63	0.62	0.029	0.042	0.081	0.048	0.0005	1		
rs1177274	2	61.34	TCTTTTCTTATTTCTGCTTTTACAGTCATCCTTTTTCCAAAAGGAAGGTATACAT[A/C]TTCCTCCTTGTCTAACAATGGCACATTGCCCCAGAGGTGTAAAGAGGCCAGGGGTGAC	A	C	0.55	0.41	0.41	0.37	0.41	0.60	0.36	0.53	0.46	0.33	0.38	0.010	0.000	0.006	0.023	0.0013			
rs1729662	2	61.37	TTTGACCACTTCATGGAAGAATATTCATGTCACCAACATTGTTATTAACCATAAT[A/G]TGTGCTTTTATACCACTGTTTTAAAATGTCATCATGATTGGATTATTGCTACT	A	G	0.64	0.59	0.60	0.63	0.59	0.45	0.54	0.47	0.54	0.67	0.63	0.000	0.000	0.008	0.015	0.0009			
rs766447	2	61.71	CAAGTTTATATTGACTATTTGATATTGCTGTGTTTTTATTTGAAATGGGACTGTTA[A/G]ACACATTTTCTTATATTGAGCAATAGAGCTGGAATATTAAAGATGTATGCATTAAC	A	G	0.50	0.66	0.61	0.68	0.55	0.50	0.53	0.46	0.57	0.68	0.65	0.004	0.012	0.019	0.030	0.0015			
rs1919481	2	62.03	TGTCAGTGTCTCTCTCCCTCTTTTTTAACTTTGGTTCTGTGTAACCAGATCAT[A/G]TCTAACAAAGTGCTTCAAAAGTTATTTCTAACCAAATAATATTTAAATCACAGTGTATC	A	G	0.29	0.31	0.33	0.26	0.41	0.82	0.83	0.41	0.57	0.31	0.35	0.000	0.155	0.014	0.151	0.0012	1		
rs1553832	2	62.99	TACAGCTAACATAAAATGATGAAGTAACACTATGTGGCAAGGCTAGGTCAAGAA[C/G]TGTGATGCAACTTACACTGGGAGAGCTAGAATAGTCATTCTTGAAGTCTTTAGCTGCC	C	G	0.31	0.35	0.28	0.27	0.46	0.32	0.27	0.25	0.43	0.43	0.34	0.004	0.003	0.004	0.001	0.0007	1		
rs992214	2	63.46	AGAGCTAGTGACATTGATGCAGAATCAATAACCTCTGGGGTTGGTAAGAATTAAC[A/G]CTATTTTTAAGTGCTTTTATATTTATCAATAGTTAGTATCATTCTATTTATGTTTGGA	A	G	0.88	0.46	0.52	0.59	0.45	0.95	0.74	0.60	0.46	0.58	0.48	0.044	0.009	0.000	0.091	0.0020			
rs262472	2	63.81	AAAATAGACATTTTTCTCCAGCTTTAAAACTATATGATTTCAGCTTCAGGTCATT[A/G]AAAGTTTTATGATTGGAAATTAATCTGGAAGGAAGATTCAGCTCTTCCCACTAGACAC	A	G	0.98	0.68	0.69	0.76	0.64	1.00	0.98	0.75	0.82	0.74	0.67	0.069	0.084	0.000	0.106	0.0014	1		
rs890478	2	64.51	GAAGGCATAACAAAATACTCTTTGCTACAGTAAACCACTCCCGTGCATGGAAATATATAGTGAGCTGCTCA[G/T]GGGCTCTTTCCAAAGATTTGGAACAGACTC	G	T	0.52	0.47	0.52	0.52	0.55	0.14	0.31	0.59	0.57	0.42	0.42	0.018	0.012	0.026	0.071	0.0041	1		1
rs1050676	2	65.29	TCTCAGGCAGAGGTGCAACCTGTCGGCCAAACACTGGCTAGTGAGAAAAAAA[A/T]CCTTCAAGAAGTCTCCTTGCAAGAGCCATTCAACACTGAAGTCAAGGGGGAA	A	T	0.40	0.28	0.31	0.28	0.41	0.36	0.30	0.32	0.29	0.36	0.30	0.006	0.001	0.000	0.008	0.0002	1		
rs2008312	2	65.43	CAGCAGAACCGGTGTCCTTCACCTGCCTGCGTCTTCTGCAGTTCGTTAAGCC[A/G]GCGCGCCCTTAGCTCCTGTGCACTTTAGGGAGAAAGCCGCAGCACCGGCGATGTGGC	A	G	0.70	0.72	0.71	0.66	0.63	1.00	0.99	0.80	0.61	0.72	0.68	0.037	0.106	0.020	0.106	0.0013			
rs268866	2	65.47	TCCTGGCTCCAAAGCCATCCCATCAAAATTAATTAGGTGGCTATTTAACTCA[A/G]GGTTACACAAATAAATGCTTGCTGTACAAATGCTTCTGGGTGAATTCCTCTATAACCTG	A	G	0.50	0.53	0.51	0.56	0.68	0.00	0.29	0.31	0.68	0.57	0.53	0.000	0.031	0.023	0.202	0.0001			
rs1000756	2	66.77	TTTCGTTTCATAACAACCAATCAATTTACATCATTATAAAACGTTTGGAGAG[A/G]TGGAAAAAAAAGACCAACAGAGAAAAAAGTAACTCTTAAATAATATGCTCAGTTACAAT	A	G	0.19	0.34	0.35	0.33	0.29	0.32	0.46	0.16	0.29	0.36	0.37	0.016	0.010	0.022	0.000	0.0001	1		
rs1106577	2	67.02	AAAAGCTCAACATCAAGGAGATTTGGTTGATGAACACAGAAGAGTAGTTAGTGTACAA[A/G]TGGAGGAGAAGTCCAGAACATCAAGGACCTTTGTGTCTCTTGTGAGAGCTCC	A	G	0.55	0.49	0.53	0.56	0.55	0.36	0.13	0.25	0.50	0.59	0.55	0.005	0.072	0.017	0.005	0.0011			
rs736779	2	68.55	ATATTTACCAAATAACTTTCGGAATGGGATTTGGCCAGAATCATCTGAGTTC[A/T]GCCCAAACATCTGATTTCCAGAATTCTATAAAATAAAATATGGATTATTTCATTTTGTTT	A	T	0.50	0.60	0.53	0.48	0.52	0.55	0.59	0.70	0.75	0.52	0.55	0.000	0.003	0.025	0.147	0.0011	1		
rs1050181	2	68.60	GCGCACAGAGTGGATCAAAGCCATCCAGATGGCCTCCCGAACTGGGAAGTAAAGA[C/G]ACTCCTGCATTCCTCCTCCCCTCCTGAGGGAAGCCCATGGACAAGCTCAGTCCA	C	G	0.64	0.38	0.47	0.48	0.43	0.18	0.16	0.22	0.21	0.45	0.51	0.005	0.089	0.046	0.067	0.0001			
rs9902	2	69.03	GTGCTGCCTCAAATGTCTTCACTGGACGTGGTCATGCCTGAGACCAGAATGGAAT[A/G]AACAATCCATTTGAATACAGCGATGGATGGTCAGAGATGTCCACACACATCTCTG	A	G	0.21	0.51	0.44	0.62	0.61	0.14	0.20	0.49	0.43	0.35	0.45	0.027	0.036	0.004	0.046	0.0006			
rs2002879	2	69.71	AAGAAAATATGTAGCATTAGTTTAGTGGGATTAACTTGTTTCAATCTTCAGTTT[A/G]GACATAATATTGAAGAACTGGCAGAGAATATAAGAAGAAGAAAAAAAGGTCCTC	A	G	0.07	0.34	0.39	0.44	0.38	0.55	0.67	0.29	0.50	0.42	0.43	0.063	0.041	0.001	0.017	0.0006	1		
rs726920	2	70.13	TCCTTACAGTGGAGTCCTGATAACATAGAGTAACACTAATTATTTA[C/G]AATCTCTAAAGTGCCATAGAAAAGGTTTCCATTCAAGAAAACTAACCCAAGTATAATCAACTCTGCA	C	G	0.14	0.59	0.59	0.56	0.66	0.41	0.68	0.38	0.39	0.63	0.60	0.119	0.001	0.023	0.021	0.0004			
rs897119	2	70.14	CTTGGCAACTTGGGGAGTACAGCATTTTCTGAGCTAGGAAGAGGTAAT[A/G]GCACAAGGGCTTCTACTATTTTGTATTTTCAATTTCTTCTTGAAAAACTGTGCATGTG	A	G	0.14	0.59	0.59	0.56	0.66	0.41	0.68	0.38	0.39	0.63	0.59	0.118	0.001	0.023	0.021	0.0003			
rs1986601	2	70.18	CGTAAACTCATTAGCAATGAATTAATGCCCACTAAAGCCACATTGTTATTATGCT[A/G]TTTTCTCTTTTAAGTTGGCAAAGTCTTTTTCTCATTCCCTCAATGCTATTTGTGGGA	A	G	0.12	0.63	0.59	0.63	0.57	0.18	0.34	0.50	0.39	0.63	0.64	0.120	0.023	0.000	0.076	0.0063		1	1
rs1448927	2	70.68	GGCAAAATACTTCTGTGTCATGTTATCTGATTTTCTTCTCCCTGTTGGAAAAT[A/G]AAAAGGTACCAAAATGATGCAAACTCTGCTTTTGCCTTCAAAGTAAGCATGATGAACCC	A	G	0.45	0.53	0.46	0.50	0.46	0.45	0.79	0.52	0.39	0.47	0.56	0.001	0.050	0.001	0.000	0.0061	1		
rs1048753	2	70.86	GGTTTTGCCTTTCTCTATGCTAACACTATTTTGAGCCACTGACGTGCAGAT[C/G]GGTGGTTAGTTTCTTGGGCTTTTATTTTGCAATTTTATATATATACACAT	C	G	0.35	0.64	0.61	0.54	0.57	0.77	0.71	0.53	0.68	0.57	0.56	0.049	0.000	0.007	0.003	0.0011			
rs2058899	2	71.55	ACAGCACAACAGAATGGGGCTCATCTAAGTAATGAATTTTTCACTACTAAGTG[A/G]CCAAATGATAGGGATTTGTGAAGATGGATTTCGATGGTACACACTGCCTCTCTTTCTC	A	G	0.24	0.53	0.56	0.53	0.58	0.36	0.69	0.64	0.43	0.59	0.58	0.060	0.000	0.005	0.002	0.0002	1		
rs227771	2	71.83	GGAACTTTATCTGAGAATTCTTTGGGGCCTGGGTGGAAGTTACATTCTTTTA[A/G]AGAGGATATGTGTTTACTTTTACCAGATGTCTGGAGACACTATAAACACAGATTAACTTA	A	G	0.21	0.51	0.46	0.40	0.41	0.36	0.66	0.39	0.64	0.58	0.54	0.032	0.023	0.002	0.005	0.0033			
rs975612	2	72.36	CTTCTAGTTCACTAAGTAGCCAGGGAGGTGGTAAGATGATAGAGTTGCTAAAG[A/C]AAAATATTCCAGAGGAGGATTGGGTGAAGGAAGATGGGGGAATTCAATTTTGGACAT	A	C	0.14	0.67	0.57	0.65	0.52	0.91	0.73	0.61	0.77	0.60	0.60	0.118	0.012	0.072	0.078	0.0006	1		
rs1653249	2	74.12	AACTTCTAAAGATCCTTCTTATTCTACAATTCCATGATCTCTGTGATTTAGGGA[A/C]TATGTAAAGAGATTCTACGGCAACAAAAAAATCAATTTATTATCTGGTTTGCTCTAGCA	A	C	0.57	0.35	0.32	0.41	0.36	0.50	0.44	0.47	0.18	0.42	0.43	0.025	0.001	0.015	0.010	0.0055	1		1
rs831547	2	74.14	GTCACAATAAAAACAAAATATATATTCTGCTCAGGAATAAACAAGAATACTACTGG[A/C]TAAGTAGATAACCATTTTGTGAAAAACTAAAGTCTTATAACTTGAAAGTCCACATAA	A	C	0.50	0.68	0.73	0.66	0.77	0.41	0.58	0.55	0.82	0.66	0.65	0.031	0.013	0.003	0.056	0.0044			
rs828869	2	74.30	AGGGCATGACACCAAACTCAGAGTGCCTCTAAGTGAAAGGGTTAATAAAGTTAGT[A/G]CCATAAAAATATAAGTTTTCTGTTCAGCGTAATAAAACACCATAATAACAATTGAAA	A	G	0.10	0.56	0.62	0.46	0.57	0.45	0.52	0.53	0.71	0.52	0.58	0.164	0.003	0.000	0.009	0.0014			
rs741418	2	75.34	GTGATTGCCAGAGGATGAAGCAGCACAGATTGTGAATGTCAGGATTGCACAGGC[A/G]GAGAAAAGCACCTCCCCAAGCAAGAGGAGCATCACATGGTGTGGGCAGGGGTTTG	A	G	0.79	0.60	0.57	0.62	0.55	0.32	0.16	0.45	0.50	0.60	0.58	0.017	0.140	0.015	0.052	0.0023	1		
rs1015645	2	75.84	GATGTCCCAATTTTAAAAGAAAGGAAGAGAGAGACTTTTATAAGGGAA[A/G]AAAGAGAAAGCTAGAGGGGACCTAGACCGTCCAGCCGTTGCTGTGTGTGTCTGTGGCC	A	G	0.62	0.52	0.47	0.50	0.29	0.73	0.53	0.49	0.32	0.51	0.51	0.001	0.004	0.011	0.007	0.0001	1		
rs730150	2	76.33	CCAACCTATACTCTTCCAGTTTATAGCTCCTACACTCATTCAATCCCCTCCTG[A/G]TAGCAAATAAGCCTTCCTTACCAAACATGGGACTAAATGTCTGAACT	A	G	0.07	0.36	0.37	0.48	0.48	0.20	0.45	0.35	0.14	0.32	0.33	0.073	0.003	0.000	0.017	0.0010			
rs1916870	2	76.41	STAAGAGAACTAAAAATTATCTCATGAACTTCAAGATCATCCCCTGGGTCCCAGAATT[A/C]ACAATGAGAATACAGAGAATGCTTCTGAGAAAACTGAAAGTAACTACTTCATTTT	A	C	0.88	0.80	0.81	0.80	0.84	0.95	1.00	0.98	0.75	0.76	0.71	0.003	0.070	0.033	0.026	0.0091		1	1
rs7563300	2	76.66	AGAAGCCAGTAGGAATAGCAACATCAGTGACAGCAGCAAACTCATGTATTGCTGAC[A/G]TGACATTAGAACATGACAGGTTACATTTCAATGCTTTAGATATGTTAAATTTTATCT	A	G	0.19	0.52	0.46	0.34	0.34	0.50	0.54	0.41	0.54	0.41	0.52	0.040	0.000	0.005	0.000	0.0020	1		
rs2860827	2	76.77	CCTGCTCATAATTAATGCATCTATCTTATATTTTATCTCCCTGACATCTCCAAACCC[A/G]TATTCTACATTATTCCTTTGCCTTTTATAAATATCTTTTAAAGGAGGGAGATCATGT	A	G	0.62	0.32	0.33	0.36	0.36	0.80	0.68	0.53	0.46	0.34	0.26	0.042	0.077	0.018	0.126	0.0028			
rs985343	2	76.89	GGAGCAAGTCTTTCTGGAGAACCATGACATTTTCAACCCCAGCAGTGTCCATACAA[A/C]ATGACTGAACTCTTATGTTTTGTGACAGAATACACACTTACATAGAATTCTAAATC	A	C	0.43	0.26	0.22	0.28	0.21	0.23	0.13	0.35	0.18	0.18	0.26	0.028	0.002	0.013	0.005	0.0020			
rs977744	2	77.01	CAAGGAATTTAAAATTTAAGAGGTTTAGTTCTAGTTATTACACAACATTACACAT[A/C]TCTTACATTTCATTGACACATACTGTGGGTTATTCTTGAGTAAATGATTCAATGAGAC	A	C	0.40	0.67	0.64	0.59	0.66	0.27	0.21	0.43	0.54	0.65	0.59	0.023	0.063	0.007	0.037	0.0012			
rs1929452	2	77.09	AAAAATTTATTTGGTAAAGAATCATTGACTCAGATGAAAATGTGATCCATGAATT[A/G]GAACATTTTATTTTGGGGATTTTTAAGATTTCTCATTGATTTGTATTGGAGTTGA	A	G	0.24	0.29	0.27	0.29	0.29	0.80	0.76	0.42	0.38	0.28	0.30	0.005	0.082	0.000	0.094	0.0003			
rs3820749	2	77.30	GAGAAACAATTTATACTGAATGTTAAGTAATATAGGGAGAACAATCAGACCAACATGT[A/G]TGAATGTCTGTTTTGTAGTAGAAACTGAAGAACCAATTGTTTGGGTTGATAATGATT	A	G	0.00	0.13	0.15	0.18	0.16	0.09	0.00	0.05	0.25	0.20	0.20	0.055	0.070	0.017	0.010	0.0015	1		

rs870560	2	99.94	CAGTACTCCAAAAGCAAGTGTGAGAGATGCCTATCTAGAGATTATTGTTGCACAG[A/G]TTAGATTAGGGTTTTCATGCTGGTGGGAAGTTCAGAGATGGGCTAGCTCTTGAAA	A	G	0.43	0.58	0.48	0.59	0.52	0.05	0.40	0.48	0.46	0.47	0.47	0.000	0.003	0.000	0.118	0.0001			
rs1519662	2	100.77	CTCCTCCTTGCACCTGCAGTGCTATATGATGATGATCTGTCTGTGCCTCTTAGTCTTGTAGCTCCTTAATAGCAGGATTGTTTATCATC[A/G]TGTTCCCAGCTGGCCCAAATATT	A	G	0.95	0.53	0.53	0.43	0.37	1.00	0.95	0.81	0.57	0.47	0.51	0.150	0.175	0.071	0.237	0.0002	1		
rs1567804	2	100.97	CCACTCCCTCTCAATACACCCTGCTTATTTCTCATTAACTGGATCTGAAGGCAGCC[A/G]AAATATCGATTAACTTAGGGGTTAATTTGTGTGGTTCTGATTAGTCAAGATTTTCTT	A	G	0.69	0.26	0.32	0.33	0.30	0.61	0.65	0.44	0.36	0.32	0.37	0.076	0.065	0.013	0.051	0.0053			1
rs882272	2	101.20	TGATAGTTTCATATCTTCTCAGACACCTGTCAGACTTGTTTTTCATTTGTTTC[A/G]AGAAGTGCCGAGAAAGCTATTTGTCACTTCCTTGTGGACTATTAGTATGTTTTCCTCCAG	A	G	0.19	0.34	0.45	0.45	0.34	0.05	0.06	0.27	0.39	0.48	0.51	0.045	0.098	0.008	0.112	0.0014			
rs1024794	2	102.37	TGCCAGCCAGGATTGAGTAATAGGCCCTAGATTTACTTTCTCATAAGAAACAACTAA[A/G]TTACTCTACAAAATATGCAAAACCAGGGTTTTTAAGAATTGGACATCAGCTAAGGA	A	G	0.88	0.64	0.66	0.66	0.70	0.55	0.49	0.79	0.57	0.78	0.71	0.050	0.007	0.012	0.002	0.0018	1		
rs1558622	2	102.55	CTTCCTGGTTTTCTGGGAGTGCAGAGGCAGACAACTGCAAGGGAGAGCCCC[A/G]CAAGGATTTGGGTGATGACTTTGAGCCAGGCTGACATCCATGTCCAAGGGCAAATGCAC	A	G	0.45	0.54	0.49	0.54	0.54	0.36	0.21	0.33	0.57	0.62	0.52	0.001	0.063	0.015	0.015	0.0000			
rs759382	2	102.72	CTGCACTATCTTTATGTAGCCTGCATTCAGCCAAAGGACCTTGTCACTCTGAAGG[A/C]CTATTACTTGTCTTCTCATTCCGTTGTCACTGCTTGGATTGTCCTTTTCCTTGGTCC	A	C	0.98	0.79	0.75	0.82	0.76	0.50	0.25	0.45	0.86	0.76	0.76	0.047	0.169	0.045	0.056	0.0007			
rs1051783	2	102.95	AACCAAAGACTGATCTTAGAATATAGAGTAGAGAGACATGTTTGTTATTCTCCACT[A/C]GTGACTTTAGTATTTTGTTATGTGATGTTTTTTAGGTGCACCTTTTCTCATGACTCCT	A	C	0.64	0.46	0.49	0.43	0.52	0.55	0.38	0.27	0.36	0.58	0.53	0.010	0.010	0.042	0.001	0.0018	1		
rs1022298	2	103.76	CCAAATGAATCCCTGAGGATTTAAGAGAACACACTTGC[A/C]AAGTCACCGTTTTCTCCATCACCAGTCTTTCACCTTTTCACTCAAATGTTTTGTAGGTTTTCAGAGGTAGTTGCA	A	C	0.36	0.54	0.59	0.41	0.55	1.00	0.94	0.74	0.50	0.50	0.49	0.030	0.081	0.008	0.156	0.0048	1		1
rs264963	2	103.88	TCTTCATCAGTATTCCCAGTGTCTCTCCAACTCATGGCTCCACAATCTCCTACAGG[A/G]GTGTGGCTTCTTCCATATGAGGAAATATTTAACTCTTCATAACTTTACTTTTGCCACC	A	G	0.65	0.51	0.47	0.61	0.50	0.14	0.15	0.23	0.54	0.57	0.57	0.021	0.051	0.028	0.057	0.0046			
rs1026220	2	105.13	GTCCATGAACTTACAGCAACCCCTTTCACATGCAAAAGCATTTTATTTTCAGGCTTT[A/G]AATGGAGGTACTAAAAACAGTGTTAAAAGCGGGGAACACTTAAGAAGTGCAACCTT	A	G	0.45	0.37	0.38	0.41	0.39	0.18	0.40	0.52	0.43	0.30	0.31	0.003	0.000	0.004	0.025	0.0034	1		
rs1568261	2	105.76	AGGTCAGTAGTAAAGTAAACAACACACAACTTATATGTTCCAGATCTGTTATTTGTTCAACTCTGGCATGTG[A/G]CAAACAACTTTGATTATAGAATTTTAGGAGTTGATTTTGTTT	A	G	0.33	0.37	0.38	0.30	0.39	0.36	0.34	0.55	0.39	0.45	0.45	0.003	0.002	0.004	0.001	0.0020	1		
rs955799	2	105.96	AGTAGAAGGAATTTGACCACAGAAAGAAGAAAATGTCATGTGATGATGGAGGCAGA[A/G]GGAAGCACAGTCAGAGAAAGGATGTTATACACTGACTTCAAAGACGAAGGAAGC	A	G	0.17	0.35	0.38	0.38	0.21	0.18	0.32	0.21	0.32	0.23	0.23	0.028	0.004	0.018	0.022	0.0149		1	1
rs1448110	2	107.03	ACATGACTGTGTTTAGCCAGTAGAATGTGAGCAGAGGTGGACAAAAGGATATGA[A/G]TGGATATTTGGGGAGCTAGCAGAATGATGGACTAGGACCGAGACTGCTGGCATCAC	A	G	0.26	0.41	0.47	0.49	0.39	0.27	0.51	0.44	0.54	0.55	0.54	0.016	0.006	0.001	0.015	0.0029	1		
rs726000	2	107.23	TTCCACCAAAGGATGTCAATGTGAACAGTGACACTGCCCATGGCTCTACCCCAC[A/G]TTTGTGAAGAGACCTGATATAAATGCCTGTGGTCTAATATTTCCTGACAGCAAAGAT	A	G	0.33	0.43	0.42	0.43	0.43	0.45	0.56	0.51	0.36	0.46	0.51	0.000	0.026	0.023	0.007	0.0058			1
rs972881	2	107.64	ATAACAGGTAAAATGTTATACCTAACAATAGCTTTGAAATATGTTGGTGGAATAT[A/C]ATGAAGTACATAGTTTGAAATGTCAGTTAAATAAATTCAGGCTAGAATCCTTGTGTGA	A	C	0.07	0.33	0.28	0.24	0.23	0.55	0.72	0.28	0.32	0.27	0.28	0.039	0.101	0.000	0.040	0.0000	1		
rs266209	2	107.69	AGATGCCTACAGGTTGTCAGACACTATCAAGAGAGGCA[A/G]GCCATAGTAGGATGAAGGATGAAAGAGAGAGAGAGAGATAATACCACTCAGTGTCTGACATCTCCAGCTCTT	A	G	0.29	0.29	0.35	0.39	0.36	0.18	0.09	0.43	0.21	0.31	0.33	0.003	0.045	0.003	0.021	0.0006			
rs931326	2	108.36	TATCAGAATTTGCACTGCTCCACACCCATCTTGCCACAGGGAAAAATCTACAGAA[A/G]TCTCTATTTTGGCAGCTTGTTTCAAGTTTTGAACCTCATTTGATAATTTGTATTCTTTT	A	G	0.71	0.52	0.50	0.54	0.50	0.95	0.76	0.72	0.57	0.43	0.52	0.031	0.066	0.022	0.186	0.0024	1		
rs1003019	2	108.38	CCAAAGTAGTACACTTGTTCCAGTTGCTGAATCTGTATTGACACATTATCACTC[A/G]AAGTCCATAGTTTAGCTTAAGATATGCTTCCAGTTTTGCACATTCTTTGGCTTGTGACA	A	G	0.43	0.55	0.56	0.56	0.55	0.18	0.45	0.57	0.46	0.61	0.56	0.015	0.014	0.002	0.097	0.0012			
rs855004	2	108.39	AATCAAAAGCCAGAATACTGCCTATCTCCATGCATTGCTTTCCAGTACCTTATCA[A/G]GAAAAAAAAAAATTTCCTCCCTCAAGATGTGCTCATATTTTAAGTGGATAAAATATGT	A	G	0.43	0.45	0.44	0.41	0.43	0.67	0.32	0.39	0.50	0.37	0.43	0.001	0.002	0.000	0.034	0.0008			
rs1718031	2	108.41	GTGATAGACCACAGAGATGCAGTAGCAATCGGAGGGTACTGACACTTAGAAATCT[A/G]TGGAGGCTTATTACTGGCTTCCTATGAATAAAATAGATGGGTGGCCAAAAATGACA	A	G	0.24	0.36	0.34	0.41	0.33	0.14	0.27	0.36	0.36	0.36	0.36	0.019	0.012	0.003	0.049	0.0001			
rs1864474	2	109.48	ACTATTAAGGAAATTGAATTTAGAGTGTCGTGATTTGTGAGAACAATGAAAT[A/C]GGCTGTTCATATTCCTGTAGCGTCAGCAGCAGATGAGATGGGAGGTGTTGTCTTTCAGG	A	C	0.93	0.67	0.64	0.67	0.75	0.77	0.96	0.75	0.64	0.63	0.63	0.087	0.107	0.009	0.017	0.0000	1		

rs6745185	2	241.91 CCAGGACCGTACGCGGAGCCTGCTGCCTGACGCTCAGCAGCTAATCCTTTCCACA[A/C]CTCGAACGGCACTTAAATTGCCCTCCTATTTTCACCCCAGCCCTTAGAAAAGCTG	A	C	0.15	0.19	0.25	0.20	0.29	0.00	0.03	0.15	0.11	0.39	0.40	0.006	0.031	0.001	0.071	0.0180		1	1
rs1864868	2	241.97 TGCTGTCTCTTCAGAGTTTGGGGTTGCAGTATGCCCTGCAAGCTCAAAGCTTTGAT[C/G]GGTGAAAGAAAACACTGATTTTCAGGTTTGTCAGCTTTTCTTGCTGTAAGGATGA	C	G	0.48	0.51	0.45	0.38	0.48	0.50	0.78	0.65	0.54	0.40	0.35	0.000	0.058	0.015	0.001	0.0072			
rs4129943	2	242.07 GTGTGTGAGAGTGTTTTTGCTGTGTCCTTGCTCTGGGTGGTAGTGTCTGGGGT[A/G]CTTCTGTTCCTAAGTAGCTTTCCTGGGAACTCTAAGGATAGGTCACCATGGCTTTCT	A	G	0.19	0.34	0.28	0.28	0.36	0.27	0.17	0.24	0.32	0.23	0.24	0.014	0.017	0.006	0.003	0.0018	1		
rs4293564	2	242.16 GGAACCCCCGTGCAAACACACACGACACGGAACACGTCATCTGTCTTGTTACAGGC[A/G]TTTGGAGCTGGAAGACCTAAGTGAGGTCTCCCGACTTCTGTGCCCCAACACACT	A	G	0.21	0.23	0.26	0.35	0.29	0.20	0.61	0.43	0.43	0.32	0.30	0.006	0.001	0.003	0.001	0.0003			
rs4430951	2	242.22 GACTAATGCAGACGCTCCACACTTCTGAGAAACCCCCAGGGTTTAGAAGCTTCTCC[A/G]AGGATCCAGGACAAAGACCTGATACATCTTCTGTTATGCAACACCCTCCAAAG	A	G	0.79	0.68	0.66	0.60	0.64	0.77	0.69	0.59	0.75	0.61	0.58	0.010	0.000	0.000	0.006	0.0039			
rs4675833	2	242.27 TCAAGGGTAGGGTTTTACAAAACAGACTTCGGGTTACCATCTCATATGGGTCTCT[A/G]TGTTGCCTATTTCTTTCAATGTTACGCCACGAGCATTTACCCATGCCCTAACTTTTCT	A	G	0.95	0.68	0.75	0.65	0.75	1.00	0.86	0.71	0.71	0.82	0.73	0.061	0.029	0.001	0.112	0.0001			
rs6437235	2	242.34 CTGCCCATCCCCAGCAGGCTCTCCTGGTCTCGTCCTGTCATCTCGTCCTTTTTC[C/G]CTTTGCACTCACCACCTGCCACACCACACCACACTCTCACTGCTGTCTGTTGTCT	C	G	0.71	0.91	0.93	0.84	0.88	0.77	0.94	0.95	0.71	0.92	0.91	0.048	0.000	0.001	0.024	0.0000			
rs2305071	2	242.50 CCTCCCCAGCTTCGTCCCCATTCTCCTGGACAACTGGCTCTGTACTGTGAACTA[C/G]AGAGAAAGGGGAAAAGGGACAGCTGACAGCTGCTGGAAGAGACCCCACACCTTG	C	G	0.02	0.14	0.13	0.10	0.16	0.09	0.00	0.08	0.14	0.11	0.12	0.031	0.055	0.005	0.004	0.0004			
rs4675966	2	242.57 TCTATGTATATGCTCATGGTAACACAAAAGGCATTAATATCAACTTTTTATAGGAGA[A/G]GAGACAGGCCCAGGAAATCAAGATCTGTAATTATTTCTTCTTTTTGTTATCTTGGC	A	G	0.58	0.50	0.45	0.44	0.46	0.14	0.13	0.35	0.39	0.52	0.45	0.015	0.055	0.003	0.050	0.0004			
rs3821280	2	242.66 CTACCTTATTACACTCCCATAAAACCCATTCAGGGTAAGAGTTGTGTTTGTTCTTCA[A/G]TTGAGGAAGCCGAGCCTAAGCATACCCACCAAGTGGAAACCAGGATTCCTACTCC	A	G	0.83	0.89	0.91	0.91	0.86	0.91	1.00	0.92	0.89	0.89	0.90	0.003	0.041	0.000	0.001	0.0001			
rs1995496	2	242.77 GGATGTAGCAGCAGCTGTGGCCTGGATGATCACTCGGGCCACTCAGCTGACTCG[A/G]GCGGTGGACACCTGTTCTCCACATTAGCCCTGGGGCTGCCAGGTCTGGGGCCTC	A	G	0.45	0.58	0.53	0.48	0.61	0.65	0.21	0.66	0.64	0.52	0.44	0.002	0.059	0.010	0.008	0.0071		1	1
rs4247179	2	242.91 AAGTTGGCTTCCTTGCCTATAAAGTTTGGTGATAAACCTAAGAAATACACCTGCT[A/G]TGCATTTTACTGCGTCCGTGATGAAAATCCCTCAACTTCACAGCCGCTCAGGCCTCC	A	G	0.48	0.53	0.45	0.49	0.46	0.91	0.56	0.73	0.54	0.33	0.43	0.000	0.006	0.050	0.125	0.0010			
rs1106639	2	243.01 GGAGCCTCTGCTCACTCTGCCTTCCTTGCTCACTTCTAGAGAGTCCGTTTTAC[A/G]TCCTCATCGAGACTTCAGGCTCCAACGCAGGCCATGACGCTGAGAAGCTGGGCCAC	A	G	0.31	0.25	0.23	0.23	0.20	0.23	0.14	0.14	0.18	0.32	0.26	0.007	0.001	0.003	0.003	0.0007			
rs16747	2	243.08 GACACTGGACTGGTCCTGGAAGGTGACCCCCTCCTGGCGTTCAACTCTGCGGGGAGAGGCCCTACCTCCTACTCCTCCAGCAGGAAGTTCAGAGAGCAC[A/G]GCAGACCACT	A	G	0.38	0.39	0.39	0.37	0.46	0.30	0.28	0.52	0.39	0.37	0.46	0.005	0.030	0.002	0.021	0.0016			
rs1516337	3	0.17 CTGTTCAAAGAAGGAATGGGCCATACTACGAGGAAGTGTTCACATTGGCTAAATACTTCTGTCAAAAATGTTAGGGATTAA[A/G]TGGGGCATGATGGACAATGAAGGCAGCTCA	A	G	0.24	0.34	0.33	0.46	0.32	0.23	0.08	0.36	0.29	0.31	0.33	0.004	0.045	0.000	0.004	0.0000	1		
rs1516350	3	0.17 CACAGATAAGAGAAAAAATTTAAAAATAACATAAGTATAAAGTACTTTAGAAAGGT[A/G]GGAGTGCAACAAAAGAAAATGAACCGAGTGGGTGCATTAATAAAGGTAAAAGTG	A	G	0.31	0.27	0.33	0.21	0.29	0.36	0.36	0.49	0.29	0.30	0.33	0.000	0.002	0.029	0.001	0.0000			
rs1516348	3	0.18 AACTATAAGAGAAGCTGGTAGGAATTAACAGGTACTGTAGATTTAAGTGAGGC[A/G]AGGGAGCTGGACTAATGAGTCCCATGAAGTCCCATTAACATATTTTATTTAGAATTTAT	A	G	0.60	0.56	0.54	0.54	0.57	0.77	0.92	0.69	0.75	0.51	0.49	0.003	0.109	0.013	0.034	0.0005			
rs1850264	3	0.20 AAGTGGTAAAGTAAACTTACATAAAAGTCATTAAGAATTTAAAAGAAATGGGAA[A/G]ATATACACAGGCACACAACTACCAACCAGATCCATTTTGTGAACCTCAGACTTTGCAAC	A	G	0.36	0.40	0.35	0.29	0.43	0.55	0.61	0.15	0.46	0.32	0.34	0.000	0.032	0.022	0.019	0.0002			
rs902118	3	1.16 GGTTATAATACACATAGACCCAAATACCACAGATCTCCAAATCTCTAACACCCA[A/G]AAACAGCCCCTACTTCCTTGGGCTAGAAAATTATTTAAAATACCCAATCCTCCGGAGGG	A	G	0.29	0.49	0.47	0.50	0.52	0.55	0.48	0.35	0.71	0.44	0.44	0.014	0.000	0.000	0.006	0.0006	1		
rs1551995	3	1.38 AAAAAAAAAAATGGCCCTAGAGCATAAGTGACCACTGAGCTAATTTAGAGAGAGAAT[A/G]CCTAAGAAGAAGAGTAGCCTAATTGCTTCTTTTACGGTCAGAATAGAGTCAAGGT	A	G	0.15	0.38	0.46	0.48	0.39	0.09	0.25	0.39	0.43	0.45	0.42	0.034	0.007	0.000	0.054	0.0010			
rs1499260	3	1.51 ATTTGTTTAAAACATTATGTGATCACTTTAGAAGAGTA[C/G]CTAGCACTCACAATTTACAAAAATAAAATACAATCAGCTGTCATGACATCAACTAAAGATAACAATCTGGTATAT	C	G	0.64	0.84	0.77	0.80	0.71	0.73	0.83	0.77	0.86	0.78	0.68	0.003	0.009	0.000	0.000	0.0067		1	1
rs968057	3	2.37 TCCTGGATTACTCTAGCACAGCCTAAATAATTCTGATTAATGCAGCCTCCCAAC[A/G]CATGATATAGTCTGCAATGAAAATGGCCCAAAATATATACCTTAATTTCTGTACAATGT	A	G	0.48	0.42	0.47	0.46	0.48	0.82	0.36	0.55	0.54	0.55	0.53	0.000	0.003	0.007	0.077	0.0000	1		
rs1024147	3	2.71 TTTGGAACACGTGGGATCTTTTCTATTTTTCCTTTTGCATCTTTTTTAGTATGATA[A/C]AAATTAGGATTAAATTTATCTTTCTGAGGCCTGTTGCTGTTTATTTCTTTGACTAAAAT	A	C	0.67	0.49	0.47	0.58	0.46	0.36	0.63	0.80	0.79	0.48	0.47	0.018	0.006	0.042	0.008	0.0000			
rs1153459	3	3.13 TCAGGAGTTTCTCAAGAGGAACTCCACACTCACCACCATCAAATCAGACAGTCCA[A/G]GTTAAACAATTGTACTGTTTTGTGAAAATAATAATTATGACAATCATGGAATTAAGGT	A	G	0.60	0.42	0.48	0.54	0.48	0.77	0.71	0.68	0.32	0.53	0.42	0.007	0.029	0.019	0.060	0.0033	1		
rs1508729	3	3.52 GGCAGCAGAAGCCCATCACCATCATATATCAGCCTCCAAGCCAATAACTGGCCTGCA[A/G]AAATAATAACTGTACTCCCATGCACCTGAGCCCACAGGGAGAAAACTCATTTTA	A	G	0.95	0.87	0.80	0.82	0.73	0.86	0.76	0.89	0.79	0.84	0.86	0.041	0.000	0.017	0.005	0.0035			
rs1488403	3	3.66 TGAAGCCTAGCTCCTGTAACTTGTTTCAXAACCCAATGCTGAAATTGCTGTTGCT[A/G]CCATTGTATTGTAACAGAAGGGAAAAGAGAGAGTGGGTTAGGATTTTATATCTTCTA	A	G	0.10	0.38	0.39	0.46	0.30	0.35	0.15	0.25	0.39	0.41	0.35	0.056	0.029	0.004	0.000	0.0003			
rs795734	3	4.08 AAAGCAAAAATATCTTGCCTGAAGTCACACAGCTGGAAGAGGAAGCTGAGGTT[A/C]AAATCTTCCATTCTAAAACTTGTGTTCTTCCCTAGGCCATTAGCACCACATGGAGGAA	A	C	0.71	0.65	0.71	0.66	0.66	0.55	0.25	0.67	0.68	0.66	0.68	0.000	0.131	0.000	0.023	0.0001	1		
rs902982	3	4.57 CAGATCTTTCTAATCTCTCAGCGTTGCCAAGAAGTTGTCCATGCTTCTAATCTTTATCT[A/G]GAGAGATTATTCCATGTTTTAATTATAAAGGGTTGAGTAGCTGTTTCAGGGCTGACGTTG	A	G	0.62	0.39	0.42	0.33	0.48	0.55	0.09	0.47	0.31	0.45	0.51	0.037	0.047	0.007	0.019	0.0090		1	1
rs1435359	3	5.78 ATGTTTCTCCACCTCTACTTTCTGTGCCTCCTCTCCTCCTATAACGTTAATCC[A/C]CCTTCCCAGCACGTTTGGACCCAAGTGCAAGGCTATTTTGGCACTTCTTTAAAAGACA	A	C	0.60	0.22	0.26	0.26	0.20	0.09	0.13	0.34	0.11	0.38	0.35	0.071	0.019	0.011	0.022	0.0119	1	1	1
rs1504034	3	6.11 GTTCCTGGAAGGGGAGAGCTACAATGTTTGTGAGTGCCTTCATAAGTGCTCTAGG[A/G]CATAACCAATAAGCAGCCATTACGTCTGTCCTGAGTTGGAGAAAGAGGATGTT	A	G	0.07	0.16	0.16	0.15	0.23	0.18	0.29	0.11	0.21	0.14	0.13	0.001	0.038	0.000	0.010	0.0006			
rs465152	3	6.89 ATGATAAAATAGCTTTGG[A/G]TTTGTTATGAAAAGTGGGTATGTTCATGGACTTCATGGTTCTATATAGATATGTAGCCATCAACCTGCAGCTACCTGCCATC	A	G	0.45	0.56	0.54	0.51	0.50	0.82	0.77	0.53	0.82	0.58	0.54	0.001	0.041	0.006	0.067	0.0016	1		
rs502758	3	8.11 AGAAACTGAAATAAAATGAAGAAAAATAAAGAAATAAAAATAATATGATGAAGAGAGACAGATGTGCATGACACTTTCC[A/G]ATGAGATGGTAATGGAAAAATCTCTGAG	A	G	0.05	0.26	0.29	0.28	0.29	0.36	0.45	0.29	0.07	0.32	0.27	0.060	0.009	0.000	0.001	0.0000	1		
rs463659	3	8.19 GTAGCAGACATCTTTTAATCACGCAGGCTCTCATGCTGGGCACAGACTTAAATC[A/C]ATAGAAGGAAAAGCAGTTCTGTATCCTCTGCTGACCCCTGCTATGAGCCCCTAGCC	A	C	0.79	0.42	0.41	0.33	0.41	0.95	0.47	0.64	0.43	0.41	0.39	0.068	0.000	0.012	0.166	0.0001			
rs164466	3	8.72 TCCAAACAGGCATCCCGCAAATAAATAGCAAGCTCTGTTGCTGCCATAGGTC[A/G]CTGCAAAAGAAACGACTGTTGGAATCTAACTTCCTTTACTACCTTTTGGGACACCCTCC	A	G	0.43	0.60	0.61	0.46	0.70	0.59	0.24	0.68	0.64	0.57	0.56	0.012	0.080	0.000	0.000	0.0007	1		
rs1005989	3	8.87 TGCTGGACAGAATTCTAAGGTAGCCCCAAGATTCCCAGCTCCCGATGCACA[A/T]GCTCTGTAAATCTCCTCCCATTGGGTCCCCAGCGGTCAGGCCCTGGGACATGAT	A	T	0.28	0.41	0.38	0.30	0.36	0.64	0.45	0.20	0.46	0.30	0.31	0.010	0.001	0.027	0.025	0.0045			1
rs749477	3	10.63 TGGTGGGATCAGAGGCCAGAGGTGAAGGCAGGAAGCACCCTAAACCACTATT[A/G]TCAGGAGAGCTTGGATTTTAGCTTCAGGACATCAGGAAGCTGCTGGAGGGTTACAG	A	G	0.45	0.60	0.52	0.59	0.61	0.14	0.46	0.36	0.32	0.66	0.56	0.004	0.004	0.013	0.105	0.0010	1		
rs892605	3	10.68 TACCTGAGTCCCCTGGGCACGGAGTCCCCAAATACACTAACTCAGAAGTGCTATC[A/G]GTTTTGGTGGGTCAGACATTCCCTCTCCCCTGGCCCTACAGCTCCCTGCTACTAC	A	G	0.85	0.71	0.74	0.71	0.64	0.86	0.81	0.74	0.71	0.64	0.66	0.002	0.000	0.001	0.004	0.0079		1	1
rs1039202	3	11.71 ATTTGAGGAGAATAAATAGTATGAAAGAAAAAGCCCAGAGTAAGCAGACTTTGGGG[A/C]GCATAGCAGAGCCAAAAACATGTTAGTCGGTATCTTCAAAGAGATTTGAGCTGG	A	C	0.21	0.64	0.63	0.57	0.61	0.23	0.47	0.45	0.57	0.58	0.67	0.065	0.003	0.008	0.070	0.0020	1		
rs709160	3	12.50 TGTGGGGGTAGTCAAGGAAAGAAGAAAGGGGTAAGACAGGGAGTTAGGAATT[C/G]GCGGGGCCCCTAACGCTGTTGGGGTGCGCAGTTTATTTTATTTGCAGCTGTCGTCT	C	G	0.67	0.51	0.52	0.50	0.48	0.18	0.16	0.23	0.43	0.47	0.48	0.002	0.101	0.081	0.090	0.0031	1		
rs299629	3	12.55 CTATAACCGAGGCCCATTTCTAAAGTTGATCTTAAAACTGGAAAATTTGAGTCT[A/G]TCTAATTTTGTGAAATATAGGGATGTAAAAGTCATATTTGTAAAAT	A	G	0.43	0.62	0.62	0.70	0.59	0.86	0.90	0.86	0.79	0.65	0.64	0.004	0.091	0.072	0.071	0.0025			
rs904453	3	12.68 TGTGGGACCCGGGCTTGCACCCTTCTCTCAGGGTACTTCAGTCAAGTGACAC[A/C]CTTTTAGAGACGACGTGAGGAATCGGGTAAGAGAGGAGGAAACTGGCCAGTGCCCTAC	A	C	0.38	0.49	0.41	0.48	0.32	0.86	0.51	0.76	0.61	0.48	0.50	0.002	0.014	0.068	0.136	0.0076		1	1
rs357116	3	13.30 GCAGGTGCTTAATAAAAGTCTCCAAGGGGATTGGTTATCCCATGTTCCTT[A/G]GGCATAAATCACCTTGCTCTGCAGATGGGCAGTGGGATACATCCAATCCATGGTTTGCTGA	A	G	0.74	0.44	0.47	0.44	0.48	0.50	0.33	0.52	0.46	0.49	0.51	0.033	0.021	0.000	0.000	0.0008	1		
rs729639	3	13.80 AAACAGCCCAAATGCCCATAAATAGGGGACCAGCTAAATAAATCACAAACTATCC[A/G]TACTCTGGGACCCCTCACAACCACTAAAATGAATGGGGGAGGGCTGTGAAATTTGA	A	G	0.12	0.36	0.36	0.41	0.27	0.64	0.75	0.27	0.25	0.30	0.36	0.048	0.077	0.004	0.037	0.0005	1		
rs353087	3	15.17 CAACTTAGTTGTGTAAAATAATCATTTTGTTATGTTCACAGACTCCGTGGGT[A/G]GGGACACTGAAAAAGAAGCAAAAGAGGGGCCTCTTTCTTCCATGCCTGGGGTCTCAGCT	A	G	0.31	0.57	0.62	0.61	0.50	0.45	0.39	0.38	0.57	0.57	0.56	0.063	0.035	0.031	0.022	0.0051	1		1
rs1106130	3	15.31 GTTTCTTCCATTTAAAACTTTCATGGGCATGGGGGGAGTTTTCTACCTTCTCAT[A/G]GAGGATGGAGCACCTGTTTGCTTCAGTTAAGGCAATGATGGGCTCTGGTTAGGAAGCCTC	A	G	0.81	0.70	0.77	0.76	0.70	0.59	0.42	0.73	0.82	0.81	0.77	0.000	0.085	0.004	0.038	0.0007			
rs826423	3	15.32 ATTTCCTACAAATAAGGACCTTCTCTCACATAATAATAATGTAACCATCAAAAGT[A/G]GAAAATGCATTGATACATGACTATCATGTAATTATCATTATTTCTTTTTTTTGAAAAAAA	A	G	0.74	0.39	0.50	0.52	0.48	0.60	0.38	0.51	0.57	0.54	0.52	0.028	0.010	0.005	0.004	0.0000			
rs905946	3	16.23 AATCAAAGTAAGTGATAGGACATTTCCAAAGCATGAATTAATGGAAATGCTCAA[A/C]AGAGAGAGAGAAATAAAAGGAGCCCTCTGCTAAAGCTCTTCTCACTGACCAATAAGGGT	A	C	0.83	0.66	0.64	0.54	0.48	0.73	0.95	0.69	0.57	0.68	0.65	0.022	0.087	0.002	0.006	0.0000	1		
rs895941	3	16.82 CATTGGCTCCTCCACATCTGGATGCAGAGCAATATAGGCAGCCAGGCATTTAGTCA[A/C]AGGAGCAAGGAAAACAGAATGAAAGAAATTTTAAAAGCTAAATCTCCTCAAATT	A	C	0.40	0.73	0.65	0.74	0.66	0.82	0.61	0.81	0.71	0.82	0.72	0.019	0.001	0.027	0.032	0.0067	1	1	1
rs1441035	3	16.98 GGTTCTCAAGCTGGAAGAGACACTGTAGTGGGACATCTGGTTGAAAAAGTTCT[A/G]AACACAGATGGGATATGGTATTGACGACCAAGTAGGTGTTCAGATAGGACCACAA	A	G	0.21	0.49	0.47	0.41	0.41	0.32	0.27	0.35	0.32	0.43	0.49	0.032	0.017	0.009	0.009	0.0002			
rs1375818	3	17.25 CATCTATCTTTTCTTAATTATTATTTCTGATATTCTAAAGACACATGTTTGCATGCAGGTACCCAATCCAACAGCGTTTGACTTTTGTGGTCA[A/G]GCTAGAGAATGTCTATTCCA	A	G	0.38	0.58	0.54	0.59	0.48	1.00	0.96	0.68	0.54	0.47	0.55	0.005	0.155	0.041	0.230	0.0011			
rs1374197	3	17.37 AATCACAGTCCAACACAGACATATCATTATTTGGCTCTGGGAAAAGAAAATCC[A/G]TGGCACTGATAAATGAAGTTACCTGCAAGAGACTTGGTATTACAATGAATTTGGTTAAC	A	G	0.24	0.55	0.52	0.56	0.44	0.77	0.94	0.60	0.46	0.48	0.53	0.032	0.132	0.016	0.048	0.0010			
rs717939	3	18.60 TGAACATTAGAACACAGTTTTATCATTTTAAAAATCATATCATGGTGTAGCCCCTGAGTTTAGCAGTAGCAGCAGGAAAAGAC[A/G]CATCTTTGTAAAGATAGTGAGGCAGACT	A	G	0.79	0.72	0.73	0.74	0.89	0.73	0.31	0.81	0.93	0.75	0.71	0.001	0.097	0.001	0.001	0.0000	1		
rs336610	3	18.63 TTGGAAAGGAAAATGTTTCCAGTCTCCATGAATGCCTGGGGAGGTGTTGCAG[A/C]GTTGAAGACAGTGTAACTGAGCTCTGCAAGTTTGGAGTTGCTTTTCTGAGAAACT	A	C	0.53	0.67	0.65	0.60	0.61	0.18	0.48	0.55	0.61	0.48	0.63	0.012	0.023	0.012	0.142	0.0000			
rs396506	3	19.84 TCATCTTATAACGCTTTCCAATTCCCAAATACCTTTAAGGTTT[A/G]GTAAAAATAATATACCTTCCACAAGTCATTTGATTTTTGTGGGCCTTAGTTTATCGACCTATTTGGATAG	A	G	0.17	0.42	0.40	0.40	0.43	0.09	0.18	0.36	0.42	0.34	0.39	0.048	0.032	0.002	0.068	0.0000	1		
rs269384	3	19.89 TCCTAAATTTGGTGGGAGCTATGAAATCTTGTATGCCTTGTTACATCTTGACTT[C/G]CTAAAATTGAAGCTCAGCCCGCTGAGTCACTTCAGTTAAGTGACTATGAGTTTAAGA	C	G	0.86	0.92	0.86	0.87	0.88	0.91	1.00	0.74	0.93	0.84	0.84	0.001	0.065	0.003	0.008	0.0007			
rs1356793	3	20.36 GCACACAAAAAAGAAAAATGGAAGGAATGCCCAATTCTTATGCATTCTATAGGTCAGGCATAACTCAGG[A/G]GTTTTGCAGAAATTTCAGTGATTGTTGCTCACATGAGGTT	A	G	0.81	0.48	0.51	0.41	0.64	0.55	0.70	0.67	0.61	0.48	0.56	0.029	0.003	0.002	0.003	0.0004	1		
rs259560	3	21.90 ATTTCAGGTTCCAAACTTAGTGATCCTAATTATTTCTTGCAAAATGGTAACAA[C/G]CAATCAGATATGTCTTATCCTATGTCTCCCTTTTCTTTGCAAACCATCCGCATTATATTG	C	G	0.52	0.57	0.56	0.50	0.59	0.45	0.69	0.71	0.46	0.60	0.48	0.004	0.002	0.006	0.013	0.0031	1		
[illegible]	[illegible]	[illegible]	[illegible]	[illegible]	[illegible]	[illegible]	[illegible]	[illegible]	[illegible]	[illegible]	[illegible]	[illegible]	[illegible]	[illegible]	[illegible]	[illegible]	[illegible]	[illegible]	[illegible]	[illegible]	[illegible]	[illegible]	[illegible]
rs1405796	3	39.66 AATTCTGCCCATTATGGCCCAAAGATCCTATATGGAAACCCCAC[A/G]TCCCTACTTGTGTTCCCCACAACCCTCTCTTATAATAAGAAATATTTATTTTGAGGTTAG	A	G	0.03	0.16	0.13	0.10	0.15	0.00	0.15	0.08	0.07	0.14	0.10	0.042	0.003	0.016	0.078	0.0031	1		
rs1405793	3	39.72 TGCCAGAAATGATAAATACATGGTTAAACAGAAAAGCTTTTCTCATTCATAACAGA[A/C]CAAGAATAATAACAGTGTGGTGTGGGATATATAGCTTATATAAAAATAAAGTGGGT	A	C	0.33	0.83	0.74	0.78	0.59	0.86	0.86	0.73	0.86	0.76	0.72	0.090	0.012	0.000	0.015	0.0009			
rs1996562	3	40.10 AAAAGGGAAATGGTAACAGTCTTGTTCATAGGTTGTGATAAAAATTAAATAATC[A/G]GCTATTAAGACTTATTTCTTGAGCACCTATCATGTACCACAAACTGTGGAAAACAA	A	G	0.88	0.66	0.75	0.73	0.66	0.77	0.84	0.71	0.86	0.71	0.80	0.011	0.019	0.000	0.001	0.0011			
rs749932	3	41.34 AATGCAATCATCATAATTTGAGGAATATAGGACCTTAAATCATAAGACCTGGATCT[A/G]AGACCTGAATTTGCTGTTGATTCATGATGCGGCATGTAGTCTCTCTGAGCCTCAGT	A	G	0.19	0.58	0.59	0.62	0.66	0.82	0.55	0.29	0.57	0.53	0.52	0.109	0.004	0.102	0.014	0.0056	1		1
rs1495704	3	41.46 ATTTACAAGAAAATCTTTTACAGATTGTCATCAAGGTATCCTGTAA[A/G]AGTAACTATCAACTACATCTCAAAGAATTACAAAAATCTACCACTATGGAAACTGAAATAAGAAACT	A	G	0.50	0.54	0.55	0.55	0.52	0.36	0.53	0.26	0.50	0.46	0.47	0.006	0.034	0.028	0.020	0.0061			
rs1995137	3	42.09 CCTGCCTGTGGCCAGCGCTCAGTGAACATTTGTTGGGTGTATCAATTGTGTC[A/G]ATTTGTGCAGTGAGTACTATTATCTCTCTTGTGTAAGTGAAGAGTCTAAATCTCGAAAG	A	G	0.39	0.43	0.40	0.41	0.45	0.64	0.29	0.33	0.32	0.39	0.41	0.000	0.007	0.000	0.023	0.0001	1		
rs477078	3	42.33 CAGACTTGCTGTAGGGATCAGAATCAACTACTGCCACCTTAGTTGACCCATAGAC[A/G]TGCAGTTAGGAGCCACAATAGCCACCCTGGCCACCACAGCCTAGAAGTGGAGGCA	A	G	0.73	0.44	0.35	0.39	0.39	0.59	0.44	0.51	0.46	0.33	0.27	0.085	0.007	0.011	0.036	0.0034			
rs697342	3	42.44 GGGCAATTAGTTTTCATAGAAAGGAGAGGCTTCCCATTTACAGAGTTGCAATC[A/G]CAGCAGTTTTATTTTTATGAACCTTTCATTTCCTCAATCTCCAGATTAATTTTTGCAATG	A	G	0.88	0.55	0.64	0.59	0.64	0.50	0.42	0.38	0.39	0.55	0.65	0.048	0.017	0.032	0.009	0.0001			
rs339681	3	42.76 AGGACTTTGGACTTGATTGTCCTGTGTTATGTATATACCCATTGAGCAATTC[C/G]CAGATGGCTAATAAATAAGAAAGTATTATAGATGTCACCAATCGGAGAGCAACAGGG	C	G	0.55	0.18	0.22	0.20	0.20	0.41	0.41	0.34	0.21	0.23	0.21	0.086	0.057	0.040	0.048	0.0000	1		
rs1351631	3	43.48 CTAAGTTCCACAAAGTGATCTAACTCAAGATTTCTTGGGAAGATGGTGAAGTA[A/C]GGGAAAAAACCGATAAAAACTACAAAGAAAAGGAAAGTAAGGAGAAACTCCATAAA	A	C	0.73	0.42	0.42	0.44	0.46	0.18	0.43	0.39	0.29	0.48	0.51	0.032	0.003	0.009	0.058	0.0035	1		
rs737516	3	43.52 AGTAAGCACCTCACTACTATCCTTGACTTCAGTTTCTTTCTCTCCAGTCTAAT[A/G]TGTGTGAAGCACGGTTCTGACTATATCCCTTGCAAGCTGAAAAAGCCTTTGCTGGTTTC	A	G	0.70	0.42	0.42	0.44	0.46	0.18	0.42	0.39	0.29	0.48	0.51	0.028	0.002	0.007	0.054	0.0039			
rs1013758	3	43.59 AATGAAAATCAAATTAAACGTAAGATCTGGGGAGTGTTTCTCATGAAAGCCCTG[A/G]CCACCTTTCAGTCTTGAGGGGTATCACATTGGGCAGACTAAAGCCTCTCCTCTCAATT	A	G	0.11	0.57	0.57	0.56	0.54	0.73	0.25	0.58	0.71	0.51	0.48	0.108	0.027	0.005	0.028	0.0037			
rs954282	3	44.71 TAGCCTTCTATTCAGTGCCCCCCTAAATGCATACACTTGTATTTCTTTCGTAG[A/G]TATTGTTAGGAAGATACACACAAGTAGCATCCCACCCAAGGACAACTGTGAAAGACCCA	A	G	0.53	0.51	0.57	0.48	0.61	0.95	0.67	0.72	0.57	0.56	0.60	0.004	0.000	0.002	0.080	0.0002	1		
rs2056321	3	45.04 GAAAATGCTTTTTGGATGAGGACAGGCAAGCCACACAGCTAGGCCAAAGGCAA[A/G]TTAGGAAACTAGGGCTTCCTCTAACCTCTAGGGTTTTCTTAAACCAGGTTGTCAGTGG	A	G	0.28	0.48	0.59	0.59	0.43	0.05	0.45	0.26	0.38	0.51	0.49	0.051	0.008	0.052	0.200	0.0052			1
rs1860264	3	45.88 GACACCGCGACCTTCAGGGCTATTCATGGTAACTGAATTTAGCAAAAATGACT[A/C]CTTTCTCCTCATTCCTGTCCTTTAAGGGTTTTGATTACTAACTGCGACACACGAAGAA	A	C	0.19	0.56	0.64	0.65	0.72	0.73	0.93	0.41	0.71	0.64	0.58	0.160	0.043	0.044	0.000	0.0049	1		1
rs737452	3	45.99 CCTACGTCCTTTCAGAACCTCTCATTCTTCCCTTAGATATCCAGTGCAAGGTT[A/G]TAATGTTTGCTGGCAAAGTAGATAGCAAGACTAGAGTTGGCTGTGGTGGCAGCCGGC	A	G	0.10	0.41	0.39	0.35	0.59	0.68	0.86	0.40	0.57	0.39	0.42	0.066	0.122	0.000	0.041	0.0001			
rs1520483	3	46.47 AAAGATACAGGGCCCAAGGTCTCCCATCTGCCAAGCTATTGTCCAAGCGTGA[A/G]TTGATGTCTCAGGAAGGCCTTTTCTGCCTTATCCCACCTGAAGCCAGTTCTACTCAGA	A	G	0.12	0.39	0.42	0.27	0.46	0.32	0.32	0.40	0.50	0.39	0.41	0.070	0.012	0.008	0.013	0.0005	1		
rs1402152	3	46.54 TGAAACCCACATTGAGTTCTTTCAGGTTCTTCAAACAACCTGTCAGCAGAAAAAGTT[A/G]ACATCCATTGAATAGGTCTTTAAGGTCTTTTAAGAACAAAAAGCAAACTAAGAA	A	G	0.64	0.58	0.69	0.72	0.66	0.73	0.92	0.60	0.42	0.59	0.63	0.001	0.036	0.003	0.001	0.0057			1
rs1014228	3	47.61 ATGCTTGATTTTTATACTACTTTCAGAGATCTGGATTTTCATTTTCCTGCAT[A/G]TTCAGGTTGATTTCTTTATCATCCCTGAATTAAATTAATTTAGGATAGGCTTCATTCTCT	A	G	0.36	0.67	0.67	0.61	0.69	0.55	0.17	0.46	0.82	0.63	0.67	0.049	0.165	0.032	0.012	0.0000	1		
rs319682	3	47.88 CACAAATAAAGGCCTTAAGTATGGTGTGAAGAACGCTAAGGCTGGAATCAGGG[A/G]AAGTGGTTCTGCTACCAGCTCTGCTGGAAACATGTAGGATTTTGGCCAGAGGATTCC	A	G	0.35	0.75	0.71	0.61	0.74	0.64	0.64	0.55	0.79	0.66	0.69	0.059	0.003	0.015	0.002	0.0005			
rs1865741	3	49.32 GTGCAGTGGTGGGATCGTAGCTCACTGTAAACCTTGCTGAGATTTTTAAATA[A/G]GTTATTTTTAAACTTCTTTGGATCTACATTTCCTTATCTTCATTATCATAGACAAATGGGC	A	G	0.50	0.49	0.49	0.34	0.43	0.10	0.00	0.08	0.54	0.51	0.50	0.000	0.216	0.127	0.102	0.0003	1		
rs1049256	3	49.72 TCCTTCATGCTTTCCTCAGCAGAACACTTCCACTTCTGAGGTGAGCTTTTGCCCC[A/G]TGCCCTTCCTCCACAGGTGTTGCCTTTTTATAAAGACCTGATAGCAGAATAAATTGC	A	G	0.40	0.43	0.46	0.50	0.55	0.32	0.05	0.30	0.43	0.35	0.45	0.000	0.137	0.017	0.011	0.0004			
rs7061	3	50.07 TTCTTAATCGAGTTCTTTATATCGAGCAAACATGACTCTTCGAACAGCATCTCG[A/G]TAAGTCTCGTTGGACTGTCTTTTGCTGGTTCTTCCTGAGGCTCCAACACTGGGGCCC	A	G	0.67	0.57	0.55	0.60	0.57	0.73	0.98	0.88	0.57	0.64	0.53	0.004	0.169	0.076	0.019	0.0005	1		
rs1005678	3	50.17 GGTGGGCAGCCAGGGTAAGAACAGGGCATCCAGGCTCCATGCCAGAGTCTGC[C/G]GGCTGTCCCAGATATACCCGGTCACACCCCTATCTGCTCATTAACCCTTTTAAGCCC	C	G	0.45	0.64	0.59	0.61	0.61	0.50	0.27	0.85	0.57	0.64	0.60	0.013	0.047	0.037	0.004	0.0001			
rs11235	3	52.70 GTTTTACATAATGGTTTTGTTAGATTTATTTTTCCATCAGTGAAAACATTTCTTAA[A/G]CACAAATTTCATTTCATTTAAGCAATTTGTAAGCAAAGTCCAGGTCCATTTAGTTTT	A	G	0.69	0.60	0.60	0.65	0.52	0.32	0.75	0.24	0.54	0.63	0.69	0.014	0.010	0.082	0.038	0.0053	1		
rs3617	3	52.79 CTCTTTGTCTCTCCTCCAGACAAAGGAGGCCCTTCTCAGAATCCTGGAAGATATG[A/C]AAGAGGAAGACTATCTGAATTTCATCCTGTTCAGTGGAGATGTGTCCACATGGAA	A	C	0.69	0.51	0.48	0.54	0.45	0.32	0.75	0.28	0.54	0.54	0.59	0.043	0.043	0.013	0.008	0.0088		1	1
rs312482	3	53.48 GAGGGAAAATTGGGTCCTTTGCACACCAGGAATTCTAATTAAGGAAGATAAACAGA[A/T]CCTGAAGGCTATATGCAGAACATATCCTTAAACAAACTGTGTAGTTGGGTTTGAAT	A	T	0.00	0.51	0.47	0.48	0.52	0.27	0.31	0.48	0.71	0.49	0.44	0.183	0.012	0.001	0.015	0.0003	1		
rs893367	3	53.87 AAAGACACGAAGCTACTGTTTACGTTTCCCCTATGGGAAAGCGCATAAAACAT[A/C]CCAGGACTTTCAGGGGCTGCTGATGTAAATTCCTATTAAGAAGTTCCAATGGGAAGG	A	C	0.76	0.40	0.42	0.46	0.50	0.95	0.94	0.77	0.43	0.39	0.45	0.066	0.196	0.087	0.201	0.0006	1		
rs2030395	3	54.56 TAACCAGGTAGAAAACGGGTCCCACAGAGGGCTATAAAAGATGAACTGGTCAAG[C/G]CTCACAGGAAAAGGGATGACTGTGCAGCCCTTTGATCCTCTGCCTCCTGGGAGA	C	G	0.65	0.41	0.44	0.56	0.46	0.77	0.39	0.45	0.68	0.42	0.39	0.013	0.010	0.003	0.031	0.0013	1		
rs920891	3	55.23 CAGGTGCTTTTTAAAAGGCAATGATTTTGTTAGACA[A/G]TAAAAAAAGGTGTGTTCTGGAAGCCCTTTGTGTCCAGGCCCAGATCCTTTTGACCACATGACATCCAGTCACCA	A	G	0.35	0.26	0.33	0.35	0.30	0.14	0.13	0.29	0.32	0.30	0.31	0.004	0.014	0.004	0.008	0.0000	1		
rs1392702	3	56.79 CTGGATCTAGACTATAAAATATGACTTATTTCCTCGTATGGGGATATCCTG[A/G]TGTGAGAAATAAGGAAGGGGTTGGAATAGTTTTGGAAATAGTTGGTAAATATTTTTCAGA	A	G	0.88	0.39	0.47	0.40	0.41	0.86	0.35	0.54	0.21	0.60	0.58	0.111	0.000	0.019	0.134	0.0068	1	1	1
rs536036	3	57.28 TTGTTTTTACATGTGGTTTAGCTCTTTTTGGAAACATGATCTCTTTTCTTCA[A/G]TGCTACATTCATCAAATTTAAAGGATTAAATATTTATTCCTGGAATAAAATGCTTTTGCAC	A	G	0.74	0.65	0.63	0.67	0.61	0.91	0.86	0.55	0.68	0.58	0.59	0.003	0.013	0.010	0.031	0.0011			
rs844438	3	57.93 GGCAACTTCAAATTTCAGCTCATCTTTCTAGGCCATGTCTAGGTGTTCTCAG[A/T]GCGAAGGTTGACTTGGTATGTCCACCAGGGTGAGAAGCAGGAAATATTTGCCCCAAACC	A	T	0.64	0.65	0.60	0.70	0.69	0.55	0.59	0.49	0.50	0.58	0.51	0.000	0.002	0.011	0.006	0.0061	1		1
rs1444185	3	58.64 AATTAATACACAATTTATCTTTTCCTTCAGGTTATTTATTTCTCAGTCAAAACT[A/C]TTGGTTGCCTGCAAGAAACGGATGCTGGCCCTGTTAAGCATAGGGAATTTATTGGAAG	A	C	0.36	0.59	0.61	0.57	0.55	0.73	0.42	0.65	0.64	0.67	0.68	0.043	0.025	0.000	0.004	0.0009	1		
rs1472653	3	59.54 GGAACCCTGGTGAAGCCTAGAACAAAGTGAGGCAACCTACAGGGAGAGCAGA[A/G]AATGATGTGGATTCATAAAGAAAGAGAGACAAGGGAGAGGGAGAGAGTGCTGAGTTC	A	G	0.57	0.41	0.44	0.49	0.30	0.32	0.18	0.26	0.29	0.48	0.53	0.007	0.037	0.016	0.008	0.0028	1		
rs1447971	3	60.42 CTTAATGACATAGCACAAGACATTACTTATGTGTTTGCAGTGATGCTGGTATAAAT[A/G]AACCTGTGCTGCCAGTCATATAAAGTATTGCATATTAGGTACAGCAGGGGTCCCC	A	G	0.64	0.32	0.35	0.29	0.39	0.59	0.73	0.56	0.18	0.38	0.46	0.033	0.073	0.018	0.026	0.0044	1		1
rs1898752	3	61.04 GATGATGGAGAAGAGGGTGCAGGCTTAATTGAGCTTGACGTAGTGGGTCGTTGGGATGTAATGGTGTAAGAAAAATAGTTCTTGGTAT[A/T]TGTACCTGCTCAGTATCTGTAC	A	T	0.83	0.57	0.55	0.44	0.59	0.95	0.85	0.59	0.68	0.54	0.42	0.043	0.051	0.002	0.118	0.0081	1		
rs748325	3	61.44 ATACAAGTTAAGATTTCTGAAACAAAATTAAGCAACAACAAATTACATTATTAT[A/T]ATGATTATTACTGTAAGGGTTAACAGCATGGTGCTCTTTAGTCAGCCTAGCAATGTACA	A	T	0.29	0.64	0.61	0.66	0.73	0.82	0.70	0.55	0.79	0.70	0.47	0.054	0.007	0.000	0.029	0.0139		1	1
rs1320173	3	61.48 AGATGACTCGAAAGTGGGGGATTCCTGCCATCACCACATGATAGCATTTGTCTTGG[A/G]ATAAACCACTCATGTAAAATGTTATCAAAGTGCCTAAAATGACACAGAATAGCGA	A	G	0.74	0.54	0.55	0.59	0.46	0.32	0.63	0.44	0.39	0.47	0.63	0.023	0.004	0.002	0.022	0.0044			
rs624228	3	61.69 GATTGAAATGATTTTGAGATTTCAAATATACATCACTAATCAGTGTTTCTTTT[C/G]TGGGCATTGTGGGCCTTGGAGGCTTTCTTGTTTCATATCTTCTGGCACCACAGGTGACTG	C	G	0.29	0.42	0.43	0.55	0.43	0.18	0.16	0.41	0.36	0.44	0.47	0.015	0.061	0.001	0.043	0.0000	1		
rs1528198	3	62.36 TGGGGCTTAATCACATTGGGCTCATTATTTATGTATCTTGAAGAAAGAAATGGTC[A/G]GAACTGAGAAATTCAGGTACATAAGAGAGCCATTCCTTTTCTCTTACATATCACAGTT	A	G	0.24	0.29	0.24	0.22	0.34	0.27	0.48	0.18	0.36	0.27	0.23	0.001	0.043	0.002	0.004	0.0005	1		
rs1534724	3	62.39 CATAATTAATCCATAAACACTTAATGAGTACAGATCAGGGCACCCTGCATTTTG[A/C]TATAGAGTCCACTTACTGCCCTCCCATCTTTATAATAAATCTGAACCTAGCTTGCAGGA	A	C	0.81	0.52	0.54	0.51	0.57	0.41	0.31	0.53	0.46	0.58	0.64	0.045	0.027	0.000	0.010	0.0050			1
rs1374679	3	63.03 TCATGATCCCCATCTCTGGCTATCAGGAATGACTATAATTCATTAGATATTGAA[A/G]TTGCCTGACAAGTCCTTGTTAGTCACACTCTTAAGCTTTTGTCTCAGCTGGTGGATGAGAA	A	G	0.81	0.57	0.55	0.53	0.52	0.77	0.64	0.55	0.65	0.56	0.66	0.037	0.004	0.000	0.023	0.0041	1		1
rs1874925	3	63.15 GCCAGAAATGTCCAGTTATTTCCATAAAAAGATGTGCAACATCATATTTCAGAT[A/G]AGGGTATTTTCTAACTGAGGCAAGTAATACTTTGCATACTCCTATCAGCAGGTAACAAA	A	G	0.23	0.55	0.49	0.52	0.48	0.36	0.76	0.49	0.43	0.49	0.47	0.042	0.042	0.000	0.010	0.0001			
rs1403699	3	63.37 CATGTCTATTAAGGAAGAATTGTTCCCTAGGGATTCTGGAAAAAAGATGGCTAAAATGTTGCAGTCCTAAATGGTGCCAGTCCTGGTAATAC[A/G]GAGATGAGCAGAAGTGCTAAGA	A	G	0.74	0.43	0.46	0.45	0.46	0.82	0.46	0.59	0.57	0.49	0.43	0.047	0.000	0.021	0.077	0.0015			
rs935794	3	63.39 AGTCCCCAAATGCATTCCTTTGCACCTGAGCAGTG[A/G]GCTCCCCATTTTAAATCCACTTGAGCCAATAAAATTCAGATGGATTTTATAGGGGGAAAATGCAACTGACATAGAAT	A	G	0.67	0.69	0.68	0.72	0.70	0.59	0.41	0.79	0.57	0.82	0.68	0.69	0.001	0.059	0.014	0.008	0.0002		
rs1376478	3	63.40 ACCAACAAAACTGCCTTTTATTTCT[A/G]TCAGAGTTGGTGGGTCCAGAAGCTCTCCTCTTTCTGCAGCGGGGCCTCTACCTGGGCTGTCTTGTGGGTGGCCTAGCTGAGT	A	G	0.26	0.33	0.41	0.38	0.34	0.41	0.34	0.38	0.32	0.36	0.39	0.021	0.005	0.005	0.001	0.0006			
rs744414	3	63.42 GAGTTATTCTGGACAGGAGGTTATACCTGGGGGGGTGTTACATTTAAGCTAA[C/G]ACCTGGAAGATGAGTGAGGGGAAGCCCCGCAAAAAATGAGAGTTGAAAGTAGGATCC	C	G	0.55	0.53	0.56	0.65	0.57	0.50	0.69	0.54	0.61	0.55	0.55	0.003	0.007	0.003	0.004	0.0001			
rs1390436	3	63.52 ATGGCTTGTAAATGGTTGGCCAGAAGTGAAGTGAGAACTGTGAGGTCCAGG[A/G]CCTACGCTGGTAATCACCACCTTACATCATCCCAAGGGAAGAGCAGGAGGTGGTGATT	A	G	0.45	0.47	0.51	0.51	0.50	0.68	0.83	0.45	0.54	0.46	0.58	0.001	0.070	0.002	0.022	0.0019			
rs853276	3	64.25 CTACCCCATTACCACTTTTAAATGAGATAATTGTGTCTACATAGGAGTAGATGG[A/C]TTTGATCCTACTACTCCCTCAGGCTCCAAGAATTGGCCCCGGCCCTAAATCTGGC	A	C	0.79	0.24	0.25	0.16	0.18	0.95	0.84	0.47	0.21	0.31	0.31	0.146	0.169	0.010	0.298	0.0013	1		
rs894741	3	64.38 TATGGGGATTTTCTTGAGTGATCATATACCCTTTACATTTTTGTTGTCACCCAC[A/G]CTTTTAGGTAGCAAATGTGGGGTTTTTTAGAATTTCTAAAAAGAACAAAAACAAAAAAC	A	G	0.38	0.46	0.46	0.45	0.48	0.05	0.49	0.46	0.61	0.51	0.55	0.005	0.000	0.001	0.129	0.0042			1
rs877523	3	64.79 CCAGCCCCCCCAAAAAGTATTGCATATATATCATGTTGAGTGTTTTTCTTCC[A/C]CACCATTTAGGATTTTGCTGTTGGCTAGCTGGGTGTACAGAGCTCTGAATTTGAGAA	A	C	0.38	0.51	0.52	0.54	0.61	0.23	0.32	0.63	0.46	0.47	0.45	0.010	0.016	0.010	0.044	0.0022	1		
rs1983357	3	64.95 ACTAAATTTCTATGTAAATTCATAGAAAAAACCTGGATAAAAAGCTGGACATTTGT[A/C]TAATAATAAGTAGATATCAGAGTGATGCAGCTCCTTTTGAGTAAGGAGGCAGATCT	A	C	0.69	0.82	0.85	0.78	0.84	0.86	0.58	0.81	0.82	0.70	0.77	0.009	0.033	0.001	0.002	0.0044			1
rs1864509	3	65.90 GTGACATCCTAGTTTTCTTGGAATTGTTTGTACTTATTGCTTTATTAGAAACC[A/G]CCTATAGTCAGATGTCTTGCCCACCCTTCTCAGTTCTTTTCCCCCAAGATGTCAAAAC	A	G	0.62	0.43	0.45	0.40	0.36	0.50	0.03	0.77	0.50	0.55	0.51	0.020	0.109	0.078	0.005	0.0027	1		
rs332364	3	66.32 AATTTTGTGTAGATGGAAGTGGCTCCTAATGTATCACTTTACATGTTAGAGTGCCA[A/G]TTAAATAACACTATTTTGTAATGAAGATAATGGCAATGTCAAATTAGTCTGTAATTC	A	G	0.38	0.82	0.81	0.81	0.75	0.32	0.31	0.52	0.68	0.80	0.87	0.080	0.119	0.037	0.116	0.0054			1
rs783512	3	66.38 ATGAGGCCAGGGGTCCATGAAGGAGCCAGTGCAGGGGTTAGGCTGCGCAGCAGGAG[C/G]GGGGAGGGTATTTATCCAAATACCAACTGAATTAACTGGGAGCACATCCACT	C	G	0.25	0.53	0.56	0.51	0.50	0.09	0.27	0.36	0.54	0.61	0.61	0.029	0.033	0.017	0.114	0.0037			
rs1490265	3	67.39 GCATGTGGGCCAAAAAAAAAAAAAAAACAAACTCTACTTGTCCTGACTTGATAA[A/C]TAGGCATAAGAAAATCCTGTATAATTAACATGAAGAACGAGATAATGAAGAGAATTCA	A	C	0.88	0.63	0.70	0.67	0.71	0.64	0.81	0.60	0.54	0.83	0.70	0.026	0.027	0.000	0.027	0.0007	1		
rs1463233	3	67.60 ATCCA[A/T]CTCTGTGTTGCCTCCCCAAGCCTGTGTGCCTTCAATCTTTCCGGCCCTTTGTTAAATTCATTAAATGACCTCACAAAATCCCTGTCTTCCTGACTGGCCCTTACTGA	A	T	0.64	0.50	0.44	0.51	0.43	0.45	0.05	0.35	0.50	0.49	0.55	0.010	0.130	0.003	0.000	0.0063		1	1
rs262180	3	68.35 ACTTACTTTTATCTAAACCCAAGGGAAA[A/G]TGAGGGGCAACACATTTGTGGAGCAGTTACTATTCAGAAGCAAGGCAGCATCACACAAGAATGTATTCTGAATTCGTTGCTTAT	A	G	0.40	0.46	0.36	0.37	0.45	0.00	0.33	0.10	0.46	0.41	0.35	0.000	0.004	0.086	0.150	0.0001	1		
rs1463957	3	69.03 TATACCTAAAAGTGAAATTTTCTATATCAAAGAGTATGTAGATCCCCAACTAG[A/G]TATTGCCTAATTGTTTTCTCAAGGGGTTTCTCAACTTACACTCCCACTAGCTATTTGGTTC	A	G	0.83	0.61	0.69	0.61	0.55	0.39	0.59	0.71	0.50	0.62	0.67	0.010	0.007	0.000	0.039	0.0001	1		
rs736333	3	69.56 CTATATAGAATGTACATCTGTTTATAAATGTATCAATGTCTAGAGATAAACATACCTA[C/G]TATTTGGTTCCTGTGGGAGAAGCAATTGATGTTAGGAGAGGAAATGAAGG	C	G	0.43	0.58	0.55	0.59	0.46	0.50	0.94	0.50	0.50	0.41	0.49	0.009	0.083	0.015	0.004	0.0019	1		
rs874947	3	69.68 TACAAATGTAAACTTCAAAGTTCTCACCCACAAAATCTGATATCATTAGAGTCT[C/G]GCCTAATTTCTTTCCAACACCAGGACAATGGCATGAGAGATATTTGTATAACTGATGA	C	G	0.45	0.48	0.48	0.49	0.45	0.18	0.06	0.27	0.54	0.53	0.51	0.001	0.088	0.017	0.036	0.0005			
rs1996818	3	70.25 AGAAGGGAGCTCGGTAGATAAATATTTTATAAATTTCTGCTAGGGCTCCCTTGAGTCTTTTGCTG[A/G]ATACCATCTACATGTGTGAAGTGAAACCCTATGAGGCTAGGCATGAC	A	G	0.69	0.49	0.47	0.52	0.44	1.00	0.76	0.76	0.61	0.46	0.51	0.019	0.045	0.060	0.231	0.0009	1		
rs1008411	3	70.38 CCTTCTCAAACTCTGTCTTCCCCAACACTTCCCCAACACTGTCTTCCCAACAC[A/G]ATAACTGGAGATGATGTCTTTGCATTCCTGCTCCTTGTAAGAAAAAAATAAATCAGCGTT	A	G	0.03	0.16	0.19	0.19	0.17	0.00	0.00	0.13	0.18	0.18	0.18	0.077	0.113	0.024	0.113	0.0018			
rs1019374	3	70.40 TGCAAGCAGGAATAATAATCCCATCAAAATAAAAAGCCCA[A/T]GCAAGTTAACTACAATATTACTAGGTCAGAGATGTTCAAATACCCTTTAGGAAAAGTCTG	A	T	0.90	0.64	0.67	0.66	0.75	1.00	0.99	0.80	0.68	0.67	0.65	0.020	0.082	0.000	0.082	0.0020			
rs1491607	3	70.51 TCAAATTTTCTCAAGAGTAAAAGATAGCTGAGCCACTTGAATCCTTCCTGATTCC[A/G]AAACTACTGCTTTTTTTCTTTCTATCATGAAGGACTGGGTCCTATTCACTTTTACATTT	A	G	0.64	0.68	0.64	0.68	0.82	0.18	0.27	0.68	0.42	0.75	0.61	0.62	0.000	0.000	0.016	0.124	0.0003		
rs1002200	3	70.79 AAGGGAACATGCAGCATAGGCATAGTTTTTAAAACCATTCAGGAAGGTTCAGTATCATTCACAGATTTATTATGGCCA[A/T]GCCACACACCTTTGTGTTCAACTTCATCCAATCC	A	T	0.69	0.56	0.57	0.53	0.52	1.00	0.97	0.67	0.64	0.52	0.51	0.008	0.116	0.000	0.157	0.0017	1		
rs17108	3	71.45 CCTCTTGCTCTTGTTAGAGACACTTTGGTTTTCAGAGACAAATAAGAAAAGGTGAGGGCCTTTAT[A/G]TATCACCAGAGCCTTGAGCTAGTGTTGCAGATACAGCTTTCTTCCT	A	G	0.38	0.32	0.35	0.40	0.43	0.00	0.01	0.35	0.36	0.44	0.42	0.001	0.164	0.002	0.164	0.0011	1		
rs1024008	3	72.60 CAGCAAAAGTGACATGGCGCCAGTTCTCAGTTGACGCCTCAAGAGACTTC[A/G]TGCTCTTCTGTGCTTGCTCTTGGAACCCTTCCACCACCAAGTGAGCAAGCCCAGGTTGGC	A	G	0.55	0.52	0.53	0.54	0.45	0.14	0.28	0.39	0.43	0.40	0.51	0.002	0.054	0.026	0.128	0.0015	1		
rs1388276	3	72.93 CACCCTTGATCAGGCTCTAGAAGGGCAAAGTCATG[A/T]GGCATTTTCCTCTCTCAAACGTGACCACATAGGTCTGCTCCTTCTACAAGGGCAATTTCT	A	T	0.26	0.29	0.28	0.33	0.39	0.45	0.05	0.41	0.11	0.36	0.27	0.000	0.049	0.006	0.014	0.0002			
rs7064	3	73.35 TCCACAAACCGTCTCCATCCACAACTTTCCTGTACATGCAAATTCTTTCA[A/G]TGGGCTGCAATATTTGCAAACATGCTTTAAACTTCCATAAAGATGCAAGA	A	G	0.48	0.80	0.75	0.56	0.86	0.20	0.19	0.76	0.71	0.73	0.77	0.000	0.036	0.000	0.016	0.0048	1		1
rs1441443	3	73.84 GTCAGGGAGAGAGCAATACAATCAAACACTCAAACCAAACCTAAGAGCCATTTATT[A/C]AACTCAGCTTTATAATAATTTTTGATTATTGATGAAGAAGTGTATTGCCAGAATAAAG	A	C	0.55	0.61	0.56	0.76	0.52	0.73	0.80	0.45	0.57	0.58	0.50	0.037	0.188	0.001	0.153	0.0009			
rs36003	3	74.17 TCTTTCCTTGTACTACCAGATTTGATGTGTATGGCTTAATAACGCAAGTAGAC[A/G]TGAATGTTAATAACTTCACAATTCATTGCTTTGCTATTTTTAATGCTCATGAAATTTAGA	A	G	0.48	0.55	0.56	0.67	0.54	0.68	0.95	0.46	0.46	0.54	0.55	0.033	0.042	0.002	0.110	0.0000	1		
rs931582	3	76.15 ACTATTGTGAATTACATTGTGCTCTCTTACACTTAATCTCTGCTTATCACTCC[A/G]CGACCAACCAATGCCCTTTGAATGACTATAGTACACCTAAAGTCAGGAGGCGCAAATTA	A	G	0.71	0.60	0.62	0.35	0.61	0.77	0.97	0.77	0.61	0.59	0.58	0.012	0.029	0.001	0.013	0.0013	1		
rs1562499	3	76.52 CTTTCAGGGATTGTAATTAGAGTGAGAAAGGGGTCAAACTGCATTGAGAAGGAG[A/C]AACATTTTAAGGATGGGCAGAGGAAGACAAGGAAGGAGCAGACAGTGTGGCAGA	A	C	0.83	0.52	0.57	0.55	0.59	0.45	0.45	0.50	0.54	0.57	0.54	0.001	0.024	0.007	0.009	0.0003			
rs1470569	3	77.45 TGAATAAAACACAGTTTTGCCTGGTAAGAAATGATATTAGTGGTCACTGAACGTTA[A/G]CATTGAAAGCCCACAAGTATAGCTCTTCTATTTAAAGAAACACTCATTGCTATTTTT	A	G	0.30	0.53	0.49	0.57	0.43	0.55	0.64	0.46	0.50	0.48	0.55	0.002	0.129	0.004	0.013	0.0000	1		
rs876675	3	77.60 AGAAATTACAGGCTTATTCCTGAAGACAGAAACAAGCCTTTATGTTTACTTTCTA[A/G]CCCAAACAGAGTGCAATGAGCTGCAATAAATAAATATGTTGTATTTTATTACAATCAA	A	G	0.26	0.20	0.22	0.52	0.05	0.36	0.25	0.23	0.32	0.17	0.20	0.001	0.001	0.005	0.000	0.0010			
rs1913081	3	78.36 ATCAGAGAAATTTTGCATGGTTGTTCATTGTTATAGATTGGGAATGTATTCTGGT[A/T]CACATCTAAACAGCAGAAAGAGATAATACTGGTAATGCAGTGTGGAGTAATTATTTAA	A	T	0.17	0.34	0.31	0.60	0.43	0.27	0.34	0.38	0.25	0.34	0.35	0.008	0.113	0.015	0.019	0.0005	1		
rs1383407	3	78.83 CACTTGGAAACCCTGCATGGCATAGGACAAGAGTTCAACAGAGTTGTGGAATT[A/G]ATGAGTGATTTACGCACGTTGAGAAAAAAGGAGGTTTGAGATCAAAGCAGTTCAC	A	G	0.48	0.39	0.42	0.61	0.43	0.40	0.06	0.49	0.36	0.39	0.45	0.039	0.008	0.000	0.005	0.0013			
rs189022	3	78.90 TTAGATAAGAGGATTTGTTTAAATAATCTTTCAGTTCTCCAGTGTCTAACCATAA[A/T]GGAAGTAAAGAGAAAAGGTAGTGAAAGCAATAGCAAAAACAATTGAGACTTATGAAT	A	T	0.33	0.36	0.39	0.44	0.38	0.27	0.07	0.41	0.43	0.38	0.43	0.015	0.014	0.001	0.001	0.0039			
rs1905343	3	79.93 CTTTGTAATCACTGCATTTACATTCATCCAAGAACAAGACAAATCTGCTGTGTTTCT[C/G]CCATGGGCAGCAGAGTTATAAAAGCAAACACGGTAGAACCAGAAGGATGAAAGA	C	G	0.67	0.75	0.74	0.23	0.63	1.00	0.98	0.79	0.68	0.66	0.72	0.001	0.002	0.001	0.012	0.0000	1		
rs2061719	3	81.17 CAATGGGGTTGTGAGGTGGCTTCACAATGAGGCAGTGAGGTGGGTTCCTTCCCT[A/G]TAAGTAATTAGCTCACCTGTTCTCATCATGACCAAATGAGAATGCTTGACCAAATGA	A	G	0.86	0.48	0.48	0.56	0.54	0.77	0.18	0.47	0.57	0.43	0.48	0.052	0.096	0.001	0.086	0.0003	1		
rs820273	3	81.47 GAAAACAAGAACAGAAGGAAAGTGAAATGTTGCATAAAGAATAGAAATGTTCATA[A/G]TTGAAAGAAAAATAAAAAACTGCCTTCTGCTCATTTCTTGAGAAACAGTTTTGAATCC	A	G	0.88	0.56	0.57	0.60	0.63	0.77	0.78	0.70	0.61	0.60	0.67	0.012	0.029	0.001	0.193	0.0042			1
rs17114	3	82.13 TCAGACCAACTGGTCTGAGACCTAAATAGTTCAATCAAAACCGTCAGAATAGATTCTATAAGAAAAGGTATTTTCTAAGCTTTT[A/G]GATTGAAGGACAATCAAGCATAGGCAC	A	G	0.26	0.50	0.51	0.37	0.46	0.64	0.84	0.42	0.54	0.43	0.55	0.025	0.000	0.000	0.003	0.0003	1		
rs936035	3	82.62 TGTTATCTGACAGTCGCCATAAAAGAAATTCAATAAGAATGATGAAAACCAACACC[A/C]TTAATGGAGTAGGTACCATGTTGAATAAGATCTTTGGCACTGTTACATGCATTATT	A	C	0.62	0.41	0.35	0.23	0.36	0.45	0.73	0.41	0.32	0.37	0.38	0.022	0.007	0.002	0.008	0.0005			
rs349148	3	83.31 TTTNTCTGCCCTAGTCCCATTTTATTCTCCTTTTAGGATTTCGGTTACAGGT[A/G]TGTTAAACTTTGGGCCTTATATCATAGATTTCTTGATTCTCTGTTAATTTTCTTCAATTTTGT	A	G	0.67	0.63	0.66	0.51	0.66	0.91	0.64	0.69	0.82	0.65	0.65	0.004	0.016	0.001	0.031	0.0011	1		
rs1524793	3	83.63 TATTCTTGCAAGTAAGATTATATTATTTGGTGTTTTCATCAGAAATGTCTATTAT[A/G]TAATGATGAGTCTATCTGTCTGTCTCTATCTATCATCTTTACAATATCACACAATTGATT	A	G	0.48	0.36	0.37	0.51	0.32	0.60	0.79	0.39	0.18	0.42	0.38	0.000	0.100	0.005	0.000	0.0002			
rs1524783	3	83.72 ATTACAGAAGGAGGAAAACATGATGAGAGCTGAAGAAGTATTTGAAGAAATTAGGA[A/C]TTAAAACGTTCCAATTTTGTCCAGAGATATAAAGCTACAGATTATCATATAAATAAAG	A	C	0.48	0.36	0.37	0.44	0.32	0.59	0.79	0.39	0.18	0.42	0.38	0.001	0.062	0.001	0.002	0.0009			
rs1385654	3	84.47 AGCAGTTTTAGACTTTCATTGCTAAGAATCCAAAGCAATTTTAATGTCCCTCAACTG[A/G]GGAATGGATAAACAGATGAAACAAATTTATCTAATAGAAACTAAACAAAACACACA	A	G	0.62	0.79	0.74	0.78	0.77	0.77	0.64	0.70	0.82	0.70	0.76	0.000	0.099	0.014	0.138	0.0006	1		
rs1470797	3	85.24 AGAAAATAACAGAGAAAGGCATGGAAATGACAGAGTCCAAGCCAAAATGGAATGTT[A/C]TTTCCACTTATCAAACACCCTCAGAAAAAGAGGGAAAGAAAATCCTTTCCTCATT	A	C	0.60	0.66	0.72	0.45	0.66	0.45	0.89	0.67	0.75	0.71	0.72	0.069	0.073	0.000	0.028	0.0007	1		
rs1562626	3	85.85 TGTCTTTACCAATTTTCCAGAAGATTGATTTAGATTGTTTTATGTTGTATGACTGT[A/G]AAAGAACAAAACAGAAAAGGATTTGCTTGTTTGATATTTTATTCTGTCTGTAATACAAA	A	G	0.02	0.27	0.26	0.55	0.25	0.15	0.16	0.08	0.25	0.16	0.25	0.078	0.028	0.012	0.028	0.0079	1	1	1[9]
rs1494302	3	86.48 AGAAAGCAGCATTCCATCTGGCTTATCCCACTGGGTTTCAAATATGCCATCTG[A/G]CCCATATTCCAGAATTCTATTCATCTCTTCCAACTACCTCATAAGACTGCAAAAGAAAC	A	G	0.57	0.59	0.58	0.41	0.61	0.50	0.53	0.49	0.61	0.55	0.63	0.018	0.084	0.000	0.020	0.0009	1		
rs721246	3	86.75 AGAGTTAGCACGGCATGAGTGTTGCCAAAGCTTACTACTTATACTCTGCTCGAGGCACAGATGGGACAGCAGGAGGAGCACTGTG[A/C]TCTGAATGCAAGGATCAGACACTCGA	A	C	0.90	0.66	0.60	0.38	0.73	0.45	0.45	0.19	0.72	0.64	0.64	0.54	0.000	0.001	0.000	0.044	0.0007		
rs1437049	3	86.77 GGTTATTTGTAAAAAGTCAGGCACCAGAATATTTAATGCCAACATTGTTTACATTGTCCTTCCTAGATACTCTGGGTTTGACATCTTTGG[A/C]CATAGTAGTCATTATCTCAATG	A	C	0.52	0.42	0.43	0.38	0.54	0.41	0.02	0.54	0.43	0.45	0.40	0.037	0.072	0.000	0.007	0.0001			
rs1370038	3	86.89 TGCCAGCTAAGATTGAGTTAGCAATCTCTATTCCATTCCAAATATTAACAGCCTTGA[A/G]AAGTAACTGACAACCAAGCAAACTTAAAACCAAGAGGAAAAATGTTTATGAATAG	A	G	0.33	0.49	0.45	0.62	0.54	0.64	0.30	0.54	0.71	0.45	0.38	0.000	0.000	0.001	0.051	0.0003			
rs13356	3	86.91 TTTTACTGCATTGAAATACCATGAACTTTCAGACTTGTTAGTTCTACAAG[A/C]AGTTGTGCTACCTTAATTTTGTGTTTCCAGAAATAAAAATTAACCTTAGT	A	C	0.73	0.78	0.79	0.38	0.83	0.95	1.00	0.74	0.79	0.74	0.72	0.008	0.108	0.000	0.032	0.0000			
rs1436640	3	86.92 AATAACTGTTACTTTCATAATCTTAATCTTCTCCTAGCAGTGTCTTAATGATAATTT[A/C]AACTTTATTTAGATCCTGAGAGCTATATCAAGTTAAATCCTCAAATTTTTATTTTAC	A	C	0.40	0.38	0.35	0.38	0.43	0.50	0.40	0.35	0.36	0.40	0.37	0.009	0.110	0.001	0.030	0.0000			
rs1025631	3	86.94 TTTATTTTTCCCTGGAGCATTTTAAAACTTTGTTCAATCTTTATGGAGTGCATTTTCAAAAATAGATTTCTACTTTCA[A/G]TAATAATGTCTTCACTAATCATGGTACAGTACTTCAT	A	G	0.67	0.66	0.67	0.76	0.80	0.95	0.99	0.70	0.64	0.67	0.62	0.005	0.001	0.001	0.005	0.0012			
rs1509781	3	87.56 ACCATATCATCTAGGCTTTATAAGTATAGGCTATAATGTTTGCACAATGACAGAATC[A/T]CCTAAGGACACATTTCTTAGAACGTATCCTCACAGTTAAGTGAAGCATGACTATAA	A	T	0.76	0.60	0.53	0.71	0.52	0.73	0.89	0.63	0.46	0.47	0.51	0.018	0.019	0.002	0.042	0.0003	1		
rs1157607	3	89.43 CTTTTTCCCCATAATTTCCTTTTTTATCCTATAGTCTAGCAAAACCGTATTATGC[A/G]ATGTGATTTGGAAGTATCCTACCTATTTGCATGTCTTCCCTTTTCTGTAGTGTTCCCC	A	G	0.52	0.47	0.44	0.28	0.41	0.60	0.52	0.46	0.36	0.40	0.41	0.060	0.001	0.008	0.001	0.0000	1		
rs1352463	3	95.54 GGCCAGCAGCATTCTAAGTGGGGGCACCAGAAGTTTCCAATTTGATTTGTAGTCCAACAACTGGCTAA[A/G]TGGTAAGTGTTATGCATTCAAAATATTTTTGTAATTATTATAT	A	G	0.50	0.47	0.45	0.62	0.48	0.27	0.57	0.49	0.64	0.47	0.37	0.000	0.000	0.000	0.002	0.0014	1		
rs2048861	3	96.30 ATCTGTAACAAGCAAGAGGGCTGCTTTGTGATCATTT[C/G]CATTACCTGAGATAAATTTATCTGTGTAGCAGGAGGGCCTTTGTTCAGATTGCATTTACTCCTTTTACCCTCCCG	C	G	0.52	0.28	0.23	0.68	0.20	0.00	0.00	0.17	0.11	0.29	0.31	0.080	0.080	0.005	0.006	0.0028	1		
rs494331	3	96.46 TGTGAAGGTTACACAGCACAGTGTGGCGGGGCTCTGACTGGAGTACAGATTTTGT[C/G]ACAAATCCTTTTCAATTAGCCCACCTGCCACTCTCAATTCAAACATGGGAAGTGT	C	G	0.31	0.39	0.44	0.66	0.45	0.64	0.07	0.32	0.25	0.49	0.44	0.135	0.096	0.016	0.014	0.0003			
rs1519159	3	96.49 TGCAAGCATCATTTTGTTTAATGTGGGTTGTTGCTATTTTCTTAGTTCCAC[A/G]TGCCAACTCAACTCCACATGCATCAGAATTGGCCTGAGCAACATTCCAGAGCATTAAGTTC	A	G	0.79	0.63	0.62	0.45	0.63	0.82	0.84	0.64	0.54	0.67	0.74	0.010	0.125	0.006	0.000	0.0000			
rs1368516	3	97.00 ACACCATTAATTAGTTACCTCTTTGGGTCATTTCACTGTGTCACATGGACGTTGACATGGTGTTGCAAGTGACAGATCA[A/G]TTTTTCACCTAAATTTAACATAAGTCTTCCAGTC	A	G	0.74	0.64	0.61	0.46	0.70	0.50	0.48	0.50	0.54	0.56	0.60	0.008	0.010	0.002	0.016	0.0042	1		1
rs1567058	3	98.81 TTTGAAAGAAAAGACATTTGAAAATAATATTCCAGTTTAATAATTAAAATTTACTGT[A/G]TAAAATATACTGCAAATTATGGCTGAGGATACTTAAAGGTACTGAAAGGTTAACAG	A	G	0.43	0.32	0.30	0.67	0.20	0.14	0.25	0.31	0.14	0.28	0.28	0.000	0.104	0.001	0.049	0.0004	1		
rs877965	3	98.85 TATTAGTAAGACTTCTCCAGCTTGATTCACTCGTCTAACCTGTCTCA[A/G]GCAATAATTGGCAGCTATAAATTAAATTATGTAGGTCACATTAGGATCAGTGCAGCAGTTTCTCC	A	G	0.40	0.65	0.55	0.28	0.61	0.73	0.59	0.60	0.46	0.59	0.49	0.017	0.077	0.003	0.013	0.0015			
rs1688378	3	99.04 TCAATTACAATTTGCCCTTGAGGATGTCAAGATCGATACTGCAACCTGGATGTTC[A/G]TCTCTGACTATCCCTACTAAACCACCACTCTCTTTTCCTCTCCATGTATGACCCATTT	A	G	0.52	0.53	0.60	0.30	0.63	0.41	0.15	0.66	0.61	0.59	0.59	0.001	0.000	0.000	0.008	0.0000			
rs1546223	3	99.09 TTGTTATTGTCAAGAATTTGGCAAATCAACAGCACATATCTTGATTTTCTCACTCAGAAT[A/G]CGATTCTCTTACAAAACAGCGTTGCTGCTTTCCTTGTGAATAATTAAAATAA	A	G	0.50	0.41	0.43	0.68	0.34	0.73	0.97	0.56	0.50	0.33	0.34	0.000	0.123	0.001	0.067	0.0008			
rs13038	3	99.55 GTGAGACTCTAATAAAGAACCAACTAGCTGAGCCCAATCAACTATGGAACTG[A/G]TAGAAATAAAATGAATTGTTGTTTTGTGCCGCTATGTTTGGGGTCATTTGTTATGCAG	A	G	0.29	0.49	0.49	0.54	0.59	0.09	0.16	0.42	0.43	0.53	0.47	0.037	0.079	0.005	0.019	0.0008	1		
rs1675521	3	99.57 TAGGGTATATAAATTCTGCAATGAAACTCTAATGCTGTGGATCTATGAAAAACC[A/G]TATTTTGTAGTTGCATTATTCTTCTTCCAGTAGAATTATAAACTGTAAAATTTTAGAGCTT	A	G	0.57	0.59	0.52	0.44	0.43	1.00	0.71	0.67	0.54	0.68	0.57	0.008	0.001	0.000	0.013	0.0014			
rs1017967	3	101.23 ATCAACTCCAGGCAGATTTCTGGACATCCCAGACCGCATGAGCTCCACTCTG[A/C]AAGAAGTAACCATGGAAGCTGATCTATTCCCTCTCCCGACATGGCTGCTGGCAAAAT	A	C	0.38	0.41	0.49	0.44	0.57	0.14	0.18	0.28	0.39	0.53	0.52	0.000	0.001	0.002	0.034	0.0044	1		1
rs591728	3	101.75 TAACTTCTGAAAATGTATATAAGCATATTACATAATCTGAAAACTCTCTACAATAAAG[A/G]AACACTTTTGTCTTTGTTTAAACCAGCTTTCCCTTACTTAGTTGACCATAAAATCCT	A	G	0.40	0.29	0.30	0.61	0.34	0.60	0.59	0.33	0.39	0.32	0.38	0.033	0.123	0.015	0.123	0.0007	1		
rs938988	3	103.18 TGATTTCCCTTCCACAAGCTTTCTAAGTCTAACACATCCTAAGTTGATGTCACC[A/G]GAATATTTTTTAAAAACAGGAATCTAACCATTTTTCTCCTCTGTTCAAAAACTTTCAAGG	A	G	0.10	0.34	0.42	0.24	0.31	0.05	0.02	0.11	0.39	0.40	0.34	0.008	0.064	0.001	0.076	0.0024	1		
rs1479371	3	103.34 AAGCCACAGGTTCTTTCTACTTTCATCCTGCTATCCACACTCTATTTTATTCTAAA[C/G]TTGGCTCCCCTTATGTTTCCAGTAGGTTCTCAGTGACAAAAAAGGCTAAATGTTTTC	C	G	0.21	0.47	0.53	0.28	0.45	0.50	0.53	0.44	0.36	0.45	0.41	0.037	0.075	0.002	0.075	0.0040			1
rs1464116	3	103.69 GGGGAAAAATTAGAGGGCCAATAATGGTATTCAGTAATTTGGTGATTTATGCTCT[A/T]GGCAATAGGAAGACTCAAATGCTTCCTTAAAGCAATGGAACAGTATGATCATATTG	A	T	0.21	0.30	0.34	0.44	0.37	0.09	0.49	0.19	0.57	0.40	0.40	0.014	0.104	0.014	0.015	0.0007	1		
rs1920325	3	105.19 ATAAATCTTTTACTATTTAATCTGTAACTTCCAAGCCTTTTCTTTTTACTCAGGTGT[A/G]TTAGGTTCCTTCTACACTGCTATAAAGAAATACCTGTAACTAGTCTCTGTTTGAGAAT	A	G	0.29	0.43	0.34	0.70	0.43	0.18	0.39	0.49	0.39	0.31	0.37	0.025	0.064	0.004	0.050	0.0131	1	1	1[9]
rs777955	3	105.78 TGATTGTTTGGGTCTCTTACCAGAAAGGAAAGGAAGTATTAGAAAAATATTCCA[A/T]GGGATAAAGACACTAGGTGAAGAATGGCAAGCTACAAGAGTTGATCTGTGTGTGACA	A	T	0.64	0.64	0.69	0.33	0.63	0.64	0.98	0.81	0.36	0.64	0.67	0.010	0.012	0.000	0.078	0.0001	1		
rs1436326	3	105.84 AGTAGGTCAGAATTAATATCTTTAACTCAACACAGAATCAACTCAACTGAAAACA[A/G]AAGTCGCAAGTAAATCCTACTCAGTTAACATTCAAAATATATTTCTTGCCCTCCAGA	A	G	0.71	0.64	0.57	0.61	0.56	0.68	0.61	0.78	0.64	0.65	0.66	0.018	0.005	0.000	0.002	0.0000			
rs1436340	3	105.92 AGCTCTGGACTCCTGATGATTCAAAATATTCACACTAAAGAGAAGTTGAATTCTAT[C/G]TGTTTAGGTCGTGGCACTTGAGGATTCCTTTTTATTTTTATAAAAGAAGCCTAAAC	C	G	0.81	0.45	0.52	0.27	0.66	0.59	0.39	0.51	0.32	0.52	0.43	0.003	0.008	0.000	0.032	0.0005			
rs2030515	3	106.60 ATTCAGTACAGTTGTGTTTTTTCTGATCCCTCAGCCTTTTACCTAATGGTTGTC[A/G]TATTCACTATTGACCTAGATGGAAAAGCCATTTTCTGTTTTCACTGAGGACACACGCAT	A	G	0.26	0.61	0.62	0.55	0.59	0.95	0.93	0.48	0.57	0.59	0.53	0.003	0.010	0.003	0.038	0.0017	1		
rs1550711	3	106.79 AAATCTCTTCTCTACACAGCCTATTCTCGCTACGGCACTTGTGACCATTGTAAAA[A/C]ACAAATGAAATAATTCTTTTGTTCAAAGCCTTCTAACTTTGAATAAAAGTCAAAGTCC	A	C	0.05	0.43	0.41	0.55	0.29	0.14	0.29	0.29	0.39	0.38	0.42	0.000	0.079	0.000	0.004	0.0004			
rs986692	3	107.60 TGGGGAAAGGGGTTTGCAAAGCTATTGAATAAATGCCTGGTATATTTTCTGGC[A/G]TAGATACACCAAATTTGGTCTTGAGTAATGCTTGTGACTCTAGACT	A	G	0.73	0.46	0.48	0.41	0.44	0.73	0.78	0.57	0.57	0.47	0.57	0.012	0.032	0.072	0.084	0.0000	1		
rs1283097	3	108.29 AAAAAGTGTGACCTTAGGTGAAGTGGTTTTCTACAGCTATGCCAATTCCTGAA[A/G]AGGGCTGACAGCTGCAGGCTGCCTGCTAACATCACACCCAGCAAGTGGGGAGCAAGC	A	G	0.64	0.50	0.50	0.40	0.38	0.32	0.76	0.28	0.64	0.46	0.48	0.019	0.265	0.016	0.090	0.0004	1		
rs1429	3	108.51 GAAAAGTCGTTGAGATCAGAACAGCATCTTTCTCAGCATGTTCCTTTCAATACCTAGCACAATCTCTTACTCGAAGTAGGC[A/G]TTCAACAACAAAGGTTAAGTGTAAGTGC	A	G	0.64	0.53	0.50	0.51	0.42	0.50	0.43	0.42	0.43	0.57	0.53	0.020	0.064	0.000	0.097	0.0001			
rs1516825	3	108.53 CAAATAAATAGGTCAGATTAAGGAGCAAAAAGAAAAATAACATTTACAGCACATC[A/G]AAGTAATGCAAATTAGCATCTCAGTGCCTCTATTTAATTATCAGTTGGTGTTTAAGGA	A	G	0.57	0.55	0.54	0.61	0.59	0.95	0.33	0.69	0.64	0.49	0.53	0.000	0.008	0.003	0.196	0.0006			
rs326361	3	109.14 ATCTTGAACAGCCCCTGGGTTTGACCGCTCCCACACGCTAACTGCTGCTGCAG[A/G]AGCTGCAACTTCCCTCATCTCTTCCGCAACCACAAGCCCTGGGAACCGCAATGGTA	A	G	0.95	0.68	0.66	0.48	0.70	0.59	0.59	0.47	0.71	0.58	0.74	0.001	0.009	0.000	0.007	0.0017	1		
rs327154	3	109.18 AAGAAAGGACAGGAAATGATTGGATTTGCATGGAAGAATGAGATGCTGGTAGG[A/G]ATCACTATTTTCAGTGTGTTTCAGGGGAAATGATTAAGTGCTAGGCCTACCAACTCT	A	G	0.55	0.59	0.57	0.51	0.45	0.86	0.98	0.70	0.68	0.61	0.51	0.004	0.000	0.027	0.105	0.0063			1
rs1447713	3	109.89 CAGTTTGACCCTCAGAGAGAGAAGATATATTTACT[A/G]TAAGAAAAGCGACTTAATAATCATTGAGCTCTTTCTGAAAGCACACAGAGAAAGGTATGTAGAGTGGTGTTTC	A	G	0.95	0.74	0.77	0.51	0.74	0.73	0.94	0.78	0.68	0.79	0.77	0.007	0.063	0.025	0.095	0.0009	1		
rs1732207	3	110.70 ATAAAATAAAGCAACAAATGTAAAAGTAGTAGTTGGTTCATTGCAGTTGACACAC[A/G]AGCAACATTTTGGAATGAAATTTTCTGAATTCAGTTTCAACATATTTTTGTATAAAAG	A	G	0.24	0.45	0.43	0.32	0.41	0.00	0.56	0.36	0.50	0.44	0.41	0.007	0.043	0.000	0.050	0.0031	1		
rs716534	3	111.26 TGCAAATTTCAGTCCAAGGGCAGGAAAAGTCCAGTGTTTCAGTTCAACAGTCAG[A/G]CAGATAGAAAAAGGGAAAGTCTCATTTCTTTTCCCTTTTGTTCTATTCAGGGCCTC	A	G	0.50	0.52	0.57	0.45	0.59	0.82	0.55	0.53	0.61	0.61	0.51	0.019	0.000	0.006	0.000	0.0007	1		
rs1512532	3	111.64 ATTGACTTTGCATTGAAATAGAAATAGAGGTACGTGGCAAAATGTGAATACAAATGACTAATCTCTACATGACTTCTAAATTGGTTCTTGTTT[A/G]ATTTATTTCATCCATTTTAGT	A	G	0.21	0.47	0.42	0.33	0.48	0.45	0.17	0.52	0.43	0.51	0.47	0.059	0.126	0.046	0.090	0.0053			1
rs1398748	3	112.60 TCCACCATGTGAGGACATGGCATTTCTCCCCTCCAGAGGATTTAGCAAGCAG[A/G]CCCTCACCAGATGTCAATGGCTTGATCTGGACATTTTCAACCTCCAGAACTGTGAGA	A	G	0.24	0.52	0.48	0.38	0.55	0.41	0.76	0.55	0.46	0.40	0.47	0.062	0.000	0.007	0.021	0.0111	1	1	1
rs1907642	3	113.06 TCAAAATATTCACAATTTTTAAAAAAATGATAATGCTGATAAGAGTGTATGGAAAT[A/G]GCCACATATACTTAGCAGGTGAGGCGCTAAACTGGTAGTATGGAGGGTAGTTTGAC	A	G	0.53	0.44	0.49	0.32	0.41	0.40	0.32	0.57	0.46	0.52	0.41	0.022	0.009	0.015	0.046	0.0006			
rs1317244	3	113.37 TTTAGCCCCGGTCACCTCCCTGTAAACATATAAATACTGTGTTTTCAGATTGGTC[A/G]TATTTTCAGTTATTCTGCACAGACTCGAGGAAAAAAAAAACCTGTAAAATAAAAATA	A	G	0.83	0.59	0.61	0.39	0.55	0.95	0.82	0.52	0.68	0.53	0.58	0.005	0.002	0.007	0.021	0.0011	1		
rs12736	3	114.85 ACCATTGCATAATTTCTGTCAATGTTCTCAAAGGTTAAAACAAGTTGTAT[A/G]GAAGAATACCTTTCCTCAATAGCAGGCCACTGTAAAAACATGTAATCTGC	A	G	0.26	0.37	0.40	0.55	0.30	0.23	0.22	0.34	0.54	0.38	0.35	0.001	0.095	0.016	0.000	0.0001	1		
rs1025398	3	114.91 ACAAAGCAGTAATCATAACACTGTATTGCTGGGTTT[A/G]TGGGATAATAACATTAGGTTGAAGCAAAGGTCAATATTTTAGCAGAATTAAATTAGTATGAATCTGAAGAATACTATA	A	G	0.50	0.42	0.49	0.68	0.40	0.45	0.41	0.58	0.50	0.53	0.49	0.014	0.001	0.045	0.011	0.0091		1	1
rs2046496	3	115.16 TCTTAATCAGGAGAGATAGCTCTTCTCTTACATGATGGCGACAGAAAGAATA[C/G]TCCCTTGCCTAATTTTGATGATAAATGGATAAGTACAACAACCATGACTGAGAAACAG	C	G	0.24	0.49	0.53	0.44	0.46	0.18	0.17	0.45	0.39	0.46	0.53	0.029	0.029	0.016	0.001	0.0067			
rs2062834	3	115.27 GAGGGGACAGGCACATGGAGGACCTGAAGTAGGGCAGGTTGTAGTAAGGATCAGAAGCTCTAAATACCCAAATGATCAATCT[A/G]GGCATGAGCATGGTGAGGGCTTCTAACT	A	G	0.60	0.63	0.52	0.45	0.57	0.95	0.63	0.42	0.64	0.51	0.42	0.018	0.000	0.041	0.012	0.0022			
rs1039524	3	115.33 ATCTTCTACTCGAATCTTATTCTTTTGTCTTGTTTATCTGGACTGAGACTTT[A/G]TTGAATTCTGGCTTGATTTCTCTGGTGCAGTAGGATCAGAGGAGATTAGAGGAGAGAGC	A	G	0.10	0.29	0.25	0.59	0.33	0.36	0.38	0.22	0.29	0.19	0.23	0.074	0.082	0.021	0.086	0.0014			
rs714697	3	116.68 ACCAGCTGGTTGCTTCAGCAAATAATGGTCACCATTTTATAGAGAGATACTTTAGT[A/G]TAGTCAGAAAGATAGCCGTCCTTGACACTATAAACTTCTTAATATCTGGGCTAAGG	A	G	0.55	0.37	0.44	0.45	0.43	0.23	0.28	0.47	0.50	0.45	0.41	0.025	0.020	0.013	0.003	0.0001	1		

rs1461131	3	117.32	[illegible]	[illegible]	[illegible]	[illegible]	[illegible]	[illegible]	[illegible]	[illegible]	[illegible]	[illegible]	[illegible]	[illegible]	[illegible]	[illegible]	[illegible]	[illegible]	[illegible]	[illegible]	[illegible]	[illegible]	[illegible]
rs2055426	3	118.54	[illegible]	[illegible]	[illegible]	[illegible]	[illegible]	[illegible]	[illegible]	[illegible]	[illegible]	[illegible]	[illegible]	[illegible]	[illegible]	[illegible]	[illegible]	[illegible]	[illegible]	[illegible]	[illegible]	[illegible]	[illegible]
rs1348969	3	119.39	[illegible]	[illegible]	[illegible]	[illegible]	[illegible]	[illegible]	[illegible]	[illegible]	[illegible]	[illegible]	[illegible]	[illegible]	[illegible]	[illegible]	[illegible]	[illegible]	[illegible]	[illegible]	[illegible]	[illegible]	[illegible]
rs1983380	3	119.53	[illegible]	[illegible]	[illegible]	[illegible]	[illegible]	[illegible]	[illegible]	[illegible]	[illegible]	[illegible]	[illegible]	[illegible]	[illegible]	[illegible]	[illegible]	[illegible]	[illegible]	[illegible]	[illegible]	[illegible]	[illegible]
rs1000198	3	120.43	[illegible]	[illegible]	[illegible]	[illegible]	[illegible]	[illegible]	[illegible]	[illegible]	[illegible]	[illegible]	[illegible]	[illegible]	[illegible]	[illegible]	[illegible]	[illegible]	[illegible]	[illegible]	[illegible]	[illegible]	[illegible]
rs538136	3	120.63	[illegible]	[illegible]	[illegible]	[illegible]	[illegible]	[illegible]	[illegible]	[illegible]	[illegible]	[illegible]	[illegible]	[illegible]	[illegible]	[illegible]	[illegible]	[illegible]	[illegible]	[illegible]	[illegible]	[illegible]	[illegible]
rs1147696	3	121.44	[illegible]	[illegible]	[illegible]	[illegible]	[illegible]	[illegible]	[illegible]	[illegible]	[illegible]	[illegible]	[illegible]	[illegible]	[illegible]	[illegible]	[illegible]	[illegible]	[illegible]	[illegible]	[illegible]	[illegible]	[illegible]
rs519336	3	121.84	[illegible]	[illegible]	[illegible]	[illegible]	[illegible]	[illegible]	[illegible]	[illegible]	[illegible]	[illegible]	[illegible]	[illegible]	[illegible]	[illegible]	[illegible]	[illegible]	[illegible]	[illegible]	[illegible]	[illegible]	[illegible]
rs338988	3	122.32	[illegible]	[illegible]	[illegible]	[illegible]	[illegible]	[illegible]	[illegible]	[illegible]	[illegible]	[illegible]	[illegible]	[illegible]	[illegible]	[illegible]	[illegible]	[illegible]	[illegible]	[illegible]	[illegible]	[illegible]	[illegible]
rs1472621	3	123.33	[illegible]	[illegible]	[illegible]	[illegible]	[illegible]	[illegible]	[illegible]	[illegible]	[illegible]	[illegible]	[illegible]	[illegible]	[illegible]	[illegible]	[illegible]	[illegible]	[illegible]	[illegible]	[illegible]	[illegible]	[illegible]
rs9740	3	123.33	[illegible]	[illegible]	[illegible]	[illegible]	[illegible]	[illegible]	[illegible]	[illegible]	[illegible]	[illegible]	[illegible]	[illegible]	[illegible]	[illegible]	[illegible]	[illegible]	[illegible]	[illegible]	[illegible]	[illegible]	[illegible]
rs1127343	3	123.45	[illegible]	[illegible]	[illegible]	[illegible]	[illegible]	[illegible]	[illegible]	[illegible]	[illegible]	[illegible]	[illegible]	[illegible]	[illegible]	[illegible]	[illegible]	[illegible]	[illegible]	[illegible]	[illegible]	[illegible]	[illegible]
rs1500530	3	123.60	[illegible]	[illegible]	[illegible]	[illegible]	[illegible]	[illegible]	[illegible]	[illegible]	[illegible]	[illegible]	[illegible]	[illegible]	[illegible]	[illegible]	[illegible]	[illegible]	[illegible]	[illegible]	[illegible]	[illegible]	[illegible]
rs1456116	3	124.41	[illegible]	[illegible]	[illegible]	[illegible]	[illegible]	[illegible]	[illegible]	[illegible]	[illegible]	[illegible]	[illegible]	[illegible]	[illegible]	[illegible]	[illegible]	[illegible]	[illegible]	[illegible]	[illegible]	[illegible]	[illegible]
rs1997422	3	125.30	[illegible]	[illegible]	[illegible]	[illegible]	[illegible]	[illegible]	[illegible]	[illegible]	[illegible]	[illegible]	[illegible]	[illegible]	[illegible]	[illegible]	[illegible]	[illegible]	[illegible]	[illegible]	[illegible]	[illegible]	[illegible]
rs634265	3	125.42	[illegible]	[illegible]	[illegible]	[illegible]	[illegible]	[illegible]	[illegible]	[illegible]	[illegible]	[illegible]	[illegible]	[illegible]	[illegible]	[illegible]	[illegible]	[illegible]	[illegible]	[illegible]	[illegible]	[illegible]	[illegible]
rs641189	3	125.55	[illegible]	[illegible]	[illegible]	[illegible]	[illegible]	[illegible]	[illegible]	[illegible]	[illegible]	[illegible]	[illegible]	[illegible]	[illegible]	[illegible]	[illegible]	[illegible]	[illegible]	[illegible]	[illegible]	[illegible]	[illegible]
rs986909	3	125.60	[illegible]	[illegible]	[illegible]	[illegible]	[illegible]	[illegible]	[illegible]	[illegible]	[illegible]	[illegible]	[illegible]	[illegible]	[illegible]	[illegible]	[illegible]	[illegible]	[illegible]	[illegible]	[illegible]	[illegible]	[illegible]
rs13975	3	126.12	[illegible]	[illegible]	[illegible]	[illegible]	[illegible]	[illegible]	[illegible]	[illegible]	[illegible]	[illegible]	[illegible]	[illegible]	[illegible]	[illegible]	[illegible]	[illegible]	[illegible]	[illegible]	[illegible]	[illegible]	[illegible]
rs651630	3	126.13	[illegible]	[illegible]	[illegible]	[illegible]	[illegible]	[illegible]	[illegible]	[illegible]	[illegible]	[illegible]	[illegible]	[illegible]	[illegible]	[illegible]	[illegible]	[illegible]	[illegible]	[illegible]	[illegible]	[illegible]	[illegible]
rs713161	3	127.26	[illegible]	[illegible]	[illegible]	[illegible]	[illegible]	[illegible]	[illegible]	[illegible]	[illegible]	[illegible]	[illegible]	[illegible]	[illegible]	[illegible]	[illegible]	[illegible]	[illegible]	[illegible]	[illegible]	[illegible]	[illegible]
rs1799404	3	127.48	[illegible]	[illegible]	[illegible]	[illegible]	[illegible]	[illegible]	[illegible]	[illegible]	[illegible]	[illegible]	[illegible]	[illegible]	[illegible]	[illegible]	[illegible]	[illegible]	[illegible]	[illegible]	[illegible]	[illegible]	[illegible]
rs2053820	3	127.82	[illegible]	[illegible]	[illegible]	[illegible]	[illegible]	[illegible]	[illegible]	[illegible]	[illegible]	[illegible]	[illegible]	[illegible]	[illegible]	[illegible]	[illegible]	[illegible]	[illegible]	[illegible]	[illegible]	[illegible]	[illegible]
rs730257	3	127.96	[illegible]	[illegible]	[illegible]	[illegible]	[illegible]	[illegible]	[illegible]	[illegible]	[illegible]	[illegible]	[illegible]	[illegible]	[illegible]	[illegible]	[illegible]	[illegible]	[illegible]	[illegible]	[illegible]	[illegible]	[illegible]
rs1658037	3	128.72	[illegible]	[illegible]	[illegible]	[illegible]	[illegible]	[illegible]	[illegible]	[illegible]	[illegible]	[illegible]	[illegible]	[illegible]	[illegible]	[illegible]	[illegible]	[illegible]	[illegible]	[illegible]	[illegible]	[illegible]	[illegible]
rs594323	3	128.77	[illegible]	[illegible]	[illegible]	[illegible]	[illegible]	[illegible]	[illegible]	[illegible]	[illegible]	[illegible]	[illegible]	[illegible]	[illegible]	[illegible]	[illegible]	[illegible]	[illegible]	[illegible]	[illegible]	[illegible]	[illegible]
rs1127030	3	129.66	[illegible]	[illegible]	[illegible]	[illegible]	[illegible]	[illegible]	[illegible]	[illegible]	[illegible]	[illegible]	[illegible]	[illegible]	[illegible]	[illegible]	[illegible]	[illegible]	[illegible]	[illegible]	[illegible]	[illegible]	[illegible]
rs852223	3	131.97	[illegible]	[illegible]	[illegible]	[illegible]	[illegible]	[illegible]	[illegible]	[illegible]	[illegible]	[illegible]	[illegible]	[illegible]	[illegible]	[illegible]	[illegible]	[illegible]	[illegible]	[illegible]	[illegible]	[illegible]	[illegible]
rs903048	3	132.07	[illegible]	[illegible]	[illegible]	[illegible]	[illegible]	[illegible]	[illegible]	[illegible]	[illegible]	[illegible]	[illegible]	[illegible]	[illegible]	[illegible]	[illegible]	[illegible]	[illegible]	[illegible]	[illegible]	[illegible]	[illegible]
rs1355782	3	132.81	[illegible]	[illegible]	[illegible]	[illegible]	[illegible]	[illegible]	[illegible]	[illegible]	[illegible]	[illegible]	[illegible]	[illegible]	[illegible]	[illegible]	[illegible]	[illegible]	[illegible]	[illegible]	[illegible]	[illegible]	[illegible]
rs6792114	3	132.86	[illegible]	[illegible]	[illegible]	[illegible]	[illegible]	[illegible]	[illegible]	[illegible]	[illegible]	[illegible]	[illegible]	[illegible]	[illegible]	[illegible]	[illegible]	[illegible]	[illegible]	[illegible]	[illegible]	[illegible]	[illegible]
rs2199351	3	132.93	[illegible]	[illegible]	[illegible]	[illegible]	[illegible]	[illegible]	[illegible]	[illegible]	[illegible]	[illegible]	[illegible]	[illegible]	[illegible]	[illegible]	[illegible]	[illegible]	[illegible]	[illegible]	[illegible]	[illegible]	[illegible]
rs1919987	3	133.00	[illegible]	[illegible]	[illegible]	[illegible]	[illegible]	[illegible]	[illegible]	[illegible]	[illegible]	[illegible]	[illegible]	[illegible]	[illegible]	[illegible]	[illegible]	[illegible]	[illegible]	[illegible]	[illegible]	[illegible]	[illegible]
rs719300	3	133.82	[illegible]	[illegible]	[illegible]	[illegible]	[illegible]	[illegible]	[illegible]	[illegible]	[illegible]	[illegible]	[illegible]	[illegible]	[illegible]	[illegible]	[illegible]	[illegible]	[illegible]	[illegible]	[illegible]	[illegible]	[illegible]
rs1402455	3	134.04	[illegible]	[illegible]	[illegible]	[illegible]	[illegible]	[illegible]	[illegible]	[illegible]	[illegible]	[illegible]	[illegible]	[illegible]	[illegible]	[illegible]	[illegible]	[illegible]	[illegible]	[illegible]	[illegible]	[illegible]	[illegible]
rs2370409	3	134.33	[illegible]	[illegible]	[illegible]	[illegible]	[illegible]	[illegible]	[illegible]	[illegible]	[illegible]	[illegible]	[illegible]	[illegible]	[illegible]	[illegible]	[illegible]	[illegible]	[illegible]	[illegible]	[illegible]	[illegible]	[illegible]
rs1355776	3	134.33	[illegible]	[illegible]	[illegible]	[illegible]	[illegible]	[illegible]	[illegible]	[illegible]	[illegible]	[illegible]	[illegible]	[illegible]	[illegible]	[illegible]	[illegible]	[illegible]	[illegible]	[illegible]	[illegible]	[illegible]	[illegible]
rs12595	3	134.82	[illegible]	[illegible]	[illegible]	[illegible]	[illegible]	[illegible]	[illegible]	[illegible]	[illegible]	[illegible]	[illegible]	[illegible]	[illegible]	[illegible]	[illegible]	[illegible]	[illegible]	[illegible]	[illegible]	[illegible]	[illegible]
rs1984630	3	135.74	[illegible]	[illegible]	[illegible]	[illegible]	[illegible]	[illegible]	[illegible]	[illegible]	[illegible]	[illegible]	[illegible]	[illegible]	[illegible]	[illegible]	[illegible]	[illegible]	[illegible]	[illegible]	[illegible]	[illegible]	[illegible]
rs36178	3	135.97	[illegible]	[illegible]	[illegible]	[illegible]	[illegible]	[illegible]	[illegible]	[illegible]	[illegible]	[illegible]	[illegible]	[illegible]	[illegible]	[illegible]	[illegible]	[illegible]	[illegible]	[illegible]	[illegible]	[illegible]	[illegible]
rs750543	3	136.35	[illegible]	[illegible]	[illegible]	[illegible]	[illegible]	[illegible]	[illegible]	[illegible]	[illegible]	[illegible]	[illegible]	[illegible]	[illegible]	[illegible]	[illegible]	[illegible]	[illegible]	[illegible]	[illegible]	[illegible]	[illegible]
rs1502186	3	136.57	[illegible]	[illegible]	[illegible]	[illegible]	[illegible]	[illegible]	[illegible]	[illegible]	[illegible]	[illegible]	[illegible]	[illegible]	[illegible]	[illegible]	[illegible]	[illegible]	[illegible]	[illegible]	[illegible]	[illegible]	[illegible]
rs1052620	3	137.90	[illegible]	[illegible]	[illegible]	[illegible]	[illegible]	[illegible]	[illegible]	[illegible]	[illegible]	[illegible]	[illegible]	[illegible]	[illegible]	[illegible]	[illegible]	[illegible]	[illegible]	[illegible]	[illegible]	[illegible]	[illegible]
rs930984	3	138.26	[illegible]	[illegible]	[illegible]	[illegible]	[illegible]	[illegible]	[illegible]	[illegible]	[illegible]	[illegible]	[illegible]	[illegible]	[illegible]	[illegible]	[illegible]	[illegible]	[illegible]	[illegible]	[illegible]	[illegible]	[illegible]
rs768496	3	138.56	[illegible]	[illegible]	[illegible]	[illegible]	[illegible]	[illegible]	[illegible]	[illegible]	[illegible]	[illegible]	[illegible]	[illegible]	[illegible]	[illegible]	[illegible]	[illegible]	[illegible]	[illegible]	[illegible]	[illegible]	[illegible]
rs751357	3	139.40	[illegible]	[illegible]	[illegible]	[illegible]	[illegible]	[illegible]	[illegible]	[illegible]	[illegible]	[illegible]	[illegible]	[illegible]	[illegible]	[illegible]	[illegible]	[illegible]	[illegible]	[illegible]	[illegible]	[illegible]	[illegible]
rs564800	3	139.58	[illegible]	[illegible]	[illegible]	[illegible]	[illegible]	[illegible]	[illegible]	[illegible]	[illegible]	[illegible]	[illegible]	[illegible]	[illegible]	[illegible]	[illegible]	[illegible]	[illegible]	[illegible]	[illegible]	[illegible]	[illegible]
rs331577	3	139.72	[illegible]	[illegible]	[illegible]	[illegible]	[illegible]	[illegible]	[illegible]	[illegible]	[illegible]	[illegible]	[illegible]	[illegible]	[illegible]	[illegible]	[illegible]	[illegible]	[illegible]	[illegible]	[illegible]	[illegible]	[illegible]
rs1426054	3	140.94	[illegible]	[illegible]	[illegible]	[illegible]	[illegible]	[illegible]	[illegible]	[illegible]	[illegible]	[illegible]	[illegible]	[illegible]	[illegible]	[illegible]	[illegible]	[illegible]	[illegible]	[illegible]	[illegible]	[illegible]	[illegible]
rs868534	3	141.60	[illegible]	[illegible]	[illegible]	[illegible]	[illegible]	[illegible]	[illegible]	[illegible]	[illegible]	[illegible]	[illegible]	[illegible]	[illegible]	[illegible]	[illegible]	[illegible]	[illegible]	[illegible]	[illegible]	[illegible]	[illegible]
rs1863868	3	142.41	[illegible]	[illegible]	[illegible]	[illegible]	[illegible]	[illegible]	[illegible]	[illegible]	[illegible]	[illegible]	[illegible]	[illegible]	[illegible]	[illegible]	[illegible]	[illegible]	[illegible]	[illegible]	[illegible]	[illegible]	[illegible]
rs1709	3	142.65	[illegible]	[illegible]	[illegible]	[illegible]	[illegible]	[illegible]	[illegible]	[illegible]	[illegible]	[illegible]	[illegible]	[illegible]	[illegible]	[illegible]	[illegible]	[illegible]	[illegible]	[illegible]	[illegible]	[illegible]	[illegible]
rs1479137	3	143.94	[illegible]	[illegible]	[illegible]	[illegible]	[illegible]	[illegible]	[illegible]	[illegible]	[illegible]	[illegible]	[illegible]	[illegible]	[illegible]	[illegible]	[illegible]	[illegible]	[illegible]	[illegible]	[illegible]	[illegible]	[illegible]
rs765695	3	144.97	[illegible]	[illegible]	[illegible]	[illegible]	[illegible]	[illegible]	[illegible]	[illegible]	[illegible]	[illegible]	[illegible]	[illegible]	[illegible]	[illegible]	[illegible]	[illegible]	[illegible]	[illegible]	[illegible]	[illegible]	[illegible]
rs723490	3	145.01	[illegible]	[illegible]	[illegible]	[illegible]	[illegible]	[illegible]	[illegible]	[illegible]	[illegible]	[illegible]	[illegible]	[illegible]	[illegible]	[illegible]	[illegible]	[illegible]	[illegible]	[illegible]	[illegible]	[illegible]	[illegible]
rs1405597	3	145.64	[illegible]	[illegible]	[illegible]	[illegible]	[illegible]	[illegible]	[illegible]	[illegible]	[illegible]	[illegible]	[illegible]	[illegible]	[illegible]	[illegible]	[illegible]	[illegible]	[illegible]	[illegible]	[illegible]	[illegible]	[illegible]
rs800065	3	145.71	[illegible]	[illegible]	[illegible]	[illegible]	[illegible]	[illegible]	[illegible]	[illegible]	[illegible]	[illegible]	[illegible]	[illegible]	[illegible]	[illegible]	[illegible]	[illegible]	[illegible]	[illegible]	[illegible]	[illegible]	[illegible]
rs1527732	3	146.62	[illegible]	[illegible]	[illegible]	[illegible]	[illegible]	[illegible]	[illegible]	[illegible]	[illegible]	[illegible]	[illegible]	[illegible]	[illegible]	[illegible]	[illegible]	[illegible]	[illegible]	[illegible]	[illegible]	[illegible]	[illegible]
rs1398775	3	146.87	[illegible]	[illegible]	[illegible]	[illegible]	[illegible]	[illegible]	[illegible]	[illegible]	[illegible]	[illegible]	[illegible]	[illegible]	[illegible]	[illegible]	[illegible]	[illegible]	[illegible]	[illegible]	[illegible]	[illegible]	[illegible]
rs1024080	3	147.03	[illegible]	[illegible]	[illegible]	[illegible]	[illegible]	[illegible]	[illegible]	[illegible]	[illegible]	[illegible]	[illegible]	[illegible]	[illegible]	[illegible]	[illegible]	[illegible]	[illegible]	[illegible]	[illegible]	[illegible]	[illegible]
rs1707465	3	147.17	[illegible]	[illegible]	[illegible]	[illegible]	[illegible]	[illegible]	[illegible]	[illegible]	[illegible]	[illegible]	[illegible]	[illegible]	[illegible]	[illegible]	[illegible]	[illegible]	[illegible]	[illegible]	[illegible]	[illegible]	[illegible]
rs1523336	3	147.46	[illegible]	[illegible]	[illegible]	[illegible]	[illegible]	[illegible]	[illegible]	[illegible]	[illegible]	[illegible]	[illegible]	[illegible]	[illegible]	[illegible]	[illegible]	[illegible]	[illegible]	[illegible]	[illegible]	[illegible]	[illegible]
rs1870424	3	147.55	[illegible]	[illegible]	[illegible]	[illegible]	[illegible]	[illegible]	[illegible]	[illegible]	[illegible]	[illegible]	[illegible]	[illegible]	[illegible]	[illegible]	[illegible]	[illegible]	[illegible]	[illegible]	[illegible]	[illegible]	[illegible]
rs1503733	3	148.40	[illegible]	[illegible]	[illegible]	[illegible]	[illegible]	[illegible]	[illegible]	[illegible]	[illegible]	[illegible]	[illegible]	[illegible]	[illegible]	[illegible]	[illegible]	[illegible]	[illegible]	[illegible]	[illegible]	[illegible]	[illegible]
rs1027695	3	148.54	[illegible]	[illegible]	[illegible]	[illegible]	[illegible]	[illegible]	[illegible]	[illegible]	[illegible]	[illegible]	[illegible]	[illegible]	[illegible]	[illegible]	[illegible]	[illegible]	[illegible]	[illegible]	[illegible]	[illegible]	[illegible]
rs1381768	3	149.13	[illegible]	[illegible]	[illegible]	[illegible]	[illegible]	[illegible]	[illegible]	[illegible]	[illegible]	[illegible]	[illegible]	[illegible]	[illegible]	[illegible]	[illegible]	[illegible]	[illegible]	[illegible]	[illegible]	[illegible]	[illegible]
rs1009621	3	149.51	[illegible]	[illegible]	[illegible]	[illegible]	[illegible]	[illegible]	[illegible]	[illegible]	[illegible]	[illegible]	[illegible]	[illegible]	[illegible]	[illegible]	[illegible]	[illegible]	[illegible]	[illegible]	[illegible]	[illegible]	[illegible]
rs2013929	3	150.35	[illegible]	[illegible]	[illegible]	[illegible]	[illegible]	[illegible]	[illegible]	[illegible]	[illegible]	[illegible]	[illegible]	[illegible]	[illegible]	[illegible]	[illegible]	[illegible]	[illegible]	[illegible]	[illegible]	[illegible]	[illegible]
rs1561026	3	150.67	[illegible]	[illegible]	[illegible]	[illegible]	[illegible]	[illegible]	[illegible]	[illegible]	[illegible]	[illegible]	[illegible]	[illegible]	[illegible]	[illegible]	[illegible]	[illegible]	[illegible]	[illegible]	[illegible]	[illegible]	[illegible]
rs1450344	3	151.51	[illegible]	[illegible]	[illegible]	[illegible]	[illegible]	[illegible]	[illegible]	[illegible]	[illegible]	[illegible]	[illegible]	[illegible]	[illegible]	[illegible]	[illegible]	[illegible]	[illegible]	[illegible]	[illegible]	[illegible]	[illegible]
rs1920395	3	152.29	[illegible]	[illegible]	[illegible]	[illegible]	[illegible]	[illegible]	[illegible]	[illegible]	[illegible]	[illegible]	[illegible]	[illegible]	[illegible]	[illegible]	[illegible]	[illegible]	[illegible]	[illegible]	[illegible]	[illegible]	[illegible]
rs325762	3	153.21	[illegible]	[illegible]	[illegible]	[illegible]	[illegible]	[illegible]	[illegible]	[illegible]	[illegible]	[illegible]	[illegible]	[illegible]	[illegible]	[illegible]	[illegible]	[illegible]	[illegible]	[illegible]	[illegible]	[illegible]	[illegible]
rs755763	3	153.32	[illegible]	[illegible]	[illegible]	[illegible]	[illegible]	[illegible]	[illegible]	[illegible]	[illegible]	[illegible]	[illegible]	[illegible]	[illegible]	[illegible]	[illegible]	[illegible]	[illegible]	[illegible]	[illegible]	[illegible]	[illegible]
rs701265	3	153.88	[illegible]	[illegible]	[illegible]	[illegible]	[illegible]	[illegible]	[illegible]	[illegible]	[illegible]	[illegible]	[illegible]	[illegible]	[illegible]	[illegible]	[illegible]	[illegible]	[illegible]	[illegible]	[illegible]	[illegible]	[illegible]
rs1388007	3	154.58	[illegible]	[illegible]	[illegible]	[illegible]	[illegible]	[illegible]	[illegible]	[illegible]	[illegible]	[illegible]	[illegible]	[illegible]	[illegible]	[illegible]	[illegible]	[illegible]	[illegible]	[illegible]	[illegible]	[illegible]	[illegible]
rs902432	3	154.62	[illegible]	[illegible]	[illegible]	[illegible]	[illegible]	[illegible]	[illegible]	[illegible]	[illegible]	[illegible]	[illegible]	[illegible]	[illegible]	[illegible]	[illegible]	[illegible]	[illegible]	[illegible]	[illegible]	[illegible]	[illegible]
rs9438	3	155.34	[illegible]	[illegible]	[illegible]	[illegible]	[illegible]	[illegible]	[illegible]	[illegible]	[illegible]	[illegible]	[illegible]	[illegible]	[illegible]	[illegible]	[illegible]	[illegible]	[illegible]	[illegible]	[illegible]	[illegible]	[illegible]
rs1025192	3	156.15	[illegible]	[illegible]	[illegible]	[illegible]	[illegible]	[illegible]	[illegible]	[illegible]	[illegible]	[illegible]	[illegible]	[illegible]	[illegible]	[illegible]	[illegible]	[illegible]	[illegible]	[illegible]	[illegible]	[illegible]	[illegible]
rs1872413	3	156.35	[illegible]	[illegible]	[illegible]	[illegible]	[illegible]	[illegible]	[illegible]	[illegible]	[illegible]	[illegible]	[illegible]	[illegible]	[illegible]	[illegible]	[illegible]	[illegible]	[illegible]	[illegible]	[illegible]	[illegible]	[illegible]
rs359573	3	156.64	[illegible]	[illegible]	[illegible]	[illegible]	[illegible]	[illegible]	[illegible]	[illegible]	[illegible]	[illegible]	[illegible]	[illegible]	[illegible]	[illegible]	[illegible]	[illegible]	[illegible]	[illegible]	[illegible]	[illegible]	[illegible]
rs986963	3	157.29	[illegible]	[illegible]	[illegible]	[illegible]	[illegible]	[illegible]	[illegible]	[illegible]	[illegible]	[illegible]	[illegible]	[illegible]	[illegible]	[illegible]	[illegible]	[illegible]	[illegible]	[illegible]	[illegible]	[illegible]	[illegible]
rs1384542	3	158.08	[illegible]	[illegible]	[illegible]	[illegible]	[illegible]	[illegible]	[illegible]	[illegible]	[illegible]	[illegible]	[illegible]	[illegible]	[illegible]	[illegible]	[illegible]	[illegible]	[illegible]	[illegible]	[illegible]	[illegible]	[illegible]
rs1074864	3	158.45	[illegible]	[illegible]	[illegible]	[illegible]	[illegible]	[illegible]	[illegible]	[illegible]	[illegible]	[illegible]	[illegible]	[illegible]	[illegible]	[illegible]	[illegible]	[illegible]	[illegible]	[illegible]	[illegible]	[illegible]	[illegible]
rs1373118	3	158.61	[illegible]	[illegible]	[illegible]	[illegible]	[illegible]	[illegible]	[illegible]	[illegible]	[illegible]	[illegible]	[illegible]	[illegible]	[illegible]	[illegible]	[illegible]	[illegible]	[illegible]	[illegible]	[illegible]	[illegible]	[illegible]
rs963354	3	158.71	[illegible]	[illegible]	[illegible]	[illegible]	[illegible]	[illegible]	[illegible]	[illegible]	[illegible]	[illegible]	[illegible]	[illegible]	[illegible]	[illegible]	[illegible]	[illegible]	[illegible]	[illegible]	[illegible]	[illegible]	[illegible]
rs1472578	3	160.13	[illegible]	[illegible]	[illegible]	[illegible]	[illegible]	[illegible]	[illegible]	[illegible]	[illegible]	[illegible]	[illegible]	[illegible]	[illegible]	[illegible]	[illegible]	[illegible]	[illegible]	[illegible]	[illegible]	[illegible]	[illegible]
rs1515628	3	160.56	[illegible]	[illegible]	[illegible]	[illegible]	[illegible]	[illegible]	[illegible]	[illegible]	[illegible]	[illegible]	[illegible]	[illegible]	[illegible]	[illegible]	[illegible]	[illegible]	[illegible]	[illegible]	[illegible]	[illegible]	[illegible]
rs1402276	3	160.62	[illegible]	[illegible]	[illegible]	[illegible]	[illegible]	[illegible]	[illegible]	[illegible]	[illegible]	[illegible]	[illegible]	[illegible]	[illegible]	[illegible]	[illegible]	[illegible]	[illegible]	[illegible]	[illegible]	[illegible]	[illegible]
rs937478	3	161.13	[illegible]	[illegible]	[illegible]	[illegible]	[illegible]	[illegible]	[illegible]	[illegible]	[illegible]	[illegible]	[illegible]	[illegible]	[illegible]	[illegible]	[illegible]	[illegible]	[illegible]	[illegible]	[illegible]	[illegible]	[illegible]
rs1599365	3	162.18	[illegible]	[illegible]	[illegible]	[illegible]	[illegible]	[illegible]	[illegible]	[illegible]	[illegible]	[illegible]	[illegible]	[illegible]	[illegible]	[illegible]	[illegible]	[illegible]	[illegible]	[illegible]	[illegible]	[illegible]	[illegible]
rs1478568	3	162.34	[illegible]	[illegible]	[illegible]	[illegible]	[illegible]	[illegible]	[illegible]	[illegible]	[illegible]	[illegible]	[illegible]	[illegible]	[illegible]	[illegible]	[illegible]	[illegible]	[illegible]	[illegible]	[illegible]	[illegible]	[illegible]
rs1515723	3	162.69	[illegible]	[illegible]	[illegible]	[illegible]	[illegible]	[illegible]	[illegible]	[illegible]	[illegible]	[illegible]	[illegible]	[illegible]	[illegible]	[illegible]	[illegible]	[illegible]	[illegible]	[illegible]	[illegible]	[illegible]	[illegible]
rs482314	3	162.79	[illegible]	[illegible]	[illegible]	[illegible]	[illegible]	[illegible]	[illegible]	[illegible]	[illegible]	[illegible]	[illegible]	[illegible]	[illegible]	[illegible]	[illegible]	[illegible]	[illegible]	[illegible]	[illegible]	[illegible]	[illegible]
rs901816	3	164.05	[illegible]	[illegible]	[illegible]	[illegible]	[illegible]	[illegible]	[illegible]	[illegible]	[illegible]	[illegible]	[illegible]	[illegible]	[illegible]	[illegible]	[illegible]	[illegible]	[illegible]	[illegible]	[illegible]	[illegible]	[illegible]
rs1492174	3	164.29	[illegible]	[illegible]	[illegible]	[illegible]	[illegible]	[illegible]	[illegible]	[illegible]	[illegible]	[illegible]	[illegible]	[illegible]	[illegible]	[illegible]	[illegible]	[illegible]	[illegible]	[illegible]	[illegible]	[illegible]	[illegible]
rs1369276	3	165.48	[illegible]	[illegible]	[illegible]	[illegible]	[illegible]	[illegible]	[illegible]	[illegible]	[illegible]	[illegible]	[illegible]	[illegible]	[illegible]	[illegible]	[illegible]	[illegible]	[illegible]	[illegible]	[illegible]	[illegible]	[illegible]
rs820417	3	166.21	[illegible]	[illegible]	[illegible]	[illegible]	[illegible]	[illegible]	[illegible]	[illegible]	[illegible]	[illegible]	[illegible]	[illegible]	[illegible]	[illegible]	[illegible]	[illegible]	[illegible]	[illegible]	[illegible]	[illegible]	[illegible]
rs769276	3	166.36	[illegible]	[illegible]	[illegible]	[illegible]	[illegible]	[illegible]	[illegible]	[illegible]	[illegible]	[illegible]	[illegible]	[illegible]	[illegible]	[illegible]	[illegible]	[illegible]	[illegible]	[illegible]	[illegible]	[illegible]	[illegible]
rs1899169	3	167.22	[illegible]	[illegible]	[illegible]	[illegible]	[illegible]	[illegible]	[illegible]	[illegible]	[illegible]	[illegible]	[illegible]	[illegible]	[illegible]	[illegible]	[illegible]	[illegible]	[illegible]	[illegible]	[illegible]	[illegible]	[illegible]
rs1996489	3	167.40	[illegible]	[illegible]	[illegible]	[illegible]	[illegible]	[illegible]	[illegible]	[illegible]	[illegible]	[illegible]	[illegible]	[illegible]	[illegible]	[illegible]	[illegible]	[illegible]	[illegible]	[illegible]	[illegible]	[illegible]	[illegible]
rs877439	3	169.12	[illegible]	[illegible]	[illegible]	[illegible]	[illegible]	[illegible]	[illegible]	[illegible]	[illegible]	[illegible]	[illegible]	[illegible]	[illegible]	[illegible]	[illegible]	[illegible]	[illegible]	[illegible]	[illegible]	[illegible]	[illegible]
rs953834	3	169.14	[illegible]	[illegible]	[illegible]	[illegible]	[illegible]	[illegible]	[illegible]	[illegible]	[illegible]	[illegible]	[illegible]	[illegible]	[illegible]	[illegible]	[illegible]	[illegible]	[illegible]	[illegible]	[illegible]	[illegible]	[illegible]
rs1025217	3	169.53	[illegible]	[illegible]	[illegible]	[illegible]	[illegible]	[illegible]	[illegible]	[illegible]	[illegible]	[illegible]	[illegible]	[illegible]	[illegible]	[illegible]	[illegible]	[illegible]	[illegible]	[illegible]	[illegible]	[illegible]	[illegible]
rs3863100	3	170.24	[illegible]	[illegible]	[illegible]	[illegible]	[illegible]	[illegible]	[illegible]	[illegible]	[illegible]	[illegible]	[illegible]	[illegible]	[illegible]	[illegible]	[illegible]	[illegible]	[illegible]	[illegible]	[illegible]	[illegible]	[illegible]
rs1920122	3	170.83	[illegible]	[illegible]	[illegible]	[illegible]	[illegible]	[illegible]	[illegible]	[illegible]	[illegible]	[illegible]	[illegible]	[illegible]	[illegible]	[illegible]	[illegible]	[illegible]	[illegible]	[illegible]	[illegible]	[illegible]	[illegible]
rs1563436	3	173.11	[illegible]	[illegible]	[illegible]	[illegible]	[illegible]	[illegible]	[illegible]	[illegible]	[illegible]	[illegible]	[illegible]	[illegible]	[illegible]	[illegible]	[illegible]	[illegible]	[illegible]	[illegible]	[illegible]	[illegible]	[illegible]
rs1353894	3	173.13	[illegible]	[illegible]	[illegible]	[illegible]	[illegible]	[illegible]	[illegible]	[illegible]	[illegible]	[illegible]	[illegible]	[illegible]	[illegible]	[illegible]	[illegible]	[illegible]	[illegible]	[illegible]	[illegible]	[illegible]	[illegible]
rs623021	3	173.21	[illegible]	[illegible]	[illegible]	[illegible]	[illegible]	[illegible]	[illegible]	[illegible]	[illegible]	[illegible]	[illegible]	[illegible]	[illegible]	[illegible]	[illegible]	[illegible]	[illegible]	[illegible]	[illegible]	[illegible]	[illegible]
rs874671	3	173.78	[illegible]	[illegible]	[illegible]	[illegible]	[illegible]	[illegible]	[illegible]	[illegible]	[illegible]	[illegible]	[illegible]	[illegible]	[illegible]	[illegible]	[illegible]	[illegible]	[illegible]	[illegible]	[illegible]	[illegible]	[illegible]
rs7433573	3	173.89	GAATTCGAGAGCTATTCAAAACCCAGCAGCTTAAAAAACCAGAGTATCTGTTCTCCT[A/G]TCATCACTCTGAATTTGTTCATCCTGACTGCATCATGACTCAGCTCTACCTAATT	A	G	0.25	0.29	0.30	0.28	0.23	0.09	0.26	0.39	0.46	0.36	0.30	0.002	0.000	0.010	0.028	0.0001		
rs649695	3	174.47	AAAATTGAGACAAAAAGGTCAAAATGTGGGGAAATTGAATCATTTTTTCTTTT[A/C]TTTGAGTCAAAGTTAAGTTGCCATCAGTTTAAGTTGTTATATCCATAAAATATTTTGTA	A	C	0.64	0.80	0.75	0.78	0.73	0.82	0.78	0.75	0.64	0.63	0.74	0.007	0.002	0.000	0.008	0.0001	1	
rs2046718	3	174.79	ATTCTAAGCCCAGGGGATACAGCACAAATAGCCAAAAATCCTTACACTAATAGAACT[A/G]TTAGTAGAGATATTGATAATACACTAAGTAAATAAGTAAATTAAGTAATATTTTAG	A	G	0.52	0.23	0.24	0.33	0.32	0.09	0.08	0.35	0.39	0.19	0.27	0.073	0.002	0.052	0.004	0.0055		
rs1488547	3	174.85	TTGTTTCACCCATTTTTTTATGCGTGTTGACATTCTCGAGCCCAAATTTTTTATAGTTC[A/G]ATGAATTCTATAGGTTGGTTTTAAGATGTGAAACTAAAAGGTGATGAAATTATT	A	G	0.48	0.24	0.30	0.29	0.25	0.91	0.33	0.42	0.32	0.29	0.33	0.021	0.002	0.023	0.238	0.0014		
rs753293	3	176.43	CAATTCTACTTGTCTAACATTGACTTTTTCTATTTGTAATGCAGCACAACCCA[A/G]TAGGATTTCCCATTGTTTCTTCTAAAACCGTGCACCTGCACCATCGATTATGCCAGGA	A	G	0.88	0.81	0.78	0.85	0.73	0.59	0.40	0.79	0.75	0.79	0.74	0.013	0.064	0.000	0.014	0.0008	1	
rs1549114	3	176.44	CAGAGTCTTTTTTTATTTTTTGAAGCTGTGCAGTAGAGGTATACAGAAGTGA[A/G]TTGTCCCTTGGAAGTAGATTAACCATTGTTTTGTAGATGTCTAAGAGATGCTTTATTTG	A	G	0.62	0.32	0.35	0.35	0.25	0.27	0.25	0.29	0.29	0.30	0.30	0.049	0.008	0.000	0.003	0.0004		
rs1545866	3	177.34	GACAGCGATCAAAGGCTAAAATTTGTTTTTTATTGTTGTTGTTCCTTTTACCCCC[A/G]CAAAGAGGGCAACATTAAGCAACAGAGACACATTGTCAATGCCAATCTTCTGTTTGTG	A	G	0.14	0.47	0.44	0.40	0.46	0.30	0.24	0.31	0.75	0.41	0.49	0.073	0.038	0.021	0.021	0.0000	1	
rs2046350	3	177.34	CATGAAGATAGACAAGGCAGGATATCTGAAATGAGAGAAGAGGCAGGATCATCCT[A/G]GAATATGCCTGAAATAGGAGGACCTAGTGGTAGCTATTGGTGTCACAGAAGGCT	A	G	0.45	0.48	0.48	0.41	0.43	0.41	0.24	0.40	0.75	0.41	0.49	0.001	0.041	0.011	0.005	0.0000		
rs951461	3	177.35	GATGTATTAAATCATAATATACACGAAATATAATTTTTGATATTTTTATCTCCTTTCCTCCATTCTATTCTACTATT[A/T]ACAATTCTCCCTCATGTTGGTAAATGACTCCTCTATCTC	A	T	0.36	0.58	0.58	0.57	0.57	0.27	0.41	0.47	0.82	0.59	0.56	0.036	0.010	0.006	0.052	0.0015		
rs1483635	3	177.37	ACAGGGTGAGACTCTGTCTCAAACAAACAAACAAGCAAATAACAAAAAATGTATT[A/G]TCTATTATCATACAAAGCCCAAACAACTGCAAGTACCAATAGATAAATATACCCTA	A	G	0.88	0.68	0.60	0.67	0.68	0.44	0.24	0.51	0.61	0.71	0.56	0.043	0.091	0.006	0.020	0.0029		
rs936034	3	177.56	AAAACACAAAATCTTGCATGCTATGATAAAACCGCTGTTTTGGCCAAAATTATT[A/G]ACATTCTGTAACCCATCTATGAAGGCATTTAATTGAAAGGCATATTTGAAATGTGGAA	A	G	0.79	0.62	0.65	0.66	0.54	0.18	0.29	0.50	0.71	0.60	0.66	0.013	0.054	0.008	0.101	0.0000		
rs1349838	3	177.66	ATAGGCATTTGACTTAGGCTGGGCTAATCGTGGTACCTGACATAAATTCACAGA[A/T]GTGCCTATAACTTATAGGGGAATTAATCCGTGGGACATTTGGGAAAAAAAGGTTTGCT	A	T	0.69	0.49	0.50	0.51	0.63	0.55	0.64	0.48	0.46	0.58	0.56	0.015	0.005	0.000	0.002	0.0027		
rs1000021	3	177.79	CAACGTTACATACTCATTCCTTAAATACATCTGTCATCAAGGCACTTTGGGAA[C/G]CACTTGGCCCAATCCTTCATTACCTCTTGGATTCTACCTTAGTCCCAGCCTGAGTGTCA	C	G	0.90	0.64	0.65	0.67	0.54	0.59	0.64	0.78	0.57	0.68	0.65	0.059	0.001	0.008	0.000	0.0001		
rs1468924	3	180.30	CAGGAAGAAGGACCAGACAGACTACAGAGGAAAGGTGTTCTTCTCGCTAGA[A/C]ATGAACCAATTTTTTTGTGAACAGACAATTAAAATGAATTACTTTATGGCAAAAGA	A	C	0.62	0.32	0.31	0.45	0.32	0.05	0.33	0.28	0.07	0.41	0.45	0.037	0.000	0.001	0.086	0.0069	1	1
rs2049769	3	180.97	TATGAACCTGATTCCAGAACCCTTGAAAATAATGACAGGAGAAAATATAATCA[A/G]TAAGGAATCATTTGGCTCAACTCCACCACTCTCACTACCCTATCCCCATCTCCTTCTTA	A	G	0.90	0.80	0.75	0.77	0.77	0.68	0.53	0.69	0.82	0.76	0.72	0.014	0.027	0.007	0.005	0.0016	1	
rs1401337	3	181.76	TTTAAATATCATAAAAGCAACCATAAAAATAAAGCATTCTAATCAGAGGGAAGA[A/C]ATTCAAAGCTCGCGTCAAAAAGGAAAACAATTTACTTGTGTCGGTACCCTTAATAAG	A	C	0.86	0.45	0.50	0.55	0.52	0.68	0.48	0.69	0.61	0.48	0.53	0.101	0.005	0.025	0.017	0.0005	1	
rs1401974	3	181.89	GCACTTCTCCCATTGGAGACGTGGGTGCAGCCAGATGGTACCAGTGCTTGCA[A/G]ACAGATGTGTAGAGGGAGACTTCTTCCCCCTTACCCATTGCTCTGGATGCAACCACAG	A	G	0.07	0.57	0.56	0.50	0.41	0.60	0.59	0.40	0.64	0.47	0.53	0.157	0.001	0.026	0.000	0.0001		
rs833283	3	182.91	CCTCAATTAAGTGGGGTGGGAATGGGGCAGTTCTGGGCAAGTTTAAAAACTGT[C/G]CTCTCAATTTTTCAATAAAGGAAAATCCTGGGCTTGTGAGATCGCAAACATTGTGAC	C	G	0.07	0.55	0.57	0.59	0.66	0.18	0.33	0.24	0.54	0.52	0.52	0.190	0.046	0.086	0.112	0.0002	1	
rs1973738	3	184.57	CAGAAATTAGCAAGTTTCTTTCTGAAGCGATTGTACTCAGTTTCCTTATAAGTGTCA[A/G]TTTCTTAAGAAAATAAGAGAAGATTGCTGTGGGCTTCCAAATATCTATTTAACCCACT	A	G	0.67	0.38	0.37	0.40	0.36	0.77	0.61	0.56	0.21	0.43	0.48	0.042	0.026	0.008	0.083	0.0086	1	1
rs2054172	3	184.70	TTGCGTCTTTTGATCAATATAGCCATTTTCATATAGAAGCAGTCCTACCTATTGCC[A/G]TTTCAGGTGGAAAGCCTTACCACACAAATTGATACTGTGTCCTCACCAAGTTTAAAA	A	G	0.57	0.76	0.76	0.73	0.77	0.73	0.64	0.74	0.82	0.73	0.73	0.014	0.012	0.000	0.000	0.0000		
rs262958	3	184.81	AGTCATTGTAAATGAAGGAAAGTCTTCATTTTCCTGAGACAGGGAGACAACATC[A/G]AGCACTGTAAAGTTACACTAAATTCCAGCAATTCCCTGACATTGAATCAGATGCCCA	A	G	0.33	0.38	0.44	0.41	0.46	0.50	0.56	0.46	0.36	0.45	0.49	0.007	0.004	0.000	0.001	0.0015		
rs1401999	3	185.00	CACTTACAAATGTGGCTTACAA[C/G]ACCACATCACATGTATAGTACTAATGGAGTTCTAAGGGCGAATGGGAGGGTGAGCTTTCCAGGGAGTGTATGTTGCCTCATGAAGAATC	C	G	0.76	0.55	0.58	0.54	0.61	0.95	0.54	0.58	0.57	0.57	0.57	0.013	0.005	0.006	0.091	0.0001		
rs869417	3	185.01	ACCAACTAAGCATTTAGCAACAGAAATGAATAACAACTAATCTTATGAGGTGATC[A/G]TTTTATAAGCAAGGAAATGAAACATTCAAGAGGATGCTTTCAGGATTTTCCCCAAAGA	A	G	0.24	0.45	0.42	0.46	0.41	0.05	0.46	0.42	0.43	0.43	0.43	0.013	0.005	0.006	0.091	0.0001		
rs3892035	3	185.52	TTGAAAATAAATTTTCACTGTGGCTTTTTTCATTTCTTAACCTTATCCATGTGAAC[A/G]GGAGAGCTTCCTCGTTCCACTGGGAGATCTTATCCACTAGTAACGAAAATGTGCTCC	A	G	0.93	0.86	0.82	0.90	0.88	0.86	0.61	0.80	0.86	0.83	0.79	0.003	0.039	0.006	0.000	0.0036	1	
rs4686705	3	185.68	TCTGCTCACCTTCCCTGCCTCTCTCCCTGCCAGCCCCTAATCCAGCTGCCTTCTG[A/G]ACAAGCTAGAACTGCTCTTAACCCCATGATCATCCATCTGCTGACCCTCTGGATG	A	G	0.48	0.42	0.40	0.49	0.43	0.55	0.74	0.48	0.43	0.45	0.44	0.011	0.081	0.015	0.027	0.0022		
rs2377104	3	185.78	TGGGTGGTGTCAGATAATTACTAAGACCCCAAAATTAAAGTGGTTGAGTCCTTA[A/C]TTTTCCACGGCTGCTCAAGATGAAAATGAAAGCTGGCCTCCAGGGGGCCCTGGGAG	A	C	0.30	0.53	0.48	0.46	0.55	0.45	0.05	0.38	0.46	0.59	0.52	0.012	0.106	0.000	0.001	0.0009		
rs4432622	3	185.88	AGCGTGGTCAATTATTATCATCTCTCAGAAGGCAGACTCTACAGACTCTATTTAAC[A/C]AACTCTGTAGAGCTTCCTGGGCACTCACTTAAACTTACTCCAGTACATGGAAACAG	A	C	0.29	0.64	0.65	0.59	0.71	0.23	0.43	0.49	0.36	0.63	0.67	0.058	0.018	0.007	0.080	0.0017		
rs4572747	3	186.02	ATTGTTTTCTTTGACCACTTTTCTTTTTCTTTGATTTTCTCTGACATTATAGGC[A/C]CTTTGGTTTCCGTTATTGGAGGCAATGATGGCCCCTCAGAAGCTGTCCAGTTCAGCCAT	A	C	0.26	0.01	0.02	0.01	0.00	0.27	0.00	0.10	0.04	0.02	0.05	0.061	0.013	0.005	0.059	0.0017	1	
rs3332	3	186.09	GACTTGACAGACTGAGAGCCAGCCAGCCAGGAGACCTGCAGAACTTAAACAC[A/G]GAGCATTTATTGTTAGAAAGGGCAAGTCTTACACTCAAATAGGTTTTAACATGAA	A	G	0.52	0.43	0.44	0.54	0.32	0.77	0.59	0.53	0.61	0.40	0.41	0.000	0.003	0.000	0.034	0.0039		
rs6769709	3	186.17	GATGCTGTTCTATATCTGTTTTGTCATATATCCCTGAATTTCACTTGTCTGAGAG[A/T]CAGATCCTTCAGTCTGTGACTGACATCCTTTTCTATCATCTTGCAACAACTTCTCTAC	A	T	0.23	0.08	0.10	0.09	0.05	0.23	0.00	0.22	0.04	0.07	0.09	0.015	0.181	0.075	0.244	0.0002		
rs1837882	3	186.55	AAGAAAGTAGGTGGATTTGTGATTTAATTTCTCTGAGCAAAAAGGGAAATAGC[A/G]CACTGCAAACAATTGTAACTCAACTTACTGATTGATTTTGGATTCTGTGGTGTTTCCAG	A	G	0.43	0.42	0.44	0.39	0.48	0.23	0.21	0.23	0.54	0.46	0.54	0.000	0.017	0.026	0.019	0.0033	1	
rs713484	3	187.61	AAAGAGAGAACACCAGAATTTGTAATGAATCTTATATATTGAAGTAATCACAGTA[A/C]GAGGCTATATACTTCTTTTCGGATTATTCAAAACATCTGTGGCACTTTTCTTTCAGAA	A	C	0.45	0.83	0.81	0.82	0.75	0.90	0.74	0.75	0.71	0.83	0.81	0.062	0.003	0.000	0.015	0.0001	1	
rs266759	3	187.83	CTTAGTGGTGACTGGAGTTACTCTGGCCCTTTCCGTTCTCCCAAAGCCTTCCCAG[A/G]AAAGTATGGAACTTAGGTGTGGACAGAATGACTGGGGGCACGGGGGCCAGTTG	A	G	0.33	0.48	0.48	0.44	0.56	0.41	0.55	0.55	0.54	0.51	0.47	0.005	0.006	0.006	0.000	0.0001		
rs1568302	3	188.42	CACATGGAAAAAGCCTTTTCCATTTCAAGGGATGACTGATTTTGTTATCAGCA[A/G]CTTTTGCTGAGCCTTCTCTAGGTCAGATGTCTGTGTGCGTGAGAATAAGCTCAGTGTGTA	A	G	0.74	0.64	0.65	0.60	0.59	0.82	0.78	0.70	0.68	0.65	0.62	0.001	0.002	0.007	0.001	0.0027	1	
rs1157840	3	188.94	AACCTGACTCATGCTTCATGTTCATATTCAACTTCTTCCCTCTGTGCTTCTAACTA[A/C]ATCTAGGAATCTCCTTGTTCTTAGAACCACCTTTATCCCTTCTCAGATATTTATGTATAT	A	C	0.40	0.74	0.78	0.70	0.77	0.55	0.96	0.79	0.68	0.70	0.70	0.082	0.040	0.002	0.040	0.0034	1	
rs2030033	3	189.01	TAGATGTAAAACTCTTGCTAAAGACATATCATTCATTACTCTTCTAATGAATATACTCAG[A/G]TAATGTACTCAGTGATACATTCCTTTTCAAAATTAATATTAGGAAAAATGTCTCTCA	A	G	0.74	0.78	0.73	0.87	0.80	0.91	0.91	0.81	0.86	0.75	0.69	0.000	0.019	0.007	0.026	0.0018		
rs2030525	3	189.40	TTAAGTGATCTAGAGTCATGGCTTATTTCCCACAAGGATTCAGTGGGCAACTGTG[A/G]CAATAGTTCTTGGCTAACCTGGTCACTATGGAAGTTCTTTCCGTGGAAAAAGATTC	A	G	0.10	0.23	0.14	0.17	0.07	0.36	0.44	0.25	0.18	0.21	0.20	0.008	0.049	0.004	0.023	0.0041		
rs2049218	3	189.44	GTCCCCACCAGGGGTGATGTGGAAAAGGGGAAGAGGGGCATATTGGTTGCCCCAAC[A/G]TCTGGGACAATTATTAGAATTTAGGACCGAGGATAGAATATTCAACACATGTA	A	G	0.12	0.41	0.47	0.46	0.54	0.14	0.26	0.30	0.39	0.48	0.55	0.065	0.019	0.006	0.065	0.0027		
rs2067078	3	189.52	TAAGAGCTGAAGTTAAAGTCATGTTAGGTAAGAGCTGAAGTTAAAGTCATGTTTAT[A/T]AGCCATCCTAGGGAGCTCATGAATTTCATGATGTTTCTGTGCTGGATAAACATTT	A	T	0.55	0.04	0.02	0.04	0.04	0.41	0.14	0.07	0.07	0.02	0.06	0.189	0.017	0.002	0.113	0.0047		
rs1568598	3	189.85	ATTCTTCATGCAACCAAAGAATTCCAAAAACGTATTTCTTTGCAGCTAGCATAAATCT[C/G]CAGTTCCACAATGGTAGCAGTGGTGGATTTTCCACTCAATGTTTTAATGATGAATGTA	C	G	0.71	0.83	0.81	0.71	0.71	0.18	0.11	0.58	0.71	0.80	0.81	0.012	0.282	0.031	0.244	0.0002	1	
rs1515504	3	190.97	TCGGTATTTGGCAGTCACTCCAGTTAATTAAAGATGCATTTATAGCCTGAAAAGTTGTAG[A/G]AGTAGTAACAAGAACGCGAGGTGAAAAAGGTAGGAGAGGTTTTGGGTTTAGGCAC	A	G	0.31	0.42	0.34	0.40	0.38	0.45	0.76	0.39	0.54	0.32	0.36	0.000	0.099	0.006	0.011	0.0000	1	
rs538338	3	191.21	TTCCTTTAATTCTATCACCAACAAGCACCTCAGGTTGTTTGAGCTGGCCAAGGATT[A/G]CATGAATGGAACAGGAGATATAGAGTTGTCAAAGGCATAATCTCTCCTGTGGCC	A	G	0.74	0.89	0.88	0.90	0.91	0.91	0.79	0.78	0.89	0.81	0.86	0.014	0.002	0.010	0.002	0.0002		
rs532616	3	191.27	CAAATTATTGTGGAAGTAGAACACATTGTCTTTGTTCTTGAGGAACATTAAAGTGA[A/C]GGTGGAACCAACCCTATTGGAAAGACATAAGGACCCAAAGATTCTCCTGGAAACA	A	C	1.00	0.95	0.91	0.95	0.88	1.00	1.00	0.98	0.89	0.89	0.90	0.030	0.033	0.015	0.033	0.0000		
rs3864005	3	191.34	AATTCTCACTCTGTTTCTACAGAATTTAAATATCTGAAGGTAAGGCCAAGGCCAGC[A/G]GAAGTTTGAAAGGCAATAAAGTATCTTGGTGCTCCTGAAAATAACTAAAGGAAAAC	A	G	0.17	0.45	0.40	0.48	0.41	0.15	0.49	0.52	0.50	0.41	0.40	0.043	0.001	0.000	0.064	0.0004		
rs1559018	3	191.62	CATTTCTCTATGCAATAAACATATGTTGTATTGGGCTTACACTGTGTGCTAGAT[A/G]CAGATTCACATTATCATTATATTCACTAACCATTACAGGGATGAATGATATCATACAGTAT	A	G	0.65	0.68	0.65	0.67	0.72	0.27	0.31	0.72	0.61	0.65	0.70	0.002	0.021	0.023	0.045	0.0003	1	
rs2048417	3	192.10	TACGAGTCTTTTTCTTAGAATTTCCACTAATACATACTCAAAACAATCTAACCC[A/G]TGTGCAGATTATTTCAATTATTTTTATTTTGAATGGGGTATCATATTTTGCATTTTGAT	A	G	0.78	0.43	0.44	0.50	0.46	0.39	0.27	0.33	0.29	0.46	0.54	0.064	0.010	0.006	0.001	0.0068		1
rs975121	3	193.22	ACCATCATAAAATAATGCAACCTGTGACGTACTGGAAATGTAATGTTGGAGGTAGAT[A/G]GACACAACTACTCAATACTCAAACCCTTGATTTTTGACCTTGAGCAGTTACTTA	A	G	0.90	0.45	0.46	0.50	0.52	1.00	0.78	0.79	0.57	0.58	0.51	0.137	0.076	0.098	0.267	0.0048	1	
rs1501622	3	193.47	TCTTAACTTTCACAATTAAAAATAAGTTAGTCACATTGGAGGTGAGACTGATCATCT[A/G]TTTTACAGAAAATATTGTTAACATTTATGTTACAGGATGAAAACATTTACAGACAA	A	G	0.62	0.66	0.64	0.79	0.71	0.73	0.79	0.83	0.68	0.63	0.63	0.001	0.010	0.022	0.003	0.0004		
rs2986	3	193.85	AAAAGAAAAAGAAGTCAGAGGAAACAGTCTTTGATGTTATGAGGCTGAGACACTACTCTTCCTTCA[A/G]GACATTTCATTCTGACTATAAGTGAATAAATACATTGAAGACTTCA	A	G	0.43	0.46	0.45	0.57	0.50	0.45	0.09	0.53	0.46	0.57	0.59	0.002	0.059	0.011	0.002	0.0143	1	1
rs769592	3	193.97	TTGTTGGGTTAGCACATTACTGTTAAGAAAACACGATGTGTGCCGTACTTATT[A/C]CACCACCAGCCTCTCCCCATCCCTGCGTTAAACTCAGTCATCCTTTTGCCTACATAGGAA	A	C	0.45	0.47	0.49	0.44	0.50	0.23	0.82	0.53	0.36	0.49	0.48	0.000	0.070	0.006	0.034	0.0002		
rs720927	3	194.70	AAGTCAGTGCAAAGGGTCTGTAAGCCTAGAAGAGGAATCTGGAAACCCTTTA[C/G]AGAAGAGCCTTCTCCCACAGGATTTCAGGGCAGCATGTGTGAGACCCAGGCTGACT	C	G	0.57	0.47	0.51	0.43	0.68	0.41	0.68	0.53	0.54	0.59	0.55	0.000	0.014	0.001	0.007	0.0001	1	
rs8513	3	195.40	GTGGGCTGGCAAGCCACTTCCGGGGAAAACTCCTTCCGCCCCAGGTTTCTTCTTCTCTTAAGGAGAGATT[A/G]TTCTCACCAACCCGCTGCCTTCATGCTGCCTTCAAGCTAC	A	G	0.19	0.34	0.27	0.29	0.25	0.14	0.00	0.15	0.21	0.17	0.27	0.006	0.117	0.009	0.020	0.0001	1	
rs789993	3	195.65	GTGGCCTCGAAGAGAAAGATAAGACACATCTTATAGGAAGCAGAAAGTGAGACG[A/T]GCCGTTAAAAAAGAGAGAGAGAAAATACCACGGGGGTCCTTTTGAATAAAATGTGAC	A	T	0.16	0.46	0.47	0.46	0.54	0.18	0.08	0.18	0.43	0.54	0.55	0.049	0.092	0.030	0.032	0.0043		
rs711995	3	[illegible]	[illegible]	[illegible]	[illegible]	[illegible]	[illegible]	[illegible]	[illegible]	[illegible]	[illegible]	[illegible]	[illegible]	[illegible]	[illegible]	[illegible]	[illegible]	[illegible]	[illegible]	[illegible]	[illegible]	[illegible]	[illegible]
rs1075870	3	196.00	[illegible]	[illegible]	[illegible]	[illegible]	[illegible]	[illegible]	[illegible]	[illegible]	[illegible]	[illegible]	[illegible]	[illegible]	[illegible]	[illegible]	[illegible]	[illegible]	[illegible]	[illegible]	[illegible]	[illegible]	[illegible]
rs823515	3	[illegible]	[illegible]	[illegible]	[illegible]	[illegible]	[illegible]	[illegible]	[illegible]	[illegible]	[illegible]	[illegible]	[illegible]	[illegible]	[illegible]	[illegible]	[illegible]	[illegible]	[illegible]	[illegible]	[illegible]	[illegible]	[illegible]
rs903196	3	196.90	[illegible]	[illegible]	[illegible]	[illegible]	[illegible]	[illegible]	[illegible]	[illegible]	[illegible]	[illegible]	[illegible]	[illegible]	[illegible]	[illegible]	[illegible]	[illegible]	[illegible]	[illegible]	[illegible]	[illegible]	[illegible]
rs1862768	3	196.91	[illegible]	[illegible]	[illegible]	[illegible]	[illegible]	[illegible]	[illegible]	[illegible]	[illegible]	[illegible]	[illegible]	[illegible]	[illegible]	[illegible]	[illegible]	[illegible]	[illegible]	[illegible]	[illegible]	[illegible]	[illegible]
rs9343	3	197.29	[illegible]	[illegible]	[illegible]	[illegible]	[illegible]	[illegible]	[illegible]	[illegible]	[illegible]	[illegible]	[illegible]	[illegible]	[illegible]	[illegible]	[illegible]	[illegible]	[illegible]	[illegible]	[illegible]	[illegible]	[illegible]
rs338180	3	198.34	[illegible]	[illegible]	[illegible]	[illegible]	[illegible]	[illegible]	[illegible]	[illegible]	[illegible]	[illegible]	[illegible]	[illegible]	[illegible]	[illegible]	[illegible]	[illegible]	[illegible]	[illegible]	[illegible]	[illegible]	[illegible]
rs338217	3	198.35	[illegible]	[illegible]	[illegible]	[illegible]	[illegible]	[illegible]	[illegible]	[illegible]	[illegible]	[illegible]	[illegible]	[illegible]	[illegible]	[illegible]	[illegible]	[illegible]	[illegible]	[illegible]	[illegible]	[illegible]	[illegible]
rs718501	3	198.41	[illegible]	[illegible]	[illegible]	[illegible]	[illegible]	[illegible]	[illegible]	[illegible]	[illegible]	[illegible]	[illegible]	[illegible]	[illegible]	[illegible]	[illegible]	[illegible]	[illegible]	[illegible]	[illegible]	[illegible]	[illegible]
rs6788537	3	198.49	[illegible]	[illegible]	[illegible]	[illegible]	[illegible]	[illegible]	[illegible]	[illegible]	[illegible]	[illegible]	[illegible]	[illegible]	[illegible]	[illegible]	[illegible]	[illegible]	[illegible]	[illegible]	[illegible]	[illegible]	[illegible]
rs2686085	3	198.55	[illegible]	[illegible]	[illegible]	[illegible]	[illegible]	[illegible]	[illegible]	[illegible]	[illegible]	[illegible]	[illegible]	[illegible]	[illegible]	[illegible]	[illegible]	[illegible]	[illegible]	[illegible]	[illegible]	[illegible]	[illegible]
rs2060086	3	198.56	CTCCAACCATCAGGGGATGGGTTTGTGGTGGCTGGAGTCACCAGACCATAGGTCC[A/G]CACAGGCAAAGGCCTCCTTCCTACTTACTCTGACAAGAAGGAGCGGCTCTCTGC	A	G	0.29	0.18	0.26	0.20	0.23	0.00	0.00	0.27	0.14	0.23	0.15	0.002	0.101	0.000	0.101	0.0107		
rs1864668	3	198.63	TTACCTTGGAGAATCTTGATGGATATTTTTTTGGTGCCTCAGTGAACGGGT[C/G]GGGGGGAATGGTGGTTTCTAAGAGGCCTCCTATCTTGAGCTCAGGATCCCTTCTGAGCTT	C	G	0.57	0.55	0.48	0.34	0.48	0.91	0.94	0.78	0.46	0.41	0.49	0.005	0.137	0.032	0.114	0.0007		
rs935971	4	1.04	TAAACATTAGTTTTAGTCTCAAATATTTTTCCCTCAAAACTCAGAAGCTTAGT[A/G]AGAAAATAAAAATCAATGAGCAACGTGTCCACTCCCTGGCACTTCTGGACTTCCCG	A	G	0.93	0.79	0.78	0.76	0.82	0.73	0.50	0.74	0.89	0.77	0.79	0.034	0.034	0.000	0.001	0.0000	1	
rs963598	4	1.14	AAAAACCATGCAGCATTATGTTCACTGCCAAGTAATATGGACGTAGAGGATGG[A/C]AGAATTCTGTAAAACCACTTCTGGAGTTCAACATTTAACCGAGCACATTTTACTTACCTG	A	C	0.26	0.72	0.72	0.74	0.66	0.72	0.84	0.60	0.67	0.73	0.74	0.118	0.035	0.000	0.005	0.0001		
rs736455	4	2.24	GGCAGACCCAGCCAGAGCTCACAGCCCAGGCCAGCGTGTTCTCAGATCAGTCA[A/G]GGAGGGAGGAGGCCCAACCCCTTGGGGGAGGATTCCCTTGCAAGAATAAAACTC	A	G	0.36	0.51	0.52	0.55	0.64	0.10	0.74	0.36	0.65	0.49	0.48	0.008	0.034	0.017	0.102	0.0006	1	
rs362331	4	3.25	GTGAAGCAGACAGGAACTATGATAGGAACCCACCGTTCATGGACGGGGACTCACCGGCCTCCAGGATGAAGTGCACACAGT[A/G]GATGAGGGAGCAGGCGTGGGTCACAA	A	G	0.43	0.57	0.58	0.62	0.66	0.59	0.63	0.69	0.39	0.61	0.67	0.015	0.001	0.009	0.000	0.0028	1	
rs934498	4	4.33	AGGGTATATGCATTTATTTTGCCAAAAAATGCACCGAAATTTAGTATTACTCC[A/C]ATTTCCATCTCTGTGTTTATCTTTTCAAAATAGCTCTCTTATTGGATGCTGTCAACCAAAC	A	C	0.45	0.29	0.25	0.32	0.20	0.18	0.64	0.47	0.36	0.18	0.23	0.020	0.068	0.006	0.005	0.0011	1	
rs920683	4	4.36	CTTTACAGATGCGGGGCTGAGGCACAAAGAGGCCACACCGTAATGAAACTG[A/G]GCTAGGCTGTGGGCTCTGTCGTCTTAAGCGCTACTCTCAACTACAGCGTGGAAGGAA	A	G	0.55	0.69	0.68	0.67	0.71	0.69	0.42	0.63	0.68	0.76	0.76	0.005	0.029	0.000	0.000	0.0067		1
rs873924	4	4.39	GCACCTCCCACAGGAATGGAAAGCCTTCCAGAGTGAGCAGAAGCTGTGAGGT[A/G]ACCCCCTCTCCTCAGATCAGGGTTGTTCAGTGTCTTGGGAGGGGAGGGGAGAATCCT	A	G	0.40	0.42	0.50	0.44	0.39	0.50	0.64	0.35	0.31	0.40	0.44	0.009	0.004	0.012	0.001	0.0036		
rs726111	4	5.98	AACCCCAAGAGAAACTGTTCATGAGAAAATGTGAACAGAGCAGGTGCTGACCAAG[A/G]ACAGGTTAAACCTAGTTCAACTTAATGTGATTTTTACGGAATGCAACCTGGTTCCA	A	G	0.10	0.41	0.50	0.54	0.64	0.82	0.29	0.55	0.36	0.52	0.51	0.109	0.037	0.006	0.054	0.0001	1	
rs875864	4	6.45	CGTCTTCTCTTCCTCCCTCCCAGACCCTCTCACCTCCTTGTCTCACTTGAACCTCTAATCCTGGGAAGGCTCCACCATAAGCAC[A/C]CTACAGATAATGAAACCAAGGCTCAGA	A	C	0.81	0.48	0.44	0.38	0.55	0.36	0.33	0.50	0.57	0.48	0.56	0.079	0.008	0.003	0.002	0.0077		
rs878283	4	6.49	AGAAGGACACCAGCCATTGGACTTGAGGCCCACTCGAATCCAATATGACCTCA[A/T]CTTAACTAGTGACATACACAAGAACCCTATTTCCAAATAAGGTCACATTCTGAGGTTC	A	T	0.60	0.81	0.77	0.77	0.82	0.86	0.41	0.68	0.75	0.68	0.68	0.034	0.085	0.020	0.001	0.0108		1
rs875579	4	6.63	CATGGATTGGGGCATCGACCTGTGGACCTGTGGGAAGGGGAAATGCCTGACTC[A/G]ACACTCAACTTCCCTGTCCCTGGCCAGAGACAGGCGTCTTGCAAGAGTCTGCACT	A	G	0.05	0.38	0.36	0.32	0.25	0.86	0.39	0.40	0.36	0.30	0.34	0.108	0.000	0.002	0.105	0.0001		
rs1557816	4	7.84	TGGCTGGGACAGGGCAGGGGTCTTCCCTTGCTTCATGGAGCCCTGTGCCCCTCC[A/C]TCTGTGATCTGAGGCAGGAGTGCACACAGTCCCCCACGCTGCTTGTCTTTTGTCC	A	C	0.50	0.42	0.33	0.43	0.29	0.59	0.77	0.50	0.29	0.36	0.42	0.021	0.111	0.028	0.048	0.0030	1	
rs881641	4	9.88	AAGAACAGGGCTTTGCTGGGCCTTTCTCCATGTAATCCATACAGAGACGTTTGA[A/G]CCTCCAGAATGGGGTCTTGGGGGGCTCAATGAGACCAAGTCTGACCCTCACCCCC	A	G	0.12	0.26	0.25	0.24	0.27	0.14	0.03	0.22	0.32	0.32	0.33	0.013	0.042	0.000	0.007	0.0024	1	
rs747357	4	9.90	CTGAAAAATTGGCTTTCCCCTTATTTCTCTAGGGACAGAGGAGACTACTTTG[C/G]CTTCAAGAAATATGACTCGAGGGCCAACTGGCGCATCCACTGGATGCCAGTCCTGATG	C	G	0.36	0.46	0.49	0.49	0.46	0.18	0.35	0.43	0.46	0.57	0.49	0.005	0.003	0.001	0.043	0.0001		
rs1981635	4	10.35	GGTGTTCTTTCTACAGTGGATTATTGAAGCAAAATATTAACAAGGCTGGAAAATAAA[C/G]AGCCATCTACCTAGACAGTAAGTGGTTAAAGCCTGTTTGAAAATTACTTACAAAG	C	G	0.60	0.76	0.76	0.78	0.68	0.68	0.86	0.74	0.71	0.69	0.72	0.020	0.007	0.000	0.007	0.0014		
rs1876265	4	10.91	ACTTGTAGCAGTTGAGGGATCCATCAATAAGGCTAATGAGCAGCATGCTATTTTGA[A/C]TGTGAAACACTCACACAGGTCACATGACCATGAAATGACCAAGGAAATGATGAGT	A	C	0.26	0.24	0.21	0.21	0.16	0.50	0.73	0.25	0.29	0.11	0.16	0.003	0.145	0.002	0.046	0.0025	1	
rs1372887	4	11.75	AAATGGTATAGCTTGGATGTTCGTCTCCTCCAAATCTCATGTTGAAATTTGATCC[A/G]CAGTGTTAGAGATGAGGATCTAATGGTAGGTTTTGGGGTTTCAGGGAGCGATCCCT	A	G	0.50	0.53	0.44	0.46	0.57	0.55	0.56	0.48	0.57	0.54	0.59	0.005	0.023	0.021	0.017	0.0117	1	1
rs717376	4	11.83	GAATGTGACAACAGGAAATAAAACTGTAACCTTCTTGCACATAATGAAATTCAGTGC[A/G]TACCCACGCCTATCACAGTACCAGACATGTTATAGGAAATTAATGATAATGACCT	A	G	0.24	0.42	0.34	0.32	0.45	0.59	0.36	0.29	0.50	0.36	0.37	0.010	0.000	0.000	0.036	0.0000		
rs763318	4	12.71	AGAAAGGAAAACAGAAAAACAAACATCAGGTTCCCGGAAGAGTGAAAGGAAAACAA[A/G]TGTGTATTGAATGTCTACTTTATGTCAGGGGTTGCCCGCAGGATGCTTTTCTGTG	A	G	0.67	0.48	0.45	0.38	0.43	0.55	0.53	0.64	0.39	0.43	0.41	0.016	0.002	0.018	0.003	0.0023	1	
rs1157946	4	13.29	GTAGTATTGATTCCCTGAGAGTCAGAGAGGCAAAAGAAGCTGATGACTCGTCCA[A/G]GAGCAATCATTTGGAAAAGCTGAACCCAGTGACTCAGGCCTGACATCTTTTGATAC	A	G	0.10	0.21	0.23	0.22	0.23	0.00	0.01	0.23	0.32	0.36	0.28	0.037	0.100	0.001	0.123	0.0018	1	
rs758879	4	14.12	AATCATTGGATTTTCATGATGGGTGTTTTGTTGATAATCTGTTAATTTCTGTT[A/G]ATGATTATGTTGATGAAAGCTGATATCTAGTGATACTTGCCTCTTCTGATATAGTCCCA	A	G	0.19	0.50	0.45	0.49	0.39	0.14	0.35	0.31	0.46	0.32	0.42	0.042	0.001	0.010	0.058	0.0003	1	
rs735855	4	14.15	CTCAGTTTCCTCACTGGCTATTCAAGGTAGCTGAATTACACA[A/T]TCCAGAAGCTAATGGGAGTCAATACTCTGCAAGGAAGACTCTGACCTATTAGAGACAGAAGACAAGAG	A	T	0.74	0.45	0.53	0.48	0.46	0.82	0.65	0.45	0.29	0.42	0.45	0.026	0.012	0.005	0.058	0.0042		
rs1875355	4	14.19	AGTTGCCTCCTCAGGTCTCATCCAAGCTCTCTTCCCTTGCTAGTAGACAGAGAC[A/G]TGCTTAAAAATTCAGAGGTATATTTTTCTCATTTCCCTCCACCAAAGCTGCATTGGAA	A	G	0.48	0.52	0.48	0.49	0.54	0.50	0.35	0.47	0.54	0.56	0.55	0.000	0.007	0.000	0.000	0.0016		
rs2047205	4	14.76	GCCTAGTATTCTAAATTTTGTTACTGCTTTTATACCCTGACTTATTCCAAATA[A/G]TCAGTTTCCTATGGAGAAACCGTTAGTCAAAAGCTGGGAGTTAGCCTTAATCAAATTACA	A	G	0.62	0.52	0.48	0.50	0.45	0.50	0.29	0.73	0.57	0.55	0.48	0.007	0.027	0.031	0.000	0.0000	1	
rs1861048	4	15.23	AAAGGGTGGACTAAATATTTGCCCCTGAATTACCCAGT[A/G]ACACTTTAGCTGCTGTTTGCAAACTATTTGAAGTTGGGAACAAACTGATTTTCCTGTTTCTCTTTCCTCCAAATT	A	G	0.36	0.58	0.58	0.43	0.57	0.36	0.33	0.58	0.46	0.54	0.58	0.015	0.026	0.001	0.018	0.0001		
rs2041673	4	15.30	GGTGGGACTACTGTGTCCGACAGTTTCATAGACCTGAAAACCAGAGAAGAGAAATA[A/G]CCAATAAAAACAGCAGTAGTGTGTGCTCACGCTCAGATAATCAGTCCATGTTGTT	A	G	0.21	0.16	0.21	0.15	0.18	0.00	0.00	0.13	0.14	0.14	0.13	0.000	0.080	0.012	0.080	0.0031		
rs1357233	4	16.89	AGGGGAGATCTAATTTGGCCTAAATTGGCTTGCTACAAAAGGTGCTAAGGAGAACA[A/C]AAATTGTTCTGCAATATGGATGTTTGTGAAAGGGTTGGGCTGTATCCATTGACAAT	A	C	0.88	0.79	0.82	0.80	0.80	0.77	0.46	0.89	0.86	0.78	0.76	0.008	0.066	0.002	0.000	0.0016	1	
rs1325107	4	17.80	TCCTCAATTTTTTATTATGTAACATCAAATACAAAAAAATACATGCAATATATAAGATTTGATGCATAATCACAAAATGAACACCCTT[A/G]AGCCCACCACGAACTTAAATAGAGT	A	G	0.45	0.31	0.31	0.38	0.30	0.50	0.40	0.11	0.21	0.27	0.36	0.011	0.003	0.051	0.014	0.0013	1	
rs1477898	4	18.29	CCATTCTGGCCTAAACGTTGTGCCCAGCTTTATCCCCATGCAGTTGAAATTATCTATATTCCCATTTCAGTGACTGAGTGGCACATAGTAA[C/G]TGCTGCAGAAATGTTTGTGGA	C	G	0.50	0.51	0.52	0.51	0.34	0.68	0.67	0.44	0.61	0.41	0.48	0.003	0.024	0.001	0.031	0.0006		
rs1850548	4	18.46	CACTTGTACCCAGTGATAAAATTCCAGAGACAGCATTATA[C/G]AAGGAGTCAAACAGTGTTATTGCATAAAAAGTGCCATATTTCAAGAAAGTGACAATACTGTGCTCTGGAAATT	C	G	0.74	0.51	0.46	0.43	0.48	0.55	0.77	0.42	0.54	0.46	0.44	0.026	0.048	0.003	0.000	0.0006		
rs1517985	4	18.48	AACCCCACACGTTAGCTTTGCCTGATTGTAATATTTACTCTTTTGTGTCTTTTGGT[A/C]AACTTGATGCTTGTGAAATTCATTCATGCTGTTATGTATAGTGGTGTTTTGTTATTATC	A	C	0.21	0.34	0.32	0.27	0.32	0.36	0.71	0.31	0.43	0.26	0.23	0.013	0.073	0.002	0.000	0.0057		
rs207338	4	18.80	ATCTGGTTGGACTTCAAAAGGTTGCATGCCTCGTTATCTGTTTTGCACTCCTA[A/G]CCAAAGCTACTTTGGGAGCACAAGATTCCGCTTAAAATACGTTAGTGTTTTCCACAAC	A	G	0.45	0.56	0.52	0.41	0.30	0.55	0.69	0.33	0.50	0.54	0.44	0.002	0.019	0.011	0.001	0.0060	1	
rs516774	4	19.98	TAACCATCTTAGTATGCCTACTGCTGCCCTTAATACACCTGAACTGAAAACAGC[A/G]GTACTGAGAGCTCATGTTCATATCTGTCCTGAATATATTTTTCTGATTGCCTAAACTG	A	G	0.57	0.76	0.68	0.74	0.61	0.73	0.94	0.66	0.75	0.74	0.65	0.001	0.073	0.000	0.005	0.0012	1	
rs729918	4	20.26	CAATCAATCCAAAGACATATTTTACTGGGTATTATAAAAACTTTTTGTGTTTCT[A/G]AGGCAGTCCCCAAATTACAGATAGTGCTATATTTTAATCCTGTTGATCACAGAGATTGC	A	G	0.55	0.48	0.40	0.34	0.41	0.45	0.44	0.42	0.39	0.34	0.31	0.020	0.003	0.004	0.002	0.0053		
rs720853	4	20.85	AAGCTCGACTGGGAGGATAATATAAAACAAGAGGAAAGAAAAGTCAGATTTAGAGT[A/G]AGAACATTGTCAATAAGAATTGCCAGGGTCCCCTAGATGGTTCCAATGAGCCTTC	A	G	0.17	0.20	0.15	0.18	0.23	0.23	0.19	0.20	0.14	0.24	0.16	0.001	0.007	0.005	0.009	0.0001	1	
rs730349	4	20.92	TTTGTGAGTTCTGGATTCTTCTGACAGAGGATTCTGAGTGAAATCTGATCCCAT[A/C]GCTGTTCTGAAAATGCACTTATATTGACTTAGTATATGGAAACATGATAATGCCAATT	A	C	0.53	0.46	0.56	0.50	0.39	0.50	0.29	0.48	0.68	0.49	0.46	0.000	0.104	0.002	0.034	0.0042		
rs1968778	4	21.57	AAGTCTCTTACTATTTTCTCATCTTCCTGCCATCCTCTGATGAGGATTTGTTATGAGGACCTGTGTTT[A/G]GTCATCACAGGTGATCTCCACATCCCACCCCTATAACTATTAGT	A	G	0.14	0.52	0.48	0.49	0.63	0.23	0.01	0.33	0.62	0.46	0.42	0.052	0.151	0.006	0.023	0.0005	1	
rs216113	4	23.27	TCTTCAGCTACATGGTGACATGGAGCCTGCCATGAGGATCCAGGATGCTCGGCTGCCTCTGATCC[A/G]AGGAGCTGACACAGAAATAACTTCCATTGCTCATAGATGACTCTTC	A	G	0.21	0.44	0.47	0.40	0.41	0.27	0.01	0.31	0.43	0.47	0.48	0.061	0.231	0.026	0.048	0.0000	1	
rs1476880	4	24.49	GGGAAGAACCTCAAGGGCAATATTTTAGGGCATTTGAGCTCACGAGGATGATGA[A/C]GATGGCTGACTTGCATCCTGCAACTGGTTCAACACCTACCCATAATACCAATGGG	A	C	0.31	0.40	0.43	0.46	0.59	0.36	0.35	0.38	0.39	0.38	0.41	0.003	0.000	0.001	0.000	0.0000	1	
rs936232	4	25.56	GCTCCTCAGAGGAAACAGGCTGAGGGAGAGTGGGAGAAATGAGGGAAATGTGGAA[A/G]GAAGCAGAGTAAGTCCATAGAACTCTTAGCAGTCTGGATTGGAGCTGCGGCTTG	A	G	0.62	0.39	0.33	0.32	0.36	0.36	0.53	0.30	0.39	0.31	0.38	0.049	0.029	0.001	0.002	0.0036	1	
rs925470	4	25.77	CCTCTGCTACGGATGTCCCCTCAATCTCCTGTGGGCAAAGAAAGGGCAAGGGCACA[A/T]GTACTTCCAAGAAACAAACAATGATTTCCAGTTGTGATGCTGCATAAATACAGAT	A	T	0.55	0.47	0.44	0.49	0.50	0.64	0.64	0.60	0.39	0.49	0.48	0.007	0.018	0.015	0.023	0.0005		
rs1456087	4	27.26	TGACCTTTGAGATACATTCACTATTGGTGAATTGACTGTCAAGTTTTC[A/G]ACACCGCAAGGTGAAGAACCAGACATTGAATGATGAGAAATTCATCATGAGAATCACTACCACC	A	G	0.83	0.64	0.58	0.60	0.64	0.50	0.78	0.43	0.75	0.62	0.63	0.055	0.040	0.007	0.001	0.0032	1	
rs980458	4	27.57	CCTCTCAGCTCTCAGAAGTCAGTTTCAGATCCCCATCACATGGTCCCTCCACAGGT[A/T]GTTCACACAAAGATGTTTCTCCCAGGCCAGTAGGAGGAATTTTTCAAAATGTTAC	A	T	0.90	0.68	0.67	0.72	0.46	0.73	0.44	0.74	0.68	0.66	0.66	0.046	0.018	0.034	0.006	0.0000		
rs890459	4	29.19	AATATTAAGGTTAGCTTGGCTCTAAGATAATAAACATATATCCATATTTTGCT[A/C]TTTATGTTGCTCACTTTGAACTCAGTAAGGAAAATAAAGTAGGAAAAATTCATTTTAAAT	A	C	0.55	0.33	0.34	0.32	0.30	0.68	0.65	0.69	0.58	0.42	0.36	0.032	0.067	0.069	0.075	0.0003	1	
rs902659	4	32.09	TTTTGGCCCATGTCCTCCAAGAGTAGGCCACATAATTGTAAGATGCATGCCAT[A/G]GATTATCAGTATTTTCTGGCAAGGAAGCTATATACACATTCCTCAAACATATGAGCGAT	A	G	0.21	0.55	0.58	0.65	0.59	0.50	0.17	0.63	0.50	0.58	0.52	0.051	0.069	0.004	0.000	0.0004	1	
rs725292	4	37.04	TCACTTTGTGGCTACTTGTTTTCATTTTTATGGTGCCTTTGGCTGAAAGAATCTCA[A/G]TTTTAGCGTGAAAACTTATCAATGTTTTCTTTGTATTTGTGCTTTATAAACTTTAAG	A	G	0.14	0.35	0.35	0.29	0.32	0.23	0.43	0.14	0.43	0.39	0.37	0.032	0.000	0.047	0.013	0.0000	1	
rs12142	4	37.51	GGAGGAGGAGAAACACAAGACCTGTAAACATCAGTTGCTTTGGGACACA[A/G]GAATTCTCATCAGATAGTTCAGTATAAACCAGTAAAAGCGTATGTGTTG	A	G	0.10	0.42	0.36	0.46	0.43	0.00	0.00	0.42	0.46	0.36	0.32	0.034	0.134	0.011	0.134	0.0000		
rs1039559	4	38.73	AACATACTAGCTGCAGCCACCTGAGCAAGGGCTAACAGAGGGGGCAAGCAGCAGACACATCAAAAAGTCTGGGAAATATGCAAAGATATATGGA[A/G]AAAGATATGTGGGAG	A	G	0.88	0.58	0.52	0.62	0.55	0.86	0.36	0.46	0.46	0.64	0.60	0.094	0.010	0.000	0.085	0.0051	1	
rs974734	4	38.80	AGATCATTCACAGTAGTAGACAAGCTTTGAAGATTTATAAATTGCCTGTGGAAAGTAA[C/G]GAAAGAAAATAACCACATGTTCAATTGCAATTGATGTCTACTCAGAACCTCATC	C	G	0.52	0.54	0.51	0.59	0.48	0.45	0.16	0.42	0.46	0.60	0.58	0.002	0.061	0.001	0.000	0.0052		
rs1046655	4	39.02	GGAACTTCCCCACCGTCCGGTACATGGCAGGCATTCCACAAATGTTTTCT[A/G]AAAGTTGAAATAGACATCTTTTCATGAACTCTGTAATATTTGAAATTATT	A	G	0.52	0.61	0.59	0.68	0.54	0.50	0.72	0.57	0.69	0.54	0.66	0.001	0.007	0.002	0.006	0.0035		
rs278973	4	40.31	CTTCCTTTTTCCAATCAGGAAACAGCTCTCAAACTCCCTCTCTCTTTCAATCAGT[A/G]CTTCATTGTTTTTGGAACTATGGAAGCCTCAGTGCCAATTTTATAATCCACGCCAGAC	A	G	0.14	0.43	0.43	0.38	0.55	0.45	0.26	0.34	0.50	0.51	0.45	0.062	0.015	0.001	0.000	0.0000	1	
rs2035383	4	40.60	ACAAACTACCATGAA[A/G]CGAAATTAGCCATTGAATCACATACATTTCTCTATCCCATGAGAAAATAAAAATGGAGCAACTCAAGCAATCCCTTCATACTCTCCCTAAATTAAACA	A	G	0.38	0.48	0.50	0.48	0.61	0.05	0.09	0.40	0.50	0.55	0.45	0.020	0.142	0.012	0.193	0.0047		
rs790142	4	40.65	CAGAGAAACAGACAGCAGGATTCAAGAGAGAACAAAGAAACAAGATCAATCT[A/G]GTTTGCCAAACAGTGGCTCCCTGTGTCTGAAACAATTGGAGAACTTTGCAGCAATTT	A	G	0.55	0.38	0.40	0.35	0.32	0.14	0.81	0.43	0.61	0.34	0.33	0.005	0.064	0.000	0.071	0.0032		
rs951149	4	40.94	ACTCTTGCTCAGATGCTACATGGGGTAACAGCTGAGATAACTAACTGATCATTGATC[A/G]CCCTTTCTCTGATATATCAAGGATATCCTTTCCTTTTTTTCAAGGAAAGGAAAGAT	A	G	0.29	0.32	0.41	0.40	0.43	0.18	0.41	0.47	0.57	0.41	0.38	0.003	0.007	0.018	0.010	0.0001	1	
rs1565114	4	42.24	ACCTTAAATACCCAACACTCAGAGGCAGAACAGGAAGGAAATAACCAAAATAAAA[A/T]CTTCCATCTCACATTCTCCTATTTCTTTTCACATGCCACTGGTCACAGCAAGTCACAT	A	T	0.38	0.30	0.29	0.37	0.38	0.36	0.17	0.34	0.32	0.26	0.26	0.001	0.022	0.001	0.001	0.0031	1	
rs969992	4	45.03	CATAAGAATTAATCACATAAGAAAATTAAATTGAATCCAATATGCATTCTATACACA[A/G]CATTTTATAGTGATTACCATATTATCCTTTCCTTCTTTTAAAACATAAATTAAGGGC	A	G	0.62	0.58	0.62	0.63	0.63	0.50	0.31	0.31	0.64	0.55	0.58	0.000	0.059	0.046	0.005	0.0001	1	
rs1504491	4	45.91	CTCCACTTAGAGATGAGAAAACTGAGACGTAGACAGGTTATGTGAGTTGCCC[A/G]AGATCAGACAGCTAGCAGGTCACACTAAAAAATAATCTGGTCCCAGAGTCTGGAGGCT	A	G	0.83	0.57	0.64	0.67	0.64	0.45	0.52	0.83	0.54	0.62	0.59	0.027	0.008	0.030	0.017	0.0003	1	
rs1866989	4	47.02	AGGGTAATACAAAGTATACAGTACTCCCCTCCACGTTTGGCAATATAGGTAGC[A/T]GATTGTGAAGATACATCCATGGTAAGGAATCTCTTTCTTGGGTTTGAAAATTCTATCAA	A	T	0.52	0.64	0.61	0.63	0.68	0.68	0.93	0.50	0.68	0.66	0.61	0.002	0.108	0.001	0.006	0.0000	1	
rs1350123	4	48.92	CACATGAGAATTAAATGAATAATAATTACAGGGGAAAATCTTAAAATAGTGCCT[A/G]TGATGTAGTGCATGCTATGGTTTTGAGCTTAATTTATGTGACAGCTACTTAAATGAGTC	A	G	0.45	0.74	0.73	0.79	0.80	0.36	0.84	0.52	0.79	0.80	0.70	0.047	0.005	0.057	0.089	0.0005	1	

rs1899130	4	68.19	ATAATTTAAAGACATTGAAAAGGGCTGTT[A/G]TTTCCAAATGACTCTCCTACAGGTTGTTCATTTATTCTTTATTCAGTAGATATCTTCATTCAGTAAACATTTATTCAATAAATA	A	G	0.19	0.32	0.30	0.23	0.30	0.23	0.36	0.35	0.32	0.39	0.28	0.006	0.013	0.003	0.001	0.0001	1		
rs1560605	4	70.83	AATATTTCAAAGTCTCCTGTATGTCATTCTAAAAACATTTTAATCTAATAAGCAATT[C/G]AGGACCATCTAAAGGGAAATATTATGACCTGATTTTCATTTTACGTAAAACACTTGG	C	G	0.17	0.26	0.14	0.17	0.21	0.18	0.10	0.24	0.21	0.26	0.17	0.001	0.000	0.024	0.005	0.0018	1		
rs2063749	4	71.30	AGCCAAAGCAAGAACAAAAACGAACAAAAAAAGTGTTTGCCTACAGTACCCAAG[A/G]ATGGCTGGATTTTGCCCAAGTGCCAAAATGCTAATTTCTAGGTGTCTGTAATTGTTG	A	G	0.14	0.71	0.66	0.67	0.79	0.27	0.45	0.60	0.64	0.54	0.54	0.170	0.046	0.007	0.100	0.0105	1	1	1
rs1490470	4	71.75	ATGGTTAATTTTGCATGTCGACTTGACTGGACACCAGGGTCAGATATGATTCTGG[A/G]TGTGTCTGTGAGGGTGATTCTTGGATAAAATTAACATTCAGATCAGTTGATTGAGTA	A	G	0.60	0.61	0.58	0.65	0.61	0.41	0.71	0.42	0.58	0.54	0.59	0.000	0.009	0.029	0.024	0.0001	1		
rs1043903	4	72.14	CTAAATCTAAACAACCTGATATTGAAGGTTTGCTTTATAGCATATCTTTGGAA[A/G]GGCAACTCATTTTTATGATTAGTGATACTGGGGTGGATTTAAATAGGAGAGAGAATCCAGC	A	G	0.38	0.64	0.67	0.71	0.70	0.36	0.74	0.47	0.54	0.68	0.63	0.044	0.005	0.016	0.045	0.0019			
rs1894292	4	74.82	TTGCAAAAAATAAATGTAGCTTATGTGAACAAACATAAATATGCTCCAATTAGT[A/G]TATGCTCATTGGGAGGGAATCCATGATTTTAGGTGGATCTGGAGATGACCTAAATGCT	A	G	0.25	0.53	0.42	0.55	0.34	0.50	0.34	0.52	0.36	0.44	0.44	0.005	0.000	0.011	0.014	0.0014	1		
rs9131	4	75.43	TCACATGTCAGCCACTGTGATAGAGGCTGAGGAATCCAAGAAAATGGCCAGT[A/G]AGATCAATGTGACGGCAGGGAAATGTATGTGTGTCTATTTTGTAACTGTAAAGATGAAT	A	G	0.12	0.60	0.59	0.62	0.70	0.27	0.77	0.47	0.71	0.54	0.57	0.124	0.024	0.007	0.051	0.0001	1		
rs718607	4	75.74	CATGCATGAGTTTAAAAAGCAATTAAAATAAAACTGTATGTGTGCATGTATAAT[A/G]ATCACCCATCAGCAACTCTAAGAAGAATAAGGGTGGCTTTCCCTTAGGTTAGGTAAAA	A	G	0.33	0.51	0.57	0.52	0.55	0.14	0.93	0.50	0.61	0.64	0.51	0.022	0.131	0.004	0.080	0.0004			
rs717239	4	77.63	ACAGTAGAGTTGGGTTAAGCCCTTAAAACAACACCTACACATCTATGCAACAAGTA[A/G]GAAGTTTCTAGGACTCACTAGAGAAGAATTGGGGCCAGGCTGGAATCTCTTAAAA	A	G	0.73	0.41	0.48	0.43	0.54	1.00	0.96	0.72	0.50	0.46	0.45	0.021	0.130	0.013	0.202	0.0010	1		
rs1511817	4	77.85	CTCCAGGACTTCCCATCAGCCTGGCCAAGACTTTCTCAGAATCACACTGCAGGCC[A/G]AAGCTCCTTTTCCTCAGTCCTCCTGCCTTCCCTCCTGGCTTTCTCTCCCCTTTC	A	G	0.38	0.67	0.66	0.65	0.75	0.82	0.91	0.80	0.57	0.71	0.65	0.031	0.045	0.007	0.014	0.0000			
rs2016985	4	78.37	TAACTTTGATATTTCTCTTAAC[A/G]CTGCCCTCATTCGACTTCACCAAAATTTGTAAGCGCCTTGTTGTTTCAACAGAAGTCATACTCTCCAACCTTCTCATTTTCAGATAAGGAA	A	G	0.38	0.57	0.60	0.56	0.54	0.50	0.52	0.55	0.46	0.66	0.69	0.040	0.007	0.014	0.014	0.0046	1		1
rs881997	4	78.56	ACAATCTCGTTGGCTTCAACAAACCAGGAGTATGGCTTTTAAAGATTTTAAGAATT[A/G]GATAACTAGGTCTGCTTCTCTCTGTGCCATCTTTTCATGTTTTTCTAACAGCATCA	A	G	0.02	0.30	0.27	0.23	0.32	0.27	0.28	0.17	0.54	0.34	0.26	0.069	0.003	0.001	0.002	0.0000			
rs1566485	4	79.00	AAAATCTTGGAAGCAATTTTTTTAGTTTGCTTTTCTGTTTACTTCTCAGTGTTCCAGTACTGGCAGGTTTCTATTTGCTTTTCCT[A/C]TTTGCTTTTTTGTGTTCTAGGACTGGCA	A	C	0.88	0.36	0.43	0.45	0.39	0.18	0.33	0.45	0.39	0.45	0.39	0.111	0.019	0.000	0.046	0.0029	1		
rs1426138	4	80.16	GATGTGGGTAAGATTTACTGAGATGGGTGTTGGGGGTTATTGGATTGTTAGTAATT[A/G]GAGGCCAGGAATTTACTACAGGGTTCTTTAAAAATAGCTCAGTATTTCTCTTAGAGT	A	G	0.07	0.19	0.18	0.23	0.27	0.14	0.28	0.25	0.14	0.16	0.19	0.002	0.009	0.008	0.000	0.0003	1		
rs550592	4	83.21	TACCTGTGTTTGGGTGTGTTTTTCACTCTCAGCCTGATACCACCAGACAGAG[C/G]CCAGGTAGGTTTGATGAATGTCAAATACAGTTGAAAGCTAACAGCCATTTAAAGGAATG	C	G	0.14	0.73	0.67	0.70	0.73	0.55	0.59	0.54	0.68	0.65	0.72	0.182	0.007	0.008	0.009	0.0000	1		
rs1880719	4	84.08	CAGCGCTCTCGTCACGGGATTATCTCAGGAATGCTGCCTCAATGCCTTATCAC[A/G]CTCCAAAGATACAATGTCTACAGGAGTCATTGAATCAGCAGTTTTAATTAATGATAATA	A	G	0.10	0.46	0.50	0.43	0.41	0.41	0.64	0.39	0.32	0.30	0.38	0.105	0.016	0.003	0.005	0.0063	1	1	1
rs1565908	4	84.81	ATATAGTCCATGGAAACCAGTACTTGTGGCTATGCCTTTAAGTAATTCTAAAGT[A/G]TGTTATATCCTTGTTATATCTCCAAAATTGATGTAACTTTAAAACCTGCAACATTTACAAATAT	A	G	0.60	0.40	0.42	0.40	0.43	0.64	0.82	0.51	0.50	0.34	0.38	0.017	0.098	0.005	0.031	0.0017	1		
rs992832	4	85.11	GCAATACTCATATATTGAAAACAATACCCATATACAATGTGGACTCTAAGTGAC[A/G]CCTATACCCTCAAAATATGAATTGATTGCTTACTATGTGCCTGTAGCACACAGTAA	A	G	0.36	0.42	0.45	0.35	0.50	0.27	0.35	0.39	0.39	0.33	0.40	0.007	0.006	0.007	0.025	0.0028			
rs1383972	4	86.84	CTTCCTAATTTTTGTCTTTGAAATCTCTGTCTTTCTCAGCATTTTCCACAAGT[A/G]TTCATACTGACTGTGTTGACCACTTGGTGTTTATGTGATCAATGCTAGATTTGGTTCTATA	A	G	0.14	0.60	0.67	0.65	0.73	0.77	0.95	0.79	0.82	0.64	0.65	0.138	0.064	0.007	0.008	0.0000	1		
rs896656	4	87.22	TGTACATTAAAGTTTCAGAAGAGCTGTTCCAACTCACAGAGTAGAAGCCCAATTCTCAAATGCCAAAAAGGAGGGG[A/G]AAAAGGACTGTGCTGATGTGCAGGGACTCCCTA	A	G	0.68	0.69	0.72	0.85	0.80	0.27	0.58	0.60	0.61	0.78	0.70	0.000	0.012	0.004	0.089	0.0007			
rs1031739	4	87.69	GGAAAAAGAAAAGGGGAAAAATAAACTTGTAAGAGGACATGATAATTGAATATCTCTAT[A/G]CCTCTATTTTAGATAAATTCCTGCAGGCCATCCCCACTTTTGCAAGTGAGA	A	G	0.29	0.45	0.42	0.43	0.30	0.32	0.42	0.29	0.25	0.37	0.37	0.011	0.007	0.014	0.058	0.0018	1		
rs1965907	4	88.90	TAGCACAGATACCATAGTAGGACTTGAGCAAAGAAAATAAATATGGCCAAAATAATGTCTCCCACTATAACACAGG[A/G]CAGGGTGTGGTGGACCCTTTCTGAAGGATTGGCA	A	G	0.24	0.48	0.51	0.60	0.38	0.32	0.43	0.29	0.46	0.54	0.56	0.040	0.000	0.032	0.013	0.0015	1		
rs891674	4	90.62	TAAAGTAAGGAAAAAACTGAGCAAGTTCCAAGAGTCTACCAAGAAAACTGTTTTGC[A/G]CTTCATCTGTTGAATGAACCTTAGGTTTCACAAATAAGGTATACTAAATGCCTAAT	A	G	0.31	0.41	0.41	0.44	0.41	0.41	0.13	0.55	0.62	0.35	0.42	0.001	0.036	0.024	0.002	0.0035	1		
rs1875863	4	90.71	AGGGGGTGGGGTACCAGGAACAACGAGTGGAAAGACCGTGTCCTTGGAAGCAAAAC[A/G]TCAGCTCAAATCATAGCCTACCCCTTATCACACTGTATTGTTTGAGCAGTAACA	A	G	0.26	0.66	0.52	0.70	0.54	0.45	0.26	0.51	0.50	0.57	0.62	0.038	0.039	0.003	0.007	0.0045			1
rs729685	4	90.89	TGTTTCTCCTGTTGATGTAGATGATTCCCTTTCCTTCTCTGTCCAGTTTATGGCA[A/G]CTCAACTCTGTATCCCAAGCTGCCCTCCACAGGGGTGTTGTTGAAGGTTTAACAGC	A	G	0.57	0.70	0.65	0.66	0.61	0.75	0.67	0.88	0.61	0.65	0.63	0.002	0.000	0.051	0.011	0.0003			
rs1156115	4	90.95	GAACATCTGTAGGGAATATATTTACACTTATAATTCCCACTATTTTTGCACTTCA[A/G]TGCCATATAAAACTTCAAACTCAATATATCTAAAGAAGAAATTATTATGTTCCTCTTGA	A	G	0.81	0.80	0.75	0.76	0.82	0.82	1.00	0.90	0.75	0.80	0.72	0.003	0.106	0.041	0.006	0.0003			
rs1381355	4	91.74	TTTGTGAAGATTACATTTGGGGGAGAAACAATAATTAAACCTTTGTAACAAAAT[A/G]AGCTTAGTAAAATGTCAGTATAATTTGAGTTAATAAATTGCAAACAGGCATCAGGGAA	A	G	0.33	0.43	0.52	0.46	0.54	0.36	0.76	0.30	0.46	0.50	0.40	0.024	0.025	0.029	0.015	0.0066	1	1	1
rs1461605	4	92.42	GTTTTCACTGATAGTTTTGCCCTTTCTACCTGGGAGCTCCTAAAGGTATTTAAAAG[A/G]GATGAGAGACATATTTTTAAAAAGCTTTATTTGGAGGGGAATAAAGTGTTATCAATG	A	G	0.65	0.57	0.52	0.61	0.70	0.09	0.63	0.57	0.46	0.48	0.45	0.015	0.004	0.006	0.114	0.0026	1		
rs2046015	4	94.14	CTCCTTCCTTGTACTATTTTGGGATGCTTCCCTAAATGTTCAGTAAGAGGACTGCTTCAAAATCTTTGACTTTTTTTTTCTTTTTGACCAGGC[A/G]AACAAATGGCTAGGTTAAAG	A	G	0.14	0.48	0.51	0.46	0.54	0.64	0.44	0.38	0.61	0.33	0.42	0.076	0.008	0.008	0.008	0.0029	1		
rs1433666	4	94.71	CCCCCCCATTTCACTCATTATCTCTCCAGTATTATATCCTCTGCTTCCCCCTTTTCCACTTCATTCTGGAGACTCCAGA[A/G]ACACTATGTTCCTTGAATATTTCTCTCTGAGAA	A	G	0.79	0.53	0.63	0.48	0.59	0.73	0.33	0.55	0.79	0.54	0.56	0.010	0.054	0.004	0.005	0.0014	1		
rs1509948	4	95.45	AGAATGTCTAAATATGTGTTTAATTTGCACATGCATGCAATTTTTGACACAATATCAGGGCCCAAC[A/G]TTTTTGTTTGTAAGCTATTTACAGTTTCTATTTCCTGCATCACTGCTG	A	G	0.64	0.42	0.50	0.52	0.52	0.45	0.67	0.38	0.57	0.49	0.44	0.010	0.011	0.004	0.001	0.0010	1		
rs1841925	4	95.49	GACAATAGGAAGAGCAGAGAGAGTTGAGTTGCTATGTCTTAGGTCTACAAAT[A/G]GGACCATTTGGAAAGGCATTTTTCCTGTGGAACCTGTCTTCTCTGACTGAAACCTATTCA	A	G	0.21	0.54	0.54	0.55	0.48	0.27	0.22	0.39	0.71	0.53	0.59	0.048	0.060	0.003	0.031	0.0014			
rs1378876	4	97.35	TGGTATATGGAATGTAAGG[A/G]GTCGATATTCCAATGTGGGGGCATAAAACATATGCTTTATCAGCGAGATTGTGAAATAACTAGGGGGCTGTCTAAGGGGATTCAGTGAAAC	A	G	0.83	0.53	0.54	0.54	0.70	0.95	0.85	0.78	0.50	0.65	0.52	0.075	0.068	0.053	0.141	0.0001	1		
rs1011495	4	97.40	AAGGTTCTTCTGATATGTGGCAAAAAAAGTGGTTCACAAAAGCAGTCAGCAAAAGT[A/G]TACCCTGAAGAATAAAAGTAAACGAAATGTGAAGCAAATGGTTCATTTCATTAGGTG	A	G	0.31	0.35	0.28	0.34	0.46	0.64	0.63	0.53	0.25	0.35	0.30	0.002	0.046	0.040	0.063	0.0001			
rs2048022	4	97.69	AAGTTGGCTAGAACCAAAGAACTTATGTCACCAGGAGTTGCTACTAACTCTTC[A/G]GCATACAGATCTGGGAAGCAAATAAAGAGCACTAATGCTGTAGTTTCCTTAAAAATGAA	A	G	0.60	0.71	0.69	0.56	0.63	0.58	0.46	0.48	0.69	0.66	0.71	0.003	0.026	0.030	0.005	0.0001			
rs1922858	4	98.21	TGTCCTTGTGCAGATTTACATTAGGTTCTGTCCCTTTCAGTTTGTTGGAATAA[C/G]TGCACTTAGGAGGATCTAGGTGGATGTGAACCATAACTTCAAAGATAAAAGCAGATTTT	C	G	0.07	0.45	0.49	0.49	0.39	0.64	0.31	0.47	0.50	0.46	0.41	0.118	0.024	0.000	0.010	0.0032	1		
rs1350970	4	98.75	CCATCCAACTTTTTCTCATTTCATTTCAAAATTCACACACAGAAATCCAAAGAGCTA[A/C]ATGTTTTTCTGCTCTGTAAGAAAGTTGTTAAATTTGTGTATTTATCATTGTGTAATGA	A	C	0.74	0.37	0.30	0.32	0.34	0.41	0.27	0.19	0.46	0.21	0.26	0.109	0.000	0.018	0.008	0.0000	1		
rs732299	4	99.45	GGGAGACCAGAAGACAATGGAATATTATTAAA[A/G]CATGAAACCAGCTTATCATTTTATACCCAGAGATATCATTCAGAAATGAAGGTGAAGTAAGGATGTTTCTTTATCATGA	A	G	0.52	0.36	0.37	0.35	0.27	0.18	0.04	0.31	0.25	0.34	0.46	0.010	0.103	0.012	0.032	0.0047	1		1
rs501110	4	99.64	CACCTCGATTCAGCCATCTTTCTCCAACAGGTTGTACTGCTGACTGCACAGT[C/G]TCCCCTGAAGGACTCCGGTCGCTGTAGCCTTGGAGAGCGACCAGATTCCTCACCCCA	C	G	0.79	0.36	0.30	0.38	0.48	0.55	0.56	0.48	0.46	0.41	0.33	0.088	0.005	0.000	0.006	0.0002			
rs1528545	4	100.02	CTGATCCTTTTTCATTTTCTGAGCTATTATAAGTGCTTTCTTTTCCTTTAACT[A/G]TCATAAATGGTTTTTCTTCCCTTAACTAAGTAAAGATTTCTGGAACAATGTTTTGACCAGA	A	G	0.55	0.48	0.52	0.54	0.48	0.32	0.67	0.61	0.54	0.46	0.48	0.001	0.012	0.007	0.017	0.0023	1		
rs1037475	4	100.41	TTGGATTACAGAGAGAAGGTGTTATGTAATGTCTTAAACTGGCTCCACCAAACTTTGTGACC[A/G]TGGGTTATTCTAAGTCTCACCTCCAAGATGCTGGAATCATAGGTTTGTGT	A	G	0.71	0.40	0.42	0.43	0.43	0.91	0.73	0.23	0.54	0.44	0.44	0.051	0.073	0.023	0.169	0.0012			
rs980972	4	100.74	GCCCATATTCTTTCCACTAAAAAAAAAAGGAGTGGGGCTGGAAAGAGTTGAGTA[A/C]GACTTTCGGCTATCTACCCTGAATGATAGCTCCACACTGCACAAGGCTTGAGGAAA	A	C	0.14	0.50	0.40	0.46	0.54	0.05	0.26	0.39	0.57	0.25	0.36	0.055	0.017	0.000	0.112	0.0008	1		
rs749407	4	100.83	CACTTTCTGATGTCCTGTGTTTTTCTGGATACATTTGGTACTCCTATACACAC[A/G]CTTTCTTCACTTCTCCTTGTTGACCCTGCCTGGAGAGGAGAGATCCTATAATATTCCTTT	A	G	0.19	0.38	0.41	0.41	0.30	0.23	0.48	0.27	0.32	0.36	0.41	0.022	0.005	0.011	0.015	0.0000			
rs1491233	4	100.93	ATTAAAAATTATTGGATAAATGTCATCTGAGCCTGGTGATACATCTTTTATGCA[A/G]TCAATGTGGCTTACAAAATCATGACTGCCAGCTATTGTTCAATTTTGTACCTGTGACCTG	A	G	0.86	0.41	0.50	0.38	0.46	0.73	0.09	0.66	0.39	0.53	0.54	0.052	0.115	0.012	0.017	0.0001			
rs1057613	4	100.96	TGAAAGGCATTATTAAAAGGTAAATTTCTCATCAAATTATAAGGGATTACAAAC[A/G]TAATGTAACAAAGCAAGTCATCAAAGCATGATTGGATGAATTCAGGAAAGGAGACAAT	A	G	0.86	0.41	0.50	0.38	0.46	0.73	0.09	0.66	0.39	0.53	0.54	0.052	0.115	0.012	0.017	0.0001			
rs716556	4	101.14	GAAGAAGCCATAATATATATACTAGCCAG[A/G]TTAAGTTCAGAGATAGACTCCTACAGCCTGGTGCCCAGAATGACATCGTGTGAACTGATAGCCTTGAAAAGGGATTAAAGCAA	A	G	0.31	0.62	0.65	0.52	0.64	0.45	0.54	0.24	0.65	0.70	0.73	0.049	0.003	0.073	0.015	0.0039			
rs1961323	4	101.23	ATTGCTTGTCCTTGCAGCTTTACAGCCTAGCCTGGCTCATGGCACATCTTACAT[A/G]TTCATTGCAGTGCTACATCTCCTCAGAAATAAAAATCCTGAAAATTGACTGAGAAAGTT	A	G	0.38	0.59	0.60	0.50	0.52	0.68	0.85	0.78	0.43	0.48	0.49	0.031	0.059	0.021	0.006	0.0069			
rs1365492	4	101.25	GATGGAATACAGGCATCATTAGAGGGGAAAGGTGATTC[A/G]GAGCACATTCTTGACTGTTACTCAGCCCCTCCTCAGCAAAATCTAAAAGAGCTATTTCAC	A	G	0.62	0.42	0.38	0.50	0.45	0.32	0.15	0.21	0.54	0.55	0.53	0.031	0.063	0.024	0.008	0.0132		1	1
rs981272	4	102.11	ACTGCCCTGTCACTTTTTGCTCCCAGACAAGCTTCTTGAAAGCTAAGCCTACCCTCACCATTCGTCCCTTAGCTCTGC[A/G]ACATAGCCTCATCTGCCATTTTCTCCTCAATTAAG	A	G	0.38	0.50	0.54	0.56	0.45	0.23	0.02	0.43	0.39	0.50	0.52	0.011	0.211	0.010	0.065	0.0004	1		
rs713455	4	102.61	CTTTTTTCGTTTTTTTGACAGCTGTTGAAAGGATCTTTATAGAGACTCCTTCA[C/G]GTAGCAAAACTTTTTTATGTATCAGTAGTTGCAGCCTCTGACTTAGACCATTTGTGTTA	C	G	0.33	0.28	0.24	0.20	0.29	0.77	0.75	0.56	0.25	0.24	0.28	0.007	0.125	0.057	0.159	0.0031	1		
rs871061	4	103.14	AAATTATCCTTGAATCTACTGTGTTGCTGAGG[A/C]CTTATCTATTATTTTGAACTGAGTTAGCACCCTCCCAACAAATAAAGTATGGCAAGCACAATGAAACTCCATCATCAAAC	A	C	0.43	0.38	0.44	0.39	0.43	0.59	0.43	0.31	0.46	0.37	0.41	0.001	0.000	0.015	0.010	0.0044	1		1
rs1027012	4	103.36	CTTGTGCACTTCAGGCTTTCATATTATCCTCTCAGTACTATTCTTTGCTATTAT[A/G]CCCTTGCCAAAGCATTAATGCTATCCAATATGTTATTTGGATTACTTTTTGACAAATTAAT	A	G	0.21	0.42	0.42	0.33	0.46	0.45	0.46	0.61	0.36	0.59	0.41	0.058	0.000	0.012	0.002	0.0016			
rs238510	4	103.49	TGGCTTCAGGTTTCCTTAAATTACAGGCAGTTCCTCAAAATGGCACTTCAGAGA[A/C]AGAGAGAGAGAGAAAACTCCAAGGTAATTAATCTACCAAGAGCAATTTAAAAAAATGGT	A	C	0.40	0.21	0.19	0.15	0.14	0.41	0.30	0.19	0.04	0.21	0.21	0.018	0.000	0.007	0.012	0.0001			
rs230490	4	103.85	CTAATTAAATCTTCATGTCTTACAGGGCTTGAGTTGGGGTCTTCTCAGACCTA[A/G]GCTGTCAACTTCAACTTCTTTGAACTTCTCATCCCACAGAATATGTAAAATTAATGGTCC	A	G	0.17	0.41	0.43	0.48	0.30	0.59	0.64	0.40	0.54	0.34	0.41	0.040	0.026	0.002	0.014	0.0000	1		
rs747559	4	103.87	CAGGGACAGAAGAAAAGAAAATGTCTCTGAAGGGAAGAGAGCCAGAATTGTGATT[A/G]CATTAAACCTAAAGATTTTCAACGATGTGACCTGATGAAATTCCATAATGGTTAAG	A	G	0.43	0.63	0.61	0.51	0.59	0.55	0.16	0.77	0.50	0.66	0.67	0.012	0.084	0.024	0.000	0.0028			
rs65671	4	104.18	GTCTAAAAATTAACCCAGGAAAGCAAAATCAGAATTTTCCTAGAGTGGAAACCTCAG[A/G]ATTCAAAGCTCTATTAAGTTTAGGTATATCATACCATTCTTGGTAAAAAGGCCGC	A	G	0.79	0.42	0.49	0.54	0.41	0.59	0.81	0.42	0.46	0.52	0.56	0.042	0.054	0.000	0.007	0.0008			
rs223383	4	104.21	ATTGGTTGATTGGGCTTTCTGTTTACAAGTTTGTATTTGCATAAGTGGACTTAC[A/G]TTTCTGCTTATATTTTGCCATGGTTAAAATCAAAGAAACATAGCCTAGAAGCATTTTG	A	G	0.31	0.58	0.52	0.45	0.59	0.41	0.19	0.58	0.50	0.48	0.44	0.017	0.054	0.000	0.007	0.0008			
rs223334	4	104.25	CTCTAAAGGAAATCCCATCAACCTTTTAAACATCACTTTGTATACACCATCTTCT[A/G]TACTGCCCTAGCTCAAAACATGAACCTGTATCTAAACGTGTTTGTAGAATTTCCATT	A	G	0.28	0.58	0.52	0.45	0.59	0.41	0.19	0.58	0.54	0.48	0.44	0.025	0.054	0.000	0.007	0.0007			
rs1031804	4	104.52	TAAAGGGACCGGAAATAAAAGAGGAATCAAAAACTGCTTATATTCAACTGAGTACCA[A/G]TAAACTGTTAGTAGACAGAATATTTCAGAGAAGACAGCTCAAGTAGCAAATATTC	A	G	0.64	0.41	0.37	0.40	0.41	0.36	0.27	0.34	0.43	0.45	0.45	0.035	0.007	0.000	0.000	0.0024	1		
rs1384401	4	105.02	TTGTACTTGCAGAATGAGTAGTATTAACAGATAACCCATAGAGAACCTTTTTCA[A/G]ATGATTGCCAAACACTGAAAGGCTTTAGATATTTCCTCTAATGTTTATCTTTTCTTATT	A	G	0.62	0.34	0.35	0.26	0.36	0.32	0.16	0.33	0.11	0.32	0.35	0.046	0.013	0.000	0.000	0.0003	1		
rs1528381	4	105.60	TTATACTAGATTAACAGGCCAACTCAAAAATTCGAATTTTTCATAGTTTTATCTTTGTTTTCATTTGCAAAAAACTATGGTTCCTTTGAGCATCCTATGAG[A/C]ATTTCCTTTTCATT	A	C	0.38	0.37	0.43	0.33	0.46	0.82	0.84	0.53	0.64	0.33	0.37	0.001	0.084	0.003	0.080	0.0040	1		1
rs960345	4	105.69	TATAAACAATAAAACTTGACAACTAGCCTAGAAGATTTTTGTAAAACATTA[A/C]ATTTTGTAAGAAGTTAGAAAAATAATGGATAATGGCCAGATGTAAAGAGGAATGCAAATGT	A	C	0.71	0.55	0.49	0.56	0.39	0.18	0.14	0.36	0.36	0.54	0.52	0.022	0.073	0.007	0.058	0.0007			
rs1024461	4	106.12	CATGATAATGAGTGCCATAAAAAAATAAACCCAGAAGGAGCTAGAAATTGGCTGGG[A/G]TTGGGGTGGGTAGTTTCTCTTTATATTGAAATAGTAAAAGAAGGAACCTCTGATA	A	G	0.05	0.21	0.19	0.26	0.07	0.09	0.38	0.25	0.18	0.18	0.19	0.047	0.007	0.003	0.023	0.0001	1		
rs1548484	4	106.21	TTTTTTGTTTTTTTGTTTTTTTTTTTGGTCGAGATGCGTCTTGCTACATTTCCAA[A/G]CTGGCTAGAGAAACTTGGCCCAGGTTTAGCGAGTTGCGGAGTATATAATGTAAAAAC	A	G	0.52	0.31	0.33	0.23	0.38	0.73	0.68	0.44	0.43	0.32	0.44	0.015	0.064	0.013	0.084	0.0036			
rs1357249	4	107.96	CACCTGGATGTTTGCTGAAGGGATGACAGAGGTTTAGTCTTGTGGGTCAAAT[A/G]CAGGATGGTATTTTGGAAGTGCATGCTAGGAAAGTGCAAGGATCCGGTTCCAAAACTCA	A	G	0.26	0.32	0.34	0.28	0.25	0.36	0.59	0.36	0.39	0.35	0.27	0.000	0.060	0.013	0.008	0.0012	1		
rs447372	4	108.31	AAATCTGGGATTTATAGTAAAACATTTTCATTGTTGCCTTATAAATTTTCCTGAT[A/G]TCATGACCTAATTTTACTTCAGAGAAGTAACATGGTAGGGCAAAATTTGGAGTTATACT	A	G	0.64	0.37	0.43	0.45	0.43	0.36	0.02	0.32	0.68	0.36	0.44	0.019	0.145	0.001	0.004	0.0000			
rs1393589	4	108.70	TGGAATGGACTCAGTGATTACAAAGGAAGAGTTTTGCAAAGGAGAACCAGATAAAAA[A/G]CAGTCAGAAAAGATAATAAAAGAAAACCTACAGGAAAATCATAAAATAAAT	A	G	0.33	0.55	0.59	0.50	0.48	0.27	0.21	0.69	0.57	0.60	0.58	0.022	0.059	0.023	0.034	0.0000	1		
rs991013	4	108.77	GGCAAGGCTCATTTATTGACAAAGAAATACAGAGTCTCCTGTATTCTCTCACCTCA[A/G]ATGCCTCCCAACTTTAGGCCAATCCTACAGGCAAAGGAGGTGGGAGCAAATGAGT	A	G	0.60	0.81	0.78	0.88	0.70	0.73	0.60	0.86	0.82	0.76	0.82	0.005	0.003	0.043	0.002	0.0068		1	1
rs970919	4	109.04	TTTGAATAACAAAGTATTGGACACATGTATGAAAAGCCCTCCATCAGTGCCA[A/G]TTTTGCATGGTTGTAGAGGTTACCTGACAGATTTGGCTTAAAACAAATTAAAGTGATTAA	A	G	0.64	0.59	0.55	0.63	0.52	0.39	0.36	0.47	0.54	0.68	0.66	0.001	0.035	0.019	0.025	0.0039			
rs995387	4	109.91	AGCAAAAGCCTTGAGTCATCTTTGAATCATCCCTATCACATTCAACAT[C/G]CAATCCATCAGCCAATCCTTCTTGCCTTTACTTTTAATATCTATTTAGAACCTGTCCACTTCTTAT	C	G	0.55	0.58	0.62	0.61	0.55	0.73	0.45	0.42	0.61	0.53	0.61	0.008	0.029	0.020	0.002	0.0005	1		
rs1865845	4	109.99	TGGTTATTCCTCAATTAATCAAATAGCTGGTGTATCCCTAAAGTAGTATAAAGTGAC[A/G]CACTGTGTGAAATCAATTTTTCTTTCCAAAATCATTAAATCCAGTAGCCTGTGACTA	A	G	0.19	0.38	0.29	0.30	0.21	0.45	0.73	0.39	0.39	0.30	0.32	0.011	0.083	0.003	0.009	0.0008			
rs1812590	4	110.19	AAATATTTTGAAATGAAAAAAGTCATTAAAGTGTTTATTAGTACGGGGTTAGCA[A/G]AAACCAAACTCCACTAAAAATTTAAGTTAGAGGAGTGTCTCTGCTGTGTCAATTTTATA	A	G	0.71	0.39	0.42	0.39	0.54	0.41	0.32	0.43	0.21	0.45	0.39	0.031	0.018	0.001	0.004	0.0026			
rs965935	4	110.24	TTTAAAAAAAGACACTGAACATATGGGTATTGCTTTCTTTATAATTGAGTT[A/C]ATTGAAAGTGGAAAATTCCATTAGCTGGATGGGAACAGAGGGAAAGTAGACAGGGGGAG	A	C	0.69	0.35	0.46	0.43	0.48	0.05	0.20	0.32	0.21	0.48	0.45	0.030	0.033	0.006	0.118	0.0000			
rs1541373	4	111.09	GCTATTTTTGGGGTAGGACAACATGCTCTCCCCAGGAAACACCATTATCTAGAGACCCTCATCATTCTGTGAATTTCTGCATG[A/G]TTCTGTCCCTGAATTAGCTCTACCTATGA	A	G	0.81	0.65	0.59	0.59	0.57	0.36	0.71	0.80	0.68	0.53	0.60	0.018	0.002	0.034	0.035	0.0006	1		
rs721412	4	111.41	AAATGAACCTTTCCTACTCAACTTTAATAGTATCTGAAGCAGTGGTTTAAGTGGCT[A/G]CACTTATGAAACTTATGAAGTTCCATAATTGTCAGTTTTGATTTTTTTTTCCACTATGC	A	G	0.40	0.73	0.65	0.55	0.63	0.55	0.48	0.56	0.57	0.64	0.73	0.024	0.010	0.000	0.002	0.0048			1
rs2035415	4	111.54	ACCATTCGATCTTACAGTTAGTTCTCCCATTGCTCT[A/G]TAAGTATCTTATGACCCTGGCAAATTAAACCACTGGAATATCTCTCTTCTTTCTCTATTTCTGAAACTTAGAAAGCC	A	G	0.83	0.53	0.48	0.51	0.46	0.36	0.47	0.69	0.39	0.50	0.53	0.083	0.001	0.046	0.003	0.0000			
rs244059	4	111.67	AGGAACTGATGGCCACGTCCTTGGGCTCATATGCAAGGGGCAAGGATATCCAG[A/T]GAGGACGAGCCCACTTTCAAGGTGCTGGCCGACCCCAGGTACCAGCTCCTGAGCA	A	T	1.00	0.77	0.71	0.72	0.79	0.77	0.62	0.73	0.79	0.84	0.77	0.117	0.006	0.003	0.003	0.0017			
rs243985	4	111.73	GTAAATATTTGGTAAATATTTTTTAGACAACACAATTTAAAGCATCTAAAGTTAT[A/C]TAATGGCATCATATTCTAACTACTAAAAACAAGCAAAAAAACACTCCTGGAAAGCTTA	A	C	0.40	0.45	0.48	0.44	0.45	0.18	0.38	0.32	0.46	0.45	0.55	0.000	0.000	0.008	0.036	0.0030			
rs1879053	4	111.86	AAGTGTTTTCTTTTATTTTTTTCCCTCATATTATGTAAACATTCCAATTCTGTCCTTTG[A/G]AGAAGTCATATCCTGGAATTGGGTGCTCAGGGTGATTCAAGGACCACCTAGGT	A	G	0.43	0.38	0.48	0.48	0.41	0.23	0.19	0.45	0.42	0.46	0.46	0.002	0.060	0.001	0.044	0.0001			
rs2046895	4	112.57	CTTCGTCTTTGCTATTTTTCCTTGCATCTCAGTATTTCCTTTGAAGTCATTTT[A/T]CTTTTCCTAAAAAATACATTTCAAGCATCCTTTTAGAATATTTGGATAGTGATAAGCTCTTC	A	T	0.29	0.57	0.56	0.65	0.63	0.41	0.45	0.63	0.64	0.52	0.55	0.031	0.000	0.011	0.003	0.0001	1		
rs1450782	4	112.97	CTTTATTAACGTGCTAATAAGTCTTATGATC[A/C]TTAAAATGGATTGTGGGCTCAAACTGTCTCTGTTTATACTTGAACTCTCCTTGACAACCACAGATACAAACTTATAAGGCTC	A	C	0.26	0.42	0.47	0.30	0.43	0.23	0.38	0.33	0.36	0.38	0.32	0.016	0.012	0.006	0.034	0.0111		1	1
rs722052	4	113.30	ACTAAGGCAAAAAGGCAGAAATATATTTGAGATATCTGTACATCAGTTAAATTTTT[A/G]TATGAATGAAGGATGTGAGTCAGGGAACCATAAGAAATACCATCACTGAGGTTGTG	A	G	0.36	0.71	0.67	0.60	0.61	0.36	0.50	0.68	0.64	0.64	0.76	0.064	0.012	0.001	0.058	0.0050	1		
rs701760	4	113.90	GTGTCTCTCTTTTCTTCTTTCATTTTCATTTCTGCACTTCTAACACATGAAATTA[C/G]CTGTTCTAAAATAGTCGAGGAGGAAACTGGCAAATGATGCTTTTATATTTTGGGAATTT	C	G	0.45	0.49	0.49	0.46	0.45	0.50	0.72	0.54	0.57	0.50	0.41	0.000	0.047	0.011	0.002	0.0031	1		
rs967099	4	114.58	TTTATTTGGAAAAATACAGTGCTAATCAGAGCCAAGTCTGCTGACCAAGCCTTGA[A/G]TGTGATAATATGGCTGTGGAGATGTAGAGAGAGAAGTCAGGCACTCTGGTCTCTA	A	G	0.33	0.43	0.40	0.33	0.52	0.59	0.77	0.43	0.25	0.38	0.51	0.003	0.086	0.005	0.018	0.0029	1		
rs29327	4	114.67	TTAGGCTGAGAAAGCTAAGCCATGTTCTTCAAAATTCATGATCTCCAGCC[A/G]TGGTGCTACTTCTGGAAATCAAAAAGGACACTGACCTTATTTCAAAAATG	A	G	0.76	0.42	0.48	0.31	0.46	0.59	0.24	0.30	0.39	0.46	0.53	0.037	0.040	0.029	0.008	0.0004			
rs766184	4	115.32	GCTGGCTCCTCTTAATTAAAGTTGTCTAAGCAAGTTTCAGCCCACAGTGATGTA[C/G]CTTGGCCAAAAGGAGGTTAGTGGCTTCCATATAAACCTTGACACTGATTGGGTTTGA	C	G	0.74	0.54	0.41	0.48	0.55	0.59	0.46	0.50	0.64	0.45	0.49	0.056	0.000	0.001	0.018	0.0010	1		
rs1551740	4	115.84	TACTCTGTTCCAGAAAATTGACTCAACAGTTGTTCAACACTTTCCTATAACCA[A/G]TAAAAGGTATACTCCATGAATTTGCGAAGCTGACCTATTGGGGGGTGGGGTGGGAACA	A	G	0.90	0.46	0.44	0.34	0.48	0.55	0.44	0.40	0.54	0.46	0.34	0.157	0.004	0.000	0.013	0.0050	1		1
rs1380931	4	115.89	ATTTTGTAATGAGCACCTTTTTACAATTTATTGTAGGTATTGTTTTTCTTAAT[A/G]GTTTTGTCTTTTTAACCCTGGAACTAGTAATAAAATTGAGTTACACACAATCGTTAAAGTCCT	A	G	0.88	0.47	0.50	0.46	0.52	0.41	0.49	0.35	0.50	0.45	0.42	0.115	0.006	0.003	0.000	0.0025			
rs1948983	4	116.76	GAGAGGCAGCAATGATCACGTCTTTAAATAGACAGGAAAAATATGTTTTTGAGATCA[C/G]CTTCCTGTGGAGAAAACAGTTATTATGAGGGAGTGTCAATGTAAAATTACAATTCA	C	G	0.38	0.63	0.61	0.68	0.64	0.77	0.41	0.48	0.61	0.60	0.61	0.016	0.009	0.010	0.023	0.0000	1		
rs1430934	4	117.26	CAGAGTAATAAGTCTGCCACATCTCCCCTGTGCTTCCATATCACTGAGATCTA[A/G]GCCATCAATGTCTACTTTGGCAAGTCTGATTTTATTTTCCTGCTTAGGGCCATATTATTG	A	G	0.43	0.52	0.46	0.52	0.54	0.64	0.33	0.44	0.50	0.46	0.52	0.001	0.008	0.000	0.013	0.0024	1		
rs814397	4	117.46	CTTATCAGTTGGACTTACTGGGCACCCAACACCAAAAAGGCAGATAAAGATG[A/G]TATGAGGTGTAGACATTCCACTTCTCCCTCCACTGGTGTCAGAGAGGCACAGTTGGGAC	A	G	0.14	0.38	0.35	0.41	0.34	0.45	0.08	0.20	0.27	0.34	0.35	0.020	0.032	0.009	0.013	0.0000			
rs1459531	4	119.20	ACATTTCACTTAAAAAAAGATCATTCATCTGTTCTCCATTGCTCTGGGA[A/G]AGTAAGTTTAACTTGTAGTGCTTGCTTTAGCTAACTAGAAATTCAGGAAAACCATAAATAAAT	A	G	0.12	0.60	0.61	0.59	0.39	0.64	0.39	0.60	0.75	0.59	0.52	0.149	0.032	0.007	0.000	0.0039	1		
rs972813	4	119.70	ATGTAAGGATTCTGGATTAGATACCAAAGATTGGTCTTTTATTTTTTTTTGTCCCTAGT[A/G]AAAGCATTATTATTAATCCCCACTTATT	A	G	0.79	0.72	0.73	0.66	0.63	0.36	0.48	0.61	0.64	0.73	0.73	0.001	0.038	0.006	0.083	0.0001	1		
rs298991	4	120.10	CAAGTGGGGTTAACTGAGCCACACATGCACAACAATGCCTCTCCACCTGCCTCCT[A/G]TGTAATTAAGAGGAGCGAGGGTATTTCTCACTTCTTCCTCTCAGGAGGTAAACCC	A	G	0.52	0.73	0.74	0.71	0.64	0.59	0.83	0.53	0.82	0.66	0.68	0.018	0.006	0.016	0.012	0.0010			
rs1459062	4	120.51	AAAATCTCTTTTCTTTTGAACTTTGCTCTTCC[A/G]TTTCTGTTCCAGTGTCTTTTCTTTTCTTATTCATTCATTCATTTATTCAACAAACTTACTAGGCGCTGAATGTTTTACTCTC	A	G	0.33	0.54	0.50	0.44	0.50	0.50	0.57	0.39	0.50	0.45	0.47	0.010	0.011	0.001	0.001	0.0004	1		
rs843571	4	122.10	ACAGTTACACAATCAGAGAGAGCAGAACAAAAAATAAGGCACTTGTAATCCCA[C/G]GCCTGTGTCTTGGGTGACTCTGCTTCCTACCTGAGCCTCTTCCCTAGTCTATATCACT	C	G	0.48	0.59	0.53	0.41	0.38	0.77	0.88	0.49	0.50	0.48	0.38	0.000	0.122	0.000	0.048	0.0052	1		1
rs1398908	4	122.73	CACTAGGGAACTTGTTTTTGCTGCGAATATACACACAAACAGTTCTTACAAAGT[A/G]ATTGAAGGAAGCACTCTGAAGAGAACTACAGAATTCATATGAGCTGTGGCTCCCACA	A	G	0.93	0.75	0.71	0.78	0.77	0.77	0.93	0.91	0.75	0.80	0.78	0.052	0.042	0.044	0.005	0.0049	1		1
rs1513703	4	122.80	CCATTCACTGAACAAAAACCTAGACAACCTTTGGTGAAAGTCTCTTCTACTCTTA[C/G]GTCCATTTAAATTTTTTCCTATTACCTCTAAGAAAACCTTAACCTTAGGGGGCATCAA	C	G	0.64	0.61	0.60	0.63	0.63	0.73	0.98	0.90	0.61	0.60	0.60	0.001	0.146	0.092	0.017	0.0002			
rs951974	4	123.29	TAGATCCTATTT[A/C]AGGTATTTGCTTTGCGTAGTTGGCAATGAATAGAAAGCTCCATTTTTAATCTCTTCTCTCTTTTTTCTAGCTAGAGATAAATGGCTCCCTTCTGACCCTC	A	C	0.88	0.70	0.64	0.63	0.70	0.68	0.40	0.70	0.65	0.68	0.74	0.036	0.026	0.000	0.000	0.0074	1	1	1
rs1880863	4	123.34	AAAGCTTTGAATCTTGGCTTAAAGGTGATTTATTCTGCAAAAGGAGCTCTGCCCA[A/G]TGGACTTTTCTGGCACCCTCTGAAGGCTGACACTTTCTGTAGAGCCCAGGAAAAATA	A	G	0.57	0.53	0.48	0.50	0.38	0.14	0.01	0.35	0.50	0.45	0.47	0.004	0.163	0.001	0.062	0.0000			
rs377070	4	124.09	CAGGGGCAGTGATGTATCTGGCCTTTGGGTACGTGGAGCTAAGATCAAATTCT[C/G]CTAGTGTTTTCCCCTTTCCTCTCTTGCCCCTGCTTCTCTCAGAACACTTATGCTCTCC	C	G	0.07	0.42	0.44	0.38	0.38	0.14	0.10	0.08	0.57	0.30	0.36	0.092	0.068	0.078	0.050	0.0028	1		
rs2048266	4	125.97	AAGCTGCATTGAAGGTGAAATTATAATAAACA[A/C]ATAAAAGAAGGGGCTTTTTTTGCCTGTTTTGTTTTGTCAATAAGATCTAATGTCAAATAGAACTAATCCAGTTTAGAA	A	C	0.90	0.67	0.69	0.68	0.77	0.59	0.46	0.39	0.82	0.73	0.74	0.040	0.043	0.049	0.010	0.0011	1		
rs1495127	4	126.05	ATACACTGCAGCTATATTTATCCCACTTTGCTTAATGATTTTTCTTCCTCTAACAAA[A/G]TTATATATACTCTTTCCTTTTTGCTGCTTCTGTCTTTGTAAATATATTTTAGGGATATA	A	G	0.26	0.20	0.21	0.21	0.13	0.32	0.32	0.25	0.14	0.21	0.24	0.000	0.010	0.002	0.007	0.0000			
rs1498040	4	126.40	CAGTGAAGTGTATAGAAGGGGTCAAAGTTGTTCTATTAAAAATTGAGAGATAT[A/G]TGAGGACATGGGAGCTGAGTCCCCATTAACAACATATACACTATCAACCTGACCTAGT	A	G	0.26	0.53	0.57	0.55	0.61	0.41	0.17	0.46	0.46	0.58	0.63	0.066	0.110	0.026	0.024	0.0018			
rs1320244	4	127.69	AAATGTCCAGAATAACTGGAGTTAGGGAGATCTGCAACTATAAGATTGCTATAA[A/C]TAACGATGTTCAATGGCAGAGCAGAGTAGGAGATAAGGGCTCTATATATGTTTTGTGTA	A	C	0.33	0.40	0.44	0.55	0.43	0.73	0.47	0.44	0.36	0.41	0.53	0.006	0.003	0.000	0.048	0.0061	1		1
rs1157308	4	128.14	CACTTGTGGGCCTACAGTGAATAGATACAAATTTTTCTTAGCTTTATGTAACAC[A/T]GGGCAATTTTCAAAATGTTTCTCACTATTTTATTTGGTGCTCACAACAACTTTGTAATTA	A	T	0.40	0.48	0.52	0.44	0.54	0.00	0.07	0.35	0.43	0.44	0.41	0.011	0.122	0.022	0.230	0.0058			
rs724950	4	128.80	ATGTCAACCTCCTTTAAACTTTCCTTTTCCTTCAGAATAACTATCTGTGCAAATATC[A/G]TTGAAGTGTATCCCAAGCTCTATTGTGTTGGTTTAAATTGCTCATATGCTGAAAAAA	A	G	0.33	0.52	0.52	0.68	0.54	0.77	0.20	0.59	0.57	0.49	0.53	0.018	0.053	0.006	0.038	0.0001	1		
rs1514298	4	129.00	CATCATCCTTGACTTTCCAGC[A/G]GAAATATTAAGTTCCCTTTGCTTCCGTGATTTAAGTCCTTTCTTTCTCTGGCCACTTCTGTGTGTCCTTTCCAGGCATATATTCTTCTATCCT	A	G	0.33	0.52	0.47	0.48	0.55	0.41	0.27	0.70	0.50	0.60	0.56	0.011	0.024	0.020	0.003	0.0036			
rs1982904	4	129.74	ATTTGGATACGACATCTCTGGGGGGCCATTATTCTGCCCACCACACTCAGTAACTA[A/C]TTGGAAACCTCTATCCAGCTCTTAAGCCTTAAATGCCACCAGGAAAACATAGAGC	A	C	0.67	0.17	0.22	0.23	0.27	0.23	0.33	0.28	0.25	0.23	0.25	0.090	0.008	0.001	0.000	0.0001	1		
rs291079	4	129.76	ATTCGCTCCGTCATTATCCCCTAACTTTGGCACCAATCCCTTCAGCCCTATTCA[A/G]TACAAACGCATCGTGACTCCACTTCCAGGTTACCTTCTCCATAAAGTCCCCTATCCTG	A	G	0.60	0.37	0.31	0.37	0.34	0.59	0.54	0.44	0.46	0.38	0.38	0.030	0.011	0.000	0.024	0.0003			
rs1106860	4	129.81	CTGCACTCTTAATTTAGGCACATATTTCCTGAAAACTTATATTTACATTTGCCAA[A/T]ACAAAGCAGTAATTTCCCCCCAAACCATGCTATTGCTAGAACCATGAACTTCTAAACT	A	T	0.69	0.64	0.70	0.66	0.66	0.73	0.46	0.57	0.50	0.61	0.61	0.000	0.021	0.002	0.002	0.0020			
rs318539	4	130.29	TAATACATCACAATAATGCACACAGAGAAGCAGGATGCTGAGCCCTAACCTGAAAA[C/G]GCTCACTTTTGAAAGCGAGTAATAACACTTTTGATATGAATGCTAGAATATATAAT	C	G	0.87	0.70	0.69	0.65	0.66	0.77	0.75	0.59	0.68	0.72	0.75	0.028	0.006	0.000	0.009	0.0026	1		
rs1396758	4	131.66	TATACATAGTGAAACAATATGTTCTTCCGAAATGGAGCACTTAGTTATCTTTAT[C/G]TGCAGAGTGAAAGACAAATTAAGGGAAGAATGCTGTGCAAGCGGTTTTATTGCTTTT	C	G	0.14	0.48	0.57	0.45	0.50	0.05	0.03	0.30	0.39	0.55	0.55	0.096	0.188	0.052	0.179	0.0001	1		
rs2055392	4	132.33	CATTTCTTCATACATATGCTTTTTCAGAGAGCTCATTCTATTTTTCTGTAGCAAGCA[A/C]CTGATCGTTCAGTAAATTTAATCCTAACGACCAATAAGGAAAATCAGTATTTACCAT	A	C	0.29	0.39	0.39	0.40	0.54	0.00	0.07	0.26	0.46	0.36	0.35	0.010	0.090	0.026	0.189	0.0013	1		
rs645076	4	132.56	ACTTTAGCTGCCAGTACAGATTATATCTTTCAAGAGATCCAAGTGTTTGAAAGTAC[A/G]GGAAAAACTAATTATAAAGTGCTAAGCATATGCACACTCACAGGAAACATTTTAGAA	A	G	0.40	0.32	0.34	0.28	0.48	0.18	0.03	0.22	0.39	0.32	0.30	0.001	0.093	0.010	0.021	0.0010			
rs349867	4	133.29	AGAACTCTGCATTTTCATTTTGAGAGGAGAAGTCGGTGTGTTTTATAATGGGA[C/G]GAAGATTAAAGCAGATACTTTGGCATCAGAAGGACTGACCTTGACAGAGACTGGTGAC	C	G	0.26	0.39	0.35	0.40	0.34	0.14	0.14	0.24	0.29	0.41	0.33	0.006	0.028	0.012	0.029	0.0002	1		
rs1499328	4	133.62	ATTTAGAAAGTAAGAACACAGGCACATTACAGATACAGAGGTATCTTGATAATGGGA[A/G]TACAAATAAACCTTATCATCTAAACTGTGGAAATAAAGACCTACTATTTTAAATGC	A	G	0.76	0.52	0.45	0.48	0.46	0.45	0.58	0.53	0.64	0.47	0.51	0.052	0.013	0.001	0.000	0.0011			
rs1425566	4	134.74	TCAAGGGGGTCTCAACCTCTTTTTATTAGCCAGCATGCTGACATGGTTTGGTA[A/C]CTTCAATTAGCTTTGTTCAATTATATGAACTTGTGATTAGCAGAGGCTAACTGTCAGCC	A	C	0.24	0.54	0.56	0.57	0.54	0.45	0.59	0.59	0.54	0.58	0.55	0.040	0.010	0.013	0.000	0.0005	1		
rs1389331	4	134.79	ACTAACACATAGAAAGATGCCTTAGAAAAGGGTTAGCTGAACAATCTCGAGAGTC[A/G]AAACATCTCCTAACACATCAAATATCTAGTAAAATCCTGAGAATCGTATTGCCTTAGT	A	G	0.14	0.51	0.48	0.52	0.45	0.45	0.95	0.58	0.57	0.50	0.43	0.059	0.181	0.019	0.000	0.0002			
rs906372	4	134.99	CAGCCTTAGTCTTGATTCTCTACAGCTTCTTATTGCCATTGTGAGATTCAGCGAT[A/G]TTCATCTTCTCTGGTATGTCTAGATTCTCTGTGGAGAAGATAAACTCATAGGTCCT	A	G	0.17	0.30	0.35	0.29	0.43	0.23	0.25	0.52	0.21	0.44	0.36	0.022	0.009	0.029	0.011	0.0001			
rs1494996	4	135.46	CTCGATCAGTAAAGCTGGCAGTCAAGAAATGTTATTTATAAGGAGACATATAGGTTA[A/C]AGCTTATAACAAAATGTCAAAGGAAAGTGGAGGATAAACAGAGGAACCAATTTCA	A	C	0.98	0.78	0.74	0.76	0.84	1.00	1.00	0.79	0.79	0.73	0.78	0.062	0.096	0.000	0.096	0.0012	1		
rs1017507	4	135.60	ATTAATTAATATTTTATTATAACTTATTTATTTAGATATTTATGTCTGTTTCCCT[A/C]CTTTCACTAGGCTATATGTACCTGTGGGTAAGAATTGTGTCTAGCATCATTATTTTCCAGT	A	C	1.00	0.47	0.44	0.49	0.52	1.00	0.97	0.60	0.54	0.48	0.46	0.250	0.195	0.008	0.259	0.0001			
rs2055664	4	136.26	TTATTTTTTGTTCTTGCAGTCAATATTCAAAAGATAGTTACTTTGGAAAGGATTAC[A/G]ATTCACATTGGTCATAACTATAGACTGGTTTTGTTGAGGCTGTAAGTCTACCTTAAATC	A	G	0.48	0.51	0.52	0.59	0.48	0.55	0.40	0.43	0.58	0.45	0.42	0.002	0.019	0.002	0.001	0.0037	1		
rs1992695	4	138.27	TAACTTTTTCTAAACTTATACATTTTCTAAACTTTTTCTAGTTTCCAGTGCTATTCTTTTACCTGCATTATGGCCACTTGCTTATTTGAAGATTAATT[A/G]CTTATGGGCAACTTAA	A	G	0.50	0.52	0.56	0.57	0.64	0.50	0.45	0.65	0.39	0.49	0.55	0.000	0.000	0.019	0.000	0.0005	1		
rs1373895	4	138.31	CATTGGGTTGAATATGAGCCAGGTTCATATTGTAAGGATTTCATTTATTACCTC[A/G]CCCTATCAGCTCTGACTCTACCAATAGGCCCAGGATGATGCTTTGGGGCTCTAGGTA	A	G	0.33	0.41	0.40	0.38	0.25	0.50	0.41	0.44	0.43	0.45	0.37	0.003	0.001	0.002	0.006	0.0015			
rs1344976	4	139.64	CTCTTCATTTTCCTTCCGTTAGATAAACAGACCCAAAAGCAAATATCTTCTCT[A/G]TGCCTAAGAAAATGTTCAAAATAAGCAGCCAAGGCTTGGAGAAAGGGCGTCTTGTTTTGC	A	G	0.38	0.46	0.42	0.40	0.54	0.23	0.35	0.55	0.21	0.48	0.52	0.001	0.001	0.015	0.020	0.0035	1		
rs426029	4	139.86	CCCAGGTGGAAGTTCATTTTTCTATTCCAAATGTCCTTTGAACTTTAGCCTCAC[A/G]GCTTTTATGCGTTTTCTCACTATTACAGCAACTCACAAATGTGTATCATCCCAACTAGA	A	G	0.26	0.39	0.42	0.33	0.38	0.27	0.19	0.57	0.50	0.44	0.40	0.018	0.045	0.003	0.017	0.0003			
rs1365372	4	139.91	AATTGGACACGTCTTATCAGTAAGTAATGAATTTGTAGTGCCTTGATTCACAG[A/G]TATGTAAGGAAAATTAATGCAATGGGTAAAAGGTGAGGCTAAGGGAAATACAAAGTCT	A	G	0.98	0.86	0.88	0.88	0.80	0.68	0.50	0.72	0.85	0.92	0.94	0.022	0.089	0.020	0.024	0.0099		1	1
rs732823	4	140.16	GTCCAACTAGCTACCAGGAC[A/G]CTCGCCTCATTCCTCTCCTGCTCCATTGTCAGAAAACCCAAAAATCTCCTAATGCATTTCTTCTGTTTCTCTTCTGACTTATTATTGTCAATT	A	G	0.26	0.64	0.60	0.63	0.71	0.68	0.95	0.59	0.75	0.63	0.56	0.056	0.098	0.000	0.006	0.0017	1		
rs978752	4	143.62	TAAATACTTAAGGTCTCATAAACTCTTTGCTTAGGATATTCAGCTGATATTTATC[A/G]TGAATAAGAAACATGGGATACCAAACTTTAAGGATCTTTTTAAAAATAAAGCTGTCCC	A	G	0.26	0.22	0.24	0.28	0.16	0.05	0.11	0.25	0.25	0.17	0.20	0.000	0.026	0.004	0.067	0.0028	1		
rs1018092	4	145.12	CTTCTTCTGGTATTAGTGACACATTCTCTGTCTACAACCAAAGTGTCCAGCAGA[A/C]AATGGTGCTAAAGTCTTCTAGATGTGAATGAAAGAGATGAGATGGTAAACAGCCCAGT	A	C	0.14	0.46	0.50	0.43	0.50	0.00	0.46	0.45	0.32	0.43	0.44	0.059	0.000	0.002	0.202	0.0014	1		
rs1822842	4	145.36	ACTGGACCACTGTTTACACTATATTTTTAATTGCCTGTTTCATTTGTCAGTC[A/G]CCTCACCTTAACAGTTTCAAACACAGGGACTAACTCCTCCTGCTCACCATGGTGTTCATTC	A	G	0.52	0.52	0.49	0.48	0.39	0.64	0.40	0.34	0.43	0.37	0.45	0.000	0.003	0.012	0.010	0.0011			
rs2202507	4	145.84	GGAACTGTTTATGATAATTTATTTTAACTCATGCAGCTTCACAGCAGCGATTAAT[A/C]AAAACTTTAAAAACTAGTGTCGTCTAAAAGAAAAGTAAAAATAGAAAAAGACCTCAGA	A	C	0.83	0.49	0.51	0.54	0.43	0.45	0.37	0.45	0.54	0.64	0.56	0.055	0.010	0.004	0.003	0.0001	1		
rs987246	4	145.85	ACCTCTTCACCTCAGCTTAGTATCCAACAGGTCCAGTTCCACCCACATGG[A/G]ACCCACTACCCTTCTCTTATTCTGACTTGGTTGCATTTTTCCATTGCATGGCTAGACTATGA	A	G	0.55	0.73	0.74	0.66	0.80	0.73	0.78	0.85	0.75	0.71	0.75	0.012	0.004	0.024	0.001	0.0003			
rs1505762	4	145.92	GAACCTCCAGAAAAGTATGGAGTTCAGTTATGGACATCCCTTGTTAAAGCAAA[A/T]GCTGAGTATTTCAGGTGCAGTCTGGGATTCTAGTTCTTTCCAATTTGGCTACTGTTGCT	A	T	0.69	0.56	0.55	0.70	0.66	0.68	0.50	0.64	0.57	0.58	0.57	0.009	0.000	0.001	0.008	0.0001			
rs2035901	4	146.10	GTCTAAGAAAATGGAAGGATAGAATTGTCATTGATTAAGGAAATAGAGTAAAATAC[A/G]GTATTACAGATTTCAAACTCGTGGTGGAGAAGTACTTCTTAAATAAAACTCCAAGTG	A	G	0.29	0.53	0.54	0.52	0.39	0.23	0.21	0.19	0.57	0.47	0.53	0.036	0.090	0.080	0.060	0.0000			
rs1444086	4	146.89	CATACGTACAACTACACACTTCCCCTCTTTTTCCCTTCCTCCAACGTTTGGAGTT[A/C]AATCAATTTTTAGTGGTTTTATCATGACTATATAACTACTATTCATTGACCACTGAGCTC	A	C	0.31	0.43	0.41	0.37	0.30	0.64	0.26	0.26	0.36	0.43	0.42	0.008	0.012	0.035	0.023	0.0002	1		
rs714195	4	147.02	TACTTTGTATGATCTATAGACTACTGTCTTAAAATTTCAAATTAAAGTAGATTTGGC[A/G]ATAGAAAAATTCTGCCCAGAACAAATCACATTGTAGTTGTCTAGATGTCTGCTTCC	A	G	0.31	0.42	0.40	0.46	0.46	0.73	0.76	0.40	0.39	0.58	0.46	0.007	0.047	0.009	0.044	0.0029			
rs1426888	4	147.56	GCAGTAGATAGTAGGCAACAATGACTCAGGCACAGAAATCTGGGGAAGAAATA[A/G]AGAACGAACTTGAGAGTGGGAGAGAAATCTAGAACTGACAGCATTCAGGTTTCTGGT	A	G	0.50	0.38	0.38	0.34	0.46	0.18	0.23	0.24	0.39	0.26	0.31	0.005	0.015	0.024	0.029	0.0028	1		
rs1960018	4	147.78	AGATGCTGCTAAAATTATGGACTAGTAGATTTTTACCACCAGGCTAAAGAAAA[A/G]TGAGGGAGTGTCTGTCATTTTGTGCTATTATTTTCTGATCCACATAAAGAAAGAGAG	A	G	0.40	0.51	0.48	0.49	0.46	0.55	0.87	0.51	0.36	0.44	0.47	0.002	0.108	0.002	0.003	0.0001			
rs1857721	4	148.17	CAAGCTAGTCAATCATAGGAGAAGGGAGCATTTGCTTCTCTCATCTAAAACCTGT[A/G]GTATTCAGGGACAAACATCATGAGGCACAGCTATTTCTGTCAGGATACATTTAAACT	A	G	0.05	0.37	0.43	0.45	0.38	0.14	0.06	0.32	0.32	0.45	0.36	0.095	0.082	0.004	0.042	0.0051	1		1
rs1437261	4	148.47	TTCCATTAACATGTCTTTCCTTTTATGATGGCGAGAGTTAATATTAAGGTGTGC[A/G]TATATGTTTGTTAAACGATCTCATTTTGCAATTAAAAGTTTGCACTTATATTAATACATTC	A	G	0.12	0.51	0.50	0.50	0.43	0.11	0.02	0.27	0.36	0.48	0.50	0.078	0.152	0.025	0.080	0.0000			
rs1040288	4	149.63	AAACACTGGAGGTACTGAATAGAGTTGTATGACTCTGGAAAAAAGGGCCAAAAT[C/G]ATTACTCAGCTTGGCACACAGAAAGGAGAAGGCCTGAGTTTTGAAGTAAAAAC	C	G	0.64	0.51	0.46	0.35	0.41	0.36	0.43	0.57	0.50	0.42	0.54	0.019	0.000	0.001	0.003	0.0015	1		
rs1512327	4	149.90	GTGGGCAACACACTGGTGAGACTCACAGTATACCCACCATGTGAACCAACTAGATGAGAAAATACATCTCTCTCTTTCATTGCATCTACTATGT[A/G]GAATCATCCTCACATGGT	A	G	0.19	0.48	0.46	0.48	0.34	0.82	0.82	0.64	0.43	0.40	0.40	0.051	0.082	0.022	0.067	0.0008			
rs1032328	4	150.04	CACAGTTGTTCATTTGCTTCTGGGAATGAACATCAAGCATCAAGCATCTT[A/G]GAGATTTGTTGGTCAGTTGGTTATTCCCAAGACTATATTTTAGGATTCCCAAAGAGA	A	G	0.35	0.47	0.43	0.35	0.61	0.91	0.89	0.55	0.39	0.30	0.42	0.005	0.094	0.002	0.108	0.0001			
rs764005	4	150.49	TTTATCAGCAGTGTGAAAAATGGACTAATACATGACATAAACTCTCACATTTTTATGCACTTGGCTTTTAATCTATGATATC[A/G]CTCATTATTTCCATTTGATTCCAGAGATGA	A	G	0.38	0.41	0.38	0.48	0.39	0.91	0.81	0.57	0.39	0.29	0.33	0.000	0.094	0.018	0.149	0.0028	1		
rs538317	4	150.75	ACCTATTCATTTGTAGGCACTGTAGACACAAATATGTGACCAATATCAGAAAAGC[A/G]TAAGTGACTACCATCTGAGGAGATAGAGGGATGGTTAACATACCAATAAGAGTTAC	A	G	0.38	0.52	0.54	0.45	0.50	0.36	0.23	0.38	0.54	0.49	0.51	0.014	0.040	0.015	0.015	0.0003			
rs1394048	4	151.21	AGTGCCCAACATTATTCTTAGATAATAAATTATCCTATTGCCAGTTATATCCT[A/G]TAGCAATTGAGTACTTTCCTGCTTCTTCCAAAGATGTGTGTCTGTGTCATTTTC	A	G	0.10	0.32	0.31	0.23	0.21	0.14	0.07	0.20	0.46	0.27	0.35	0.052	0.057	0.013	0.035	0.0001	1		
rs991529	4	152.07	ACACATACTGCATTTTGTTTTATAATTTTATGATTCAACCCCTATCAAGCAATA[A/G]TTAGTATACTCAGGATTTTTCCCAAGTTGCTACTTCATTGTCCTCCACAGTTGTCTCCAA	A	G	0.78	0.49	0.52	0.52	0.46	0.73	0.34	0.60	0.46	0.51	0.53	0.051	0.012	0.009	0.040	0.0007	1		
rs1516822	4	153.91	TTTCAAGTTTTATTTTTTGGCATTCTCATAAACAAACCCTGTGGACAGCTAGACTGA[A/G]TTTACCCATCATCCCTAAATATGCTGCATAAGCAAATATATTAATGAGAAAAAAAA	A	G	0.63	0.70	0.75	0.73	0.75	0.82	0.74	0.67	0.71	0.67	0.75	0.004	0.000	0.042	0.001	0.0001	1		
rs1878449	4	155.47	GGGGTTGCTTCACACAAGATAAATCTACACACTTAGAATTAAAATATGGCACA[A/G]TTTCCCTGATAAAATATGGCATGGTTTTATTTTGCTATATAAAGTATCCACAGAATCCAG	A	G	0.29	0.57	0.62	0.56	0.63	0.23	0.21	0.52	0.57	0.58	0.59	0.065	0.120	0.008	0.105	0.0025	1		
rs716428	4	157.08	CACCTTGTCTCTTGAAAGTTTTAAGGGCAATTGCATAACTTATCAACCAAACT[A/G]GAAGAGTATAATAGGGTGCAAGTAAAATTATGACAGAATAACACCCCAATCTGGACCGT	A	G	0.62	0.41	0.44	0.52	0.46	0.27	0.54	0.39	0.50	0.43	0.48	0.010	0.001	0.007	0.025	0.0007	1		
rs2090870	4	158.06	GTGAACTGCAATACCAGCCACGAGAGAGCCCCTTAAACTCTATGGCTCTGAA[A/G]CTAACATAGGAAACTGCCTGGAGACTGGAGAGTAAGCAATGGCACTGCTCCAGAGA	A	G	0.52	0.64	0.63	0.60	0.57	0.18	0.36	0.57	0.77	0.64	0.65	0.011	0.058	0.006	0.144	0.0000	1		
rs1554472	4	158.07	AGCATACATTACCAGCCGGCTTTTGTCTTCTCTTCTCTTCTCAGTCATCAGAAGC[A/G]TTTAATTACCAGCTTTCTCTAATTATTCCACTTCATTGTACTTGCCCTGCTAATTCCTTG	A	G	0.50	0.49	0.53	0.55	0.50	0.55	0.55	0.44	0.50	0.59	0.57	0.002	0.000	0.013	0.000	0.0000	1		
rs1352695	4	159.14	CCTATAAATTATAGCTGTGCTCTAGGAGAGAATAAACTGTAGAAATAATGGAGAA[A/G]CTAAAGAGATGTGGTTTAATGAGGACTAAAATCAAGACTTAAGAGAATTTACACAT	A	G	0.26	0.59	0.60	0.52	0.48	0.73	0.92	0.39	0.63	0.50	0.53	0.047	0.114	0.003	0.020	0.0026	1		
rs510719	4	160.58	GTTTCCCCAGAAATGAAAAGGATGATGGGTATGGCAAACTGACATATTTTCAAAAT[A/G]TGACTGCTCCTCAAGGCTATGGTTTTAAACAAAGTTGAAAGAATCTTCAGAAAAGA	A	G	0.74	0.59	0.55	0.54	0.55	0.41	0.60	0.58	0.75	0.63	0.58	0.024	0.002	0.002	0.007	0.0002	1		
rs1439408	4	161.97	CATGTGTAAAAGTCAGTTATTTCACTTCCTCCGCTAACTGGTATTCTCAAGGTTTTTC[A/G]TTTAAAGCAATTCTGATAATTCTCTAGAAGTGTATCATTTGTAATTTTTATTTGCATCT	A	G	0.88	0.63	0.65	0.73	0.66	0.45	0.24	0.64	0.54	0.71	0.65	0.030	0.118	0.006	0.031	0.0002	1		
rs2014158	4	163.31	TAAAGATTTACCTCCACTGTCAGTCAATAATACCTAATCAAGGTCTCAGAATGGGC[A/G]GCATAAGGAATAACAAACACTGAGCTAATATTGTTGAAAATGTAGACTAGGATGG	A	G	0.33	0.44	0.43	0.38	0.46	0.50	0.25	0.53	0.46	0.41	0.45	0.002	0.011	0.009	0.004	0.0004	1		
rs4139	4	163.97	AAGTATCCCGTGCCAGACACTACTCTATGTAGAATGTGCTTTTATTTATCTTTT[A/G]TTTTACCATGTAACCCTCATAGTCACCTCTCTAAGAAGTGTGATTAAAAGAAGCAGA	A	G	0.93	0.74	0.78	0.74	0.82	0.95	0.68	0.78	0.68	0.69	0.70	0.028	0.002	0.003	0.045	0.0060	1		1
rs1458149	4	164.27	TTTTTCTTTTTGAGACAGAGTCTTTCTCTGTCACCTACTCTGGAGTGTAGTGCC[A/G]TCATAGCTCACTGTAACCGCGAACTCCTGGACTCAAGCAATTCTCTCCGCTTTAGCTT	A	G	0.81	0.66	0.73	0.68	0.64	0.25	0.04	0.64	0.58	0.71	0.70	0.006	0.308	0.008	0.131	0.0019			
rs2054210	4	164.36	AGGAAATAAATAGGAATTTAATTTAATTCAATTCCTATTTCAGGAAGAAATGAGTT[A/C]AACAATTAGAGTGTTTAAGCTGATGTCATAATCCAGCCTAATCCCAGAAGTGACAG	A	C	0.31	0.53	0.52	0.56	0.57	0.59	0.80	0.60	0.57	0.46	0.50	0.043	0.037	0.000	0.000	0.0003			
rs924909	4	165.86	ACTGATTGCATTCAGTCCAAGAATCTGTTACTCATTCGTGGCAGGGAATGAT[C/G]AAGACATAGTAATCAGCCTGGTTAACTGGATGTCAACTCCACTTCCTTTTAAATAATAAT	C	G	0.33	0.54	0.54	0.56	0.61	0.82	0.95	0.70	0.68	0.52	0.52	0.039	0.122	0.006	0.029	0.0000	1		
rs1001045	4	166.96	GGGGAGCTGCTTGCCTGGAGAGGTTTTGTGGTGACTATGGAAATTAATCTT[C/G]TTGATGGGAATCAGCTGTTCACCATACTGCTGCCTTCATTCCCAAAGAGAGTTTCCAT	C	G	0.71	0.55	0.55	0.56	0.63	0.59	0.94	0.57	0.57	0.52	0.58	0.010	0.092	0.002	0.000	0.0003	1		
rs1039180	4	167.66	GCACTCTTAGCCATTTCTCTCAGATTTGCTTTTAACAGTGTTAATTCGTTTTT[A/G]TGCACCAAATCCTTTTGGTCTGTTTAATTTTACTTCATCTGAGTGTTTATGCAGAGGTTA	A	G	0.55	0.52	0.50	0.41	0.54	0.86	0.59	0.53	0.61	0.45	0.53	0.005	0.015	0.005	0.105	0.0008	1		
rs954722	4	168.44	CTAGTGTCTCTGCTTCAGAGTCCTGTATCAAGTCCTCTGGATTTGGTCAGAGAT[A/G]AAGGAAAATAGTAGAGAAAAGGGAAAGCTGTTTTCAGTTGTCTCAGCTTGAAAGTG	A	G	0.31	0.26	0.23	0.15	0.23	0.27	0.43	0.17	0.21	0.24	0.26	0.006	0.023	0.013	0.001	0.0011	1		
rs1385513	4	168.85	TCTCATTGTTTGAATTTTAATGGCCTGTAGAGCCATAGAATACTTTATTTGGAGT[A/C]GACATAGAAACAAATTGTTGTATTTACTTTCTAACAACAGTAGAAGTTGATAGACCCTC	A	C	0.68	0.86	0.85	0.84	0.77	0.68	0.63	0.86	0.86	0.78	0.84	0.022	0.040	0.001	0.020	0.0000			
rs1478224	4	169.13	AAACTATATAAAATTTAAGATGTTCCTTCCAAAGAATCATTTGTGTCTGGAGGTTAA[A/C]AAACAGGCAAAATAAAATCTGAATAGAGGCACATAGCACCTCTACATAAAATAAGA	A	C	0.60	0.62	0.54	0.49	0.61	0.73	0.66	0.59	0.69	0.45	0.58	0.000	0.010	0.000	0.013	0.0001	1		
rs1566499	4	170.43	CTTCCCTCCAAAGTCAGAGGTTAAGGACAAAAAAATTGAGTGCTCACAGGTAAG[A/C]TGACGGAGATCTCCTAGATAGAACAGATGTTGAAAGACATGAGATTTGGTGACTTA	A	C	0.62	0.41	0.46	0.45	0.36	0.18	0.01	0.44	0.50	0.49	0.49	0.011	0.163	0.000	0.043	0.0004	1		
rs931145	4	171.64	GCAAAGGATGGTTAATACTCAGATGTTTTCTGGAAATGAATTTCAAGGTCCTCAAAA[A/G]TGTTTTGAAATGGAGAAAAACAGGTTACCTTTGAGAAGTTCTAACCAGAGCTGGA	A	G	0.17	0.58	0.52	0.46	0.59	0.00	0.46	0.31	0.54	0.41	0.44	0.055	0.004	0.014	0.223	0.0024	1		
rs1533837	4	172.34	GCATAACAGATACATCAAGCGGTGTGTGAAACTGTGGTCATTCCTGACTAGGA[A/C]AGCTCCTGCTTGGGTAAAATAAACCAGAGCCACGTGATCCACTTTGGGGAATTCAA	A	C	0.14	0.68	0.71	0.73	0.84	0.55	0.33	0.84	0.79	0.59	0.59	0.153	0.077	0.024	0.011	0.0065	1	1	1
rs2178299	4	173.08	TTATTATATGTTTAAGAGCTAAAGTCAAAACTTTTCCTTTTTGCGTAACTAAGATATGTATTTAATCTATAACCCAATACAATGTTCAGTTGT[A/G]GAACTCATCATTGAATC	A	G	0.38	0.76	0.76	0.71	0.82	1.00	0.89	0.72	0.96	0.70	0.69	0.094	0.009	0.005	0.080	0.0042	1		1
rs1569214	4	173.17	GAAATTTTATTGAGGCTTGACAGATGGATAAGAGATAACTGTGAGGCAGATAGCA[A/G]AAAAGAGCTTGTTGATTTGGACGAAGCAAAGCAGCAAGAGCAGAGCAAGTAG	A	G	0.79	0.83	0.85	0.87	0.89	0.55	0.61	0.69	0.79	0.83	0.83	0.002	0.036	0.013	0.052	0.0007			
rs1472370	4	174.18	TTGAAATAGAGCTACGGAATTTTCTTAAAGAATAAAAGTTAATGAGTTCTTTAA[A/G]ACACGAGAGGGTAATGCACAATGTGATCTAATAATAGAAAATAAAACTAAATGAGACA	A	G	0.05	0.59	0.56	0.58	0.52	0.64	0.88	0.55	0.61	0.52	0.55	0.170	0.061	0.001	0.001	0.0012	1		
rs1824347	4	174.46	GGAGGCTGTCAGCTGTCATGCTGAGAGCAGAGTAAATGGATCTGAAGCAAC[A/G]CTTTCCAGGGTTTGTGCATGTTCATTAGGGGTCGATCATGAGAGGCCTTACAGCAGC	A	G	0.14	0.61	0.52	0.57	0.63	0.95	0.85	0.56	0.61	0.56	0.56	0.084	0.064	0.001	0.135	0.0000			
rs1014381	4	177.25	ATGCCTAATGATCTCTCATTTTTGGCCTCTTTACCTCATTTAATGACTTTTCTTTC[A/G]TTCATCCACACTGGTTCAACTGTCATTTCAGGTAAAACCTTTTCTTTACTGTCCCAT	A	G	0.38	0.39	0.44	0.51	0.45	0.32	0.15	0.40	0.46	0.52	0.55	0.000	0.051	0.000	0.008	0.0058	1		
rs1472328	4	177.34	GATAAGACCAGCTCCTTCCCTTATTACTCTTAATTTTCAAATGCAACAAGGCT[A/G]TAAGATGAACATTCCATTCCATATTGTTTCTTTTAGGTAATAAAGAATTGAAACACAG	A	G	0.62	0.92	0.86	0.80	0.80	0.73	0.92	0.77	0.79	0.86	0.78	0.043	0.004	0.013	0.017	0.0070		1	1
rs1993281	4	179.32	TTAATATCAGCTTTATCAGACCCATATGCTCCAGGAAATCAAAGAACAACAGAAAAACTTCCCTCCATTTCT[A/G]CATGAACCCATGTACTCTAATGGTGAGATCACTGACTAGT	A	G	0.21	0.41	0.42	0.48	0.29	0.55	0.08	0.31	0.50	0.39	0.42	0.019	0.073	0.002	0.017	0.0002	1		
rs2044868	4	180.62	ATTATAGTATTGAAAGAAGTGCTATAGTGAGTCAACCTGGCTCACAATACAG[A/G]CACTTGAAGACTAGAGCATGCTCTTGGGTTCTTAATAAACTGTGATCTGTTTGTACCTC	A	G	0.62	0.54	0.48	0.45	0.46	0.86	0.70	0.42	0.50	0.52	0.45	0.022	0.055	0.000	0.116	0.0000	1		
rs1363207	4	182.33	AGTGTTGTAATAGAGGATTTTAATATAAATAGTTTGAAATTGTATAAATATTTTCTG[A/G]TTGTGATACTTGTGCTATTTGGAATATCAGAATACCAGGAAATGTGGATGTATAAAT	A	G	0.24	0.60	0.61	0.66	0.46	0.73	0.94	0.67	0.54	0.52	0.56	0.046	0.103	0.008	0.015	0.0000	1		
rs724659	4	183.02	ATCCCTCTGATCTTTCAGTACATCACGCACAGTTTTGCACCATTTACCAGAATTTCTCCCAC[A/G]TGATTAAGCTGTTGTCATCCACTGTGGTAGCTGATTTAATGAGTTCCCCT	A	G	0.71	0.50	0.49	0.52	0.43	0.55	0.74	0.68	0.46	0.59	0.52	0.027	0.031	0.015	0.001	0.0001	1		
rs2045405	4	184.01	TTAATTACCTCAGCTCCGTACGGTAGAGCAGGTGGCTGCAGCAATGGAGAGCC[A/G]CTTAGAGGAAGAACGACCCTTCACTGTTCACATCCCACAGCCTTATATGAAAATGTG	A	G	0.43	0.84	0.86	0.87	0.84	0.73	0.35	0.75	0.81	0.81	0.81	0.107	0.134	0.013	0.014	0.0014	1		
rs1516538	4	184.02	CAGAGGAAGCAGTGATTTTATGGTGGAAAAAAACTTTTCAGTAACGAAATAACA[A/C]TAGTGAGAATAAATAAGAAAGCTCTTGGAGCTCCCTTAATTAGAAAAACAGAGCTTT	A	C	0.24	0.41	0.45	0.39	0.34	0.18	0.65	0.35	0.39	0.50	0.43	0.021	0.018	0.006	0.039	0.0003			
rs2063906	4	184.07	CTTTAATCCAGCCTCCACACTGTCGTCACAGTTGTCATTCTGAGTTGTGTTTT[A/G]TCAGTCCCTTGGTGACCATACCTCACCCTCTACTGCTCCTCCACTCCCACCTTCGCAC	A	G	0.88	0.76	0.74	0.72	0.63	0.86	0.94	0.80	0.89	0.66	0.71	0.036	0.073	0.017	0.035	0.0005			
rs1514483	4	184.26	CTCCAAGGTTCTGGCAATTGCTGGACTATGTCACCAGCTGTCATCACCCTTCCTT[C/G]CCATCCATTTTTGGCAGGATCTGGGGACTCAAGCATGCCAGTAGAAATAATAGTAG	C	G	0.95	0.57	0.57	0.62	0.55	0.95	1.00	0.69	0.62	0.68	0.61	0.089	0.170	0.007	0.103	0.0005			
rs2016910	4	184.66	ATTCCTTGCTGTGAATGTTGCTTTGGGGTCATCCCCGTTTCATTTTTACAATGGTT[A/C]CATAAGCTTTCCAGGGTTCAACTACTTTGGCTTCAAGTCTTTCAACCTCTGCTTGTCA	A	C	0.64	0.69	0.67	0.76	0.59	0.77	0.64	0.61	0.57	0.70	0.71	0.000	0.000	0.000	0.009	0.0019	1		
rs907362	4	185.02	AGAGTGACTCCTTCCCAATTAGGAAGGTGACAAAGGACATCACTAAGCACTGA[A/G]GCTGGCAGGGTGAGTGGCTTAGTGCCAACAACCACAGTGGCCTTTTACACCAAT	A	G	0.55	0.54	0.53	0.49	0.61	0.27	0.33	0.51	0.57	0.47	0.49	0.000	0.032	0.003	0.051	0.0009			
rs4241779	4	185.30	GCAATTGCTCAGTTCCGAAACACATCGACTTGTGTAAGAAAAGATTGGAAGTGC[A/G]GAGCTGTCTTTGAGCATGATGCATGGATGTCTAAACAGTATGTTTTACATTGTCC	A	G	0.33	0.64	0.57	0.63	0.57	0.59	0.42	0.77	0.50	0.61	0.65	0.039	0.016	0.017	0.000	0.0009	1		
rs1983503	4	185.53	CAAGCTATTCTCTCGTGTTCACAACGAACACTGCCGCACAAATGACCTGCAACTTATAC[A/G]CCTTTATTGTTCACCTGAAAGCCCTCTGTAGTTGGAATACCAAACCATTAGCCC	A	G	0.14	0.28	0.37	0.26	0.27	0.14	0.21	0.27	0.27	0.36	0.37	0.020	0.004	0.000	0.023	0.0031			
rs1921565	4	185.82	ATTGAGGAACATGTATTTCAAAACTCTTTGAATGTGAATGTATTTCCCTAAATCACAA[A/G]TGGGGATTTAATCAATATTGAACAAAATGTCTGCTTGTTCTACTAGAGTTGAGCA	A	G	0.74	0.39	0.44	0.52	0.45	0.00	0.00	0.65	0.64	0.46	0.42	0.035	0.202	0.013	0.202	0.0008	1		
rs13478	4	186.31	AGCCATCTTGCGAAATATCAACCATCAGTTAGAGAAGCTCCTTGACCAGGGATGAGAA[A/G]AGCAGTCTACTAAAATGTGCCTATAGGAAGACTAGTCTCATGCTGTTACCTTCT	A	G	0.07	0.16	0.13	0.17	0.11	0.68	0.66	0.36	0.04	0.16	0.09	0.003	0.168	0.047	0.194	0.0028			
rs768614	4	186.63	AAATCTCAATTGGTTAGATTCATGTAAAAAATGAAGTGAACTGAAACTGTA[A/T]GCAGGGGGTGCAATCTGAGGCCGGTGGACAAACAAACGAGGCACATTGGCCTGCTTAC	A	T	0.76	0.77	0.76	0.82	0.84	0.91	0.81	0.80	0.93	0.82	0.79	0.000	0.001	0.003	0.008	0.0020	1		
rs1564986	4	186.65	ATTAACCAGGTGAGATGGGAAAGAAAAGGAAGAAAGAGGTTTCAGGGAGACAGAAC[A/C]TCTTTGTGAGGGCTCCAAGGCATGCAAGAAGCCCTTTTGAGGAGCTGGTGGTTG	A	C	0.73	0.34	0.41	0.38	0.34	0.68	0.67	0.47	0.32	0.38	0.38	0.076	0.058	0.016	0.065	0.0000			
rs1545299	4	187.03	GTCTTGGTCATTAGAGTGTACTCAGGGCAGCAGATCATCTGCAAAAGCTGTGGGCCA[A/T]CCAGCTTCCATAGAAGTTCCTCCTCACAAAAGAGAAATAGTTCCTTAGAAGAACCTGG	A	T	0.69	0.29	0.28	0.24	0.21	0.27	0.08	0.44	0.14	0.35	0.31	0.087	0.042	0.006	0.001	0.0025			
rs1158465	4	187.17	TTTGAAGCAAATACTCTCAATGAAAGAAAATACATATTCTTTGCAACACCCTGGAATTCA[A/G]GATTGTCTGGGCATATGTGAAGTACTTGTGAATCCACTGGGTACTAAATTGGTATT	A	G	0.83	0.41	0.44	0.52	0.50	0.50	0.68	0.63	0.43	0.60	0.49	0.072	0.016	0.005	0.000	0.0000			
rs1566347	4	187.43	CAATTGGAAGATAAAAAACATGACCGATATGCTTTGGGGTTTTAAAACCAATATTT[A/G]CTGTTTGAGCAGACTACGATGGCTTGTTCTGAACCGTCCTCACAGCCAACAACATT	A	G	0.36	0.33	0.42	0.33	0.30	0.27	0.30	0.32	0.21	0.27	0.31	0.004	0.008	0.006	0.015	0.0035	1		
rs830835	4	187.61	AGATTATCTGTAGTTGAACAGGGAGTTCACAAGCTTTCCCCATGACTCAGAAAATT[A/G]TAACTGCCTGAAGAATTTTTAGAAGGCTGTAAGTGAGGAGATGGTCCTGTGGGAC	A	G	0.93	0.86	0.84	0.85	0.77	1.00	0.98	0.81	0.73	0.87	0.78	0.006	0.023	0.002	0.050	0.0023			
rs1473597	4	187.82	CTAGAAAGAGAGGAAGCAGATGAGCAACGGCACAGAAATGGAAAGGTTTCTGC[A/G]TGGAGGGCAATAGTGGAGAGTGAGAGACCGCGAGAACTCACTTCTCATTCACTGG	A	G	0.55	0.67	0.61	0.56	0.63	0.73	0.63	0.39	0.79	0.55	0.53	0.000	0.003	0.007	0.011	0.0043			1
rs1864604	4	189.05	ACAGGCTGTTCTGGGATAAGGCTGAGAGTAGGTGAGACTCGTTAGGACTCTAAAAG[C/G]CATGACAGTATGCAAGGAAAACATCATTATTTTTGTAAAGGCTGTGTGTTAGTCCATTTGTATTGCT	C	G	0.14	0.28	0.22	0.23	0.18	0.32	0.28	0.33	0.18	0.20	0.22	0.008	0.004	0.011	0.009	0.0001	1		
rs996026	4	189.31	TGTAGAATTTCACTATGTGGCCAATAATGATGTCTGTACAACATACTTTGATTTGC[A/G]GCCTCTTGTTTGTAGTTGGCTTTTACTAAACAAAGTGACTTCTTGGAAGTTAATCTG	A	G	0.31	0.30	0.31	0.28	0.41	0.59	0.14	0.50	0.32	0.34	0.38	0.002	0.015	0.040	0.052	0.0041			1

rs539718	5	4.00	GTGTACCAGTTAGCTAGGGTGTCATAAATATCCACAAACTTGGGAACTGCAAACA[A/G]TAACTCTTTATTACTGCTCAGCAGCCTATGAGCCAGCTGGGCTCAC	A	G	0.64	0.07	0.08	0.06	0.09	0.00	0.00	0.04	0.00	0.06	0.05	0.209	0.018	0.005	0.018	0.0046			1
rs959937	5	4.28	ATTCTTGTTGCTCCTTTCTGTGACATGTCAGCCTGGGTCCATGTGTGAAACTG[A/G]TGATCGAATGATTAAATCAAGAGAGTTTTGATTAATTTTTTTCCCTCC	A	G	0.33	0.26	0.25	0.28	0.25	0.50	0.26	0.42	0.27	0.31	0.35	0.004	0.003	0.030	0.034	0.0042			
rs261198	5	4.45	TTGCAAGGAGCAATGCTCTTAAATTCCCGTACCCCTTAACCTCACAACTCTCCA[A/C]ACTTAGGTTTATTAACCACCTCTGTCCCAAGGACAGGAGGCTCAGG	A	C	0.83	0.93	0.87	0.90	0.95	0.73	0.80	0.83	0.92	0.90	0.85	0.000	0.000	0.000	0.009	0.0001			
rs2052886	5	4.63	TGCCAAGATAGCTAGCCCGGGGACATGGCGAGGGAGTTATTAAAATCAGCTCAG[C/G]GGTTCGCAGGAGAATTCTGAAGAGGGCACATTGTCCAAAAGGAC	C	G	0.43	0.35	0.29	0.21	0.25	0.55	0.27	0.36	0.25	0.29	0.26	0.018	0.000	0.019	0.046	0.0003			
rs816480	5	4.89	GCAGCCCTGGCCCAAGCCTCTGAAGGCCTAGAATGCCAGATGGAAGGCATTGGTGAAC[A/G]AGGGGTATATAACAAAGATTTCCAATAACAATACAGATGCT	A	G	0.74	0.08	0.13	0.21	0.21	0.23	0.43	0.17	0.04	0.16	0.22	0.240	0.074	0.005	0.012	0.0054	1		1
rs171610	5	5.07	ACTGACATCCAATTCACAGCTTCTTTAGGAACCCCTGTGATGACATTGTTTGGCA[A/G]CCCAGGAATGTCTAAGAATTTAGTTCCAGTCTGCTAGCAGAGAGC	A	G	0.83	0.89	0.89	0.89	0.93	1.00	1.00	0.94	0.93	0.85	0.89	0.002	0.045	0.002	0.045	0.0000			
rs1560062	5	5.27	ATTTTTATCCTGGCATTCTATCAAGTTGTCATTCCTGCAAGCAAATAGAGCTGGCC[A/G]GTTGTCTGTTTGGAGAATTACATTCAGGACCCTTGAATTCATGGG	A	G	0.86	0.86	0.85	0.87	0.89	0.73	1.00	0.91	0.93	0.90	0.87	0.000	0.050	0.004	0.014	0.0011			
rs4702266	5	5.45	CAGCTTTGAGGGTGATACGCACCTTTGCCCCCACACATTAGTGAGGCTCACAGC[A/G]AGTTAGGTAGATGGGATCTGAGTCTTGATCAGCCATCAGCATGTG	A	G	0.88	0.64	0.61	0.75	0.64	0.95	0.66	0.79	0.57	0.71	0.61	0.054	0.007	0.014	0.096	0.0002			
rs246765	5	5.63	GTAGTCAATGTGCTTGGAAGACATATCTAXAAACTAATCTATGGTCTTCCCAACC[A/G]ACTCTCATGTCATCGTCCCTGCCATGTCCTGCCCATATTACTTCTC	A	G	0.05	0.06	0.07	0.05	0.05	0.05	0.20	0.18	0.11	0.10	0.09	0.002	0.018	0.013	0.004	0.0008			
rs378932	5	5.66	GATCTCTTTGATGTAGGAAGGGTCATGAACCCCCATACCACCTGGGAATCTG[A/C]GTGACTCACAGCAGTGGCGGGGGTGGCAATGTGGTCAGTGGTCCTTC	A	C	0.76	0.72	0.67	0.72	0.68	1.00	0.90	0.74	0.82	0.74	0.69	0.005	0.053	0.014	0.140	0.0002			
rs2173946	5	5.96	TCTCAGGCAGGAGTGCTAGAGTCCCAGATAAGCGTCACACTCCGGTTTAAGTTAATC[A/G]TGAAATCGAATGCCTGGGTCTTCCTGGGTCTGGTGAAGGCCA	A	G	0.38	0.39	0.34	0.29	0.21	0.36	0.15	0.20	0.39	0.37	0.27	0.001	0.061	0.031	0.004	0.0039	1		
rs307195	5	6.12	GCTGGTACATTTACACATGAGACGTTTATACCTAATGTGGGCATACCTGGAGTGC[A/G]TGTAGGCTTTCTCTACCATTCTTCTGCATGTTGATTACCCAAGGGA	A	G	0.10	0.40	0.42	0.37	0.27	0.75	0.31	0.38	0.50	0.30	0.37	0.088	0.005	0.000	0.052	0.0012			
rs3846569	5	6.32	CAGTTTACCATTGTATACTGTGCAAAGAAGCAACGACTCTAAATAAGTTTGCTCTGC[A/G]AGCCTTCACACCATTGTCCTTTGTCAGGTATTCTTTTTTGAATCT	A	G	0.12	0.05	0.09	0.05	0.07	0.09	0.14	0.07	0.07	0.10	0.10	0.000	0.000	0.002	0.002	0.0005			
rs720846	5	6.66	CTCTCTCTTTCTCCCAGTTCCTGTGGCAGTTTGTCCAGGTGCCCAAGAACAATTC[A/G]TACCCTCCTTTCTCGTTATTTATATACTTGTCTTTTTGCCCCTGCCG	A	G	0.24	0.36	0.37	0.30	0.36	0.23	0.50	0.23	0.54	0.29	0.32	0.005	0.021	0.002	0.003	0.0023	1		
rs875347	5	7.74	GTTTTTATTTTGGAGGAAGAAATGCACATTAGTACAAAGTTGTCCCTGTACTC[A/G]GTGGGATCATTCCTGCTCTGTGTAATTCTGACACCACTGAAACGACCAC	A	G	0.43	0.49	0.39	0.43	0.45	0.82	0.17	0.59	0.46	0.44	0.44	0.000	0.012	0.035	0.124	0.0001	1		
rs1394641	5	8.56	TAAAATAATCATGTATCTTGGCTATATAGTTATATGTCATTTTTTAAAAATGCATTGC[A/G]TAGATAAAACCTAGTTAACCAAGTAGTTGTGGGAGTTTTAGGAG	A	G	0.90	0.56	0.52	0.52	0.52	0.82	0.47	0.70	0.43	0.65	0.54	0.097	0.000	0.025	0.058	0.0000	1		
rs756757	5	8.62	GTCTATTTCATATGCAACGTATACTTGTGCTTTGTTGGGAGACAATTCCTCATGAGTCTCATATTTCTCCATGTCTTGTAAGCAGAGAGTGCCT[A/G]TTGTTTCAC	A	G	0.67	0.68	0.70	0.68	0.71	0.77	0.91	0.56	0.75	0.62	0.69	0.002	0.020	0.013	0.000	0.0004			
rs40725	5	9.44	TGTCCCATGCATGCCACAGCAGGACAATCCTTAGTACCAAAAGGCTGAAGCGATTGCT[A/G]CATGTGTGTCTCTTAATTAGGAACAGTTACAGATTGTCATAAT	A	G	0.17	0.07	0.05	0.11	0.07	0.09	0.02	0.13	0.04	0.07	0.07	0.018	0.018	0.023	0.003	0.0014	1		
rs1506030	5	10.11	ACAGCCCAGCAAGATAAAGTACTCAGGACAGCTGGATTTTAAGAACACCTTTGTGA[A/G]TTGGGTCTTTATTGGGATAATCAGAGAAAATGACTACATTTAGG	A	G	0.33	0.58	0.63	0.55	0.63	0.09	0.38	0.45	0.43	0.58	0.48	0.034	0.037	0.029	0.176	0.0091	1	1	1
rs717259	5	12.34	GAATGAATGAGAAAGAGCCATACACTTG[A/G]AAAGGTAGAGGCCAGAGGGCCAATGTCCTAAAGTTGGAACAAGCTTGCGTGTTAAGACCTAGGTAACTAAA	A	G	0.88	0.57	0.56	0.50	0.71	0.60	0.70	0.48	0.61	0.60	0.58	0.074	0.012	0.000	0.002	0.0013	1		
rs917042	5	12.50	AATTGAAGGACACAGTGGGAAAAAGCAATGATTCCAGAACTTGTATTGGCTTTT[A/C]AAGAATCCCTGCAGTTAGGGTAGCAGAAGTAAGAGTTTAGGAAGA	A	C	0.57	0.49	0.49	0.44	0.59	0.59	0.70	0.42	0.50	0.47	0.56	0.002	0.022	0.001	0.004	0.0045			1
rs170856	5	13.64	AATCTGTTAACATATTCAAAAATCAGGTCTTTAGGGTTTAGGTTCTTAGCATATG[A/G]CCTCTTCCACATTCCCTGGAAGTTAATTCCTAATCTACAATGCAGCC	A	G	0.69	0.33	0.39	0.32	0.55	0.73	0.32	0.51	0.36	0.39	0.44	0.030	0.012	0.001	0.028	0.0000	1		
rs1373942	5	13.71	TTTCAAAATTTTTCTTCCACCTGACTTTTTCTCATCATTATTTAAGCGGCCCA[A/G]TTTCTCCCCTCTTTAAAACAAAACAGTACCAATGACAAAACCTCCTTCCA	A	G	0.31	0.50	0.42	0.49	0.46	0.64	0.68	0.51	0.39	0.45	0.45	0.002	0.051	0.021	0.042	0.0008			
rs2034222	5	13.73	TCTCTCCTCTGTACCTGAATTGACCTGTAACAGGAGAAAGAAACTCACCGTGG[A/G]TGAGCCCTCCTTTGTCCTGGGTACTTTCAACTTGTTTGTCTTTATTGT	A	G	0.57	0.52	0.57	0.49	0.55	0.40	0.49	0.41	0.61	0.56	0.62	0.001	0.004	0.016	0.023	0.0012			
rs879253	5	14.85	CCCTAACATCATTCAGGAAGCAAAACAACTAGTCAGTTCCACATGTTGGTTTACCA[A/G]TCTCAAGGCCCCATCTGTTCAGGAGCAAGCTTCTTATATGACTTG	A	G	0.81	0.53	0.58	0.48	0.57	0.77	0.90	0.70	0.71	0.52	0.51	0.040	0.067	0.026	0.028	0.0018	1		
rs1004673	5	14.89	TACATGCCCTTCACAGTCAGTAGGAAAGTGGAAGGTTCTGACACCTAAAGACA[C/G]CCAAGGGGCATCCTGGAAGGTGACTGCAAGGGCATCTATGGAAAG	C	G	0.36	0.30	0.32	0.29	0.38	0.36	0.51	0.33	0.32	0.34	0.29	0.000	0.008	0.000	0.000	0.0008			
rs1550825	5	14.96	AATGAAACATCTGTCAGCATTTGATTGTGTCATAGACAATGCCCTTGCCACACCC[A/G]TACATTAATGCTCGAAGAAAAACTGTGGCGTGTATATAATGGAATG	A	G	0.62	0.41	0.35	0.40	0.36	0.30	0.33	0.56	0.39	0.39	0.41	0.024	0.000	0.020	0.001	0.0016			
rs924455	5	15.80	TAAGAAAGATGGGGTAATATTTATAAGTGGGAGGATGAGTTACAGATGGAA[A/G]ACCTGGATCCCACGATTCCCTTATGGCTCTACATCAGGCAGTCATCTTAT	A	G	0.52	0.42	0.46	0.48	0.41	0.27	0.53	0.41	0.54	0.35	0.45	0.002	0.002	0.014	0.056	0.0002	1		
rs1505034	5	15.83	TGTAAATAGGATCCATGTGCAAAAGAAGGAAAGGGAATCTAAGAACATGGAAC[A/C]ACAGTGAACATTTATGCTCTCCAGATCTTTCCTTTCTTCTATTCATTTG	A	C	0.40	0.60	0.55	0.52	0.57	0.73	0.47	0.65	0.50	0.66	0.53	0.001	0.001	0.026	0.048	0.0000			
rs162848	5	16.53	TTAGGACAAAGAGCTGAAAAGTCTAATTCACTGTCATCTTTGTGACTTTTTTCTTT[A/G]TCTGCTTCTGTTGAGGAAAAAATTTGGAAGCTTTCAGTTTCCTAGC	A	G	0.79	0.67	0.67	0.66	0.66	0.73	0.54	0.61	0.68	0.67	0.70	0.010	0.004	0.002	0.003	0.0003	1		
rs298552	5	17.39	TGCCCTCTAACCAAGGTCTTTCCATTCTAACCAATCAAGTATATTTTCTTTGTCTT[A/G]CTCGTGTGTCCACCTACAAAAGCTCTCTGCCCACCCTGCTGTAGCA	A	G	0.60	0.45	0.37	0.37	0.46	0.36	0.48	0.40	0.43	0.45	0.44	0.045	0.012	0.005	0.000	0.0017	1		
rs905176	5	18.84	TAGAAACAAAGACAGACATTAGAGATACTAATATTTGTAATAGAGGTGATTTTTCCAGGTAAGTATTGATAATGAGATATGC[A/G]CAAATTTTGTTGAAAGGTTTT	A	G	0.14	0.39	0.33	0.35	0.29	0.09	0.30	0.19	0.50	0.32	0.34	0.030	0.000	0.009	0.036	0.0001	1		
rs1498106	5	19.83	CTTCTTATCTAATTGTACCTTATTAATATTTGGTGGAACTGGGATGTGAAAAGAAGCCTATCACAATCTCAAGTTCATGTTCTTTCCACTGCTGTATCTT[C/G]CC	C	G	0.48	0.45	0.45	0.46	0.39	0.64	0.43	0.59	0.61	0.42	0.40	0.000	0.001	0.005	0.011	0.0014	1		
rs720189	5	20.60	TACAATTTGAACTAACATTAGGACTCTATAAATTTTCAACTCTTTAGGAATCTC[A/C]CTCCTCCTAGTAGCCCCCATATTTTTAAATGTAATGTTTTACTATTTTCT	A	C	0.81	0.50	0.52	0.51	0.61	0.45	0.53	0.58	0.71	0.51	0.54	0.040	0.000	0.006	0.002	0.0000	1		
rs1442470	5	22.94	CATGAACTTTGGGATTAATTGGCTATGCGGAATACAGGGAATAGAGGGAAGCAAAT[A/G]CTCTCCTCTCATAGCATTAGCACATAGGACGCTGGGTTTTGGGA	A	G	0.07	0.30	0.24	0.29	0.32	0.00	0.36	0.07	0.43	0.25	0.26	0.034	0.010	0.020	0.085	0.0004	1		
rs919332	5	23.29	ATACCTTAATTATCTTGATTCCAGCAGTTTGAATGGTCATGAAGCTTTATTACGTTT[A/G]CAATTGTTATCAAGTTTTAAAAAAGACAACAATTATATACATGGCA	A	G	0.30	0.28	0.24	0.29	0.34	0.36	0.28	0.31	0.11	0.24	0.23	0.002	0.000	0.001	0.006	0.0005			
rs1354168	5	24.09	CAGGTTAAATGTTGCTAATGACAAGTACTCACATAAAATTTTGGAAGTAAAAGG[A/G]AATTAAATCCTACTTGTCTGATGTCTACTGTGGGCATGTATGTGTGCT	A	G	0.07	0.46	0.48	0.48	0.46	0.32	0.19	0.34	0.39	0.53	0.55	0.105	0.064	0.020	0.013	0.0017	1		
rs1857844	5	24.57	ACTCTGGAAATTTGTTCTCAAACATTTTATTTTTAGTTTGACTTCTTTGTGATT[A/G]TTTGATTTGTATTCTTAGGCACATGCTTAGAATCTGAAAATATGTCTGCT	A	G	0.55	0.46	0.50	0.54	0.54	0.60	0.21	0.54	0.29	0.55	0.55	0.003	0.043	0.006	0.008	0.0019			
rs1910966	5	24.58	AAATAGATTAGGAATAGGTAAAGAACCTTTAGCTCCTGACCATAGAAACACAT[A/G]CATTTATTCTTCTTTCTTGTACAAATTTGGTAGATTACATGTCTGCTCG	A	G	0.43	0.46	0.50	0.54	0.52	0.60	0.21	0.54	0.29	0.55	0.56	0.001	0.047	0.005	0.006	0.0023			
rs1428242	5	25.61	ACCACAAGTAGATTTATCTCTCAAATGTTTCATCCCTCCTATTACACTAATTTAC[A/C]ACATCTGAAGGATAAATCCAGATTCACAATTTATTAATGTGTAGTGAA	A	C	0.76	0.65	0.67	0.74	0.59	0.82	0.89	0.64	0.75	0.70	0.70	0.000	0.028	0.001	0.010	0.0001	1		
rs1428589	5	27.96	TTTGCATATTTTGACCAACCTCTCCTCTAACTTTTCACCCCGTATCCCCAAACTCA[A/G]GCTCCTGGTAATCACCATTCTACTCTCTGCTTCTATGGGTTCAAC	A	G	0.31	0.45	0.45	0.50	0.43	0.14	0.21	0.47	0.36	0.48	0.41	0.017	0.059	0.004	0.090	0.0002	1		
rs1862559	5	28.78	GAAGTACAAATACAGCAGTACTTTCTTTTTATCAAAAATAAAGATATGTGTTCCATAGCTAACAATACTTGTGTGTTCTCCACCAACTA[A/G]ATTTTTATTATGGG	A	G	0.45	0.49	0.33	0.49	0.45	0.15	0.33	0.41	0.39	0.48	0.36	0.012	0.000	0.002	0.014	0.0005	1		
rs1423287	5	28.81	ACAGCAATGTCACTTAAATGTTATGAAAATAAATTGAGATACATTCAAAATAGAAGATATTCATTATTTTACATTGTTTTGTAAGGCAATAAACAT[A/G]ATATATG	A	G	0.45	0.49	0.33	0.49	0.45	0.18	0.33	0.41	0.39	0.48	0.37	0.012	0.000	0.002	0.008	0.0008			

rs1864003	5	141.81	ACATCCTAACTGACCTGTTGCTACTTTGCAGAAACGAGCGTAGAGAAGGCGAC[A/G]TTTTGGGGAGCTAGCCCTGAGGTCAGTGGTCGACAGAAGGGCTCTATCCAAGTAATC	A	G	0.83	0.42	0.43	0.52	0.48	0.73	0.32	0.64	0.50	0.52	0.51	0.118	0.002	0.038	0.064	0.0026			
rs33388	5	142.73	TTATGGGTGAAAGTCATGGATGGATTATGAGTTA[A/T]TCACACACCTAGAGAAGCATGTAAAATGTGCAGGTAAATTACACCCATTCATTCAGGCAGACGTTTCCTGGCACCTGA	A	T	0.55	0.52	0.53	0.52	0.50	0.23	0.18	0.48	0.35	0.53	0.43	0.000	0.090	0.006	0.060	0.0067	1	1	1
rs464727	5	143.32	CTTCATCTCTGAAAATGAAAGGAATAACTTGTGAAAAATTATGATTGCCATTTTTAT[A/G]TGTAATGAGGTGTATGTATAGGGAAAAGCAGTTTGATGTAATCCTGTAATACTTTTA	A	G	0.57	0.15	0.19	0.13	0.14	0.41	0.13	0.35	0.32	0.16	0.20	0.080	0.003	0.017	0.038	0.0000	1		
rs29900	5	143.78	GCTCCTGATTCTGGGCTAACAGCCCTCCAAAGGGCCTGCCATGGTATTTCATT[A/G]TCTGGCATTTCCATATTTCTGTCTTGTTTTTCTTACTACTCCCTGGCCAGGCCTCATAC	A	G	0.79	0.72	0.70	0.72	0.73	0.77	0.90	0.94	0.89	0.67	0.67	0.007	0.038	0.059	0.003	0.0010			
rs248771	5	145.47	GTGTGTGTTGTGGGGAGAGGTGGAGGGAGGTACAGGAAGGTGATGGAGGGAGGCATAGTTG[A/G]GTGGAATGAAGTGCCCAGAGTCATGGGACTGGTAGTCAAACAGGCTG	A	G	0.26	0.39	0.41	0.44	0.38	0.27	0.44	0.25	0.27	0.29	0.27	0.007	0.002	0.009	0.011	0.0105	1	1	1
rs1480150	5	146.48	TGACTTAGTTGAGTTATTGCAAGACACTTACACATTTAAAAGTTCTGATTTACC[A/G]TGTGCAATGCTTGTATTTGATTTGCTTAGCAGGCATATTTCATATAACATATGATTTCAT	A	G	0.74	0.56	0.60	0.65	0.71	0.82	0.51	0.65	0.64	0.61	0.64	0.008	0.010	0.000	0.024	0.0013	1		
rs728937	5	146.51	CAGTGGTTCATCCAGCATAGTGAATACTTTTCTTCCTCCCTCCTTCTAGCAAAC[A/G]TTTATTAAGCATCCTGTCAGCATGTGTGATGCACTATACTAGGCATTGTGACCAAGATG	A	G	0.86	0.41	0.46	0.29	0.43	0.79	0.45	0.46	0.23	0.49	0.49	0.096	0.000	0.000	0.053	0.0002			
rs1016256	5	147.56	GTCTATGGCTAACAGCCTAAATAGAAATAGCTATCCTCAAAATTATCCTGTGGAGGAAGAAAAGTATAGGAGATTCATGAAAATGT[A/G]ACCAATATATATTTCCTGCTTGAGACT	A	G	0.14	0.53	0.47	0.44	0.59	0.14	0.02	0.43	0.36	0.51	0.49	0.062	0.202	0.008	0.071	0.0001	1		
rs1989154	5	147.88	CACAGTGTGCACAAGGATTGATGTTTAAACAAAGTTAAGATCACAGTGGTACAGC[A/G]TTAGAGTGAGCTGATGACTATAAAAAAAGCTAACATTTTGAAGTTAGTTTATAAAAGG	A	G	0.64	0.38	0.42	0.51	0.38	0.86	0.94	0.45	0.50	0.43	0.35	0.038	0.198	0.004	0.141	0.0014			
rs783779	5	148.76	GTGACATAACAGGAAACCGAAAAGAGATGACTATCTTTAGCCAGTGGTGTTTTT[C/G]AACTACTACAGTTTTTTTCTGCCCAGTATACCTAGCAAGGAGTGGAGGAGGGATGAG	C	G	0.36	0.55	0.51	0.50	0.57	0.64	0.74	0.66	0.43	0.45	0.49	0.007	0.049	0.004	0.013	0.0000	1		
rs887346	5	149.62	CCTCATATTACAATGACACTGAACCTGTGGGCCCCATATGTGAGAACTGAGCTGG[A/C]TGCTGCAGATTCACCAGGGAGTTTGCTAAACACACAGATGTTTTGGGCCCCACCC	A	C	0.62	0.41	0.35	0.37	0.30	0.50	0.53	0.50	0.39	0.35	0.32	0.034	0.015	0.008	0.006	0.0002	1		
rs2053053	5	149.64	CTTAACAGCCTATTTCCATGCTTTTATGCAACACTCAGGTATATACATGCACAC[A/G]CACCTGCGCACCCTCCTTGTGCACAGTTTTAAACGTGCACACATGTGTACAAGTGCAT	A	G	0.21	0.41	0.35	0.34	0.32	0.27	0.55	0.32	0.36	0.33	0.32	0.008	0.023	0.004	0.005	0.0005			
rs1560657	5	150.02	GACTCATCTGAAGTTACCCACTATTCTGAGGCTGAACTGAAGCCTT[A/G]TCTCTCCTGAGACCACCCAGTGTGCCCAGCTGGAAGTTCTTCCCAACCTCTCACCACTACCTCGT	A	G	0.60	0.63	0.61	0.59	0.55	0.64	0.76	0.74	0.61	0.64	0.66	0.000	0.015	0.010	0.001	0.0029			
rs906678	5	150.81	TGGTTTTCAAATATTTCTTATCCTCTGAACCTTTCGAATTAAATCACAAAAAGAAC[A/G]AAGTATTTAAAATAAATCAGGGTTATTCTTACATAGCTCAGAAAAAATACACACATCCC	A	G	0.45	0.61	0.60	0.54	0.55	0.73	0.95	0.47	0.54	0.61	0.67	0.010	0.120	0.004	0.013	0.0023	1		
rs357608	5	150.87	TAGGTGTAGAAGTACTTCCATGTATTTTGTTCCTTGGCAAACTAGATAAAAGGGT[A/G]GGGCAAACCAGAAATATAAAAGGAACCCAGCTCAAAAACTTGTCTCTAACACTTCCG	A	G	0.31	0.55	0.53	0.57	0.55	0.36	0.16	0.74	0.71	0.52	0.51	0.030	0.105	0.024	0.020	0.0010			
rs2016865	5	151.77	TGAGTTCCAAGTTGTGTTTTATATTGGTGTAGGGGGCATTTTTTCATGTTTCTACA[A/G]AGAGTGTTTAAAGGTCAGTTTGAAAAATTGTCTGACTCTTAGAATATTAAAGGGTGA	A	G	0.64	0.44	0.49	0.49	0.38	0.05	0.52	0.51	0.39	0.48	0.42	0.001	0.005	0.002	0.207	0.0041	1		1
rs1438952	5	152.50	GGGCACCAGGAAGGAACTGGGTGTCCCAAGAGAAAATCTGGTGACAGCTTCTCCAC[A/C]GGGTAATCTTTCTAGGCAAACTCAGTAGTGAGCTGAGTTTACTAATACTAATACA	A	C	0.83	0.53	0.59	0.56	0.57	0.59	0.21	0.67	0.64	0.62	0.60	0.034	0.085	0.000	0.000	0.0003	1		
rs1461224	5	153.21	CCTTCTTTCTCTTTTCTGCCCCTCTTC[A/C]TCTCGTTCTTTCATCCATGAAGAGTGAGGAAGATGTTTTTAAGCACTGTTGAGCCACATGAGAAGATGACCCTGAACGAGTTTAG	A	C	0.64	0.60	0.51	0.44	0.50	0.91	0.79	0.78	0.54	0.57	0.58	0.019	0.039	0.049	0.097	0.0054	1		1
rs444984	5	154.48	AGGACTCCCACAGGATTAACGGTCATCACCCAAGAGAGTTGCTATCCACTGTCAC[A/C]CATGGCAGAGTGCAGGGTCCTGATGGAGTGGGCGCGGGAGGAGGCAACATAGAG	A	C	0.26	0.55	0.51	0.56	0.64	0.32	0.38	0.47	0.46	0.56	0.55	0.035	0.004	0.000	0.011	0.0009	1		
rs716651	5	155.12	TAGGAGTCCTGTTTTTGTTTTTGGAGGATTAGCTACAGTCATGTAAAGCATCCTTAA[A/G]CTAAAAGTAGCTTTTCAAGACTTCGGATGAAGAACTTTAGGGCAGAATTTAGAAGA	A	G	0.43	0.49	0.38	0.49	0.38	0.55	0.04	0.32	0.42	0.45	0.34	0.000	0.120	0.003	0.007	0.0028	1		
rs1432812	5	155.26	GTGTAAGAAGTCCACATACCCTGGGGCTATTCTACAGAGAGGAAGCTTAAACC[A/G]GCCACTTGGAAAGGCGAAAGAGAGATAGAGAAGACGGTCCAGCCAGTCCCACCTGT	A	G	0.95	0.83	0.76	0.78	0.75	0.86	0.27	0.93	0.71	0.83	0.73	0.031	0.125	0.026	0.007	0.0021			
rs728693	5	156.77	AATTTTAAAATAGTATATGCTAGTTTTAGAAAAACTGGGAAATACCAGAAAGTGC[A/G]AAGGAAAAAGTAAGAAATCTCTTGCCCATTTTACTTTTCCATTTTTTGTCTTTTTCTGCC	A	G	0.74	0.55	0.58	0.66	0.57	0.23	0.17	0.29	0.29	0.55	0.55	0.008	0.103	0.048	0.083	0.0010	1		
rs1039322	5	157.17	AGAGCAAGGTACTGACTGGTCTAAAGCCACTCTCCCCGGACACTGCTAACTCT[A/G]GCTAACCATCTCACAGACATCCTGCCTCTGATCATAAGATGGTCCCTGGAAGGAAGC	A	G	0.86	0.66	0.63	0.78	0.68	0.55	0.64	0.72	0.68	0.70	0.70	0.037	0.000	0.000	0.007	0.0049			1
rs949602	5	157.37	GATGAAGATGAGGGTTTGAGAGAGGATCCTTCTCACAGCAACATAAAGCAAAG[A/C]ATTACAAGTCCTCAAAAGGGTGGAATTTTGAGCTGCATTTCTGTTCTTTGAATGGGTGC	A	C	0.50	0.43	0.43	0.40	0.27	0.73	0.21	0.40	0.50	0.38	0.37	0.004	0.027	0.002	0.051	0.0006	1		
rs270664	5	158.54	AAATCAAGCTCAAGGCCCTCGTACTCAATGCTTCTCTCTGTCTGCCACCCTGC[A/G]TACTCTTTATTGCAATATGAAATTACTTTTTTGGGGTTTACTTTTTGTCTATCTTTCCCAT	A	G	0.45	0.45	0.52	0.49	0.46	0.73	0.38	0.50	0.61	0.48	0.49	0.014	0.037	0.010	0.005	0.0043	1		1
rs9216	5	159.76	TTTTGGATGAATAAAAAGGTTTGGATTTAATGAAGGGGAAAAAAAGAGGC[A/G]GAGGAGGTGAGCCTGAGGCTAGAACCGCTCACTCGACCCCCCACCCCCAG	A	G	0.57	0.48	0.45	0.46	0.66	0.68	0.51	0.69	0.50	0.46	0.42	0.006	0.004	0.042	0.028	0.0020	1		
[illegible]																								

rs910563	6	88.05	[illegible]	[illegible]	[illegible]	[illegible]	[illegible]	[illegible]	[illegible]	[illegible]	[illegible]	[illegible]	[illegible]	[illegible]	[illegible]	[illegible]	[illegible]	[illegible]	[illegible]	[illegible]	[illegible]			
rs2223877	6	88.14	[illegible]	[illegible]	[illegible]	[illegible]	[illegible]	[illegible]	[illegible]	[illegible]	[illegible]	[illegible]	[illegible]	[illegible]	[illegible]	[illegible]	[illegible]	[illegible]	[illegible]	[illegible]	[illegible]			
rs1051131	6	88.22	[illegible]	[illegible]	[illegible]	[illegible]	[illegible]	[illegible]	[illegible]	[illegible]	[illegible]	[illegible]	[illegible]	[illegible]	[illegible]	[illegible]	[illegible]	[illegible]	[illegible]	[illegible]	[illegible]			
rs632385	6	88.55	[illegible]	[illegible]	[illegible]	[illegible]	[illegible]	[illegible]	[illegible]	[illegible]	[illegible]	[illegible]	[illegible]	[illegible]	[illegible]	[illegible]	[illegible]	[illegible]	[illegible]	[illegible]	[illegible]			
rs1884831	6	88.89	[illegible]	[illegible]	[illegible]	[illegible]	[illegible]	[illegible]	[illegible]	[illegible]	[illegible]	[illegible]	[illegible]	[illegible]	[illegible]	[illegible]	[illegible]	[illegible]	[illegible]	[illegible]	[illegible]			
rs2610715	6	89.26	[illegible]	[illegible]	[illegible]	[illegible]	[illegible]	[illegible]	[illegible]	[illegible]	[illegible]	[illegible]	[illegible]	[illegible]	[illegible]	[illegible]	[illegible]	[illegible]	[illegible]	[illegible]	[illegible]			
rs2248529	6	89.28	[illegible]	[illegible]	[illegible]	[illegible]	[illegible]	[illegible]	[illegible]	[illegible]	[illegible]	[illegible]	[illegible]	[illegible]	[illegible]	[illegible]	[illegible]	[illegible]	[illegible]	[illegible]	[illegible]			
rs1040676	6	89.52	[illegible]	[illegible]	[illegible]	[illegible]	[illegible]	[illegible]	[illegible]	[illegible]	[illegible]	[illegible]	[illegible]	[illegible]	[illegible]	[illegible]	[illegible]	[illegible]	[illegible]	[illegible]	[illegible]			
rs205224	6	89.74	[illegible]	[illegible]	[illegible]	[illegible]	[illegible]	[illegible]	[illegible]	[illegible]	[illegible]	[illegible]	[illegible]	[illegible]	[illegible]	[illegible]	[illegible]	[illegible]	[illegible]	[illegible]	[illegible]			
rs1980986	6	89.76	[illegible]	[illegible]	[illegible]	[illegible]	[illegible]	[illegible]	[illegible]	[illegible]	[illegible]	[illegible]	[illegible]	[illegible]	[illegible]	[illegible]	[illegible]	[illegible]	[illegible]	[illegible]	[illegible]			
rs7773548	6	89.82	[illegible]	[illegible]	[illegible]	[illegible]	[illegible]	[illegible]	[illegible]	[illegible]	[illegible]	[illegible]	[illegible]	[illegible]	[illegible]	[illegible]	[illegible]	[illegible]	[illegible]	[illegible]	[illegible]			
rs6934871	6	90.20	[illegible]	[illegible]	[illegible]	[illegible]	[illegible]	[illegible]	[illegible]	[illegible]	[illegible]	[illegible]	[illegible]	[illegible]	[illegible]	[illegible]	[illegible]	[illegible]	[illegible]	[illegible]	[illegible]			
rs1179900	6	90.32	[illegible]	[illegible]	[illegible]	[illegible]	[illegible]	[illegible]	[illegible]	[illegible]	[illegible]	[illegible]	[illegible]	[illegible]	[illegible]	[illegible]	[illegible]	[illegible]	[illegible]	[illegible]	[illegible]			
rs1055403	6	90.63	[illegible]	[illegible]	[illegible]	[illegible]	[illegible]	[illegible]	[illegible]	[illegible]	[illegible]	[illegible]	[illegible]	[illegible]	[illegible]	[illegible]	[illegible]	[illegible]	[illegible]	[illegible]	[illegible]			
rs285619	6	91.05	[illegible]	[illegible]	[illegible]	[illegible]	[illegible]	[illegible]	[illegible]	[illegible]	[illegible]	[illegible]	[illegible]	[illegible]	[illegible]	[illegible]	[illegible]	[illegible]	[illegible]	[illegible]	[illegible]			
rs790605	6	91.10	[illegible]	[illegible]	[illegible]	[illegible]	[illegible]	[illegible]	[illegible]	[illegible]	[illegible]	[illegible]	[illegible]	[illegible]	[illegible]	[illegible]	[illegible]	[illegible]	[illegible]	[illegible]	[illegible]			
rs1145739	6	91.41	[illegible]	[illegible]	[illegible]	[illegible]	[illegible]	[illegible]	[illegible]	[illegible]	[illegible]	[illegible]	[illegible]	[illegible]	[illegible]	[illegible]	[illegible]	[illegible]	[illegible]	[illegible]	[illegible]			
rs1145774	6	91.65	[illegible]	[illegible]	[illegible]	[illegible]	[illegible]	[illegible]	[illegible]	[illegible]	[illegible]	[illegible]	[illegible]	[illegible]	[illegible]	[illegible]	[illegible]	[illegible]	[illegible]	[illegible]	[illegible]			
rs1884260	6	91.98	[illegible]	[illegible]	[illegible]	[illegible]	[illegible]	[illegible]	[illegible]	[illegible]	[illegible]	[illegible]	[illegible]	[illegible]	[illegible]	[illegible]	[illegible]	[illegible]	[illegible]	[illegible]	[illegible]			
rs2144363	6	92.04	[illegible]	[illegible]	[illegible]	[illegible]	[illegible]	[illegible]	[illegible]	[illegible]	[illegible]	[illegible]	[illegible]	[illegible]	[illegible]	[illegible]	[illegible]	[illegible]	[illegible]	[illegible]	[illegible]			
rs6454855	6	92.07	[illegible]	[illegible]	[illegible]	[illegible]	[illegible]	[illegible]	[illegible]	[illegible]	[illegible]	[illegible]	[illegible]	[illegible]	[illegible]	[illegible]	[illegible]	[illegible]	[illegible]	[illegible]	[illegible]			
rs1979797	6	92.49	[illegible]	[illegible]	[illegible]	[illegible]	[illegible]	[illegible]	[illegible]	[illegible]	[illegible]	[illegible]	[illegible]	[illegible]	[illegible]	[illegible]	[illegible]	[illegible]	[illegible]	[illegible]	[illegible]			
rs1391503	6	92.54	[illegible]	[illegible]	[illegible]	[illegible]	[illegible]	[illegible]	[illegible]	[illegible]	[illegible]	[illegible]	[illegible]	[illegible]	[illegible]	[illegible]	[illegible]	[illegible]	[illegible]	[illegible]	[illegible]			
rs1496252	6	92.65	[illegible]	[illegible]	[illegible]	[illegible]	[illegible]	[illegible]	[illegible]	[illegible]	[illegible]	[illegible]	[illegible]	[illegible]	[illegible]	[illegible]	[illegible]	[illegible]	[illegible]	[illegible]	[illegible]			
rs1373376	6	92.89	[illegible]	[illegible]	[illegible]	[illegible]	[illegible]	[illegible]	[illegible]	[illegible]	[illegible]	[illegible]	[illegible]	[illegible]	[illegible]	[illegible]	[illegible]	[illegible]	[illegible]	[illegible]	[illegible]			
rs2555778	6	93.17	[illegible]	[illegible]	[illegible]	[illegible]	[illegible]	[illegible]	[illegible]	[illegible]	[illegible]	[illegible]	[illegible]	[illegible]	[illegible]	[illegible]	[illegible]	[illegible]	[illegible]	[illegible]	[illegible]			
rs1427123	6	93.23	[illegible]	[illegible]	[illegible]	[illegible]	[illegible]	[illegible]	[illegible]	[illegible]	[illegible]	[illegible]	[illegible]	[illegible]	[illegible]	[illegible]	[illegible]	[illegible]	[illegible]	[illegible]	[illegible]			
rs1040155	6	93.56	[illegible]	[illegible]	[illegible]	[illegible]	[illegible]	[illegible]	[illegible]	[illegible]	[illegible]	[illegible]	[illegible]	[illegible]	[illegible]	[illegible]	[illegible]	[illegible]	[illegible]	[illegible]	[illegible]			
rs1338248	6	93.58	[illegible]	[illegible]	[illegible]	[illegible]	[illegible]	[illegible]	[illegible]	[illegible]	[illegible]	[illegible]	[illegible]	[illegible]	[illegible]	[illegible]	[illegible]	[illegible]	[illegible]	[illegible]	[illegible]			
rs1324103	6	93.90	[illegible]	[illegible]	[illegible]	[illegible]	[illegible]	[illegible]	[illegible]	[illegible]	[illegible]	[illegible]	[illegible]	[illegible]	[illegible]	[illegible]	[illegible]	[illegible]	[illegible]	[illegible]	[illegible]			
rs1548297	6	93.98	[illegible]	[illegible]	[illegible]	[illegible]	[illegible]	[illegible]	[illegible]	[illegible]	[illegible]	[illegible]	[illegible]	[illegible]	[illegible]	[illegible]	[illegible]	[illegible]	[illegible]	[illegible]	[illegible]			
rs491112	6	94.17	[illegible]	[illegible]	[illegible]	[illegible]	[illegible]	[illegible]	[illegible]	[illegible]	[illegible]	[illegible]	[illegible]	[illegible]	[illegible]	[illegible]	[illegible]	[illegible]	[illegible]	[illegible]	[illegible]			
rs1873219	6	94.28	[illegible]	[illegible]	[illegible]	[illegible]	[illegible]	[illegible]	[illegible]	[illegible]	[illegible]	[illegible]	[illegible]	[illegible]	[illegible]	[illegible]	[illegible]	[illegible]	[illegible]	[illegible]	[illegible]			
rs1488318	6	94.68	[illegible]	[illegible]	[illegible]	[illegible]	[illegible]	[illegible]	[illegible]	[illegible]	[illegible]	[illegible]	[illegible]	[illegible]	[illegible]	[illegible]	[illegible]	[illegible]	[illegible]	[illegible]	[illegible]			
rs4302647	6	94.76	[illegible]	[illegible]	[illegible]	[illegible]	[illegible]	[illegible]	[illegible]	[illegible]	[illegible]	[illegible]	[illegible]	[illegible]	[illegible]	[illegible]	[illegible]	[illegible]	[illegible]	[illegible]	[illegible]			
rs952577	6	94.98	[illegible]	[illegible]	[illegible]	[illegible]	[illegible]	[illegible]	[illegible]	[illegible]	[illegible]	[illegible]	[illegible]	[illegible]	[illegible]	[illegible]	[illegible]	[illegible]	[illegible]	[illegible]	[illegible]			
rs2493964	6	95.37	[illegible]	[illegible]	[illegible]	[illegible]	[illegible]	[illegible]	[illegible]	[illegible]	[illegible]	[illegible]	[illegible]	[illegible]	[illegible]	[illegible]	[illegible]	[illegible]	[illegible]	[illegible]	[illegible]			
rs169125	6	95.56	[illegible]	[illegible]	[illegible]	[illegible]	[illegible]	[illegible]	[illegible]	[illegible]	[illegible]	[illegible]	[illegible]	[illegible]	[illegible]	[illegible]	[illegible]	[illegible]	[illegible]	[illegible]	[illegible]			
rs2890370	6	95.88	[illegible]	[illegible]	[illegible]	[illegible]	[illegible]	[illegible]	[illegible]	[illegible]	[illegible]	[illegible]	[illegible]	[illegible]	[illegible]	[illegible]	[illegible]	[illegible]	[illegible]	[illegible]	[illegible]			
rs2716091	6	95.90	[illegible]	[illegible]	[illegible]	[illegible]	[illegible]	[illegible]	[illegible]	[illegible]	[illegible]	[illegible]	[illegible]	[illegible]	[illegible]	[illegible]	[illegible]	[illegible]	[illegible]	[illegible]	[illegible]			
rs1133503	6	96.10	[illegible]	[illegible]	[illegible]	[illegible]	[illegible]	[illegible]	[illegible]	[illegible]	[illegible]	[illegible]	[illegible]	[illegible]	[illegible]	[illegible]	[illegible]	[illegible]	[illegible]	[illegible]	[illegible]			
rs4839952	6	96.17	[illegible]	[illegible]	[illegible]	[illegible]	[illegible]	[illegible]	[illegible]	[illegible]	[illegible]	[illegible]	[illegible]	[illegible]	[illegible]	[illegible]	[illegible]	[illegible]	[illegible]	[illegible]	[illegible]			
rs716192	6	96.56	[illegible]	[illegible]	[illegible]	[illegible]	[illegible]	[illegible]	[illegible]	[illegible]	[illegible]	[illegible]	[illegible]	[illegible]	[illegible]	[illegible]	[illegible]	[illegible]	[illegible]	[illegible]	[illegible]			
rs7772067	6	96.62	[illegible]	[illegible]	[illegible]	[illegible]	[illegible]	[illegible]	[illegible]	[illegible]	[illegible]	[illegible]	[illegible]	[illegible]	[illegible]	[illegible]	[illegible]	[illegible]	[illegible]	[illegible]	[illegible]			
rs3827786	6	96.71	[illegible]	[illegible]	[illegible]	[illegible]	[illegible]	[illegible]	[illegible]	[illegible]	[illegible]	[illegible]	[illegible]	[illegible]	[illegible]	[illegible]	[illegible]	[illegible]	[illegible]	[illegible]	[illegible]			
rs2472922	6	96.88	[illegible]	[illegible]	[illegible]	[illegible]	[illegible]	[illegible]	[illegible]	[illegible]	[illegible]	[illegible]	[illegible]	[illegible]	[illegible]	[illegible]	[illegible]	[illegible]	[illegible]	[illegible]	[illegible]			
rs521608	6	97.20	[illegible]	[illegible]	[illegible]	[illegible]	[illegible]	[illegible]	[illegible]	[illegible]	[illegible]	[illegible]	[illegible]	[illegible]	[illegible]	[illegible]	[illegible]	[illegible]	[illegible]	[illegible]	[illegible]			
rs2095348	6	97.21	[illegible]	[illegible]	[illegible]	[illegible]	[illegible]	[illegible]	[illegible]	[illegible]	[illegible]	[illegible]	[illegible]	[illegible]	[illegible]	[illegible]	[illegible]	[illegible]	[illegible]	[illegible]	[illegible]			
rs1206147	6	97.61	[illegible]	[illegible]	[illegible]	[illegible]	[illegible]	[illegible]	[illegible]	[illegible]	[illegible]	[illegible]	[illegible]	[illegible]	[illegible]	[illegible]	[illegible]	[illegible]	[illegible]	[illegible]	[illegible]			
rs1206126	6	97.66	[illegible]	[illegible]	[illegible]	[illegible]	[illegible]	[illegible]	[illegible]	[illegible]	[illegible]	[illegible]	[illegible]	[illegible]	[illegible]	[illegible]	[illegible]	[illegible]	[illegible]	[illegible]	[illegible]			
rs876012	6	97.80	[illegible]	[illegible]	[illegible]	[illegible]	[illegible]	[illegible]	[illegible]	[illegible]	[illegible]	[illegible]	[illegible]	[illegible]	[illegible]	[illegible]	[illegible]	[illegible]	[illegible]	[illegible]	[illegible]			
rs6569342	6	98.66	[illegible]	[illegible]	[illegible]	[illegible]	[illegible]	[illegible]	[illegible]	[illegible]	[illegible]	[illegible]	[illegible]	[illegible]	[illegible]	[illegible]	[illegible]	[illegible]	[illegible]	[illegible]	[illegible]			
rs1494781	6	98.98	[illegible]	[illegible]	[illegible]	[illegible]	[illegible]	[illegible]	[illegible]	[illegible]	[illegible]	[illegible]	[illegible]	[illegible]	[illegible]	[illegible]	[illegible]	[illegible]	[illegible]	[illegible]	[illegible]			
rs1997936	6	99.48	[illegible]	[illegible]	[illegible]	[illegible]	[illegible]	[illegible]	[illegible]	[illegible]	[illegible]	[illegible]	[illegible]	[illegible]	[illegible]	[illegible]	[illegible]	[illegible]	[illegible]	[illegible]	[illegible]			
rs7776325	6	99.91	[illegible]	[illegible]	[illegible]	[illegible]	[illegible]	[illegible]	[illegible]	[illegible]	[illegible]	[illegible]	[illegible]	[illegible]	[illegible]	[illegible]	[illegible]	[illegible]	[illegible]	[illegible]	[illegible]			
rs2894891	6	100.26	[illegible]	[illegible]	[illegible]	[illegible]	[illegible]	[illegible]	[illegible]	[illegible]	[illegible]	[illegible]	[illegible]	[illegible]	[illegible]	[illegible]	[illegible]	[illegible]	[illegible]	[illegible]	[illegible]			
rs761713	6	100.65	[illegible]	[illegible]	[illegible]	[illegible]	[illegible]	[illegible]	[illegible]	[illegible]	[illegible]	[illegible]	[illegible]	[illegible]	[illegible]	[illegible]	[illegible]	[illegible]	[illegible]	[illegible]	[illegible]			
rs761711	6	100.67	[illegible]	[illegible]	[illegible]	[illegible]	[illegible]	[illegible]	[illegible]	[illegible]	[illegible]	[illegible]	[illegible]	[illegible]	[illegible]	[illegible]	[illegible]	[illegible]	[illegible]	[illegible]	[illegible]			
rs240153	6	101.11	[illegible]	[illegible]	[illegible]	[illegible]	[illegible]	[illegible]	[illegible]	[illegible]	[illegible]	[illegible]	[illegible]	[illegible]	[illegible]	[illegible]	[illegible]	[illegible]	[illegible]	[illegible]	[illegible]			
rs239229	6	101.15	[illegible]	[illegible]	[illegible]	[illegible]	[illegible]	[illegible]	[illegible]	[illegible]	[illegible]	[illegible]	[illegible]	[illegible]	[illegible]	[illegible]	[illegible]	[illegible]	[illegible]	[illegible]	[illegible]			
rs239189	6	101.17	[illegible]	[illegible]	[illegible]	[illegible]	[illegible]	[illegible]	[illegible]	[illegible]	[illegible]	[illegible]	[illegible]	[illegible]	[illegible]	[illegible]	[illegible]	[illegible]	[illegible]	[illegible]	[illegible]			
rs846773	6	101.34	[illegible]	[illegible]	[illegible]	[illegible]	[illegible]	[illegible]	[illegible]	[illegible]	[illegible]	[illegible]	[illegible]	[illegible]	[illegible]	[illegible]	[illegible]	[illegible]	[illegible]	[illegible]	[illegible]			
rs2207958	6	102.03	[illegible]	[illegible]	[illegible]	[illegible]	[illegible]	[illegible]	[illegible]	[illegible]	[illegible]	[illegible]	[illegible]	[illegible]	[illegible]	[illegible]	[illegible]	[illegible]	[illegible]	[illegible]	[illegible]			
rs1953817	6	103.09	[illegible]	[illegible]	[illegible]	[illegible]	[illegible]	[illegible]	[illegible]	[illegible]	[illegible]	[illegible]	[illegible]	[illegible]	[illegible]	[illegible]	[illegible]	[illegible]	[illegible]	[illegible]	[illegible]			
rs1431218	6	103.37	[illegible]	[illegible]	[illegible]	[illegible]	[illegible]	[illegible]	[illegible]	[illegible]	[illegible]	[illegible]	[illegible]	[illegible]	[illegible]	[illegible]	[illegible]	[illegible]	[illegible]	[illegible]	[illegible]			
rs500204	6	103.67	[illegible]	[illegible]	[illegible]	[illegible]	[illegible]	[illegible]	[illegible]	[illegible]	[illegible]	[illegible]	[illegible]	[illegible]	[illegible]	[illegible]	[illegible]	[illegible]	[illegible]	[illegible]	[illegible]			
rs1155126	6	103.85	[illegible]	[illegible]	[illegible]	[illegible]	[illegible]	[illegible]	[illegible]	[illegible]	[illegible]	[illegible]	[illegible]	[illegible]	[illegible]	[illegible]	[illegible]	[illegible]	[illegible]	[illegible]	[illegible]			
rs6929015	6	104.15	[illegible]	[illegible]	[illegible]	[illegible]	[illegible]	[illegible]	[illegible]	[illegible]	[illegible]	[illegible]	[illegible]	[illegible]	[illegible]	[illegible]	[illegible]	[illegible]	[illegible]	[illegible]	[illegible]			
rs860933	6	104.55	[illegible]	[illegible]	[illegible]	[illegible]	[illegible]	[illegible]	[illegible]	[illegible]	[illegible]	[illegible]	[illegible]	[illegible]	[illegible]	[illegible]	[illegible]	[illegible]	[illegible]	[illegible]	[illegible]			
rs201204	6	104.78	[illegible]	[illegible]	[illegible]	[illegible]	[illegible]	[illegible]	[illegible]	[illegible]	[illegible]	[illegible]	[illegible]	[illegible]	[illegible]	[illegible]	[illegible]	[illegible]	[illegible]	[illegible]	[illegible]			
rs880177	6	105.90	[illegible]	[illegible]	[illegible]	[illegible]	[illegible]	[illegible]	[illegible]	[illegible]	[illegible]	[illegible]	[illegible]	[illegible]	[illegible]	[illegible]	[illegible]	[illegible]	[illegible]	[illegible]	[illegible]			
rs2040431	6	106.12	[illegible]	[illegible]	[illegible]	[illegible]	[illegible]	[illegible]	[illegible]	[illegible]	[illegible]	[illegible]	[illegible]	[illegible]	[illegible]	[illegible]	[illegible]	[illegible]	[illegible]	[illegible]	[illegible]			
rs1475270	6	106.73	[illegible]	[illegible]	[illegible]	[illegible]	[illegible]	[illegible]	[illegible]	[illegible]	[illegible]	[illegible]	[illegible]	[illegible]	[illegible]	[illegible]	[illegible]	[illegible]	[illegible]	[illegible]	[illegible]			
rs2050042	6	108.35	[illegible]	[illegible]	[illegible]	[illegible]	[illegible]	[illegible]	[illegible]	[illegible]	[illegible]	[illegible]	[illegible]	[illegible]	[illegible]	[illegible]	[illegible]	[illegible]	[illegible]	[illegible]	[illegible]			
rs217532	6	108.57	[illegible]	[illegible]	[illegible]	[illegible]	[illegible]	[illegible]	[illegible]	[illegible]	[illegible]	[illegible]	[illegible]	[illegible]	[illegible]	[illegible]	[illegible]	[illegible]	[illegible]	[illegible]	[illegible]			
rs1260658	6	109.47	[illegible]	[illegible]	[illegible]	[illegible]	[illegible]	[illegible]	[illegible]	[illegible]	[illegible]	[illegible]	[illegible]	[illegible]	[illegible]	[illegible]	[illegible]	[illegible]	[illegible]	[illegible]	[illegible]			
rs351755	6	109.58	[illegible]	[illegible]	[illegible]	[illegible]	[illegible]	[illegible]	[illegible]	[illegible]	[illegible]	[illegible]	[illegible]	[illegible]	[illegible]	[illegible]	[illegible]	[illegible]	[illegible]	[illegible]	[illegible]			
rs736830	6	109.76	[illegible]	[illegible]	[illegible]	[illegible]	[illegible]	[illegible]	[illegible]	[illegible]	[illegible]	[illegible]	[illegible]	[illegible]	[illegible]	[illegible]	[illegible]	[illegible]	[illegible]	[illegible]	[illegible]			
rs1046943	6	109.83	[illegible]	[illegible]	[illegible]	[illegible]	[illegible]	[illegible]	[illegible]	[illegible]	[illegible]	[illegible]	[illegible]	[illegible]	[illegible]	[illegible]	[illegible]	[illegible]	[illegible]	[illegible]	[illegible]			
rs781499	6	111.31	[illegible]	[illegible]	[illegible]	[illegible]	[illegible]	[illegible]	[illegible]	[illegible]	[illegible]	[illegible]	[illegible]	[illegible]	[illegible]	[illegible]	[illegible]	[illegible]	[illegible]	[illegible]	[illegible]			
rs1022092	6	111.47	[illegible]	[illegible]	[illegible]	[illegible]	[illegible]	[illegible]	[illegible]	[illegible]	[illegible]	[illegible]	[illegible]	[illegible]	[illegible]	[illegible]	[illegible]	[illegible]	[illegible]	[illegible]	[illegible]			
rs1158747	6	112.55	[illegible]	[illegible]	[illegible]	[illegible]	[illegible]	[illegible]	[illegible]	[illegible]	[illegible]	[illegible]	[illegible]	[illegible]	[illegible]	[illegible]	[illegible]	[illegible]	[illegible]	[illegible]	[illegible]			
rs1994672	6	113.71	[illegible]	[illegible]	[illegible]	[illegible]	[illegible]	[illegible]	[illegible]	[illegible]	[illegible]	[illegible]	[illegible]	[illegible]	[illegible]	[illegible]	[illegible]	[illegible]	[illegible]	[illegible]	[illegible]			
rs1565528	6	113.72	[illegible]	[illegible]	[illegible]	[illegible]	[illegible]	[illegible]	[illegible]	[illegible]	[illegible]	[illegible]	[illegible]	[illegible]	[illegible]	[illegible]	[illegible]	[illegible]	[illegible]	[illegible]	[illegible]			
rs1491074	6	113.73	[illegible]	[illegible]	[illegible]	[illegible]	[illegible]	[illegible]	[illegible]	[illegible]	[illegible]	[illegible]	[illegible]	[illegible]	[illegible]	[illegible]	[illegible]	[illegible]	[illegible]	[illegible]	[illegible]			
rs1033391	6	113.81	[illegible]	[illegible]	[illegible]	[illegible]	[illegible]	[illegible]	[illegible]	[illegible]	[illegible]	[illegible]	[illegible]	[illegible]	[illegible]	[illegible]	[illegible]	[illegible]	[illegible]	[illegible]	[illegible]			
rs773676	6	114.09	[illegible]	[illegible]	[illegible]	[illegible]	[illegible]	[illegible]	[illegible]	[illegible]	[illegible]	[illegible]	[illegible]	[illegible]	[illegible]	[illegible]	[illegible]	[illegible]	[illegible]	[illegible]	[illegible]			
rs2030926	6	114.11	[illegible]	[illegible]	[illegible]	[illegible]	[illegible]	[illegible]	[illegible]	[illegible]	[illegible]	[illegible]	[illegible]	[illegible]	[illegible]	[illegible]	[illegible]	[illegible]	[illegible]	[illegible]	[illegible]			
rs963884	6	114.18	[illegible]	[illegible]	[illegible]	[illegible]	[illegible]	[illegible]	[illegible]	[illegible]	[illegible]	[illegible]	[illegible]	[illegible]	[illegible]	[illegible]	[illegible]	[illegible]	[illegible]	[illegible]	[illegible]			
rs548388	6	114.28	GACATAAAAAAGATCCAAACAACTTCAACAGATGAAAAATACAATGTCTAAGATAA[A/G]ATAATAATGGATAGGACTAAAATCAAATTAAATACTGCAGAAGAAAAGATTAGTGA	A	G	0.43	0.70	0.69	0.65	0.73	0.64	0.96	0.64	0.64	0.64	0.58	0.047	0.069	0.010	0.002	0.0073			
rs1415428	6	114.74	GTTTGTCACTCATGAAAGCCTGATTACAAGTAGAGAGATACTTGGTAATGACTGTTCTCCATGTGAT[A/G]TCTCAGCTGATGCAAACCAGTCTGCTTGAATATTGTGTATCTAC	A	G	0.60	0.41	0.35	0.44	0.41	0.18	0.35	0.40	0.36	0.41	0.28	0.030	0.001	0.005	0.014	0.0029	1		
rs2032558	6	115.82	AAAAAATAAGTGATGAGTCTTTATCTGTTGTCCCAGGGCAGAAATTCTCAAATCTT[A/T]GTCAAAGTAAGTGTGCCAAATAATCAACTGGTTCACTTATTGTAGGCCAGTAACAA	A	T	0.60	0.54	0.55	0.51	0.48	0.68	0.67	0.54	0.43	0.57	0.53	0.000	0.000	0.005	0.001	0.0007	1		
rs1041883	6	116.73	CAGCTAAGGTGAGAGTGGGGATACCTTTTCTAATTCTTACAAAAGTGCCAC[A/G]TACCCATGTACACAGGCTGCTACCCTGGGACAACAGACAAAAATAGGACAAGGTAACA	A	G	0.29	0.57	0.45	0.50	0.61	0.91	0.99	0.71	0.75	0.57	0.45	0.011	0.259	0.039	0.143	0.0000	1		
rs2040405	6	116.86	ATAATATAAAATAAAAATATTATATAACCCTGTAGTTATGCTGTGTAGTTATGCTGTGTAACTTCCTCAAG[C/G]CCTTTATCAGAGAAACCCAGCTTTACACAATGGTGATGAAAA	C	G	0.60	0.66	0.69	0.56	0.59	0.77	0.66	0.43	0.68	0.60	0.57	0.009	0.008	0.047	0.000	0.0080		1	1
rs544047	6	117.65	CACAATTGCTCTAACAGCAACACCAGTGAAGCCATTCTGTGTATTCAGTTGCA[A/G]TATTCAGGAAATTTGCTAGATATAGAGAAGAACATTTGATTATAGCATTTGCTAAA	A	G	0.26	0.64	0.57	0.61	0.71	0.45	0.66	0.49	0.46	0.60	0.57	0.067	0.004	0.011	0.013	0.0001	1		
rs1321807	6	117.73	TGCTGCTCAGGTATAACTCTCTAGTATCTATTATCATTTGCTGTGCATGTGTTT[A/G]TCTCCCTCCTGTTTTATGAACTCCATCAGAGAAGCATCTCTTCTATTCCTTGTTAAAT	A	G	0.24	0.49	0.56	0.54	0.48	0.36	0.14	0.39	0.57	0.49	0.53	0.049	0.110	0.015	0.023	0.0002			
rs1535330	6	117.76	GCAAGCTTCTGAAAGGATTATTTT[C/G]CCAATGGAAGAGTGTGATTTGGTGTCAAATGTTGTATGAACAGGCACTCTTATCTTCAATGGTGTGTCACTAATAGAGG	C	G	0.64	0.59	0.65	0.61	0.55	0.36	0.14	0.54	0.59	0.54	0.59	0.062	0.155	0.024	0.047	0.0006			
rs1321803	6	117.77	ATGACACAGAGGGTTCTGAGCTGTCCAGTTTTCTCTT[A/T]CAATACCACTCTCTTACAGGCGGGGGGAGCCAATGCTCAGAGCATGTGTCTGTGGCACACACAGAACCATCCATT	A	T	0.93	0.47	0.43	0.52	0.50	0.64	0.60	0.73	0.61	0.43	0.44	0.142	0.010	0.034	0.015	0.0000			
rs210646	6	117.89	AAAATATTCCACACTTACTGTAATAGTTCTTTGGGAGCAATATATGGTGAACA[C/G]TGGCAATGCAGGCATCTGCTGTGATTAGTCAGAGCTGATTGGCTAAGAGGAAAATGAA	C	G	0.64	0.58	0.57	0.50	0.57	0.64	0.97	0.64	0.54	0.58	0.60	0.003	0.152	0.017	0.005	0.0000			
rs210617	6	117.91	CTTGGAGGTAGGTAGAGAAGCACCATGTAAACAATAAGCGTATTTTCAACT[A/G]CGAGTCATTTACAGTCATGAAACTACTCCTGCACTTGGCACTTGTGTTATTTTCTGCACC	A	G	0.67	0.58	0.56	0.45	0.48	0.68	0.64	0.31	0.39	0.52	0.56	0.008	0.006	0.009	0.017	0.0000			
rs1541317	6	118.07	ACCCACAGCTTTACTTGCCTGAGGTTGTAGCCCCAGTTAATACATGGCAAGA[A/C]ATTTAACAGTGAGATCCAGGAAATAAGGGCTCAGATAAACTCTCACATATCCATGGC	A	C	0.90	0.39	0.38	0.33	0.27	0.73	0.59	0.90	0.50	0.45	0.42	0.128	0.011	0.086	0.031	0.0000			
rs1012509	6	119.49	AGCAAATTCGTATTGCCAGGTAGACAGGATGCTTTGGTTTGCTGTGTTCTCA[A/G]GGGGTTGCTTGTTTATGAGATCATAAGGTTGGTTTATGTGTATGATTTTTATTTCTGT	A	G	0.36	0.27	0.30	0.34	0.23	0.59	0.26	0.52	0.43	0.31	0.29	0.002	0.001	0.024	0.048	0.0006	1		
rs2036256	6	119.82	GTCTTGTCTGTACTTAAGTCTTATTCAGTATCTCGGATTCAAACATAGATGTC[C/G]CCTTACAAAGCCTTCTGATTTGCCAATATTGGATTAGGTGTCTTGTCTATAAGATTCTAT	C	G	0.62	0.49	0.55	0.50	0.50	0.77	0.72	0.74	0.43	0.53	0.54	0.003	0.022	0.028	0.034	0.0002			
rs937091	6	120.18	CTCATCATCTCCACTGAAACCACCAGAGTCCAAGTGCAATAGTGTCATAACC[A/C]CTCTTGGCTCCCTATAGTCATCCCCTCATGCCACACACCATCATCCCCATCCCCATTA	A	C	0.60	0.51	0.44	0.49	0.57	0.59	0.31	0.28	0.57	0.53	0.48	0.011	0.012	0.013	0.012	0.0004	1		
rs1486198	6	120.79	AATTAAATACAGTTATCTTAAATTATTGGGGGTGAGGGTGGTAGGGAGGAGTACAAAAGTAGATAGACAAAT[A/G]TAAAGAAACTTCACTCATCCCATCCTTAAATTTAAAATATA	A	G	0.38	0.55	0.55	0.51	0.41	0.32	0.50	0.30	0.50	0.48	0.57	0.007	0.000	0.024	0.020	0.0012	1		
rs2063875	6	121.28	TAGACATTTCCTCAAGCATTCTTTTATAGGTGTTCCAGATAAAAGAGTTGGTGT[A/T]AGGCCGGGATTTAAAAGACTGAGAGAAAGAAAACCATGATAATTTCCCTTATGGCTTT	A	T	0.12	0.62	0.62	0.67	0.61	0.00	0.01	0.24	0.57	0.55	0.62	0.160	0.270	0.086	0.299	0.0000			
rs763075	6	122.19	TTGACCAAAGTCAGAAAGAATGGGAGGCTTTTTGTTGTACTACAAGACAGTCAGC[A/C]CTTTCTGCTCAATGTTTCTGCCTGGATGGAAAGCCTGTTGCTGCAACTAGAGAAG	A	C	0.12	0.52	0.44	0.41	0.43	0.25	0.93	0.52	0.61	0.41	0.40	0.060	0.147	0.010	0.017	0.0015	1		
rs1563512	6	122.46	GAAGTTCAAACTCATTGGCACTCAGCTACTCTGACAATTAAGCCCTGGTCCTC[A/G]TCCTAAGCTTACTCTTTCCCTCTCATTGCACATGATAATTGTCTCTTTACAAACTTCCAAC	A	G	0.43	0.38	0.27	0.30	0.29	0.36	0.91	0.50	0.46	0.30	0.24	0.012	0.227	0.015	0.002	0.0010			
rs873460	6	123.71	GGATATGGTTTTGGAATGGAAAAGAGCATAGAAGGAAGCCTACCTTTAGAGCT[A/G]TAAGGTGGACAGTAACAAAATAGAAGTGAGTGGAATCCATTCCTGTGACAAATGTGG	A	G	0.19	0.43	0.41	0.32	0.41	0.18	0.43	0.14	0.32	0.44	0.42	0.022	0.005	0.043	0.021	0.0005	1		
rs1321084	6	123.88	TGTGAACAAAATTATTCTGACTTTTTTTATAGATCTTTGTTCTTCAACAAGGC[A/C]CTAGGTAGGGATCAAGTCATCTATCCTTTCATAGCACTTAGCATAGCACATTTGCACAG	A	C	0.57	0.51	0.49	0.49	0.55	0.41	0.60	0.48	0.57	0.46	0.43	0.003	0.006	0.001	0.002	0.0010			
rs941815	6	123.90	AGTTTTACCTATTATGCATTCTGAACATTGGTTGAAGAAATAAATTTCTGAATT[A/G]TTTACATCTCATGGCTTTATTGCAATGGTAGTATGAATGAGTTTAGCATCAGTTGTGTA	A	G	0.60	0.42	0.40	0.33	0.45	0.45	0.17	0.49	0.39	0.39	0.42	0.019	0.026	0.007	0.004	0.0003			
rs1407529	6	124.67	AACAATATATTAGAGAAAATAAACGGGAAAACTGGAACTAATACATTGAAAGAGA[A/G]ATAATTTAGCCCCAGAGAACTGGAATTTATGTAAAGAATCAAATGGAAATTCTAAGA	A	G	0.60	0.55	0.52	0.56	0.55	0.55	0.60	0.52	0.61	0.59	0.62	0.004	0.006	0.000	0.002	0.0042	1		1
rs659491	6	124.81	AACATTTCTCTCTATCACAACTATGATATAAATCCCTGCAGCATTTTGTGTAT[A/G]TATTTGTATATATAGATGTACAGAGGGGCAGCTTTACACATGACTTTTTAATAGACATGTA	A	G	0.21	0.52	0.57	0.59	0.63	0.50	0.72	0.61	0.64	0.55	0.54	0.083	0.009	0.000	0.008	0.0005			
rs911966	6	125.37	AATGAACTGCTAAACCTACAATGTTTTATGACTAAATCATGTACATCTGCCCA[A/G]AAGTATTTCTCCAGTTTACCAAGAAATGAAGGCTTACTTTATTGTTTAAGCAGCAT	A	G	0.40	0.50	0.52	0.50	0.36	0.29	0.04	0.47	0.43	0.52	0.54	0.005	0.152	0.001	0.092	0.0008	1		
rs524394	6	125.51	TTCCTGAGATTGGTTTGTTCCCCAAAGTTATACCCTTTCCAATATCACTATACT[A/C]CTTGCAATCACGGATCATTAAGCAAAATCGGGATGAATTTGCTGGACAGAGCC	A	C	0.26	0.60	0.52	0.63	0.48	0.27	0.26	0.42	0.75	0.44	0.43	0.035	0.040	0.014	0.040	0.0032			
rs1569741	6	126.36	TATAGTTCATGTTGTGGAAAAATACCAATTTTGTTCTTAAGGCGTAGAGGGAGG[A/G]CACTTTTGAATCACTACAACTTTACACTTTGTTATTGTTCCAATTTTATTTACAGGTTT	A	G	0.75	0.55	0.42	0.41	0.54	0.95	0.98	0.54	0.50	0.35	0.48	0.096	0.312	0.025	0.257	0.0045	1		1
rs1340952	6	126.93	GAGAAATTATCTTTTTTATTTCTACATCTTTCATAACACACTTAACGGTTCATGAATGAAAAT[A/G]TTACTTAATCAAGCCCTGGGTGATAAATCACTTGTGCTCTGCTGGATGAA	A	G	0.40	0.51	0.54	0.52	0.48	1.00	0.94	0.66	0.43	0.46	0.48	0.008	0.143	0.006	0.202	0.0035	1		
rs1538956	6	126.94	ATTTTGATTTAATGTCTTTAACACTCTAGCACCTAATTCACAAGTTTTCTTTCTACTGTGATTTCTCCCGT[A/C]TGCTATTCTTGGTAAATTCAGCATTCCCATAAAAGGCTCATTT	A	C	0.26	0.51	0.54	0.54	0.48	1.00	0.94	0.66	0.43	0.46	0.47	0.038	0.142	0.006	0.202	0.0040			
rs1022573	6	127.04	GGTTAAACTGACACACGTTT[A/C]ATGATATGCGTAGTCATTGCTAAGTAAGTAGGCATAAGAACAAGGTAATTTAAATAAGTGATTATAATTGGTACAATATTTTGAAGTTTTTAAT	A	C	0.74	0.49	0.46	0.44	0.52	0.00	0.04	0.32	0.61	0.51	0.52	0.041	0.143	0.010	0.202	0.0041			1
rs988693	6	127.28	TCTGTGGTGCTATCTGTGAAATCCAATACTGTCCTGATTCCCTTGAATTGGAC[A/G]GTACGTACATGCTCTTTGGGCTTGTCCAAAGTTTTCAGGTTTTTGAAGGAACTGCTAT	A	G	0.52	0.56	0.50	0.48	0.61	0.14	0.64	0.60	0.54	0.47	0.50	0.000	0.012	0.000	0.085	0.0003			
rs1543432	6	127.68	TACCCACAAATTGATGGTCTCCAGGAACAGACCTGAACCAGTGACTCACTTGT[A/G]TGGGGTCACAACACAGCTGTCATCATGCCCTCAATAGAATCAGCCTTTTACACATGTG	A	G	0.24	0.23	0.25	0.16	0.27	0.27	0.20	0.38	0.25	0.23	0.21	0.005	0.006	0.003	0.002	0.0012	1		
rs482607	6	128.97	CAGCTCTGCTTCTTTCTTCTTCTAACTTAAGATTAACTGTTGTCTGCCTGA[A/G]ACAGAAAAAGTGCTGTCTGTAGCAAATGTGATTCTTGAAGGACTGACGGCTACACCTCCTGTT	A	G	0.60	0.28	0.36	0.35	0.39	0.55	0.59	0.43	0.36	0.27	0.40	0.019	0.023	0.000	0.015	0.0000	1		
rs1894641	6	130.00	GCATTTCAAGTTCTTATCAACACAGTAGTTCCTGCTTACAAAAGGCTCATAA[A/G]AGCAGGCTGCTTTGCTTCCACCTCTAAGAGACTGGGGACTTTTTGGTGGGTGTGGACC	A	G	0.29	0.13	0.18	0.16	0.13	0.36	0.17	0.08	0.11	0.22	0.24	0.005	0.001	0.014	0.023	0.0026	1		
rs2007339	6	131.33	TTACTGTGTGCTGGTGAATAGCTCTTGTGGTTGAT[A/G]TGGTGCTTTGGCTTGTGCTCCTCATTTCTGTCATAGAATGGCTTATGACATGCCATTCTACGACAGAGATGCTTAG	A	G	0.24	0.16	0.25	0.30	0.21	0.05	0.29	0.22	0.21	0.34	0.27	0.000	0.000	0.002	0.045	0.0002	1		
rs942150	6	131.68	AAAAAGCAAGCTGTCCTAATAGTTTCTTTATAAACATGTTTAGACCCAGATAAAAGGCTGAACCCTAAACCGGGTATAAAATGGCTGATTCCTTCGAG[A/C]CCTACAGGGTGT	A	C	0.14	0.41	0.52	0.45	0.41	0.77	0.67	0.58	0.39	0.46	0.46	0.080	0.010	0.001	0.025	0.0057			1
rs1033540	6	131.72	GGCTCATTAGAGTCCAAATGGGAAATGAGGAAAAATACCAACGCTGCTTGTC[A/G]GAGGTGATGGAGAGTGATATCCCCATTTGACTACAAGAAGCAATACTTTTGGCTGGGT	A	G	0.14	0.41	0.29	0.43	0.45	0.00	0.22	0.36	0.43	0.30	0.34	0.023	0.003	0.001	0.112	0.0011			
rs1409181	6	132.13	AGGAGTCCTGCTGTCAGTGGAGGTCGTTCTGGATTCCGAGTGGGATCGGAAA[C/G]TGATGTCCCGGGATTAGAGGATGTGTTTATGCTGGCTTTCAGGATGTATCCAAGGCAT	C	G	0.60	0.46	0.46	0.54	0.41	0.50	0.80	0.38	0.64	0.43	0.51	0.011	0.073	0.007	0.001	0.0026	1		
rs48385	6	133.29	ATTTTTTTTTTTTTTTTAATCAGGCCACTATACAGCTGCAGGCCACTGGTCA[A/G]CCTATAAAACAGCTTTCATTGGATTCCACACTTAGACTCCCTAGCCATGTCAGAGAATTCA	A	G	0.79	0.61	0.53	0.44	0.64	0.27	0.39	0.35	0.57	0.49	0.50	0.045	0.004	0.010	0.028	0.0008	1		
rs969282	6	134.19	GTGGCCCTCGGATGGCCCATCAGGTTTCTAC[A/G]GTAGAGATGAGATTTTTATTTTAACAAAGTCCTTCTGGCGATTCAGTTACTTAAACCATCTTAGACAGGGTTCTAAGCAT	A	G	0.81	0.61	0.52	0.52	0.52	0.55	0.53	0.71	0.61	0.58	0.45	0.075	0.000	0.020	0.002	0.0013	1		
rs229919	6	134.21	GTTGCTGCTTCTCTTCTGCTCTGGCTAACTTTTAGATAGCGAGAGTCTGAA[A/G]ATTTCTCTCCTGTGGGCTCAACAATAAGTTCTGCAGGCTCTTTTCCAGAGGGGAATGCA	A	G	0.08	0.28	0.27	0.28	0.23	0.00	0.01	0.09	0.29	0.26	0.23	0.029	0.089	0.023	0.112	0.0002			
rs162192	6	134.25	ACAGCTATCTTCTTACCAGTGAAGTTTATGCTTTTCCCCAAAATGAGGAAAGGC[A/C]GTCCTGGGAGTAATGTATCATCACTGGCTATATTATTAATCCTCATAATCAATTCCAC	A	C	0.76	0.38	0.45	0.49	0.41	0.86	0.91	0.73	0.50	0.56	0.44	0.062	0.168	0.078	0.141	0.0004			
rs1616619	6	134.48	CTAACAGAGGCTAGGTTCAGCTGGATCTTGTTCCTCTTCTCATTCCTGCAGGATAA[A/G]CTTATTGACCTAGAAAAAGCACGATAAGTATGACTGGCCTCTGATCTTCTTTCAAT	A	G	0.15	0.49	0.43	0.46	0.46	0.14	0.20	0.40	0.57	0.52	0.40	0.055	0.029	0.001	0.062	0.0006			
rs1981093	6	134.56	GACAAACCTAGAGTTGAACCAACATCTGGCTAAAAAGCAAATGCACCATGACAAAT[A/G]TTCATGGATCAGCAAAGTGCTAACCCAGTTATGGAAGTCTAATATAGTGTTACAG	A	G	0.18	0.09	0.10	0.02	0.14	0.09	0.33	0.09	0.07	0.14	0.12	0.004	0.041	0.003	0.001	0.0006			
rs1041480	6	135.35	ATTGCTCTGTGTTTCTCACATCAGGCACTTGTCACATGGTTTTGTAATTGCC[A/C]ATTATCTTGTGTGTATTCCTTGTCAGATTTTAGGTGCAGTGATAACTGGAAATGTGTTCAC	A	C	0.50	0.54	0.40	0.51	0.54	0.45	0.51	0.50	0.46	0.48	0.44	0.003	0.004	0.016	0.099	0.0001	1		
rs2064430	6	135.62	AAACTACGACTAAACAAAAATATTATTGATTCTAACCTAAGTTTTCAATTTTG[A/G]CAGCCAAAGAGATGAAGGAATGGAAGAATTTCCTTCTTGCTGATTACCGGATGCTGGGCTAA	A	G	0.57	0.49	0.49	0.37	0.50	0.45	0.44	0.51	0.39	0.39	0.49	0.001	0.015	0.000	0.006	0.0004			
rs975676	6	136.37	CACGTCCAGAAAAGCTCTCAGTAGTACAGCCATCTGTCCGAAATACTCTAACCT[A/T]CAAGGTGGCCACGGTCTCTTCAGAGGGTCATGGAGCTCTGCAAAGCTTGTCTTAAA	A	T	0.62	0.52	0.58	0.61	0.54	0.86	0.50	0.45	0.50	0.63	0.58	0.001	0.005	0.007	0.050	0.0000	1		
rs1028331	6	136.66	TATCAACAGCATCATATGAACATTTCAAAAACGAAAAATAAGGGTAATCATAGCCT[A/G]TGCCTAAAAATCCAATCATAGAGAACTTGCAGAAATATTTTTAATAATGTTCAAAA	A	G	0.63	0.77	0.85	0.88	0.86	0.57	0.75	0.83	0.86	0.79	0.78	0.013	0.002	0.007	0.024	0.0013			
rs1570056	6	137.04	ACTGAACCGGTCCCTGCTTTGGAGCTGACGTGGCTACAGGTTCCTTAGGCC[A/G]TCAGCCGTCTGTTCCAAGTAGAGCTAGCTGTATGTGGTACCCTGACTGTTCTCACCAGC	A	G	0.26	0.49	0.52	0.48	0.63	0.41	0.88	0.56	0.54	0.55	0.52	0.028	0.107	0.002	0.003	0.0001	1		
rs2040034	6	137.28	ATGCTCCCTTTGGTTCTGGAGGCTCCGCTTATAGCCAGCAAAAAAGGACTT[A/G]GACCACAGCCTTCCAGACGTATTTCCTTTTAAGAAGTCCTGTTCCCAGCAGGCCTCCAC	A	G	0.18	0.34	0.27	0.27	0.23	0.25	0.17	0.28	0.21	0.21	0.29	0.002	0.015	0.001	0.000	0.0009			
rs999638	6	138.05	TTTCCTCCCGTTTCCATTATATATTCAGGCAGTGACCATTGCTTCCAGGACCC[A/G]CTGCTATTTCTGGTGGTGCCTCAGATCTGGCACAATGGGTAGATCAGAATTAAAAGCTT	A	G	0.62	0.51	0.51	0.56	0.52	0.50	0.31	0.39	0.54	0.49	0.51	0.004	0.017	0.010	0.000	0.0001	1		
rs1018513	6	139.15	GGTTGGGACTTCGTGTACTTGGAAAGTTTCAGTGAAATTTATCATTGGCAGAA[A/G]CGAATTGACGTTTGTTTCACTTTACCTTACTTCTGGTTTTCCTTGTTTAGCTTACCTGTC	A	G	0.64	0.52	0.48	0.50	0.45	0.55	0.66	0.45	0.46	0.35	0.41	0.008	0.016	0.000	0.000	0.0028	1		
rs1358716	6	139.43	TTCGGCTGGCTTCCTTGTCACC[A/G]CTTTTCCTATCAAATGTATCCTGAATGTTACTATCAGAGTATTCTTCCAAAACGATGTTTTCATCACATCATTCTTATCTAGTAACTTACAA	A	G	0.48	0.48	0.42	0.43	0.38	1.00	0.73	0.64	0.46	0.40	0.34	0.003	0.059	0.041	0.274	0.0050			1
rs2032017	6	140.01	CTAGAACCTACGTCCTATATTTTAAATATTTTAAATTCAAGCCCTCCCCAAAGCACA[A/G]TTTTCAAATAAAGAGGTAAGACACAATTCCTACAAGTTTTGTAGTCAGGAAGACCT	A	G	0.79	0.72	0.62	0.76	0.66	0.50	0.34	0.69	0.64	0.69	0.70	0.008	0.071	0.001	0.018	0.0030	1		
rs2027195	6	140.93	AAGAGAATCGTGATTTCCATTTAAATTATTCAAAACAAAATTAGCTGGGGTAAA[A/C]ATATTTTAACAAAACATTCTGCACTTATTATTGTCTTCTGAGCTTGACAGAAGAGTAA	A	C	0.71	0.51	0.55	0.51	0.57	0.59	0.31	0.73	0.46	0.70	0.60	0.007	0.059	0.003	0.001	0.0002	1		
rs647875	6	141.12	TGTGTGTTTTACTGAAGAAAAATACATATTAATTCTGCATCTAAGTTACATGAT[A/G]GATATGCTCAGGAGAGCGAAAAATGAGAATGTTCCCTCGAAACATCAGAATTGTTCA	A	G	0.57	0.34	0.36	0.41	0.45	0.36	0.28	0.48	0.32	0.41	0.40	0.015	0.017	0.004	0.002	0.0004			
rs1349710	6	141.48	GCCAACATTCGGTCTGACTAATGCAGTACAGCATTTTCTAATGAAGGGAGAT[A/G]TAATGCCAAGGGAGTGTGGCATGATGGCCTTTGGAGTCAGAGAAAGTTGAGTTTCTC	A	G	0.17	0.35	0.34	0.26	0.32	0.14	0.11	0.26	0.32	0.36	0.28	0.016	0.038	0.001	0.029	0.0007	1		
rs225604	6	142.43	ACAGATCCATCAAGAGGTAGAGATGATTTCTCAAATCTAGGTTCATTTTGAAACTTTCTTTGCCTAATGTAACATTAGCAAAC[A/G]TAAAGCAAGCAGAGTCCTAAACAATGCTT	A	G	0.38	0.40	0.53	0.41	0.45	0.05	0.03	0.53	0.43	0.57	0.47	0.013	0.171	0.000	0.142	0.0025	1		
rs1931992	6	142.58	TTAGAGAGAGAACAGAAGCTCATGAGGGAGCCCGTACCTGAGGGAACAGAGGCCCATGAGATGCCGTAAAGGAAAGGAGAAAA[A/C]AAGGAGGGGCTTTTAAGGGAC	A	C	0.64	0.51	0.61	0.59	0.55	0.68	0.78	0.51	0.57	0.51	0.51	0.004	0.039	0.000	0.007	0.0033			
rs839556	6	142.85	GCAAGAATGTAAATGTCACCATTTTCTTTCAGGAGGTACACATTTCACTTTAAATC[A/G]GCATGTATTATTCATTTCACTTTAATTCTGTGGTGCCGACCCTGGAATTGCATATCC	A	G	0.21	0.54	0.58	0.62	0.50	0.68	0.92	0.60	0.64	0.55	0.56	0.088	0.060	0.004	0.001	0.0000			
rs200148	6	143.33	TTTGGGACTGACTGGCTAACTTGGTAGCTTTTAGTATGGGGTCAAACCTAATG[A/G]GACTTGATATTAAGAATGGGACTTGATACTGAGGCTTCTGCCTGGGGGATTAGCACAG	A	G	0.35	0.60	0.60	0.59	0.59	0.00	0.02	0.32	0.79	0.53	0.56	0.032	0.241	0.034	0.290	0.0009	1		
rs736784	6	144.05	TTCTACGGTTCTAGATAGGGACCATGGAAAAGCTGGAAAGTAAAATCTAGTATTTT[A/G]TGACTTCTCAAACATTAAGCCTATTATTATATATGCTTAGGTTAAAAAATTGTTTT	A	G	0.88	0.67	0.58	0.66	0.63	0.64	0.44	0.69	0.71	0.67	0.69	0.054	0.015	0.013	0.001	0.0051	1		1
rs612928	6	144.58	ACAAAGTCAACTATTTTGTTTTACAAAATTAGGTCACATTGCTTTTCAGTATA[C/G]TTCAAATTACCACAGACCTCAAACTGGTGGCCATCTGCCCGAGAATGAAATGGTATTGC	C	G	0.38	0.66	0.60	0.60	0.66	0.45	0.52	0.47	0.57	0.55	0.60	0.016	0.000	0.001	0.009	0.0008	1		
rs1832378	6	145.67	ATGTTGACATTGTGTCCTTCATTTTCAAAAAGAACTAGTATTTAAAAACAATT[A/G]TATGTGTAAGACCTGTGCACTTTTGTGAAGCTGCTATTGAGGGCATCGCTACTAGGTTG	A	G	0.67	0.57	0.63	0.54	0.57	0.41	0.29	0.46	0.54	0.54	0.44	0.000	0.111	0.040	0.048	0.0173	1	1	1
rs391524	6	145.80	AGTCTTAGGAAACACCTTGAAAATAAACAGTGACTATTTCTCATCATGTATATAC[A/G]TAGTATTTAGCAAATTAGAAGTGCTTAATAAAAGTCAATGAATCCCAAATAAATCATC	A	G	0.43	0.49	0.48	0.48	0.54	0.68	0.60	0.47	0.61	0.53	0.56	0.001	0.022	0.002	0.034	0.0028			
rs725258	6	146.77	TTTTATTCCATCTGGTCCTCATGCTAACATGATTGCAGTCTCTGGGATATAG[C/G]CTTGAGAATAATTTCCCACCTGCTGAGAACACAGTGTCAGAGAAGGTGGAATATGGATC	C	G	0.52	0.37	0.40	0.46	0.41	0.68	0.19	0.63	0.39	0.32	0.41	0.004	0.019	0.019	0.048	0.0001	1		
rs730862	6	147.95	TTAGGAGAGTTGGGAGTGGACCTGGGAAATATAATTCCAAGACGCATTAGAGAT[A/C]AATGGACAAAGACAGTTTACTCACATTCTTTGGAAAATATTTTTGAAGTCTCTAAAAT	A	C	0.74	0.50	0.50	0.56	0.57	0.95	0.85	0.44	0.71	0.57	0.48	0.019	0.053	0.004	0.112	0.0000	1		
rs1572856	6	148.42	TAATGATATTAACATTTTATGTAACTCATTCATTAAGGTCTCAAGTGGATGCTCAA[A/C]GCTATTACAATTCAGTACAAGTCACTTAGTATGAAACACACGCCTCCCACAGCACT	A	C	0.95	0.88	0.90	0.91	0.89	1.00	0.99	0.99	0.93	0.86	0.88	0.007	0.023	0.041	0.041	0.0002			
rs6570858	6	148.81	GCCCGGGGAGCCTCCTGTTTTCTGAAAGTCAAGCCATGGTACGTCCTCACAAAGG[A/G]CCCAACATCGAGTATCTTGATAAATACCTGAATGACTCCCTGAGAGGCGTGGCA	A	G	0.12	0.30	0.32	0.24	0.25	0.18	0.10	0.17	0.31	0.35	0.35	0.030	0.034	0.010	0.014	0.0006	1		
rs6570867	6	148.91	ATTTAAAAAATGAAATTTCAAAATTAATGAAATGTGTTTCAAACCTCCAGGCTGAC[A/C]CATTAAGCGAATCAACATGGTATATGGGTGAGGGGGGGTTGAGGGTTGAAAATTG	A	C	0.67	0.80	0.80	0.89	0.88	0.86	1.00	0.88	0.68	0.82	0.83	0.005	0.090	0.013	0.008	0.0003			
rs4131485	6	148.98	AAAGATGTTTTAGATGAGGCTCTCTGGAGAAAAACCTCAAGGGTCATCAGCAATT[A/G]GGAATCTCAGTAATACTGTGGCCCTGGCAGGGCTCTCAAGCATTTCCCATGCCAT	A	G	0.67	0.65	0.68	0.68	0.59	1.00	0.97	0.72	0.82	0.74	0.76	0.000	0.100	0.001	0.140	0.0065		1	1
rs1934221	6	149.36	AGGGTCATATTTTTCCCTGTCTT[A/C]TATGTTCCTCTTTGTGGCTATAAACCCAAACTTATGTGTTGTGTGTTTTTTCTATGACAATTATGGATATGACTTATTTACTATATGCTCTA	A	C	0.29	0.42	0.36	0.32	0.41	0.14	0.03	0.32	0.32	0.35	0.33	0.003	0.100	0.001	0.032	0.0015	1		
rs237012	6	149.71	GGAAACATTTTATAGAATAATTGTTTTAATCTGGGGATCCATCCTACTTACTA[A/G]AATTTTGGTCTCAGGCATTAGAAAACATTTTCACCTGACACGTTTTTAGTAAAACCTTGT	A	G	0.29	0.50	0.48	0.51	0.61	0.18	0.30	0.35	0.57	0.53	0.53	0.024	0.016	0.012	0.058	0.0015			
rs369643	6	149.78	TACTGGGGCCCTGGGAGAAGGAGGGCATGTCTATAGGTCACCTACATGGTGTCA[A/G]CTATGTGGAAATACCTGTTGGAACAGCTCTAGATTTAAGAATTAACTTTTTAGTTTC	A	G	0.10	0.44	0.41	0.46	0.50	0.14	0.29	0.29	0.61	0.49	0.48	0.077	0.012	0.016	0.058	0.0020			
rs2151913	6	150.16	TGTGCAAGAAAATATTTTTAAAATCATTTCTCCAATAAGCTGCCTTTATCCTATA[A/G]TATTGGATTATCATTAAATGACAAATCATAAGTCTGCTTCCTCTTAATATATTCTTCCA	A	G	0.88	0.36	0.38	0.37	0.32	0.91	0.91	0.47	0.32	0.41	0.35	0.166	0.175	0.012	0.183	0.0011	1		
rs1389717	6	151.07	ACACCCCCACCCCCACCCCAACCTGCATTTTAAAACTTCAGTTTAAAGATGTGTCC[A/G]TTACTTTGAAAGTGAAAAGATGTGTCCCTTTACTTTGAAACAAGCCCATGATTCA	A	G	0.31	0.36	0.34	0.32	0.36	0.09	0.04	0.25	0.21	0.29	0.33	0.000	0.080	0.001	0.036	0.0000	1		
rs2064356	6	151.12	CTGTAAGGGCACTCTTTGTGCAAAGGACAATGGCTGGAAAATCAAACCCATGTTT[A/T]TTCTTAGAGCTTGGGCCCTAGCAAGAACAAAATGAGCCAGCCTGAGCACATGGAA	A	T	0.71	0.22	0.24	0.29	0.23	0.41	0.54	0.31	0.25	0.23	0.24	0.120	0.043	0.001	0.018	0.0001			
rs1738575	6	151.32	AGAGAGAGAGAATAACTCAACAAACTACCACGTTTAAAAGCTTTCTAGTTTAGAT[C/G]TGAAAGGCTTCACGCTATCTCTCTTCCTCTACAGTCAAGCTGGTGCCACTTATACATGAA	C	G	0.29	0.65	0.53	0.43	0.52	0.41	0.13	0.58	0.57	0.53	0.56	0.027	0.071	0.001	0.004	0.0010			
rs1003027	6	151.52	AACTTTGCTGAAACAATGCTGACACCAGCTATTTTTGGATCACAAGATAATTTT[A/G]TATCTTAAGGGAGAGACTATCCTGTGACTATAGCGGTTTACAGGAAATCTTAGCGTTA	A	G	0.55	0.39	0.35	0.30	0.29	0.14	0.39	0.43	0.39	0.27	0.31	0.016	0.000	0.008	0.042	0.0015			
rs967726	6	151.64	TTACCCAAGGAGGAAAGAATTACAATCCAAGTTCCACACCTGAAGGATTTGCAA[A/G]TTAAACATCTCTCAGTCTTTGCTTTGAATGAGCTAAGAGCCAAGCTAAAACCTCACA	A	G	0.83	0.54	0.57	0.60	0.48	0.05	0.01	0.26	0.50	0.58	0.60	0.037	0.231	0.062	0.186	0.0028			
rs10002	6	151.75	GCCCAGGAGTTGCAGATCGGCCAGGACAAT[A/G]TAAGAAAACCCCATGTATTAGAAAAAAGA	A	G	0.64	0.21	0.22	0.19	0.16	0.73	0.47	0.34	0.25	0.25	0.27	0.072	0.025	0.001	0.112	0.0017			
rs718527	6	152.50	AGGTTAGGTTTAGGTGAATTTTCATTCTTCC[A/G]TTTTCCTAATTGCTACCATAATCACATCTTACTTATAGAGATGTTATTAGAGTATTTGGAATCTTCTACGATCAAACCTATTT	A	G	0.40	0.51	0.42	0.45	0.50	0.23	0.21	0.28	0.50	0.48	0.48	0.000	0.033	0.020	0.025	0.0027	1		
rs2029413	6	152.71	ACTTCAATATTAAGAGAAAACTTTCTCTCTCAAAATTAATGGCCCAGTGCTTGTA[A/C]AGAGTTTAAACTCAGGTCTTTAGAAGGAAGATTGCTAAGGATGAGAGAAACATCAAGTT	A	C	0.67	0.36	0.35	0.38	0.39	0.55	0.35	0.49	0.39	0.40	0.36	0.022	0.029	0.001	0.196	0.0004			
rs1407491	6	152.97	AGAACACGTTAAATATAATTCTATACCTTATGTAGAGCCCATTTAGACATAAGTCCCAGAGTAAGAC[A/G]TCTCTTCTTTCAGGCCTCCCTCAATCAGCCTCCCTCTCTGTT	A	G	0.69	0.72	0.73	0.65	0.63	0.73	0.43	0.55	0.82	0.64	0.73	0.002	0.053	0.035	0.000	0.0004			
rs686761	6	153.24	TCTCTGTTTCTGTGTGTTTAAAAATTTCTCAAAAATACGACAACAGCAACAC[A/G]TTCCCGCCTCTCTTGCTCCACCCTATCCAACCCCCTCCAAGAAGCTCAATAGTCACTGA	A	G	0.33	0.63	0.52	0.44	0.54	0.45	0.21	0.42	0.43	0.52	0.53	0.020	0.057	0.014	0.005	0.0000	1		
rs717687	6	153.28	ATCAGAACATAGTCAACCATCTTTGTTTTTATTTCCATGGTCACAAATAGATCCATGTGGGTCTCTG[A/G]CACTTCAAATACTTAATTTGGTTACTTAAACTTAAGTGATTCCTAA	A	G	0.62	0.35	0.44	0.54	0.41	0.50	0.78	0.56	0.57	0.44	0.39	0.016	0.061	0.013	0.003	0.0016			
rs316296	6	153.55	GCTCCAGTCAAGTTGAAATGTCATCTCTTCTCTTCCCTGTGGTTGGCCCAA[C/G]TTCATTGCAGGCTACATTGTTGTCTGCTCTGCTCATTGTCTGTCTGAGGTCTGCTTATTG	C	G	0.71	0.55	0.55	0.49	0.48	0.45	0.21	0.59	0.54	0.47	0.52	0.024	0.052	0.017	0.001	0.0003			
rs790266	6	154.57	TTGCAAAACGCCTTTGCTGGAGTACATCAAACTTAAAATTGTAGATCCCTA[A/G]CTCCATTTCCAGGGTGGTTCTGTGAAAATAAGTGGGCTGTGTGCAGAGTACAAACTGCC	A	G	0.50	0.42	0.49	0.44	0.54	0.23	0.88	0.60	0.50	0.38	0.48	0.000	0.086	0.018	0.041	0.0001	1		
rs1361218	6	154.75	CACAGGAACTAAAGGAAAGAGATGGTCTGAGCACAGAGAATGAATCTAGGA[A/G]AGAGGTGACTGGTTTGGGCTGCATCAGGAAAATGCGAAGGCTAAAGACCAGAGCAAG	A	G	0.62	0.46	0.42	0.39	0.45	0.82	0.95	0.60	0.56	0.40	0.36	0.026	0.209	0.034	0.107	0.0016			
rs1385733	6	155.51	TTAGGAAAAGGGGGTGGGATGTCTTGGATTAAAAGGAACTGATTTTAGGCATT[C/G]TAGTTTTTGATGTTAGAAAAAATATCTCCAGAACACCTTGATAAATGTGGTCAGGT	C	G	0.62	0.52	0.45	0.52	0.43	0.64	0.31	0.47	0.57	0.46	0.40	0.018	0.010	0.000	0.016	0.0001	1		
rs912722	6	155.61	CATTCAAAAACTAAAATATTCTAATGCCATCTATTATCTGCTGACTGACTTTA[A/C]TGATTTGGATTGATTTTTAAGCCATTGTGAAACTGTGTTTTAAAATTAAGTGACCATCAAG	A	C	0.36	0.40	0.35	0.45	0.36	0.00	0.06	0.39	0.39	0.40	0.45	0.000	0.064	0.001	0.134	0.0028			
rs231958	6	155.79	TACCTCATCAGGGCTTTCTCCTTTACCACTAATGGTCTTCCCTAGTAATTAC[A/G]AAGTGTCAGCCTTATGGTTACGATGAGTTGCTGGTTAAATGGACATAAAGTGCTT	A	G	0.57	0.58	0.66	0.56	0.61	0.32	0.74	0.61	0.54	0.66	0.62	0.000	0.000	0.010	0.097	0.0040			1
rs1902064	6	156.75	ATAAGTCTGGATAGATTGGAAAGGAGGATGTTGTCTGGCTATGAAGGGCCCTGA[A/G]TGACTGGCTGAAGATTTGGATTTCATTTTGTGTTTAGAGTTAGGGCCACGACAATT	A	G	0.21	0.52	0.50	0.48	0.63	0.27	0.34	0.25	0.36	0.42	0.56	0.015	0.041	0.025	0.036	0.0025	1		
rs767860	6	157.45	GAAGAGAGCACCCGTGTCCAAGACAACTTAGCACTCCCCAGTGCTCCATGGGC[A/G]TGCCTGAGAAGGAGGCCTGTCTTCCCAGTTAGGAGCACATCATTTCTCACAGG	A	G	0.43	0.43	0.43	0.49	0.50	0.55	0.41	0.60	0.29	0.49	0.48	0.000	0.000	0.013	0.006	0.0014	1		
rs1133482	6	157.65	GCTGGCTCTCCTCCAGGGAGAAAGCCTGTCACTTATTCCTCAACACTGTT[A/G]GGAAGTTACCTAGAAAAGTACAGACAGAAGACCTAGGCTTGCGTTTTAGA	A	G	0.76	0.55	0.56	0.57	0.52	1.00	0.91	0.82	0.61	0.53	0.53	0.021	0.123	0.053	0.209	0.0016			
rs602890	6	157.98	AATGAGGATTCACTGAGGCTGATAATCAAATGAAGTTTGCATTCCACAAGC[A/G]CATATCGGTAAGGTCTGGGTGCACAGTGGGTCTGGCTCTGGACACAGTCATGGTGGCTTT	A	G	0.26	0.49	0.56	0.63	0.39	0.18	0.69	0.38	0.54	0.44	0.52	0.047	0.022	0.006	0.067	0.0017	1		
rs262827	6	158.59	CAGGAGCTTAGGTCAGTAATTTCTCTAGTTCTTCTGCAAAGAATTGAACTGTT[A/T]CCCCTCCTTGCATTCTGTTATCCTTTTCAGTGAGATTCCGGAAGAGTCTGAGAGAAGG	A	T	0.60	0.65	0.69	0.68	0.68	1.00	0.98	0.84	0.64	0.65	0.70	0.001	0.110	0.036	0.134	0.0026	1		
rs1866896	6	158.85	GTTCCACAGTAGCCACTACCAAGTCCC[A/G]GCCACTTCTCCTGCAGAGCTCCCTCTCCCTCTGCTGCCTGACCTGGGCCAGCACTCACTCTCTTCTGCCTCTTCAGCA	A	G	0.12	0.34	0.41	0.34	0.45	0.64	0.66	0.40	0.32	0.38	0.47	0.025	0.082	0.012	0.063	0.0071		1	1
rs923459	6	159.47	TAAGCTTAAAGCCATTGTTCAAAAAGTCACACTATTGCTGACATTCAATGGT[A/G]AAGAATCTCAGTGAACCCAAGGAGTGCACATGCTTGTTTAAAGTTTTTTAAAAAGCACTTT	A	G	0.64	0.52	0.45	0.53	0.54	0.36	0.29	0.49	0.46	0.51	0.56	0.017	0.019	0.001	0.005	0.0049	1		1
rs294882	6	159.51	GCTTCAATTCTCCCAGGCTATCAGAGAGCAGTAGAAGGAAATTGATGGGTGG[A/G]GTGGAAAGTGGTATTAAAAACAGACTGGAGCCAGGGCACATGTTACAGGAAAAGAG	A	G	0.36	0.45	0.53	0.49	0.43	0.56	0.79	0.47	0.57	0.51	0.44	0.017	0.039	0.002	0.000	0.0031			
rs409359	6	159.85	CATTAAAAAAATACACGTGCAACAATGAAGCTAATAGATGGTATTCAGATT[A/G]CCAGCCTTGTGCTTTGTCTGATATTCTGGTTGTCTTTTAGATTTCTTGAGTTTTAGATTC	A	G	0.38	0.54	0.51	0.50	0.43	0.59	0.51	0.60	0.57	0.52	0.44	0.002	0.000	0.003	0.008	0.0032			
rs8031	6	160.01	ATCAAAGTTTAATTATTACAAAATAGTTCAAGCAACAGATAGAATTTCAAAAC[A/T]GTATTTGCTTTGCTTCCTTGTTTTGCTCCAACACTAATCATGCTGAGGTTTTTGAAGCAC	A	T	0.74	0.52	0.46	0.51	0.48	0.77	0.43	0.50	0.57	0.58	0.56	0.033	0.001	0.001	0.048	0.0045			
rs927450	6	160.09	CACCTCTGGGGCCTACACTCTTTACCTTCTCTGATTATCTGACCCATCCGC[A/G]TATCTCTGCAAAGTCTTAATGTAGGTTTCCAGGATTTAATGATTCTTTCAATCAACTTTAA	A	G	0.79	0.44	0.39	0.40	0.39	0.73	0.34	0.49	0.46	0.50	0.52	0.068	0.004	0.000	0.044	0.0077			
rs927451	6	160.12	ACTGTGTGATGTATTATTTATTATTGAACAAGCATCATACCTGTATCACTTG[A/C]TAGGCATTGCTGTTAGAGGCAGGAGTTACAAAGATAAAGTAGATGTTATCCCTGTCATCA	A	C	0.29	0.54	0.60	0.59	0.57	0.27	0.66	0.51	0.57	0.47	0.45	0.034	0.008	0.003	0.024	0.0089		1	1
rs1003737	6	160.34	GGGTATGGGGTTCTCAAGTTTTTTGGATATTACCTGGTGTTTAAAGGGGACAT[A/C]ATTGTAAAATTATTATGATTGTTCTTATTATGGGCATATTGTATGTAATTTGTTGATTTT	A	C	0.50	0.55	0.63	0.60	0.46	0.27	0.49	0.49	0.46	0.60	0.55	0.010	0.019	0.004	0.073	0.0040	1		
rs675162	6	160.69	GGGCACCTGACAAAATGGTATGGAGGCTGGTGCCTCTAAGAGCATCCTCCCCT[A/G]GGTCTGGTACACAAGGTCTCTGTGCCTCCACCCCATGTCTCCGCCTATAGAGGCTT	A	G	0.48	0.45	0.47	0.45	0.59	0.64	0.76	0.66	0.62	0.51	0.52	0.002	0.060	0.021	0.025	0.0017			
rs1406888	6	161.00	ACTTTCTCAGAGGTGCTGGTGAGGACATTATGAGTCTGGAGCCCTGAAAAGC[A/G]TTCGACTCTGCCACTAGCCAGACAGACCTGGACTAGGCACGTTAACTCTTTGTATG	A	G	0.31	0.59	0.52	0.50	0.45	0.36	0.08	0.32	0.71	0.55	0.59	0.040	0.156	0.022	0.023	0.0016	1		
rs11902	6	161.08	TTTGTCCTTTCTCTTCCTTTCTAAACATGTATCTCTCCCACAGGTCATTCTCCTGT[C/G]ACCGTGTGTACTGCAGACTGTTTCAAAACCGGGCAGGCAATTAGCAATGGGAAATA	C	G	0.48	0.50	0.49	0.56	0.66	0.95	0.95	0.72	0.43	0.49	0.49	0.001	0.147	0.011	0.153	0.0009			
rs783182	6	161.08	CCTGGAAGTGTGTTCTGACTTTGCCACTAGCTAGATAGACCTGGACTAGGCAC[A/G]TTACCTCTTTGTACCACTCAGCTCTAACCCCTCATTCAAAACCCAGCATTTTCAAGT	A	G	0.43	0.49	0.50	0.54	0.64	0.95	0.95	0.72	0.39	0.50	0.55	0.004	0.148	0.011	0.153	0.0007			
rs1080085	6	161.62	AATTCTGGGAGATTGGACATGGACCCTGGAGCGATGCTGACATTTATTCATC[A/C]TGGTTATCTGCATCATGGTTTTCTCATCCGTAAAGCAGGAAATGGGGACAGATGTTCCT	A	C	0.83	0.64	0.68	0.67	0.61	1.00	0.97	0.90	0.89	0.71	0.73	0.008	0.069	0.016	0.117	0.0020	1		
rs577876	6	161.83	ATAAAAGCTTTTTTATGAGTTGTGTTAAAAACTGCTTTTTGTGGATCACATGAAGC[A/G]ACTCTGAAATGCATTATCTCTGATCATCATTGTTGCTAATCATGTCCATTTTGGGCCA	A	G	0.76	0.57	0.53	0.60	0.61	0.73	0.81	0.51	0.75	0.59	0.59	0.037	0.055	0.003	0.031	0.0035			
rs1893098	6	162.19	ACCCTGCTTGATTCTGCTTTTTTAACTGTACACTTTCTTATGTTAGATAATATTTAC[A/G]AAGGATTTTTATATCTATAGCCTTATGTAAAATTGGCCTGTAATTTTCTTTTCTTACAT	A	G	0.48	0.54	0.46	0.54	0.50	0.73	0.34	0.65	0.64	0.52	0.45	0.000	0.005	0.010	0.034	0.0005	1		
rs1954948	6	162.51	TTCTCCCTTCCCCACATTGACTGAATGATATGTTGTCGGTTCCTTTTGTTCCA[A/G]TCAGGTAGTTTTCAAAATTTCATGGCAGGGGAACTGAGTTAACCAATATATACATAGCCT	A	G	0.57	0.57	0.64	0.70	0.63	0.64	0.93	0.52	0.64	0.59	0.58	0.001	0.073	0.012	0.000	0.0030			
rs1333962	6	163.18	CTCAGGCCTCTTCCTCCTTAAAACCTTTCAGGGCTTCTCACTAGCCTTTGAT[A/G]TAAAGTCCAGATCACTCAACATGTATATAAAATACTTGATGGTCAGGTTTCTTTCCACTT	A	G	0.19	0.57	0.55	0.66	0.50	0.18	0.19	0.40	0.50	0.58	0.60	0.079	0.074	0.015	0.071	0.0012	1		
rs942731	6	164.57	CATCAGATAAATCGTCATTTAAAAATAGGGAATTTGGTAGATTCCCTCCAGGCTG[A/G]CAACAGCTGTGTTCATTCTCCCTTGCTCTTAGGGTATTTTTTATTTTGAGTGCCCAC	A	G	0.52	0.43	0.48	0.39	0.57	0.32	0.68	0.36	0.50	0.46	0.46	0.003	0.003	0.011	0.031	0.0016	1		
rs1886672	6	164.63	CTGTGTTTTCACAATGTCATGGAAGAGGAAGTGGCGATGGCTGAATCAGGTGGGG[A/C]GCAGCCCTAAGTATTCACAATCCACCACACATTTTGATGGTTCACAGGGACTTTG	A	C	0.38	0.49	0.54	0.51	0.39	0.05	0.20	0.33	0.61	0.42	0.51	0.008	0.059	0.019	0.159	0.0012			
rs727811	6	164.95	TAATCTATCCTACATGTGTTTCTTTTTCTCTTACCGGAACTTCAACGACTTA[A/C]AATCATCTGCATCTCCCAGCAATCTCATGATTTCATTCACTTTATATTTTTGCTCTGTGGGC	A	C	0.31	0.47	0.61	0.54	0.46	0.59	0.41	0.59	0.36	0.58	0.61	0.056	0.026	0.001	0.001	0.0000			
rs646444	6	165.63	GAGCAAAGCTGTCTCAACAAGAAAATGATTTAAAAGAAATTTTATTGTGTGAT[A/G]TCAGAATGCATTTAGTGCTCTTCATGTACAGGTGAGTGGGCAGGTTGAGAGCGTGGTC	A	G	0.26	0.54	0.54	0.51	0.54	0.64	0.48	0.54	0.54	0.49	0.62	0.024	0.001	0.001	0.013	0.0053	1		1
rs491253	6	165.68	CTCTCTCTATCCAAAATTAATACTAAAACACAGAAGGAGTGTTTATTTCATTTG[A/G]AGAAATAAATGGCAGACTTGGCCTGAGAACTCAAGTTGCCCAATGTATAGAGCCAGGC	A	G	0.71	0.35	0.40	0.39	0.38	0.32	0.45	0.45	0.36	0.38	0.34	0.031	0.000	0.000	0.015	0.0049			
rs875591	6	165.98	CTGACTGACAAAGCCTTCATCCGGCCCATTTGCCACTTCACTTGCCACTGCCT[C/G]GTGGGCTCAGCTCCAGCACGGCCGTCACCCCGATCCTCTGCCTTGTGAGCCCCCTG	C	G	0.21	0.38	0.38	0.37	0.43	0.45	0.56	0.49	0.46	0.42	0.45	0.025	0.005	0.001	0.000	0.0058			
rs3002430	6	166.20	TCCCCAAAGTTCAAAGTTACCACTGCATGGAATATTACTTATTGGGGACCTTCA[A/G]TGGTTAAAAACCTGCAGCCTCCCAGTTCTCTTTAAGAGTGGCTGCCAAGCCTCCGTG	A	G	0.71	0.80	0.75	0.61	0.75	1.00	0.94	0.89	0.88	0.72	0.66	0.003	0.026	0.017	0.090	0.0073	1	1	1
rs1445282	6	166.43	CTATCGGAATGGTAATGTCTAGTGGTAGTGCTGACGAGAGTGTAGGTAAGAA[A/G]GTATTCTGCATGCTGAAATGGGTTGTGCAAATTGGTACAACCTCTTTCAGGGTCAGTT	A	G	0.07	0.23	0.30	0.38	0.39	0.36	0.35	0.33	0.25	0.29	0.26	0.056	0.000	0.002	0.000	0.0027			
rs6910121	6	166.77	ATTTCCATCCTATTTCCTTCCCTGGCCTTTGGAGAAACATAGGTGTGTAAAGT[A/G]GACGTTGTCATGACAGTGCAGGCTGCCATGGTGGCTTTCTGTCTTAGTTAAGAGCGGG	A	G	0.81	0.74	0.67	0.79	0.71	0.95	0.73	0.88	0.71	0.65	0.66	0.008	0.002	0.027	0.071	0.0007	1		
rs880900	6	167.05	TTTTGGGGAACCGACAGATTTACTAAATAGCACCATGCTGAATTTGAAACAGCC[A/G]CGCCCCAGAAGCTGATCAGAAAAGATTGTTTTCAGGCCAAGAGTAAGATGTAAGAAT	A	G	0.30	0.71	0.74	0.78	0.68	0.95	0.80	0.79	0.68	0.78	0.78	0.096	0.003	0.002	0.045	0.0015			
rs533810	6	167.10	CTCCTTTTTTGTTGTGTTTTATCCACCACTTCTAGTGGGTGTAGCGAGATC[A/G]TTCTCTGTCATTTCTGCCTGGCGTCTTGCTGGAAGTAGAAGTCCTCTAAGCTGTACTTTCTC	A	G	0.19	0.61	0.70	0.65	0.64	0.55	0.43	0.57	0.75	0.64	0.73	0.167	0.051	0.011	0.019	0.0000			
rs916331	6	167.38	TAAGTTAAAATCTCAGTGACTAAGCACACTAGAACCTTCTTACACACATAAAA[A/C]CCCAATCATCTAATCAGCTTTTCTGAGGAGGAGAATCTGCTCCACACAGTAATTCAGCA	A	C	0.83	0.65	0.59	0.52	0.52	0.83	0.79	0.79	0.79	0.58	0.66	0.020	0.010	0.027	0.017	0.0007	1		
rs2981963	6	167.62	CTGTTTCATTGTTGCTGACACCAAAAGCAATAATGAGTAAAAATTGCTATGCATCA[A/C]CTTCACGGCTTCCCTTCTTCAAATGCACTTAGCAGGCATGACATCGCATGAGAAAA	A	C	0.33	0.19	0.20	0.24	0.23	0.23	0.02	0.14	0.18	0.16	0.19	0.005	0.064	0.020	0.000	0.0001			
rs595491	6	167.94	GCCCTGACAGCCACCCACTACTCCCGAGGGTGAACATCCAGAAGAGAGAAGGTG[A/C]ACCTCTCAGAGTCGGTCTGGACCGTAACAATGGGAACGGTTATGCTCTTTCAAAA	A	C	0.21	0.15	0.16	0.17	0.16	0.27	0.27	0.26	0.18	0.14	0.17	0.005	0.010	0.027	0.014	0.0007	1		
rs1132306	6	168.10	TTCTTTCAAAATGATACTGACCACATCTGCAGCACCTCCTTTCACAACCGACTTCAC[A/G]TAGATTCCTAGTTTATCTTGACCAGCACCCTATTTTGCAAAACAAGGCGAAAACAC	A	G	0.55	0.50	0.56	0.53	0.55	0.14	0.45	0.45	0.61	0.64	0.63	0.000	0.015	0.002	0.165	0.0032			
rs1532145	6	168.31	ATTGAAAACATCTATGTTCACAACATATAACAAGACAACATGTGTTCAAAACAC[A/G]TGGCAAGATGTCCTTCTTCCTGCAGTGGAGCTTCTAACCAGGCTTCTTACCAGACAC	A	G	0.76	0.77	0.77	0.80	0.80	1.00	0.88	0.86	0.89	0.84	0.78	0.001	0.019	0.003	0.070	0.0000			
rs1884477	6	168.71	TTGAGGGAAGTGCCTGAGTCCCCTTAAGCCAGCAGCCACCTCAGAACCCCAT[A/G]GCGAGTCTATGGTAGGCCCGTCCACTGATTTCCACCTGCAAAGCTAGCGCGGTGCA	A	G	0.26	0.28	0.23	0.27	0.23	0.23	0.24	0.36	0.46	0.24	0.24	0.003	0.002	0.006	0.001	0.0007	1		
rs1590289	6	168.92	TGAGAAAACATACTGGCATCCAGGGAAAAGTGAAATGCAACTTTTCTCACCTATT[A/T]TGCATTTTTCTGGGACCCTTAATGGTTAAAACCCAGGGGAAGCAGTGCGACTGAGT	A	T	0.38	0.75	0.73	0.63	0.75	0.68	0.71	0.73	0.68	0.80	0.82	0.049	0.000	0.000	0.001	0.0067		1	1
rs4235911	6	169.10	GTAGCAGGGCGAACCACAAAGAAGCTACGATTAAAAATTACCAGAAAAACCTGAAA[C/G]AGTGGCACCAGGAATGAGAGTCACAGAAAGCAAACCAAAAATGAGCCCAAGG	C	G	0.45	0.51	0.51	0.38	0.57	0.27	0.43	0.55	0.39	0.57	0.45	0.001	0.006	0.001	0.034	0.0026			
rs6917113	6	169.27	GGTGAGACCTGGGGTGAGATTAGCTGTGGGATGAGAACAAAGAAGAGAACCAGGA[A/T]GACCCCTATCTGCCTGGATGAGCATGGGAGAAGGGTCTCATCCTGAAGTAGGG	A	T	0.71	0.82	0.84	0.88	0.80	0.95	0.95	0.93	0.82	0.83	0.88	0.008	0.027	0.021	0.023	0.0026			
rs727619	6	170.56	AGAGCTCAATGCCTGTCCTGGGGGCTCCCCGTGGGCTGGGGGCCTACAGC[A/C]CTGACTTGGCGGATCTGTCTGCAGCTGGCCGTGCACATTAGGGCTGCTTGGGAAGAG	A	C	0.31	0.64	0.60	0.59	0.71	0.09	0.65	0.52	0.89	0.62	0.70	0.046	0.002	0.002	0.169	0.0082	1	1	1
rs2294183	6	170.66	TGGGCTACCCCAACTCCACTCCCCAGCCTTACAGCCATGCCCTGGGCCTCCAC[A/G]TGTCCAAGAGGGTCTTCAGACTAGGTGGGGGACAGTGTCCAGGACCTTCCTCACA	A	G	0.28	0.43	0.42	0.50	0.43	0.43	0.34	0.30	0.27	0.48	0.53	0.007	0.002	0.001	0.002	0.0077			
rs909475	6	170.67	TGTTCCCTCCCTTCAGGGGGCCTCTGAATATTCTCCAGTGAGTTACGACGTTGGT[A/G]CCATATCTCAGGAAGTCACACCAACTAGACACTTGTAGAAGCCAATGTGTTCCCTT	A	G	0.24	0.40	0.39	0.46	0.39	0.65	0.41	0.28	0.32	0.43	0.45	0.012	0.000	0.001	0.042	0.0018			
rs909472	6	170.67	AGGAAGACCCTAAGGCTCCTGCATTTTGCATGTTGATGGCCGTTGTGTTATGAC[A/G]TTTTTTTCTGCTCACTTCCTTTTGTTTCTGGAGGTTTGTGCACCCCTGTGGCCCTGG	A	G	0.26	0.27	0.27	0.24	0.34	0.23	0.15	0.44	0.39	0.22	0.22	0.000	0.006	0.011	0.001	0.0012			
rs1474555	6	170.69	CCACATCAACTGAAGCCACAGTGACACC[A/G]AGTCAGCATCCTGGGTGCAGCTGCCACCCTGTCACTCTCATCACATTCCACCATTCCCAGGCCACAGAGGCAAGAGGATA	A	G	0.29	0.48	0.49	0.46	0.52	0.18	0.66	0.65	0.54	0.42	0.40	0.022	0.021	0.014	0.054	0.0025			
rs760909	6	170.71	CAAAGAATACAGTCTGAAACAGGTTTGGAAGAGGCAAGCTTAGTTCTAGGTCAG[A/C]CCCACAGGACGTGGGATGAGGGATATATACAGGCATTCGTTAATGCTGCATTGTTCT	A	C	0.48	0.49	0.49	0.49	0.52	0.20	0.66	0.65	0.54	0.42	0.40	0.000	0.021	0.014	0.047	0.0028			
rs756519	6	170.71	TCACCAAAACTAGCAACACTGTTACATG[A/G]AAATCAAAGAACAGGGCTCTGAAGTGAAACAAAAGCCAAATCTGAATCTTAGGTCTGCTGCTTAGTAGGTATCAGCTTTCCCAC	A	G	0.17	0.40	0.40	0.46	0.39	0.68	0.36	0.28	0.32	0.43	0.46	0.029	0.000	0.001	0.052	0.0018			
rs2076318	6	170.73	AATCCGCCAAATTATTGTAGCCCTAGATTTTATGGATGTGAATTATCACCATCATCA[A/G]TCATCACTATTTCTGTATTTTAGCCCATGCATTTCTACTGAGAGGAAAACTAACAA	A	G	0.69	0.51	0.51	0.54	0.48	0.82	0.34	0.35	0.46	0.58	0.60	0.015	0.024	0.016	0.050	0.0024			
rs8770	6	170.74	TTCCATTTCTGGGATTTTATCCTAAGGAAATACTTATACCAAAAATAGAGGTGCAGAGATGTTGAC[A/G]GATTGCTTACACAGTGTCTACTTATTAGTGAAACAAAAGTGTTCAGT	A	G	0.40	0.49	0.49	0.48	0.52	0.18	0.66	0.65	0.54	0.42	0.40	0.003	0.023	0.016	0.050	0.0025			
rs2021899	6	170.76	CCTTAGGGATCTCCAGGATTTATCCCAGAGTGCCCATAATGGACAGTACAAAA[C/G]TGGGAAGAGTTTCCAAGAAAGCAAATACCCCATCATCCACCACAAAACACACTCTCAC	C	G	0.71	0.51	0.51	0.54	0.48	0.82	0.34	0.34	0.46	0.58	0.61	0.020	0.023	0.019	0.050	0.0029			
rs1127460	7	0.62	TTCCAGAGTTCCTTTCAGTCTTTATTTGCAGCAAACTACCACTTTATATG[A/G]CAGGTTTGTGTGTCTGTACACGCACATACACACACATATCCTTAAGCTCG	A	G	0.14	0.24	0.25	0.23	0.13	0.23	0.00	0.31	0.11	0.22	0.31	0.014	0.106	0.000	0.002	0.0008	1		

rs1881114	7	0.67	ATGATCTGTAGGACAAACCCTAGTCACTAGTTTACCTATATACCAAGCCTGCACACA[C/G]ACCTGAGGCTAAAATAAACATTAGCATATTTAAAAATAAATCAACAAAGCCTGCA	C	G	0.40	0.34	0.42	0.37	0.43	0.50	0.38	0.38	0.36	0.43	0.41	0.000	0.001	0.001	0.005	0.0000			
rs1057558	7	0.81	GAGGAAGTAACTGGGGAGGGAGGACGGTTGGGGGGGATTCACCTTTAAAG[A/G]ATTTGAGAGCTTGTTTATTTTGCAGAATAAGAGTAAATTAATCCCCTGTGTG	A	G	0.76	0.82	0.80	0.76	0.84	0.91	0.64	0.88	0.79	0.85	0.85	0.001	0.016	0.017	0.015	0.0028			
rs1133043	7	0.88	CGCAGTTGTCCCCAGCTGTTATTTCTGTAGAAACAAATTCGTCAGCCACA[C/G]GTGCTGGAGTATTTCATTGTCCTGGCACCCGACTAACCCACACGGCAGCT	C	G	0.81	0.68	0.61	0.65	0.64	0.23	0.57	0.60	0.64	0.59	0.64	0.018	0.001	0.002	0.083	0.0000			
rs1543985	7	0.96	CAGCGTAGCTGGGGGTGGAAGATTCCGTCCCCTGGATCCACACAGTCACAGC[A/G]CACTGTTGCCCACTGATGGCAGCCTTGAGGCCGCCATCTCCTCTATCCTTGGCTCT	A	G	0.14	0.32	0.39	0.35	0.34	0.67	0.40	0.31	0.31	0.39	0.38	0.027	0.000	0.000	0.047	0.0002			
rs1362136	7	2.25	ACGCCTGGCTCCCATGATGCACAACTTACTTATTTGTTCAATCCCAGTGTTC[A/G]TTGTTTCAGAATTGCTAAGATGTACCCCTGGGAGAACCAAACCTACTAACTAGAGTCCA	A	G	0.53	0.26	0.27	0.32	0.23	0.83	0.62	0.44	0.18	0.30	0.22	0.041	0.126	0.018	0.254	0.0003	1		
rs798485	7	2.55	ATTCACTTTAACCAAGACTCTAAAGCAACAGCTCAGCTGAGCTCCTTCTCTCTCC[A/G]TCTCCCTCGCTCTCTGCCAGCCATGGAGGCCACTGAGTGGGGATCTCAGTGCAGA	A	G	0.14	0.28	0.27	0.29	0.20	0.38	0.18	0.08	0.32	0.21	0.33	0.017	0.013	0.038	0.004	0.0016			
rs1044701	7	4.05	CCGAACACAGCACACGGTCAACTCCGCGGGACACGAGGACACGGGACGGC[A/G]TCTCCAGAATTGCTTGTTACGTAGGAAGCGTGCATTGTTAACCAGAGTAT	A	G	0.19	0.41	0.50	0.46	0.29	0.82	0.73	0.45	0.18	0.43	0.38	0.045	0.040	0.001	0.076	0.0092	1	1	1
rs1470539	7	4.25	AGTTATAAATTCTCAAAGAATAGGGAGCATTTTTCTTCTTATATTCTCTCC[A/C]CAGCTCCATACGCAACACTGCGTGTAATAACTCTGTAATGGCTGTTGATTGACTGGGTAT	A	C	0.74	0.66	0.59	0.59	0.61	0.86	0.65	0.61	0.79	0.57	0.69	0.011	0.002	0.001	0.042	0.0054			
rs1553960	7	4.64	GTATCTGTGAAAGACTGGATATCATAGGTGAGATCAATGCTGAGAATTTCAGA[A/G]TTATCTGGCTTTTTTAGCCCTAACCCAATCACCCACATTGTACGAAATTCTTCCATATCA	A	G	0.40	0.65	0.60	0.54	0.68	0.55	0.59	0.48	0.46	0.60	0.65	0.011	0.000	0.000	0.000	0.0019			
rs852432	7	5.31	ACTGATGGAGTGCACCGCTACAGTCACCTTCCAGTTGTTACTGATATGCCCA[A/G]TTCTCAAATGGGGATATGGGCAGTGATGTGACTTCCCAAAGCCACATAGACAACGTG	A	G	0.67	0.64	0.62	0.61	0.71	0.82	0.63	0.74	0.68	0.70	0.67	0.002	0.003	0.023	0.032	0.0002	1		
rs1468996	7	5.48	AATAAATGTTCAAAATCAAAACAGGAGTAGTTAGTAGACAAATGCTTAGCTATTTT[A/G]ACAACACCTAGAACACTTTGAGAGAGTGGCTGTAAAATTTCTGCCCAGTGCACCCT	A	G	0.19	0.45	0.41	0.34	0.54	0.82	0.63	0.44	0.46	0.46	0.42	0.031	0.025	0.002	0.081	0.0002			
rs4560	7	5.81	CAGTTACGGCAATTCACTTAAGGAGCTTGAGGGCCGTGTTAAAAGGAGCCAG[A/G]TTTTCACAAGACCTCATCCACCTCTGCACATTGGCTGGCACTGTCACACTGCAGCCTC	A	G	0.19	0.39	0.40	0.43	0.41	0.41	0.29	0.32	0.39	0.45	0.41	0.020	0.005	0.000	0.001	0.0003	1		
rs558030	7	5.84	CAACTAAAGACTCTATTTCACTTTCATCAAAATTGTGGACTCTTTCACCTAGT[A/T]AGAGTCAATGTTTTAAGCATCTCTTACTCTATGCTAAGTAACTTAGCTACCCCGTATCTC	A	T	0.19	0.40	0.40	0.44	0.41	0.41	0.22	0.32	0.39	0.46	0.41	0.020	0.019	0.000	0.001	0.0003			
rs758263	7	6.97	AGCAGTAGATTTTGTTGTATCCCCCTTGCGGCTTGTGTCTGCTACTCTCCGC[A/C]TTTCTGATCCTGCTGTAAGGTTATGCACTAGCTTCAAGACCTTGAGCGGGACGAAAGC	A	C	0.40	0.53	0.58	0.54	0.61	0.05	0.22	0.45	0.61	0.64	0.63	0.021	0.097	0.019	0.215	0.0025	1		
rs1299548	7	7.05	CCACTACCTCAACCAACTGCTCCTCAGAGAAGTAAGATGGATTTGTGGAAAT[A/G]ATTTCCTTGAGCCATTTTTCCAAAGATAGTGGGGCATTCTTAAATGGTATTCATTTGA	A	G	0.74	0.45	0.54	0.40	0.39	0.09	0.32	0.51	0.32	0.52	0.56	0.018	0.036	0.001	0.137	0.0000			
rs719423	7	7.10	ATTCATTTGCCTTTCATTCTGAGTTCCCATGAAGCTAACCTGGCAGTAAATTCTA[C/G]CTCAACCTTACTTGAGGCACCTCCCAGTCCTCCTGCCTGGATAATGTCAAGTTTCCA	C	G	0.50	0.59	0.66	0.61	0.57	0.32	0.22	0.51	0.64	0.53	0.59	0.010	0.112	0.015	0.070	0.0008			
rs1882600	7	7.11	TTCACAGTGTGAGAATGACAGGGCTGGATCAGACAATAGCCCCGAGCATGACTAA[A/C]CTGCCCTGGGTGTGTTTTCGGTCATGCGAATATGAACATTGGCATTGAGATAAGC	A	C	0.24	0.65	0.65	0.60	0.70	0.68	0.71	0.73	0.71	0.63	0.60	0.113	0.000	0.002	0.000	0.0022			
rs2042545	7	7.62	AAACAAGCAAATAAAGCAAGATATTACTCATAACTAAATGGAACTAAAAACTAAT[A/G]GACTACAAACTAAATGGAAACAAAACCTCAGAAAACCTAAAATAAGATAACTGATG	A	G	0.30	0.51	0.51	0.59	0.57	0.44	0.38	0.54	0.46	0.57	0.50	0.008	0.004	0.005	0.028	0.0006	1		
rs37995	7	7.77	AGAATAGCTCCATATTTCTCGTTAATATTCTTTATGTGAGGCTCATCTAAAATCACTGTG[A/G]ATAAAGTACAAGACAGTGAAAAAAACAACTTGAATTCAGGTCCAATTGTCATT	A	G	0.62	0.54	0.52	0.54	0.63	0.64	0.85	0.67	0.50	0.50	0.49	0.006	0.081	0.007	0.008	0.0005			
rs1558557	7	8.05	ATTGCAGCTCAGCCTTTATCCTCATCAATCCAGTTGTCTGCCCCATGACATTGGATTGGACCTTCATTTCTCATGAA[A/G]TTGTATTCTGAAACAATGATTCTGATCTGTGGGT	A	G	0.57	0.48	0.45	0.34	0.54	0.59	0.44	0.35	0.54	0.43	0.51	0.003	0.000	0.002	0.008	0.0009			
rs1032873	7	9.40	GTACTTCTCTGATGCTTCTGGGCTTGTTGAAATTTTATACCTTGATGCAAAGGTC[A/G]TGGTATCTGTACAGTCTCTATGGGTAGTCAAAGCTTTTGAACACCTCTCATTTTGCT	A	G	0.21	0.57	0.46	0.46	0.57	0.80	0.30	0.51	0.50	0.44	0.52	0.020	0.001	0.019	0.097	0.0039	1		1
rs719679	7	11.79	AACTCTGGCTGTCTCCAAAAGTAGTAACAATGAAACAGAGAGATGATTTTCCAACA[A/T]TCTCTTCAGTCAAAACTTTCATTTTCTGAACTACAGATAAGGATTGGGGAAAATCT	A	T	0.33	0.50	0.56	0.51	0.48	0.36	0.38	0.31	0.32	0.42	0.56	0.028	0.020	0.023	0.020	0.0002	1		
rs769110	7	11.80	AGGTGATTTGCATTTTTCCCTCAGTATGGCTTATTCTCACCATCTCTAGGGCCTTTT[A/T]TGACCTAACAACAGAGAGTGGTTTACAAAGATAACAGACAACCCACTTTTTTACTAT	A	T	0.55	0.65	0.67	0.59	0.61	0.77	0.68	0.57	0.64	0.63	0.71	0.004	0.002	0.000	0.013	0.0031			
rs769111	7	11.80	GAAAGGTGATATGACTTGCCCAAGGTCGTGTAATAAGTTAGTAACAAAGCTAGAA[A/C]TGGAATCTATGCCTCCTTACTGTAATCAGGGATGGTGCTGATATGTAATAAAGGTCC	A	C	0.55	0.64	0.66	0.58	0.59	0.75	0.68	0.57	0.61	0.61	0.69	0.003	0.002	0.000	0.011	0.0026			
rs2051930	7	13.58	GCTTCACTTGATTGCTGAATTCTGTGGTTCTATGGAACTTTGGTTCTCTTTTC[A/G]ATGGCATAATGAAACTCATAACCAACCTTGGAGACATACTTAAGAGAAGATATTTCTCA	A	G	0.76	0.71	0.72	0.76	0.64	1.00	0.95	0.79	0.71	0.71	0.70	0.000	0.051	0.000	0.096	0.0014	1		
rs1367781	7	14.31	GAACTATAAGAAGGAAACAGTTCTTCAATGTAGAGCACTGACCTATTTTTCAGCCA[A/G]TTAAGAAGGCTGCACCTGAGGGAGCATGCATGGCTATGGTCCTCATACCAGCTATTG	A	G	0.21	0.34	0.35	0.37	0.32	0.41	0.83	0.44	0.39	0.35	0.30	0.010	0.130	0.004	0.002	0.0022	1		
rs917440	7	15.47	AACCTACATGATAGATTGTTCTTCATGCTTCCTGAACTGAGCCATTGCCCTTAA[A/G]TTTCTCAGGGCAAACAATTTTCATACAAGCACATGTTATTTTGTTTGGGGAAAACTGAC	A	G	0.25	0.42	0.44	0.45	0.41	0.39	0.49	0.32	0.57	0.37	0.51	0.011	0.004	0.002	0.001	0.0029	1		
rs726395	7	15.48	CTGAAAAACACACCTGGCTCTTCTTTATTCAAAAAGGACATTGCCAACCAAGCTGTTTTAAGTGTATCAACAGTTA[A/C]TTAACAGTGATGCTCCTGATACACAAAGGAATAACA	A	C	0.57	0.46	0.51	0.49	0.38	0.35	0.55	0.38	0.54	0.31	0.43	0.005	0.001	0.006	0.014	0.0012			
rs1029718	7	15.79	TCCTACCGGCCTAGAAGGCTGGCCCACCCTAGTGGCTGGGCACCTACTTGTTT[C/G]AAGAGTTAAACCAAGGCACGATAGCCCACTTTGTCAGCTGCCTAGAGGGAAGTT	C	G	0.14	0.46	0.37	0.48	0.50	0.68	0.77	0.48	0.50	0.36	0.36	0.027	0.084	0.010	0.052	0.0002			
rs1723804	7	16.53	TTAAAGTGGCCAGCTGAGGTTTATTTAAATAAACTGTCATTGTGTTTAGAAAATG[A/G]AAGTAACCCTGGCATTTTATAAGGTGATTAAACTGTTCTTAAGTTTTATAAAACATCT	A	G	0.17	0.46	0.44	0.48	0.41	0.59	0.48	0.39	0.36	0.42	0.42	0.048	0.001	0.003	0.007	0.0001	1		
rs1375237	7	17.31	TGAAGATTTGCTCCAGTAAAACAAAGGTAAAAACCAAGAAAAAAATGGGGGAAATATAC[A/G]GAAAAATAACCCAGTTCTATTGAAAACAAATCCCAGCG	A	G	0.38	0.43	0.40	0.43	0.48	0.36	0.49	0.38	0.46	0.42	0.34	0.000	0.008	0.000	0.001	0.0017	1		
rs957960	7	18.62	TGAGCGGTGGATGAGATTTTTTTCTAAAGAACCTCTTTGAACACAACCATTCCATTA[A/C]ATAATGTAAACGCAGATATTAAGTGCACATATTTGTTTTGTGATTTAAATGGTAAATC	A	C	0.60	0.35	0.38	0.34	0.39	0.41	0.51	0.55	0.39	0.32	0.34	0.026	0.026	0.016	0.003	0.0005	1		
rs714392	7	19.36	TGATTACTAAATAACTCATGTCAGTCATAGCTAAATTATTTAATGGTGTTTGATA[A/G]GAATCAAAGACCAAATTATAGAATTTGTTCTATGCTAACATACAATTATCTTTTTATGA	A	G	0.43	0.39	0.41	0.30	0.38	0.68	0.72	0.71	0.25	0.38	0.34	0.000	0.043	0.052	0.031	0.0024	1		
rs1858941	7	20.38	CTGTGCTTAGCCTACTGTATGGATAACTCATGCTGATGCCTGGCATATGACTTA[A/G]TTCTTTTCTTGGCTTTTAGCTGATAACCCCCTCTTAGTAAGCAATGAGTCAATCAAT	A	G	0.38	0.40	0.41	0.41	0.43	0.55	0.25	0.29	0.50	0.42	0.44	0.000	0.006	0.005	0.015	0.0009	1		
rs2024046	7	20.48	TCCTAGGAAGGGCTGGCAGACCCAGAATGACTACTTGCATTGT[C/G]CTTTCTCTGTATAAAACCTGAATTTGTGTAACATAATCCTTCTCTGTAATAACATCAGAAACAGCA	C	G	0.76	0.59	0.60	0.57	0.75	0.82	0.84	0.72	0.61	0.60	0.58	0.015	0.061	0.012	0.032	0.0003			
rs1859805	7	21.93	TCTGTGCAGAGGGGAAGTAGAGGCAGTAGCAGAAGTAGTACCCCTGCATTTGAAA[A/G]TCGGGTAACTTCAGGAAGGAAGGAATATTTGACTGAGGTCTTGAAGAATGGTAG	A	G	0.71	0.58	0.49	0.41	0.54	0.85	0.78	0.50	0.71	0.53	0.54	0.024	0.072	0.001	0.087	0.0019	1		
rs955484	7	21.94	TCATAAACATCATCCAGTACATTCTATATATGGTCTGGAGAAAAAAGACAAATGA[C/G]AGCTCAGTGGTTGAATGCCCACTTTGTAGGGATTAAGACTCATACCATGGCTATCC	C	G	0.50	0.24	0.33	0.26	0.30	0.50	0.18	0.43	0.43	0.32	0.36	0.009	0.030	0.003	0.010	0.0000			
rs740295	7	22.22	GCCACATAGGAGAACTTCTATTGGGCAGGAAAGATTGCAAAGTATACCTATACA[A/G]TTAGGAAGCGTATATTGGAAGATGTGAAAGGATAGACAGATTTAGGACTGTGCTATT	A	G	0.88	0.66	0.69	0.68	0.64	0.95	0.57	0.82	0.75	0.77	0.75	0.021	0.002	0.017	0.071	0.0037			
rs858239	7	23.03	AGTCATAAGCACATGAGTTGTAAGAGGTTGAACTTGAGAGACCAGATCAGGCA[A/G]TGCCGCTTAATACCATCACATGATCCTCCCCGAGGCCCTGTATTTAATTAAAATAGAG	A	G	0.79	0.40	0.37	0.37	0.38	0.23	0.09	0.44	0.43	0.41	0.41	0.076	0.082	0.001	0.028	0.0006	1		
rs798646	7	23.36	GAAACTGAACATCTCCCATAATCCCATTCTTCAAGACAACTGGTTTATCAAGC[A/G]AATAAGTTTTAAAAAGTCACTCAGGGGCATAGAAATGAGATGCTCATACCCCTCAGTTC	A	G	0.52	0.37	0.43	0.44	0.48	0.55	0.56	0.45	0.57	0.38	0.40	0.009	0.023	0.005	0.019	0.0000			
rs1469000	7	23.55	TGACTATATGTATTTGAAATTATCTCCTTGTCTGCATCACCTTGATCCTAAATGAAATCACACAC[A/G]TAGAATTATACACAAAGGAATATCCAAAACAGCTAAGGCCTCCAATAA	A	G	0.57	0.48	0.49	0.51	0.52	0.77	0.28	0.42	0.43	0.60	0.60	0.001	0.026	0.019	0.034	0.0071		1	1
rs39453	7	24.88	TGTCAAACGTGGAGCTGCCAGAGCCACGGTGACTGGGAAAGAGGAGCAGACGGT[A/G]CAGGCTAGGAAACAGAGAGGAGGCTGATCTTCCCACCTTTCCTCAAGTTGGGGT	A	G	0.86	0.65	0.67	0.62	0.75	0.59	0.70	0.69	0.50	0.61	0.60	0.018	0.000	0.000	0.005	0.0017	1		
rs766838	7	25.77	AGCAAGGATTGGCTATCCTACCCTTGGAGCCATACTAAGTGGTTTCTGTCAAAAA[A/G]CTGAGTATCAGACTGGGACATAGGCACTCTTACGTCCTGAAGGGAGGCTCAGATG	A	G	0.29	0.54	0.52	0.59	0.41	0.59	0.59	0.36	0.61	0.55	0.47	0.022	0.006	0.006	0.007	0.0024	1		
rs1725074	7	26.89	AGGGATATCACTATTTGAACACGTTTCTGAAATCTACTCTTTAGCTGAGGGAGGG[A/G]TGGTTTGGAGGCTTCCTCCTTCCCCCAGGGCTAAGATTTTAAAATGAGATTCTAAT	A	G	0.38	0.14	0.14	0.09	0.16	0.05	0.38	0.16	0.21	0.15	0.17	0.032	0.020	0.001	0.023	0.0003	1		
rs1404282	7	27.33	ATACTTTATTAAAATCTTGGATTTTCTTGTCTGATTCCTCCATTATAAACTTCCTA[A/C]AAAGTCAGCACTGAGGTCATGGCTTGCCAGCATCCCCATTATCTATTATAGTGCATG	A	C	0.10	0.24	0.26	0.34	0.30	0.09	0.22	0.21	0.14	0.23	0.25	0.034	0.002	0.005	0.033	0.0002			
rs1860759	7	28.14	AACAATGCCTCAGCATGTTAGAGGGAATATAGAGCCATCCTGAGATGAAGCCT[A/G]AAGGATTCTTCCCCCTTAGTTCTTTGAGGTGCAAAGTGACTTGGTATACGTATTGGGG	A	G	0.50	0.58	0.60	0.56	0.50	0.82	0.53	0.56	0.50	0.57	0.49	0.004	0.003	0.000	0.032	0.0052	1		1
rs7313	7	28.81	ATTTTCAATCCCGCTCTCCCATTCCCACAACCTGCCTGCTCCCAAGCATCTTCTACCTGATGGAAGTCACCC[A/G]CTTGCCGGATGTAACCAGCCACTTCACTGTCAGATTTA	A	G	0.31	0.42	0.50	0.48	0.34	0.05	0.34	0.33	0.42	0.45	0.49	0.025	0.019	0.022	0.141	0.0005	1		
rs1568658	7	28.88	TGAATTAATACATTGTGGTAAATTCGTAGAGTGCATACTGTTGAGCAAAATAATG[A/G]TATGTATTGATATAAAGAGATCTTCACAACATATTGTTAAGTGAATAAGCATATATAGA	A	G	0.36	0.72	0.69	0.63	0.75	0.27	0.92	0.65	0.82	0.64	0.67	0.044	0.059	0.001	0.075	0.0002			
rs245906	7	28.94	GTTTTATGAAACATTAGTCTAATAGGTTCCAGAATGTACTGCTCATGTCTGTCACC[A/G]TGAACATCTGGCACCAACTTCACACACTGTGGGTGGCCAGTACATATTTAGATCA	A	G	0.74	0.60	0.58	0.72	0.54	0.77	0.53	0.52	0.50	0.51	0.58	0.016	0.000	0.003	0.038	0.0000			
rs13834	7	29.70	[illegible]	[illegible]	[illegible]	[illegible]	[illegible]	[illegible]	[illegible]	[illegible]	[illegible]	[illegible]	[illegible]	[illegible]	[illegible]	[illegible]	[illegible]	[illegible]	[illegible]	[illegible]	[illegible]	[illegible]	[illegible]		
rs255139	7	30.49	[illegible]	[illegible]	[illegible]	[illegible]	[illegible]	[illegible]	[illegible]	[illegible]	[illegible]	[illegible]	[illegible]	[illegible]	[illegible]	[illegible]	[illegible]	[illegible]	[illegible]	[illegible]	[illegible]	[illegible]	[illegible]		
rs2007818	7	31.40	[illegible]	[illegible]	[illegible]	[illegible]	[illegible]	[illegible]	[illegible]	[illegible]	[illegible]	[illegible]	[illegible]	[illegible]	[illegible]	[illegible]	[illegible]	[illegible]	[illegible]	[illegible]	[illegible]	[illegible]	[illegible]		
rs2049758	7	32.82	[illegible]	[illegible]	[illegible]	[illegible]	[illegible]	[illegible]	[illegible]	[illegible]	[illegible]	[illegible]	[illegible]	[illegible]	[illegible]	[illegible]	[illegible]	[illegible]	[illegible]	[illegible]	[illegible]	[illegible]	[illegible]		
rs724291	7	33.54	[illegible]	[illegible]	[illegible]	[illegible]	[illegible]	[illegible]	[illegible]	[illegible]	[illegible]	[illegible]	[illegible]	[illegible]	[illegible]	[illegible]	[illegible]	[illegible]	[illegible]	[illegible]	[illegible]	[illegible]	[illegible]		
rs731844	7	33.89	[illegible]	[illegible]	[illegible]	[illegible]	[illegible]	[illegible]	[illegible]	[illegible]	[illegible]	[illegible]	[illegible]	[illegible]	[illegible]	[illegible]	[illegible]	[illegible]	[illegible]	[illegible]	[illegible]	[illegible]	[illegible]		
rs325462	7	34.59	[illegible]	[illegible]	[illegible]	[illegible]	[illegible]	[illegible]	[illegible]	[illegible]	[illegible]	[illegible]	[illegible]	[illegible]	[illegible]	[illegible]	[illegible]	[illegible]	[illegible]	[illegible]	[illegible]	[illegible]	[illegible]		
rs343024	7	35.23	[illegible]	[illegible]	[illegible]	[illegible]	[illegible]	[illegible]	[illegible]	[illegible]	[illegible]	[illegible]	[illegible]	[illegible]	[illegible]	[illegible]	[illegible]	[illegible]	[illegible]	[illegible]	[illegible]	[illegible]	[illegible]		
rs2041632	7	36.21	[illegible]	[illegible]	[illegible]	[illegible]	[illegible]	[illegible]	[illegible]	[illegible]	[illegible]	[illegible]	[illegible]	[illegible]	[illegible]	[illegible]	[illegible]	[illegible]	[illegible]	[illegible]	[illegible]	[illegible]	[illegible]		
rs2058675	7	36.52	[illegible]	[illegible]	[illegible]	[illegible]	[illegible]	[illegible]	[illegible]	[illegible]	[illegible]	[illegible]	[illegible]	[illegible]	[illegible]	[illegible]	[illegible]	[illegible]	[illegible]	[illegible]	[illegible]	[illegible]	[illegible]		
rs918980	7	37.34	[illegible]	[illegible]	[illegible]	[illegible]	[illegible]	[illegible]	[illegible]	[illegible]	[illegible]	[illegible]	[illegible]	[illegible]	[illegible]	[illegible]	[illegible]	[illegible]	[illegible]	[illegible]	[illegible]	[illegible]	[illegible]		
rs2007664	7	37.76	[illegible]	[illegible]	[illegible]	[illegible]	[illegible]	[illegible]	[illegible]	[illegible]	[illegible]	[illegible]	[illegible]	[illegible]	[illegible]	[illegible]	[illegible]	[illegible]	[illegible]	[illegible]	[illegible]	[illegible]	[illegible]		
rs722336	7	38.27	[illegible]	[illegible]	[illegible]	[illegible]	[illegible]	[illegible]	[illegible]	[illegible]	[illegible]	[illegible]	[illegible]	[illegible]	[illegible]	[illegible]	[illegible]	[illegible]	[illegible]	[illegible]	[illegible]	[illegible]	[illegible]		
rs2043786	7	38.84	[illegible]	[illegible]	[illegible]	[illegible]	[illegible]	[illegible]	[illegible]	[illegible]	[illegible]	[illegible]	[illegible]	[illegible]	[illegible]	[illegible]	[illegible]	[illegible]	[illegible]	[illegible]	[illegible]	[illegible]	[illegible]		
rs1949880	7	39.33	[illegible]	[illegible]	[illegible]	[illegible]	[illegible]	[illegible]	[illegible]	[illegible]	[illegible]	[illegible]	[illegible]	[illegible]	[illegible]	[illegible]	[illegible]	[illegible]	[illegible]	[illegible]	[illegible]	[illegible]	[illegible]		
rs715283	7	39.96	[illegible]	[illegible]	[illegible]	[illegible]	[illegible]	[illegible]	[illegible]	[illegible]	[illegible]	[illegible]	[illegible]	[illegible]	[illegible]	[illegible]	[illegible]	[illegible]	[illegible]	[illegible]	[illegible]	[illegible]	[illegible]		
rs2036041	7	40.02	[illegible]	[illegible]	[illegible]	[illegible]	[illegible]	[illegible]	[illegible]	[illegible]	[illegible]	[illegible]	[illegible]	[illegible]	[illegible]	[illegible]	[illegible]	[illegible]	[illegible]	[illegible]	[illegible]	[illegible]	[illegible]		
rs756328	7	40.77	[illegible]	[illegible]	[illegible]	[illegible]	[illegible]	[illegible]	[illegible]	[illegible]	[illegible]	[illegible]	[illegible]	[illegible]	[illegible]	[illegible]	[illegible]	[illegible]	[illegible]	[illegible]	[illegible]	[illegible]	[illegible]		
rs722790	7	40.79	[illegible]	[illegible]	[illegible]	[illegible]	[illegible]	[illegible]	[illegible]	[illegible]	[illegible]	[illegible]	[illegible]	[illegible]	[illegible]	[illegible]	[illegible]	[illegible]	[illegible]	[illegible]	[illegible]	[illegible]	[illegible]		
rs963779	7	40.81	[illegible]	[illegible]	[illegible]	[illegible]	[illegible]	[illegible]	[illegible]	[illegible]	[illegible]	[illegible]	[illegible]	[illegible]	[illegible]	[illegible]	[illegible]	[illegible]	[illegible]	[illegible]	[illegible]	[illegible]	[illegible]		
rs1548630	7	41.07	[illegible]	[illegible]	[illegible]	[illegible]	[illegible]	[illegible]	[illegible]	[illegible]	[illegible]	[illegible]	[illegible]	[illegible]	[illegible]	[illegible]	[illegible]	[illegible]	[illegible]	[illegible]	[illegible]	[illegible]	[illegible]		
rs37262	7	41.16	[illegible]	[illegible]	[illegible]	[illegible]	[illegible]	[illegible]	[illegible]	[illegible]	[illegible]	[illegible]	[illegible]	[illegible]	[illegible]	[illegible]	[illegible]	[illegible]	[illegible]	[illegible]	[illegible]	[illegible]	[illegible]		
rs273093	7	41.18	[illegible]	[illegible]	[illegible]	[illegible]	[illegible]	[illegible]	[illegible]	[illegible]	[illegible]	[illegible]	[illegible]	[illegible]	[illegible]	[illegible]	[illegible]	[illegible]	[illegible]	[illegible]	[illegible]	[illegible]	[illegible]		
rs1203539	7	41.49	[illegible]	[illegible]	[illegible]	[illegible]	[illegible]	[illegible]	[illegible]	[illegible]	[illegible]	[illegible]	[illegible]	[illegible]	[illegible]	[illegible]	[illegible]	[illegible]	[illegible]	[illegible]	[illegible]	[illegible]	[illegible]		
rs2007475	7	41.63	[illegible]	[illegible]	[illegible]	[illegible]	[illegible]	[illegible]	[illegible]	[illegible]	[illegible]	[illegible]	[illegible]	[illegible]	[illegible]	[illegible]	[illegible]	[illegible]	[illegible]	[illegible]	[illegible]	[illegible]	[illegible]		
rs2024031	7	41.68	[illegible]	[illegible]	[illegible]	[illegible]	[illegible]	[illegible]	[illegible]	[illegible]	[illegible]	[illegible]	[illegible]	[illegible]	[illegible]	[illegible]	[illegible]	[illegible]	[illegible]	[illegible]	[illegible]	[illegible]	[illegible]		
rs1476666	7	41.74	[illegible]	[illegible]	[illegible]	[illegible]	[illegible]	[illegible]	[illegible]	[illegible]	[illegible]	[illegible]	[illegible]	[illegible]	[illegible]	[illegible]	[illegible]	[illegible]	[illegible]	[illegible]	[illegible]	[illegible]	[illegible]		
rs757911	7	41.81	[illegible]	[illegible]	[illegible]	[illegible]	[illegible]	[illegible]	[illegible]	[illegible]	[illegible]	[illegible]	[illegible]	[illegible]	[illegible]	[illegible]	[illegible]	[illegible]	[illegible]	[illegible]	[illegible]	[illegible]	[illegible]		
rs861677	7	41.82	[illegible]	[illegible]	[illegible]	[illegible]	[illegible]	[illegible]	[illegible]	[illegible]	[illegible]	[illegible]	[illegible]	[illegible]	[illegible]	[illegible]	[illegible]	[illegible]	[illegible]	[illegible]	[illegible]	[illegible]	[illegible]		
rs846319	7	41.83	[illegible]	[illegible]	[illegible]	[illegible]	[illegible]	[illegible]	[illegible]	[illegible]	[illegible]	[illegible]	[illegible]	[illegible]	[illegible]	[illegible]	[illegible]	[illegible]	[illegible]	[illegible]	[illegible]	[illegible]	[illegible]		
rs846263	7	41.86	[illegible]	[illegible]	[illegible]	[illegible]	[illegible]	[illegible]	[illegible]	[illegible]	[illegible]	[illegible]	[illegible]	[illegible]	[illegible]	[illegible]	[illegible]	[illegible]	[illegible]	[illegible]	[illegible]	[illegible]	[illegible]		
rs846294	7	[illegible]	[illegible]	[illegible]	[illegible]	[illegible]	[illegible]	[illegible]	[illegible]	[illegible]	[illegible]	[illegible]	[illegible]	[illegible]	[illegible]	[illegible]	[illegible]	[illegible]	[illegible]	[illegible]	[illegible]	[illegible]	[illegible]		
rs1010233	7	42.87	[illegible]	[illegible]	[illegible]	[illegible]	[illegible]	[illegible]	[illegible]	[illegible]	[illegible]	[illegible]	[illegible]	[illegible]	[illegible]	[illegible]	[illegible]	[illegible]	[illegible]	[illegible]	[illegible]	[illegible]	[illegible]		
rs1029482	7	43.00	[illegible]	[illegible]	[illegible]	[illegible]	[illegible]	[illegible]	[illegible]	[illegible]	[illegible]	[illegible]	[illegible]	[illegible]	[illegible]	[illegible]	[illegible]	[illegible]	[illegible]	[illegible]	[illegible]	[illegible]	[illegible]		
rs2014453	7	43.88	[illegible]	[illegible]	[illegible]	[illegible]	[illegible]	[illegible]	[illegible]	[illegible]	[illegible]	[illegible]	[illegible]	[illegible]	[illegible]	[illegible]	[illegible]	[illegible]	[illegible]	[illegible]	[illegible]	[illegible]	[illegible]		
rs740094	7	44.41	[illegible]	[illegible]	[illegible]	[illegible]	[illegible]	[illegible]	[illegible]	[illegible]	[illegible]	[illegible]	[illegible]	[illegible]	[illegible]	[illegible]	[illegible]	[illegible]	[illegible]	[illegible]	[illegible]	[illegible]	[illegible]		
rs1852210	7	45.74	[illegible]	[illegible]	[illegible]	[illegible]	[illegible]	[illegible]	[illegible]	[illegible]	[illegible]	[illegible]	[illegible]	[illegible]	[illegible]	[illegible]	[illegible]	[illegible]	[illegible]	[illegible]	[illegible]	[illegible]	[illegible]		
rs2054789	7	45.99	[illegible]	[illegible]	[illegible]	[illegible]	[illegible]	[illegible]	[illegible]	[illegible]	[illegible]	[illegible]	[illegible]	[illegible]	[illegible]	[illegible]	[illegible]	[illegible]	[illegible]	[illegible]	[illegible]	[illegible]	[illegible]		
rs1486155	7	46.04	[illegible]	[illegible]	[illegible]	[illegible]	[illegible]	[illegible]	[illegible]	[illegible]	[illegible]	[illegible]	[illegible]	[illegible]	[illegible]	[illegible]	[illegible]	[illegible]	[illegible]	[illegible]	[illegible]	[illegible]	[illegible]		
rs723149	7	46.32	[illegible]	[illegible]	[illegible]	[illegible]	[illegible]	[illegible]	[illegible]	[illegible]	[illegible]	[illegible]	[illegible]	[illegible]	[illegible]	[illegible]	[illegible]	[illegible]	[illegible]	[illegible]	[illegible]	[illegible]	[illegible]		
rs2013523	7	46.86	[illegible]	[illegible]	[illegible]	[illegible]	[illegible]	[illegible]	[illegible]	[illegible]	[illegible]	[illegible]	[illegible]	[illegible]	[illegible]	[illegible]	[illegible]	[illegible]	[illegible]	[illegible]	[illegible]	[illegible]	[illegible]		
rs921630	7	47.54	[illegible]	A	G	0.60	0.51	0.54	0.57	0.54	0.41	0.24	0.65	0.64	0.57	0.62	0.000	0.035	0.021	0.002	0.0040	1		1
rs1474283	7	47.69	TACAAATGTTAACTGTTATGCTTGTTATTAAAAATTCATCAAGTGTCCGTTC[A/G]TTCTTATTTTCCAGAAGGAGTCTATATGCTCAAGGCTGTTATTTATAACGAGTTTCATGG	A	G	0.21	0.36	0.39	0.41	0.43	0.23	0.18	0.48	0.36	0.41	0.35	0.027	0.039	0.001	0.031	0.0003			
rs1529867	7	48.56	AACTGCCTTTCACGTTGTAGTTTTCATCATCTTGCTCCTCTGTGTGGTAACT[A/G]GGGAAACCTCCCCTAGGCTACAGGTTAGGCAGATTCCAATATCCAATTAGTTGGTATA	A	G	0.26	0.50	0.48	0.50	0.52	0.55	0.39	0.50	0.43	0.38	0.46	0.038	0.008	0.000	0.000	0.0022	1		
rs1529590	7	49.11	TGTTTTGCTCATTTATGTTTTGCATATCAGAAATACATTTCTCAAAAGTGTAA[A/G]ATGATTAGGGCTACAGATGGCTGCACTGAAGTAGTGATAGTTTTATTATCAGCTTTATA	A	G	0.40	0.42	0.45	0.33	0.36	0.50	0.94	0.49	0.29	0.41	0.39	0.000	0.168	0.002	0.003	0.0006	1		
rs730652	7	50.49	TATCAGATTGTCTATTAATGTTAACTTGACGCCAAAATATTTTCCATGGGGCTG[A/C]TCCCACTGGGAGGAATAGTGAAAGGTGAGAAAAAACATTTATTATTAAAAAAGTTTGA	A	C	0.50	0.54	0.53	0.50	0.64	0.27	0.60	0.54	0.39	0.53	0.53	0.014	0.000	0.002	0.048	0.0002	1		
rs2043731	7	50.91	TGTAACAAATTGGCAGACGAGATTATGAGGCCAAGGCCAAAATGGGAGGA[A/C]TTGCTTCCAACATGACTCAGAGAAACTCACTGTAGTTATTGTTTTGGGGAAAGTTTCC	A	C	0.67	0.60	0.63	0.59	0.51	0.50	0.56	0.61	0.71	0.65	0.58	0.011	0.004	0.004	0.012	0.0035			
rs616185	7	51.58	TCTATGGTGGTAAGTAGTTCGTCAAGCGAGGATAGCCCATAATTTATCAAAGACCTGGA[A/G]ACATTTCCAATACAATTGATTTCATTTCTCAATTAAAAAGGTCAATGTCCTTGAATT	A	G	0.76	0.65	0.59	0.59	0.54	0.59	0.74	0.54	0.36	0.60	0.65	0.013	0.014	0.003	0.000	0.0000	1		
rs169902	7	52.39	TATTGCCAGCCTATCTAGGTATACCAATTTCATGGAAAACCTCACCTATCTGTGA[A/G]TATGTTTATCTTTCTCTAAATTGTAGGGCAAGAAACAAAATGTTCAAAAATATTGGATTC	A	G	0.52	0.51	0.45	0.52	0.54	0.73	0.56	0.46	0.46	0.40	0.52	0.002	0.013	0.000	0.051	0.0031	1		
rs2049410	7	53.95	ATCTTAATTTTAAATTTTTCAATGGTGAATTCTTACGATGAGAAGATCTTGTC[A/G]GAATGTGATCCACTTTGCAGTCTAGTCTTTTTAAAGTACATTTAGGTCTTTGTTAACCT	A	G	0.40	0.24	0.26	0.20	0.23	0.27	0.57	0.28	0.32	0.18	0.18	0.014	0.047	0.001	0.000	0.0023	1		
rs1532083	7	56.34	TAATTTTCTGATGTTGAGTAAGACACAAGCATCAGTTAAATGCTTTGTCACACTTATTAC[A/G]TTTGTAGAATTTCTCTTCATGTATGGATTCTCTGGTGTTATGTAAAG	A	G	0.57	0.62	0.75	0.71	0.73	0.77	0.68	0.79	0.68	0.70	0.77	0.037	0.011	0.004	0.001	0.0004	1		
rs1105305	7	56.38	AAAGAACAATAAATATTGTGAACTGGTAATTTTACCTCAAACAGAATATCAAGGAA[A/C]GAACACTAGAACCTAACTGAGAACTCACGGAGAAACCTGAGGAACCGAAGGAA	A	C	0.60	0.67	0.75	0.73	0.73	0.95	0.73	0.86	0.68	0.71	0.78	0.031	0.004	0.000	0.026	0.0003			
rs2009526	7	57.66	AACAATCGTGTGACACGGATCTCCAGGAAGTGTCTCCTGACGGACTGTCGAAGTC[A/G]TCTTTCTTGAAGACATTTGGCCAGAGCGAGAGGCATCCAGGCCCCTGAGAAACAC	A	G	0.86	0.70	0.69	0.71	0.66	0.82	1.00	0.76	0.79	0.76	0.73	0.030	0.129	0.006	0.043	0.0015	1		
rs517258	7	64.15	ACTCAAGGCCAAGTAAGAAGTCATGTCATCTGGCTCCAGCAAACTCAAATGCAAT[A/C]TCAGTTGAAATCTCATCAGCCCAAGGACCCAATAAAGAAATTATTTATCTTACAA	A	C	0.60	0.64	0.58	0.63	0.66	0.73	0.77	0.64	0.69	0.66	0.62	0.000	0.102	0.002	0.108	0.0014	1		
rs1543851	7	64.22	TGGAAAAGTTACTATTACCATTTGGTTTTGTATTTTCTTTCTGTGTTTCCTGTGGAT[A/G]TGTTTTTCTTCATTTGCTCTGTGTCTTCATTTTTTTTGGCGATTTTGTAGTGACATGCT	A	G	0.86	0.65	0.58	0.61	0.70	0.73	0.69	0.68	0.69	0.64	0.64	0.046	0.006	0.006	0.009	0.0025			
rs1352081	7	64.23	CTGACAAACATATTGCTAATTTCAAAACACTTAATTCTCAGATGCCCTAGGAAAAA[C/G]CATAGGCAGGGGAAGTATCACTGCAAAGTAGCACACAGAAGACTCTGGAGGGG	C	G	0.48	0.24	0.20	0.22	0.30	0.59	0.70	0.41	0.27	0.30	0.22	0.040	0.141	0.029	0.071	0.0006			
rs13536	7	65.43	TCACTTCGTCTTTGTGGTCGAAGTAGATGCAGTACCCAATAAAAAGGGCCCCAGC[A/G]TCATTCTTTAATTAAACTTCATTTAGCTGCCATCTGTCTCGGTTCGAAATGGCAT	A	G	0.64	0.67	0.63	0.65	0.59	0.36	0.55	0.60	0.61	0.51	0.66	0.001	0.000	0.002	0.023	0.0011	1		
rs801193	7	65.44	TACCTAAGAATACTGAATGATGCATAAGTTTTGCAGAAAATACATGAACTA[A/C]AAGGACAGATTTGGGCACTGCAGTGTATACTTGTAGTCCCCACTACCCAGGAGGCGGC	A	C	0.45	0.50	0.52	0.46	0.59	0.86	0.49	0.61	0.50	0.61	0.51	0.005	0.004	0.001	0.058	0.0006			
rs956523	7	66.24	AAATGCTGAGGGCAGCCCCTGCCTGTCTCCTGTGTCAATGATGAGAAATCGCC[A/G]ACCTTGAACAAGTGGATTAAGTCTCCCACAGGCTGTATTAGACTCCAGATCCCTCAGAG	A	G	0.45	0.61	0.67	0.70	0.68	0.95	0.86	0.75	0.71	0.59	0.56	0.025	0.033	0.009	0.076	0.0041	1		1
rs1842746	7	66.79	TAAAATGTTTATGATTCTCTTCCTG[A/C]AAACTTAGTCCTTGCTGGAATAGAAGTTCTGGTTTCTAAGGAGGAAATTCTTCCACTAGGGGTCACAGTAAGAATTCCACTAATGTT	A	C	0.69	0.76	0.65	0.66	0.80	0.57	0.79	0.67	0.68	0.61	0.52	0.001	0.003	0.000	0.007	0.0060	1		1
rs1874243	7	67.24	GAAGATTGTTGGTTGTTGGTTAGGAGGACACATTTTCAG[A/G]CATTTGCCATCTCTTCTGAAGGGAATTGATTCAAGATCCTAAAGAGAGTGATCGTAAGACCCTGAGAAACTCA	A	G	0.45	0.61	0.54	0.61	0.66	0.86	0.84	0.64	0.46	0.53	0.52	0.003	0.061	0.007	0.062	0.0001			
rs1468588	7	68.52	AATGAACAATATTCTGAGGTCAAATGAATTTCAAAAATGCTGGGTTAAACCAAA[A/G]TATTTGAAGGCAGGTAATCTCAGGGCATTCAATATGCTAATATTAAATTGAATTTCAAG	A	G	0.19	0.31	0.33	0.32	0.30	0.18	0.25	0.33	0.43	0.28	0.30	0.013	0.006	0.001	0.016	0.0011	1		
rs740166	7	69.55	GAGCAGTCTGCCACTTCTGCAGAAAGAGACAGAAAGGGCCACTGTGTTTGTTAATTAGGAGGTCCTCGGCCAGAGACCTGCTAAGCTC[A/G]TAAACGTTAAAGGCCAGAGTT	A	G	0.40	0.60	0.63	0.60	0.68	0.64	0.64	0.73	0.64	0.59	0.61	0.039	0.006	0.003	0.003	0.0002	1		
rs678798	7	70.60	GTCATCCAAACTCGTGCCTACTCTTGACTGCTGTGAGATAAGTATGAGTGAT[A/G]ACAGCTTCCTGGAAGAAAGCCCCTCGATCTTTACGGAGGACATAGCCACTGCTTTCCA	A	G	0.36	0.48	0.45	0.46	0.41	0.09	0.46	0.24	0.43	0.46	0.43	0.009	0.006	0.048	0.131	0.0002	1		
rs3135677	7	73.07	GCCAGGAGTCGAGAGCAGCCTGGGCAACAGCAAAACCCTGTCTATGAAAAAAA[A/G]GAAAGAAACTGGGTTGGCCCATTCAGATTGAATTGTCATGTGCCCAGAGGGCC	A	G	0.67	0.70	0.70	0.68	0.77	0.95	0.75	0.74	0.79	0.68	0.72	0.000	0.002	0.001	0.081	0.0000	1		
rs1859293	7	74.99	CTTTAGCTGGGTCCAGAGAGTGACGAGGGCACCAAGACAGAGAATGGGCTCAAAGA[A/G]GCAATGTTATTAAGAATATCATCTGCTTGTCTGGTGTTTTTCCTTGGAGGTACACC	A	G	0.29	0.40	0.41	0.39	0.30	0.55	0.38	0.44	0.46	0.43	0.45	0.011	0.004	0.003	0.003	0.0009	1		
rs917424	7	75.60	AATGCCACTCAATTGCACTTGAAATCTGATGTGACTATGTGACAGTTATTTAC[A/G]CAACTTTTACCTCGATAAAAACACATCGTTTTCTCCACTCTTTTCAATAATTTAAAGATT	A	G	0.19	0.23	0.21	0.16	0.20	0.14	0.32	0.41	0.14	0.20	0.15	0.000	0.013	0.020	0.020	0.0034	1		
rs1860148	7	75.62	ACATTGAGGTTCAAAGACACACAACAAAT[A/T]CATGTCCACCAAATTCGGATCCCCTGCTGCTGTGGGATGCCAAAATGAACAGGATGTGGACTCTGTACCCAGAGGAGACA	A	T	0.90	0.79	0.80	0.84	0.80	0.82	0.64	0.60	0.86	0.82	0.83	0.008	0.026	0.020	0.000	0.0013			
rs740158	7	76.67	AGATATCGCTTTCCCATGGACTCTAATTTGTCTCCATAGTCTTCTAGCAG[A/G]TCCAGAGATGGATTCTTGACTAAAAAGATAGCGTGCTGATTGTCTTCTTAAAGAAAAAT	A	G	0.86	0.51	0.51	0.52	0.55	0.91	0.68	0.75	0.64	0.54	0.49	0.046	0.002	0.025	0.078	0.0001	1		
rs798332	7	77.52	ATCTGAACACAGGAACATGGGTTAGTCTCCTTAATGCTGGCTCAGGCCTATCA[A/G]TTGGTTTCATCAGCTCCCACCTGACTTCTCCCTTCTTATTTCCTAGCTGCAGCTAAC	A	G	0.83	0.60	0.66	0.62	0.64	0.77	0.64	0.79	0.61	0.68	0.68	0.016	0.000	0.014	0.009	0.0000	1		
rs714438	7	77.59	GATCGATAGAAAACAACATTTTTTAATGCATGCCTAGTGGCTCTGAATTTTTTGAA[A/G]TTGAATCTATGTACCTTGTAGGTCCTGGGTGCTTCATGACATGATTGATGGTGCAC	A	G	0.38	0.24	0.27	0.29	0.25	0.68	0.73	0.40	0.43	0.26	0.29	0.001	0.072	0.003	0.065	0.0004			
rs1034699	7	79.32	AGCCACCATGCCCAAATACAACTTCTCTGATGATGACTTTTTCATCCTCCAGATT[A/G]GAACTTTTCTCTTGGTTATATCTTCTCATAACTCCTGTAGTCTCCTTTGCATCTGTAT	A	G	0.55	0.72	0.68	0.76	0.80	0.68	0.50	0.70	0.75	0.67	0.69	0.018	0.030	0.001	0.001	0.0003	1		
rs1468242	7	79.41	TTAAAATGATACTAGAAATACAAAAGGATTCCCTCAGGAGGAAATTTTAGCCT[A/G]TTAAGTCTCCAACTTGCAAACGTTAAAAGGTTTTAGGCCTTTTAACAGTCACAACAGAA	A	G	0.45	0.61	0.60	0.62	0.68	0.68	0.73	0.65	0.54	0.56	0.61	0.014	0.004	0.000	0.001	0.0001			
rs2030711	7	79.83	AAACAAGTATGAACAGAAAATGTATAAGTTTTCTTTATCATCGAAGAGACTATAGCA[A/G]TGAGTTATTAAATGGCTATTTGAAATGTATCTCACACACAAATATAAAGATATCTT	A	G	0.76	0.68	0.63	0.62	0.79	0.77	0.80	0.60	0.68	0.65	0.71	0.008	0.015	0.000	0.011	0.0023	1		
rs2040902	7	80.67	CTTAAAAGAATAAACTCTAGTGGAGGATTAAAGGATTGTTGGGGGATGGATAA[A/G]ACCAGAGTAAAGGCACCAGTAATGACTGTTATGATATGGTCATTGTGTATGTTGCCT	A	G	0.33	0.68	0.53	0.57	0.61	0.36	0.03	0.44	0.71	0.48	0.58	0.029	0.202	0.018	0.025	0.0008	1		
rs1029847	7	81.21	TTTTTATTAAGGGAGTTAAGTTGTTTTCATCTTTACATCACCTATTGGTAATATTT[A/G]TAGTTTTTCCTAAGTGAACACTATAAAGTTACCATTATTCAGTTTCTCATATGAAAAC	A	G	0.86	0.74	0.77	0.74	0.79	0.86	0.84	0.72	0.79	0.78	0.82	0.008	0.003	0.002	0.007	0.0023	1		
rs258651	7	81.33	TGATTCTAATGAAGAATATCTTATGATGTGAGTGTTTAAATAAAATTAGGAGATTAAG[A/C]AGAAGGAATATTGCTTTACACAACATAAACAGATTTACATTCTAATTGCTCTATGTA	A	C	0.55	0.68	0.64	0.61	0.68	0.64	0.70	0.73	0.73	0.54	0.68	0.71	0.001	0.014	0.007	0.001	0.0043		1
rs28156	7	82.26	TCTTGACATGGTTTAAGGCACTACATAAGGCATTTGAATTGCCATTGGCTCTGCT[A/G]TCTGTCCTAGGGGGTGCTAGTTTTTTGCACAGCCTAAAGGGACAGTTGATATGTAA	A	G	0.67	0.38	0.40	0.33	0.48	0.73	0.96	0.46	0.46	0.45	0.40	0.054	0.209	0.002	0.064	0.0006	1		
rs42002	7	82.87	TGGGCAAATATGCCATTTTATTTGATTGCTGTCAGCCCTTCTAAGGATTTTCTCAT[A/G]GACTCTGTTGAACCAACTTTTTCCTTTATTTCATGCACGTGGTTTGTTTGCTGAAGTT	A	G	0.88	0.61	0.53	0.55	0.41	0.55	0.79	0.70	0.57	0.54	0.58	0.071	0.044	0.023	0.000	0.0011	1		
rs917089	7	83.34	TTTACACTGCAATTGCATTTGTATCAGTA[A/C]AAACACATCTTAGCAATTTAGATATGATATATCTTCTGGCTTGTTATAATTGCATGTGTATGCTTTTACAGCAAGACTGTTAGA	A	C	0.31	0.51	0.52	0.49	0.50	0.45	0.53	0.38	0.57	0.49	0.56	0.017	0.003	0.014	0.000	0.0000			
rs473880	7	83.52	CAACAAAAGGTTTTCTAATATGAAATAGTAGACTATATGTGAAAACTTCTTGAA[A/G]TTAGTCAATACCTAATAGTTGGAAGCCAGTGTATTCATCTAGGAATGTGTGATATTGGC	A	G	0.45	0.54	0.56	0.50	0.61	0.77	0.45	0.46	0.46	0.52	0.53	0.011	0.011	0.019	0.015	0.0005			
rs612774	7	83.59	GCCACATCAGTTATTAATCATTCATTACATCCACAACTATTTATTGAGATCAAAT[A/C]AAGAAACTGATCTGTCATGATGAAAAGGTATGAATAAGAATCTTTGGATTGGTAAAG	A	C	0.74	0.52	0.52	0.60	0.55	0.59	0.81	0.70	0.32	0.60	0.51	0.035	0.075	0.036	0.012	0.0007			
rs764077	7	84.38	CAAGTTTTTAGAACCACATTTTTTTTTCTAACATCACTTGAGAATATCTAGAAAACCCCTGTGTGAGAGAAGGGCCACGAGATAATGGT[A/G]AGAATTTGAACTTTGGAGTC	A	G	0.81	0.47	0.48	0.44	0.54	0.65	0.22	0.57	0.57	0.47	0.53	0.079	0.025	0.019	0.026	0.0018	1		
rs1024516	7	85.89	AAGTCCCACTTCCCCCAACACCCAAAATATATTTTCACTACCTTCCTTGGTTCATA[A/C]AATTTTCCAAATTTTAAACTGAAGTATTTCATCCAAGAAGTACTAAAGAACATATAG	A	C	0.88	0.70	0.65	0.71	0.80	0.27	0.29	0.36	0.64	0.64	0.65	0.044	0.058	0.037	0.069	0.0001	1		
rs1922242	7	86.79	ACAACAGGCTAGTTAAAAACTTTTATATGTACAATTCTTACATACGCACAAAAATT[A/T]GTAAAGGAATATATCCTATCCTTATTCCTTATCAATGAATAAGTGATGACAATTTTGCT	A	T	0.58	0.58	0.60	0.61	0.43	0.68	0.61	0.74	0.57	0.61	0.56	0.000	0.000	0.016	0.004	0.0007	1		
rs1202169	7	86.81	GACTAGCATTAAATGAAGGACTGGGCTTTGCCAGAATGAAGAAATCCTCTGAG[A/G]ATGTGCAGTAGAGCAAACAAGATACTTTCTGAGGAAATTTCTGAGCAATTTGAAATT	A	G	0.76	0.58	0.52	0.50	0.75	0.41	0.49	0.41	0.50	0.51	0.58	0.037	0.000	0.004	0.004	0.0016			
rs1637503	7	87.20	TGCCAGGAGGCAGCTTCCCAGAAACCCAGTGGGGGAAGTTCGGAGCGACAACCC[A/G]CCCAGTTTTCAGAAGTGAACACGGATCTTTCCTCCTAGGGTATCATTTGATAAATTAAATAA	A	G	0.52	0.49	0.42	0.50	0.46	0.50	0.59	0.56	0.36	0.53	0.53	0.009	0.020	0.016	0.006	0.0056			
rs1688886	7	87.22	CAGAAACATGGGGAAGGTAGGTTCCTGAATATTGGAAGACCACAGAAAATGC[A/G]AAGAAACCAGGCTCACATTTATTTTAGTGACCACCACTGCAAGTTTGGTTTGGGTGAG	A	G	0.70	0.51	0.58	0.49	0.54	0.50	0.46	0.42	0.64	0.47	0.46	0.006	0.008	0.017	0.005	0.0068		1	1
rs1637489	7	87.23	TTTCTGAGATTAGTCTCTAGCTTTTAATCTTATAATTACATTCCTGCCACTG[A/G]TAGATCCCCCTGCAGTGACCTGCAGACAGCTGCTCTGCCGGGCAGTTTCCCTTCACTAGT	A	G	0.76	0.51	0.58	0.49	0.54	0.50	0.46	0.41	0.64	0.47	0.46	0.015	0.008	0.017	0.005	0.0062			
rs1468121	7	87.96	GACAAACCAGGGAGCCAGCATTTTGTAGAGCATATTAGCAAGAAGCAAAATCACT[A/G]CAGAACTTACCTAAGCTACAGTATTAGACTCAACTTTGAGTACTTGTTGATGTAAA	A	G	0.31	0.71	0.70	0.70	0.73	0.32	0.65	0.67	0.68	0.67	0.68	0.056	0.001	0.000	0.061	0.0000	1		
rs717474	7	88.40	TATGATTTTGTCATAAACAGCATGTTGATAATGATCTACACAGAATAATGAATGA[A/G]TAATGTGATGAATAAGAGGAACATATTTAGGATGTGTCATATGATATGTCATCTTTGAA	A	G	0.38	0.48	0.51	0.54	0.48	0.05	0.11	0.43	0.39	0.54	0.49	0.008	0.075	0.001	0.135	0.0001			
rs1023564	7	88.45	TTTTCTTCTTCTCCAAGCGTTCTGCTGTGACTTCCTGTAACAAAAGACAAATAACA[A/C]GATAAGAAACAGAAGTTTGATGACATATGTATACCTCTTGTGCACATGGGAGATAACCT	A	C	0.38	0.43	0.32	0.37	0.43	0.82	0.57	0.35	0.43	0.41	0.35	0.001	0.026	0.003	0.118	0.0001			
rs194506	7	89.45	CACTTTTTATTGTACTGGTGCTATGGAAAATGTGGTGAATGAACATAGTTGTATTTTA[A/G]TACTGAAAAAACAGGACAGGTGAAGACAAATTTTTAAAGAAAGGATTGTAAATGA	A	G	0.88	0.90	0.82	0.88	0.86	1.00	0.98	0.97	0.89	0.81	0.79	0.001	0.030	0.026	0.060	0.0001	1		
rs194518	7	89.47	TGATAGAAGTCCATGGTATTTACTCTTTTGTTATATTTTGATGCCTATTTTCTTAA[A/G]ATATTATTTAAGTGCCAGGATTCATTGTTAGCTAATTTGTTCTAGCTCTCATTTAATGAT	A	G	0.19	0.64	0.54	0.51	0.54	0.23	0.53	0.29	0.57	0.49	0.48	0.075	0.001	0.030	0.065	0.0017			
rs42611	7	89.56	CCGTACACAGCCGTCTGCCACCTCCCTAAAAAAGTTGGGCTGAAGAGAGAAATCATA[A/C]GAATCAAATCTAAACAGGACATTCAAGTCATTTCACATCGTCGAAAATTGTGCA	A	C	0.15	0.66	0.60	0.55	0.61	0.27	0.40	0.41	0.57	0.55	0.53	0.114	0.020	0.011	0.060	0.0005			
rs714498	7	89.78	TAGATAAGACAACCAAGAAAGCTTAAGTTACATA[A/T]CATGTGAAAATCCCTCCTGACACAAGATTGCCCTTCAAGCAACATATCCCTTCAATATTTTTTCCTAATAAAAGGAA	A	T	0.26	0.66	0.59	0.57	0.61	0.86	0.54	0.59	0.43	0.55	0.50	0.067	0.007	0.000	0.032	0.0025			
rs929168	7	89.95	CTAAAACTCAATGTGTAAAAAATTGA[A/G]TTTATAATAGTTTCCTCTTTTCTCCAAGCTTGTATTCTTCCTGTGTTCACTGGTTTACTTTATGAACCAGCCTTTTACCTAGTTATAC	A	G	0.79	0.54	0.49	0.59	0.64	0.82	0.76	0.69	0.50	0.52	0.47	0.042	0.030	0.018	0.058	0.0002			
rs758706	7	90.11	CTCACTCATTCATCCCAAGCCCACAGTGCCCTCCTGTTTCTCACCTTATGCC[A/G]CTCACTAATATCTGCTCTAATTTCCAAGGCACTAATGTTTAATAATTATTATTGCCTTTC	A	G	0.38	0.42	0.37	0.43	0.50	0.68	0.60	0.45	0.50	0.39	0.33	0.001	0.035	0.002	0.052	0.0000			
rs9008	7	91.11	TAGAGACTTTACTAAAGGACTGTATTTTTGACTTCACCTATAATGCAACTAATCAT[C/G]TACTTAGTTGCTATGTTAATCTATTTGCTTCTTCAGCAAATTATCACTGACTCAGTGC	C	G	0.76	0.46	0.48	0.46	0.48	0.55	0.33	0.65	0.46	0.60	0.52	0.037	0.010	0.005	0.002	0.0000	1		
rs722263	7	92.50	TGCAGACAGGTCTTCAATTAGCTGCTGTTATCTGTACAAATGGGAGAAGGTATTGCTTTTGATGATATTTTGTTCTTTTAAGCTG[A/G]ACAATATTTCAAGAATAACTATGGAATCT	A	G	0.19	0.62	0.60	0.65	0.63	0.27	0.29	0.65	0.79	0.67	0.68	0.083	0.046	0.009	0.051	0.0054	1		1
rs1326152	7	94.05	TTGGTAGATTGCATTATTGGCCCAATTATTCAACTACCTCTGTATGACAATCTGTTTT[A/G]TGTCTAGTTTGGATGAGATAACAAAATACCATAGACTGGGCAGCTTAAACAATAC	A	G	0.24	0.57	0.52	0.46	0.38	0.27	0.25	0.30	0.46	0.54	0.48	0.046	0.053	0.034	0.037	0.0006	1		
rs854731	7	94.25	AGTATGACGGCCAAAAAAAAGCTATTGGAAGGAGAGGAGAGAGGACACACCT[A/G]GGTTACATTTGGCCTCTTTTAAGTCTGTGAGACATGTTTAAAAATCTTTAAGAAAGC	A	G	0.55	0.45	0.47	0.49	0.48	0.77	0.63	0.74	0.32	0.43	0.48	0.006	0.029	0.074	0.069	0.0001			
rs961262	7	94.34	TTAATATTGCCACATAAATACACAATCCTATAGAGCATTT[C/G]AGGAATGGATAATCTGGGACCCATATTTCTATCTAGAAGATATTATATGTTTTTAGATAGTTAAGCTTTTA	C	G	0.38	0.53	0.48	0.55	0.54	0.91	0.84	0.74	0.50	0.54	0.49	0.002	0.084	0.080	0.137	0.0001			
rs1859121	7	94.59	CCTTGAGGCAATGTGGCCACCATTGTGCTGCTGGGTCTAGGTTCTTTGAAAA[A/G]TAACCTACAGCATGTTTGACTGGCCCAAAGTTCAGTTAGACCTATAGTGCTATACC	A	G	0.45	0.50	0.53	0.52	0.52	0.32	0.10	0.27	0.43	0.48	0.49	0.002	0.102	0.029	0.017	0.0001	1		
rs1053275	7	94.61	GGAAAATCTTCTTGATGGATCTGAATGAACAAACCCAAGGGCACAAGC[A/G]CTAGAATCAGTGGTGGATTTGACAAGAATTATTTAATCCACATGGAT	A	G	0.55	0.50	0.48	0.48	0.48	0.68	0.91	0.73	0.57	0.52	0.51	0.002	0.111	0.029	0.017	0.0001			
rs1917486	7	95.85	TCACAGCAGCCCATTCTATGTCTTGGTTGGCAGGATGCAAATATCAGGTGCCA[A/G]ACATTTTTTACAGATGAATTTACCATTTCCTTGGAGCAGAATTAGGATAACAGTCACC	A	G	0.40	0.70	0.71	0.80	0.70	0.73	0.78	0.69	0.64	0.65	0.69	0.039	0.002	0.000	0.001	0.0001	1		
rs1229560	7	97.04	GTGTGGAAGCAATGAAGAGCACAGATACATTTTGCTTCTCTTTGGTTTTCCT[C/G]CAAGGAAGCTGGTCAGACTCAGGGGATGGTCATCAGCATCCATAGCAAAAATGTGTTA	C	G	0.60	0.52	0.55	0.45	0.66	0.68	0.65	0.65	0.39	0.68	0.65	0.005	0.021	0.019	0.022	0.0063	1	1	1
rs1047035	7	98.06	TGGCCCAGCGACCTGGGCTGTGGCTGAGACAATAATGAGGCCAGAGTAGCC[A/G]GAGCCTGTCAGGAAGGGCAGGGGAGGACTGTGGGGTCTGGGCTCTGTCGCTGTA	A	G	0.10	0.19	0.22	0.23	0.23	0.27	0.13	0.25	0.36	0.24	0.29	0.008	0.001	0.000	0.009	0.0057	1		1
rs219798	7	98.26	GTCCCCTAGGACACTAGCCCTAAGGCGCTGGTCTTGTGACTCCTCCATGGTGAC[A/G]CCATGCCTGCCTGCTCTCGTCCCTTTCAGAACGGTGTATGAAGACTCCGGGCCTG	A	G	0.24	0.66	0.61	0.60	0.70	0.45	0.86	0.49	0.54	0.61	0.56	0.073	0.037	0.011	0.013	0.0036			
rs1865472	7	99.12	TTCCTGGATCAAGGCTTCCTTA[A/T]CTATAAAATGAAGACAACTGGACCAAATGTCTCTAAATACCATCCCAGCTCTTGAGTTCTATGAATTTCAGTCACTGTAACTTCTGCAA	A	T	0.90	0.52	0.52	0.48	0.46	0.77	0.73	0.57	0.57	0.63	0.62	0.114	0.031	0.002	0.048	0.0042	1		1
rs1617640	7	99.93	CTAAGGTGTGAGAGACCAGCTAGTCTTGGTCTCCTGCTCTGGGAATCTCACTC[A/C]TCTGGCTCAGGGTTTCCAGAAGCCATAAAACCTTAGCTGTAAATCCCCAGCCCCCATC	A	C	0.64	0.55	0.62	0.57	0.55	0.82	0.82	0.65	0.57	0.65	0.67	0.000	0.023	0.000	0.021	0.0016	1		
rs11178	7	100.34	GAAGACAGAAATAGTTTCTACCAGGCACACAAAAGCTCCTGTAAGCCCCGTAGTTCC[A/G]TCCTGCAAAGGGCCTCAGTGGGAACCAGGTCTGCAGCCCGAGTGGGCAGAGAG	A	G	0.58	0.63	0.57	0.59	0.57	0.36	0.83	0.51	0.61	0.55	0.62	0.002	0.017	0.012	0.042	0.0001			
rs201492	7	101.31	ATATGACAAGAGCATGGGAAGTGAAAGAGTCTTACTATTCCTTGGCTCATTTCC[A/G]CTCCTCAGATGGAGTTCATTTGTATTTTCTAAAATGTTTCTGCATGTACAGAAATACC	A	G	0.67	0.61	0.61	0.54	0.59	0.05	0.17	0.54	0.46	0.64	0.67	0.002	0.116	0.001	0.193	0.0022	1		
rs727708	7	102.89	GGGGTACACTGTATCAGCTC[A/G]TCATCTGCAGTTACTAGAATAGCTGTTCCTGCCATGGCAGGGCCTCTGTCATGTGGTTGTGGATGGGTGTTCCACATTTAAGAACGAAAG	A	G	0.60	0.55	0.48	0.51	0.54	0.55	0.72	0.34	0.71	0.41	0.39	0.002	0.017	0.027	0.000	0.0037	1		
rs39400	7	103.05	CCTTCAATATTGCTTTTTCATTCAAATTCTATCATTCCAAGCATCAATCTGAAT[A/G]AGTATATTTCTACCATATGGCTTCTTTTAGGCTTTATGTAGAAAATATTATCTTCATGTG	A	G	0.43	0.34	0.39	0.44	0.34	0.00	0.15	0.14	0.18	0.43	0.44	0.002	0.036	0.055	0.164	0.0020			
rs1010340	7	104.39	CACATTCCTGAACACATTCAGTTGTGTAACCCAAAGTTTATATTGTTTGAATATAGATGGGC[A/G]TCATACTTGCAGTCATTCCAGATGTCAGTGGCTCTTGTCCTCACTTGTCA	A	G	0.52	0.48	0.43	0.49	0.43	0.75	0.48	0.42	0.35	0.54	0.56	0.000	0.000	0.005	0.034	0.0059	1		1
rs234	7	105.12	TTATACTGATGCACCCATCACTCACAGTTACTAGACATGTGGCAGAGACTGAAT[A/G]AAGGGTTGACCCAGGGACACTCCTTGAATATGTCCCCTTTTTTGATCAGCATATCCT	A	G	0.40	0.50	0.49	0.50	0.57	0.32	0.21	0.60	0.54	0.48	0.47	0.001	0.042	0.004	0.017	0.0003	1		
rs41261	7	105.17	GGCAGAAAGGGCATATCCAAGGACAGTCTGGCTTTCACCATACATGGGACAGGC[A/G]GTTCCCAATAAAAATTGCTTGAGAGTCACTGGCTATATCCACTTCTTCAAAATATC	A	G	0.64	0.44	0.43	0.45	0.32	0.64	0.45	0.31	0.39	0.51	0.47	0.019	0.000	0.005	0.018	0.0001			
rs176481	7	105.29	ATGAAAATTGGATAGGAGTATTGAAAATTATAAAAATTGAAGTAGACACCATGAC[A/G]TACAAAAATATAATCCCAAACTGCTTAACGACTAAAAAGTGAAAAACCATATTCAGA	A	G	0.19	0.31	0.33	0.36	0.50	0.27	0.28	0.59	0.29	0.43	0.32	0.012	0.000	0.053	0.000	0.0007			
rs887882	7	105.40	GGAAAAGGGCACAGATCTGAAGGACTGAGGGCCACACTGGACACTCTTTCCA[A/G]TTATGCCACAGCCCGCCTGCTGGAAGTGGAGCCAAACCAGCATTCAGACTCTGTAG	A	G	0.69	0.50	0.62	0.56	0.68	0.23	0.60	0.58	0.54	0.61	0.55	0.012	0.001	0.001	0.056	0.0008			
rs1024761	7	105.65	GATACTGTAACATTCTGAGATCTTAAGATACCCACAGAAGCTGAGTTGGTGT[A/G]AGCTGGAAATGGAGTGCCTGGGGACTGAAAGGCCACAAAGCTCTGTCTAGGAAAGG	A	G	0.05	0.41	0.40	0.50	0.39	0.05	0.49	0.26	0.43	0.46	0.51	0.112	0.003	0.013	0.118	0.0053	1		1
rs342296	7	105.93	TGCAGGACAAGATTGGGTGAAGCTCAATTTGCCAGAGCTCTGCTTTGCCCAGC[A/G]AAGTTTCTTTCAGAGCACTTCCTCCCAAAGACCGCGTTAACCTTTCTCCCTATTGTCA	A	G	0.17	0.46	0.45	0.55	0.48	0.23	0.41	0.35	0.43	0.48	0.38	0.055	0.000	0.017	0.031	0.0005			
rs1476878	7	106.26	TGCCTCATTTAAATAAAGTAACAATTTTCAATACTTCTACCTACAGGTTCCTAA[A/G]TTGTTCAAAAATTACCTGCTAAACCTTAAATTTAAGACCATAACTTGGAGACTGTGTAC	A	G	0.17	0.45	0.49	0.63	0.39	0.36	0.35	0.28	0.39	0.40	0.53	0.055	0.004	0.026	0.004	0.0002	1		
rs257376	7	106.36	ATTATGAAAAGGAACATCGCTACCTATGAAGAACAGTTAGTTGCCCTGTTTGGAAC[A/G]AACATGGATATTGTTGAACCCACTGCATGAAGCAAAAGTATGGAGCAAGACCTGT	A	G	0.48	0.52	0.56	0.60	0.46	0.59	0.60	0.34	0.36	0.61	0.58	0.004	0.002	0.011	0.001	0.0000			
rs1858929	7	106.89	AGAAGTGTGGTCCCAAGTCACATCCCAACTAGCAGGTGATGTCATGCTGAGTC[A/G]CCAACTGTTGGATCAGGGTTTCCAACATCCTGTCTGTCTACAGATGGGGTTCATTC	A	G	0.52	0.43	0.55	0.55	0.57	0.64	0.64	0.49	0.54	0.51	0.54	0.000	0.010	0.001	0.010	0.0006	1		
rs2028030	7	106.92	AGGTGAGCTGTCACAGTAAGTCTTTGGAATAGGCCTGCAAGCTCAGGAACTCT[A/G]AATGTCTTATGTACTTAATGACTGCCATTCTCAAATTATGTTAACCTTACTCAGCATC	A	G	0.45	0.40	0.49	0.52	0.48	0.64	0.56	0.44	0.46	0.47	0.48	0.001	0.004	0.003	0.013	0.0007			
rs441534	7	107.39	TCTAGTATCCTTATGACATTGTCCTTCTCCTTATCTTATTTCTAGGCCCTAGGCTT[A/G]TGTCACCAACGTGAGAAGCTCACATTGTTGAATGGGCAACTTTGTAGCAGGGACTAA	A	G	0.65	0.78	0.76	0.74	0.86	0.77	0.94	0.81	0.69	0.74	0.78	0.007	0.028	0.003	0.000	0.0000	1		
rs916691	7	108.45	TGTCCACAGAGTTTCATGTAGATTATGGACTATGGTGATAATCGGAAGAAAGAT[A/T]GTATTCTCTCTCTAGAAATATGCATATATAAGCAGACATTAAAAATCAATTATACAATTT	A	T	0.79	0.63	0.61	0.59	0.57	1.00	0.99	0.70	0.71	0.55	0.59	0.015	0.127	0.001	0.152	0.0001	1		
rs1013920	7	109.43	CAGAGAATTGAAAGACTTCAAGTTTGGAATAGTTAACAGAAAGCCAGACAGAGA[A/C]AATAAATAGAAGGAAAAACACACAGAGGGAAGTAGGGGAGGATGGTGTAAACAGC	A	C	0.74	0.53	0.56	0.54	0.54	0.73	0.54	0.73	0.36	0.64	0.52	0.016	0.001	0.009	0.011	0.0009	1		
rs719530	7	109.45	TGAACTTTAATAAAGTTTTGTCA[A/G]ACAGTGCCTTTAGTAATTACAGCAAACCTGGTGTTCACTTGCTATCTCAGAATCCTTTAAAGGCATCTATACCTAAGGCCTGATACGGA	A	G	0.47	0.53	0.47	0.46	0.54	0.39	0.40	0.25	0.51	0.57	0.37	0.43	0.002	0.013	0.005	0.000	0.0001		
rs214459	7	110.41	TCTTTATTCATCAGCTTAAAGTTTTCAAGCTGGGTTTATTTCACGTGTAAACTTACA[A/G]TTGCAAAAACTAACAAAATTCCTCAAAACTACTCTTGCTATTTCTAGGTAGAAGTCTT	A	G	0.52	0.51	0.48	0.50	0.52	0.70	0.54	0.79	0.36	0.61	0.53	0.000	0.001	0.041	0.019	0.0018	1		
rs214468	7	110.42	AGGCCAAAGAGCCAGAATAACAATGATAACTATGAAAGATAGTACAAGGTAGAA[A/G]AACGTCTCCTGCCAGATACTGAAACTATCATTATTAGAATAGCATAGCATTAGGC	A	G	0.60	0.51	0.48	0.50	0.50	0.68	0.54	0.80	0.32	0.60	0.53	0.004	0.001	0.041	0.015	0.0018			
rs37742	7	110.47	CTCTGAATCTCACAATATTGCACGTGCACCTCCCTGCATCCTCAAGGTAGAAGTTT[A/G]TATGCTTGTTTCATGATGACCCCATACCCATGGCAGGAACTGTTGTTCTATATC	A	G	0.67	0.51	0.48	0.49	0.52	0.65	0.55	0.80	0.36	0.61	0.54	0.013	0.001	0.041	0.010	0.0018			
rs1476517	7	111.06	AGACCCTGTTCCTGACCTCTCGCTATCACGCCCTAAACTATGTCTCTGCTACCC[A/G]CTACACTTCCTCCTTCCCCAACAACCTCTCTGCATCAAATCTACCCTTTTGTCATTGC	A	G	0.33	0.33	0.34	0.22	0.30	0.45	0.30	0.11	0.36	0.30	0.29	0.000	0.000	0.026	0.013	0.0039	1		
rs7817	7	111.68	GTACATTAAATTATAAGCAATATAACCACTGGCTACAAAGTTATCTAAGTTAATCCA[A/G]GATAAAAACTGTGTCTAGAGTTTAAATACATTGAAATAGAAAAAAAATAGAAATT	A	G	0.45	0.53	0.50	0.46	0.54	0.09	0.21	0.48	0.31	0.51	0.53	0.002	0.045	0.006	0.119	0.0004	1		
rs43077	7	111.80	TCATTTGAAAGCAGAGGATCCCAGAGCTGGACTGTCTTGGGGAAAAGAACTCCT[C/G]GCATTAAAAAAGAGGAGAACAACCTATCTGAGGGAAACAAAGAACATTGGAATACA	C	G	0.55	0.47	0.50	0.54	0.43	0.82	0.53	0.55	0.57	0.60	0.48	0.006	0.001	0.011	0.076	0.0001			
rs2040587	7	114.23	TATATATGCAACATGTCAACAACATTCCCTTAGTTTAAAAATTATGTTGATGTC[A/G]TTTTCAGTTTGCATTTTGTATCTTCTGGTGCTTCCAACTCAGCTAGAAGTACAGATTT	A	G	0.60	0.70	0.66	0.60	0.61	0.59	0.44	0.58	0.71	0.61	0.60	0.005	0.030	0.011	0.010	0.0009	1		
rs2056865	7	115.78	GGTATTGTTTTAGTACTAGTTTTGGTAGCAAGTGAAGAGCACATAAAATTTTCTT[A/G]TTGTTCCAAAATGTCCACCACTTTGTAATAAGAACAAAGTCTGCACAGATCTAAAGG	A	G	0.29	0.65	0.69	0.73	0.75	0.50	0.22	0.55	0.61	0.67	0.61	0.076	0.126	0.018	0.018	0.0052	1		1
rs41736	7	115.99	TTGTAGGAGTCTTCTCCCTTGCACAAGTAAACAGTTATATCAAAGGTGTTTAC[A/G]TCAGGATAAGGTGGGGCTCCTCTTGTCATCAGCTCCCAGAGGAGCACGCCAAAGGA	A	G	0.10	0.45	0.45	0.41	0.39	0.50	0.59	0.46	0.57	0.43	0.48	0.092	0.008	0.000	0.000	0.0000			
rs885993	7	116.68	TTCCAAAAAGAAGCACTACATTAACTATACACAGTTTTTAGAAATTAACAATATTAC[A/G]TACTTATTCATGCAATCACCTACCACTTTCTTCTTCAGCATCGTAATTAAAGTTTTA	A	G	0.24	0.55	0.61	0.55	0.61	0.50	0.51	0.52	0.54	0.70	0.56	0.081	0.005	0.015	0.008	0.0050	1		
rs1896887	7	116.75	AGGGCAGGGAATATGTTACTATGAAGTTTATTGACAGTATACTCCAAATAGTGTTT[C/G]TGATTCAAAAGCAATATCTTTGATAGTTGGCATTTGCAATTCCTTTATATAATCTTT	C	G	0.21	0.19	0.19	0.20	0.21	0.50	0.47	0.42	0.29	0.18	0.22	0.002	0.036	0.041	0.051	0.0057			1
rs38831	7	117.27	GACATCACTATGTTTAGCATATGATTTTTTAGAGAACAGCAACATGGAACCCTA[A/G]AGAGATTTGACATTTAATCATTTGAAACTCCACATAAGAGTTCTGGAAAAACATCAGT	A	G	0.29	0.35	0.32	0.36	0.43	0.68	0.67	0.34	0.46	0.43	0.49	0.002	0.058	0.001	0.056	0.0192	1	1	1
rs1510504	7	117.84	TTTGTCATTTTTGTTGGAATTTGGTAGGACACTGCACAGTTACTAGTGTCTC[A/C]AAATTCAGACGCTGAGTTTGTTGAGATTAAGAAGAGCAGGATGGGTTCATTGCCAGTC	A	C	0.40	0.51	0.57	0.48	0.61	0.23	0.31	0.47	0.50	0.43	0.51	0.009	0.023	0.000	0.048	0.0018	1		
rs846427	7	119.05	ATCGAATTTAATTCGTTTTCAATATTCAGTTGACTTGAACCTTTGTTAAATAGAGGA[A/C]GGTAGAAGTATAAACATCCTTTTACTGGCCAGAAATTTATTTTTCAGTGT	A	C	0.05	0.42	0.44	0.46	0.38	0.41	0.69	0.70	0.46	0.41	0.41	0.130	0.048	0.012	0.001	0.0005	1		
rs868053	7	120.33	ACTGCTCCATGCTCAGGCCACCTTACTTGGGAAAGTACATAAAGTATAAGAAGA[A/C]ATACTATCCTCTGCTCCATCCTGTCATTAGAGTTATCTAGAATATTACTTCATATCATTG	A	C	0.29	0.33	0.41	0.46	0.32	0.14	0.05	0.39	0.29	0.38	0.35	0.002	0.059	0.006	0.026	0.0019	1		
rs12217	7	121.27	TTGCATATAAAAATTAACTCAAAGTTGACCTAAGATCTAAATGTAAGAGCTAATATT[A/G]TAAGTATCTTCAAAGAAAACATGGCGTAAATCTTTCTGACTTTGAATTAGGCAATGA	A	G	0.93	0.51	0.53	0.51	0.57	0.77	0.50	0.61	0.39	0.47	0.46	0.118	0.001	0.011	0.056	0.0057	1		1
rs1320393	7	122.33	CCAAGGTGTTTACCTCCAGAGATTTTAGATTGACAGACGGAAATTATCAGCC[A/G]CTCCCCTGGCAAACTAGCATACATTCTGATTAGTATGTTGGGATTAACACAGTTAACT	A	G	0.62	0.55	0.62	0.61	0.46	0.70	0.22	0.51	0.64	0.53	0.56	0.001	0.049	0.000	0.010	0.0002	1		
rs1880180	7	122.39	CTCCACAGGTGGGGCAATTCCCAGCACTCCATTATGCACAGTCCCTCCTCTTA[A/G]TGGGCAAGAACCCTGCTGATGCTCTCCTCTAACAAGTACTAATCGAGGTTACACCAGT	A	G	0.60	0.59	0.67	0.55	0.52	0.68	0.23	0.63	0.50	0.57	0.67	0.006	0.118	0.007	0.001	0.0000			
rs1121030	7	123.26	GAATTGTATTGACCACAGAACTCTTTCACTCCAAAACTGCAGATTTTTAATACTAT[A/C]CTCTCTGGCTACAAACTCTAACTTTCCACCAATATGTCCATATGGACAGCCAATAG	A	C	0.93	0.70	0.78	0.81	0.71	0.68	0.91	0.82	0.69	0.73	0.69	0.034	0.024	0.002	0.003	0.0067	1	1	1
rs1860487	7	123.27	AGCTTTGTACAAAGACAGAGAAATTCATTCCGATGTTTCTAGGCAGGATTTTGACA[A/G]TAGTACTTTCAATATTCATCTCACTTTTCTTATTAATGCCTCAACGGCTCACACCTT	A	G	0.48	0.52	0.50	0.57	0.52	0.77	0.46	0.57	0.57	0.56	0.57	0.003	0.001	0.010	0.038	0.0016			
rs648619	7	124.36	GGTGCAAAATGTTTGTCCTTAGAGATAAAGAAAAGAAAGGAAGGGAAGGAAGGAA[A/G]GAAATACTCGGCAGGAGAAAAAATAGCTTCTACTTCTGTTAGCTTTACTACTTTTCTT	A	G	0.71	0.51	0.48	0.52	0.43	0.50	0.53	0.63	0.32	0.60	0.56	0.027	0.000	0.010	0.000	0.0018	1		
rs1833083	7	124.77	GTCTCCAACTGGAGGATAGGCCAAGAGATCCTAGATCTTTTGTCAATCTCCACCCA[A/G]ATTAATTTTGGGGGCAAATCATTCTCAGCCATTCAAGCTCAGGTTTCTCCTTGATAT	A	G	0.83	0.61	0.65	0.66	0.71	0.82	0.88	0.65	0.46	0.65	0.60	0.022	0.053	0.003	0.026	0.0021			
rs996867	7	124.80	CACCAAATCCTACTTCTAACAACATGCACTGCCTACTTTTCCATGTTGAAAATC[A/G]TATTTTCAAGTAAAGCTTCAAAACTAAGCAGCAATAAGTAATCCCAGAAGAAGATTATA	A	G	0.86	0.57	0.59	0.60	0.70	0.77	0.66	0.58	0.46	0.60	0.59	0.046	0.008	0.002	0.028	0.0000			
rs1419607	7	124.95	TATGAATTTTACTAGCT[A/G]CTAACAACTTATACTATAGCAAGGGTAGTATTTGAGAGGTCTCACTTGTCCAAATTCTTGCCAACACTTGATATTGCAGACTATAGTTTAGTACA	A	G	0.62	0.43	0.44	0.52	0.50	0.60	0.59	0.58	0.32	0.47	0.48	0.026	0.023	0.024	0.020	0.0009			
rs719319	7	125.27	AGCTACAAAGGAAATTTAATGGCTTTTGAGTTACAGACTTTCAGGTATAAATC[A/G]TACAATGACACCACAGAATTTATGTAGTAATAATATATCTGACATGCCTGAATAGAGAAA	A	G	0.79	0.62	0.66	0.62	0.66	0.55	0.35	0.57	0.71	0.58	0.56	0.007	0.054	0.001	0.007	0.0081			
rs1419511	7	125.31	GTCAGTGTATGTGTGTGGTACATGCACTTGAATTTGTGTGTTTGGCTAAGGTGTA[C/G]TGGAAATGGCACATGGCAAGTTGGAGCTAATCAAACAATGGTGAGATACAACACCT	C	G	0.52	0.15	0.16	0.17	0.30	0.23	0.06	0.16	0.21	0.17	0.18	0.099	0.000	0.007	0.019	0.0006			
rs766240	7	125.69	TGAGCTGATCTTTACACATTCGTTCTCAGTATAAGAATTTAACCAGATAGTTGCT[A/G]TCTTTACAAACAAAACTTTTGATACCCCTAATGATGAGGACCAGATGAAAAATAATAA	A	G	0.25	0.39	0.42	0.40	0.46	0.50	0.23	0.42	0.25	0.41	0.28	0.027	0.035	0.001	0.000	0.0129		1	1
rs1419438	7	126.18	TTATGGACCTACTGGTCCTACCTAAATGGCCAAAGCCAACTTGACTATTTGAGTG[A/G]ATTCACTGGTCAAATTGGACAGAAATGCAAAATGAACTGTTAATAAGAATATTCAGGT	A	G	0.69	0.64	0.65	0.66	0.73	0.77	0.92	0.63	0.75	0.63	0.66	0.001	0.045	0.003	0.006	0.0000	1		
rs1419437	7	126.21	AAGCTAGTAAAATTCATTCCTACAGAACAGATCCCAACCTAATTAAGTGTAACAC[A/G]AAAAGTGAGTTTCTACTTAAATTCTGAGGCAAAGATTATAAATACACAGAAGCCCAAA	A	G	0.83	0.60	0.58	0.65	0.64	0.68	0.64	0.64	0.61	0.61	0.59	0.040	0.001	0.000	0.005	0.0000			
rs322812	7	127.30	GGATTATTGAGTTGTGTGCAGTGTAGACTAACAGGAGATGGAGAGCAATCCG[A/G]TTGACTGTATCACCGTTCCTGAGACAAGGGCGGGAAGTGATGGGGGCAGGGGGAGAGTA	A	G	0.43	0.52	0.53	0.48	0.45	0.73	0.40	0.56	0.64	0.54	0.49	0.003	0.006	0.006	0.031	0.0013	1		
rs691183	7	128.66	GGTGAACATTGCCAAGGGATGAGCTTGCCAAAAGAGAGACGGAAACAAAGAGA[A/T]CAGTGGAGCATTTCTTCAGATAGGCTCATAAGTGGGACTCTGGAGGTTTCCCTGG	A	T	0.50	0.66	0.63	0.71	0.68	0.91	0.77	0.71	0.61	0.60	0.70	0.007	0.014	0.002	0.060	0.0025	1		
rs1464890	7	129.22	TTTTCATTCCTATCTAGTTCCTCTTTGGAATCCATGCAGCTCTCCCTGATA[A/G]CATGTTCGCAATGTATGAGGAAGGTGGGGCTCTGGGGCTTCCAGCAGATTGAATCGTCCA	A	G	0.93	0.43	0.47	0.41	0.43	0.32	0.52	0.56	0.32	0.48	0.44	0.118	0.001	0.002	0.028	0.0012	1		
rs2030974	7	129.46	AAATTTCTTTCTCTGCTTTCGAGTTTTTTTCTTAAACAAAGATTAGACCATATC[A/G]CGTATACTGTTCTTTAAACAAAGGTGATCTTTTCTTGTTCATAAACATAGATCTACATTAA	A	G	0.57	0.73	0.71	0.65	0.71	0.91	0.98	0.75	0.86	0.64	0.70	0.006	0.075	0.003	0.033	0.0000			
rs729332	7	129.47	CCATGACCCCACATATATTCAAGATGAATGACAGCTTCAAGTTTATTTTTAGA[A/G]AGAGCTAACTACGGTAGCGTTTTCAAAGACATTTTCAAAGAAACTGATACTAATACTAGT	A	G	0.50	0.44	0.37	0.32	0.39	0.09	0.28	0.29	0.50	0.36	0.35	0.019	0.003	0.000	0.044	0.0002			
rs1809627	7	129.52	CAATTCAGCCTAGCTTTACCATTAACCTCGGCTTTGGGCTTATAAGCCCTTCATGG[A/G]CAGAGCCAAGGTTTTATTCATTTAGAATCCTCAGCACCTAAGGTGCATCATAAAGA	A	G	0.21	0.27	0.30	0.38	0.36	0.23	0.08	0.45	0.36	0.35	0.34	0.000	0.010	0.051	0.001	0.0018			
rs1990790	7	129.59	GAATGAGAATTACAAACTTTTAATTATATTGTAGGGGGAAAAAAGTAGGCTGTTTTCCTGATAGGTCTAGCCATTCATTCA[A/G]TAAACCATATTGATATGATTGTGCCTGATCT	A	G	0.79	0.47	0.50	0.46	0.48	0.59	0.32	0.50	0.46	0.48	0.47	0.051	0.014	0.001	0.007	0.0004			
rs15956	7	130.60	CCCTGACCCCACATTGCCACAGAAGAGGGGGTAAGAGTAGAACCAAACAAGTTAGC[A/G]TTTTCTCCACACATTGAGTCTCATCTGAAATACTACTGAGGTGCAAAATGTGTC	A	G	0.43	0.49	0.58	0.63	0.45	0.50	0.60	0.61	0.39	0.67	0.58	0.002	0.010	0.008	0.000	0.0001	1		
rs541186	7	131.81	CTAGGTGACCTTGGTTAGCCTCTCTGGCCACTGTTGACTCCTCTGTAAGAAT[C/G]ATGGCCACTCGATCTACTTGGAAGGCTTATTGTAAGAAACAAATGAGATTATTCTGTGTC	C	G	0.64	0.52	0.50	0.39	0.46	0.55	0.26	0.57	0.46	0.52	0.49	0.017	0.017	0.005	0.005	0.0000	1		
rs1468974	7	132.00	GTTACTCAGGAATGCACAATCCAGGAGCAATCAGCAAGTATTCTTATTAGAAGCTC[A/G]AAAAGTAGGAGGCTACTAGAAGGGCTTCAGTCATCCATGTCTTAATCAACAGTGA	A	G	0.19	0.23	0.25	0.29	0.36	0.23	0.20	0.24	0.14	0.28	0.31	0.008	0.008	0.010	0.006	0.0005			
rs889826	7	133.01	TACCAAAAGGTACACCTATTTTCCTCTTCCTCTCTTGG[A/G]AACAGATACAAATAGTTACACAGTTGGCTCTGTCTGTGGCTCATTGAGCTGTGAATTAATGAGTACATTATGGTTCT	A	G	0.33	0.52	0.66	0.61	0.71	0.45	0.83	0.34	0.68	0.63	0.46	0.042	0.019	0.033	0.017	0.0150	1	1	1
rs1646656	7	133.45	CAGTAAAAGGACATCAGGTTCTTAGATTTAATATATACCCCGAGGTATGCTTC[A/G]GATATCTTTCTGATGGATTTACAAGAAGTCCACCTCACCCCATCAATAAAGTCAGGAC	A	G	0.45	0.54	0.56	0.49	0.61	0.18	0.08	0.33	0.68	0.52	0.51	0.004	0.133	0.024	0.076	0.0037			
rs1962522	7	134.20	GATGCCTTTCATCCTCACCACCACCCGCCAAGAAAATAAAAAGACATTCTGGT[A/C]ACTTCTGCTTCCTAGACATGGGGTTTTCAGTTGAAAACCATCTTTTACTTCATCACTCTT	A	C	0.43	0.48	0.56	0.46	0.68	0.18	0.45	0.71	0.43	0.53	0.49	0.012	0.009	0.005	0.090	0.0038	1		
rs2059367	7	134.46	CAAATTTAGAAAGCAAAACCAAAAGGAAA[C/G]ACAGAAGAGATTACAATTTTAAACTCCTTCCAAATAAGACCTTTTAAACCAATCCTCCCCCAACAACAAAGAACTTATTGGCA	C	G	0.74	0.73	0.72	0.70	0.71	0.50	0.58	0.75	0.61	0.75	0.71	0.000	0.004	0.011	0.018	0.0000			
rs1559534	7	134.46	AAAAAAAAAAAAAAAGAAGTATTTTCAAAGGCAGCTCTTCCTGCTGGAAAACTGCCT[A/G]TGACTGTCTGAACTAAATTAACAATTACTGAACAAGCACAAAAAATAGAGTACTG	A	G	0.31	0.14	0.12	0.07	0.13	0.14	0.01	0.06	0.18	0.15	0.16	0.027	0.041	0.003	0.001	0.0029			
rs1863005	7	134.47	ACTACATAGTTCTCCCCCTCCTCCCACACCAAACCTGAAAATCTCACAACAGGTTG[A/G]AACTATCTTTAGTATTTCTGCATAACACTGTACTAGTCCAACTGTTTACTAGGAAG	A	G	0.55	0.48	0.51	0.56	0.54	0.73	0.43	0.35	0.57	0.47	0.46	0.000	0.015	0.044	0.013	0.0037			
rs959520	7	135.69	TAACCACAAACCCCCTTAATTTCCAACGGTGACTACCGACTACCTATTTCTAAATAATGCCATTGGATATAAAGGGTAGGGC[A/G]AATAAAGGTAAATGTTTCAGAATGACTCGT	A	G	0.40	0.45	0.47	0.46	0.45	0.88	0.63	0.29	0.57	0.43	0.38	0.000	0.020	0.010	0.112	0.0034	1		
rs1424387	7	136.01	TATACTCACTCTAAATTACAAATACAGAAGTGTAGTGAGTTAAAGTCCCTCTCACAACATGTGTGAAACTGTCAGAATTCAAA[C/G]CTAAGCTCCTTGATTCTAAACCCGTGCTCT	C	G	0.93	0.71	0.68	0.66	0.71	0.95	1.00	0.89	0.68	0.71	0.64	0.059	0.146	0.043	0.086	0.0028			
rs748260	7	136.90	GAGTTGGAAATGGGATCGGCTTTCCTGAATCTCATTAGCT[A/G]CTAGAAAGAGAAAAAAAAGATCAAGGTTTCAAAAGGAAGGAGCAAGGGGAAATGGATTTCATAAAACAACT	A	G	0.33	0.56	0.49	0.44	0.66	0.73	0.69	0.53	0.71	0.45	0.46	0.011	0.032	0.001	0.040	0.0001	1		
rs273954	7	137.02	AAAAAAAAAGTGAATACAAAAAACAAAATGAAGATGGGTGATAGATAGAATCAGTT[A/C]TTCCAATCTAGTGAGGCCATTCTCAAAGTGTGCACATTCGTAAAACAAGTCAAGAC	A	C	0.36	0.33	0.43	0.28	0.36	0.00	0.10	0.28	0.43	0.36	0.36	0.001	0.087	0.011	0.176	0.0025			
rs1424376	7	137.10	AAAATACTTGCCAAGGAAACAACACAATAGCTAATACCTGAATGT[A/G]CCATTGTAAGAAACTTCTAGAGCTAACTGGCAAAGACCTGCTTTTATATTTAAGATAGGTAAACTAT	A	G	0.45	0.70	0.61	0.60	0.73	0.32	0.36	0.54	0.61	0.67	0.59	0.022	0.054	0.003	0.065	0.0003			
rs1371463	7	137.70	AAGTCTCCAACTATTTGTGGATGTCTATTTTTCCTTTTATTTCTAACAGTTTTGCT[A/G]AATGTATTTTGAAGCTCTTTTATTTGATGTATAACCTTCAGTATAGCTACGTCTTCTTG	A	G	0.05	0.25	0.26	0.32	0.30	0.64	0.57	0.38	0.36	0.22	0.21	0.042	0.078	0.027	0.086	0.0034	1		
rs1015343	7	137.77	CTAGTCAGCGGGCAAGGTGAACCCGCTGGGCTGTTACATTCTTCCCAGTCAC[A/G]CCCTCAAGCTCTAGCTCAATAAACCTCAGATGACTGAGATTTTTGCCTCAATATCTCAT	A	G	0.38	0.52	0.54	0.51	0.68	0.41	0.58	0.53	0.71	0.52	0.43	0.008	0.000	0.000	0.008	0.0055			1
rs13068	7	138.52	GAGTGTGAGATAAGAGGCCTACAAATCACTCTGTCCTTTACAAACTCCCT[C/G]CTCATTCTCTGCATTTGCCTAGGATATTCACTCTATATGGAATATCTTTT	C	G	0.24	0.77	0.79	0.77	0.77	0.68	0.88	0.74	0.89	0.64	0.69	0.161	0.007	0.005	0.009	0.0040	1		1
rs1464798	7	138.75	ACTGATCTCTGTACACCTCACAGCACCCAGCGTACAGCCTTGTCTACTGGGGT[A/G]AGAGGGTCGGTGTCTGCAGACATTGGGTGTCATTGTTCACACTCATGAGCTGGAAGA	A	G	0.52	0.43	0.48	0.43	0.46	0.05	0.03	0.42	0.54	0.48	0.48	0.001	0.158	0.001	0.135	0.0001			
rs880290	7	139.33	GGGTTTTCTAAGTCTATGTTCTGGACTCTATTTCCAGAACTCCAGCCACTTCCT[A/G]AGCCTTCAAGCTGCTGTTTTCCCCTAACCCCAGAAAACCCCTGAGGTTGTGTAATTA	A	G	0.19	0.26	0.25	0.26	0.21	0.00	0.00	0.29	0.21	0.36	0.26	0.003	0.101	0.000	0.101	0.0003	1		

rs768055	7	140.82	CATTCTTTAATGAATCACGCAATCTGGCTTCTGCTTCTACACTAAAATATCATGGG[A/C]AGAGATGATCAGAACTGTAGAAGTCTTTTAGAAAGAGCCTCACAGTGCAATGCTTA	A	C	0.45	0.45	0.44	0.54	0.46	0.09	0.27	0.41	0.54	0.36	0.42	0.000	0.008	0.001	0.078	0.0001	1		
rs1476640	7	140.82	TAGAATTATGAGTAACCCTTTCAAAATCTCAAATAACATAGTTTGAGATAAGAC[A/G]TATGAAAAGAACTTCTGCTTGGAAGCAGAGGAATTTGAAAATTTATTGAAAGATGACAC	A	G	0.40	0.43	0.39	0.38	0.39	0.64	0.47	0.39	0.43	0.49	0.41	0.001	0.004	0.000	0.035	0.0003			
rs1859646	7	140.90	GGAAATTTAAAAGCAAAGCAATATGGCACACAGAAATAAAAGGACACTCATAGGT[A/G]TGATTGGAGCCCCAGAAGGAGATGGAAAAAATAAGCACAAGGAATGAAATATTTG	A	G	0.48	0.46	0.48	0.41	0.48	0.86	0.49	0.59	0.39	0.50	0.45	0.001	0.001	0.016	0.105	0.0002			
rs17229	7	141.73	AATTGGCTGACATCTCATTGTTACTTCTTGTCCCATCTCTGTCACCTTGT[C/G]CCTTGGTGTCTGGGTGACTCTAGCATCTATAAGCTCTGTGAAAAGAGATA	C	G	0.26	0.57	0.51	0.49	0.55	0.64	0.06	0.40	0.64	0.50	0.45	0.024	0.148	0.011	0.008	0.0034	1		
rs940864	7	142.51	ATTAGTTCAGATCTGATGGGGAGAGTGGGAGGTTCTGAGGCTGAGACTGAGCTTAG[A/G]GTTAAAGTTTGGAAACACTGTTTTAAATTATTATTATATTCTCATGCACCTAGTAG	A	G	0.40	0.58	0.55	0.68	0.57	0.86	0.91	0.60	0.64	0.58	0.59	0.019	0.075	0.002	0.050	0.0005	1		
rs727714	7	143.49	ATCTCCTAATTTGGGGGTACTCACCCCTTGTGAGTTCCCTTTTCCCAGACACCA[A/G]GGGTATGAGTTTGAGGGACTGTTCAGGTATCTCTAAGGGATCATGTTGGGGCTGCG	A	G	0.76	0.54	0.57	0.59	0.52	0.91	0.73	0.80	0.39	0.59	0.55	0.049	0.050	0.037	0.114	0.0005	1		
rs969356	7	143.57	GAACTCTGAGGACGGGCTCACCATTGGAAAATAGAAATGGAAGGGTTTCCTTA[A/G]TTCAAGTGAATATGTTAAAAGATGGGAAAAACAGGAAAGCTAAAGATCTGGAAGGTC	A	G	0.55	0.57	0.64	0.56	0.63	0.23	0.07	0.32	0.57	0.65	0.66	0.016	0.229	0.075	0.117	0.0000			
rs2056553	7	144.65	CACACAAGCTGCTGAATATATATTTTTTTCAAATAACTACAGCAAAACTATAGAAAC[A/G]GGGGTTATACCAAATTAGGAAAATTACCTGAACCACAGGCCCTTCTAAAAGTTCAC	A	G	0.55	0.47	0.45	0.44	0.70	0.36	0.58	0.36	0.46	0.54	0.50	0.011	0.015	0.000	0.000	0.0006	1		
rs850545	7	144.79	TGAGTCACTAACTTGCGTGTTTGGGCTAAGTAATAGGGAATGGAAGAATTGACCT[A/G]ATTAAAGTTGTATTGCCATTGGAATGTTGGTGTGAGATTAGTTTCGGTCATTTCACT	A	G	0.48	0.38	0.53	0.46	0.39	0.59	0.90	0.60	0.39	0.38	0.43	0.007	0.047	0.001	0.000	0.0099		1	1
rs1860482	7	144.81	TGCCTTTTTGGTAAGTCTGGTATAGAGGAAGGTTTCTTGCTTTCCCTGCA[A/G]GAGTAGGAAATCTCTAAGGTTACTACAGAAGATCCTCAGCCCAGGATCCTTTCTTGCC	A	G	0.50	0.23	0.32	0.33	0.23	0.23	0.20	0.35	0.14	0.28	0.24	0.006	0.015	0.003	0.015	0.0059			
rs802200	7	145.50	GCAAAAAATGTACACAACAAGCAGCACTTATACATTCTTTAGCACA[A/G]AAGGGACTGCCTCAGAGACCTCTTTAAATTCTTAAGGCAAATTTAAAATCTTGGCAATTTCCTCGA	A	G	0.81	0.40	0.44	0.33	0.41	0.18	0.50	0.22	0.43	0.43	0.51	0.064	0.003	0.014	0.036	0.0022	1		
rs700273	7	145.57	AACCTTTTTCCTGATGAATTTAAGAGATGTGTTTATTGAACAGAAGCACAATT[A/C]ATAGATAACTCATCTTTATAGGGCGTCTGTTGCCATGGTAATAGTGATGTGACATCGTT	A	C	0.83	0.41	0.43	0.34	0.45	0.15	0.59	0.23	0.39	0.42	0.51	0.079	0.009	0.016	0.051	0.0042			1
rs1524341	7	146.10	CCAGTGGACTTTCAAGCTGTTATTTTGAATTTGCAATTCAATTGAATGAAAG[A/G]AAATACATATAAATATGGATCATTTCAGATAAAAATAAGGCTTTTGTTTTAGTAGGGAA	A	G	0.83	0.55	0.52	0.60	0.66	0.68	0.36	0.78	0.64	0.47	0.54	0.051	0.013	0.036	0.009	0.0002	1		
rs1024676	7	146.11	GCAGGTTCATACACATATCTGTCTTTGGCTTATCAACATTTAGAACTATAGCG[A/G]CTCTTTATTAATCTCCTTAGTGCCTGGTTATCCAAATACTTTACTATTTGTTTAACTGC	A	G	0.43	0.34	0.35	0.37	0.45	0.50	0.14	0.46	0.50	0.29	0.35	0.005	0.035	0.007	0.010	0.0000			
rs903898	7	146.24	CATATATTATGCTTAATTGTGTACAATATTGTCTCTATGAATATTCTTTACCAATGGCTCGTCTTCCATTTATAT[A/T]GGGCATTAAATCTTTAATTGTAAATAGTGCACTCTGTTAA	A	T	0.57	0.55	0.61	0.56	0.45	0.55	0.27	0.73	0.68	0.67	0.69	0.003	0.083	0.006	0.006	0.0032			
rs1496542	7	146.30	GACTTAACTTAGATGGTTTTATTTATGACTATTTATTTTGCTTCGGATTTTCTC[A/G]GAAGACCACAAACCCCTCTAGATATTTTAAATAGAATGAGATACAATACAGAGAATCAGA	A	G	0.60	0.46	0.40	0.45	0.57	0.45	0.67	0.25	0.32	0.36	0.30	0.032	0.059	0.009	0.007	0.0051			1
rs1861010	7	146.99	CGGGGCAGAACAAAAGCCAGCAAAGGGAT[C/G]GAAGAAACCCAAAATAGATCTTTCTTGAACTGATTTGTATCAGAGTTATAAACAATTCCAGGTATGAAAGAAAAGTAGACAG	C	G	0.31	0.43	0.35	0.37	0.43	0.50	0.53	0.38	0.43	0.28	0.34	0.000	0.039	0.007	0.024	0.0001	1		
rs963314	7	147.25	TTCCAACTATGTGCTTCTATACAATGTGCCTATACTCAATAATAGCAATAATC[A/G]TAACAGCTACCAGTAACTAGAATTAGTGCAAAGAAGTGCATTATCTGTTATTAACACACT	A	G	0.83	0.43	0.44	0.44	0.52	0.91	0.54	0.58	0.57	0.48	0.49	0.079	0.002	0.007	0.125	0.0008			
rs740136	7	148.81	CTCGGTCAGCGCTTGATAATTCTCAGGCTGGCTCTTAGAGGAGGAGCTGGAC[A/G]TTCTGAAAGGAAAGTGGGGAGGGAGCGAATAAATAGAGGATGGAGCTCTAG	A	G	0.14	0.32	0.39	0.37	0.46	0.77	0.86	0.47	0.39	0.28	0.40	0.043	0.144	0.002	0.085	0.0000	1		
rs757723	7	148.84	GGCTGGTGCAGAATCTGTAAGGGCTTCTGAAGCCATGGCTGCAGCACTCACACA[A/G]TGTCACTTCAGCCATATTCTTGATCAAATCAAGTTACAAAGCCCCCCAAGGTT	A	G	0.57	0.21	0.22	0.24	0.32	0.68	0.83	0.43	0.11	0.25	0.30	0.071	0.230	0.021	0.123	0.0047			1
rs1104881	7	149.53	AGGCATCTGCCCCTAACCCAATAGAATGGAGACTTCAGCCAGAACTTGGGACAG[A/C]GGAAACTGTCACCCATAGTCCCCATTCATGCAGGGTAATTAATTGGCTGGCTGCA	A	C	0.48	0.26	0.26	0.24	0.38	0.27	0.25	0.25	0.25	0.32	0.37	0.025	0.000	0.000	0.001	0.0063	1		1
rs1547958	7	150.03	TGGGGAGCAGTGAAGGTACACACGAAACTCAATGCTCTTCGCATGCTGGGCTGT[A/G]AGCTGAGCCAACTCCCCGCTCCCTGGTGCTGTTCATCTGGTCTGGC	A	G	0.10	0.18	0.24	0.23	0.13	0.05	0.00	0.21	0.14	0.26	0.26	0.022	0.101	0.003	0.049	0.0001	1		
rs875588	7	150.56	CAGGGTTGCTGAGTGTCAGATGTATGCACATGTGAATGGATTGCTAGGAAAAAACA[A/G]GAGTTCTTTGTGTTCATTTATGTATGTTAAAATGGTGAGTTTGTGACATTTACT	A	G	0.64	0.40	0.48	0.51	0.57	0.41	0.33	0.55	0.64	0.48	0.53	0.012	0.005	0.005	0.001	0.0021	1		
rs2014611	7	151.57	ACTCTGAAATCCACAT[C/G]TCTATTTTGTTTTTTGTTTTTGAGACGAGTCTGTCACCCAGGCTGGAGAGCAGTGACACGATCTCAGCTCACTGCAACCTCCGCCTCC	C	G	0.57	0.32	0.31	0.44	0.34	0.27	0.54	0.46	0.21	0.36	0.28	0.051	0.041	0.026	0.000	0.0001	1		
rs880310	7	151.59	CTC[A/G]GGGGGCACACTAGTCCCTCCTGGAGGTTTCACGACCTGGCACCAGATGCAGACCCTGGTCAGCTCTGTTAAGGAAGGACTCTGTGAGCAGGTGCTGGTGTCAGC	A	G	0.71	0.52	0.50	0.54	0.34	0.05	0.01	0.34	0.43	0.44	0.51	0.022	0.182	0.006	0.141	0.0000			
rs1735093	7	152.39	GCCAGATCACAGAACTGTGAAGTGATCCTGGGCAGAAGTGGCTGA[A/C]GTTGTGTTCATACCATTTCAGAAAGATGGGAGCTCATCATTGAAATTAAC	A	C	0.43	0.44	0.43	0.46	0.48	0.77	0.76	0.48	0.57	0.46	0.42	0.000	0.060	0.001	0.069	0.0001	1		
rs38993	7	152.97	GTGGTGATGTGATGAGATTTGTACTTCAGATATCTCTAGATGAACTCTGGAGA[A/G]GGAAAAAGTAGATGTGAGTGGATGAAAAGAATGAACAGCAGTTGTCCAGCAG	A	G	0.69	0.56	0.60	0.54	0.68	0.45	0.43	0.58	0.50	0.65	0.54	0.001	0.023	0.002	0.020	0.0033	1		
rs286833	7	153.49	TGCATCTGCATCTTTTCTCAGGGTCAAAAAATCATAAATCAATAATTTGGGGTTGTA[C/G]TGAACAAAGTTAAGCCACGGGATTCTGGGTGCTATTTTCCAAAGTTGTCATTATTT	C	G	0.10	0.05	0.04	0.05	0.07	0.00	0.01	0.00	0.04	0.05	0.04	0.007	0.004	0.014	0.000	0.0000	1		
rs7800754	7	153.77	TCACGGTGGGACACGGAGCCCAGCCCCTGATGGCACCATCCATGTGTTGCTCTC[A/G]ATCCCTGTCTCTAGATTAGCTAAGCCAGAAAAATTAGCCTGATTTGTCTAAGCGT	A	G	0.17	0.07	0.08	0.02	0.13	0.14	0.01	0.07	0.18	0.05	0.08	0.005	0.020	0.000	0.002	0.0000			
rs306278	7	154.18	GAAGAGACAGAGATACTGTCCCTAAAGCAGTACAAGGAAGCAAGCCAGAGAC[A/G]AAACATGTTCCAAAGAAGAGGGTGCCCCAGCTCAGGACCTCATCCAAGGCAGTTTC	A	G	0.86	0.39	0.45	0.40	0.63	0.59	0.41	0.42	0.36	0.36	0.34	0.096	0.002	0.001	0.012	0.0106	1	1	1
rs1657290	7	154.35	CCAAAAGACAGGGCTTGTAAGCCTAGCCTTCACCCCATAGGAAAGATGTCCATGC[A/G]TGTGGAAAGCAGCCTACCTCAAACATGCAGAGCACAGGGCCGCGTGCAGGAT	A	G	0.57	0.42	0.48	0.39	0.54	0.86	0.57	0.48	0.50	0.58	0.48	0.001	0.001	0.003	0.071	0.0001			
rs749539	7	154.64	TAAGTTTAAGGCTTCAGGTTATGTAAAACAAATTAGGTCTCTGCTTCATCAATTTAA[C/G]TCCCCAAACCTTGCCACTAAGTTTACGTAGGTTGTATATTGTTCAGTCAAATAAC	C	G	0.31	0.07	0.06	0.12	0.16	0.68	0.08	0.20	0.11	0.07	0.12	0.056	0.000	0.035	0.230	0.0039			
rs6953751	7	154.79	ACTTGGTGGAAACGTGGAGTCCTGGGGTCGTTGTGACTTGCTCCACGTCAGTCC[A/G]TTATTGATGTTTCAGAAACACTTTCTACTGGGTGTCCATTGTGTCCCAGACTGGAAC	A	G	0.45	0.34	0.34	0.27	0.29	0.41	0.47	0.39	0.46	0.26	0.19	0.005	0.016	0.005	0.003	0.0192		1	1
rs1920454	7	154.92	TCCAGGAACTCTGGTAAGCAAGGCAGGAGATGTCACCACTTACCCTGCTCCAG[A/G]AACTGAGGCCTGAGAACAGGACAGGCCCACAAAGAGCCTCCAGGAGAGGCTGGG	A	G	0.33	0.28	0.28	0.33	0.18	0.32	0.86	0.20	0.57	0.35	0.19	0.002	0.233	0.000	0.006	0.0026			
rs1343750	7	154.94	CTGGGTTTACAGGTGTGAGTCACTGCATCTGGCCAAGAGAAATTATTTTAACTTGT[A/G]TTCTAACTATATAATATCATTAGAGAAGGACCATGCTGACTGGCTTCTTCACCCTG	A	G	0.75	0.80	0.81	0.80	0.88	0.68	0.71	0.85	0.71	0.68	0.84	0.006	0.014	0.000	0.020	0.0000			
rs1531381	7	155.11	GTGGCTTTTAACCTTTGGGTTGTGATCTGTGGGTTAAAACATCCTGAGATTCAGAT[A/G]ACTCGTTTATGGGTCACCGTGCCCTGTTCTTCTTACCAACCCTTGACAGTTTC	A	G	0.14	0.20	0.21	0.17	0.21	0.23	0.49	0.25	0.14	0.19	0.15	0.001	0.067	0.004	0.003	0.0027			
rs7784332	7	155.23	TGTCCTCAGCTCTGTCCCTGTCCTCCCTCCTGCTCAACCAAATATCTCCCAGCCAA[C/G]TGACACATGGAAAGGAGCTAATAAATTCATGCCAGGATGTATGTGACAAATGAT	C	G	0.31	0.53	0.48	0.49	0.57	0.31	0.33	0.52	0.54	0.47	0.48	0.009	0.015	0.001	0.010	0.0000			
rs1389240	7	155.47	AGAATAGACACAGAATTGAGACATCACAGAGTTGGAAGAAGGAGCCCGGGGTCTCATTAC[A/G]TCAGTGATTAATCACAAAGTGGGTCCTGCTTCTGAACTCA	A	G	0.50	0.59	0.56	0.63	0.55	0.23	0.66	0.52	0.64	0.60	0.63	0.000	0.012	0.001	0.044	0.0037	1		
rs1182378	7	156.47	ACCAAAATGAATATAATATTTTAAACCTATTTACTGGCATAGCTGAGATTATT[A/G]AAGGTGCAAGCCCTCAGTGCAGATGGGTAATGAAAACAACCGAAGACAATCTCTGGC	A	G	0.79	0.35	0.38	0.43	0.41	0.41	0.46	0.34	0.29	0.37	0.31	0.076	0.002	0.004	0.000	0.0036	1		
rs1182414	7	156.48	CCTCACCATTAATAGGCAAGATGAGCGAGACCCATTATCAGATGAGAAAAGCAC[A/G]GCACGGAATACTGTGTCCAATGTGCTCACCGTCTCAATTTTAAACCTAAAGGAGGG	A	G	0.52	0.66	0.62	0.57	0.59	0.68	0.64	0.71	0.71	0.64	0.70	0.002	0.002	0.012	0.005	0.0041			1
rs1894811	7	156.72	CTTTTGGGTTTGGGGAACAA[A/C]AAAATTGCTTTTCATAAGTACTCAATTTAGAATTGTTTGCTAAAATTAAGCGCTTGTTCACCAGACATTTTAGACTGAGTAATTTTTAACCC	A	C	0.79	0.51	0.53	0.55	0.61	0.86	0.86	0.73	0.65	0.60	0.60	0.032	0.066	0.010	0.058	0.0024			
rs10772	7	157.84	TGCCTAGTGCCTGCGTAAATAATATATTTAATATAAAGGACTCCAGGTAT[A/G]AATGGTGTAGAATCCATGATTCCAAGAAAAAACACTTTTCTAGCAAACC	A	G	0.52	0.62	0.57	0.63	0.45	0.50	0.49	0.49	0.75	0.55	0.55	0.005	0.006	0.007	0.004	0.0000	1		
rs372746	7	158.37	GACACCAAGCACAGCTTCCCGAGGGTCCCCAAGCCACCACCCACAGCAACGCCCC[A/G]GAGCACAGCACTGTGATCTCTGGCTGGTGACTCAGAGCACTTCATTTTTCCATT	A	G	0.12	0.26	0.34	0.38	0.16	0.14	0.01	0.36	0.18	0.27	0.32	0.038	0.134	0.000	0.029	0.0021	1		
rs455030	7	158.44	TTGCCTTCTCTGTGTATTGCAATAAAGACCATTCTCATTTCTCTAAATTTGCTCTT[A/G]ATAACCATTAGTGAAGGCTATATGGCACTTGACATCATACAGACACTCTAGGTTG	A	G	0.31	0.68	0.67	0.50	0.70	0.82	0.75	0.78	0.64	0.69	0.68	0.043	0.009	0.029	0.029	0.0009			
rs2003213	8	0.38	AAACTAAAAATACAGTACCCCTGGAAGTATGTGACACTGGATGTGCTATCTAATTGGT[C/G]CACCACAGGCAGAGAATGAAGTACTTCAGATAATTTCATCAAATAACCAAAATTA	C	G	0.62	0.79	0.78	0.88	0.89	1.00	0.85	0.91	0.89	0.80	0.81	0.012	0.006	0.009	0.090	0.0015	1		
rs13429	8	0.43	TCACAATTCTATGATTGACGTGAGTTGCAGGCACTT[A/G]GCGTTCCAACAGAGGCACGGCAACTGCAGCCATCATCACAGCCACAGAGCTCCCCACAACATGGGAAGCGATCCCCA	A	G	0.55	0.49	0.55	0.46	0.48	0.73	0.75	0.60	0.50	0.50	0.49	0.000	0.019	0.000	0.017	0.0026			
rs935559	8	0.47	GGGAGGGGGATAGAAAATGAAGCAGTTGAACTGACACAGGAGGGCTCTACAGGG[A/G]AAGAGAACTGGGAAAATCCCATTAAGGAGTGGAGGCCTCATTTCTTAGCTGAATGTTG	A	G	0.07	0.12	0.12	0.10	0.14	0.27	0.16	0.10	0.11	0.10	0.10	0.001	0.015	0.001	0.036	0.0009			
rs2166842	8	0.51	CAGACAGGCTGACTGAACAAATTTAAACGGATCCATTCACAAAGCAACTCACCTCA[A/C]CATGTCCCAAGGTCTTTTCGAAAGATCCTTGCCCATTAAAAAGAGATTCTCTTAG	A	C	0.67	0.90	0.84	0.84	0.89	0.86	0.61	0.91	0.82	0.84	0.82	0.037	0.061	0.000	0.001	0.0007			
rs1669694	8	0.56	AGTTCCTCTTGACCTGGAATTGTCCAAGTCAGCACCACAGCTGGTTAAGGCAG[C/G]CCCTCTCTCTTTTCCTCTTCTTATTTTCTTGGCTTTAAGTCTGCTTAGGACTCCTGGAA	C	G	0.48	0.50	0.48	0.55	0.50	0.45	0.28	0.40	0.61	0.47	0.37	0.000	0.018	0.002	0.001	0.0106		1	1
rs763869	8	1.36	TACAAACATTTTAAAGAATAAATATGATGTGGCTACTCCCTCATAATGTAATGCA[A/G]TTGAGTTAGAAATAATATAAACAAACATCCTCATATACTTGAAAATAATCAAATGTCAA	A	G	0.45	0.56	0.53	0.39	0.57	0.45	0.82	0.74	0.46	0.57	0.48	0.013	0.042	0.004	0.013	0.0035	1		
rs4242539	8	1.68	AAATGTCAAGAAAATAACACTACAGAGCACATATGGAGGAAAGGCAGACAGTGC[A/G]AAGATTGCAATAACTGTGTTCCTCCAAGTTATAGATGTGGAAATAAGACAAGTGA	A	G	0.38	0.24	0.23	0.20	0.23	0.23	0.70	0.18	0.38	0.22	0.20	0.010	0.106	0.006	0.000	0.0007			
rs7833068	8	1.74	CAAGCGCATAATGCCAGTATCTCACCTGAGATCATGTTAGCATATCATGAAAGTTC[A/C]TGACCTATTGCACATGTGCAGAATCACCCATGTAGCACACACACAGATTGGAAGA	A	C	0.43	0.64	0.54	0.61	0.66	0.68	0.39	0.56	0.46	0.61	0.64	0.002	0.010	0.003	0.015	0.0042			1
rs2272711	8	1.82	GGTTAGGTCATGAGAAAGCTACAGCATAAAGAGACACAGTCTTTTCACAAAAA[C/G]GCTCTTTTTGGTTCTTTGGGACAATTTACATTGAGAGGATGCTGGCAGCGTCTGCA	C	G	0.10	0.09	0.19	0.05	0.09	0.45	0.22	0.42	0.18	0.17	0.11	0.006	0.002	0.036	0.052	0.0037			
rs1478959	8	2.14	GTACATTTCTTGACTTA[A/C]TACAATTCAAAAAAAATGAAACAAATTACATGAACAGTGGCTATCATTTCCCCCTTGTGAATGGCATAGTAGCAGTCTATACAATTTTGGATGT	A	C	0.31	0.18	0.21	0.26	0.21	0.09	0.16	0.07	0.18	0.19	0.19	0.002	0.006	0.020	0.023	0.0006	1		
rs897445	8	2.28	CACAAAGTTCTGAACTTTATTAAGAACATGCCCCAGAGTTCTCAGATGGTTTTATATAAATGAACGCATCAGACATGGTGATGTAGCCCCTTTTAC[A/G]TCATCCGCGCTGCCAAG	A	G	0.35	0.36	0.41	0.45	0.39	0.36	0.51	0.51	0.36	0.45	0.45	0.000	0.003	0.005	0.000	0.0010			
rs1016667	8	2.57	CAAGGTCCTAGACTGCAGCGGATGACATTCTCCAGTTGTGTGACATTTCTCCCC[A/G]TCAGAATATAGAGAGCTCGGAGTAATGCAGGTGAGACCCTGGACCCTAACTTTCT	A	G	0.57	0.90	0.86	0.90	0.86	0.91	1.00	0.90	0.86	0.85	0.92	0.063	0.027	0.001	0.000	0.0049			
rs2978328	8	2.63	TGTAAACATGTCTGAACCCAAAGATGAGAACAGGTTAGACTCAAAATGGGTTCGC[A/G]GCTGCAGAGTTAAGGACTGAATTACTTATCCTTCTGAAGTGGTGATTTTCCTCCCT	A	G	0.38	0.86	0.87	0.88	0.75	0.68	0.21	0.74	0.89	0.77	0.74	0.158	0.273	0.034	0.050	0.0106		1	1
rs2978315	8	2.69	TCCAGGACTGTGGAGGAGAGGTGGTATCAGTGTGACTCTTTTGTATTCTGTTC[A/G]TCTTGTACAATCTGGCAGCGCATGTGATTCTGACTCCATCTTCAGGATTTTTGGGTA	A	G	0.12	0.36	0.32	0.18	0.30	0.64	0.10	0.42	0.29	0.21	0.23	0.016	0.018	0.005	0.067	0.0063			
rs3112	8	2.94	GTTAACTCAAGAATATATTCAGTCTTTATGTTACTAAAACATTGTAATAGTGTTTCATCAATGATGCCGAGGTCACTGCT[A/C]TACAAAGATTAAAGAAACTTACCATCAAACAC	A	C	0.81	0.63	0.65	0.54	0.57	0.68	0.49	0.67	0.75	0.57	0.57	0.016	0.021	0.000	0.000	0.0045	1		
rs922798	8	3.47	TATTTTTATTATTAATTAAATTTTATGAAAGGAAGGCAACCTTTTAAGAAATAAA[A/C]ATAAGTTTTATTAACCAAAAGTGTCTCTTTAGTTACAAAGCCCTTCAACGCATAATGTC	A	C	0.79	0.59	0.53	0.56	0.61	0.59	0.69	0.64	0.57	0.55	0.46	0.032	0.019	0.002	0.001	0.0016	1		
rs972422	8	4.01	AGATTATGAAAAATGAACAAATTTGTTTCTTTGCACAATTCAAAATATCTGATT[C/G]CTTGAAACTGGTTCCAGAATCAGTAAAAATGATGGCAGCAATGGTTTTCCTTAGAT	C	G	0.71	0.46	0.43	0.52	0.54	0.82	0.53	0.53	0.46	0.52	0.53	0.045	0.012	0.020	0.118	0.0032	1		
rs899145	8	4.57	AGTTATTATCACCAAAAGCTTTCTCTTCTAATGACTAAAAATAGCACTCTAC[A/C]TTTTAGTGTAGTTCACAGAATTTGTTTTTAATACTCCAGCTCTTGTGCTCCTAAGTCT	A	C	0.24	0.66	0.63	0.63	0.57	0.77	0.74	0.59	0.61	0.60	0.54	0.059	0.025	0.000	0.031	0.0033	1		
rs763630	8	5.45	CGGTGTGCCAAGGAAGAGTTCTTGTTTATGTGTATCTTGTTTAATTTTTTACC[A/G]ATTAGCCTACTAGGATGATCTATTCCCCTTTTTAACTTGGTTTAGATTAATTACAGTATAT	A	G	0.40	0.45	0.48	0.45	0.48	0.27	0.85	0.40	0.46	0.35	0.42	0.002	0.120	0.000	0.007	0.0003	1		
rs1920469	8	5.82	CATAGAAACAGAGAGTAGAATGGTATTTGCCAGGGGCTTAGAGAGGGAAAAAAG[A/G]AGATGTCTTCAACAGTTAGAAAGTTTCAGTTTCATAAAACGAGTATGTTTTTGA	A	G	0.36	0.68	0.66	0.70	0.61	0.90	0.85	0.43	0.68	0.68	0.68	0.072	0.008	0.060	0.026	0.0001			
rs890016	8	5.97	TGTAGACTGGATTAATCGCCTTCCCTTTTATCCACACACAGAGGCACTGTAT[A/G]TCCTCATTCCCTCCCACATGTTGGCAGAGAAAATCTGGAAACGCACTGGCCTTTCTCT	A	G	0.93	0.57	0.56	0.62	0.71	0.82	0.65	0.68	0.64	0.58	0.66	0.101	0.003	0.000	0.032	0.0072		1	1
rs2442475	8	6.34	ATTTCAAGTGTATTCTGGGGACTTCTGAAAATATTAGCCAATGTTAGAAACAAAGTC[A/G]CAAACCCAAAGGGATTGCTTTTGAATTTAGGCTTGTGATCCATCTTCTTTTAATTCA	A	G	0.12	0.27	0.23	0.24	0.28	0.09	0.18	0.11	0.43	0.22	0.18	0.009	0.002	0.027	0.012	0.0027	1		
rs2442567	8	6.45	ATGAGTAGGTTTTGCAGATTTGACAGCAATGTCCTGGCCTAATCCTTTGGAAAGG[A/G]CAAGAAATTCCAACCTGAAGATTTCTGTGTCTCCAGCAACTCAGCTCCAGATATT	A	G	0.23	0.00	0.02	0.04	0.00	0.50	0.06	0.01	0.07	0.01	0.01	0.035	0.000	0.002	0.155	0.0033			
rs2911981	8	6.56	GACAAAAAGTATTCCAATTAAGAGCGAGAATCTTGAAGAGTTGAACCTTAT[A/C]TCCAGATGACATACGGAATGGCAGAAATAGAATTACACCTAGGTTTCTCACT	A	C	0.93	0.95	0.95	0.98	0.98	1.00	0.98	0.98	1.00	0.96	0.96	0.001	0.002	0.005	0.018	0.0006			
rs890232	8	6.65	TTTCACGGGTATCAGGCCCTCCCCAGAGGCTACTGCTGAGGGTTTTCTTCT[C/G]CAAGCCCTACCTAATAGGCCCTTGGCTGTTCTTCCAAAACCTCTTCTCTATGCTGAAAA	C	G	0.67	0.55	0.55	0.54	0.50	0.80	0.72	0.74	0.68	0.56	0.50	0.003	0.013	0.019	0.027	0.0034			
rs890230	8	6.65	AAGTTAACATCTCTCAGGGAGGAAATCTTCCATGGAAAAATACCATGGAAAAATCCTACTACTTTGACC[A/G]CACACATACATTCATTATGGCACAACCGTAATTACAACCTTGCC	A	G	0.21	0.43	0.45	0.46	0.50	0.25	0.28	0.20	0.32	0.47	0.48	0.019	0.011	0.040	0.013	0.0020			
rs751009	8	6.74	ATCAAAGCAAACTGCTTGTGTTGTGTCCATAAGCGGCAAGCACTGCCAGACACTC[A/C]CAGCCATCTGGAAGAGCAGAGCTGGATGCTCCCAGCAAGGCCCAACCAGAATCTTC	A	C	0.64	0.80	0.78	0.80	0.71	0.73	0.28	0.66	0.54	0.88	0.78	0.008	0.145	0.018	0.001	0.0002			
rs4392921	8	6.90	TCCTCGGGCCCAAAGCTATTAAAGTGAGCATCAAGTCTCTGGTCAGGAGGACAGCA[A/G]TGTAATGCAAAAACCAAGATAGACAGAGATGTTCTTCACCCTTCCTTCAGGCAAG	A	G	0.48	0.50	0.47	0.56	0.50	0.36	0.29	0.48	0.50	0.54	0.56	0.000	0.014	0.001	0.004	0.0072	1	1	1
rs433960	8	8.81	AGACCACATTAAAAGCAACTAAATGATGATACAGACGTTTAGCATCTTGCGGCTTT[A/G]GACACTAGCTAGAGATGTGTCGGGAAATTCTGTATGGAAGGAGCACTCTGGGAGC	A	G	0.36	0.68	0.65	0.70	0.61	0.86	0.96	0.84	0.75	0.67	0.56	0.044	0.100	0.047	0.032	0.0088	1	1	1
rs2028806	8	8.97	CAGAAAACCTGCTAGACAAATTCCAAAAGAGCTGTAACACAGCGATTTCAGAC[A/G]TTTAATCACCTCAAGAGAAAACCTGAAGGATCTCCGTCGCCGCCTCTATCTCTGTC	A	G	0.17	0.30	0.31	0.32	0.21	0.59	0.81	0.60	0.61	0.27	0.27	0.027	0.102	0.013	0.021	0.0010			
rs753012	8	9.02	GTATTTGAGTTTTATTCCAATGTCCTGCCATTAGGACCAAGATCAGGCTT[A/G]TTTGGATTCACAAGCTGCCACACTGGGTTCTTCATAATCACTGGCAACATTGTCACT	A	G	0.10	0.30	0.38	0.39	0.25	0.50	0.67	0.47	0.21	0.34	0.33	0.077	0.033	0.003	0.004	0.0019			
rs735449	8	9.14	TGCAAGTAGTTTCTAATTCTTTGTTCATTCAATGACTGTTTATTGGACGGCTA[A/C]TATTGATGCCAGATGCTATTCTGGGTGCTATACGGGTATCAGTGAATCACGTAGAGTTCCT	A	C	0.29	0.42	0.40	0.41	0.43	0.09	0.36	0.32	0.75	0.43	0.40	0.006	0.001	0.003	0.064	0.0001			
rs718742	8	10.65	TAACTGAACTGGAAGAGGGACAATCGGAGTGTATTCGGGAGACATGAGCCAGT[A/C]GGTCTTCCAATCAGGGGACCCTTCCCAGCATTCATCCCTGAAACAAGCAGTCCTGC	A	C	0.52	0.75	0.68	0.78	0.73	0.41	0.54	0.79	0.82	0.73	0.68	0.010	0.007	0.016	0.033	0.0004	1		
rs1544981	8	10.72	GCATACTGACTGCAAGGAAAGCTAAATTCACAGCTCACCCTACTCCCGCTATGCC[A/C]TCAAGATGCAATTCCTGGGTCTCTGGGTTCTTCATTTAAAAATAGATCTCATTTGC	A	C	0.17	0.18	0.29	0.18	0.29	0.45	0.03	0.20	0.14	0.26	0.29	0.018	0.018	0.001	0.001	0.0000			
rs1047950	8	11.22	CTCATTGTTTAAAACCTTACTGATCTAATACCATCTACACAAAAAAATGTTGTA[C/G]TTGCAGAACTTAGTTTATAAAAACAAAGAATGTGTATTTCTTCAATAGCCGGGTATTTGC	C	G	0.55	0.53	0.54	0.59	0.38	0.91	0.90	0.70	0.64	0.51	0.55	0.005	0.051	0.000	0.056	0.0010	1		
rs1390950	8	11.63	GAGAAGAATCTATACATGGATTTGTTTATATACAGAATGAACATTTTAGGAGGACAGACAAGAAACTGATCAT[A/G]CTGGTGTTTCCAGGGAGGTGTCTTGGTGGCTCACGG	A	G	0.90	0.61	0.58	0.51	0.84	0.95	0.52	0.60	0.58	0.70	0.74	0.077	0.000	0.001	0.123	0.0197		1	1
rs12898	8	11.74	TGTGGTTCCCCACGGGGTAGCATCTTGGTTATGTGAGATCACCAAGACACCAA[A/G]CCTGTTTTATGAGCCTGAATCCTCAGCTTGTTGCTGGATTTGTGGCTGATAAAAATAC	A	G	0.36	0.63	0.62	0.63	0.70	0.55	0.25	0.39	0.50	0.67	0.65	0.015	0.038	0.004	0.001	0.0019			
rs2203837	8	12.83	AAGTAGAACAAACTATCAGACAGATCATAAATTCTCTGGCTCAAAAATCCTA[A/G]GGAAATTATGTAACCAGACTTTTATTGCAGTGAGGTTTCATCATAATGTATGTATG	A	G	0.67	0.56	0.51	0.48	0.55	0.86	0.94	0.50	0.61	0.48	0.45	0.014	0.108	0.002	0.070	0.0027	1		
rs721956	8	13.20	TCTTAAAATTTAAAAAGTAGTTTATAGTTGAACTCTACTGTACTTTAGTCAA[A/G]TTCTCTACCCATTTGCCTGATTTCCTTCAATCCTGCTAGCTGCTATTTCTCACACTCCTTT	A	G	0.21	0.38	0.39	0.33	0.36	0.59	0.13	0.21	0.32	0.46	0.36	0.011	0.045	0.013	0.026	0.0000			
rs1478043	8	13.95	AGAGATCTTAAATTATATGAGCTCATTGCATGCTTCAAAAGGTCTCATTTCAGAGAA[A/C]AAAAGGTTTCCATTGCCAAAACTTTGCTGCATTTTCTCTCCCCAAAATATACC	A	C	0.45	0.86	0.89	0.93	0.88	0.68	0.78	0.84	0.79	0.92	0.87	0.138	0.015	0.001	0.037	0.0018	1		
rs7841810	8	14.03	CAAGCATTCAATTAGAACGGAGGCAAAGGACTGATAGTCTTGGCTTTCAAACAAAC[A/G]CTAGGGCACTGGGTAGATGCAGAGTTGGATTTCACGATTCTTCAGGTTTTAGT	A	G	0.50	0.32	0.35	0.27	0.45	0.18	0.33	0.32	0.46	0.41	0.29	0.007	0.000	0.000	0.012	0.0059			1
rs1125265	8	14.87	TTATCAAAAAAGTATCTGTTAGTTCAAGTTTGGTGGGAGAAGAATTTCTCATTCC[A/G]TTTTATGTTGTGCATGGCAATTAAAATAAAATAGCACAGCAATTATTCCTAAGAATCT	A	G	0.38	0.51	0.51	0.61	0.52	0.41	0.44	0.49	0.64	0.46	0.52	0.002	0.001	0.001	0.000	0.0006	1		
rs990544	8	15.32	GAGGAGGAAAAAAACTCAACGACAACTTGAAAACGCTTTAAACATTTTGTTGTAT[A/G]TAGTTCAGATCTTGTAATAGAGAACATTCTTTTAAATAAAATACCAAAAGCATTGAC	A	G	0.64	0.24	0.26	0.20	0.16	0.35	0.05	0.42	0.25	0.30	0.22	0.072	0.055	0.009	0.002	0.0035			
rs765262	8	15.91	GTTCTTGATCTTCTAGGACACAGCACATGAGTAGTAAACATCCTCTCCTTCAAT[C/G]ATCTCACAGAATTGTTTGGTTGAGAAATCCTGTGTTTAATCACCACATATTCTTATCTGC	C	G	0.81	0.73	0.68	0.70	0.64	0.91	0.96	0.69	0.89	0.68	0.75	0.014	0.071	0.000	0.046	0.0018	1		
rs1393045	8	16.63	GTAATGATGTGCTTAATACGTAAGAATATACAGGAACCAAATTAGGCCAGGCCTA[A/G]GGGGAGATGAGGACTGCTCCCAATTAGGTTACCCTCACTAAAACAAGGTTACAGAGGACGG	A	G	0.24	0.43	0.48	0.46	0.36	0.59	0.48	0.55	0.46	0.46	0.43	0.040	0.000	0.000	0.002	0.0001	1		
rs1043883	8	17.51	CAGTCCTGCAGACCTGCATCAAAATGCTTTCAGAGAGTCTGTGGACTTGGGGA[C/G]TGTGTCATCTGGGTTGGGTGAAATGCTGGGGCTAGGGAAGGAGCCCGAATTCCTTGGTGA	C	G	0.33	0.53	0.62	0.56	0.63	0.50	0.25	0.44	0.54	0.72	0.70	0.036	0.079	0.015	0.008	0.0021	1		
rs2979784	8	17.61	AACCCTGTCCCTTGGTGGTGAAGAAAAAGCTCTGGTCCTTACTGTAGTTGTCCA[A/G]TAAGGCTCATGGTGCTTGCAGAGAATGGGAGCTCCTTCCTCTCTGTGAGGAAC	A	G	0.74	0.85	0.80	0.74	0.84	0.73	0.90	0.84	0.79	0.83	0.78	0.002	0.011	0.001	0.002	0.0001			
rs450904	8	17.70	GAAGAAGAGGAAACATAGGAGTAAGAGGTCAAAGAGCTAAGAACTTCTCGGGCCAA[C/G]TGGTCAGGAAGAACTTAGGAAAATTCATTTTTATCAGTGAGTAACTGGAAAGAG	C	G	0.10	0.17	0.19	0.26	0.14	0.41	0.67	0.52	0.11	0.17	0.12	0.004	0.143	0.079	0.038	0.0039			
rs3739406	8	17.75	CAGCTTTTGCAGGAGAATGAAGTCCAGTTCCTTGATAAAGGAGATGAGAATACTGTC[A/G]TTGATCTTGGAAGCAAGGGCAGTATGCAGGTCAGAATGGACTTTTCTTAAGTC	A	G	0.78	0.29	0.32	0.44	0.29	0.40	0.88	0.56	0.21	0.35	0.31	0.113	0.177	0.029	0.004	0.0000			
rs208770	8	17.81	AGTAAAATGTGCCCCAGTTCACCAGTAGGTGAATAAAGTAGCTCATTATTTACA[A/G]AAACTAGTGGCATTGGAGAAGAGCAAATAAACATGAAGCATAATAGGTAACCATTGGA	A	G	0.74	0.41	0.35	0.54	0.36	0.77	0.83	0.57	0.25	0.42	0.32	0.114	0.188	0.059	0.137	0.0001			
rs208062	8	17.83	TGAATCTATGGCCAGCCAGCCCAGTAAAGAATGAAAATAATGAGTCAATTCCTAT[A/G]TGCTGTCTTTACACTGTAAACGTATAACCCCAACTCTTCATTAGTTCATCCACTTCA	A	G	0.33	0.61	0.66	0.45	0.64	0.23	0.17	0.40	0.75	0.61	0.70	0.086	0.196	0.083	0.144	0.0001			
rs6991832	8	18.72	TATTCTTCAATCCCAGGCATCTTGAAAATGGACATAAATATCAACGGACTAGGCTCA[A/C]TTCCCCTCTTTTAGCATCGTTGACCTCACTTATCCCTGCCTGTTGAACATCACAA	A	C	0.31	0.47	0.47	0.43	0.52	0.09	0.66	0.33	0.61	0.42	0.48	0.021	0.008	0.032	0.114	0.0000	1		
rs334206	8	18.92	TTAGTCTGGAAACTGTTTCTTGTAGTTTCTCTAGCTGTGCTTTATTACCTAAACTCA[A/G]ATGTCATTTGAGGAATGGGGTGGAGGCTTCATTGTTAATTCAGCTAATTCCAGGAAG	A	G	0.07	0.35	0.37	0.33	0.23	0.55	0.31	0.26	0.39	0.29	0.36	0.056	0.000	0.003	0.032	0.0003			
rs871667	8	19.16	CCTCCAGGTATCCTCACTCTTCTTCCCATTCCTCAAGTATTCTTTGTTCTGTTCCA[A/G]ACAGCCACCCGTCTTTCAATTCCAGGCCATGTCTACAGAGAACTGAGTTTAGCTTC	A	G	0.55	0.58	0.56	0.63	0.57	0.17	0.34	0.38	0.68	0.53	0.60	0.000	0.025	0.024	0.083	0.0012			
rs1552779	8	19.54	CCCACACATGGCAATTTTTTCCATCAAGCTCATGGAATTTCTTCGGCTTTCCCAT[C/G]TCTCCATCAGCTTCTCTGTATATCAAATACTCTATGTATCAGAGGAGAACTCTTCCTT	C	G	0.62	0.42	0.42	0.58	0.39	0.64	0.65	0.52	0.46	0.47	0.37	0.031	0.035	0.013	0.031	0.0008	1		
rs966773	8	19.76	CAAAGCTATCAAAAGGTTT[A/G]CAAATTATTCAGGTACTTGATGTCATATAGGTAACTTACCTCATAACTTCTATGTTGTCTTTTGGTGTAATACAGACAGACTCAAAGTCATGAGGC	A	G	0.67	0.34	0.28	0.26	0.41	0.27	0.28	0.44	0.36	0.29	0.32	0.095	0.001	0.022	0.001	0.0032			
rs7814176	8	19.93	AAAGCCACTTCCTGTCAAGGTTACTAGTGATGGGAGGCAGATCCCACCTTAACCTC[A/G]ACTGTGCTGTGGATACTCATACCTCCTATTTAATACGACATTATCAGGCCAGGTGCCAC	A	G	0.17	0.31	0.36	0.48	0.45	0.09	0.16	0.23	0.21	0.40	0.38	0.020	0.036	0.011	0.051	0.0017			
rs4922151	8	20.15	GGTGTGGAGGAAGAACTGAAGTAGAAACAGGCTGTCTGCTTGGAAATGGCTTCCA[A/G]TGTAAACTGCTGGGAATCTAAGCCACTCTGACCTCAGCACCATCCTATCAGCTATG	A	G	0.31	0.38	0.38	0.39	0.36	0.27	0.40	0.42	0.32	0.40	0.40	0.001	0.002	0.007	0.002	0.0007	1		
rs1868660	8	20.50	CTCTATTCCATCCCTCACTGATCTATCCCTTTGGCCTTGAAGTTCTAATTATTTC[A/G]GTGGTGACTCCTTGCAAGCTAGGCCCTTGTCTTGTAATTCTAGGTATTCCACGGAGTG	A	G	0.48	0.29	0.22	0.18	0.23	0.00	0.33	0.30	0.21	0.20	0.21	0.034	0.002	0.006	0.090	0.0007			
rs557838	8	[illegible]	[illegible]	[illegible]	[illegible]	[illegible]	[illegible]	[illegible]	0.48	0.43	0.85	0.74	0.43	0.79	0.55	0.54	0.100	0.060	0.000	0.103	0.0046	1		
rs898249	8	[illegible]	[illegible]	[illegible]	[illegible]	[illegible]	[illegible]	[illegible]	0.43	0.50	0.00	0.06	0.34	0.39	0.38	0.27	0.074	0.131	0.022	0.189	0.0096		1	1
rs1904899	8	[illegible]	[illegible]	[illegible]	[illegible]	[illegible]	[illegible]	[illegible]	0.61	0.43	0.31	0.74	0.49	0.57	0.54	0.54	0.071	0.000	0.010	0.008	0.0033	1		
rs7017274	8	[illegible]	[illegible]	[illegible]	[illegible]	[illegible]	[illegible]	[illegible]	0.17	0.18	0.09	0.09	0.02	0.04	0.12	0.12	0.027	0.000	0.014	0.000	0.0001			
rs4427168	8	[illegible]	[illegible]	[illegible]	[illegible]	[illegible]	[illegible]	[illegible]	0.87	0.84	0.95	0.66	0.77	0.68	0.83	0.84	0.129	0.027	0.000	0.016	0.0005			
rs4871996	8	[illegible]	[illegible]	[illegible]	[illegible]	[illegible]	[illegible]	[illegible]	0.82	0.89	0.86	0.88	0.93	0.86	0.86	0.84	0.005	0.003	0.017	0.001	0.0005			
rs2306518	8	[illegible]	[illegible]	[illegible]	[illegible]	[illegible]	[illegible]	[illegible]	0.61	0.52	0.68	0.78	0.48	0.82	0.63	0.60	0.051	0.026	0.007	0.001	0.0003	1		
rs900267	8	[illegible]	[illegible]	[illegible]	[illegible]	[illegible]	[illegible]	[illegible]	0.55	0.52	0.68	0.78	0.40	0.82	0.56	0.56	0.030	0.046	0.009	0.007	0.0001			
rs2466216	8	[illegible]	[illegible]	[illegible]	[illegible]	[illegible]	[illegible]	[illegible]	0.79	0.80	0.91	1.00	0.93	0.82	0.85	0.78	0.025	0.065	0.014	0.008	0.0023			
rs3924018	8	[illegible]	[illegible]	[illegible]	[illegible]	[illegible]	[illegible]	[illegible]	0.70	0.79	0.77	0.97	0.92	0.64	0.67	0.63	0.037	0.112	0.036	0.002	0.0030	1		
rs310319	8	[illegible]	[illegible]	[illegible]	[illegible]	[illegible]	[illegible]	[illegible]	0.34	0.48	0.14	0.04	0.24	0.54	0.46	0.49	0.000	0.137	0.034	0.066	0.0001	1		
rs879958	8	[illegible]	[illegible]	[illegible]	[illegible]	[illegible]	[illegible]	[illegible]	0.56	0.43	0.68	0.64	0.61	0.29	0.57	0.45	0.005	0.023	0.015	0.037	0.0002	1		
rs901170	8	[illegible]	[illegible]	[illegible]	[illegible]	[illegible]	[illegible]	[illegible]	0.68	0.61	0.73	0.80	0.42	0.68	0.66	0.67	0.080	0.014	0.025	0.005	0.0000	1		
rs719587	8	[illegible]	[illegible]	[illegible]	[illegible]	[illegible]	[illegible]	[illegible]	0.38	0.38	0.32	0.38	0.49	0.36	0.46	0.45	0.005	0.007	0.000	0.007	0.0015	1		
rs385351	8	[illegible]	[illegible]	[illegible]	[illegible]	[illegible]	[illegible]	[illegible]	0.40	0.43	0.32	0.43	0.67	0.36	0.57	0.46	0.007	0.001	0.022	0.006	0.0006			
rs388047	8	[illegible]	[illegible]	[illegible]	[illegible]	[illegible]	[illegible]	[illegible]	0.32	0.34	0.27	0.43	0.60	0.25	0.39	0.27	0.047	0.006	0.050	0.000	0.0001			
rs1561817	8	[illegible]	[illegible]	[illegible]	[illegible]	[illegible]	[illegible]	[illegible]	0.35	0.48	0.41	0.80	0.56	0.46	0.62	0.58	0.023	0.063	0.004	0.001	0.0073		1	1
rs9797	8	[illegible]	[illegible]	[illegible]	[illegible]	[illegible]	[illegible]	[illegible]	0.46	0.50	0.09	0.27	0.38	0.50	0.54	0.48	0.006	0.040	0.011	0.131	0.0012	1		
rs2015443	8	[illegible]	[illegible]	[illegible]	[illegible]	[illegible]	[illegible]	[illegible]	0.28	0.36	0.41	0.47	0.38	0.21	0.24	0.29	0.023	0.004	0.000	0.001	0.0002			
rs352436	8	[illegible]	[illegible]	[illegible]	[illegible]	[illegible]	[illegible]	[illegible]	0.57	0.41	0.82	0.69	0.67	0.54	0.58	0.55	0.009	0.002	0.004	0.026	0.0010	1		
rs189523	8	[illegible]	[illegible]	[illegible]	[illegible]	[illegible]	[illegible]	[illegible]	0.67	0.73	0.73	0.76	0.66	0.82	0.74	0.70	0.000	0.006	0.000	0.005	0.0051			1
rs241202	8	[illegible]	[illegible]	[illegible]	[illegible]	[illegible]	[illegible]	[illegible]	0.57	0.46	0.27	0.34	0.45	0.46	0.53	0.51	0.000	0.026	0.009	0.040	0.0051			
rs1872923	8	[illegible]	[illegible]	[illegible]	[illegible]	[illegible]	[illegible]	[illegible]	0.38	0.41	0.73	0.41	0.57	0.57	0.44	0.48	0.061	0.008	0.007	0.025	0.0007			
rs1836851	8	[illegible]	[illegible]	[illegible]	[illegible]	[illegible]	[illegible]	[illegible]	0.29	0.32	0.05	0.32	0.13	0.21	0.35	0.30	0.080	0.010	0.005	0.037	0.0001	1		
rs1383895	8	[illegible]	[illegible]	[illegible]	[illegible]	[illegible]	[illegible]	[illegible]	0.65	0.54	0.36	0.34	0.42	0.68	0.63	0.58	0.030	0.021	0.006	0.013	0.0029			
rs1481747	8	[illegible]	[illegible]	[illegible]	[illegible]	[illegible]	[illegible]	[illegible]	0.71	0.75	0.77	0.99	0.73	0.64	0.80	0.80	0.026	0.084	0.002	0.002	0.0006	1		
rs7848	8	[illegible]	[illegible]	[illegible]	[illegible]	[illegible]	[illegible]	[illegible]	0.68	0.71	0.68	0.25	0.58	0.57	0.66	0.64	0.021	0.062	0.001	0.009	0.0013	1		
rs8685	8	[illegible]	[illegible]	[illegible]	[illegible]	[illegible]	[illegible]	[illegible]	0.66	0.61	0.64	0.20	0.52	0.54	0.59	0.62	0.008	0.076	0.000	0.006	0.0019			
rs1522845	8	[illegible]	[illegible]	[illegible]	[illegible]	[illegible]	[illegible]	[illegible]	0.82	0.88	0.82	0.76	0.77	0.82	0.85	0.84	0.173	0.006	0.002	0.000	0.0002	1		
rs930706	8	[illegible]	[illegible]	[illegible]	[illegible]	[illegible]	[illegible]	[illegible]	0.37	0.46	0.55	0.52	0.50	0.32	0.47	0.40	0.027	0.018	0.022	0.028	0.0007			
rs23242	8	[illegible]	[illegible]	[illegible]	[illegible]	[illegible]	[illegible]	[illegible]	0.38	0.32	0.36	0.29	0.22	0.46	0.40	0.33	0.001	0.000	0.004	0.005	0.0000	1		
rs1000236	8	[illegible]	[illegible]	[illegible]	[illegible]	[illegible]	[illegible]	[illegible]	0.43	0.46	0.41	0.54	0.51	0.50	0.53	0.53	0.013	0.000	0.003	0.003	0.0001	1		
rs7295	8	[illegible]	[illegible]	[illegible]	[illegible]	[illegible]	[illegible]	[illegible]	0.34	0.39	0.14	0.34	0.31	0.29	0.36	0.25	0.000	0.005	0.000	0.010	0.0016			
rs1901386	8	[illegible]	[illegible]	[illegible]	[illegible]	[illegible]	[illegible]	[illegible]	0.54	0.45	0.59	0.59	0.75	0.46	0.52	0.49	0.028	0.008	0.063	0.008	0.0002	1		
rs1481058	8	[illegible]	[illegible]	[illegible]	[illegible]	[illegible]	[illegible]	[illegible]	0.52	0.57	0.41	0.52	0.38	0.64	0.57	0.51	0.023	0.000	0.016	0.012	0.0002			
rs1451687	8	[illegible]	[illegible]	[illegible]	[illegible]	[illegible]	[illegible]	[illegible]	0.60	0.61	0.27	0.67	0.63	0.46	0.61	0.56	0.034	0.007	0.005	0.040	0.0000	1		
rs868586	8	[illegible]	[illegible]	[illegible]	[illegible]	[illegible]	[illegible]	[illegible]	0.50	0.50	0.86	0.41	0.53	0.36	0.50	0.48	0.048	0.008	0.000	0.062	0.0000			
rs1127379	8	[illegible]	[illegible]	[illegible]	[illegible]	[illegible]	[illegible]	[illegible]	0.61	0.48	0.36	0.57	0.38	0.64	0.60	0.58	0.000	0.000	0.019	0.020	0.0000	1		
rs749540	8	[illegible]	[illegible]	[illegible]	[illegible]	[illegible]	[illegible]	[illegible]	0.51	0.46	0.32	0.14	0.35	0.32	0.65	0.62	0.001	0.085	0.024	0.028	0.0039			
rs474059	8	[illegible]	[illegible]	[illegible]	[illegible]	[illegible]	[illegible]	[illegible]	0.38	0.36	0.23	0.20	0.35	0.36	0.57	0.54	0.000	0.020	0.003	0.020	0.0094		1	1
rs801100	8	[illegible]	[illegible]	[illegible]	[illegible]	[illegible]	[illegible]	[illegible]	0.45	0.25	0.18	0.18	0.31	0.43	0.35	0.31	0.050	0.008	0.001	0.012	0.0005			
rs1955185	8	[illegible]	[illegible]	[illegible]	[illegible]	[illegible]	[illegible]	[illegible]	0.78	0.73	0.73	0.74	0.71	0.68	0.76	0.74	0.179	0.004	0.001	0.002	0.0010	1		
rs1548128	8	[illegible]	[illegible]	[illegible]	[illegible]	[illegible]	[illegible]	[illegible]	0.32	0.32	0.27	0.01	0.54	0.36	0.42	0.28	0.197	0.100	0.033	0.001	0.0001	1		
rs902619	8	[illegible]	[illegible]	[illegible]	[illegible]	[illegible]	[illegible]	[illegible]	0.32	0.32	0.32	0.01	0.54	0.36	0.42	0.28	0.191	0.100	0.033	0.000	0.0000			
rs715906	8	[illegible]	[illegible]	[illegible]	[illegible]	[illegible]	[illegible]	[illegible]	0.49	0.55	0.36	0.29	0.36	0.36	0.48	0.43	0.029	0.031	0.046	0.013	0.0010	1		
rs898520	8	[illegible]	[illegible]	[illegible]	[illegible]	[illegible]	[illegible]	[illegible]	0.66	0.73	0.91	0.86	0.25	0.86	0.66	0.71	0.001	0.026	0.015	0.040	0.0003	1		
rs718251	8	[illegible]	[illegible]	[illegible]	[illegible]	[illegible]	[illegible]	[illegible]	0.59	0.64	0.50	0.60	0.65	0.50	0.66	0.73	0.196	0.000	0.001	0.008	0.0021	1		
rs185596	8	[illegible]	[illegible]	[illegible]	[illegible]	[illegible]	[illegible]	[illegible]	0.57	0.34	0.50	0.44	0.54	0.36	0.43	0.36	0.100	0.004	0.015	0.008	0.0037			
rs716583	8	[illegible]	[illegible]	[illegible]	[illegible]	[illegible]	[illegible]	[illegible]	0.74	0.61	0.64	0.59	0.72	0.57	0.68	0.66	0.000	0.001	0.017	0.000	0.0005	1		
rs1837630	8	[illegible]	[illegible]	[illegible]	[illegible]	[illegible]	[illegible]	[illegible]	0.56	0.48	0.68	0.53	0.39	0.39	0.54	0.55	0.059	0.001	0.005	0.009	0.0003			
rs731239	8	[illegible]	[illegible]	[illegible]	[illegible]	[illegible]	[illegible]	[illegible]	0.51	0.45	0.27	0.15	0.41	0.57	0.58	0.46	0.007	0.060	0.005	0.020	0.0001	1		
rs1425911	8	[illegible]	[illegible]	[illegible]	[illegible]	[illegible]	[illegible]	[illegible]	0.49	0.57	0.91	0.99	0.64	0.43	0.45	0.58	0.059	0.196	0.009	0.092	0.0007			
rs1437785	8	[illegible]	[illegible]	[illegible]	[illegible]	[illegible]	[illegible]	[illegible]	0.74	0.82	0.50	0.72	0.70	0.79	0.73	0.69	0.083	0.002	0.003	0.012	0.0001	1		
rs1011925	8	[illegible]	[illegible]	[illegible]	[illegible]	[illegible]	[illegible]	[illegible]	0.60	0.59	0.82	0.69	0.73	0.61	0.57	0.52	0.109	0.002	0.001	0.021	0.0027			
rs1013087	8	[illegible]	[illegible]	[illegible]	[illegible]	[illegible]	[illegible]	[illegible]	0.56	0.59	0.64	0.63	0.77	0.61	0.54	0.56	0.114	0.005	0.021	0.005	0.0001			
rs919023	8	[illegible]	[illegible]	[illegible]	[illegible]	[illegible]	[illegible]	[illegible]	0.60	0.59	0.55	0.94	0.52	0.64	0.57	0.65	0.009	0.068	0.017	0.006	0.0000	1		
rs985942	8	[illegible]	[illegible]	[illegible]	[illegible]	[illegible]	[illegible]	[illegible]	0.49	0.43	0.14	0.34	0.50	0.36	0.43	0.40	0.014	0.008	0.004	0.071	0.0010			
rs1317890	8	[illegible]	[illegible]	[illegible]	[illegible]	[illegible]	[illegible]	[illegible]	0.72	0.71	0.64	0.90	0.61	0.64	0.71	0.73	0.250	0.019	0.013	0.006	0.0014	1		
rs1873104	8	[illegible]	[illegible]	[illegible]	[illegible]	[illegible]	[illegible]	[illegible]	0.69	0.55	0.64	0.52	0.53	0.43	0.64	0.66	0.015	0.007	0.007	0.000	0.0011	1		
rs997493	8	[illegible]	[illegible]	[illegible]	[illegible]	[illegible]	[illegible]	[illegible]	0.32	0.16	0.23	0.05	0.26	0.25	0.39	0.28	0.007	0.042	0.005	0.000	0.0005	1		
rs595255	8	[illegible]	[illegible]	[illegible]	[illegible]	[illegible]	[illegible]	[illegible]	0.38	0.34	0.59	0.41	0.58	0.54	0.39	0.33	0.105	0.001	0.005	0.008	0.0034	1		
rs344278	8	[illegible]	[illegible]	[illegible]	[illegible]	[illegible]	[illegible]	[illegible]	0.59	0.54	0.45	0.24	0.53	0.43	0.48	0.51	0.012	0.040	0.001	0.003	0.0008	1		
rs718710	8	[illegible]	[illegible]	[illegible]	[illegible]	[illegible]	[illegible]	[illegible]	0.66	0.48	0.55	0.73	0.61	0.71	0.61	0.62	0.060	0.014	0.001	0.000	0.0004	1		
rs1351353	8	[illegible]	[illegible]	[illegible]	[illegible]	[illegible]	[illegible]	[illegible]	0.10	0.07	0.27	0.39	0.22	0.32	0.14	0.15	0.021	0.047	0.016	0.025	0.0015			
rs1485506	8	[illegible]	[illegible]	[illegible]	[illegible]	[illegible]	[illegible]	[illegible]	0.46	0.48	0.68	0.58	0.44	0.46	0.45	0.38	0.004	0.015	0.002	0.031	0.0004	1		
rs884839	8	[illegible]	[illegible]	[illegible]	[illegible]	[illegible]	[illegible]	[illegible]	0.89	0.75	0.82	0.58	0.85	0.93	0.85	0.79	0.071	0.028	0.003	0.000	0.0010	1		
rs1108	8	[illegible]	[illegible]	[illegible]	[illegible]	[illegible]	[illegible]	[illegible]	0.55	0.52	0.73	0.25	0.29	0.46	0.43	0.51	0.009	0.048	0.025	0.020	0.0003			
rs1172	8	[illegible]	[illegible]	[illegible]	[illegible]	[illegible]	[illegible]	[illegible]	0.55	0.48	0.27	0.48	0.44	0.50	0.49	0.42	0.109	0.004	0.006	0.044	0.0100	1		
rs1393901	8	[illegible]	[illegible]	[illegible]	[illegible]	[illegible]	[illegible]	[illegible]	0.66	0.66	0.73	0.50	0.63	0.68	0.59	0.53	0.144	0.021	0.014	0.000	0.0122		1	1
rs1434929	8	[illegible]	[illegible]	[illegible]	[illegible]	[illegible]	[illegible]	[illegible]	0.67	0.63	0.36	0.72	0.54	0.58	0.70	0.70	0.001	0.002	0.004	0.054	0.0001	1		
rs766871	8	[illegible]	[illegible]	[illegible]	[illegible]	[illegible]	[illegible]	[illegible]	0.66	0.52	0.45	0.75	0.44	0.54	0.63	0.61	0.001	0.013	0.013	0.013	0.0000			
rs933938	8	[illegible]	[illegible]	[illegible]	[illegible]	[illegible]	[illegible]	[illegible]	0.61	0.57	0.32	0.22	0.34	0.75	0.58	0.55	0.076	0.058	0.025	0.025	0.0000			
rs695167	8	[illegible]	[illegible]	[illegible]	[illegible]	[illegible]	[illegible]	[illegible]	0.50	0.52	1.00	0.77	0.71	0.68	0.48	0.49	0.065	0.048	0.013	0.202	0.0002			
rs166537	8	[illegible]	[illegible]	[illegible]	[illegible]	[illegible]	[illegible]	[illegible]	0.11	0.16	0.41	0.78	0.26	0.14	0.16	0.12	0.099	0.193	0.001	0.026	0.0049	1		1
rs1440369	8	[illegible]	[illegible]	[illegible]	[illegible]	[illegible]	[illegible]	[illegible]	0.48	0.50	0.91	0.32	0.69	0.46	0.53	0.45	0.037	0.030	0.026	0.103	0.0003	1		
rs1007750	8	[illegible]	[illegible]	[illegible]	[illegible]	[illegible]	[illegible]	[illegible]	0.71	0.80	0.18	0.17	0.50	0.68	0.66	0.58	0.000	0.132	0.010	0.112	0.0005			
rs1532577	8	[illegible]	[illegible]	[illegible]	[illegible]	[illegible]	[illegible]	[illegible]	0.79	0.75	0.67	0.80	0.66	0.64	0.73	0.73	0.005	0.000	0.007	0.007	0.0017	1		
rs2016354	8	[illegible]	[illegible]	[illegible]	[illegible]	[illegible]	[illegible]	[illegible]	0.44	0.61	0.64	0.22	0.33	0.29	0.60	0.58	0.040	0.041	0.014	0.008	0.0047			1
rs10701	8	[illegible]	[illegible]	[illegible]	[illegible]	[illegible]	[illegible]	[illegible]	0.58	0.55	0.91	0.94	0.70	0.61	0.57	0.64	0.018	0.082	0.008	0.064	0.0011	1		
rs1464092	8	[illegible]	[illegible]	[illegible]	[illegible]	[illegible]	[illegible]	[illegible]	0.55	0.59	0.32	0.11	0.60	0.61	0.63	0.62	0.047	0.139	0.000	0.041	0.0002	1		
rs1380229	8	[illegible]	[illegible]	[illegible]	[illegible]	[illegible]	[illegible]	[illegible]	0.33	0.46	0.50	0.64	0.30	0.46	0.43	0.46	0.086	0.036	0.001	0.012	0.0058	1		1
rs1483813	8	[illegible]	[illegible]	[illegible]	[illegible]	[illegible]	[illegible]	[illegible]	0.44	0.46	0.50	0.73	0.45	0.54	0.48	0.45	0.096	0.031	0.001	0.000	0.0005	1		
rs906998	8	[illegible]	[illegible]	[illegible]	[illegible]	[illegible]	[illegible]	[illegible]	0.55	0.45	0.45	0.68	0.42	0.61	0.47	0.48	0.003	0.024	0.004	0.001	0.0000	1		
rs879256	8	78.94	GTTTCCACCCTAGCAGGGTCAGAAAACACAGCTAGCCAGTGAAAGTAAAGGA[A/G]GCAGAAAGACATGGTCCCCAATCTTACAGGAAGCAGCACATCCCTCCCTTCCTCTTGA	A	G	0.10	0.38	0.34	0.34	0.32	0.18	0.40	0.20	0.43	0.31	0.32	0.046	0.001	0.007	0.016	0.0003			
rs603733	8	79.97	ATTCACTAGAGCATAGACAGTAGCTGATTTATTTTATCTCTCAGGTAATGGGGCT[A/G]TTTCCACATGACATCAAAACAACCTTTGAAAGAGGGAGAAAAATGATCTAACATGTTT	A	G	0.68	0.67	0.67	0.61	0.70	0.56	0.81	0.67	0.79	0.67	0.64	0.001	0.011	0.002	0.006	0.0000	1		
rs2098898	8	81.02	TCAACTATTCGTCTCTCTCACTCAGGAATCTAGTTTGAGAAGTGAGATGACATTGC[A/G]AGTAGAAGCTGAAAAAACTTGAAGAAGCCTTGAGGTATAGGTAGGCTACAGTGAG	A	G	0.65	0.34	0.39	0.59	0.41	0.36	0.51	0.41	0.36	0.36	0.38	0.043	0.006	0.000	0.000	0.0009	1		
rs2162229	8	81.10	GAGCCTAAGGTGGGGAGGGGAGGATGCGGTGAGCCACA[A/G]GTTCTCAATGTTCAGAGAGCCAGTGTCCTTAAATAGCGAGGTGAAGAGCTCAATTAGGAACCAAAGCCAAA	A	G	0.45	0.48	0.42	0.41	0.43	0.82	0.56	0.40	0.61	0.40	0.40	0.000	0.005	0.001	0.076	0.0012			
rs717340	8	81.10	GAGGCCTCAGTTCCTACTGTGTGGACCTCCACTTGGAGCTGCCTGAGTGTCTTCATGACACACTCTCTCTCGGATCAGAAAGGAGAC[A/G]TTAGTAATGCCAAGGATACTCCTT	A	G	0.79	0.53	0.58	0.59	0.57	0.14	0.36	0.60	0.39	0.60	0.59	0.037	0.013	0.001	0.100	0.0007			
rs915	8	82.62	TAGACTTATTGTAGGTTCGTTTGAGCTTTGTGTTGTAAAATTAAAAATGCTTCT[A/G]TAAAGTTTTCAAGGTAGGGAGTGATTTTATTATTGTGTATATCTAATATATTAAGTATGTG	A	G	0.48	0.36	0.34	0.30	0.25	0.77	0.29	0.24	0.43	0.37	0.38	0.011	0.002	0.005	0.089	0.0031	1		
rs1020490	8	83.89	GAAAACTATAAGAAAAAAAAAAAAGACCTTAAGCAATCAGCACAATTA[C/G]GGTGGGGCAGTTAGCTGATTGAAAGAAACTGGGGAATGATTGGAAGCCAGGGAAATGGAGATG	C	G	0.62	0.40	0.40	0.41	0.41	0.09	0.10	0.41	0.57	0.47	0.44	0.018	0.082	0.000	0.087	0.0009	1		
rs729336	8	85.35	TTTGTGAGCTTCTTTCGTAATAAAGACTCGTCAAATTTTCTACGTTATTTATCTTT[C/G]CCACCTTACGTGATTGTAGGCATCCAGAATTTTCCCACTGAGAGTGTTTATTAATTTG	C	G	0.17	0.43	0.46	0.41	0.29	0.55	0.22	0.67	0.29	0.40	0.42	0.051	0.029	0.042	0.005	0.0014	1		
rs20571	8	86.43	GACCACTGGCATGAACTTTTCCCAAATGCCAAGGGGGAAAACCAGTCGCCC[A/G]TTGAGCTGCATACTAAAGACATCAGGCATGACCCTTCTCTGCAGCCATGGTCTGTGTCT	A	G	0.19	0.63	0.57	0.61	0.66	0.50	0.25	0.46	0.79	0.63	0.60	0.108	0.097	0.029	0.012	0.0000	1		
rs1458132	8	87.72	TTCCATTATATTTTAAAAGTTAAAAAAAAAGAAAAACAGGGTGTCTCAATCTTTATA[A/T]CTCTGATGCTCAATAAATAGGTTTTCAAAAGCAATAGAGCTTTGACATTAGAGTTCC	A	T	0.81	0.53	0.53	0.57	0.57	0.64	0.60	0.48	0.50	0.44	0.49	0.037	0.007	0.003	0.005	0.0014	1		
rs1902866	8	87.81	TCTATTTTAGAGTTTTTTCTAACTAATCTTGTAAGATGATCAGTGAAAATATATT[A/C]CATTGCTCCAGAAGAAAAAGAAATGGAAGAGATATCTCTATTAGAAGATGGCAAGGTTC	A	C	0.48	0.58	0.59	0.59	0.48	0.33	0.39	0.47	0.50	0.67	0.62	0.014	0.038	0.029	0.047	0.0014			
rs1519938	8	89.13	GTGGTACTGGTGGCTATGGGGAGAATTAATTCATTTGTACATCCTCGATTCTAACT[A/G]TGTGTGAAACTATTTTTGTATCACAAATATGCATGGCACAAATCTGACATCTTCATG	A	G	0.55	0.57	0.58	0.67	0.46	0.50	0.56	0.59	0.54	0.52	0.56	0.001	0.001	0.001	0.005	0.0005	1		
rs1879202	8	89.25	GCAACATTAATTTTTAAAAAGGAAAAAAAGTCATTGGAAGGATTCAGTTGAGG[A/G]GGAGGGGTGAAATATATAAATAGAGACAAGATCATCAAAAGTTTTATCTCTTCAGTAAG	A	G	0.26	0.67	0.66	0.72	0.63	0.75	0.58	0.66	0.71	0.60	0.65	0.074	0.008	0.001	0.005	0.0003			
rs999544	8	90.25	AAATAGTTATTGGGAAGGTACTAGAAATTTATAGAAAGCAGTGACATAGATTT[A/G]CAGTTTAGCAAAACAGGAGGATGTGGAGCAGGTGACGTGGAAAGAAGAATCTGTAGGT	A	G	0.55	0.65	0.70	0.71	0.64	0.59	0.67	0.57	0.61	0.60	0.70	0.016	0.000	0.003	0.004	0.0000	1		
rs150615	8	90.59	CTTAAGGGAATCCCATGTGTCTGGGTCCTAGGCACTTCTTACCAATGACAGAT[A/G]ACACCCTAAGAAAAGGAGTTATGGCCAAAAACCATGCACCATATTCCTTGCTGAAAAA	A	G	0.50	0.49	0.46	0.58	0.41	0.27	0.32	0.14	0.61	0.47	0.42	0.000	0.014	0.076	0.020	0.0034			
rs160437	8	90.64	TTTGGATTAGAGGGGAGGCAGGCAAAATAATTTATCTGACAACTTCTTGTAATCTCCTGTTCCTCCTT[A/G]GCTAATATTTTTCCTCCAGGGCCTTAACCAGACCAAACTCCTCT	A	G	0.55	0.42	0.41	0.52	0.50	0.27	0.39	0.28	0.54	0.41	0.40	0.015	0.000	0.016	0.009	0.0005			
rs218902	8	90.69	TTAAGGAAGCTAATAAGAGTCCAAGATTCTCTGAGTGCCTTTGTGAATTATTTC[A/G]TCTGATTCATGTATGTACCTTCATATAATGGCTTTTAATATTGAGGAATATTTGGAGATT	A	G	0.95	0.53	0.57	0.66	0.54	0.36	0.41	0.33	0.68	0.56	0.51	0.104	0.023	0.052	0.028	0.0028			
rs40380	8	90.77	ACAGAATGTGTAATTAGTTTACAGAAAAACAAAATCTGGAAACCTAATCACTAGA[C/G]TGTGAATTTAGAAAGCAGTGAGGTAAAAAGGAGAAAAAATCCTCTCTGTTATGAAAAC	C	G	0.64	0.52	0.55	0.50	0.55	0.59	0.71	0.71	0.39	0.60	0.55	0.008	0.024	0.022	0.003	0.0002			
rs28144	8	90.78	AAATATGATGCTTTGAAAAGAATGCTCTTCAACAATATTTAAGCTGGCCATGC[A/G]TCAATAACATAATACTGCTCTTAAAATGGTAAACTCTTGCACAGGGATGAAATTAGTAG	A	G	0.74	0.53	0.53	0.60	0.46	0.36	0.34	0.21	0.68	0.46	0.56	0.019	0.026	0.065	0.020	0.0000			
rs997898	8	91.86	AGTCTTTAATGCCCTGACTATCTTCTCCCATTTTTTTTATCTGAACTTTGAAGCTAT[A/G]CTTTACACCTATTGTTGTTACATAAGATTTCAAATTATACTTCATTACCACAAGGCAG	A	G	0.83	0.32	0.37	0.34	0.38	0.86	0.81	0.30	0.32	0.35	0.34	0.108	0.094	0.019	0.116	0.0001	1		
rs997899	8	91.86	GTTCAGATAAAAAAAATGGGAGAAGATAGTCAGGGCATTAAAGACTATATACAG[A/G]CTGGTCAACTTCCCTAAAAGAGAGATTAGGTGGGTTGTTTCTTACAGTGCAGAGGTTC	A	G	0.24	0.68	0.63	0.66	0.63	0.14	0.19	0.70	0.68	0.65	0.66	0.073	0.094	0.019	0.116	0.0001			
rs1025908	8	92.86	TTACAACTAACAACGCTATTGTATGTAAGTAGATGGGGTTTGATGTACAGCCAG[A/G]TGTACCAATCCTGAGAAAAGACAATGCTGCATTGCATTATCAATTTAATAAAGCATGT	A	G	0.17	0.57	0.52	0.49	0.55	0.18	0.15	0.17	0.43	0.41	0.48	0.055	0.054	0.052	0.046	0.0002	1		
rs536161	8	93.47	TTTGTTCTAATGTCGTTAATAAGGCGGCTCCACAAACAGGACTAAGTAGGGA[A/T]GTGATCAGGTTGCAGAGTTGGGAGTGTTGTTTTCTTCTGACCACAGGCAGAACTCATCA	A	T	0.57	0.26	0.27	0.26	0.23	0.45	0.65	0.47	0.18	0.21	0.27	0.058	0.085	0.008	0.020	0.0004	1		
rs1449233	8	93.51	CAGTTCTTTTTGACTCCAGGTTCAACCCAACTCTTGGTTCCATTGTTTTAGAAAAT[A/G]CAAAAGGTTTGGTCCTAATCTACAGAATCCTTTAACTACATTTCCAGGTTTTGTTTTC	A	G	0.88	0.53	0.53	0.53	0.55	0.50	0.42	0.61	0.39	0.56	0.50	0.074	0.015	0.000	0.002	0.0025			
rs1483457	8	94.10	CAGCGGTGGACTATTATAACATGGGACATCATGGCTGGCTTACTTGGCTGTTA[C/G]TCATATGATCTAGACAGTACTTTCATATGATAATAGCAAGAGCCATTGCGCTTCTGTC	C	G	0.81	0.70	0.68	0.70	0.66	0.59	0.75	0.80	0.50	0.65	0.56	0.003	0.001	0.001	0.010	0.0056	1		1
rs931476	8	94.35	AATGGGGCTGACATCTAAGCTGGAAGAAACCAGGAACTCAAAGCACAAAGAAAA[C/G]TATTCTTGGCTGAGGCAATGGTATGTAAGAAGTTTCTGAGGCAGGAAAGAACTTGG	C	G	0.43	0.43	0.40	0.51	0.48	0.50	0.77	0.47	0.42	0.38	0.44	0.001	0.051	0.002	0.003	0.0007			
rs911	8	94.89	TGTGTGGCAAAATAGAAAAT[C/G]TTTCAATTACATTAGGAAATCGGGTGGATAACGGAGTATAGTATTATTCCACTTAAGAAGCATTCCAGTCAAATAATCACAAAAACAAATTCAG	C	G	0.38	0.58	0.58	0.54	0.64	0.23	0.06	0.47	0.61	0.45	0.57	0.008	0.149	0.001	0.052	0.0000	1		
rs1051624	8	95.10	AGGTACTCATGCCCGACTGTCTACCAGGCACACAGACTTTGAGGAGAGGG[A/C]GTATGTCGTCTTGATCCGCATCAATGATGGGGGTCGGCCACCCTTGGAAGGCATTGTTT	A	C	0.67	0.39	0.47	0.41	0.38	0.59	0.66	0.45	0.25	0.48	0.46	0.014	0.014	0.000	0.003	0.0002			
rs11785	8	95.67	CAAAGAGCCAGGTATGCTGCATGCTGCTTGCTGTAAGTTACGATTTGGCTTCACT[A/G]GCTCAAATTTTTTCACTCCACCAAAAGATAAGGCACAGGCCCGTTTGTCCAATCAA	A	G	0.07	0.39	0.37	0.43	0.36	0.82	0.45	0.50	0.36	0.42	0.40	0.061	0.013	0.005	0.140	0.0013	1		
rs1441394	8	96.20	CAGGCTCAGAGCCCTCAATGGACATGCCTGCAGACACACAGCATTGGTATAAG[A/G]AACTTTTTAAAGGAATAAAAGAGTCTTGGAAGTTATCAAGTCCAGCCAGGGGTTGCT	A	G	0.69	0.51	0.49	0.49	0.43	0.77	0.89	0.41	0.57	0.54	0.55	0.040	0.012	0.002	0.100	0.0026	1		
rs714552	8	97.14	TAATGTGTCCTGCAGAATGGGCGTATGTGGATCTGAAAGATAAAGAAGAAAAAT[A/T]AAACCCTTGAGTGAGTTTATCAGCTCTTCTGCCTTCCATGTTCTCCAACCACCTCTGC	A	T	0.74	0.66	0.66	0.56	0.66	0.32	0.08	0.55	0.50	0.61	0.60	0.014	0.142	0.001	0.037	0.0003	1		
rs874643	8	97.50	CCAGAAAGATGTAAATAAGCAGAATAAAGCTGGGAGAGGGAAGGCGGTAATGCCT[A/G]AGGAACTAGATGGGCTGGCATATGAGGGACCAGTTAAAAATGGAATCTCTGTGG	A	G	0.86	0.80	0.84	0.77	0.77	0.36	0.36	0.55	0.75	0.84	0.88	0.001	0.132	0.050	0.131	0.0031			
rs714046	8	97.59	CCTCTCCTCTTCTCAGGATATCGGGAACTACTGTCATTATCCATTCCCTCAAT[A/C]AACCTCACTCCTTTTCCTTCACTCCTTTAGCTTTTAACATTATACCTTGGCTGGCTTTGTC	A	C	0.17	0.21	0.22	0.20	0.27	0.14	0.09	0.35	0.32	0.20	0.17	0.001	0.016	0.015	0.005	0.0014			
rs736191	8	99.15	AATTGACAGTTACATAAATATATCTCTAAAACTGTCAGGATGAAAACCATCCC[A/G]TTCTTGACAGGAAGTGAAGTCACAAAAAAAGCTAAACTGACACTGCTATCGGGGGCCTC	A	G	0.50	0.53	0.54	0.48	0.54	0.64	0.45	0.29	0.54	0.54	0.50	0.000	0.005	0.042	0.008	0.0010	1		
rs2453628	8	101.10	CCACAAAAATAAAGTGCTAAATTTAATCATTCCTCTTGTTTTGGAACCTCCTTGGGA[A/G]CCCACAGTACATCCATCAATAAATGTTTGGTCAAYTTTTCTTATTGCTTTCCCTCAC	A	G	0.76	0.61	0.49	0.45	0.39	0.64	0.94	0.60	0.68	0.48	0.53	0.025	0.131	0.007	0.010	0.0002	1		
rs2022922	8	101.43	AGCCCCATAAACCGCCTACCGCCTGGACTTGGCTTCTCTAACAGTTGCTCAAGAAC[A/G]CTGGCACACAGATTGAGAGGCTCAGCACAAAACGCCTCGGTTTTCTTCATTTTCT	A	G	0.64	0.84	0.79	0.87	0.70	0.41	0.69	0.48	0.79	0.70	0.72	0.016	0.014	0.056	0.083	0.0013			
rs1609	8	101.54	AAGGTTTTTCCTTATCTAGCTTCATTTCTCTCATGGTAATTAATGTAATTATGT[A/G]TAGAATTAAGACCACAATAAGGGAGATGATCAGAGGAATCTGTATAGTGTAATTAGAAAA	A	G	0.74	0.51	0.60	0.55	0.63	0.90	0.75	0.76	0.61	0.61	0.64	0.011	0.009	0.015	0.068	0.0002			
rs2155881	8	101.60	CCAGGAAAGCTTAATTTGAAATAAAGTGCTGGGATGGGACTTGCCCTAAAGGAG[A/G]CTTTAGTAATCTCATCAACCCTGAACTGGCGGCTCAYCTGCCTTAACTGTGTTTCCT	A	G	0.50	0.64	0.67	0.70	0.71	0.73	0.56	0.63	0.79	0.69	0.70	0.023	0.077	0.010	0.183	0.0000			
rs614961	8	102.24	TGATGAAATTTTCAGTTAATGATTATTTTAATGGCCCATTGTCTCTGTGTCTTTGTG[A/G]ACTTCCTTATATCTTCAAAAACTTATAAATCATAAAAACAAAATTAAAAAGTTATGAT	A	G	0.57	0.63	0.64	0.73	0.66	0.68	0.77	0.78	0.75	0.64	0.68	0.008	0.008	0.002	0.000	0.0008	1		
rs500318	8	102.25	GTGAGGACACAGACAAACTGTATCAGTACATCTCACAACACCTAGAATCCAGAC[A/G]CCAAGGATGATGCACAACATTTCCCTGTTTTCCCCACCAATTCACCCTTAAGTCTATG	A	G	0.43	0.58	0.61	0.55	0.50	0.50	0.61	0.81	0.57	0.58	0.63	0.035	0.002	0.005	0.024	0.0000			
rs718262	8	102.93	TTCAAATGAGGGCATCACAAAC[A/G]CAGCCTATCAGCCCTGCAATCTCAGGAAGAAGAGAAACCACCTGGAAAGATATTACTAAGAAGTCAATCATGATAATAGTGTGGTACAG	A	G	0.38	0.46	0.49	0.49	0.50	0.32	0.22	0.43	0.57	0.56	0.56	0.001	0.025	0.000	0.009	0.0065	1	1	1
rs930266	8	103.64	CCAGCCACCCGGAGTCCCAGCAGACCACAAATGACTTTTGGAACTGAACACC[A/G]CTGCTGCTGTGCTGATGGGACCTTAGAGCCAGGCCCTAGATGAACCCGTTTCACCAC	A	G	0.43	0.54	0.47	0.55	0.50	0.45	0.40	0.50	0.79	0.60	0.51	0.005	0.008	0.001	0.003	0.0009	1		
rs2131858	8	104.29	CATGTGACAACTACAAATCTAGTCCAGAAGTAATCAGAACACATTTGACAGAAG[A/G]GCAGATGGCTGAAGAAGAAAGGTCTCCAAGGGAAAGGAGCGGGGATTGAGATTGAC	A	G	0.12	0.36	0.37	0.30	0.34	0.64	0.26	0.34	0.39	0.29	0.44	0.074	0.024	0.007	0.011	0.0020	1		
rs1370009	8	104.31	TTATCAACACAGGGCTAAGAAGAAAGCAGAGCCTCTTCTTTTTCACTTTATTTACAAATAGATTTACCAAATACCTCTGTAGTTTGTCTGAGTGA[A/G]TGCTCATTTAGTTATACC	A	G	0.26	0.44	0.46	0.41	0.39	0.72	0.80	0.50	0.43	0.40	0.49	0.041	0.032	0.002	0.014	0.0004			
rs1370008	8	104.31	GAAAAGATACAAGGAGGCAAAACATGAATTACTGCCAAAACTCTGGAAAGAAATAGAAAAGAATTGGAAGCAGCACAGAAAA[A/T]ACACATTTCCTATAACATGATAATGGACA	A	T	0.24	0.44	0.46	0.41	0.39	0.64	0.76	0.47	0.43	0.40	0.49	0.049	0.018	0.007	0.003	0.0004			
rs1460239	8	105.77	GTACATATAGAATATTTCCAATTACACGGAAGACAGCAAAATGTGATCCCTTAAT[A/G]TACTGTATGTGCAACGTGGGTAAGTTAATGCTGTTATGAGAATGTTAAATTTTACAAC	A	G	0.38	0.60	0.56	0.71	0.59	0.36	0.47	0.60	0.54	0.53	0.62	0.014	0.004	0.003	0.013	0.0027	1		
rs169566	8	106.02	GAAGCAGTAAGGATAACTGGTATCACAGAAAATTGGGCTCATGATGTTGAAGAGA[A/T]CTGAAGCTGAGAATACATCTGTAATGCAGAAGAAAATAATACAGAAGAGAAAAGGC	A	T	0.81	0.59	0.61	0.55	0.61	0.73	0.51	0.49	0.79	0.49	0.58	0.016	0.005	0.005	0.003	0.0005			
rs1375956	8	106.52	GGTATCATCTTGGAAGTAAGAGGACTTGGCTAGCAAGCACAGGAAACAGCAGTGG[A/G]ATGTGCAGCAGCTAAAAGTCAGTCTACCAGCTGGAGGGCCACAGAAAGGTCCGT	A	G	0.10	0.46	0.43	0.52	0.48	0.27	0.43	0.28	0.50	0.40	0.40	0.092	0.000	0.015	0.017	0.0006	1		

rs1872283	8	107.77	[illegible]	A	G	0.81	0.60	0.63	0.77	0.50	0.64	0.50	0.63	0.46	0.72	0.64	0.025	0.008	0.000	0.000	0.0000	1		
rs1954728	8	108.08	[illegible]	A	G	0.90	0.61	0.64	0.57	0.59	0.95	0.51	0.86	0.57	0.67	0.68	0.040	0.008	0.043	0.076	0.0002			
rs1370294	8	108.71	[illegible]	A	C	0.21	0.52	0.47	0.39	0.41	0.59	0.06	0.39	0.43	0.41	0.47	0.015	0.087	0.006	0.018	0.0005	1		
rs1457210	8	109.22	[illegible]	A	C	0.64	0.60	0.55	0.60	0.64	0.50	0.35	0.52	0.61	0.56	0.58	0.001	0.046	0.006	0.010	0.0002	1		
rs5772	8	110.06	[illegible]	C	G	0.45	0.57	0.62	0.63	0.48	0.50	0.83	0.72	0.61	0.53	0.61	0.011	0.022	0.012	0.008	0.0000			
rs1783152	8	110.44	[illegible]	A	C	0.64	0.50	0.46	0.49	0.48	0.55	0.23	0.25	0.46	0.54	0.45	0.014	0.038	0.034	0.001	0.0002			
rs1019603	8	113.70	[illegible]	A	G	0.62	0.25	0.28	0.28	0.34	0.41	0.32	0.29	0.24	0.27	0.27	0.052	0.000	0.001	0.012	0.0006	1		
rs996252	8	113.97	TGAAGAAAAAGCATAACTTTGGATTAAGTTTAAAACTAGGATTTGATTCCAGGGAAT[A/G]AGATTGCAGAAACATGATTCTACGATGCTATAGAAACCAGCATTGCTACTATCTTC	A	G	0.10	0.51	0.48	0.50	0.48	0.05	0.00	0.23	0.36	0.42	0.34	0.070	0.170	0.027	0.107	0.0079		1	1
rs727678	8	114.65	AATATACAAATTCACTAATCAGTTGTTGAGTAGAGTAATTAAACTTTTGGGAAATTG[A/C]TAAATAATATTAGTGGTTAATTATACTTACAAATGTAAAAATTTTGCCATATGCTT	A	C	0.29	0.65	0.64	0.68	0.63	0.32	0.16	0.49	0.54	0.58	0.59	0.076	0.151	0.013	0.065	0.0003	1		
rs925760	8	115.18	CATACAAATTGTAAGTGGCTTCTTATTGCTCAAGTAACACAGGTGTCCTTA[A/C]CATGTGTCTTCAATGTACATGTTTAGTTTAATTTTCCATTACTTCCTCCTACGCATGCTA	A	C	0.05	0.49	0.46	0.49	0.46	0.18	0.27	0.37	0.46	0.46	0.45	0.160	0.023	0.019	0.067	0.0000	1		
rs1353277	8	116.04	CCCACTATGTCCCATAATCAGCAGTTTTGTCAACTATCTTTATGAATGAAGCCCC[A/G]ACTAGCTCTCATGAAATTCATAAATTTGTGCCTGCCCAAAGATATGC	A	G	0.50	0.79	0.75	0.79	0.77	0.29	0.54	0.75	0.72	0.77	0.77	0.019	0.063	0.011	0.065	0.0003	1		
rs1494754	8	116.12	AGTCCCAAATGTGACTTCAATTATAAAAGGACCATCCAGCTTGTTCCAGTCAACT[A/G]GAAGAATCATGAGCAATAATACATTTTTGTTTGTTTGTACCCACCTACATTTTGGTTTG	A	G	0.40	0.42	0.50	0.32	0.52	0.45	0.63	0.41	0.38	0.40	0.45	0.010	0.008	0.002	0.002	0.0014			
rs1384810	8	116.44	ACTCCTTAGGGCATAAAGTCAGAGAACAGCAGCTATTCCAGACCACTCCTCTAAAT[A/G]TTAATGAAACTTACATAAATCATACATAACATATACTATCCCTTGTGCTGGAAAA	A	G	0.88	0.55	0.53	0.54	0.64	0.95	0.84	0.67	0.79	0.57	0.60	0.086	0.050	0.002	0.123	0.0028			
rs1628373	8	118.25	GGCATATTCTCCTGTTCAGGAGAATGTACCAACCTTACCATTTAATGTCATTTACT[A/G]TACATTATGACTTACCTTGTGCATCACTTTGTCTAAGAAGTCTAACTGATCCCTTTC	A	G	0.69	0.58	0.52	0.54	0.48	0.36	0.53	0.32	0.71	0.54	0.47	0.035	0.002	0.007	0.005	0.0018	1		
rs963014	8	118.29	CATTTCTTGAACTCTGAGAGACTTTTTCTAGTATCAGTTCTGAGGAGCATTGCT[A/G]TTTTCCTTCCTATCATCTGGAAGACCTAGACTTTAAAACCAACCTGAGAAAAACTT	A	G	0.45	0.51	0.55	0.65	0.61	0.95	0.68	0.54	0.68	0.56	0.59	0.007	0.000	0.002	0.086	0.0014			
rs769322	8	119.63	AAGACACATCCTTTTTATAGGAAATAGGCATGAGAAAATCTCACCCACTTTAA[A/G]AAAGAATCGATAATTGTAATTCACTCCCAAAGCAGTCTTATTTATAAGCTTT	A	G	0.79	0.34	0.36	0.37	0.32	0.59	0.25	0.46	0.25	0.45	0.33	0.105	0.009	0.001	0.024	0.0010	1		
rs715954	8	119.78	AATTCAAGGAGAAAATATTTCTTAAACAAGTGCCAATCAGCACCATTCATGAATGTCCATCC[A/G]AGTATTGTAAACTACAAG	A	G	0.29	0.34	0.27	0.30	0.21	0.45	0.44	0.26	0.39	0.28	0.20	0.000	0.024	0.002	0.034	0.0043			1
rs1346014	8	120.18	CAGAACAGGCAGCTTTAATGAGGTGGGGACATTTGAGGTTAGCCTTCAATAATGAGCCAT[C/G]TTCATTAGAAAATGGGAAAGAGAATTC	C	G	0.07	0.26	0.25	0.26	0.23	0.55	0.33	0.27	0.25	0.27	0.23	0.049	0.000	0.000	0.032	0.0015	1		
rs2034843	8	121.30	ACATAAAAATAACACACATACCTATATATTCTATAATTGAGGAAGATGTTTTT[A/G]TAAAGAACATCACTTGCTTTCTGCCTTAAATCCTTCCACCAGGGAATGAGGAGCAAACA	A	G	0.33	0.62	0.56	0.55	0.68	0.64	0.54	0.51	0.61	0.61	0.62	0.014	0.005	0.003	0.018	0.0047	1		1
rs2034844	8	121.30	ATGAAAGACAGATCCCTCTGATAGAGATTCCCTGCCCAAGACTCTCTAGGTC[A/G]TGAAAGATACTAATTCATCTTTCATCTGGTCCTTAATGACCAATAATGGATCCTCTTT	A	G	0.21	0.57	0.52	0.54	0.64	0.54	0.49	0.61	0.57	0.56	0.54	0.034	0.009	0.005	0.025	0.0036			
rs1433396	8	121.96	TTATTGTGATACAGTATGGAAAATGTATAGTTCCTTAGAGAATAATTGCATTTT[A/G]TGCTGCTTATGGCTATTGGTGGTAATAACAAGGAAGAAATGAAATGAAGGAAAGACAAA	A	G	0.05	0.31	0.35	0.35	0.16	0.00	0.04	0.08	0.18	0.34	0.35	0.064	0.073	0.036	0.123	0.0002	1		
rs1021898	8	122.20	CGATCTCTTAATTATCTTGGACCATGTAAGTGGTTTTCAGTCCTCTCTCTCT[A/G]TTAAATGTGGTGCTTCTTGTGTGTGTGTGCAGAAAATAGAGTTCTAGAATCAGGGAA	A	G	0.12	0.53	0.44	0.50	0.64	0.09	0.26	0.17	0.29	0.46	0.40	0.064	0.023	0.051	0.082	0.0015			
rs755649	8	123.71	CAGGCAGCCAATCCTTCATTGCCTGAGGCCCTTCAGGCTGCACCCTGT[A/G]TGAGGTAGAACAAAGAACACTCCACTAGGATGCAGCAGATTCTCCTTCTGGTTTGACGT	A	G	0.62	0.39	0.45	0.56	0.43	0.32	0.29	0.34	0.36	0.47	0.54	0.029	0.012	0.000	0.004	0.0080	1	1	1
rs748856	8	123.77	AGCTTGGACTAGCAGGGGCCATGCAGGCAGCCTGCTTGCTCAACACCATGC[A/G]GGCTTTCCACGGAAACGTCATGACAGTTCTGTGGGGGCAATCTGGGTGGAAAGGCAA	A	G	0.48	0.45	0.53	0.60	0.45	0.36	0.75	0.46	0.61	0.52	0.55	0.008	0.006	0.026	0.035	0.0002			
rs2833	8	123.94	TATCTGGTGTACGTGTTGCAGTATGAACTCTGGTTGCAATCCCTCCCCCTCCCACT[A/G]ACAATGGGGAGTCCCTCACGTTTTGAGTACAGAGCGGCTGCACAAGTCAAACGCTAGGAAGTTTGAATAAAAC	A	G	0.33	0.54	0.57	0.39	0.61	0.73	0.52	0.55	0.54	0.45	0.53	0.036	0.008	0.005	0.007	0.0002			
rs2001945	8	126.43	TCACACAGGCTCTCAACCAAGCCTTTCACCAACCCAGCAAGGAAAATTCAGAA[C/G]CTTTTACTGAGACTGGGAAACTGATGCCCAGAAAGAGGCCCCAAGACACACAGTTAC	C	G	0.29	0.41	0.52	0.45	0.39	0.35	0.55	0.47	0.32	0.46	0.47	0.031	0.000	0.003	0.020	0.0018	1		
rs921720	8	126.49	TCTGCAGAAGCCTAAGTGTCTTAGTTTGGAAAACTCAGTGTCCTCCTTCTTCCAG[A/G]CCATTCATGCTGTGTGTGGAAATTCCAAACTGTAGCTTAGTTGCCTTGCCCTGAAACT	A	G	0.71	0.35	0.40	0.37	0.34	0.77	0.27	0.66	0.31	0.46	0.40	0.058	0.014	0.024	0.056	0.0000			
rs2124036	8	126.60	ATGCTTTTGCAGAATCCTTTGCTCTTCTTAGGGCAGGTTCAAAGAAATGTCAGCAGAGAGT[A/G]	A	G	0.24	0.79	0.83	0.73	0.80	0.55	0.66	0.56	0.82	0.76	0.75	0.228	0.031	0.074	0.076	0.0090		1	1
rs1488019	8	127.20	TTTCATAAAGAACAGATGAAGGCATAGGGTTGATCCGTCTCCTATCCCTCTGCC[A/G]CGTTCATGAGAGAAAGAACATTCCTTTGATTAGTTCACATTCCGAGGTGCACATTTTT	A	G	0.45	0.27	0.27	0.26	0.27	0.50	0.30	0.31	0.18	0.27	0.27	0.037	0.006	0.011	0.051	0.0005	1		
rs7814955	8	127.38	ACACACCTAATGTGTGTCAGTAACTGTGCTCTGAAAATGCAAAGGTAAGTAAGAC[A/G]TCCATCAAGGAGCTTGAAACCTGAGTGGAGAAGATCAAGAAACAGACTCAGTTTTA	A	G	0.90	0.60	0.64	0.63	0.61	0.41	0.40	0.67	0.64	0.67	0.66	0.056	0.026	0.001	0.024	0.0011			
rs974450	8	127.66	CCATGTTAAGAGGTTTGGGCTTATCCTAGAGACAAAAAGCAATCTCTGAAGAATTTT[A/C]AGCAGAAAATGATGTTGCCATCGGATTTGTTGTTTCAGAAACATCGCCCTGGTAAC	A	C	0.38	0.34	0.38	0.33	0.32	0.45	0.16	0.27	0.21	0.40	0.45	0.000	0.032	0.010	0.002	0.0021			
rs901592	8	127.95	TCTTTGAGTGACACATGGGTAACTGTTTAAACATGACTGTAATGTGAGAATATG[A/C]GTTAAATTAGTGTAAAAACTGTATGCCTCACAATTTCCAGATGTTAGTATTTCGTAATGT	A	C	0.75	0.45	0.42	0.39	0.52	0.50	0.47	0.40	0.36	0.35	0.32	0.054	0.045	0.004	0.001	0.0030	1		
rs283710	8	128.25	GAGTTCCTTGGATTGTGCATAAGGTTGATGGCTTGAAGAGGTGGCTAATTGCCT[A/G]AAGACAGGCTTGATATTTTTACCAACACAAAAAGAGATCAAGTGATCCAGGAAG	A	G	0.79	0.78	0.74	0.80	0.79	0.32	0.33	0.52	0.64	0.77	0.74	0.000	0.095	0.037	0.109	0.0009			
rs1562435	8	128.46	AGAAAGAGAGAAAGTGTACTACAATATAAATGAGTAATTGCCGCTAAAATTA[A/G]AAATTTTCCTCAGCCACAAAATGCTGCAAAGGCTTGGCCCCTGCACTTAACAATGA	A	G	0.64	0.65	0.66	0.50	0.73	0.95	0.70	0.66	0.75	0.65	0.58	0.002	0.000	0.001	0.058	0.0016	1		
rs4517084	8	128.62	CCCAGATTCAGAGCTGGCTGGGAAGACCCACTCATTGAGTTTGGAAGTACTGAT[A/T]AGCAGGTGGTTAGAGTGTGGCCTTTAGAGACAGGTGGACAGAGTTGTCTTTGGAAA	A	T	0.58	0.54	0.54	0.58	0.71	0.59	0.65	0.61	0.43	0.49	0.59	0.001	0.004	0.000	0.000	0.0031			
rs755520	8	128.93	TTGCCAGAGGCACATGGAGGGGTAGCTGCTTGGTTGCATTAAATTAAATTGC[A/G]TTACTTAGAGTCCCTATAAATGCTGGTGGATCAATGTAGCTGATGGTTTCATAACAA	A	G	0.67	0.54	0.63	0.65	0.54	0.59	0.41	0.52	0.50	0.52	0.62	0.001	0.032	0.006	0.001	0.0006			
rs766811	8	129.96	CAAATTACCACTACTTACGACCAGGCAGAATCCAACTCTTCCTTTCTTCCAGTATT[A/G]GAATACCATTTCACTTGGGATGAAGACATAGTGATTTGAAATGTGCAGGAGACCTACAT	A	G	0.48	0.70	0.68	0.70	0.64	0.55	0.77	0.53	0.71	0.67	0.67	0.024	0.001	0.014	0.015	0.0000	1		
rs384752	8	130.83	GAAATGGTGGCATTTTGGAAACTTTCTTCTCAGAAGCAGGAGTCATACAAATG[A/G]AACAATAGATTAAATTCAAAAATGTGGATTCTATTTGAGTCCATGTTTTTCTACGTATG	A	G	0.36	0.52	0.51	0.44	0.59	0.55	0.33	0.38	0.61	0.42	0.51	0.017	0.026	0.013	0.000	0.0003	1		
rs298609	8	130.84	CGACTTCAATTTTAGCAGTATATTGACTCTTTTTCTACCCTCCACCCTAT[A/G]GAAATTAGTGTGAAGTTAATAAAATTTTTAATTTACCTCAATAAGAACAATTGAAACATGC	A	G	0.38	0.52	0.51	0.45	0.57	0.55	0.33	0.39	0.64	0.41	0.51	0.013	0.026	0.012	0.000	0.0003			
rs3057	8	131.29	CACTAAACTGTGTGAAGGTGTATTGATAGTACCTCTTGATGGAGTACTAAA[A/G]TGATGATTGGCTTTCCATTTGGAGGAGAAAATCGTGTGACATAAAAACCTTCCTTCTG	A	G	0.67	0.42	0.54	0.51	0.52	0.55	0.58	0.72	0.54	0.71	0.62	0.010	0.005	0.022	0.001	0.0048			1
rs925688	8	132.69	ATACCACCTTTCCCAAGGGTGTCAACCTTAAAAATTCCCATACTTGTGTCCTTA[A/G]TAGTTTGAGAATAAGAAACCTCCAGAACCAAAAGGTAAGTCTCGAATGGTGAAGTTTG	A	G	0.74	0.51	0.54	0.52	0.59	0.73	0.57	0.71	0.79	0.61	0.46	0.030	0.003	0.026	0.025	0.0029	1		
rs1062064	8	132.97	AGAGTACTCCTGCCCTCCTGTCCCTCTTTTGTTACCTCCAGCCTGGCTACT[A/C]TAGATCCTCATTCCATCAACTGTGGATTTATACAAGGATCTCCTAACTGG	A	C	0.18	0.30	0.36	0.30	0.41	0.73	0.53	0.43	0.46	0.40	0.29	0.032	0.010	0.000	0.056	0.0019			
rs977939	8	133.10	GAACCTATCTCTGAGGAAGTGATTCCTCTCTCCAACATAGTGCCAGACTCTC[A/G]TATTAGGAAATGATGAAACCATCAACCAAATGTCTGCCATAGCTACTGTGGCTCTGAC	A	G	0.26	0.43	0.48	0.45	0.39	0.05	0.11	0.33	0.32	0.44	0.45	0.041	0.102	0.021	0.166	0.0019			
rs2138248	8	133.49	CAAAATGTTCTTATTCATGTTTTCACTAATATAATCCCATTTATATAAAATTACAC[A/C]TACGCAGGTGTGGCTAGTACATAGCTCTTTGATTAGTGGAAGATCAAAGGGA	A	C	0.83	0.64	0.65	0.65	0.55	0.86	0.38	0.60	0.57	0.69	0.73	0.029	0.026	0.000	0.054	0.0029	1		
rs760327	8	134.46	AGGCACTAAGGTGCAAAGAGGAAGATTTGCAAGCACATCGAAGGTTCTCCCAA[C/G]CTGCTGCTAGCTGCTGGGCTGTCGGGCCTGGGAAATGGGCAGGCGCCGTGAGAAGGA	C	G	0.45	0.42	0.44	0.34	0.50	0.32	0.53	0.30	0.39	0.35	0.39	0.001	0.013	0.007	0.005	0.0010	1		
rs1375432	8	134.62	GGGCAGCCCCTGCAGGTAAGCTGCCAGGATCACCCCATTATTGAGTAGGTAG[A/T]TGCTGGCATCAGAGGAGAGCCTGGACACGAATTACTGCTCCACTGTGTATGAGCTACAT	A	T	0.12	0.41	0.40	0.37	0.54	0.36	0.49	0.30	0.46	0.47	0.42	0.033	0.030	0.002	0.005	0.0008			
rs894358	8	135.57	ATCCTGTCTCTGGACACCATGAGGCACACCTTGATTCCTCCTGTGGGTGCT[C/G]TTGCAATGAGGTTTATTATGTCTAACTTCCCCTGAGGTGCTCTGACTCCTTTGACT	C	G	0.31	0.43	0.33	0.28	0.29	0.64	0.89	0.54	0.61	0.38	0.34	0.009	0.136	0.008	0.023	0.0006	1		
rs1563322	8	136.45	TCTAACCTCCACTTCTTACCTGTAAAATGAGAACAACAAAACTAAATTCACGTG[A/G]AAGTCATGGATTAAATGAAGAAATGTGTGAAGAACCTTACAGTACAGTGCCAGGCATACC	A	G	0.60	0.37	0.37	0.30	0.43	0.27	0.21	0.34	0.32	0.51	0.38	0.011	0.033	0.013	0.015	0.0004	1		
rs1470615	8	136.96	CCTATTAGAAAATTAGTAGTTGCAACCCCAAGGAAATGAAAAGTTTAACTAGTGTG[A/C]AGGCAGAGCTGGTGTTTAAATGGGGGAAAAGCATTCACACAGAACCCTAATTGG	A	C	0.14	0.28	0.29	0.30	0.30	0.14	0.11	0.26	0.18	0.40	0.29	0.016	0.029	0.002	0.017	0.0004	1		
rs2282	8	137.09	TTAGAAATGCAGGTGGCATCTCAGAAGGTCTCAGGCTTTAGAATAAGTTGTTACTTTGATTCTTTGCTCTGAC[A/G]CCAGTTAGCTGTGTGATTTTGCAGAAGGTTACATTTGT	A	G	0.67	0.43	0.43	0.50	0.46	0.73	0.84	0.72	0.50	0.54	0.53	0.020	0.077	0.034	0.031	0.0079	1	1	1
rs1399173	8	138.44	AATAAAGTAAGATGTCATGAGAAACTTGATTTAATAGAGGACACTGGATGTGA[A/G]AGGCAGAAAGTAATAAAGAGAAAAGGCTACATAGAGACCAGAGGGCCAATCCAAAA	A	G	0.36	0.60	0.59	0.46	0.59	0.45	0.76	0.38	0.57	0.54	0.45	0.010	0.049	0.002	0.000	0.0053	1		1
rs1398296	8	139.26	AGATCAGTCTGAGTCAGAGCCTGTGCAGCTTATAAGTGCTAAATTCTGCCTTCCA[A/G]TTGGAAGATTCTTCATAGAAGAGGTAGCATTTAAGAGGTCACTGAGAAACAGGCA	A	G	0.29	0.36	0.29	0.35	0.29	0.23	0.58	0.41	0.32	0.22	0.23	0.001	0.026	0.000	0.006	0.0036	1		
rs1398297	8	139.26	TTTTTAGTCCACATTATACTTGGATTCTTTGCAGTATTCTCCAATGTTGTTCC[A/G]CTCTCCCCCTTGAAATACCCTCCCTTGGCCGCCGTGAAATGAAGCTTCACTATTTTCC	A	G	0.36	0.15	0.12	0.12	0.20	0.05	0.01	0.07	0.17	0.11	0.10	0.023	0.070	0.024	0.026	0.0004			
rs741465	8	140.54	ATGACCACACCATGAGCTCTCTAGCCTCCACACCAGTGAGTGCCTGTTTTGGTCTAACCCATGGCTGC[A/G]GATATGCTAGGCACATCAGCTAGTCACTGTGGAATC	A	G	0.31	0.42	0.46	0.37	0.45	0.18	0.48	0.50	0.43	0.51	0.50	0.012	0.000	0.003	0.046	0.0000	1		
rs983740	8	140.66	CATGCCCAGAGCTCCAGGGGAAGGAGTGAGAAGCTGGGAGAGCAGGGCGGAGCC[A/C]GGTGGGTGAGGTCCACACATCTCCTTACTTTGAACTTGACCCGGCAGACTTCCT	A	C	0.50	0.61	0.56	0.59	0.61	0.20	0.78	0.66	0.61	0.58	0.46	0.000	0.057	0.032	0.036	0.0019			
rs1868280	8	141.87	GAAGATAATGTGGACAGGATTAGCTAATGTATTCGATATGGCTTGAGAGCAAAA[A/G]AGGAATAAAAGACGATTGCAAATGGATCTGAGTAACTAAAAGATGAGGTAACCCTTT	A	G	0.19	0.49	0.54	0.63	0.52	0.32	0.07	0.42	0.43	0.58	0.61	0.064	0.128	0.004	0.025	0.0056	1		
rs1375062	8	141.94	TTGCTATGCTTCCGATCTTTTAT[A/C]GTTCATTTCCCCCAAGTTTTTCTGTTTTCCTTGTTTGTTGGTTTCTATCTTCAGTGTTAGAGGCTTTTCTTATAGCTTCAAAAATTCAGTTA	A	C	0.19	0.49	0.54	0.63	0.52	0.32	0.07	0.41	0.43	0.60	0.62	0.064	0.128	0.005	0.025	0.0066		1	1
rs879429	8	143.01	TTCCAGCCCTTCATTCAGACTAGGGGCTTGTGAAAGGTAGGGAAT[C/G]GGGAGGGCCCTGATCTCTCTCCCATCCCTGGCCTGGGCTGGCCTCCTAGGTCTGGATGAA	C	G	0.62	0.60	0.65	0.61	0.50	0.45	0.26	0.57	0.68	0.66	0.61	0.000	0.073	0.006	0.017	0.0002	1		
rs4246828	8	144.27	CTGCGAGATGCACCTGGAGCCTTGAGCCATTGTAAAGCTGTTCCTGTTCTCTAAC[A/G]GCACCAATGCCATTAGACATCAGTCCTCCCTAATTTATGAAATGTTTCATTTAAT	A	G	0.45	0.71	0.71	0.72	0.75	1.00	0.95	0.79	0.79	0.79	0.77	0.040	0.055	0.000	0.112	0.0008	1		
rs7386971	8	144.42	AGAGGGGAGGGTACCCAGGTTTCCACACTAGTTGAAACCAGTCAGTACATACCT[A/G]TCTTCTGTTTCTGCAGGGTTTGAGCAAGGGCTGTGCCAAAACACACAATAAAAA	A	G	0.79	0.58	0.53	0.56	0.54	0.41	0.08	0.28	0.61	0.63	0.58	0.055	0.083	0.012	0.000	0.0022			
rs380904	8	144.67	ACGTGGTGGCCACGCAGGCCTCACGATGGCTATGGCCACCTCATCTCACAGAAAG[A/G]ACCCTCTTCTAACTTACTCCTCACAAGTCTGGCCCCTCATCCTCCAAACCCCTAT	A	G	0.38	0.33	0.29	0.34	0.20	0.18	0.18	0.26	0.11	0.23	0.26	0.002	0.023	0.011	0.018	0.0001			
rs3750203	8	144.84	ACTTTCCACCTCTTGGATATGGATTTTATCCTGCCCTGATTCAGGAGATCCCCATCT[A/G]TTAAGTAAGGGGAAAGATGATTTTGTTGTCTTTTGTTTCAGATTCTAATGTAGGAA	A	G	0.12	0.70	0.69	0.58	0.71	0.05	0.35	0.47	0.61	0.67	0.67	0.155	0.050	0.014	0.238	0.0001			
rs4874100	8	144.99	TCTATTTATAACCTGTAAGAATTGTATCTGAAGCGGGTGTTGAGTTTTCTGA[A/G]GTCCTCTTATCTGCTAAGTAGGGTTGGTTCAGCCCATGGAGGTTTCATCAGCGGGCTT	A	G	0.14	0.13	0.08	0.09	0.11	0.00	0.03	0.08	0.11	0.10	0.10	0.007	0.010	0.003	0.031	0.0003			
rs4557742	8	145.51	TACAGTTGACCTGACAGTAACATGCTGTTTCTCACATGTGCACACCATTCTTGGGG[A/C]AAATTCCAAGAGAATTCTAGGTCAAAGGATAAATGCTTTTTTTGTTTGAGACAG	A	C	0.43	0.52	0.56	0.49	0.45	0.72	0.02	0.40	0.43	0.47	0.56	0.010	0.211	0.015	0.012	0.0001	1		
rs748195	8	145.69	CATGACATCATTCCCCTGTGATGTCATACATGTGACGTCATTCCCGTGCTGTC[A/G]CCACATGATGTCATCCCCTGTGGTATCACCTCTGTGATGTCAATACCTCTTTTAATGTC	A	G	0.88	0.63	0.50	0.60	0.48	0.50	0.33	0.49	0.75	0.53	0.53	0.086	0.020	0.000	0.002	0.0001			
rs1735169	8	146.07	GCGGCCTGGATTGTCAGCCTCTTGAAAGTCAG[A/G]GAGAGAGTGCGGAAGGATGTCCCAGAGATGCGAGGAGTGTGGCAAAGCCATCAGAGCCA	A	G	0.67	0.32	0.35	0.43	0.46	0.68	0.41	0.50	0.57	0.40	0.40	0.061	0.006	0.017	0.080	0.0053	1		
rs1618523	8	146.07	CCCTGGGGCTGCAAGTTTCTGGAGGCCCCCAGGAGGCCTTCCAGACATCTGC[A/G]TTCCTTCTGGACTCTCCAAGTAAGCACAGGCTTCTGTCCTACTCTGTAACCCTAGAG	A	G	0.45	0.27	0.32	0.43	0.45	0.55	0.05	0.46	0.46	0.36	0.37	0.012	0.043	0.017	0.038	0.0039			
rs1735173	8	146.08	CACACCGGAACTTCTGTGCACGTGGAGTATGTGTGCAGTGTCCCTGCCTTG[C/G]TGTCACGTGGCTGCTGAGAAGGGTCTTGGCAGGTAATGCTTAGCCAGGTGGTCACCT	C	G	0.33	0.70	0.66	0.57	0.54	0.32	0.58	0.50	0.43	0.62	0.59	0.061	0.007	0.017	0.080	0.0064		1	1
rs572098	9	0.25	TTGTCTGCTTTCCTCATTGCAGTATCCCCAGAACTTTGAGCAAGTGCACACTCGAT[A/G]AATACTTGAAAGAATAAATCAATGTCAGACATCTGGGCTTATGTCTCATCTTTCTAC	A	G	0.34	0.48	0.49	0.50	0.50	0.64	0.78	0.51	0.54	0.51	0.44	0.020	0.035	0.002	0.003	0.0032	1		
rs1532309	9	0.59	GGGATGGGATGAGTAGGG[A/C]AATTTCTTACACAATGCCACGGTCCAGAGGAAAACATCCTTGAAAAAGATTCATCTTTCAAATCCCGATATAAATCTACTCTTAGATCTATA	A	C	0.90	0.50	0.53	0.50	0.48	0.91	0.97	0.47	0.71	0.52	0.50	0.077	0.134	0.001	0.078	0.0008			
rs1532310	9	0.59	AGGCACATTCCACGTGTGGCTGATGGGGTTATCATAGTTACAAATCATATTT[A/G]TCCACAGTGATAATTATCTTCCTTAGCGTTTTGTTCTTTCACTTCAGAGCTCATTTGTGGGCC	A	G	0.90	0.52	0.56	0.54	0.48	0.91	0.97	0.48	0.71	0.52	0.52	0.073	0.128	0.001	0.073	0.0009			
rs999924	9	1.32	AAACTTGAAACTGTCAAGGTTAGTTGGAATGTCCTACATTCAACATTCCTTTTC[A/G]TGACCACAGACTAAGCCAAGGCATTAAGAAAGACAGCCTGCCTTCTACAAGAGTG	A	G	0.30	0.30	0.27	0.30	0.36	0.50	0.77	0.40	0.46	0.24	0.25	0.002	0.036	0.000	0.002	0.0001	1		
rs6474795	9	1.39	AAGTTCTTTTTTATTCTCTAAATGTAAATATGACCTATTTCCACAGCACCTTTAGAA[A/T]GTACAATGTTTTCTGCCCCCTGTAGCAACACAAGCTTTTGAAATCTATTTATATAG	A	T	0.43	0.73	0.77	0.77	0.80	0.91	1.00	0.84	0.86	0.81	0.77	0.054	0.096	0.008	0.023	0.0014			
rs1412256	9	1.45	AGTTTTCCTTAAGAATCTGTAATGTACATTGAACCCTGAGTGCAGCATGCTGAAA[C/G]GGAGTCTTTTTGGAATGTAGTGAAGTCAGGGCTATTGAGACATGTGTTTACGTATAT	C	G	0.67	0.98	0.92	0.93	0.95	0.90	1.00	0.91	0.96	0.92	0.98	0.037	0.045	0.001	0.001	0.0130		1	1
rs1535842	9	2.20	AATATTATTTTAGAGACCCTTGAAAAAGTTTAGAGGGGCAGGGGAGATGAATAA[A/G]GGAAAGAAATTGAGATTCTATGTCCACACTAACATAGTCATTCATTGTACTAAGCAC	A	G	0.71	0.39	0.46	0.46	0.50	0.55	0.33	0.53	0.43	0.48	0.53	0.036	0.014	0.000	0.002	0.0041			1
rs1331829	9	2.37	GTTCAAAGAACTTTTTGGTAGGGCTGTAGTGTTCAGTTGTGCAGACTGGGC[A/C]CTGCACAAAGGTGCAATGTGACTTCAGTGTTTTGCCTACCCACAGTTCTTTCTTTCTTC	A	C	0.76	0.45	0.45	0.45	0.38	0.68	0.49	0.50	0.29	0.45	0.46	0.065	0.002	0.002	0.031	0.0004			
rs729367	9	2.59	AGTCCTTCTAGGAGTACTACCAGAGGCTCCAAAGGAGAAGAAGTATAGTC[A/G]ATGGGTAAATTCCACCCATGTTCCCAGAATTCCAGGGACCTGGTCAACACCAGAA	A	G	0.52	0.30	0.24	0.23	0.23	0.50	0.53	0.39	0.39	0.17	0.26	0.037	0.052	0.006	0.034	0.0004			
rs1455175	9	3.79	TTAACAGACCCGATGTAAACATGCAAATGTGCCTGCATCTCTAGTACCATCCTA[C/G]TGCTACCCAAAGATGTTTAATGATTTTCCAGAGTTTTGAGAGCCCATTGGGTATG	C	G	0.50	0.77	0.73	0.80	0.84	0.77	0.75	0.86	0.75	0.80	0.77	0.031	0.000	0.018	0.002	0.0006	1		
rs4131424	9	4.33	ATCTCACCTGTAGTACCCTAAGTAGCCCGGGGTTAAACAAGGGCACTTTCAACAC[A/G]CTTAGGGTTTTAGTTTAAACCTGAAACATCAGGATCTATTTAGACCTCTCTTAGA	A	G	0.21	0.15	0.13	0.15	0.18	0.09	0.02	0.13	0.07	0.17	0.22	0.003	0.020	0.000	0.000	0.0074	1	1	1
rs7851353	9	4.40	TAGTCCACACGACCTCCCAACTGTAAAAAGAAATACAACCTCAGGCACAAAATATT[A/G]CCGGCAGTACTGGGTTATGAGGTCGTGATACAGATGACCCATAATGTTCTTGTAC	A	G	0.33	0.54	0.48	0.49	0.52	0.36	0.16	0.40	0.64	0.53	0.44	0.008	0.050	0.005	0.004	0.0010			
rs913258	9	4.87	GCTGGGATCAGAGGGAGAGAATAAATGACCCTGATACTCTTACTTAGGAAGGCAT[C/G]CTCCCCCCTCCCTCCCCTCCCTCCCCTCCGAGAATCATGGCTTTAAAGACACAGAGATGAC	C	G	0.24	0.81	0.83	0.79	0.89	0.18	0.65	0.67	0.79	0.87	0.87	0.211	0.042	0.020	0.255	0.0002	1		
rs958480	9	5.72	GGGTACACGAAAGGCTTGGGAATTCAGGGGTCTTTGAAAGGCTAAATTAAATGT[A/T]GTAGAAGTGGTTCAAAACCTACTTAAAGAAGCAACCAGCTCCTCCAGGACACTTCT	A	T	0.79	0.51	0.57	0.61	0.54	0.18	0.47	0.45	0.57	0.54	0.50	0.029	0.004	0.013	0.080	0.0010	1		
rs1028702	9	5.78	TTAACAATGCTCTGACAGTGTACATCTTTCTGATCCTAAAGGGCAGCTATTGAAGT[A/G]GATTGGTTTGGCTTTCCTTAAGGTCATAAATGCCGTGTGTAGTGTGGCAG	A	G	0.14	0.36	0.31	0.22	0.32	0.64	0.43	0.29	0.32	0.23	0.35	0.018	0.012	0.000	0.058	0.0011			
rs695013	9	6.08	AAAAGCAAACAAAAAATATTTATGATATATCTGAAAAATTTTGAGCACTGCAATTG[A/G]AAATTGATAATATTCAGGAAATGGTACTCATTTTTAGATGTGACATATTTCAGTGT	A	G	0.69	0.79	0.81	0.87	0.80	1.00	0.98	0.74	0.93	0.79	0.81	0.001	0.084	0.000	0.106	0.0001			
rs1407862	9	7.02	CAGGCCTTGCCTCCTCTGCTCAGCTTTTGTTTTTGGAGGGGAAAGAAGGCCAA[A/G]CCACTGACCTAATCCTGAACAATGTATTTGACTAGTTTTTGTTGTTTTTACATTGAAAA	A	G	0.45	0.53	0.49	0.40	0.50	0.32	0.68	0.54	0.43	0.38	0.31	0.005	0.008	0.000	0.034	0.0313	1	1	1
rs1156793	9	8.37	TAACAGTCTCAGCTGGAGAGCAACCCTTTCGGTTTAAAATAAACTAATGAAATCCCT[A/G]AGGACAAATATCACTATGATATGCACAAAAACAGCACATTAATGCAACAAAATGA	A	G	0.31	0.75	0.83	0.79	0.77	0.45	0.31	0.43	0.82	0.83	0.83	0.153	0.172	0.092	0.090	0.0000	1		
rs765800	9	8.78	GAGATTTTAAGTTCACTGACTTGCCTCAGAGGAACTTAACAAGAAAGAGCAAACC[A/G]AGGAGTAAGTTATTTTAGATAATTGCAGCAACAATGCAATGACTCAGGATTTAGGGA	A	G	0.38	0.40	0.42	0.45	0.32	0.50	0.71	0.45	0.43	0.44	0.37	0.000	0.039	0.000	0.003	0.0010			
rs768224	9	9.76	TACATTAAATTGGTGACAAAAATAATACTGTGTTCAAGGATTAGTTTTATTTC[A/G]CACTGAACATATTGCATCTTTATATAACTAACCATATACAATGTATGTTCTAGTTTGC	A	G	0.45	0.41	0.34	0.41	0.30	0.59	0.61	0.52	0.57	0.34	0.34	0.005	0.032	0.007	0.026	0.0001	1		
rs1322304	9	10.51	AGGCAGTGTGTATTCCTGCATGGCCTGATATATTAGGTGTCTATGAAAGC[A/T]TGCTCCCAAAGCAACACTCTCCTACAGCTTCCTTTGAGGCCTCTACTGTTTCAGTCT	A	T	0.83	0.51	0.48	0.51	0.59	0.86	0.90	0.63	0.46	0.50	0.48	0.058	0.105	0.001	0.075	0.0002	1		
rs1334022	9	11.01	TGATTATTTAAAATAATGCTGTTATAGAAACATTAAGATTAGACAAAACAGAT[A/G]AAATAAATTAATATTTAATGAGGAGGCAGAAGGATATTCACTAGGAGCCAACATTTGTG	A	G	0.55	0.71	0.62	0.63	0.59	1.00	0.98	0.89	0.82	0.66	0.65	0.004	0.110	0.043	0.134	0.0017	1		
rs901986	9	11.19	TGTAGCCTTTAGAAGTGAATATAACTTGAATTTATTTTATTGTGAACTTAGA[A/G]GGAAATGTTTAAGCTGTTTTTCAAACGTGAGTTCAAATCCATATCAAGCTTTACTATTG	A	G	0.21	0.24	0.24	0.24	0.21	0.00	0.02	0.11	0.43	0.24	0.29	0.000	0.052	0.020	0.085	0.0031			
rs1854908	9	13.13	TAAATTACTGTTTTTTAATTTAAAAAATACAATTGCTCTGAAGCACTTTAATATC[A/C]AGTCAAAATATGGAGAATTCCTTACTAGGATGCAAAATAAAAGAATAATGAAGGTTG	A	C	0.81	0.28	0.32	0.40	0.25	0.50	0.39	0.47	0.14	0.38	0.44	0.123	0.002	0.016	0.016	0.0061	1		1
rs1556031	9	14.43	TGTTCCACGTGAGAGAGCCTGGCTTTCTCGCCTCTCCTCAGGCTTCCTGG[A/C]GCTGCTCTTCTGATGGAGTTGTTCCTCTCCCTGGTTGTTTATAATGGATTTCTAACAGAGGA	A	C	0.74	0.46	0.44	0.43	0.41	0.45	0.20	0.50	0.46	0.46	0.49	0.047	0.041	0.002	0.000	0.0032	1		
rs1143025	9	14.60	TGCAGTCAATTCTGAATGGAAAAAGAAATCAAGTCTGAAATTTATGTGAC[A/C]TAGTGGCTTCAGATCATTTGAACGATATGTAGAAATCAGATGAATAT	A	C	0.74	0.62	0.57	0.60	0.57	0.64	0.85	0.68	0.54	0.63	0.56	0.016	0.046	0.004	0.003	0.0001			
rs1328273	9	16.01	CATGTTAGTAGAGCAAAATGATTAACATCTCAAGAAATAATGAACATTGCC[A/G]CACTGACTTAAGAATCACCACCCTGATTTATTCCTATAGCTTGAGGCAGCACCACCTTCTC	A	G	0.07	0.39	0.36	0.45	0.34	0.45	0.82	0.36	0.54	0.43	0.34	0.077	0.118	0.000	0.003	0.0008	1		
rs2151065	9	16.24	AACCTCCTTTAAGCACTTTCAAGTAACTTGCCTTGACCAGAGAGCTAAGAAGTA[A/G]GCATTTCACTACCTTTTCAGCAAATGGTGATTTCTTTGTCAGCCCCATAAAGTGAAA	A	G	0.40	0.45	0.53	0.51	0.52	1.00	0.81	0.49	0.71	0.52	0.53	0.023	0.035	0.009	0.170	0.0001			
rs1888952	9	16.25	CTGTGACCCTCAGCGGTTAGCCTGAAATCACAGGCAGGTCTGTAGCAGAGTCGTG[A/G]CTTGAACCCAGGTCTTCTGTCTATGATCTGGGAAGGAGAGTGAACTTCTGCCCAA	A	G	0.76	0.46	0.40	0.41	0.48	0.00	0.21	0.45	0.18	0.43	0.39	0.089	0.024	0.000	0.164	0.0001			
rs1029015	9	16.65	TTAAAGGTGGAGCAAAAGTGGTCAGTGGCATCAAATGTCACAGAGAAATAAGTAAAAAAA[A/C]CAGAAACGCTTCTACAATATGCTTCAGATGAGAGGTCAGCGCTCCAGAGC	A	C	0.74	0.44	0.45	0.45	0.34	0.18	0.28	0.57	0.43	0.39	0.39	0.047	0.029	0.014	0.043	0.0018	1		
rs263645	9	17.01	CTCTGGGAGATCAATGATCACCTTCTGAACGTCCTTCCTCCTTGCTCTCATT[A/T]TCCATCATTCGGCTTAGAGACTTGGGGAATGGAAACCATGAGTATTATTTTAAAAATC	A	T	0.24	0.53	0.48	0.60	0.61	0.55	0.31	0.36	0.50	0.55	0.56	0.049	0.014	0.017	0.001	0.0040			1[g]
rs960232	9	18.16	GTGAAACGTGTCATAAATGTCTGTTCTGGAGTACTAAGGTTTATAAAATC[A/G]ATATGCTGCTCAACCTTTTCATCTCACTGACTGA	A	G	0.67	0.61	0.62	0.48	0.54	0.68	0.74	0.68	0.68	0.67	0.62	0.002	0.078	0.013	0.001	0.0016	1		
rs2076902	9	18.88	AGGGTGACTATGATTCATTCAATTAAGTAAAATTACTGGATAACAAAAGTACCT[A/G]TTTCTTCTGTTTATGTCCTATACTAACTTTAAAAAAAGATACAATTAATCTTCATTCAT	A	G	0.52	0.55	0.51	0.54	0.59	0.41	0.30	0.52	0.61	0.60	0.58	0.000	0.020	0.000	0.005	0.0002	1		
rs748530	9	19.63	TCTTAGCCAAGCTTTTATACTAGAGGAGATGTGTCTGTCTCAGTTTCCTCATGTGA[A/G]CTTGAGAATTGCTAAGGCAAAATGCTGTCATCATTACAGATGGAAATAGGTTATCC	A	G	0.29	0.21	0.30	0.26	0.20	0.09	0.03	0.27	0.32	0.30	0.19	0.000	0.069	0.002	0.036	0.0059	1		1
rs717081	9	20.28	AGTTGAATAAATGTCATTTTTCTCCTAGTGAAAGCATGGTGGAGGATTTGTGCTTATTT[A/G]TACAGGTTTGGTTGTCTTAAAACAATGTTTCCCGGAATGCAGCTCATGCATCA	A	G	0.52	0.58	0.59	0.57	0.59	0.45	0.22	0.40	0.50	0.58	0.55	0.021	0.153	0.064	0.046	0.0021	1		
rs1332184	9	21.49	TAACCAGAATAGCTTATATAATAAAAGCACTGCTGGAAAGCCTGAGCAGAGAAAT[A/G]TGTCCTCATTTATTGAAAGCAACTGCAGCATTGTGTGTGTGTGTGCGTGTGTGTCT	A	G	0.50	0.40	0.36	0.39	0.45	0.36	0.61	0.44	0.36	0.34	0.34	0.005	0.031	0.024	0.031	0.0005	1		
rs720974	9	21.51	CAAAACATTTATATTATCAGGCTATGGTTGCCTGTGACCCACTAAGACCAAAGT[A/C]AGACATAATGCACACATTTCAGGAGAATTTCCCAACTACCCAAAAGTCAAGGC	A	C	0.33	0.53	0.60	0.48	0.55	0.68	0.49	0.54	0.57	0.58	0.59	0.029	0.008	0.001	0.005	0.0001			
rs1414237	9	21.72	CTATAACGGAAGTACCAAGTCCACATTGCAGAAATCTCTGTTCCTAAATCAATG[A/G]CTAATAACAGACTGGACTTCTCATTCAGGGAAATAAAATGA	A	G	0.93	0.57	0.48	0.54	0.61	0.73	0.79	0.32	0.54	0.40	0.52	0.123	0.042	0.014	0.028	0.0004			
rs1360590	9	22.03	CTAGACAAAGTTTAATGTTCCCTTTTATATGTTTTCCTGGTAAACAAAAATTGTCTCAGGGTTATTATGCATATATGATATTGTCAAGAAACTTTCTGGGT[A/G]CTGTGGGGCAAA	A	G	0.29	0.64	0.49	0.55	0.54	0.23	0.08	0.41	0.46	0.44	0.49	0.007	0.043	0.002	0.008	0.0003	1		
rs1333050	9	22.12	GAATTTTCTTTGGCTATAGTATGAAAGAGAACTATAAACAATTTGCTCTAGATTTTTC[A/G]TTTAACAATATGTCTGGCAGATCTTGGAATATCTGCTTATATCAACTATTTATTT	A	G	0.24	0.61	0.67	0.52	0.57	0.59	0.24	0.46	0.50	0.77	0.69	0.107	0.103	0.018	0.005	0.0000			
rs1327063	9	22.35	AGCAACTAATGTGAGTAGGCTTTTGTATGTCTGTAGCT[C/G]CTGGAAGTCCTTTAATCATCAGGAAATGCCCTACGTATCCATCAATATTCCTTGAAAAGTTGATTTTCAAATC	C	G	0.67	0.52	0.50	0.54	0.52	0.05	0.55	0.30	0.61	0.43	0.58	0.010	0.001	0.028	0.153	0.0019			
rs702223	9	23.80	TGTAAGGAGCAGTTTAACATGATTCAAACATTATCTGTAAATACAAGAGATCAAA[A/C]TGAAATATAAAACCTTTAGAACTACTGCCTTTGTCTTTACACTTAACTTTTTAATTCACGT	A	C	0.29	0.36	0.35	0.34	0.45	0.45	0.79	0.46	0.39	0.28	0.33	0.003	0.096	0.007	0.004	0.0022	1		
rs1332677	9	24.66	TTGCTTTCAATACAACCCAGGTTTACTTCTGCCTTCTGCTATGTTTCATTGCGG[A/C]GGTTGTGAGTTCTTCCTTTATAGGCATCCAGGGATAAAGTGATAGAGCAGGCA	A	C	0.62	0.56	0.49	0.59	0.50	0.36	0.27	0.28	0.71	0.44	0.47	0.003	0.048	0.042	0.018	0.0010	1		
rs2169325	9	24.90	AAGAAGTCATATTTTCCACCCAGGATGTGGTCTATGAGCCAAGATCAAAGCTTT[A/G]GGTAGTGGGTTTCAGCTGCCCTGTCCTTGTTATTTTGTGCATTGCACAC	A	G	0.14	0.29	0.30	0.34	0.29	0.59	0.30	0.36	0.29	0.36	0.28	0.003	0.005	0.014	0.089	0.0011			
rs1128957	9	25.67	TTTTTTTCCTGTATTTTGGTTCTGAGGGATGAGTAATGTGTTTTTAGTT[C/G]TCGTTGAGTTTATCCATTCTCAATCCCCTAAAGCCTTCAGCTATTCTTCA	C	G	0.45	0.60	0.59	0.59	0.52	0.85	0.71	0.66	0.57	0.43	0.51	0.011	0.005	0.005	0.044	0.0018	1		
rs757099	9	25.73	TAGTGTTATTTCAAAGCAGAAAATGACCTAGTCTGCTGAAGAAGTTTTAAAGTAGT[A/G]CTTCCATTACTACAATTCACAAGAAGATGTTAATTCCCATAGGGGGTACATGTCTAC	A	G	0.76	0.63	0.53	0.54	0.63	0.64	0.66	0.48	0.61	0.52	0.43	0.025	0.008	0.000	0.010	0.0041			1
rs1220087	9	25.78	ACCAAGGTGAGGTTATCAGTGGTATCATGTCTTCCTGAGGAACATCATAAGCACAA[A/G]TTTGAAATGGACTGATGTTTCTGTTGTATTGACTTCAAGTATTGGTTGAAAATAAG	A	G	0.18	0.48	0.55	0.59	0.46	0.35	0.49	0.45	0.39	0.43	0.56	0.095	0.015	0.005	0.021	0.0001			
rs666478	9	27.16	GTGCAGTTCACAAGCTTTGGGAGGAAAAGAAAAGATGATTCAGAGTCAAAT[A/G]CAAACCCCTTGAAATACATAATTCTCTGGTTGAAGTCTAGTGAATATGGGA	A	G	0.43	0.52	0.49	0.59	0.63	0.20	0.52	0.44	0.43	0.52	0.56	0.003	0.001	0.001	0.051	0.0024	1		
rs1555454	9	27.22	CATCTCTAGTTGAATTACCACTTGGTTTTGATGACTATTTCCTTAAACTGT[A/G]TTTACTTTTATTTTGGCTCCCAGATCGGTACAGCCAACTTACTTCTAGAAATTTTATGGAG	A	G	0.74	0.74	0.86	0.83	0.80	0.68	0.80	0.90	0.82	0.77	0.78	0.023	0.013	0.003	0.043	0.0070	1	1	1
rs9103	9	27.54	CAGTACAACAGTCATCTGATTCAGTGGTTCGTCTAAAACATCAGCTTCC[A/G]CATCTTTCTGATTCATGATGGGAAAGCTTTCGGAA	A	G	0.83	0.79	0.76	0.73	0.84	0.77	0.97	0.86	0.71	0.83	0.78	0.003	0.042	0.007	0.000	0.0004			
rs994367	9	28.08	CTTAGGGTGAGATTCTTTCATTCTTCATTCATTCTTGGTCCTATCTGGGGAA[A/T]CTTTTCCTTGGAATGATCATGAAAATAGATCCCTATGTGTGGTGTTCAGCACCCAGATACAG	A	T	0.45	0.45	0.47	0.48	0.54	0.68	0.40	0.53	0.50	0.42	0.49	0.000	0.004	0.000	0.017	0.0006	1		
rs560764	9	29.58	AATGACCTTTAGATAGTCCTGTTACAGTGCTTCCCAGATTATTCTCCAGTGTATGATTTTTAGGGTGTA[A/C]TTGTCACATCACTGAGAAGTTTCAGTAGTAAGAAATATGTTTTTCA	A	C	0.12	0.49	0.49	0.44	0.45	0.50	0.61	0.39	0.61	0.45	0.54	0.074	0.018	0.000	0.005	0.0013	1		
rs1018254	9	30.13	ACCAGAGCTCTACATGTGCTACGCTTTTAGTCAGCAACCTGATGTCACCAAAAAC[A/G]CATGCACCTGTGCATCTAAAAATTATATATATATATATATTTTTAATAATTTTGGTC	A	G	0.15	0.35	0.33	0.37	0.18	0.09	0.10	0.20	0.29	0.29	0.32	0.018	0.038	0.009	0.047	0.0000	1		
rs1475656	9	30.90	TTCTATTTTTTTTTCATCTTTAAAAAGTTCCAAGAAGGGTTTTGGATA[A/T]GGTCCTCAATGAGCTTTTAGTCAAAAATTCTCTGCTAAAAAGTGTGCGGAATATCAGACCA	A	T	0.29	0.32	0.38	0.48	0.43	0.55	0.48	0.43	0.29	0.46	0.41	0.005	0.001	0.001	0.011	0.0000	1		
rs1412340	9	31.61	CTTTTCTGTTTCCTAGTGCATATATGATTCTTCAGAAGCTTTAGCAAGTCTTC[A/G]AAAGCTTGAGACACCACAAAAATGGTTTTAAAAGCCCAGTAATAATTCAGAATATTGTTGGCAGA	A	G	0.43	0.30	0.27	0.28	0.34	0.05	0.45	0.14	0.36	0.28	0.34	0.010	0.017	0.023	0.049	0.0007	1		
rs288886	9	31.73	TTTTTCCTGATATCCTCATGTCTAAATGATGGTGAGATCCCATTCTGTAGGTATGATAA[A/G]CCACTATCTGTAGGTTAACATTCATGGAATGTAGGATCAAGCTTTACCCTTTCCAGA	A	G	0.55	0.34	0.36	0.52	0.41	0.32	0.39	0.21	0.43	0.36	0.43	0.022	0.002	0.009	0.000	0.0028			
rs671018	9	32.55	AGAAACTGGAAGCTGTTCAGTGCACTGGCAGCTGAACCCTGCTCCTGCTCC[A/G]TTTATCAGAGTTACTATCATCTCAGATAATATGGAGCTCATGTCAGCAGTGCAGGTGGT	A	G	0.60	0.40	0.40	0.37	0.32	0.80	0.73	0.43	0.46	0.33	0.36	0.023	0.063	0.003	0.102	0.0002	1		
rs855543	9	33.63	ATGGAGGCTTTCCTATGCATTTGCTGCTAAGCTTTTTGACCAAAGAGTTAAGG[A/G]GACTTCATACAATATTTGGAGGTCTTATTCTGCATAGCTTCCTCTGCAGTAGCTTAT	A	G	0.26	0.47	0.46	0.48	0.48	0.65	0.76	0.54	0.57	0.46	0.47	0.018	0.054	0.001	0.016	0.0000	1		
rs471371	9	33.86	GGATTGAGGAGAAAGAAAAATTCAAGGACAACTCTCAGTTTTCTATATTGAGG[A/G]AATAGTGAACAATGACAGATCCAAGTAGTGATAAATAATACTCATTAAGTGCTTACT	A	G	0.43	0.36	0.40	0.39	0.43	0.09	0.26	0.23	0.29	0.38	0.43	0.001	0.013	0.011	0.064	0.0001			
rs2781	9	33.91	CATCTGTCATGGTCTCCTTCCACCAAATCTT[C/G]ATCTGGGAATAGCAGGGTATCCCTCCACCCAAATGTGCCACCTGTTTGTCTTCATTCCTGGTCTG	C	G	0.52	0.60	0.64	0.67	0.64	0.77	0.69	0.50	0.39	0.65	0.70	0.013	0.000	0.033	0.005	0.0001			
rs885451	9	34.99	TGGTATTAGCAAGCTGACGGCAGGAGACATTGCTAGTGTACTACAGTAGCAT[C/G]GAATCTCTACATATCTTTCAAGTAGCACTCAGGTCACTCTCACAGCACTGCAGTGGA	C	G	0.12	0.53	0.52	0.46	0.48	0.00	0.41	0.27	0.39	0.49	0.51	0.090	0.008	0.025	0.216	0.0001	1		
rs944700	9	35.10	CCCCTCCATCCAACCCCAAGTACAGGGGTAAGCACACAGCAGGGCTCAAAAAAGTAGAATGCAAGTCCCAGACATCTG[A/G]CAGCAGTCCTCGAGGTGGAGAGAAAAT	A	G	0.71	0.46	0.45	0.52	0.45	0.41	0.45	0.58	0.54	0.51	0.48	0.029	0.001	0.001	0.005	0.0000			
rs1359885	9	36.12	GCCCCAAGATTGCTGGACTGCTGGAAGGACTGGCTCATCCCTGGGAATGAC[C/G]CCTCTGCCTTCACTTTCTCTCAGCTTGCAAAGTGCACAACTCAAGCTCTTCTGCAGCT	C	G	0.83	0.58	0.59	0.60	0.57	0.77	0.50	0.68	0.71	0.67	0.68	0.037	0.006	0.001	0.017	0.0054	1		1
rs954779	9	36.41	AGAACTCTGAAAAAATATATAATTGAGGCTACAAGTTAATTCACCTCAACTGAG[A/G]GAATAGTCTTATCAAATTCTTCAATATTTTATCTCATTCTCAGGTCAGACTAGTTCCC	A	G	0.33	0.21	0.21	0.22	0.16	0.32	0.28	0.27	0.18	0.26	0.21	0.030	0.019	0.008	0.020	0.0002			
rs735741	9	37.93	CTAGAGTAAATGGGTCTCAAACACTTCTAGGTCTGACGTCAGAGACCTTTC[A/G]ACTGATGTAAGGAATGTCGGGGAGGAGATGGGAGGACATGGCTGGGAGTCCCATGG	A	G	0.38	0.52	0.40	0.53	0.50	0.05	0.30	0.34	0.46	0.51	0.51	0.000	0.001	0.001	0.081	0.0052	1		1
rs1138374	9	37.96	ATCCAGTTATATGACACCCCTTACGAACCTGAAGGCCAAAGTGTTGACTC[A/G]GACTCGGAGACACAGTCAGCCCCCACTGCGGGAGAGCAAGCTGCCCCA	A	G	0.24	0.53	0.41	0.55	0.54	0.10	0.51	0.48	0.50	0.53	0.49	0.012	0.013	0.018	0.051	0.0037			
rs716933	9	38.32	CACAGAACAAATGCTGTACCTGGTATTCAAGGCCTTCATTATCTGACTGCAAACC[A/T]GCTTTCCCTCCTCCCGCACCTCAGCCCCCACCACCTGTTCTCTTACCATGGAATG	A	T	0.40	0.48	0.42	0.50	0.38	0.41	0.72	0.34	0.50	0.50	0.39	0.002	0.048	0.005	0.001	0.0000			
rs1004604	9	38.35	GAAAACAATGGAAATGTCAACTTCATTGTCCCTATAGAACTGTTTTATTTGTTA[A/G]ATGTTAGTGAAAGGCATTTCTAATAATGTACTGAGTCTTTTAAGAGAAAAATAGTAGCC	A	G	0.57	0.71	0.69	0.70	0.54	0.90	0.74	0.59	0.68	0.79	0.66	0.009	0.000	0.009	0.029	0.0002			
rs987187	9	38.37	GGTGAGTGAGTAATAAATGCAACTTAAGAGGCAGACAAAATATATTCCAGCAACA[A/G]GCCCAAGGGGGTGGGTAAGGAAAAGGTCTCTGAGGGTGAAAAGATAAGGACTTC	A	G	0.60	0.26	0.23	0.24	0.18	0.18	0.03	0.25	0.21	0.23	0.18	0.080	0.042	0.000	0.001	0.0034			
rs923784	9	66.57	GGACAAGGGAAGAAGGTCAACAAGAAGCTAATAATAGCAGCCCTGCTAATAAATTGGATGGAAAGAAAACAATTGT[A/G]GCTTGTTTCTGGTTACCTGTGATTTTAAAC	A	G	0.93	0.40	0.49	0.52	0.38	0.41	0.35	0.58	0.39	0.54	0.59	0.118	0.016	0.018	0.004	0.0039	1		1
rs2026349	9	66.65	CACACTGCAGCATCTCAGCCTTCCAGAGTGCCTTTATTCATTCCACCCTGGG[A/C]CCATAGTTGAGAATCAGAATGTACTACTTTTTGTTTCCTTGTTTGTTTTTAAGACTTCA	A	C	0.67	0.40	0.41	0.37	0.43	0.18	0.24	0.53	0.50	0.46	0.44	0.048	0.006	0.040	0.016	0.0017			
rs1414944	9	66.91	CAGAAGAAGGAAATGATATATGGAAAATCTTAC[A/G]TGGGGTATAGAGTCAAGTGATCCCTAGGTTACAGTTGAGAGCTATAAATAGGCCAGTGACTTCATGTGTACCATGGC	A	G	0.40	0.28	0.31	0.34	0.36	0.68	0.48	0.28	0.19	0.29	0.35	0.002	0.014	0.003	0.065	0.0001			
rs1889915	9	68.62	AAGAAGACAGTGATGCAAGTTGCCTAATTGGATTTGGATTCATTTGAGACTTA[A/G]CAAGGAATCTAGGCCCCTTTTCCCACCTAGATTTATACTGAGAATGTCATCTAGGCCCA	A	G	0.60	0.44	0.45	0.50	0.48	0.00	0.32	0.63	0.50	0.48	0.51	0.008	0.017	0.008	0.202	0.0013	1		
rs1074670	9	69.47	TTTCTAAGCCTATGTCAAAATAAAGCTATGCTCAGATTTGACTACGGACTACTCA[A/G]TAATTTATCCAGCTGAGCAGCCTTCAAGCAGAGACAAAAATTGAAATTCTAAAAGTTC	A	G	0.62	0.52	0.52	0.60	0.61	0.86	0.57	0.40	0.57	0.53	0.52	0.008	0.008	0.001	0.090	0.0002	1		
rs1404195	9	69.84	GGGATTCTTATCATCAGCCAAGATTTGGAAACAGTAATTTCTTATACAAAGTCCT[A/G]AACTAATACCCTTGAAATAGGGGATTCCCCTTCACTGCCGGGTGTGTCGCGGTTAC	A	G	0.07	0.57	0.56	0.63	0.57	0.55	0.47	0.48	0.46	0.55	0.51	0.147	0.006	0.005	0.001	0.0024			
rs1570637	9	70.06	TATAGGCCATAAGCAGGTCATGAAACTGACTCAAACAAGATTGTGCATCAGA[A/G]TCCCCTGGAAAGCTTGTGAAAACACAGATTCCTGCCCCTACCCCATAACTCTGATTCC	A	G	0.81	0.35	0.29	0.28	0.29	0.73	0.68	0.54	0.46	0.30	0.31	0.144	0.074	0.033	0.089	0.0000	1		
rs2031197	9	72.63	TTGGGATCTTATGATTTCCTACAAAAACCAGAGTAGGCTGAGCTATGGAGCCA[A/G]TTTAGTTTCTTGACCCAGGCGTAAACAATATGCAACCATCTAGAACTGACATGAAAT	A	G	0.74	0.54	0.54	0.52	0.54	0.23	0.66	0.59	0.61	0.56	0.53	0.014	0.002	0.002	0.069	0.0000	1		
rs1333342	9	72.96	GTTGGGGAAGGAAGCGGACTGCGGCGGGGCGGGTGTTTACCGTAACCGGCGCT[A/G]TCATCGCTCCGGGACTCCTGCACGGGGAACACTGGGCGCACGGGGACGCGCAG	A	G	0.12	0.42	0.52	0.50	0.50	0.45	0.63	0.47	0.32	0.37	0.40	0.078	0.004	0.002	0.003	0.0073		1	1
rs2026406	9	73.53	TCCTTGGATGTCAGCAGCTCATTTAATATGCATAGCACTTGACAATTTACAAAA[C/G]TTATCACGTCTTTCTCATTTGGCTTCCCCACAATTAGTTCCAGAATCAGTAGAAGA	C	G	0.43	0.54	0.60	0.59	0.54	0.59	0.53	0.30	0.61	0.47	0.57	0.013	0.001	0.021	0.000	0.0014	1		
rs927632	9	74.62	CTTACTCTATTCTCTGCAACAGGAAGAGAAGGATGACTAAAAATCACTGGAGG[A/C]TCTAATCAATGGAGAAGGCACTTAGACTTACATCTGCAAAACACCTTGCTCTTGTTTAC	A	C	0.26	0.37	0.36	0.39	0.36	0.14	0.06	0.26	0.46	0.40	0.40	0.004	0.078	0.008	0.046	0.0002	1		
rs1891835	9	74.88	GAGTTATTTTGCTGATTTGGGGACCTGACCTTGGTTTAAATTAGTTTGTCTGTAAC[A/G]TCCCTGAGAATCATACACTGATTATCTGATATCAACCTGTC	A	G	0.90	0.63	0.63	0.66	0.70	0.65	0.54	0.72	0.71	0.69	0.68	0.043	0.028	0.001	0.006	0.0005			
rs2839899	9	75.81	TTAGGCAATTTGCTCCTCCAAATCTCTGACCAAACTAAACCCAGTGAAATATTTCC[A/G]TTCCTGGAACTGCACCTAGTTAATTTATCCAGTTAATCCAGGCACATCTG	A	G	0.21	0.75	0.69	0.72	0.66	0.55	0.35	0.66	0.71	0.70	0.72	0.119	0.042	0.000	0.009	0.0017	1		
rs1887773	9	75.89	GAGAGGAGTTAAGAGAGAGCCAGAGAGATTCAAAACTGAGGGTGGAGGGTAAATAA[A/G]CAGACAGGAGAGACATAATGAATGGGAACAGGAATGATGGATGAGACAGCA	A	G	0.10	0.12	0.09	0.09	0.11	0.23	0.15	0.21	0.18	0.14	0.14	0.000	0.007	0.029	0.019	0.0047			1
rs722642	9	76.75	AGCTTAGGTCAGATGTCTGAACAAATCAATGTGTGCAGCATAAACATACCA[A/G]TGGGCCAGTCTGAATACACACCTACCCTTGGAATGCAAGTGGTGTCAACTACA	A	G	0.12	0.33	0.33	0.41	0.34	0.41	0.06	0.49	0.33	0.34	0.30	0.028	0.056	0.027	0.003	0.0006	1		
rs713478	9	76.77	AGAATTACAGAGAGCCTGGTGTTGATTAGGGGAGGAATAACAGGACACCAGC[A/C]ACCTTAAAACCTGTCATTGTGCAACAGAATGGCAAATAGCCATTCATTCCTGGTTCA	A	C	0.07	0.30	0.31	0.39	0.30	0.32	0.68	0.20	0.31	0.28	0.25	0.052	0.058	0.011	0.000	0.0037			
rs735914	9	77.27	AGGAACTCAGCCACCTACCTGGTTTCCAGAATGTCCACC[A/G]TAGTTGTGCCACTTGATACAGAGAAAATCCTTTTAGCTATATGCTCAAGGACAGACTGCCATCAGATGGGAT	A	G	0.76	0.52	0.53	0.43	0.43	0.64	0.81	0.46	0.61	0.51	0.55	0.025	0.036	0.001	0.005	0.0001	1		
rs1417080	9	77.59	AGTAGACTAGAACCTTCAAAAAGTTAAATTCAAATGGAAGGATTCACAAAGTAATAT[A/G]ATATTTCTGCTTGCAAAATATGGTGGAAAAACTGTTTCTTAGGGTACAGCACATC	A	G	0.31	0.74	0.76	0.79	0.73	0.86	0.73	0.79	0.75	0.80	0.76	0.122	0.002	0.000	0.002	0.0002			
rs870713	9	79.68	TTTCTCATTTGTGTAAATGGCAGCACCAGGAAGCCAGGGGAATTAAGAGTATTCC[A/C]TACCCAATTTACAGAGGGCAAACCAAGGCACAGGCACCAAGTGTTTACCTGACTT	A	C	0.83	0.61	0.51	0.55	0.59	1.00	0.74	0.50	0.64	0.46	0.55	0.068	0.032	0.002	0.216	0.0001	1		
rs2378592	9	79.95	AGGAAGCAGTGAGCAGAGGAGCGGGCACAGGGTGACTTCCAGTGCTTAGGAAAC[A/G]TTTAAGAAAGCTTGAAAACCTTTTCAGACTTATTTCTTTTAGCATACATTAT	A	G	0.17	0.22	0.19	0.17	0.38	0.14	0.13	0.07	0.21	0.23	0.24	0.002	0.010	0.033	0.010	0.0010			
rs485522	9	80.80	ATCAAAAGGCATATGTCCTGGGTTTTAGAAAGGGTGTTTCATTTCAGGCAGTG[A/G]GAAATCATACATCATAATTTCTCAGCTCAGATTAGCATCACAAAGTCCAGGATCTATTTC	A	G	0.55	0.46	0.48	0.62	0.45	0.73	0.71	0.35	0.64	0.42	0.39	0.001	0.022	0.017	0.025	0.0037	1		
rs7846911	9	81.22	CATGCATGAAGAAAGGCAGTGGATTAAGGCAATGGATTTTTAGGCTTAAGCAGA[A/G]CTTGGGGAGGAGAGATGTGTATCAAATCCTAACTCACCTTCACCAAGATGTGGT	A	G	0.10	0.19	0.21	0.21	0.21	0.05	0.26	0.13	0.04	0.16	0.16	0.006	0.013	0.001	0.019	0.0013			
rs4877742	9	81.32	AGGATGACTTAGAAGTCGTATGAGAAAAGAAGGAGAAAAACACAAATTTCAATTTCAAA[C/G]CTAGCCATCTGCAAGCTTCCAAAATAAATTGTTGAGGATTCCAATCACAGAAA	C	G	0.10	0.20	0.19	0.20	0.18	0.00	0.15	0.04	0.18	0.21	0.27	0.007	0.001	0.017	0.060	0.0093		1	1[g]
rs1359168	9	81.37	TTTTACTGCAAGAAAGAAATGTAATTTACCTTGTGACTCAGCCTGCATTGTGCACACA[A/C]AATATAAGAAAAGCTTCACAAAAGAATAGTTACTTTCACTATGTGTATTGCATTC	A	C	0.76	0.40	0.43	0.34	0.48	0.41	0.46	0.39	0.25	0.33	0.44	0.040	0.000	0.008	0.002	0.0000			
rs944947	9	81.76	CTGTTGTTTACCAACAGCAAATACTTGCTTACTCATTCAACATATTCCTTC[A/G]TTTTGGTGCTATTCTGAATACATGGTAACTTCTGTCAGTGCCAATTAATAGTGTAATATT	A	G	0.69	0.78	0.83	0.83	0.88	1.00	0.99	0.73	0.82	0.81	0.79	0.018	0.033	0.022	0.050	0.0021	1		
rs918223	9	81.87	TGACTTTACAAACGTTCTAGCCTCTAAAGCAAAAACATAG[A/G]TCCTATTTTATTAAAGCAAACAAATCTCTTTGAGGTGTGCTTTCACTCC	A	G	0.67	0.45	0.39	0.35	0.38	0.27	0.05	0.53	0.54	0.40	0.40	0.036	0.078	0.029	0.006	0.0012			
rs1187356	9	82.76	GCAGAACACAGTTTTGAAAGCTCTTGGCCTCCAGTCAGGACAAATCATTTCACA[A/T]GAAGAAAATTTTTGGAACTAATACTTCTTCTCCAGCTTCAGGATAGGTCAAGCATGG	A	T	0.57	0.48	0.45	0.41	0.54	0.27	0.19	0.35	0.50	0.53	0.42	0.000	0.093	0.036	0.056	0.0018	1		
rs1778970	9	82.85	AGAAAGCTATGAGCTATGAAGTTTTCTGGCTTGGATGGGTCAGTGATAATTTC[A/G]GGGGTATGGCAGACTTTGTGTATTTTCTTGGGAAGTAAAAGAAAAAGGTATGGTTC	A	G	0.71	0.31	0.35	0.33	0.32	0.32	0.26	0.29	0.36	0.42	0.34	0.058	0.014	0.006	0.004	0.0001			
rs675837	9	83.73	TGATTTTCTTTTTATTTATGTCTAACTCCTCAGCAAAGGAAGGCCTTACTAAC[A/G]TGATAAACATTCACATTTTATTTTAGTTAAGCATTTCTTTAAATGTAAGAAATTGGAGGC	A	G	0.76	0.34	0.34	0.32	0.41	0.86	0.55	0.58	0.36	0.43	0.37	0.102	0.026	0.027	0.154	0.0000	1		
rs88644	9	84.56	GTCTAACAGAAACCCTAAGATGGCTGCCTCCACCACACTCCTCTCCC[A/C]GGAATCCTAGTTGGCGTGGAAGGAGGATAGCTGTTGGGAATTTCTCCTCTCCCAGCTCCCTTGA	A	C	0.55	0.53	0.51	0.56	0.50	0.82	0.90	0.82	0.58	0.57	0.48	0.008	0.155	0.103	0.085	0.0007	1		
rs912067	9	85.42	GGTGATAGTAAGTGGGAAATGATACACAGTTCTCTGTGATTATTCAGGAG[A/G]CTTAGTGAAATCACCTGGTTGATGGAATTTGAGAGATGGCTTTCTCCAACAGATGT	A	G	0.69	0.66	0.63	0.66	0.70	0.59	0.69	0.59	0.68	0.66	0.65	0.001	0.000	0.008	0.005	0.0001	1		
rs729958	9	85.74	AGGTTTCTTCCAGCAACATTCCCCTCTGTAAACCTTTTACAAACTGTGTGT[A/G]CACACACTGACATTAAAATTCCAAGAAAACTAATGTGTGACAGGCTCTGTTGCACAAATGA	A	G	0.79	0.41	0.44	0.48	0.46	0.50	0.23	0.40	0.27	0.42	0.48	0.083	0.010	0.004	0.012	0.0009			
rs560019	9	86.04	GGGGTCAAGCAAAGCTTCCTGGTGGAGGGCAGGTTTAAGCATGAGATGTTGAG[A/G]AGGTGTTCGCTGAAGAGAGAAATGGTGGGCAGAGGCCTGGAAGAGAGGACCCAG	A	G	0.57	0.60	0.56	0.65	0.71	0.32	0.24	0.78	0.64	0.66	0.57	0.000	0.064	0.013	0.041	0.0001	1		
rs4077800	9	87.79	AATCATAGCTAAATTCCTACTCTGAAACCTTTCTCTTTTGCTAAATTATCTGGAA[A/G]TCGTAACTTCAGATGCCCCATGTTTTTGGTTTTTTTTTTTTTTTTTTAGACAGAGTCTTG	A	G	0.02	0.26	0.22	0.27	0.13	0.00	0.04	0.15	0.32	0.25	0.22	0.028	0.021	0.001	0.065	0.0019	1		
rs7037744	9	88.25	ACTTTTTGCTGGGGTGCCTGGCCTTCTTCACATCTCCTTTAAATATCCTGCTGCCC[A/G]AATGAGTTTTCTTTTGAGGTTCTCTGTGCCCTTAAAAAGATCAAATCTATATTCT	A	G	0.76	0.40	0.39	0.30	0.41	0.60	0.30	0.63	0.39	0.41	0.41	0.077	0.003	0.035	0.023	0.0000			
rs890427	9	88.55	CATATCTCCTTTCATTCTCAGGGAACATGAAAATTCTGATGTGTTCTC[C/G]CCTCCAGATACCTTCGCTGAAGAGTCATGAACAACCCATGGGGTGAGCAGGCCTCC	C	G	0.95	0.80	0.84	0.85	0.86	0.77	0.91	0.78	0.82	0.76	0.84	0.023	0.008	0.000	0.001	0.0001			
rs2780701	9	88.91	TATCAATTTGAGGTTTTTTTTCTTTATGAATCAATTGTGCAAGGTTTGCCCTAC[A/G]TCTCTTAAAAGGTGGTTAAACTGGAATCTTAAAGACAGTTGAATTTAGCCAGACCA	A	G	0.79	0.53	0.58	0.72	0.61	0.50	0.56	0.55	0.25	0.49	0.51	0.042	0.002	0.000	0.000	0.0021	1		
rs751279	9	89.14	CGATACCTGGCTGTGTCCAGCAGAGGGGAAGTCTCCTGGGAAGAGGCCTTTGAG[A/T]GGTTTATACACATGTGCGATGTGGGCCAGCGGGCGGCCCCGGGAGCCAGCAAAG	A	T	0.62	1.00	1.00	1.00	1.00	0.86	1.00	0.86	1.00	0.99	0.99	0.155	0.000	0.049	0.050	0.0020			
rs4744070	9	89.71	CAAGGATACTACCAGACTACAACCACACTGCTTCTTTAAGAGCTTGAGTTCAGAT[A/C]TCCAATCCTGCAGTGGGTCCAAAGTTTGACTTCTTGCAGTGCAATTCTAAGAATTTC	A	C	0.55	0.14	0.14	0.10	0.13	0.36	0.04	0.17	0.25	0.19	0.16	0.091	0.020	0.000	0.040	0.0007	1		
rs1316268	9	90.08	GAAAAAAAGCAGGGCGGGTGACAGACCACCCAGGAGTGGCACGGAGCCAAAGAAACCCCCACTCCCAGCCAGGGGAAGCAGTGAGTGATTGTGTGACACC[A/G]CCTGAAAA	A	G	0.19	0.56	0.63	0.61	0.73	0.50	0.21	0.56	0.57	0.58	0.63	0.123	0.127	0.011	0.016	0.0003			
rs3780346	9	90.32	GAATTTACCAAAGACAAAGGATCATGTTAAAGAATACAGGATCCAATATGGCCAATGAC[A/G]AAACTCTGAGCATCCAAAAATATAACCCAAATGCAATTGATTGAAATATACTC	A	G	0.90	0.47	0.42	0.50	0.36	0.23	0.36	0.41	0.46	0.49	0.47	0.157	0.000	0.000	0.020	0.0005	1		
rs715846	9	90.61	GTTTTAGTAAAGTCCCATGGAATGATTCACTTCTCCAGTGATCATATTCAAATTTCA[A/G]TCAGGTTTCGGAATGGACAGAGAATTTCCAAAGCAAGAAAAGGAGGTAATCTCTTAC	A	G	0.98	0.97	0.98	1.00	1.00	0.73	0.57	0.96	0.93	0.97	0.98	0.002	0.143	0.000	0.083	0.0014			
rs1048510	9	92.66	GCACCTTGTCTAAGGACCCTAAATGAACGATAAACAGAGATGGTAGCTATGAGTAT[A/G]CAAAAGGTAAATCCACTTAATCACATACAGAGAGCAACAACAACTGCTTACGA	A	G	0.62	0.51	0.50	0.48	0.43	0.86	0.23	0.74	0.46	0.40	0.49	0.002	0.066	0.014	0.054	0.0003	1		
rs1027710	9	93.44	TATGCCCTTGTGAAGTTTAGCAGAGTGAAGGGAATTTCCTTTAATTTATTGAAGT[C/G]TGCTCTTCTGAAGCATAAATCAATAGATACATAGTTGTCAAAATGAGGTAAGCTCA	C	G	0.36	0.44	0.37	0.48	0.41	0.50	0.85	0.49	0.36	0.32	0.47	0.000	0.136	0.000	0.006	0.0034	1		
rs1335049	9	93.83	CTGTAGAACGGGCACATTTTAGTTTGCAAGAACTCACCAGTAAAGCCCAAACTAAT[A/G]AACCAGGTCCTTCACATTTCCTTTAATTATATAGGATTTTCTTTGTGTTTCTGTCT	A	G	0.64	0.64	0.55	0.56	0.63	0.55	0.71	0.56	0.64	0.64	0.55	0.012	0.016	0.000	0.000	0.0000			
rs700962	9	93.96	CCCTAAGTACCAAGTGGGAAGTAATGGATATGGACTTATTCATGCTGTCGTGGT[A/G]CAATGTAAGGACGCTGAGTCTGCAAGGATAGATAACTCATTTCCAAATAGGGTCCC	A	G	0.83	0.77	0.70	0.70	0.71	0.45	0.69	0.68	0.68	0.63	0.63	0.016	0.000	0.002	0.025	0.0022			
rs734631	9	94.20	CCAGGTCTGTGTAAGCCAGCCCTGTGTGCCATGACCTTTTCTCATGGCTTTA[C/G]ATTCTTCCCTTCTGTGCCCTCTCCCTTACCTCTAAGGGTGGGGCTAGTGGTGATAGTG	C	G	0.98	0.76	0.74	0.79	0.73	0.45	0.94	0.72	0.71	0.66	0.68	0.081	0.043	0.000	0.038	0.0018	1		
rs1020884	9	95.54	TCCACCTGCAAATTGGTAAGGTGGAAAGACACTTCACACACTTGTAGGAATCTC[A/G]GAGAAAAGACCATTTCATTGTTTCATATCACACCAGGCCATGAGCATTTTTGCATGT	A	G	0.79	0.49	0.59	0.54	0.68	0.68	0.23	0.50	0.71	0.56	0.66	0.020	0.066	0.000	0.008	0.0019	1		
rs952765	9	95.75	AAGATCTGGCCAGTTGGTGCCTTGACCCCTTGGTCAGCA[A/G]TTCCTCAGTGTCCCGCAGTGGGAGCCAGGAGCCCCTGACTGATAGCAGCGGCCATGAGTCCTCTACGTCA	A	G	0.81	0.66	0.64	0.71	0.64	1.00	0.89	0.79	0.68	0.75	0.66	0.016	0.046	0.014	0.140	0.0010			
rs894673	9	95.99	AGAGGGGTCCAAGGAGTCAAGGAAAATTCC[A/T]CCAGATTCCTTGACGGAGATTCCCCTGCATGCAGACGCAGAGGTAACCAGTTCTTCATTAAAGAAACCATGGAAATTCA	A	T	0.62	0.65	0.60	0.66	0.63	0.91	0.60	0.66	0.54	0.55	0.62	0.001	0.000	0.000	0.073	0.0010			
rs814027	9	96.29	GCCTCACATATTCCTATATAGCAATGCAAAATGGACTAACACACCCAGGAGCCACT[A/G]ATCAGAGCTTCTCATACTTTAGTGTTACAACACAGATTCTGATGTAGTGGGTCTGA	A	G	0.52	0.52	0.53	0.52	0.43	0.68	0.88	0.65	0.57	0.60	0.56	0.002	0.053	0.011	0.006	0.0000	1		
rs879354	9	96.47	CTACCCTGCCAACCTCCCAGGTCTGAGGTGCACTGCTGCCAAGAGCAAAC[A/G]TGCAGGATAGATTTACACCTTCCAGTGCACTTCCATGGCCATCCACTCATTTAATCCTG	A	G	0.38	0.42	0.43	0.52	0.55	0.50	0.24	0.44	0.46	0.49	0.42	0.001	0.017	0.003	0.003	0.0002			
rs1167768	9	96.71	ATGTGCTAAATGGCCCCCTGGAGACCAGTGCAAAGAAATGGCTCCAACCC[A/G]TGCTGGATTACTGGCCTCTATTCTCTTTCTTAGAGAGCAGCAGCAACAGTTTCCAGCTG	A	G	0.19	0.34	0.29	0.32	0.21	0.73	0.47	0.30	0.61	0.34	0.27	0.006	0.021	0.000	0.100	0.0002			
rs1555519	9	96.95	CAACACACATAATGCCTAGCACACAGAAGGTATTTAGTAAATGCTGGCTGAGCCT[A/G]GTACCGTGGCACACACCTATAGTCCCAGCTACTCTGGAGGCTGAGGCAGAAGAAT	A	G	0.33	0.63	0.64	0.63	0.55	0.45	0.40	0.54	0.46	0.59	0.72	0.051	0.035	0.016	0.025	0.0039	1		
rs1529191	9	98.23	GCAGTCACAGATCTACTTCAATTTCACTAGTACATGCCTTGCTCCAGTCAATGG[A/G]AAAATAGCTTGTCAGAAGATGAGACACCTTCTACCAATTAGCATGGGAAATTTGGAAT	A	G	0.19	0.39	0.44	0.40	0.45	0.95	0.96	0.74	0.54	0.47	0.47	0.032	0.234	0.069	0.207	0.0005	1		
rs1338121	9	98.39	AACAATAACAATTGTTCCTTTAATGCTTTTATTCTTATGGAAATGCTTCCACATAC[A/G]TTGTCTACATCAGATTTTTCAATAAGCTTGTAAATTGGACTGGCTAGGAATCAGTCCC	A	G	0.67	0.63	0.59	0.63	0.57	0.05	0.04	0.24	0.43	0.57	0.55	0.004	0.241	0.081	0.215	0.0004			
rs1930243	9	98.50	CGACCATAACCATAAATATTTGGGAGTCATTGGCATTATGTGGCATTATATTC[A/G]TTTTAAAGCTATGAGACTAGATCAGAGAGGAAAGATTTATTGCCAACCAGACAAGTCC	A	G	0.81	0.46	0.50	0.45	0.52	0.95	0.96	0.80	0.43	0.50	0.49	0.037	0.158	0.049	0.135	0.0000			
rs2026999	9	98.52	AGTTGACTCCCTCTACCAATG[A/G]GAGTTCCTGTTTCCCTAAATCCTTACCATTCTCACATGTTTAATGTGTTTGTGTTCCATGTTTATGTGAACTGTATAAATGGTAACAAGTGAAT	A	G	0.50	0.73	0.68	0.67	0.64	0.05	0.05	0.24	0.64	0.65	0.64	0.009	0.256	0.102	0.246	0.0004			
rs1338740	9	99.00	GAGAGAAAAGCTGTACAGCGCTGGCCACCTCCCAGGAGTTGTGGCATTTTGAA[A/G]TACTGGTAGTACCTAACCTGTAGAGACCCAGAAGGAAGGAGACTAGAATAATAAAG	A	G	0.21	0.58	0.52	0.49	0.41	0.00	0.24	0.36	0.43	0.52	0.52	0.042	0.043	0.016	0.230	0.0000	1		
rs1329088	9	99.26	CCAACCAGGCAACACCTATGTCCCATCTGTGAAGTGGCACCTGAGATCCTG[A/G]ACATTATTGCCCACAGCAGGTCATAGCCTCTGTTTATCAGCTTCTCCCCATCTCTGT	A	G	0.64	0.49	0.48	0.44	0.50	0.77	0.76	0.49	0.36	0.42	0.41	0.019	0.052	0.000	0.056	0.0026			
rs1323421	9	99.80	CTAAAATTATAATCCTGAAAACCTTCGCTGCCTATTAGTCATTTGGTACATGAC[A/C]TAAAGCAAAATATATTCTGCAATTTTGGACTTCAGTTTGGCTCCTCAGTAGACTGACTAC	A	C	0.52	0.49	0.48	0.51	0.63	0.35	0.52	0.42	0.38	0.47	0.56	0.000	0.001	0.001	0.018	0.0012	1		
rs2000182	9	100.17	GGGTACATGTGATATTGTATAATAATGAAATGTGGAATGATAAAATCAAGGT[A/G]ATTGGATATTTATCACTGCATGCATTTGTCATTTCTTTGTGTTTAGGAACATTCCAGTTTA	A	G	0.29	0.52	0.52	0.48	0.55	0.45	0.40	0.29	0.39	0.49	0.51	0.024	0.001	0.010	0.000	0.0001			
rs884416	9	100.70	TCCAGAGGAGTTAATTTGCAGAGAAGAGACAAAATTCAAAAGCAAGTTATAACTC[A/C]ATTCTTAGAAGTGACTGAGATTTTCTACTTGTTTATTAATGGATATATCTTCTTTTAAAT	A	C	0.33	0.55	0.57	0.55	0.61	0.35	0.84	0.65	0.46	0.55	0.51	0.020	0.076	0.013	0.008	0.0006	1		
rs949471	9	101.75	ATTATTATTTTACAAGCCCCATTAGATACATTTTAAATGAGTATTGATGCAATA[A/G]TTTATTGATATTTAAGGAACTTAGGCAAATGTGTTATGAAATTATGCTTATGGA	A	G	0.55	0.41	0.40	0.46	0.54	0.41	0.00	0.55	0.36	0.50	0.54	0.014	0.164	0.028	0.000	0.0094	1	1	1
rs363717	9	102.92	TATGAGAGAACTATTGTTTGGGAAGTTCAAGTGATCTTTGAATATTCCCAG[A/G]AATTTGAATATTAATGCCTAAAGGTGTAAGACTTCAGATTTC	A	G	0.95	0.85	0.85	0.83	0.71	0.91	0.99	0.82	0.75	0.78	0.86	0.011	0.037	0.000	0.004	0.0000	1		
rs6477450	9	103.82	TTTACAAATTGATTAGCCAAGAGGTGAAAGTAAGTAAACTGGGAAGAGGAAGAAG[A/T]GGGGCCTTACTTTTTCACAGGGAGAAACAGCCTTGCCATTGTGGTCTTTATGTGC	A	T	0.79	0.36	0.36	0.30	0.39	0.64	0.45	0.52	0.36	0.47	0.41	0.061	0.001	0.001	0.018	0.0003	1		
rs1961970	9	104.06	GGAAGTCATTTTGGTCTCTGCTCTCCCTTTTAGGCAGGGGATAAGTGAGCATCA[A/G]GGAGAAGCAATGATTCATCACCCTTGTGGCTAGCACAAAATATTGAGAACAGGTAAA	A	G	0.69	0.26	0.27	0.23	0.27	0.55	0.35	0.28	0.14	0.32	0.27	0.096	0.072	0.000	0.044	0.0001			
rs1516882	9	104.29	AAGTGCTTTTAATTGTAGTTAACACAAAGGTACATCATTTTGAGGAGGGCACAGATT[A/G]CTTCATTGCACCTCATATTCTAGGCTCTATCTTTGGGCTATCTTAAATTTTCTTA	A	G	0.79	0.64	0.72	0.70	0.61	0.45	0.79	0.86	0.75	0.64	0.65	0.000	0.000	0.000	0.066	0.0062			1
rs7850024	9	104.48	CTATGGCACACACATGGAGTGAGAACACAGTACCATAGTAATGGCAACAGTAAG[C/G]GGGGTAGGCAGGGAGTAAGGAGAGTAAATTCACAAGGCCAACATGCCCTTGTTTG	C	G	0.60	0.67	0.71	0.73	0.57	0.41	0.56	0.80	0.75	0.67	0.66	0.017	0.023	0.000	0.071	0.0033			
rs1491100	9	104.73	AGGTTTAAAATTAATTGATTTAAAGAGAAATATTAAGTTCAAAAACACTGGTG[A/G]GGCAAACCTGTGAAGTGGTGCCTCAGGGTGAATGATTGATGGAAAAATAACTGATTTC	A	G	0.19	0.69	0.60	0.60	0.64	0.77	0.97	0.36	0.64	0.62	0.59	0.068	0.131	0.024	0.028	0.0000	1		
rs1819730	9	105.61	TGGGCAGGAGAAGAATTGGAATATATAAACCTAGCAGTGACGTGAATGACAGT[A/G]GCCTTAACCAATAAACTAGAGATAAAAT	A	G	0.74	0.84	0.82	0.84	0.84	1.00	0.97	0.90	0.75	0.83	0.88	0.002	0.039	0.001	0.070	0.0012	1		
rs940287	9	105.82	GACAGGAGTTTCATTTCCAGAGTCAAGAAAGAAGACCATCCTGAAACATCCTCA[A/G]TGACCCTACCTGAGCAGATGGGGCGGAGCAGCAGGGAGGCCCTGGCATTCCTA	A	G	0.55	0.28	0.31	0.28	0.45	0.19	0.22	0.36	0.32	0.32	0.37	0.029	0.002	0.003	0.007	0.0006			
rs1407392	9	107.77	AACTCTACCTCATTGGACTTCA[A/G]TTATGCAACTAGTGAGATTCATCATATTTTTTGGGTTTCTTGGGATTCTCACCTTGTACCGGCTCAAGTTTGCTGGCTGTGACTCAGGC	A	G	0.55	0.46	0.51	0.55	0.57	1.00	0.92	0.54	0.79	0.57	0.47	0.001	0.071	0.007	0.164	0.0018	1		
rs1923433	9	107.78	GTGGTACTGTGTTGTCACAGAGACTGTTTCAAGATGGAAGGGATGACTCTAAATATTTACCCCAAA[A/G]AGAATACATTTATTCACATAAAGCCTGTACATGAATGTTTATA	A	G	0.21	0.34	0.43	0.45	0.48	0.59	0.75	0.41	0.50	0.40	0.44	0.029	0.045	0.010	0.005	0.0003			
rs1327532	9	108.51	ATACCATCTGCCTGTCAAGTATTGTTTTAAAACATTA[A/C]ATTTGCTAAATATTTACCTAGTGTTAGATGTAGGCCAAAAAGCAGCAACAGTGGAGTAGGAAGTTCATGAGTTTT	A	C	0.31	0.32	0.29	0.38	0.38	0.68	0.78	0.36	0.36	0.29	0.29	0.000	0.119	0.001	0.070	0.0000	1		
rs984071	9	108.52	AGAAACAATTT[A/G]AAATTCCAGGGAGGTATGGATAGTGATGTTAAATTTTTAATATCTCAACTACACTTTATTTCAATATCTTAAAATATATATAAGGAAAATGGTTTCATAT	A	G	0.14	0.30	0.37	0.24	0.39	0.27	0.00	0.36	0.25	0.33	0.36	0.030	0.117	0.003	0.000	0.0002			
rs1410049	9	108.55	CTCCTGCTGCAGTCCTAAAAAATGTCCTCTCCCGGAAAACATAACACATATACTTGT[A/T]CATGGGGACGATTTCAGTGTGAATGGCAAGTTTCTGTCATGTGCAGAAGGGTAT	A	T	0.57	0.46	0.44	0.57	0.43	0.45	0.53	0.36	0.61	0.49	0.36	0.054	0.000	0.032	0.005	0.0067		1	1
rs1413219	9	110.04	GTATTGGATGGCAGTCTTAGGGAATTTCAATATCAACTGATTCTTTAATTCTGA[A/G]TTTGAGAATTTCTTTTTATAATACACACGGTCGCTAAGGCGCTTAGAGATCTGTGAGGC	A	G	0.36	0.32	0.43	0.35	0.41	0.36	0.04	0.36	0.32	0.44	0.51	0.000	0.088	0.001	0.000	0.0065	1	1	1
rs1324935	9	111.14	GTTGGAAGATAATATCAATGGATCCTAGAATTATGTCCTAAGTAGTCTTCAG[A/G]AATTCTGCGATAGAGAAATGTTGATACTTCTAAGGTAACAAAGTAGACTATTTACAAAGT	A	G	0.64	0.30	0.35	0.46	0.25	0.77	0.39	0.52	0.43	0.41	0.38	0.053	0.003	0.021	0.117	0.0006	1	1	
rs1128786	9	111.30	GATTTTCTCTCTTTTCCTTTTTTATAAAGTGAAGAGGTTCAACTATCCAGA[C/G]AGTCCTATCTAACTCAAAAAATGTAATGGCTTTGGTTGGATTGCATCTTC	C	G	0.19	0.30	0.32	0.33	0.39	0.14	0.17	0.32	0.39	0.37	0.34	0.014	0.015	0.000	0.026	0.0000			
rs1040989	9	111.51	ATCCTTACGGTATCTATTGACTTCATTATATTTAAGTGTTTGTATAGTTCTTTGCCTAATATCCGTATCCTCATTGAGCCTGTA[A/T]ATGCCCTAAGGGCTGGAAATCTATC	A	T	0.48	0.60	0.72	0.61	0.54	0.30	0.58	0.73	0.69	0.65	0.69	0.051	0.026	0.003	0.125	0.0009			
rs14419	9	111.55	GGGCCAAGTGGGTCTATGAAAAACAAAATTCAGTTAATGCTTCTGTCAAGTGAAGAGAGAGAAC[A/G]GATTTTGTTATTCTCCCACAGCATTTTAGTAGTTTCCTAAAGCTTAC	A	G	0.38	0.44	0.34	0.38	0.43	0.06	0.02	0.19	0.32	0.39	0.30	0.005	0.066	0.003	0.039	0.0001			
rs1871692	9	111.88	CTCATCTATTCCTCTTTCCCTAGTTGAGCAAGCATGAAGCATTGTGTTGGTG[A/C]TGTGTCTGCCACATCTGTCCCTGAGTGGTCTTTCACTATCATGCAGAGTAACTTCTTT	A	C	0.40	0.66	0.73	0.71	0.73	0.27	0.17	0.66	0.73	0.72	0.71	0.080	0.173	0.004	0.125	0.0029	1		
rs1000709	9	112.61	TGGAGCTGGTTATAGTTTTATAGCCCTTTTCAGGAGTGTCCTTCTCTGTGCC[A/G]TGGTATGAGACCTGTCCCGTGAGGCTGAAGACAAAGTACAGAGGGAAGAACAGATGTA	A	G	0.33	0.45	0.51	0.50	0.61	0.59	0.39	0.56	0.57	0.48	0.49	0.018	0.013	0.002	0.001	0.0001	1		
rs726657	9	113.07	CTTCTAGAACTTTCTCTTTCCATAAACATGAAGCATAGATTGAAGGTCAACCCT[A/G]CATATATGACCAGCAGCAAGAACGCAAGGTGGGATGCAAGGACTTAGAAGACTAG	A	G	0.74	0.52	0.40	0.49	0.48	0.36	0.49	0.41	0.42	0.42	0.50	0.038	0.002	0.008	0.006	0.0042			
rs1060545	9	113.20	TTGAGGGATTTAGAGATAGAAAAGCACCATTTCCAACTAGCAAAAGACTCTCAG[A/C]AGTAATAATCTGTGTTCCACCAGAACTCAAATATAGGAGGGAAGCTTTTAGTATC	A	C	0.38	0.54	0.49	0.59	0.57	0.82	0.75	0.67	0.46	0.59	0.71	0.016	0.017	0.014	0.036	0.0226		1	1
rs944510	9	113.23	AAGACCTGGCACCGCCTTCAGAGCCCAGCGAAAGCTTTCAGGAGCACACACTC[A/G]GATGGGGAAAACCAGATTGTCTTCACACATCGCATCAACATCCCCCCGCCGGGGCTG	A	G	0.60	0.39	0.40	0.43	0.54	0.27	0.59	0.29	0.46	0.49	0.38	0.030	0.039	0.000	0.002	0.0002			
rs1250019	9	113.23	CTTGTTATATGAGGTCATGGTTTAGAAGAGGTAAGTAGCTGTCAAAGTT[A/G]CAGGGCTAGTGAGTGAGATTTCAAACCCTCTGTTCTGAGTTTTTCCCAAAGCCTAACATT	A	G	0.64	0.52	0.50	0.61	0.57	0.41	0.63	0.40	0.50	0.65	0.65	0.015	0.013	0.003	0.001	0.0157			
rs1757096	9	113.23	ACAACAAATGGTTCTCAAGCCAGATGGAGCCTGAGGGTGCAACCGTCCTTC[A/G]CTGCTGAATTTGAATCTGGGTGCCCTGGGTAGGTAGACACAGAAAAATGGCTGCTAAGCCTC	A	G	0.55	0.61	0.60	0.59	0.46	0.73	0.38	0.71	0.57	0.51	0.62	0.005	0.046	0.000	0.002	0.0003			
rs1250288	9	113.38	TGTAATTAAATTGAAGCTGTCAAGATTAAGAGATCATTCTAGATTATCTGACTGGAC[A/G]CTTAATACAGTGACAAGTGTCTTTATAAAAGGCACACAGAGAAAAAAGGAGGAA	A	G	0.55	0.40	0.35	0.39	0.36	0.82	0.96	0.34	0.54	0.34	0.38	0.040	0.309	0.004	0.173	0.0019			
rs1405	9	114.33	CCAGGCAGTGGAGAGGGTGAGGAGTGGTGTATAGAAGAGAGC[A/G]TGGAGTTTAAGGGGTTATTATGGCTGAGATCCAGACCATGAGCAGAGAAAAGTTCAGTTTATCTCACC	A	G	0.90	0.33	0.44	0.41	0.45	0.82	0.86	0.65	0.54	0.55	0.41	0.132	0.102	0.012	0.071	0.0004	1		
rs1334071	9	115.19	CATCAAGGGCCCTTGCAGGTTTAAGCAACTCTAAGGAGGTCTGGCCTAAGGAAGC[A/G]GGACCCAAGGTTCTTGCTCAGCACCCAAACCCTCATTCTGGTAAAGAAAAGAATT	A	G	0.38	0.58	0.53	0.56	0.46	0.64	0.10	0.57	0.43	0.51	0.49	0.011	0.107	0.001	0.008	0.0003	1		
rs1572306	9	115.45	GTACTTCTGGGTCCTGGCCTAAGCCATTCCTTTTACTGTCCTCTGCCACTG[A/G]CTGCATCTACTACTTATGGTAGCTGACCAAAATCAGTTATTTAGCCTAAGACAGCAG	A	G	0.07	0.21	0.23	0.21	0.25	0.14	0.32	0.14	0.29	0.23	0.24	0.028	0.007	0.006	0.008	0.0000			
rs734096	9	116.10	GTCCACTGGTTTCACATATATCTCCATCCATTAGAGTTAGTATCTGGTTAT[A/G]TTTTGGTTCCATGACTTTGCCACATCATAAATACTATTCTGTTTTTAAAATTTATTACCTT	A	G	0.63	0.65	0.56	0.60	0.57	0.65	0.48	0.66	0.75	0.58	0.58	0.003	0.003	0.010	0.004	0.0003	1		
rs724975	9	117.16	AGTTGGCTAAATCAGGGCTCTGTGGTTTGAGACAGAATCCCAATTATTTCCAAT[A/G]GAGATAGCAGCGACAGCTGCTCTTAACCCAACCACTCCTCACTGCTGCTAACCTG	A	G	0.02	0.15	0.14	0.10	0.16	0.36	0.26	0.16	0.11	0.14	0.16	0.025	0.015	0.000	0.040	0.0000	1		
rs230146	9	117.25	AGCATCTCTTTTCATAGTTGGGTTGATAGTAACAGATGGTCATGTTCACTCAGCAC[A/G]GACAAATAGCTCGAATGATAAACACAACCAGGGTTGAGAGGTGCAGGAGGGAGATT	A	G	0.86	0.73	0.76	0.73	0.75	0.55	0.55	0.61	0.82	0.78	0.82	0.002	0.046	0.040	0.052	0.0016			
rs914652	9	117.34	CTGGGTTTGGACGCCCTGGTTTAGTGTATGGTAAAGCCAAAGGCAGGGTTTGCTC[A/G]TCAAGGTTGAGGGTAGTAGACCAACATTGAAATATATATACTGTAGTGTGCTGA	A	G	0.40	0.18	0.17	0.24	0.27	0.23	0.47	0.26	0.21	0.24	0.17	0.031	0.074	0.003	0.007	0.0000			

rs1013324	9	118.39	ATCAAGGTAGTCATTCATTGCAGCTACAGGTGAGATCAATTTGTCCTCTAGGAGAT[A/G]ACCTCCTCCCTCCCCAAACTGCTGCTTTGTTTTAGTTTGTATTGTTTTATTTTATTA	A	G	0.57	0.19	0.14	0.21	0.21	0.00	0.04	0.11	0.18	0.15	0.15	0.127	0.005	0.001	0.031	0.0002	1		
rs769012	9	119.61	CCCTGAGATGACGAGTTTGCTGGGTAAGATAGTCTCAGGCCTCTTCCCTCTAA[A/G]TACCATATGAGCCAGCACATACAGCCCCAGACCCGCTGGCAAAGTCAGAAAAGGCAC	A	G	0.45	0.60	0.62	0.57	0.69	0.55	0.61	0.76	0.68	0.57	0.48	0.018	0.000	0.003	0.003	0.0130	1	1	1
rs656328	9	119.71	GGCTGACCTGACCGAGCTGGGATGAGGTCGCCCTGCAATTACCACCTCTTCCG[A/T]GGCCAACAGGACTTCTCCCTGCTGAGTCCCAAACCTGCTGCCCCACCCCCCCAT	A	T	0.69	0.76	0.74	0.70	0.75	1.00	1.00	0.91	0.75	0.75	0.64	0.001	0.106	0.020	0.106	0.0043			
rs4679	9	120.29	CTGCGGCCCATCACTATGGAGCTCAATGTGATAAGCCCAACGAAGGA[A/G]TTTATGCTCTGCCGCTGGGAAGAGAAAGATCCGAGGCGGTGTTTAGAGGAAGGCAAACTGGTC	A	G	0.14	0.63	0.65	0.61	0.46	0.27	0.24	0.49	0.61	0.61	0.59	0.123	0.083	0.012	0.073	0.0015	1		
rs913932	9	120.33	TGGGGGCCAGGCCCTGGGTCTGATCCCCAAGTGAAACCCTGGTGGATGCAGGG[A/G]CTCAACAAGGCCTGTGTCTAGGGGGAGCTTAGGGGGCCTAAGGCTCTAGGTGC	A	G	0.48	0.57	0.51	0.50	0.61	0.77	0.97	0.54	0.57	0.46	0.54	0.000	0.181	0.002	0.056	0.0001			
rs917783	9	122.17	TACCGCGCACCAGGCTGGGGCACCCCGGCCTGGTGTTAGTTGTGTCCTGATG[A/C]TTCTCTGTCTGAACTTCGCCTTCTTGTCCCAGACACACAGTCCAGAGAACTTGAGGCA	A	C	0.67	0.60	0.58	0.51	0.63	0.59	0.28	0.74	0.54	0.66	0.58	0.006	0.050	0.008	0.000	0.0000	1		
rs2148742	9	122.25	GAGAATCCCAACCTAAGATATGAGATAAAGATTCCCAGAGACCTGGCCTCAGCAAC[A/C]ACAGGACATGCTCCCTGTATGTCTCCCCCTATACATGAGGTACACTAGGTGGGC	A	C	0.38	0.67	0.73	0.67	0.71	0.64	0.71	0.60	0.71	0.73	0.73	0.099	0.005	0.021	0.014	0.0004			
rs1984001	9	122.51	GTTATATTTGGTTGTTACATCTGTTTCGGTATCTCGTTTGTCTGGGGGGAA[A/G]AATGTGTATTTAGACCCCAAATTATTTTCTCATTAAATGCTTTTAAGGATTTATGCTAGGCC	A	G	0.62	0.40	0.42	0.49	0.38	0.77	0.59	0.66	0.46	0.45	0.42	0.020	0.015	0.058	0.074	0.0006			
rs12009	9	123.37	TAACCCTATTGCCCCAGGGTACACAGCACTGTAGGTCATCTTTAATGGCT[A/G]TATTGGGCTTGGCCTGAGACCAGTATTCTGCTCAGGGTCTGCCCAAATGC	A	G	0.40	0.56	0.56	0.50	0.57	0.45	0.98	0.52	0.71	0.54	0.61	0.015	0.140	0.000	0.006	0.0013	1		
rs1055352	9	124.29	GCCTGGGACCAGAGAGGAAAGTGGGGTGAAACCAGGTGGAACAAATCTGT[A/G]TTTTTACACCATCTTCGGGCACCTTGTTGGTTTGCAGGGGACCCAGGAGA	A	G	0.12	0.46	0.50	0.35	0.54	0.23	0.35	0.34	0.43	0.42	0.46	0.094	0.010	0.014	0.041	0.0022	1		
rs741028	9	124.56	AGTAGATTCCTTTTCCTGTATCTTTCACTAAGACAGTTGGCTCCCATCATG[C/G]CTGCTAAGGGTGAGACGTGGCACAGAGTTGCAAGAGACACCAGTGGAGACGATATGTAG	C	G	0.65	0.56	0.54	0.60	0.55	0.77	0.38	0.51	0.50	0.53	0.61	0.007	0.010	0.003	0.034	0.0025			
rs1336980	9	124.75	GCCGGCGGCAGAGCGGGCGCGGGCCGGGCCCGGCAGGGGGCTGACCGGGCTCGAGTGCCGGGCGGTGGCGGGGAAGGAGGACGAGAAG[C/G]GTCTACTGTTGGTAGTC	C	G	0.88	0.56	0.61	0.69	0.73	0.80	0.98	0.67	0.73	0.61	0.61	0.036	0.110	0.001	0.012	0.0000			
rs624903	9	125.02	CATATATAAAATTTAATAAATAACCCTGACCAGTCTAGAAGCACCATTCAAGG[A/G]GCATGTGCCCTTGGAAAATAGTTTAGGTGTTGCATATTTGAGTCCCTTTCAGTGGAGG	A	G	0.17	0.08	0.14	0.16	0.02	0.00	0.01	0.01	0.07	0.18	0.14	0.001	0.024	0.021	0.041	0.0001	1		
rs947626	9	126.15	CACAGTCCTGCCTGAGCCTCCTTCCAGCACGGGGGTTGATCCCAAGAGTGAGTA[A/C]TGTATTTCAAGAAAACAAGCCTCCAAGTGTAAACCCTTATTGAAACTCTGCAGCA	A	C	0.45	0.18	0.15	0.15	0.20	0.59	0.12	0.47	0.07	0.27	0.21	0.089	0.000	0.102	0.144	0.0040	1		
rs878400	9	126.28	TACCTGGCTCCTAGAAGCCTAGCCAGTCACCTCCTGCCCAGCCTTCAAACAC[A/G]TCAGAGGGTTAACCATTCATGGCCACGCTGAACACTACCTCACAGATCCGGAAACCAC	A	G	0.21	0.51	0.58	0.62	0.59	0.59	0.77	0.63	0.57	0.54	0.65	0.076	0.032	0.001	0.001	0.0053			1
rs751340	9	127.07	CTGCAGTTCCAACAGGATGGATTCCTGGTGCTGGAAGGATTCTTGTCTGC[A/G]GAAGAGTGTGTGGCCATGCAACAAAGGATTGGCGAGATAGTGGCTGAAATGGATGTCC	A	G	0.52	0.39	0.35	0.33	0.43	0.14	0.42	0.43	0.39	0.38	0.43	0.014	0.001	0.001	0.039	0.0023	1		
rs913275	9	127.29	GGGGCCCATTGTATCTGGAGAGCCTGGATTCCTCTTGGCAGTCTAGGCCC[A/G]GCCACTTCTGCTACCTTTGCGCTGCTGTGAGCCTCACCCTGGGCCCCTGGCCTCCTAAG	A	G	0.38	0.71	0.70	0.68	0.75	0.68	0.57	0.66	0.71	0.69	0.69	0.056	0.010	0.000	0.000	0.0002			
rs1220789	9	127.63	GAGCTGGGATTTGACCCCGAGTCTGCGGGTTCTGAGCTGTACTCCTCCCCAG[A/G]GCAAGATTCTGCTTCTGCTGGGAGGCGCTGCTTCTAGGACTCTCCGTCTCTCCGG	A	G	0.07	0.40	0.42	0.44	0.39	0.18	0.26	0.48	0.57	0.45	0.42	0.072	0.009	0.006	0.023	0.0004	1		
rs1054879	9	128.37	TTCGGCAGCCGGAGAGGCTGTGGTGGGCCGAGGGTGTCTAGGGGTTCTGCCT[A/G]GTCAACGTTATTTGTCGTCCCATGTTTGGCAGCAAAACCACCTGCGTGG	A	G	0.26	0.50	0.50	0.55	0.41	0.09	0.30	0.60	0.64	0.55	0.49	0.026	0.019	0.010	0.097	0.0001	1		
rs818053	9	129.24	GGTGCGGAGAGGCCCTCAGCTGACCTCTGGTTCAGGCTCGAGACGAACTCA[A/T]CAGCCAAGTGTCCGAGGATGGTGAGGAGCAGGGAGGGGCGCCATCCAGGAGGGGGA	A	T	0.74	0.49	0.48	0.50	0.64	0.71	0.33	0.67	0.54	0.61	0.53	0.030	0.012	0.014	0.027	0.0015	1		
rs818052	9	129.25	AGCTTCTCCTTAGGTGAAGTCCCACCAACCCCCGTTCAGGATAATGAGGA[A/T]CTCTGGATCTAAGGCCAATAATGGATGACCGGTGCCACCCCCAACCTAATGGGAGA	A	T	0.26	0.52	0.53	0.50	0.36	0.55	0.67	0.34	0.50	0.41	0.47	0.035	0.012	0.011	0.001	0.0025			
rs735107	9	129.47	TATAGAATATAATTGCTTTTTTTCCTCCAAAATATGTCTACATTTTTACAAAC[A/G]TGTCGAAAAACATCCGAAAGGAATAACTAAAATATTTTTTCTTTGTTTCCTTGTATGTT	A	G	0.45	0.60	0.61	0.62	0.73	0.50	0.48	0.61	0.54	0.67	0.65	0.022	0.024	0.004	0.018	0.0022			
rs565325	9	130.63	AAGAGGAGGGTGGGTCACTAGGGGAAGGTTTCTCAGAGCATGCGGTATGGAC[A/G]CAGTGAGTAAGGGTTGAGTTAGACTTTTCTGATAGTCAATCTTGAGGGTGTTCCTG	A	G	0.29	0.57	0.55	0.51	0.59	0.45	0.17	0.50	0.57	0.59	0.60	0.027	0.074	0.000	0.003	0.0032	1		
rs456396	9	130.84	AGGGCCAGCGCCCCAGAATAACAGTTCTAGTCAC[A/G]CTCTACACTTCTTGGTATTGTGTTGTCGGGGGTGGGGGGTGGGAGTAGGCCCCTGCTGCTCTTTACGGTGTTAC	A	G	0.57	0.41	0.38	0.48	0.34	0.59	0.78	0.43	0.39	0.33	0.43	0.027	0.079	0.004	0.024	0.0034			
rs215156	9	130.94	TTTCCTGAAGTTAATAATCATCTTGCTTTTTCACTTGTCTAGTTTTCTTTGTACCT[A/G]TTAGTTAATTATTTCCACATCTCATAATAGCCTTTCAGAACAATTTCCCACAAAGTCC	A	G	0.45	0.33	0.33	0.40	0.27	0.23	0.25	0.21	0.36	0.41	0.37	0.001	0.013	0.019	0.020	0.0002			
rs886017	9	131.25	CTTCACTCCCTTGGTGAAGGTCCGCTTCTCAGGACAAAGTCATAGTTGGC[A/G]GTAGAGTTCATGGCATAGAAGACGATTGGCGTTTTCAGGGATCTTCAGCTCTGGTGGGGA	A	G	0.43	0.51	0.52	0.61	0.55	0.64	0.71	0.66	0.54	0.49	0.47	0.003	0.024	0.004	0.008	0.0032	1		
rs12335	9	131.47	CCTCTGTCTGGTAACAGTCACACCAGCTCCTGTTCAGTTACAGTCTTTGTCTC[A/G]TAACCGGAGAGTTTCATCAAAAGAATTTTATTTACAGCTTTATCATCCATATGCCACTA	A	G	0.43	0.46	0.46	0.50	0.43	0.55	0.60	0.58	0.36	0.58	0.56	0.000	0.012	0.010	0.007	0.0027			
rs1611122	9	131.85	CTGACTCCAGTTGGGAAACCAGAATCAGGACCCACTGAGGGCATAAGGTCCTG[C/G]GGCACTTGCGTTTCTGTGGAAAAATAAAAAAAAGGCTTGAAGCATTTCAGACATCACA	C	G	0.55	0.55	0.46	0.45	0.48	0.91	0.94	0.72	0.77	0.55	0.44	0.010	0.209	0.041	0.156	0.0000	1		
rs877954	9	132.77	CTACCAAGCAGAACCACCAGAGGCCAGGCGACACCAGCACATAGTAGGTACTTC[A/G]CAAGCATGTGTAGAATGGATGGCCAGATGCCCACACATACTAAACACTCTCTACA	A	G	0.79	0.35	0.37	0.29	0.36	0.41	0.34	0.51	0.18	0.35	0.41	0.124	0.002	0.028	0.007	0.0029	1		
rs914400	9	133.73	AGTCGGGGCTGAGACACATGTTCCAGAAGAAAGTGCCCTCTCTGCAGAAAGAGTGGGACCTCGG[A/G]CTGTGTCGGGAAGACAGAGAGAGGAAAGCAGGTAGCTGTGCGC	A	G	0.24	0.45	0.45	0.41	0.43	0.36	0.23	0.52	0.46	0.42	0.41	0.025	0.025	0.008	0.002	0.0003	1		
rs705670	9	133.91	CTCACAAGAGGGCCCTGTCCCAGCCTCTGGCCTGGCTCCCCTTCTGCTGA[C/G]GGATGTGGGGCTATCAGGGACCAGGACTCCTGACTGCCCCTTGAATATCTCTGTCTAG	C	G	0.57	0.65	0.78	0.76	0.64	0.41	0.44	0.59	0.68	0.72	0.74	0.028	0.063	0.032	0.083	0.0012			
rs710411	9	134.80	GTAGGGCCCCTTCACC[A/G]TTTGAAGCTGACTGTAAAAACATCTGACCTGTGTGCAAAGCTCAAGCCCAGAGGCCTCCAGGGTGTCCAGCCTCCAGGGTGCCTCCTACGCA	A	G	0.38	0.62	0.59	0.61	0.73	0.65	0.17	0.58	0.62	0.58	0.67	0.035	0.120	0.004	0.000	0.0023	1		
rs945383	9	134.98	AACAGATATATGCGGTATTTTAAAAACTGATTTTACAAAAT[A/C]CAGCCATGAGATGTACAACTCATTCCCTAGGAAACAGCCCAGGAATGGCCATCTTCCCAGCCCCGTACG	A	C	0.33	0.27	0.22	0.28	0.32	0.23	0.47	0.18	0.25	0.18	0.17	0.005	0.032	0.000	0.000	0.0027			
rs908831	9	135.18	ATAGAAGCCCAGTATGTTAAAGACAAACTTCACAATCATTAGACGAAAACATGACAGTGGTTTCCAGCCTCAGGGAAGGTCAGGATGTTCTGAGTC[A/G]TCGGAAATAGCA	A	G	0.57	0.61	0.61	0.59	0.66	0.86	0.40	0.74	0.71	0.61	0.55	0.000	0.010	0.038	0.071	0.0009			
rs1018330	9	135.34	AGCAAGTGTCACAGGGGGCAGAGACCAGCAGAACCTAAAGATGACCCTAGAAGAGCAGGAGGCCAGGGGAACAGAGTTAGACGAGCCA[A/C]GTGCTAAGCAGGACGTC	A	C	0.29	0.64	0.73	0.74	0.75	0.32	0.35	0.77	0.61	0.73	0.76	0.093	0.054	0.003	0.065	0.0004			
rs7028989	9	136.13	TAGGAGTGGAAGGAAGGTGTCTGCATGTAGTTATCATGAAGCCATGGATGGAATATC[A/G]TAGCAGAGCGACTCACGGTGAGTTCACGCTGGAGACAGATGAGCTGCGAGAT	A	G	0.60	0.12	0.13	0.20	0.07	0.55	0.06	0.29	0.18	0.14	0.16	0.118	0.007	0.036	0.113	0.0001	1		
rs7860423	9	136.17	TCCAACCCTGGCTGCCGTGGTAATCCAGTCGCAGTGAAGACTGCGCACAGGAGC[A/G]TGTTCATAGGAGCATGGAGAAAGGCCCAGTGAAGCCCCTGATTCCCAGAATTATG	A	G	0.74	0.14	0.15	0.14	0.13	0.59	0.06	0.27	0.21	0.14	0.17	0.196	0.007	0.028	0.134	0.0003			
rs766374	9	136.22	CCTAGCA[C/G]CACCGTCAAGGTGTGCAACAGCTGATCACTCCCAAATTCCTCTGTGCTCCCTTGAGTCGACCCCTCGTCCTACTGGCAGCCTCTGCCACCAATGATGTTTT	C	G	0.12	0.54	0.51	0.62	0.54	0.30	0.71	0.52	0.61	0.45	0.45	0.107	0.021	0.000	0.024	0.0026			
rs7909028	10	0.13	AAAAGAAAGTAAAAAAATTCCCGCACAGATTCTCATTTGCCATTGCCACAAATAGGT[A/G]GCAATGAGAAGGGAATTTCTGTAAAATGTAACATGGAAGAAGCATGTTCACAGC	A	G	0.62	0.70	0.65	0.73	0.55	0.86	0.77	0.74	0.71	0.57	0.56	0.003	0.008	0.005	0.017	0.0027	1		
rs1476130	10	0.23	TTATGGAACACAGGTTTGGTATCTACTTGTTCCTTTCCCCTTCTACATAACACT[A/T]GTTCTCTAAAGGAAGAACAAGACTGAACGGCAGAGGCAGAATGCGTTCTTCAGCGGAA	A	T	0.36	0.30	0.35	0.24	0.45	0.23	0.23	0.26	0.36	0.41	0.42	0.002	0.007	0.004	0.002	0.0013			
rs2018975	10	0.27	CCACGACAGTGCATCCTGCTGTCTCTGCTCTGAGCTAAACTGCAGGAGAACCCC[A/G]CCTTTAGGGACCTTTTCATCCCAGGAACCCCAGCAACACCTAGGCTCCAGTACTG	A	G	0.36	0.38	0.31	0.32	0.21	0.55	0.64	0.42	0.32	0.28	0.23	0.000	0.050	0.002	0.019	0.0042			1
rs6560842	10	0.45	GGGGCCCTCGTCAATGAAGTGAGTCAGGAGAACTAAAAGGCACAGACTTGGAAGGC[A/G]TGAGACACTTGATTTACCGAAGACACAGGGCCTTATTTAGAAAATTTTTAGGAA	A	G	0.45	0.96	0.98	0.94	0.96	0.95	1.00	0.98	1.00	0.94	0.95	0.220	0.000	0.000	0.016	0.0009			
rs816570	10	0.63	TTAGTAGAGACAGGGTTTCACTCTTAAGGACGTTGTTCAGTAAGTACTAATTGGT[A/G]GCCTGCCGTTGAACTAATTTCAGTGTAGTACTGACATCATCAGCCAAGGCAGAAAC	A	G	0.07	0.26	0.35	0.26	0.41	0.05	0.18	0.24	0.35	0.30	0.45	0.077	0.023	0.010	0.086	0.0046			
rs1057304	10	0.81	CTCCACGCATGCACATTTAGGATCACCGTGTCTTCTCGTTGGACTGATCTGTC[A/G]TTAGGACCTCGGACCCAAGTAATTGTCTTTGCTCTGAAGTTTTGACAGTAACAAAGGC	A	G	0.79	0.55	0.52	0.52	0.57	0.77	0.56	0.49	0.38	0.60	0.62	0.055	0.018	0.005	0.060	0.0076		1	1
rs1029182	10	1.92	TCTCTGCTTTGTCGAGGAGTCTGAATTGACTTCCTGCAGAGAGAAATCCCC[A/G]CACCCCGTTTCAAATAACGTTGGTTCCTCTGGGGAGAGCTGTGGGTCTGTCTGTTCTCA	A	G	0.50	0.66	0.68	0.70	0.61	0.59	0.50	0.57	0.75	0.71	0.67	0.019	0.014	0.005	0.004	0.0000	1		
rs729397	10	2.49	TGAACAGAGATGAGGTTTCCCCAAGAAGGTTCCCGGTGTGGATACGCTCCCCA[C/G]GATAGGACTGCAGGATCTGGGGCAGCGCCTTCTCATGGCGCAGGGTCTGCATG	C	G	0.24	0.21	0.30	0.34	0.21	0.09	0.02	0.16	0.25	0.28	0.30	0.005	0.105	0.017	0.044	0.0000	1		
rs729260	10	2.49	AGAAAATTCCAGACTAAAGACTGTGGAAATGATTCCAGTTTTACCATCTATAC[A/G]TCAGTAATTTGGTATTAAAAACAAAGGTTTGCTCATATCGTATTTAGTTATTCTTTTGCA	A	G	0.79	0.60	0.67	0.67	0.52	0.64	0.83	0.74	0.50	0.62	0.73	0.008	0.005	0.016	0.005	0.0014			
rs4328141	10	2.84	GATGGAGAAACCCAGCTGACCATTTTCAGATGGGAAACATTTACTTGATAATTGCTC[A/G]ACTTTCCCCAAGGAAAGGATGGGCTATTTGAAAGATTGTTTCTATCTTCCGT	A	G	0.12	0.58	0.55	0.54	0.55	0.68	0.84	0.43	0.69	0.50	0.55	0.111	0.060	0.015	0.011	0.0002			
rs7084192	10	3.01	GAAGGGGTAGAACCTTACAACCTTGGGTAGGGATGTTAAGGTTCATGAAGTTGAG[A/G]ACCTCCAGTGCCAGACTTCCCTGAACCCCCAGGGCCTGCAGTCATGCCCCCACCT	A	G	0.69	0.14	0.13	0.09	0.13	0.09	0.02	0.20	0.04	0.16	0.18	0.197	0.013	0.020	0.000	0.0023			
rs3814595	10	3.16	GGCATGGTTCCGACCTAGTCTCTGGAAACCACCGGAATTACAAAGTTTTGTATGTG[A/G]TATATTTGCCGAGGTGCATGTTCATAGTACCATTCTCACACTCAAAGGGACCTC	A	G	0.33	0.37	0.26	0.28	0.21	0.32	0.64	0.20	0.29	0.30	0.25	0.008	0.114	0.004	0.009	0.0000			
rs735155	10	3.33	GCTGAAGGGTCGGGAGAGGCCTGCTGGGCCGCTGGAGAGGGGAGACCTGCTG[A/G]CTTCCCGTTGAATTCGGTGACACTCTCACACAGACCGTTCATTCTCCCGACTAGC	A	G	0.40	0.47	0.43	0.49	0.57	0.86	0.52	0.59	0.39	0.45	0.48	0.000	0.002	0.021	0.124	0.0009			
rs705466	10	3.60	GTCAGAAGATGGGCTACTCCCCTCTCCACAGGCAGGTGAAGCTCACTTCCGATCT[A/G]TTTTTCTCAGTTAATGTGGAAAAGTCATCCTTGGAGGAACCTCTAACAGTCCTT	A	G	0.93	0.81	0.74	0.76	0.84	0.18	0.43	0.76	0.65	0.73	0.74	0.029	0.043	0.000	0.158	0.0002	1		
rs3750858	10	3.76	TAGCAGAATGCGTGGTCCAAGAGAAGCTCTCCACAGGTGGTGGCTATTATTATT[A/G]GCTTTTTATTCAGCCCCATCTCGGGCATTTATCTCTCTGCAAGGTGTGAGATGAACA	A	G	1.00	0.78	0.84	0.85	0.91	0.95	0.91	0.95	0.86	0.90	0.91	0.063	0.004	0.017	0.019	0.0076		1	1
rs2031573	10	3.96	ATTGTCTTCTCCTCCTCTTTTCTTTTTCCTTTTCTAGGAGCATTGTGTCTATC[A/G]TTGGCTGTGTGGGGGTTTTTGAGGTGACGGGGAGAAAAGGAGATGAAGTTGTGGA	A	G	0.00	0.15	0.18	0.21	0.20	0.00	0.06	0.09	0.11	0.25	0.22	0.067	0.019	0.005	0.070	0.0009			
rs313476	10	4.54	ACTGTCAAGAATAATTCACAGAGTATCAGGTGAGTCTGCTTAGAAGAGGGAACCTACGTTAATCTTTTCTGACATTAATAGAATT[A/G]GACCTTATTAATTCTGACATGATTTATT	A	G	0.73	0.60	0.56	0.43	0.61	0.91	0.86	0.78	0.63	0.60	0.56	0.027	0.074	0.031	0.103	0.0002	1		
rs1993181	10	4.85	CCAAGCTAGAAGGTTTCTACACGTCCCTGCACATCTGGAAGGCTGGGCAGGGAGGTGGATGAGAAACTGAGGAAT[A/G]AGGCAATGGCAATGATACTGGTCGCAGTAACAAT	A	G	0.26	0.22	0.22	0.21	0.20	0.23	0.10	0.25	0.07	0.27	0.27	0.001	0.015	0.004	0.000	0.0011			
rs4750217	10	5.71	TCCCCGCTCTGACACTGAAATCAAGAGCGATAGAGCTGTGTTTCTGATTAAACAC[A/G]GAGGATCTTCTCTCCCCAGCAAAGATCTCACTCAACTGAGAGTACCTGGATG	A	G	0.45	0.61	0.58	0.56	0.48	0.55	0.36	0.57	0.54	0.61	0.65	0.005	0.032	0.000	0.001	0.0033	1		
rs713588	10	5.89	CACTTCGTCTTTGGGTGAAAAGAGGAAATTATTATTTACCCCTAAGGCCTGGGA[A/G]GAGTTTTTATTTCAGCTTGTCTCATTTTTGGATGCTGGCAGATGGAAAAGCCAACAA	A	G	0.31	0.61	0.54	0.61	0.45	0.91	0.88	0.78	0.64	0.57	0.55	0.032	0.062	0.025	0.078	0.0000			
rs591510	10	5.95	TGTGAATAAGATGATTTGCTGTTGTTTTCCTCTAAATAAACTGGTCAGCAACATT[A/G]CAGAACCAGCACAAACCAGCACAAACCATCAAAGTGGAGGGGGTGTTTGCTTTTCT	A	G	0.90	0.61	0.60	0.62	0.70	0.23	0.11	0.44	0.55	0.59	0.64	0.070	0.133	0.009	0.069	0.0009			
rs1887027	10	6.12	GACAAGAAGTTCATCCAGTACCAGTCAGGCCAATATCCCAAAATTAGCATAACTG[A/G]CAGAAATGCAGAGGCATCAACAACTCCTCTTGACAACAGGGTTGGTAGAGAATC	A	G	0.31	0.18	0.23	0.20	0.21	0.18	0.19	0.35	0.11	0.20	0.26	0.003	0.000	0.008	0.000	0.0006			
rs2031220	10	6.39	CCATTTCTAGATAATTCAGGAGACTAGGCAATACAGACTAGCCTGCTTGCAGACA[A/G]GCTTTCTTAGTCAAGCCTAGGTCTTCACATAGGACTCTTATTCAAGGAATTAGACT	A	G	0.57	0.27	0.35	0.33	0.32	0.59	0.72	0.44	0.19	0.28	0.25	0.049	0.079	0.003	0.041	0.0031	1		
rs3793730	10	6.53	ACATGTTCAGGGCTGTTGTCACATCATTGACTTCGGCCTTTCCCAAAGTCAATA[C/G]AGAAACAAATGACAAATGACTGAATTTTAGCAACTGTGATTCAGAGATCATTTTAG	C	G	0.90	0.84	0.87	0.84	0.96	0.95	0.75	0.97	0.77	0.94	0.88	0.002	0.019	0.021	0.008	0.0001			
rs1041226	10	6.65	GGAAGCCTTTAATTAGAGACAGAGATATATTCAGCCACCGGTGTTGATCTTATC[A/G]TTCTGGGGAAAGCTCGTGGCAGTCACAAAATGTTGAAGACCCTGAGGCTGATGTGA	A	G	0.02	0.18	0.19	0.18	0.13	0.32	0.80	0.28	0.23	0.08	0.17	0.041	0.184	0.009	0.009	0.0001			
rs2439903	10	6.84	GAATTCTGAAGCTGCAAGGGAAGGACATAGGAAAGCTAAGCTGAGTACATCAGAA[A/G]AGCTGGGTCGAGTTTTCTGCAGTGTGACGATGCCTGGAGACCCAGGTTAGAGT	A	G	0.21	0.02	0.02	0.04	0.02	0.00	0.07	0.09	0.04	0.04	0.05	0.052	0.007	0.017	0.009	0.0017			
rs4747368	10	7.00	TCCTTTCTTTCCTCTCCATAGAAAAGCATAGAAAAATAAGTGCTTTGCACATTATGG[A/G]CACCTGGAGAGGCTTTTGCTTTGTGGTTTGCAAAGGAAATGTTCTGAGTTGTTGC	A	G	0.26	0.33	0.32	0.28	0.30	0.20	0.16	0.26	0.23	0.37	0.43	0.004	0.027	0.020	0.020	0.0040			1
rs942432	10	7.17	AACGTTGTTTGCTCCTACCTCGTTCCTTCCCTTTATGAGCAAATAATTCTTCTAAA[A/G]GCCAATGGGGATTCTATGTTGCAGAAACAAACATACAAAAAACCATATTCCTTGATG	A	G	0.26	0.39	0.43	0.34	0.36	0.77	0.53	0.35	0.38	0.29	0.34	0.012	0.010	0.002	0.074	0.0027			
rs1969825	10	7.23	TAGAGACTTCCCTCTCCGTGCCTTTGAAGAGGGACTCCAGGACCCACGTTGGG[A/G]CGGTTCTGCTTATGCTTTGCATTTTCGAAAAAAAAAAAAAAAAAAAAAAAAAAAA	A	G	0.43	0.93	0.87	0.87	0.98	0.77	0.83	0.92	0.82	0.92	0.92	0.134	0.004	0.001	0.012	0.0030			
rs942434	10	7.24	TGCCTGTTTTTGTTGTGAGTGATAGGATGAGGACACAACAACTGACAAAAATAC[A/G]GAGAGCTGATGGGCTTCTTCGAAGCTGCCTGCAAGCCTGTTTCACGTGACGTGTTC	A	G	0.07	0.35	0.39	0.41	0.36	0.09	0.21	0.56	0.46	0.46	0.48	0.059	0.010	0.027	0.047	0.0039			
rs1537626	10	7.66	CAACCACACTTTTACTTGCACGGTACATGGCTTAGTTCTCAGACTTCAGACCTGG[A/G]AGTAAAGGCTGCCACCACCATTTATTAGCCAACTTATCTACCTTTGCAAAGCCTCA	A	G	0.26	0.23	0.21	0.18	0.25	0.23	0.16	0.16	0.18	0.21	0.31	0.004	0.001	0.000	0.001	0.0067	1	1	1
rs3858256	10	7.77	GCAGTACCTTTGGAGGCCACAGTAGCATATTCCCTATTATTCTCCACACTTCTCCC[A/G]GTTGCACCTGGAGAAATGACTGCTCTGACACAACCTTGAAGTTAAACATTTTTG	A	G	0.60	0.21	0.23	0.20	0.27	0.45	0.46	0.31	0.29	0.22	0.23	0.089	0.030	0.011	0.038	0.0003			
rs1244459	10	7.93	GGACCAGGTCATGCCAGTCCTTCAGAAGGGAATGAAACACTTGGTAAACATTAGGG[A/G]GCGGCTGGTTATGTCTAATGTGGCTGACTCATCTCAAAGTGTCAACAGGGCCAC	A	G	0.90	0.84	0.81	0.83	0.80	0.86	0.97	0.83	0.85	0.85	0.76	0.017	0.065	0.003	0.013	0.0010			
rs2671303	10	8.08	TTCGATATTTTCTTGGTGCACATGGGAGGAGAGAGACCGAAATCTTCTCATCT[A/G]GCCGAAAGCCAGCAGTCATTCTCTGTCATGAATATTAACAGAGGAGAGACCTGTA	A	G	0.83	0.63	0.64	0.68	0.63	0.86	0.72	0.59	0.77	0.65	0.55	0.037	0.015	0.000	0.050	0.0024			
rs516769	10	8.22	AGTAAAATTTTTTTTAAAGTTTTAATGCCTATCTCCAATACTAGATTTGGAGCTTCTG[A/G]AGGGCAGGAATGATGCCATTCATCTGTCTTCAGCACACCCCAGTCACAAGTACAC	A	G	0.52	0.51	0.43	0.57	0.50	0.59	0.44	0.38	0.57	0.52	0.51	0.004	0.000	0.000	0.019	0.0054			1
rs827626	10	9.03	GCAAGGTGTACAGTGAAAGTTAGCTGAACTGGAAAGAGAGTGGACTATCAGAATA[C/G]TGCCCATTTTTCACTTTTGAGAAAGCCCAATCAGCGTCCAGGCTGCTTAGAAAGCAC	C	G	0.36	0.18	0.22	0.23	0.18	0.40	0.42	0.30	0.14	0.21	0.29	0.020	0.044	0.014	0.032	0.0100	1	1	1
rs1623807	10	9.08	GAGGAGATGTAAGATTATGGAGAAATCCAGTTGTGAGTAGAACCTTGAAAGAA[A/T]TCTAGGACATCCAGGATGATGTTGAAAAGCTATGGTCACAGGGAGCCATCCTGCCTT	A	T	0.73	0.77	0.71	0.68	0.84	0.95	0.94	0.74	0.75	0.74	0.70	0.001	0.028	0.002	0.043	0.0003			
rs1033912	10	9.60	ACAGTCCTGCACTGCTTGTTGTTCAATATCTGAAAGCCATTGTATCTATTT[A/G]CTTTCTACTTGTGTTTTGTGGGAGGATGGTGTGGTCCTTGTTATTCCACCTTGTCTA	A	G	0.79	0.43	0.45	0.44	0.48	0.27	0.69	0.43	0.42	0.45	0.36	0.065	0.027	0.001	0.025	0.0029	1		
rs913375	10	10.74	AATCATGAAAGACAGTACAAAAATTAAAGACCCATCCTTACAC[A/G]CCAACACAAAAGGAAGGGTGTCTAGAGGCAGTCTTTTCAACAACACCCACACACTGTCATCACCATCT	A	G	0.50	0.52	0.52	0.48	0.43	0.50	0.10	0.44	0.58	0.53	0.63	0.002	0.139	0.020	0.005	0.0085	1	1	1
rs725024	10	10.87	TAACACACTGTGTGACCTTTCAAGTTCAAGGATTCCCTCAAA[C/G]TGGCATACAATGCTTTCTAAACTGTAGGTGACAGAAATAACATAAGGGCTTTTGTTATCTGAGCTATGTC	C	G	0.50	0.59	0.57	0.66	0.57	0.36	0.22	0.68	0.46	0.63	0.57	0.007	0.079	0.006	0.031	0.0008			
rs1892302	10	12.45	TAAAACTCTATCATTCTAACTTTCAAAGGAAGTTATTCATCAAATATACCTCAATCTCAGCA[A/G]CCTGTACAATACATAATGAACAGTAACAAACAGCTTTCTCTCCTAG	A	G	0.36	0.32	0.27	0.34	0.29	0.23	0.33	0.42	0.23	0.24	0.30	0.008	0.008	0.000	0.000	0.0031	1		
rs1111060	10	12.58	ATGGGGACTGAGACCTGAGCTCCTTGTATCTTGTGGACTGTCCTCCTCCAG[A/G]ACCTTGGAGTCCTTAGCTGCATTTAGCAAGCAGCGGTGAACTAGAATGGCAGGTCATGTGT	A	G	0.33	0.38	0.39	0.32	0.29	0.59	0.42	0.28	0.43	0.40	0.47	0.001	0.000	0.006	0.030	0.0039			1
rs652029	10	13.07	TATACTAGAGTGTGTTGGTGCCAAAATTTTGATTCAGGGTGAAAAGCAGGAAGTCC[A/G]TCTTAGATCAGGAGCCTAAATCACATGCTTCAACTCAGCATTTACCAGAAAGCCCA	A	G	0.45	0.09	0.13	0.16	0.20	0.18	0.00	0.20	0.15	0.19	0.17	0.059	0.062	0.005	0.000	0.0011	1		
rs7899305	10	13.20	AAAAAAAAAAAAAAAAAAAAAGACCTCTGGCCCTGCTACCTTCAAAAAGAACTTAATTAA[A/G]CCTCGAAAATCTGATAGGTCTGCCGATCATGCCAAGCAGATGCGAGAACTCTT	A	G	0.29	0.28	0.30	0.25	0.21	0.45	0.65	0.22	0.08	0.36	0.35	0.000	0.073	0.002	0.018	0.0001			
rs4615920	10	13.35	TCACTCTCCTAACGTCATGGCTTCCATCTGTTTAACCCTACGTGACTGCTCCTTCC[A/G]CTGACAGCTGAAAGAATAAACCTCCGATTTAAACAAAAGTTTGCCGGGCGCGG	A	G	0.74	0.74	0.79	0.79	0.80	0.82	1.00	0.72	0.93	0.71	0.74	0.002	0.075	0.007	0.000	0.0011			
rs1890876	10	13.49	GGAAGCGAGCGATTCGCGGCTGCAGCCGCTCAGCATCGGAGAAGTGATTGTTGTCC[A/G]TCTGATGTGTTGTTTCATAGAAGCAGGGCTTAACCTTTTAAGTGAAGATTAT	A	G	0.07	0.35	0.35	0.26	0.32	0.77	0.42	0.33	0.71	0.39	0.31	0.080	0.000	0.001	0.070	0.0011			
rs13005	10	13.69	TTGAAATGCTTTAATAAGTGTTGACAACACTGTTTTGCAAATGTAAAG[A/G]TACTATACAAATCTTAATACAAAAGAATGAATAAATTAAAAGCAGATTTCTT	A	G	0.55	0.55	0.61	0.55	0.52	0.68	0.72	0.52	0.68	0.59	0.53	0.003	0.001	0.018	0.000	0.0034	1		
rs1001201	10	14.05	GTACCACTCCAGAGTTTTCATTCTCCAAGCTGGTGTGGAAAGCAGGATTGCAAAT[A/C]TCTATGCAAATGGTCAGGACTCCCAGGCGTTTCAGCATCCTATGCAAAATGGCAG	A	C	0.60	0.71	0.70	0.67	0.70	0.23	0.44	0.41	0.71	0.64	0.72	0.005	0.026	0.028	0.094	0.0001			
rs873849	10	14.51	AGAGAAGTGTTGATTCTCCTTCATCCTGCCCCAGACCACGACACAGTATTGGCAC[A/C]AGGAGGGCCCAGTGAATGAGAAGCAATGCCCAGAGGAAGATGTGAAGGTACGTG	A	C	0.14	0.40	0.39	0.39	0.46	0.32	0.62	0.41	0.25	0.35	0.39	0.065	0.012	0.039	0.008	0.0003	1		
rs2298033	10	15.65	GAAGTCTTTCTGGAATACTTACTCGAAGGTAAACGATGAAATCCTGGCACTGGTGG[A/G]AGTTTCTGCTGGCTCGGTGGTGTTGCAAAAAGGAGGGAAGCACGCTGGATGC	A	G	0.07	0.02	0.02	0.00	0.05	0.09	0.07	0.01	0.25	0.01	0.00	0.008	0.010	0.009	0.011	0.0035	1		
rs1341106	10	15.71	AATTGCTCTCAGTATTCTTGAATTCAACTTTTATTTCTTATTTCCAGTTCAGATAATAAGC[A/G]TCCCCAGACTTAACCATAACAGAAAAGTTTAATCGGCT	A	G	0.45	0.23	0.17	0.21	0.30	0.41	0.46	0.17	0.29	0.24	0.22	0.044	0.054	0.000	0.038	0.0034			
rs1341100	10	15.76	AAGAAAGGGTAAATAATCACTTTCTTCAGTGTTTATGGGTCTAGGAAAGTTTCTC[A/C]ATTTGCAGACCTCAGGCCAATACACTAAACACCACAGACCTAAACTCTGGCCTTTC	A	C	0.81	0.79	0.79	0.86	0.75	0.91	0.73	0.77	0.77	0.73	0.78	0.004	0.000	0.014	0.033	0.0002			
rs811912	10	16.86	ACGCGCCACTTCGGCCCAATCAGCGTCCCAAACCGTGGGGTGGACGCCAC[C/G]GGGATAAACTATCCTCAGCGTGGGGCGGGGTCTCGAAGCCGAATAACGAACGTGAT	C	G	0.12	0.45	0.57	0.60	0.52	0.14	0.16	0.51	0.50	0.63	0.64	0.120	0.098	0.005	0.116	0.0002	1		
rs780838	10	16.93	GATGTGGATCATTAGATATATGTTCAAGTTAGCATCGCCATGAAAT[A/G]AAAGCATTTTGTTCTTGTGGCTTGTAAATGAAAATGGGAAAATATTTCACATTGTTAATCATCAGAC	A	G	0.81	0.41	0.43	0.33	0.39	0.41	0.76	0.50	0.50	0.38	0.43	0.075	0.044	0.001	0.001	0.0000			
rs1810205	10	16.97	TCCTTTACCACACTGTGAACTCCTTGGCAGAACTCGGTGGTTTGCATGATGTG[A/G]AACCACTTGTAAATGTTTGACTAATTAATCAGGCCTATTAATCAGGTGAGAATAAA	A	G	0.36	0.62	0.60	0.70	0.64	0.59	0.24	0.59	0.42	0.65	0.60	0.030	0.073	0.006	0.001	0.0003			
rs1276709	10	17.04	GCACGGAACTAACAATGTTGAATGCTGCCCTCATTACTGTCTCTGTCCTCCACT[A/G]AAGACAATCACCATAGCAACCTTGGAAACGTAAACAAAGCAAAAATCTTTTAATTGA	A	G	0.29	0.53	0.55	0.52	0.52	0.32	0.29	0.55	0.42	0.53	0.65	0.042	0.033	0.000	0.025	0.0022			
rs359280	10	17.32	ATACTGTTGGGAATAGAACTGGTAAGGGAAGAAAAAGCTCTCTAAAAAATAGGA[A/G]AACAGGCACAGTGTGGTGGATTCATTATCACAAGGCCAGAAAGTTCACATCCTGAG	A	G	0.38	0.52	0.56	0.56	0.52	0.14	0.20	0.28	0.46	0.53	0.49	0.008	0.042	0.008	0.058	0.0013			
rs359327	10	17.40	TCTTCCTTTCCTTTCTTTTTTTGGACAAAAATCAGTCTCTTCAAATCCAGAG[A/C]TGTATGAAAGTCACTGCTGCAGATTATGAGGTATAGATCTGTATAGATGGCTGGTCAT	A	C	0.10	0.36	0.39	0.35	0.29	0.00	0.07	0.32	0.39	0.53	0.53	0.056	0.065	0.007	0.146	0.0093		1	1
rs729245	10	18.74	ACCAGGTCTGCTATCATGCTGGGATATTCTAATATCTTCTGCTAAGGT[A/G]TAGTCCAGTAAGAATCTACCAGGTATACTCATTAAAGGAATGACTAGAGTCAGCAAAAGC	A	G	0.45	0.27	0.30	0.30	0.34	0.41	0.36	0.29	0.29	0.27	0.27	0.005	0.000	0.009	0.001	0.0008	1		
rs1339048	10	19.23	CAACCAACAGAGAAAACTACATTTATTCATTATTTCCAGAGTTCAAAT[A/T]TGAAGTCTTTCAGGAGAAAACCTTCAGATTCTTCAG	A	T	0.40	0.68	0.65	0.62	0.66	0.68	0.85	0.50	0.65	0.56	0.60	0.060	0.003	0.052	0.006	0.0029	1		
rs949857	10	20.53	TAGTCTCATTCAATGATCGGTGCTTTTAGGCATGTGTATGTGCAGATATCTTA[A/C]AGGGAAAGTTTTAAGAGATCAAGGCTGCAGTGAGCTTGATCCTACTACCACTCCA	A	C	0.57	0.46	0.39	0.34	0.52	0.73	0.93	0.38	0.61	0.36	0.41	0.013	0.195	0.004	0.056	0.0009	1		
rs2038915	10	21.08	AGGGTCTTGGGCACCTGGCCTTTACCCAATTAACAAAATGGCACTGGCTACTCA[C/G]GGTATTTCCAATTAAATGATTCAGCCTGCATACTCCAGACTGTAGTCTTGCCTTG	C	G	0.31	0.54	0.56	0.65	0.50	0.23	0.49	0.29	0.68	0.46	0.50	0.038	0.001	0.058	0.060	0.0037	1		
rs893882	10	21.35	AGAGCTACCACTCCCCACAGCACA[A/T]CTGCCTGTAATACATGTGGTCACATTCCATCTCGACTCATTCTTCATATACATTAGGCAGTATCAACATTCAAAACAAAAGAGGGG	A	T	0.43	0.35	0.41	0.37	0.32	0.27	0.20	0.46	0.43	0.40	0.42	0.001	0.011	0.003	0.005	0.0002			
rs8341	10	22.83	ATCTCCCACTGTGGGAAAACCAGGCAGAGATCCAAACTCTCAATTCTCCTGTTACC[A/G]AATGCAGATCTGAATTATAAGATGTTTATGTTGACCATTGTTCAACAATGGGGT	A	G	0.14	0.37	0.36	0.41	0.50	0.41	0.06	0.21	0.43	0.29	0.35	0.030	0.067	0.000	0.004	0.0009	1		
rs927751	10	23.51	TCTTAAATGAACACAATATCAGTCTGCAACCAGGCAGCAAGGTTTGGGATGA[A/G]ATCATTGTCACAGGGTAGAGAGGCTGCTGGAATCCAACTCATGGCTCAACTGGACTAA	A	G	0.74	0.51	0.47	0.45	0.52	0.91	0.83	0.57	0.54	0.58	0.56	0.024	0.045	0.001	0.087	0.0077	1	1	1
rs1928365	10	25.79	ACAAAATCACCTCAACTACTTTTTGTAAATAAAGCAGAAAGTCATCTGTTTTGTT[A/G]AGTCTAATTAAACAATCTGGATCTGACTAAGCTGTCAGAAAATATACACTTTCACAGC	A	G	0.48	0.46	0.50	0.44	0.43	0.09	0.58	0.48	0.54	0.54	0.46	0.000	0.008	0.000	0.087	0.0013	1		
rs1339812	10	26.32	GAATTTTTAACAGGATAAACAAGACTACATCTTGT[A/G]TCTCAGAATCAGAAAGTGTTGAGCATAACACTGTGCCCAATAATCATCTGAAACTTACAGCACAAGCAGAGAATCTA	A	G	0.17	0.38	0.36	0.37	0.43	0.18	0.21	0.41	0.32	0.38	0.30	0.013	0.003	0.005	0.008	0.0000	1		
rs1521034	10	26.41	GGTTCAATTCTGCAGACACTTAAAGCAATAAAACAGCATGAGCAATCTGCAGA[A/G]ACTAGAAGGAAAATTAAATGCCCCTCAATTTTAGTCATGGAATCATAGATTCGAAGTG	A	G	0.60	0.40	0.43	0.41	0.48	0.82	0.79	0.51	0.46	0.41	0.40	0.008	0.049	0.001	0.067	0.0025			
rs1935484	10	26.46	TTTCCTTCCCTCGATTATGGATAGATTAACCAATAGAGCAAACTCTAGCAGG[A/C]GACATAGTGTCCTCCAAGGAAAAACAATGATGGAAGGCGATATACAGTGATGGGAAG	A	C	0.60	0.55	0.56	0.56	0.57	0.68	0.73	0.54	0.61	0.49	0.57	0.000	0.004	0.002	0.002	0.0002			
rs1999240	10	26.47	TTATCAAAATGCAAACAGCATGGAAAAGAAAGAAGACATCTGTAGTTACCCAG[A/C]GTGCACCGATATGCAGCCAGGAGGAAGGCAGAGGCCGTCTGAGGCATGAGACAC	A	C	0.40	0.56	0.57	0.56	0.55	0.45	0.72	0.58	0.65	0.62	0.56	0.007	0.027	0.000	0.001	0.0003			
rs994502	10	26.50	ATTGCTGAGAACCCCTGCCAATGTTTACCTTCAGATTCTGCAGCTTGTCTGG[A/G]AAGTGGGGTATTCCCTAAAAATGCAGCTACATAGATTCATTCACTCCCATTGATTCATT	A	G	0.55	0.83	0.83	0.87	0.84	0.64	0.78	0.72	0.86	0.74	0.83	0.052	0.002	0.003	0.023	0.0000			
rs638324	10	27.73	CCACAGCCTCTGTAGTCAGATGACTGCTCCTCCTCTATCCCGGTAGGAACA[A/C]TATCTTCCCTTGAAGCAGTCAAGCTAAGGATGCCTGGGTTCAGGAGCACCAGACCCAC	A	C	0.57	0.66	0.69	0.68	0.70	1.00	0.72	0.85	0.79	0.71	0.71	0.003	0.008	0.027	0.140	0.0003	1		
rs1979720	10	27.88	GCATCTCTCTGGGGAAGCAGTGATGCTTTAGATGATCTGAGTGTGGCACCAAAG[A/G]GGACAAATATGTCACCTTGAGCAAATGGCATCTACTTGGGTGAATGGTGAAGTTA	A	G	0.31	0.20	0.21	0.20	0.18	0.14	0.10	0.25	0.18	0.27	0.30	0.016	0.001	0.011	0.001	0.0105		1	1
rs1494201	10	27.89	CAGCCTTTATCGTTGCCCTTCTCACTGCAGCCTGTACACTTGCTGTACCGCTC[A/G]GGCACATCTGACCCACACACCCCATTTTACTAGGTAATCTCAGCCCTGAAAGACATTG	A	G	0.88	0.59	0.49	0.52	0.50	0.86	0.70	0.55	0.50	0.57	0.56	0.111	0.036	0.012	0.095	0.0055			
rs997750	10	27.92	CTTAATGTGCCAGTCAGGTTTTGCATCTACAGAGGTAGATAGAATTGAATAGAAGT[A/G]AAGAGCTAAAAGGCTGCCTACACAAAATTCGAAGTCTGTTGAGAGAAAGAGGTCA	A	G	0.45	0.62	0.52	0.59	0.57	0.36	0.64	0.43	0.58	0.60	0.45	0.001	0.005	0.001	0.010	0.0031			
rs997462	10	27.93	TTTCAGTCTTTCCTTCCAAATTATCTCTGTATTAATAGAAACAATCAATAGAAACACTGCTTTTTCCCACTGACTTGATTTA[A/T]GGTATAATTCTTTTCTCTCTATTCCTTTGTTTG	A	T	0.45	0.62	0.52	0.59	0.57	0.36	0.64	0.43	0.57	0.60	0.45	0.001	0.005	0.001	0.010	0.0031			
rs332188	10	28.92	TTTTGCCCCACATTTTGATGCCTTGACATTTTATTACCTTTATGAACTAAAAAA[C/G]TGATAAGCTGAAAATACTACATGGTTGGTGAGGGCTCTCACTTTTAAGATGTCAAGAG	C	G	0.74	0.54	0.55	0.49	0.59	0.50	0.81	0.59	0.29	0.63	0.53	0.016	0.028	0.000	0.006	0.0003	1		
rs788065	10	29.26	ATCTGGTGCCCTGTAAGACAAACAGAACAATGGCCTGGGCTCCCAGAATGCTAAA[A/G]TGATGGTGCCTGGCCTCTTCCTGCTACCTGGTTGACTTATTTGCTTACTCGGAGTTA	A	G	0.29	0.49	0.44	0.40	0.41	0.45	0.47	0.46	0.36	0.44	0.42	0.015	0.001	0.007	0.001	0.0001			
rs1889735	10	29.37	TGTAAGACAAATGTGCATTAGAGCTCAGGACTGCTGAACATCAAATCTTTCCAAAAA[A/G]CCATTTCTGGAAAACATAAATCCTCCCATTGGCTTTAATAATGGATGTTAACATG	A	G	0.31	0.63	0.63	0.66	0.64	0.50	0.92	0.50	0.61	0.65	0.67	0.056	0.052	0.043	0.016	0.0006			
rs950000	10	29.42	AAGCTATTTCTGCAAGGTAGCAAATGAACCATCTTGTAACCGCATCAAAAG[A/G]AAGGTGAGGCCTTCCTACAGTCAGCTGAACCTTGGCCTGACTCTGACCTGTGCTTCATG	A	G	0.50	0.45	0.48	0.39	0.48	0.55	0.53	0.55	0.39	0.51	0.51	0.002	0.003	0.005	0.006	0.0016			
rs913034	10	29.77	ACTCTTTGTTATTAATTGTTATTGTTATTGTTTGGGGAGATCCACATTATCTT[A/G]TCTTCAAGTTAAAAACAGTAATTACGCTGTTCCTGCTGACAATGTCAGTGATGAGTATT	A	G	0.38	0.56	0.56	0.55	0.39	0.75	0.90	0.69	0.75	0.52	0.44	0.026	0.074	0.001	0.015	0.0071	1	1	1
rs1328327	10	29.87	AGTGTGCAAAACAAGTTTCACAGAAATATTTCATTGCCAGAATCCTGAAATAAA[A/G]TCTCCACAGAGGAAAACTAAGAAATCCAAACTTCTTGTCTCACAATTGTTACCTAGGC	A	G	0.64	0.45	0.52	0.38	0.38	0.50	0.73	0.43	0.46	0.41	0.48	0.010	0.027	0.002	0.000	0.0011			
rs867992	10	31.39	CAAATATGTCAATCATTAACATTTTCTCTAAAAATTAACAAGTGCAATTAGCATGTA[A/T]CAATGAAATAATTGCTTGAGTTCACTATTTTTTTAAGCTGAATCTCCCTTTGAGTCAG	A	T	0.21	0.57	0.52	0.54	0.59	0.36	0.65	0.32	0.57	0.41	0.38	0.045	0.018	0.026	0.004	0.0103	1	1	1[9]
rs1272045	10	31.43	GCAAACCCAAGAAGGAAAGGCAGGGACTGGTTTAAAAATGGGCTGGATTCAG[A/G]CGGCAGATGATCAAGGGAGAGCCAGGTATTCAGTCCCAGGCCTGAGGGGCCAGAG	A	G	0.38	0.59	0.66	0.66	0.57	0.73	0.54	0.58	0.79	0.71	0.66	0.049	0.008	0.001	0.002	0.0000			
rs727345	10	31.90	ACCCTCCCTTGAAGAATAAATCCTAAAACAACTGGTGATGTTTATTGCTTCTGT[A/G]TTAGCTTGATAATCTTAAGACAATCCTTGATGCCATTTACACAACTTTTTCATTCCTCCATG	A	G	0.74	0.37	0.31	0.32	0.29	0.64	0.27	0.57	0.54	0.41	0.41	0.082	0.000	0.029	0.054	0.0066	1	1	1
rs944824	10	32.77	CAGTGGTATTCAGAAATTGGAAAACAGTGAATGCAGTACATATGGAAATAAA[C/G]TGGAAGGCCCTGTGATGACCCCAGTTTGCTGTCTAGAATTGCCAGTCCACAGTGTAG	C	G	0.19	0.48	0.44	0.51	0.50	0.09	0.21	0.35	0.54	0.45	0.46	0.037	0.051	0.000	0.082	0.0006	1		
rs1335066	10	32.98	CCCTCAGGCAATTTTTGGAGACCAATCTCTAGACACTTGTGGTTTAGTAAGTT[A/T]ACAGTCTTCCACATGCTTCACTAAAATGACCTTAACCACAGTAGTTCCCCTTAGGGCTT	A	T	0.19	0.48	0.44	0.51	0.50	0.09	0.21	0.35	0.54	0.44	0.44	0.037	0.051	0.000	0.082	0.0001			
rs1360456	10	33.51	ATATCCAGAGGTACTTTCTGAGTCATCAAGGA[A/G]GATTAAATTTATGCTCATTCAGGGTCTCCCATCGAATGCAGTTATTACTTTCTCATTCGT	A	G	0.17	0.28	0.25	0.27	0.29	0.50	0.56	0.35	0.29	0.23	0.26	0.002	0.067	0.009	0.051	0.0001	1		
rs927099	10	33.53	TTAATTGCGCTACGTCTCAGCTTATTATCTTATAAACCTCTCTGCTGATGAGT[A/G]GACTGATGAAGTTAGTAACCTCTATTTTTGACCCAGTTATGATCAATACCTGACACATA	A	G	0.19	0.43	0.50	0.49	0.52	0.36	0.43	0.51	0.50	0.45	0.53	0.061	0.008	0.003	0.020	0.0004			
rs1441027	10	34.59	TAAGAATTGGAAACTAAACCTAATTCTTAGAACAGGAAAGAAAAGATTCCCACA[A/G]AGGGAGCAGGTTCAAGTCAAGAGGCAAACACCCGCCATCTACCTGTGTCACAAATGCC	A	G	0.48	0.43	0.52	0.57	0.52	0.32	0.24	0.57	0.46	0.49	0.51	0.000	0.043	0.003	0.022	0.0000	1		
rs731544	10	35.66	TTGCCATTCTTTATCTGCTACCAAGTGCTCATGTTT[A/G]TTATTCTCATGTATGTGGTACATGGCGGTTGTCATTTTATACTCTGTTCAGATGTCACAGGCACACCTCTGCTAGTG	A	G	0.17	0.59	0.52	0.44	0.52	0.23	0.39	0.34	0.64	0.39	0.41	0.068	0.001	0.000	0.025	0.0059	1		
rs1345561	10	35.77	ATGAGGGCAAATATAAAATCCTAACCAGGATGGATGCTTTAATGTCCAAGAA[A/C]TCTAACACATAGTCCCCTAGAAAAGCAGCAGTGGAACACATCATGCATGCAGTTCCC	A	C	0.52	0.74	0.69	0.62	0.64	0.41	0.59	0.68	0.82	0.61	0.56	0.013	0.001	0.000	0.026	0.0075		1	1
rs913167	10	36.24	GATAAAAGAACGGTCCACCTTGAAGGCGGAAACGTGAGCATGGGCTTTGAGGTA[A/G]CGTCCTCTAGGCATTAAGAGAATGCCCAGTTTTCCCAGTCCCTGCATTTTGCAAGC	A	G	0.45	0.62	0.53	0.55	0.52	0.45	0.51	0.35	0.54	0.58	0.54	0.002	0.000	0.028	0.002	0.0000	1		
rs1208789	10	37.73	ATTTCCACCAATGTGAAGACAGTTAGCAGCCTTAAGGATGAAAGGATGGTGCAA[A/G]TACCATGATCCCAGTGAGTGACAGATATCAAGGCTGGTAGAGCCTGATGAAAGCACA	A	G	0.48	0.48	0.42	0.50	0.54	0.55	0.38	0.75	0.50	0.46	0.40	0.000	0.003	0.046	0.002	0.0005	1		
rs1892143	10	38.61	CCACTGCATGTTTTGGCCCATAATACCTCTTACCTGTCCTAAGGAAGTCC[A/G]CAGCCTGGTGGAGTCCTCTATGGCCCCCTGCCTCCAGCACCTTCTGTTCCCAGGCTCCGC	A	G	0.36	0.37	0.47	0.45	0.54	0.68	0.59	0.32	0.16	0.45	0.53	0.008	0.005	0.007	0.025	0.0018	1		
rs1892144	10	38.68	ATTTCCATGAAAATTACAGGAAAAAATGCGCAATGAATTGTTCTTGTACAGATCTAAAATCCTTCACCACTATTCTAAAGTCCAAACA[A/G]TCTATGAAAATTTAGTCTTTTCA	A	G	0.36	0.37	0.47	0.45	0.54	0.68	0.59	0.32	0.18	0.45	0.53	0.008	0.005	0.010	0.025	0.0018			
rs1937988	10	42.24	TTATGGCTCCAAAGTAAACTAATTCATCCATTCATTCAATACCTATTTAGTC[A/G]AGGACCCTTAGCAAACATCCTAGGGTTCTGGGGGCTATTGCTTCAGTTAT	A	G	0.50	0.46	0.35	0.35	0.29	0.23	0.31	0.48	0.54	0.34	0.27	0.012	0.001	0.001	0.017	0.0030	1		
rs1255411	10	42.25	TACATCATGGAGGTGAATCATCAGTGCCAGTCTCATGCATACCCAGAG	A	G	0.86	0.55	0.62	0.57	0.57	0.64	0.81	0.49	0.68	0.59	0.62	0.062	0.023	0.001	0.004	0.0000			
rs210284	10	42.37	GATTTTGTTGCAAATGCTTTTTCTGTACCTATTGATATGATCATATTTAATCTTT[C/G]AACTGCTAATGCATGCAATGGATTGCACTGAAATATCAAATGTTGAACAAGGCTTGCATTCC	C	G	0.12	0.42	0.36	0.38	0.29	0.41	0.16	0.43	0.29	0.40	0.40	0.064	0.035	0.000	0.000	0.0002			
rs624804	10	42.54	GCCAGAGGTTTGAAGTCAGGACTACAGCTTCCCATCTTACTCTTGGGATACAA[A/G]TCAGCTGTTTTCAGATGAGAGAGTCTGAGGTTCCAATTTTATAAGATGAGAAGGGCTG	A	G	0.45	0.59	0.60	0.54	0.64	0.50	0.38	0.55	0.61	0.60	0.56	0.018	0.032	0.001	0.005	0.0014			
rs1126487	10	43.17	TTGACCACAAAGCCTTCACCTCCCTCAGGGCCCAGCATCATGGACACTTG[A/T]CAGGGTGGGTGTCAGGTGATCTGGGTGTGGCTTTTTTGTGGCTTAAAAA	A	T	0.83	0.41	0.42	0.48	0.45	0.18	0.32	0.48	0.36	0.44	0.41	0.075	0.014	0.000	0.050	0.0007	1		
rs1009769	10	43.65	TGTCAAGGGCAGCTGTTTCAGGTTCCAGCAGAA[A/G]TCACAATGTGGAGACACCCATCAGTCACTGTTACCTGCAGACCTGTGCTCAGGACACAGGGCAAGTCGGTGTGGG	A	G	0.81	0.57	0.54	0.54	0.59	0.82	0.94	0.41	0.61	0.53	0.61	0.045	0.109	0.026	0.050	0.0037			
rs77839	10	44.12	GAAGAACTCGTGGTTCACCTGCGTCCTTCTCTGGGAACGAATATTTGGGAAAT[A/G]AGAACCTTGGCACACCTCTAACATGTAGAAACATCCACGGACTGAAAGCGGGACAC	A	G	0.38	0.65	0.62	0.54	0.57	0.82	0.58	0.78	0.39	0.58	0.61	0.020	0.000	0.040	0.046	0.0000	1		
rs1011388	10	45.04	AGAAATATAATTTGAGAATGGCAGAGAGAAGATGATGGATGTTTTAATGGAAG[A/C]ACTTATGCTACAACTGAAATTTAGTTATTCAAGATTTTGATAGGAAATGAATTAGAGAG	A	C	0.52	0.40	0.44	0.50	0.54	0.77	0.88	0.60	0.50	0.48	0.38	0.002	0.110	0.010	0.052	0.0019	1		
rs733488	10	48.04	GCAAGAGGCCAGAGTCACCCAGCTTACCTGGCAGGTACACTTAGGGCATCA[C/G]CAGGAGAACCTGACGAGGGAGGCTGTCAGGGGTGTAGGCAGGTGTCCTGCCTTCCTCGAA	C	G	0.17	0.42	0.45	0.45	0.36	0.00	0.56	0.06	0.39	0.41	0.44	0.048	0.004	0.105	0.189	0.0008	1		
rs1822861	10	49.18	CTGGGACACTCAGAATTCACATTCACACTCAGCATGTTTCTGCAACACCTA[A/G]TCCAGTTTTCCACCCTGGCTCTGGTCTGAAGGAAGGGCCAAGCAAGAAAACAAATCA	A	G	0.38	0.53	0.48	0.54	0.45	0.45	0.13	0.50	0.57	0.41	0.41	0.004	0.010	0.001	0.000	0.0016	1		
rs1917810	10	50.21	CTCTTTCCCCTTGAAAATGTCAGTTGAGCCTGCTAGGGGTCTAGTTCCTGTCTCA[A/G]AACGATTTCTAGATTATGGATCCAGTTTAAATGAATTGATCCTGAGCCATGAAATA	A	G	0.60	0.58	0.53	0.51	0.57	0.23	0.76	0.50	0.82	0.54	0.59	0.007	0.041	0.000	0.054	0.0034	1		
rs1268722	10	50.30	GGAGGGCCCAGGGCCAGGCACCCCTCCTCCAGCTGAGACAAGGGGCTACCCC[A/G]AGCCTGTGGAGGGGTGGAGCAGGTACCACTTGGCAAGCATTGGCCACGCTGCAG	A	G	0.24	0.51	0.45	0.51	0.46	0.86	0.70	0.48	0.46	0.41	0.41	0.040	0.014	0.002	0.075	0.0039			1
rs1203406	10	51.54	AATTCCCTCGCCCTTTCAGGACAGGTTCTGTGACTGCTTGTAGCCAATAGA[A/G]TAAGGCAAAAATGACAGGATGTATGTGGGTACATTACATAAGATTGTAACATCTACTCT	A	G	0.60	0.43	0.39	0.46	0.41	0.55	0.64	0.71	0.54	0.36	0.35	0.019	0.018	0.062	0.007	0.0003	1		
rs957717	10	52.14	TCTTTATTCCTCAGGCAATGAATAAATAAGTAGTTTGTTGGTAATTTCCATTAATC[A/T]TGTGAGACAGACAGAATATTATCATGCACTTCACAGTGTGTGTCAGCAATTCTGCATT	A	T	0.57	0.65	0.59	0.59	0.66	0.73	0.81	0.63	0.61	0.61	0.61	0.001	0.054	0.001	0.028	0.0024	1		
rs1912607	10	53.73	TGCAGTGAGTTGTTGGAGCAAGATATAC[A/G]GCAACTGAACAAGGAGGAGAAACTCTCTCTCTTGCAGTGTCCTTCTTCCCACATATCTTACTGACAATGGCTGACTCAC	A	G	0.29	0.39	0.41	0.37	0.45	0.27	0.50	0.57	0.46	0.42	0.41	0.009	0.008	0.032	0.009	0.0001	1		
rs1000218	10	53.79	GTGTGAAGAATAAATTTAAGGAATAGAAAAGCAAACCATCCATACACTGTTTGGGG[A/G]TAGTCATTAGAATCTTGAAATTTTGCATTAAGTTTGAAGTTAAACATTTCATTACACT	A	G	0.55	0.57	0.58	0.65	0.54	0.45	0.48	0.58	0.62	0.65	0.65	0.001	0.000	0.011	0.001	0.0030			
rs1443138	10	53.85	CAAACCTGCACTGGGAAACCAGGGCAAATCATGTCTGCTGAGGGTGGGT[C/G]GGCAGGCTCAAAGCAATCAAGAACTCTCATATCCTCCCTCATATCCTTGGCTCTTTACTT	C	G	0.26	0.54	0.60	0.56	0.64	0.82	0.76	0.52	0.54	0.58	0.68	0.099	0.002	0.008	0.012	0.0036			
rs1900423	10	55.03	TTGTCATTCTTTTTAATCATTATTCAGAACACAAGTCTCGTAGTCA[A/G]TTCTTTCCCAATCTAATTCATTTTACTCCAGGAACTTTCGTAAGTTCAATTAATCAAT	A	G	0.88	0.66	0.68	0.74	0.88	1.00	0.97	0.81	0.68	0.76	0.72	0.015	0.065	0.001	0.101	0.0001	1		
rs996320	10	55.35	ATTTCTCTAGTTTTTGACTTTACTGCCTATATTACAGAAGTACGTATGT[A/G]TTTGAAGAATTTAAGGTCCACCCATAGCTCAAATATGTCTTTATGGAAAG	A	G	0.33	0.59	0.71	0.68	0.80	0.25	0.51	0.47	0.65	0.75	0.79	0.042	0.020	0.009	0.083	0.0041			1
rs1338799	10	56.97	GGACAAAGATGTAATTTGGTCTACTGTACACTAATTTTTGGCTTCTCCAGCTT[A/G]TGATTCTTTTCTGAGGCTGAACCTCTCTATAATCAATTTGTTAGACTAAACTACTCAGAC	A	G	0.86	0.60	0.59	0.63	0.59	0.50	0.72	0.58	0.57	0.68	0.67	0.040	0.010	0.000	0.006	0.0038	1		
rs1904764	10	58.49	GATTATCATTGCTTCTTTTGAAGATGAAGGAAAGGGCCCATGAGCCAAGAAATG[A/C]GGTGGCCTCTAGGAGCTAGAAATGCATCTCAGTTTATGACCAGCAAGAAAATAGG	A	C	0.19	0.19	0.32	0.35	0.27	0.00	0.00	0.29	0.35	0.30	0.34	0.018	0.164	0.013	0.164	0.0002	1		
rs1427209	10	59.61	CGAAAGCCTCCCTTCAATTCCTTCTACCTGAGTCTCTCCATAGAGCAACACACA[A/G]CATAGCAGCTGGCTTCCATCAGAGTGAGCTGGCAAGAAAGCAAGAGAGAGTGAGTAC	A	G	0.55	0.39	0.33	0.58	0.48	0.50	0.63	0.59	0.39	0.40	0.42	0.027	0.050	0.042	0.018	0.0067	1		
rs1346300	10	59.63	AGCTTGGTGTCAGGCTCGGGTGGGTTAGAATTCTCAGTGAGAATGCAGGTGTT[C/G]CAAAGCTCTGCCTTCTACTAGCTTTGGTTATGCTTTCCTCAGTTTCTCCCTTTACAAAA	C	G	0.35	0.50	0.55	0.56	0.41	0.77	0.79	0.38	0.50	0.49	0.66	0.007	0.046	0.008	0.034	0.0071		1	1
rs1897620	10	60.24	CATGTGGATTCCCGGGAAAAAACTGCCTCAGATCATTTGTAAGAGGAAAGAATAGTAAGTGACCTCTCTAACCAAAACTG[A/G]CTAGTAGATACTACATCTTTCCCGTGGCATT	A	G	0.81	0.63	0.60	0.63	0.66	0.82	0.91	0.75	0.61	0.60	0.59	0.027	0.063	0.024	0.029	0.0004	1		
rs1999668	10	60.43	TTCCCACAGCTCTGCCCAGCTGTTCCCATTTACCCCTTTGTATGAAGGCATTCATATGATTTTGTTGTAGAGGCTTCGATGTAT[A/G]TAGACTTCTTGGCAAAAGCCTCACCATG	A	G	0.31	0.66	0.64	0.64	0.68	0.55	0.69	0.48	0.50	0.57	0.61	0.078	0.000	0.016	0.009	0.0017			
rs284642	10	60.43	TATTAATAGCATTCTCCCCAGCCCACTGCCCCAAAGAAGGTGGTATTTTAC[A/C]TCTGCTGAGACTTAGCCTGGCTTATTCTTTCACTCTGAACCAGACCTAGGTCTGGAGTGT	A	C	0.38	0.66	0.64	0.65	0.68	0.55	0.70	0.57	0.50	0.57	0.62	0.052	0.000	0.006	0.009	0.0014			
rs579142	10	60.47	TAGGGTGTTCTAGGTTGGCAATTCCACCAACATGCCCACATTCTGCTTCCAC[A/T]TGCAACATTGCTCGAACAGTCAATCCCAGTACCCTTTCTGGCTGGACTGGGACCACACCT	A	T	0.71	0.44	0.49	0.45	0.50	0.32	0.79	0.54	0.61	0.52	0.47	0.029	0.056	0.001	0.017	0.0002			
rs284604	10	60.48	TGAGTGATTCGCTCTTTCAGTGAAGAAACCCTTTTGGAAGGTGATCTTCCT[A/G]CAGGTCCCATGCTTAGGAGCCACATGATCAGAGATGAGGTGTCCAGCTGAACTCTC	A	G	0.65	0.41	0.46	0.41	0.46	0.35	0.42	0.58	0.51	0.45	0.45	0.020	0.022	0.004	0.010	0.0000			
rs1906457	10	62.28	CAGAAATAAATAACACATGATCTCTGTCCTCAGTTTAGGAGGTTAACTCTAGTT[A/C]TCACCATTATATTAAGAGCAGACTGAAATGAGTGTTTAAAAAAGATATTGTAGAAATA	A	C	0.14	0.49	0.42	0.44	0.46	0.32	0.31	0.40	0.46	0.46	0.35	0.055	0.010	0.000	0.008	0.0007	1		
rs1906455	10	62.28	AGCTATTTGCAGTATTTCAAAAAGCCTAATGCAAATGGCACACTTATCTGTGTAACTGTTATAGTA[A/G]GTTGAGCCTACATGATCCAATATCTACATTGAATCACATTCAAGCAAA	A	G	0.53	0.43	0.51	0.50	0.48	0.68	0.70	0.41	0.32	0.46	0.56	0.000	0.024	0.012	0.017	0.0000			
rs224136	10	63.82	TTTCTTCTATCACTATCATTCATCCAAGTGCCAAGTGAAGAACTTGAGTGC[A/G]ACTTCAACTCTGCCTTCTCTTTCTCTCATTTTCCACATCCAGTTGTGAACAGCCCTATTAA	A	G	0.67	0.18	0.16	0.20	0.18	0.36	0.20	0.23	0.36	0.18	0.20	0.127	0.001	0.002	0.020	0.0008	1		
rs377859	10	63.86	AAACGCCTTTGAAGATCATGGGTTTGATAAATAAACAAGATATAACAACTTTTGAGAAGTAGAATTTTTCT[A/C]AGAGCATCCCAAGTCAAGTTTAAAGTTTTTAAGAGGATGCTAC	A	C	0.88	0.63	0.65	0.54	0.64	0.59	0.34	0.60	0.69	0.63	0.62	0.030	0.064	0.012	0.007	0.0000			
rs911610	10	63.96	AAAAGTCAGGAGGTCAGGTTTGAGTCAGTCCCAGGTACCTAAATGGAAGACCAA[A/G]ATTGGGGCAGGCAAAGCAGCAAAACCACAGAAGCCAAGGACGTGCATAGGAAATA	A	G	0.12	0.41	0.42	0.32	0.45	0.14	0.15	0.28	0.46	0.40	0.33	0.078	0.094	0.034	0.080	0.0046			
rs907	10	64.28	TTTTTGAGGTAAGTATAAATACCTTTACATGGCTAACCTTCTAAC[A/G]CTTGAAAATCAATTTCAAGGGACTCTTTAATCAGTTAAATAATCTGCTTTAGAAGGCACAAATGATCA	A	G	0.53	0.24	0.16	0.22	0.18	0.32	0.72	0.22	0.14	0.15	0.26	0.106	0.237	0.034	0.050	0.0151		1	1
rs1866311	10	65.17	CTGGGTTCAGCCTCACCTCTACCAGCAGTGTGATATACTTGAAAATGGCAGGCAG[A/G]GATTTGGGAATTGATGGCAATCCTCAACTTCAGAATTACTCTTCACCTACCTGTTCTAACA	A	G	0.26	0.78	0.77	0.82	0.80	0.86	0.79	0.56	0.89	0.76	0.77	0.167	0.005	0.073	0.000	0.0000	1		
rs1544237	10	66.63	AAAAACATTACAGGACCTAATTGGGATCTTTTTTTAGCAAGTAATTAATGAA[C/G]TCATTGTGTCTGGTCTGTATGTATTTGCATTTGAAAAAAGGTATTAATAAGCAAACTTACA	C	G	0.57	0.65	0.55	0.50	0.59	0.86	0.96	0.50	0.64	0.61	0.56	0.001	0.131	0.007	0.062	0.0002	1		
rs1925622	10	68.11	GGCTAAAAACATATCCTTTTGCATCAGAGACTTTCTATGTTATGGTTTGTAC[A/G]TACTGTCTCTTCTCTTTCACTCCTCTTCATCTCTGGCTCTCTGAGAAGTAAC	A	G	0.36	0.57	0.55	0.70	0.45	0.27	0.16	0.54	0.57	0.71	0.64	0.019	0.085	0.000	0.040	0.0034	1		
rs1904645	10	68.31	TGGACTCTGCTAGTTCCCATCACCTGATAAGAACTC[A/G]CCTTTAAACATGTGAAATGGAGGATATAATTGCAATTAAAGTAAAGCCTGATAATTTAGGTTGATTATTGACATC	A	G	0.29	0.71	0.79	0.80	0.80	0.77	0.66	0.59	0.65	0.77	0.81	0.146	0.009	0.032	0.001	0.0002			
rs876489	10	69.32	ACACTTGATATTGCACAGTGCACTATTTGGCCAAAGCATGGCCTTACCA[C/G]CTGTTCTGCACTATTTTCTTTTGGGTGGTGACTGTTTTTCTTGACTTTGCATATCCCCT	C	G	0.17	0.31	0.32	0.28	0.30	0.59	0.28	0.43	0.36	0.41	0.32	0.018	0.003	0.006	0.026	0.0000	1		
rs1227938	10	70.50	GATGAAGCAGGACCCACAAGAGCAGCCTGGAGACCACGTCCAAAGGACGCCCCCC[A/G]CCCCCAATCACTGTACTCATCCACAGACAGAGTCAAGGGCAAGTGTTACATGTT	A	G	0.28	0.33	0.37	0.44	0.27	0.09	0.08	0.43	0.54	0.33	0.43	0.009	0.059	0.000	0.064	0.0014	1		
rs736594	10	70.57	GAAAATCAGGACAAGAGAAGATGCATGCGACAAGCCAACATGAATGGAAACCCTGTT[A/G]GGAAAGGAAAGGGTGGCAGCCGGGAAGGAAGGTCAGGAACGGGCTCCCAG	A	G	0.21	0.48	0.47	0.45	0.36	0.14	0.38	0.49	0.54	0.43	0.45	0.034	0.004	0.001	0.058	0.0001			
rs7463	10	71.53	CTCGCTCAAGGGGAATGCAAAGGGCTAAGAGCCCTGGCTTCAAGCAGCTGTTAT[C/G]CTAGATGAGGAAAATGCAAACAGATTCAATCTCTGGGATATTGCTGCCAACATGCT	C	G	0.88	0.53	0.53	0.56	0.64	0.36	0.60	0.75	0.46	0.61	0.62	0.090	0.007	0.052	0.006	0.0064	1	1	1
rs877783	10	72.66	GTGGAGCAGTAGTGAAAGAAGAGTTTAAGAGAAAGTTTGTTAGACTATC[A/G]TGATGGTCAGGGCTTGGGAGACTTTGCCTTGGAGCAGATGATGGTGATTTGGGGGA	A	G	0.50	0.74	0.71	0.73	0.75	0.36	0.52	0.73	0.71	0.73	0.77	0.012	0.006	0.003	0.042	0.0063	1	1	1
rs1867998	10	72.68	TGGCACATGTGGTGAATGCTATAAACGTGATTGTTACTTTATTGGGCCTGGAAT[A/G]CTGTGTGCATCTGATGACTGAACTCAGGGGCCTTTCCAGAAAATGCACATCCACGTG	A	G	0.13	0.52	0.48	0.46	0.43	0.09	0.40	0.35	0.25	0.40	0.42	0.082	0.005	0.015	0.103	0.0011			
rs2227551	10	75.01	GAGACTTGCTGGGAGCCTCAGCTGCCAGCCTTCCTCACGACAGCGAGCTCGA[A/G]ACTCATGGATCTATAGGCTTCAAGGAAGCCAACCTTGCTACTTCTCACCTCCTTAGGGCTT	A	G	0.50	0.66	0.71	0.76	0.79	0.14	0.13	0.56	0.75	0.77	0.83	0.023	0.168	0.042	0.168	0.0056			1
rs4065	10	75.02	TCTGGGCAGCCAGCACCCAGGCCTATCCCTGAGGGTAAAGCTATTGTCGTTC[A/G]CCCCTGGTGGGTGGGCAGCAGGCAAATCCATCTGTGCAGAGCCTCTCCCAGTCTCTT	A	G	0.43	0.51	0.55	0.63	0.63	0.14	0.04	0.25	0.61	0.61	0.64	0.007	0.162	0.048	0.085	0.0025			
rs1040934	10	77.41	GAGGACAATGTGTCCTGGACAGCTTAGGTAAGCCCCTGAAGATCCATGCCCC[A/G]TGTCATGTGTGTGACTTAGGATGTGGAACTGAAGACCTACAGACTTAGGACTAGCTGCT	A	G	0.36	0.74	0.86	0.85	0.86	0.15	0.24	0.31	0.79	0.75	0.83	0.122	0.208	0.175	0.270	0.0010	1		
rs1907308	10	77.64	TGAAACATGAATAGGATGGAGCAAAGTGAAAACACATTAAAAATTATGTCAGAT[A/G]CCCAAATGCAGTAAAGCTAACTGCATCTCTGACAGGTAATCAAGAATTGGGTGACTCTC	A	G	0.71	0.65	0.60	0.62	0.63	0.95	1.00	0.75	0.61	0.78	0.74	0.004	0.170	0.027	0.107	0.0086		1	1
rs1655630	10	79.75	TCCTGCAGCTCCACCAAACTGGGTCGGCTCCATCTTTCTCCTGCTTCACAAGCCC[A/G]GAGAAAAACAAAATAGCACAGGTGCATCCATCAGAGCAGAACAGCAAAGGGCTG	A	G	0.30	0.16	0.17	0.16	0.16	0.14	0.00	0.11	0.15	0.13	0.16	0.010	0.065	0.001	0.001	0.0001	1		
rs703990	10	80.28	GAGGGCTCGCGGGGTCATTCTGCAGTTCCAATGCTGCAGGCTTTCATGTGT[A/G]TTAACTATTAAGGCTCCCAGATGCCTCAGAGGACTGCAGGACTGCCCCCACCCACTC	A	G	0.07	0.17	0.17	0.29	0.14	0.23	0.69	0.50	0.14	0.18	0.16	0.008	0.176	0.067	0.005	0.0000	1		
rs720262	10	82.14	CATGATTAATATTTCCATCTAAGAAGCAATTAAGGAATTATTATCCAGGGAGATTGTGACAAAAGGAATTAAGCCATGCC[A/G]GCTCTGTTCAGGCAGAATGAGGCTTAGCTTTA	A	G	0.55	0.42	0.44	0.46	0.38	0.77	0.55	0.46	0.46	0.43	0.35	0.011	0.010	0.003	0.078	0.0033	1		
rs1336439	10	82.50	GATAAAGAATTTTTAAGTTCTTCTCTAGAGAAAAATTTAAAACCCTATTAGAG[A/G]CGGAAAATAAGACCTGATTAAATGGGGAGGCACTCCACTCAAAGCCTGGATAGGAAATCAT	A	G	0.38	0.69	0.65	0.51	0.66	0.64	0.66	0.73	0.57	0.60	0.63	0.052	0.001	0.001	0.003	0.0004	1		
rs989422	10	83.47	ATATATAATATAAAGCAAAGCAACTGCCCAGAAATGGACCAGAGTGTTATACCT[A/T]TCCTATGTTATTCCATGTGTGGAGGTGACAGGATGATTCTCCAGTGTGCAAAATACT	A	T	0.86	0.55	0.62	0.62	0.52	0.73	0.56	0.42	0.64	0.59	0.53	0.025	0.016	0.037	0.000	0.0059	1		1

rs1900500	10	118.11	CTTAACTGATCTGGCTATTTTTTCTCAGCT[A/G]TGGACACAGTCCCTTGGCAGATTCTCCTGGCTGACTGCAAGGCAGAGGAGCAGAGTAGTTGGTGTAAACACCACCTCCTCA	A	G	0.60	0.43	0.51	0.46	0.57	0.25	0.40	0.50	0.50	0.53	0.48	0.003	0.008	0.002	0.041	0.0003	1	
rs1638432	10	118.13	GAGGCCGGAGCTCCATCTGGACTGAGATGGTCCTACTGATGCTTAATGTC[A/G]TGGGGTCGGGAGCAGGTCCGGGCTCAGCTGGGAGCGTGGCCACCTCTCCAGTGGGTG	A	G	0.33	0.51	0.43	0.51	0.39	0.41	0.33	0.42	0.43	0.40	0.44	0.003	0.001	0.002	0.000	0.0001		
rs758367	10	118.15	ACCCTGGCAGTGGGATATCCCTCTCACCCTGTGACTCGCTGGGAGTAGTTCTG[A/G]GTTTTGTGTCCCAAGATAGCGCCCCTTCTGCCACCCCCACTCCTTCTGCCAGCAAAG	A	G	0.69	0.61	0.50	0.56	0.59	0.68	0.57	0.73	0.57	0.54	0.52	0.011	0.000	0.021	0.006	0.0002		
rs14240	10	118.70	ATTTTTGCCCAAATGTTTGTTAATGTAAGTTAAAAAATTATATTTGGTTTGGATTCAA[A/G]TATTTATAGATTTACAATACCTATTTCATCAAAATATATTGTATGATATTTAATAA	A	G	0.64	0.51	0.44	0.46	0.61	0.41	0.28	0.48	0.61	0.56	0.48	0.023	0.015	0.003	0.002	0.0006	1	
rs703422	10	119.49	GTCGTTCTTTCCCTTTCTTCACTTTTTCCTTTTGCTCATCATTTATGTAATCC[A/G]TACAATGAGGTACTACTTTTTTTTTAGGGTATTGACTAAGGATTCAGCTTTAAAAAATGTAT	A	G	0.81	0.38	0.40	0.49	0.34	0.73	0.83	0.49	0.23	0.47	0.50	0.087	0.122	0.007	0.073	0.0052	1	
rs853617	10	119.61	TACAGAGGCACATGTCATCACATATCTTGGTACATACATTAAACTGGGATGTAAT[A/G]GTACATTCCAAAGATTTGAAGAACTGCCCTAAATCCCTTTTTTAAGTGACATTTAAAA	A	G	0.31	0.59	0.60	0.50	0.66	0.27	0.18	0.51	0.69	0.57	0.52	0.038	0.103	0.004	0.064	0.0027		
rs915272	10	120.56	TGGCAACAGTCACTAAGTGGGACAGGAGTCTGACTGC[A/G]TGATTTCTGTTGTCCTGGAGACAAGCAAAGCAAAATTTTGACAATCTAGTTCCCCATGCTCAGCAACAACCTCC	A	G	0.29	0.43	0.41	0.32	0.41	0.36	0.22	0.31	0.50	0.37	0.33	0.006	0.018	0.003	0.000	0.0043	1	
rs1467813	10	120.95	TACAAAGAGGAAGGA[A/G]GTTCACCATGTGTCAGGCATGGGTCTAGACCCTCGGAGACAGTGGGGAAGAAGAGGCCCCCCACTCCCAGATTCCAGAAGACAAACCACACAGA	A	G	0.45	0.24	0.31	0.32	0.21	0.09	0.20	0.27	0.38	0.36	0.30	0.012	0.006	0.000	0.033	0.0004		
rs1041517	10	121.57	ACTTCTGATTCCAAAACGACTGTGTCAACGTGAAACCTTATGTTCCGAAGTTTACC[A/G]CATTCCCCAGGCAAAACACTGCACTGACTTCTACAGCCTGTAATGTCAAATCAGCA	A	G	0.66	0.78	0.76	0.76	0.68	0.86	0.97	0.60	0.82	0.76	0.72	0.006	0.056	0.015	0.008	0.0004	1	
rs934321	10	122.38	CTTGTAGCCACATTGCACTTTTCTTTCTTGGTAACCATCAAAATATTTTGCATTGT[A/G]TCAGGCACATGATGGAAACAAAGTAAATATCTGAGTGACTGATTGATATAGCTAAGCCAC	A	G	0.33	0.18	0.23	0.23	0.30	0.23	0.25	0.43	0.29	0.27	0.30	0.002	0.000	0.019	0.000	0.0027	1	
rs1649183	10	123.04	CCGGAAGAAAAACTGGTGGTGCAGGGCTTATGGGAAACAGCACATAGTCTCCAAGC[A/G]TCTCTCCAGAGAGTTCCAAGGGGAATTCTGACTAAACAAGGTTTTTCGCCTTCAC	A	G	0.67	1.00	1.00	1.00	1.00	1.00	0.99	1.00	1.00	1.00	1.00	0.132	0.004	0.000	0.000	0.0000	1	
rs1696837	10	123.09	GATTGACAAGCTGCAGACTAGTCAGGTTGCAGCACGCTATGAAGCTGTGAAATTG[A/G]TGTCATCTACAATGTATCTCTCCAAGTTTTCAAGTGAAACCTCTTGCTACATGCCT	A	G	0.38	0.69	0.69	0.66	0.70	0.55	0.45	0.51	0.71	0.54	0.65	0.64	0.056	0.044	0.002	0.046	0.0006
rs2901297	10	123.19	CAAGATACACTTTAAAATAGATTGTAGAGTGAGTGGGAGATGAGGGAATGGCCATTA[A/G]TGTAAATGCTCTTTAGAGAAGGAAAGAAAAATAGAATTAAGATAACTGGAGGATGA	A	G	0.38	0.13	0.17	0.10	0.14	0.41	0.28	0.16	0.19	0.11	0.08	0.032	0.017	0.000	0.048	0.0103		1
rs911788	10	123.61	GCAGGACCCTGAGAACTATCAATTTTTGGATGCGTGAAATAGACCAAACATGAAACCT[A/G]CTCTGGACGCAGGTTCTCTCTGAATCAGGCAAAGCTTCAGGCCTCTGAAGGC	A	G	0.74	0.65	0.59	0.65	0.57	0.77	0.64	0.63	0.65	0.57	0.65	0.008	0.001	0.001	0.017	0.0007	1	
rs11190	10	124.29	ATGAACAAGTGCTTCCTCACAACTTGAAGCTTCTAGCAGAAGAACTTAAC[A/C]AGCTCAAAGCAGAGTTTTTGGAAGACCTAAAACAAGGAAACAAAAAATAT	A	C	0.93	0.57	0.61	0.61	0.66	0.91	0.79	0.78	0.57	0.69	0.64	0.046	0.001	0.009	0.029	0.0002	1	
rs1571423	10	124.79	AACACAAACCTTGCAATAATTTCAGCCACTAAACAGTGAAGCAGATTAGGCGTGA[A/G]TTTTTTACAGAAAGAACAAGCTTGTATTTTCAATAATTATCCCAAAGTGAAGAAAATG	A	G	0.10	0.26	0.21	0.20	0.18	0.41	0.45	0.14	0.39	0.27	0.23	0.017	0.010	0.004	0.010	0.0000	1	
rs870577	10	125.20	CCTGTCACTTCTGCTATTCATGAATCTCCTTTTCTGGCCAAGTTCAGTATCAC[A/C]CTCTTGTGCATTCACACATCCAGCACTCCTACCTTCTCATCAGGCATTCCCCTCTGCCA	A	C	0.14	0.35	0.34	0.35	0.20	0.59	0.26	0.40	0.54	0.26	0.32	0.023	0.006	0.000	0.029	0.0002		
rs10899	10	125.38	GATCCTCCTACCCCCGAAGCCTGGTCTGTCTCACACATACAAGTTAGAGCTGGGT[A/G]GACGGGTGCTTGCACTTTCTGGCTTTGGTGGGCGAGACCTTGGCCTGAGAACTT	A	G	0.60	0.48	0.55	0.52	0.45	0.41	0.73	0.53	0.57	0.68	0.63	0.001	0.020	0.001	0.012	0.0007		
rs876352	10	125.91	CGGCTTCCCACAATTGCTAATCCCGGCCAGACCCCTTAGCAGCAAAAGGAGCCCC[A/G]AACAGGCGGGACAGGTGGACTAGCTCTGAGTTCTGTTTCATCTGTAGGACCTTG	A	G	0.07	0.29	0.27	0.23	0.25	0.27	0.41	0.35	0.39	0.32	0.28	0.026	0.009	0.013	0.000	0.0002	1	
rs4962424	10	126.30	CAAAAATTAACTGATTAATTCTCTTCCCCCCTTGGATGACGCAGCTGATGTGGAA[A/T]TCTGTTTTTACAACAGAGTCAAGGAGGGGCCAGACTGCAGTTTGACCATCAGAGCTT	A	T	0.69	0.65	0.71	0.63	0.68	0.73	0.66	0.76	0.79	0.68	0.71	0.001	0.000	0.009	0.006	0.0000		
rs1152690	10	126.37	TGGTAGAATAGTTGCAGCTGTGGTTTTGCAAAGATAGGACTGTTTCCGGCTGCC[A/G]TGCATGCTGTATTATGTATTTGACAGATCTCCAGGATGATGTAGAAGCTTGGCCTG	A	G	0.64	0.92	0.94	0.91	0.95	0.82	0.57	0.82	0.86	0.92	0.89	0.066	0.080	0.007	0.010	0.0032		
rs7919596	10	126.47	GTGCAGCAGCCTCCAACAGGTCTGGAGTCAAGACCTGCATGTGCCACTGGACTTG[A/G]GGTACCATGGGAAAATTGCTTATACTCTCTCAGCCTCCATATTTGTTTTTGTAAAA	A	G	0.55	0.66	0.69	0.63	0.59	0.82	0.93	0.89	0.82	0.76	0.70	0.004	0.057	0.030	0.021	0.0001		
rs1278305	10	127.39	TACAGTTAAGTGTTCAGCTTATTTAGATCCGATGTAAAATAGAAAATGTTGCA[A/G]TACAAAATGAAATGTTACTGCCATTTTCTTTTTAGGGCAGAATTGATGGTATTCATTCCA	A	G	0.45	0.39	0.36	0.27	0.41	0.27	0.52	0.44	0.36	0.43	0.47	0.008	0.018	0.006	0.002	0.0022	1	
rs728104	10	127.56	CAGCTTTTTTTTTTTCCCGTTGAAACATTAGAAAAATCAGCCAGTCATGAAGCCCAGAC[A/G]TACTTATGAAAACTTGGTCCTCTATGTAGAAGATGCTCGACGCCAAATCTCCA	A	G	0.38	0.14	0.14	0.15	0.20	0.14	0.28	0.17	0.18	0.27	0.19	0.043	0.021	0.001	0.000	0.0018		
rs873957	10	127.58	AAATGAGACACACTCAAATCAATCAAGAGATACGACATGGACATTTTCAGGAAAA[A/G]AAGATTTGTCTGAGAGCCCATTGTCTATATAAAATTTTCAATACTCTATTTCAAACCT	A	G	0.43	0.65	0.65	0.63	0.64	0.68	0.45	0.72	0.64	0.59	0.67	0.030	0.029	0.000	0.000	0.0013		
rs1936875	10	128.93	TTAAAATTGCAATCCACACCTCTCACCACCCCTCCTCTCTTCTACTTAAAAAA[A/T]TAGTACTTAGCATCTTCTAACATACTTCAATTTACTCATTATATTTATAGTTCATTTTAAG	A	T	0.48	0.41	0.36	0.37	0.32	0.68	0.32	0.65	0.50	0.39	0.45	0.002	0.027	0.029	0.025	0.0032	1	
rs1926143	10	129.05	TTGTCTAATGACATGGCTCTCAGAATGTGTTTCAATCATTAAGGTACACATGAC[A/G]TGTACTTAAAAATGTCTCTGGCAGTTTCTGATGCAAATACAGGGAGGTTCAACTTTTCTC	A	G	0.71	0.70	0.63	0.62	0.63	0.64	0.61	0.50	0.75	0.61	0.71	0.001	0.005	0.011	0.000	0.0039		
rs1255135	10	129.59	AAGACTGGAAATTAGGATAAAGCAGATTTCAACATGAAAATGCTGGAGACACTGTCA[A/C]AGTCATCACCCTCAGGTAGTGGTCACACATATTCCCAGCTTCCCAAATCC	A	C	0.38	0.58	0.60	0.54	0.45	0.50	0.32	0.41	0.36	0.56	0.55	0.018	0.032	0.011	0.001	0.0008	1	
rs765934	10	130.22	GGGATGACAGGAAGCCCACCTTGACTGCCCCCAGACTGAACTGCCTGCGTC[A/G]AGATAAGAAATGACTCAATGTCCAGTGACTCAGATGGTCGTGGGAAAGCAGCGTAT	A	G	0.67	0.58	0.65	0.60	0.52	0.50	0.72	0.57	0.64	0.70	0.61	0.000	0.001	0.002	0.014	0.0017	1	
rs1869251	10	130.29	CTCAGTATGCTGCCTACAATTGTTCAAGTTCACAGTTACTTGTAAATATTCT[A/G]TGCAAGTCTCCCCAAGTGTAGCAGCAGCCTAGATTTTTGTAATTCCCCTAGTCTTTTAGA	A	G	0.52	0.53	0.48	0.59	0.63	0.59	0.66	0.71	0.57	0.50	0.62	0.002	0.021	0.032	0.010	0.0069		1
rs1998825	10	130.48	CATTTTAGTGCCTGGTAGTCTGTTAAAT[C/G]GACATTTGAAATTAACCCCACCCCTCAACTGCAAAAGAACCCCCAAAACAATAGGGGGAAGGGCAGGCACTCAGAGGGACTCA	C	G	0.57	0.72	0.68	0.66	0.68	0.95	0.65	0.64	0.68	0.68	0.65	0.008	0.000	0.003	0.062	0.0014		
rs570967	10	130.90	GCACCCTACACTACGCAGAGTAATCAGCCACTAAGGAGTAGTGAAGGATCTGACA[A/C]CCAGAGAGCTCGGTAAGGACGATGCCATCCGGCTGGCTCCTCTGCAGGTGGCCA	A	C	0.60	0.42	0.41	0.51	0.57	0.18	0.63	0.40	0.39	0.47	0.46	0.021	0.038	0.002	0.021	0.0010	1	
rs6482747	10	130.97	AGCAGGCCCTGTCTGTAGGCATGCCTTCTCATCTACTGGAACATACCGTAAGACC[A/G]CGAGGACAAAGCCACTCCTGGGAAATGGAAGCCAGTGGTCACCCCGAATCATG	A	G	0.57	0.62	0.66	0.65	0.66	0.64	0.20	0.73	0.71	0.63	0.61	0.003	0.113	0.001	0.001	0.0007		
rs1980849	10	131.01	ACCATGGCGGGCACTCTGGAATGCTCTGTTTTGGCAAACTTTCTTCCCCAAGAAC[A/G]GATTTGACTTTTGAAAAGTGGTCTGAAAGTCAGCCTCCCCAGCCCTGGTCACAGCA	A	G	0.12	0.49	0.46	0.39	0.45	0.41	0.19	0.44	0.38	0.46	0.49	0.074	0.045	0.002	0.001	0.0002		
rs1536087	10	131.39	CATCCCCTGGCCTCGAGCAGGCACCCACTCTGGGCCCTGCCTGTGCCGGGCGCC[A/G]GGACACACAGATAAGCACACACAGGGCACCCGAGCTCCCTCAGCAAGTCCAGG	A	G	0.50	0.52	0.38	0.39	0.45	0.68	0.57	0.46	0.46	0.38	0.45	0.013	0.036	0.012	0.075	0.0041		
rs1703711	10	132.12	CGCGAGGCCCTGGTGGCAGGTGTCCCTGGTCTTGGAGCCAGCACTGGGATCAAA[A/C]CTCCTTCTCCAGCCTGGGATTCCTGAGCTAGTTGTGTCACTTGGTACAGATGCCC	A	C	0.67	0.55	0.56	0.52	0.63	0.59	0.45	0.72	0.46	0.62	0.58	0.007	0.008	0.014	0.000	0.0001	1	
rs1036268	10	132.16	ATGAGAAGTAATATCTCAGGCTAGAATACTCATAAGTAGAAACTGAGTTTACACTG[A/C]GCAGCAATACTTTTTATTGCCTCTACTTTCTCCTTTGGTTTTCTGCAAAAGTTATGA	A	C	0.52	0.60	0.65	0.68	0.68	0.18	0.28	0.67	0.61	0.72	0.69	0.003	0.039	0.003	0.090	0.0040		
rs893509	10	132.49	TGTCGGGCTTGGGGTGAGCCTTGGATAACTTATAGTCTGGAGGCTTTCAAAT[A/G]AAGGGAAATCATTGGCAGGTAAGGACCAGCACTGCTTTCACATAAAGTGGCATTGAGT	A	G	0.33	0.56	0.56	0.55	0.70	0.45	0.55	0.66	0.54	0.54	0.49	0.020	0.001	0.009	0.003	0.0029		
rs754575	10	132.51	AACACGAAGACTTGCAACCCATTAGGTGTTACCGGGTTCAAGGCCCTCGCTGCCT[A/G]CAGGGAAATATTTAGGAGAGAAGCGATTGGACTGGCTGAACCATGGAGTTGACA	A	G	0.43	0.79	0.71	0.70	0.73	0.73	0.79	0.81	0.79	0.70	0.69	0.030	0.006	0.012	0.002	0.0000		
rs1530064	10	132.52	TGACAGCCAGGACTCGGTTCACAACCCATGTGTGTCTCCTCAGGATTCCCTG[A/C]GTTCGTGGAATGAGATTCCCCAAGTAGGATATACCGGCCACCGTTCCTGTGGAGGG	A	C	0.21	0.35	0.29	0.28	0.39	0.50	0.45	0.52	0.39	0.34	0.34	0.001	0.022	0.072	0.040	0.0031		
rs7894	10	133.20	ATATTCCAGGACAAAATCAACTAGAGACGCGAACGTGA[C/G]GACCAAGTCTTGTCTTGCATAGTTAAGCCGGACATTTTTCTGTCAGAGAAAAGGCATCATTGTCCGCTCCATT	C	G	0.19	0.32	0.24	0.20	0.32	0.55	0.30	0.21	0.15	0.28	0.26	0.002	0.001	0.002	0.046	0.0011	1	
rs1888120	10	133.30	TGCCTTGTATGGGAATGATCAGGGTGGAGCTAATGTAGGCTGTTACCATAATCAAC[A/C]CCCCCCAAATCTCCTTCCTAAATTATGTAAAATAATTTTACAACCTCAAGAAATC	A	C	0.55	0.58	0.55	0.54	0.43	0.82	0.94	0.72	0.39	0.45	0.56	0.000	0.088	0.004	0.036	0.0000		
rs954042	10	133.57	GGCTAATTCTTGAGGGAGTGAGTGGCTTCCCGCTCTCTCCGGACAGCATTAA[C/G]TGCCACAGGTTGGGTTATTATAACATGAGGCCACCCCTCATGTTTTGTCCCACTCCCC	C	G	0.81	0.68	0.69	0.73	0.66	0.45	0.96	0.84	0.69	0.69	0.62	0.018	0.077	0.022	0.019	0.0041		
rs3793670	10	133.97	AATATGAGGAAGAATTTTTCTTGAGTACAGCTGTACCTGGCTAAAAACATACACA[A/C]ACGTATGCAGCCACTGTTACCCTCTCACTTTGAAACGTCCGAGTGGAGATCATATG	A	C	0.05	0.23	0.26	0.23	0.23	0.45	0.09	0.21	0.18	0.20	0.33	0.031	0.002	0.003	0.063	0.0043		
rs761774	10	134.40	GTTATCGAATGCCGAGGAGCCCAGGATGCACTTCCGAGGCTCACTGGTGACTTTCC[A/G]GAGATACTTAGGCAAATGGACATAAATAGCTCTTGGATCCTAGCAGGAATTCTCA	A	G	0.17	0.13	0.14	0.19	0.09	0.45	0.47	0.34	0.18	0.18	0.17	0.000	0.048	0.020	0.057	0.0005	1	
rs880340	10	134.53	AGATCTGCCCTGCCCAGCCCCCAGCATGGCTGTCTTCACCTGTTACCCGTGGGC[A/G]CGAGCTTGCCCCATGGGGTGGCATGTTGAGCCAAATGCCCTTACCCTCTATGTCA	A	G	0.33	0.60	0.52	0.43	0.45	0.64	0.41	0.73	0.50	0.38	0.35	0.018	0.000	0.048	0.011	0.0153		1
rs741737	11	0.28	AAGGGGACTTCAGGGAACCCCGGCCCCCGCCGCGCCTGCCCCTTCTCCCTTATTGGGCGTTTTGCCGGGAAGGCTC[A/G]TGGACGTCTCACTCGCACGGGTGTTTGGCGT	A	G	0.98	0.74	0.75	0.75	0.70	1.00	0.96	0.83	0.68	0.80	0.80	0.037	0.035	0.000	0.065	0.0000	1	
rs7481525	11	0.37	GAATTCCTGCTCTGCTCGCAACCTTCCATCATGCACACAAGAAACAGTCACATGC[A/G]CTGTGCCTCCATCCTCTGCCCCTGCCTTTGCTCTGCTCGCAACCTTCCATCACA	A	G	0.33	0.61	0.61	0.52	0.63	0.55	0.51	0.61	0.61	0.62	0.62	0.024	0.002	0.002	0.000	0.0007		
rs885454	11	1.21	TGTAGATGACGCTCGTCAAGGAGGTTTACCACATAGCCCCCCGGAGCCCACCC[A/G]ACACCAGCCGGAGGTGCTAGGCTTCTGCGGCTCCCACCTGGGGCAGGCGGAGGA	A	G	0.05	0.08	0.07	0.06	0.09	0.22	0.50	0.15	0.21	0.09	0.08	0.000	0.143	0.018	0.027	0.0008	1	
rs1105350	11	1.47	CAAATTTGTGTCTAGCAGGGATTGCCTCACTTCCCAGTAGGGATCAGTTAC[A/G]TGCTGGGTGCCAAGGAGGCCGACTGGCACGTCATGGGTACCACTGGCGAGAATGGGA	A	G	0.64	0.60	0.60	0.52	0.50	0.45	0.57	0.53	0.68	0.60	0.48	0.008	0.003	0.001	0.003	0.0049		
rs2029463	11	1.63	TGCGAGATGAAAGCTGCCCACCGCTGGCACAGGACTAAGGGCTGTGCACAGCAC[A/G]GGAAGACCCCTCCCTTTAAGAGTTTCTCCCGATGCCCTCTTCCCCCTGCAGCCCTC	A	G	0.12	0.13	0.14	0.17	0.14	0.41	0.28	0.11	0.11	0.05	0.13	0.001	0.050	0.010	0.100	0.0008		
rs1004446	11	2.13	AAGGAAAACCTATTTCCTTGCAAAAGCCTCAGTGGCTTTGTGCAGCCCTAAAG[A/G]AGAACCCTCTCCACAATGGCCTTTCCTGCACACCCCATCCCTGAAGTTGAGGCTG	A	G	0.52	0.41	0.37	0.33	0.36	0.23	0.16	0.25	0.38	0.29	0.22	0.017	0.024	0.005	0.013	0.0103	1	1
rs451041	11	3.03	ATAGAAACACCACCCTCCATCTCCCACCTGCCAAGTAGTGCCCAAGGCCTAAC[A/G]TGATCTTTCTGACGCTTAATGAAATGAGCCCTGGGGGCCCCAGCTTCCCCTGCAAGC	A	G	0.62	0.53	0.52	0.54	0.61	0.32	0.13	0.48	0.68	0.45	0.55	0.013	0.081	0.000	0.015	0.0016	1	
rs956453	11	3.37	TTCTCCATCAGTCCCTACTTCTGATCCCAGTCCCAGTGAGGTATCCTCCAGATTG[A/G]CAAATTATATGCTAATCTCTGCAGAGATGCACCTTGCTTCTCATAAAGGCAGACTT	A	G	0.36	0.64	0.63	0.66	0.55	1.00	0.95	0.70	0.71	0.64	0.63	0.047	0.105	0.000	0.146	0.0001		
rs879114	11	3.61	TAGCACATTCTCAATATATGTCTGAATAGTGCGTGACTGAATCTGAGAAAGG[A/G]GAGTCACATGTGGGTGGAGCAGTCTAGGAAGGGCTCACAGGAGGGAGACACCCAG	A	G	0.26	0.18	0.18	0.22	0.18	0.18	0.10	0.29	0.21	0.26	0.19	0.014	0.004	0.020	0.000	0.0003	1	
rs1451724	11	3.82	CCAGCATTGACCGTACTTTTCATTGTGCTGAACTAACTGGATGCTTCAGGGG[A/G]AGGGTAGTCTGACCTTCTGTTTATTCCAGAGCCCAGGGAAGGGCTGAGCAAAGCT	A	G	0.15	0.47	0.48	0.44	0.52	0.50	0.54	0.48	0.36	0.39	0.41	0.059	0.003	0.003	0.001	0.0012		
rs1609812	11	5.21	ATATGAAACCTCTTACATCAGTTACAATTTATATGCAGAAATATTTATATGCAGA[A/G]ATATTGCTATTGCCTTAACCCAGAAATTATCACTGTTATTCTTTAGAATGGTGCAAAGA	A	G	0.86	0.79	0.86	0.84	0.70	0.55	0.53	0.59	0.57	0.84	0.88	0.001	0.064	0.036	0.057	0.0012	1	
rs892336	11	5.57	GGCAATGCCCATGTGCTCACAAAATGTGTGTGCAATGAT[A/G]TTGATCTGGCAATAGTGCAGGTGCTCAAGGAGAAGATGGATGGAAACATAATGATGAAGCTGCGGCTCAG	A	G	0.19	0.55	0.51	0.56	0.54	0.00	0.17	0.21	0.43	0.52	0.48	0.072	0.080	0.039	0.237	0.0012		
rs906895	11	6.24	GGAAAAGGGCTCCCGGGAGGAAGCTTCTGACTTGAGTGTTGAGGGCAGTTGGG[A/G]TGTGGTGGCAAGCACTGACAAGGATAAACCAGAGTAAGGTGAGGATGGGATATAAT	A	G	0.21	0.48	0.58	0.50	0.52	0.73	0.77	0.61	0.57	0.59	0.53	0.088	0.022	0.000	0.006	0.0026	1	
rs960647	11	7.26	TGCTCATGTCTTACTTTCCCTGTTGTCCTATTAAGTTCAAAAGTCGACTTAACAAAA[A/T]CTAAACAGAAAAAAGAGAATGGGGGAATAGGAACAAGAGAAGATTACATCCATTTT	A	T	0.12	0.28	0.33	0.29	0.23	0.32	0.41	0.47	0.25	0.38	0.37	0.033	0.005	0.001	0.000	0.0006	1	
rs2200650	11	7.29	TATTTGGTTCTACTCTGCATGTAACTTTTCTGGTCTGAGTTCCTCCAATATATGGT[A/G]GTAGTACTGTGATCTTGCTAGATTTGATCTGGGGACTCTGCCATAATTCTAACAAAC	A	G	0.17	0.34	0.28	0.40	0.38	0.50	0.57	0.43	0.39	0.34	0.30	0.016	0.039	0.032	0.024	0.0001		
rs905290	11	8.72	GCCAACTCCTTAGGCAGCTCCACCCTCCAGCAGTGGCATCACATCAATAATTGGC[A/G]TCTACCATGTCCCAGCTCCAAATCCATATGCAGATCTTGGCAACTACAAGGAACA	A	G	0.90	0.55	0.54	0.62	0.59	0.59	0.46	0.67	0.50	0.69	0.66	0.052	0.013	0.000	0.001	0.0052	1	
rs10279	11	8.93	CCAGCCTCATCAAAGAGCTGACTTACTCATTTGACTTTTGCACTGACTGT[A/G]TTATCTGGGTATCTGCTGTGTCTGCACTTCATGGTAAACGGGATCTAAAA	A	G	0.76	0.77	0.81	0.76	0.82	0.68	0.67	0.72	0.68	0.75	0.76	0.001	0.008	0.000	0.006	0.0034		
rs1525064	11	62.74	CAATGGTGATCTTGCAAGATTGGGTGTCTGGCACGC[A/G]GGCACATCAGCACAGTATCAACAAGCAATTTATCCATAGTGTGCAAGTCCCTCCCCTGGTTCCTCATAGGCTG	A	G	0.76	0.42	0.45	0.32	0.39	0.36	0.48	0.41	0.25	0.45	0.35	0.062	0.000	0.002	0.004	0.0048	1	
rs630759	11	62.95	GTTGAAGGTATTTTACAAATATGCAGAGATCCAAACACCAGATTTTCATCCCTGGG[A/G]AACCCTCAAACAAGTGGGTACCTGTAAGCTGTCTGAGATTGCCAGTGCTTACAGG	A	G	0.24	0.77	0.72	0.77	0.89	0.09	0.22	0.46	0.61	0.75	0.63	0.107	0.106	0.038	0.206	0.0076		1
rs626333	11	63.37	TTCTCAGTTTCTTCTTGCCGCTCACTCTCAGCTCCCAGAGTTGGAACACGTC[A/G]TATCATTGTCTGCCTTTCCCTTTCTAGAATGTAAGGGCCTGCAGGTTAGGGTGTTTGCGTG	A	G	0.21	0.59	0.55	0.45	0.46	0.59	0.85	0.28	0.46	0.49	0.57	0.065	0.057	0.027	0.000	0.0005		
rs728919	11	63.48	GGTCTCATGTGAGAAACACATCTGTAAAGTATAAGATTGAGGCCCTAAAGGAA[A/G]CAAGGATCGCAGAATCTCTGGGGCATATTTCTTTTTTCCTTCACCCTGTCTTATGGTGT	A	G	0.24	0.60	0.55	0.50	0.48	0.82	0.96	0.59	0.54	0.59	0.60	0.062	0.108	0.001	0.036	0.0021		
rs562865	11	63.55	GCAGGCTCGGCTCCTGCAGTTTGAGCGGGGTCGCTAACCTCAGGAAGGCCCC[A/G]GACAGAATGTGGGGAGGTTGGGGATGGGGTTCCATCCTCTCCTGCCCGCCTGCCAG	A	G	0.21	0.59	0.55	0.50	0.48	0.82	0.96	0.50	0.54	0.59	0.60	0.065	0.119	0.001	0.043	0.0024		
rs477138	11	64.24	GTGGCCTCAGACTCAGAGGTGGGAAACCAGTGCCACCCTAGAGACATG[C/G]GGCAAAGAGGGCAATGTGAACCATCTGCCACGGGGTCCAGGCCTTTGGCCCCTGGC	C	G	0.10	0.29	0.21	0.30	0.30	0.18	0.15	0.18	0.25	0.23	0.30	0.026	0.008	0.005	0.008	0.0031	1	
rs931127	11	65.18	AGAGCGCGCTAGTGGAATGGTTGCCCAGGGCCCAGT[A/G]CTTAGCAAGTGCTCCATAAAATAGTGGCAGACTCTTGAGCACCTGCTGGCCGGGAGCTGGACGCTAGGTGGC	A	G	0.38	0.53	0.57	0.61	0.55	0.50	0.19	0.65	0.64	0.57	0.56	0.014	0.068	0.000	0.000	0.0000	1	
rs1671063	11	66.05	AGTCCACAGGGGATGTGGCCGGAGGCGGGCCCTGTACGAGGGGTATGCAAC[A/G]GTCACTGATGCGCCCCCGAGTGCTTCCTCACCCTCAGGGACACGGTGCTGCTGCG	A	G	0.14	0.42	0.42	0.41	0.23	0.50	0.85	0.65	0.25	0.47	0.37	0.046	0.116	0.015	0.004	0.0020	1	
rs1530891	11	66.49	ATGGGCTCATGGTGGGAATGGCCGTAACGTGGAAGAACTCCACCCTCCACGA[A/G]TTTCACCCCCTTGTTCTCTCTCTGCTTGCTCTGTCCTCTTATTCTTGGCTGCTAGAGTT	A	G	0.15	0.40	0.46	0.46	0.36	0.45	0.79	0.69	0.36	0.55	0.48	0.073	0.054	0.046	0.000	0.0000		
rs614080	11	67.12	CCCCAGCGCTGCTTCCCAAGGTTCGGAAGGGGAAACTCTGACCTCAAGCTCT[A/G]TTTGAAGAGCGATAGCAAAGGGGAGAGGTTGGCTGTGGTTTGGTGGTGCTTATCTCC	A	G	0.18	0.48	0.53	0.48	0.55	0.73	0.23	0.68	0.50	0.52	0.49	0.061	0.014	0.009	0.003	0.0002	1	
rs947894	11	67.13	AGCCCTGGTGGACATGGTGAATGACGGCGTGGAGGACCTCCGCTGCAAATAC[A/G]TCTCCCTCATCTACACCAACTATGTGAGCATCTGCACCAGGGTTGGGCACTGGGGG	A	G	0.57	0.67	0.70	0.67	0.59	0.86	0.43	0.73	0.65	0.72	0.65	0.008	0.036	0.000	0.017	0.0003		
rs1531514	11	67.17	AATGTTAGGGGGAAGGGAGAGGTAAGGCAGGGTTTTCGGGAGTTACCCCAAAGCAAGAGGGCACAGTATTC[A/T]GGCTGCTGGGGTTGGGAGATAGAAGTCCTGGTTCCA	A	T	0.79	0.41	0.38	0.50	0.46	0.27	0.77	0.27	0.46	0.41	0.46	0.072	0.060	0.015	0.011	0.0022		
rs4255548	11	68.75	GTTTTAATAACACAGGGAAACACATGTTGATGGGTGGGTCTTTAGCTCATTCTG[A/G]TTTCTCTAACTCCCTCTCTTTCTCCTCCTTTCTTTCCGTCTTTCTGCCTGCCTGCCTGC	A	G	0.21	0.42	0.40	0.30	0.34	0.59	0.60	0.48	0.39	0.30	0.38	0.015	0.050	0.014	0.033	0.0001	1	
rs7122786	11	68.80	CCTCAACACCTTCATGGATTAACAAACAGTCTCATGCTCAGTAAATGGAAAGTCC[A/C]GTCCAGTCTAAGTCAGAGCTTCAGTCYTTTTTTTTTTTTTTTTTTTTTTTTTGAGACGTA	A	C	0.69	0.83	0.80	0.74	0.86	1.00	0.98	0.97	0.89	0.73	0.83	0.008	0.047	0.041	0.080	0.0003		
rs4077638	11	68.86	GGGGCCCTGCCTGCTGGTGAGACCACCTCAGGCTTAATGAAGAATGACTCCTATAAC[A/G]TTGAGAGTGAAACAGAGATTCCAGATGTCAAGCAGTGCTCGGGGACTTGGAC	A	G	0.14	0.00	0.00	0.00	0.00	0.18	0.42	0.07	0.00	0.00	0.01	0.052	0.164	0.030	0.068	0.0020		
rs1562194	11	70.37	TCAGGCATCCTGTTGCCTCAAGATAAGCAGAGTTGAACCAGGTAGACCACACGGG[A/C]TTTACACTGGGCAGCAATCACACTGAGCACTGGCACAGGCCAGACCTCCAAGCC	A	C	0.40	0.64	0.75	0.74	0.70	0.73	0.31	0.76	0.61	0.79	0.73	0.050	0.099	0.002	0.000	0.0001	1	
rs749811	11	70.66	GCTTCTGCACAAGGGACAAGTATGCAGCATCGGCCACAGACGAGGGGGACTGTG[A/G]AGGATACAGACCCAGCCCTCAGCCAAGACAGCCAGGGAAGGCAGGAGCACCCT	A	G	0.38	0.32	0.25	0.29	0.36	0.36	0.43	0.42	0.32	0.33	0.27	0.004	0.012	0.007	0.005	0.0011		
rs1660861	11	70.75	CACCTGCTGCTCAGGGGTCAAAGTTTAGGAGTTAAGGTGCATGTTCTTCAGAAAG[A/G]GATGCTGTCAGGGAGCCCTGACACCAACTATCTCCTTGGCTTCACTCTGTGGACAC	A	G	0.31	0.59	0.63	0.60	0.66	0.50	0.65	0.51	0.46	0.65	0.63	0.038	0.001	0.018	0.004	0.0000		
rs514933	11	71.66	TGGAAATGGCACTTAGTGAGGGTGGGAAAAGCCAGCGACTCCCTAGTCTGTC[A/G]CCCACCAAAGGAGGAGCCTAACCCACTCCTATTCCTACCCCTCCACAGGGGCCATTC	A	G	0.33	0.65	0.67	0.57	0.63	0.64	0.94	0.70	0.71	0.64	0.55	0.058	0.052	0.000	0.001	0.0096	1	1
rs593753	11	72.42	ATTCTTATCTTTGCCCTTCTTCAAATGAAATGAAATTGTAACTCTGATGAGCCCCC[A/G]TAACTGGATAATCTGTATCTAAGCAGCCAAGTACTGCTGAAGAAAAGTTTCGACGG	A	G	0.14	0.24	0.24	0.18	0.14	0.23	0.33	0.05	0.19	0.28	0.21	0.005	0.029	0.026	0.003	0.0004	1	
rs591804	11	73.09	GCTGAGGGGGAGGTGTGCACACCTGCCTCAGGTATCTGGTTCCTGGTCTTTAGC[A/G]GCCTCTGAGGTGAGCCCTCACCTGAGGGTGATGCAAAGCAGCAGAGCAGCCTAGT	A	G	0.50	0.74	0.63	0.60	0.64	0.86	0.85	0.66	0.71	0.68	0.58	0.010	0.028	0.000	0.029	0.0013	1	
rs539343	11	73.88	CCCCTTTAAATCTGCATTCGCAGTTTGTTTTCACCTAGATAAGAATGCTGGGA[A/G]GGGAGAGCATACTGTTTTTTTTTCCCCCAGTCCTACTATTTTAGGATTCTAACAACACA	A	G	0.43	0.48	0.50	0.43	0.38	0.45	0.78	0.41	0.50	0.40	0.31	0.003	0.060	0.007	0.001	0.0267	1	1
rs589149	11	75.61	CTCCACCCTCACAGCTGCTCCCACTGGAGCCAGGCTCTTGCCTGGAAGAACTGC[A/G]GGTTCCCTGGGAGACTCCCCAGAGCCCCTCCTTAGTGGACCCAGGCCCACCCTG	A	G	0.71	0.77	0.71	0.74	0.75	0.55	0.94	0.67	0.86	0.67	0.77	0.000	0.052	0.000	0.009	0.0027	1	
rs1945085	11	76.46	TCAGCTTTTGAGTTGACTTCCAGAAAGTTGAGATCTTTTGACCATTTTTTCTC[A/G]TGTCATATAAAATGTGCCACATGGTAGTTGCATGTGGTAGTCATGTACACTTTTTT	A	G	0.81	0.41	0.41	0.35	0.32	0.68	0.84	0.56	0.50	0.49	0.47	0.079	0.099	0.003	0.028	0.0005	1	
rs1945465	11	78.08	TAGCCTCTTGCAGCAAAAAC[A/G]GAACTAGCTCTTCCTGGGTCCAAGGCTCCAGATAAACTGGCCTGAGCTGCCATCTACCGGCCAACTGTGCACACTGCAGCAGATACTGC	A	G	0.50	0.20	0.23	0.26	0.27	0.82	0.48	0.58	0.25	0.20	0.33	0.068	0.040	0.085	0.224	0.0082	1	1
rs1783953	11	78.18	TAGAATTCAACATATGTTTAAGAATTTATAACTGTTTTAATCTCATACTACCCA[A/G]TCCAGCTAAAGCAGCCCTACTGCACGTGGCTTCAGAATCTTAATGGAGCTCATGCAATA	A	G	0.79	0.24	0.32	0.17	0.41	0.45	0.76	0.28	0.36	0.31	0.30	0.140	0.138	0.002	0.018	0.0001		
rs1247726	11	79.69	ATTTTCCATTAGCTCCCTAATTACTGATGCACTCCAGGAGACATTCTCTTAGACAG[A/T]CACCCACCTGATTATGAGAGACTTCAGCTGATTTATGGTGTTGTGAATGTCCTCTG	A	T	0.33	0.38	0.38	0.34	0.50	0.64	0.65	0.53	0.36	0.36	0.36	0.006	0.020	0.001	0.015	0.0002	1	
rs1459937	11	81.25	TTGCCTTTATAACAAACCTGCACATGTACCCTTGACCCTAAAATAAAAGTTAAAAA[A/G]TATCTTTTGGGGAAAGGAAGACATTAGAGAAAAGAGCAGTTCATAAAGACATGCAG	A	G	0.31	0.34	0.36	0.30	0.26	0.23	0.03	0.55	0.25	0.34	0.33	0.001	0.106	0.042	0.011	0.0004	1	
rs1945906	11	81.29	GGCATTTCATCATTAGTCAGTGTTCTTGACTTTTGAACAGAA[A/C]TATATTACCAGAATGACCTATAGCACTTATTTTACAATTCTCTAGTGCCACTATAAATTAATGAGAATATTGA	A	C	0.69	0.71	0.71	0.67	0.66	0.55	0.27	0.77	0.46	0.70	0.68	0.000	0.081	0.030	0.004	0.0004		
rs1459952	11	81.31	TTGGCTTTGGACCGGAGCATTTTCACATTAAAGAAAATCCATGAAATTTCTCC[A/G]TTTTGGCAGAGTTTCTCATGCATGTGTTTAATATATGAACACCTCATGACCTTTTCAAT	A	G	0.07	0.33	0.29	0.29	0.32	0.36	0.56	0.15	0.32	0.25	0.27	0.059	0.020	0.034	0.000	0.0006		
rs2077889	11	82.08	CCATGAAGACCAGAGAGATGTG[C/G]GGTTGAGAGTATATGCAGAGAAAGAGAAAATCACCACCTCTTGCCTGAGAAACAAAGAAAGAATAGCTGCTTTTGTTTAAATTTGACC	C	G	0.29	0.18	0.25	0.24	0.18	0.36	0.24	0.31	0.14	0.29	0.30	0.002	0.000	0.004	0.012	0.0015	1	
rs1278402	11	82.70	TCAAATATTTAAAAAATACATGTAATAATCAGAGCTCAATATTTAGCAACTCTACAG[A/G]TATTTATTAAGTGTTGTAATTTGCACTAAAGCACACATTTATTATTTAGGTAATGG	A	G	0.93	0.76	0.73	0.68	0.70	0.95	0.98	0.89	0.75	0.74	0.74	0.046	0.084	0.033	0.054	0.0002	1	
rs948142	11	84.00	TCAAAAATGCCAGGGTTTTTGAAAAAAAGAAATATCAACATTTATGGTAAACATAAA[A/C]AACATAAATCACAATAGTACTATGCATAGTGTTCCTTGCAAATGTGGATGTGTTGT	A	C	0.43	0.30	0.34	0.30	0.36	0.36	0.02	0.57	0.21	0.33	0.35	0.009	0.095	0.027	0.000	0.0002	1	
rs1914375	11	84.64	AATAAAGCAAACCTTTATTACTTATATACCATGTATTTGGTGCTTTCATATTC[A/G]TTATATCAAAGAAATATCAATAACTCAATTTTTTGCCAAAAAGGAGTTTGAGACTTCAAGTG	A	G	0.35	0.34	0.35	0.32	0.37	0.32	0.64	0.47	0.25	0.24	0.32	0.001	0.046	0.016	0.000	0.0002	1	
rs561646	11	85.47	GAGAACGTGTATTAAATTGGCTAGTTGAGCATTTACTTGCTGTATGTTTTA[A/G]TGGATCCTTATCCTTTGCAAAAATTTAATTCTAGTCATTAATCTGTCACTAGCTCCTGTGA	A	G	0.69	0.68	0.72	0.65	0.70	0.59	0.58	0.55	0.68	0.68	0.69	0.001	0.011	0.009	0.010	0.0006	1	
rs1986778	11	86.95	TGATGAATATAGTATATGTTTTTCCA[A/G]TTTCCAACTCTTCATCTAGTGGTGCTATGTGTCCTAACCTCCTTCCCCCTCCCTTCTTCCTTTTCTTTTTTATTTCTCCTCCTCTTT	A	G	0.36	0.45	0.47	0.54	0.41	0.18	0.27	0.29	0.50	0.51	0.54	0.002	0.021	0.004	0.029	0.0042	1	
rs1880116	11	87.02	AGCTTTGGTTGGTACAGATTCCCTGCCTGGAATTTAATAAATATTGTGGGCTCTGT[A/G]TACCCCAACATTTTAGGAAAATCTTTTTGTTTAGAAAACTGTTAGTTGAAGGTCAAA	A	G	0.69	0.76	0.81	0.79	0.73	1.00	0.96	0.85	0.81	0.77	0.78	0.005	0.030	0.005	0.070	0.0006		
rs160195	11	87.98	CCACGTTAGAATTGTCTGTTTAATTGAAAGACTCTTTTGCACCATCATCTCTAG[A/C]CTCGCCACAGACACACACTATACCATGAACTTATTAAAGAGAGGAGCTTACTCATTTTC	A	C	0.26	0.60	0.69	0.60	0.75	0.64	0.44	0.63	0.75	0.65	0.60	0.114	0.055	0.005	0.005	0.0052	1	
rs496972	11	88.92	AAAAACAAAATACATGACATTAGTTTAGCACATTAGGGGGCAGGTAGAAAGGACCT[A/G]CAGGCTAGAAAGTGATGTCCTTTGATATGCCATTACAAAACATATGCCAACTTGG	A	G	0.52	0.81	0.80	0.87	0.88	0.64	0.33	0.50	0.57	0.80	0.88	0.040	0.107	0.041	0.014	0.0037	1	
rs1404532	11	90.23	TGCGTGTGTGCAAGTGTGTGTGTGCATGTGTGTGTGTTTGATAGCACAGGTCT[A/T]CTTGTGACAAATTCTTTCAGCTCTCAGTTTTCCCAAAAATGTCCTTATTTTGTATGTATT	A	T	0.48	0.48	0.46	0.40	0.38	0.32	0.17	0.25	0.31	0.50	0.56	0.000	0.057	0.047	0.015	0.0027	1	
rs1388472	11	91.33	CTTGCACCAGAAAAGAAACCTATCTTTGCCCAAAGACTTTTATAAGGAAAAGT[A/G]CAAACTAACCACCTAGCTGTTGTCTCCTCCAGCTCCATTTCTTACAGATGCTCACATCA	A	G	0.50	0.50	0.42	0.40	0.46	0.14	0.28	0.19	0.42	0.42	0.45	0.005	0.007	0.017	0.042	0.0002	1	
rs1027454	11	91.52	AGTCTTACAGATGAAATGTGCCTCACCAAAAGACTGGCCTACGTAAAATACAC[A/C]TGATGTTATAGCTACCTGTTCAACTTTAGTGCCTTTGTAGAAATGAGTTAAATCAGTCTC	A	C	0.60	0.54	0.54	0.54	0.43	0.27	0.38	0.38	0.50	0.54	0.59	0.006	0.002	0.003	0.022	0.0023		
rs543704	11	91.92	ACCTTTCCCAGGTCTGGGGTGTTGCCTTCAGTGCCTCTTCTGGCACTCTCCAAT[A/C]AAACCAGAGAGTCTCTTAAAACTCAACTCTCATATTATAAAACATAACCCCTTTCCTCC	A	C	0.33	0.54	0.52	0.60	0.52	0.18	0.11	0.34	0.50	0.51	0.51	0.029	0.140	0.021	0.090	0.0003	1	
rs536417	11	91.96	GCTCATGCTTTGCATCCATTTCAACCTAACCCTGAGCGGGAGCTGGTTTTGC[A/G]AAATGACATTTATTTGCCATTGAGGGCCCTGAGACCGTCTCTATCTCTGACAGTCTTTGA	A	G	0.33	0.43	0.43	0.44	0.41	0.64	0.68	0.51	0.38	0.47	0.44	0.001	0.041	0.003	0.023	0.0009		
rs586699	11	91.98	CATGCCTCCCTACCCATTGAAAAGCTGTGGTGATTTGTTAATTAATTCATGT[A/G]TTCATCTTCTGATATCAGAAATATCTGTGAGGCCCTACACTGAGCGTGGTACTCTCCAAGC	A	G	0.50	0.57	0.52	0.54	0.48	0.82	0.90	0.72	0.57	0.59	0.53	0.000	0.074	0.018	0.043	0.0010		
rs1944108	11	93.15	GTATTGCATTTCTATTACAGTTAATCAACTGTTCCCAATAAGCTGATTGCTAGCAA[A/C]GTAGCAGATAGAAAACCTACTGGGAAATTTTTTTATGGTCATGTCCACAGCAAATGA	A	C	0.57	0.57	0.65	0.66	0.59	0.64	0.54	0.49	0.71	0.58	0.60	0.000	0.001	0.015	0.001	0.0025	1	
rs584109	11	93.70	CATTTCATTCATTAGACAGACCATATAATGGCCATGAATGGAGGCTCCAAATCC[A/C]AGCTCTCTCTCATATTAGCTGTGTGAAAAACTAGCATTTTAATAGTGCCTGTTTCATCA	A	C	0.31	0.51	0.52	0.51	0.50	0.23	0.67	0.20	0.57	0.55	0.52	0.025	0.014	0.057	0.041	0.0007	1	
rs541821	11	94.85	CTTCTGTAACACAACTACATAATATCCAAAGACAAAGTAGAATGGCAAGAACTTC[A/G]CAGAGCGGAATAAGCCTTGATGGTAAAGGGAAACATCCAAATAAGCAAGCCCTATGC	A	G	0.55	0.25	0.27	0.33	0.38	0.55	0.32	0.21	0.23	0.31	0.28	0.024	0.002	0.003	0.022	0.0000	1	
rs1255182	11	95.16	GAGTTGCCCTCAGCTAAATTCACAGACCTGTGGTAGTGTGGCCCAAAGTGTTC[A/G]TCTTGATGTTAAGAATAATTTTAAAACCCCTTAAAAAAGAGCTTTTGGTGAAATTCATC	A	G	0.71	0.47	0.51	0.52	0.46	0.41	0.39	0.40	0.43	0.52	0.52	0.015	0.005	0.004	0.007	0.0000		
rs483884	11	95.60	AAAACAATAAAAGACATCCGTGGGTTTGTGCAACTCTCTCAGACAGTTTTATG[A/G]GGAGGATCAAAGATGTGGGCCTCGACTCTGTGATAAACCGAGGTCGGCTGCCATAATC	A	G	0.36	0.42	0.45	0.53	0.54	0.27	0.34	0.55	0.39	0.53	0.53	0.000	0.000	0.029	0.006	0.0028	1	
rs921562	11	96.84	ATCTATTCTTCCAATTTCTCAACCTGAGTCATAACCGTCAGAAATATTTCCTCC[A/G]TATTATGTGATATTACCTTTTAAATCCCTATACAAATCAAAATGAAAAGATGATACTCCT	A	G	0.76	0.56	0.56	0.51	0.66	0.64	0.67	0.61	0.57	0.65	0.58	0.025	0.005	0.010	0.006	0.0005	1	
rs723513	11	97.62	AGGCTGTAACAAAATATTGCACGTTCAGCCAAATTTGAGCTATCTCTTTACTATCC[A/C]TTACTTTTTAAAATATTTCAGCATGAATGGCCATTCATGCATTTATGTTTCCAAATGT	A	C	0.33	0.46	0.47	0.39	0.48	0.55	0.16	0.46	0.64	0.39	0.44	0.005	0.051	0.001	0.011	0.0001	1	
rs1002122	11	99.60	GCAAGCAGCCCAGGTATCAGGTTGCATTTTCATACTCTTGCTACTCTGGTGGCAAC[A/G]CAGAATAACTGCCATAAATCATCTCTTGGCTGTATACAATTAATTTTGTGTTCTGTA	A	G	0.43	0.46	0.53	0.41	0.52	0.64	0.75	0.69	0.46	0.56	0.49	0.006	0.022	0.017	0.005	0.0011	1	
rs622675	11	100.20	GTTAATTGGATGCAAGAGAGACATGCTTTCCCAATTCTCATCGTAGTTTTAAAATAT[A/G]TAAAGCCCAAACTGATCAATTGTTGAAACATTTGATGGTATATTACCTAGTATTTAA	A	G	0.90	0.80	0.81	0.80	0.75	0.73	0.96	0.84	0.68	0.82	0.85	0.009	0.042	0.003	0.002	0.0006	1	
rs618291	11	100.22	TGGAAAATGTGGCCACCGTATGAAAACCTTACCTTTTTTACATTCCAAACTTCT[A/G]CCAAACCTTTGTATCTTTCACATATTTTCAACAGCAGATACACTGCTATTTGAAAAATGA	A	G	0.55	0.50	0.52	0.51	0.46	0.68	0.80	0.58	0.42	0.61	0.56	0.001	0.054	0.003	0.022	0.0014		
rs1943752	11	100.63	GATGATTGACTAGAGATGCTGAGCATGCATGTCTGTGCACAAGAAAGGACCAA[C/G]GCAACAACAAATAAACAGCTAAGATTTGACTGGAGTGTCAAAGGGAGAGGACTGGAGT	C	G	0.03	0.30	0.30	0.26	0.27	0.00	0.17	0.21	0.25	0.23	0.31	0.057	0.004	0.000	0.090	0.0010		
rs948734	11	101.47	ATCTCAGGGAGTCCACAAAATGTTCCGG[A/G]GGTGGAGGGCATCAGGCTGAAGGCTGGACCCAGGAACAATCCAAAATCATATGCAGAACTGCCCTCATGAGGAAACTGTCA	A	G	0.81	0.42	0.48	0.43	0.48	0.77	0.88	0.40	0.38	0.51	0.51	0.064	0.134	0.003	0.052	0.0011	1	
rs1711399	11	102.00	TCTTCAAGATAGTCAATGATTTCTGAATTTTATGCAAAGTGACTTGCAAATCTG[A/C]AGATCCTACCCCTTTTCAATCATATTGCCCCTCTACCCTGAATCACTCTTTCTGTACAG	A	C	0.43	0.54	0.56	0.48	0.43	0.45	0.50	0.69	0.46	0.61	0.58	0.004	0.002	0.015	0.005	0.0003	1	
rs1784405	11	102.01	TCATGAACTAAGCAGCCCTGGTTAATGAGTCCCATAGAATGAATGGCTTCTCAGCC[A/C]ATGACATGACAGTTTGTACTTCAAAGTTTCCAGTGAAGCAAACTAAGAAGGAAACT	A	C	0.57	0.46	0.44	0.52	0.57	0.55	0.50	0.31	0.54	0.39	0.42	0.004	0.002	0.015	0.005	0.0003		
rs1276278	11	102.06	TCAAGTGATATCATAAAATGCTCCAAAATGAAGTGCATGGAGACCCTGTAGTA[A/G]TAAAAGGCCTGGCAAGATAGAGATGTTCCACATTTGGGTATAAGCCTCCTCCCATCAT	A	G	0.50	0.58	0.54	0.43	0.54	0.82	0.78	0.67	0.79	0.48	0.51	0.003	0.024	0.002	0.032	0.0008		
rs1298740	11	102.09	TGTAGGAGAATGCTGCCTTACCTTCTGCCGAATGGTCTTTGAGCTGAGTTTCTT[C/G]GGGGCCTTGTGGGCTACAGAGACTTTATTTTAATGGGTATTATAAAGATTACTGGCAT	C	G	0.48	0.43	0.46	0.59	0.46	0.18	0.22	0.33	0.21	0.52	0.49	0.002	0.024	0.002	0.032	0.0010		
rs536107	11	102.44	CATTTGTTAAATTTTTTTTCTGGGTAAACAGTTTTTAATAGAGCTAAATTTAAC[A/G]GAGTATTGTAGGCATATCACCTTCTACTCTTTTTGCTAATCATTTTTTTTTTCCTGAGTA	A	G	0.74	0.45	0.49	0.50	0.45	0.73	0.57	0.63	0.46	0.47	0.49	0.024	0.004	0.003	0.022	0.0000		
rs601535	11	102.64	CTTTAGTTCTTCAGGTTTCTAGAACACATCTTCCATGGTAGAAAGAGTATATG[A/G]GCTGGAACATATTTTGAGATTTGCATGTCTAAAAGTGTCTATAATATATTATTGTATGTGA	A	G	0.55	0.64	0.66	0.65	0.70	0.68	0.88	0.52	0.57	0.68	0.62	0.011	0.026	0.020	0.001	0.0014	1	
rs559345	11	102.84	CTAATGGGCTTGAACCCAGGTTTGTTTCACCTCAAGCATTTGATTGATTGGTC[A/G]CGTTAATGCGCAGAAATTAGTGCGACTGCCTCAGTAATTGTTTTATGTTCTAATTTATAT	A	G	0.67	0.56	0.54	0.48	0.52	0.55	0.67	0.77	0.57	0.58	0.54	0.011	0.016	0.070	0.003	0.0002		
rs540819	11	104.40	ACTTCACCACCGATATAGCTCTTTCATTTATTTCACCCTCCAACAACTCTCAAT[A/T]CTTACATTTTCAATTGCCAGGAAAGAGGTAGAAATCTCTTGTCAAGGTTGCTCGTTCTAT	A	T	0.95	0.53	0.59	0.52	0.57	0.41	0.87	0.63	0.57	0.43	0.44	0.108	0.068	0.004	0.012	0.0077	1	1
rs1785872	11	104.51	AACTCGGTTATTGATGTCACCATCACCAGTTCTTTAAACCAGGAACTTGAAAC[A/G]CATCTTGGCCCCTTTCCTCTCACGCATCTCCAATATTCAAGCCACCGCCAAGTTTTATG	A	G	0.50	0.40	0.42	0.41	0.45	0.55	0.89	0.70	0.31	0.45	0.45	0.004	0.177	0.071	0.017	0.0006		
rs1483025	11	104.54	TACCACAAAGCTAAGAGGGAGCTGTGACATTAATGGCTGTGTATTCAAGACATC[A/C]ATACATGGCATAAATAATGTCACTTACCTCCTGATCATAACACCTATCATAGATTGGA	A	C	0.71	0.40	0.42	0.41	0.45	0.55	0.88	0.70	0.36	0.45	0.46	0.048	0.158	0.071	0.017	0.0008		
rs1822982	11	105.06	AATTGAGAACAATGGAATGGTAAAAACATCATTTAAGACAAAATCAAAAGCCAAGAATT[A/G]GATGAAATAATCTTAATGGCACACATTGTTTTCTAAATGAATTCACATTCTATAT	A	G	0.36	0.56	0.57	0.57	0.61	0.14	0.81	0.58	0.54	0.54	0.39	0.019	0.033	0.000	0.105	0.0148	1	1
rs1938961	11	105.15	TAGAGCTTTAAACCCCTACACATATCTTACTAATCTTTGATTTATGAAATCTATGAAATGAGGTGTCTTCCCACCCACGCACCTGCACATCTCATTC[A/G]AGGCCAACCTTATAT	A	G	0.79	0.63	0.62	0.65	0.64	0.45	0.76	0.68	0.75	0.68	0.58	0.027	0.024	0.012	0.009	0.0007		
rs2000633	11	105.40	GAATGTACCATTCAATGTAGCAAAATAGTTTAATATATGTCTTTGGATCTCTC[A/C]TCTCCATGGGGAGCTAAGATGTTAAGGGGAGAATTAAAGCTTCTCTTTCTCAGAACTTC	A	C	0.40	0.46	0.41	0.40	0.45	0.55	0.27	0.34	0.54	0.42	0.40	0.002	0.027	0.011	0.003	0.0004		
rs1504747	11	107.38	ATTTTAAAGTTTTAGTAAATGAAAGTGTGGTATTAGCTCAACATTAAACCAATAAT[A/C]AGTGGATCTGAATAGTCTGGAAACAGATTCTGTATAGATACAGATAGAGCTAAAATAT	A	C	0.16	0.23	0.24	0.35	0.23	0.23	0.21	0.39	0.50	0.31	0.28	0.007	0.009	0.008	0.003	0.0016	1	
rs627418	11	107.67	TAAATTGGTATGACTATTTTGGAGGACAATGACAATATCCATCAAAAAGTAAAATTC[A/G]CATTCCTTTTGAACAAGCAAGTCTACTGCTAGAAATTATCCTACATCCATCATACA	A	G	0.31	0.46	0.42	0.45	0.39	0.36	0.41	0.33	0.39	0.36	0.40	0.011	0.000	0.006	0.002	0.0003		
rs882524	11	109.74	CCATGCCTGGCCAGCATTTATTCTTCGAACCGCCACCAAGAAGCACAATATGACA[A/G]AAAGGCAATGATGCCTCGAAGGGAGCGCCAATGGTCTCTCATGATCAGCCTCTGT	A	G	0.83	0.72	0.65	0.68	0.63	0.70	0.56	0.43	0.58	0.63	0.69	0.037	0.000	0.007	0.007	0.0001	1	
rs1469170	11	110.28	GGAATGAAAGGAATGACTGTGAGTAGCAGGCAGGGGTGATACAAAACCAAACCAT[A/G]CTGGATATGAAGTTTGGGTGTTTTGTCCATTTCCACCTTCCATGGACTCAGTGAA	A	G	0.85	0.28	0.30	0.33	0.32	0.50	0.51	0.57	0.32	0.31	0.34	0.128	0.012	0.025	0.010	0.0012	1	
rs1940528	11	111.08	TCAGTCAACCCTTCCAGGTGAGACTCCAGCTCTGCTTTGTTCATGTAAGCTTC[A/G]TCCACATCCTTCTTGATGAAGACAAATTCGTTTTCCTTCTCTGTATGCTTGTTGATCTCA	A	G	0.98	0.82	0.81	0.80	0.75	0.41	0.61	0.84	0.79	0.88	0.91	0.047	0.027	0.002	0.083	0.0121	1	1
rs720023	11	112.38	CTTTGGTCCACTTGGACCCACTTGCTTGGGAATATAAACTAATCGTTCCAGTTTATTAAACGTGGACCTCATAGAGGTTGT[A/T]TGTGTAGTGCTTTGAAGATTAGAATCATATAT	A	T	0.52	0.59	0.60	0.56	0.48	0.18	0.30	0.45	0.57	0.71	0.61	0.010	0.072	0.018	0.137	0.0000	1	
rs2011505	11	112.53	CCCACTAATCCCCGCAGCTCAGACCAACTATTTGTGTTGTGAAAGGTCTTATTGGC[A/G]TTTTTGCTTTAAACCTAAACCAAAATGACTACAAAAATTTTGGTGACCATCCAGAAC	A	G	0.50	0.55	0.56	0.51	0.50	0.09	0.23	0.47	0.57	0.65	0.53	0.002	0.089	0.000	0.143	0.0000		
rs869451	11	113.29	AATGAACTCATTAACTTTACCAAAAGCTATTTCAATTCTCCACACACTACATTGCCT[A/G]CATTACACATTTGACGCAGGTTGGAAATCCATTTATTTTCAACCAACCATAGATCC	A	G	0.57	0.42	0.44	0.41	0.34	0.73	0.78	0.51	0.54	0.42	0.44	0.013	0.071	0.002	0.048	0.0001	1	
rs1013582	11	114.77	TACATGGAAGGAATTGATTTAAGCAAGTGATTACTTAAAAAGGAAAATTAAAAG[A/T]GTGGAAAATGGATAGGAGAGAATGTTAATTGGGAGGGTTTCAAGTATAAAGGAGACT	A	T	0.83	0.51	0.56	0.56	0.57	0.91	0.79	0.75	0.57	0.72	0.62	0.055	0.034	0.026	0.103	0.0013	1	
rs1009746	11	115.66	GAAGGGAAACCTCCACTCCACTTTTCCAGTCATGTCCCAGAATGAAAAGCAG[A/C]AAGCCCTGATTTTGTTCATGTGCTGATTCTTCAAGTAAGGTGGAGCCAGGGCTGCTTC	A	C	0.74	0.53	0.46	0.51	0.50	0.55	0.85	0.31	0.61	0.53	0.52	0.050	0.140	0.019	0.009	0.0031	1	
rs906540	11	115.98	AATATCTCAACTCTCTTTCCTGCAATTGCTACAGCAGAAATTCCAGGCAATG[A/T]GAGTTTGAGAGTCTCTAGGACCACGAAGATGTTGCCCAATCTCTGACGGCCCAAGGATTC	A	T	0.74	0.40	0.47	0.45	0.41	0.14	0.10	0.44	0.50	0.52	0.38	0.053	0.068	0.000	0.050	0.0030		
rs609177	11	116.31	TAGAAATGCTTTTATTTATGTATGTGTACCCATGTGACAGGCTAGAAATCCCTC[A/G]TGCCCCAGGGACACCATGCTCCCTGAAATCACAGCATCTGAAAATGTTAAGCACCCA	A	G	0.86	0.42	0.30	0.45	0.41	0.82	0.67	0.75	0.50	0.40	0.30	0.148	0.058	0.130	0.131	0.0003	1	
rs236919	11	116.63	AACTGGCCTGCACAAAGACTCTGAGAAACGGGATGCCCTCTTCATACAGTCCC[A/G]GGAGAACTGCTGCAAGTTGACCCATCTTCCCACTCCACCTCCACGCTCAGTCTCCTT	A	G	0.81	0.46	0.33	0.45	0.34	0.73	0.68	0.66	0.50	0.41	0.30	0.118	0.054	0.081	0.078	0.0019		
rs947889	11	117.41	CCTTCATGTGGAATACTAGTTTCTTTCACATCACTTATCACTGATTGTAGTAATAC[A/G]TTTGTTGTGTTTATTTGCTTGGTGTTTATCTTCATAATCAGATGACAGGCTCCATGAA	A	G	0.67	0.49	0.50	0.46	0.48	0.91	0.81	0.61	0.43	0.54	0.64	0.005	0.019	0.002	0.073	0.0046	1	
rs7389	11	118.07	CCACAGGGCCCTTCCCCTCTGCTGCATCATCTCCTGCTCAGGCTTTCTCTCTAGG[A/C]GCATTGGAGGAATCCTCTTTCCCTGCTGGAACTCAACACTGCAGAACTCCAAG	A	C	0.86	0.63	0.68	0.72	0.61	0.64	0.14	0.82	0.75	0.64	0.73	0.032	0.140	0.017	0.000	0.0033	1	
rs658922	11	118.54	TCTACATATTCCAATAAAGACCTTTTTTGTCTTCAACTCCTGTCACTTGGA[A/T]TCCAGGACTTCTTCCATCCCTCATGTTTGTTCCTTACTTTGCCAGCCTCGGCCATTTCTGTA	A	T	0.36	0.48	0.45	0.54	0.45	0.91	0.11	0.74	0.54	0.55	0.55	0.005	0.063	0.058	0.137	0.0016		
rs644252	11	118.58	GGCTCTGGGTCCAAGCCTATCTGCACTGCTAGACTTCTGGTTTCCTGAGATA[A/C]AGGCCCCACACTGGACCCCCAAGTTTAGAGGCTTGTCTGACTCTCACACCCTTCCCAG	A	C	0.14	0.51	0.50	0.46	0.54	0.23	0.86	0.22	0.46	0.43	0.42	0.076	0.070	0.066	0.048	0.0010		
rs1073636	11	119.51	GTTGAACAACTCCAAGCTCCATGACTTGCACAGGTAAACACAAAGAGCCTTGAA[A/T]GTACTATTTCACAGGTGTAATTATCTCTATCCTGCAAGTGAGTTAAATAGCACATTCT	A	T	0.48	0.50	0.52	0.55	0.45	0.00	0.17	0.29	0.50	0.51	0.53	0.001	0.070	0.035	0.230	0.0000	1	
rs481703	11	120.59	CCTCTCTGGGTCAAGTCTATTAAATACCATTTTAAAATGCTAAATGCTACCAC[A/G]TCCTTCAAAGGAGACATAAGCCCTATAATCTGGGTTTGCATTTGTAGAGTGTCTCATAT	A	G	0.30	0.67	0.67	0.70	0.52	0.82	0.81	0.41	0.54	0.66	0.67	0.063	0.013	0.012	0.021	0.0000	1	
rs665035	11	120.62	CTCATGGATCAAAAGTACTTCAGTCCCATAGTAATGGTGGTGGGATCTGTCTTGG[A/G]TGTGAGAGTGCTCCCAGATCCTGCTGCCTCCTGGCCTGGCAGACAGAAGGAGATG	A	G	0.31	0.39	0.41	0.43	0.38	0.64	0.59	0.40	0.39	0.45	0.48	0.003	0.008	0.001	0.028	0.0027		
rs575030	11	120.63	CTCCGATGAGGGTAGTAGATAGTAAATGATGAACTCAAGACACTTTGA[A/G]GGAAGTACATCAAATATTTTGCAGATTGACTCACTGCCTGGCTACAATAGCCTCTTA	A	G	0.50	0.67	0.65	0.70	0.66	0.86	0.88	0.45	0.50	0.67	0.70	0.012	0.035	0.012	0.035	0.0010		
rs596437	11	120.91	GGCTCCTCCTTCTGCACATGGATGAGCCACCAGATTCCAGGGTCAGTGGT[A/G]TTAGTGAGGAGATGTAGGTAAGATGAGTTAGGCCACCACTGAGAGGTGTTTCCCAAATT	A	G	0.67	0.51	0.56	0.50	0.57	0.41	0.67	0.49	0.68	0.62	0.59	0.008	0.016	0.002	0.004	0.0011		
rs617847	11	120.94	TAGCTGTGACTGAGACTGAGGTGCACCCCATGACTGGGACTTTTCCTTCGG[A/G]CAAAGCTTTACCAGGATATTCATGACTTGTGTGTGTAGCTTCTCTAGGTTTTAAATCTCC	A	G	0.25	0.44	0.45	0.46	0.45	0.59	0.45	0.50	0.36	0.38	0.40	0.029	0.003	0.003	0.003	0.0025		
rs676943	11	121.59	CCTGAACTTGTCCCACACTCTTTCTTCATCTACCCTAGAGGACCAACTGCAGGCCTG[A/G]ATACATGGTACTCAGTAAAGCCTTGTTAACTTACATTGAATGTTCTTTGTATTAAT	A	G	0.62	0.47	0.44	0.49	0.43	0.68	0.75	0.52	0.50	0.52	0.44	0.029	0.109	0.010	0.052	0.0000	1	
rs668183	11	121.80	ATGAATCAGTTGCCCAGACAATCTCCAACTTTGATGAGCAGCCCTGGTCACATAG[A/C]CACTTGGTATCCTACTCTCATTCAGTTACTTATGAGCTTCTTTTCATATGAGAATAAT	A	C	0.64	0.81	0.77	0.75	0.77	0.77	0.59	0.68	0.79	0.84	0.82	0.012	0.019	0.001	0.000	0.0021		
rs1894078	11	122.14	GCCTGTCTCCATGAGGATGCAGAGTCGTTGAGTTTACATGGGGAATATTTAGCC[A/G]TCCACGTCATTTCTGTGGGCTTCCCTAGGCTGACCCTGGAAAGAATTCCGGATGCT	A	G	0.17	0.41	0.45	0.50	0.46	0.18	0.05	0.41	0.35	0.47	0.45	0.075	0.168	0.013	0.067	0.0000	1	
rs930983	11	122.37	AAGTTGGATCAGTGATCAATTCATAGCATTTCTGTTTCAAATATTCAAAAGCAAAA[A/G]TAATCTGCCAATTAGAAAACAAAAAACTTCAAACTTAAGTGATGTAATGATGGAGC	A	G	0.60	0.49	0.40	0.39	0.43	0.77	0.92	0.59	0.38	0.38	0.37	0.014	0.176	0.011	0.078	0.0015		
rs485345	11	122.61	TCCCGCCTCTTAATGACAGAGCACCATCTTCTGAAGCCTTTAAAGATACAGTCTA[A/G]CTCATGCCTCTGAAGTGAGCAGAGATGGATAAAGAAGCCACCTCATAGACACAAA	A	G	0.40	0.74	0.74	0.78	0.77	0.45	0.53	0.83	0.77	0.76	0.73	0.061	0.022	0.013	0.036	0.0007		
rs1943467	11	122.80	GAGGCTGGGGTCGATGAATGGACCCAGCTGTGGTACAGAGGAAAGTTCCTTTC[A/C]CCCCTCCTTGCTACTCCTTGGCTTCCCTGACCCACCAGACTCCTCCATGGXAAAGTC	A	C	0.87	0.34	0.27	0.35	0.23	0.09	0.31	0.29	0.32	0.29	0.34	0.224	0.005	0.004	0.020	0.0055		
rs1148109	11	123.04	AATGCTGAAGACAACCTGGATAAATGAGAAGCTATACACTCCTGGTGGGTGTTCGT[A/G]TACATATATGGGGAAATGATTATTTTAATCTGCAGAAAAGGTTGTTTTTCTCTTG	A	G	0.60	0.49	0.58	0.45	0.41	0.73	0.51	0.71	0.54	0.61	0.60	0.003	0.000	0.028	0.022	0.0004	1	
rs6590016	11	123.29	AGCTAACTATTTATCTCAATGATCTTGAAATCCTTTTCCTCTTCACTGGCCCCTAC[A/C]AAATGTTAACTAATCTTGGAGCGTGTAATTCTAACTCCTAAATTGGACCTCTTTAGTC	A	C	0.19	0.41	0.46	0.43	0.43	0.45	0.25	0.47	0.50	0.45	0.42	0.051	0.034	0.000	0.001	0.0005		
rs2512161	11	123.67	TGGTTGTCAACAGGATTTAAAGACTTACAAAATCAGGTACATGCAAGTTATCATAT[C/G]TGTGTGCGATTTACCACATAAGGAAGTCAAGGCTAATAGTTCAAACTCCTTGTATA	C	G	0.36	0.57	0.54	0.55	0.57	0.41	0.59	0.31	0.54	0.52	0.53	0.012	0.001	0.017	0.005	0.0000	1	
rs1893702	11	123.82	ATTTAATCATGATTTGTTTTCAACAATAGCTCTCACAATGAAGCCAACCTCATTCTGG[A/G]AATGTGTCACTTCTTTAGAAACATTGTGTAAAATAAAAGACGTTATCATGTTACTTT	A	G	0.79	0.58	0.53	0.50	0.57	0.73	0.58	0.43	0.54	0.54	0.59	0.020	0.007	0.015	0.005	0.0004		
rs2276189	11	124.04	AGGAACCTAGAGTACATAGTGTGAAAAATACGGTTCAGGCAATGATTATGTTAATGA[A/C]GCCATGCACATTCATTTTTAGTGGCTGAGTAATTTAAAACATGATGTGGGAATTC	A	C	0.31	0.00	0.00	0.01	0.00	0.18	0.00	0.02	0.00	0.00	0.00	0.122	0.000	0.000	0.068	0.0018		
rs6590098	11	124.19	CGTATTGCTTCCCAAATACGAAGGTGTTTCCATTCATGAGACCAATGTTTAAAAGG[A/G]AGACTGTGCAGAGCAGGCTTCCCCTGAAGCAGTAGGCCACTTTATTCCTTTCGTC	A	G	0.05	0.00	0.00	0.00	0.00	0.00	0.00	0.00	0.00	0.00	0.00	0.017	0.000	0.000	0.000	0.0000		

rs4936975	11	124.46	CAAAAATAGAGAATAGCATGATGAAACTCCACAAACCTGTCACCTAGATTCACTATT[A/G]TCAAGCTTTGGCCACACTCACTTCATCTATACTGAATTATTTTAAAGTAAATCTCA	A	G	0.67	0.86	0.86	0.87	0.91	0.91	0.93	0.65	0.86	0.89	0.87	0.037	0.000	0.081	0.001	0.0002	1		
rs593290	11	124.62	TGAAGGATTTAGCAAGCATGTTGGTAATGGGACAGGAGAAAGTGAATATGAGAGAC[A/G]TATGTGGAAATTGGGCCAGTTAGGGCTTAAAATGTGCCTGTGTGGGAATGTGTG	A	G	0.55	0.43	0.47	0.51	0.52	0.45	0.73	0.47	0.32	0.39	0.38	0.007	0.039	0.000	0.000	0.0045			1
rs3740892	11	124.79	TACCCTCTGACCCCCTTCAAAATGCCCTAGAAATGATCCTTTCATTAATTTACCAAC[A/G]AAAGGTTTTGAGTGCCCACCATGAGTCATGCACTAGATGCACCAGTCTTGCAGCC	A	G	0.62	0.67	0.69	0.72	0.73	0.59	0.26	0.61	0.73	0.72	0.72	0.008	0.124	0.015	0.017	0.0000			
rs4627097	11	124.99	ATCCTCTTTCCTTTATTCATTATCAGCGCCAATGAAGCAAAGACCCTGGCAAAGC[A/G]TAGTAAGTATTAGCCAGTGATGAAATTTTTGCTGTGTAAAGGGTTGGGGCTGTAACA	A	G	0.64	0.60	0.67	0.72	0.64	0.64	0.89	0.72	0.61	0.55	0.65	0.000	0.035	0.000	0.000	0.0000	1		
rs1425842	11	125.15	GTCTAGCTGTTCTGATTGAATCCAGAGGGACACAGATGTGTTATCTTTGGGCCACCA[A/G]TACCTTTGGTACTTAAGAGGTTCATAAAAAAAATCACCCAGAGACATCAAGGATG	A	G	0.14	0.15	0.14	0.16	0.07	0.05	0.61	0.16	0.11	0.09	0.12	0.000	0.107	0.001	0.019	0.0000			
rs528638	11	125.41	CCCTTCTCCCAAAAACACACCCATCTTTTACCAGGAATCTGACTTCATTTTGGA[A/C]ATAAAAGTAGTACGTTGTTTGCTCTGCACTCTTCCTCCACAAAAGGAGTGAAAAATAGA	A	C	0.62	0.56	0.54	0.58	0.34	0.50	0.56	0.61	0.43	0.46	0.49	0.006	0.000	0.000	0.001	0.0001			
rs663714	11	125.59	CTGGATACAAAGCCTCAGGCGGCCCCTTCTTCATTGTACACCTCTGCATCCAGCA[A/G]AAGAGTTTTCCTTTCTCTTGTGAGCAGAGGAAGTGGGCTTGGCTGGAAGGAGTACA	A	G	0.95	0.69	0.73	0.76	0.57	1.00	0.99	0.91	0.86	0.71	0.76	0.064	0.089	0.018	0.112	0.0030			
rs586566	11	125.74	AGGTCATAGGTGGTTTTAACCCCGCTTTCATACGTCAGTGAAGAGGGTGGGCTGG[A/G]ACTCAAAAGAGTCATCCAGTATCACACCGTGAAACGTGGCAGAGGCGAACCTGGA	A	G	0.17	0.27	0.30	0.23	0.16	0.00	0.22	0.24	0.25	0.26	0.22	0.007	0.003	0.000	0.101	0.0045			1
rs2156449	11	126.07	GGCTCCCTTTGGAGCTCTCTGGCAATTCATGATTGTTGAGCAAGAAAGATGGGGA[A/G]TGAGGAATTAGTTTTCACTGAATTGCCACAGGGCCTGCTCTGACTATAAGCATAG	A	G	0.81	0.70	0.66	0.72	0.73	0.68	0.45	0.73	0.64	0.73	0.67	0.006	0.047	0.000	0.002	0.0001	1		
rs1944819	11	126.59	GCTCTGATAAATAGAGGCTGCACGCAAGGATTGGGTGAAAGGCCCAGGAAGCTGA[A/G]TAACTGTGACAGAGAGCCTCAATGTTTCCCTGCAGCTCTCTCCATCCCCACACTT	A	G	0.45	0.52	0.41	0.49	0.54	0.86	0.75	0.47	0.36	0.33	0.35	0.001	0.060	0.003	0.111	0.0009	1		
rs949095	11	126.60	TTGCTGCTTTACCCCCATCCTCTCTCAAGCCCCATTTCTGGCCCCTTCCCTAGT[A/C]CCCCTGTCCAGCCCCCTCTCCCGCTGCACTGTGCCTACCCTCTGAGAAGAGCTGTG	A	C	0.74	0.72	0.56	0.61	0.63	0.86	0.77	0.49	0.42	0.52	0.55	0.026	0.039	0.000	0.071	0.0001			
rs762827	11	128.19	TTTTGTCATTGTCATTGATGACTGATTTTATTGATGCATGATGTTACTGATTT[A/T]GACAACTGTACGTTCGTACATCTCAGAAATTTCTTTCTGACGTGTCCTCATTCAGTCCTCT	A	T	0.19	0.54	0.52	0.50	0.50	0.36	0.10	0.59	0.46	0.44	0.47	0.054	0.099	0.010	0.011	0.0003	1		
rs1027323	11	128.76	ACCATGTAGAAGTCCAGCTGCCTTCAGAGACCACTATGTTGGGAGGAGACCC[A/C]GACCAGCGATGTGGCGAGAGAGGGGCCATGTGGTAGAGCACCAGGCATGTGAATGG	A	C	0.88	0.55	0.55	0.50	0.48	0.55	0.25	0.64	0.62	0.51	0.59	0.067	0.044	0.002	0.000	0.0020	1		
rs923811	11	128.95	AGAGCTGCTCTTGGCCACTTGGGATTCTTCTCCCAGGTGCAGCTTTCTTGTTG[A/C]CCTGAGCAGCATGTCCACTGAAGTGCTTCTCAAGGAGGGAGCTTCTCAGTCATCACTC	A	C	0.50	0.69	0.60	0.48	0.64	0.73	0.59	0.60	0.79	0.58	0.53	0.000	0.004	0.000	0.022	0.0016			
rs1043654	11	129.31	TTCAGTCCAGTGTGAGTATTTTGGAGTAGGTTGGATGTAGATTTTGTTTTTATC[A/G]TTGATTTGTACCGACAGAAATAGACATTTCATCATGTAAAATTCCTGTTATTCTGGAAA	A	G	0.17	0.54	0.54	0.57	0.50	0.45	0.35	0.63	0.61	0.56	0.60	0.075	0.016	0.005	0.003	0.0015	1		
rs2001625	11	130.14	ACGGTGAGCCACAGGACACCTGCTGTAGGCATCGAATTCCTGAATGGTGGGAC[A/G]CTGTGTGTCTCTGGCCCTATTCCCTCTCCAGGACAAACCTCACCCTTTCCTGCAAAT	A	G	0.83	0.42	0.43	0.35	0.52	0.45	0.42	0.36	0.50	0.49	0.46	0.113	0.000	0.001	0.000	0.0016	1		
rs1944142	11	130.26	ATTGGGATAGGCAGTGTAAAGGAGTCAGAGCTTCTGTTCATCAGAATCCATAC[A/G]GCAGAGGTGGGTCAGCCTTTTGGCACTCTAGGGCCTCCAAAATCCTTTTCAGCCCTG	A	G	0.79	0.53	0.52	0.49	0.61	0.55	0.44	0.59	0.46	0.45	0.48	0.025	0.016	0.000	0.003	0.0017			
rs965493	11	131.18	TCTGGGCTGCTCAGCGGGGACTTCTGGTCTCCTCTGTCAGGGGACAGGCAAATCG[A/G]GTGACTAGAATCCTGCTACCTGGTTAGCTGGATAAAGGATTTAATAAAACAAATG	A	G	0.79	0.50	0.49	0.45	0.57	0.23	0.20	0.42	0.46	0.54	0.44	0.055	0.039	0.004	0.034	0.0020	1		
rs3345	11	131.38	TCTTAACTCTGCCTCTGAGCTATGTGACCTCAGTGATGGTCCTTAATCTTCTATCT[A/G]TATGTAAAACTGATATATTAAAATGACCATCTCAGCTTGAGCAACATTCTGAGACCC	A	G	0.40	0.58	0.63	0.60	0.59	0.18	0.48	0.63	0.43	0.59	0.55	0.017	0.006	0.003	0.085	0.0033			
rs2044727	11	131.68	CTGCATTCCAAAGCAAGAGGGGAGAAAAGCAAAATTGAATCTGAAGGCAGACTCCATGTGACCACTTTAATTAAATGTAAGAGTGG[A/C]TGCTGTGTGAGGCAAAAGGAAAAAA	A	C	0.36	0.32	0.26	0.23	0.16	0.18	0.16	0.17	0.21	0.33	0.40	0.003	0.008	0.013	0.005	0.0092	1	1	1
rs570969	11	132.53	TCTAAAATATTTGAGATTACAAAACGTCTTCTATTTGACAAGTGGTTTCAGAAA[A/C]AATGCCCCAAGCGACTTCTAATGTTAACATTTCTGTCCCCAGGAAAGTGATCATTTATA	A	C	0.21	0.51	0.51	0.57	0.55	0.45	0.70	0.57	0.50	0.51	0.56	0.042	0.008	0.013	0.002	0.0002	1		
rs475056	11	132.58	TTAATATCTGTCTCTACTTGTAGACCCCAACATTCTTGGAGTCTGAGACTCAT[A/G]GAGAGGGTATCGATAGGAGTACAGATCCCAGTATGTTGCACACTGTAAGAACTTAAAAG	A	G	0.52	0.39	0.46	0.49	0.50	0.55	0.90	0.29	0.32	0.51	0.50	0.000	0.079	0.034	0.000	0.0001			

rs2287570	12	113.87	CATCTCCTTTCATTTGTCAGATCAAATGTAGAATAAGGGAGGTTTAAGGCCCACC[A/G]TGAGTTTCCACAACAGCTAATATTTGGAGGAATTATAAGTTCTGGAATGACAGCCA	A	G	0.57	0.43	0.45	0.40	0.57	0.50	0.16	0.31	0.43	0.33	0.51	0.003	0.068	0.015	0.000	0.0013	1		
rs2141693	12	114.19	AATTCCATCTCGAAGGTTAATTCTCTACCTTGCTCTGCCCTCTTTGTGATGCAC[A/G]CAGACTATGAAACTCATGTTCCCTTACCCCGGCTTTCAGTTGGGTTTGACCAATGAT	A	G	0.50	0.92	0.96	0.90	0.96	0.91	0.93	0.97	0.86	0.93	0.92	0.164	0.016	0.000	0.017	0.0008			
rs2008370	12	114.74	AGAAAGCAGAGAATTCTTCCCCCCCCCCCCGCACCCAAAGAAAGGAGTGTAG[A/G]GCTGTGTAAAGAGGAAGGAAAAGAAAGAATTCTAAGAGCCAAGTCTAAGCACCACC	A	G	0.33	0.59	0.61	0.61	0.64	0.45	0.72	0.60	0.57	0.52	0.54	0.039	0.015	0.000	0.007	0.0019	1		
rs1865788	12	114.76	TTATCCTTAGTAAAAATACCAGCTACTGTTGTCAAGGGCTTGCTGAGGCCAG[A/G]CACTGGGCTTTACATCCTTTTGTTTGCTGTTGTTTAATGCCTGCAATAGCTCTGGGAAG	A	G	0.14	0.39	0.44	0.38	0.46	0.45	0.40	0.47	0.32	0.44	0.43	0.069	0.001	0.001	0.000	0.0001			
rs4767387	12	115.05	ACCACAGAGGTCCTCTGGGTGTTTCCACAGAGCAAAATTGAAAACAGGGCCAATAA[C/G]TAAGGTGCAATTAACAAAAAAAAAAAAAGTCTGACTGGCCCAGTCTTCGGCTTC	C	G	0.31	0.05	0.04	0.04	0.04	0.36	0.68	0.14	0.04	0.03	0.02	0.062	0.233	0.010	0.082	0.0019			
rs2657297	12	115.35	TGTGGAGCGCTTTTCAATTGTCTCCATTCAGTTCCTCAAGTGGATCTCGTATGCC[A/G]AATGAGATCTTTATCATTTGTCACAGGGGTGATGCATGCTGGGCCTGCTACCGTGG	A	G	0.50	0.55	0.57	0.56	0.59	0.68	0.86	0.47	0.43	0.65	0.56	0.003	0.044	0.012	0.005	0.0003	1		
rs728861	12	115.50	AAGATTTCAACAAATATTTGTGCCAAAACCCTTGCTGATGCTGGAGCTGGGGATA[A/G]GACTCGCAATCTAATCAGAGAGAAATGCTTCAGGCGGCAGCAACAGGGAGGCAGC	A	G	0.52	0.40	0.38	0.27	0.27	0.59	0.73	0.30	0.27	0.38	0.33	0.012	0.063	0.007	0.026	0.0018			
rs903770	12	115.70	GGGCCTCTGTGGGGCACTTACAGTGCCCACTACAGGGGCTAACTGGAGGTTTTGC[A/G]GACCAAAAATGTATAGGCTGATTCAAGAAGGGTTTTCCAGGGCGCCAGATTATGA	A	G	0.17	0.75	0.75	0.76	0.79	0.45	0.90	0.46	0.75	0.71	0.69	0.196	0.007	0.094	0.060	0.0032			
rs2393111	12	115.79	CTGATGCTTGCCATCCATCCAAAAGTTGAGTCTCACTTCTAATGAAGAGTGGTAGC[A/G]GGTAGGTTACAGTACCCTGTACTCTTTAACATCCCTTAGGGCCACTTCAAGACTC	A	G	0.36	0.85	0.89	0.87	0.84	0.77	0.95	0.80	0.82	0.85	0.84	0.163	0.004	0.007	0.012	0.0023			
rs885487	12	115.95	GATCCTTGGAGCAGCACGAAGGCAGAAAAAACCAAGGCACAATTTATGCATTC[A/G]TTCAATCATTTATTCATTGCACATTTATTGAGCACCTACTATGTTCAGGGCTCTTGCT	A	G	0.43	0.52	0.45	0.45	0.45	0.86	0.91	0.71	0.57	0.49	0.47	0.000	0.115	0.028	0.100	0.0000	1		
rs1995257	12	116.27	AACAAGATCTCACTGGGGAGTCCTACCATATCCAATGGCTTCTGATCTAGCCCC[A/G]TGAGCCATCTTACTACGTTTACCTCTGGGTGTGAGTGACAACTGGCCCTAAGTAGA	A	G	0.95	0.90	0.91	0.95	0.89	0.86	0.96	0.94	0.86	0.93	0.91	0.001	0.000	0.007	0.008	0.0000			
rs1530541	12	116.64	TAGAACCAGAAACTTCTCTTCTACTTCTCTCCCCATATTTCTCTCTACTCCCAAAA[A/G]ACAGATTGCACAGCACTATGCCCACAATGAGTGCCCACTGCATAAATACATGGAGC	A	G	0.29	0.57	0.57	0.45	0.43	0.18	0.45	0.26	0.50	0.47	0.47	0.036	0.006	0.046	0.067	0.0055	1		
rs714323	12	116.78	TGGGTGGGGACACAGCCAAACCATATCAATGCTGCTATGAACATTGGTGTACAA[A/G]TATCTGCTTTCAAATCCTAGAGTATATTCCTACAAATATTAATGCTGTTTCTAACCTG	A	G	0.69	0.60	0.60	0.70	0.61	0.86	0.96	0.74	0.50	0.55	0.56	0.002	0.083	0.002	0.029	0.0004			
rs4590911	12	116.97	GTAAAGGCAAAAGATGTCTTCTGCAAGTAGCAAATGAGTGATGCACACACCAATGCA[A/G]AGAGCTTGTTAATCAGCCTAAGAGAGCAGCATCTAAGTAGCAGGTGAGTAAGC	A	G	0.31	0.52	0.48	0.43	0.59	0.18	0.56	0.32	0.25	0.39	0.44	0.025	0.000	0.024	0.076	0.0015			
rs404566	12	117.13	TTGTTGATACAATAACTTCCAAAACAAATATGGCCCATTCTACTGTGTTTCTTATT[A/T]TCAGTATGGAATGCTCATTCACCATTAGGCACGTTTCTCTACCTGTACCTAGGA	A	T	0.28	0.40	0.39	0.48	0.41	0.23	0.30	0.40	0.39	0.40	0.46	0.004	0.001	0.007	0.009	0.0083		1	1[g]
rs7295288	12	117.27	TATCCTCCTTAATCATGCACCAAATGAACAATATCCTCAATCACAAGTTTATTTCAAG[A/G]TTGTTTTTCCACCCAAGATCCATTCTATTCACTTGATTTTTAATGACTTTTATCTGC	A	G	0.69	0.87	0.84	0.89	0.84	1.00	1.00	0.96	0.86	0.78	0.77	0.010	0.080	0.041	0.080	0.0040			
rs2730550	12	117.47	TTGAACTGCTGTATAGGCCTTCGTTTTCAAACTTGGAAGAATGATATTGAGGTAA[A/T]TTGGAAAGCATGTTCTTTCCCTGTTGGCATCTGTGATGGTTCATATTGAGTGTCAATTG	A	T	0.62	0.75	0.80	0.77	0.66	0.50	0.49	0.61	0.75	0.72	0.78	0.032	0.084	0.031	0.079	0.0015	1		
rs2730753	12	117.58	GTGTGGTCCTCCTGTGGGGCAGGTGATGGATAAACTATGTACACCGCTAGAAAAG[A/G]CAGTGCCAATTTGAGGGAGCTCTGAGTGCCACAGGTGGGAAACAGTGCTGTGG	A	G	0.33	0.52	0.44	0.40	0.52	0.50	0.75	0.57	0.39	0.55	0.51	0.014	0.030	0.001	0.000	0.0011			
rs1568923	12	117.83	CGCTGTCCAAAGTGCTGAAACGTGGATTCCAAACGGGGGCAGCACCCAGCTCC[C/G]GGCTCTGTCCGGGTGAAACCCAGAGAGGGGTGGGGTGGGAGAGATCGGAGTTCA	C	G	0.69	0.52	0.60	0.70	0.55	0.73	0.79	0.65	0.50	0.71	0.65	0.004	0.032	0.008	0.007	0.0010			
rs1426626	12	118.10	ATTTTGAAATTTTTGTTTCACTATCAAATCATTTTGTATTTTAAAAAAGGTGGAAA[A/G]GGCCTGGGGTCTTCACATTGTAGAAGAAAAGAGACTACTAGGGGATTTTAATGCAA	A	G	0.36	0.38	0.42	0.35	0.36	0.77	0.91	0.21	0.36	0.47	0.42	0.001	0.140	0.032	0.069	0.0005			
rs477467	12	118.34	CATTTATTACCCATCCCCTAGATCTCCTCCACCCCCAAGTCATCTCTTACACCA[A/G]GACCCAGAGAAACTAACCTCAAATGCAACCAGCAATATCATGCATCCATTGATAGAT	A	G	0.67	0.33	0.32	0.39	0.46	0.73	0.75	0.64	0.43	0.49	0.44	0.069	0.088	0.054	0.084	0.0068		1	1
rs4213	12	118.53	CCTGGGCTAGTGGGTACATCTGTGGCGACTCCCGGGAAACTGAGCTGCGTATA[A/C]AGAAGTCTGCTTTGGTCCAAAGGACATCTTCCTACCTGTACCAGACTCAGAAGGCTGCTG	A	C	0.58	0.72	0.69	0.66	0.66	1.00	0.93	0.78	0.79	0.68	0.67	0.003	0.059	0.008	0.138	0.0001			
rs2422	12	118.98	CCCCTCCCCAGACTTGGGAACTGCCAGAGCTGTGAATCATTTTCATCTGACAGGG[A/G]TCAGATCCATTCAGACCTCACAATATTGAAAAGCCAAGCCAGACACACCACACTGC	A	G	0.53	0.59	0.62	0.62	0.68	0.32	0.91	0.66	0.46	0.55	0.55	0.002	0.077	0.008	0.031	0.0021	1		
rs610694	12	119.72	ATTGCTAAATCTTAGGAGTACATTGTAGAGTTTGAGAGATAATGGAGGTTACCT[A/G]TGACTGACTCATGACTTAGGATGGATTATCATCAAGCACTAATGTTTTAAAAAACGT	A	G	0.45	0.52	0.50	0.54	0.45	0.41	0.86	0.53	0.61	0.52	0.55	0.005	0.050	0.000	0.010	0.0009	1		
rs1179992	12	119.91	AATGGTGCGCCTCCCATCAAGACAGTGCCTTTCAATCAGTAGAAGAACTTTATTAT[A/G]ACATATGTTAAAGTACTTGAACACCCTCGAACCCATTCTTAAGAGAATACGCATTC	A	G	0.90	0.33	0.27	0.24	0.43	0.14	0.05	0.33	0.27	0.22	0.27	0.216	0.051	0.000	0.020	0.0000			
rs169631	12	120.00	TTTTAAGATGAGAAATGATGTTTAAATGCAGATGGAAATGGGACAGGAGAGAAA[C/G]GGAAGTTGATGGGATAAATGAAAATTATCATCATCATCTTCCTCACCATCACCAGCA	C	G	0.07	0.73	0.72	0.80	0.54	0.68	0.92	0.54	0.57	0.84	0.78	0.267	0.028	0.013	0.002	0.0016			
rs1064951	12	120.29	CCGCTGCTCCGGACCCTGGATGTCCAGTGGGTGGAGGGACTAAAGGATGCCCA[A/G]ATGCGGGATCTCCTGTCCCCGCCCACAGACAACAGGCCAGGTGAGTTGCCAGGC	A	G	0.24	0.41	0.38	0.34	0.32	0.45	0.53	0.40	0.61	0.23	0.37	0.009	0.010	0.000	0.003	0.0000	1		
rs1473553	12	121.42	GGCCAGAATTCCAAGTG[A/C]TTTCTGTCACCCGCCGGCGGACCGAGAGTCTAGGGTTCGGGGCGCGCAGGCTGCCTCGGCGCAGACCTCTTCCCCTCAGGGAGGCCGTCTC	A	C	0.95	0.75	0.79	0.68	0.75	0.82	0.63	0.84	0.75	0.73	0.81	0.031	0.017	0.002	0.000	0.0004	1		
rs1790094	12	122.02	AGCCTTCTCCCTCCTGCTCTGGGATGCAGCACTCTAACACACCTTGCTGGGG[A/G]CTTCCTTGCATTTCTGTGGCAATTCTCAGGTGTAGGCAGAGTCTTGTGACAACTGAGG	A	G	0.88	0.30	0.27	0.37	0.23	0.32	0.47	0.48	0.43	0.36	0.33	0.201	0.014	0.013	0.000	0.0017	1		
rs989892	12	123.64	GCCTGCATGGACCCACGCACGTGTTTCTTCCAATGTTATTACCAAGCACGCCGAG[A/C]GAGATCTTCCCAGCTGTAAGTGGTAATTAATTTGTAGCTTTATGGCATATCTAGCC	A	C	0.74	0.49	0.57	0.54	0.57	0.86	0.92	0.54	0.25	0.53	0.53	0.021	0.082	0.000	0.062	0.0005	1		
rs4765621	12	123.67	ACGTGTGATGTCCCTGAGCTCTTCCTACTGGGTACCTGAGTCACTTGTGAATTGC[A/G]TGGTCCTCTTCCCCACTTCCTGCTCAGTCCACGAGGGCAGGAATCCTGTTGGATTA	A	G	0.43	0.29	0.33	0.34	0.38	0.45	0.80	0.32	0.32	0.38	0.42	0.002	0.105	0.000	0.006	0.0067			
rs2070628	12	123.75	TAGTTCAAAACCGGAGCTTCAGCTACTTAAGAAGATAGGTACATAAAACCGACCA[A/G]AGAAACTGACGCCTCACTTATCCCTCCCCTCACCAGAGGTCCGGCGCCTGTCGAT	A	G	0.81	0.99	1.00	0.99	0.98	0.64	0.46	0.93	1.00	1.00	0.98	0.071	0.237	0.024	0.146	0.0032			
rs7132145	12	123.85	TTCATCAGGGGAGCAGCTGGCGCGTTCTGGAGGCGAAACGAGCTTGCTTCCGTGTC[A/G]AAGCTTTTCTAGTTCTGTTTGATGCGTAAGCTTCAAGGCATCCGAAGGCCTTTG	A	G	0.24	0.11	0.16	0.20	0.25	0.27	0.33	0.38	0.07	0.15	0.23	0.015	0.022	0.068	0.021	0.0120		1	1
rs922873	12	123.88	GTTCCCATGGTGTTTCT[A/G]TGACTGTTATGCAAGTTGTTGGACTTAGTGCAAATTATAAAACATTAAATGTTTGCCTCTAATTCTTTAGGAACTAGGTTGTGAATTTCA	A	G	0.67	0.78	0.77	0.72	0.79	0.86	0.94	0.90	0.79	0.68	0.77	0.004	0.035	0.026	0.010	0.0001			
rs2197777	12	124.18	TTGGATCAAAAGATAGAGGAGGGAGAAATGATTTTGAAGAATTAGCCTCATCT[A/G]AAGTTTTAGCTGCTGCCGCCACCAAGGGGCAATCTGTATTTTGACAATGTCGAGCAAT	A	G	0.64	0.68	0.68	0.72	0.75	0.77	0.53	0.54	0.86	0.71	0.60	0.000	0.004	0.006	0.011	0.0026	1		
rs999702	12	124.27	ATGCTCCCTCCCAGGCAGCCCCTCCTGAGTGGATGGTGGGTGCAAAGACATCTGT[A/C]TTGAATTTTAGGTGTTATCTGAGTCTCTCTCTTTTGTAGAAGCTTATAGCCAAATC	A	C	0.76	0.39	0.42	0.48	0.29	0.64	0.53	0.50	0.29	0.49	0.52	0.062	0.008	0.005	0.025	0.0049			
rs6489020	12	124.65	CCTTTTGACATAAAGTTAAGTGACTATATGTGAGGCCTTATTTGAGTTTTACACCT[A/G]CTTAAACATGTCTTCCCCACCTTCCTTCTCTCCTACCCTAATAGTACACTCTGGAAA	A	G	0.63	0.49	0.48	0.55	0.61	0.59	0.82	0.59	0.57	0.60	0.59	0.008	0.053	0.000	0.002	0.0070		1	1
rs225553	12	124.79	TACTTACTTCTGTTGGTGAAAATTGGAATATAAGTGTTGCGGAAGCAAAGAAAAGA[A/C]TTTCTGATGGCAAAATATATCCTTACACTAACAACTGAAAGTCTTGCTGAAGAATCT	A	C	0.19	0.06	0.08	0.05	0.11	0.05	0.01	0.07	0.04	0.14	0.13	0.020	0.006	0.007	0.000	0.0055			
rs918044	12	125.23	CACATCTGCAATTATATTTGGACGAACAAATGCTGAACATGTCCAGTTTCACT[C/G]CACTTCCCCAGCACCAACGTCCCCATCATTTACATGCAATTTCCGTCCCAGCATCAGCA	C	G	0.60	0.49	0.48	0.45	0.43	0.68	0.98	0.62	0.42	0.46	0.52	0.003	0.196	0.018	0.020	0.0024	1		
rs2058290	12	125.43	CAATGACACTTTTAATCAGTAGAACACATTACATGGCCACACAATTACAAAAATGGC[A/G]CAAATGCTGATATCAAGAAGGTCATCCTTGTAGTCATGGTCAGAGCTTAGCAAAA	A	G	0.12	0.14	0.14	0.13	0.18	0.00	0.01	0.03	0.08	0.15	0.16	0.000	0.033	0.012	0.050	0.0001			
rs7133178	12	125.57	ACACACCTTATTCCCAGAGAGATTGAAACATAATCACCAAAGTCAGATGAAAATAT[A/G]CCTTCAATCTGCAGCTCTGGAGAGTCATTCCATTCCACAGTCTCTCTGTGAAAA	A	G	0.33	0.11	0.11	0.12	0.16	0.09	0.19	0.11	0.12	0.10	0.11	0.047	0.020	0.005	0.000	0.0000			
rs1672299	12	125.78	TCTTATTTAAGAAGCTTATAATGCTCTATTTATGAATAACAGGCTATGTGGGG[A/G]ATATTTCCCACCACTTCACGAATTATTTTTTCCAGGTACTTTCTTGCAAAAATCCTACTTA	A	G	0.64	0.55	0.55	0.57	0.50	0.41	0.32	0.48	0.42	0.43	0.51	0.002	0.027	0.002	0.012	0.0010	1		
rs6489151	12	125.92	TGACAAAAAAAATACTATTTGAAGAAGACACATTAAACCTGCAATGACCTAAATCA[A/G]TGGTGGCTTGGGGTGCTTAGACATCACATAACATAATTGGGTAAAGAATAGGAGGA	A	G	0.69	0.67	0.64	0.66	0.61	1.00	0.83	0.78	0.75	0.65	0.67	0.003	0.017	0.006	0.140	0.0008			
rs2712530	12	126.05	CTGCAAGAATTAGTGAAAGTTTTGACTTTCCAGGGGAGGAAAAGGGTGGCTCTTAG[A/G]AAAATAAAGTTTCACTCCAATGTGAAACAATGTGTGACTTTAGGAAATTCAAATAC	A	G	0.64	0.34	0.36	0.37	0.38	0.15	0.89	0.35	0.46	0.53	0.42	0.050	0.152	0.000	0.034	0.0026			
rs1910780	12	126.23	CTACAAGGGATGCTTGGTCCTGTAAGATCAAATTTTCAATTTCTCTTTGGGCTGC[A/G]CTGTGATTTGTAACCAGAACTAGGGAACTGCTTCCAACACCTGCCAGGTTCTCAGC	A	G	0.60	0.76	0.76	0.65	0.79	0.50	0.52	0.60	0.79	0.59	0.66	0.007	0.016	0.002	0.024	0.0038			
rs1388149	12	126.49	TCTGCTTACCCATAAAGACCATGGCTTTCAAAAAACGTTTAATTTTCCCTTTTAC[C/G]CCTGACCACACACAGCACAGAAGCCTGGGGAGGGGCATCACTGCCCCCCAAGAGTG	C	G	0.24	0.24	0.34	0.39	0.30	0.14	0.54	0.39	0.32	0.35	0.34	0.008	0.018	0.000	0.029	0.0004	1		
rs866781	12	126.71	ACAAACAAACACTCCAAATTTTAAGAAGAGCATATTGTGGCCATTTTGGAAAAGACA[A/G]TCTGCCATAGTAATAAGATTCATGATTGTCAGAGTTAACATTCATCACATGCTTC	A	G	0.24	0.01	0.00	0.00	0.00	0.05	0.00	0.03	0.00	0.01	0.00	0.091	0.000	0.018	0.016	0.0000			
rs6489226	12	126.89	GATTTTACAGTTGGGGAAACTGAGGGTCGGAGTGTGTAAGTGGCTACCTTGTAA[C/G]TAGGCTTGCTTGGACTGTGAGCCCAGCTCTGCAGATACCCAATTCCCATGCTACGT	C	G	0.07	0.47	0.50	0.52	0.43	0.41	0.16	0.54	0.50	0.50	0.50	0.152	0.090	0.000	0.012	0.0002			
rs952093	12	127.20	TCAGAAACAATAAATACCTGTGGAACAAAATGCAGGCACAACCTCCAAACCCACCAC[A/G]GTGATTGTCATTCTACCTGCAATTTAACAGAATTTAAATTTAAAATTTTAAAAATT	A	G	0.79	0.89	0.87	0.90	0.82	0.68	0.67	0.91	0.92	0.86	0.83	0.002	0.011	0.002	0.014	0.0001	1		
rs897978	12	127.34	GTGACTGGTTAAATGGGACTGAATGAGAAGTACTTGAGGATCAGAGCAGATGGA[A/G]CATTCTATCTACATGTGTTTCTATTCAAAGAAAGGGAGCATCCCAGCCCAAACTC	A	G	0.60	0.52	0.52	0.49	0.61	0.64	0.51	0.31	0.43	0.56	0.55	0.009	0.001	0.010	0.020	0.0004			
rs7963526	12	127.59	CCTGGAAGGTGAGCTGTAATTACAGATCCTAGAATGTCATGTGATGATTTTTACC[A/G]AGAACACACATTCCCCAGGTCCCAGGTTACTGACACATAAGTCTACTGTTTGGCTT	A	G	0.40	0.40	0.43	0.44	0.44	0.36	0.10	0.33	0.32	0.44	0.46	0.001	0.099	0.037	0.011	0.0001			
rs1465727	12	127.76	GACACACTGAGAAGGGAGAAACATCACTTCCCCCATATTCTTGATGTGCATGGAAT[A/G]TTCATTCATATACACAGAGGGGGAAGAGGAAAGGAAGGTACAAGGAGAA	A	G	0.50	0.66	0.69	0.63	0.66	0.27	0.53	0.36	0.69	0.59	0.63	0.017	0.014	0.050	0.100	0.0013			
rs621830	12	127.90	CCTTCCTCCTCAGATTAAGCTTACTTAATGGAAGCCTTCCCCTTAACCAGGTTTC[A/T]GCCAGTCATATTCCATACAGTTGATAACTCCAGTCTACTGGTAAGTAGACATTTTCC	A	T	0.19	0.23	0.22	0.28	0.30	0.32	0.23	0.25	0.27	0.33	0.26	0.003	0.001	0.001	0.004	0.0014			
rs1486629	12	128.01	GGCTCCCAAGTTCAACCAAAGGAAACTATGACCAGTGTGTTCACTGGAAGGGAGC[A/G]CACATATGTTGGAATTACTCCAGACTCCACTTGATGTTTTGCAAGGGTGAGGAGT	A	G	0.71	0.67	0.64	0.63	0.71	0.50	0.31	0.78	0.82	0.76	0.68	0.006	0.035	0.026	0.006	0.0003			
rs7134835	12	128.26	GACAGCAGGACATGAAGAGAGAAGGAGCTGCCATGCAGACCCTTCGAGAAAATGA[A/G]TTCTTCCTCTGATTTGAGTTACAGGCAGCTGAACACAATCTCCATCTAATACAA	A	G	1.00	0.64	0.66	0.74	0.61	0.41	0.49	0.78	0.75	0.68	0.62	0.131	0.018	0.006	0.040	0.0027	1		
rs1543045	12	128.43	GCATTTCTCTGCACCTCCCAGTGCTTTAAAAGGTGTGAAGCACAGGCTTTATCTC[A/G]GCACAATGCCAATTTATTTTAGTCTGCAATACTGAGTATTGAGCAAGTAAAGGAAA	A	G	0.71	0.94	0.91	0.94	0.89	0.91	1.00	0.92	0.93	0.93	0.94	0.042	0.010	0.010	0.000	0.0013			
rs896511	12	128.56	CAGAAATTCGGAGACGATGACTGGGGAGGTGTGTCTGGAGTGGAGGTCTAAT[A/G]TAGGAGGTACATCTGCAGCCCCTTTCTGTAAACCAGATTGTGCCAACGTCTCCCTAG	A	G	0.40	0.65	0.54	0.60	0.64	0.36	0.10	0.54	0.42	0.60	0.66	0.009	0.113	0.000	0.013	0.0072		1	1[g]
rs2047709	12	128.71	GCAACTGAATAGAGGAATTAATCCTATTTGTGTCACCCGCTGTGACACTGAGA[A/C]GTCACTAAACTGGAAAGTGATCTTTTATTGGTCTGTGATTGAGGTTGGTGGAAGGGA	A	C	0.52	0.79	0.88	0.85	0.84	0.36	0.80	0.97	0.73	0.84	0.86	0.069	0.008	0.017	0.168	0.0007			
rs826716	12	128.89	CAAAGAGTTTTGTGTGGGTTTTGGGGTTGCTTTTCTCTGTTTGCTTTAATCAC[A/C]CTATGCCCGGCGTCTAGATCAGGGTCGGCATATGCATTCTCTGCATTCTCTGTCCCTG	A	C	0.31	0.24	0.24	0.26	0.27	0.05	0.28	0.19	0.29	0.26	0.26	0.010	0.012	0.000	0.023	0.0001			
rs4760075	12	128.98	GCGGGGCCACAGGGTCAGTGAGAGCCGAGGCATTCCACACTCTGCTAGTTTAGAA[A/C]CACAGGCAATGAGACCCAAAGACCTGACCACTCAAGCCCTTCCATTGCTCTAC	A	C	0.17	0.23	0.25	0.30	0.18	0.73	0.78	0.26	0.32	0.26	0.28	0.002	0.181	0.002	0.132	0.0031			
rs6486532	12	129.09	CAGTCTGTGTCAGGTGTGCTCAGCCCAATACTCTTCCATTTTAGTGATTGGCAGGAA[A/G]TGTCAGCCATAAAATGCTAAAAATGGGTCTGAAAATCCCAAGCTAAAGAAATCATA	A	G	0.79	0.56	0.63	0.46	0.71	1.00	1.00	0.76	0.64	0.64	0.61	0.008	0.146	0.007	0.146	0.0001	1		
rs428715	12	129.09	TCATCCCCCCTGGGGCGAGGAAGATGTAAGAGACTGTTCTGGGGCCTCAATTT[A/G]TCAGTGTTTGCATTCCTACTAGCTGAATTAGCTGGGATTCTGGAGTGTGGAGCC	A	G	0.81	0.84	0.80	0.82	0.79	0.73	0.88	0.89	0.92	0.81	0.84	0.000	0.004	0.005	0.005	0.0013			
rs1487602	12	129.46	GCCAGATCCTTGTACAACTGGGTGCAGCTAAAATCTCTTCTCCATCCCGTTC[A/G]TGCACAGTCTCCCATTTACATTGCTTTATGTTGTTGAAATTTTGACTTTAAGATTATTTAC	A	G	0.55	0.45	0.45	0.45	0.54	0.59	0.70	0.53	0.25	0.41	0.44	0.000	0.010	0.001	0.001	0.0010			
rs1195569	12	129.58	GATTTAGCATCTATGGCTGCAAGAGATAGGAATTCTAGAAAATGATTCCCT[A/G]ATTGTCAAGACCTCACAAAGAGGTGGAAAAGAAACATCATCTCACGGTCAAGCGGAACC	A	G	0.48	0.54	0.51	0.52	0.39	0.41	0.35	0.25	0.71	0.53	0.51	0.000	0.009	0.032	0.002	0.0014			
rs1545610	12	130.67	TTTTCCAGTTTAGCATGGCTTTAGGTTTTCTGAGAAGGTGTGAAGACTGTAC[A/C]GAGTCCTCCCAGCTCCTGTCCCGTTTCCCTGTCACTAACCTCTTTCATTACCACGGTACA	A	C	0.74	0.56	0.57	0.54	0.61	0.82	0.72	0.78	0.57	0.62	0.57	0.021	0.016	0.034	0.046	0.0000	1		
rs737434	12	130.72	GATGCCTGTGAGCAGGCAGTGAGGGTGAGTCTTTAAAACCGTATGGGCATTTTT[A/G]CCTCTGAATTATTCGCACTGCAGATCTTTTTCTACTAGACTATCAGAATTTTTTCC	A	G	0.40	0.08	0.12	0.09	0.11	0.09	0.02	0.34	0.04	0.15	0.19	0.071	0.016	0.057	0.001	0.0048			1
rs1278601	12	131.74	CTCCCCCCTGAGGCTGTCTTCTAGAGACTGGGGTTTCTTACTAGGTTTGAACAGT[A/G]GGAGATACCAGAGGTGGGAGGAGAAAAGTCTTATTACCGTTCCCTTCCTCCTG	A	G	0.31	0.48	0.48	0.52	0.43	0.41	0.31	0.41	0.32	0.47	0.51	0.019	0.025	0.006	0.007	0.0001	1		
rs1278602	12	131.74	CAGAGGTAGTAAAAGACTTCATAAGTCTTTCTATTACTGAAGAGGAGCTTAGTAATAACT[A/G]CAAGTCTATTTATATATGTAGCAGATGGTAACAATCTCCAGA	A	G	0.29	0.48	0.48	0.52	0.43	0.41	0.31	0.41	0.35	0.47	0.51	0.024	0.023	0.006	0.007	0.0001			
rs4758930	12	131.82	TGACAGGGCTCTGCCCTGCAGTTCCATGGTGTATGTGATTCTTTCTCTCACTGC[A/G]TGCAGACTCACCCAGAAGTCCACTCACTGCCTCTTCCCCTGGGGTCTCCCTCCCTG	A	G	0.29	0.03	0.04	0.02	0.04	0.00	0.00	0.00	0.04	0.04	0.02	0.054	0.013	0.013	0.013	0.0016			
rs937538	12	131.85	TCATCTGCTATCTGATTAGGTGGTGGAGCTTCATCGTCAAAAGACCAATCTAAAAT[A/G]TGGATTCCTTCTTTTTCAACTGGAACTTTACCAAATGTAGCATCACAAACTCAAAC	A	G	0.43	0.57	0.57	0.51	0.54	0.77	0.84	0.44	0.46	0.60	0.55	0.017	0.029	0.013	0.013	0.0006			
rs595241	12	131.89	GAGAGACAAAAAGAAAAAGCCCATTATACAAAATGGAAGAGGGCAAAAAAAGAG[A/G]AATTCACTGGCCTGGTGAAAAGACATGAGACATGAAAAAAAAGTCCTTGTGAAGG	A	G	0.19	0.51	0.48	0.43	0.48	0.77	0.81	0.44	0.46	0.53	0.44	0.068	0.031	0.005	0.025	0.0050			1
rs632610	12	131.90	CTCACAGGTGTCTTTTTTGTTCCC[A/G]TTGTAGTTCTGTCTGGTCTCTGAATTCATTTTCTTTTTGTTTTTTCTTTTCTTTTCTTTCTTTTCAAATGGACATACATTTCTTATTGCCTGAA	A	G	0.81	0.49	0.51	0.57	0.52	0.20	0.19	0.55	0.57	0.47	0.56	0.068	0.033	0.005	0.033	0.0049			
rs1025	12	131.92	AGATTGGAGAAAGCCAGAGATTAGCCCTCATTCCGCATCTGTCAACCAGGACAGAA[A/T]GCATGGACAAGGGATGAGCTTTACAAAGATGATGCACTTTGGAGATCAGAAAAT	A	T	0.21	0.44	0.40	0.41	0.46	0.77	0.81	0.44	0.42	0.40	0.41	0.032	0.065	0.001	0.056	0.0006			
rs2270928	12	131.97	CCAGAACTTCTAGAGATAGTGGTCACTGGCCCTCCTCACAGGCCCTTCTTCCTGG[A/G]AAGACTGAGTTTATCTGGCCCCTGTTTCCCCACTGCCAGTCTTTACATTCCATTTG	A	G	0.14	0.33	0.28	0.35	0.43	0.09	0.17	0.28	0.33	0.27	0.30	0.006	0.001	0.007	0.012	0.0019			
rs7960480	12	132.02	AATTTTTTGTTACATTGTAGAAAGCTTTGTTTTTGTTTCAGTTTGATTTCTCTG[A/G]ATGGTTTTAAGAACTGCTTCTTGTCCTGTTAAGGAATGATGTTCCCTCTCAGTTTATTTT	A	G	0.40	0.35	0.32	0.39	0.46	0.09	0.17	0.28	0.35	0.30	0.32	0.010	0.002	0.004	0.017	0.0011			
rs1838114	13	17.29	CCATGATTTTCCTTTGGAGACTCCAAATCCTCAGTCCAAATGACTTTATTCCACTT[A/G]TAACTCCATAGAAGCCATGAAATCCAGACCTCAGTACAGACGGTGACTTCCATGAA	A	G	0.63	0.41	0.47	0.55	0.36	0.91	0.63	0.41	0.46	0.48	0.38	0.014	0.018	0.000	0.125	0.0047	1		1
rs1867248	13	17.33	GTGGGAGGAGGGTGGGCAGGTGACCATGTGTCCTGGGAGGAAAGTGGGTCTGCG[A/G]GTGGAGGACAGATGGCTCATGGTGGGATGACACTGTGTGAGCACAGAAAGGCT	A	G	0.29	0.23	0.27	0.30	0.20	0.68	0.93	0.27	0.30	0.26	0.24	0.008	0.298	0.006	0.120	0.0001			
rs1334958	13	17.34	GTGAGTCACACAAATTTTTTGGTTTTCCACTGCATATAAAAGTTACGTTTACACTAT[A/C]TCTTCAGTCTATTGAGTGTGCAATATCATTATGTTTAAAAACCTAATTTAAAATACTT	A	C	0.55	0.39	0.36	0.46	0.29	0.68	0.95	0.55	0.32	0.42	0.38	0.035	0.257	0.021	0.075	0.0007			
rs1157025	13	17.35	TTTTAAGTCAGGGGCCACAGGATCTGTTCTTGTGAAACTTGGTTAGGATAAA[A/G]GAAAAGGGGGCGCATTTGGCTCAGAACTCAAGAGGTGCTCCACAAGGGATGCCCCAC	A	G	0.67	0.55	0.53	0.56	0.45	0.86	0.98	0.70	0.43	0.52	0.58	0.022	0.204	0.027	0.105	0.0025			
rs1974047	13	17.50	TCCAGAGGATTCATATCATGAAAGCAGGTCTTAGGACAGAGTCCTGGTGCATGTC[A/G]CTTTATAAGGCTTGGGTGATAAAGGAAAAAGAGAAGCCCCAGATCTTCACTCTG	A	G	0.50	0.26	0.26	0.29	0.23	0.50	0.45	0.36	0.32	0.31	0.27	0.055	0.217	0.002	0.162	0.0008			
rs2006655	13	17.54	AGCACACTGCCAAGGGAACATTTTGAAAGGACACACACCACAGGTCCATTTAG[A/G]GAAAGGTAGCCACGGCCAGTTCACACCTGGCCTGGACAGCAAACCTGGTCTGCCCTC	A	G	0.14	0.21	0.20	0.21	0.20	0.41	0.44	0.31	0.25	0.24	0.21	0.001	0.038	0.008	0.034	0.0002			
rs1572372	13	18.64	CCATTAAAACTCCTTCTCAGAAGCAACCAAAGGAAAATTGTGGGTTTGTGTGT[A/G]TGTTGTTGTTTTGCTCTGGAGTTTGTTCTTGGAGTTGTTTGGAGGGGTCGTTTTTGCTTCT	A	G	0.21	0.45	0.43	0.39	0.50	0.41	0.27	0.45	0.46	0.32	0.35	0.013	0.006	0.004	0.001	0.0033	1		
rs7787	13	19.10	TTTTTTTTTTATCCTTCAGCAAGCCTCTTTTAATGATTTAAAAAAGAACT[A/T]CTAACTTTTACAAATAATTGTGTTCAGAGACAGACTTTCAATCTAAAGAA	A	T	0.67	0.50	0.47	0.46	0.40	0.59	0.91	0.73	0.46	0.48	0.57	0.016	0.097	0.047	0.003	0.0046			1
rs264729	13	20.00	CCAGCTTCCCACTCAGAGTAGGGGTGCCTTTTGAAATATCCTCAACAAATGGCC[A/G]ATCAGATTTTTCTTGAACATTTCCTGAGTGGAAAATGTAAGGCCTCCCAATGAAGCC	A	G	0.26	0.64	0.62	0.56	0.66	0.32	0.54	0.53	0.64	0.54	0.61	0.041	0.000	0.002	0.034	0.0005	1		
rs955783	13	20.10	GTGACTCTTGAAGCTACTTTCTGGACAATTGGCTTGAGGGAGGCAGGAGAG[A/T]GCTAATATTTACGGAGCATCTAGGAAAAATTGAACCTCCACTCGCTTATTTAAGGAGCT	A	T	0.29	0.48	0.39	0.34	0.50	0.20	0.05	0.30	0.58	0.44	0.42	0.002	0.087	0.004	0.014	0.0032			
rs292476	13	20.83	TGCTTATGTGGTTACCTGTGTCCCAAGGTGGGTCAGCCTGTGTTCAGGATCTCACA[A/G]AGTCTCTCATGAAAATAGTTGTGGGCTTCAGACTCATTCTTTGCTCCCAAATCAAA	A	G	0.62	0.76	0.80	0.83	0.89	0.55	0.30	0.65	0.68	0.83	0.84	0.018	0.100	0.022	0.026	0.0030	1		
rs4132778	13	20.89	AGAATTTTCTGCTGTTTGTTGTAAAGTACTGAAATGTTACGCTTGACTCTTCAAAG[A/G]GCAGGCAAAATCTTTTGGAGTATTTAAGGAATGAGGAAAGAAAAATATACATCCT	A	G	0.93	0.84	0.93	0.87	0.95	0.95	0.99	0.76	0.92	0.91	0.91	0.001	0.018	0.014	0.000	0.0019			
rs6490738	13	20.96	CAGCCCTCGGACAGCTCTGTCCTCTACTCCAGCAGGTGTAGACTTTGTCGTAGC[A/G]GAAAAGGCAGGGTGCTGAAATGTCTCCCAGTAATGAGCAACAAACAGTGTCTCC	A	G	0.64	0.73	0.76	0.66	0.77	0.91	0.93	0.83	0.79	0.73	0.76	0.012	0.032	0.004	0.020	0.0004			
rs475992	13	21.67	CTTGCAAACAAGTTAGAAAAGTATATTTAACTGCTATGATAAAAGTACAACTTCT[A/G]TTGGAGGCATAGGCAAATAAATATAAAATCTTCCCAGGGTAATCTGGGATGATACCC	A	G	0.55	0.38	0.43	0.48	0.50	0.14	0.15	0.41	0.32	0.40	0.40	0.008	0.043	0.001	0.054	0.0012	1		
rs1008814	13	21.76	CGGGCCCTCTCTTTCCATCTTGCATTTCCTATGTAATGGCCAAAGACACTTGGTA[A/T]GCCTCTCCCCAAAACAGACTATGGCATACTTGGACATATGGAGGGATGACAGTGG	A	T	0.48	0.42	0.51	0.49	0.55	0.86	0.56	0.65	0.64	0.52	0.54	0.001	0.002	0.002	0.080	0.0016			
rs7322794	13	22.86	TTTCCCAAACATGCAAAATTTCACATCTATAAACTCCTCTGAAGTCTAGGACAC[A/G]AATCTCTGATCTTGGCCAAATCCCTGCATGCACAGATGAGGGAACACGGACCAAGAG	A	G	0.93	0.83	0.87	0.90	0.84	1.00	0.99	0.88	0.79	0.90	0.90	0.004	0.034	0.004	0.052	0.0010	1		
rs8000533	13	22.99	AGCTTCCCGTGTGCAGATGTGGCTCCCCACATGCCTGTGTGATCTCTGGGTTA[A/G]AGGCCATGTCTCTATAGTCAATTACATGCACAAAGCATTTATGGGTCATCTAATACC	A	G	0.45	0.43	0.42	0.37	0.43	0.27	0.22	0.40	0.36	0.42	0.43	0.000	0.041	0.020	0.031	0.0000			
rs6490970	13	23.03	TACAAAGTTCAAATCTCAGTGGATATATTTCTGAGAAACTTTCTCTTCCTCCTTAT[A/G]CCCAGGAGTTTTATCTGGTTTGGGAGGAGCCATGCCTCTGACCTAGACAGGTTGTT	A	G	0.64	0.41	0.44	0.48	0.61	0.77	0.48	0.60	0.36	0.43	0.46	0.012	0.002	0.003	0.034	0.0002			
rs977655	13	24.10	CATATTTATTACATTTTAGACACCAGAGGTAAAAGCAGGACCAGTGTAATAGAA[A/G]GTGATCCCCACACATAGCTGAAACTGCCAAGCCATGATTCCCTTTTGTTCTTCAAAA	A	G	0.74	0.36	0.42	0.40	0.52	0.80	0.77	0.57	0.39	0.44	0.49	0.053	0.056	0.013	0.077	0.0022	1		
rs9901	13	25.44	CTACTCAACTGAGTGTGTTTAATGTCCAGAAATCAGATGCCTAAGAAACA[A/T]CTTCCTTAGGTGGAAGTTCAGCCCTTCATCTCTCATTTCATTTTTAGTTT	A	T	0.64	0.55	0.63	0.63	0.71	0.27	0.75	0.41	0.68	0.60	0.52	0.000	0.015	0.019	0.048	0.0072	1	1	1
rs1886204	13	25.97	TCACAGAGCTCAGTCTAGCACAAAGCATTTACTAGCCAATGACACAAAATGATC[A/G]AGAAATTTCACATTCTTTTGTCATATTATGTTTTTGAAATTTTACATGTCAAAAACAG	A	G	0.45	0.29	0.27	0.26	0.29	0.32	0.21	0.33	0.32	0.36	0.31	0.014	0.006	0.000	0.000	0.0008	1		
rs535534	13	26.01	AAATCTTAAACATGAAAAGAATCAAATGAATGAGAGAGTAACTTGTTAAGAACCCA[A/G]ATAAAGGTTTACTGACAATACTTGGTTTCTGAAAACACCATTTTTCATATTATTAAAC	A	G	0.88	0.39	0.42	0.49	0.39	0.32	0.02	0.47	0.50	0.44	0.43	0.120	0.140	0.003	0.008	0.0001			
rs1572881	13	27.01	GAAACTTGAAGGGTATTCCCTACACTGGAGGGGAAAACCCAGCAGTGGTCAACAC[A/G]ATTAACAAACCTTTCAAGTCTCTTTGGATTTTTGTTTGTGTTTTGAAACAGGGCCTT	A	G	0.12	0.18	0.15	0.13	0.27	0.27	0.71	0.19	0.21	0.07	0.11	0.002	0.170	0.001	0.007	0.0011	1		
rs1617234	13	27.11	ATGACACGCCCTCTACCTTTCCATATTTCTCTTCGGGGAAGAACATTTTGACAGC[A/G]TATCAAGAAGGTGCCTCTTACTATTACAGCTTCTTTGAAGTACAAAACTAAAAAGT	A	G	0.52	0.42	0.57	0.49	0.55	0.86	0.66	0.69	0.50	0.52	0.64	0.000	0.002	0.009	0.062	0.0018			
rs954108	13	27.17	ACCAGACAAGCATATTCTAAGCCATGTGATTCTAAGCATTTGCTTCCTAAATGATC[A/G]TTGATCTCTGGCCGCTGAAGAGTTCATTGTCTATCTGTGAACAGTCACTTGTTCC	A	G	0.36	0.64	0.56	0.56	0.61	0.09	0.45	0.59	0.50	0.64	0.49	0.023	0.001	0.003	0.125	0.0043			1
rs720083	13	28.15	GCCAAAGCCAGCTCACTGCTTACAGCATGAATACTGTTGAGTTTGTCATCCTG[A/G]TGTTCCTACATAGTGTTTGATGACCAGGTTGTCTGTAGAGTCACCATAAAACCTTTATT	A	G	0.88	0.61	0.58	0.65	0.54	0.82	0.66	0.48	0.57	0.58	0.51	0.074	0.005	0.001	0.046	0.0008	1		
rs604269	13	28.41	ATGTATTAGCTGGAGAATAATCTGTAAACAATAAATTACTAATACTAATAGACAATCA[A/G]TATAGCCTAAGGAGTTTGTTAACGCCAAGTTCTATGAGACACACCCCTCACTGCAT	A	G	0.64	0.44	0.53	0.55	0.56	0.82	0.77	0.55	0.58	0.63	0.52	0.004	0.028	0.000	0.046	0.0004			
rs478410	13	28.45	CTTTCCACACAATGATGGGAACTTGTGACCAAGGTTGAGCCCTCTCACTGTTG[A/G]AAACATGGCTGTCTTTTGTTCAGCACCCACCCACTCCCATTTTGTTTGTTTTATTAAG	A	G	0.43	0.71	0.77	0.80	0.73	0.27	0.19	0.68	0.64	0.74	0.77	0.041	0.125	0.003	0.094	0.0006			
rs717651	13	28.97	CTCACTAAAGGAAATGTTTTATGTAAAGTGAATAACAGTACCTGGCATAAAATA[A/G]GTGCTCAATAAATGTTAAGGCCTTCTCTCCCTCTTCAACTGGCCTCCTCATTTTTCACA	A	G	0.21	0.61	0.53	0.59	0.64	0.91	0.88	0.52	0.68	0.45	0.44	0.055	0.068	0.000	0.103	0.0049	1		1
rs213584	13	29.23	GCAGTGGACAGCAGGTTTGTCCCCAGGCCAGCATCTCAGTGGACTGATTGTTTAG[C/G]CAAACCCTCTCAGAGATGTGAGCACACAAAGAAGGGATCCTGGTGCCCCATGCAC	C	G	0.68	0.77	0.72	0.72	0.75	0.59	0.82	0.85	0.86	0.66	0.76	0.000	0.019	0.019	0.005	0.0001			
rs1854950	13	29.34	CAGGTTGGAGCATCAGACAACGAGGGGGGATGGGGCTACCCTGAAGTGGATAC[A/G]CAGGGGCACTGTCACCCCTTGGCAAGGATGGAGTCAGGAAGAGTCACACATATGAC	A	G	0.21	0.38	0.34	0.38	0.29	0.14	0.17	0.43	0.36	0.37	0.34	0.019	0.021	0.003	0.039	0.0014			
rs436227	13	30.43	TTTATAAAACCAAGATGGAGCCAATATTGGAGAATGTTAAGTGTTCTGCCTTCA[A/G]AAGAAAACAGGTTTAGGTTAATGAGGTTGAGAAGGAAAATGCAAAGTTACTTAAAAAC	A	G	0.29	0.31	0.35	0.33	0.32	0.30	0.22	0.28	0.32	0.30	0.34	0.002	0.016	0.009	0.003	0.0003	1		
rs390704	13	30.44	GGCTGTTCAACCTTTGGCTGTTCAACCTAGTGGCATACTGTGACTGAATAGATT[A/G]AACTTTAAAAATCTTCCAAGCTGTCTTTTTGAATCTATCTTCATATTCTCCACAGAACT	A	G	0.74	0.46	0.45	0.48	0.50	0.59	0.25	0.33	0.57	0.45	0.41	0.035	0.034	0.018	0.004	0.0035			
rs1329344	13	31.90	TAACAACTATATATGTTCTCATTACAAATTTTCTCATGTTGTAATGAAACACTAGTT[A/C]ATGCTAATGTTAAGTTTGAAAAAACAAACAAGAAAAATAGATAATTTGTGAACACAG	A	C	0.95	0.66	0.58	0.62	0.55	0.82	0.63	0.66	0.69	0.61	0.55	0.117	0.001	0.001	0.039	0.0004	1		
rs912675	13	32.07	TGTTCAAGTTATTTACCACATCTAAGTCTTTTAATGTAGCTTTTATATGCTGG[A/G]CCAAATGTTTATTCTCAAATGGAAAACAACTGAATTGTGTTTGCACGAATATGTTTTAACCT	A	G	0.43	0.37	0.44	0.40	0.41	0.41	0.08	0.22	0.50	0.36	0.55	0.000	0.074	0.026	0.000	0.0075		1	1
rs20411	13	34.03	ACTAATTATTTGAACTGGTTGGAAATCAAGCAACATTGAATATCTTTTCCCTCAAAT[A/C]AAGATCACATCTTGTTCAAATTTAGAACAGAGACTCGTATGTACAATAGACACTCTC	A	C	0.36	0.30	0.26	0.24	0.32	0.59	0.48	0.26	0.30	0.33	0.27	0.004	0.026	0.002	0.040	0.0002	1		
rs1517894	13	34.90	TTTTCACAATGCTAAATTTCCTTCCCAGTTTCAGACTAGATGAAATATGAAGGAA[A/G]GCAATTAAGCAAAGAATGTGTATCAAGGAGTCAAATATTTCATGAAAAAGCCAGTGGC	A	G	0.33	0.37	0.39	0.45	0.45	0.68	0.45	0.47	0.50	0.38	0.45	0.000	0.014	0.015	0.070	0.0010	1		
rs1517895	13	34.91	CTGGGTTATTAATTAACTGAAAGTCAAATATCAGAGGGAGTTGAATAAGTTTATA[A/T]TACAGTATCCACGCCCTGAATTCCTTAGCTGTCAAAAATAACCACTTTCATTTATCCA	A	T	0.48	0.43	0.43	0.46	0.38	0.00	0.14	0.27	0.36	0.47	0.41	0.001	0.068	0.012	0.209	0.0000			
rs9469	13	35.38	AAAAAAGGAACAAAAAGTAATGCAAGACTCAAAATTTTGGAGTGGTGGC[A/G]TGCCTCTCTTCATTTTACTTTTTGACTGGCTGCCTGTATGCCGATGATGA	A	G	0.10	0.37	0.40	0.40	0.39	0.77	0.49	0.55	0.29	0.33	0.35	0.066	0.010	0.020	0.099	0.0019			
rs1781670	13	35.49	TATTCAACAGAGCTATGGTAGTTTGCAGGACTGGAAGGAAATAGTAATATGATC[A/G]TATTTTGTTCTTTTAGGCCACCTACAGAAATATTTTGTGTTATAGAGGAGGATTTAACTT	A	G	0.33	0.52	0.54	0.61	0.52	0.18	0.44	0.46	0.71	0.63	0.56	0.036	0.018	0.024	0.118	0.0001			
rs668103	13	36.55	GATAGGCAATTTAAATTTCAGATACATACTTAGAAGAAGACAATAGACAATTAAT[A/C]AGCTGGAGAGTTCATCACAGCCCTGCTCACATTGTCCTCTAGAAATCCGCACATCTA	A	C	0.79	0.55	0.72	0.68	0.61	0.55	0.33	0.58	0.62	0.69	0.61	0.007	0.093	0.013	0.017	0.0070	1	1	1
rs980285	13	36.84	ACAAGTAAAGAAAAAAACAAATACAAAACAACAACAAAAAAATAAAATGGCAGGCTT[A/G]TGCCCTAAAGTATGAATAATTATGTTATATTAAATGGTGTAAAGATACCAATAAAAC	A	G	0.45	0.30	0.35	0.38	0.18	0.68	0.51	0.70	0.29	0.40	0.32	0.014	0.036	0.066	0.085	0.0000			
rs737645	13	37.83	CCATGGGAAAGAAAGTCTTGCTTTCAAGGACTAATATTGGAGGATAGAATGGTCA[A/G]TAGTTGTTTCTCCTTAATGTTAAAGTATTGAAAATTACTGACGAGTCAGCTACCTTC	A	G	0.75	0.47	0.46	0.51	0.48	0.86	0.46	0.71	0.46	0.56	0.55	0.064	0.004	0.058	0.122	0.0070	1	1	1
rs732390	13	38.33	TATCTAGCCTCACTGAGCTTTCAGTCTAGGTTATAAAATCGACCTGATTCACAAC[A/C]CAACAAGGGAGAAATCTTACTCTACCAGGAGGCTTCAGTAATGAGATTGGTCTTGC	A	C	0.71	0.65	0.65	0.61	0.55	0.82	0.38	0.88	0.46	0.70	0.68	0.009	0.023	0.078	0.032	0.0019	1		
rs2004000	13	39.18	GGGGTATGTTACCTGAGCATCAGTCAGTTTTGCCAGGAGACCTGATACTTGTCCTTGTTTCC[A/G]TTAGTCTATTTAGGAAAAGCTGGGAATGCATCCTTCTATGACTTGTTGGT	A	G	0.48	0.57	0.60	0.57	0.68	0.36	0.40	0.55	0.61	0.50	0.55	0.009	0.035	0.006	0.035	0.0037	1		
rs7799	13	39.33	GGTGCTGATATTCTCAATAGTTATGCCGGTCTAGCCTGTGTGGAAGAGCCCA[A/G]TGACATGATTACTGAGAGTTCACTGGATGTTGCTGAAGAAATCATAGACGATGATG	A	G	0.67	0.30	0.32	0.30	0.39	0.86	0.31	0.45	0.42	0.23	0.32	0.058	0.003	0.004	0.141	0.0002			
rs912875	13	40.48	ATTAAGTGGACTGCTCAAATGAGATTTTTTGCCTCCCTTTCCAGAAGAAGTCTGC[A/C]GTCTGGGATCAGGAGATTGTTGTATATTTATTTGGGGTTATAGTGTGGGTAATTGG	A	C	0.57	0.52	0.51	0.48	0.45	0.55	0.93	0.70	0.54	0.60	0.58	0.005	0.149	0.021	0.003	0.0023	1		
rs994856	13	40.51	CTAAGGCCAGCCAGGCTTCAAGGGGGGCCACAGACATTAAGACAGTCGTGTCA[A/G]AGGATTTGGGACATGATTTAAAACCTACACACAGTAAGATTACTCTTCCTTTCTTGTG	A	G	0.76	0.56	0.59	0.59	0.63	0.91	0.48	0.52	0.65	0.55	0.56	0.006	0.016	0.002	0.051	0.0004			
rs238349	13	40.60	TTGTATCTGCACTTACGAAGTCAGAGGTCTCCTTTTTCAGTCCCTCTGATAGT[A/C]CCACCTAAGTGCTGTCCTGCTCCTGTGAACACTTTCCTGTGCAACCAAACAGGAGGTG	A	C	0.55	0.49	0.47	0.55	0.50	0.59	0.43	0.48	0.54	0.48	0.52	0.001	0.001	0.001	0.008	0.0027			
rs1449505	13	40.61	CTGTTTGTGTAATTATTTCTGATATTTTTAGGAAAAGAAGTTTAACAGAGTAACTCA[C/G]ACTGAGACTGTTAAACTCTGTTCATGATAAACTCCATATTACATAATATAGGTGAAC	C	G	0.21	0.31	0.33	0.27	0.25	0.27	0.07	0.19	0.25	0.32	0.32	0.006	0.055	0.015	0.001	0.0003			
rs1169791	13	41.07	TAGCAAATAACTGAGATATCCCAATGGCATTCAACGTTAGTGTTTATTGCTC[A/G]TGCTCCTGGGGCAGTTGGATAGGTGGCTTTGCTGCTTTTGATTGTGCTCATTCACGTGTA	A	G	0.31	0.64	0.56	0.65	0.52	0.68	0.24	0.54	0.61	0.59	0.62	0.027	0.050	0.001	0.009	0.0020	1		
rs1544295	13	41.30	CTTGTGGCTGTCAGGGAGGGAGAGGGAGAACAGGAGTTGGATAGCTTTACCA[A/G]CTGGAGTTTCGCCTACCAGGCTGGTCTTCCTTTCCGTTCGAGGGCTCCCTGTATCCTC	A	G	0.12	0.20	0.20	0.21	0.23	0.09	0.22	0.14	0.27	0.08	0.13	0.001	0.007	0.000	0.003	0.0036			
rs1408875	13	42.81	CACCTGCATGTGAGGAAACTACCCGAGGCTGGGGAAAGAACCACCCAAAAGAA[A/G]CAAGCAGAACAATTTTCAGAATCACATAGGGTAGGCACCAGTTGGTATTACCACC	A	G	0.24	0.39	0.42	0.28	0.54	0.68	0.61	0.46	0.39	0.52	0.51	0.030	0.008	0.001	0.025	0.0007	1		
rs2050638	13	43.14	TGTATTTTGAAGGGGACATTTAAGCCATGGCAGC[A/G]TATACTCACAGAGAAGGTAGAAGTTCCATACTGAAGAATTAACTCGATACCAAAATTAATGATGGTCCTTAATACATAC	A	G	0.52	0.74	0.70	0.68	0.79	0.55	0.54	0.55	0.57	0.71	0.73	0.015	0.014	0.018	0.013	0.0001			
rs4941527	13	43.78	ACTTCTCACACAGGCAGTTTCTTCTCCAAACTCTTCCCCTAGGTTTTATTTGGAGA[A/G]TGCTATCACCAAAACCCCTGCAGCAATTCCCAAGGGGAGCCTCCCCTTAATAAAG	A	G	0.19	0.40	0.46	0.44	0.38	0.18	0.30	0.25	0.50	0.42	0.39	0.048	0.016	0.043	0.050	0.0029	1		
rs2985981	13	43.89	ATGTATGAAATAACAAGGAGACAGCTATGCAATTGTTCATTAGAAACTTCTTGTTGG[A/G]TCCATCAACGCTAATATGCCTGAAGCCTGTGGTAAAGACAGAAAATACGAGTTTT	A	G	0.21	0.16	0.15	0.20	0.25	0.23	0.01	0.15	0.14	0.23	0.15	0.002	0.065	0.002	0.002	0.0004			
rs1886040	13	44.52	ATGAGAGTCAGGTGAAATGAGGTTACTCTTAGCCAAACTAAAAAGATGTAACCC[A/G]GGGCAGGCAGATGGTATTGGTGCCTGACACCTGCATGTGACTTTCACTGACTTCAA	A	G	0.21	0.43	0.50	0.43	0.36	0.64	0.20	0.47	0.21	0.60	0.59	0.051	0.048	0.000	0.008	0.0055	1		1
rs625052	13	44.60	TAATTGTTCATCATTTAATGGATTCCAATTATCCATGAAAATATATTCATGAAGGC[A/G]TCTATCAGCACTGGCATTCTACTTCTGGAGTTCTTCTAGATCACCTTTGAATGACTG	A	G	0.43	0.27	0.26	0.32	0.25	0.50	0.46	0.34	0.36	0.31	0.37	0.023	0.032	0.002	0.046	0.0051			
rs644061	13	44.86	TTACTACCCCAACAGAATTGCAAGACAAAAGATTCTAAGAACCAACTGTGGGCCG[A/G]TGAGGTGGCTTATGCCTGTAATCTCAGCACTTAGGGAGACTGAGGTGGGCGTAT	A	G	0.12	0.72	0.62	0.70	0.55	0.41	0.26	0.58	0.68	0.56	0.56	0.155	0.059	0.000	0.021	0.0011			
rs720277	13	45.33	GAATACTTAGAAATTGGCTTTAAGTAGGACTGATTTACCTAGCACAGTTATCT[A/G]TGGTCTCATAAAACTGGCTTAGTTTCATGAAATACCTTGTTTTCTCTCTAGTCAGTCCTT	A	G	0.45	0.63	0.62	0.59	0.64	0.45	0.61	0.57	0.71	0.47	0.59	0.007	0.003	0.001	0.001	0.0003	1		
rs973908	13	45.51	AACTCACTGCTACCTCTGTGATTTTAAAAAAGAACATCTTAAAATTGTCTTCG[A/G]TACTTATCCAGTTAGGTTTCATGAATCATCAAGGTGGGGGATGTCTGGATAGCCTCAAGT	A	G	0.81	0.54	0.45	0.51	0.65	0.59	0.72	0.58	0.50	0.48	0.48	0.075	0.039	0.002	0.018	0.0009			
rs1853987	13	46.00	TTGGGTTTAATCTGGTATTTTAATAAAATACTTTTGAAGCAGCAGCTATCAACT[A/C]TGAAAAGCAATTGATGTTTCCATTAATCTTTTTCTGAGGAAAACCTTGGTTCCAAGGT	A	C	0.36	0.24	0.27	0.26	0.27	0.45	0.46	0.35	0.27	0.27	0.30	0.002	0.016	0.004	0.011	0.0000	1		
rs912278	13	47.08	TAAAGGGTGGAGGTGATATGGCATTCTGAAAGTAGGGAGGGACTAAGTCAGTC[A/G]TCATACTAAACAAAAATCCAGTACCTTTCCTTTATTTAGCTACCAGAATGATGACAG	A	G	0.86	0.51	0.56	0.48	0.59	0.50	0.56	0.51	0.46	0.57	0.51	0.046	0.001	0.006	0.002	0.0028	1		
rs1427167	13	47.26	TTCAAGAAAGGATTATGTTCTCTGCATCATTCAGGAGCAGTACTCATGCATTTC[A/G]GGAAGGTTTCCATCCCTATCAGACATACCTGGCTGTATATCAAAGTCCATGCTCCTC	A	G	0.43	0.61	0.63	0.71	0.55	0.18	0.45	0.36	0.64	0.57	0.54	0.013	0.013	0.031	0.080	0.0016			
rs7446	13	48.07	TGGATCAAGACACTTTGGTATGATGCATATACAAGTTGGAAGACTAAAGAGGTGCA[A/G]TGTGATCTGAGCCTCCATCATTGTCCTCCACAAACATATTTCATATTCTTATGTC	A	G	0.12	0.24	0.35	0.29	0.34	0.18	0.54	0.19	0.36	0.32	0.34	0.044	0.023	0.013	0.014	0.0000	1		
rs1407441	13	48.09	ATTTTCCAAAAGCAAAAGACAATACAGATTTTGGATTCGTCAGTATTTAAGGGATTGAAAACCTGGGCATTGAATGAGATCACCTGG[A/G]GAGGCTGTATTTCGGAAGGA	A	G	0.18	0.11	0.13	0.17	0.13	0.00	0.00	0.07	0.08	0.17	0.12	0.000	0.075	0.020	0.075	0.0010			
rs1239684	13	48.96	GGGTTCAGACCTCTACTAGGTACTCAATCCTGAAGAGTGGCTGAACTGTCCC[A/C]CTTTGTTTCTCCCCATCATGGAACAAAGTGAATAAAGACTGAAAAGCTCAAATGGAAT	A	C	0.64	0.55	0.52	0.55	0.52	0.64	0.08	0.75	0.35	0.57	0.61	0.004	0.152	0.023	0.001	0.0062	1		1
rs675305	13	49.12	CCATCCTGGCTAGTGCTTAACAAACACCCAACTAGACCATGCTCCTCAAAATCAAGC[A/G]CACAAACATTTGTGTTTCATTAAACAGAAATTAATAGACTCCAAGAGAAAGAAG	A	G	0.83	0.40	0.45	0.43	0.54	0.50	0.52	0.28	0.46	0.47	0.51	0.061	0.000	0.026	0.000	0.0008			
rs1014934	13	49.87	GAAATCTCGTCACTGGCCTGTGTTAATGTTCTCATTTTCCTTGTGGTGAATTT[A/G]TAATCTTTTCTAAACTCTCCATAAAAAGAAAACTCATAGTCTTTCCTTAATGTTTCGATC	A	G	0.43	0.67	0.65	0.71	0.64	0.50	0.47	0.70	0.64	0.60	0.55	0.027	0.024	0.000	0.018	0.0063	1	1	1
rs1058142	13	50.14	GTAGTATTTATTTTGAAATAAAAGTTCCCATCCCTTGTAGGCCTCCTGT[A/G]AGGCACAACGTCTTCGAGGGGAAGTTGAAGTGGGGTCTTATTCACTG	A	G	0.24	0.32	0.23	0.21	0.29	0.14	0.59	0.38	0.25	0.14	0.25	0.001	0.059	0.009	0.017	0.0007			
rs747781	13	50.35	CGGGTCCCTGCCAACAGCCTCATCTGAGCCCCTAGCC[A/G]GCAGCAAGCATCCCATTGGCAGCCATGTGATGAACTATCTTCTTGGAAGTGAGCCAGCCCCATT	A	G	0.50	0.45	0.53	0.48	0.68	0.73	0.62	0.31	0.71	0.52	0.57	0.001	0.002	0.050	0.013	0.0001			
rs3368	13	50.51	GATTTTAAGTATTAAGAGTTTCCTCTGCTTCATTCTGTTTCCAAAGGAAATATACGTCGCATGAACTCATGATCATCTCAGCATGAACTCCTGAGTGAAG[C/G]CTCTCTCAGC	C	G	0.81	0.46	0.36	0.40	0.30	0.41	0.81	0.66	0.36	0.42	0.33	0.113	0.108	0.063	0.002	0.0001			
rs1322938	13	51.53	ACAAAGGCTTCTTTTTATTTTGAAATGCTTCTGTCTCTTTTAGTTCAATCTGGC[A/G]ATGTTTGTGCTAGTACAATTCTCTTTAAAACTTTGTGGGTTTCTTAGGTATCTTATTAGG	A	G	0.67	0.45	0.50	0.44	0.34	0.59	0.20	0.38	0.39	0.41	0.48	0.016	0.048	0.004	0.007	0.0002	1		
rs1573217	13	52.28	AAAAATAAAATGAAAGGACTATTCAAGTGTGAGAAATAAAATAAAGGTATACCT[A/G]TATAAACATAGGCTTTCACCTCGTCATGTATTTCATTCTTATGGAGCAAAAATGG	A	G	0.64	0.53	0.53	0.57	0.64	0.41	0.52	0.49	0.57	0.57	0.60	0.015	0.001	0.000	0.004	0.0050	1		1
rs1361026	13	52.82	GGAAAGGGGCAAAAAATGCTACTGTCATGAAAATC[A/C]AAAGAGAATAAATTTTCAACTAGAAAAATTAATAAGAGCACATTCTGTGGAATACTCCGAATGAAAGAAAATAGTTA	A	C	0.40	0.32	0.34	0.33	0.25	0.27	0.13	0.33	0.32	0.46	0.38	0.002	0.029	0.000	0.002	0.0001	1		
rs1340580	13	52.92	GGTAAAAATTTCTAACACCATGTGAATGCACATGAAAATTTCTGAGATCCTC[A/C]ACCCTTTTTTCTGTCTTTTCCAGAGCCATCCTTAAAAACCTCAAGTACAATCCCATCAT	A	C	0.21	0.44	0.49	0.44	0.52	0.23	0.30	0.32	0.36	0.55	0.51	0.051	0.019	0.015	0.034	0.0000			
rs1382461	13	52.96	CTAAAATACAAATTTATCTTGGAATTCTGTGTTTTATCGAGCAACTTTATTCAA[A/G]AACAGTTTCCTACTCTAATATCCTCCTAATTCTTTAGGTTTAGCCTTAATACATACATTT	A	G	0.31	0.51	0.55	0.57	0.54	1.00	0.96	0.83	0.54	0.57	0.55	0.035	0.109	0.040	0.164	0.0001			
rs1336229	13	52.96	AGATGACAGTCAGCTTTACCTCCAGGTATATAAAGCAAACCTTCAGATG[A/G]TTCTGGCCTCCAGACTTTGAGCTACACCAGATGACACTGCATAGAGCACAGACAAACT	A	G	0.98	0.81	0.72	0.70	0.79	0.86	0.99	0.70	0.85	0.79	0.83	0.073	0.089	0.001	0.017	0.0084		1	1
rs641385	13	53.67	CTTAGGATTATGTACAAAGATCTAATTTGTCTACTTCCTTCCCCAACTCTGATA[A/C]CTCCGTAGAATTTAGTTGCATATTTCAGAACTCAAAGCTACTAACTACTTTAAATTAATA	A	C	0.83	0.50	0.55	0.48	0.46	0.77	0.56	0.74	0.54	0.58	0.61	0.048	0.000	0.020	0.025	0.0021	1		
rs1936318	13	54.93	ATGCTCCTGCCTCTACCTCTAAGTTCTGGGATTTCAGGTGTAAGCCACCATGCCTG[A/G]TCTTAACTAAAATTGGTATATCAACTGATAATATTGAATTCATTGAAGATGTCTTTT	A	G	0.21	0.44	0.30	0.31	0.34	0.36	0.66	0.23	0.35	0.39	0.41	0.008	0.058	0.011	0.001	0.0047	1		1
rs1409239	13	55.78	GCTTCCAAAGAAAGGGGAAATGCACAACGGTTTTTAGACAATGATGCTGTTGCC[A/G]CATATGAATTATGTTTTCATTTTCTATTGTCTTGAATATGGCAAAGAGGAAAATCAGG	A	G	0.74	0.53	0.58	0.46	0.57	0.05	0.01	0.53	0.46	0.60	0.64	0.012	0.274	0.000	0.200	0.0010	1		
rs986642	13	55.84	GACAACATTAGGACTGGAAGCCCTGCCACTCAGCTTTCTATCTCCACATGCT[C/G]TCCATCTGGCTGACTCTAGATAAAGGCTCCATGCACTCGTTGCTTTTGAGGCATTCA	C	G	0.83	0.60	0.61	0.63	0.73	0.41	0.31	0.73	0.46	0.80	0.77	0.029	0.054	0.021	0.026	0.0096		1	1
rs717256	13	56.22	ATTAAATCACAATAAGTGATTGAGTCATTATAAAGCAATTTATGCTGTTGAGGA[A/G]GGTGTGGGTCAAATTAGAAAAAGATTAAAGGCAGGAGTAGTAGTTGAAGAGATTTTAA	A	G	0.74	0.41	0.41	0.46	0.43	0.27	0.20	0.38	0.50	0.47	0.49	0.041	0.048	0.003	0.022	0.0025			
rs1413119	13	57.14	GTACTCAAATATAAAAGCCTTGGCTAAAAAGAACAGCATAAAACTTAGAGACA[A/G]AGGTTAGCCCTGGGCAAGAAGAGCCTCTCAGAAGAAAGTTAAAAGACTCATTGAT	A	G	0.26	0.40	0.34	0.45	0.25	0.68	0.40	0.56	0.43	0.33	0.38	0.002	0.002	0.018	0.057	0.0002	1		
rs901510	13	57.67	TGCATCAAGATTTACCTCGTGGAAATCAGAAAATCTGGTATAGTTGTTAAAAA[A/G]TTTTACATTCTGCTTTCATTTCTTGATGAAACCTCAGGAAAAACTGGTTTCAATCATGTT	A	G	0.57	0.46	0.48	0.44	0.57	0.86	0.76	0.57	0.32	0.41	0.44	0.013	0.100	0.030	0.161	0.0002	1		
rs1536289	13	57.68	TGGGGACCATTAGTAGTGTTCCTGTCTTCGCAAGAAGACCATGCCTAGAACA[C/G]AGTCTCCCTTCACTACTTCCAGGAAGAATTTTTTAAATACTCATATGCATCCATAGGT	C	G	0.25	0.50	0.59	0.50	0.46	0.14	0.14	0.28	0.57	0.46	0.49	0.079	0.153	0.081	0.154	0.0047			1
rs340210	13	58.23	AATAATTGAAATTATTTTCTTGTGTTAAATCACCTGAGATAGGGCAAAAGGGCATTGAACTGAATGGGTTGTAAACATTCA[A/G]AATCACTCAGTGATATTCAAAATAACTTTTAC	A	G	0.45	0.76	0.77	0.72	0.86	0.82	0.99	0.70	0.82	0.71	0.68	0.086	0.046	0.014	0.000	0.0072	1	1	1
rs2077961	13	58.93	TATGTTCCCTACTCTGATTTCTGGGCAAGCTTTTGGAATCTATTAATCTTTAG[A/C]AATGGGTTTCAGAGAGGTACACATTTTTAAAAATTCTGAAATCGTTCCTGTACTTAACTA	A	C	0.62	0.49	0.49	0.49	0.54	0.86	0.53	0.39	0.50	0.55	0.57	0.014	0.003	0.004	0.080	0.0032	1		
rs301653	13	59.25	GTTTCTGAAGAAATCATGTTGACCTTGCCTCCTAAGTTAGTGTTTCTCAAAAT[C/G]GACTTCACATTCCTTAGAGGGCAGGGATGGTGTCTCTTTTCTGCTTTATCATTATGACT	C	G	0.86	0.88	0.87	0.87	0.88	1.00	0.98	0.88	0.89	0.84	0.88	0.000	0.031	0.012	0.060	0.0000			
rs310935	13	60.83	CATGTTTAGGGAGGGAAAGATCAGTAGTTCACATTGTACAATTCAAATTCATCAA[A/C]TCAAGTTTTCCACTCCAAGTGGGGATATCAAGAACGAAATTTGGATATAACTTAAAGACA	A	C	0.14	0.42	0.41	0.44	0.36	0.95	0.71	0.46	0.54	0.49	0.48	0.040	0.063	0.003	0.215	0.0031	1		
rs1553161	13	60.95	AATGGTTCATTTGATGGGTTACACATTTTTCTCCTGGGCAACCATTGTATAAT[A/G]CGATTCTCAATTGTTCTATTGCCAACAATATTTTATTGAGTTGTTGCTTCTGGTTTTCTG	A	G	0.17	0.42	0.41	0.45	0.37	0.91	0.71	0.47	0.54	0.51	0.49	0.034	0.060	0.002	0.164	0.0037			
rs1335686	13	61.29	TTACTTTTCTAAGAGGTGAAACTGCCTCCTTCCTTTTTAATAAACTCCACTAT[A/G]GATTTTTCAGACGTTAACTGCACATCCAGGGACATTTCTATAATCACATTTTGTATGCACA	A	G	0.67	0.44	0.47	0.44	0.50	0.91	0.97	0.45	0.54	0.45	0.45	0.022	0.190	0.000	0.125	0.0001			
rs188481	13	61.61	TCTTGAGAAAGATACTATCTTTATATCTGTCAGAATGGAGAATACTGGAGA[A/G]GCAAGTGTCTACATTCTGTATGTCTGCGAAGCATGATGAGACTCTGCTGTGACAGCTCCC	A	G	0.17	0.51	0.47	0.50	0.57	0.05	0.10	0.35	0.54	0.47	0.49	0.061	0.097	0.004	0.141	0.0010	1		
rs1545963	13	62.65	TTTTGAACATTTTTCATTGTGTTTCCAGACTGCTTATGTTTTGTTTTAATTTCC[A/G]AATTTTCTATACATTTTCAGAACCCATTTGCTTAGAGTATAACCAAAAAACATTCATCACAT	A	G	0.67	0.44	0.39	0.48	0.34	0.64	0.19	0.52	0.46	0.42	0.40	0.036	0.023	0.007	0.038	0.0000	1		
rs1022239	13	63.22	TTATGAGATTTTAATCGTCTAATTTGACATGCAGTTCATTTTTTAAATAATTGT[A/T]CCAAATCTCATCAGCAACATCATATCATTGGTGACATAATGTGAATTAATCCTTTTAAAC	A	T	0.69	0.37	0.39	0.38	0.38	0.18	0.59	0.36	0.32	0.34	0.44	0.043	0.018	0.000	0.039	0.0027	1		
rs1572875	13	63.36	ATCTCCTGGTCTCAAGTGATCCTGAGTGAGTGGTACTACAGGCATGTACCACTATA[A/T]CCAGCTATATTTGCTCATGTCTATTCATTGGCCTAGATACATTTAATTATTTTGCATG	A	T	0.74	0.80	0.85	0.84	0.77	0.95	0.91	0.80	0.92	0.82	0.84	0.009	0.002	0.003	0.014	0.0009			
rs1831972	13	64.74	AATGATAATCAGGTATATTTATTTTGTCTTT[A/C]TTGCACTCTGGTAGTGATGGTTGAGAAGAAATAGACTTATTAAGTTAAAAAGTAGAGAAAAGTTATCAAGAGGTTTTTAGG	A	C	0.33	0.46	0.48	0.49	0.48	0.64	0.94	0.56	0.43	0.52	0.40	0.013	0.143	0.003	0.013	0.0061	1		1
rs1547143	13	65.43	CAAAAGTATTCCTATAAATGAGAGCGAATAGACTTTCATGCTTTGAAGATGGTGGGT[A/G]ATCTTAAATAGCAATAACAGCATGCCCATGATCAATGTCAAAGAAACACTGTG	A	G	0.93	0.77	0.74	0.77	0.63	0.91	0.78	0.82	0.64	0.73	0.72	0.026	0.000	0.001	0.026	0.0009	1		
rs735600	13	66.80	GTCCTCTCCTAGGTAAAACAAAATAAATCTGTTGGCCTCTCAATGCTCTATTT[A/G]TTCATCTATATCATCTCCCCCTTCTTCTTCCTCATTAATAAACATCCACAGAGGCACTGC	A	G	0.40	0.67	0.61	0.50	0.66	0.68	0.76	0.71	0.71	0.55	0.60	0.033	0.023	0.001	0.001	0.0000	1		
rs1340745	13	67.31	GAGGTACATAATATTAGAATTGACTAAGAGGTAGTGTTGGGCCTAAAAGAAGGAA[A/G]AATATATGGCATCAATAGTGGAGCCAAAGATGATATCAATTCTAATGATAAGAAAAT	A	G	0.62	0.40	0.43	0.41	0.34	0.50	0.26	0.51	0.38	0.43	0.41	0.018	0.019	0.006	0.003	0.0001	1		
rs1341518	13	68.77	ATCAAAGTTACAGACAGTATTTTCCTAGGAAGGAAATATTGAACAACATTTTT[A/G]TTGAAATTTAACTACCATTTTTCTTACTTGCTTTTTCTTCTCCTTTTTTGAACATAGGAAA	A	G	0.24	0.28	0.23	0.22	0.27	0.14	0.01	0.23	0.42	0.20	0.25	0.001	0.050	0.004	0.002	0.0001	1		
rs768826	13	69.57	AAGCAATCATACTAGGAAAAAAAATTTAATCTGTTTGTGATGATTAAGTGAAATCTAG[A/G]AGCCAGATATGGTAATGAATCTTGAACAGAACTTGTTAAACATATTTTTAAAAAGTG	A	G	0.40	0.57	0.57	0.54	0.46	0.59	0.89	0.80	0.69	0.59	0.55	0.007	0.065	0.052	0.001	0.0000	1		
rs1023102	13	71.62	CTCACTGATCTCATAATTCTATAGAGCTATACTTCTAGCAAGAAGGTTACTATCATTATTACTATTTGGATGGTGTTTGAGAGTC[A/G]GGAGATGAATGTGGAGCTGCCTCATTT	A	G	0.43	0.58	0.60	0.52	0.50	0.50	0.26	0.38	0.61	0.53	0.51	0.007	0.030	0.017	0.000	0.0061	1		1
rs1331769	13	72.64	GCTTTTGTGCCGGCTGATGGCAAAGGGGACATGAAATGAAGGAGTGGGGAAGGCATATTC[A/G]TCTTCATGATTTTCCATAAACAAGAAGTGTAGTCACTTCTGTATTATTT	A	G	0.83	0.45	0.50	0.49	0.41	0.23	0.11	0.47	0.46	0.51	0.55	0.065	0.089	0.000	0.038	0.0001	1		
rs1318725	13	72.66	GCAGGAAACAGTCTGATTTCAAAGGAGGGCTAGGATTAGGCAGAAAACCGTG[A/C]AGAGTCAAGAATGGAAGCCTCCGCAGCCTAGACTGGTGGGTTTCAGCGGCATACA	A	C	0.21	0.22	0.24	0.29	0.30	0.18	0.00	0.15	0.29	0.22	0.23	0.002	0.101	0.003	0.005	0.0004			
rs2038946	13	72.92	AATAATAAATTAAATTCCAGTAGCATGAAAGAACTGCAGACAGAATTCTGAAAACT[A/G]TAATTCAGGAGTACTAGAACTCACCCAGATACACAAAATAAGGAATAATGGGTAA	A	G	0.83	0.36	0.50	0.49	0.48	0.23	0.53	0.55	0.43	0.57	0.55	0.058	0.000	0.000	0.048	0.0009			
rs1011026	13	73.53	CTGTAAGCCAAAATTTCTGCATCTTTAATATGAAGATCATCAAACTGACCTCAC[A/C]GAAATAATGTCTGTGAAACACATACAGCAGTGAAGACTCCACAACAGTTAATAGTTAT	A	C	0.83	0.34	0.44	0.41	0.36	0.50	0.54	0.41	0.39	0.54	0.42	0.122	0.013	0.000	0.006	0.0000	1		
rs713381	13	73.63	CTCCTGAGAAAATGATGTTTAGCCTGAGGTTGAAGATGTGTAGAAACTACACAAGA[C/G]GAAAGAGAATAGGGCAGAGAGATTATGAGCATCCCAGGTAGAGAGAATAATAAG	C	G	0.36	0.44	0.46	0.46	0.59	0.68	0.71	0.66	0.43	0.43	0.51	0.015	0.019	0.001	0.009	0.0005			
rs1560540	13	73.68	CCATTTATGTAAAACATTCCATTTCCTTATATTAACATACACAGCTGAGAAAT[A/G]CTGTGGGGTGCTGGGGAAAGCCCGAGAACCACGATGAATAAACCTGAGTTTTGGTACTT	A	G	0.71	0.52	0.54	0.59	0.46	0.50	0.91	0.26	0.43	0.56	0.55	0.020	0.091	0.019	0.000	0.0005			
rs953109	13	73.86	GCATGGGAGAAACACTGAGGTCTTGCAGGTTTCCTGGGCTACCCTTGGGAAA[C/G]AGCAGCACAGCTTCTGCTCTGGAGACCTATGCCCCCCTCCACTGACTTAACCAGAAGA	C	G	0.36	0.54	0.57	0.55	0.61	0.55	0.45	0.63	0.68	0.53	0.61	0.025	0.010	0.001	0.002	0.0016			
rs1394695	13	74.73	CCCTCTCCTTAAATATAAGAATACAACCTGTTCTCAAGTAGCTCAAAATCCAGT[A/C]GCTCAAGATCTCTGTTAAAATCATTGTTTATTGTACTGTTAGAGATTGTGATTTCTCAT	A	C	0.57	0.49	0.56	0.52	0.52	0.36	0.48	0.67	0.71	0.51	0.53	0.000	0.002	0.003	0.020	0.0011	1		
rs1886037	13	75.96	ACACAAGTTTCAAGAACATCGTTCATCAGAATCTGTATCTCTTAGTGGGAAACTT[A/G]ACTTGGAACAAACATGAACAAAAACATTTTTAATATGGTTTATATGTATATTATA	A	G	0.36	0.24	0.25	0.29	0.39	0.50	0.18	0.25	0.29	0.34	0.33	0.020	0.000	0.003	0.061	0.0082	1	1	1
rs5351	13	76.27	ATCGTAAAAATTCTCTCATCCCTATAGTTTTACAAGACAGCAAAAGATTGGTGGCT[A/G]TTCAGTTTCTATTTCTGCTTGCCATTGGCCATCACTGCATTTTTTATACACTAATG	A	G	0.62	0.36	0.33	0.33	0.39	0.73	0.45	0.45	0.43	0.42	0.41	0.029	0.001	0.002	0.056	0.0030			
rs1372177	13	77.37	CAGCATAGTTTCTTTCAATGTTGGGGGAAAGAAGATGAACCTATCAACATGACT[C/G]CAATAAAGGCTTAGTAATACAACCATATTTGGGAAATATTCATATAGCAAAACAGAGA	C	G	0.95	0.52	0.51	0.59	0.54	0.86	0.86	0.46	0.57	0.42	0.41	0.145	0.087	0.000	0.090	0.0038	1		
rs767778	13	78.00	ACACTTTAAAATAGCAGAAGAGTCAAAGACAGAATCCTAAAACCAAGGTTATTCAA[A/T]CCAAGGTTCTTCAAATCAAGGGCACAGTATGTGTTTGACATTCATAGCTTCTTCAT	A	T	0.86	0.36	0.33	0.41	0.48	0.68	0.94	0.44	0.39	0.35	0.38	0.182	0.268	0.016	0.091	0.0020	1		
rs1892096	13	78.94	CCGAGAAAGCTTTTTACAGTGGCCCACTTTGGAGCCATCGATAATTAACTAAACAAATATTTCAGAGTGTATCCAACT[A/G]GTTTCAGCCAATTCCTTAGGGAGTTGTGATACT	A	G	0.36	0.57	0.46	0.50	0.63	0.45	0.71	0.24	0.54	0.40	0.40	0.007	0.028	0.038	0.000	0.0028	1		
rs1176270	13	79.12	ATTTCAGAATTTGATTTATCAGATATTGAAGAAAATGAAATTGCTCTACACACA[A/G]TTTTGTTTTCTTTTTGCTCCTTCTAGCACACAGTAGTGAAAGGGGAGAGTAGATTTACAGT	A	G	0.29	0.38	0.39	0.27	0.46	0.45	0.31	0.48	0.46	0.34	0.48	0.009	0.007	0.000	0.001	0.0085		1	1
rs768218	13	80.06	GGAGAAAGAGGGAGGTGGACAAGAGCAGAGCCC[A/G]CAGAGCAGTTCAGCATGTACTGCAGTGACTGGTGTCCTGAACCATAAATACCTGCAACTTTTATGCAGTGATCAGCAC	A	G	0.52	0.53	0.57	0.50	0.58	0.40	0.46	0.70	0.54	0.63	0.55	0.005	0.013	0.000	0.008	0.0002	1		
rs984518	13	80.48	CGTGGTTAAATACTATAGTTGAACTTAAAGGCAAGTCCTGTATAGAATATTGAAC[A/G]TTTGCTCTCTCATGATTCCCAAGTCTGGAGCCCTTGTTGTGCATCATAGATAAGTAG	A	G	0.12	0.38	0.35	0.30	0.36	0.50	0.30	0.52	0.29	0.37	0.37	0.028	0.001	0.022	0.018	0.0031			
rs1572096	13	81.06	ACTAAAAGTAACATTGGTCATTTTTCACTGAAACTCTCCTTTCAAGCACACAAACAC[A/G]CCACAGTATAACAAAATATGTTTCACAGCTCTATTAAAGCATTCTGTAAAATAAGT	A	G	0.33	0.38	0.31	0.24	0.34	0.68	0.61	0.48	0.43	0.23	0.30	0.001	0.018	0.009	0.041	0.0012	1		
rs1334857	13	82.32	CATAATACTGTTAGGTAGGACAAATCCCCAACAGCTATAAAACCTGTGTCTTATC[A/G]ATAAATGCATGGGATGAAACAGAAGGAAGGCATCAGTGCTTCTTTACAGCCCTGAG	A	G	0.62	0.84	0.85	0.79	0.80	0.41	0.61	0.67	0.85	0.83	0.77	0.023	0.023	0.017	0.095	0.0046	1		1
rs1348840	13	83.00	TGAAGCAGAAAAAAAGCTTAAAAATACTTAATATCCTCAAAAGGGAGGGATATTAAA[C/G]GCTCTGTAGAGTAAAACATTCAGCAAACACTTAAAGATGCCTTTGAAAGTGAAAC	C	G	0.21	0.48	0.50	0.49	0.63	0.64	0.65	0.63	0.75	0.60	0.56	0.051	0.008	0.007	0.005	0.0007	1		
rs996297	13	83.81	TACTTTGCTTCAGGTTTCTGTCTATTGCTAAATTACATCTACTGTGTATCTTGT[A/G]TGTGTTTCTTTGTTCATTTAGAATATATTTAGCGAATAAGGGAAACTTGAGACATATGAA	A	G	0.21	0.24	0.33	0.30	0.41	0.23	0.33	0.39	0.19	0.41	0.28	0.006	0.002	0.004	0.003	0.0008	1		
rs722952	13	84.03	TCAAGTAAAAGGTGCTTAGGATAATATTAGGGGTTTAAAACCACTGAAAGATCA[A/G]GAGAGTGGTAGTGAAGAGAGCTTCCACAATAGAGGATTTCACACCAACGTTGTCAAA	A	G	0.62	0.46	0.47	0.48	0.50	0.50	0.79	0.56	0.39	0.41	0.45	0.011	0.070	0.004	0.001	0.0005			
rs1411310	13	84.25	AATCCATGTTAATTTCATAAATGTTCCTATAGACAACACACATTTCATATAGAT[C/G]TTCAGTACATTATCTTATATTGTTGCTTGCTAACTAATTAAAATTATTTTTTCTATAAT	C	G	0.26	0.62	0.59	0.62	0.57	0.64	0.42	0.54	0.71	0.58	0.56	0.070	0.026	0.003	0.000	0.0011			
rs2042766	13	86.54	TGTTTTTTGCAAGGTATTAAAGAAACAGATGACCTCAGGAAAACAAAATAGCT[A/G]TGCAAACAGAAATGTACAGAAATAGAGGAAGTCTAAAAATTTTGGGGCCTTGGGAGA	A	G	0.60	0.33	0.38	0.38	0.27	0.18	0.22	0.52	0.13	0.40	0.39	0.015	0.016	0.019	0.026	0.0005	1		
rs1762899	13	89.12	ATGGTATCTTAATTCACAAATGAAGTATCATCTGTTCATCCTCAGTTTCCTCTTT[C/G]CTTTGGATTTGATGCTATTCACCATTTTAATTTTTTAAAGACTACTATTGAAAGTCTGCT	C	G	0.21	0.35	0.27	0.26	0.29	0.64	0.54	0.29	0.46	0.30	0.27	0.004	0.029	0.003	0.058	0.0000	1		
rs898271	13	89.44	CTTGAATAAATGTTCTTACATTTTTGTTATAAAACATGACTTTTTATGTCAAAC[A/G]TTCTCCTTAATACTGTTGATTTGTTGGTGACACGTGGCTCCCTTTCTAGAGTAGGATGGT	A	G	0.71	0.47	0.42	0.56	0.54	1.00	0.84	0.71	0.61	0.45	0.39	0.076	0.136	0.081	0.315	0.0001			
rs1411316	13	89.66	AACCATCCAACTTTAGGCTCATCCTTAGTATTCTCCCTGTGTGAGAGTCTGGG[A/C]AGAGGGAGGGACTTGGCTGATTTCAGGGCTCTGAGACTCTAATAAATCTCAAATCTGG	A	C	0.62	0.51	0.46	0.50	0.39	0.50	0.49	0.49	0.43	0.52	0.47	0.006	0.000	0.001	0.000	0.0000	1		
rs716623	13	90.68	TGCTGCCACCATAAGACAAAATTCAAATAGTATAAAGTTAGAGGGGAATTGCC[A/G]TTCTTCCCTCTGTGTTATTCATTCACTTTAATTTGAGGAGTTTATTTAAATAAAAACAT	A	G	0.60	0.67	0.64	0.61	0.48	0.41	0.88	0.47	0.68	0.45	0.55	0.000	0.049	0.013	0.024	0.0047	1		1
rs1867438	13	91.47	GAGTATCTTATGGCTTGTTCTGTAGGTTTGTAGCTTTGTACTATATAACAAAA[C/G]AGATTGAATATGGGCTACTTTGCTATTAGTTAGAAAGAAAGATTTACTTCCCAACTCCCC	C	G	0.31	0.38	0.47	0.49	0.32	0.45	0.79	0.44	0.39	0.47	0.44	0.004	0.085	0.000	0.002	0.0004	1		
rs1408209	13	91.71	ACTGTCACTTAGAAGTGAAAGGTGTGTGCTACTGTAAGGCTACAGGGTAAGA[A/G]CACACATGACACTAGGTGAAATAGGATGTGACATAAGCTAAGTGATAGATTCAAAAAG	A	G	0.55	0.32	0.32	0.29	0.25	0.91	0.64	0.54	0.25	0.34	0.27		0.050	0.004	0.176	0.0029			
rs719223	13	92.43	AATAAACTAGGAAAATGGAAGATGCTAGGAAATAATTCACAAGCAACACAGTCAGT[A/G]AGTGTAAAATGTGAGAAAGGCATGTAGTAGGGCAGAGAGGGGAGAGCTGACT	A	G	0.02	0.35	0.36	0.40	0.32	0.05	0.17	0.20	0.32	0.38	0.37	0.119	0.046	0.016	0.118	0.0000	1		
rs766606	13	93.35	CTCTGATCAGAATCACCCCTTATTCCAGAGATCCCAAGTCAATAGATACAGG[A/C]TGGCTGCACGTGAGAGAATTTCTTGAAGTTCCCAGCTGATTCTAATGTGCAGCCAGGT	A	C	0.75	0.52	0.53	0.56	0.46	0.82	0.85	0.33	0.43	0.47	0.51	0.016	0.059	0.034	0.036	0.0002	1		
rs1327634	13	94.56	GCATGAGTTTGTGCACACTCCAAAGTTATGTAACAGATAATTTCGGATTTTGA[A/G]TTTGGCTTGGATACAGAACTACCATAGACTTGTCATTGTAAATAAGTTTTGAATGCG	A	G	0.62	0.48	0.48	0.40	0.46	0.55	0.23	0.46	0.61	0.42	0.48	0.007	0.041	0.001	0.001	0.0006	1		
rs1925121	13	94.64	CTTTCCTAGCCACAACCACTGTTAACTCTGTGGAGAATATCCTACTGCATATCTTGTTGTTATCTGTATGTCTTCATGCCTATAGACCTA[C/G]AGATTCTATTCATCTGTGTTTTG	C	G	0.26	0.53	0.48	0.53	0.54	0.41	0.76	0.42	0.43	0.48	0.48	0.021	0.048	0.001	0.001	0.0000			
rs729174	13	94.75	AAAAACATTAAGTGGTGAGGGTGGCTAAAATGTTGAAATAATTGCCACCTCT[C/G]CCTTGTTTACACTGTGAATATCTTTCTATTAAGGCCTCATTTGTTCATCTATTGCTTTGTGG	C	G	0.67	0.47	0.52	0.44	0.46	0.36	0.07	0.54	0.54	0.55	0.50	0.007	0.146	0.000	0.023	0.0005			
rs1164591	13	94.79	GAACTGACTACAGCAGGAAGATACTGAATAAGCAAAGGCAATGCCTCAATCT[A/G]AAATAATAATCTACCTTTGTATTCAAATGTCCCCCTCATTTCTGAAAACAGACATTGAT	A	G	0.67	0.41	0.37	0.34	0.39	0.59	0.72	0.51	0.43	0.39	0.34	0.024	0.024	0.000	0.005	0.0028			
rs1415707	13	96.19	CTGGTCTGGGGTTCTGGAGGAAGTGATATGTCTGCCCAGGGGTAAAGAAT[A/G]AGTAAGAGCTAGCCAGACAAAGAGGTGGAGAAAAGAGTGACAGGCAGGGGAACACC	A	G	0.83	0.48	0.44	0.49	0.39	0.68	0.86	0.50	0.43	0.48	0.51	0.091	0.118	0.004	0.034	0.0038	1		
rs1415708	13	96.19	TTATTCCTTGGTGTGGTGTTTATTCACCTAGCTGTCA[A/G]GCTTTCTTCCTATCTTCATCTCTCATTAAAACCTGTTCAGGTGCTTCATTTGGACTCCAGTGAATATGAACTCAGA	A	G	0.83	0.52	0.52	0.50	0.66	0.95	0.88	0.63	0.71	0.49	0.48	0.065	0.091	0.003	0.153	0.0004			
rs1933121	13	96.25	TATATTTATTGTGTAAATTAATTAAGAGCTCCACATATTTATATTAAAATAAAAT[A/C]CCATTCATGCAATTGATCTTTATCATTTTCAAGGAAGCAAGTCCTGTAAAGAAAGTG	A	C	0.31	0.18	0.14	0.20	0.09	0.55	0.23	0.26	0.29	0.27	0.12	0.024	0.008	0.006	0.090	0.0001			
rs1626391	13	97.03	GCCTGGGTTCAAATCCTGGCTCAGCAAGCAGCCAGATATATGGCCTTGGACCA[A/G]TTTACTTAAATTTCACGTTCTCCAGTTTCCTTGTATCCAAAATAGGAATAACAAACCTT	A	G	0.83	0.49	0.45	0.33	0.45	1.00	0.80	0.71	0.50	0.49	0.46	0.083	0.084	0.041	0.251	0.0000	1		
rs1324984	13	97.34	AAGCGAAACTCCATCTCAAAAAAAAAAAGAAATTACTTGTTTGCATCCCTTTTAGTCC[A/G]CTTTCAGACATCGTACTCTTGAACTAGATTGTGTGTTCCTTATTTCCCAAACCTTC	A	G	0.40	0.55	0.52	0.61	0.54	0.23	0.73	0.43	0.36	0.53	0.46	0.006	0.024	0.011	0.056	0.0008			
rs1556800	13	98.51	AACTCAACTGTTTATTTGAAGAGTAATTATAAAACAGAAAAGTTTACAAATCACC[A/G]TTACACTGAATGAATGATACCTGGTTTATATATACTGCATGATATTTGAATTAAAG	A	G	0.83	0.84	0.80	0.83	0.91	0.91	0.96	0.81	0.96	0.88	0.83	0.002	0.035	0.003	0.017	0.0003	1		
rs2039677	13	98.55	TCCTCTTCTAGGTTATTCTGCTCCTTCCTACCTTTGTTGATGCCCCACAGTCA[A/G]TGTTGGGGAAAATTAATTAGGATTTATATTCCAGTCTTCTCTAATGTGTTGTCCACATTG	A	G	0.71	0.72	0.73	0.63	0.68	0.32	0.28	0.44	0.68	0.79	0.81	0.000	0.093	0.045	0.080	0.0064		1	1
rs1836845	13	100.08	CAAGCATCTTTCTTCTCTTCCATATTCAACACAGTGAATTCATAATGGAGACAATAC[A/C]TCCCTAAGTTGCTGATGGTGTTGCAAGCACCATTCATGTTTTGGACATTATGGCAGT	A	C	0.76	0.63	0.62	0.56	0.57	0.45	0.79	0.64	0.75	0.60	0.56	0.005	0.006	0.001	0.028	0.0032	1		
rs1328357	13	100.13	CACACCACTTCCATCATGGTTCATCATGGTTCTCCTTGT[A/G]TAAGTAGCTCACCTATTGAAGCAAGCCTAAACCTCCATCGCCTTGCCTCAGTCTGCAGCTGATGGTCTTCCA	A	G	0.24	0.54	0.56	0.52	0.55	0.09	0.29	0.49	0.39	0.52	0.63	0.035	0.022	0.000	0.097	0.0091		1	1
rs536863	13	100.53	GGATCTGGCTTACCCAGACTCAAACTCTGGCTATCTTTTCTCCACTCTGAATG[A/G]ATGAGAGAAAAATCAGTTTTCTAGAAAATTCATATCTTCCAAATTAATAGTTGTTTAGTT	A	G	0.38	0.63	0.65	0.66	0.57	0.73	0.74	0.63	0.71	0.61	0.63	0.054	0.000	0.001	0.000	0.0002			
rs1415055	13	100.64	CTATGCAGTTATTTATTTACTCTGGTGAATAGAGAAACAC[A/G]TTCTTGACACGGGGAAGGAATACACTCCTTTAAGCTTCTCCTAGCACCACTGTGAAAACTTCTAGACTTTTTTT	A	G	0.48	0.42	0.45	0.43	0.41	0.36	0.54	0.42	0.50	0.39	0.32	0.000	0.006	0.001	0.003	0.0111		1	1
rs677594	13	101.01	TTTATCCTGATACATATAATCAGGTAGAATTGGGAATTTTATTCAGCCTCCGC[A/G]TCGGGGAACATAGAATAACTCTTATTGACTCAAGACTGGAATTTTCAGAAAAGTTTAA	A	G	0.68	0.54	0.49	0.52	0.39	0.32	0.24	0.52	0.50	0.48	0.48	0.014	0.050	0.000	0.020	0.0004	1		
rs1412953	13	101.95	TCCAAGGATCACTTTCAATCAAAAGTTTCCCTATTTTTAGACTTCAGGGAAG[A/G]GAGAAATGATGTTGCAATCCCTCCACCTCTCTAAATTCCTTTTTAATGCTTTCTTACATG	A	G	0.19	0.75	0.76	0.77	0.77	0.68	0.99	0.61	0.69	0.74	0.73	0.186	0.050	0.024	0.011	0.0014	1		
rs1330518	13	102.76	TTGTGGGTATATGCATGTCAATGGCCTGATATTTTTGCACTGAGTCCTGCC[A/G]AAAGCTATTTCTTACCTCTGATTTTAAAGAGGGCCTTCTCACCCATCTCCCTCAGCTTTTG	A	G	0.33	0.61	0.65	0.55	0.59	0.86	0.89	0.67	0.61	0.56	0.57	0.058	0.028	0.000	0.020	0.0031	1		
rs4476030	13	103.49	GCGGATTTCACGTCATTTCCACATAGCACCCTATCCAGTGAGAGGAAATATCTGC[A/C]TCTTTGTGAAAGTGACAGGGATGTGCAGGCACAGTGCAATTCCTTGGGGAAGTGA	A	C	0.17	0.24	0.27	0.25	0.16	0.27	0.13	0.35	0.36	0.28	0.23	0.007	0.019	0.005	0.000	0.0021	1		
rs981900	13	104.19	ACAGTCCTAGTGGGGTTTTGTGAATGTTCATATAGTATTAACACACTAGCACTG[A/C]AAGAATGCAAATGATAAGTAAGGGAAACAAGTACTTAGTTTCCAATATATGTCTGAA	A	C	0.78	0.60	0.68	0.65	0.77	0.91	0.99	0.80	0.64	0.79	0.72	0.010	0.140	0.047	0.051	0.0031	1		
rs1969888	13	104.55	AGTGTGAATATAAAATAAATCTGTAAAATTCGAGGAAATTCAGTAAAGCTTGAT[A/G]GTCATCATTACGCATTCAGGAATTACCAAGCATTTGAACATATAATTCTAATAATTGAT	A	G	0.76	0.32	0.31	0.30	0.32	0.50	0.64	0.32	0.43	0.31	0.23	0.111	0.054	0.000	0.021	0.0057			1
rs2039120	13	104.61	CCACAACCAAGGTGTTACAAATTGGCATTTATGTGATCTCAAACCTAAAATATG[C/G]CCAAGATCATCCAGCCCAACCTTCTACTTAATTAGCAGACTTCTCTTCTCACTACCTTC	C	G	0.52	0.18	0.23	0.20	0.25	0.27	0.28	0.38	0.14	0.22	0.22	0.051	0.001	0.013	0.001	0.0000			
rs354439	13	104.64	GTTGCGATAGAAAACAGTGAATGATATTCAGAATATTGTGCTTAA[A/T]AGGAATGTGATACATGAGAGAGATACATAAGGGAAAGAGAAGCCACCAGAACACCCCTCTTTCATC	A	T	0.40	0.61	0.56	0.51	0.54	0.64	0.72	0.42	0.54	0.57	0.56	0.014	0.018	0.008	0.003	0.0001			
rs718831	13	104.74	GTACCTGGCCGAAATGTCCTGCAGGTCAGGAAAGATTATTAAGAAAATGTGTCAGCAG[A/G]GCCAGCTCATCAACTGTGTAGATCGTGGTTTGATGTGGAAAAGGTGAAGC	A	G	0.45	0.49	0.51	0.50	0.57	0.91	0.27	0.69	0.43	0.49	0.52	0.000	0.035	0.035	0.119	0.0000			
rs767327	13	104.76	TTTTAAGGTAGAAATCATGAATATGAAATGCCTGACACAGAGCATGTGTTCACA[A/G]AATGATTTCTATTATCAAAATAGGAGCCAAAACACCCCACCACAGGAAAAGTGGGA	A	G	0.29	0.46	0.48	0.49	0.39	0.09	0.69	0.30	0.58	0.52	0.49	0.027	0.036	0.034	0.108	0.0003			

rs1407584	13	104.84	CAGGTGCCCCTTTCATGATTTAACACCTTTGTTAACTGTCTACAACTGGCCAGA[A/C]ACTGTGCTGCTATAATTAGTATTATTTCCTTGTGCCGTATGCCTGTGTACCTGGTGT	A
rs1411736	13	105.06	ACAGTAATACTCCTCACTCCTTACACACAAGGAAAATTCCTCCTGGAAGAAT[A/G]AGAGGTGCGCCAATTTGCTAATTACAGCTACCAGTAATGTTAACAATACTCTCCAAG	A
rs951749	13	105.08	AGTTATGCCCAAGAATCAAGGCTGGTTATTCAGCTTCCATGTAAATAGCATTCC[A/C]CTTGCCAGTGTTTACACTAAACTGAGCAATACTAAACATGGAAAATCTTTGTATCATG	A
rs53248	13	105.16	GTTGATAAGTATGTGCAATTATTTCTTTTGGAGATGGAGCTATAAATCAAAGGA[A/G]CACAATGATTATGCTTAGTTAGAAAAGAAAAAACTAACATTCTAAGTAGTGGTCAGTTG	A
rs716504	13	105.76	AAGACCACAAAAAAAAAGAGGGTTCAGTTCTGTTTGGTTCATCATTGTATCT[C/G]CAACATCTAGCATAGAGCCTGGTATTCTTGCATCTCTATTTGTTGAGTAAATAAATAAATG	C
rs4253	13	105.87	CAAATGTGGAGAACAATGGCTCTGAATTCCTCCCTCCTTATGATATCCAGTACC[A/G]TTTCCTGGATAATTTTCAGAATGACCTCATTAGTTCTTCTCCTTAATCCTCTGACATCC	A
rs719185	13	105.98	GATTGTCTACTGAACATCACTTGTTGGGCAGTGACTTGCTTTTTCGAAAACC[A/G]TCTAAAACTCAGTATCTCCCCTCACCCTTCCAGTTGCTCTCTCTTCTCACTTTTTATCTTG	A
rs1876723	13	106.31	GTTGAAAAGTGGTGAATTAGACAGAGAAACAATGGAAAAGAGTTGTAGATGGGG[A/G]TTCTTGTGCCAAGTCATGAAAGTCCAAAGCAACATAAGTTGTTTTGGAAAATATAAG	A
rs1856277	13	107.15	AGTATGGCACAAAACTTCTACAACTCCTGACTTGAGAGTTTTAATTATAATATGGG[A/G]TTGGACACTTTAATTTGAGGTTGGTTTGTGACATAAAGTGATCACAGAAGTTGTATT	A
rs1010172	13	107.54	CTGTGTCCTGCAAAACAATGTAAATTAAGACACTGGCTAATGTAACAGGTTGGGG[A/G]AAGTATCCTTCTACTTGAGTCCTCTCTCCTCTGAAGGCTATGTGTGCCATGCTACC	A
rs2031836	13	107.74	TAGTGCTTGTGTTTTGCCTTCCTCGTAGCATCTCAGCGTTAAGAGTAAGACATGC[A/G]GTAAACAGCGCTTCTTCAAATCATAAGAAGCAACGATGTCACAGAAAAATTCATTGA	A
rs2076914	13	108.04	AATATAAATGTTGGAGTTATCACAGTTGGACAAGGATTATGGTGGTGTTCTCT[A/G]CATTTCTATGAAAAAGTCACTATCTGGTAGTGGCCACCGAGCTTTTATTAATTTTGTTAT	A
rs912947	13	108.85	AGAGTGAGATGTGATCTGCAAGCCT[C/G]GAGACATCCCTGCACCCCAGCAAAAGCCAGGGTTGCTGGCCTCCTTGGTCCTGACTTCCTCTCCTGTGGATGTCTGGTTCTAGCT	C
rs1894758	13	109.74	CTCCAGGACGCAGTGCTTCTGTGATGCTGATTGTATCCCAGCATCTTGCGCGCTC[A/G]CTCTGAAATGAACTTGTGTTTCAAGACCATGTTGTGGTGGAATGTGGCAGTTTCCA	A
rs1105513	13	110.02	AGACCTGGTCATTTTTTTCCATTTGGAATCATCGATTTATTTCACTTAAGTTATCA[A/C]AGTGCAAATCATAGCTCCCGGTTTCTTCCAGTCTCGGCTCCTTTTGAGCTGATGTCG	A
rs726455	13	110.60	AAGAAAGCTAAATGGCAATACTGGAGTCATCCCTGTGAAAATAGTACTTACAGC[A/G]GTTACACAGTAGTTACTATACATTCATCCTCTTGCCCATCAGTTAATTACATCACCCT	A
rs204218	13	110.98	TTGTGTATATTGTGTTCGGCCAAACAGGAGGCCAGACCTGAAAGGAAGCCAAGGC[A/G]ATAAAATAATCAAAGATGGAGGGTGAGGAATTTGATCAGATATCAAGGGTGACC	A
rs748144	13	111.05	CTGATGAGACCTGGGCCTGGCCACAGCTTGCTGTCCTCAGGAGAAGCCCTGGGC[A/G]GGAGGTGCCCAGCTACAGGCCCTGGATTCCCGACCCCATAAACTGTGCCATAAT	A
rs1885688	13	111.84	CCGTTAAGAAATAAAGTTTATAAAGCAAACTGATATGCCAAATATATAATAGTTCTC[A/C]CTGAGGAAAGGGCATGAGGAGATCATGTCTGACAAGCAGATTTACAAAAATGATT	A
rs755992	13	111.95	TTTGTAAGAAAACACACAATGAGAAATAATGTGCTCTGAGGCATTGAACTAATGTT[C/G]GCCCAAATCTAGTTTTTTGTAAGCAAAGATAAGGCCTGGGGCCAGAGGTCGGAAA	C
rs755993	13	111.95	AGAGATAAAGGACACGGCTGAGAGCTGAGAGCTGAGAACTGAATAAACCTCG[A/C]GTTGTCAGCTTGTGTGTGCAGGAGGCATCCCTGCCGGCCTCTTCCTTCCTCAGCACC	A
rs912007	13	111.96	ATCCCCATTGTCAAGCACCGACCCCCGTGTCCTTGCCCAGCCCCTGGCAGTCCCACTGT[A/G]CGCTTGTCTCCGTGCTTTGGCAACTCCAGGGTCACATAAGAGGAGTCCCG	A
rs14067	13	112.06	TCCCTTCCAGTCCTGTCCCTGCTGCCCTCTGTGACCATCTCTGCTAAGAA[A/G]CATCGACAGTTCGTCCTGCAGCCTCCAAGCACACTAACCTCAGCCATCAG	A
rs1972373	14	18.44	GTCTCTGGTCCCCCAGATTATTTATCAGTACTAAACAGAAGTGGACATAGGCC[A/G]ATATAAAACATACCAGTAAATAAATTTGTTCAGGTGGTTGTTTTTCATGAACAACATAT	A
rs944398	14	18.52	GATCTTCTGTGGGTGGGAAGCCAGTAAACCTGTCCAAATGGCTTAGATAGGTGTGTC[A/G]CCCAGTAATTCCCTGCACAGGTAGGATAGTTCTCTGACTATGGCAAAAAAAAA	A
rs1959344	14	18.68	TAGGAAGTATAATTTATGACAATTTTTGAGATACAATTTTGAATTTAAAGAAATATTATCCCCC[A/C]ATACCCCATGTTCTCTGACCACTCTTGCTAAACATGCTTTGGTGGAAAG	A
rs1057605	14	18.75	AGTGCTTCTCAAAAAAGTTGAACATGTATATACTCTATGATCCAGCAATT[A/C]CATTCTATGTCAATACTCTTAAACAGTGGTTCTCAAATTTTACTGTGCAT	A
rs1760912	14	18.81	ATTTTACATTTGTTGATTCTGTTTTCTGGTTTACATCCGATTTCTATTTCTTTTAT[A/T]GCTTTATAGAGCCCCGTGACCTAGAAATAACCCTGAGTGGTCAACAGGTCTACCTGTG	A
rs1713430	14	18.81	GATTTAGCATGACATCATAGACATCATTTCCTTCACAATACACATGAGCCTAGAAAG[A/G]GAGAGAGAGAAAAAATCAACATTAGTATAGGTTCAGCCCTGACATCCTAAATGAG	A
rs2700	14	18.82	ATGCGGTACCTTTTAAAGGTTCAGTTTAATTTCCTTCAGCTGTGGTGAATGTTGAT[A/C]TTAAATAAACCAGAGATCTGATCTTCAAGCAAGAAAATAAGCAGTGTTGTACTTGT	A
rs1713419	14	18.82	AGGGGAGCAAAAGGCAAAAGATTGAAATCAATGACTAGGATGATGCCATGCCAT[A/G]GTTCGCTGCAGGATTTCTGGCAAGGATTATGGATCATAAATTTCAAAATACCATAACT	A
rs878157	14	18.82	GAGGGTAGAAGCCCCTGTGAATTTCCGCCTTCTGAGGGAAAGGTGAGGTATCTTTA[A/G]GAATTTAAAGAAAGAGGGACACAGTAAGAGATGACAGGTTATCAGTGAGCTGG	A
rs1713423	14	18.85	GAGTCTTCTCCCTCCCTCAAGCACCATACCCGCTTCCACAGCCCACTCCATTTCT[A/G]TGTAGAGGACTGGGAGAGAGCTTCCCTCTGCCCCCATCCTATAGGATTCTTCTAA	A
rs1543494	14	19.82	GAGATTAAGCTGCTTAGAAGATAAAGTAAATCAGTACAATTTCCCTGGCCTGAAAAAG[A/G]AATGTCTATAAATGCTGTTGCTTTTCTGCAGTACATACCGAGCATCAAATATTA	A
rs2242529	14	19.99	GGGGTAGGGTGGGAATGGAAGTGAGAAAGACGAGACAAAACTTCGAATATTTTG[A/G]GGTGTGTGTGCTGGTGAGGAGTGGATGTTCTGATCTCCCCCTCTTCTTCTGGCCC	A
rs1018344	14	20.18	TGTATGGTTAACGAAACTTGATGTATTATGATGGGGAAAAGGGGTTCATTGGAA[C/G]GGATGGCATGCTAAAGTTTTATACTAAAAAAAGGCAACAATAGCAACTGAGTGATTC	C
rs718433	14	20.23	CATGGAATGGCATAAATGCATCTAAGGAAGTGACTACAATACATCCATCCTGGC[A/G]TTAAAAAGTAGAAAAGAAAGGATTTCTGATATATATAATCAGAGAGCTGCTGTATTTTA	A
rs4981390	14	20.38	CAGATAAAAAATGGAAATCAATTGATTTAGCTATTGTGAGTGCTGTATCTTTATGAC[A/G]GGTGAGTGCTTCAACCCATGTAGAAATAAAACAACTCTTACTAGTGCATACTCAC	A
rs3811310	14	20.52	AGTGCTTGGCTAGATCTCATTGTGTGXCTTGACGCTTGGGGTACTCCTGTTCCCAA[A/G]TGGGTATCATTCCATTTTACTCTCTTGTCCACAAATGGAGCATTGTCTATTTGACT	A
rs4982599	14	20.74	TGAACTCATAGATAGCTGCAGCTGGCCTGAGACTGGGAGAGAAGACAGCCCAATC[A/G]TCTGGAGCTTCCTATACAGACTAGAGAGTGGTGGCACATGCCTGTAGTCCCAGC	A
rs3751467	14	20.95	ACCCAGTAAGTTCTTCATCCTTGGTCAGGAAATCAGCCTGCATAAGATTCTGGGG[A/G]TCATAGTAGACATGCAGGCTTAGAGAATTTTCATCTGTCCTTGTTCATAACTGGTA	A
rs3102229	14	21.10	AACACTGCTGCTCTCATGTGGAATGCACTGTGGCTTTTTGGTCAAGATAATTACC[A/G]TGGAAGATCCTAGGTGGCAGCAAATCCCAGGTGAACAGACTGAGCCGTGGAGGTG	A
rs1570342	14	21.36	TCATAGCCAGCAGCTTCTACCTAAAAATAGTGCATCCTCCTTCCCTTTTCCCC[A/C]GGTCCAGAAAGCAAACTAGGATAAGATGGGGGAAGGGATGGGGGTGGTAACCAGGA	A
rs977870	14	21.72	CATTCCAAGAAGAAGAAAGACCAGTCCAGAAAACAACCAGCAAGGAGTATTAAAGAAG[A/C]AGTGATTTTGCCTGATGCCAGGGTACAAATGTTTAGCCTATATCAAACACATTC	A
rs1958315	14	22.23	CCTGGTGCATTCCAAATATATTGTTAACCCAAACCTCTCCTCTATATTTTAAA[A/T]CCAAATAGCCAACTATCTACTGAAAAATTTACCTCAAAATCAACCTGACTAAAATGGAGC	A
rs5244	14	22.97	ACAGGAACTGCATGGTCACGTTTTCTTCTCTCACTCTGAATTGGCTGTTTCTGATC[A/G]GAAGTTCCCCTAGAATCACCCAGTGTTTTTCTAGAAAGGAACTTGAGCCTGAACTC	A
rs1957528	14	23.10	CAACATCAGAATCAAATGTCATGTATGAGTGTGGGAATGTCATGTCATT[A/G]TGCTGTTCTCATTTACCTGTAAGGATTGGCCTTGGCCACTTCGAAAAGGGACTAGCTGTGGT	A
rs941738	14	23.87	TTCTGGCTGCCCTTTTAGCTCACTTATTTTCATTTGCTTGGTTTCAATAGAT[A/G]TAGTGATCATGCTGGGCCCAGCTAGCCATATTACTTCCCTACCCTACCTTCCCCAGGCCT	A
rs927263	14	24.08	CACATCATGGTAGGTATATTTAAATTCAGGGTGGTAGATGGAAAAAATTGTCT[C/G]TCAGCCCTTGTAGTCCAAATCCCCAGGCCCATACATTTCTTTAGAGAATGAGTACTTAC	C
rs1956775	14	24.08	TGTAATACAGCTGTCCACTCATTCACCTGAGCAAAGATGATATGGTGAGGGACC[A/G]TCTGGAAGCTCTCTTTTATATTTCTTTACAATGTGTTTCCAACAAAGATATCTGAGTGT	A
rs1950948	14	24.40	TGAAAAATTTCTATTAGATATCCTTTTCCTTCTTGTCACTTATCATTTTGCCAC[A/G]ATAGCCAGATGATGCTGTTTTCTGCCATGGGTCTCTCCTCAGCTATAGTCAGGCACCAGG	A
rs1956616	14	24.44	ATAGTCCAGAATTTGCATGAATGAGGCCCAGAAGAGTACTCTGTCTGAGTTTTAATGATGTTCATGAACTG[A/G]GTAGAACTTGGGAGGAAAAGATAAAGATTTTAAAAACCCAA	A
rs862952	14	24.48	ACACAGTCTCTACCCTTCAGCATCCAGGGTTCAGTAAGCATGGAGGCTGACAC[A/G]CTAAACTGCAAGTAATAACATGGTCACTCCCATGGAGCAAATAGGTCAGGGCTGCAG	A
rs2144043	14	24.63	AATAACAACAATAAATCAAAAATATTAAATGCTTCCAGGGAAACATTGTCTAATAG[A/C]ATTTTCTGCACTAACAAACATGTTTTGTCTTCATTGCTAGTGTCACTAACCACATATG	A
rs911085	14	24.74	TTGGGCCTCTATCTAGAGTCGAATTGAGGCTATGTAATCATCAGTCTTTCACAAA[A/C]TTTTTTGCAGCCCTAACATAAAAGTCATATTATAAAGCTCCACATTTGGCAAATGGTA	A
rs1951085	14	25.28	TTGGGAAAATAACATCTACTAATTTAAAATTTGTCACATCCAAAATTTGTGCT[A/G]TAATAGTGGTAAACGATGGTGTATCAGAATTCTCTCTTAGCTATTACTGATGTGTTTAATT	A
rs880433	14	26.48	GAACTTGAGGAAAATGTATGTATGTATTATTTCTGAGTTGAGAGAATTAAGTAC[A/G]TGTAAATGTTAAGGATGGCCAGTCAGAAGGTCAAGGGACAACCCAGATGTGGGCATG	A
rs176264	14	27.15	CACACTGCAATCCATGTGAGCCCCACCTTTCAGAGCCCTCCTGCAGTTGAC[A/G]ATACTATTGAACTAACTCTGGACAACAGACTGTGTACAAAAATGATGTGTGTCCACTCC	A
rs1957115	14	27.81	CATTCTGCAATTAAGATCTATAATTTTAGTGATATGCAATTAGTTTTTTTTCTT[A/G]AAGCTATTCTCCACATAGTGTGGGTCAAAGCTTTATTTTACTTTATTCTTCAAAGGGCCCA	A
rs11984	14	28.04	GATTATTTCCAGTGTTCTATGAACAACTCCAAAACCCATGTGGGAAAAAAATGAAT[A/G]AGGAGGGTAGGGAATAAAATCCTAAGACACAAATGCATGAACAAGTTTTAATGTAT	A
rs179562	14	29.21	GTTTTTCCACCGCCACCGGTCTAGAAAGGAAGTGGTAATCCTGGGGTTGGAAGCC[A/G]AGACTGTGACTCCAGGGACTGCTCTTCCCTGCATTGCCCGTCCACTTAGCAGCAT	A
rs2273171	14	29.37	TTGTGCTCTTTCCTTTTAGTTGCCTGCTAATAAAGATGCCTTTCGAAAGACATGGA[A/G]TCCCAAGTATACACTACGTAGCCATTTTGATGGAGTACGGGCATTAGCTTTTCATCC	A
rs7160965	14	29.50	ATAGCAAAAGAAAACATCTACAGACAACATCTCTACCAAAAACTATGTGAAAAAAC[A/G]TCCCCTGGAACGTTTTTTAGGATGCTAATTAATTGGTGGGGCCAAACTAAGGTAG	A
rs727675	14	29.72	ACAGTGAGAATGAAGGGGAGGGAAGCTTTGTGAGTAATAACACAAGTGGTTAA[A/G]CAAACAGAAGGACTGTGCAAAGATGAAGAGACCCTTGTGTGTAGGTTAACCGAGCA	A
rs6571428	14	29.95	TAAAAATAGGCTTAAATTTCAGTTCTTAATTCATTTGTGGCTGTGACCTTCAACCT[A/G]AGAACTGATCTTTGCAGGTTAAGGATAAATTTTCAAACCTGCTACTGTGTTTTAAAAG	A
rs2378944	14	30.19	AATGCCCAAAATAATCTTTACAAACAAATCCAGAAGCCTTACTCTACAGCAATCAAG[A/G]ACACGTAGTATGAGGCAGGCAACAGCATACAGAATTCAAAAGCAGCCACTCCAT	A
rs718212	14	66.19	ACAAAGCGGGACAACAGAGCAGACAGGGCTGGATTCTGTCATGGAATGGGAAA[A/G]AGATTTCACCCTGGGCTTCCCTTTACTCCCAGACTCTAGTTGCTCATTGAGTTCATG	A
rs961700	14	66.32	AATGCCAAGTTATGATGGTATAAATGAGTGAATGTTTCAGCCTTTCTCAACTA[A/G]AAGTTTCTCATCTGAACTACAAAATACAGTGGATGATTTGAGTGACTATTTTCTCAATTC	A
rs1950769	14	66.34	ATGAAAGTGTCAGTAAGAAAACCTTGAAAGATTATTTGTGGCATGGCTTTTTGTAGCGGGCATTGTGATCAGAAATCTGAAGATGACAGG[A/C]TATTACAAGGGCTTGTGATGT	A
rs1015023	14	66.35	TAAGCATCATGGTACCATACAATGCTTCTGAGCATAGGAGGGATGTGATCAGA[A/G]ATACACTTTGGGAAAATGATGTAGCAATGGTATGTACCCATATGTACTATTACCGATA	A
rs927221	14	66.83	TGGTTAAATCACGATTTTATTTTCTCGTTGACACCTAGTGTGTGTTATATGC[A/G]GCGTCCTCTGCCTCATGTCATTCTCCAATATGCTTTCTCAGGGCTACTTTCCCAGACCCTA	A
rs1043254	14	67.70	GTTTATCTCTAACAGGGGCTGTCCAGTATATCGGTCCTGTTAGGAGGGGA[A/G]AAAAAGTTCTTCCAAAGGCTGGAGAAGTGAACAAGGAGTCAAATTTATATT	A
rs8688	14	69.13	GTCACACGAAAAATACATGAAAGTGCTCTGATAATTTTAAAGCATCATATAAATTAA[A/C]GGTGTCATTGGTAATAATCTCATATGCATTTCTTAACCCTTCCATTTCCTTGAAGAC	A
rs987579	14	69.50	ACTTCTATGAGCTCTGAGATACTTACAAATTTGCAACTTAGACATGATTTAACATTT[A/G]GATGAGTTTCCAACTTTAATTCTTGAAACCTCAGTTTTTAACCATAACTTTTCTCATT	A
rs221924	14	69.57	CCCTTTTTATTCTAGAGGCTTTTCCTTTAATTCAGAGATTAGTGGAGATAATAAC[A/G]TCAGTCAAGGCTAATGTGGTACATGGAACTTGCTAATGGAGGTTGGACATTCTGTTT	A
rs917284	14	70.73	CTGAAAGGCTGGACACCACATCAGAGTCTTTGCCAGTTGTCCTTTCTCCCAT[A/C]CTTTCCTATTGGCAGTTCCTGGGATTATGCTGTTTTATTTCCTCTTTCTTTCTCTTCTCCC	A
rs6574106	14	71.38	AAAAAACTTGAGGCATGCAATTGTTCGAAATCTTACTGCCTCCCTGGTTTCTTCTGC[A/G]TTTGAATTCTGTTGAACACTGATAGGAGGATTCCCTTTAGGGGACACGAGGAA	A
rs7155380	14	71.47	ATTTTTCTCTTCCAATGGCCAGAGTATTATAATGTAAGGACTAATTTTGGTGCAGT[A/G]GCTGGTTAGCCCTTTGGCAAGTTCTGGTCAGAATATGAATATATCTATTATTGGTTT	A
rs977357	14	72.20	CGAGAGAGGTGGCAGGAGCCAAGGTCTGCAGGGACTTGTGGACAGGGCAGGGAGTTTAGACTTTGTCCTAAGACAGCTCAAAGAACTGACTC[A/G]CCTGGAGACTCAGTTT	A
rs1558174	14	72.29	TGTGCATCCCCTGAGTGATCACAACCATCTCCCTCCTATCAGAATTACTATTAT[A/C]CTAACTTTCATGATGTTAATTCCTTCTTGTTCTTTATAATCTACCACCTGTATTTGAATT	A
rs1468507	14	72.56	CATGAGCACAGCTGAGATTGTTCAGAATTATCTTCTACCTCAAAATTTGGTGTT[A/C]TTTCAATTTCCATAGCATTGCTTTGCAACTGATTGCCGTTTTCCTTGACCATCGTGAAT	A
rs1125221	14	72.90	ATTCTGGATAAAATATTTTTAGTGATCAAGCAAGAAAGTAAGGAATTTAAAGAAC[A/G]AAAGAAGCAACTCAAATAATTGAGCAAACCACTAAAGTTGGCTATCACCCTGAAGAT	A
rs4899553	14	73.69	AACTCTTGGCAAGACATTGTCTCTCTCAGAGGCCTTTAGAATGTCTCCAAAGCAC[A/G]CTACAGTGTATCTGCTGAACTGTTAGCCAACAGCCTCTGTATCTCATGGGCAGGTA	A
rs1063169	14	73.74	GCTTCCCTGCTTCCTGGCACCCCACTGTGAAACCATTTCTGACCTGCAGTTGCAGA[A/C]TAGGTGTGACTTGCATTTAAAATAAGATCCTCAGCAAGAGAACAAAGAAGAGCCC	A
rs2205187	14	73.83	CCATCACACCAGGATGGCCCCTGACCTACAGGCTGATCACTTTGTTTTAAGCTGC[A/G]CCATCATCAGTTGGCTCTGTGGAGCTAATTGCTGCCTTCCTTTTGTCCTTTTATTG	A
rs935340	14	74.58	GTGTTGATAGCTTGCCCTGTCTGAAGATCTGCTCATTCTAATCTGATAAAACCA[A/C]TGCAGATCAAATATTGACAGACACCTGTGGGGCTATGTTTGGCTTATGGGCCCTGGTT	A
rs760267	14	75.27	CATGGTACTGAGAGGTGGGTCTGAAAGCAGTTAGGGGTGGGAGGTGCATTG[A/G]CTGTACAGTTAATCCAAGGAGCTTGGCTGACATTTTGAGGACAGTGGGAGTTTGCACT	A
rs888066	14	75.58	GAGGAAGGCAGTAAGTGAGCAGAGGTTTGAGGAAGTTCACCTGATGCAGGTGTG[A/G]AGGACAGATTGTGCAGGAGCCTGGAGGCTGAGTCACCAGCTAGGAGGCTGGGC	A
rs888412	14	76.60	TTCACCACAGCAGGTAGAAAAGAAACGGAAGAAAGAACAAGTCTGACCCAAGAGGCCCAATCGCC[A/G]GGTTTGGATTCTAATTTCAGTTTAGTGTGTCACCTGGGAGATGCA	A
rs2043585	14	77.96	AAATAAAAATGGAGAAATTACATGCCAAGTGAGAAGACTGCACTGGAAAACAGA[A/C]ATTATCTTTTAAAAGCATTTTCAGATTGGTTGAAAAGAGAGGAGCAAAGGAGAGCAC	A
rs178384	14	78.17	AAAATAGGAAGAAGAATCAAATTTTCACGTTGCTGATTTCTGGTTATTAGTGTTCA[A/G]TGAAAGACACAAGCAAGTGAAATACCAGAAGGCTGTAAAGTCCAGAAACCTAGACA	A
rs142922	14	78.62	TCAACCATGACAGAACAGGGTAGAAACTGGGAAAGCCTGAAGAAAGCAAAAGG[A/G]TTGGAAGTAGAGACTTTTATGTGGAGCCAAAAAAAAATGTCCTGGGACAAAATAATA	A
rs1530771	14	79.02	ACATCTTAGATGTAGTTTT[A/C]GAAATGATTTCTGAAACTGTTTCCTTTGGTGTGCTGTGAAATCCACATCAGGAGACAGCAGTAATTCTGATAAAAGGAGAAAGAAAATGCCCC	A
rs2060596	14	79.18	ACCTAGACAACTTGGTCTAGGTAGCGGAAACGAAGTATCTAAGCAATGCAGACAA[A/C]CTAGTCTGAATGGAAAATAACCTTGACTTGATCAGAATATTTACTAGCCTCTATATT	A
rs1863766	14	79.34	CAGTGTTGTAGTCTCTGTGAAGTTTTTTCAGTTGTGATCAATGTTAGTGATA[A/C]CTGCAGAGCCTCAGTGGTCTAGGCTGAAGGAATTTGTGGTAGTGACAGTGACTGTGTAG	A
rs1025253	14	79.38	TAAATTCTGGTTAAAGCTGAGAAAGGTATTTCTTTCAATTGGAGAAAAAGCAGT[A/G]AACACTCTTCTGCAGCTTACTACCACCTGCTGGTGCAACAAAATTTCTTATTAACAAG	A
rs759939	14	79.65	AAGTATGATTATATAAAAATGCTTACTTAGTTTTTTACTGAACACATTTAACATG[C/G]CAAGTGAGTATTAAACTTCTTTGAACTTGTGATATCAAAGTAATTCCTAGAGCATATTA	C
rs799032	14	80.63	GAGGTCTTCTTTCTTCTAAGGTTCCTATAGTAAAAATCTGTTGGT[A/G]GCAAACTCATTCAGTCATGATTTTTGTTGTCTTGTTCTCTTGTTTTAATCTGAAAGTGACTACTTCATC	A
rs1964413	14	81.26	CAAGTAATTCCTATCTCATTATACACAGATTTCCAAGAGTTCAGTATACACCAG[A/C]TGATGCCTGCATCAGAGTCACAAACTGAGCAGGTAACACCATGAACATTAGAAAATAA	A
rs876287	14	81.41	TCTATCCACAAAGGCAAGATTACACCAAACTTAACGTTGGATGGTTTAGAAGCTGT[A/G]TTGATTGTCAGAAATGAGTTATCTCTAGCCTATTCCCTGGTGCTAAATAAATGCATT	A
rs898939	14	82.66	AGGCCACCATATCACAATTGCCTTTAGGTGCTGTTCAGCTACTTATCTGTGAAA[C/G]GGTGACGGGGGAAGGGAGAAAACAGACTTGAAAATAGCACAAAATAAGAGAATAAAT	C
rs726957	14	83.12	TAAGTCCAAAAAGAACAAAAAAATTGTGAAGACTCCGAGAAATTCATGCTGTTAAA[A/G]TGGCTGAGTATATACCCAAACTGCCTCTGATGTGTACTGGCTTCACATTTTTAATGC	A
rs1885316	14	83.85	ATTAAATGGATTTACATTGTTTGTCTGTAGGTATTCCCCTCCCTTTTAAAAAAAT[A/G]CAACTATGACTTTAACTGATTTTTAATGCATCTTTGTCTTTCTTAAAGAAAATATTTTTA	A
rs981270	14	84.17	TGTTTGGGTAGCTACCTTTCATATAACCTTAAAAAGGGCTCTATCATTCTCATAT[A/G]GAACTCATCGCTATAATGAAATAACACCATTTGAAGATGATAATTGTCTTTTATTCCTAT	A
rs1999916	14	84.45	TCCTGTACAACATCCCTTTGCTTGAATGGTTTCTGTGCTTTTCCAGGAAGCAA[C/G]ACCCATTATTACATAGCTCTAAATGCTTTCTTCTCCTGAGATGAAGCCCAAGATAAGTT	C
rs1012921	14	86.34	ATCACAGTGCAGGGCACAGAGAAAGGG[A/G]AGTACAGCATTTTCAATTTGAGGTGGTACAATATTCCTATTGTCTCCCATTTACAATCATGGATTCTAAGTGTCCCTAGGTTGT	A
rs1956411	14	87.12	GACCTGTTTGGAAATGGTGTGGCTGCCACCAGAACATTCAGTGAGCTTGTGGC[A/G]AAAAAGCTTGCCAGAGGGTGCAGCCCTCAGTGGAACAGTGAGAGTAGTCACTTGGT	A
rs719572	14	87.37	GGTTATTGACAGAATTCAGTAGGATTAATTCTAGCTCAGAATTTAGCCGGAGCT[A/G]GTGCTAACTTTTAGCCCTGGAACCCAGCTGACAGAACTAGATCATACCTAGTACTA	A
rs759364	14	87.75	TACCCCTTGTCTCCACTTACCTCCCCCTTCCCAGGACTGATGATTTCTTATCTTA[A/G]CCATCGCCACACATAAACTGGTGTCCAAGTCCTCCTTTTGGCTCCCATTATTCTTTC	A
rs942190	14	88.41	GACCGTCCTCCTATACTGGGAAACCGAGCACAGAAAGCTTAAGCGCCTGGC[A/G]CGTGGTGGGCGCTTTCCTACTCACTTTCTGGGACCACGCACCTGTAAGCTTAGGTCTG	A
rs4904723	14	89.18	TTGTTTTTTCTTCTCCTGCTGGACTAGAAATTCCACAGGGACCATTTGCTGTCTCG[A/G]CCCTAGTTCCTGACACGTAAATATTTGACAGATGAATAAATATGTGAATGGATAAA	A
rs1152431	14	89.36	AAAGTACCTGCTTCAATGTTTGCTGCTATAAAACAGCCAGACAAGCCCTATGAGGCAG[A/G]TAGAGCTCAGAGATGGAGTAAAATCCACAATTTATTATGGAAAATAAAAACATA	A
rs4904745	14	89.55	GAACTTTTTGCATTATTTCCACGTTCAGGAAAAGGCATATGCCCTCTGAGAACAGA[A/C]AAATGGACAGAAGTTTTTATTTTTCATTTTATTTTTATTTCATTTATTTATTTATTTAT	A
rs941914	14	89.78	TAAGGTGTTCCGTCAGTGGCCTCACACATACAGACCTAGGCTTTGCAGCCCCAA[A/G]GATGTTCATTATCACTTTAGTCAACAACTCAACGTCTGGTATTCGGTACCTTACTGGT	A
rs755102	14	89.85	ACTGATGTATAAACAAGAAACACACACAGCGAGCTGTCAGCCACTCGCAAAACAG[A/C]GAATGACTTAAAAAGGATCGAAACACTCAAACACGTGACATTTCAACCAAACACAG	A
rs1298989	14	90.08	AAAGTCAAACTTTCTGTCAACAATTGGTCATTAAAAGTTGTCTCACTGCCTTATG[A/G]TCAGTATCTGGGCCTCACATCCTCCAGGGAGTCATACATTAGCTAATTTCTTTGGTTT	A
rs972905	14	90.19	AGTGACTGTGATTGAAAACAGATCCTTTACTCTTTC[A/G]TAAGTTAGGAAACAAATGTGGGGGCAAATGAGGAAAAGAAAACTAAGAGGTTGCTGGGGAGAAAGAAGGCAAATG	A
rs1998136	14	90.27	TGATAAAACAGCAAGAAACCTAAGACAAGAGCACATAGAATTAGGAAGAAATGTTT[A/G]GGAAGGGAGATGAAAAGGGAAAGGTCAAAAAAAAAAAAAGTCAAGGGCAAACCT	A
rs1242119	14	91.17	CAGGCTAGGGGCACAGAACAACACCTGTTTCAATTCTCTATACGGGGGTCCCAAGCCATTGAGAGAAGTGTAGTATCC[A/G]TAAACAGACATTACCTCTTCAGGACTTTGCCG	A
rs1740696	14	91.49	TTGTGGCCGGGGCTGCCAGTTGCCATTCTAGGGGGAACATGGAGATAAAGC[A/G]AACATTGAAATATGTTTTTTATTGTCAGCAAAAATAAACATCCCAGGCCGGAAGCCAGG	A
rs1740694	14	91.50	AATGGAGGGTCACAAACTGAAGCGCCCATGGGGCAAATGTAAACCATGAGGCTG[A/G]TGGTGTGGGACAACAGAGAATGGTGGGGCCTAAGGGGCAACCAGCTTAAACACT	A
rs882023	14	91.52	TTGGGGACCTAAGTCACATCTCAGGCTGTTTCATTGGGTTATGGTCTAGAAGTG[A/G]CTTCAGGATTTTGTTGCTAAAGAAGAAGGGGAGAATGGGGGACAGGTTGGGCTGC	A
rs733559	14	91.73	TCCATTCCATTCAAGTGCCACCATGATTGGTGATCTGATACTTTACCTTCCACAT[A/G]TACCTCATTTTAATCTGCTCCTGGTGTTGTCATGCTACTATGTGCAGGGGTTATCAC	A
rs760335	14	92.80	ATCGCCGCTAGTATCAGTAATAGCCAGCTTTTGCTGAGCACCTACTGTGTACCAC[A/G]AGCACACACACTATTTCTCTTTCTCAACTCCTTAAAGAGCTAAGCCCTGAGCACAG	A
rs1004958	14	93.03	AAAAACCGAATAAACCCACGCTGCATTTCTCTACACACCAATTTACTAGAGTC[A/G]TGCAAACTTGTCACCTTCCACTAGGCTACATCACTGCCACTCACATTGGCTTCCTCTG	A
rs742893	14	93.14	CAGGCCTCAGCTCACGGGTGACAGCCACATGACCTAGGGAAGCCCCTCTCCC[A/G]CCTGGGCATCAATCTCCTCATCTGTAAAGTGGTGATGACCGCCCCATCTTGCAGGGC	A
rs1361525	14	93.26	CCCCCCAAAAACATAGAAGGAAAGAGACAATAAAGATAAGAACAGAAATGACTGA[A/C]ATAGGAGAAAAAGATCAATAAGGCTAAATCTTGGTTCTTTGAATAAAATAATAAAAT	A
rs1054195	14	93.64	CTCTCAGTAAAAACTTTCAGACATAGTCAATTAAGCCCTGCACAATAAGGAAACATT[A/C]AATCTGCTCAATAGACCAGACTCCCCCAAAATAAAACAATGATCACCACCCACTA	A
rs2369522	14	94.73	ACCCCAGCTCCTATGATCCTGTCCATCTCTTACTATAGAGGATGCAAATTTCAA[A/C]TATCTGTTATCCCTTGGAGAAAGCTTGTTGGTGAACTACACATCTGATTCATGGGAGA	A
rs1022590	14	95.55	GCCATACAATCTCTGTTGCAACTGCTCTACCAATGAAGCACAAAACATATAAAC[A/G]TTGACTGTTGAACATATGAGCAGCTATAGACAATAACTTGTAAATGAGCATAGCTGTG	A
rs8013495	14	95.70	GCCCACCTGCAAGCTCCCAATCAATTGGTTGTCTATCTTCAGTTTGGACTCCT[C/G]CAAGGATCGGAGGCCTTCTGAGGGGGGCAGCTGGATGGGTTGAAAGAGCTTCCTGA	C
rs8005578	14	96.06	TCCCCCTGACTTCCATCTAAAGTGATGCTAAGGGTCCGCATCTCATTTCCTTGACA[A/C]AAATGTCAACAGCATTATCTTATGCATGTGTTGTGCAGATAAGGGAAAGTCACGTT	A
rs1462256	14	96.18	TAGCACTTTTGGTGTATGCTGATGCATTTGCTTTTCATTGTCAAAAGAAGAAAAA[A/G]GGCCACTGCTGTTAGGAAGCTGGACTCCCGGACCAGTTCTTGCATGTACTCAGGGT	A
rs2616774	14	96.40	CTAACATTTCTTTAATGGATTATGACAACTCACAGATCTTTCTTTGTCTTTCGTTA[A/G]TTGCTGCCATTCAGACCGATTTTTGTCTTGCATATGATTTTAATACAAGCACTTGTAAT	A
rs926949	14	97.09	CTGAAGTGCAAGCTGTGACCTGAAGGTCTCATCACCTTCCAGTCAGAAATATAGCT[A/C]TTCTTGAAGGCTAGAGATTTTTCCCCTGCAAAATGACTCAGTTAATGGCAATAAAA	A
rs1955897	14	97.22	CTTCTCTTCTTTCTCTATGCTCCCAAAGAGTCAAAGAACATTGAAGTAATGCAT[A/T]CCTTAAAATTTGAGGATTTCATTGGTAGAACTATCTCAGCCAGGGATTTTGGGAGGAG	A
rs1159799	14	97.24	GCACTACCAGATGCTTCCATACACCAGTTAGCTGCTTCCCGGGCCTATTTGAAG[A/C]AACGGGTGTCAGGTCAGTAATTAGGCTCTTGCAGACAAACTGGAAGTGCCATCTGA	A
rs1257670	14	97.48	GGTTAAGCAGTGGTGTCCACATGGTCCTGCTTCCAGGGGGACCACTAAGCAGATC[A/G]CACAGGAAATGCCCTAGTACTACACACTGGGGGATTTGTTTTCATGTTGCACAGC	A
rs868666	14	97.59	CCTCTGGAAATACATCCTCCACTGAGTACAAACAGGGCCACCATGCCACTTCCT[A/G]TGTAACACCTGGAAATGTCACCCCCCGAGAACAGCAGCCCTGGAGTAGCTCAGGG	A
rs3850422	14	97.66	TTGGGAAAATTCGCGGACTCTCCCAAAGCCTGCATCTCCCACTTCTACAATGACT[A/G]GTTAAATGCGCCCAAGGACCAGGCGCTCGCTACACCAGCATGCCAGGAAAGGGG	A
rs941731	14	97.88	ACCTTTTAATTTCTAAAAAAAACAAGCTGTTGTATTAACCTAGAGGTAATCTTAGATTATTAATCCCTAAAAAAAACTTG[A/G]TGTGGAAAACAGTTCAAACCCTCCTCCCCCAAA	A
rs1951521	14	98.73	ACTTTAGCCCATAAATAAGTGAAAGAACTAGCAGTGCAGCTAGTAAATCTAAC[A/G]TGGTTCTTTTTTGACAACTGACACCAGAACCCTTAATCATTTGTATTTTACTGTTGGAAA	A
rs7159412	14	99.27	TGTATCAGAGAACCGCGGGCAAATGGCGAACCGCGGGCTACTGGCAAACCCAGTTC[A/G]GTGGTAGGACTATGTGCTCTCTTGTCCAAGGAGTAGTTAGCATCTTGGCATGA	A
rs17172	14	99.42	AAATATATACAATTGCATGAAGATGAAGAGGGTGAAACATTGTCCAATTATGAAAT[A/G]TGAGGTGAGGCCTGAAATACTATTAATATAATTTGAAAGTCCTAAGGTCCATAAGTA	A
rs1007904	14	99.95	CCTATCTCCCGCTTATCTCCTCCTGGGAGCGCAGTTGTTTGTCTCTCTTGCCCA[A/G]TGTAAAGAGGAAGGTCAGTGCAGCCCCTGTTGCTCCATCATCATGGTTGGGAGCAG	A
rs872945	14	101.47	CATCTACCTGCCTGTTTCTAACATCAAATACCACACAGTCACATCGTAGAGTCACTGTGAGAGCTGCCATTCACCAGGC[A/G]CATTCTGCGTGCCAGAGACGGTGTTGAGCCG	A
rs8548	14	102.19	TCACAGTTTATTAATGCTTTAAACAGCTTCATGTTTTAGAATTTGTGTATTGTC[A/C]ATACTTAATTGGGGGTGGGAGAGACTGAGCTACACTACTGCTAAACTATTTTTAGCATA	A
rs1547350	14	102.64	GGGGAGGGAGGGCCGGCCACGCGGTGGACAGAGCGAGGGTGCCAGGGTGACC[A/C]GAAGACCGTCACCACCCGACAGCAACGCAAGTGCCTTTGACCTTGATTTGGACT	A
rs1132975	14	103.19	CTCGGGGCCCCTGTAGGGCCAGGCTTTGGGGACTCTGGCAGGTGAACGAC[A/G]GCACAGCCTTCATCCCACACGTGGTCCAGGTAGGCCTGGGCCCCGAGGAA	A
rs6644	14	103.19	TGGGTTGGTGTTGGCAAGTCAAGAGAGTCGATGATCCAGCTGTTTTAGTC[A/G]CAGACTGAGCTGATCCCAACAGGCCCTGGCAGCGTCTGGACTTGTGTAAA	A
rs988131	14	105.16	CTGACTCACAAGTAATTGTGCTGAGCAAGAAGCCACACAAATGAAATGGAAACTGTA[A/G]GGTTTCACTTTAACCAAATTTTGAAAAATAGACCTGAAGTAGCAAACATGAGAGA	A
rs988133	14	105.16	CTGTGCATCCAAAATAACAATCAAGATAACGATGAATTACACTTCAAGGTTTCCCT[A/G]GTTTCCTCTAATTCCTGCCTCCCAGTCATTTTCTGCTCACTACACTGAGTCAGTCTC	A
rs1858691	14	105.20	GCTGGAATTTTTCTGATTCCAGCCAAGAAAGAGGCTGGGCCAGAGTCAGTGTCACGTACA[A/G]GCTCACAGGTTTCACGTCTGCCCCTTCTCCTGATATGAGACTATGCAAAG	A
rs2105992	14	105.23	AACTACACAGTTTCTAAAATATTTGCCAGATTGCTCACAGGAGTTGTACCTGGGGT[A/G]CAGAAGCAAAAGGACTTCTCTTTATCACAGCACAGGCCATGACCTAGGTAAGGTG	A
rs1989749	14	105.26	TCAGACTATTTCTCCATCTTCCCTTTTACTTTAAAAGTCTTTTATCTTCCTCAGTC[A/T]CCATGCAGGAAATAAAAATTCCCTTTACTTTCTGTCCTCCAAGTCTGGTGAACAGTTT	A
rs1562203	15	20.36	GAGTTTTTAGCAATTAAGCACTTTCATTCTCTGAAAGAAAACTGCAAATGAGCA[A/G]CTCTGAAGAAGGCTGCTTAGATTTGCCTCCCAAACAAGCGTTTGTAATCATTTCATTAT	A
rs3693	15	20.55	ACAGATACGTAGTACATCTGTAGTATTAAAATGGCATGGGAGGAGGCAGTTAGAAAAAACATCTAAAACAGCTCCTTAGAAGGCCAATAATA[A/C]AGTTGGAAAAAAGGGAGTT	A
rs999842	15	20.55	GCAAAGATGAGATTATTAAAAATGTGGTATGTGGATGAAAATCTCGCACCATCT[A/G]TGGACCTTTTGCAAAAGCATACTTTCTTTGTGTTATAAGTTCTTGTTTCTGGCAATGTT	A
rs1814767	15	21.25	TTTTTAGCCCCATAGGCCGAGGGATTCAGGAGGGCTGGCTACGGGCATTTTGGAAT[A/G]GAAGATGTTGTACCAACAAATCAAGCTTAGGTTCCTGGCAATTTGCCCACATAT	A
rs7495382	15	21.37	TAGGGGTGGGGAAGTTGCTCATGTCAAATTCATTAGCTCATTTTAAACTGGTT[A/C]CATTTGTAAGGCAGAGCAATTGGTGTGAGTCCCGCCCTGCAGTCAGATGGGCGAGTCT	A
rs1722791	15	21.50	CTTCTGCTCCATTGTCTCTTTGTTTTCTTGTATTCCAACTATGTGTATATGACAG[A/C]TTTTAACTTTGTCCACCAATTCTCAAATGTTCTATTGTGTTTTTCCCATTGTTCTTTTTGC	A

rs898368	15	21.72	TTAAATAAACCTATATTGAAACATATTCCTTCGATAATAGACCAGAATACAACAG[A/G]TCAGCAGTGACATTACACTGATAAATGTGTAGTCACAGAGAAAAAGGGTATATTATG	A	G	0.31	0.41	0.36	0.38	0.25	0.64	0.32	0.32	0.32	0.37	0.43	0.001	0.002	0.002	0.035	0.0040			
rs1966109	15	21.84	GACTTTTGAAGATACTCTGAGTTTTTGAGTTAAGAAGATGGAACTGAGAGTCAAG[A/C]ACAAATTAGCTGAGTTTCATGAAGCCATTCTCCACATTCAGTGGTAAGAATGGGG	A	C	0.76	0.39	0.40	0.43	0.38	0.64	0.83	0.47	0.46	0.57	0.51	0.073	0.114	0.004	0.028	0.0064		1	1
rs1463292	15	22.55	TACAAAGGAAAAATGTGTGTGAACTCACAGAGAAAAATCGATAATTAAACTCC[A/G]TTTGTAACACTAAAGGGAAGATCATAATTGACCTATACACAGTATCAGTAGGAGTCTC	A	G	0.81	0.51	0.57	0.62	0.63	0.73	0.74	0.52	0.50	0.60	0.55	0.037	0.012	0.006	0.013	0.0001	1		
rs975963	15	22.56	TCTGAAATTGCTATTCTTGTGATGCCTCAAATATTTTGACTTGTATACATGTC[A/G]TGGTTTCTACATGAATACTAATCATTCTTTTGTCTTTCCCAGCTTAAATTTGCATCGTTTT	A	G	0.19	0.48	0.41	0.38	0.38	0.27	0.26	0.47	0.46	0.39	0.44	0.032	0.009	0.007	0.009	0.0002			
rs2732029	15	22.78	ATGGAGAAAACTGTAGTTTACAGATTCTTCTGGAGCATTTTTATTTCTAGATTGC[A/G]TGTGGAAGTCTAACCCCCCTTGGAGATGTCTGCCTTAAAGGGTCTTTGGCCAGGGTC	A	G	0.29	0.36	0.36	0.35	0.27	0.55	0.48	0.23	0.35	0.36	0.40	0.002	0.014	0.008	0.028	0.0002			
rs2739765	15	22.99	CACACATGCTCTCCCACCATGGACTCATGAGATGAGATCATGTAAAACAGTGAC[A/G]GTCATGCACACGAGGACCCTTGGCCCTCCCTGCCACTCAACCCTGGAAGGATA	A	G	0.64	0.96	0.94	0.98	0.93	0.68	0.99	0.82	0.96	0.93	0.95	0.066	0.017	0.017	0.043	0.0000			
rs1557874	15	23.14	GACAAAAGTAGACCAGAAAAATTCAGTCATAAACAATGGGAAACTAGTAACACCACATT[A/C]CAACACAAAAGGAGAATGCAGCAGCAGACAAGACAGACCACCAGTGATGAG	A	C	0.10	0.09	0.14	0.13	0.14	0.00	0.02	0.07	0.07	0.09	0.15	0.006	0.065	0.014	0.065	0.0001			
rs969860	15	23.26	CAGTTTTCCTTTGACTTTGTGCCCCAGGTTCAGTACCTGGGTTCTTGCCATGTT[C/G]TGGTGACTAATTCCCATCTTCTAACCCACTTCAGGAACTGGGGCACAGCTACTCTTG	C	G	0.83	0.91	0.87	0.87	0.86	1.00	0.97	0.93	0.92	0.91	0.87	0.000	0.046	0.014	0.065	0.0000			
rs952121	15	23.42	AGCTGGGGCTGCCTGGCTCTTGGGAACAAACACCCTGTGGCCCTGTGGTGATGGG[A/G]GTCATGAGGTTCTGTATCCCATAGTTTTGACAAAGTTGTGCTTCAGACCGTCCT	A	G	0.33	0.50	0.50	0.37	0.48	0.64	0.62	0.49	0.71	0.53	0.65	0.022	0.001	0.005	0.004	0.0124		1	1
rs872537	15	23.60	TGTGGCTGTGTCCTCGTGACCCGGATTTGCCAGGAATTATCTCACCTCACAAAGAC[A/T]CAGCCCTTGACCATAAGAGCACCTGGAGGAAACTAGGTTCTAGGCAAAACCCCT	A	T	0.52	0.42	0.41	0.50	0.41	0.14	0.08	0.41	0.42	0.52	0.42	0.003	0.077	0.001	0.058	0.0000			
rs2055893	15	23.78	TTCTGTACTGTATATTCTCTCCTGACATTTAGAGGGCATTTCAAAATATAGTCATGAC[A/G]TGGAAGGTGGTGCAACTCAGAGCAGAGCATCGGCCTAGGCAAAGAAACAAGGGT	A	G	0.45	0.54	0.54	0.43	0.48	0.18	0.50	0.17	0.57	0.38	0.38	0.002	0.000	0.083	0.076	0.0116			
rs3922665	15	24.14	CCTCACCTGAGGCATTGAGTTTTCAGTACATAAAAATTATTCAGCAGCTCATTTC[A/G]GCCCCATTTCCTTTAGCTCACTGAACCCATCCCACATCCCTCTTCAGTCACTCTCTTA	A	G	0.38	0.51	0.50	0.48	0.61	0.45	0.98	0.39	0.43	0.58	0.61	0.004	0.184	0.006	0.000	0.0062	1		1
rs7169488	15	24.19	CAGAGGGGAAGGCACCCCTCCAGGACAGAGGGCCTAGGGATGCTGCACAGAGAAGG[A/G]GGGCTTTGAACAAAATACAAATAAAACCTGGTTACTAGGAATTAATTGGCATC	A	G	0.67	0.85	0.87	0.91	0.88	0.95	0.96	0.97	0.96	0.86	0.86	0.058	0.001	0.004	0.002	0.0018			
rs1365591	15	24.31	TAGGGAGATTCTGGGGTTCCTAAGGTCAACAAACGTATTTTTTCATTTACATGT[A/G]CCCAGCAGCATGTCTGTTGTATACGGGCACTCACTGGACAGCTGGAGAAATGAATAAC	A	G	0.05	0.17	0.12	0.10	0.23	0.00	0.00	0.16	0.11	0.15	0.11	0.000	0.022	0.022	0.022	0.0001			
rs2928714	15	24.31	CAGGGAAAGGTGGAACTATAGTCAACACTACAAAGAAAAATATAGATGGTGAACAC[A/T]GCAGCCAGGAAGGGAAGTAACCCAGGGGGGAAAAGCTGTAGCACTAGAGAAAC	A	T	0.45	0.35	0.34	0.28	0.34	0.36	0.00	0.30	0.34	0.42	0.27	0.26	0.005	0.003	0.004	0.000	0.0035
rs1863459	15	24.44	GAGTGCACTGTCTCCTTTCTATGACAAGCAAATTTACAGACCAATGATCTGCAC[A/G]AAGAACAAACTCAAAATGCATCCTCCACCTGAATGTAAGATCTGAAACCATGAAAC	A	G	0.67	0.80	0.80	0.85	0.89	0.23	0.40	0.58	0.93	0.80	0.81	0.005	0.062	0.033	0.159	0.0019			
rs970408	15	24.54	TCTTAGAGACGAGTGCTCAAAGCATACAAATTGCTGTTGATATTTGTGTGCATCT[A/G]CATTCATCTCTGGTGACCAATCTAATCACAGCTTGGAGTCAGTTGCTTGGTAGGAAT	A	G	0.21	0.11	0.07	0.10	0.04	0.23	0.08	0.19	0.07	0.12	0.11	0.010	0.000	0.000	0.015	0.0013			
rs1571281	15	25.37	TTTCCACCTTTGCTGCAGCATCACGGCCTCTGTTTAATTCCTCCCTGCATCT[A/G]CTCAAGGCAGTTCTGCCAGGGCCAGGTGGAGAATGGGTCTCAAAGCATGGGGTGGAC	A	G	0.17	0.49	0.51	0.46	0.46	0.32	0.02	0.53	0.46	0.48	0.39	0.051	0.128	0.015	0.005	0.0056	1		
rs1979372	15	25.48	GACCTGGGATATTTTAAGTGACTAATTCATGTTTGAGGAATCAAAGAAGGAAA[A/G]AAGGTCTGTGGGAAGGAAGCCAAAGAGAAGGAAAACAGAAGGAAGGAAGGCGA	A	G	0.24	0.47	0.40	0.52	0.46	0.09	0.04	0.31	0.36	0.39	0.32	0.006	0.073	0.001	0.040	0.0023			
rs1871017	15	25.49	TAAAATATGACATGGAAACTCCGTACACCTTTGAGACAAATTACACAACGAT[A/C]AGCTTCACACTGAGCACATCCACCCTGTTCCCAGCATGGCCACTCTTCCTCTTGACAC	A	C	0.43	0.47	0.53	0.41	0.46	0.91	0.38	0.61	0.54	0.52	0.53	0.005	0.013	0.006	0.097	0.0003			
rs1077037	15	25.49	ACACTTTATGCCAGAACATTGAAATAAATGTATTAATATGTGTTTACAGGGCTACTGTAAAAATGACCACGAAATGAAAAGTGGGATA[A/C]ATCTTCCAGGATGGATAACTGAGA	A	C	0.26	0.47	0.43	0.50	0.48	0.05	0.08	0.33	0.32	0.46	0.40	0.011	0.071	0.004	0.101	0.0006			
rs1871009	15	25.51	AGTGTGCATGCCTGGAGTTTCCCTAAACAGAT[A/G]GTACCTTACATGGAGACAGTGTGTAGTCTGCCTGAGGTACAGAAATCAGGTGTAGGTCACTCTGCTTGCCCTGCAAT	A	G	0.60	0.47	0.59	0.43	0.54	0.41	0.79	0.58	0.69	0.61	0.73	0.002	0.012	0.003	0.033	0.0103			
rs1498521	15	25.66	GAAAGAGGCTTCTCACACATATTTCACACTCAAAGGCTGGAAATAGCAAATCCA[A/G]TGCATCTTCAGAAAGCACTGATCTAAAGAAACCTCTTAAAACTAGAGCCAAGAGGA	A	G	0.50	0.46	0.41	0.54	0.30	0.68	0.56	0.25	0.32	0.24	0.24	0.002	0.005	0.019	0.031	0.0176		1	1
rs1520942	15	27.37	AGGCTTTGTTACATGTTTCCTGGGGTGCTTCCCCATTGGACCTGTAAGTGTCTAA[A/C]CCTGGGCTGATTCTGTAACCTGCCTGGTCAGCAGTCCTTGTTCTATCTGTCCTGG	A	C	0.17	0.15	0.15	0.15	0.14	0.36	0.08	0.20	0.25	0.11	0.11	0.000	0.012	0.003	0.034	0.0006	1		
rs1719013	15	27.87	GAGTTTGACAGTCATTGTGGGTGCTCCACCCACAGCTCCTGTCAGGAAGTG[A/G]CTGTGAAACCGGGTTCCTCAGAGCCATCGTGGTTCTCTGGAGGTAATCTGGGTTGGG	A	G	0.64	0.36	0.30	0.37	0.23	0.36	0.22	0.29	0.46	0.28	0.25	0.053	0.003	0.000	0.005	0.0012	1		
rs2873	15	28.95	GAACAACAAGATAACACATCTTCTTGCTCATCCCACTTGAACTCAAGTCATCA[A/G]TTTTAGGCACAAAGGTTTAGTTTTCTCGGGAAATCAAGTTTTAACCACTTGAGGTTAC	A	G	0.81	0.51	0.58	0.61	0.52	0.14	0.27	0.48	0.50	0.54	0.54	0.042	0.031	0.005	0.085	0.0001	1		
rs1524876	15	28.98	AAAATAGTGTGTATGTAAAAGTGCAAAGAATGGGGCCCTGGCTTTCTCTTCTC[A/G]GAGTGTTCCACAGTAACAACATGAAGACAATCCAGGTACACAAGTTTGTATGTGCTT	A	G	0.19	0.49	0.42	0.38	0.50	0.86	0.73	0.52	0.45	0.45	0.46	0.040	0.035	0.006	0.090	0.0003			
rs278357	15	29.21	AGCAGATGCCTTGAGCCTTCCTGCATGAGCCCTCGGGCCAGATGGAGTAGGTA[A/G]GCAAGGATCTCAGTCACACAAGGTAGTGTCCACCATGACTTCTGCCTCCTGTCAGC	A	G	0.33	0.49	0.52	0.49	0.59	0.27	0.57	0.45	0.46	0.51	0.51	0.031	0.000	0.015	0.048	0.0004			
rs904951	15	30.13	CGGTCATACATGGTCCCCCTGGATCACTTGTGAAAGACTCTGTCCTGCTGGGC[A/G]TGAATGCCAGCTTGCATTCCTTTAATTAGATGGAAACTAGGAGGTATTTGGTGGCTGC	A	G	0.23	0.47	0.52	0.56	0.46	0.45	0.36	0.30	0.46	0.41	0.48	0.029	0.002	0.018	0.000	0.0000	1		
rs1562933	15	30.88	CCCTATCCACCTAGGAGCCACGCTGAGGTTGAGCTATCTGGGAAGAATCTA[C/G]GAGATAAGGATATTGGCAAAACTCCAGTCCCCTTTCTGCAGGGCTAAGGAGGTCT	C	G	0.48	0.42	0.48	0.40	0.46	0.55	0.77	0.51	0.64	0.54	0.40	0.001	0.062	0.001	0.005	0.0021	1		
rs343913	15	31.06	CACAATACTCCCCATCTCAAGTTTGATAATTTCCTGGAAAAGACTCACAGACCT[A/C]AAAAAAAAATGCTGTCTGTATTGTTATAATGTATTATAAAGAATACAAATGAGCTGCCA	A	C	0.45	0.34	0.35	0.32	0.32	0.82	0.28	0.52	0.29	0.38	0.35	0.004	0.005	0.007	0.112	0.0006			
rs1026752	15	31.11	GCTTCCAGAACACTTGTCTTCTCTAAAACTAGGCACACACTACAACATTATTCT[A/G]AGAGAAACATGACCAGCTCAAGAGAAAGAACTGGGAGACAATGACACAGGAGGTTCC	A	G	0.14	0.31	0.36	0.32	0.34	0.09	0.47	0.30	0.29	0.27	0.27	0.037	0.003	0.004	0.064	0.0039			
rs1878299	15	31.46	GTGAAACTCTTTCCGGGACTACTTCTCAGGCCCTTGCGTCACAAATACCTGACGTTTGTGGCACCACTT[C/G]CCTTTCTAAACCTGTGGAGACGAGGATAATCAGTGACCAC	C	G	0.31	0.47	0.49	0.44	0.48	0.36	0.43	0.41	0.62	0.47	0.44	0.019	0.002	0.003	0.005	0.0027	1		
rs2033610	15	31.47	TACAAATGCAGATACCATAGATCTGATAACATACAAACACAGATACCATTGCACA[A/G]TTTGTCAGGATCTCCCTGCTAGTGCCATGGTGGCCTCAGACTTAGATGCTCTTCTG	A	G	0.29	0.50	0.51	0.45	0.50	0.45	0.56	0.51	0.54	0.51	0.48	0.031	0.002	0.000	0.000	0.0017			
rs1495280	15	31.64	TCAGCTATTGTGAGAAGAAAGGCGAACAGAGGGTGTGGAAGGGAAAGAGAGAAC[A/G]TAGACCTTCAATAATTAGGTAGCACTACTCAAAAGAATTTGATGAGAACATCTAAG	A	G	0.52	0.49	0.49	0.41	0.30	0.55	0.85	0.59	0.50	0.44	0.43	0.001	0.095	0.015	0.007	0.0006			
rs2088142	15	31.70	CCTAAGAATGTTTTTGTAATGTCATTCATTGATCATTGAGAGATTATTGGTTC[A/G]TTGAGTTAATAGAACTTCAAAATCTTGACACATTTCATTATAAAATAGTTTAAAAATCAC	A	G	0.20	0.64	0.55	0.55	0.52	0.33	0.80	0.47	0.60	0.54	0.45	0.054	0.043	0.000	0.019	0.0035			
rs965471	15	31.80	GGGGCTAGGAGAGGTGCATAAACAATAAACAACCCATATAAGTAAATTATATAGAGC[A/G]GGGTAAGGGGAATTAGGAGGGTCAGGGTGGGGTGGAGTTGTGTTTTTAGATA	A	G	0.48	0.70	0.66	0.62	0.68	1.00	0.24	0.85	0.79	0.63	0.67	0.010	0.064	0.043	0.158	0.0006			
rs2059956	15	31.84	AAGACATTTTCTTTCCATGAAAAATTTTTAGTTTAAGAGGGAAAGCAAAATGTA[A/G]CTAGATAAATGCAAATAAAGTGTGAACATTATAAACAGGTCCAGTGGGGGGACCCCAA	A	G	0.50	0.55	0.42	0.54	0.43	0.77	0.59	0.73	0.50	0.43	0.48	0.001	0.024	0.063	0.080	0.0017			
rs580839	15	32.71	CTCCACCTGGGACAGGTTTATAACTCCCCTTGAAGATAACACCAAGAACAG[A/G]GGGTGGACAGTCTTGAAATTGGTGTTGTGCCTGCTTCTCTTGTTCATCTATCACAGGAAG	A	G	0.69	0.46	0.41	0.38	0.48	0.45	0.28	0.68	0.32	0.41	0.41	0.040	0.026	0.039	0.000	0.0004	1		
rs1055356	15	32.86	GCAGAAATTCTGAGTTTTCTGCCTTATTTTTCTTGGGATAACCAGCCACA[A/G]GGAGACCTTCAGGTAAAATGTGGTTCAAATATGGAAGGCAGAGGTACCT	A	G	0.86	0.42	0.43	0.49	0.54	0.77	1.00	0.53	0.64	0.40	0.45	0.133	0.307	0.024	0.094	0.0018			
rs717941	15	32.91	AAGTAGAATGCCCTCCACTCACCCACTTCAAATTCTAAGATGCTG[A/C]AAGTATTTTCTTCTTTCTCTAAATTCTTACTATTTCCTTCCATAGCAGTCATGTAATGTTTACGATGCA	A	C	0.83	0.39	0.38	0.43	0.46	0.59	0.93	0.48	0.50	0.35	0.44	0.148	0.229	0.025	0.043	0.0041			
rs1551650	15	33.01	TAGTCCAGACCCAGAGATCCAGAGACATTGTCATGGAGATGGGGAGAAGTGTACC[A/G]ATTTAAGAAATGTTTTGAGGTTGTATTAGAGTTTTCCAGAGAAATAAACCAATAG	A	G	0.86	0.34	0.31	0.35	0.39	0.27	0.88	0.41	0.36	0.34	0.42	0.195	0.207	0.016	0.000	0.0094		1	1
rs732165	15	33.07	TATTTGTTAAATGCCATACGAAGTTCCTTGCAGTATGTGCATATTCGGCCTCCT[A/G]GCTCTGATAAAGGAGACATATACATGGGTTATTCTAAAGATACTAATTCCACACAT	A	G	0.02	0.42	0.38	0.38	0.36	0.14	0.09	0.24	0.25	0.33	0.39	0.138	0.097	0.041	0.075	0.0000			
rs1989223	15	33.84	AGTTAAGTGAAAAGAAATAGTGTTTACAGCATGTGAATGGCAACTCTAAAAATAGA[A/G]GGGCCATTTAGTACATACTTTATGACAAAAATTTCCTCAGGTGCCCTAGGTCAGCT	A	G	0.90	0.75	0.68	0.68	0.70	0.64	0.97	0.76	0.68	0.78	0.75	0.046	0.089	0.005	0.003	0.0036	1		
rs988311	15	34.25	TATTTCACCATTTTAAATGTATTATCCCTATTCTGTGAGCCAGGGAGCTAAT[C/G]TGAGGATTATGTTGGTGGCTGAGTTCATCTATCATAACTCTGTATTCCAGAAAATGGGAA	C	G	0.50	0.70	0.77	0.78	0.75	0.18	0.19	0.64	0.68	0.84	0.76	0.023	0.133	0.001	0.137	0.0001			
rs957515	15	35.25	AGTACCCAAGTGGCCTGGCAATTTATTAAGTGAGCAATTTATTCTTTTCTTGCT[A/G]CTAAGATCAAATCTTTCACCATTCAAATTCTCCACATTCCTCAGCGATAACAAGGTAGG	A	G	0.57	0.58	0.51	0.54	0.63	0.05	0.32	0.39	0.54	0.49	0.48	0.003	0.019	0.011	0.153	0.0005	1		
rs1003223	15	35.26	TGAGTAGTAATTTAAAATAATATTTTCAAAGAATAATACTTGGGAAAATGTATTCA[A/G]TATGCTGCTAAGTGTGTAAAAGCAGGCTGCAAAATTAAATATACAACATAATTCCAA	A	G	0.79	0.53	0.56	0.55	0.46	1.00	1.00	0.88	0.54	0.60	0.62	0.029	0.183	0.077	0.183	0.0023			
rs1108081	15	35.82	TGATGGTTGGTTGGGCGGAGGTAGTGCTGGTTCGGGAAGGATGTAGAAGC[A/G]TTTTAGGGAGTGATATTGTTCAGTGTGTGCTTCTGGCAGCTCCCATATCCCATCAGTG	A	G	0.14	0.53	0.53	0.51	0.52	0.95	0.65	0.59	0.50	0.55	0.53	0.105	0.002	0.000	0.118	0.0002	1		
rs723049	15	36.77	AACTAAAAAATACCCACAGAAAATGCAGAAGCTAAAAAGAAGAGAACCCACTCTCTGGTGGC[A/G]TAAAACAGAAAGAAGCCAAGTCTGTGTGACATCACATCTTACCAGTCCT	A	G	0.57	0.39	0.44	0.39	0.43	0.41	0.52	0.50	0.43	0.42	0.41	0.013	0.015	0.009	0.000	0.0002	1		
rs768269	15	36.95	TCCTCCTTAGGGCTCAAAGTGGCCAGAAGATTTGCTGGAGTTCACACAGATGCT[A/C]AGTGACAGAACCCGGATTTGAAGCCAGGACTCAATTCTGAACCCATGCTTTATCCAC	A	C	0.57	0.38	0.46	0.49	0.45	0.27	0.38	0.35	0.29	0.42	0.40	0.013	0.001	0.002	0.013	0.0003			
rs1433887	15	36.95	AAAATGGTGTGCAACCAGA[A/G]CTGCTTCAACATAGAAATTCATGGGGTGATGGTCCCAGGGTCACACTGCCTCTCAGAGAACAGGACTCTCATTTAAATAACCCTAAATTCAA	A	G	0.48	0.63	0.55	0.51	0.54	0.73	0.68	0.68	0.71	0.60	0.62	0.007	0.007	0.004	0.013	0.0011			
rs1433895	15	36.96	TAACCTCCATTTCTCAGGAATCAGCAAAGAAATGAACAAAGACTTGCTGTGGGCTTTAGGTAT[A/G]CAACAAAGGGAGAACTTAAGACTGCGTCTGCAACAGACCAAGACTGG	A	G	0.52	0.64	0.63	0.58	0.61	0.32	0.35	0.63	0.54	0.64	0.66	0.003	0.038	0.000	0.048	0.0003			
rs1158417	15	36.97	TTTTGAATGAACAAGAAAGTATAAATAGTAGTTACCTTTGAAGGTCAGCCAAGTA[A/G]AAGACAAGAGGATGTTTACTTACAACTTTACGTCTTTCTTCATAGTCCTCAGTACATA	A	G	0.19	0.54	0.56	0.62	0.55	0.68	0.77	0.61	0.71	0.59	0.53	0.068	0.042	0.004	0.017	0.0004			
rs2004175	15	37.05	CCTTGGAATTCATCATGAACCACAGGAGGAGAAGCTCAATGTAATGAGCAAG[A/C]TTTATAGTGATCTGTTCTCCTCATTTGGCCCCTGTATCTACTTTGCAATTAAGGAAAAT	A	C	0.90	0.42	0.45	0.38	0.41	0.77	0.85	0.70	0.43	0.62	0.44	0.123	0.080	0.026	0.048	0.0000			
rs276855	15	37.25	AGGGATTAAGTCTGTGTAGTTTAGTGCAAAAGGCATGGCTCTCTTACAAGCTCA[A/G]TCACCTTATCTTCTCCAAGACGTTTATTTTTAATCTACTTAATTGAGGGGGATAAGAC	A	G	0.45	0.54	0.59	0.55	0.63	0.86	0.27	0.65	0.62	0.54	0.68	0.003	0.056	0.003	0.058	0.0065		1	1
rs1565863	15	38.15	ATTTCACCGGTTTCAATAAAAAATGCATGATTTTGGTAGACACTTGACAGATTTA[A/G]TGAGTAAGGCCATTTGGTAAGAGAGAGAGTTGGGCTACACCAAACTCAGAGAAGT	A	G	0.28	0.73	0.70	0.71	0.70	0.25	0.60	0.45	0.79	0.68	0.63	0.085	0.003	0.026	0.094	0.0006	1		
rs1869907	15	38.97	AACAACACGGGTGGCAGAGATGCATGGCAGGCCCCAAAGCCAGATCTAGGTGGTCAGG[A/G]TAGGGAAGCTTCAGGGAAGGCTTCTCAGAGAAGGTGA	A	G	0.48	0.60	0.54	0.59	0.48	0.73	0.93	0.46	0.57	0.45	0.52	0.000	0.170	0.002	0.056	0.0000	1		
rs9796	15	38.99	AGCTTGCTGCCCTCCAGTAGAGACAGAGGGGCAGTGTTTGCA[A/T]GGGAGGTGTTTCTGCCTGTCCCTGCCTGATGTCCCTGGTGGAGCAGGTACCTCCGGGCGG	A	T	0.12	0.40	0.45	0.41	0.54	0.27	0.05	0.44	0.43	0.55	0.46	0.116	0.190	0.017	0.061	0.0003			
rs1009913	15	39.05	CATGAGAAGTAGTATTCTGGTAGCAGGTTCAGTAAGGCTGCTAAATTACCAGA[A/G]TCATTATATCAAACCAGTTGGGATCTTTGTTATTTGTATTTTATGAAGATTAACCTCAG	A	G	0.29	0.60	0.54	0.59	0.50	0.73	0.94	0.44	0.54	0.45	0.52	0.015	0.176	0.003	0.060	0.0001			
rs1197668	15	39.86	CAGACTCATACAAATGTGAGGCGCTGAGACCATCTGTTATTACTGCTGAGAAGGGCCATGTCATTCTAGGCTCTTGGCTGTACATG[A/G]CAAGGGCTGGTACAGAGCTCGCTC	A	G	0.45	0.43	0.47	0.39	0.34	0.75	0.32	0.40	0.46	0.38	0.42	0.000	0.006	0.000	0.048	0.0038	1		
rs614215	15	40.09	CACCTCGTCATGCTGCGGGGTGAGGGCGTAGGGAGGTAATGCTAGCTTTAGGTA[A/G]AAATAGTATCTCGACCTCAATGTCTACACAGTCTTAAATGGACAATAAGGGAATCT	A	G	0.67	0.61	0.66	0.71	0.63	0.75	0.87	0.49	0.71	0.63	0.70	0.000	0.036	0.005	0.005	0.0019			
rs1995939	15	40.73	GTCAGATGGAAGACACTATAGAACCAGGTTGGACAGATTTGGGGTCAGGGTTGG[A/G]GGCTGAGTTCTTTTGAGGACTGGTTGGAGTGGCAGCCAGATGGTGGCTGCTGAC	A	G	0.14	0.77	0.79	0.74	0.70	0.68	0.99	0.52	0.89	0.76	0.76	0.208	0.064	0.021	0.006	0.0001	1		
rs573615	15	41.33	TAGATGAATAGCTCACAAGGTATCCCTTCCTTCTCCAGAGTCATCATGCCCT[A/G]TGGAGATTTTGGTTTTTCTTTTTCAATCAGTACCTGAATTAAGATGCACAGACTAATCC	A	G	0.83	0.27	0.30	0.27	0.32	0.55	0.26	0.46	0.29	0.37	0.39	0.159	0.004	0.015	0.031	0.0028	1		
rs488756	15	41.33	CAGACCATATACTAAATAATCCAGCCTTTCCTCATTGATTTGAAATGTCACCTTATC[A/C]CACAGTCTGTTTTTAATTCTTTATTCCTTTGATTTATTTATTGTGACATCAATACCAA	A	C	0.38	0.85	0.83	0.87	0.84	0.45	0.74	0.71	0.93	0.79	0.80	0.119	0.003	0.008	0.083	0.0002			
rs1648282	15	43.14	TCTGCTCCCTGCTCCACTGCTTTTGGCTCCCTGCCATGTCTCCCCCACTGA[A/G]CAGCTTAAAGATCTGGCTTGAGTTGGACACTACTTTGCCCTGGTTTTTCCCTCTGCAC	A	G	0.17	0.59	0.57	0.55	0.45	0.32	0.63	0.36	0.50	0.54	0.51	0.072	0.012	0.014	0.020	0.0018	1		
rs1648312	15	43.17	ATGGTGGGGGGGCAGTGTCTAGGGACTAGAGTGAGAAGTAGGGGAGCTACTG[A/G]TTTGGGGCAAAGTGAAACCTCTGCTTCCAGACTTCAGAAACAAATCTCAGAAGACA	A	G	0.26	0.56	0.58	0.54	0.43	0.05	0.00	0.28	0.46	0.47	0.52	0.033	0.223	0.020	0.153	0.0017			
rs1648308	15	43.19	GAAATCTTTCAGCATTTCAGCAGCATT[C/G]TGGCTATATCAGAGAATGTGCCTAAATACCAGCCGGTGGTTTATTATTTATTTATTTAAGTTTTGAAAGTCTTTAATT	C	G	0.83	0.45	0.43	0.48	0.59	0.95	1.00	0.74	0.54	0.53	0.48	0.065	0.216	0.025	0.147	0.0011			
rs1426932	15	43.40	TGCTACACCCCATCCAAAAAATGGGATGAAGTCTTGATTTCTCTTTCTAAG[A/G]CACTAAAATCAGACAGAGGAGACAGAAATATCTGTTTGCTGCACCATTATTTCTCACTGA	A	G	0.21	0.61	0.60	0.68	0.66	0.05	0.00	0.38	0.57	0.61	0.58	0.072	0.259	0.019	0.186	0.0002			
rs1030588	15	44.44	GAATTATCACCAATACGAATACTGGACTTTTAATTGTCTCTCTTCATCTCAATATC[A/G]TAAGCTCTAATAAGACAAAAAAATTGCCTTGTCATAATTAAGCCCAAAGATCACAA	A	G	0.05	0.53	0.56	0.59	0.52	0.27	0.30	0.24	0.64	0.55	0.58	0.186	0.046	0.071	0.056	0.0000	1		
rs1367814	15	44.51	CAAATGGGTATCCTTGTTGCATTTCCAGTTTGGGGCTTATCAGCTTTCACTTA[A/T]TCAGTTTGCCAATCACAAAACTCATGATGTTAAAATGGTGTTTGGAGGAAACTCCCCTC	A	T	0.60	0.65	0.64	0.55	0.68	0.82	0.97	0.72	0.57	0.63	0.55	0.001	0.098	0.003	0.024	0.0024			
rs623549	15	45.35	AAATGAGCTCACTATTGGGGAAACTGACCTTCCCAGAGGCACAAGGAATATCAC[A/G]AGTCTGAAAGCAGTGCCCTCTGAAGCCCTATAGCTATAGAGTTACTCTGAGTAAT	A	G	0.17	0.60	0.63	0.65	0.61	0.50	0.49	0.70	0.68	0.54	0.65	0.108	0.008	0.008	0.005	0.0003	1		
rs281265	15	45.39	AGGAGGATGTGAGACGATAGTGCCTCCCATGACGAGGGAAGATGGGCACAAG[A/C]ACTATGATTGGTCTCTAACTCCTATGCCCTGGCTCATAGTTGATTTTGAAACCGGCCA	A	C	0.21	0.66	0.67	0.68	0.63	0.55	0.52	0.71	0.68	0.56	0.67	0.100	0.008	0.005	0.004	0.0000			
rs3198	15	45.40	AGGCAGGATAGATATTTGGACCCCACTCTCCCTGGTTCTTATACCTACTCTCTTAATCAGTTTGCT[A/G]TCTTCATCCTCCTGGTTGCTAAAGCCAAGCTGTAGCCCTTGTCATG	A	G	0.76	0.53	0.46	0.33	0.46	0.50	0.53	0.66	0.61	0.46	0.45	0.046	0.003	0.022	0.000	0.0000			
rs890153	15	45.54	ATTCTCATCTTAGTTACAGCTTAAGTAAAGACAAAACTGAAAATGCATAAGAATTT[A/G]GTTATTCCTCTGAACACTGATATGGTGAATAAGTGTGGAGGAGCTGAAGCATTGTC	A	G	0.29	0.58	0.60	0.54	0.55	0.41	0.59	0.67	0.62	0.58	0.62	0.042	0.001	0.026	0.010	0.0002			
rs514327	15	45.74	AGCAGGAATAATTGTGGAAAAAGGCAACAAAATAAATCAAATGGCAAGAACTGGGTA[A/C]AGTAATAATTACCAAGTTTTTTTATGCATTGGAAGTTACTTATCTGCCTTCCCATG	A	C	0.88	0.57	0.60	0.50	0.59	0.73	0.74	0.73	0.64	0.62	0.64	0.041	0.006	0.015	0.006	0.0010			
rs537848	15	45.81	CTTGGGCAAGTCACTTAACCTCTCTGAGCTCCAGCCTTCTCATCTGTAAAATG[A/G]GGATAATAGTTATGCCTCCCTCCTTGAGTTGCTGTGAGACTGAAATGGAGTTAAACAG	A	G	0.50	0.64	0.59	0.76	0.52	0.23	0.24	0.53	0.50	0.54	0.51	0.002	0.056	0.002	0.065	0.0037			
rs1062124	15	46.83	GCTAGGCCAGAGGATTTGGGCCCCAATCCTTTTAACCAGCAAATGGGACAGTCCA[A/G]TGAAACAGACAGCCATGTTAGTGGCTTTTGATTTGATTCCACTGACATACACACAC	A	G	0.60	0.63	0.54	0.56	0.50	0.64	0.83	0.66	0.61	0.54	0.52	0.003	0.039	0.007	0.004	0.0001	1		
rs877007	15	47.04	GAAAATGATTCCTTAGTTTCTACTATTGTCTAACCCTCAAGACTCTTGATTAA[C/G]AGCCTCAGAATATTAACTTGAACATTTAATATTTGAGTAGTTGAATGGCCTTATATACCA	C	G	0.60	0.42	0.37	0.29	0.30	0.75	0.81	0.44	0.35	0.43	0.39	0.028	0.103	0.003	0.073	0.0000			
rs1048975	15	47.13	AGTTACTGCAAAGAAATCTTTGGCTGCCACAAGTAGATGCTCTCTACTAC[A/C]AGAGCTGGATTTCCATTACTCACTCTTGCTCTTACATTAAAGTTGTTGAT	A	C	0.60	0.40	0.51	0.48	0.43	0.36	0.17	0.47	0.46	0.43	0.45	0.012	0.032	0.004	0.001	0.0000			
rs489813	15	47.15	CATAAGGTAACTTTATACATTTGGTTTGAGTAATCTAGTCATTTCAAATTTAAG[A/G]TAATTACAATGTTCCTGTCATACATAGTTCCTCTTTTACAAACTATATCAGGAACTATAT	A	G	0.57	0.64	0.53	0.52	0.57	0.77	0.86	0.63	0.54	0.61	0.58	0.002	0.020	0.000	0.006	0.0001			
rs1864299	15	47.32	TGCATGAGAGATTGCCATGGCTTGCAATCTCTTGAACGGTAGTAACTCAGACA[A/G]CAAGGGAAAGGGCAGAAAGGGCATCTGTTTTCAATTGTGATATAAAAATCTTGCCAAA	A	G	0.57	0.64	0.53	0.52	0.57	0.77	0.86	0.63	0.57	0.61	0.58	0.002	0.022	0.000	0.006	0.0001			
rs1981995	15	47.33	CTACAATTAGTAATGGAAATGTAATTAACCACTTGGCACTTGTGGCTTGCCTCAGTCATTGCCACACTCCCAGCCTGAAAAGTTTAAACCAAGT[A/G]ACTCCAGTGATCACAAAT	A	G	0.57	0.64	0.53	0.52	0.57	0.77	0.86	0.63	0.54	0.61	0.58	0.002	0.022	0.000	0.006	0.0001			
rs981612	15	47.38	ACAAACCTTTTAAGAGTTAAAGAAAGTTACTTGGGTTGATTTTTGACATTAGAC[A/G]TTAGGAAATATTAGGCATTAGCTTGAAATACTAAAGGCAGGTAAGCATTTATGTCACC	A	G	0.55	0.64	0.53	0.51	0.57	0.64	0.86	0.63	0.63	0.61	0.59	0.003	0.022	0.000	0.001	0.0000			
rs479062	15	47.81	GTGACAGGCAAAGGAAACGACAGTGCAGAATCAAACTGTGTTGAAGACCAGTGG[A/C]AACTGCATAATTTAACTTGGATTTAGGCGGAAATGAAAGATGGGAGGAGTAAAGAA	A	C	0.38	0.48	0.48	0.40	0.39	0.59	0.68	0.59	0.46	0.45	0.48	0.007	0.020	0.006	0.003	0.0001	1		
rs2630	15	48.57	ACTTCCTCTTGAAGAGATGGGTCGTAATGTTGTAGTCTTACATGCTTACGTAGAC[A/G]ATCAAAGCAAGAATACTCAATAAATGGCTATTTACCACTTGAAAGAAAGATCAAGTAA	A	G	0.81	0.59	0.60	0.67	0.57	0.55	0.54	0.65	0.54	0.56	0.50	0.018	0.007	0.001	0.006	0.0063	1		1
rs1147129	15	48.92	TCTGAAAGCGACTGTTTCTGCTCCTACTACAACACTGGTAGGTTTGCATAGTCA[A/G]TGCCAACACATTTGAATTTGAAATCTAGTCACTGTGGACTCAATAGTGGCATCTAACT	A	G	0.76	0.53	0.54	0.50	0.63	0.32	0.72	0.64	0.50	0.63	0.50	0.040	0.037	0.019	0.013	0.0000			
rs2014638	15	49.84	GATTTAAGTTAGTTATGTGCAGCCTCCCTTGGTTTGTAACATTTTCATGCAGCTC[A/G]TGTCTCCTCTCCTTTTCTGAGAAACCTTCAGATTAATTTTTTGGCAGATTAAATTGAT	A	G	0.52	0.54	0.55	0.56	0.46	0.59	0.19	0.55	0.54	0.64	0.56	0.001	0.067	0.001	0.001	0.0000	1		
rs2017176	15	50.72	AAAAAGGAGAGGTAAAAAAGACAGTGGCTGGTTTAAATGACTAATCACACTGG[A/G]GAGTCCATCAACATCTCAAGCCAATCAAATTACTAAGGAGCCCAGAGTCACGATTCTA	A	G	0.48	0.56	0.46	0.56	0.45	0.64	0.75	0.44	0.68	0.44	0.44	0.000	0.022	0.010	0.005	0.0000	1		
rs690054	15	51.72	AGGTTTTCCCAGGGTCTCAGCCACAGAGTAAGACTGCTGTGCTGAAGTG[A/G]GAATAGCTGGGATAGAAACAGCCTAAGAATTGCAAATAAAAAATGTGTGTGCATGAACTAAGA	A	G	0.29	0.45	0.46	0.54	0.50	0.36	0.13	0.48	0.25	0.54	0.53	0.022	0.073	0.004	0.010	0.0018	1		
rs1858359	15	52.37	TTACAGCTTATAGGTTTTATGTTATGTTGTAAACTACAGTCCCCACTTTGAGATTATTTTTAAAAATCTCATCCGATCTTTCC[A/G]TCTAGAATTTTTAGAGTATTTTTAGGTTAAAT	A	G	0.50	0.45	0.40	0.39	0.46	0.86	0.31	0.49	0.36	0.46	0.34	0.001	0.009	0.002	0.105	0.0014	1		
rs472579	15	53.38	TTAATGTGATGTAAGTATGTAGGTATAAAGTTCCTGGATATATCAGTTATTAA[C/G]CCACAATTATAACTAAGCGTGAGTTATGGCAGAGACAACTTGAAACACTGCTAGTGATT	C	G	0.64	0.48	0.52	0.46	0.52	0.00	0.02	0.51	0.54	0.50	0.53	0.004	0.216	0.002	0.244	0.0008	1		
rs1384126	15	54.56	GGATATTTTTGAGCACTCACCAGTTGATAGGCAATGTGCCAAGTTGATACATTCA[A/T]TCGTCTGAACAATATTCTGAGAGAAGCACTTTAGTTATATCCATTTTATAGATGACAA	A	T	0.55	0.62	0.59	0.61	0.54	0.55	0.81	0.55	0.69	0.56	0.48	0.000	0.039	0.000	0.000	0.0023	1		
rs1037958	15	55.24	TGCAGTCATCTGTATATCAGCCAGTATTTTAAAGTATTAGGAAAAGAATACCT[A/G]TATTCAGGCCTTATTTAGGCATTTTTTTTGGCTAAATTAAAATTTAGCTTTATAATTTTC	A	G	0.90	0.46	0.49	0.40	0.48	0.64	0.84	0.61	0.31	0.44	0.49	0.081	0.077	0.006	0.006	0.0000	1		
rs11856	15	55.56	GGAGTGGGAAGAGTTGAGACTGAAGTTAAGAGAAGGGTTTTCAAGTCCC[A/G]GCTCATCTCACCCCAGTGACTTTGACAGTGTGCTCACTCTCAGAGCCATG	A	G	0.14	0.18	0.23	0.24	0.16	0.09	0.54	0.18	0.18	0.15	0.20	0.012	0.039	0.010	0.033	0.0025			
rs723967	15	56.41	CAGAACCTAGCTTGGTGTCTGGCACATAGTAGGTGTGCTCATTACATATTTGTTGGATTACTGTGCCGTGCCT[A/G]TTTGTCTATACCAGCTTTGTTTGCACTGCACCATGAGCT	A	G	0.83	0.54	0.46	0.50	0.50	0.77	0.57	0.72	0.50	0.51	0.50	0.091	0.015	0.054	0.069	0.0006	1		
rs347117	15	56.72	TTATAATCACTTTGCACTAGTAAGTTTCCAAAATAGTCTATATTGAACCAACAA[A/G]TCAAATATAAACACAAGGTCTAGCAAATCAAGAATCTTAGTTATCATGGTGTGGACAG	A	G	0.74	0.29	0.35	0.27	0.27	0.77	0.75	0.51	0.43	0.28	0.25	0.099	0.117	0.023	0.137	0.0037			
rs383902	15	56.75	AGGATTTGTTAAGCTTGAGGGACATCAGCAAGCGGGGAAGGCTTTCTGAGGT[A/G]GAAGAGAATGACACATAAGATGCTTGTAGGTTTACTTGCCTGGAATCGATAGTCTGTG	A	G	0.74	0.31	0.35	0.28	0.29	0.77	0.75	0.52	0.46	0.29	0.26	0.099	0.117	0.023	0.137	0.0032			
rs395601	15	56.78	CTCAGCTCCAGTAGGAAACATCCTCTCCACTATGTGGCCGGAAGTTGAACTCC[C/G]GGGCAGCTCCCTTGTGTGCGGCTTCTTTAAAGGGAAGGCAGGAGGCGGACAGCTTG	C	G	0.26	0.64	0.60	0.67	0.68	0.18	0.02	0.41	0.54	0.63	0.61	0.082	0.282	0.033	0.137	0.0002			
rs387812	15	56.79	CAATGAAATCTTTATACAAAACATAATTGACAGAACTTTGTGACCAGTGAGGAAAGAATAATAAAGTTGACTCTAAGGCTCAGGAACTCTGAAA	A	G	0.29	0.62	0.60	0.67	0.68	0.18	0.02	0.41	0.58	0.63	0.63	0.072	0.281	0.033	0.137	0.0000			
rs347115	15	56.80	TGCTTCAGTTTTCCTATTTCCATCCTTCTACTTGTTCAAACCAAAAATGAGATA[A/G]TCTTGACTCCACTTTCACTCTATATCCAACTGATCAGCAAATTTCTGTCAACTCTGTATC	A	G	0.69	0.31	0.34	0.27	0.29	0.77	0.98	0.51	0.43	0.29	0.24	0.078	0.318	0.023	0.137	0.0039			
rs610877	15	56.82	AGGGGTGGCCCAAGGGGCCCACAATAAATAACACAGTCACTCCTATTGGTACAG[A/C]AATGCCAAGATTTAGAAGTTATTTCATAGGAGCTGGGACAAGGTCAAACCTCTCTT	A	C	0.71	0.30	0.35	0.27	0.30	0.77	0.98	0.51	0.43	0.29	0.24	0.084	0.309	0.020	0.130	0.0055			1
[illegible]	[illegible]	[illegible]	[illegible]	[illegible]	[illegible]	[illegible]	[illegible]	[illegible]	[illegible]	[illegible]	[illegible]	[illegible]	[illegible]	[illegible]	[illegible]	[illegible]	[illegible]	[illegible]	[illegible]	[illegible]	[illegible]	[illegible]	[illegible]	[illegible]
rs999868	15	91.55	TAGAAAGAGATGGGACAGGCTATTCTCAGGAAGACACTTAGCTGGCTTCTTC[C/G]GCTGCATCAACTCAAATCTGCCACTTTGGAAAATCAAGTTGAATAGGAATTGGGAGGG	C	G	0.12	0.41	0.46	0.51	0.39	0.00	0.18	0.23	0.54	0.35	0.35	0.102	0.055	0.037	0.223	0.0125	1	1	1
rs2066390	15	92.10	TTCCAGGGTCCAATTAGCCCTGTGAATTGTGGATGACAAAGAGAGCGGGACA[C/G]TGCCAAGTGACTATGAAAAATCTTGGCCTTCATATGTTTTGGCTGATGTGAGCGGTTT	C	G	0.40	0.18	0.20	0.24	0.14	0.18	0.19	0.14	0.07	0.21	0.18	0.028	0.001	0.000	0.002	0.0004	1		
rs918921	15	92.87	ATTTTAGCTATTTGGCCTAAATTGTGAGGTTTGGCCTGAATATCATGTTGGGTCCTCAGCAAC[A/G]CAGTCAGAGTAAATAGGAGGACTTCAGGATTGGTGGGGTAAGGGGAT	A	G	0.52	0.45	0.43	0.51	0.27	0.55	0.57	0.67	0.36	0.51	0.44	0.005	0.005	0.026	0.002	0.0000	1		
rs1559571	15	93.70	GGATCTGCGGGAGCCCCAAAGGCAAAGAGAATCTCTCAGCGAAGCCACTTA[A/C]AGGGGAATTTAGATGATTACGCAGAGGCTGGGAGTAGAGGGAGGAGATTTCACAG	A	C	0.36	0.56	0.45	0.50	0.57	0.27	0.50	0.45	0.54	0.48	0.48	0.008	0.000	0.003	0.022	0.0007	1		
rs2639197	15	94.04	GGCGCTAAACAGTAGATGAGCCTCTCTATTGAGAAGGTGTGGTCCAGGGAAAAACC[A/G]TGCCATTTACTCAGATTTAAGAGCATGGAGGTGCAATCTTATCAGCATTATTACG	A	G	0.65	0.74	0.72	0.71	0.86	0.86	0.97	0.82	0.64	0.75	0.67	0.001	0.088	0.018	0.032	0.0008			
rs8036556	15	94.20	TTTCCAGCTCCTGTCCTAGGGAGCTTGAGGACAAGTGCTTCTGAGTGCCCAGAAG[A/T]GACAGAATAAAGCCCATTTAGAAAAGCTCTGGGAGCTTGATGAGACTTGCAGGTA	A	T	0.36	0.42	0.38	0.43	0.29	0.14	0.29	0.33	0.19	0.38	0.39	0.002	0.014	0.009	0.062	0.0000			
rs1007328	15	94.43	CTATTCCAGTGCATGAAAAAAAAAATCATGTGGAACTAGTGGGAGAAACTTA[A/G]CATACAATGTGCCTGAGTGATTGGTTTCCCTCTGCTTTTTTTTTTTTTTTAATGATTTCT	A	G	0.64	0.51	0.57	0.46	0.52	0.36	0.73	0.68	0.46	0.57	0.59	0.001	0.004	0.006	0.031	0.0000	1		
rs1558389	15	95.32	AAACTCTAAGGCTGTGAGACAAAAGACCTAGGTTTAATATCTACATTCACTGCA[A/G]ATTCAACACAGAGCAGTAAGAGTTAACAGATATCTATTATATCTAAGGTCTCCTATCA	A	G	0.48	0.43	0.47	0.50	0.39	0.27	0.49	0.51	0.50	0.40	0.41	0.000	0.000	0.001	0.001	0.0009	1		
rs288394	15	95.86	CATAAGATGGGAGCTTAGACTGGGGCTCCCAAAGAGGGATCCTAGTCTGCCTT[A/G]AGGTCAATGTTAACTCATTGGCTGTGATCACACTCCATGCAATTCCCTCTTCCTCATA	A	G	0.29	0.66	0.65	0.68	0.52	0.64	0.46	0.55	0.68	0.59	0.55	0.084	0.030	0.012	0.003	0.0096	1	1	1
rs289470	15	95.94	TACGTGCTTCTCTTCCTGCAAATGTTCAATGAGTCCTTCTAGAAACCATTTGT[C/G]TGCAGTTCCCAAATAACATTGTCAGATAATTTTCTTCCCAGGCCTTCAGCCAGCTGGA	C	G	0.36	0.48	0.60	0.61	0.64	0.77	0.90	0.63	0.57	0.74	0.69	0.027	0.059	0.004	0.023	0.0082			
rs906310	15	96.03	TTGTGCTGCAGAAGTGGAATCAAGCGCAGTGATTTCTAACACATTAGGAATCCA[A/G]ACACCTCCGAATGAGTTGCTATTCCAGCATAAGCATTGCCTGTGAAATCTGTCTCCA	A	G	0.38	0.48	0.43	0.45	0.50	0.68	0.40	0.59	0.54	0.42	0.35	0.000	0.000	0.005	0.037	0.0023			
rs1021393	15	96.14	TATCAGAACTGCAATAATAAACATTGTACTAGAAGGAACTGGAAAAATGTACATTCTAT[A/G]TGTCTCCATGTGCATATTGTAACTTTCCAGTTATATTTCAAAACAACAAATTT	A	G	0.98	0.48	0.48	0.57	0.50	0.82	0.86	0.65	0.38	0.46	0.58	0.138	0.068	0.002	0.032	0.0022			
rs1372840	15	96.54	CATGGATAGCAATGTAACATTGTAATCTGATATTCAAGCCTCTGGTCAGTGCACTCATCTCACTGAACAGAGTATGAGCCTGTTTAGGCCA[A/C]CCCAACTCATGTCTAACTCCT	A	C	0.26	0.39	0.37	0.49	0.32	0.32	0.50	0.38	0.36	0.33	0.35	0.004	0.010	0.000	0.001	0.0001			
rs2045112	15	98.04	ACAGACTAAATAACAAATCCACCAATGCTCCAGTGTATTCTGTGTCCTCACCT[A/G]GAAGGTAAGAATTTGTTCTTCAGTTGTATTGCTGCATTTTGATCCCACCTTCCAATTCC	A	G	0.24	0.25	0.28	0.29	0.21	0.36	0.75	0.34	0.25	0.32	0.28	0.001	0.105	0.001	0.005	0.0000	1		
rs729478	15	98.42	AGGGCAGCATGCCCCAAATCCTTGGTTCTCAAAGGGTTCACAGGGAAGCATCT[C/G]GTCTTCTGCATTTTCTTCCCCAAAAGAGCGTGAGTGTGTTGGATAGAGTTCAAATAAT	C	G	0.52	0.54	0.55	0.49	0.55	0.14	0.27	0.51	0.57	0.63	0.52	0.000	0.032	0.000	0.111	0.0004			
rs8024825	15	98.85	ACCAATCTAGTTATACTACCTGAGAACTGCCCAGCCAGCCAGGTTTCCTAGCGAT[A/G]CAAATGCAATCTCCTTTTCATGTAAGTCAGGGTTAAAGAAAGGAACTGCAACAGG	A	G	0.62	0.48	0.53	0.60	0.45	0.77	1.00	0.55	0.68	0.59	0.56	0.005	0.196	0.003	0.031	0.0000			
rs7168512	15	98.92	AGGGTCAGTTTCTGTGAATGCCCTTGGTCAAACACATGGAACGGGAGAACACCCTC[A/C]TTAAAGAAGTTTGAGTAAGAGAATACGCGGAGAAGTACACCGCACCCCAACCAG	A	C	0.79	0.49	0.61	0.71	0.55	0.05	0.07	0.27	0.46	0.44	0.59	0.023	0.163	0.033	0.193	0.0001			
rs4965706	15	99.01	AGAAAAGCACTATGGATACTAGGCTTGCTTCTCATCTAAGGATACCCCTGTTG[C/G]GAGGGAGCCACCAACACAGCATGCTGGGGCTGAGCCGAGACCCCTTCCTCGCAGCT	C	G	0.38	0.19	0.19	0.18	0.23	0.14	0.24	0.13	0.19	0.18	0.16	0.043	0.015	0.000	0.000	0.0000			
rs11433	15	99.45	CAAAACCAAATACATTTTTCCCCAACGTTTTGATTAGGTTGTATGTGTGT[A/G]TGTTTCAGTCTGTACATCAGATTTATTGTAATAATTCATCTTTGTCAT	A	G	0.14	0.36	0.43	0.41	0.38	0.05	0.17	0.27	0.36	0.37	0.40	0.043	0.032	0.002	0.101	0.0000	1		
rs2949	15	99.62	TGGATCTCTCAGCTCCCGCCCCCAGAGGCTCAGCACTTTGGACATGGCTCACAAGCAGTGTTGATTGACTGCATGAATGC[A/G]TGTGCGTGCAAGCATGAACCTTGTTTAAA	A	G	0.31	0.48	0.51	0.56	0.45	0.59	0.77	0.55	0.39	0.50	0.54	0.015	0.054	0.000	0.004	0.0016			
rs4965892	15	99.78	CTCGCCCTATACACGCGCACACTTAATGACATCTCTGATCATGTCTTCAGGACGC[A/G]TACCCTCTTTGACTTGAAAAGTAACAACTGTTTGTGGCCTCTCACAGGCCGAAA	A	G	0.63	0.46	0.52	0.49	0.63	0.59	0.58	0.63	0.50	0.52	0.52	0.005	0.000	0.001	0.001	0.0000			
rs1446119	15	99.85	CTGCGGTGAGTGTGGCAGATGTTGGACCACCGCCCCTCCACTCCAGCATCCTTG[C/G]GGATATCCTCTGGGGGGCTGGAAAGTTCAAGCCCACGTTTCTCAGAACCACTTGC	C	G	0.29	0.40	0.37	0.41	0.36	0.18	0.73	0.30	0.46	0.42	0.45	0.006	0.068	0.000	0.018	0.0021			
rs7168948	15	99.89	ATAATAACATTGGCCCCACGACGTAGACCTGTGGCGGCTCAGGGTTAAGAGACGG[A/G]AGCAAACCCCTGCTTCCGTGGATCTCGCTCCAGCCCTCTCTCCTTGCTTCTGCA	A	G	0.36	0.41	0.42	0.49	0.39	0.32	0.68	0.40	0.71	0.48	0.46	0.009	0.012	0.001	0.015	0.0000			
rs1192113	15	99.95	GACAAAAGTGGAGCCTCATGCCTCCACACCCCTATAAATTATAAGTGACCCTCCC[A/G]GACACGTGCAGAGGTGAAGGAAAGGCAAAAAGGAAGTAGTAAGGCCTGCCCTCT	A	G	0.19	0.30	0.20	0.17	0.30	0.73	0.74	0.33	0.46	0.32	0.31	0.001	0.111	0.006	0.119	0.0042			1
rs8466	16	0.04	CTCAAAAGGTTTATCTTTCCACCACACTAGCAAAGACCCTCAGGACACAC[A/G]ACTCATCTGCCAGCTAAGCATCTACCCCGAGGGACAAGGCAAGCACACA	A	G	0.60	0.85	0.87	0.80	0.79	1.00	1.00	0.93	0.86	0.84	0.77	0.050	0.055	0.005	0.055	0.0057	1		1
rs1211375	16	0.18	GACATTTTCTCCTTTACTCCCACAAATTTAGGTTTAAAATATTTCCATCAGTT[A/C]ATCTCCAATGGCTAGGTCCTTCCAATGAGGTCTCCTGAAAACATGTGGCCTGGATACTC	A	C	0.24	0.36	0.37	0.37	0.29	0.77	0.11	0.32	0.46	0.39	0.40	0.010	0.055	0.003	0.083	0.0012			
rs1203957	16	0.18	TTTTCTGTGTTTTTGGTGTGCCCCACTCGCTGTCGCACATTCTGTGCTGGTGT[A/C]TGTTGTAAAGACTGGAGAATCATTTGTAGGACTTGAGTGCAGCACAGTCAGTGTGCA	A	C	0.38	0.08	0.12	0.12	0.07	0.14	0.18	0.24	0.11	0.10	0.17	0.052	0.006	0.024	0.001	0.0041			
rs1203974	16	0.22	CAATTCAACTGGATGATTTGCAGACTATCATTTCTGTGTTCTCCCCATCAAAT[A/G]CAATGAAACTGATCTAAAGATATTTACAACTGGAGTGGAATACACATTTTTTGAGG	A	G	0.62	0.52	0.56	0.61	0.48	0.82	0.29	0.65	0.57	0.56	0.61	0.003	0.036	0.005	0.043	0.0037			
rs1204001	16	0.25	ATTTTAGGGCATAAAGTTTATCACTTGAGTCCTTGAATGAAAGGAAGTGTAAA[A/C]CCTGACGAGTATGGAGTGTGGACTGTCTAGATGCCCCCTGGTCTAGGGAGGGATTAT	A	C	0.10	0.16	0.19	0.18	0.16	0.14	0.18	0.31	0.21	0.15	0.17	0.005	0.002	0.040	0.000	0.0005			
rs926694	16	0.47	AGCACCTTCCTGGGTTGTCTTTCTGGTTCCATGAGGTGGCCACCTTGTGAG[A/G]CGAGAATTTATAACCCCCGTTTGCGGCAGGCCTCCAGGTGGTTGAGTCACACGTCTGA	A	G	0.81	0.46	0.49	0.40	0.55	0.73	0.89	0.58	0.61	0.40	0.49	0.051	0.074	0.001	0.017	0.0002			
rs2038227	16	0.48	TGGTGAGAGAACTTGCTTTCCTTGACGTCATGTGATTAAGTAAACCATATTCAAA[A/C]ATTTTAAATTTATACCTGGATTTATGAAAGTTGGGAGAAGTCCCTCTTCTCCAGCCA	A	C	0.71	0.64	0.60	0.63	0.52	0.60	0.23	0.46	0.57	0.66	0.55	0.009	0.053	0.003	0.002	0.0015			
rs763053	16	0.68	ATGGCCCCAGGGTTGGGCAGTGAGACGCGGCTGGACCGCAGCAAAGGAGATGC[A/G]CGGAGCGACACAGTTCTGCTCGACTCCTCGGCCACACTCATCACCAATGAGGGT	A	G	0.38	0.65	0.77	0.83	0.77	0.18	0.28	0.61	0.64	0.76	0.81	0.082	0.145	0.028	0.197	0.0005	1		
rs2072042	16	0.98	AAGGCACAAGTTTCCGTGAAGTAGGCGCTATTGTATGCTCTATGCTCAGCACAC[A/G]GGGGAAGCAGTGCAGGTGAATCAGGTATGACTCGTCTAGAACTGAGGCCCTAACG	A	G	0.31	0.50	0.55	0.56	0.50	0.73	0.50	0.33	0.50	0.60	0.56	0.028	0.001	0.030	0.019	0.0000			
rs2745165	16	1.20	CTGCCCAGGACGGTCCCTTGGGCCACTGGGGTCACCAAGCACCTGCTTCCCAC[A/G]GCCTGTCATAAAGAGCTATCGTCGTACTTCCTCCTGTGCTCTGGGTTTGCTCTG	A	G	0.00	0.12	0.19	0.13	0.13	0.05	0.00	0.16	0.11	0.09	0.11	0.063	0.060	0.001	0.019	0.0051	1		
rs8767	16	1.44	CCCGGGCCTCAGAGTCGGCGCCGGAAACTAGGTCTGGGTTTCCCTCTGTGGTGGTT[C/G]TGTACTGAGGACCTGGAAGTGATCATATTTTGGATATATTCGGTTAAATAAAAT	C	G	0.69	0.52	0.62	0.60	0.57	0.68	0.67	0.56	0.64	0.61	0.51	0.001	0.000	0.001	0.001	0.0073		1	1
rs3178656	16	1.65	AAACAAAACAACAGTCAGGCTGGAAGTGGTCCCTGGGCCATAGTTTTCAACCCTG[A/G]TTCTAGTTTGAACAAAGAACACCTAAGATTTAGCAGGCACTTTCTTCTAAGACCC	A	G	0.69	0.96	0.93	0.89	0.82	0.86	0.35	0.92	0.82	0.97	0.94	0.050	0.221	0.000	0.005	0.0009			
rs757601	16	2.00	CACATGACAGACTGATAGAATCTCCAAATGTCTTTTTCCAAAAAGGGCACAGACTATA[A/T]GCCCACAAATTGCTCAGAGAATTGCAGCATCTCTTCAACTCCTTTGATGTAAACC	A	T	0.21	0.62	0.65	0.60	0.57	0.73	0.49	0.67	0.69	0.64	0.66	0.096	0.010	0.007	0.005	0.0003	1		
rs4786287	16	2.49	TTAAAAATAAATGTTAAACATTTATTCAGCACAAAGTGGTCTATTCAAAAAAAT[A/T]AAAAAATAGGCTAGGCGCGGTGGCTCACGCCTGTAATCCCAGCACTTTGGGAGGCTG	A	T	0.52	0.49	0.43	0.48	0.46	0.83	0.67	0.31	0.29	0.38	0.45	0.004	0.024	0.020	0.083	0.0004	1		
rs879416	16	2.92	GTTGCTTATGCTAAGGGGAAGCACCACCTTGAAAGAGTCATTGTAGAGTGGAGGT[C/G]GGGAGGGCAAAGTTGAGATACTGAGGTTTTGGGGTTTGGTTTAAGGCAGGTCCTT	C	G	0.07	0.15	0.13	0.15	0.13	0.59	0.52	0.18	0.04	0.09	0.10	0.001	0.099	0.005	0.134	0.0000	1		
rs1063677	16	2.95	TTACAACCCATCCTTTCTGCTTCTCCAGGCCAGAAGCCAAGGAGTCCTCAGTGAC[C/G]TGTGGGATCCACAACATCTCCACATCGCTGTCCCCACCCAGCCAGGGCAGCGCCA	C	G	0.62	0.51	0.55	0.41	0.43	0.05	0.34	0.16	0.75	0.61	0.59	0.001	0.035	0.130	0.193	0.0008			
rs11228	16	3.27	TTTAATTATATATTTGTATAGTCATCTCACTGTTGTTAATTTTCAATAGTTT[C/G]TTGGTTTAGTTCTGTTAACTTTTGGTAAAATGACACCATCTACAAAGAATAGTTTTTGTTTCT	C	G	0.40	0.51	0.50	0.50	0.59	0.77	0.86	0.77	0.43	0.49	0.52	0.007	0.064	0.032	0.034	0.0003			
rs859302	16	4.78	AACAGGCTGGTCCTGCCCCTGCTACACCACTGCACTAGCAGACTGATAACCCA[A/G]TGCAGTCAACCCCGGGAGTAACTCAGACAAATTTATTTGCTGAAATAATGTATGCTT	A	G	0.45	0.65	0.57	0.59	0.68	0.45	0.44	0.50	0.61	0.57	0.58	0.005	0.011	0.001	0.005	0.0000	1		
rs1012259	16	5.95	CCCCCAGGATCTGCTGCTCACCTCCAAGCTCTGCCCATGGACACTTTGAC[A/G]CGTATCGAAGTGACCATGTGCTAACCTTGCCCTTGTCTCTACTACAGTTCTTATCTCAG	A	G	0.05	0.41	0.38	0.33	0.43	0.50	0.42	0.50	0.36	0.39	0.35	0.083	0.003	0.016	0.014	0.0002	1		
rs2043631	16	5.97	GAGATTGGGAAAGGAGGTAAAGAAAGAAATAAGAGAGGGAGGAAGGTGAAACCGA[A/G]AATTAATACAAGCTGATAGACAGTAAGACAAAAACAAATAGAGACAAACTCCAAC	A	G	0.29	0.36	0.39	0.33	0.43	0.41	0.38	0.52	0.25	0.42	0.39	0.004	0.002	0.016	0.004	0.0000			
rs1035564	16	6.11	TTTACAACACCATAGGCAAAGCATGAAGCAAGCATCAAAGTATATGGAACTGCT[A/G]ACGTGAGAGGTCGAACCTGAAAATACACTCTGTGAAAGGTCAACCAAAAGCATGACA	A	G	0.38	0.69	0.60	0.62	0.41	0.59	0.57	0.52	0.50	0.62	0.70	0.016	0.001	0.000	0.000	0.0056			
rs933478	16	6.28	GCATTCATCCCACTCATGAGGGGAGGAGCCTGTATGAACCAGTCACCTCTTAAAG[C/G]CCCCACCTCTTTTCACCATCACAGTGGCATTAAGTCTCATTGCCCTTCACCAGC	C	G	0.55	0.33	0.36	0.30	0.30	0.27	0.14	0.32	0.32	0.41	0.26	0.016	0.022	0.000	0.002	0.0074		1	1
rs716508	16	6.34	CTTTAAATATATCCTTCAAGTCAACTCTACCACTATATAGTTGCTGAGCAAATATT[A/G]CCATGTATGGGAAAAATGTAATCCTTGTTCTTACAGATGTTGCATTCTAGTGCAGGG	A	G	0.33	0.65	0.70	0.68	0.59	0.91	0.84	0.66	0.54	0.66	0.66	0.058	0.041	0.001	0.051	0.0005			
rs1019141	16	7.72	AACTTTCAGAAGGAGGTCATTTGAGAGAAGAGGTTGTGGCTTGCCAGTTAC[A/G]GGGATCACGCAGGTCTTTCAATGACTATGTGCTTTGGCAGAACTAAAAAATGCCCGAGG	A	G	0.74	0.54	0.42	0.37	0.54	0.32	0.24	0.73	0.25	0.48	0.43	0.056	0.023	0.046	0.008	0.0000	1		
rs763649	16	7.73	TAAATTAATTAATAAGTAAATGGACAGAAAGCAATCTTTAAAGTTGTATTTCTG[A/C]TGTTTTAAAATGTCTATTAAATGGTGCTACTTCCTTTTATCTGTAATAAGCAGATATTGA	A	C	0.52	0.56	0.47	0.40	0.57	0.95	0.41	0.79	0.57	0.42	0.45	0.004	0.001	0.060	0.172	0.0002			
rs1507038	16	8.04	AAAGTCCTGCTGTACGGGGAAGCAAACAGGCAGGATGAGCAATGGATATATTTAA[A/C]ATCTCTAGCAGCTTCGAAGCTAGTGTTCTTCAATACAAATCAACCTCAATTTATTTT	A	C	0.14	0.43	0.35	0.39	0.36	0.36	0.45	0.31	0.39	0.40	0.44	0.020	0.004	0.005	0.001	0.0040			1
rs1532930	16	8.09	TGCCTCAATAACTATCAAATATCTCCCAGCTCAAGTTCAAATTCTTCAGCCTGG[A/G]GGACAAGGTGGTGCAAATAGACAGAATGGGAATAGGCTTTGGAAACAGGATGAATAC	A	G	0.24	0.49	0.45	0.52	0.43	0.50	0.54	0.44	0.36	0.45	0.39	0.015	0.011	0.000	0.006	0.0006			
rs997097	16	8.22	ACCATTCTCTAACCTACTTAGATGGAACTGCCATGGGGACTTCTCCTATTGCCT[A/G]CCCTTCACCAGAGATAAAGCCAATCGCTATTCTACAATGACATTTGTAAACTTGTGAG	A	G	0.24	0.31	0.36	0.51	0.30	0.59	0.67	0.26	0.25	0.39	0.36	0.006	0.054	0.004	0.029	0.0001			
rs933488	16	8.66	GCAGTGCCACACTCTGGCTCTCCAGAGTGACATCTATGCATGCGACGTCCCCAAAACACACCAGGCCAGGTTCCCCTGTAATTCCATAATGTCA[A/G]GGCAAAAATAGTAGC	A	G	0.52	0.38	0.39	0.37	0.48	0.27	0.33	0.28	0.54	0.46	0.46	0.003	0.007	0.012	0.011	0.0010	1		
rs1420040	16	9.82	ACCTCTCTTTCTGACTTCTATTCTGACCTCCCTGGAGGAGACCAGGCACTTTGA[A/G]AATATGTCTAACGTTAGGGATTTACTAGCTAATTCTAGTTGCTGACGAGGTTGAGAA	A	G	0.71	0.52	0.56	0.56	0.46	0.59	0.71	0.41	0.61	0.51	0.52	0.022	0.019	0.002	0.003	0.0007	1		
rs2024495	16	10.38	GGCGAGATGTGAGAAAAATTATGGCTGACATGGGGGCTAAGTCAGATTGATTTGTTTTA[C/G]ACAAAAATGAATTTTATATCTGAATATTTTGGAGAAAATTCATGCAACTCCTA	C	G	0.19	0.32	0.40	0.34	0.41	0.09	0.51	0.23	0.21	0.34	0.28	0.016	0.016	0.004	0.050	0.0067	1		
rs10221	16	10.54	TGGAAGTGGAGCTGTGACTAGTACAAGCAGCCCAAGAAACTCTGAACGGG[A/G]CCCAATGGAGGCAAACTTGAGCAAATAATTGGGATGATAAGAAACAAAAA	A	G	0.29	0.62	0.60	0.53	0.46	0.18	0.55	0.47	0.57	0.43	0.45	0.076	0.007	0.022	0.131	0.0119		1	1
rs741175	16	11.13	TGACCCACTCTTGCTACTTGTGTGCCAGCCACCCAAGTTTCTGTGAGGACCCTGG[A/G]ACACAGCACCCTCTCTCCCACCTCAGCCTCACCTCTGCCATCCTTAAAAGGCAGT	A	G	0.38	0.64	0.54	0.48	0.46	0.82	0.88	0.47	0.61	0.48	0.53	0.018	0.070	0.003	0.039	0.0000	1		
rs887864	16	11.13	AAGCAACACTCTGAGAACAGTGTCCTGAGACATTCAGGTCATTGCTC[A/G]GATGATGGCACTGTTTTGAGGGGTAAGAAGATATAGCTCTGGCTGCTTTGGCCAGAGGAAGG	A	G	0.50	0.73	0.63	0.57	0.52	1.00	0.97	0.53	0.71	0.60	0.63	0.017	0.090	0.008	0.129	0.0000			
rs918738	16	11.41	CAGACCCGCCCCGCCCCCGTTCGAGTGTCAACAACAAGATCGAATCTGGCCTC[C/G]GCAACTTCTCGGCGGGCAGCAGGGAAGAGGGCGCCCGGTAAGGCCCGGGCATTAC	C	G	0.43	0.59	0.55	0.62	0.48	0.32	0.06	0.24	0.50	0.49	0.56	0.009	0.174	0.052	0.031	0.0004			
rs1035579	16	12.50	CTTTCACCTGAGTCCTG[A/G]CAAGGTGTCACACACTGTGCTCTCAGCTCAGGATGGAGTGGATGGGCCTGAGGCCCTGCCCACCGCATTCCCTGTCTCATAGCGGGGAGAGT	A	G	0.62	0.51	0.49	0.50	0.46	0.36	0.53	0.76	0.43	0.49	0.55	0.006	0.001	0.055	0.008	0.0019	1		
rs1002970	16	13.40	CTAAGGAAAGCACCCTAGGCTATCTATGATGTCTCCTCTTCCTATCTGTACAAT[C/G]TGCTCAGCCTCAACACATACTCTTTTCCAGCCACTTCAAATATCAGCAGATATTTGGC	C	G	0.24	0.76	0.68	0.74	0.75	0.91	0.71	0.75	0.64	0.71	0.73	0.073	0.004	0.007	0.060	0.0022	1		
rs734826	16	13.46	GGTGAGACAGTGTCCTCCACTAGCCAACTCCCCGACCCTCAACCAGAAATCAAT[A/C]TTAAGGGCTCATGGCATAACCTGGTTTCTCTCAGACAGACTAGACCCAAATCCTCTT	A	C	0.07	0.45	0.39	0.52	0.36	0.36	0.57	0.30	0.46	0.42	0.47	0.059	0.023	0.007	0.000	0.0030			
rs1001937	16	13.50	CCATAAACAACTCGGTTACTGTGATTCTACTGAGAAGATCCTGATAGCTTGCAATTCCCTCT[C/G]CTGTGAACTCCCCCACTGAATTCTGGGGGCCCAGAGCATTTCTCAAGTC	C	G	0.69	0.44	0.39	0.49	0.52	0.18	0.38	0.18	0.39	0.36	0.43	0.049	0.001	0.032	0.032	0.0004			
rs1547405	16	13.56	AGGGTCTTTGAAAGGCATTTAAGCCAGAAAGAGGTGTGATATATTCATGGCAAGC[A/G]TGAGCATGTCAGTACATATGACAGCACCCTCTTCACCAATTTTACAGCAATATTCTC	A	G	0.83	0.63	0.62	0.63	0.71	0.55	0.99	0.68	0.61	0.66	0.64	0.025	0.116	0.006	0.006	0.0002			
rs1158123	16	13.68	TCTCAATGAGACGTCATGGTGGTTTGGACAAGGGTGAGCTCAGCAGAGATGGG[A/G]AGAAGGAGACATAGTATAGTCCAAATTTGTTTTGAAAACCCTGGCTGCTGATGGAG	A	G	0.26	0.57	0.60	0.52	0.70	0.77	0.85	0.46	0.54	0.54	0.52	0.050	0.028	0.013	0.013	0.0020			

rs2384933	16	15.78	CAGGGCCACCCATGCTTACCATTTCCCTACTGAGGCTCTTAGGATACTTTTGT[A/G]AGATATTAATAGCTACTTGTGGCCATGAAACAAACTAATAACTAGCTTCTGGCAGCTTT	A	G	0.02	0.31	0.35	0.33	0.41	0.18	0.30	0.21	0.32	0.29	0.36	0.081	0.000	0.024	0.012	0.0008	1		
rs212090	16	16.20	ATTCCCATGAGGCCATCTCCTTAATATTTACCCCACATCAATCATGGTGGGA[A/T]CAGTAATAACAGCATTGATTGTTCTTACAATGTTGAACAAAAGAAAAAAGGGAAAGCCTG	A	T	0.93	0.57	0.57	0.50	0.59	0.82	0.53	0.66	0.54	0.53	0.58	0.085	0.002	0.003	0.026	0.0000			
rs1472426	16	17.16	CCATGATACGGTGTTTGTGGCTTGCTGAGTCGATGCGTTAAGCTTCTAAACAT[A/G]GAGCTGATGAGAATAGCAATGGCTAACCCTGTGTTAGAAAGCACTGGTAACGGACGC	A	G	0.17	0.42	0.42	0.43	0.36	0.55	0.23	0.31	0.43	0.42	0.37	0.048	0.040	0.009	0.003	0.0018	1		
rs936347	16	17.24	GTGTGGGACCTTTAGAGCCAGGTTAAGGAGTCCAAGCTTATCCTATAAGCAATGGG[A/C]AGCCTTTGAAAAGTTTTCTATTAGAAAATGCCATGATGAGATTGGTTCAGTTTATA	A	C	0.07	0.56	0.54	0.61	0.55	0.50	0.32	0.34	0.68	0.47	0.59	0.123	0.024	0.020	0.000	0.0016			
rs1457907	16	17.31	GAATTAAACCCCATTGCCCAAGCTGTCCTTGACTTCAATCTGACCTGGTTC[A/G]TTGCCCTGAAACCACTAGGGGTTCCAGTTGAATGACCAGAGACCCTAGGGTGTGTGGCT	A	G	0.29	0.53	0.39	0.44	0.30	0.64	0.35	0.34	0.50	0.44	0.35	0.005	0.000	0.000	0.045	0.0000			
rs1379652	16	17.36	GGAACCTTTTGAAGTTCTGTGGTTTGTTGATTCATCTCTTTACACCCATCCCT[A/C]TTTCAGGAAGGGTTTATGGCAGTCTGCATGGGTGTACTAAACAAAATAAGACTGCAAAA	A	C	0.81	0.47	0.50	0.43	0.57	0.41	0.71	0.56	0.57	0.50	0.51	0.051	0.021	0.006	0.005	0.0001			
rs1389504	16	17.90	AGAGGGTAAAAAAAAAATGCCATCATCCTCCCACCCCCCAGACAAATTAGCAAGA[A/C]AAATGTTAGCAAAAAGCTCTGCAGACGCACCATTATTTACAAAGGGAGAGAGAAC	A	C	0.14	0.37	0.42	0.34	0.29	0.32	0.24	0.30	0.36	0.27	0.34	0.069	0.025	0.016	0.013	0.0040	1		1
rs757189	16	17.95	ATCTATTTTATGTCTCTAGCCAAGGAAAAATGCAATGCCTCCTTTACAGGTTTGTTCTGCTGGAGAT[A/G]GTGATTATTAAAGCAATTTGTTAGAGGCAGATTTTGCGTACATATT	A	G	0.36	0.51	0.44	0.49	0.55	0.45	0.57	0.51	0.54	0.60	0.50	0.000	0.011	0.006	0.002	0.0008			
rs904821	16	18.81	AAGACACACTGAAAAAAATCACATCCAGGTGAAAG[A/T]CTTCTTGAGGTAGAACATGGCTAATAAATTTCTTGGATCACTCACATGCACCATTTAAATAGTAATTCTGAAGATGT	A	T	0.15	0.46	0.40	0.45	0.45	0.82	0.55	0.47	0.70	0.38	0.42	0.055	0.014	0.004	0.001	0.0001	1		
rs1544357	16	19.18	GCTTCATAACGACTAAGTCTGACTGCC[A/C]ACAATAACCCTTTAAATTAAGTACTACTGAGATCAGTGTTGTGGTGGTAAATCTTTAGTTCTCGGGGTGGGGTGGGCAGAGGT	A	C	0.12	0.36	0.35	0.24	0.18	0.50	0.42	0.30	0.29	0.28	0.36	0.041	0.001	0.002	0.006	0.0004			
rs724307	16	19.23	TCTGCAGGCCTTACCTCATCCCCAAAGCTCATGACGTAGATGAAATATTGTAA[A/C]TAGCTCTGTAGACAGTTTGCAGTGTACATCTTCCCCCAAGCGATGCCAGCTTCTCTGA	A	C	0.24	0.59	0.66	0.61	0.63	0.77	0.43	0.71	0.61	0.64	0.60	0.081	0.021	0.009	0.013	0.0028			
rs2023762	16	19.24	ATATTTTTTGTACATTTGGTAACATGCTCAGTATGCATAGGTGGACCTCATTTAC[A/G]TGCAAATTACTTACAAAGAGAAATAATAGGAATGGAAGCTTTGCTTAATAAATGAAAT	A	G	0.79	0.52	0.50	0.46	0.50	0.05	0.05	0.26	0.39	0.46	0.53	0.040	0.141	0.027	0.147	0.0028			
rs2106671	16	19.24	GAAGTATGGGAATTTTGGTAGCAAACACAGTTAAAAAATGTTAAGCCTTGTTTAAA[A/G]CTTATGGGGAAAGGGATCAGTAAGTGGAAACTTTACTCTCACAAAATGCTTTTAGT	A	G	0.69	0.53	0.46	0.45	0.46	0.27	0.60	0.39	0.50	0.42	0.49	0.017	0.006	0.006	0.025	0.0018			
rs179209	16	19.27	ATTTGCTGCTTCTCATCCATGCTAAGGGAGAAACTGCTGAAAAAGTCGTCCCA[A/G]GCAGCGATTCAGTAGAGCCTTTTTGTGTTGTAGGCAGTGCTCAAAAGTCCCTACAAAG	A	G	0.19	0.53	0.48	0.46	0.50	0.09	0.33	0.33	0.57	0.36	0.42	0.051	0.026	0.025	0.125	0.0020			
rs179219	16	19.28	TATTATTAGTTATAATAAACTATAATTCTGCCTCAAGGAGGCCAGTGTTTTTTAAC[A/G]TTCCATATCAGTTCATCTTTCTGAGAGTGAGCAAGGCTTGTCTCACTCTTCCACTTTC	A	G	0.81	0.45	0.49	0.51	0.48	0.82	0.67	0.67	0.43	0.59	0.54	0.058	0.032	0.031	0.080	0.0013			
rs875648	16	19.63	GTGATGAAGCAGTAGGCAACACAGGCCTAGGAGAAGGGAAGAAACATTAACGCT[A/G]GCAGTGACAACGGTGGCAGAATCATACTCCTGTTTTCATCCCTCATGTATTTCCAAC	A	G	0.12	0.59	0.64	0.70	0.68	0.73	0.67	0.61	0.73	0.58	0.62	0.139	0.000	0.001	0.001	0.0004	1		
rs227761	16	19.83	TGTTTTGGCAAAGTGTAAAAACAGGGAGAAATGTCAGTGGGAAAATACGGTGC[A/G]GCACATTTCCTATAAATTTCCATTATGCAAATTTGAAATTGTTCAATACCAAGTTAATC	A	G	0.07	0.50	0.49	0.45	0.50	0.18	0.47	0.44	0.57	0.45	0.43	0.105	0.000	0.001	0.043	0.0021			
rs727590	16	20.01	TAAAAATTCTTATAATACACACACCTGCAAACCTGGTGTGCTCATCACTCAGATTT[A/G]ATACATTTTAACATTTTGCCATATTTACTTCAGCTCTCTTTAAGAAAGAAATGACA	A	G	0.90	0.71	0.77	0.82	0.75	0.68	0.98	0.80	0.69	0.84	0.76	0.013	0.039	0.004	0.011	0.0005			
rs2031077	16	21.20	TAAACTCCAAGCATTGTGTTGAAATAAAGCCAGAGTAGAATAATGATTGAGAACAT[A/G]GGGTCTGAAGTCAGAGACTGACAGATATTAAGCCCCAGATTGGATCTTCCCATTA	A	G	0.26	0.27	0.32	0.33	0.25	0.32	0.34	0.39	0.21	0.34	0.28	0.000	0.003	0.019	0.002	0.0007	1		
rs741720	16	21.21	CTTCCTCTGTCTGTTCCCTAAGCCACAATCACACTACTTCTAGTTTGTAAAAACAT[A/G]CTTTATGGGTTGCAAGAAATTTTATTTCTTCATCTTTACTAAAGTTAGTTGATGCTG	A	G	0.38	0.43	0.43	0.34	0.43	0.59	0.65	0.43	0.46	0.34	0.34	0.006	0.021	0.001	0.010	0.0061			1
rs208965	16	22.83	GCTGTCACCTGTGAGCTCAGTTTGGGCTTTCAACCAGAGTGACTACCCATCG[A/G]CCTTTCTGTAGGGCCTGGGCTTCCTCTGCACTGGCAGCTGGGTTCTGGGAAGGAGCATC	A	G	0.00	0.52	0.61	0.39	0.57	0.00	0.52	0.29	0.43	0.52	0.59	0.262	0.000	0.021	0.237	0.0003	1		
rs1858799	16	23.08	AAGGCATGTGACTTGTTCAGGTTCGAGGGCTGGTTGATGACAGGCCGGACC[A/G]GAACCTGCATGCCCAATCCAGTGCTTTTTCACTAGACAGCGTGGCTGAGCATATAAT	A	G	0.81	0.74	0.67	0.71	0.68	0.82	0.51	0.72	0.61	0.73	0.73	0.008	0.021	0.001	0.010	0.0015			
rs152733	16	23.33	GGTCACTGTATGGGCTCCAGGTTTGAATCCTGGCATGCCACTTAATATCTTGGGC[A/G]ACTTAACCTCCCTGAGGCTCTGTTCTCTCATCTACAAAACAGGGGGATAAAAATA	A	G	0.74	0.81	0.80	0.84	0.79	0.32	0.78	0.83	0.86	0.77	0.79	0.001	0.003	0.000	0.146	0.0002	1		
rs194790	16	23.76	TAAACCTTTCGTGATGACAAGAGCCAGCCACCAATTATCTCATCCCTGGATTG[A/G]CATTATTGTAAGCCACAAAGTACCAAGCTTTCCTTGTGCTTAAACTGTTGAGGAAAATG	A	G	0.67	0.54	0.48	0.56	0.48	0.59	0.20	0.67	0.54	0.59	0.61	0.020	0.036	0.037	0.010	0.0107	1	1	1
rs411103	16	24.20	TCAGTGGGTTCCTGATTTTTACCTGACCACATCGCTGCTATGTAGGCCTCATC[A/T]CAGTCTCTCGGACTGGAGGGACAACCCCATGGCTGTTTTATTACAAGACAACCAGGG	A	T	0.31	0.53	0.64	0.59	0.64	0.32	0.54	0.71	0.50	0.69	0.57	0.070	0.009	0.000	0.080	0.0054	1		
rs991911	16	24.26	GTATGTGCCTTGAGCTCATTCTCTCTACCTTTACATGCAACAGTCAGGAAATT[A/T]AGTTGATTCCAGCTTTCAAGCATCTACCAAAGTGATGGCAATGAGTCCATCTCCCTT	A	T	0.62	0.67	0.63	0.62	0.66	0.64	0.82	0.42	0.73	0.55	0.63	0.003	0.007	0.074	0.003	0.0002			
rs1011078	16	24.86	CTGGCTTTGATTCTGGCCTCAGTTCCCTGCCGTTCAC[A/G]CATGCAGTGAGTCAGCCTGAGTGTGGTGAGATCCTGGTGTTGGGTACCACTTAACCCCACCATCCTTTTCTATA	A	G	0.14	0.44	0.53	0.51	0.52	0.45	0.47	0.39	0.36	0.43	0.55	0.084	0.002	0.023	0.006	0.0014	1		
rs874562	16	25.07	GTTTCTTGACATTTAGCTGTGAGCTCCATAAATAATGGCCACATTAACCAAGATCTGAGATCCTCAATACCTTGGATGAAGTCC[A/G]TCCTAGGATATGTGGTTTATACCTGGGG	A	G	0.43	0.53	0.49	0.52	0.54	0.77	0.78	0.51	0.25	0.39	0.42	0.003	0.039	0.000	0.034	0.0023			
rs2157857	16	25.48	TCCATGGAACTAAGCCAGGTTTCTCTGGGGTCTCAGATGCTTCATCGGGTGTCAACTGGGT[A/G]CCTCTTTGGCTCTCAAACTTCACATTCTTGCCTTTTCACATTTGTATTTTCTGGT	A	G	0.71	0.58	0.54	0.41	0.54	0.86	0.55	0.56	0.50	0.59	0.59	0.006	0.008	0.007	0.032	0.0016	1		
rs1022455	16	25.50	CATAAAAGGAACGACTGTACTTTTCAAAAGTGTCAACTCTGCCTACTGTTTTTTGAATCTAGCTCACGGTTGGGAAAGACTTCAATA[A/T]GTTTACATTCCTAAGATTCTCCACCG	A	T	0.31	0.58	0.53	0.41	0.55	0.86	0.55	0.46	0.50	0.57	0.58	0.032	0.002	0.009	0.050	0.0018			
rs205162	16	25.56	GGGGAGGAGCTGGGGGTCTTTGTGGCAATGTATTAGAAATTTTGAGGGATCTGC[A/G]GGTCCTACTCTTATCCCCATTTAAATAGTAATTGTTAGCTACTGTATCTGCTTCTGA	A	G	0.47	0.24	0.22	0.28	0.23	0.41	0.50	0.41	0.43	0.20	0.17	0.063	0.091	0.046	0.054	0.0011			
rs2078274	16	26.57	TATTATCTGCAAATAATAATTATTGCATTGCTTCCTGTCTAAAAATTTAACTTA[A/T]TCCTTTATCTGTCCTTCTGCACTGGCCATAATGTCCAGACTATAAACTGTTCTTATTTATT	A	T	0.57	0.59	0.59	0.62	0.66	0.27	0.52	0.60	0.57	0.63	0.55	0.000	0.002	0.001	0.056	0.0015	1		
rs739480	16	26.58	GGACACCCCCCCTCCCACTATGCAGGGCATTACAGCATGGCATTTAACAGCCCCAA[C/G]ACTAGAGCGAGATGGCCTGGGTTGAAGTCCCATCTTTGCTGCTTTTGTTGGCAAA	C	G	0.60	0.79	0.78	0.88	0.84	0.86	0.85	0.76	0.86	0.79	0.73	0.014	0.017	0.001	0.017	0.0001			
rs237131	16	26.61	ACTCTTAGGTCAAGAGAAACTAGTTTGGAGATTGAAGGGAAAGGTTTAACCAG[A/G]ACTTCAGATGGGCTACAGATTTTTTTTAAGTCCTTATGCTTTTATTTCTACAAATGGGA	A	G	0.02	0.39	0.40	0.34	0.46	0.45	0.38	0.09	0.35	0.37	0.38	0.124	0.000	0.055	0.003	0.0000			
rs2057768	16	27.29	GTCCCACTGTTCAAGTTCCTGCCCAAGATGCCCAGACTTTATCTGTGACTGCTCC[A/G]TCAGTCCTGGGAGCAGGTCCAAAGGCACCAAGATAGTAAAACCTCTGTTCCCCCCA	A	G	0.24	0.26	0.34	0.29	0.18	0.50	0.19	0.38	0.21	0.30	0.28	0.009	0.017	0.000	0.010	0.0034	1		
rs8832	16	27.34	CGGCTGTCAGGGGTGTTCAGTTAAGGGGAGCAACAGAGGACATGAAAATTGCT[A/G]TGCTAAAGCAGGGACAATTTGCTGCCAAACACCCATGCCCAGCTGTATGGCTG	A	G	0.83	0.36	0.46	0.39	0.41	0.45	0.29	0.47	0.25	0.48	0.51	0.065	0.038	0.000	0.001	0.0007			
rs3093291	16	27.41	CCTGCTTCCAGGCTTCTTGTGAGAATTCAAGGAGGTGATTTGCATAAAACCCTGC[A/G]GATGGCCCCCATCACATAAACACTCAAACAACAGCTGTAATTGATGGAGAAGGTA	A	G	0.69	0.65	0.69	0.70	0.61	0.45	0.45	0.45	0.50	0.66	0.76	0.002	0.035	0.027	0.022	0.0034			
rs1646129	16	29.18	AGTTGTCTAACCTCTCTGTGCCTTCATTGCCTGACTCGGCTGTTTCCAATGCA[A/G]CTGATTTTAACGGCACCTGTTTCATGAGGCCACAGCAGGATTCTGGGAGGCAAAGC	A	G	0.17	0.40	0.29	0.38	0.36	0.55	0.11	0.38	0.39	0.28	0.31	0.006	0.017	0.010	0.040	0.0000	1		
rs11901	16	30.02	CCAGGGTCTGTCAGGGGCCAGGAAGCCCTCCTTTCCCAGTCCAAGGTTGA[C/G]AGGGTCTCTGTCATTTCCTGTCCCAGTAGGTGGTTGGGGCATTTAGGCCC	C	G	0.26	0.47	0.46	0.55	0.43	0.55	0.14	0.57	0.54	0.41	0.49	0.016	0.055	0.007	0.005	0.0006	1		
rs3574	16	30.45	TTTATTTAATGGGCTCAGAGAGCAAGGGAA[C/G]CACACAAAATTTACAGTCTGAGTTTTGCGCGCAGAGACCCCTCTCCACCTTTTTCATGCCTGTGTGTACACACACACTGTC	C	G	0.62	0.63	0.63	0.56	0.66	0.95	0.93	0.66	0.61	0.61	0.66	0.000	0.095	0.002	0.112	0.0029			
rs889551	16	31.30	CTCTTCCTTCTGTTTGTGGTCCCCCCCAGGGTGAGAGATTGAGAGGAAAGAATTG[A/G]AGGGATGGCAGGGGTTCCACATGACTGGTACCCATATGTGGTTGGAAATGCCCTG	A	G	0.21	0.30	0.28	0.32	0.27	0.36	0.46	0.63	0.32	0.26	0.28	0.004	0.010	0.054	0.001	0.0003	1		
rs13143	16	31.53	TGGGCGTGTGGGTTTAGGTCTGGCTGGGCCGTTTCTGAACTTGACCCCTGAG[A/G]TCATCATGTACCTTAGGGGCGGGGCTCTCAGTGGGTGGGGCCTCCCGCTCTGAG	A	G	0.26	0.27	0.26	0.24	0.18	0.18	0.22	0.11	0.18	0.17	0.26	0.001	0.011	0.024	0.012	0.0002			
rs1534507	16	31.70	TGGTCATGTACTGGGTGGACTTTATCTTCTCAACCTCTTTAACCCTGCTATGG[A/C]CGTATGACCCACACGTTGTCCTGAGTGTGCAGAGAGCTGTGCTCAATGCCTATGCCA	A	C	1.00	0.78	0.70	0.76	0.75	0.80	0.23	0.82	0.71	0.71	0.76	0.108	0.138	0.003	0.002	0.0016			
rs189378	16	35.89	GTGACTCTCATATGTGAATCTGAACCCAAGGTAGGATAGTGAATCTCAGACC[A/G]AAATTCAGCACACCTGTGAGGCTGTGACTACACTACTGCCTCACTGTTCACAAAGCA	A	G	0.00	0.13	0.10	0.06	0.07	0.09	0.15	0.10	0.04	0.14	0.10	0.036	0.004	0.002	0.000	0.0000	1		
rs700582	16	46.50	TGACTGGGTGGAAGAATTATTCACACTTTGTTTCTGTATATGTTTCCACACTTTTAT[A/G]ATTCATCACAATGAAAGAAATATTGCAACAAAATTAAGAAAGAAAATCACTTACTCT	A	G	0.48	0.15	0.11	0.26	0.14	0.00	0.00	0.05	0.25	0.17	0.14	0.086	0.050	0.012	0.050	0.0013	1		
rs40355	16	46.57	TATTGGTTGCTTATTCTTCATAGTAAAATGTCTATGACCTGTCTCATCCTGGCC[A/C]TACAGAAAGATGATAAAGCAACAGCCACAGCCCTGCCAGGATGAATCAAGGAGCTC	A	C	0.48	0.15	0.11	0.26	0.14	0.00	0.00	0.05	0.25	0.16	0.14	0.086	0.050	0.012	0.050	0.0013			
rs36471	16	46.57	AAAAGGAAATTTGAGGGGTAACCCTGTGTTCCAGTCCCTCTGGGATTCACC[A/G]TGCACCTCAGGAGGACCTCATCTGCCACAGAGACTCTCTCCCACCCACCGAGGGCAGG	A	G	0.52	0.85	0.89	0.74	0.86	1.00	1.00	0.95	0.75	0.83	0.86	0.086	0.050	0.012	0.050	0.0013			
rs1978320	16	46.67	AGACCAGATACAGACACTTAGGGGGACTCTCCTGGTGCAGGAAAGATTGCT[A/G]TGTTTGGGCTCTCCTGGCTGGATGACCTCCATAATCGCTTTAGCTGGCCTCCCTTA	A	G	0.19	0.63	0.64	0.57	0.71	0.41	0.33	0.76	0.54	0.57	0.61	0.100	0.026	0.020	0.012	0.0005			
rs1566467	16	48.29	ATGGTGAAAAGGTCAAACATACATGTAATGGGATTTCAGGAGAGAAAAAGAAT[A/G]GGAAATGGGCAATGTTTGACGAGCCTACTGCCAAGTGATAAAAGATATCAAGTCAC	A	G	0.33	0.50	0.53	0.54	0.52	0.14	0.38	0.39	0.54	0.55	0.62	0.029	0.018	0.036	0.122	0.0030	1		
rs1224	16	48.35	AGACAGTACACAATCAGTGGCAGCAGCACCAGCCAGAGTGGCAAGTGCTCAAAGC[A/G]GCAAGACACAAAGTGCTGTGCGGTCCACAATCATGGGGATGCTTCTGGCAGAAGC	A	G	0.57	0.34	0.32	0.30	0.25	0.36	0.59	0.45	0.43	0.32	0.29	0.035	0.035	0.005	0.001	0.0016			
rs1510986	16	48.94	GGACGCAGCAGAGTTTCATCTTCTGCCTGGTTGCCACAGGCTAGCAGTGTGGAAC[A/G]GAGCTCATGCTCAAAATATCAGAAAGAATGTGAGAACAGCCCCCACTCCTGCCCA	A	G	0.76	0.55	0.56	0.62	0.50	0.59	0.75	0.76	0.68	0.52	0.53	0.025	0.038	0.026	0.003	0.0004	1		
rs1861659	16	49.34	AGCACCAGGGACAGCCTGCGGCCTGCGCATCTAGTATCAGGTCAAGTCCTGAGGTCCTGGGGCTCAGACACACACACTGTAAATGGGCGCGGTA[A/C]ATGGTCCTTGGAGA	A	C	0.29	0.49	0.47	0.46	0.55	0.50	0.80	0.46	0.57	0.52	0.45	0.018	0.059	0.000	0.000	0.0007			
rs1064448	16	50.13	GGGCAACTCATCCCAGGACCCCAACCAAGTCTGGTCAGTCACCCATCTGC[A/C]ACCTGTAATCATCTGTAAAGGCTCTGATGCAAGACTTTGTGTAG	A	C	0.90	0.52	0.55	0.48	0.39	0.86	0.49	0.63	0.46	0.46	0.42	0.088	0.004	0.000	0.050	0.0109	1	1	1
rs745230	16	50.45	GGTGTGAGCCTTCAGGGCTGCTCTAGGGTCAGAGGAACTGGTTTGCAGATCA[C/G]GGCTGTGGCACGGCTGCCCAGCCCCAATTCCTCACCTTGCTGCTTTCATTACTTCCTG	C	G	0.48	0.29	0.31	0.26	0.30	0.68	0.90	0.50	0.29	0.29	0.38	0.018	0.237	0.028	0.080	0.0019			
rs2063099	16	51.24	AAAATTGACCTGTCTCTAACATGTAATTAGGGGCATGGATAATTCATCTCACTCCT[A/G]TTCTACAGTTTAATATGTAGGGACTTGGAAACTTTGCTCACATGTTCCACAAATCTT	A	G	0.62	0.48	0.50	0.52	0.52	0.00	0.04	0.21	0.46	0.52	0.49	0.161	0.056	0.237	0.038	0.0038	1		
rs1123428	16	52.36	GTTAGCGCTTATCTTTAAATTTATTTACAAACCGTGAATAATAGGGTTTCATTCATT[A/T]CTCTCTTTAACAAATGGCCCAGAGTCCTTGTATCAATTAATATGCATGTGTATTATCTT	A	T	0.29	0.59	0.51	0.55	0.61	0.41	0.42	0.58	0.54	0.51	0.44	0.009	0.000	0.011	0.000	0.0027	1		
rs1990637	16	52.82	AACTGAATACCTTTCAATCTTTTTTAAAAAAATCATTGTAGAAGGGTAAATGTTG[A/G]AAATATTTACAATATACTGCCAAGTGAAAGGAGGCACAAAATATGTAGGACATGATT	A	G	0.52	0.22	0.32	0.26	0.43	0.00	0.19	0.30	0.25	0.30	0.30	0.010	0.025	0.004	0.158	0.0001			
rs1946155	16	53.41	TTGCTCTCATCCAACTCTGCAAATCTTCATAACAACTCGTTCCTCTGGAACCTTCT[A/G]GAGACCACAAACATCTGCAGACTAAACCTCCAGTCCTAGTAAACAACCACATGT	A	G	0.50	0.25	0.29	0.18	0.32	0.73	0.01	0.44	0.21	0.26	0.36	0.017	0.110	0.001	0.078	0.0012	1		
rs1420558	16	54.54	CTGGCCTCATCTCCCCCACTGCTCCTTCGTGCCCTCTGCTTTAACCACACCAGC[A/C]TCCCCACTGTTCTTCAGACACACCATGGGTATTGCTACCACAGGGCCTTTGCACTT	A	C	0.52	0.70	0.66	0.56	0.55	0.27	0.75	0.63	0.71	0.55	0.66	0.005	0.013	0.002	0.060	0.0009	1		
rs837529	16	55.26	TCTTCCCCTTCATCCGGCATTCTGCAGTTTTGGGTTGCGCACCCAGGCGAC[A/G]TTAACCAATGCCTTAACTTAAGATGCAGAGAAGACTCTGCAGCCCACCTCGCTCCAGTC	A	G	0.36	0.59	0.60	0.63	0.64	0.64	0.64	0.63	0.68	0.66	0.60	0.019	0.005	0.001	0.003	0.0010	1		
rs243836	16	55.31	CTCAGCATTGTAGGAGCCCTGAATCTCCAAGTCTTAGAATTGTGGCTGGTGAGCC[A/G]CACAGACTCACAGAACGTGAATCTTAGAATCCTAAAGCCCTGAAGTTTTCAAATT	A	G	0.45	0.42	0.44	0.50	0.52	0.73	0.25	0.60	0.79	0.52	0.48	0.000	0.126	0.013	0.058	0.0001			
rs1203490	16	55.97	AAATACTGAATAGCAGGTAGGGAGCTTGGTATGAACTCAAGAAATAAAGAGAAT[A/G]TAGAATGGTTTTTGAGGTGAGGTTAAAAAAAAATCAGAAGATAGTAGTTGACAAGCA	A	G	0.36	0.43	0.51	0.41	0.45	0.36	0.24	0.47	0.32	0.49	0.51	0.025	0.078	0.015	0.038	0.0010	1		
rs1809348	16	56.06	CTCCCCAGCACTCCTCTCCCCTCCCCAGTGGATATGCCTGGATTGTCTGTC[A/G]ATGCCAGGAAAGTGCGATGGTTTCATTCTAGATTGGGTTCTGCAGCACTGTGTGAGA	A	G	0.48	0.47	0.55	0.48	0.43	0.45	0.38	0.68	0.46	0.52	0.58	0.004	0.020	0.002	0.009	0.0000			
rs735144	16	56.66	AATTTCAAATACTGTGTATTTTAGAAGAAAGGAAAGAGCCAAAGT[A/G]CAGCCTTTTCTCACTTGATCAGTAACTTGTGTTAGAAACTCAGTGGGGAGAATTCTTTGCGTTTT	A	G	0.76	0.66	0.58	0.60	0.63	0.27	0.75	0.41	0.68	0.52	0.46	0.025	0.022	0.014	0.040	0.0062	1		1
rs41383	16	56.89	TTGGCTTCTGTTTAGGATGTTCTCCCATTGTCTGACCTAAATCCCTCTTGCT[A/G]CAATTGACACTCTTACATCCTGTCTTTGGGTCCCCAAGCTTCTAGGCCCCAGGTTGTT	A	G	0.76	0.51	0.54	0.44	0.46	0.41	0.25	0.32	0.32	0.47	0.51	0.032	0.045	0.022	0.007	0.0001			
rs247041	16	57.65	TTTTCCTTTAGGCCTTGCAAACATGTCTAAAGGTATTCAATTTAGGTATTTCCA[A/G]TATTTTCTATACCCTCAGAAGCTCCTGCCAACTGCCCAGATCTGACTCACTGAGCACCA	A	G	0.31	0.55	0.62	0.60	0.48	0.59	0.30	0.40	0.42	0.65	0.59	0.046	0.039	0.026	0.000	0.0005	1		
rs37358	16	57.97	TATCCTCTGTAGGCAGGGTAGCAGAGCAAACTTGCTATAAATCATTGATGTCTATGAC[A/G]CAGCACCAAACTAGGTCTCACTGACCACAGAAGCGGGCAAGAAGCTTGATGTCGTTCA	A	G	0.41	0.45	0.42	0.41	0.46	0.14	0.09	0.32	0.38	0.49	0.45	0.017	0.079	0.009	0.066	0.0019			
rs1862802	16	58.25	AAGCCTAGAAGAATATTTAAGGTTTAATTTCACAGGCAAAAGAAACAAGCATG[A/C]GGAGCCCACTGTCTCCCACAGCAAGATGAGGGATGCTCTGGGGCATGAGAAG	A	C	0.81	0.47	0.48	0.45	0.45	0.68	0.93	0.57	0.50	0.32	0.44	0.058	0.138	0.010	0.025	0.0012	1		
rs6993	16	58.52	AGATAAAGTACAGTTTCCTACTCTCCACTATTCTTTGCTGAAAAGGGTGAGGGTAC[A/G]GCATCAGCAGAAAATTATGTTCTTCATTCTTAAATAAATCTCGATAGGATGGAGTT	A	G	0.33	0.38	0.31	0.39	0.32	0.77	0.96	0.32	0.32	0.27	0.43	0.001	0.201	0.015	0.069	0.0027			
rs990813	16	58.85	TTTCAGATTTGCCAAGGGACAAGACAGGTGACAAGATATACAATTTTTCTG[A/G]AAATTGAGCAGCAGGAGTATTGCAGGATCTCTTCCCAGTGAGAATAGAGACTCCGAAAG	A	G	0.74	0.31	0.41	0.49	0.43	0.32	0.38	0.39	0.36	0.38	0.34	0.056	0.002	0.001	0.005	0.0040	1		1
rs1482258	16	59.29	AGAATACAGGAAAGCACTGTGGCCTGATGAAGTCTAGGACCCAATCTAAATGCT[A/C]ATTCCTGCTCAGGGAAGGTAAAATGCGAATATATAATCAGGTACAAATTTAAAGCTTT	A	C	0.60	0.46	0.56	0.54	0.57	0.36	0.67	0.60	0.61	0.54	0.50	0.011	0.023	0.007	0.005	0.0000			
rs715262	16	60.60	AAAATAGTAATTCAATTGTTAGCAACCCCTCACAAATAACTATCCAACCCACTTG[A/G]TTTAATACCTCAGCGTCCTTGCATGCCTTTCCTTTTTGAAACACAAAGCAGTATTGA	A	G	0.76	0.58	0.64	0.62	0.55	0.55	0.72	0.74	0.54	0.63	0.69	0.008	0.007	0.002	0.003	0.0001	1		
rs716455	16	61.33	AGGCCTTTTACATTTTTGTTTATT[C/G]CTGCTCCCGAAACTACCAACTTTAAATACTTTTTTATATTTGAATTCTTTAGACATGTTTTTCAGTTATATAATCATTATAATTTCCTGA	C	G	0.50	0.37	0.39	0.45	0.48	0.68	0.74	0.59	0.54	0.45	0.44	0.003	0.039	0.019	0.031	0.0014	1		
rs1027277	16	62.46	GCAATGAGAAATACATTTGAAAGACTGTGGAAGATTTTTTGGCTCTTTGACTTAGGGAAG[A/C]TACCAGTTTCTCTAATACATTTTTTAACCATTTTTTCATGTGATATATATAT	A	C	0.17	0.46	0.48	0.41	0.46	0.55	0.56	0.35	0.54	0.45	0.43	0.058	0.010	0.003	0.002	0.0022	1		
rs1424032	16	62.74	GGAGCACAGATCTTTGTTGGACAGCTCTGTACATCTGCACAGTTTCTTAAGACTG[A/G]GATCTTACTGCATATTTTATTAGTGTTGTATCATCAGCAGAGACTTACTCTTCTGAAGA	A	G	0.88	0.82	0.80	0.85	0.89	0.95	0.95	0.88	0.93	0.77	0.83	0.005	0.022	0.020	0.030	0.0005			
rs153672	16	62.78	ACACTACAAAAGAGTGTTTCTGCAGGGCTTATTAGAACACACTGATTGTTTT[A/T]CCTGCACATGGATGACTAAGAATGATATTTCACTAAAACACCTGGATTGCTAGGGTTT	A	T	0.24	0.62	0.68	0.67	0.82	0.73	0.94	0.66	0.64	0.64	0.71	0.093	0.048	0.001	0.001	0.0004			
rs918703	16	63.31	GGAAGAAATATATTATATTGTAACATGGGTTTGCTAAATAGTCATGCTTGCATC[A/G]TAATTCAATTCTATATTGACTCTGCATCTTTTCTAGAGGCAAGATTCTTCAAAGCCTAAG	A	G	0.52	0.54	0.53	0.44	0.46	0.59	0.91	0.63	0.62	0.48	0.58	0.005	0.069	0.001	0.001	0.0005	1		
rs1423832	16	65.33	GGGTTCAGAGTTGGTTAACTTTGAGGCTCACTTACATCGTCAATGACCTAAGCTCTTTCCACCTTTCC[A/G]CTTCACCATCCCCAGTGGGTTAGCCTGTCCCATAATGGCTGCT	A	G	0.40	0.36	0.39	0.37	0.34	0.23	0.48	0.31	0.43	0.30	0.32	0.001	0.003	0.002	0.017	0.0026	1		
rs1359839	16	65.47	GGAGAGAGGTGGGGATAGTGTGGCAGTCCCCAAACTCCAGCTCTGGACCTAGGCC[A/C]CTGGAGCTCAAAGCCTGCAAGCCAGCTTGATGATCAAGCATTCTCGTGACCTGG	A	C	0.53	0.26	0.24	0.35	0.21	0.18	0.03	0.28	0.35	0.33	0.27	0.044	0.038	0.003	0.001	0.0004			
rs461785	16	65.59	CACGGCCCGCCACACCTGGGGAGGAAGGGAGAAAACCACACCTGCTCAGTGC[A/G]AAGAATGGGTTTTGAAGCTTAATGGTGCACGGAATTTCTGTGTCTTTTTGCTGT	A	G	0.19	0.53	0.50	0.44	0.34	1.00	0.85	0.53	0.50	0.36	0.45	0.048	0.067	0.001	0.216	0.0023			
rs149156	16	66.69	CCCCGACACCAGATAATCCCTCCGTCCCTTCTGGTCCTAACATTGTGGCATTCC[A/G]GTGATGACCTGCTTCCTTATAAATTATTTTCACAGAACTCTTGTAACTATGGTAAGTC	A	G	0.12	0.52	0.53	0.41	0.46	0.36	0.31	0.31	0.57	0.40	0.41	0.129	0.032	0.026	0.031	0.0065	1	1	1
rs9033	16	66.96	GCATATATGTGCTTTAAAAATATTAATCCTTTTGAAAAGAACTGAGAAGA[A/G]AAATGTATAATTTTATCCCATTTTTAATATTTTGGTCTAGCAACTTGTGA	A	G	0.12	0.51	0.53	0.50	0.52	0.50	0.57	0.39	0.50	0.43	0.50	0.120	0.000	0.006	0.002	0.0000			
rs1050356	16	67.47	AATGGAGCCTGGGACTAGTGACCAAGAGTTGGGGCAGACCCAGGCACTCACCTG[A/G]CAGCTTGGACCCGAGCACAGAGGACGTGCAGGGTGGCTCATCTCATCTGG	A	G	0.12	0.47	0.49	0.48	0.54	0.73	0.47	0.38	0.39	0.45	0.41	0.129	0.011	0.018	0.005	0.0039	1		
rs1177648	16	68.37	TTACCAAAGCAGTAACTACAGCATAGATTTCAAGCACAGCACATCTCTCACTCA[A/G]GATGAAAGCACTCTTACCGAAGTGAAATCCTCACCATTGTAATGACTGTGGGGAGAC	A	G	0.74	0.38	0.38	0.46	0.38	0.23	0.41	0.22	0.21	0.40	0.47	0.070	0.000	0.023	0.013	0.0009	1		
rs1541979	16	69.10	CCCAGGCCCATCTCTCTAGCAGCCATAGGACTGTGTGTGTTACTCAGAGCA[A/C]CTCAGAAGGGAGTAGGTCTTTCAGGTTAGACCTGGCCTGACAGCCCAGAACTGTCTCA	A	C	0.48	0.31	0.35	0.20	0.32	0.05	0.07	0.31	0.29	0.23	0.27	0.004	0.078	0.005	0.091	0.0039	1		
rs246142	16	69.24	ACATTTTAGATAGGCACAATTTACGTATGTACATTACACCTCAATAAAGTTTTAAAT[A/T]TTTAGAAGTCTATCCTGTTTCCTTAAAAAAAAAATTAGTTGCACCATGGTCTCAAAA	A	T	0.05	0.23	0.18	0.18	0.19	0.41	0.24	0.16	0.14	0.08	0.15	0.018	0.004	0.000	0.034	0.0008			
rs244415	16	69.44	CTAATGTAGTATACTCTTCATGAAATTAAACCAGAAACCCACTAACGAGAAAG[A/G]TAATTCACCTACAGATGCTGTGAGCTAAAATGTTGTGAGTTGATCTTGGTTATTCTTTAT	A	G	0.19	0.43	0.35	0.38	0.48	0.23	0.26	0.29	0.36	0.38	0.35	0.018	0.006	0.002	0.013	0.0001			
rs722579	16	70.30	GGAATAAACCCAGTGAATCCAC[A/G]CTTACTAGGGCTGTCAGTACAGGGGAAACTTTGAAATCAATACCATTTGAACTCAAATCCAGAAGTCCTATCGACAGTTATTCTTGGTG	A	G	0.38	0.38	0.44	0.48	0.38	0.60	0.43	0.65	0.14	0.49	0.42	0.003	0.004	0.016	0.008	0.0016	1		
rs235987	16	71.03	ATTTCACCCCAAAGTTTTAGCATAAGAGCTTACATCCTTTCAGTAGGAGTGGCA[A/G]TTGTGATGATTTTCAAATAGTGCTATCATATTTCTTGTACATTCATTCATCAGCTAGAAT	A	G	0.05	0.21	0.20	0.27	0.16	0.05	0.01	0.18	0.46	0.28	0.23	0.041	0.085	0.003	0.037	0.0000	1		
rs1018910	16	71.05	CACAAAACCCTTAATAAACAGTCTTAACTTACGCTACTGGCAACTGTAACTGCCAG[A/G]GTTATCAAGAGCCATGGAAGACTGAAACCTTCTCTACCATGAATAAATGGGACTC	A	G	0.71	0.77	0.76	0.78	0.73	0.68	0.21	0.70	0.89	0.81	0.75	0.008	0.230	0.023	0.020	0.0002			
rs310334	16	71.35	CAGAGGTGGTTGAGTCTACCACTGTCTGTTCCCTCAATGGCCCTCTTCACTCT[A/G]TTCACACTGTCATCTGCTGTTCTGGTCTTATTTTCAATGCTGGGAAATTTTACTCTGAC	A	G	0.93	0.69	0.70	0.63	0.64	0.14	0.22	0.60	0.61	0.68	0.76	0.081	0.052	0.000	0.105	0.0030			
rs719353	16	72.82	TCTCTGTGCCTTCTGAAAATTTTATCTTGGGCATAT[A/G]CTGCTTGCTTATTTCAGAAGCAAAGTCACCCCTCAATAAATGGAATAAAACCATCCCAGATACACCATCTAAGTATAT	A	G	0.60	0.38	0.45	0.37	0.48	0.95	0.78	0.48	0.64	0.42	0.38	0.006	0.034	0.002	0.141	0.0030	1		
rs918735	16	74.03	GCAACATAGCAAGGTACCCTTCTCAAAAAAATAAACATGTCAAGGGATGGTTTT[C/G]TGGTAGTTCTTATGATTGTTGATGCTTCGTATTGTAATGACAGCCACATATCTATTGT	C	G	0.40	0.49	0.55	0.45	0.57	0.59	0.28	0.36	0.57	0.41	0.50	0.015	0.032	0.020	0.001	0.0010	1		
rs1019572	16	74.28	GAAGCCATGAGACTTCCGGGAAATCGTCAAGGCATGGCCAAGAATCACTAACTAGTTGA[C/G]GAACACTAATATCATAAGAAAGATGATAAATAAAATGAACCAAGGAGTCAA	C	G	0.24	0.29	0.33	0.30	0.23	0.14	0.22	0.17	0.21	0.39	0.22	0.002	0.004	0.008	0.023	0.0045			1
rs877375	16	74.51	TCCCTTCTTCCTCCTCTGTCCTTTCCACACTCTTCTGCCTTCCCCAAAGTCACT[C/G]GGTAACTACGCTGTACACGTCAGTACCAAGCCTTCTTTAGGGGCTGGGGATTCAGAA	C	G	0.83	0.60	0.65	0.52	0.55	0.36	0.08	0.47	0.43	0.53	0.62	0.018	0.191	0.003	0.038	0.0005			
rs1046371	16	74.53	TTTTGGTTCCTTTCTTACCCACAAACCATTACTACTTGAAACTTAAAAAA[C/G]TCGCCAAGTGTAAAGGCTAAAGAGAAGCAGTTTGACGGACCTTGTGATTT	C	G	0.64	0.65	0.67	0.68	0.68	0.95	0.97	0.75	0.79	0.73	0.73	0.000	0.088	0.006	0.081	0.0029			
rs3636	16	74.72	CATAAGAACTGCCCCCCGACCCATACACACACAATTTATAGCAGGTAAAACCAACTGAAAGGAACAAAGTAATGACTTTCTTGAACAAA[C/G]TGATTACGAAAGTGAAAGGCTA	C	G	0.89	0.58	0.59	0.61	0.63	0.50	0.17	0.58	0.54	0.59	0.62	0.075	0.116	0.000	0.004	0.0001	1		
rs5842	16	75.04	AGGACCTGGCCACCCCGAGGGCTGAGCCTGGTCCCACGAGGGTGCCGTGTCCCCTGACAGGGCCAGTGCAGTTTGGTGTGTCCTCCGCCTT[A/T]CCAGGAGAAGAACC	A	T	0.83	0.42	0.42	0.39	0.38	0.32	0.08	0.44	0.36	0.49	0.40	0.095	0.088	0.001	0.006	0.0003			
rs1549660	16	76.15	ATATTCAGTGAATGTAGAGATAGATTGAGCATATGTATTCAAGAAACAGAAGC[A/G]TAGTACATACAAGAGACACATCATGGCTATAAGATTAATGATAGCTCTAAGGTCCACT	A	G	0.67	0.59	0.57	0.49	0.57	0.23	0.74	0.52	0.54	0.48	0.49	0.014	0.022	0.001	0.052	0.0052	1		1
rs1506819	16	76.25	AAGAATTGGCCAAGGATCTGAGGGAATTGTGTCTGCAGGAGCACGGACC[A/C]TTGCTCTCTGATCTTCTTCTAAATATTCCCTACTCAATTTCTAGCCTCTCTGGACCTTGAA	A	C	0.26	0.34	0.44	0.44	0.34	0.41	0.24	0.34	0.43	0.55	0.41	0.035	0.024	0.006	0.003	0.0004			
rs1002077	16	76.28	GACAGTCGTTCCCCCTGGAAAGTTCTATGTAGATAAGGCTGCTCTCATCACT[A/G]GGATCTGTGTTGTCTAGGTGGAAAGTGTTTTTTAGATAATCTTCAGCCAGTTTATGTT	A	G	0.79	0.60	0.52	0.50	0.55	0.55	0.84	0.53	0.50	0.48	0.59	0.051	0.065	0.000	0.002	0.0019			
rs1506842	16	76.29	GTTTGGGTATGTTGATATAAGCATTTGTTCAAGTTCACAGCAATGGAAGGATG[A/G]CAATGTCCAAGGAAAAGCTTTCGTGGCTAGAAGAAGTCCTTGCCAAGGAGTTAGGTA	A	G	0.13	0.41	0.49	0.50	0.45	0.50	0.16	0.47	0.63	0.53	0.42	0.103	0.070	0.001	0.000	0.0019			
rs1502441	16	76.78	TGTTTAAATCTCAAAAGGTTAGAGTTTGAAAAATTTCTGCTTGTTTTACTATTAAAGACCCTACATCCTTTATT[A/G]TCACAAACCACAAACAAGTCAACAATTTTGAAGCTTAGTA	A	G	0.55	0.48	0.41	0.49	0.50	0.59	0.47	0.18	0.46	0.41	0.47	0.006	0.001	0.055	0.010	0.0019	1		
rs1948719	16	77.18	AAAGCATGGCAAGGGAAATGTCACCACATACATCTTCACAGTAATGAACATGGTA[A/G]GCGGGGGTCCACAAACCTTCTGGCTCTCCCTGCCCAGGTATGGAAAAATGATGGATTCT	A	G	0.57	0.53	0.53	0.57	0.57	0.91	1.00	0.68	0.64	0.56	0.56	0.000	0.189	0.022	0.087	0.0001			
rs1922604	16	77.87	CTGAAACGTGACACTTCAAAAGATATTTCAGATACAGTGGGTGAGCTAGTAA[A/C]TCTTTTGCAATGAAAGGTTTGGCTCCTTCTTGCGGCTTGAGAGTGGCTAATGTAT	A	C	0.17	0.35	0.40	0.31	0.41	0.32	0.45	0.50	0.18	0.41	0.42	0.022	0.005	0.005	0.001	0.0010	1		
rs1074963	16	77.95	AATAATGATTACTCTTATAATGGAATCTTCTACAAGAAGCAGGATTTTTGTT[C/G]CCTTCTACTCTTAAAGATTTTCTGATACAGTGAAACAAGGAAGAGGTTAGCTGAA	C	G	0.26	0.42	0.38	0.46	0.45	0.32	0.30	0.39	0.32	0.42	0.39	0.007	0.002	0.001	0.002	0.0007			
rs1125671	16	78.47	TGAGGTGGGGGGAAGGGGACTTTCTTGCTTCCTCATAGTTCTCCCCATCCTGTAAAGGAGCCT[A/G]TGCTTCCCGACTCCCAGGGCCCATGCAGCATGTCAAGAACAATATT	A	G	0.24	0.54	0.52	0.48	0.52	0.27	0.62	0.44	0.61	0.58	0.55	0.017	0.001	0.016	0.060	0.0000	1		
rs1079635	16	78.64	TGTAGGATCTTGGGATACTCTGAACCTCTGGGTCTCAGTTTCCTTAGGTTGGGGA[A/G]GATAATGACTCCTAGACCTGTTAGGGGAACTGAATGAGACATGACTTTCTAAAATC	A	G	0.90	0.70	0.73	0.70	0.59	0.59	0.73	0.73	0.57	0.58	0.74	0.032	0.002	0.001	0.005	0.0004			
rs1079638	16	78.69	TGCCAACCTGCTCTTTTGTGCTGAGGAACTCT[A/G]GATATTTCAACTAGTTTACATGACTTTACTTGTACTTATAATCCTTTATAAA	A	G	0.74	0.71	0.73	0.75	0.71	0.59	0.49	0.64	0.71	0.61	0.63	0.000	0.034	0.008	0.014	0.0040			
rs1125630	16	78.73	AGCCTATATGACAATTTCTTAAACTTTTTTTGTTTTAAAAAACAAGATCTCACTTA[C/G]GATATCAGTATTTCTGATCTAGATGTATTCCTTGTCAGTATCATTTTCATTCCACAAT	C	G	0.19	0.72	0.71	0.77	0.63	0.41	0.79	0.78	0.89	0.67	0.69	0.123	0.008	0.007	0.036	0.0008			
rs1424110	16	78.95	ATCTCATGCTGGACTATCTAAGCAAGCATGTAGGAAGAGTTTTCTTCCTGCT[A/G]TGGCCATAGAGGTTTGGTTGTCACTATGAAGAATGGGCCTGTGTTTTTTTTTTAAAA	A	G	0.86	0.72	0.73	0.66	0.73	0.55	0.77	0.65	0.75	0.76	0.78	0.011	0.000	0.005	0.018	0.0000			
rs424074	16	79.01	TTTTTTTGCCTATTACAGTTGGGATTAGACTAACCCTCCCTAAGCAGCTATT[A/G]AGCCTCACACTCCCTGGACAAAGACATGAAAACTCTGGATGATTTGGAAGGGAGATC	A	G	0.57	0.64	0.74	0.56	0.71	0.27	0.34	0.33	0.71	0.63	0.64	0.014	0.077	0.093	0.106	0.0076		1	1
rs728929	16	79.45	CCTCATCTCTACTATTGCAGCAACCAAATAGCGAGTAGAGCTCCTGCCACCCAGTGTGCTCTTCCACTGCC[A/G]AGAAATCCTTCTGAAATGCTAATCTGGGTATATATTTCCC	A	G	0.55	0.37	0.41	0.54	0.54	0.36	0.89	0.52	0.21	0.41	0.44	0.007	0.099	0.009	0.004	0.0003	1		
rs1424234	16	79.46	AGGAATAAAGGTTTGAGCTCAACATGCAGTGGAATCTTGTAACAATTGCAGCTTTT[A/G]TGCAGCACTCATTCAATAGAAAATAACATGAGGCATACATAACAATTGTGTAGCAG	A	G	0.64	0.45	0.47	0.62	0.61	0.68	0.86	0.68	0.29	0.57	0.54	0.012	0.076	0.022	0.022	0.0013			
rs1125733	16	80.23	ATGCATATCAGAGGGAGTCCAAACTCAAGTCAGTGAGTTTTTTTAAAAGCTTGT[A/G]TTGTATATTTGATGTGCACCTAATTAGACCAGAGCATCTTTAATTAAGTGTGTCAGC	A	G	0.90	0.45	0.52	0.61	0.63	0.50	0.93	0.79	0.64	0.56	0.56	0.118	0.128	0.070	0.001	0.0031	1		
rs2059280	16	81.11	GTAAACTTAAGTAGGGTAAG[A/G]CAATGGGGAGTGTGTGATGGAGGGGCAAGCTGTGATGTTAAATTAGGGTCAGGAAATCCTTACTGAACAGGAGATTCGAACAAACTTG	A	G	0.75	0.72	0.79	0.72	0.79	0.95	0.99	0.82	0.75	0.78	0.74	0.000	0.079	0.007	0.049	0.0006	1		
rs1037973	16	81.18	ATTGTTGTAACCACTGAGCAGTATTCTTATTGTTGCAGTAATATGTTTAAATCCT[A/G]AGGGAAATTGCTGTGCTAAACGTAATTTATTAGAGTAGTTCAGTCGTGCTACCAGAAG	A	G	0.38	0.39	0.31	0.34	0.32	0.23	0.28	0.25	0.42	0.35	0.35	0.000	0.013	0.010	0.017	0.0018			
rs2012502	16	81.51	TGGACTTTAGCTAAATGGGTCAACTGGACCTAGTCTGGAGGTGGAG[A/C]ATGTGTGTTTGAGAAGGAATGTGGCACATGGACACCAGGTCTTGCCATGTCCAACCAT	A	C	0.64	0.45	0.39	0.38	0.34	0.09	0.11	0.20	0.32	0.32	0.40	0.041	0.043	0.016	0.047	0.0002			
rs11443	16	81.81	CCTGTGGAATGCTGCCTGGATATTTTCAGAAACTGCTTCAGGATGCTGTGTAAGA[A/G]TAATACCCACCGTAAAATATCGCTGTGTCCTTAACCAAGGAACGAAG	A	G	0.81	0.63	0.58	0.57	0.59	1.00	0.82	0.68	0.39	0.61	0.61	0.040	0.056	0.012	0.216	0.0008	1		
rs967955	16	81.93	GTCTGTTTCCACAGCCTCCAAG[A/C]TATTACATTTCAAGAATGCTTTCCACAATATAAATAACAGTTTCCACTATTAGTCTTCAGACAGTAAGAAATAAAGTCAAAGCACTGGCTT	A	C	0.19	0.48	0.53	0.50	0.50	0.32	0.13	0.29	0.32	0.48	0.42	0.068	0.102	0.018	0.025	0.0056			1
rs2967305	16	82.10	AGTAGTCGTATTTACTGTCTGTGCATTTCCTTCAGCACTGTGTCTTAGAGACTT[A/C]CGTGTTGGGACCAACAAACCTACCTTACTGGTTACGTACTGCATAGAATTTCATTAT	A	C	0.19	0.78	0.78	0.82	0.82	0.64	0.35	0.61	0.88	0.76	0.81	0.167	0.078	0.004	0.008	0.0009			
rs725514	16	82.63	AAATAGCCTTATGCCAATATTATAAAGACAATTTCCTCTTGGAAAGAACACTTACTC[A/C]TTTTGCCTTAAACAGTCATTAATGTTCCAAATTCCCCCTCAGAATTTCATGTCAATGC	A	C	0.78	0.53	0.62	0.54	0.64	1.00	0.85	0.66	0.58	0.70	0.59	0.011	0.015	0.001	0.129	0.0019	1		
rs7190151	16	82.89	AAAAGGCAAAACCTCCTTGACCTTCACCCAGGCCACAGATTGCTGCGGGATCTCA[A/G]TAAAATAAATTTGATGAGGCACTTCAGCTTCCTTGAAAAGGATGAGACAGATAT	A	G	0.81	0.97	0.96	0.93	0.98	0.95	0.99	0.86	0.96	0.95	0.98	0.030	0.009	0.013	0.002	0.0012			
rs1424189	16	83.11	ATAGATCCACAGCCTTCCAGAAGTCCCTCTCTCCTTCATTCCATCTTCTCAGAGC[A/G]ATTTCCCAAACAAATATCTTATCTCATCATTTCCTCTATTTAGAAAGTCCCCAATGGC	A	G	0.36	0.32	0.48	0.40	0.43	0.27	0.41	0.21	0.46	0.38	0.47	0.007	0.001	0.024	0.017	0.0010			
rs723919	16	83.12	TTTAAAAGGCTTTATGAAAACATTAGTGCTGAGCCTGGGTGATTTATGGCACA[A/G]ATCCATGGAAAGAACCTTTCCTAAGCCCACATTTCAGCTAGGGCTTGCCTGGGAGAA	A	G	0.31	0.42	0.59	0.50	0.52	0.50	0.42	0.35	0.54	0.50	0.55	0.027	0.004	0.010	0.001	0.0009			
rs1387370	16	83.31	CTTCATCAAGAGATTTTCCAGTTATTGAACTTAAATAACATTGTTCTAAACCCCAA[A/G]TCCTCTAAATTCCTGTTGTCAGTGAAAGAGGTACCTTCTAATTTGGAAAGAATATCTA	A	G	0.64	0.44	0.46	0.39	0.45	0.18	0.41	0.50	0.46	0.47	0.49	0.017	0.005	0.000	0.050	0.0004	1		
rs41103	16	83.62	ACCCACCCCTACCCGCCCCGGGAGCCCACATGGCTCCTGTCCTGTTCTGTCATCT[A/C]CCTTCATGAAGGTGGCCAGCGTCTGGAGTTTAGCCCAGTAGGTTCATTGTGCCC	A	C	0.40	0.37	0.39	0.36	0.45	0.23	0.07	0.35	0.32	0.40	0.30	0.001	0.057	0.002	0.009	0.0048			1
rs999783	16	83.87	TGGTCAGGTCATGGCTGTGTGTGCTTATCTGGCTGGGCTGGGCCACTGAGAATGCAT[A/G]TCTTATGAGAATAGGGGAATCTGGAACATGTGGATTTACTTTGACCTAAACTC	A	G	0.14	0.22	0.23	0.29	0.18	0.45	0.48	0.24	0.46	0.29	0.26	0.006	0.025	0.003	0.023	0.0012	1		
rs39552	16	84.03	CCAAGGTGTGTCCTGCTGGGAGAAGCCAGGATTTGACAGAATCTGCAGGATTAA[A/G]CCCGTGAAGGAGGGGCTGTCTTGCCTGAATGGCTCAATGGCTCCTCATCCC	A	G	0.83	0.53	0.50	0.61	0.46	0.64	0.71	0.56	0.65	0.57	0.56	0.087	0.046	0.014	0.028	0.0017			
rs922450	16	84.21	CCTTGTTTTTAAAAAGCCAATAACAAGAAAACTGTACTTGACTGCTGGTAGAGGAAAC[A/G]GGGAGAACAGAATTGATTTCTAGATTCTCTTGAATAGTGAAAGAAAATCTAGCTCATT	A	G	0.24	0.52	0.52	0.55	0.50	0.36	0.86	0.65	0.46	0.58	0.55	0.052	0.055	0.005	0.020	0.0004			
rs247892	16	84.26	CTCAGGAGGTCTGGCATGGAACCTGAGATTCTGCATTTCTAATAAAGTCTCGCGTG[A/G]TAACAACCGCTGCTACTCCAGGGACCCTGCTTTAAGAACTTCTGTTTTAAACAAA	A	G	0.75	0.63	0.67	0.65	0.64	0.91	0.84	0.78	0.64	0.71	0.66	0.004	0.013	0.004	0.040	0.0001			
rs454087	16	84.31	TTAAAATACAGATTTTTTTGTCCTGCCTTTGGTTATATTAGCTTGGGCACTA[A/T]GTTCTCAAACCCAGACTTTGTGACAATTATGGGTGTGTTTCAGTAGGTCATGGGCGTCACC	A	T	0.24	0.58	0.59	0.67	0.59	0.55	0.52	0.73	0.64	0.50	0.50	0.062	0.001	0.021	0.000	0.0057			1
rs2052904	16	84.42	CTGCTGTGTGGCCCATTCTTGGGAACACCACACTCTCTTCCTGGGTGACTTTG[A/G]TCACTCATCCCCCCAACACATATAAACTGAGCACTCACGGTCTGCTGGGCACTCGTG	A	G	0.55	0.57	0.56	0.48	0.54	0.94	0.44	0.54	0.50	0.56	0.51	0.000	0.002	0.001	0.125	0.0001	1		
rs889613	16	84.48	TCCTGCTCAGAGCCAGGTCTGAAGTCCCACTTGATAGCCTCATAAAATGGCAGAAC[A/G]AAAATACCACCCTCAATTGCGTGCTAACCACAGTTGATAAAATAATCTCTGTGCATTT	A	G	0.57	0.66	0.65	0.66	0.64	0.55	0.96	0.54	0.64	0.70	0.74	0.014	0.042	0.019	0.023	0.0011			
rs873857	16	84.48	ATGAAAGGTTCCGAGAAGTCTCAACCCAGCATTTCCAAACTTCTTGCCTAAAA[C/G]TCGTTTGTCTCATACCTAGTCCCAGCCCACAAAACATGTTTTGCAGAGACTTCTT	C	G	0.40	0.70	0.71	0.63	0.57	0.64	0.44	0.67	0.54	0.70	0.63	0.059	0.050	0.001	0.003	0.0023			
rs1874014	16	84.64	CACTGCCTGCTCTATAGCTGGGCACTTTTTACAAGGAGTTAGTTGAGGCTCAG[A/C]ATTATTAAGGTGTGTGCCCAACATGACACAGCCAGGGCGGGGTAGAGCTGCCGTC	A	C	0.45	0.60	0.60	0.63	0.63	0.86	0.94	0.61	0.50	0.53	0.60	0.014	0.114	0.001	0.050	0.0003			
rs751339	16	84.78	GCAACCTCTCTCAGCCTCAGTTTTTCCCCATCTATGAGATGGTGATGATTGC[A/G]CAGATTGATTAAGGGAAACGGTGCGTGTTAAATCAGCACAGGACAGGCAGGTTTTCTCATAAA	A	G	0.12	0.48	0.50	0.46	0.55	0.27	0.08	0.31	0.64	0.38	0.47	0.094	0.111	0.025	0.028	0.0004			
rs1566463	16	84.91	GATGAAGAAGCTGAGGCTCAAAGAACCATCTGGCTAAGGGCATACAGTCAGCTG[C/G]CGGGAGGTGGAACTGGATCCAGGCCCTTCCAGGTCCTGCCCCGAGCTGCCCAAA	C	G	0.62	0.66	0.65	0.72	0.71	0.90	0.76	0.74	0.82	0.65	0.69	0.003	0.000	0.003	0.026	0.0009			
rs998624	16	85.14	CAGACTTCCAGAAACACTCAGACTTCCCAAGCCTGGGGTAAGAAGGGAGGAGGGA[A/G]ATAGAGAAAGACAGGGAAGATGTGTCCTGCCCTGGGCCCCAAGGGCTCATGCTGGC	A	G	0.31	0.28	0.29	0.28	0.16	0.18	0.00	0.28	0.32	0.27	0.27	0.000	0.101	0.006	0.005	0.0007	1		
rs408126	16	85.56	AACCCAGTTCCTTTGGCTGGAGTTGTAGGAATTCTAATCTGCTGCAGAAAGAAA[A/G]TGGCTTTCCTAAAGAAGGAAGAAAGCACCATGTGTGTCGTGAGCCTGCTGGGAG	A	G	0.33	0.46	0.47	0.50	0.41	0.10	0.10	0.25	0.32	0.46	0.57	0.018	0.151	0.041	0.113	0.0018			
rs7197068	16	85.82	CTGTGGTAGGCAGAACGTCCTACCTCTCCTCCTCCGGGATGTTTGTCTCTTAT[C/G]TCTAGGACCTGTGGATATGTTACATTATATGGCAAAAAGAATTCATATAATTAGGGT	C	G	0.95	0.72	0.73	0.73	0.64	0.82	0.99	0.88	0.75	0.84	0.75	0.054	0.089	0.055	0.008	0.0001	1		
rs889593	16	86.30	ACGCACACCCAGGAGTCTTAGCCTCAACCCTGAATTCCTCTTTTGACAAATGGAAA[A/C]TCATTCCACTCTTGCCGATGCATCTTCTAATCCAGAGCAGAACATAATCCCCAT	A	C	0.19	0.56	0.52	0.57	0.48	0.41	0.26	0.32	0.58	0.53	0.49	0.037	0.017	0.015	0.001	0.0000			
rs299956	16	86.58	ATCCTGAGAAGAGAGGGGTGACTTCAGCGAAGGGGCACATGCTGAGCCTCCTCTC[C/G]TGGGGAGAATTAGACTTAAGAAGCCACTTGCTGATAAGGGTTAATCATCGGCTA	C	G	0.48	0.21	0.32	0.25	0.20	0.18	0.44	0.36	0.36	0.21	0.27	0.007	0.004	0.000	0.018	0.0033	1		
rs2076962	16	86.94	AAAGACTGACAGGCCGGCGCAGAGGGCCACCTACAAGGACCAGGGGCAGGCA[A/C]GTTAGAAGCTCCCATCTTCTGAGTCCTCACTACCTGCCAGCACTTTCTTTTTTCTTG	A	C	0.14	0.35	0.30	0.32	0.29	0.32	0.50	0.31	0.43	0.30	0.30	0.016	0.016	0.001	0.001	0.0001			
rs750740	16	88.55	GCTACAAGACTTGGAGGTCCGGCCCCGGGAATCTCTGAGCTATGGCCCCCTC[A/G]CACGGACAGGCATGAGCTGGGGCCTGCGACACCCAACGTTGACTGCTCAGACCCT	A	G	0.64	0.47	0.48	0.45	0.41	0.78	0.66	0.43	0.57	0.42	0.38	0.008	0.008	0.001	0.001	0.0076	1	1	1
rs463701	16	89.34	TGGCCAGGGGCCCCCGCGAGCAGCTGCCGTGTGTCTGTTGTAGGGAATGG[A/G]GCTGGTAGCTTTAGTTGTCCTTGGTGTAGAACTTTGTCTGGCCCGGGTACAGGAAG	A	G	0.60	0.62	0.55	0.56	0.55	0.41	0.74	0.46	0.54	0.47	0.44	0.003	0.026	0.002	0.005	0.0059	1		
rs452176	16	89.40	CCTCTGCCGTCGCTATTGGGGATGGTCTTGGGTGAGGGTCTCCCTGGTAGGT[A/G]TTCAGAGCCACCACTCTGGGGGGCCTCTGCTTGTTCCTGCCCCAGGCCCAGTGGGAC	A	G	0.21	0.52	0.59	0.62	0.57	0.30	0.75	0.28	0.61	0.58	0.63	0.076	0.020	0.058	0.036	0.0010			
rs1006547	16	89.59	ATCATTCTTCAGTGAAAGAAAATGTTTCAGTACATAATGATTGTGCAGAGAGGAGA[A/G]CTGAAGGAAAATGAAAGAGCTCTGGGAAAGCCTCATGCTGCAGAACTTGAGAGTA	A	G	0.64	0.63	0.68	0.65	0.73	0.18	0.09	0.57	0.61	0.74	0.72	0.003	0.202	0.017	0.151	0.0020			
rs1800330	16	89.60	ACCCAGCATAACTCACGTCATGTTCCTGCTGGCTGCCCTGCCTTGCTCTGTGACT[A/G]TGTCATGGTTCTTAACTAGAGCTGGGTGGCGCGTTTGATGGCCCAGGCATTCGAGAG	A	G	0.55	0.62	0.67	0.63	0.73	0.18	0.09	0.46	0.57	0.70	0.69	0.014	0.202	0.040	0.151	0.0006			
rs870856	16	89.83	GCAAAGCCTCTCGTGAGCTGAAACCCTAGGTCCCAGGATCCAACAGACCGAGTCT[C/G]CCATTCCAGGATGAGAGTCCCCTTGACGCATGTTCACTCTTCTGGGACCGTGCC	C	G	0.26	0.26	0.30	0.35	0.29	0.14	0.14	0.43	0.32	0.44	0.43	0.000	0.012	0.026	0.013	0.0177		1	1
rs8577	16	89.85	AATATACTTATGCATAGAGAAAATCTGGAAAAATATATGCCAGCTATAAAGTGGTT[A/C]TTGCTCATGGGATAAATTGTGGGAGACTTTTCCCTTTTTGCTTCTCTGTGTTGTGA	A	C	0.38	0.33	0.23	0.30	0.25	0.77	0.50	0.40	0.43	0.22	0.20	0.025	0.084	0.050	0.221	0.0014			
rs869048	16	89.86	GCTTGCTACCTCAGTCTTCCCATGTGTAGAAGGTGAATTATGGTGCTGGCCCAC[A/G]GGGTGTATGGAGAATGAAAAGAGTGGAAATAATGTCCAGCGTACTGCCTGGCACA	A	G	0.63	0.40	0.28	0.38	0.32	0.50	0.81	0.42	0.35	0.22	0.23	0.076	0.185	0.025	0.042	0.0042			
rs1609550	17	0.09	CACCTGTGTCATTTTAAAACATCTTTATACCGTATTTTTACTGTACCCTTTCTAT[A/G]ATTAGCTACACACATAATTCCACGGTGTCGCAGTTGCATCGTGCACAGGTTTGT	A	G	0.26	0.52	0.51	0.55	0.57	0.68	0.60	0.56	0.39	0.48	0.49	0.030	0.008	0.007	0.025	0.0002	1		
rs741677	17	0.45	GTCAGTCCTCCACTGCTGGTTGTGGTTCTGAAGAAAGGAACAGGAACAAGTCAGC[A/G]TGGAATTTCTAATTTGCTTCCTGAACTGGAAACTACATTATTTTAAGATACAGAAA	A	G	0.24	0.58	0.58	0.62	0.55	0.86	0.33	0.47	0.79	0.53	0.60	0.062	0.038	0.019	0.039	0.0000			
rs7813	17	0.63	GGGGTTTGGCGCCGTTCTTCAGGGCCGATGCCCTCAGCAATGGACTTTAAGAGC[A/G]TGCTCGTGTGCCCTCTGGAGCAAGGAGCTGACAGAAGGGTTGGTCTTGCTCAAA	A	G	0.90	0.59	0.58	0.65	0.52	0.73	0.73	0.65	0.64	0.68	0.55	0.069	0.014	0.002	0.015	0.0008			
rs1045491	17	0.63	GTGCGGAGCGAAAAAGCTGGGCTTCAACATGGCAGGTCTGCAGGGGTCAGCCC[A/G]AGCAGCCTGGACTTTACAGTTATGTGAAACTGTCCACAAAAGTCATGGCAGATA	A	G	0.07	0.29	0.26	0.24	0.30	0.09	0.06	0.22	0.14	0.24	0.30	0.034	0.062	0.005	0.033	0.0012			
rs910924	17	0.64	CGCGCTCCTGCCGCGCCCCCCCCCTCCACAAGCAGGCTTGCGGATCCTAGAC[A/G]CTTTGACGCCTGTCCCCGAACCCTGAGCCCCAGAAATGGTGG	A	G	0.07	0.29	0.28	0.28	0.32	0.09	0.04	0.24	0.14	0.24	0.31	0.046	0.090	0.010	0.044	0.0005			
rs592294	17	0.69	GCTTGGCCGATGCCCACCACAGCACCTGCCTCCCTGCGTTCTGGTAGTGATGGCC[A/G]TTGATTCAAGGACGTACCAAGGGGCGAAAAGCAAGCAGGAAGTCACCTCGC	A	G	0.48	0.45	0.46	0.51	0.43	0.23	0.10	0.45	0.39	0.49	0.41	0.002	0.062	0.019	0.013	0.0009			
rs11062	17	1.89	CTGGAGGAAACTGCTCAATAAAACGGCTCCATATTTACTTCTCTGGTCAC[A/G]GTTCATCTCCACGATTTTAACAAAGGAGTCGAGGAAGCTAGTATCTGTA	A	G	0.36	0.37	0.31	0.35	0.52	0.45	0.83	0.42	0.25	0.23	0.28	0.007	0.203	0.021	0.030	0.0000	1		
rs216219	17	2.35	AGTTTTGAGTATTCCTTTATTCCTTGGATGGATCCGAGTTCTTCCTCTCTGC[A/G]TAAAACTAGTGATCCTAAGCCCAACGTAATGCCTACACGCCAGAGCAGATGGATGCCTC	A	G	0.50	0.34	0.33	0.30	0.36	0.27	0.23	0.33	0.29	0.38	0.40	0.023	0.008	0.006	0.002	0.0078	1		
rs216223	17	2.36	TGAGGAATCTGTATTTTTTATAAAAGCTGTTCAGGTGATAGACATACACTCAGG[A/G]TTGTGGAATTAGCAGTACTGATCTCACTCATTTAACTGTTGAAAGAACAGCCTAGAAC	A	G	0.50	0.66	0.67	0.70	0.64	0.73	0.77	0.69	0.73	0.62	0.59	0.023	0.008	0.003	0.002	0.0084			
rs1984749	17	2.46	GCCAGCGAAAACTTCCACACAAGGAAAGTTTCTACTCGCAGGGGCCTACTGCAT[A/G]TCCAGCTTCTGGACAGACAGGTTTAGGAACAACCACAGCAAACATCAACAAAGCA	A	G	0.74	0.63	0.66	0.57	0.73	0.64	0.81	0.56	0.71	0.57	0.51	0.003	0.011	0.015	0.002	0.0125		1	1
rs405923	17	3.45	TATATTTATAAAATGTATCAAAAGAAAGGCTAGATTAATATTCCAGGCATACAAG[A/C]AAAGGAAGAAGGCACAAAGAAATATCAACAGATTTGACTGCATAAACATTTTTTAAAA	A	C	0.21	0.45	0.50	0.48	0.55	0.50	0.04	0.33	0.32	0.44	0.48	0.051	0.175	0.010	0.001	0.0007	1		
rs1068692	17	3.48	TGGCCCTGAGACAAATTATAAGCCAGGGAGTGAGATTACCTTGAGACTGGTGT[A/G]GGCTAATCAGCCTCTGCAACTGGTCCTGAACATTAGTGGGCCACATGATTCTTCCAC	A	G	0.10	0.39	0.43	0.44	0.39	0.00	0.03	0.18	0.32	0.39	0.39	0.092	0.158	0.028	0.202	0.0016			
rs395357	17	3.64	GAGGCAACAACATCCTTCACGCCCTGGTGACCGTGGCCGAGGACTTCAAGAC[A/G]CAGAATGACTTTGTGAAGCGCATGTACGACATGATCCTACTGCGGAGTGGCAACTG	A	G	0.40	0.50	0.52	0.55	0.43	0.27	0.01	0.46	0.50	0.53	0.52	0.004	0.216	0.002	0.040	0.0002			
rs222775	17	3.79	AGAGGAGTTTATACAAGACAAAGAGGCGGAAGGACATTACGGAGGGAGGAA[C/G]AACCTGTCACATCTGGGAAATGCCCGCAGGGCCTGTGCGGGAAGAGGGAGATGCGC	C	G	0.81	0.46	0.47	0.51	0.43	0.41	0.60	0.47	0.54	0.42	0.45	0.051	0.003	0.002	0.005	0.0001			
rs1319344	17	6.37	AGAAGAGTCAAACTTTTGAACCCCTACTGTATCCTATGATGAAAATGTTCTCTT[A/T]CATGGATGTGATTTAATTCTTTATTGGTTATATGAACCAGCTCAGAGACTAGAATCCTTA	A	T	0.40	0.38	0.38	0.38	0.27	0.55	0.69	0.43	0.54	0.32	0.30	0.000	0.035	0.005	0.013	0.0019	1		
rs1443417	17	6.69	CCCTCTTTGTCCCACCGGGTATGC[A/G]GCACAGCATCGGTGACTACTGTAGTCGTTTGAGCAGTACCTTCGGATCATATCTCATGAGGCTGTAGGCTCCTTCAACCCGTGGC	A	G	0.29	0.46	0.46	0.41	0.45	0.14	0.09	0.33	0.46	0.43	0.53	0.022	0.113	0.019	0.090	0.0015			
rs1848550	17	6.95	GGTGTCTTGTCTTTTGCCATTTGTCAAGCACAGCAGCTGAGGTTGCTGAAGC[A/G]GGTGACTTGCTACCAGAAGCCGAACCCACTGCAAGAAGCTCTCCTGGAAAGGGGCATCAAG	A	G	0.53	0.55	0.52	0.56	0.55	0.23	0.40	0.40	0.57	0.49	0.52	0.000	0.081	0.011	0.044	0.0001			
rs222850	17	7.34	CGTCACGTGATCCCTCTCCCAATCACGTGACCGTCTGACTCCACCTAGTGCC[C/G]CCGCCACATGATCGCTGCCTTCAGCAACGGTCTCCCAGCCCCCAAACGTGTACCCCA	C	G	0.93	0.64	0.63	0.67	0.64	0.73	0.59	0.67	0.85	0.73	0.66	0.077	0.001	0.025	0.020	0.0000	1		
rs4562	17	7.36	TCCCAGGTCACACATACTCCTTGAGGAAGCTGGGTCCGGGTAGGTCAAGGAGG[A/G]CACGGTACCAGCCTTGCCTCCCATTCCACCTCAGGACTTCCTGGAGAGGACGTGCACCT	A	G	0.10	0.38	0.37	0.35	0.36	0.23	0.40	0.32	0.14	0.29	0.35	0.066	0.001	0.029	0.031	0.0000			
rs9217	17	7.56	GACATTTTTTTTGGGAGAATGAGATTGTAGACATTTTTTTTTCTTAA[A/G]TGTAAGATCAAGGAAAATAGCTTCCAGAATGTGGTGGTCTGGGCAACAAAT	A	G	0.40	0.58	0.65	0.60	0.64	0.50	0.69	0.50	0.75	0.71	0.69	0.030	0.003	0.007	0.012	0.0003			
rs858526	17	7.70	CTGTAGGAATCATGGTGGTAGAATCTCACAGTCCCAGGCACACGACCCTTTGC[A/G]TAACTTAAATAAGGAAAACTGTGTGATATTAATAGAAAGCTAGACCACTCTGGAGTG	A	G	0.76	0.38	0.41	0.50	0.45	0.50	0.70	0.34	0.64	0.37	0.44	0.069	0.056	0.008	0.003	0.0001			
rs1565816	17	7.97	ATCTTGGCTTCCAGGATAAAGATCAGTCAGGTTTCTAATTCTGTTGTCATTCTT[A/C]CAGCCAAACCGTGTACTCTACTGACCCATGATCAAGTGGCTGTTCTCCCAACAGCA	A	C	0.62	0.76	0.81	0.83	0.79	0.23	0.48	0.66	0.64	0.78	0.81	0.028	0.061	0.011	0.192	0.0002	1		
rs1391766	17	8.56	TCTAAGCAGATATGGAGGCCAAGACACAGGTCTCATGTTTGCCTGTTTCA[C/G]CTCATCATAGGGGTTATTTTCTAACCATCAGATTAAACCTGCATTTTTAAGGTTAAAATA	C	G	0.40	0.43	0.38	0.40	0.45	0.41	0.41	0.52	0.46	0.36	0.35	0.000	0.000	0.009	0.000	0.0003	1		
rs422173	17	8.67	CCCCTAAAAGACATTACACACACACAAATAAAAGCAGATGTACGTTAATCTAAT[A/G]TCTTTACTCAGTCATCAGGGGAAAAATATTCTAAAAAGAAACTGCATCTTTTCCTCAG	A	G	0.48	0.43	0.38	0.40	0.45	0.45	0.43	0.52	0.50	0.36	0.36	0.004	0.000	0.007	0.001	0.0002			
rs2043155	17	8.83	CTGCTTTGGGGACAAAAGCACAGGGCCTCTGCTTTCTGGGAGGCAGACTGACGA[C/G]GGGGAGAGACCAGGCTCAGGAAGAGCAGACCCAGGGGTGAATCCTGACCCTGTC	C	G	0.14	0.18	0.19	0.26	0.13	0.05	0.01	0.10	0.14	0.25	0.27	0.003	0.045	0.006	0.023	0.0060			1
rs1997095	17	9.18	TCACCTCTGTCAGCGGTCATGAGGGAGCCTGCTCTCT[C/G]TGGGGACAGATCTATTTTGGTGTAACACGGATCGTGTGAACACTGATAACCTCAGACAGAGTGATTTCTTC	C	G	0.71	0.55	0.58	0.54	0.68	0.55	0.49	0.55	0.68	0.51	0.59	0.007	0.004	0.000	0.001	0.0003	1		
rs726598	17	9.26	CCAACTCTGCCCATGTGGCCTGGACATGTGGAAATGTGTCAACAATCATGACAGATCTGAGAGCAGT[A/G]CCTGTCCCAGTGCGCAGGCAGCCAATGGCTGGGGCCTGCAG	A	G	0.31	0.38	0.45	0.40	0.38	0.45	0.47	0.50	0.46	0.47	0.53	0.012	0.001	0.000	0.000	0.0056			
rs1047365	17	10.02	GTGAGGGCAGAATTCAACAAGCACAACTCACTGGTCTTCAGTCAACGTGCTAGAA[A/T]CCGATGACTTATTAATCTCTAATTTTTGGCGCCTCTTCATTGAATGAGAATTGCT	A	T	0.60	0.55	0.50	0.49	0.54	0.41	0.56	0.60	0.71	0.51	0.52	0.002	0.003	0.002	0.004	0.0000	1		
rs758140	17	10.57	TCTGTAATTGATCAGAGTTTTAACTATTATGTAAAGAGTCCTCACTATCTGTAAT[A/C]CTATAACTCATCTATGTATCTTGGTCTCATTCATATTTTCTTCTTTCATCTTTCAACTTCA	A	C	0.24	0.51	0.53	0.46	0.45	0.14	0.17	0.40	0.36	0.52	0.45	0.046	0.103	0.013	0.111	0.0011	1		
rs1266160	17	10.66	AGATCTTACCAAAGCAAACGATTCCAACTTGGCATTCAGGAAATCAATAAGTG[A/G]AATAAAGAAACTGAATTTCTGGAAATTATCTTCAATTGAATCGCTGATGTTGGCTGA	A	G	0.33	0.42	0.39	0.49	0.54	0.91	0.83	0.42	0.50	0.41	0.44	0.000	0.138	0.009	0.183	0.0017			
rs995362	17	10.68	CTGTTCTGCCAGAAGCTTTGAATGTTCTTCCTGCTTTGCAGGCAGTTTCTCCCTAAAGAACTACTGAATATTATGTCATACTCCCA[A/G]CAGGGTAATATATGTGATGTGCACTG	A	G	0.24	0.43	0.39	0.49	0.50	0.77	0.81	0.48	0.62	0.39	0.43	0.012	0.122	0.007	0.094	0.0014			
rs1860300	17	11.34	AGAACTGCTTTATATATGTCAGGAAGGGAAAAGAAAACCCCACAAAACTTTCAAT[A/C]ATTTGATGATGTAATACAATCTTACTCTTAAGATAGTCAGTTGATAACCAGATCAGTT	A	C	0.38	0.57	0.57	0.52	0.45	0.23	0.90	0.44	0.43	0.61	0.61	0.014	0.077	0.008	0.048	0.0025	1		
rs205060	17	11.49	GCCTGCCACCTACATGGGCCAATGGTAGCAGGCCCACAAACAAGCATTAAACAG[A/G]GATATGAGTGAGTCTCCTGAGGGCAAATGGTGGTGCTACCTTAGAGGTGGACT	A	G	0.24	0.48	0.41	0.34	0.29	0.68	0.51	0.43	0.64	0.36	0.37	0.019	0.004	0.000	0.037	0.0001			
rs2193112	17	11.64	GTTTCCAGTCTTCAGGAATTAGTACTGATAAACAAAGAAACAGCTCAGATAC[A/G]GTTAGTGAGGGTTAGGATGGCAGCTCTTCTGTTGGTCTGTCTGGTTCATCATTACGCAG	A	G	0.62	0.39	0.44	0.45	0.34	0.55	0.67	0.54	0.46	0.48	0.40	0.020	0.018	0.000	0.003	0.0012			
rs1984661	17	12.85	GACAAGACAGCCTGGCTAATTCTTACTGATGGAAATGGATGACTTTTCTCTC[A/G]GTTGTCTGCTGGTACCCGCGACTTGACTTTCCCAAACTGAATGAACTTGCCACTGTCC	A	G	0.52	0.48	0.56	0.60	0.57	0.86	0.77	0.65	0.75	0.47	0.52	0.001	0.033	0.003	0.058	0.0000	1		
rs2215054	17	13.04	GGGAACTGAATGAGATTAGGAAATATTTTTTAAGTAG[A/G]AAAATAATGCAGGTATGGAATCGATTTTAGTTAACAAACAGAATTGACACTGCAGAAGGT	A	G	0.74	0.74	0.68	0.71	0.77	0.73	0.95	0.73	0.82	0.71	0.69	0.007	0.082	0.011	0.007	0.0000			
rs1468191	17	13.34	AAGGGTTTACAGAACAACTTAAAAATTAGACTGGTCCCAAGTTATGGAATAAACA[A/G]AATTTTCTCTGTAAGGAAGTGGGATAATTACTTTCCAATTTAGGAAATTACTTTGGC	A	G	0.50	0.34	0.41	0.37	0.41	0.77	0.89	0.43	0.39	0.43	0.30	0.006	0.165	0.001	0.078	0.0058			1
rs1558120	17	14.01	GAAAAATTGATATTTTGTTTCTCCCCTGGCCTCTCCATGTTCTCATTAGAAAAA[A/G]GAAAAAAAAAAAAAGAATGAACTCTCTTCTTTTCTTAGTTTTGGACTTCCTTGACCAAAGC	A	G	0.74	0.62	0.55	0.55	0.70	0.91	0.92	0.69	0.64	0.52	0.58	0.011	0.066	0.006	0.064	0.0003	1		
rs983424	17	14.35	ATGAACTTGATGAAGAATTGTAGTATCAAGTTGGGAATTAGGG[A/G]CAGTTAGAAAATGTCGCAGACTCAAATCTGCTATTGAGGGAAGCGCCGTTTGATTCTGGTC	A	G	0.50	0.46	0.53	0.52	0.55	0.50	0.14	0.65	0.61	0.49	0.48	0.001	0.107	0.001	0.001	0.0024			
rs917310	17	14.45	AACCTCAGTTTTTAAACCAGAATGCTTCTACCATAAAGAATTGTGATTTCAGTT[A/G]TACTTCCATCAAGGAATATGTGGGAAGATACATATATTGTCAAATGGTTGGGATGC	A	G	0.86	0.43	0.39	0.41	0.41	0.45	0.65	0.43	0.32	0.45	0.45	0.128	0.035	0.001	0.003	0.0021			
rs612066	17	14.61	TTTCAAGACCCCTTAAGAAAGAAGGGTCCATTGTAAACCTTTGAAACAGAAC[C/G]CAGACATGCTGAGAGCCTTGGTGATGTTTCAAGTAATATGAAACTGCTTTTCATCACG	C	G	0.62	0.54	0.48	0.59	0.43	0.27	0.66	0.39	0.68	0.48	0.54	0.008	0.008	0.014	0.031	0.0007	1		
rs2058237	17	14.68	GTTGAAAACATCCATGCACCAAGAAATGCTGATGGAAGAAGTAC[A/G]TGTTGTTCTTATTCCTTTTTCTGTTGGATTTATTCTTCCTTCTATCTTTCATTTTATTTGGTGTTATAAGTTC	A	G	0.31	0.43	0.48	0.40	0.50	0.68	0.78	0.57	0.46	0.58	0.48	0.019	0.050	0.003	0.020	0.0004			
rs917541	17	15.12	AGAAAGCGCTCAAAGAGACATAGGTGTTCATGGTTGGAAGCTGTCAGATTGAT[A/G]TGTGCAGAAATGGTGACAGCTAAGATGATACAAGGGGGAAGAGTACCAACAGCGTT	A	G	0.52	0.51	0.49	0.40	0.55	0.50	0.30	0.54	0.58	0.52	0.54	0.003	0.009	0.014	0.001	0.0016	1		
rs231018	17	15.37	CCCTGTCTAAGGTCTTATAGACCATAAAGGCTCTCCTCGTTGTATTACTCCAAG[A/C]CCCATTGGAGGGAAACAGGACACACAGGGGACCTGTGCCCCTAAACAGAGCCCCA	A	C	0.36	0.51	0.52	0.48	0.46	0.32	0.35	0.53	0.64	0.44	0.45	0.021	0.018	0.004	0.034	0.0013			
rs1045599	17	16.08	TGTTGCAATGTTCTGTGGTTGGCAGATGCTTTTTGTTTTGTTAAAAGTGCAACA[A/G]TAAAATTTATCAATACTTTTCTGCTACTCAGGAAACTTGGACTCAGCATAACTTACTT	A	G	0.69	0.59	0.59	0.62	0.52	0.45	0.39	0.54	0.57	0.60	0.51	0.004	0.028	0.000	0.015	0.0045	1		1
rs178815	17	16.29	TTTTTCTAATTTCAGAGAATGCTGTTCTAATTATTTTTCAAGTCTGTCTCTT[A/G]CCTGTGGATATGGGATGTTGGAACTTCTTGCTGTAGCTCCCATTTTTAACTTTTTCCT	A	G	0.40	0.58	0.58	0.63	0.52	0.41	0.36	0.46	0.61	0.59	0.52	0.017	0.032	0.001	0.021	0.0020			
rs1869771	17	17.76	TGGCCTCTGCTCTTTCCTTGGGGCA[A/C]CCTCTTCCTTTCAACCCTCTACCCAGTCTGAGCCTGCTCACCCCTCCTGGTATCCTTCCCATGTCTCATGCTCTGCCTCTTCTCAT	A	C	0.43	0.50	0.44	0.48	0.43	0.00	0.04	0.11	0.32	0.45	0.44	0.000	0.108	0.063	0.176	0.0008	1		
rs1037037	17	18.97	AGGAACGTCAGAGACCACATCAGGGTGTTGCAGGCCATTTTAAACCATGAGAG[A/G]TAAGGAAGACTTCAGGAATATCCTGGAAAAATATACAAAGGCAGGAGAGCTATC	A	G	0.40	0.46	0.44	0.52	0.45	0.68	0.50	0.71	0.54	0.36	0.44	0.000	0.001	0.037	0.028	0.0017	1		
rs1860302	17	20.08	GCCTGATCCTTGTTATGAACACAGATAAAAATGGGGTAAACTATATGCCAA[A/T]TCTCAGATACCAAACCCTAAATAAGATGCTAGCTTATTGATGTGAACAATCCAAAGTGCA	A	T	0.48	0.51	0.44	0.46	0.45	0.82	0.53	0.70	0.61	0.52	0.42	0.007	0.012	0.108	0.118	0.0001	1		
rs1478999	17	21.85	CCAGGAAATAAACCTAATTAATGAACTTTAATTATACATCACATATTCAATAGGAGA[A/G]TGGAAGAAAAACTTTGATTGACAAAACCCATTGATTGTGGAGGGGTGGATATTTGAA	A	G	0.24	0.38	0.42	0.52	0.50	0.05	0.14	0.21	0.35	0.40	0.41	0.006	0.026	0.013	0.076	0.0000	1		
rs1564296	17	21.88	AACAGCGATTCCGTCGCGTTTACTTGGGAGGAAGGCGGAAAAGAGGTAGTTTGT[A/G]TGGCTTCTGGAAACCCTAAATTTGGAATCCCAGTATGAGAATGGTGTCCCTTCTTGT	A	G	0.02	0.32	0.40	0.49	0.50	0.00	0.13	0.15	0.54	0.36	0.37	0.081	0.021	0.014	0.112	0.0000			
rs1877497	17	22.09	CTCACCTTGAGTGA[A/G]TGAGGACCATGCTGCTTGGAAGCGTAATAACTTAAACCACCTTGCCTATGTCTAGTGATTAAAAACTAAGCTCTACTCAGCTCATGTAATGCCCCTGAC	A	G	0.38	0.32	0.24	0.23	0.30	0.60	0.66	0.27	0.25	0.24	0.25	0.010	0.063	0.000	0.055	0.0000			
rs1521563	17	25.52	AGATTATAGGGGTAAGTCACCGAACCTGGCCAGAATTTTTTTTCTTTACACAGGTG[A/C]ATAATATTCCATGTTTGTGTGTACAATTTTGCTTCTCCATTTACCTGTTGATAGGCA	A	C	0.71	0.74	0.77	0.73	0.70	0.45	0.38	0.80	0.71	0.78	0.82	0.001	0.052	0.001	0.038	0.0020	1		
rs1402726	17	25.54	TTATCTCCACCATCACTGACTTCTTTTTATGTTTAGAGGATCCAGATGCTCAGTGTCATCTTC[A/G]AAGATGCCAGGTCACATTGGCAAAGGCCGCAAGAACTGATATGAAAA	A	G	0.50	0.30	0.28	0.29	0.29	0.55	0.63	0.31	0.32	0.30	0.22	0.023	0.042	0.000	0.031	0.0030			
rs1060618	17	25.79	CACTCCAACCTGGACAACAGAGCGAGACTCCATCTCAAAAAAAAAAAAAA[A/G]TTGTGTTGCCTCATACGAAATGTATTTGGTTTTGTTGGAGAGTGTCAGAC	A	G	0.55	0.62	0.58	0.55	0.57	0.50	0.67	0.56	0.64	0.62	0.67	0.001	0.030	0.003	0.001	0.0064		1	1
rs727999	17	27.69	AAGGGTACTCTGGAAACGGACGTATGCCCCCTGGTGGTCAGAAATTAGAGGCTAGGCGCT[A/G]CAGCCAGCAGGGTCCCGTCAACCGCACAAATT	A	G	0.50	0.40	0.40	0.32	0.39	0.05	0.58	0.31	0.46	0.38	0.40	0.000	0.000	0.013	0.159	0.0008	1		
rs1060636	17	28.02	TGTGAAATGCAAAGAAGGACAGCAGCACATTTGGAATGGGAACCAAAAGA[C/G]AGCTGTCTGGCAATACACCAGGACAGCCAACATCTTGGTAAGTTATATAT	C	G	0.10	0.16	0.16	0.24	0.04	0.56	0.16	0.18	0.38	0.19	0.24	0.000	0.005	0.002	0.127	0.0063		1	1
rs1057993	17	28.92	GGAGGATTATTACTAGAACGTTCTAGAGAGCCTGTTAAGCAACTTGAAT[A/G]GATTGCTGCGAAGTTTAGGGCTGGTTATACCTTTGACTCTCCTTTAGTGG	A	G	0.67	0.46	0.42	0.45	0.50	0.82	0.64	0.64	0.46	0.45	0.50	0.024	0.018	0.042	0.090	0.0022	1		
rs739632	17	30.02	CAGAGGAACTGCCAGCCTACC[A/G]CAGAATCAGGAAGGTAATAAATCATTGTATGCCTTAGGATTTGAGGGTGGTTTGTTCATGGATACAGGAGGCC	A	G	0.48	0.34	0.44	0.39	0.41	0.32	0.51	0.51	0.29	0.35	0.35	0.003	0.005	0.006	0.002	0.0058	1		1
rs757288	17	30.27	GCAACAACCACAATACAAGGCCAAAGAGGGGAAGGGAGTGGATGCCAGGGAAGCA[A/G]AAACAAGGCATCCCCTAAAGGGCACATAACTCTGGTTATTAATCAGGGGCTGC	A	G	0.79	0.82	0.80	0.78	0.73	0.64	0.66	0.76	0.75	0.77	0.72	0.001	0.014	0.001	0.023	0.0047			
rs894723	17	30.73	CATGCCGGTATCTGTTCTCTGAATGAAAATCAGCGAAGCAGACTGAAACTG[A/G]GGGGCTGGCTCAAAGGGTCTGAAGGTGACCCATAACTCTTCAGCCTCGCCCACCATCA	A	G	0.07	0.52	0.44	0.54	0.46	0.41	0.54	0.35	0.50	0.42	0.44	0.114	0.001	0.010	0.002	0.0001	1		
rs1990823	17	31.23	CTGGTACATTCACTTTTAAAAGCTTTCGATGCTGTAGTTTGCAAATCATTTTA[A/T]CTATTGAGAACTTGATACTCAAGCACAAAGCTGTTTTATCTGGAGTGGGTATGAAAGGCC	A	T	0.76	0.57	0.55	0.59	0.54	0.32	0.56	0.43	0.36	0.52	0.57	0.015	0.001	0.014	0.041	0.0002	1		
rs2052412	17	31.26	GATGTGCGTTCTACAGATATACATACACATATGCACAAAGAAATAGCTACCATT[A/G]TATTTATACTTGCATTGTTCATATGTAACAAACTAAATGTCCAATAGGGACTGATTAAT	A	G	0.38	0.44	0.45	0.41	0.46	0.68	0.42	0.57	0.64	0.48	0.44	0.000	0.000	0.012	0.037	0.0001			
rs28955	17	31.47	GTGCCATGTCAGAGCCAGAGCACCTCCGTTTTCCCTCACCTCACCTGTCC[A/G]CTTGGGCTGGGTTTCCAGTGGACTCCAGAAAGCCCAGCACCAGAGGCTTA	A	G	0.05	0.27	0.20	0.17	0.18	0.23	0.09	0.25	0.39	0.19	0.22	0.031	0.009	0.005	0.000	0.0012			
rs870589	17	31.76	CTCTGTTGGTTTCTGTTCCAACCCCCTGGTTCTTCTTCCAAAGCCCCAGTGCTCAGATAGCTC[A/T]GCCTCACTGTTTTGCATCCCACCCAATTACTGGGAAGGCTCCCTTCTTC	A	T	0.38	0.46	0.45	0.46	0.39	0.91	0.54	0.31	0.42	0.52	0.53	0.002	0.008	0.021	0.143	0.0013	1		
rs758408	17	33.08	TGCCCGTAAAACAGTACAAGGGCCCTCCTCTCCCCACTCACTCGCTCTCTCTCTCT[C/G]CTCCTTTCCTGGCCTCCTTCCTTTCCTCCTCCGCCTTCCACACAGCAGGCAGCAGC	C	G	0.79	0.63	0.60	0.61	0.55	0.86	0.86	0.73	0.79	0.66	0.70	0.032	0.057	0.004	0.046	0.0057	1		1
rs758299	17	33.28	TTAGTACCTGATCAGGCCATACCTAGCCCCTCTATTTTTTCAAATGTCTGAGCATC[A/T]CCCTTAAATGGGATATGAACAAGCTGCAGCATTTTGGAGAAGGATGACCTAAGGAT	A	T	0.43	0.54	0.54	0.54	0.55	0.82	0.97	0.75	0.57	0.56	0.57	0.004	0.149	0.036	0.054	0.0010			
rs225284	17	34.08	CTAGGAATGTTCATGTTTTAAGCATTCTGTATACCAGTGTGTGTCATCCAGGG[A/C]AATTCAAACCATGTGCTGAGCTTTCTGTTAGTGGGGCTTGGTGCATCCTTGCCAGATGC	A	C	0.57	0.45	0.52	0.54	0.48	0.50	0.29	0.57	0.54	0.55	0.48	0.000	0.043	0.001	0.002	0.0011	1		
rs226426	17	34.20	TGTTTTATACTTTTGTACATATGAGAAATCAAGGGATTGATGCAACCAGTAGA[A/G]GCATTGAAATGACTGTCATTAACCACACAGTCCTGGAGGCAGAGATGCAGTTACCTAC	A	G	0.50	0.74	0.65	0.56	0.70	0.55	0.76	0.73	0.54	0.69	0.67	0.007	0.018	0.024	0.002	0.0008			
rs1005552	17	35.15	GGCTAGCTTGCGGTAGGGGAGTAGGTGAGAAGGCAAGAGTGGCTTCAGAATTAC[A/G]GAATCCAGTTGTTTAATCCCTGGCCTGATCCTCTGGGCTCCAAGGCTGGGGCTGG	A	G	0.81	0.55	0.54	0.61	0.45	0.82	0.86	0.70	0.54	0.51	0.55	0.061	0.099	0.037	0.076	0.0001	1		
rs1859212	17	36.23	GACTGATATCTCTGAGAACCTGTGGGAAGAAGGACAACCAGTTGATCTCTTG[A/G]GGACTGAGAGTCAGCCCACATGATATGAGAGGCTGTTGCATCCCAATGCCATTGTCTC	A	G	0.10	0.50	0.41	0.49	0.43	0.27	0.51	0.26	0.43	0.48	0.47	0.077	0.004	0.011	0.015	0.0011	1		
rs1008723	17	38.44	GCGATGGGGTGCTGTGTTCCTTGGTTTCCAGTCTCAGAAGCGATTAATCTT[A/C]AATAGGAGCTGGCTCACAGAGAGGGAAAAGAGTCAGTGGGAGTAAGGCCCTGAGAT	A	C	0.24	0.51	0.54	0.59	0.45	0.55	0.40	0.36	0.68	0.47	0.49	0.062	0.011	0.029	0.000	0.0013	1		
rs1526601	17	39.21	TCTATTCTCACCCTGACAACAGTTTTCTTTTTCATATAGGCTGGACTAAATTCGC[A/G]AAGTTCATTAATTTTCAAATGTAATTGTTTGCAATTTAAGCCAAAAGACATCTTAAAAT	A	G	0.55	0.59	0.56	0.46	0.52	0.50	0.10	0.42	0.64	0.55	0.52	0.001	0.107	0.011	0.000	0.0012	1		
rs1468259	17	39.43	TTGACACCTAGTAAGTCCCCAGCAAGGTGTAAAGCCCCGGATTTATAAGGCTA[C/G]GAAACATATTACAGGAATAAAACCCTAAACCACAAAAAGGTGTACTGTCTACAACGG	C	G	0.12	0.48	0.54	0.56	0.55	0.00	0.03	0.21	0.39	0.44	0.55	0.094	0.155	0.045	0.216	0.0004			
rs1008753	17	40.10	ACAGAAAGATTCATGTTTGGGTCCTGACTCTGTCCAGGTCCATCTCCATATATGTTG[A/G]TGGGGGCCGCTCATTTTAACTCTCTTGTTTTCTTTCTTTCTTAGTGAATCCTCCG	A	G	0.48	0.44	0.39	0.45	0.46	0.05	0.01	0.30	0.43	0.42	0.44	0.008	0.129	0.000	0.071	0.0004	1		
rs13851	17	41.85	TCAGACAGAACGATGAACACAGGTGAGCAGGGAAAGCTAATTTTGAAGTAGG[A/G]TGCATGGAGAGGGAGAAGTGGTGACAGAACTTGGTAACTAGCTGGCTGGGGGAACGA	A	G	0.86	0.70	0.65	0.65	0.66	0.55	0.74	0.57	0.64	0.67	0.72	0.037	0.007	0.000	0.001	0.0056	1		
rs739769	17	42.21	TGGTGGAATTGAATATTTCAATTATGCTGTCTCAGAAATTTGCTTCCGAGTCTAG[A/G]GAACTTCTGGCAATCATTTTGTTCCAGTTGTCATCAGGAGCATGGAGCTCAGAGACCTGC	A	G	0.29	0.30	0.32	0.34	0.23	0.09	0.05	0.34	0.21	0.27	0.21	0.001	0.073	0.001	0.056	0.0121		1	1
rs744281	17	43.49	TCTTACGGGCTGGGGTGCCATTTACTCATTGGACACAGCACTCTAGGTGACA[A/G]TCAGTTGCTGGGCAGGCACTGGGTGCAAACCCTTAACCCTAGGGATTGGAGCAGAAG	A	G	0.52	0.35	0.44	0.38	0.30	0.32	0.41	0.49	0.46	0.46	0.31	0.000	0.007	0.000	0.025	0.0122	1	1	1
rs736604	17	45.50	CTGACAGCTTCTCCAGCTCTCATCAGTATCTCAGCAGCATGAACGTAAGTTGTA[A/G]GGAGCTCTGAAGGTGTGTGGCAGTGACAGCCTCTGCCCTGCCTCCGCCTGGTTGG	A	G	0.43	0.46	0.49	0.44	0.41	0.55	0.52	0.53	0.50	0.50	0.53	0.003	0.002	0.001	0.001	0.0015	1		
rs2013383	17	46.27	TGTGGCGATGCGCCCGAGGGTGGCTTGTTCCCTGTGAAGTTGTCTTCCCCCA[A/C]CCCTGCATGTCTGGCTTTGGGAGAGTGAGTGGGTCCCCCACGTGTGTCTTTCCAGCC	A	C	0.26	0.46	0.45	0.34	0.34	0.45	0.74	0.40	0.54	0.41	0.44	0.012	0.052	0.000	0.000	0.0001	1		
rs2051821	17	46.62	TGCCAACTCCTTGGTCTATTCTCCACCTCTCCCCAGGTGAACTAGATTTTTTGTTTCCTTAGTTGAAGAGCATCT[A/G]GAATTAAGTAAAAAATACTGTGCAACCTATCTCCATTC	A	G	0.67	0.80	0.83	0.83	0.79	0.73	0.93	0.81	0.79	0.76	0.80	0.026	0.002	0.000	0.025	0.0036			
rs733920	17	47.11	ACCCCCTCCCTGCCCATACACCTACCAGCCTTCCCAGTTGCCTCCCCCTCAGTTGGAGAC[A/C]AGCATTCCCCCAGATGGCCAGCAGGGTCCACAGGGCCAGACTGTGTCC	A	C	0.38	0.20	0.14	0.18	0.09	0.18	0.17	0.27	0.18	0.17	0.16	0.066	0.011	0.044	0.013	0.0010	1		

rs948384	18	18.26	GAAGACAGGGACTAAATCGTGAATGGTTTCTAGGTCAGGCTAAGGAATTTAAATTC[G]TTATTCAAAGTCATTGGAGAGTTTAAGCAGAGAAGTGGCATGATCTGATTTATCTT	C	G	0.24	0.56	0.52	0.48	0.65	0.73	0.83	0.30	0.57	0.43	0.41	0.049	0.045	0.028	0.017	0.0069	1	1	1
rs1010800	18	18.86	GAGAACCATCTATGTGAAGAGTATAAAACAGTTCTTGACACACAGGAAGTGGCCA[A/G]TCAATGATAACTATTATTTTTGGGAGATTATCTGACTACCAGCAGAGAACAAAAATC	A	G	0.83	0.77	0.74	0.80	0.86	0.45	0.48	0.77	0.62	0.80	0.74	0.025	0.006	0.011	0.013	0.0006	1		
rs1972602	18	20.37	TTCCAACAATTACAACTAATCTTTTCACTAATTAGTAATAACTCTAGTCTCCA[A/G]CTAGGACCAACCTCCAAAGTTTCTTTTTTTTTTTTTTTCTTTTTTTTTTTT	A	G	0.05	0.23	0.28	0.20	0.21	0.00	0.00	0.22	0.25	0.23	0.27	0.075	0.129	0.003	0.129	0.0003	1		
rs736209	18	20.82	GGTGAGGAGAGGACATTCCTACATTAGACACTAGTTTTCGATATAGACCTGGGCT[A/G]TGGACCTTTGGTCATGGTAACAGACTGTGTGTGATGGATACACCACAGGGGAAAA	A	G	0.14	0.32	0.40	0.35	0.45	0.05	0.04	0.11	0.25	0.43	0.45	0.049	0.109	0.055	0.101	0.0028			
rs1459098	18	21.78	GGTCTCACCATGTTGCCCAGGCTGCACTGGTAACTTCTTAATTGCTAAGCCACTAGCTTTGGCTCTATTCTTCTCAACTTGGAAAATTCTAT[A/G]GCATTTGGTACCCTTGGTCT	A	G	0.83	0.45	0.45	0.41	0.50	0.36	0.87	0.27	0.43	0.46	0.44	0.087	0.110	0.020	0.003	0.0000	1		
rs1185007	18	22.84	TTCCCTCCTTAATCCTCGCCTGAGGAAATTCTCCTTCCTCTTTGTTTGCTCGC[A/G]GTAATTTGCTCTTCCTTTTTACAAGCCTATCTTCATTGTGTTCTCCCATTATGCCTG	A	G	0.86	0.55	0.58	0.62	0.63	0.32	0.45	0.78	0.71	0.60	0.54	0.062	0.013	0.038	0.037	0.0008	1		
rs665781	18	23.83	CCTCTACAGGTGCTTCATAATCTACAAACCTGCTGGGTCTCCACTGTGGTA[A/G]CATAGCTCTCCATGCCCTATAGGATGAGTTAGAGCTTTTAATAGTGCATTCCAGACCC	A	G	0.48	0.45	0.53	0.49	0.54	0.18	0.78	0.57	0.46	0.51	0.52	0.004	0.018	0.000	0.090	0.0001	1		
rs1548755	18	24.12	AATATGATTGGGTTTGCATGAGCAAAGGAATATGAAGCTAT[A/G]TTTATTTTTATATTTTTGACACTTTGCCATAGACACTCAATTTCAA	A	G	0.14	0.39	0.36	0.39	0.43	0.32	0.24	0.57	0.21	0.44	0.40	0.023	0.005	0.035	0.000	0.0040			1
rs1941207	18	24.82	AGATGGAGGCCCAGAGGTGGAGGCTTCTCCTGATTATATAGTACTATGCCAGGGGTAAGGATTGTGATGAGAGAGTATTTCTAAT[A/C]ATTTTGATGTGGCTGC	A	C	0.63	0.65	0.73	0.62	0.57	0.73	0.78	0.45	0.79	0.76	0.71	0.008	0.000	0.068	0.002	0.0007	1		
rs1972603	18	24.98	TAAGACTCACTCAGAAATTTTTAATTTTTATCAGGAAGACAATAAATATATCACTTT[A/T]CTTTTCCTTTCCTCTATGTTTTTACTCTAGAGAATGAAAAACAAACAAACCAAAAAA	A	T	0.14	0.63	0.60	0.62	0.48	0.64	0.56	0.42	0.39	0.62	0.66	0.128	0.005	0.012	0.000	0.0032			
rs2011596	18	27.03	GCCAGAGCCCAAAAGCTGGTCCCCAAGGACATTTATTAGTCATAGTGGACAACT[A/G]GAGTGTTCTCTCCTTAATGCTCTTCTGTTCTCCTGGAGGCAGCACAGACCTTCCTC	A	G	0.33	0.37	0.40	0.44	0.29	0.14	0.04	0.45	0.43	0.38	0.34	0.000	0.087	0.000	0.039	0.0024	1		
rs1426311	18	27.15	TCAACTCATTGTATTACATCTCTGACACTGAAGTCATTGCTAAGTCATGCTGG[A/G]AAAAGACTGAATTTAGGAAATTCCTATCGCTAGTTAGTAGCACTTTGTCGTTCAAAATCT	A	G	0.14	0.53	0.49	0.53	0.48	0.00	0.01	0.50	0.57	0.48	0.38	0.073	0.189	0.000	0.216	0.0096		1	1
rs454768	18	27.88	TCCTGGGAGCTAGAGGAAGTTAGAAAGAAACATAAGGATGAATGATAGTGACT[A/G]CTATCACTGAGTTTACTGAGTTATAAGTATTTCTTGCTTCTAGCAACTCTGTGA	A	G	0.55	0.66	0.59	0.65	0.55	0.68	0.71	0.59	0.64	0.64	0.63	0.004	0.009	0.000	0.004	0.0000	1		
rs2015877	18	28.34	GAACAGAAGAACAAAAAACAGCTCATGATGGTTTAATGCTGG[A/C]AGTTGAGGCTTTTTCAGATTTGAAAATGATCCCCGAAAGCATGAGATTGAATTGGATTAACC	A	C	0.35	0.20	0.30	0.22	0.20	0.09	0.70	0.13	0.11	0.29	0.31	0.002	0.082	0.015	0.040	0.0001			
rs1389532	18	29.64	AGGAATGGACTTGTTTTATGACTTTATGACCTACCCACAAGAACAGGTAGTT[A/G]TAAGGCAGGTCCGCCTCCTTGTTCCCTGGCTCTTGCACATACCTGTTCACCTTTCTGCT	A	G	0.83	0.57	0.45	0.39	0.39	0.91	0.94	0.47	0.57	0.49	0.46	0.095	0.162	0.005	0.137	0.0005	1		
rs1617318	18	30.70	GAGCTAATGGTCCAGTTGGAGGGTCTCATGAAGCTACTAAAGGTAAGACCTGCCA[A/G]ATAAATTTTCCTGAGCTTATTTTCATGTCATCTGTGAAAATTTTTGCACATAATGTC	A	G	0.52	0.30	0.39	0.38	0.32	0.00	0.00	0.33	0.18	0.35	0.30	0.008	0.170	0.005	0.170	0.0051	1		1
rs680798	18	30.77	GGAATCACACAATGCACACATAAAATTGAGTCTTGTGTTTTGCTTTATCACT[A/G]AAATTTTTCCCATTTGCTTCAGGAGTAGTTGATCAGAGACTTGCTTTGTAACATCAAGT	A	G	0.86	0.66	0.60	0.57	0.68	0.86	0.54	0.69	0.61	0.65	0.66	0.020	0.007	0.002	0.026	0.0009			
rs1131709	18	31.14	GTATGAAAAAGGTTTTAATCAGTGTTCAACTCTTATGCTACATTAAAACC[A/G]CACTGGATCCGGATACGTGTGATGGCTCACGCCTGTAATCCCAACACTTT	A	G	0.50	0.49	0.45	0.43	0.50	0.73	0.81	0.45	0.57	0.43	0.55	0.005	0.103	0.004	0.064	0.0005			
rs1789501	18	31.87	TATATTCATGGGGAGAAGAAATAAGGAAAAAAAAGTAACATTTTAGTGATGTG[A/T]CCTATCATCTGCCAATTGTATGGAACTTGCAAACTCCTGATTGAACAGGAAACGTTAA	A	T	0.86	0.33	0.38	0.39	0.43	0.45	0.50	0.32	0.39	0.30	0.35	0.143	0.005	0.002	0.003	0.0001	1		
rs607856	18	32.02	TCCCCCTCTGGCCGGAGACCCAGGGTCTGTGGAGTGCAGCGCTAGGGTGCAC[A/G]TCGCCCAGGTCCCAAGCTGCCCGCTCCCCACTCCCGCTCCCACCTCAGCCGGCCCAGCCCGCTCTCTCCCC	A	G	0.60	0.47	0.54	0.61	0.57	0.55	0.56	0.56	0.43	0.48	0.48	0.006	0.000	0.004	0.001	0.0004			
rs873201	18	32.57	TGTATCAAACACAGACAAAATGATTTGCCTGAAGCTAAGCTGGATCCACCAAGGC[A/G]GAGCTAGAGAAGAACCCAGGGGCTACCTCTAGTTTCTAGCACTAGCTCCATAGTT	A	G	0.45	0.30	0.32	0.29	0.30	0.27	0.46	0.43	0.11	0.32	0.32	0.006	0.008	0.008	0.001	0.0001	1		
rs948438	18	33.10	TCTTCACCTCTGATCCCCAACTTCAAGGGATACACATGAGCCCAGGCAGTGGCCA[A/G]CGCTCAGGTCCACATTCTCAGAGGGACCCTGCTTCTCACATGAGAGGAAGGCCCTT	A	G	0.64	0.53	0.51	0.50	0.52	0.77	0.60	0.67	0.46	0.55	0.55	0.005	0.000	0.012	0.028	0.0005	1		
rs1662910	18	33.10	TTTCCTTAATCCCATTCCCCATTCCAGAGCTTGCCCCATAATATATCCCTCA[A/G]TCCACCAAGAAGAGGCCAGAGGAGTCTAGAGAGTGAAGTGATGAGGCTCTAATGATCTC	A	G	0.64	0.52	0.51	0.53	0.52	0.77	0.60	0.67	0.46	0.55	0.55	0.005	0.000	0.012	0.028	0.0004			
rs894772	18	33.13	TGACAGTTCAACAGCTTCCTTTAGGAAACAGGAGGACATAGAATGATGCTGGACC[A/G]TCAATGGGGCCCACAGTTAGGGCACTGGTGTGGGCTGGAGCAACAGGCAGGGCT	A	G	0.67	0.42	0.43	0.33	0.43	0.36	0.64	0.70	0.32	0.52	0.48	0.022	0.016	0.031	0.006	0.0007			
rs650680	18	33.21	ATTAACTGCCAGTTTACAAACAATGTTGTAGTAGACAGCACAAGATCAGGAGA[A/G]TGCCCACAGGATCCGCTTGAGAATGTAGGAGCAGCAGCGAGAACAATGAAGTT	A	G	0.50	0.34	0.43	0.28	0.34	0.68	0.49	0.55	0.46	0.43	0.53	0.004	0.004	0.010	0.034	0.0073			
rs972900	18	33.59	AGAGCCAAAGGCTCTCACACCAGGCTGTGTTAGGTGGACACAAGAACTGGGATGGAGGAAAGCCGGTGCCTGATAGCA[A/G]AAGCGCAATGGGAAATAAGT	A	G	0.71	0.63	0.71	0.72	0.77	0.27	0.56	0.64	0.68	0.80	0.72	0.002	0.073	0.004	0.009	0.0074		1	1
rs1943410	18	34.47	CATAACAGCAACACTAATCAATTGCAGAGCCAATGTGATCAAAAATGTAAATGT[A/G]AAAGAGGGGGGCTGAAATCACTCAGATCTCTACTAATCATACATAATTACTTGAAGT	A	G	0.57	0.51	0.47	0.46	0.55	0.82	0.40	0.56	0.54	0.53	0.42	0.007	0.003	0.002	0.076	0.0009	1		
rs1943413	18	34.49	AAGCAATTATGCATCCAAACATTACTTTTGCACTTAGTGATATTCTCCTTG[A/C]AGTGTTGTGTGCTAGAGAAATGAGTTCTCCCACTAGCAGCTCCTGGTCTTGCTCTACAGGAG	A	C	0.45	0.57	0.64	0.62	0.57	0.18	0.65	0.50	0.57	0.59	0.68	0.014	0.000	0.003	0.107	0.0013			
rs307144	18	35.77	GATGGGTCATTCAGGAGGAAAATAAATGTGAGAACCTATGACCTGCTTTTTCT[A/G]CTGACACTTTTTGTACTTTCAATTCTAGGTGTTTACTATGAATCTGACTGTTCAAGTTTC	A	G	0.81	0.52	0.60	0.57	0.59	0.70	0.82	0.62	0.50	0.58	0.54	0.016	0.018	0.002	0.002	0.0022	1		
rs2017595	18	36.01	GTGCCCCAGCAGCTTGCACAGGCAGGGAAGTTTGATCTTTTTCGGATTTTGCAC[A/G]TGTTATATTAGATGAGATGTCCCTGTAAGCTAATTACTTTGGCAACCAAGAGGGCACCAA	A	G	0.95	0.61	0.53	0.50	0.66	0.91	0.88	0.68	0.54	0.59	0.60	0.150	0.102	0.009	0.108	0.0092		1	1
rs203151	18	36.29	AGTCTTTTCCTCTGTGTAACAGAGTATCTTCAGCATTTTCTGGTCTCATGGAGGCAG[A/G]GAAGTCAATCTACCAGCACTCAGTATTCTATTCCAGCATACGTTGATAATAGACC	A	G	0.69	0.59	0.52	0.54	0.55	0.95	0.92	0.69	0.32	0.48	0.44	0.027	0.150	0.036	0.179	0.0012	1		
rs52911	18	37.81	CTTTCATTTTAGCAGCTCTGCCTGTACTTTGCAGCAGATTTTACCTTCAC[A/G]TTGCATTGTTTCTGCTGTTCCCTGCCCTGCCCAGGCCCTGCCCTGCCCTGCCCTTCTGCCCCTCCCTCCCTCCCAGCTTTCTCACTC	A	G	0.71	0.38	0.35	0.33	0.32	0.77	0.51	0.55	0.50	0.42	0.40	0.080	0.014	0.027	0.099	0.0004	1		
rs1941531	18	38.02	GAAGGGCACTTTGACCCTGAGGGGTATAGCAGAAGAGTGTACACATTTTCATTAT[A/G]CAGCTTCATAAACAGGGTGTCTTTCTCTATAGGTATGTGGTAATCAGTTGTGAGCTGT	A	G	0.88	0.71	0.75	0.66	0.82	0.77	0.53	0.56	0.75	0.71	0.68	0.019	0.017	0.027	0.002	0.0005			
rs1878677	18	38.98	AGCTAACCTTGGGACAAACTGTTGCAAAGTGGCAACCCCTACTCTGGTTGAACTTA[A/G]TGGTATCTTCATGTGTATTTGTCTTATTTTAGATTTCAAATTGCTGAGGGGTAGAA	A	G	0.50	0.36	0.40	0.41	0.41	0.50	0.81	0.29	0.54	0.37	0.28	0.003	0.089	0.035	0.003	0.0116	1	1	1
rs1460508	18	39.73	CCTACTTTAATTAGATCCCTGCATCTAAGGAAAAAGTGGTCTCTTTTGCTTCTGT[C/G]CCAACATCTGTAGCTGCAACAAAGATCAATTACAATGCCCTTGACAAGGACTTCAGTT	C	G	0.29	0.35	0.36	0.33	0.38	0.59	0.47	0.48	0.25	0.40	0.39	0.001	0.010	0.005	0.040	0.0005	1		
rs1054986	18	41.27	TGGAGCCAGCTTGTGGTTGCAATAGGAATAGAAGAGACTTCCTTACTCCA[A/G]TCCCACCCTACCCCCTCATCCTGCCTCCAACCAGTCATGCAGAGAGATGCT	A	G	0.90	0.45	0.51	0.55	0.59	0.64	0.52	0.73	0.61	0.58	0.59	0.073	0.000	0.025	0.002	0.0011	1		
rs1345493	18	41.38	AGCTGGCACCAGGCTGGGCCCTTGGAATGTTTGAGTAATTGATTTCTTGG[A/G]AAAGACTCCAAGTCCTGGGCTACAAAGTCAATACCCCAAGTCCTTCTCCTAGCCGACGCT	A	G	0.21	0.26	0.23	0.15	0.09	0.32	0.16	0.11	0.11	0.16	0.15	0.003	0.000	0.000	0.024	0.0050			1
rs920783	18	42.15	GAAAGGAGATGTTCTCCTTCTCTGCTGAGACTTCCTCACTCAGATGAGAGGACT[A/G]TCACAGGAAGCTTCATTTCTATGGCCACCTGGAAGCTGCCTGTTTGGAAGACTCAGGA	A	G	0.12	0.57	0.54	0.51	0.52	0.60	0.61	0.52	0.58	0.59	0.59	0.098	0.000	0.006	0.003	0.0019	1		
rs545344	18	42.62	ACTTTCCCTCCTTCTATCCATGGTGTTGGATTTGTCTGAGCTAAAATAAAATGC[A/G]CTCACTGTAACTTTGTGTTCCTTTCTCTAAATTCAGGTAATTTGGGGCAGCTGGACAGCA	A	G	0.57	0.54	0.48	0.40	0.61	0.86	0.80	0.64	0.81	0.51	0.42	0.002	0.041	0.001	0.062	0.0016			
rs2010834	18	42.81	GCACTTGCCCAGGAGGGCACCTTGGGGTTTCTCCTCTTTAAGCAGGCCCCA[A/C]ATGATTTCTCCCTGATCAAAGTAGGGGAGTGCCAATCTCCCTGGGCACACAAGGGGCG	A	C	0.38	0.51	0.40	0.34	0.54	0.82	0.80	0.64	0.68	0.47	0.38	0.003	0.060	0.006	0.062	0.0003			
rs2137289	18	43.00	GGCAAAACTTTAGCTGTGCTCCAGATAAGAAGGAGGAGCTTGGATGAGGGGAGGC[A/G]CTCTGGACTAGAGGAACACGGTGTGCAGAGGTGGAGGGAGAGGGGTGAGAGTG	A	G	0.93	0.53	0.64	0.70	0.50	0.23	0.57	0.54	0.50	0.57	0.67	0.085	0.000	0.001	0.074	0.0013			
rs1434511	18	43.08	AGGAACTGACATGAACATTTACTACCATCAGGCATAATTTATATGATTACAG[A/G]TGATTAAATAAAATGTACTTGAGTGAACAGGCAATTTGTTTAAGCCTACATCTAGACGCAGCC	A	G	0.64	0.49	0.51	0.52	0.36	0.68	0.55	0.28	0.39	0.48	0.51	0.015	0.006	0.007	0.028	0.0000			
rs1502609	18	43.57	AATTACTGTAACAGAGGCAGGCTCCATCTCTATGGCCTTAATGAATGCTAACATTAACAGTA[A/G]AAGTTTAAGAAAAACATGAGGTATGTTCATCCAAAATACTATTA	A	G	0.76	0.70	0.82	0.74	0.66	0.50	0.25	0.75	0.71	0.82	0.78	0.005	0.146	0.001	0.051	0.0034	1		
rs1981	18	43.61	CAGTCCCCAAAATATTTTACATCACTAACTAGTGGCTAGTCCTCTAAGGCTTGCT[A/G]AAAGCTAGGGTAAACGTGTTAACATCTATTCAAAGCTTCAAAGATGGTGGCAAGAGCTCATTT	A	G	0.43	0.46	0.44	0.52	0.55	0.05	0.07	0.33	0.46	0.43	0.42	0.001	0.085	0.002	0.113	0.0001			
rs1787190	18	43.61	GAATCAAAATTACAAAAGGGAGAGATACCTAAGAATCTGGCTTGTTTATATT[C/G]TTTAAAAGATCGCATTTGATTGAAGGTGGGTGCATATTTTTATATCCACTCTTCCCCAT	C	G	0.43	0.46	0.44	0.52	0.55	0.09	0.07	0.33	0.46	0.43	0.42	0.001	0.085	0.002	0.082	0.0001			
rs1319951	18	44.66	ACAGCCTTTGACAGGGAGTCAGGGGCCAGACCCTCCTTGTCACCCAACCCAGTT[A/G]CAGTTCAATAATTCTGTCTCAGAACTTAGAGGAGTTTAAGAGGGAGAAAATGTTTA	A	G	0.43	0.27	0.24	0.26	0.16	0.00	0.00	0.11	0.43	0.26	0.28	0.018	0.101	0.018	0.101	0.0007	1		
rs1873191	18	44.72	ACCAGGGTGGGAGCACTGCCAACAGTAGGAAATCTGATTTCTGGTCCCCTGGTT[A/G]TTGTCATCCTCAGGATCATGTGTGAGCAGTAGCTGGAGAGCAACAGTGAG	A	G	0.86	0.68	0.69	0.66	0.71	0.77	0.78	0.75	0.71	0.69	0.71	0.018	0.002	0.002	0.015	0.0013			
rs521861	18	45.62	TTCTAACTAGAGCCAATGGCCAATCCACAGCATCTGCTGGACCTATAAAATG[C/G]CCAATGTCAACCATGTACTCAAGAGTATATGGCTCTTTATTAACTAGAAC	C	G	0.60	0.54	0.52	0.44	0.54	0.41	0.69	0.42	0.46	0.60	0.59	0.000	0.004	0.019	0.021	0.0002	1		
rs2045531	18	46.34	TCCATTCATGGACTGTCTTCCCTGGGGTTGATTAATGTCACCACTCCAGGTTTC[A/G]GAGGTCTCAGCAAAGTCTCAGTAGCCATTTACTGTGTGCAAGGCA	A	G	0.24	0.48	0.50	0.52	0.45	0.23	0.65	0.41	0.54	0.45	0.51	0.035	0.006	0.001	0.044	0.0008	1		
rs1470325	18	46.53	TCTGGGTAGTGTGGTTGCATGTGATCTCAATGGTGTGGCTGTTAAGGGCTAGAG[A/G]TTGGCATCTGGGAACAGATTAAGGATGGGAAAAGGGGTCTGGGGGTCTTCTCTGCA	A	G	0.24	0.56	0.56	0.60	0.57	0.50	0.64	0.58	0.68	0.60	0.53	0.065	0.000	0.000	0.003	0.0002			
rs1811455	18	46.54	GGAGATTTAGAACTGAGAACAGAGATTATGAAGCAAATCAAACCCCTAGTATGACT[A/G]TTGTGTAAAATCTGGGAATAATTTGTGTGCATTAGCATTTGTCTTAGCCAGCATTC	A	G	0.95	0.89	0.83	0.90	0.86	1.00	0.95	0.88	0.82	0.88	0.85	0.018	0.020	0.000	0.055	0.0000			
rs1822459	18	46.63	CAAAAGCAAAACATCTGTACCACTCATTACATTACATCAGTAATTATAATGCATGGAGATGTCACCTACTCCATGGGCTTCA[A/G]TGTTTCTCCATGCATTTGGCTGTAATTT	A	G	0.79	0.42	0.41	0.54	0.50	0.68	0.00	0.27	0.39	0.43	0.37	0.083	0.170	0.044	0.037	0.0006			
rs869224	18	49.23	TAACTGGCCAGTTAGTCTGTGTCAGCCAGTGCTGGGAGTCAGCAATAATCTC[A/G]CAGCCATTCTCTGCCTCTTTGACTTTCTAGTCCTTTTAAAATTCAGCCTGATGTGTCTTT	A	G	0.64	0.42	0.48	0.38	0.41	0.68	0.89	0.56	0.21	0.48	0.49	0.105	0.005	0.003	0.015	0.0001	1		
rs1145315	18	49.94	CCAGGTCCTTATATGTGTTGACGGAGGTTCTCAGGGTCTAGCAGTTAGTTATGGCTTCAACACCAGTTCTTTTGATAGTTG[A/G]GAGAACTATCTCAAAGCCAATTCTGG	A	G	0.26	0.28	0.37	0.34	0.36	0.59	0.57	0.49	0.54	0.38	0.39	0.007	0.005	0.006	0.035	0.0000	1		
rs1942569	18	51.00	CAGAATTTGGCATTTCAGAACATGGCGTCTTGCATCGCTTTAAATTGTTTCATGTGTACATA[A/C]AAGTTATGTCTTGCCAACTGGACTTTAAACTCTTCACAGTCACTCTCCA	A	C	0.83	0.53	0.44	0.56	0.50	0.45	0.68	0.47	0.46	0.43	0.37	0.068	0.018	0.000	0.000	0.0021	1		
rs1792737	18	52.00	GCTTCATGGGAAAAGTCCCATCGCAGAGTTAGTGAATTTGACCTTTGCCCAT[C/G]TGGGGGCAGCCAGAGAACTTTATCTCATTTGTGCCTTTAGAGCTTACAGTTTGGGACC	C	G	0.24	0.36	0.36	0.37	0.27	0.77	0.40	0.44	0.23	0.34	0.39	0.017	0.000	0.006	0.060	0.0001	1		
rs917711	18	52.01	AGCTGTCTGGGTTGACGGATCACTTCACTGGATGATCACTAGCTCTGCCCTCAA[A/C]CACTCAGATGGTCAGAAACAATTCATTCCATTCAGAGGAGCCTAACCAATGCCTTCT	A	C	0.38	0.55	0.48	0.49	0.43	0.77	0.42	0.59	0.75	0.51	0.56	0.005	0.002	0.001	0.041	0.0054			
rs1792723	18	52.02	GAGCAGAAAGAACACAATTAATGGTGAAGGACTTGTCCA[A/C]ATGATGAGAAAAAATTCACAATGTGGGCCCACTCTACATCTCTGAAAGGAAGAAGTCAGCAAACATCCCTAA	A	C	0.48	0.45	0.51	0.48	0.57	0.23	0.58	0.41	0.27	0.47	0.42	0.001	0.003	0.001	0.038	0.0064		1	1
rs2010962	18	52.10	TTATAGTTCTTAGAAAGGTATACATCCACCCTGGCATCATGCAGAAAACTC[A/C]AATATTTTGGAAGACTGCTCAGTCTATCTGTTTCATCCAAACTGGCTTGAAGTAATGAGTT	A	C	0.71	0.47	0.50	0.39	0.64	0.73	0.25	0.53	0.43	0.55	0.48	0.018	0.033	0.001	0.022	0.0012			
rs652437	18	52.34	AATCATATCAAAGGAAGGCAAGTTTGGGGGCAAGAAATCACTCAGAGATGCAT[A/G]TTACACTCCACATGAATTACCGGCAAAATGAAGGATGCATGTGAAATAAACAA	A	G	0.88	0.54	0.54	0.59	0.38	0.68	0.19	0.61	0.68	0.55	0.51	0.060	0.079	0.015	0.009	0.0000			
rs625628	18	52.35	GACTATAGCAATCATCCTCATTCTGGCCACCTCCCTTAAAAGAATCTTTT[A/G]GACCCAATATCTGAACTTAATTTCAAAGGAATCCCAAGCATCAGGCTGGT	A	G	0.14	0.57	0.52	0.55	0.79	0.50	0.81	0.48	0.42	0.60	0.57	0.069	0.053	0.008	0.000	0.0004			
rs663220	18	52.35	CAATTTGTTTAGAAATTTGAAATAGTTCCTTGTAACAAAGACACTCGATGGGAACA[A/G]TGGAAATGGTTTAAGTATACAAGTGCTCAGAAACCTACAGCATTTGGGCAAAAGTA	A	G	0.95	0.58	0.58	0.61	0.39	0.68	0.20	0.68	0.71	0.57	0.54	0.104	0.071	0.022	0.008	0.0001			
rs732982	18	52.88	TATCCATAAAAAATCGATGTAATGCCCTACCTGTAATATAGGGAGAAATAAAAC[A/G]AAAGTCATTTATAATAAAGTAGTTCAATAAGTAAATACGCAGGCACAACTGTACTC	A	G	0.14	0.34	0.39	0.40	0.29	0.00	0.01	0.32	0.32	0.28	0.40	0.055	0.151	0.014	0.176	0.0000	1		
rs13732	18	53.36	GCCTGCTCCTGAAATGGGAACAGACAGGCCAACTTCCCCTCTCCAGTCTCCCCTG[A/C]GGGTCAAAGCTTTACTTTCCTGTCATGTTAAGAAAGAATAGATTTAACCTTGATAA	A	C	0.10	0.47	0.48	0.54	0.50	0.18	0.05	0.19	0.54	0.42	0.45	0.101	0.149	0.053	0.054	0.0014			
rs4941043	18	53.57	TTCTCTCTCTCTCTCTCTCTTTTTAAAACTGAACCTCCCACTTTTGGGCTTTCT[A/C]TGTCATTTCCTCGTGATTTAATTCAAGGTCTCTGTTCTTGCTGGAACATCACATAGGTA	A	C	0.57	0.60	0.61	0.63	0.66	0.45	0.53	0.76	0.61	0.58	0.52	0.004	0.007	0.016	0.022	0.0045	1		1
rs1547869	18	53.74	TTTATATGCAACGTGTGAACAGTAATTTAACAATTCTCCCATTCAGGATGTGTA[A/C]ATTTGTTTCTTGTTTTTCAATTTATCAATAATTTACTGGCACAGCCTTGCACCCCTGC	A	C	0.75	0.84	0.80	0.85	0.84	0.91	0.52	0.93	0.82	0.73	0.85	0.012	0.049	0.012	0.003	0.0016			
rs734779	18	53.98	CACTATTTCCCAGCTCAGATACCATCTTTTAGGAGAGTACCTGCCATTTGTGAAAAT[A/C]AGGATAAGGATAACTAGGATTACCTCCCTTAAGACAAAGTCATAGGAAGAATGGTA	A	C	0.57	0.50	0.50	0.40	0.48	0.68	0.28	0.55	0.57	0.50	0.45	0.000	0.044	0.000	0.011	0.0027			
rs1864921	18	54.19	GGGAGGAGGCCGGGAAAGGGGGGCAGTGGGGAGGGGCACAAAAGGAGATTTTTA[A/C]AGGACTTGATCATTTACATTAAGCCACAAGGTCTCTTGCCTCTGGTGCCTGAAG	A	C	0.36	0.63	0.60	0.59	0.70	0.55	0.66	0.51	0.54	0.57	0.60	0.035	0.003	0.001	0.001	0.0007	1		
rs1948617	18	56.82	ATTCCTTACTGTGGTACCGACATTCTCCCTTAATATATTTCATTTGGCTCTCTATA[A/C]AAACTAAATCTGACCTAAACCATATATTCAAACTCTCTCATTTGTCTCCTTAAAGCC	A	C	0.40	0.48	0.54	0.51	0.57	0.59	0.49	0.68	0.46	0.57	0.63	0.007	0.001	0.025	0.004	0.0031			
rs955427	18	57.04	ATGTCCTTGCCAGATGCCAGACCCTTGATCTTGGACTTCCCAACCTCCCAACTGT[A/G]AGAAATAAGTCCCTGCTCTTTATAAATTAGCCAGTCTTCGATATTTCATTCTTGTA	A	G	0.60	0.24	0.25	0.29	0.32	0.14	0.66	0.29	0.36	0.32	0.33	0.067	0.096	0.002	0.005	0.0033	1		
rs929525	18	57.25	TATGTTGTTCAAGTACAGAGCAAGCCAACTCAACTTCTCCCATGACTTCAGCTCC[A/G]AAGAGAAATCGTGTTTTTCTACTGTAGCCACAACTTAATTTTCTTTTAGCTTTTGTCA	A	G	0.69	0.73	0.75	0.79	0.68	0.50	0.48	0.76	0.68	0.76	0.78	0.005	0.031	0.002	0.038	0.0000			
rs1943329	18	57.32	CTCACATATGTAAGGAAGCTCAGAGTTGGTCATCTCCTTTGGACATGATATCCTGA[A/G]CTCACAATTAAATTTAGTTCACTTTCTATCTCCATGAATTGCATTTTTACCAACTTA	A	G	0.69	0.68	0.66	0.67	0.71	0.82	0.49	0.71	0.54	0.65	0.67	0.000	0.023	0.002	0.014	0.0000			
rs1984372	18	57.56	GCCACCGCGCCTGGCCACACCTCTGTTTCTAATAACTGCCCGATCCACACCTCTGC[A/G]CTGTTGTGAGAATCAAATAAACTATGTGAAATACTTTGTATGCTTATTTATCTCTT	A	G	0.38	0.54	0.54	0.54	0.41	0.59	0.76	0.50	0.54	0.53	0.56	0.007	0.031	0.000	0.003	0.0006	1		
rs1506350	18	57.97	GAAAGTCAAAAGAATC[A/G]TAAGTTAAACCATTGTTAAGCTGGGGACTGTCTGCAAACACTTAAAATGCTTAAGACAGTAAATTCTGTATTTTGCATATATATATATATATATTTT	A	G	0.48	0.63	0.63	0.68	0.46	0.73	0.80	0.59	0.64	0.62	0.61	0.008	0.018	0.004	0.005	0.0004			
rs884205	18	58.20	TAGGGACAGAAAATACTTGCTTTAATAATATATATGCTGTTGATTTCACAAAAATTT[A/C]TTAAAATACAGCCTGGGTACCCAGTGAAAGAGCTGAAATGGAAATGGAGTATCATG	A	C	0.02	0.24	0.25	0.17	0.41	0.23	0.19	0.26	0.36	0.21	0.19	0.062	0.003	0.001	0.000	0.0009	1		
rs624821	18	58.71	GGGCATTGCAGGACACGTTCAGCTCTGCCAGGGTTGGAATACTGCAGACTGCAG[A/T]GGGAAGTCCCCTAAATGATTATTGGAAGGTTAAGACTCTTCAACTTGGTGAACCT	A	T	0.48	0.59	0.64	0.57	0.75	0.36	0.51	0.46	0.61	0.66	0.60	0.014	0.010	0.019	0.035	0.0004	1		
rs1472948	18	59.24	CAGAATCAATTATCATGGGTTAAGGAGTTCTTCCATTACCTTTGATCGCCTCT[A/G]TCAGGCACTCCAATAAATCTGCTTGTTCAACAAACTACCGAAGTCCACCACGGGGACTCAA	A	G	0.38	0.48	0.43	0.52	0.38	0.82	0.82	0.72	0.46	0.48	0.47	0.000	0.109	0.041	0.101	0.0006	1		
rs715351	18	59.40	CCCATGATAAGGCAGGGGGGTAGGCACAAGAATCTCCACGGGCCTCAAGTTCAA[A/G]CAGTGCTCTCAGATCCAACCTTAGCAATAATTAAGCCGAGACTTGAATCCTTATCT	A	G	0.57	0.27	0.28	0.32	0.27	0.77	0.53	0.38	0.29	0.30	0.26	0.035	0.036	0.001	0.117	0.0003			
rs1015416	18	59.72	TTATTTCAAAGGGCTGTAAATATTGAGACATGGCACGGTTCCTGGCCCATAGTA[A/C]GAACTAAACAAGTGTTTTAGAATTATGGTTGTCATTACCATTGGTTGTCCATCTTACTA	A	C	0.36	0.79	0.78	0.70	0.73	0.64	0.45	0.66	0.64	0.78	0.66	0.090	0.024	0.004	0.005	0.0061			
rs1944268	18	59.80	CCTGAGTGACCACATCATTCAACACGCTGTACAGAGCTCCTCCCCAGTCCCA[A/T]GTGTGGTTATTCGGCTGCCTCCTGCTGAGGTAGCCTCTCAAATTCTCTTAAATTTGCCC	A	T	0.18	0.22	0.29	0.37	0.32	0.55	0.55	0.48	0.14	0.42	0.40	0.014	0.030	0.020	0.035	0.0117			
rs1529340	18	59.82	ATTTCTCAAGTTCTCTAGGGCTTTTTTCCCTCTTCTCTATACCGCTTTTATTTT[A/C]TATCAATTCAACTTACTGAGAGGAATTTTCAAGGCTTTTTTCTTTTCAGCAAGCCTGTTAG	A	C	0.40	0.35	0.35	0.37	0.36	0.50	0.00	0.27	0.39	0.49	0.49	0.003	0.123	0.002	0.018	0.0143			
rs1460967	18	59.83	TCACTTTATTTGAGATAAGCTAACTGAAAACCACATTTGGCAAAACACTGCTGTTC[A/G]TTTCCAATCTAAAAGATTTTTGTCTTTTATAGTAGGGAAATTACACTTAGTTTGATTAT	A	G	0.38	0.35	0.35	0.37	0.36	0.50	0.00	0.26	0.39	0.49	0.49	0.001	0.123	0.006	0.018	0.0143		1	1
rs1517162	18	59.95	TACAATTTCTGTGTGTTTTAAATCTCTTATTTAAGTTCTGCCATTCTGCCAATCAAAAGAATAATCATTATCATCC[A/T]CCTGTTCTACCTTTTCAGTTACCCAATCAATCAATAAT	A	T	1.00	0.75	0.72	0.63	0.66	0.82	0.89	0.63	0.50	0.70	0.74	0.135	0.028	0.001	0.016	0.0016	1		
rs565973	18	61.23	CATAAAGCTGGTGACACTCTAAGCCTAAGCTATAATGCCTCAGGGCCAACCTGG[A/C]AAACAAAAGTACAAGCTCACACTGAGAACCATGTTCTGCATTCCTTACTTCTGAACTC	A	C	0.38	0.59	0.55	0.59	0.39	0.82	0.55	0.63	0.46	0.51	0.54	0.016	0.000	0.002	0.039	0.0006	1		
rs978418	18	61.91	TCCCCTTTCTTTTTACATATTGTTAAGAACACAATATCATTCAGCATTATCAGA[A/G]TGATTCCTTCAGGGTTTCTCCTTGAATTATAACTGCAGGTGACTCAGCTGACTCATTCT	A	G	0.23	0.60	0.63	0.59	0.64	0.59	0.55	0.56	0.54	0.57	0.53	0.087	0.005	0.007	0.001	0.0034	1		
rs923641	18	62.08	TGAAACAAAAGAGGGGAATTTTTATGCAAAAGAAGGCAATAGGTATGTCAAAACA[A/G]TTTTACAGCTATTAACATACAATATGATATATGATAGTACATAAAAACAACAAGTAAA	A	G	0.10	0.35	0.36	0.35	0.39	0.00	0.52	0.31	0.27	0.38	0.33	0.056	0.014	0.000	0.140	0.0006			
rs531162	18	62.33	TGCTTCAAATAGAGATATAATTCCTGAGTCCCAAAATTTGCCTAAGGGTATGCTGA[A/G]CTTCCAGAGATGTCACCAAAGTAATGTTTTTTCACCATTTTCAAGAGGGCCATATA	A	G	0.19	0.29	0.26	0.29	0.20	0.45	0.44	0.13	0.39	0.17	0.23	0.010	0.004	0.013	0.013	0.0014			
rs1157329	18	62.79	GAGCAAGGGCTAATACTGAGAGTATTGTAATTAGCTGAATGAATGGTCATCGTAGACAGGAGTGTCCAAGCTGGAGCATACACAGGGGGCTGGGTCTCTTA[A/C]AGCTCATCTT	A	C	0.48	0.70	0.65	0.62	0.63	0.36	0.54	0.49	0.50	0.67	0.61	0.008	0.001	0.009	0.025	0.0002	1		
rs664144	18	64.08	ATAAAATAAATTAGATCAGCCAATCAGTTCATTTGGAAAAGGTTTGATAATGGTGCT[A/G]TGGAATGGCTTATTTGATAGATACCCATATCCTAAGTTAACTAAGAATATATTCTAC	A	G	0.67	0.34	0.35	0.37	0.39	0.45	0.38	0.53	0.21	0.38	0.34	0.054	0.002	0.020	0.007	0.0000	1		
rs593418	18	64.08	AAGGGGGTGATTACACAGTCATCACTGGTCTCCAACTGTCCCAGAGAACTCT[A/G]CTGAGGTTACTTAAATTAACCTTTGCTGGAATAAGTTTGGTTAATATGCTACCTCCAGA	A	G	0.45	0.38	0.38	0.37	0.50	0.45	0.14	0.21	0.46	0.37	0.33	0.007	0.034	0.010	0.009	0.0000			
rs2032245	18	65.01	GTGAAACTTAAAGAATTTTCATTGAGTAAAGACACTCATAAACATAGCAAGGGAAT[A/C]AAAATGATATCATTGGTAAAGCCTTTTAAACCTTTCTCTCTGCCCCTTTCTAGTCAT	A	C	0.76	0.79	0.81	0.82	0.77	1.00	0.99	0.84	0.86	0.70	0.79	0.002	0.059	0.000	0.080	0.0000	1		
rs1539964	18	65.77	GAGCTGAGACCTGCTGGATGGGAGAGG[A/G]ATAAGAGGTGAGGAAACTCCACGTGCAAAGGCCCTGCCCCATTAGAGCATTGTCCTCAAATTACACAATGTAAGTTGGATGA	A	G	0.17	0.60	0.56	0.59	0.55	0.86	0.71	0.63	0.54	0.43	0.57	0.095	0.127	0.013	0.094	0.0000	1		
rs1942399	18	67.49	TAAGTGATGGAAAGAGGCAATTGGGTATGAGTGAAGTATTAAAGTGCTTTTGT[A/G]TGAACCTATTGATTACACATAATAAATGTGTAGATGGGCTATTTACTAATGTTAGCTGC	A	G	0.50	0.51	0.47	0.39	0.36	0.55	0.46	0.54	0.50	0.45	0.45	0.000	0.002	0.000	0.001	0.0002	1		
rs1394984	18	67.63	CCAGACGAACTAGTTGTGGCCATAATGGGACGTGAGGAGTCAGAGATGTCTCACT[A/G]TAACTTCCTCCTTTGAAAATTTAGGCACAACTAATCAGCACTAACATAAAAATAAA	A	G	0.69	0.68	0.65	0.61	0.63	0.86	1.00	0.54	0.81	0.64	0.70	0.004	0.170	0.003	0.050	0.0037			
rs1943664	18	68.69	ACTATGTGCATTTGAATTAGAAGGGCTTGTACCCTACGCCAATTCTTTTTAGG[A/G]TTTTGGAAGTCCATATGATCTTTCATTGTTAGGAAGCTGCAAGCTGAGAAATAAAACCA	A	G	0.40	0.65	0.61	0.66	0.64	0.36	0.41	0.65	0.75	0.70	0.77	0.017	0.032	0.000	0.031	0.0174	1	1	1
rs1430553	18	69.60	CGACTGCCTGATCAAATGCTGAGGTCCTATACCTGTGTGGAATCTGAGGGCAGA[A/C]TGAGTGACTTCAGAGTCATTGGCGCACATTGAACAATAATGTTCAAGAAAATACAA	A	C	0.67	0.64	0.63	0.68	0.54	0.75	0.86	0.61	0.65	0.66	0.72	0.002	0.044	0.000	0.015	0.0032	1		
rs1943919	18	69.80	TGTGCCCTACTCCTAAGAAAAAATTATCTCACAGTATAATGTCAGGATTGCAGGCACTGGGTTGTAGCTTCCTGGCACTCCACAAAACACCCTACA[A/G]AAACATTTGTCTTCT	A	G	0.74	0.34	0.35	0.49	0.39	0.23	0.76	0.53	0.36	0.38	0.41	0.104	0.125	0.020	0.002	0.0047			1
rs2850510	18	69.81	TGTCCCACACTGGTTGTCAGAGTAGGGGGTGGGCACAGGAGGGGGAGGAGTATGCTC[A/G]GGTGGTGACTATTTTCTGTCTGTGAAAATAAATGAGGATCACCCAAATGAGAACAA	A	G	0.52	0.93	0.93	0.95	0.96	0.91	1.00	0.85	0.96	0.89	0.92	0.092	0.031	0.007	0.000	0.0000			
rs1539978	18	70.00	CTCATGTTTTTTAATGAAGGGAAACGGCTTTAAGTTTTTGTAAGTCTTTGGTAAC[A/G]ATGGGCTAAATTTCCCCAGCAGTATTTCGGAAGACCTTCTTAACTTGCCTCATTACT	A	G	0.38	0.52	0.47	0.40	0.54	0.00	0.45	0.24	0.35	0.51	0.42	0.007	0.001	0.050	0.237	0.0016			
rs734559	18	70.33	TTTAATTTGGGAAATGCTTGCTTTGCTCCCTGAGGGAGCAAATTTTGATTTGG[A/G]AAATTGTCAGGCCTTGTAAATGCTCTTTTGTTGAATTTACTGTCACATGATAAAAATAA	A	G	0.14	0.20	0.28	0.23	0.23	0.32	0.46	0.23	0.14	0.21	0.13	0.023	0.008	0.011	0.000	0.0237	1	1	1
rs8090565	18	70.51	GTCAAGACGACCTTAGATGGGGAGACCTATGGGCTTTACAATTGGAGTAGAATCC[A/C]GGCCTCAATGAGCTACAAGCACACTTGGTCATGTCGCTTATCCTAAGTCTGAGTT	A	C	0.43	0.59	0.58	0.65	0.48	0.73	0.85	0.69	0.71	0.62	0.51	0.012	0.054	0.009	0.020	0.0050			
rs999647	18	70.70	TTTGTAGGAAAGTAGAAAGATAATTAAGGAGGAAGCAAATCTTTTCATGAGAAAGT[A/G]TAGGATAGGTTTCACAGCGTTGTCCTGAGAAGGAGAATTGCTTGATATTGGAATAC	A	G	0.76	0.38	0.40	0.44	0.38	0.50	0.72	0.68	0.32	0.49	0.52	0.069	0.050	0.028	0.005	0.0055			
rs906507	18	70.89	GATATGAGGGCCTGCTATTTGCA[A/G]GCATATATTTGCAAATAACATATTTTGCTTGTGAACAGTTATGATTCCTAAAAACTGAAAAAATTAAATTGGAGGGTGGGAAGGGGCAG	A	G	0.93	0.90	0.82	0.88	0.89	0.82	0.74	0.78	0.89	0.90	0.87	0.014	0.003	0.001	0.000	0.0019			
rs1866730	18	71.05	GACGGTGGGGAGCGCGGGGAATGGAAAAGCCAGAAGGTCTCGCAACAACCCGGT[A/G]TCACCTGAATGTCTGCAAAACGTGCCGGCAAAGGCAGCATGCCCCCACCCCG	A	G	0.48	0.51	0.53	0.51	0.52	0.45	0.32	0.42	0.64	0.54	0.53	0.000	0.020	0.020	0.005	0.0003			
rs872994	18	71.30	GTCTGATCATACAATCTTCTGCACTTTCCATGGCATGTCTGGAGAGTGTTTGGCC[A/C]GCCCAGTGTGGTAATCCCTGCCTGCTGGGACGGCCTTTCCTTCAACTATTCATGTT	A	C	0.29	0.43	0.38	0.23	0.41	0.00	0.18	0.41	0.32	0.31	0.35	0.002	0.021	0.005	0.134	0.0001	1		
rs1943193	18	71.61	AGGGCTGAAGTCGGGCTGAAGTCAGGCAGAAGTCTTAGCTCCAACCATCCATCC[A/G]CAACCGTGGGACTGGTGACTCCCCAGGATGGTCCCAACTTCCTCCCCTGTCCCA	A	G	0.45	0.68	0.60	0.57	0.50	0.91	0.70	0.66	0.46	0.58	0.64	0.004	0.004	0.004	0.078	0.0051			1
rs744148	18	71.72	TTTAATGAGTAAAAAACAAACTTTGCTGT[A/G]TTTTGTGGCTGTTCGTCTTTCTCTCTACTCCCTGTAACCTAGCCTATCCAGCTACTCAACAGAACCATTGTAAAGCCAGATTTA	A	G	0.90	0.55	0.62	0.48	0.61	0.68	0.60	0.48	0.61	0.52	0.54	0.043	0.007	0.037	0.000	0.0025			
rs2042717	18	72.32	GTCGCGATGAAGGGAAAAGCTGGATCACATATGTACGTCTTAGGATTCCCTCCC[A/G]TTGGTAGATGTGAACCAAAACTTAGGATGTGAGCTTTCTGGCACCTAAAACAAAAAC	A	G	0.55	0.53	0.62	0.56	0.82	0.77	0.85	0.41	0.50	0.61	0.59	0.001	0.034	0.019	0.013	0.0003	1		
rs953830	18	72.49	TTTATTACTTCAAGTGAAGTGTGGATATCTCCCTCCCATTAGTTTGTGTTACCTTC[A/G]TCACTTAAAAAATAATCAACATGATTTATACAACACAGGAGGGGAAGGATAATCTTT	A	G	0.12	0.34	0.31	0.28	0.34	0.14	0.14	0.11	0.36	0.24	0.32	0.015	0.011	0.023	0.008	0.0004			
rs552139	18	72.73	TCAGCGAGATTAACTCCTAGCCCTCGATAATGTCAGTGATCCTGGGAATTCCA[A/G]GCTTTCCCATAACTGATCATGCGTGCAGCTTTCTGTGGGGACCACAGGGGGTCCTGC	A	G	0.81	0.65	0.66	0.57	0.57	0.55	0.48	0.58	0.77	0.71	0.66	0.008	0.026	0.006	0.013	0.0001			
rs470473	18	72.89	ACAGGATTTCCCTCTGAAGATTAATAGATCTAATTCCTTGATTGCATAATACATAC[A/C]TTATTCACTGGCCTGTTCTGGTAAATAATCAGTTCATTTGCTAGCATTTGGAATTAAA	A	C	0.69	0.27	0.24	0.29	0.29	0.73	0.67	0.32	0.07	0.30	0.38	0.078	0.072	0.000	0.100	0.0100		1	1
rs1893828	18	73.08	GCCCCTCCACCCCATGAGTAGGGGGACATCTGGGGATGAATCTGCTTTACCATAGA[A/G]TGGTGAAACAATGTTTCTCGAAGGTAGGGATGCTGGACAGATAAATATGTACTTC	A	G	0.88	0.76	0.75	0.79	0.80	0.68	0.32	0.69	0.64	0.84	0.83	0.015	0.088	0.000	0.002	0.0035			
rs1944566	18	73.20	CTAAGGACGTTCTTAGAATAAGCAACTTTGAAG[A/G]AAACCAGAAGTTGGGCTAAAGTAGGTAGTTACCAGGTGGTGGGAACGGGGCCTTTACCAGGCACTTAAGATGCAGAG	A	G	0.79	0.82	0.87	0.83	0.88	0.55	0.57	0.53	0.68	0.85	0.78	0.008	0.081	0.056	0.083	0.0090			
rs1472672	18	73.57	TGTGATCTTAGCAAGTGAATCTAATTATCTTTCTTTGTCTCAAACACCTCATCTCT[A/G]AAACAGGAACATAGCACCACCTAATTCACAGGTTTGGGGAGTACTAAATGAATTAAT	A	G	0.48	0.76	0.75	0.72	0.84	0.77	1.00	0.82	0.79	0.76	0.77	0.043	0.090	0.001	0.000	0.0005	1		
rs1866338	18	74.40	AGGCTTTCCAATTTTGATAAACTGTACTGTGCATTCATGCCTGTTCATTTGAGTGACTGCAGATCTTAAAAGAATC[A/G]TTTTCGGAAGACAAAAATGCATGTAAAATAAGGCTG	A	G	0.19	0.52	0.43	0.44	0.52	0.50	0.60	0.34	0.54	0.35	0.40	0.018	0.054	0.001	0.016	0.0004	1		
rs1075470	18	74.77	GCACTGTCATCGCTTGTCAGAGCAGCTCGTCCATGGGTTGGACACCCTCATGTCC[A/G]AGAATTCCGGAACATGCACACACATCGCCAAGAGGCTGAGCCAAGTATCATTTTA	A	G	0.38	0.66	0.68	0.72	0.63	0.45	0.42	0.47	0.71	0.71	0.58	0.056	0.063	0.061	0.035	0.0030			

rs1616169	18	74.96	ATGAAATAGTGGTGGTGACTGTGACTTCTATTCTCAATTGGGAACATCAGGTCAATT[A/G]AGTAAGGATATGATTAGGATTCAGACGAGGGCTGAAAAAAATGGGAAAATTTGG	A	G	0.55	0.68	0.67	0.66	0.66	0.36	0.76	0.53	0.68	0.67	0.66	0.009	0.002	0.037	0.058	0.0001			
rs503347	18	75.59	CCCTTCCTCTACCACTGGAGCAGCCAATGCCCACCTAACACACTGACGCGCC[A/G]GAAATGGCCAGAGATCTCGACACCCCTCACTCAAAGGCCCCCTGACGGGCTGCACTT	A	G	0.55	0.53	0.54	0.61	0.59	0.23	0.53	0.55	0.68	0.63	0.66	0.000	0.000	0.001	0.056	0.0121	1	1	1
rs1805272	18	75.89	AAATTTCCATCCTCTCCTCTTAAATAAGACCCATCTGTATACCATTCTATTTTAGC[A/G]TGATCCAGTGGCATCTCTTGTAGGTCTGTCCTGGAGGTGAGAAGTCAGTCAGTCATC	A	G	0.74	0.47	0.57	0.60	0.59	0.30	0.13	0.32	0.46	0.59	0.52	0.019	0.095	0.041	0.029	0.0014			
rs1020382	19	0.22	GGGACCGGCCGGCGCTGCGGAGTCGCCAGCGCTTTCATTCGTGTTGTAATAGCTCATTTGTCTCACAGCACTGAAGTAGTTGAATCA[A/G]TATGGAAATATTTAGCAATAGGCA	A	G	0.81	0.52	0.64	0.58	0.61	0.18	0.38	0.41	0.46	0.68	0.56	0.040	0.032	0.013	0.080	0.0040	1		1
rs7256434	19	0.27	TTCATGGCATGTTTTTCCAGGTTTGCAGGGTGTTTTCTTTCAACACTTGAAAA[A/G]TTTCACTCCATTGCGTTTTGGCTGTCATCGTTTCTGTTGAGAAGTCAGAGGACGGTCTT	A	G	0.79	0.91	0.93	0.91	0.86	1.00	0.74	0.95	0.96	0.89	0.92	0.019	0.036	0.004	0.027	0.0000			
rs2012035	19	0.35	GTGTTACTCCTCCTCCTCGGATGGGAGAGACCCACTGGATCAGTGTTCTGTCA[C/G]CTTAAAAAATGAATCAGCCGGACGTGGTGGCTGACGCCTGTAATCCCAGCACTTTGC	C	G	0.12	0.52	0.58	0.62	0.59	0.19	0.11	0.41	0.50	0.65	0.55	0.120	0.112	0.008	0.078	0.0002			
rs731714	19	0.35	GAGTGTGCGCAGGCCCCTTTGCCTTCTGTGCCTCGGTGTTTCCATCTGTG[A/G]ATGGCAGTAGGCAGCGTCTTGCTGAGGATTCAGCGGGTCCTCCCGCCCACCCACCCACC	A	G	0.87	0.42	0.37	0.30	0.38	0.38	0.34	0.41	0.40	0.32	0.38	0.135	0.004	0.002	0.000	0.0001			
rs351111	19	0.80	CTCGGTGGCTCAGGTGTTTCTGAACAACTACGACGCGGAGAACAAACTGAACGAC[A/G]TTCTCCTCATCCAGGTGGGCGGGCAGGGCCGCGAGGGCTCGGAGGGGCACGGC	A	G	0.64	0.38	0.47	0.55	0.39	0.41	0.72	0.43	0.32	0.46	0.42	0.015	0.043	0.000	0.000	0.0002	1		
rs757228	19	1.05	TGACTCCAAGAACTCAGCCTCTAGGGGCTGGGCGGGAGCAAAGCCATGTGAACAG[A/G]CTTATGCAAGACCAGGATTCGGCCAGCCCTGCTCAGGGATCCTTGCTTCACTGC	A	G	0.48	0.46	0.52	0.63	0.45	0.36	0.45	0.56	0.32	0.51	0.53	0.001	0.005	0.000	0.015	0.0008			
rs266805	19	1.33	CACGTTTGCGTTTGGACCTGTCTGTGCGTTCTCCTGCGTGGCAGTCCTGATTTCCAT[A/G]CTTCTGGAGAATCCATTTCGTTAACACTGAAAGCCAGTTCTCTTTTCCTGGCAGT	A	G	1.00	0.63	0.78	0.70	0.66	0.61	0.71	0.67	0.61	0.83	0.83	0.104	0.001	0.002	0.007	0.0027	1		
rs197198	19	1.46	GCTTCCAGGCAGCTCTGAAATACGTCCCACCTGTGCAGCTATGTGACTCCCTCCC[A/G]CACACGCTTAAGACACCTCTGCATGCAGTCAAAGCCACTGTCACAAGCCGGCAGC	A	G	0.40	0.58	0.55	0.50	0.64	0.55	0.94	0.43	0.50	0.57	0.45	0.006	0.142	0.004	0.001	0.0016			
rs1875960	19	1.49	AGCCACTTCTCCCTCTTTTATGATGAGTTTCTGGGGTTGATAACCGGAACTTGGAAGT[A/G]GAACTTCATGCAGAATCATGCTGCTGGCAGGGATTCCAGAGAATACATGCCCC	A	G	0.43	0.32	0.28	0.33	0.32	0.05	0.39	0.26	0.29	0.24	0.31	0.036	0.036	0.008	0.019	0.0019			
rs887270	19	2.50	GAACTAAAGGAATTGGAGCAGTGGACCTGGCCTGCAGGGGGCACGGGAGAG[A/G]TATCTGCTGAGTCGCTGAGTAGGGGGAGCTGAGGATCAGGGAGGCGTCAGTGGTGGC	A	G	0.86	0.52	0.55	0.56	0.59	0.82	0.79	0.71	0.61	0.57	0.53	0.049	0.015	0.006	0.029	0.0003	1		
rs1688128	19	2.98	TCTGAGAGGGGAGACAGCCCAGTCCACTGGTGATGGCAGTAGGAACACAATTCCC[A/G]TCTGTGGAAGTTTGCTCTGTGTAGGGAGAGGCTGACAGCGTTCATGCACCATCTC	A	G	0.36	0.55	0.54	0.60	0.61	0.77	0.47	0.57	0.64	0.49	0.42	0.019	0.009	0.001	0.028	0.0087		1	1
rs306048	19	2.99	CTGGTCATTGCAGCAACCCCATCACCCACACCACGTGTAACCCCTCTGTACC[A/C]AGTTGGGGGTTCACCCTCCTAGGATCGCCGTGGACTCCCGTCTCTATCTCCACCCTCC	A	C	0.67	0.45	0.51	0.37	0.52	0.32	0.15	0.47	0.39	0.53	0.58	0.016	0.068	0.000	0.015	0.0029			
rs4900	19	3.07	GTTGAGGAAGAGGATGACGGAGGAGTTCTGGAACCAGGGGTAGGTGATGAT[A/G]GTCCGGAACAGGGCTTTGCTCTCCTCCATCCGGTTCTGGCGGGAGCGAAGGCAGAGG	A	G	0.29	0.46	0.45	0.43	0.46	0.45	0.33	0.45	0.36	0.41	0.46	0.009	0.007	0.000	0.000	0.0005			
rs1715093	19	3.51	GGGTCGTCTCGGGATTGTGGGGGAGCTCCCCAGGACTTGTTAAAGGGCAA[A/G]GTGAGTCCTTAGCCACCAGCCCCAGACTTGGGGGTGAAATACAGAAGTGGGTGCCATT	A	G	0.48	0.47	0.59	0.60	0.50	0.18	0.09	0.48	0.29	0.50	0.48	0.009	0.152	0.007	0.090	0.0066	1	1	1
rs2108389	19	3.54	AGCCGTGTGGACCTGGGTTTCTGAGCTGGTCTGAGTTCTTGAATAGGATTTG[A/G]TTTGGGAGCTGTCTCTGAATCCTGAGCTGACTGGGGTTCCAGAGCTGGGCTGCTTCAT	A	G	0.14	0.42	0.48	0.34	0.41	0.82	0.88	0.63	0.61	0.46	0.45	0.062	0.087	0.018	0.071	0.0005			
rs2052059	19	3.75	GTGTTTTCCTCCTACTCTCCTGGGTAATTAGAGGTTTGGCTGACAGTATTGT[A/G]GGTGGATACTTTTCTTTTGCCCAATGTTTTCCAGCTTCTAGTCTTGGGCAGCTGAATGATC	A	G	0.50	0.41	0.43	0.44	0.52	0.27	0.06	0.38	0.32	0.45	0.52	0.005	0.108	0.002	0.013	0.0044			
rs352500	19	4.14	TTTTTTCCCCAAGCATTCCTCAGAAGTAGTAGCCACTGGTAAACCAAGCCATAT[A/G]ATTGCTTAATTTCCAAAGCATAGTTATATTTAATATTTAAGTAAATCTTTCAAGAAGTGT	A	G	0.60	0.52	0.50	0.50	0.61	0.45	0.06	0.51	0.46	0.49	0.48	0.006	0.132	0.002	0.002	0.0001	1		
rs243404	19	4.34	ATGCCACAGGGGACCGCTCACTGCCAGGCTGTGTGAAAATGCAGAAACTTGGA[A/G]ACTCTAAATGTCCACTGGCTGGAGAACGGATAGACACACTGTGGAATAACCACGAGC	A	G	0.17	0.21	0.27	0.28	0.38	0.55	0.74	0.29	0.29	0.27	0.28	0.008	0.099	0.000	0.035	0.0002			
rs741923	19	4.40	CCCCATCCCTCCTGCTCTCGCAGCCCCATGATGGACGTTCTGGAACCACTCCC[A/G]GGCCTCATGTTTACAAAGAGACCGAGAGGCCCATCGTCCAGGGTCACAGGATGGC	A	G	0.29	0.51	0.40	0.43	0.39	0.36	0.09	0.46	0.61	0.42	0.40	0.007	0.065	0.001	0.000	0.0006			

rs1547429	20	47.23	ATTTGAGTGGGGACACAGAGCAAAACCATATCAAGATCCAAACTCACCATCTCAAT[A/G]AGGGAAAGGCTGGGGGCCCCTAAACTTCTGTCCTCAGAAGGGGACCCCTAAACT	A	G	0.24	0.44	0.44	0.38	0.43	0.77	0.69	0.51	0.50	0.42	0.45	0.023	0.023	0.000	0.048	0.0001	1		
rs761272	20	48.00	CCAAACACTACTACAAGGAGGGGCTGATCGTCTCACTTTACAGAAGAGAAAAC[A/G]AAGGCTCAGGGAGGCCCTCTAGAGGAGGAGCAGTGGGAAGCCCACACACGGGC	A	G	0.21	0.61	0.54	0.57	0.52	0.45	0.47	0.29	0.65	0.52	0.43	0.058	0.000	0.017	0.002	0.0079	1	1	1
rs968478	20	48.03	TTCTCGCCTCTTCCCTCTTCCTACCCAGGCTCCTCAACATTGTGTGCCCACCCC[A/G]AGTGGGAGTTCAGCTCAAACAAGAGGGCTCAGAGGGCCACGGGGACCCCTGCAAG	A	G	0.36	0.71	0.63	0.59	0.59	0.55	0.64	0.49	0.61	0.57	0.52	0.032	0.002	0.003	0.000	0.0065			
rs911411	20	48.40	ATAGTATTTAGGCACAGTTTTGGGGTGGGGGTCATTGAGGGGAAAGGAAAAGTA[C/G]AGTAGAAAAATGAACTACTTTAGAACAACAAGGCTAACAGGCAAAAGCAATGAATCA	C	G	0.36	0.41	0.41	0.46	0.63	0.32	0.35	0.34	0.46	0.46	0.45	0.004	0.003	0.001	0.008	0.0004			
rs756529	20	48.70	GCCTGCACTGGGGTCTGGCAGGAGCAACCAAAGCCCAGGAGTTGACACATAAAGT[A/G]AACAAGCGGTCAGATGTCAGCATTTGAACAAAATTCCTGAGGATCCCACTCTCT	A	G	0.48	0.43	0.46	0.38	0.25	0.50	0.50	0.40	0.50	0.39	0.41	0.000	0.001	0.012	0.001	0.0021	1		
rs119416	20	48.72	ACTAAATCAAAGTGAGAGATGTTGACTTAGTGTAGCCATGGCCTGATCTGAATC[A/G]ACGTTAAAGTTTCCCAACAGCCCATGAGATGGTAGAAAGGATGAAATGATACTATTA	A	G	0.26	0.27	0.33	0.37	0.41	0.27	0.61	0.41	0.25	0.35	0.41	0.002	0.032	0.000	0.003	0.0038			
rs610412	20	48.76	AATGTGCCTGCCAGAATTCTGATCATGAAGACTGCCGAAGTGTTCTGACCTTG[A/C]ACCTCAGAATTCCAACCCCGTACATTAGTTCCAACTGGGACATCAGCATTCTGACCT	A	C	0.79	0.64	0.58	0.57	0.64	0.82	0.78	0.59	0.71	0.62	0.54	0.029	0.031	0.010	0.054	0.0013			
rs718630	20	49.80	CATCTAGCACCTCCCCCCCCATTACACACCTGCTTTGTGCTAGGCACTTTAC[A/C]TGTGCAACAGGATGATGTTAGGGGTGCTCTGAGTCTCTTGGCCTAAGTTCAAAGGTCAT	A	C	0.45	0.61	0.54	0.51	0.52	0.50	0.43	0.55	0.68	0.64	0.59	0.010	0.015	0.002	0.005	0.0017	1		
rs995480	20	50.46	TGATCATTAGAAATAATAAACCATCATACCAAGA[A/G]CTGATGCTCACTGAGAGCATGCAGTCTACCTGCCGGGTGCTGAGGGCTTCACATGCCCTCTTCGATGAGACTTCGAA	A	G	0.26	0.51	0.46	0.57	0.55	0.59	0.42	0.57	0.54	0.49	0.49	0.019	0.004	0.003	0.005	0.0008	1		
rs926910	20	50.63	TGAGAGCCCCAGAGTCCACTCTGATGGCTTTTCCCAACTTCAGGTCATGGG[A/G]AGCAATGATGGATATCTCAGTTCCTTCCTTCCCCTAGAGAACCTGGCTCCACATGCAAAC	A	G	0.33	0.59	0.53	0.59	0.54	0.64	0.73	0.38	0.71	0.61	0.54	0.013	0.012	0.019	0.002	0.0001			
rs228835	20	50.75	CATTTGGATGTTCTCTGAAATTTAAAAAATGTGTAACCCTTCAGGCAAAGTTCAC[A/G]CTGGGAAGTCCAGGCTGTTCCTGACTTCAGTGGGAACCCTGGCCCTTGGCCACAGG	A	G	0.26	0.63	0.61	0.56	0.61	0.41	0.63	0.61	0.75	0.54	0.52	0.060	0.000	0.001	0.024	0.0024			
rs737116	20	50.97	CTCCAAGGAAGAGTTTGCAAGATGACCCACTGGGGTACAGGAAGCG[A/C]CTCTTTACATTTCAGTTCCATCTCCAAAATTAATGCAAAATTAAAATCTTACTAATACTATTGAGG	A	C	0.71	0.52	0.48	0.44	0.41	0.14	0.22	0.42	0.61	0.45	0.52	0.027	0.043	0.004	0.071	0.0017	1		
rs1022689	20	51.51	TGGAGGTCTGGCCAGAACTCCCGCTGAGTTCATTACCAGGTAGTTGTTCTGATTT[A/C]TGTCTTTATATAACCTAGTTACTTCTGTACTTTCTCATCTGTGCCCTTTTAGTGAATG	A	C	0.26	0.33	0.23	0.26	0.39	0.45	0.30	0.30	0.36	0.34	0.30	0.000	0.001	0.002	0.025	0.0014	1		
rs200613	20	52.64	ATTTCCAAAGACAAAGAAACTCAGTGGGATTGCATGGGAGAAACTGTGTTG[C/G]GCACTCCTATGAAGGATCCCCATGTAGCCTTCACAGTTTCACTTAGATTCTGTCTCTTC	C	G	0.29	0.48	0.56	0.46	0.50	0.55	0.88	0.71	0.68	0.62	0.60	0.051	0.040	0.013	0.004	0.0008	1		
rs1476720	20	53.21	CTAGCCCAGGGCAGGGATGTGTGCTGGAGTTGAAGGCCCTCCCTACAAAGGCCACCTGCAAGGCCACTACCACTTC[A/G]GAATTTGTGAAGGCCTGCATCGCTCCAGTGC	A	G	0.69	0.68	0.77	0.72	0.84	0.32	0.40	0.72	0.64	0.74	0.74	0.014	0.130	0.018	0.155	0.0002	1		
rs394732	20	53.36	ATGAGTGTTCCCCAAAGAGTTGAAGACCAGAGAATGAACCAGAGCAGAGACTTAC[A/C]AGGTAGTGTAGTAACCATTACAGGGGCATCAGCAGAAAAAAAAAAAAAAAAAAA	A	C	0.79	0.43	0.42	0.44	0.43	0.73	0.20	0.54	0.43	0.36	0.45	0.092	0.021	0.016	0.078	0.0021			
rs964293	20	53.50	TCCAGTCAATATACTGACCATTTCTGTCCTTATATCTTCCTCACGGAGTGTTC[A/C]TGATGGTAGTCACACAGTCTTTTATTATAATTTTATGTAGTTCTTGTTGGAGCGATTAAG	A	C	0.60	0.35	0.33	0.39	0.30	0.59	0.19	0.35	0.43	0.38	0.36	0.015	0.031	0.001	0.016	0.0004			
rs1418927	20	53.51	CACTCCCCAGGCCCCCAAAACTTTCTACTCCTAAGCTGTTTTATTTTCATCATCAC[A/G]TTGTCACCATTGAACATTATCTTGTTCATCTTGTTTTTTGCTCTCTTCCTCCACCAG	A	G	0.29	0.29	0.22	0.18	0.29	0.14	0.04	0.40	0.32	0.18	0.24	0.000	0.055	0.008	0.013	0.0000			
rs767971	20	54.39	GGATAATCACAATTCAGAGGTACAGGTGAGAG[A/G]TCCAAAGAAACTTCAATGTCATAAGCAAGGGAGGAAGTTGTATCTGTGTCTCTAGAGAAATGTGAGAGGCAACATTCC	A	G	0.17	0.81	0.77	0.73	0.66	0.82	0.97	0.80	0.82	0.69	0.74	0.217	0.034	0.001	0.000	0.0021	1		
rs914433	20	55.20	TGTGAGTGCAAAGAATCATAAGGTCTGGAAATGTCTGGGCTGGAAATACTCCAGTT[A/C]AGCTTGGACTGTGGACATTCCTCCTCCCCCAAAACCTTTGTGAATTTTATAATAT	A	C	0.10	0.39	0.50	0.44	0.46	0.14	0.00	0.39	0.36	0.53	0.52	0.114	0.230	0.006	0.090	0.0001	1		
rs1884910	20	55.71	TCCGCCGCTCCTTCAGTGGCTCCCCATTTCTGTTACCACTCAAGGTCCTTCA[C/G]GATTCTTTTGAGCCTCATTTCTTTCGACTTCCACCACGCTGACCCCACCTGGTCTGCCA	C	G	0.64	0.38	0.34	0.37	0.59	0.36	0.57	0.33	0.39	0.38	0.31	0.044	0.029	0.001	0.000	0.0004	1		
rs1570530	20	55.84	TCTGCTCCAGGAGCCCCTGAGAGCGTCAAAAAGAACAAGGTGGGTGGGTTG[A/G]GGGGAAACATCACTGAGAGGCTACCTAGGCCTGGATCCCCGGCAGATGCAGGTCCAC	A	G	0.43	0.47	0.45	0.39	0.48	1.00	1.00	0.83	0.32	0.47	0.48	0.000	0.274	0.095	0.274	0.0012			
rs186659	20	56.49	GCATGTTCATTCATCCTCATTGTCATATGATAGTCCATCGTTGTATTAGACC[A/G]CAATCCATGTATCCATTCTACAGTCATAGGCGTTCAGGGAGTTTCCAGATTGGGGCTCTC	A	G	0.93	0.56	0.60	0.57	0.52	0.14	0.56	0.60	0.57	0.73	0.63	0.101	0.000	0.002	0.095	0.0005	1		
rs1328757	20	56.82	CTCCTGCAGTCCACTGGCCACTGTTCTCTTCCCTGGCAAGTCTTAGAGTTTAGG[A/G]AGTGGAGGGGGCTTGGCAGTTTTGCTAGATTATATGCTTAAGCAATGAAACCTTTAT	A	G	0.31	0.45	0.48	0.45	0.34	0.55	0.06	0.46	0.39	0.45	0.50	0.015	0.114	0.001	0.004	0.0005			
rs511145	20	57.59	TGCTTGGCCTACCTACTAGCAACTCTTGGGCTAAATAATTGTTCAAAAAAAAGA[A/C]CAGGAAGAAAGGAGGAAGAGCAAAAGTAGTTTTATTTTACACACATATTAGCATTACC	A	C	0.26	0.59	0.53	0.63	0.50	0.59	0.36	0.31	0.54	0.59	0.66	0.021	0.010	0.017	0.008	0.0152	1	1	1
rs910860	20	57.69	AAACTCTTTTATAGTCTGCCATGGTAAAAAGTACATAATTTCTGTCACAATTTGCAC[A/G]TATGGAGCTGAAAGAACTAATGATTAGCAACATTCTGTCATGTTTTATAAAGTTCC	A	G	0.69	0.67	0.69	0.66	0.70	0.64	0.98	0.65	0.57	0.67	0.74	0.001	0.111	0.005	0.000	0.0024			
rs2208087	20	57.77	TACAGCCTTGGTCAGAGATAAGTGGCACAGCTGGCCACAGTGATTCATCTTC[A/G]AGAAGCACTTCACACAGTAGCAAGAGGAAGTCTCCATTGATAATTTTTTTTGTTTGTTT	A	G	0.12	0.54	0.53	0.60	0.64	0.18	0.75	0.50	0.79	0.49	0.49	0.124	0.015	0.006	0.085	0.0000			
rs259998	20	58.46	TGCAGCTGAGGGTACAGGACTCAAAGCCCAGAATGTTCTTCAGTGAGAGGCAC[A/G]TGGAAGACCCAACCTCCTTTCCTGCATAAAGCTGAGACCTCTGAAAGGCATACTCTC	A	G	0.45	0.79	0.77	0.73	0.79	0.77	0.79	0.73	0.61	0.82	0.79	0.051	0.000	0.002	0.000	0.0002	1		
rs259966	20	58.50	TATCAGTTGGAAAGAAAAATAAGAGACACATAATCATATTGGTTCTAAAATTCA[A/G]ACTCAAAATAATGAGGAAAACCCCCAAAGTGTATTATACCTGAATATGCTCAGGCAAA	A	G	0.29	0.48	0.50	0.44	0.57	0.14	0.41	0.24	0.57	0.43	0.46	0.031	0.006	0.056	0.095	0.0010			
rs1407538	20	58.54	TTCACTCATCCAAAGCCATCAGTCACCATTCACCCACTTTTCTGTTTGGAGTTATTTA[A/G]TTTCTTTCTTGAATGCTTCCCCATCTTGCTCAAATTTGCACCCTGTCTCTGTCCTTC	A	G	0.67	0.70	0.72	0.60	0.73	0.23	0.59	0.40	0.68	0.63	0.70	0.002	0.015	0.064	0.144	0.0001			
rs155004	20	58.56	TATAATGTGCTGCTTCGGGGTCCAAATGGCCACTCTTTCTCTCTTCCCTGCAT[A/G]GTTTCTACTCCCACCAGAGATGGTTTTGAACGAGCACTTATGTCTTGCCTGCTAGAAC	A	G	0.62	0.59	0.56	0.74	0.59	0.91	0.78	0.88	0.43	0.64	0.60	0.005	0.031	0.116	0.097	0.0021			
rs748543	20	58.67	ACTCCTTATGAGCATCTGAGGGAGAGGAGCTGGATCCACACTCTGGACTCTTGA[A/C]TCCAACTTTGAAATAAAATATTTAAACAGCCCATCTCATGATATTCAGAACAATAGTC	A	C	0.57	0.62	0.58	0.55	0.52	0.27	0.37	0.46	0.57	0.58	0.66	0.001	0.016	0.005	0.044	0.0020			
rs1570160	20	59.43	TGACTTTCCCTGGATGTTGGTTATATAAAGACGTTATTGTCATATGTTATGCTG[A/C]TAATGGTATTGTGGCCATTTAAAAAGTCTTGTATTTTAGAGATACGTGTGAATTATTTCC	A	C	0.76	0.55	0.48	0.54	0.52	0.95	0.89	0.64	0.54	0.69	0.58	0.040	0.114	0.012	0.153	0.0020	1		
rs1381100	20	59.53	ACACAGTTTCCTCCAGCTGTATTTACTATATGCACAAACTGTTGCTTGGCCTGGGA[A/G]ATACTGAACCTGCAATGCACCATACAAAGCCTGTGGATGTAATGGTTAAAATGTT	A	G	0.62	0.24	0.22	0.17	0.16	0.32	0.29	0.25	0.25	0.20	0.21	0.111	0.009	0.000	0.011	0.0000			
rs354731	20	59.64	GATGTGAACAGACCATGTTGGAGAATGCCTGGCATCAGCACTCTGACTTGCAG[A/G]TAAAACAGTCTACCCTAAGAGACATTCTAAGTTTTTTAGATCTTCTCCATACATTTTG	A	G	0.26	0.63	0.63	0.68	0.57	0.77	0.94	0.66	0.57	0.64	0.49	0.078	0.068	0.000	0.009	0.0064		1	1
rs1001583	20	60.74	TCTTTTATTCATAAGGTAATTCTTATGAAAATAATTGGTCATTTTAACC[A/G]CACAGATCTTTCTGGCACACACTGCCAGCAATCAGAGTGGCTTCATTTAGTACATTCAGGTG	A	G	0.69	0.52	0.56	0.48	0.46	0.86	0.16	0.58	0.29	0.60	0.59	0.015	0.070	0.000	0.071	0.0009	1		
rs2026162	20	60.96	TCTAGCTGGGGACGCTCGGTTCCCTCGGAATTTGCACCAACCTCCTTCGTCC[A/G]ATCCTTCCCCTCTCTGCAGTTGGACTGCTGAGCTGGGCTTTGCCCCAGTGACTTGGCC	A	G	0.40	0.49	0.48	0.39	0.52	0.23	0.10	0.33	0.39	0.46	0.51	0.003	0.093	0.012	0.038	0.0013			
rs910928	20	62.45	TGGGTGTGGCTCATCGCAGCATATGTGTGAGTGACTAAGTTCCGGGAGGAC[A/G]CAGGCCTGCCTGACAGGTGCTGGTACTTTTCCAGGCCTGGGGGTGGCGTGACTGACA	A	G	0.48	0.47	0.42	0.40	0.36	0.09	0.33	0.26	0.43	0.41	0.44	0.005	0.008	0.031	0.087	0.0014	1		
rs736379	20	62.50	CGGGAGAGGACTTCAGAGGTACGAGCGCTGCTCTGGTCTCCTTTCCTTGCTTCT[A/C]CTTCTCTGCCAAGGCGATCGCAGTTGCTTGCTGTCTTGCTGTGCTGTTACGGCAAT	A	C	0.21	0.46	0.47	0.51	0.36	0.55	0.69	0.63	0.54	0.45	0.51	0.037	0.033	0.012	0.003	0.0003			
rs816943	20	63.40	TCTTCACATGTGCCCTCCTTGCCTGCCCCAGCAGGACCTTGCTGGGGTGAA[A/G]TGTGCTCTTCGTCTGGTGTGTGGCTGGGTGTGTATGTGTGTCTGTGCTGGTGCTGGGAG	A	G	0.31	0.63	0.52	0.48	0.59	0.95	0.61	0.39	0.33	0.53	0.54	0.017	0.010	0.007	0.153	0.0016	1		
rs379042	20	63.52	ATTATGGACTGAACCACTGGCAGAGAAAGCAGAGGGGGGTGAATGTGGTGGTTGT[A/G]ATGCTCTCTGCCCTTTTGTGTGTTTTGCTTTTTAAAAAACTCAATCATGCTGCAC	A	G	0.17	0.29	0.31	0.21	0.30	0.32	0.44	0.27	0.43	0.26	0.26	0.008	0.002	0.006	0.000	0.0003			
rs912239	20	63.57	TGTTTGACACCCCTGCCTGGCCTCACACGGGGGCCAAATCTGCGGTTTGACATTTTATC[A/G]GTGATTTAGATCAAAGAGGGAAAGGATTGAATTAGCAACTGACAGGAAGCTC	A	G	0.31	0.25	0.23	0.23	0.23	0.14	0.13	0.26	0.11	0.23	0.23	0.010	0.003	0.010	0.001	0.0002			
rs990141	21	15.01	AAACATGTGATATGAACGAGATGGTGCAGATTCTTCTTTCTCTGTTTCTCCCCAACT[A/G]AGTACAATTACAAACCCTGACAATTACATACAGCAACCATAGAATGACACAGAG	A	G	0.52	0.65	0.61	0.57	0.61	0.50	0.75	0.64	0.61	0.57	0.56	0.004	0.025	0.002	0.006	0.0007	1		
rs926130	21	15.14	GCAACTAAAAGTCACAATACCCAAATAACTATTTCCCATAAGTGTTTTCTCCAGCT[A/C]ATCTAAATATTGTAAAGAAAAGACAATGATATGGTAGTACTGGGAAGGGAAGACCA	A	C	0.52	0.54	0.51	0.56	0.70	0.32	0.56	0.46	0.43	0.58	0.48	0.004	0.018	0.000	0.000	0.0000			
rs2178895	21	15.26	TCAAGGATGTTTTGCCCTTCTTAGAAAAATAAAGGGGACAAAAAAGAAGGTGGCT[A/G]GCAAGTTAGTGCACTAGAAACAGGGTAATTAAGGCATAACTCAAAGTGACAATCA	A	G	0.95	0.66	0.67	0.63	0.68	0.95	0.76	0.49	0.71	0.63	0.60	0.079	0.002	0.014	0.076	0.0014			
rs1882959	21	15.47	TGTCTCCAAAATAAAAAAAGTGTTTGATGAAGAATGTTTTAAGTCCACAGAAGACATA[A/G]CCCTACATGCGCTGGAAATCTAATTGACTTTTTGTTATATCTTGTATCAAATTAAT	A	G	0.95	0.77	0.76	0.71	0.70	0.95	0.70	0.76	0.68	0.82	0.80	0.061	0.000	0.010	0.071	0.0043			
rs7275650	21	15.62	TCTTCCAACGTCTCCTGTAGTTTAGCCATCCTCCGTCTTACTACCTCCCTTACTAC[C/G]CCCATGAGAGAAGATCGGATCTCAATCAGTTGTCCTTTTGCTTTATAGTGCTATTTT	C	G	0.17	0.12	0.11	0.10	0.04	0.05	0.04	0.11	0.14	0.08	0.11	0.002	0.007	0.003	0.008	0.0000			
rs8127275	21	15.79	TTCCCTGTAAATAAATTCCTCTGTTCATTCAAGCACTTCACCCAGCTCTCCCTCT[A/G]TATAGTCACCTCTTTAACGTGATTCCTCCTTAAGAGATGAGACTTCCTATTTTGTCCT	A	G	0.64	0.99	0.96	0.99	0.96	0.86	0.96	0.99	1.00	0.96	0.99	0.080	0.002	0.022	0.008	0.0043			1
rs2823392	21	15.92	AGAGAAAGCATTCTTCAGGAAAAGGGGACTTGATTGTCTTTTCAAATTAAACAGAGA[A/G]TTCATCCATCAATGTTTCGTACCTTCCCAGCTAGCAAATTTCTTTGGTATAAA	A	G	0.57	0.27	0.30	0.21	0.32	0.09	0.06	0.16	0.18	0.31	0.25	0.025	0.073	0.025	0.056	0.0021			
rs983530	21	16.50	TTAACTCTGAATCTCCTTCCAATGAGCTACGCTCCCTGCCCTATTTCAACTACT[A/G]TTCTAAGCTTGCAACTCAACTGCAAAATCTCGACCAAGGAGGAGATCTCAATTTTGT	A	G	0.76	0.67	0.63	0.59	0.82	0.95	0.72	0.77	0.68	0.70	0.61	0.009	0.001	0.005	0.071	0.0007	1		
rs189457	21	16.59	TTTTAGTCTACATCAAAAAGATCTAAAGGGTGTGGCAGATAGGTACCTCCC[A/G]AGAATGTATACAAGATGTGAAAATAATGTTTCCCTACATACTTTCACTCACCCACCTGC	A	G	0.74	0.67	0.65	0.65	0.57	0.27	0.51	0.32	0.50	0.63	0.55	0.003	0.016	0.084	0.089	0.0069	1	1	1
rs2824056	21	17.09	AATAGAGATTCTTAGTACCATACAATGGCTCGTGTATAAGTCTGATGCTGGGATAC[A/G]CACTTGCATGCATATCTATATACCAGAGAGAGAAGCCTTGAAGTATAGCTTAAGG	A	G	0.36	0.54	0.60	0.73	0.54	0.55	0.72	0.54	0.61	0.58	0.63	0.025	0.014	0.000	0.000	0.0008	1		
rs202855	21	17.55	CATGATTTCACTGCTTGCCAGGTTAAACCTGCAAAAGATGGTGTATCCATATTTC[A/G]TGACTTGGCTCTGGCCAGTTAGAAAAAAGACAAGCATATGGTATTACTTTCCATGAC	A	G	0.71	0.87	0.84	0.87	0.79	0.95	0.97	0.92	0.93	0.84	0.75	0.010	0.035	0.006	0.023	0.0060			
rs1389157	21	17.71	TGAAGTAAAGCAAACCAGTAGGGCAGGATGGAAGTGTCAAAACTGATTGGCCGGTAT[A/C]AATTTTCAAAATGTGTGGTCAGTATTCCTGGAGGCCAAATCCACTGGGTGGCTT	A	C	0.36	0.03	0.02	0.02	0.07	0.05	0.00	0.00	0.04	0.03	0.05	0.123	0.004	0.004	0.005	0.0017			
rs12034	21	17.86	TCACTTATTCTTTTCCCTAATTGTGAATTTTAGTGATAAATACACCTGTACTACTGA[A/G]GAAAATATTCTGACACTTCACGTGTGCAAAGTATAGAACTGACAGTGTCAGTTTCAG	A	G	0.18	0.67	0.56	0.66	0.63	0.55	0.66	0.56	0.58	0.58	0.66	0.075	0.012	0.003	0.001	0.0072		1	1
rs197523	21	18.26	ACTGCTGGTTTCCCCATGTCCTGTTTAGTACTTTCTTTGTTGCATATAGACAAAT[A/G]AAATAGAAGCCAAGAACATCTGGAAACCATCAGTAAATAACAGTTTGGAAATTTCTT	A	G	0.50	0.21	0.30	0.40	0.32	0.00	0.23	0.53	0.23	0.33	0.36	0.019	0.001	0.028	0.117	0.0029	1		
rs6517799	21	18.45	AGCCTCTAACTATTAGACTCCAAATAAATCTTCTGACAGAAGTTAAGAGAAGGAAT[A/G]GCCACCATCATCAGCAAAGATGTAAAGCCTTGATTTGGGACTATCTCATGGTCTTG	A	G	0.81	1.00	1.00	1.00	1.00	1.00	0.99	1.00	1.00	1.00	1.00	0.071	0.004	0.000	0.000	0.0000			
rs2824776	21	18.66	TTATATACCACATAAAATGCAAGCAAATGCCATTTTTGTTTTATTTTATATTGGTCTTTGAAT[C/G]TAAGGCATTAATTGCCCTGCTTGTTTGGCATGGTTTACAGTGGAAAAGAGCATGTGAA	C	G	0.26	0.31	0.25	0.30	0.39	0.23	0.21	0.27	0.18	0.35	0.30	0.000	0.000	0.001	0.000	0.0023			
rs2825276	21	19.32	CAAGGATGGCATAGAGGTGCCTACTGATAAAAAGAATTACACTCAGAATCCAACT[A/G]TCAGGGACTGCTGACTTCTTACTATCTCAGGTGATGGTGAAAAAATATTCCTACCA	A	G	0.33	0.55	0.48	0.34	0.43	0.35	0.59	0.55	0.38	0.36	0.41	0.022	0.004	0.004	0.016	0.0020	1		
rs2825360	21	19.41	AAAGTTACCATTCATGTCTTCCTATCAGTGTAGAAAGACAGGGAGAGTCTCCAGGA[A/G]CTATAAGAATGTAATCAACTTGAGCAATCAGCCTGTTTTATGGCTTTGCAGCCTGT	A	G	0.52	0.40	0.40	0.37	0.46	0.18	0.06	0.46	0.33	0.38	0.36	0.012	0.009	0.022	0.022	0.0000			
rs2825416	21	19.50	AGCATATATGTTTCTCTCCTAGATTAATTGTTTTCTTATTATGTTTTTACAAAATGG[A/G]AAGTCACATATTACAAGTCTATAAACAAATATATATTGAGAGAGAGAAGATTTATTA	A	G	0.43	0.32	0.36	0.35	0.30	0.50	0.25	0.16	0.29	0.35	0.30	0.002	0.007	0.025	0.010	0.0039			
rs1808093	21	19.68	AACTGATAGGTTATATATTTTTGTATTTGTTTATTTTCTGGGTGCACCCACTG[A/G]AACTTTGTGTCATGGTTCTTCATTATGCTTAATGAATTGAAGTTATTAATGCCATTGTGT	A	G	0.38	0.48	0.41	0.39	0.32	0.18	0.26	0.28	0.39	0.42	0.45	0.001	0.032	0.017	0.046	0.0015			
rs2825875	21	20.19	AGTTAGATAACACCTATCAGTAATACCTTAGATTATTTCTCAATGTAGGCTGCAA[A/G]CATTAAAGGGTCAAGCAGTTTTCACATTTTAAGCAATTTACTTTTAATTAACAAAAAA	A	G	0.26	0.65	0.63	0.57	0.55	0.36	0.39	0.47	0.42	0.64	0.63	0.038	0.014	0.010	0.023	0.0012	1		
rs1888415	21	21.22	AAATGCCATGCTAGACATTCGACTAGCCTTCTATGGCTAGACTACTCGCCATAAAGAG[A/G]CACCTCACAGAAAAACAAGAGGCTTGGGAAAAGTAGAGTGACTTGGTAAAGG	A	G	0.05	0.20	0.19	0.18	0.18	0.00	0.42	0.14	0.29	0.24	0.26	0.020	0.036	0.007	0.075	0.0021	1		
rs2258630	21	21.90	ACAAAGACCTTGACTGTTCTGCAATCACCACTACCCCACAGAGATGCTACTTCT[A/G]AGTTGGAACTTGAACTTTACTGCTGTTTGTAACACCAGACACCTCTACTATCCCCGA	A	G	0.64	0.41	0.38	0.44	0.34	0.32	0.69	0.31	0.38	0.35	0.30	0.041	0.054	0.005	0.000	0.0033	1		
rs1783400	21	22.03	GAAATGAGTTCTTATGCTATAATTTTGCTGAAAGAAACTATTTTCTTGCCTCC[A/G]TACTCCCCATAAGATGTAGCAACCTAATCTGACTGCCCTGAAGTGAGCTTGGTTTACTGG	A	G	0.40	0.51	0.47	0.41	0.50	0.41	0.30	0.48	0.62	0.36	0.41	0.002	0.005	0.008	0.000	0.0009			
rs1155463	21	22.06	TTTGTTTCATACACACTAGTCTCCCTGGATAATTCCAAATTGGAAATATAGAGGAG[A/C]TCCTTGACTGAAAGACCTGTACCTCAATGACTGAAGTCCTCTGTAGTACTGAGCAC	A	C	0.55	0.38	0.32	0.33	0.23	0.36	0.16	0.54	0.43	0.25	0.27	0.027	0.013	0.025	0.001	0.0021			
rs767055	21	23.26	TGTTGTACTTTGAAATATCCCATTGGTGCCTAGTGTTTTTTCATCAACCACACGCT[A/G]TTGGCTTTTTCATGGATATTTTTGGCCTAAAAAATAATGAACAGGTGGTGTTGTGGT	A	G	0.64	0.64	0.61	0.71	0.66	0.68	0.52	0.71	0.77	0.63	0.60	0.002	0.003	0.004	0.003	0.0000	1		
rs1892776	21	24.63	GCTTAGATACAGGCGAAGAAGGAGGAGTGGGACTTGAAACATTAAAATTTGTCTTT[A/G]TAAGCTATATGCCTGACATTCTATGAGAGCTGTAGCCTAGGCCAGTAAGAAAGTTT	A	G	0.26	0.59	0.57	0.57	0.55	0.45	0.84	0.65	0.71	0.50	0.51	0.044	0.062	0.010	0.002	0.0004	1		
rs2829150	21	24.82	ATTGAATTTGGGCTTTGATGAAGGACAATTAGTGACACCCCTGTGTAGATAGATACCC[A/C]TTGCTATATAACAAATTATCATGAATATTTGTATCTCAAAAATAAATATTCATTTTA	A	C	0.76	0.54	0.53	0.60	0.55	0.64	0.55	0.48	0.59	0.63	0.60	0.037	0.000	0.007	0.005	0.0055			1
rs1041731	21	26.15	TTTTCCACGCTTAGTATCAGAACTCTCTACTTTTGCTTGTTTGAATCATTGC[A/G]TTAGCTTTCTAACTGGCTTCTTTAATTCCTATTTGTATATTTAAAATCTGTCTCCACTG	A	G	0.62	0.59	0.58	0.51	0.54	0.41	0.24	0.41	0.64	0.60	0.58	0.001	0.051	0.022	0.014	0.0000	1		
rs2829966	21	26.18	GGCAAAGGCGTTCTGCTCCAAGATGTCAAAGTGGGGGAGAAAATTATGGGTGTTCT[A/G]CAATCTTGGCAAAAGAAGAGTACTGTACTCCTTATCTTTTTACTGCTTCTCCATG	A	G	0.64	0.58	0.54	0.48	0.57	0.23	0.60	0.56	0.57	0.46	0.55	0.000	0.002	0.002	0.089	0.0008			
rs2830072	21	26.42	ATATCTTGAAATTTGCTTCTTAGCTCTGTTTAGAATCATTGTGATTCACGGATAGTA[A/C]ATTGTCAGAGTGGACTTCATGAACCTTATATCAGACATTCTGAAAAGAGCTAAAAT	A	C	0.88	0.45	0.51	0.41	0.41	0.50	0.92	0.49	0.27	0.46	0.47	0.078	0.086	0.001	0.001	0.0008			
rs2830252	21	26.78	CCTTGCAAAAATTCCTAGAAATCTTTCCCACATTATCAGTAGCTTTTTCATACTC[A/G]AACATAGTAAAATAGCTTATCAGTTCTGTTCTGCTCCCCTGATGATTTCCTTCTTACAG	A	G	0.81	0.43	0.54	0.59	0.57	0.86	0.69	0.57	0.46	0.57	0.63	0.058	0.022	0.003	0.085	0.0054			1
rs423048	21	27.16	TGTCATCGTATTGTTACTGGGGGGTCCTTGCTCATAGAGCTTCCAAGATGGC[A/G]GCAGCCTCGTGTTCTCTGACCTGGGGTTCTGGCCTCACGGATTCCAAGGAATGGAAT	A	G	0.55	0.46	0.46	0.39	0.48	0.60	0.36	0.41	0.46	0.45	0.43	0.000	0.012	0.001	0.003	0.0024	1		
rs2831032	21	27.92	TATTTTTGTTAAAGAATGTCATCCGAGCCATTCCAAACATGCCATAGAAGCAACTG[A/C]CTATTGAAGGAGACACCCTTGCTTTGACATTTTATAACAATGATCTATGTAATACT	A	C	0.48	0.68	0.69	0.71	0.79	0.55	0.53	0.65	0.68	0.64	0.66	0.024	0.016	0.001	0.009	0.0003	1		
rs1964799	21	28.30	AACAGGAGATAGTCAGCTGGAGGAACTGACCCCCAGAGCGGTGACATTTGACTGCTCCA[A/G]TGGATGTACAAGTGAGGTAGGATGTGCCCCTTTCCCTC	A	G	0.81	0.67	0.68	0.68	0.59	0.64	0.71	0.66	0.54	0.62	0.67	0.032	0.007	0.009	0.001	0.0025			
rs2831343	21	28.31	TGTATAAACTGTCACTGGCAGGTCCTAAAGTACCTTTTCATGTGGGCATTCAGT[A/T]GATGACATTTTCCAGGTTATGAGCATAGATTAGAAGTTATGAAATTCAGAGCCTAT	A	T	0.36	0.42	0.47	0.50	0.45	0.50	0.35	0.40	0.29	0.40	0.39	0.001	0.003	0.000	0.002	0.0001			
rs2831341	21	28.31	TTGTTTATTGATTTTGATCTGAGGATGGAGGCTTAAAAACGTTTTTGTTTTTTCTT[A/G]GCCTGTATGAAATCCACTGCGATGCAACCCTGGGATAGTGAATTGGATTCCTTTAGCT	A	G	0.81	0.67	0.69	0.68	0.59	0.73	0.71	0.66	0.58	0.62	0.67	0.029	0.005	0.007	0.009	0.0018			
rs2254796	21	29.26	TGCATTATAAAACGTAAGCATTCAAAAAAAGCAGATATTACTGCCGAGGACTCT[A/G]AAGAGCTGGTTTTAGTTCTCTCTGTTGACAGCCTAAGAAATAATTAATAACTGGATTA	A	G	0.19	0.44	0.48	0.44	0.30	0.14	0.24	0.53	0.57	0.44	0.43	0.058	0.042	0.001	0.075	0.0018	1		
rs2832181	21	29.40	CATAATTTGGAAATGAAATGATGAACAAGGTTTCTCTGATAGGATCCAGGAGGTTA[A/C]GAATGTCAACCTAGTAGGAAGTAGAATTTTCTGATTCTAGAAAATTATTTGGGTC	A	C	0.38	0.50	0.45	0.48	0.64	0.59	0.61	0.35	0.43	0.52	0.53	0.005	0.010	0.007	0.004	0.0037			
rs2832191	21	29.41	TAAAGGGCCCTGCAGAACACTTGTCTAATTTGAATTGTTTAGATTAATTTGGCA[A/C]CTGGTGAAATATCACTTTGATTCTAATTATTGTCATGCTCACCTTGAGTAAGGCTTT	A	C	0.64	0.54	0.58	0.59	0.43	0.41	0.38	0.70	0.57	0.55	0.49	0.002	0.024	0.004	0.014	0.0042			1
rs15092	21	29.64	AATAAACTATTCTTTGAAACTTGTTGGGTAGAATGAAAATTAAAGCCATAATGGTA[A/G]AAGATGGCATACTGATTATAAAAGAAGCAGAAAAACATTGATTTTTTTATATCTTTCA	A	G	0.90	0.60	0.55	0.59	0.68	0.86	0.92	0.67	0.50	0.64	0.62	0.088	0.085	0.008	0.058	0.0020			
rs2832411	21	29.91	TATCTCTTGTGTACACTAATATATCCCAACAAATAAGGACGGTCGCAGAACTAGTA[A/C]ATCCTAACTAAGTTCTTGTTGAATGAGCGATAGAATCTATCAAGGTCAATTCAAAT	A	C	0.86	0.40	0.42	0.37	0.36	0.41	0.67	0.54	0.36	0.35	0.40	0.114	0.035	0.011	0.000	0.0001	1		
rs145472	21	30.17	GTGGAGGATAGAAATGAAACCACAAACTCATTTTCCAAACAGTAAAACCACCCAAA[A/T]GTCCCATCTCTTCCTAAACGGACACAAATGCCTGTTATGGCCTTTTTTGATGTC	A	T	0.45	0.71	0.69	0.66	0.77	0.45	0.64	0.46	0.75	0.66	0.67	0.032	0.000	0.027	0.023	0.0011			
rs1977904	21	31.59	GACATGAGATGGGGACCCCAAAAATGTAATCTAAGGCTCAAAGTTAGGTGCCATAC[A/G]TAAACTCAGAGGCTATGAAGAGGGAGGCATGCAACTTAATGAAATTCTTTATGA	A	G	0.18	0.63	0.54	0.67	0.59	0.05	0.41	0.22	0.69	0.47	0.53	0.071	0.010	0.063	0.166	0.0001	1		
rs1041756	21	32.16	CAAATTCATTGTGGTTGACTAAATTTGTAATTCACAGGGAGTCTCGTTGCAGAAGC[A/G]GACGACCATAATAAATGAGAAGGCAGAAAGGAGAAACGCAACAAAACCGGGTCCT	A	G	0.45	0.65	0.60	0.70	0.64	0.82	0.98	0.75	0.71	0.59	0.58	0.006	0.152	0.021	0.046	0.0005	1		
rs762173	21	32.75	AGAGGCTTTCTCTGTCCAAAAACAACAACAAAAAACATCTTCCTCTCATCTCCCTGCC[A/G]CCCCTCTCTATCACAGCATCCTATTTTTTCTTCATAGCAGCATCATCGTTTAGATTT	A	G	0.50	0.64	0.68	0.59	0.57	0.45	0.19	0.55	0.64	0.63	0.66	0.028	0.140	0.004	0.031	0.0002	1		
rs1476415	21	33.52	CTTATAACATTGTTATTATACATTTCACTTATTCATAAGCTATAATCA[A/C]CCAATATGTTGCTGCTATTTATTATTTGGACAATTATTCTCAACCTGATAAAGAACATCTACAGAATC	A	C	0.50	0.46	0.44	0.37	0.30	0.09	0.28	0.34	0.46	0.51	0.50	0.002	0.009	0.001	0.068	0.0033	1		
rs2250226	21	33.55	CTTTTGGAAAATTTCAGCTTTTTGACTGACCATAGTTATTTCCTGAAGTGCGACC[A/G]GACTGAAAAATCAGTAGTTATATCACTAAGGCTCTCTTGCACTTCCAGAAAGGTCTT	A	G	0.40	0.65	0.67	0.60	0.61	0.32	0.31	0.46	0.61	0.68	0.72	0.045	0.077	0.019	0.065	0.0009			
rs1044195	21	33.74	AAACTCCTTTTACTGAGTTTGCAGTACTGTATTTGCACTGTTTGTATTCCTCTGG[A/G]CCCTTATTGCTACTTTTGCTTTCCTTTGTTACACAGATTTTGTGTTGCACTTTTTCTCCA	A	G	0.05	0.22	0.20	0.21	0.25	0.18	0.30	0.25	0.12	0.26	0.25	0.031	0.005	0.001	0.000	0.0010			
rs2834380	21	34.41	GCAAAAGAGGTTCTCTACAATGAAGAATATTTGATCAAACAGTTCCTGTGGAATCA[A/G]TTTCATAGTTGGGCTTTGCTTATGTCATTAGGCAAGGCTGGCACTTCACATATTTTT	A	G	0.74	0.43	0.44	0.48	0.39	0.14	0.09	0.51	0.39	0.48	0.46	0.047	0.090	0.000	0.062	0.0006	1		
rs1892687	21	35.23	TAAAAAAACCTTTCCATCCAGGGAGTTGAAACATATCTGTGAAACAATAGCTTGT[A/G]GAAGCTGTTGTTTTAGGGAAGATGTAGGAAGATCCCAACCAGCGTCATAA	A	G	0.29	0.45	0.48	0.52	0.43	0.18	0.59	0.46	0.62	0.51	0.50	0.031	0.001	0.003	0.076	0.0000	1		
rs2051179	21	35.25	CTTTTCTCTTTTCTCCTCCCACTTCAGTCAAAGACAGCTTTTTAACCCAGGCCTA[A/T]GACTGTAGGACTGAATCTTATAAAAGTCATCTCTACTCAATTATAGCCAAATGTGTAA	A	T	0.69	0.55	0.51	0.48	0.55	0.77	0.40	0.51	0.36	0.45	0.48	0.027	0.001	0.003	0.060	0.0000			
rs2834949	21	35.80	TTTCCTCTCCCTCCTTGCATTCCCCAGGCCCATCTTTTCTACAAAGTGCTAGA[A/G]TGTCAGTTTTATGGGATGTTGAAACCTTCCCAGTATAAATTCTACCCGCATTATTCTTTT	A	G	0.12	0.34	0.45	0.49	0.45	0.32	0.44	0.59	0.29	0.48	0.47	0.071	0.000	0.011	0.009	0.0005	1		
rs1014803	21	36.01	TTGCTAATTAAAGGCAAAAATGGCCTCTGGGAAGGGAACTCTGAAATGCAC[A/G]GATTGCAAGTGAGATGATGGTGAGGAACTATTAGTATTTAGGAACTCTCTGAAACTTTGA	A	G	0.60	0.47	0.54	0.51	0.48	0.59	0.71	0.55	0.57	0.54	0.50	0.003	0.023	0.010	0.008	0.0006			
rs1012959	21	36.98	AATACTCATGCAAAAGACTTTTTTGGTCTTAGAAATGATCTCCACTGGACAAATAA[A/C]CAGAAATAGAATTACATACTGTTCAAGAGCTGTGAATGCTTCTAAATCCCTTGAGA	A	C	0.24	0.44	0.45	0.46	0.43	0.32	0.68	0.34	0.50	0.41	0.38	0.015	0.039	0.006	0.006	0.0076	1	1	1
rs2835582	21	37.38	AAAATCCTTATTATTGAAGATTCTTCAGGAAAAAAAACCCTTAGTCTGAAACTTTA[C/G]CACCAATCCCCCTTGCCCCCATTGAAATACGTATTTTTAAACATGGCTTTTGATA	C	G	0.60	0.51	0.46	0.45	0.48	0.64	0.75	0.67	0.57	0.42	0.55	0.017	0.071	0.026	0.023	0.0030			
rs2835584	21	37.38	TATTCTTACTAGTTTTTCACTAGAATCAGAACTTTCTTTGTTAAAATAGAGTTTT[A/T]GCCAGCACTTAAATATTCTTAACATTTAACAGGCTAGAATACTTCATTCATTCTTTCT	A	T	0.40	0.49	0.54	0.55	0.52	0.36	0.25	0.33	0.43	0.58	0.44	0.017	0.071	0.026	0.023	0.0035			
rs2835574	21	37.38	ATAGCAGTACAGATGGCAAATGGATTGCCACAATATATCTCTGGATCCATAGTGA[A/C]CCTGCAGAGATAAACCTGTGATGGTCAAACAATGTGAAAACTGCTGTCAGAGACAT	A	C	0.40	0.49	0.54	0.55	0.52	0.36	0.26	0.33	0.43	0.58	0.44	0.017	0.068	0.026	0.023	0.0035			
rs2835601	21	37.39	TTAACCACTTAAGTATTTCTGCCAAAGAACCAGTCAAGGATTTTCTTGTATACCTG[A/C]AAAACAGAACGTTTCCCAAAAATGGGTATTTAGCATAATAATTCAGTGGTGAGA	A	C	0.60	0.51	0.46	0.45	0.48	0.64	0.75	0.67	0.54	0.42	0.56	0.017	0.071	0.026	0.023	0.0035			
rs2835626	21	37.43	GTTCAGCAGGATTGGTTTGGTTGTGTCTCTGACTATCCATATGATCAGAAATTTAT[A/G]TAATTCCTAAGAAAGTGAAACACAGAGGTATAATTTTGCCACAGGAATTTGGGGAT	A	G	0.69	0.67	0.71	0.72	0.68	0.41	0.75	0.36	0.57	0.67	0.62	0.001	0.002	0.048	0.036	0.0032			
rs2835629	21	37.43	GGGGAAAGAATGAGGCAGTTGTACTAGGCATCTGTTAACAATAGCAATTATTAAAGT[A/T]TGGTAGAAATAAATTCTCACATTTTGTACATTTCCACATAATTAATTAACAATATAAT	A	T	0.43	0.53	0.56	0.57	0.52	0.36	0.16	0.34	0.43	0.58	0.47	0.012	0.097	0.023	0.020	0.0028			
rs764175	21	37.44	GAAAACATTCAATAAATGTCAGAGATTCCTCATTAGACAGTAGAGGGGAAATGC[A/G]TGTAAGGGAGGTGGGGATGGAATGCTCCTACCTCTCCGCCTCCTGATACCTGAGTC	A	G	0.57	0.47	0.44	0.43	0.48	0.64	0.84	0.66	0.57	0.42	0.53	0.012	0.097	0.023	0.020	0.0028			
rs2835638	21	37.45	TCACCAGTGTCGTGACTTTAACACAGTTTGAATGAATTCTTAATGCCTTCTAGCCAC[A/G]CTATATAAATGTAATCAAGATGGAATTACATCAGGTTGTCAGATGCACAGCTTTGC	A	G	0.43	0.53	0.56	0.57	0.52	0.36	0.17	0.34	0.43	0.58	0.47	0.012	0.090	0.023	0.020	0.0028			
rs2835649	21	37.47	ATCAAACAGTGTGTGCTTGGCCCCTGAAAAGCTGCATTTAATGAATTGTGTTTTC[A/G]TGTTCTCTATCAGCCTACATCAGAGGACATCAGGCTCACATTCTGTGAAGTTTGCTG	A	G	0.43	0.53	0.56	0.57	0.52	0.36	0.16	0.34	0.43	0.58	0.47	0.012	0.097	0.023	0.020	0.0032			
rs2040125	21	37.47	CTAGTTCAACTAACTGTTAGGAATGTTTCTCACCACTTTACAGCAGTGTAGGC[A/G]TATCAGTTAATCACTGTCATAATAATGCTGAGTGACTCCCAGCCACAAAACCCCAGTGA	A	G	0.21	0.49	0.52	0.52	0.50	0.05	0.06	0.31	0.46	0.51	0.44	0.065	0.155	0.032	0.172	0.0027			
rs2835653	21	37.48	GCTTTTCTCACTATTGTTTCTTACGCACATTCCCATCCCGGTTTTTGAATAATTTT[A/G]TACAGATTTCTTTTTCCCTTTGGGTTCTGTCCTTGTAATATGCGTCAAAACAGAGTTT	A	G	0.57	0.47	0.44	0.43	0.48	0.64	0.84	0.66	0.46	0.42	0.53	0.012	0.097	0.023	0.020	0.0032			
rs2835657	21	37.49	TAAAAGACAGAATTATTCACTGTTATTAACAAGATTCGGCAAATACCTCAAACAA[A/G]TAGTAAAATATGATGTGCGGACCAACAAAGCCCTGTATGCCTCAGGCATAGCAGC	A	G	0.43	0.52	0.56	0.57	0.52	0.36	0.16	0.33	0.43	0.56	0.46	0.010	0.092	0.024	0.018	0.0025			
rs2835667	21	37.50	GCCCAGAGACAGGCCGAGTCTGTCATTGCATTAGCCTCTTAGTGGTTCCTTGGG[A/G]GCCACTCCCAGCAGAACCTCTGTCCCTGCACACAGGTCTGTGCTACGAAACA	A	G	0.57	0.47	0.44	0.43	0.48	0.64	0.84	0.66	0.54	0.42	0.53	0.012	0.097	0.023	0.020	0.0032			
rs1016694	21	38.16	GATCACCCAAGTTGACAGATGCTGTGTCTAGGCTTTTTCGTGATATATTAGCTT[A/G]AACTCAGATCATAATTTCCCCTGCAGTTTGAGCTACTTAACAAAGCCTTCGCCTTTTAC	A	G	0.50	0.49	0.52	0.45	0.50	0.64	0.84	0.66	0.46	0.49	0.57	0.000	0.037	0.003	0.003	0.0004	1		
rs2836301	21	38.60	CAGTTAAGTTTGAAGATTTGTCTAATTTCCAAGGCAATTTTGTAATATATGGGGAAA[A/C]AAAGGTTGCATGTAAGACATTTAAGGTATCCTAAAAAAGTCTACACAAATAAAAAA	A	C	0.57	0.44	0.41	0.33	0.45	0.64	0.49	0.61	0.61	0.52	0.44	0.020	0.013	0.033	0.038	0.0012			
rs2836803	21	39.27	AAGTCATTCCTGACCACGCAGTCAGTGAGTGAGCAGGTTTACAAGTTCTTGAAAT[A/G]AATGTAAGCAGCACTATGGTATAATTATCTTCCAAACTGCATTTCACAACCAGAA	A	G	0.45	0.44	0.47	0.44	0.59	0.55	0.43	0.69	0.46	0.47	0.52	0.000	0.042	0.025	0.003	0.0012	1		
rs2836956	21	39.54	AAATTTATATGAATATAAATAAAATCTCAGAGGCAGTTAAACCTATTTTTCATATCA[C/G]AGGCAAAAATATTCATTCGTTTTCTGATTGGTACTATCAGGACCAATTCAAACAGAA	C	G	0.57	0.48	0.43	0.49	0.46	0.50	0.58	0.49	0.46	0.41	0.47	0.012	0.023	0.004	0.005	0.0009			
rs13625	21	39.59	AGTTATGAAGAAACTAATTTCTGGCACAGTGTGACTGCTTCAAACCTATGTTCTG[A/C]TGCTATCTCCTATAGAGGAGGGCAGACTTTTTTTCTTAGTGCATTTGCTGGTAGGT	A	C	0.36	0.30	0.40	0.38	0.29	0.05	0.04	0.19	0.29	0.43	0.42	0.021	0.128	0.032	0.107	0.0006			
rs2837121	21	39.99	TTAGTGGTCACTCTGAAACCACACAGCCATACTTCTTTGATAAGAATCTAGAATTA[A/G]TCAATGCCTTTATCCTCTTCTTGGATAATACAAAGAGTTTAAACATGTAATTATAAT	A	G	0.50	0.52	0.52	0.45	0.59	0.80	0.61	0.65	0.54	0.51	0.52	0.000	0.226	0.001	0.028	0.0004	1		
rs2837536	21	40.55	GAAAAAAGTTTAAGGATGTGATACTTTGATCCTCAATCTTGAATCATAGAAACAAA[A/G]TCTAGGGCAATAGTAAAAAGTCCCTATCAAAACTCACTTGCATGTACCTGGCCACC	A	G	0.48	0.46	0.47	0.35	0.52	0.27	0.34	0.56	0.39	0.40	0.49	0.003	0.001	0.015	0.006	0.0025	1		
rs2210267	21	40.68	TGTAAAATTTAGCCTCCCTCCAGATTTCTATATGTTGTGTTTATGCACTCAG[A/C]GATTTTTAAGCGTAAAATTGGCTTCTTATTTAAAGCCAAAGTGAGTGAATAGAATACTCAT	A	C	0.90	0.64	0.61	0.63	0.66	0.82	0.92	0.77	0.75	0.55	0.59	0.043	0.068	0.014	0.018	0.0003			
rs2837622	21	40.72	CGAGTGTTCCATTTCTTTTTTCTGTAACCTTAGACACTAACAAGGCATAAAGTCC[A/G]GAGGGCATAGAAGGTATCGCCTGGGTAATTTATGTACCCTCTTCTGACTTTGCCAC	A	G	0.78	0.45	0.54	0.43	0.57	0.55	0.70	0.34	0.42	0.48	0.51	0.029	0.010	0.020	0.000	0.0001			
rs2837710	21	40.86	CATGGGAATATGTGGGCCCTGCTGCCAAGGGAAGAGGCCTCCTGCTACAGCCCTC[A/G]TGGTAACAGAAAGGAGGACCCCCTTCTTCCAGTTGGCTCATTGTCTAGCCTGCC	A	G	0.29	0.48	0.43	0.52	0.48	0.27	0.71	0.48	0.64	0.48	0.47	0.020	0.018	0.004	0.022	0.0002			
rs2210273	21	41.26	TGGTCAACCCTCAGCAACACTTTGTTGAATAAATGAGCCCAAGATATCTCATTGTAC[A/T]CCTTTAGGCTCATCTACAGGCAGGTTCTATATTCTGAGATGAAAGAGGATATTGTA	A	T	0.83	0.42	0.40	0.33	0.32	0.27	0.83	0.45	0.46	0.40	0.47	0.113	0.160	0.006	0.002	0.0054	1		1
rs2837888	21	41.26	AGCATCCCCATAAATCAACAGGAAGACAAAATAATGCTAAATGTTTTCACTCTATTT[A/T]TCTTAATCTGGAGCTAATATTCAATGCAATCAGAAGGGAGAGAGAAACAAAAGGA	A	T	0.83	0.41	0.40	0.33	0.32	0.27	0.61	0.44	0.43	0.40	0.47	0.113	0.042	0.005	0.002	0.0054			
rs2837956	21	41.40	TTCACAGACAAGATGCTATCTGCACTAAAGATTCATAGATTTTCACAGATGATGA[A/G]TGTTGATAGTCCCGAGCATGCAGTATTTCAGCCCCGCTTGACGGCATCGGAACC	A	G	0.79	0.42	0.40	0.44	0.41	0.09	0.03	0.30	0.46	0.36	0.37	0.065	0.157	0.016	0.082	0.0012			
rs914186	21	41.54	CGCTCGCACTCCCGAGCTCATCGTGGCATGCGCTGAGCCGAAACCACGAGGTAGA[A/G]GGAATGAGATCACAACATTTGTTTGCGTTGTCTAAAATTATCCTCTGATTTCAT	A	G	0.62	0.60	0.65	0.61	0.52	0.91	1.00	0.86	0.61	0.72	0.69	0.001	0.158	0.037	0.064	0.0012			
rs458582	21	41.72	GGTTGGACTGATTCCAGGGGCCATGATTGGTTCCCACCAAAAATATGTCTTGATT[A/C]TGGGCAAAAAATTCACCACGTGGCTTCCCTACAAAGATAATATCTTAATGATGCCCC	A	C	0.90	0.43	0.49	0.41	0.39	1.00	0.95	0.82	0.50	0.63	0.61	0.128	0.036	0.010	0.015	0.0043			
rs2248865	21	41.96	TGGGGGCTGTTGGATTTAGAGTCTGAATTAATTCTGACATCTAGAGATTCAAAGC[A/G]CCTTCACGGCTCCTTGCTAGGGTGAGGGTGTATGAGGTGAAGTAATACATGAAGTT	A	G	0.26	0.14	0.25	0.26	0.18	0.50	0.46	0.27	0.11	0.21	0.29	0.001	0.026	0.000	0.046	0.0001	1		
rs2838112	21	42.04	CAAGGAGGGCTTTTTCCCCTCCCACTTTTGTTTATTTACATCAATATGCAGTCTT[C/G]GATCCCTGTTTAATTGATGGGATATTCTGATTATTTTGATGGTCAGGTTAGCCCTGATT	C	G	0.21	0.41	0.40	0.43	0.39	0.68	0.52	0.39	0.50	0.51	0.44	0.027	0.001	0.013	0.022	0.0012			
rs876498	21	42.74	ATGGCAAACTATGAAAATTGTGACGTTTTATTTGAGTAGTCATTAAAAGCT[A/G]CTTAAAAATTGTCAGAGGCTATATGGATAAGCCTCTGTGTGGATAAGACTAGCATTTTA	A	G	0.57	0.40	0.37	0.29	0.38	0.36	0.20	0.30	0.35	0.41	0.38	0.031	0.009	0.000	0.001	0.0004	1		
rs2839524	21	42.77	CCCTGCCTCCAGGCCCACACCTACGTCCAGGGGCAGTGTCCGCAAAGTTCCAGTTCT[C/G]CTCGGACTTCCTCCGTAGCAGCATCACACAAGTGTTCAGCTCGAGATGTGCACC	C	G	0.10	0.40	0.38	0.40	0.34	0.18	0.00	0.27	0.43	0.33	0.37	0.073	0.170	0.023	0.032	0.0008			
rs875060	21	42.84	CTGCCTTTCTTTGTCCTGTGTAGGACGCC[A/G]CCTTAGGTAATTATTGATTCAGGTTGTTGGTGGCGCCATTTGAAGTCTCTTCCCAGGCCACAGCAGGACAGGTTCTCCAGG	A	G	0.50	0.42	0.45	0.56	0.39	0.27	0.95	0.43	0.54	0.46	0.46	0.000	0.137	0.003	0.025	0.0001			
rs2839576	21	43.03	TTTCTCTTGCTACAAATGTTGCTGCTGTGTACAACCTGTTAAAGTCCACAGATGGA[A/G]TAAAAGCTTTGGTTTGCAAAATGACTCAATCTTTACAAACATTCTGTCATTTGTACC	A	G	0.05	0.34	0.44	0.37	0.55	0.05	0.63	0.24	0.32	0.30	0.41	0.135	0.010	0.025	0.129	0.0011			
rs234705	21	43.38	GGAGCTGGGGCTGGGTGCTCCCACCGGCCAGTGTCTTGGAGAACCTTCCTGC[A/G]CTGAGTGGGGGGTCGGAGTTCCAACTGGGGGCCTCTGAGGATCAGCCAGGAGCT	A	G	0.29	0.28	0.34	0.33	0.41	0.05	0.09	0.23	0.31	0.31	0.40	0.003	0.074	0.014	0.096	0.0014	1		
rs2838229	21	43.55	CAATCGTCTGACCCACTGCCATAGCTTCCTGGACATTACTGAAGGGCTCAGCTC[A/G]GGGTGGAGGGATGAGCACCTCTGCTGTGCCTACCTCGAGTGTGGAACTGGCAGGA	A	G	0.10	0.41	0.46	0.45	0.39	0.00	0.19	0.24	0.19	0.46	0.51	0.092	0.057	0.041	0.209	0.0039			
rs2838325	21	43.87	CAATTCTAGAATGCTCTAATACTTACATCCTGGATTATATGCATTCATGGACTGTA[A/T]CTTAGCTCTGTTGAAAAGTTTCAAAAATCCCATAGAGTAGACATTTTAATGACTGCT	A	T	0.26	0.56	0.52	0.65	0.54	0.77	0.79	0.61	0.68	0.59	0.55	0.026	0.050	0.003	0.044	0.0003			
rs1785452	21	44.69	GGTCCGTGTGCTCCTGCACCTGCTGGAAGGTGTAGCTGTCAGGGAGGAGAAAGC[A/G]ACACCTCGAGGTCAGGGTGTTTACCCAGAACAGACCCCTCTCACCGCCTGCGTG	A	G	0.52	0.23	0.26	0.23	0.25	0.14	0.14	0.39	0.18	0.19	0.21	0.017	0.031	0.001	0.032	0.0034	1		
rs2838665	21	45.01	GAACGCGCTTGTGTCTTACTCGTGCAGTTTGCTAAACTAACAACCAAAAGGCCATC[A/G]CGCCCTTACAGTTTTATTCCCCGCTGACAGATTTGAGGGGAGCTGTCAGGTGGGA	A	G	0.12	0.27	0.26	0.30	0.30	0.45	0.84	0.16	0.29	0.24	0.18	0.025	0.213	0.008	0.015	0.0104		1	1
rs235310	21	45.12	CAGACATCAGAGAGGAAGTATTTTACTCACCAACCATTCCTTTTAAAGCAGAA[A/G]TCTCAGTGTAATCAGAAAACTTCCAACTTTATAATTTTTTTTTTGAGACAGTGTTTTGC	A	G	0.38	0.58	0.62	0.53	0.64	0.32	0.51	0.65	0.46	0.51	0.51	0.010	0.000	0.009	0.017	0.0033			
rs2255761	21	45.54	TCCCAGCAGTCTAATGAACAGAGGAGGGCTGGCTGCTGTTGATGACGAGGACTT[A/G]CAGGGCCCCCTTGAGCACACCCAGGCCACACTGGGGGCCCAGGGGCTCTGGCAGA	A	G	0.62	0.66	0.62	0.56	0.77	0.41	0.80	0.33	0.64	0.56	0.63	0.000	0.028	0.046	0.018	0.0000	1		
rs10098	21	45.57	TCTTAGGACGCCTGAGTCTCAGATGTGACTTATAGGGTATTTCTTATGGCAAGAC[A/G]GTTAAAACAAACTTCAGCGTCTCGTCTGTCCTTCTATGGCTGTGGCTTCTGATGTTC	A	G	0.33	0.56	0.58	0.49	0.70	0.55	0.73	0.44	0.62	0.51	0.51	0.045	0.006	0.023	0.002	0.0036			
rs2838970	21	45.90	TCTGAAGGGCCATCTCATCCATGAAAAAAAAGACTAGGTAAGTTTCACCCTCTGCTCTAA[A/G]ATATCAGCAAAGACATTTTCCCTCTGCCTAGAGATATTAATGAAAAAAAAAAAG	A	G	0.45	0.47	0.50	0.46	0.32	0.09	0.36	0.22	0.38	0.33	0.36	0.001	0.010	0.053	0.103	0.0067		1	1
rs2968	21	46.46	GGGAGGAACAGACTCCTCTGCATTGTGATCCAATTGTGAACAAAAGTCCTCTTC[A/G]TGGAACAGGAAAAATACACTCCCTCTAAACAATGGATTGAACACAGATGTGATTTCT	A	G	0.62	0.56	0.59	0.52	0.73	0.73	0.28	0.65	0.54	0.62	0.60	0.001	0.039	0.004	0.013	0.0000	1		
rs2256207	21	46.92	TGTATGTACTTAATCTTCTTAGGGTAAGTCTCTGAATGTGAACCTGATGGACTG[A/G]GACTATGTGGCTTTTGGTTTTACCACCCAGTGCTCTCCAGAAGGTGTTCCCTGGAC	A	G	0.76	0.72	0.73	0.77	0.75	0.91	0.92	0.93	0.75	0.76	0.79	0.002	0.049	0.073	0.047	0.0020			
rs2839372	21	46.92	CACCCAGTTAATGAAAGTAAGGCTACCAAATATATCAAGCTATATCTGCTTGGAC[A/G]TAGCTTTGTTTACTTTGTGACAGGAATTTCTTAGAGTCAGCCTCCAGTAACTCAGT	A	G	0.17	0.28	0.27	0.23	0.25	0.09	0.08	0.07	0.25	0.24	0.21	0.013	0.049	0.073	0.047	0.0022			
rs2256070	21	46.92	TAGAACCCTGATTTACTACCTTCAATGGCTATACGATATTCCATAGCAGGAATTCC[A/G]CTTGATTCTTCAGCCATCCCCTTTGATAGACTTTGTCTTGGTATCCAGTTTTCTAC	A	G	0.69	0.72	0.74	0.77	0.75	0.91	0.92	0.93	0.75	0.76	0.79	0.000	0.049	0.073	0.047	0.0020			
rs2839377	21	46.93	TAAACCTTAGAAAAGCTCAAACAAACATAAAAATCACATATTAACCCAGTACCTAGAAG[A/G]AACCACTGTTATCACTCTGCAGTTTACCTTCAAGTATTTTTCTACACACGCACAC	A	G	0.36	0.55	0.52	0.56	0.61	0.59	0.59	0.74	0.61	0.63	0.53	0.012	0.010	0.056	0.008	0.0000			
rs7288876	22	15.46	CCGTGTTTAGGAATGTTCAACGCTCCAAGCCCCATAGGCTGATTCAAGAAGATAAA[C/G]TGAGACTCAAGGAATTTCGAAGTGGAACAATACACCAAAGCCTTAAACCTGAAAT	C	G	0.60	0.42	0.37	0.28	0.23	0.64	0.51	0.60	0.36	0.41	0.37	0.023	0.010	0.029	0.035	0.0001	1		
rs2845348	22	15.59	TAGGAGCTCAGACTTTTTTTTGTTTTTTGAAAAGGTGAGTACCTTGGACATTTTCA[A/G]AAACAGAGTCTAAAATTCCTTGTGCTTTGCAATTATGGTGGACAAAGCTAAGAGAT	A	G	0.95	0.53	0.52	0.48	0.46	0.50	0.72	0.55	0.50	0.62	0.56	0.130	0.019	0.000	0.000	0.0015			
rs175138	22	15.68	TTCCTTGAATTGCAAAATTAAAACCTCATAAGCTACAGTTTCCACATCTGTACAAT[A/G]ATACATCATTAGAATCTTCCTGGGGAGATAGCAGTGTAGAGAAAATAATACATCG	A	G	0.50	0.60	0.58	0.59	0.63	0.77	0.54	0.57	0.50	0.67	0.58	0.006	0.001	0.004	0.017	0.0000			
rs175139	22	15.68	GTCCTGTCAATTCTGTGTTTCTAAGAAATGATGATGGTGGTCATGATTATAATGAT[A/G]TCAATAGGAACACCTTTCCAAACTTCACTGTGTACCACGAACTCTTCTTCCTGCTGC	A	G	0.50	0.60	0.58	0.58	0.63	0.77	0.54	0.57	0.50	0.67	0.58	0.005	0.001	0.003	0.020	0.0001			
rs1860945	22	15.77	AGCAGAGTCTTTGAGTCTGCAGAGGAGAAGGCTGTTGGGGATGTCAGGGCA[A/G]GGTTTGCTGCTGAGGACGCTGGTCCATGACAGCCTAGCACCTCAGATTTCATATC	A	G	0.76	0.69	0.67	0.68	0.69	0.82	0.71	0.86	0.65	0.77	0.69	0.006	0.000	0.034	0.014	0.0004			
rs5748760	22	15.81	TAGAACTAGTGCTTGGTAATATTGCTGCAGGTGCAGCTGTTTAATGTGATAAAG[A/G]GTAATGAGGGGAAAGCTTGCTCGTGACCACACCACAGACCCCCAGCACCCTCCCATT	A	G	0.12	0.31	0.29	0.27	0.18	0.09	0.01	0.25	0.14	0.22	0.23	0.033	0.129	0.007	0.044	0.0065		1	1
rs2399153	22	15.87	GGGTGAGTTCTGATCATGCTGCCTGATCCCTTTCCTCATCCCATACTCTGCTTCT[A/G]ACCTGGATGGGGGCCCACAGGGCCAGCGTGCAGCTGAGCCAGGACTGTGCCAGC	A	G	0.02	0.17	0.16	0.16	0.13	0.27	0.09	0.19	0.07	0.18	0.20	0.035	0.007	0.000	0.006	0.0003			
rs5994180	22	16.01	TATGCTGTTGCACCCAGACCTGTCACTGTCCCAAGCACTCAGCCAGACATGTAACA[A/G]AGGCATTTTTATGTATTCTTTTTGGCAGCCCTTTGAGGAAGTGTCAGCATCTCCA	A	G	0.88	0.95	0.95	0.98	0.91	0.95	1.00	0.89	0.96	0.98	0.94	0.004	0.022	0.007	0.001	0.0000	1		
rs1034859	22	16.01	CGCACCCCAGCAGGGCTGACAGCTCCTGGGCTTCTGTGTTGTAAGATGTT[A/C]CCAGCATTTGTAACAAAAACCACGGGCACCCGCAGCTGCCCCTGGGAGTTCACCAGC	A	C	0.45	0.63	0.50	0.60	0.44	0.91	1.00	0.58	0.68	0.59	0.44	0.002	0.182	0.000	0.082	0.0027			
rs7290186	22	16.07	TTGTAGGGCCTGTGTGTCCTGGTTGAGGATCAAACCGCATATGCTATTGGAGAAAC[A/G]ATCTCTGTGGACATACATACTGAAAGAATAACAAAGGCAGAAGAGAAAAACAA	A	G	0.64	0.83	0.81	0.77	0.89	1.00	1.00	0.97	0.71	0.77	0.78	0.008	0.093	0.067	0.093	0.0002			
rs2040692	22	16.21	CCTCATTGGCTTCCCAATCTAATTGACTCCGTTGAC[A/G]TGAAAGGGTGACTCCCAGCTGCTGGCACCCCTGTCAATCCAACCAGGATGTGAAACATCC	A	G	0.38	0.51	0.51	0.45	0.38	0.68	0.36	0.54	0.54	0.44	0.46	0.011	0.029	0.000	0.006	0.0006			
rs2097596	22	16.33	AAAACATTTATCTTACAATTATTTCATCTTCCTCACAATGATAGTTTGTGGAAAAAC[A/T]GCGAAGTGTCCTTTTCAAAATTCGGAGGTTTCTAAACGATAGAAATTCAAATGCTTT	A	T	0.02	0.11	0.08	0.10	0.11	0.23	0.00	0.05	0.14	0.08	0.13	0.007	0.027	0.002	0.023	0.0022			
rs174345	22	16.41	ACTGAATACTTATGGGAGCCGGAGATGAGGGTAGGAAAGGTTTGATCTTGTAAT[A/G]TGTCACTGTGTTTCCTTAGTGGCCAGCCAGCCTTCAGAATAGCTAAAGGCCTTCC	A	G	0.67	0.57	0.44	0.50	0.55	0.68	0.73	0.38	0.50	0.41	0.48	0.029	0.039	0.010	0.022	0.0010			
rs2535694	22	16.53	TACAGTGATCTCCAGAATACATCATGACATTATTAAGTGAAATCAAACACAAGCTC[A/G]GAAATTAATTCACGGCCCATGCTATTTCTTGCAATAAAATTCTCTCTTTCCCTTCCA	A	G	0.05	0.13	0.13	0.10	0.18	0.18	0.09	0.23	0.18	0.22	0.22	0.006	0.000	0.006	0.006	0.0093	1	1	1
rs3747026	22	16.59	GTTAAACATCAAAAGCTTTCTCCTTGAAAAGATGCTTGGTGTATCTGAAAGTCA[C/G]TGGGACATTTGGTGCATTCAGAAGGTCAAATCATTCCTGACCTCTCCTCTTAGGACC	C	G	0.58	0.54	0.56	0.56	0.61	0.23	0.23	0.42	0.57	0.50	0.55	0.001	0.041	0.017	0.045	0.0007			
rs4269007	22	16.67	AGTGGCTCCTGCAGCCTGATCAGTTCCTCTTTAAATAACTCAGCAAACCAGACG[A/C]TTTATTTCGCATCCCCTCCTTGGTGCATTCGTCTCCCTTGGCCTTTCCGGCTCTTT	A	C	0.12	0.40	0.35	0.33	0.27	0.05	0.01	0.26	0.36	0.40	0.40	0.036	0.110	0.003	0.076	0.0010			
rs387399	22	16.84	AACCAAGGGTCTTAATTTCACCTGCTGCTGTGCTACTAGTAACTCCTAAGTCCACAC[A/G]GGACTATGGGCACACTCATCTCTACCTATTATCCTCATGACAGAACCCTGTGCGCC	A	G	0.43	0.48	0.46	0.35	0.52	0.41	0.22	0.43	0.54	0.40	0.40	0.000	0.039	0.000	0.002	0.0015			
rs2252257	22	17.01	TCTTTGGCATCAGAACGGATTACATGAATACACATTCCTCCTTCTCTAATTCTT[A/G]ACGATCCCCATTGTATTTTCACAGAGATTCCATCGTGCCTGGCTCACATAAGCGCTTC	A	G	0.29	0.21	0.19	0.12	0.23	0.86	0.65	0.38	0.14	0.15	0.13	0.008	0.128	0.017	0.261	0.0026			
rs737923	22	17.51	AGCCAGCCCGCGCTCCGTTCGACGTCACTATAAATGCTAGACTTTATTGGA[A/G]ATGTAGTCCTTGTCCTGCCCCGCGCTATTCATGCTTGTCAATGCTGTGAACACGAGGG	A	G	0.55	0.66	0.65	0.72	0.63	0.14	0.63	0.48	0.54	0.57	0.63	0.005	0.000	0.015	0.141	0.0006	1		
rs885978	22	17.57	TTGTGAAGTATTTGGGATCTAAAAAGAAACAGTATAGCAAAGTCTTGGGAAAGCAC[A/G]GGTAAGAGAATCAGAATGTGCCAATCAAACCTGGGAGGGCTGCTGTGGCTTTT	A	G	0.07	0.49	0.43	0.48	0.39	0.27	0.43	0.21	0.32	0.38	0.49	0.128	0.162	0.058	0.044	0.0004			
rs712948	22	17.58	CAACCTTGAAAGCTACTTCACCTCTGTCCACGAGGGCTGAGCTGGGCCCAGCATGCAGGC[A/G]TGACACCAGGTACTCTCTTCCTGGGCTGCATATCCCTGCTTT	A	G	0.02	0.40	0.35	0.35	0.30	0.09	0.01	0.15	0.25	0.21	0.41	0.152	0.189	0.065	0.108	0.0002			
rs756658	22	17.83	ACTCACTAGCTCCAGGCTTCCCAGGGGCAGAGCATTTTCCTGGAATAGTCACTC[A/G]CTGTACTTCCAGCCTTCCCACAAGCAAATTATAAGGCCCTGCGTCAAGTAGGCTC	A	G	0.88	0.49	0.54	0.54	0.52	0.68	0.66	0.51	0.54	0.51	0.49	0.074	0.005	0.001	0.009	0.0002			
rs1476445	22	18.00	TGTCGTAACCTACTTGGGCTACTGGACAATGAGGAGATTCATGGCCTGTGCA[A/G]CACCGTCACCAATTGCCTGGTGCAGCCCAAAGACCACCAATGTAAGGGCAGACCTGT	A	G	0.76	0.53	0.47	0.50	0.61	0.27	0.34	0.54	0.71	0.54	0.54	0.049	0.008	0.005	0.020	0.0025			
rs374225	22	18.40	TGACTTTCAAGTCCCAGCTTGGACATTAGAAGAAAACAAATAAGGACACAGC[A/G]TGTGACTGAGTGGAAGGGTCAGTTCCTGCAATGTCCATTGAACCTGAGGGTCTAAGAA	A	G	0.64	0.58	0.59	0.61	0.61	0.41	0.14	0.49	0.50	0.61	0.59	0.001	0.110	0.003	0.014	0.0000	1		
rs165630	22	19.14	GTAGGATGCGCCTGGCTCCATCAGTCACTTAGGCTGTGATCTCACAAATCCATTT[C/G]TGGGCAGAGCAAGGGACAGTGGTTCAGGCCCACAAGACTCAGCTACCATTAGATT	C	G	0.64	0.37	0.44	0.37	0.32	0.55	0.05	0.29	0.39	0.33	0.34	0.015	0.136	0.020	0.004	0.0058	1		1
rs743409	22	20.45	TCACCCAATTAGATACTGTCTGTGCTAAGAAAAGTCCAGAAGGCCTGGACCACAAT[A/G]AGATTCTAACAGTATTACTTCTAAACCACTGGGGGCAGAGGGTCCTCTACTCAAACT	A	G	0.33	0.48	0.45	0.44	0.38	0.50	0.21	0.47	0.61	0.41	0.35	0.008	0.022	0.005	0.002	0.0075	1	1	1
rs2012607	22	21.61	TGCACAAGTCTGGCAAGATCATTATCTCCTTTCACAGAGAGGGATGTGACCT[A/G]GCCAAGGTCCCTCATCAGGAAAGTGGTACAGCCTCCCCTCCACCCACCACCAATGGT	A	G	0.21	0.32	0.24	0.26	0.36	0.14	0.00	0.33	0.18	0.30	0.35	0.008	0.126	0.000	0.025	0.0059	1		1
rs756631	22	21.79	CCCCAATTACAGCATCATTTCTATGAGAAATGACAGCTCTGTTACCACAGTCCTCT[A/G]TGATGTGGCTTCCAGGAATCCATTAGAGCTAATTACTCCCTCAGCAGAATGTGACA	A	G	0.64	0.40	0.41	0.43	0.41	0.77	0.45	0.70	0.75	0.45	0.44	0.015	0.000	0.021	0.052	0.0000			
rs1003774	22	23.13	TAAAGCACCATGTATTAAAATGCAACTCATTCTCCATTGGTTGTTGAAGCATGT[A/C]AAAGATCCTCCAAGTCAGGGTGTATCTCAAAGTGCGCCCACACATGAAGATACCTTCT	A	C	0.60	0.54	0.49	0.55	0.41	0.59	0.58	0.40	0.46	0.40	0.52	0.001	0.001	0.016	0.002	0.0003	1		
rs743377	22	23.42	CAAGTTTTCTCCTCTTTCCATTAATTTCTCTTCCAAGGCAGACGACTTCGATC[A/C]TAAACTACCAAGATACCTGGAGCGATCAGATGAGCCTCGGCCTACTCTTGAAGCTAAAC	A	C	0.79	0.53	0.60	0.55	0.48	0.41	0.27	0.52	0.61	0.59	0.58	0.025	0.040	0.000	0.010	0.0000			
rs742163	22	23.80	CTTAACAGGGAGAGGGCAGAGTAAAATGAGGCATGAGGACAGCTTGGTAAATTCA[A/C]TAGAATGTGGAAAAGGTATCCCTAGGATTAAGGTCAAATTAAAGACATAAACTAA	A	C	0.57	0.56	0.57	0.56	0.68	0.75	0.79	0.72	0.61	0.60	0.55	0.005	0.067	0.024	0.048	0.0001	1		
rs763281	22	23.92	TGACAAGGGGTTGAGTCCTCCTCCTGCCACCCTCTGGCTGTGGGATTTCTTTTAA[A/G]CATTGGTTCTCCTTAGCTATACAATGGGAGTGGTGATCAGGACATTTTTGCAGGTA	A	G	0.60	0.71	0.65	0.65	0.75	0.73	0.42	0.70	0.71	0.63	0.67	0.007	0.041	0.000	0.000	0.0010			
rs713816	22	24.49	GTACCCCTCTCCAGTAGAGCAATGAGTTTAGTGGGGTTCTTCACTATAACTCCCTCC[A/G]GGGCATCAAGCCAAAATGGTATTGGTAGTCAGGAACAAGGAAATCTCTTGCAGGA	A	G	0.74	0.39	0.43	0.50	0.38	0.05	0.43	0.49	0.50	0.48	0.48	0.050	0.000	0.002	0.112	0.0005	1		
rs761670	22	24.74	CCCACCCTGTAGTCTGTCCAAAAGGAATATCCTTCTGGTATACCTGCTATGG[A/G]AAAGAGCCAGAGGATAAGCATCCAGCCACAAAGATGCCGGGGCTGGTTCTATAACTT	A	G	0.23	0.43	0.51	0.54	0.41	0.23	0.36	0.45	0.29	0.51	0.48	0.042	0.006	0.001	0.041	0.0003			
rs738402	22	25.01	ATAGTCACATTTATCTGATTCTTCCTTTATCACGTACAGTCGGTGCTCTACTCAAA[A/T]GCGGTCAATCAATCAAAGTGTTGGGTGTTCACTTTCTCTCCTATGGTTGACACCCGACA	A	T	0.69	0.46	0.46	0.48	0.43	0.91	0.89	0.78	0.46	0.46	0.41	0.027	0.118	0.056	0.143	0.0007	1		
rs738401	22	25.01	TAACTCCTTGAGGGCAGGGGCCAAATCTTAGAATGTTCACTCTCTGTGTCG[A/G]GTGTCAACCATAGGAGAAAGTGAACACCCAACACTTTGATTGATTGACCGCTTTGAGT	A	G	0.33	0.56	0.53	0.52	0.57	0.09	0.11	0.22	0.54	0.54	0.59	0.022	0.113	0.052	0.138	0.0009			
rs713751	22	25.51	TGAGATAAAGCACTATGAGGGAAAGGGTGTTTTCAGAGTATATGAGAAGTGC[A/G]TGTTCTATACTGGGGGTGGGGATTCAGGAAACTTTTGGAGGAGAGATACTAAACT	A	G	0.31	0.49	0.50	0.45	0.39	0.27	0.22	0.50	0.50	0.34	0.39	0.028	0.041	0.005	0.031	0.0100	1	1	1
rs84312	22	26.04	AGTCATAAATAATAACGCTTTGATATAATGAATTTACAAAGAACAAAAATCTGGTT[A/G]CCATGGTTGGAATGCCATTCAGAAGTCACTAACCAAATATCTTCGTTTTGCATTCA	A	G	0.43	0.66	0.76	0.67	0.63	0.45	0.64	0.66	0.82	0.65	0.70	0.069	0.010	0.005	0.053	0.0039	1		
rs136926	22	26.11	ACTAAATGTGGAGACAAGTGAATAGACTATGTGCCAAAGGGAGATAAATATTACC[A/G]TGCATATGCATGGCCCGAGTGCCATAGGAGGGTGGGTGCCCATGGTGGGTCTAG	A	G	0.81	0.64	0.68	0.68	0.55	0.91	0.61	0.84	0.68	0.66	0.62	0.014	0.000	0.033	0.059	0.0021			
rs5762174	22	26.19	GTTTGGCTCATGGGTATTATAGGAAGAATTGGATAGGTTGCTGGACTTAGGTAA[A/C]TGTTCTTTTGGAGCCCCTTCACCCCCATAGCCAGGATGCTAATCCTTCCACTTCC	A	C	0.15	0.65	0.60	0.67	0.64	0.23	0.34	0.56	0.82	0.49	0.51	0.134	0.039	0.001	0.090	0.0082		1	1
rs138650	22	26.72	GGCCACACCGCAAAGCAGAGTGTCTGAAAGTGTCTCAAACTTTGGGAAACAGAGGAACACCAAAAATCCTATTTTCTTA[A/G]CATAAATCCAGTTGACACATCTCGATATTGCT	A	G	0.79	0.54	0.45	0.49	0.57	0.55	0.60	0.67	0.57	0.51	0.58	0.072	0.029	0.047	0.017	0.0100	1	1	1
rs738995	22	26.97	AGAGAAGGGAGGATCCCATCTTGAATGGTTTTGACTACCTACCATGAGTCATTGAA[C/G]GGTTTAAGTAAGGTAGGCACATGGTCAGATTTGCATTTTTAATACAAAAAAAATCC	C	G	0.05	0.36	0.39	0.32	0.34	0.14	0.01	0.43	0.25	0.42	0.32	0.079	0.127	0.001	0.039	0.0041			
rs737622	22	27.20	GCTGTGAGTGACATGATCTTTCTTCTCTAGGGAATGAAGGAGGAGAACAATACCA[A/C]GAAATTAATCCAAATCCATCTATCTCAACATTTGAATTTATTCATTTACCTCATCAAGCA	A	C	0.52	0.60	0.54	0.53	0.52	0.36	0.06	0.60	0.54	0.57	0.45	0.000	0.155	0.002	0.013	0.0034			
rs1997719	22	27.95	AGGAGGGGCCGGGCCTGTGGCCTTGGGAGTTCAGGACTCACTTCCTGCCGCC[A/G]GGTCTTTTGCGTCCCAGTGTTCCGAGACTGGATTCCAGCCAGTTCCAAGGCTTGG	A	G	0.10	0.37	0.40	0.43	0.52	0.25	0.34	0.19	0.25	0.49	0.57	0.057	0.002	0.018	0.012	0.0224	1	1	1
rs140062	22	28.00	TTCAAGAAATCAAACATTACTGTCTCGGCTTAAGCAATTGACATGACTTCCTAC[A/G]TTGTATAGAGGCTTAATCTAAATATTATAGGCAAATAAAAATTCTTAACTGGGTACCCT	A	G	0.93	0.57	0.65	0.60	0.57	0.73	0.28	0.55	0.43	0.55	0.54	0.046	0.083	0.013	0.002	0.0098			
rs715494	22	28.11	AGATGATCTTCACACAATCTCTGAGGATCTGACCTACTGAAG[A/C]TGCTCCTCATGACAGTTTCTAAGAGGTCAGGAGGAGACTGGTGGGCTCAACTCAGGAC	A	C	0.19	0.45	0.41	0.49	0.45	0.27	0.65	0.48	0.54	0.53	0.50	0.022	0.026	0.005	0.011	0.0048			
rs714027	22	28.90	GGACATCCCAGGGATGCGGATTTGGCCTCAACCTATTAAAGAATTTTCCCCCA[A/G]TGCTCTAGCAAATTGCCTGGCACATGGAAGGCAATCAACATTTGACTGTTTTGATAG	A	G	0.79	0.51	0.43	0.49	0.41	0.36	0.82	0.53	0.71	0.48	0.51	0.042	0.062	0.000	0.011	0.0009	1		
rs737805	22	29.02	CCATGACAGGGGAGGGCAGAGGCTCAAAGAGAAAACAGGGAGCAGGGCCAGGGC[A/G]GGATGTAAAGCTATGTCAGGGCTGGAAGTAGGGGCCCCTCACTCAGCAGCTAT	A	G	0.38	0.43	0.47	0.51	0.46	0.82	0.79	0.78	0.50	0.47	0.46	0.008	0.029	0.018	0.043	0.0002			
rs4444	22	29.53	CTTGGTGGAAGGCCCTTCTCAGAGTCTTCATCATACACTTCTCGATTCTCGC[A/G]GTGCTCACCACACTGACTCATAAGGCTCCATTGGAGAGTCTGTCTCCTGAAAAATAT	A	G	0.29	0.57	0.54	0.50	0.54	0.68	0.96	0.64	0.75	0.55	0.52	0.054	0.098	0.000	0.002	0.0001	1		
rs762883	22	31.46	CATGATCCAAAAACCAAAAGGATAACATCAGGCACAGCAGGGGCTTTTTTACT[C/G]TGCTCCAAAGCTGTAATTGACTAGTTAGTTCCTGAACCATGTCATTTGTTAAATATTTT	C	G	0.67	0.40	0.45	0.38	0.34	0.91	0.86	0.50	0.54	0.52	0.38	0.022	0.110	0.003	0.125	0.0030	1		
rs9862	22	31.58	TGGTACTTGTTGACCTCCAGCTTAAGGCCACAGAGACTCTCGGAAGCTTCCGT[A/G]TGGATGTACTGCACATGGGGCATCTTGGTGAAGCCTCGGTACATCTGTGGGCAAAAG	A	G	0.24	0.49	0.51	0.48	0.55	0.41	0.04	0.33	0.54	0.53	0.42	0.046	0.188	0.023	0.012	0.0029			
rs137410	22	31.91	TTGCATGGCTGTGCTAATTCTTATCAGGATGTTGTCCTGTGAGGCACACCAA[A/C]GGCTACTGTAGAAATGAGAAGAAGGTTAATATTCCACTCTTTCCAGCTTTGTGGACC	A	C	0.62	0.66	0.64	0.68	0.59	0.32	0.55	0.72	0.57	0.63	0.58	0.001	0.001	0.004	0.052	0.0016			
rs762058	22	32.28	CCCATCTCTGCATGTCCAAAACCGAACTCGCCGTCCAGTGCTATTCACTCTAC[A/G]TCCCATTCATCTCTAATCTGCATCCCTTGCTCTGACTCCAAGGCTAACATCCCGCTAC	A	G	0.33	0.48	0.49	0.46	0.52	0.27	0.57	0.38	0.46	0.48	0.46	0.002	0.021	0.000	0.011	0.0003	1		
rs1844461	22	32.96	GTAGTAGTTGCGGTCCATAGCAATTCTGAAATCTCGCCAGACAAATATTGCTCTGTCTTGTACTCTGGAGATAGGA[A/G]ATTTTTCTTGATCAAGACCCAGTTTAATTCTTTGAGA	A	G	0.79	0.74	0.75	0.65	0.75	1.00	0.94	0.93	0.79	0.84	0.82	0.001	0.028	0.026	0.085	0.0039	1		
rs1380148	22	33.17	CAGGTGGGTTGTAACAACAATAGTAATCACTTTTGTTGCATAGGCTGTTACTATCCCCTGCCTCAAGCCAAAAAGTGTCA[A/G]TTTCCCTCAATGATGACTTATATGTGCTCATC	A	G	0.40	0.65	0.65	0.63	0.71	0.45	0.35	0.72	0.64	0.66	0.62	0.033	0.058	0.003	0.019	0.0007			
rs2056965	22	33.55	AGAAAGTCCTTGCCGTTGGTAATCTGATGGCAGCCACTCTTGGAACCAGTTCA[A/T]GTGTTTCTAATGCTGAGGTGTCCTGGCGTCCCCTGGTTCCTGTCCTTTCTGCCGGCTC	A	T	0.43	0.34	0.47	0.46	0.45	0.41	0.33	0.23	0.43	0.44	0.56	0.000	0.003	0.031	0.000	0.0060	1		
rs2008519	22	33.74	GATGAAGGAGATAGAGGTCCCTGACTCCAGACACCACAGTTAATGGGGGAGAT[A/G]GACTCATATGAGCAATTAGACTGCTGGCTCCATGAGGCCATGGCTCCATGGCTGCTC	A	G	0.68	0.47	0.47	0.53	0.52	0.73	0.29	0.68	0.54	0.49	0.39	0.016	0.024	0.020	0.031	0.0074			
rs138777	22	34.04	TTGGGCAGGAAGGACACTGTCATTTTCAGGTTTCAGAAAGTTCCTTCTGG[A/G]CAGTGTGGAGAATGGGTGGGAGGAGGGAGGCAGGAGGAGGGAAGAAGCTGAGGCGGT	A	G	0.75	0.42	0.38	0.27	0.30	0.41	0.56	0.34	0.46	0.26	0.24	0.077	0.009	0.005	0.000	0.0105		1	1[g]
rs738510	22	34.36	CAGTAACGTGTGGACAGGCCTTTTGCACTACACTGTCATCAAAATTCGAGGT[A/G]CTCAATCATTTGCATTTTTGCCGTCTGAGAGGTGAAACATTTGATGTCATTGCCTGCACT	A	G	0.52	0.65	0.60	0.57	0.68	0.82	0.60	0.75	0.61	0.61	0.53	0.004	0.000	0.017	0.026	0.0019			
rs972153	22	34.37	CAGGGCAAGATTTACTTTTGTCTGACATAGAGCTGGGTCTTGGTACTAATGAG[A/C]TTTTTCAGACTTTGACTTGGGAAAGGCAGTGGCTATAAGCAGGATTGGACTTTTGGGT	A	C	0.86	0.71	0.67	0.59	0.57	0.82	0.22	0.74	0.61	0.70	0.67	0.012	0.152	0.002	0.002	0.0006			
rs1540297	22	34.38	AGTGAGAAGGCATGGCTGGGCCAAAACAAATCTAGGCACTTCTGGAATTCTC[A/G]ACCACACAACCAGATAACAGTGCCTGGACTGACCACAAACTGGACCCATTAAGAGT	A	G	0.48	0.59	0.57	0.60	0.73	0.86	0.81	0.75	0.75	0.56	0.51	0.001	0.051	0.035	0.075	0.0010			
rs715550	22	34.75	ATCTGCCCCTCCCCAAAACAGAGAGGCCTCATACATCTGTCAGTAATAAAGCC[A/C]ACCCTCTCGTATTTACTGGGTAGATAAGTAACTGAGACTGAAACGCCACTGATTCCA	A	C	0.36	0.76	0.78	0.76	0.82	0.77	0.93	0.81	0.75	0.69	0.80	0.100	0.021	0.000	0.001	0.0001	1		
rs878847	22	34.77	TTAGACAGATATTGAAATCTCTGGATCTATCATCCACGTCACTTAACTTTTCTTTC[A/G]TATTTTCCGCATCTTGCTAACTCTTTCTCCATCTGCGTTCATTATGTTTTTCAGTCCAT	A	G	0.90	0.64	0.63	0.69	0.77	0.50	0.53	0.59	0.64	0.70	0.63	0.062	0.005	0.000	0.006	0.0000			
rs735853	22	34.95	CAGTATTGTATATGAATTTTCTGCCATGTGGTAGAAAGAGGGTGCCTGTCTTT[C/G]TACTCTGTGTGGAACAGGCACACCTGTTTACCTGTCTTGGTTTCACCGGTTTTATAGGAG	C	G	0.12	0.47	0.51	0.50	0.32	0.09	0.53	0.26	0.64	0.45	0.42	0.078	0.000	0.042	0.114	0.0054			
rs739096	22	35.01	CTAATTCTGAATTTCAGTGGTTAATACTCCTTAGGGACAGGGGCCTTTAATTT[C/G]GTCCACATGATAATCTTATCAAGCAACAGATGAGACACAATAGGTCTGAAAATCAGGAA	C	G	0.03	0.60	0.62	0.59	0.52	0.15	0.68	0.41	0.50	0.53	0.52	0.238	0.001	0.042	0.132	0.0060			1
rs933924	22	35.04	AAAATTTCAGTCTATTTTTATATCACGTTACAAAGGTTAAAGTCACTTTCA[A/G]GTTATTGCAACAAAAGTCTCTCAATCCCATGAAGGTGGCAGTATTAGTGTTCCTATTTTGT	A	G	0.40	0.28	0.35	0.27	0.34	0.91	0.27	0.64	0.46	0.30	0.28	0.001	0.004	0.048	0.178	0.0016			
rs2413411	22	35.26	TGTGATGCTTAGAGAAGTGCCTGATCATACTAAGAACTCTGTAAATGTTACTTATT[A/G]TTGTTGAGTTATTTTAATATTGAATTATTATTAAAATTCTCCCTTGTTAGAATTCTAT	A	G	0.90	0.30	0.38	0.45	0.32	0.41	0.26	0.39	0.25	0.39	0.37	0.191	0.001	0.000	0.004	0.0001	1		
rs760519	22	35.51	TTGGAGCATTAAATGAAGACTTGTCAATTCCTAGTGTGGCTCTTTTACCTTCTCC[A/G]TTGCTCCTGCTCCTATCTCTGGAACCCTAAGGACGCATCCTGTTTTACTCCCCAGAC	A	G	0.90	0.84	0.83	0.82	0.80	0.77	0.94	0.77	0.79	0.78	0.78	0.002	0.027	0.011	0.002	0.0032			
rs1534880	22	35.57	GAGAGGTAGCCACTGGGACGTGGTGATCACTAGGCTGTGTGGTCAGCAGGTCACT[C/G]TCCTGTCTCTTGGTGAAGTAACTGAGGTTTGGAAAAGTGGCGTGGCTTGGCCAA	C	G	0.10	0.43	0.46	0.40	0.63	0.86	0.91	0.41	0.54	0.36	0.44	0.101	0.100	0.006	0.071	0.0000			
[illegible]	[illegible]	[illegible]	[illegible]	[illegible]	[illegible]	[illegible]	[illegible]	[illegible]	[illegible]	[illegible]	[illegible]	[illegible]	[illegible]	[illegible]	[illegible]	[illegible]	[illegible]	[illegible]	[illegible]	[illegible]	[illegible]	[illegible]	[illegible]	[illegible]

rs136904	22	43.33	ACATGACCTTTGTGTGTATTTTCCCCATCGTGTGCTCTGCTTTTCTTTTGC[A/G]TTTTAGCTTCTCAGACATGACCTTTGGTCTCAGATGCTCAGCTTGGTGGCAGCACTTCACA	A	G	0.36	0.37	0.35	0.45	0.18	0.73	0.83	0.32	0.54	0.34	0.35	0.001	0.135	0.002	0.084	0.0001			
rs1540327	22	43.38	TCAGAGGGCTTGGTACCCCCACGTGCCCTCATGGCATTTTGGGGCCTGCTCTGG[A/G]TCTGGGCATCTTTTGGGAGTTTCTCTAGAGCCTATGGGGCTGGGGGAGGCAGAA	A	G	0.63	0.30	0.41	0.38	0.36	0.68	0.80	0.60	0.36	0.44	0.37	0.008	0.043	0.008	0.013	0.0054			1
rs132817	22	43.81	CAGACAAGACAAATTAATCTATATAATACTGAAAGAAACACACCCAAAGCCGAAGT[A/G]TCTAGAAACACTGAAACTGAAAGAAGCAGGGCGTAGGGGGAGAAATAGGTTTACC	A	G	0.79	0.58	0.63	0.57	0.52	0.32	0.33	0.70	0.46	0.66	0.63	0.015	0.054	0.000	0.061	0.0000	1		
rs714022	22	44.45	TTTTTATGTGGTGTTTATGTAGTATCCTTTTTCCTCTAATGCTCTTCCTTTGGTAC[A/C]TAACCTTGTAGGAAAAGTTTAGATCTGAAAGCACAGATGCTGTGAAAGGACCAATTC	A	C	0.74	0.54	0.63	0.60	0.50	0.68	0.57	0.58	0.39	0.56	0.59	0.003	0.006	0.007	0.000	0.0008	1		
rs737822	22	44.52	CTGGTCTTAAAATGATTTTTTTACTGTCCTTTAGTTTCTGTCCTTGAATGAATAA[A/G]CAAAGATGTTAGAACAAAAATTCCAGTCTTTGCAGGAAGAAAACTTTTTAAAAAATCTT	A	G	0.71	0.52	0.60	0.61	0.52	0.64	0.56	0.57	0.39	0.56	0.55	0.003	0.004	0.006	0.001	0.0004			
rs738672	22	45.78	CCTCGCTCCTTGCAAGGTGAGAAGCAGTACCCTCCCATTACGGTAACTCACCGACTCTACTGCACACTCAATTTCAGAATCTAATTTAGTCC[C/G]CCAGGTTCACTGGATGGAG	C	G	0.53	0.60	0.58	0.55	0.68	0.68	0.74	0.44	0.46	0.68	0.55	0.001	0.007	0.035	0.002	0.0001	1		
rs738486	22	46.15	CAGACAAGGCAGGGGATAGAGGCCTCCACTGCTGTGCAGAGGAAGATAGAGAAGC[A/G]GAGGGGAGCATGCACAACATTAAATTTAAAAATAGATTTTCATTTCTACAAACCA	A	G	0.35	0.53	0.53	0.46	0.64	0.18	0.73	0.57	0.39	0.46	0.45	0.019	0.027	0.000	0.067	0.0035			
rs728591	22	46.48	CAGAATGTATTATTGTCAAGCTAAGAGAGGGAAATTAATAGCCTTAAATGCATATA[A/T]AGAAAATAAATAAAATCAGTCATCGAAGTTTGGATCTCAAGAATCTTGCAAAAGAAC	A	T	0.23	0.21	0.21	0.18	0.23	0.55	0.16	0.38	0.18	0.20	0.15	0.000	0.007	0.010	0.048	0.0059	1		1
rs738704	22	46.65	AAACCCTGTGAGTATTTCATTCAGCTGTAAACTACAGACAGGAAAATATCTCAAGC[A/G]AAATAACCATGGCTTGCATTGTTTCAGCACTACATAAATTTCAAAGAGCTTTCAAATCTG	A	G	0.71	0.32	0.34	0.35	0.27	0.36	0.31	0.60	0.21	0.38	0.40	0.061	0.007	0.031	0.000	0.0012			
rs4403	22	46.93	ACACCTCTGGAGGCCCTCTTGCTGGGCCATGTGTTCTAAGGGCGTTAGGGAT[A/C]ATATGACTTTGACCCATGGTATTCCAGGTCCCAACTCTGGCAGACAGGAGGTGCTCA	A	C	0.21	0.54	0.44	0.49	0.41	0.18	0.21	0.35	0.39	0.41	0.52	0.034	0.032	0.015	0.043	0.0023			
rs761793	22	47.18	GAGAAGCCTGTTGGGCTACACAGGGTAGAGGTGATTCACACTGACCGGTCACTAC[A/G]CACACTTCAACCTTTATTCCTATACAGACAGGACCTAGCAGGCAGAGATCACTAC	A	G	0.88	0.71	0.75	0.80	0.77	0.41	0.63	0.88	0.81	0.78	0.72	0.012	0.021	0.009	0.086	0.0028	1		
rs131020	22	47.40	AACATGTGGAGGCTAAGACCAGACCGATGTCATATGGAACACAACATGTCTGTCC[A/G]CACAGACACAGACGCACACCCTGTATCACTCAGTGCTTAACTGGCAGCCAGAGA	A	G	0.69	0.58	0.50	0.67	0.48	0.09	0.43	0.26	0.65	0.43	0.52	0.019	0.005	0.071	0.125	0.0000			
rs7410750	22	47.45	AGCTGAGCCCTGTCACCXACGGGAAATCACAAAATGACTGTGGGTGTCTGAAACCAC[A/C]AAGTGCTCAGGTGTTTTGTCACATGACAGTCCATGGCCAGATCATCCAAAAA	A	C	0.60	0.37	0.46	0.33	0.48	0.23	0.17	0.39	0.57	0.39	0.34	0.007	0.065	0.005	0.044	0.0049			1
rs1474834	22	47.54	AAAACAAGAAAACACAGCTTCTTCTCGCACCCTGTGAATTACCTACAGAAAATGACC[A/G]TGGGCTTAGACACAAAACACAGCTCAAGGTGTTCTCAGGAGGTCACCTGAGACT	A	G	0.93	0.72	0.75	0.74	0.84	0.77	0.77	0.82	0.79	0.85	0.78	0.018	0.002	0.000	0.002	0.0010			
rs932497	22	47.62	ACCTCACAGCCCATGTCCTAGTTAAGAAAAAGGCCAAAGGTCAATCATAGCCC[A/G]GGTCCTTGAGTTGTGAAAATTCAGTGAACAGATATGAGAAGAGCAGAACACTTGCAC	A	G	0.74	0.63	0.61	0.58	0.52	0.77	0.51	0.86	0.86	0.63	0.64	0.014	0.000	0.067	0.025	0.0002			
rs135223	22	47.73	CTTAAGACACCTGAATTTAAAACGTGCATCGAGGTAAGGTCGTTTACAGAAACAC[A/G]TTAGCAAGGGGCAGTCCCGCGGCAGGCAGTGAGAGGAGCGAGAGGAGGCCGGT	A	G	0.31	0.46	0.42	0.43	0.52	0.73	0.49	0.41	0.36	0.40	0.44	0.011	0.000	0.001	0.031	0.0000			
rs8136406	22	47.92	GATAGAAGAAATGTTAAACAAACTCAGCAACATCTAGAAGGTTGAGGGCAGGAGG[A/G]AGGAAGAAGTAAAAAAGTGTCTGAAAGGTCTCATTAGTGGGAGGAGGAGAGTCG	A	G	0.55	0.96	0.93	0.90	0.91	1.00	0.99	0.93	0.93	0.92	0.93	0.060	0.028	0.001	0.045	0.0007			
rs7286586	22	48.02	TTTGTGAAGCATCCTCACACCTCTCAACAGGTTGGCGGGAAGTAGAGGCATAGGTTG[A/C]ATTTTCACCACCATTTAAAATACTAGAACTACTTTGTCACTTCCTTTGTGAATTC	A	C	0.50	0.84	0.86	0.85	0.91	0.41	0.55	0.56	0.86	0.79	0.87	0.095	0.088	0.113	0.165	0.0001			
rs6520124	22	48.14	TTTCCATTTCGCTTGTACACCTCAATTCATCTTGTAGACCGAGTTTAAAGACAGAC[A/G]TCACTGGAGGCCTAAACGCTGTGGTCATTCACATAAATAAATCGTTTGTTTTAGCT	A	G	0.88	0.95	0.97	0.99	1.00	0.86	1.00	0.95	0.93	0.94	0.97	0.015	0.009	0.005	0.023	0.0004	1		
rs739240	22	48.27	CAGCGCTAGCAGAATTCCAGCTGCCAGAGAGCTCGGGCATATGGGCAGGCGCTTAT[A/C]AAGGAAGAAAGCAGAGTGTTGAGGCTGTTTTTTTAAGCCACAGGTAAGAAGAAA	A	C	0.79	0.70	0.69	0.65	0.70	0.59	0.99	0.63	0.57	0.63	0.73	0.007	0.100	0.002	0.005	0.0010			
rs4824090	22	48.34	CATTAACCCTCAAGTCCACCCCAGTCTGCAGCGTGGAAACTCAGCACTGGGCTTTT[A/G]TGAAGATTTAATGAGTTGGTTCCTGATTAGAATCTGGCCCCAACCACAGGCCTGA	A	G	0.24	0.20	0.18	0.15	0.20	0.23	0.01	0.30	0.11	0.23	0.20	0.002	0.047	0.008	0.002	0.0007			
rs138853	22	48.41	TGCTCTGGCCGGGCTGTGGGGATGTTCTCTCCTCTTACAAACGTATTCTGCACACA[A/G]CAGGACGTGAGGCCTAACAGGCAGCAGTGAAGCAGAGGGGGGCCCAGCTGGCAGG	A	G	1.00	0.83	0.87	0.91	0.86	1.00	0.95	0.90	0.93	0.89	0.92	0.047	0.009	0.001	0.045	0.0030			
rs2092982	22	48.50	TGGTCCATTATCTAGAGCCCATACTTGGGCAGCATTTGAAATTTCACCTTAACC[A/C]CAGACAGGGCTCCAGGGAAGTGGAGATGTAATTCTTACAACAACAGTTCTGATCATG	A	C	0.24	0.72	0.75	0.85	0.74	1.00	0.94	0.54	0.93	0.77	0.81	0.116	0.043	0.023	0.106	0.0027			
rs137879	22	48.64	CATCACTCTCCAAGGGGAGCTGTCAGTTCTATCTGCCAGCTTTGAGTGTCTCAG[A/G]TTTGATTTTTACTTATTCAACCTGGAATTTGAATGTCTTGATGTGTGCCGCTGGTGTCAT	A	G	0.45	0.45	0.49	0.48	0.57	0.41	0.65	0.66	0.64	0.46	0.53	0.000	0.026	0.027	0.000	0.0021			
rs137930	22	48.71	AGTATGAACAATATTTCTTTTTGTTTAAAAAATATTTCTTGGAAACAACTTATTGA[A/G]GTTTCCTCAGAGTGGCCAAAGCCGATGATAATTTTATTCCAGATAAAGAACTGGTACA	A	G	0.86	0.62	0.62	0.58	0.70	1.00	0.71	0.66	0.64	0.68	0.70	0.025	0.000	0.000	0.129	0.0029			
rs6712	22	48.82	TGGCAGGAAGGACACAGCCGGCTCCTGACGCACACCTGATTTGATTCAACAC[C/G]GAGTCGGCACAGACACAACCAGAGATGGCCTGGGTCCAGGCTGGGGCTGCACGGG	C	G	0.95	0.82	0.90	0.85	0.91	0.95	0.96	0.75	0.96	0.88	0.91	0.011	0.012	0.020	0.013	0.0004			
rs6520165	22	48.99	CACACAATGACTGAGTTCTGGGAGTGTCTGGGAGTGGCTCAAAAGATGATAAAGG[A/G]TGACCACCATCCCTCTGTCCTAATGGTGCGTATGTAAGCTCTGTCTTCAGGACTT	A	G	0.67	0.71	0.71	0.71	0.70	0.32	0.41	0.65	0.54	0.65	0.66	0.001	0.054	0.000	0.079	0.0001			
rs743616	22	49.20	GCATCCACGGGGAGGGGTTACCCTGGGTGAAGAAGTGAGCCTTGTACTTTCCA[C/G]TCCGCACAGCAAAACCCCACGGACCTCGTCTGGGTAGGACGGTAGAAGAAGA	C	G	0.42	0.51	0.55	0.54	0.54	0.35	0.21	0.41	0.65	0.46	0.58	0.005	0.050	0.003	0.014	0.0013	1		
rs1001469	22	49.27	TGTGAGCGTGGGAGCTCCCTGGGCTGGCCTGAAGGAGTCAGAGAGGGCCTCAAA[A/G]CAGAGGCATTGATGGCTGAGCTGAGTCCTCTAGGTGGTCAGCGGCTGGGGCTGAGGG	A	G	0.29	0.17	0.23	0.24	0.20	0.32	0.05	0.20	0.14	0.20	0.29	0.000	0.039	0.003	0.002	0.0041			1
rs10451	22	49.29	TGGCCATTCTACCTTCTTTAGTTCAGGATTCAAAAGACAGGTAGGAGCTTGGGAA[A/G]CTCATGAGGCCTCTCCTAAGGTCCCGGGATGCTGCCTCCAGCTCCTGTCATCCTG	A	G	0.29	0.34	0.33	0.28	0.31	0.15	0.43	0.38	0.39	0.24	0.27	0.003	0.003	0.006	0.030	0.0019			
rs749706	X	3.63	AATGTCCTGCCCACTTATTACCTCAGCGCTCGCCTTCTATCAAAAAGAATGCC[A/G]TTCAATGAGCTCTCTGAGGACCCCCTGAAAACAGGCTCAGAGACGTCTCTGCTCTC	A	G	0.86	0.58	0.56	0.62	0.50	0.86	0.92	0.66	0.43	0.55	0.60	0.052	0.078	0.004	0.046	0.0003	1		
rs1882409	X	5.60	TCAATGCAGGCAATTTAGTAGACACTGAAGATCATATTTTACCTTTCCAAATTG[A/G]AGTGGATAACAACAGTGCCATTTCATGGCCTTAGAACAAGAATGACCTTTTCTGTC	A	G	0.81	0.58	0.56	0.66	0.50	0.36	0.64	0.46	0.75	0.62	0.52	0.045	0.000	0.000	0.025	0.0009	1		
rs1158755	X	5.70	CTTGTGTTGCATCGGATTTCTTAATTAAAGACGAGATATTTTAATACAAAAATG[A/G]TACGTTAAAGTGACGACATCGTTTAGCCTTAATACGTGATCAAATGTGGAGCATGTTT	A	G	0.05	0.24	0.27	0.21	0.21	0.00	0.02	0.16	0.46	0.27	0.21	0.029	0.056	0.002	0.090	0.0008			
rs1852456	X	6.42	AGCTCATCCCCACTCCCCTGTCACTCTGCATAAGATTCCAGGGTGACATTGT[C/G]AGACGTCGTCATTAATCTCCTTTTAAAAATGCAAATCCCCTCAGACATGACTCTGCTC	C	G	0.86	0.65	0.58	0.56	0.70	0.59	0.66	0.73	0.57	0.52	0.59	0.055	0.003	0.006	0.000	0.0002	1		
rs1869588	X	7.49	GTTGTTTGAATGAACTCTAATAGTCTAAAAAGAGGAAACCATGCTTCTGTGGCTG[A/G]AGGAATCAATCCACATGTATTATTTCTTCTAGAAAATATCAACCACTATTGTAGGCAC	A	G	0.33	0.48	0.43	0.35	0.43	0.64	0.57	0.41	0.39	0.38	0.45	0.005	0.004	0.003	0.015	0.0012	1		
rs1921708	X	7.71	ATTCATACCCATGACTAAGTACACACTGTGACATGATGGGATTTGGAAAGCC[A/G]CAGTTATTAGAAGAAGGCTGTTCGGCATGAGCTTTGTCCAAAGCAAATGGGCCAGGT	A	G	0.93	0.42	0.44	0.27	0.39	0.64	0.73	0.54	0.43	0.49	0.50	0.184	0.059	0.016	0.031	0.0029			
rs1159561	X	8.02	ATAAAGTGTACAAATCCCACAAATTTAAATAGGCAGCTTAATGTAATTTAATGCC[A/G]CTACATGTAACAGTTTAACTGGCCAAGAACACACAGAAGGAAGAGTAATTATTTTCC	A	G	0.50	0.54	0.51	0.60	0.50	0.73	0.43	0.42	0.54	0.45	0.40	0.002	0.004	0.012	0.017	0.0146		1	1
rs1656651	X	10.33	AAGAGACATGTTCATTATTAACAAATTTCTACGGACAAATCTCACAGCTGGATG[A/G]CAATATACCCAGAAGTAATACCATTAATTTTCTTAGGGAAAGCAATGTCTTTGGAGTTCA	A	G	0.02	0.42	0.38	0.45	0.27	0.00	0.00	0.08	0.50	0.39	0.50	0.128	0.164	0.084	0.164	0.0000	1		
rs2057652	X	10.53	GGTATTGAGGCCAACAGCTACCGCCACTCAATAGGACTCTTGCTGTTACATTGT[A/G]CTGGTAAAGTTAGATTTCTTGTCTGAAAATTTCCTGAAACCCTTTCTGATATGAAAA	A	G	0.69	0.82	0.79	0.79	0.71	1.00	0.99	0.85	0.86	0.78	0.68	0.011	0.037	0.000	0.055	0.0060			
rs720062	X	10.54	GCATAAATAAAACCATTCCAGAAACAGGCAA[A/C]ATATGGTCACTGACAATTTTACTGTAGCACAAAACAGAGCCCTAAAAGCTGCAAGACAGCAGAATGATTTGCTGGTAAAA	A	C	0.14	0.18	0.23	0.28	0.34	0.00	0.01	0.15	0.18	0.24	0.30	0.004	0.050	0.001	0.070	0.0029			
rs1933730	X	10.57	AGAGTTCAATTCTGGCACTGTTCTTTTTGGTGAAAGCATATTGTATCATGTTTT[C/G]TTTTCCTCACTGTCTAGGTATGTAACTGTATGTGAGCTGTGAAGTAACACTATATACAA	C	G	0.52	0.82	0.78	0.78	0.66	1.00	0.99	0.84	0.86	0.80	0.75	0.040	0.050	0.000	0.070	0.0002			
rs1476468	X	10.66	ATCTCTTTCCAGATCTATTATATCT[A/T]CATAAAAATATGCATGAGAATCAGATAAACTAGTGGGCCTCAACCAGGAATGATTGTGCCCTTCAGGGTACATTTTATAATATTTGGA	A	T	0.43	0.72	0.73	0.73	0.75	1.00	0.98	0.79	0.71	0.67	0.63	0.075	0.034	0.003	0.065	0.0091			
rs2238907	X	10.66	TGTCTTTTATGGTCTCTTGATTTATTCAGGAATATTGCATTGCAGTTGAGTCTCTC[A/G]CAGAGTCCACTCTTGAGGCTGCTAACATGTTGATTTAATGAATGGGTGCATTTCATGAA	A	G	0.40	0.73	0.74	0.76	0.75	1.00	0.98	0.83	0.73	0.68	0.70	0.084	0.035	0.000	0.065	0.0099		1	1
rs946252	X	10.67	ATATGAAGACATCTGATCTCATACTGTTTTAAACCCTAATTTCACCAACTATG[A/G]GCAGATTTTTATGTCCAAGTTTGTGAAATTGGACACTGACTTATGCAAGGGGTGTTTTAC	A	G	0.19	0.32	0.34	0.27	0.32	0.50	0.07	0.42	0.21	0.25	0.32	0.018	0.082	0.010	0.010	0.0000			
rs916685	X	10.70	ACAGCTTTACATGAAGGATGGCAAGTAGAATGGCTCATTTTCATTCATAATT[A/G]TTGCTTATACACTGTTGTTATGGAATTATAGAATTTTAGAGCAGGAAGGAATTTTAGG	A	G	0.67	0.76	0.74	0.78	0.82	1.00	0.98	0.80	0.79	0.71	0.66	0.013	0.031	0.004	0.060	0.0061			
rs937260	X	11.84	TATACAAATAATATGTTGGGGATTTTTTTAAGTAAAGGAGCATATTGGGCCTC[A/G]TAATTCCCTGGTTTTTACTGCTGAATGTAGACCCTCAAAATGAGGATTTACAAAATAAT	A	G	0.60	0.62	0.56	0.60	0.70	0.64	0.76	0.83	0.54	0.59	0.48	0.000	0.016	0.059	0.000	0.0049	1		1
rs2040618	X	13.23	ACAGCATCCACTATACCTGAGCTGAGAGCCATCACATTCTGGTCTTCTCGACTT[A/T]TCTCTTAATACTTCCTTTCTTGCTGTCTGGGCTCTAGCCACACTGCTTTAAATTTCTTT	A	T	0.64	0.57	0.56	0.46	0.52	0.95	0.72	0.80	0.54	0.44	0.46	0.010	0.038	0.031	0.141	0.0037	1		
rs1859004	X	13.27	CATCTCACATAATCTATGGAATGTTATAAACACCAACGGAAAACGTCATGGTTT[A/G]GGAACATTTCTTATGCAGCCAGGGCTTATTATAAAGCCCACCTTTCTTGCAGAAGCCA	A	G	0.57	0.37	0.34	0.28	0.36	0.77	0.79	0.38	0.25	0.27	0.27	0.041	0.131	0.011	0.137	0.0010			
rs929210	X	13.69	ATGTATGTTTTATATCTCTATAATGTTATAAGGTCCTGGGATGGAAATTATCAC[A/G]TATTAATCACACCTTTCTCCTTGATACTTGTTTCGCTTAGTTTCTAGAACACCACCTT	A	G	0.62	0.47	0.48	0.49	0.45	0.14	0.16	0.55	0.32	0.30	0.49	0.005	0.064	0.003	0.075	0.0001			
rs1373328	X	14.39	GGTCTCAGGAATTCCCTGCATCTTGATGAGCCC[A/G]AGTGTGGGCCAGTTAGTCCCTGCAGAGCAATCATTTTAGACTTTAGAACCAAAATCCCAAGACAAATGGCCCCTCAC	A	G	0.52	0.51	0.53	0.56	0.50	0.32	0.57	0.47	0.75	0.47	0.52	0.002	0.001	0.009	0.037	0.0005	1		
rs1444011	X	14.44	[illegible]	[illegible]	[illegible]	[illegible]	[illegible]	[illegible]	[illegible]	[illegible]	[illegible]	[illegible]	[illegible]	[illegible]	[illegible]	[illegible]	[illegible]	[illegible]	[illegible]	[illegible]	[illegible]	[illegible]			
rs401335	X	41.84	AAGAGACCCCAATATCACCCTGGCTCAGGTGATATAGCAGGGTAAGTGGCAA[A/G]CCCTTATCTAGGCATCTATTCCAAGACAGGCTACTGTGAGGTGATTTCAAAATTGCT	A	G	0.24	0.54	0.50	0.50	0.43	0.50	0.77	0.50	0.43	0.45	0.53	0.055	0.022	0.000	0.001	0.0001			
rs1155699	X	42.28	ACATTCTCTCCTATATAATTCATAATCTCTTGAACATTGAAAAGAGCCAACTCTAA[A/G]CTAGTGATAGCCTCTAGAGCAATTAAAATTCTTGAATAACTAAATCCAGAAATTGTC	A	G	0.64	0.66	0.58	0.65	0.70	0.64	0.85	0.52	0.68	0.65	0.61	0.001	0.046	0.002	0.003	0.0006			
rs1339774	X	42.39	TTCAAAACTGATTTTTATTTTCAAATATCACCTTCTGTGGATATTTCATTATGAAAG[A/C]CATTGTTGCATGATAGCAGCTGTAATATATAATTTTGTATTATGTTTTTGGATTAT	A	C	0.36	0.28	0.35	0.32	0.30	0.36	0.17	0.48	0.21	0.32	0.36	0.001	0.015	0.014	0.000	0.0000			
rs929096	X	42.45	GACTCTTGCTACCCATGCAGAGATCATATTGCCCTTTAGTGAAAC[A/C]CAGTGACTGAACAATGGGGTGAAAAGTGAGGAGTGAGCACTCCGAAGCTTGAAGGCAGGC	A	C	0.29	0.30	0.28	0.27	0.23	0.36	0.00	0.61	0.36	0.29	0.20	0.000	0.101	0.054	0.006	0.0062			1
rs2064070	X	42.65	AAGGGCATTCCTTTCAAAGTTTGAAGGCAAAGCAGGTCTTCTGGCTACAACTCAGGCAAAT[A/T]GATGTGAACTGGTTGTCTGAACTTGGAAGCT	A	T	0.19	0.37	0.30	0.32	0.25	0.82	0.64	0.68	0.43	0.52	0.23	0.017	0.050	0.040	0.061	0.0046	1		
rs979605	X	42.65	ACTACGCCAATTAATCCAAGACCTGTGCCAAATTTAAAAGGAAAAGCAGAGTTCA[A/G]TGCAAATTCTAGAAATAGTTGTCAAAATCCCCATTTCTTATGTCCTAGATAATACTTC	A	G	0.24	0.34	0.29	0.30	0.25	0.59	0.45	0.68	0.39	0.32	0.24	0.010	0.008	0.043	0.036	0.0030			
rs766117	X	42.86	GGAACAAGAAATAGGAGCATCTATAAAAACTAGAAAACAATCTGGAAAGACTGGAC[A/G]AAATGATGGAGGAGTACTTTGGCAACAATCACATTAATCTAGCGAAGGTCAAAG	A	G	0.33	0.49	0.51	0.49	0.46	0.73	0.44	0.40	0.46	0.55	0.48	0.016	0.022	0.001	0.003	0.0009			
rs1335101	X	43.13	ACTGCAGCATTACTAGACAATCCTAACTGATACATCAAACATTTTTTCTGTTATGC[A/C]TATGTGGTCTACCTCTTTCTAATGGCTAAAAAATATTGTCTGAATTAATGCAAATAC	A	C	0.52	0.40	0.43	0.40	0.45	0.45	0.51	0.36	0.43	0.44	0.33	0.004	0.001	0.010	0.000	0.0041			
rs1451512	X	43.36	GATCTTCCCCACTGTTGGCCTGCTCATAAGTGTTGATGTTGTATAATGGTAGC[A/G]CCATGAGCAAGTTTATTTGCTTATTTGTTTTGGTGGAGGAAGGGAAATTTTTTGAGA	A	G	0.07	0.18	0.22	0.17	0.29	0.45	0.25	0.33	0.11	0.20	0.16	0.021	0.000	0.024	0.038	0.0057	1		1
rs548996	X	43.70	ATTACTTTCAGGTTTTTGCTTACAAACCACACCCCTCTGATGTCCTGCTTGC[A/G]TCATTCATTTAGTCAGGTGTATGGAATTGAGTTTCATGTATGTAGGAGTGGAAATGTGGACTTATA	A	G	0.48	0.65	0.62	0.73	0.79	0.25	0.67	0.44	0.71	0.70	0.72	0.024	0.000	0.079	0.118	0.0056			
rs1033383	X	44.27	AAGTTTATGAGAGCTTAAGGCTGAGCCCTTTTCACAGTTACTATTATAGAATCCC[A/G]TCTGGAGCTCTTCATACAGGTTGATCTTTTCTGAGTTCAAGGGGGTGCCATTTTTAT	A	G	0.21	0.39	0.40	0.38	0.48	0.00	0.00	0.13	0.25	0.45	0.51	0.019	0.176	0.048	0.176	0.0076	1	1	1
rs1004705	X	44.41	TTGCTATTTACCTTGATTCTGCCTTGTCACCTCCCACCACCACTGTATTAAGAGGGTTGGCTTCAAATGTAGCTAAAGCGAGGTTGAAACCCCCCTTGGAAGT	A	G	0.71	0.66	0.58	0.59	0.63	0.68	1.00	0.63	0.60	0.48	0.66	0.020	0.196	0.000	0.011	0.0014			
rs742585	X	45.12	GTGTGTTTATTTTTTCACTCTTCATTAACCAGTTTTCTAAGGCATAAAAAGTAGGTCTC[A/G]TGAAGTCAAATTTGTTGGCTAAACTTTTTAATTAGTTTCAGTTTCCCAAAAATTG	A	G	0.05	0.35	0.32	0.28	0.36	0.05	0.00	0.14	0.31	0.33	0.42	0.081	0.140	0.022	0.081	0.0030	1		
rs1156592	X	45.72	AAAAAATGCCCCAAGAAAGTCCACTTTCCTTTGCCAAAAATCAGGAAAAGCGTG[A/G]CTCAGCAGGACAGAACATTTTGATTATACCTGCCTTATTCCAGGTGAACACCA	A	G	0.31	0.19	0.15	0.11	0.16	0.41	0.60	0.16	0.11	0.23	0.16	0.016	0.114	0.005	0.043	0.0010	1		
rs1154782	X	45.90	CAGTTAAAATGTGCATGGTTTGGCTCCCAAAGACACAGTTCCCAAGCAAGAGCCCCA[A/T]CAAGAACTTTACCAAACTGTTCCCAAGGCCACAAGGTAATGTAGAACTTCACAA	A	T	0.07	0.53	0.49	0.56	0.55	0.41	0.35	0.25	0.46	0.48	0.40	0.105	0.010	0.020	0.002	0.0041			1
rs1998837	X	46.55	GTGAGATTCAATAAAGACCAGAAAGGGCCTTGTTTCATTTCAAATCAGGTTTGCC[A/G]CCAAAGAGTTAAAAGAAGCTTTCTGCCCTTAGAGCATTTTGGATGTTGGAATGG	A	G	0.50	0.62	0.64	0.70	0.66	0.95	0.52	0.70	0.75	0.78	0.67	0.013	0.014	0.002	0.067	0.0000	1		
rs1207480	X	46.73	TTCATGCTTCTTGATTCTAGTGACTGGAATCATGCTGATGACTTGATTTCATT[A/G]ATTTATTTGAGCTCTACTGAGAAACAACTGAACTATTGAATATTGCCATATTCATCTCCAT	A	G	0.26	0.55	0.46	0.51	0.50	0.27	0.28	0.58	0.43	0.48	0.48	0.035	0.027	0.003	0.031	0.0003			
rs688770	X	46.81	GAGTGTTGGCATAAATGTGGTAAAATAGGCACACTTGTGGATTGCACATATGATCCA[A/G]TGATCAACCAATACCAATTCTATATAAACATACCTGAGGAATAATCTCACAGGCT	A	G	0.86	0.47	0.58	0.55	0.50	0.91	0.74	0.46	0.69	0.60	0.60	0.069	0.022	0.005	0.092	0.0002			
rs4824685	X	47.19	CTGGGTTCAAGGGCTCCTCTCAGCCACTGCCTCAGGACTACTGGTATGTGCCAC[A/C]ACTGGCCCATGCTTTTTATTTGTTTTGTTTTGTTTTGTTTTGAGACAGAGTTTAGTAGAACCG	A	C	0.21	0.57	0.50	0.59	0.54	0.36	0.42	0.59	0.54	0.61	0.55	0.037	0.004	0.001	0.008	0.0020	1		
rs717193	X	47.81	TCACCAATCAAGGCCTCTGTTCTCACAGGTATTATTCTTTAAGTATCCTAGCATAC[A/G]TGAAAGTTAAAAAGGATGGAACAATTTTCCACAATTTTTCAGAGCAAGAAAGGTTG	A	G	0.17	0.35	0.36	0.35	0.45	0.52	0.14	0.64	0.19	0.52	0.35	0.009	0.064	0.018	0.013	0.0000	1		
rs2064034	X	47.87	GTGGGAGGTGGGTGGCATGGAGAAAGGCAGGAGGGTTTCAAGGCTCCTCCCT[A/G]TTTTGTCATATGTGGACAGTTTTCAAGGATTCCCCTATAGGGGAATGAAAAGGGGTC	A	G	0.12	0.40	0.41	0.48	0.55	0.14	0.61	0.20	0.54	0.58	0.48	0.023	0.055	0.013	0.013	0.0027			
rs1934176	X	49.11	CAAACTTGATATCAGAGCTCAGGTCTCTTAATAGTAGCAGATGTTGCAATGTCA[A/G]TGCATAAACAGAATAGCCTTGTTCCCTCCAATTGTCATTTGATATAAATCCATGCAT	A	G	0.45	0.63	0.69	0.76	0.64	0.86	0.52	0.85	0.69	0.59	0.63	0.037	0.039	0.006	0.005	0.0027	1		
rs2050843	X	49.48	ACTTCTACTTCCTCACCTGTCACTGCCTCTTCAACGCACAGCAGCCTGCGGTGTGCTGCAGCAGGCCACTGAAATGTCTCTCAAAGTC[A/C]CCCCCCTCCATCAATCCTTTTTT	A	C	0.71	0.29	0.35	0.32	0.45	0.09	0.29	0.42	0.46	0.44	0.33	0.088	0.001	0.009	0.047	0.0000			
rs1264007	X	52.40	GTAAGAGAAGGGAGGTAAAGAAATGAATAAATGAGTTATGTAATGAGGGAAACT[A/C]AGTATTACATTAACACCTCAAAAGAGGTTCAGCAGAGATGATATTGTCTCTTAG	A	C	0.07	0.53	0.59	0.56	0.68	0.59	0.43	0.45	0.54	0.54	0.55	0.168	0.008	0.004	0.000	0.0012	1		
rs1043034	X	53.80	AGGCTGTGACAATGCCTGCCACTGAGACCAAAAAGGTCAGCCATGTGGCTGATAC[A/G]AAGGTCAATACAAAGGCTCAGGAGACTGAGGCTGCACCCTCTCAGGCCCCAGC	A	G	0.48	0.55	0.68	0.60	0.68	0.50	0.64	0.61	0.54	0.66	0.65	0.015	0.000	0.000	0.018	0.0005	1		
rs763183	X	53.81	CCACGGCTTTATCACTCAGTTCTGAAATAGGCCAAATGCATGG[A/G]CACATGAACACCAGACAGAGATACTTACTTCTGCTTCACAAACTCATCAGTGATGAGCTTCTTCACG	A	G	0.36	0.45	0.32	0.40	0.32	0.50	0.36	0.39	0.46	0.34	0.35	0.000	0.000	0.000	0.018	0.0007			
rs2005463	X	53.94	CTTGAGAACAAGAACACCCCCAGCACCCAGCACGAAGGGCCACGAACACCAATAC[A/G]TGTGTGAATGCCCCTTAGAACCTGGATGCACGGAACCCTCTTTGGAATTCCCAGTACAA	A	G	0.98	0.54	0.65	0.61	0.66	0.45	0.64	0.68	0.54	0.63	0.64	0.115	0.000	0.007	0.022	0.0003			
rs1536163	X	54.86	GGGAAATAATTAGAAAGAATGTGGAAGTCCTGGGTAATTTGCCTGTCCTACT[A/G]TTAGTCCTGCCCTAGTGCCCTTATGAGTATTTTATGGCATGAAGGGTTAGGT	A	G	0.81	0.17	0.25	0.30	0.27	0.30	0.60	0.38	0.21	0.39	0.35	0.173	0.079	0.008	0.002	0.0056	1		1
rs204141	X	56.03	AGGAGGAGCTGAGGCATGTCTGTGGGGCTCCAGTACATATAGACACAAAG[A/G]CTGAGGTCCACTGGGTAGGACATAGCTCTCAATGTTCAGAATGTTGAGGTTCT	A	G	0.21	0.85	0.78	0.71	0.75	0.73	0.66	0.54	0.89	0.63	0.70	0.183	0.019	0.045	0.005	0.0036	1		
rs204165	X	56.06	GGTAGGCTGCAGAATTTCACATGGATAGAAGAAGGAGATGAGCCCATCACCCA[A/G]TGTAAAGGGACCGTTCTGAATCAGGTGCCTATATATCCCTTTCTTTATCTGGTAAA	A	G	0.24	0.85	0.78	0.71	0.75	0.73	0.66	0.54	0.89	0.63	0.70	0.168	0.019	0.045	0.005	0.0036			
rs512119	X	56.16	TTGTTTTTCTGAAAAGTTGGTGTCACTGGGCCAAAGCTTGCCCAAGTAGGTA[A/G]ATGCTCTACCACTATACAATACCTAGTATATATTTTTTTCTGCTAGAGAATAAATGGAT	A	G	0.26	0.85	0.78	0.71	0.75	0.68	0.65	0.55	0.89	0.60	0.70	0.154	0.022	0.041	0.011	0.0036			
rs957721	X	56.28	TAGGTGTTTAAGAAATTTAGTTGTTTTGCAGTAACAATATTTCCATGTTTAAAAA[A/T]GTCAGAAACTAAGGGATAAATAAAACTTTGTATCCACTCCACTTCTCCCCTTCAGAACA	A	T	0.79	0.15	0.23	0.29	0.25	0.27	0.36	0.47	0.07	0.40	0.31	0.183	0.025	0.050	0.005	0.0048			
rs1560514	X	56.35	CTTGATTATAATTTGTCTTGGTATGAGTCTGCTTGGGTTTATCTTAGAGTTTCATTTTTAGCTTTTCCCATTTT[A/G]TGTAACTTCTTCCCCCA	A	G	0.79	0.21	0.30	0.30	0.32	0.27	0.52	0.50	0.25	0.47	0.38	0.146	0.040	0.034	0.000	0.0057			
rs980735	X	56.39	GTCTTGGAGTGGCAGGTGGTCACTGTGGCATTGTTCCACAGAAGCAAGTCAC[A/G]CATCAGGGCCCTGACTATTTTACTGCACCCAACCACCACAGCATGCCTTCTGGCAAT	A	G	0.45	0.15	0.23	0.29	0.25	0.27	0.36	0.47	0.07	0.39	0.30	0.037	0.025	0.050	0.005	0.0042			
rs2015312	X	56.39	GCGAAAAGGTGTCCCTACAGGATTCATTCTGCCCATTCGCAACATCCATGCCA[A/G]CATCGGGCTGGGTTTTCTTAGTAGGAACGATGAGGACTCCCA	A	G	0.45	0.14	0.23	0.29	0.25	0.27	0.36	0.48	0.07	0.39	0.31	0.037	0.025	0.056	0.005	0.0048			
rs1048358	X	56.48	AACTAAATGAGCATAAATTCCCCAGTTGTTTCTTCTGTTCTGTTGCTGCGTG[A/G]TTCACCCAATTAATAGAAGACACACAACACACACACATTAA	A	G	0.79	0.14	0.23	0.29	0.25	0.27	0.36	0.48	0.07	0.40	0.31	0.183	0.025	0.056	0.005	0.0048			
rs2043183	X	56.49	AAAACTAACAAACAACTGAGAGGATTTGGAAGATTGGAAAGGATACCATAGATATC[A/G]GATTCAAAAATTCTTCTACTCACAACCTAAACCTTTATATATACTTTTAAAACAAGATAA	A	G	0.45	0.14	0.23	0.29	0.25	0.27	0.36	0.48	0.07	0.40	0.31	0.037	0.025	0.056	0.005	0.0048			
rs1011526	X	64.28	ATAGCGACATAGGAGATGCGTGCTAATATCATGATGTTTGTATTCTACTTCCAA[A/G]TGATTCAACAAAAGCAAACAAACAAAACTGTATGTATGTGTGTGTAGATGTATATACA	A	G	0.83	0.18	0.20	0.28	0.27	0.00	0.07	0.18	0.29	0.18	0.22	0.177	0.023	0.000	0.085	0.0000	1		
rs760866	X	64.28	AGGCTTAGAGTCTGAACTTGAAGAAGGTATCTAGAAGGGCTAAACCAAGGTTAGA[A/T]GTGGTTTAGTAGCATGATGGAACCAGAAAATAAATAGGCCAGATGGAAAAAAAAC	A	T	0.21	0.82	0.80	0.71	0.73	1.00	0.93	0.82	0.71	0.82	0.78	0.146	0.023	0.000	0.085	0.0000			
rs1264216	X	64.30	ACTTTACATGCACTCTCTTGAGATTGGGAGAAAGTTGAAGATGACCACTACCATC[A/C]CTAGTATTGGTGATCCTGGCAAACATATATGGAAATGGAAAGGAATAAGAGATAGA	A	C	0.67	0.83	0.80	0.71	0.73	1.00	0.93	0.82	0.71	0.82	0.78	0.002	0.026	0.001	0.090	0.0001			
rs1777646	X	67.06	ATCTGACTAATTTTAAAGCAGCCATCATGAAAATCATTCATTAAGGAATTACAAAC[A/G]TGCTTGAAACAAATGAAAAGACAGAAAATCTTGGCAAAGAAATAGAAGATATAAAG	A	G	0.48	0.40	0.49	0.56	0.50	0.55	0.30	0.58	0.46	0.46	0.41	0.000	0.027	0.001	0.001	0.0014	1		
rs1317201	X	67.36	CCAAGCA[A/G]TTTGAGGTTCTCAGGAAATGGGTGGCGCTGAGAGGGGAAGAGATGTGTTGAGAGCACTGTTCATTTCTAGTTGAAAATCCCACCCTGCCTGCACCCACTACC	A	G	0.60	0.66	0.65	0.62	0.54	0.64	0.50	0.71	0.64	0.57	0.55	0.002	0.014	0.001	0.001	0.0018			
rs1171943	X	67.60	TTCTCCAGTGCACAGATGCATGAAGAAAATGCTAATAATCTCCTCCAACTGT[A/C]AGAGGTAGTGCCCAGCTGGGACTACTGAGAGGCTGGGCTGAGAAGCTGGGCCGACTGGA	A	C	0.48	0.30	0.29	0.26	0.27	0.59	0.95	0.31	0.21	0.33	0.30	0.033	0.268	0.000	0.056	0.0001	1		
rs1327476	X	68.14	CTATCATGCTTGATTTTTTTTTGCCTTCCATCACTAATGTCCTGCTTGGTTT[A/C]TTAGGCAGGCTCCATTTTAGTTTGCTCCAAAGCAAATAGGGTGAGAGAAAGCCACGAGAAC	A	C	0.31	0.35	0.37	0.39	0.34	0.55	0.83	0.59	0.32	0.35	0.35	0.000	0.133	0.042	0.028	0.0019	1		
rs6780	X	69.15	CAATCCTAATCTCTCTGATCAGGGTCAGTCAGAATCAGCAATCCTTTTCC[A/C]CAGTGCCCTGCATAGAAAATCCAATCTTTCCATGATAGGGAGTCTAAGC	A	C	0.13	0.59	0.42	0.46	0.45	0.59	0.31	0.57	0.42	0.40	0.45	0.027	0.000	0.027	0.037	0.0034	1		
rs174126	X	72.38	TCATGAAAAATTAATGGACTCTAGGAAGACAAAAGGTACTTCTTGTTTTCCAA[A/G]TTGTTTTACATTTTTGTAATGGTAGCTTTTGGAAAGTTAAGGCCAGTCCATGTCAGATT	A	G	0.79	0.91	0.88	0.84	0.96	1.00	0.84	0.85	0.79	0.88	0.87	0.005	0.001	0.001	0.060	0.0001	1		
rs242461	X	74.11	AATTTTAGGGTTTCCTAAGAGGAAAAACATTTTGAGTTTCTATCATGGATGAAT[A/C]AGCTAAGTATCAAGGTTTTGGACAATTTTTAAAAGTTCATTTCTTCCTATATCTTCCTAC	A	C	0.24	0.26	0.21	0.18	0.16	0.91	0.58	0.49	0.38	0.27	0.24	0.000	0.076	0.046	0.275	0.0007	1		
rs1432041	X	74.89	ACTATTGTTTGTGAGGTGTATAAATGCAACATTGACACTGTGAAAGAGCTGCT[A/G]AATAAACCCATGTCTCATCTCCCTGCTGTCTCTCAAGTGTTCTTCCAGCTCCCTGACTC	A	G	0.24	0.79	0.80	0.83	0.75	0.23	0.51	0.68	0.79	0.86	0.86	0.155	0.042	0.005	0.159	0.0065	1	1	1
rs232963	X	75.00	GTGGATCAGGTGGACTTGGATTTGTTTTATACTGGACTAGCTATATTCCTGAATACC[A/C]AAATTTCAAGTAAATTTAGGAAAATGAAAGGGGTGATGAGCACTTGGAATTTGTTATT	A	C	0.76	0.22	0.20	0.17	0.25	0.77	0.49	0.32	0.21	0.14	0.14	0.155	0.042	0.005	0.159	0.0065			
rs1868410	X	75.17	CTGGGTGGGCTTGGGATGGAGTTGGTGACTAGCCTAAGAGCTAACCATGCTAC[A/G]GGATCCTTTCCTGAGATATCTGTTTCCTAATCTATACCTTCAAGGTTCCTGGTCTATAG	A	G	0.52	0.63	0.68	0.73	0.68	0.95	0.79	0.80	0.54	0.76	0.72	0.011	0.008	0.016	0.076	0.0014			
rs1448986	X	75.21	GACATATTACTACTGCAATGACAGGAGAGTCCTTTAGAAATGCAAACCTTTTCTATTTGATTAGCTTACTATGCAACAG[A/G]CTCGTTTCTTCTAGAAGAAGGTATTCAT	A	G	0.48	0.38	0.32	0.27	0.32	0.05	0.21	0.20	0.46	0.24	0.28	0.011	0.008	0.016	0.076	0.0014			
rs10991	X	76.14	ACTTTGTAGAATTTGTAAAATAATGTTC[A/G]TATCATAATTGAGAATTTATAGCCAAGGAT	A	G	0.26	0.24	0.22	0.18	0.25	0.45	0.56	0.41	0.25	0.21	0.23	0.002	0.041	0.016	0.023	0.0002	1		
rs1337127	X	77.19	GTGGGAGGTTAGAAAGGGAGTGTTGCTTCCTCTCTAGGAACAGAGCAAAGAAA[A/G]CATTGGTGAGACACTGATAAATAGTCATTCCACATATCTCCTCTTTGATTTCCCAGC	A	G	0.57	0.43	0.44	0.40	0.45	0.64	0.94	0.41	0.54	0.48	0.37	0.007	0.007	0.013	0.043	0.0031	1		
rs1935074	X	78.93	AATTATAATTTTGATGCGATTTTCATTCCTCGTAAGAAACAAGTGCAACATGGA[A/G]GGTTTAGTCAAACGACACATAGTTTTGTACAGGATAGTGCATGTGCAATATTATAGTT	A	G	0.57	0.62	0.55	0.52	0.55	0.09	0.44	0.23	0.68	0.49	0.53	0.001	0.013	0.065	0.137	0.0019	1		
rs1569512	X	80.71	CAAGAACATTACAAATCAATTTAGAAAGGTCTGTGGCTACAACTCAGGCAAAAT[A/G]TTAGTTTTTAACTAGCTGTGTCTCTGGGATGATGAACGTCAGGCCTGTTTCTATC	A	G	0.33	0.55	0.54	0.65	0.68	0.82	0.98	0.74	0.50	0.54	0.51	0.024	0.146	0.018	0.043	0.0001	1		
rs1544221	X	82.21	TGATAATATTTTCACAGACAGATGAAAGGAGCATATTTCACGTGGATGGGCACAAT[A/G]TAAGAAAAGGCATGGAAATGTTCTGAAAGAAGGAAGAATCTGATACTGTTGTAAG	A	G	0.85	0.57	0.58	0.61	0.50	1.00	0.89	0.84	0.61	0.37	0.51	0.038	0.049	0.034	0.152	0.0041	1		
rs1358741	X	82.59	AGTAAAAATAATACTCATCAAAATTTCCGTATAACAGTTTTTTTTAAATTCAAA[A/G]TGCTTTCACGTACATTCAGGCTCAACTGAGCACTTAATTCAATTATGAGCAACTTTACA	A	G	0.60	0.38	0.33	0.20	0.46	0.59	0.29	0.41	0.29	0.43	0.41	0.039	0.000	0.000	0.036	0.0048			1
rs222102	X	85.67	TCACTTGTATGA[A/C]TAGTGTGTATCTTGGTTAAAATAAATGTTATGACTGTGTAGTTACACCATTTACATTCCCTTTCAAGACAGAAAACTGTATACATACATGAAATAGTTAAA	A	C	0.86	0.79	0.73	0.78	0.82	0.64	0.20	0.73	0.71	0.77	0.72	0.011	0.171	0.001	0.005	0.0012	1		
rs2031120	X	85.84	TCCAAGGCCAGGCTCTGCCATTCACTTAAAATAGCTTGCCAAAAGTTGTAAGAG[A/G]AGAGAGGTTACTTGCAACATATTGGCAATGGTAGCCAGTTGTTCCTGGTACATGA	A	G	0.57	0.31	0.23	0.29	0.25	0.32	0.55	0.30	0.29	0.27	0.22	0.071	0.058	0.015	0.007	0.0000			
rs2056969	X	85.89	TATATTGATAGCAATGGTTGAATCCATACTGGAAATACTACACAGCGTTGAAAAT[A/G]AGGTGAATCTACATGTTCTGATCTGGAGCATCTCAAAATTGTAATCAAGTGAAAAAT	A	G	0.33	0.68	0.78	0.71	0.75	0.36	0.44	0.57	0.69	0.73	0.78	0.110	0.058	0.041	0.095	0.0000			
rs2051591	X	87.14	ACAAACAAAATGTATAACGGAATTACCAGGTCTAATACATTGAATTTTAATA[A/C]GTAGATGGTGCCTAGAATTTTCTATTGATATGGAAGTGCTGGGAAAGGAAGAGCATAGTC	A	C	0.33	0.46	0.43	0.45	0.48	0.09	0.51	0.56	0.39	0.45	0.44	0.001	0.004	0.005	0.073	0.0004	1		
rs680757	X	89.34	CAAATTACTTTCCAGCAGAGGTAATTCCCATAGAGGCTAATAGTTAAAGGCTTT[A/G]TGCCAAGCCTCACTCCACACAGCTAGAGGAAAGAAGTGCCTCATTTATTTAGGAAAT	A	G	0.67	0.43	0.43	0.50	0.34	0.09	0.02	0.46	0.61	0.45	0.40	0.034	0.122	0.005	0.068	0.0008	1		
rs525869	X	89.37	AATAGGTGACAACATTGCCATAGGTATCCATTGGGGGTGCAAACCTGTTTGG[A/G]ATGAGAGTTGTCAAGACGGTTGAGTACCTGTAGGAGGGATTAAGGATGTTACTTATCT	A	G	0.10	0.58	0.57	0.50	0.66	0.91	0.98	0.47	0.36	0.53	0.60	0.152	0.117	0.017	0.064	0.0004			
rs1402076	X	91.29	CAATGCCATAGTTCAATAATAGAACCTTGGATAAATATTACTGGCACCTTCCT[A/G]TGAATGACTGTAAATGCAGCAGGTACTCCACATGTAATGCTGTTGTTTCTCATCAGG	A	G	0.19	0.53	0.40	0.43	0.55	0.18	0.15	0.22	0.29	0.48	0.42	0.029	0.065	0.034	0.046	0.0000	1		
rs2030392	X	91.33	GATTTATTTCCTAATTCCACTAAGAATGCTTTGGGTGATTAACCTTTTTTCCT[A/G]TGCCCTCCATAAACCGAATGAGTTAAGTAAAACAACGCAATTGGGACAGTTCCTTAA	A	G	0.26	0.49	0.36	0.44	0.54	0.14	0.11	0.24	0.32	0.40	0.39	0.004	0.063	0.020	0.046	0.0005			
rs1409304	X	91.54	AGTGTGTGGTGACTTGGTGGTCAGTTTTGCCAGGGGCACAGCAAAATGACTG[A/G]CATCATCCCCAAGAATTTCCTTAGGAGCAGGCTGGCAGCACACAGTCATGGCA	A	G	0.24	0.51	0.47	0.43	0.52	0.14	0.04	0.16	0.43	0.33	0.42	0.037	0.158	0.049	0.071	0.0010			
rs1207457	X	91.83	CATGTAGGGGCACTGTGTCTCTTTCTCTTTGTTCAATTTTACTTGTTCCTTC[A/G]TCTGAATCTAAATGAAAACTTAAACTCCACACACCAACTGTCAAACCCTTCCAGACTCAAT	A	G	0.48	0.45	0.45	0.50	0.39	0.50	0.54	0.51	0.25	0.45	0.53	0.000	0.000	0.002	0.000	0.0010	1		
rs980114	X	93.20	CAGAGGAGAAGTCAAGGCCTCAGTTAACAGATACTAAGAAATAATTAAGTGCCCT[A/C]ACTTTTGGATGAAAGAAATAAAGTTCACATTGAGCATAATGTGGTCACTTTAAACT	A	C	0.33	0.71	0.71	0.66	0.82	0.64	0.20	0.45	0.77	0.77	0.81	0.081	0.147	0.036	0.003	0.0048	1		1
rs927819	X	93.78	CAGAAAAATACCTCATTTTATATAAAAATTTAGTGATTGATAGACATCTAGCTCTTGGCACACA[A/G]AAATAGTTTACTGAATTATCTGGGCAGTATATTATGACCCAGAGAGGTT	A	G	0.69	0.30	0.25	0.29	0.20	0.00	0.03	0.16	0.00	0.21	0.28	0.111	0.046	0.006	0.090	0.0012	1		
rs829290	X	94.94	AAAACTAAAATTGGCTGTTTTAAGTCATTAATGTTTTCCCCTGTAATTCCTCC[A/G]ATCAAATGAGTTCCTTTTCCTCAAAATCTCCATGTAATATTTATCTCTACTGCTTGACAT	A	G	0.17	0.49	0.41	0.51	0.61	0.45	0.11	0.50	0.75	0.40	0.40	0.029	0.049	0.015	0.001	0.0000	1		
rs897918	X	95.58	ACTTCAGCAGCATCTGCTATAAGCTTTCCCATACCTTATACACTGAATAAATC[A/G]TGAAGGCTGATGGAATCCTGAAATGAACCCATAATCCAATAAAATGAGAAAGGCTAAAC	A	G	0.52	0.52	0.46	0.45	0.46	0.55	0.56	0.60	0.50	0.51	0.52	0.004	0.013	0.009	0.005	0.0049	1		1
rs241856	X	96.76	TTTGCTATTTTATAAGCAAGACATTGGATCTCTTTGAAGAGGTATACGGTTTC[A/G]CAGATAGTACAGCATATTCCTAAAAAAAGGATGATGTGACTCTTATTCAGCGCAACTTAC	A	G	0.14	0.54	0.43	0.46	0.54	0.05	0.25	0.28	0.46	0.47	0.43	0.052	0.012	0.005	0.123	0.0000	1		
rs1004006	X	98.26	TAAACACACACACATGCAAAACAATGCTTCAGTGAACAATCTTGCACAGATAT[C/G]TCTTGTGCTTACCTAATGAAATTTTTAGAATGAGAACCCATCGGTCAGTTCGAACTTCT	C	G	0.40	0.39	0.46	0.41	0.45	0.91	0.48	0.66	0.46	0.42	0.49	0.000	0.001	0.028	0.131	0.0002	1		
rs1204395	X	98.65	TAAACTTTGGTTCCTTTGGACTATTCTGTGAGGGTTTTTGTTTGTTGTTTGCAGG[A/G]AAAAAATTAACCTAAGATATTAATGCCATTTCTACTTTTCCTCACAGCATATATAAGA	A	G	0.60	0.31	0.38	0.43	0.23	0.82	0.41	0.29	0.43	0.38	0.41	0.023	0.000	0.017	0.090	0.0000			
rs761843	X	99.09	GAGGGAGTCTTTTGGGAGAGGAGGGAAATAGTGGAATTGAGTGAAGGTCCAGT[A/G]GGCATGTAGAGAAAAGTTAACATAACAGGAAAGCTCTTATGTTGTCTGTGGCAGG	A	G	0.30	0.48	0.46	0.35	0.61	0.95	0.65	0.61	0.42	0.42	0.49	0.017	0.011	0.034	0.166	0.0008	1		
rs876151	X	99.29	TATGAGATGGAGCGGGTGGTGGGGGGTGGTTAGAGAAGTGATACAAATGGC[A/G]CGCTCCAATATTTGGGTTGAATTACCATGCTGTAGTGGAACTCACACAAGGATGGAACT	A	G	0.19	0.33	0.37	0.43	0.43	0.09	0.16	0.18	0.29	0.30	0.52	0.029	0.048	0.025	0.078	0.0090		1	1
rs978853	X	101.22	GTAAATCATCTGCCTCATCCATCAGTGGGGGTGAGATCTGCACTGCCTCCCCAAACAATGAAACCCAAAGCACAGAA[A/G]AATGTTATTTCATCTAGAAAGCTGCCTAAGTTAC	A	G	0.69	0.45	0.40	0.30	0.46	0.55	0.74	0.48	0.50	0.42	0.28	0.059	0.084	0.002	0.017	0.0056	1		1
rs471416	X	101.80	TTTAAAACAACTGGCTGGCATATAATAAGAGGGGCACCTGTTGGAAGACTCC[A/G]GAGAAGGAAGGTATGGTCTCCTGGAACTTTTCCTGTGATTTTGCCTTGGGAGAGGAGA	A	G	0.71	0.70	0.66	0.74	0.73	0.36	0.86	0.51	0.57	0.67	0.71	0.004	0.049	0.006	0.035	0.0019	1		
rs988176	X	103.01	GGCTGAATACATTTTGGTCAACTAGCATTAGGCAATAAAAAAAAATGAATGT[A/G]CAAAATCAGTGTAACTTTTTATTTCCTTCAAGTGACTCTGCTCGATTTACAATGTCAAGGT	A	G	0.57	0.47	0.39	0.49	0.29	0.64	0.19	0.29	0.36	0.46	0.40	0.027	0.023	0.001	0.042	0.0006	1		
rs1757442	X	104.18	AAATCCAGGCTCCTCTATCTGATATATTCAATCGCTGTACTAAATCAACCCAAA[C/G]CTGTCAGTCTTGCAACTTTCATTGTGACAGTCACAAAGGCAGTGTCACAAATTACAGA	C	G	0.93	0.63	0.67	0.62	0.75	1.00	0.97	0.82	0.93	0.66	0.66	0.056	0.088	0.008	0.140	0.0002	1		
rs1152324	X	105.07	CTTTATCTCCAATTTTCCTCCTTAATTCAGTTTAGTCAGGCTTTTGGCCCTACC[A/C]GTTTACTGAAATAACTCTTTAAGGCCACCAGTTGCCAAAAGATGTCATGGCCATCTTT	A	C	0.79	0.20	0.18	0.26	0.09	0.50	0.61	0.24	0.00	0.20	0.20	0.197	0.116	0.011	0.061	0.0002	1		
rs980099	X	105.08	ATTTGCATATTAATTAATGCATAGCTTCTAATCCAACCTGGAAGAATAATATGC[A/G]AATTTCAGTCTTATGACAGACAGATAATATGTTGTTTTGGGGGAAGGCTTTGTAGGAT	A	G	0.24	0.80	0.83	0.74	0.91	0.55	0.58	0.74	1.00	0.80	0.81	0.181	0.045	0.017	0.046	0.0000			
rs1885643	X	105.26	ATTATCATTTATTGAATAAAGTATAAAGGAAATCTAAGTCTAGCACAACCTATAT[A/T]GTAAACAGATTTAAAGGATGGACTCTACACGGTTTTTGCACTCACTTTTTTTTCCTGA	A	T	0.57	0.80	0.83	0.74	0.91	0.55	0.58	0.74	1.00	0.80	0.81	0.041	0.050	0.020	0.050	0.0001			
rs1883411	X	106.17	TCCAGCCAAGCCAGAAGTGACAACCACTGGGGCAGGTAAGCTGTTGTTCAATGGCTCTCAGCTAGCAGACCCACATATAGC[A/G]TGTTTTATAGAGAAAGAAGAATTGTGGC	A	G	0.57	0.51	0.47	0.33	0.61	0.23	0.07	0.22	0.54	0.40	0.38	0.001	0.105	0.036	0.038	0.0047	1		1
rs537111	X	107.49	ATTGGAAACATAGACAGGAACTGATACAGGTCCAACAACATGTAGTAGAATCC[A/G]TGAACATTCTGGTGTTGTAAGTCCATGAGACACTAAGGTCAATCATTAATCATCACAC	A	G	0.83	0.59	0.48	0.51	0.39	0.23	0.07	0.39	0.46	0.39	0.54	0.061	0.126	0.008	0.044	0.0029	1		
rs697829	X	107.63	AAAACTAAATCAGGGTCTTACCACTGGGATGAAAACTGGGGAATCAAAAAATCATA[A/G]TGTTCCCACCTCCCCCTGAGCACTCCCTCCCCTCCGTCCCCC	A	G	0.17	0.49	0.41	0.54	0.67	0.73	0.91	0.85	0.78	0.54	0.72	0.027	0.043	0.009	0.060	0.0000			
rs2016878	X	108.70	TTAATGGTGTTCGGCACTGTTTGGTGGTAGGAAGGAAGTGATAAGTCAGGGATT[A/G]TGATGAAACAGAGACCAAGCCCTCCTGGGTAACTTGTGAAGGGATAGCCTTTTATGCCT	A	G	0.64	0.23	0.42	0.34	0.41	0.00	0.00	0.22	0.29	0.42	0.52	0.025	0.176	0.048	0.176	0.0059	1		1
rs1890141	X	109.11	AGTCACCTCATCATTCTGGGCCTGCTTAGTATCAAGTGAAAAGGCCTGCCTTAGTTG[A/C]CTTGGGTAAGTGTTGGAAGAACTAAATCAAATAGGACAGATGTGAAATATTTACA	A	C	0.14	0.00	0.00	0.00	0.00	0.00	0.00	0.00	0.00	0.00	0.00	0.052	0.000	0.000	0.000	0.0000			
rs926412	X	109.26	CTGGAGTATTTCTTTAGCATCAAATCTTCCTTTGCAGCTATTGTGTAATATTCA[A/T]CAACCAGGATCCCTACGGATTGTCTCAGGTGCTTGTAAAACACAGCAGACTATGTCTT	A	T	0.74	0.99	0.99	1.00	1.00	1.00	1.00	1.00	1.00	0.99	0.99	0.101	0.000	0.000	0.000	0.0000			
rs1016231	X	109.50	ACTAAGAGCCACTGATAATCCAATGTGGCTTGAGTACAGGGTATATGGAAGAAGT[C/G]GCAGGCAGTGCCTAGCTAAGGAGCTCTTCAATGCTATCCAGCAGTTAGTCCTCTTCC	C	G	0.52	0.00	0.00	0.00	0.00	0.00	0.00	0.00	0.00	0.00	0.00	0.229	0.000	0.000	0.000	0.0018	1		
rs3027802	X	109.77	TCTTGAATCCTGAAAAGAATTAAAATTATAAGGTTGTAGAGAGGCTGTCACATAAG[A/G]TCGCCCCTAAAACATCTGGAGTTTTGGGGAAAGCTATAGGATGGAAAATGGGTT	A	G	0.19	0.00	0.00	0.00	0.00	0.00	0.00	0.00	0.00	0.00	0.00	0.071	0.000	0.000	0.000	0.0000			
rs7049660	X	110.32	ATAAAACAATATATTTTGAATAACACATTCAAATCAAAAGATACTCCAAAACACATGA[A/G]GATGGTTGTATATGTTGGGGGGAAAATGGGAAAGGGATGGAGGCTAAAGGAG	A	G	0.67	0.84	0.83	0.82	0.84	0.86	0.38	0.95	0.79	0.79	0.81	0.021	0.106	0.021	0.001	0.0002	1		
rs2040497	X	110.52	TAATTACATCTGAATTAAACCTGGAAGGAGGAAAATGACATAGGATTATTTTCT[A/G]CACCCATCACGAATTCACTGCCTGTAGCGTTGAATTTTGTCTCTAAAGTGATGGGAGCC	A	G	0.60	0.77	0.77	0.83	0.86	0.45	0.74	0.77	0.57	0.90	0.81	0.031	0.001	0.001	0.077	0.0007			
rs583430	X	110.78	GATACTGCTGCAATCCACCTCTCCCCCGCTGCAGCATAGTACTTTGGGTTTCT[A/T]GCTATTCCCTTGGAGGTGATGATCAAAAATGCCTTTTAAGACATATGCTTAGAAT	A	T	0.17	0.82	0.88	0.90	0.82	0.91	0.52	0.79	0.57	0.88	0.83	0.232	0.051	0.000	0.004	0.0003			
rs1883366	X	111.00	ATAGTAAGTATGTTATAGATAGTACAGTTATAAATGGTAGGCTGTCCCAATGA[A/G]CAGGCCTATTTGTTGTGTTTTGAAGAACACTGTGAAGGACTCTAGCCTCCCAGATAGC	A	G	0.21	0.53	0.57	0.55	0.52	0.41	0.66	0.50	0.61	0.59	0.54	0.072	0.000	0.015	0.024	0.0022	1		
rs968859	X	111.14	TTTCTGGGTCCATTTTCCAATTCTCTGTCCTTTATCCTGCTATTTACAGTATAC[A/C]TACCCTGTACTTCTTGAGAGCTTTGTTTATTCTTTGGCTCTTTCCCCTCCCCACTCCCT	A	C	0.12	0.40	0.46	0.45	0.46	0.73	0.18	0.45	0.21	0.36	0.41	0.054	0.017	0.021	0.069	0.0000			
rs981780	X	111.20	ATATTGGTTCCTTTTAAAAGAGAATTTCATAGCCAGGTTATTAGTTGCTACATATAAT[A/G]TAAATTTCAAACCATGGAGATCTTTAGTAAAGTTTACTCATCTGTCTTCTAATTTTA	A	G	0.14	0.40	0.44	0.38	0.43	0.73	0.22	0.48	0.21	0.35	0.35	0.032	0.003	0.030	0.084	0.0000			
rs761544	X	111.67	AGCCACCTGGAACTGTTAGGTGAACAATAAATAAAATTCCATCTATTGAGCTATCCCA[A/T]GTTTGGGTCTATTTTATCAGTTAGCCTACTCTTAACTACCATGGGGAGAGAGA	A	T	0.57	0.33	0.44	0.44	0.43	0.23	0.35	0.32	0.32	0.32	0.38	0.007	0.004	0.011	0.031	0.0002	1		
rs1108444	X	112.10	AATTGCTCTGTCACACATCAGCGTATAAAGCAAGGTCTCAGTTTTGTGTTCTCTG[A/C]CTTATCATGATGACACTGTCGATTGTTGGTGAGAATGGGCTGAGGAATTTGGAAGA	A	C	0.26	0.53	0.49	0.49	0.43	0.86	0.90	0.61	0.54	0.46	0.47	0.018	0.152	0.034	0.134	0.0003			
rs540285	X	112.54	AAGCTAGCAGCATCAGTGGCAGGGGTAGCAGCAAATATTTGTTACCTGACATCC[A/G]TGCTGTGTGCTGAGACTCTCATTCTTAACATTAGCTACAATTTTGCCTGGGATAC	A	G	0.33	0.38	0.31	0.24	0.39	0.18	0.41	0.35	0.07	0.35	0.31	0.015	0.031	0.009	0.000	0.0006	1		
rs1577454	X	112.86	ACAAATCCTCACTTAGCTGACTTATAGATTTAATGCAATACCAATTTTAAACAATA[A/G]CATATTTTGTTTTTGGGGGTTGACAAGCTAATTTATCCTGATTTTTATATGAAAATGAAAGG	A	G	0.48	0.85	0.84	0.83	0.82	1.00	1.00	0.90	0.93	0.82	0.91	0.105	0.036	0.000	0.036	0.0043			
rs7056311	X	113.24	CTCCCTCTGTCTAGTAACTTGATCTTCATGG[A/G]TCCTTAAAATCCCTGTCTTCATATGCCTTTAAAAGGACTAATAATGTGTTTTAGGATAATTACCACATACTATA	A	G	0.52	0.23	0.28	0.32	0.34	0.23	0.04	0.18	0.25	0.30	0.17	0.062	0.030	0.000	0.001	0.0057	1		1
rs2231	X	113.36	TCTGAGAAGAACTCAGTTCTAAAGTGTTCAGTCTTTGCAAATGCTTTATGAGTTTTC[A/G]TTTCCTCCTTTACAAAATGCCATCAATTCCTCAAGGAAAAAAAAAAAACGTTTCTTCT	A	G	0.86	0.89	0.78	0.79	0.89	0.86	1.00	0.77	0.86	0.76	0.005	0.080	0.030	0.005	0.0000				
rs1523192	X	114.05	CCCTGCTTCATCTGGGTTAGGATTTTCTGGAGACCATTGGCCTGCTTCATAGTC[A/G]ATCAACACCAGCTTACCTAGGACCACTTACACCCAGAAAGTAATATAAAGTAGGAC	A	G	0.19	0.50	0.61	0.63	0.43	0.82	0.36	0.69	0.39	0.62	0.59	0.109	0.015	0.001	0.032	0.0010	1		
rs988457	X	114.44	ATTAATGGTGTTTCTGTTCATTCAGCCACCTAAACTTGAATTTCATAATCATTT[C/G]ATCTTCTGTCTTCTCTCCCAAGACTTCTTCTCTAACCTCCAATCAGTTACTACATTTTATCCTTCT	C	G	0.45	0.59	0.55	0.50	0.57	0.23	0.43	0.48	0.32	0.49	0.59	0.011	0.015	0.010	0.083	0.0016			
rs4462068	X	114.88	CTTCCATGCTCTGAGAGGTGAAAAAGTGCTAACCTGCTGTAAACTCAGAGAGTTTC[A/G]CAGCATCTTCATTCTCCAATGCCTGGTAGATATAACAACGAGCTTTAGTAGTCTC	A	G	0.48	0.35	0.43	0.43	0.48	0.64	0.67	0.45	0.39	0.39	0.46	0.000	0.026	0.001	0.013	0.0000	1		
rs1558022	X	115.26	CTTCTAATGTCCCCTCAACTAAATTTTTCATCAGCAAAACTGATTTGTGAAATCA[C/G]TCCTATTATTCCCACTTTCCATGAAATTTTGTAGTGAGTTCCAACCTCTCTCTACAGA	C	G	0.79	0.50	0.50	0.52	0.54	1.00	1.00	0.79	0.58	0.43	0.49	0.048	0.223	0.055	0.223	0.0049			
rs2040962	X	115.36	CCTTTGCTGCTAGTAGCTCCATCATTGCCTTACTCTTCCATGCTACAAACATTGAA[A/G]TCTTTCTCTCCTTCACTTAGAATATTCTGGAGCACCTCTGTTCAAAGACTAGCTA	A	G	0.31	0.54	0.51	0.52	0.50	0.05	0.04	0.21	0.46	0.58	0.56	0.023	0.155	0.066	0.159	0.0003			
rs714597	X	115.75	AGGAAGGAAGCCATATGAGCTAGGGTCTTTTTAAATTTGGCACTACAAAAAG[A/T]GGTGCTCAGCAGGAAACATTTCTAAAATTTAATTTTAGTAGGTGGCTGGAG	A	T	0.60	0.70	0.70	0.71	0.73	0.32	0.41	0.44	0.79	0.73	0.69	0.005	0.032	0.041	0.056	0.0000	1		
rs1716758	X	116.21	AAGCATGAAGGGAGGAAGCATCATGGAAAAGAGGCACCGGAAATGTGGAAATTTGTTC[A/G]TTTAAGTGACTGGAGAATTGGCATTGAAATGAGGTAAGCCAGAG	A	G	0.81	0.24	0.23	0.26	0.11	0.65	0.92	0.32	0.26	0.20	0.25	0.229	0.318	0.073	0.138	0.0006			
rs929590	X	116.70	GGACTGAACCCCTGAAAGCAGAATACTTTCAGTCAGCTACAGCATCCTAGCACTGA[A/G]ATTGAGATACTCCATGCTGCTGCTACTGTGCTCTAGCAACTGACCAAAGTTCT	A	G	0.10	0.15	0.16	0.24	0.20	0.64	0.84	0.20	0.21	0.20	0.22	0.004	0.270	0.001	0.131	0.0012	1		
rs2018358	X	117.48	TGTATCTCAGATAAATCAAGTATAAACCTCATAACAGCACAGGTGAATTTACCATTG[A/C]TCACTAGACACCTCTAATGAAATGATGTTCAGGACAATTAGGTGGTTTACAGTGCTT	A	C	0.79	0.66	0.65	0.77	0.79	0.82	0.89	0.78	0.71	0.62	0.74	0.016	0.060	0.026	0.032	0.0050	1		1
rs2748	X	118.31	GTAAACAATTGTTTTTCACCTAAGCTGCTTTGGAAGAACTGATGCTTGCTGCTAACT[A/G]AAGTTTTGGATGTATCGATTTAGAGAACCAATTAATACCTGCAAAATAAAGCATAC	A	G	0.88	0.61	0.61	0.59	0.54	0.50	0.85	0.68	0.82	0.67	0.59	0.028	0.035	0.005	0.010	0.0022	1		
rs1034606	X	118.96	ATGTTTGATGACTGACTGATACTACGGTCACACCCATTTGACGTACTTATAGA[A/C]CTTATATCATTGAACCTCATTTTCTTTATTTACATTTTATCTATCCTTGCTTTTATGCCAA	A	C	0.76	0.56	0.69	0.56	0.59	0.41	0.30	0.30	0.68	0.61	0.68	0.005	0.088	0.069	0.043	0.0002	1		
rs1522889	X	119.22	TTTGAGAGGAATTTTGGGGAAATAAGGGACCAGCTTGGAGTTCTGCAGAATAGATG[A/C]AGGTAGATCCAGAGAAATGGATGAGAATGGTAAAAGTGAGTTTCAGCTAATTGTT	A	C	0.36	0.37	0.48	0.56	0.54	0.05	0.02	0.30	0.39	0.53	0.56	0.004	0.140	0.001	0.118	0.0049			1
rs988246	X	119.42	TCTAAGATCCATGCTCCTCTTCTAGGTTGAGTACTTTTTCTTCAGCTTCCTTC[A/G]TAATTCCCCAAAAGGTAGCTTTATGTATTATCAAAGTCTAGCTAGCTTGCTTCATTGGA	A	G	0.93	0.81	0.82	0.82	0.79	0.91	1.00	0.72	0.68	0.79	0.83	0.010	0.065	0.020	0.008	0.0000			
rs783279	X	119.84	ATCTCAGAGGGATCAGAGGGGTTGACAATCTATGCTTTTGGCCAAGAGTGAAAGTG[A/G]AGGAATGTAGGGACAGGACATATAGCTTGGGAGGGGATCATTTAAGACTTAAA	A	G	0.86	0.81	0.83	0.82	0.86	1.00	0.98	0.94	0.86	0.88	0.81	0.000	0.031	0.012	0.060	0.0000	1		
rs1934070	X	120.08	ATATACCTTTATTTTTCCAAATTGGTAGCAAGTTCAGCATTTCCATGGCCATGT[A/G]TGGAAGCAAAATAACTTTTTAAAAAATCCTTTGTTGTCTAATCAGTAGATAACTAGT	A	G	0.19	0.63	0.65	0.56	0.66	0.86	0.77	0.73	0.54	0.64	0.63	0.100	0.011	0.004	0.035	0.0006			
rs7881297	X	120.31	ACTATATGATATAATAGAGGTATACACAGAGGCCAGCAGAGAAAGCATGCGTGGAA[A/C]AAACATGGAAGACTTCACAGAGCAATAAAATGGCATGTGATTGGAGAATTAATA	A	C	0.55	0.99	0.98	0.99	0.82	0.55	0.93	0.96	0.97	0.99	0.130	0.145	0.015	0.048	0.0006				
rs716209	X	120.50	CAGGATGGGGGCTAGTAACCAGAAGACCAAGACAGTCTTAGAGGATTAATTTCTAG[C/G]CCCACTTCCCAATCTTTGGAAGGGAAGAGAGCCTTAAAAGTTAAAATGATCAC	C	G	0.90	0.38	0.38	0.32	0.38	0.32	0.35	0.35	0.39	0.49	0.37	0.163	0.000	0.001	0.001	0.0006			
rs1539225	X	120.70	TGTGACTAATCTTGACACTACCACTCAAAAACTGAACATGGACTGAGAGTGGGTA[A/G]TTATCAGTCACCATATGAAAAAGTCATAAATATGGCTGTGTATATACACTGAGGC	A	G	0.50	0.74	0.83	0.82	0.82	0.36	0.45	0.74	0.68	0.83	0.87	0.055	0.068	0.004	0.109	0.0063	1	1	1
rs1293544	X	120.83	AAGGGAAGAGAAATTTTCAAGGAGGAGGAGGTGATCAACCATTTCAAATGCTAG[A/G]AGCATTCAAATAATCTCAAAGAAAAATGTGGAGCTTCAAAGTTCATTGGCCTTTGT	A	G	0.83	0.58	0.61	0.63	0.52	0.23	0.23	0.20	0.50	0.65	0.65	0.043	0.056	0.069	0.065	0.0019			
rs1476014	X	121.07	AAGCTGCTCATGTTAAGATCTTCAGAGAAAAGCACAATAAAAACCTCATAATACAT[A/G]AGCATACAGCAATCCAACCTCCAAAAAACGGGAGGTTGGTGCTTGGCAACTGGG	A	G	0.86	0.80	0.77	0.77	0.73	1.00	1.00	0.85	0.79	0.80	0.71	0.011	0.129	0.027	0.129	0.0012			
rs2227098	X	121.19	TTTCTCTCTAAGCAAAATAGGCCCTCTTTCGTTTTTTCAAGTACATTTCAAAGGGATCTGGT[A/G]AGCTGCAGAGTTCACTTCCTCAAGCTTGTTGAAGCTCCTGGCTGCCTGTTTGGGTT	A	G	0.40	0.54	0.42	0.48	0.43	0.45	0.49	0.35	0.54	0.53	0.46	0.001	0.001	0.001	0.000	0.0010			
rs9856	X	121.74	GCTGAGGAAGAATTCACATTGTTAACTGTATAAGTCTAAATGAAAATGAAATATTATT[A/G]TGGAAAAGTAAAGCAAGCTTGAAATTGGAACCTTTAGAAAGCTCAATCCAGAAA	A	G	0.62	0.53	0.62	0.59	0.48	0.73	0.43	0.51	0.57	0.50	0.52	0.000	0.008	0.001	0.011	0.0044	1		
rs1798138	X	122.02	AGGTGCCAAGTTCCTTCTCCAGAAGGCAGGCCAAATTGACTGGAAGCCAAGGCACC[A/G]GGAGAATCCTGACCACCAGAAAGAATACAGAATTTCTGACATTCAATCATTCTTTTT	A	G	0.31	0.49	0.54	0.44	0.41	0.14	0.29	0.17	0.32	0.44	0.40	0.000	0.023	0.078	0.095	0.0049			1
rs726834	X	122.31	CTCCACCTCTTCCCACTTTGCAGCTATTCTGCCATATACCTAGCACCACACTTCT[C/G]CATGCTACCTTCAGTGGTCCCACCTAGGCTTGTCTTTCTCAGGTGATCTTGTCAAT	C	G	0.65	0.60	0.70	0.66	0.68	0.59	0.56	0.69	0.61	0.67	0.71	0.000	0.004	0.003	0.002	0.0004	1		
rs7055104	X	122.77	[illegible]	A	G	0.26	0.00	0.00	0.00	0.00	0.00	0.00	0.00	0.00	0.00	0.00	0.101	0.000	0.000	0.000	0.0018			

rs2066918	X	122.89	[illegible]	A	C	0.57	0.18	0.19	0.15	0.14	0.27	0.41	0.15	0.18	0.27	0.20	0.127	0.059	0.001	0.017	0.0002			
rs767216	X	123.16	[illegible]	A	G	0.57	0.93	0.92	0.91	0.84	1.00	0.96	0.89	0.96	0.89	0.86	0.086	0.003	0.000	0.027	0.0018	1		
rs209659	X	123.79	[illegible]	A	G	0.71	0.77	0.72	0.72	0.75	0.91	1.00	0.93	0.89	0.71	0.72	0.012	0.070	0.017	0.010	0.0018	1		
rs1566588	X	124.02	[illegible]	A	G	0.71	0.32	0.20	0.24	0.25	0.36	0.71	0.29	0.14	0.26	0.24	0.123	0.120	0.005	0.017	0.0013			
rs845324	X	124.19	[illegible]	A	G	0.33	0.29	0.34	0.23	0.36	0.41	0.11	0.28	0.54	0.42	0.43	0.002	0.059	0.006	0.000	0.0009			
rs2235079	X	124.54	[illegible]	A	G	0.19	0.34	0.36	0.24	0.38	0.82	0.95	0.38	0.23	0.34	0.37	0.025	0.202	0.003	0.090	0.0001	1		
rs16309	X	125.07	[illegible]	A	G	0.14	0.56	0.54	0.50	0.64	0.27	0.22	0.48	0.54	0.61	0.60	0.114	0.069	0.014	0.056	0.0041	1		
rs1883906	X	125.39	[illegible]	A	G	0.12	0.64	0.60	0.66	0.61	0.86	0.30	0.80	0.64	0.74	0.68	0.149	0.050	0.043	0.035	0.0053			1
rs2200290	X	125.69	[illegible]	A	G	0.71	0.28	0.28	0.17	0.23	0.09	0.71	0.21	0.29	0.20	0.28	0.128	0.133	0.000	0.012	0.0000	1		
rs240170	X	126.17	[illegible]	A	G	0.86	0.15	0.19	0.15	0.18	0.18	0.47	0.27	0.25	0.18	0.16	0.286	0.042	0.024	0.000	0.0004			
rs916208	X	126.65	[illegible]	A	G	0.14	0.49	0.50	0.40	0.45	0.68	0.72	0.56	0.43	0.48	0.43	0.062	0.056	0.013	0.031	0.0021	1		
rs16318	X	127.04	[illegible]	A	G	0.17	0.43	0.32	0.45	0.23	0.32	0.83	0.51	0.36	0.37	0.24	0.016	0.140	0.028	0.000	0.0034			
rs2281277	X	127.76	[illegible]	A	C	0.55	0.88	0.85	0.90	0.86	0.55	0.86	0.93	0.79	0.90	0.91	0.037	0.003	0.014	0.038	0.0064	1	1	1
rs209213	X	128.11	[illegible]	A	G	0.19	0.57	0.58	0.46	0.43	0.65	0.68	0.36	0.61	0.56	0.53	0.109	0.001	0.053	0.001	0.0011			
rs1034422	X	128.32	[illegible]	A	T	0.67	0.44	0.49	0.41	0.48	0.91	0.93	0.75	0.50	0.51	0.54	0.005	0.104	0.040	0.092	0.0000	1		
rs3788	X	128.93	[illegible]	A	G	0.26	0.14	0.16	0.13	0.14	0.00	0.14	0.14	0.29	0.15	0.14	0.010	0.000	0.000	0.045	0.0010	1		
rs717972	X	129.25	[illegible]	A	G	0.62	0.54	0.51	0.57	0.57	0.68	0.83	0.39	0.50	0.40	0.47	0.002	0.044	0.015	0.011	0.0004			
rs859852	X	129.46	[illegible]	A	C	0.48	0.42	0.40	0.40	0.41	0.32	0.28	0.45	0.58	0.51	0.35	0.002	0.009	0.001	0.004	0.0033	1		
rs202768	X	129.78	[illegible]	A	G	0.24	0.33	0.27	0.27	0.23	0.41	0.77	0.40	0.29	0.32	0.23	0.000	0.145	0.015	0.017	0.0014			
rs1005488	X	130.10	[illegible]	A	G	0.81	0.31	0.28	0.26	0.27	0.00	0.00	0.09	0.18	0.26	0.30	0.173	0.090	0.013	0.090	0.0004	1		
rs2179978	X	130.26	[illegible]	A	G	0.05	0.18	0.15	0.15	0.14	0.00	0.00	0.01	0.14	0.09	0.16	0.011	0.045	0.025	0.045	0.0000			
rs1005580	X	130.65	[illegible]	A	G	0.38	0.48	0.52	0.46	0.52	0.45	0.98	0.48	0.46	0.42	0.41	0.002	0.177	0.001	0.001	0.0014	1		
rs980840	X	131.11	[illegible]	A	G	0.52	0.26	0.30	0.30	0.29	0.68	0.07	0.50	0.29	0.29	0.36	0.021	0.031	0.017	0.085	0.0013			
rs1474730	X	131.13	[illegible]	A	G	0.52	0.26	0.30	0.30	0.29	0.68	0.07	0.51	0.29	0.29	0.36	0.019	0.034	0.017	0.080	0.0010			
rs1003856	X	131.69	[illegible]	A	G	0.19	0.78	0.75	0.64	0.86	0.73	1.00	0.75	0.93	0.60	0.69	0.133	0.129	0.000	0.002	0.0010	1		
rs175327	X	131.95	[illegible]	A	G	0.36	0.57	0.56	0.55	0.61	0.50	0.07	0.40	0.81	0.56	0.56	0.023	0.128	0.025	0.001	0.0000			
rs757308	X	132.38	[illegible]	A	G	0.60	0.33	0.31	0.34	0.21	0.23	0.80	0.35	0.39	0.18	0.22	0.045	0.127	0.001	0.006	0.0075	1	1	1
rs1982	X	132.41	[illegible]	A	C	0.29	0.61	0.61	0.59	0.71	0.68	0.20	0.59	0.57	0.70	0.65	0.051	0.082	0.004	0.006	0.0047			
rs1984391	X	132.74	[illegible]	A	G	0.50	0.42	0.39	0.35	0.46	0.55	0.22	0.20	0.18	0.48	0.41	0.006	0.038	0.021	0.007	0.0008			
rs2254857	X	133.54	[illegible]	A	G	0.19	0.22	0.28	0.20	0.14	0.20	0.17	0.16	0.23	0.17	0.25	0.008	0.022	0.017	0.010	0.0002	1		
rs725584	X	133.93	[illegible]	A	G	0.50	0.23	0.21	0.24	0.30	0.45	0.15	0.17	0.21	0.21	0.30	0.051	0.002	0.004	0.038	0.0060			1
rs1016812	X	134.02	[illegible]	A	G	0.45	0.61	0.52	0.52	0.64	0.77	0.65	0.54	0.54	0.49	0.52	0.001	0.006	0.002	0.038	0.0003			
rs877761	X	134.14	[illegible]	A	G	0.26	0.53	0.54	0.57	0.61	0.77	0.71	0.51	0.46	0.55	0.48	0.030	0.018	0.002	0.041	0.0012	1		
rs975379	X	134.43	[illegible]	A	G	0.43	0.13	0.20	0.12	0.13	0.09	0.73	0.27	0.11	0.16	0.16	0.034	0.147	0.006	0.010	0.0009			
rs1190742	X	134.78	[illegible]	A	G	0.83	0.48	0.48	0.51	0.45	0.68	1.00	0.67	0.54	0.52	0.48	0.051	0.183	0.004	0.008	0.0002			
rs2064155	X	135.23	[illegible]	A	G	0.12	0.30	0.32	0.35	0.32	0.36	0.19	0.26	0.38	0.42	0.33	0.033	0.014	0.014	0.000	0.0000	1		
rs1206233	X	135.23	[illegible]	A	G	0.12	0.30	0.32	0.35	0.32	0.36	0.19	0.25	0.38	0.42	0.33	0.033	0.014	0.014	0.000	0.0000			
rs697831	X	135.60	[illegible]	A	T	0.26	0.29	0.21	0.18	0.32	0.18	0.26	0.18	0.29	0.18	0.22	0.006	0.004	0.000	0.000	0.0024			
rs1539270	X	135.72	[illegible]	A	G	0.71	0.64	0.59	0.62	0.71	0.73	0.41	0.53	0.64	0.72	0.66	0.005	0.018	0.004	0.009	0.0017			
rs1487921	X	136.11	[illegible]	A	T	0.57	0.49	0.47	0.45	0.41	0.23	0.42	0.40	0.50	0.45	0.50	0.003	0.004	0.001	0.038	0.0001	1		
rs2017465	X	136.53	[illegible]	A	G	0.81	0.79	0.74	0.78	0.71	0.55	0.60	0.68	0.82	0.78	0.84	0.006	0.006	0.000	0.015	0.0044			1
rs728186	X	136.85	[illegible]	A	G	0.62	0.42	0.43	0.33	0.52	0.45	0.83	0.68	0.71	0.38	0.37	0.020	0.118	0.073	0.003	0.0031	1		
rs2485729	X	137.69	[illegible]	A	G	0.48	0.02	0.01	0.02	0.00	0.32	0.28	0.03	0.00	0.01	0.01	0.203	0.117	0.018	0.125	0.0003	1		
rs367697	X	137.99	[illegible]	A	G	0.45	0.49	0.42	0.52	0.34	0.41	0.56	0.50	0.36	0.40	0.45	0.000	0.006	0.000	0.001	0.0002			
rs1323334	X	138.34	[illegible]	A	G	0.76	0.47	0.42	0.46	0.41	0.18	0.48	0.21	0.50	0.44	0.39	0.073	0.005	0.043	0.026	0.0001	1		
rs1884198	X	138.54	[illegible]	A	C	0.33	0.37	0.36	0.35	0.36	0.05	0.08	0.08	0.39	0.36	0.31	0.000	0.045	0.068	0.071	0.0011			
rs1565843	X	138.76	TTAGTTTTGCAATGTCTCCAATGAACATAACTGCAACTCACAAGGAGCCAATAAG[A/G]CTCTGCCTTTAAAAAGAGTCAAGAAGAGTGGATCCCAAACAAACCCACCTCAGGAT	A	G	0.76	0.51	0.56	0.54	0.50	0.82	0.18	0.48	0.68	0.61	0.56	0.027	0.068	0.000	0.062	0.0015			
rs910677	X	139.53	GCCAAGGAAGGGAACTGAGGTTGTGTATCAGTGTGTGGTCATGGTGCAGAGCTAT[C/G]CTGGTGCAAAAATAAAGGAGTGAGGCCTTTTCAGCTCATTCTTCTACATTAATTAC	C	G	0.19	0.15	0.24	0.13	0.22	0.14	0.15	0.27	0.18	0.21	0.18	0.001	0.005	0.005	0.007	0.0025	1		
rs17407	X	139.78	GTCCCTATGGTAATAGAATGATGAAAGGAAAGAATCACGGGAATTACAGGAATCC[A/G]TTTTAGGAGTAGAAATCAAATTCTACTTTTTATATGAGGTAGATGAAGCCAGAAAG	A	G	0.83	0.63	0.60	0.59	0.61	1.00	1.00	0.92	0.79	0.52	0.51	0.027	0.146	0.082	0.146	0.0015			
rs971879	X	140.04	TTCCTGAATCTCCCCTCTCTGGATTACAGACTGGAAACTCTCACAAAACAGGCA[A/G]GTGGGATAACTGTAGGGCTCACTTTGTGCATTCTTCATCTTTGATTGATCCACATCC	A	G	0.74	0.43	0.52	0.56	0.36	0.14	0.00	0.34	0.46	0.58	0.58	0.033	0.223	0.020	0.085	0.0008	1		
rs1555622	X	140.77	CAAATGACCTTCCAGAGGAGGCATAAATTTGGTACCCAAATTTGTTCAGCATTTCAAC[A/C]ATTTTTCTACCGTCAACATATTTTCCATGTATATTTATTCTTAGATGGTTAGTGT	A	C	0.69	0.36	0.33	0.34	0.29	0.59	0.79	0.30	0.46	0.35	0.28	0.111	0.005	0.001	0.012	0.0005	1		
rs1997686	X	140.86	ACATGCCAGAAGATATTATAAAAGACATACACAGAGGTTTGAG[A/G]TTAGTGACTTCTCTAAGGGTCAGGGTTTTATATCTGGTCCAGATTGATGGAATAGGAGTGAAAGTGATT	A	G	0.40	0.22	0.22	0.21	0.21	0.40	0.37	0.21	0.21	0.32	0.23	0.020	0.012	0.000	0.011	0.0003			
rs1569855	X	141.01	TTTGAATCAATTGAGTCCATAGATAAAATAACAACAACAAAAATCCCACATCACCTCC[A/G]ATAGAAACAAAGTCAATCTATTTTTGTTGCAATGCAGATTAAATAATCAGATAATT	A	G	0.31	0.67	0.65	0.59	0.71	0.32	0.88	0.41	0.58	0.57	0.66	0.078	0.023	0.021	0.075	0.0002			
rs926356	X	141.19	GATTCAAAATCATAGGTGGAAGCATCCTATTGGCCCTTCCTTAATATGTACCCAAA[A/G]CTTCTATTAGAACATGCAAAGTATGACATTGCCCCACTTTTGTTTTCAAATTATAAG	A	G	0.48	0.64	0.60	0.55	0.59	0.32	0.34	0.34	0.89	0.51	0.49	0.001	0.016	0.029	0.025	0.0030			
rs992566	X	141.40	TGTGATTAAAGGGACCAGGAATTGCATGGTAGCCACATTCGCTGGAGCTCTAGTTA[A/G]TGATATGGATGGAATAAAGAAAACCTAGAAATTGCATTTCAGAGTCATCCTGGGT	A	G	0.79	0.53	0.45	0.46	0.34	0.64	0.68	0.77	0.36	0.57	0.55	0.048	0.029	0.046	0.020	0.0038	1		
rs2500128	X	141.80	TGTTCACACAAAGTCACTTAATTTATCAGAACATATCCCAGTGATTAAGCAACACCC[A/G]ATTGTATTAAATTCTGTGTTCCTATGTACTAGTAGAGTGACTGGAGCCTGGTTTGT	A	G	0.69	0.93	0.96	0.99	0.96	0.95	0.93	0.94	1.00	0.98	0.95	0.079	0.005	0.000	0.000	0.0004			
rs644429	X	142.47	CTGAAAAAATAATACATCAGGTAGAATTTAAAAAGCTGAATGCAAATTTTCATCT[A/G]TATTAACATGACACACATGTCTAAACAAGGTATGTGTATGCACGGGAACTGAAAACC	A	G	0.81	0.63	0.56	0.51	0.64	0.77	0.86	0.66	0.43	0.53	0.55	0.048	0.072	0.007	0.031	0.0003	1		
rs17410	X	142.69	GAGCCAAGTCCAGGCACTGTTGCAGCCTGGCCAGGTGTGCACATGCTTGGGAC[A/G]TGCTGACACACTAGGCCTCTGCCGCCTCAGCCTCCTCCCAGACTTGGGCACCGAC	A	G	0.33	0.55	0.61	0.60	0.45	0.59	0.60	0.50	0.46	0.64	0.55	0.022	0.000	0.002	0.001	0.0008			
rs500004	X	142.93	CTGCTCAAATCGCCATCATCTAGAATGTTACTGAATGCTGTGGCACAGAGAAACACT[A/G]AGTGTAAAGGCTCTCAAAACTTCCATTGGTGCCCATAAATGAAGTGTTTCACTTTT	A	G	0.52	0.60	0.56	0.57	0.68	0.50	0.56	0.66	0.50	0.65	0.61	0.000	0.003	0.022	0.000	0.0006			
rs1151983	X	143.29	CTCTTAAATATTCAGCAGAGTATTTTGAATGCATGTGAGGAAACTCGCCTAGGTAAA[A/G]GAAAGGACTACTCAAGAAGATTAAGAACAATCCCAAGTTTCAAACAGGGCTGGG	A	G	0.19	0.46	0.52	0.55	0.57	0.41	0.50	0.60	0.29	0.55	0.45	0.083	0.001	0.005	0.010	0.0042	1		
rs2057211	X	143.58	RATATTATAATATTCAGCCGATATGTTCTCGTGTAAGAGTAGAAAAGAGGAAAAAA[C/G]GTATCCATTCAAAACGTTTCTCTGCTTTCCAGAGGTTTCTGAGGGGTTTATTAAAAC	C	G	0.88	0.89	0.85	0.87	0.77	0.82	0.98	0.67	0.79	0.85	0.79	0.000	0.023	0.050	0.002	0.0047			1
rs871156	X	143.67	TGTTGGGTCTCCCGCTGTGTTGGTGATTAGGCCTCTGGGTGGAAGCAATCTCCAGT[A/G]ATCATAGGTTGCCTTATCGGTGCTATTGAACCAGTCACTCCAGTTCTGTTGGAAG	A	G	0.57	0.44	0.56	0.59	0.50	0.45	0.67	0.51	0.57	0.55	0.49	0.000	0.004	0.005	0.011	0.0043			
rs1033630	X	144.08	CACCTCCTTCAGAAATCAGATCACTTGTTAGGGAAAATATAATGAGGAAGCTGTAA[C/G]GTGTACAAGCAGCCCCTTCTTATTACCACTGGGCAAGGTTTCTTAAAATGAGATCT	C	G	0.55	0.53	0.49	0.48	0.50	0.45	0.97	0.40	0.50	0.56	0.46	0.002	0.162	0.010	0.001	0.0006	1		
rs996435	X	144.31	GAAAATTTTAAAATGGAAAAGAAGCAATATTTACAGAAATTTTAGCAGATAATTTTCCA[A/G]GCCTAGTGGATTGCATCAAACTTAGTTTCAAGAAGCATTCTGCATCCAAGCAA	A	G	0.86	0.68	0.70	0.86	0.73	0.91	0.99	0.66	0.82	0.65	0.73	0.018	0.123	0.003	0.040	0.0004			
rs1575093	X	144.52	CTGACCTACTCTAATTGGTTATGGTTTTCTAAATAGAAAATGATGAGCAATGTAA[A/G]TCTTGTAACTCAGTTGTCCTCAAGTGGGAAAATATGGAAATGGGTGAAGGCATGGTA	A	G	0.48	0.48	0.39	0.39	0.39	0.23	0.30	0.41	0.36	0.43	0.61	0.001	0.003	0.000	0.020	0.0156		1	1
rs1336835	X	144.61	GCTTTTTTGTAYTTAATGTAGGCATGACTGAAATTAAGAGCAGGAGAAGTACGTCC[A/G]GTTTCAAGTATACAGGAAGGCAACAGGATCAGGCTTTTAGTACATGCTTAAAAGCG	A	G	0.55	0.30	0.28	0.30	0.34	0.14	0.36	0.14	0.25	0.38	0.38	0.027	0.001	0.021	0.032	0.0017			
rs2004410	X	144.72	AGGAATGGGAAGGAAAAGAACAAAAAGACAATCAGGAATGAGTAGTAAGAATAAT[A/C]GGCTGCCATGCTGATCATAGGACTGAGGAGAACACAGGACGTGAAGGGTATCAC	A	C	0.29	0.26	0.30	0.22	0.23	0.36	0.56	0.39	0.11	0.34	0.41	0.001	0.015	0.003	0.000	0.0082	1		
rs560689	X	145.24	TATATACTTAAACTAAAACAAGGATAACATCATGATGAATGTTTCATTGTTTGCTGT[A/G]GATTCTAAGAAGCTTAGATCTCAATTTCATAATAGCTGTCCTCAGAAAACATTTCTT	A	G	0.21	0.25	0.15	0.22	0.14	0.14	0.21	0.22	0.11	0.21	0.18	0.001	0.000	0.001	0.005	0.0000	1		
rs1861581	X	145.46	ACAGAATGGGAGAGATGCTTATAAAGCTAGCAGGAACTTCCATTATGGCAGAGGGAA[A/G]TGGAAGTCCCTACAGAACTACGAAATTACAGAGGTGAATGGCAGATCTATGAG	A	G	0.26	0.18	0.20	0.22	0.21	0.00	0.00	0.17	0.32	0.21	0.21	0.000	0.101	0.010	0.101	0.0001			
rs4949	X	145.67	GCTTTTTTAAAAAAAAGAGTGCTTTTTGTTGGGATGTACATTTTCCAAATGCAAAA[A/G]CATTTATGATTCTGTGTCTCTTATAAAATATGACACTCTCTACTTTTCTCTCATTTATTT	A	G	0.29	0.62	0.57	0.50	0.48	0.14	0.49	0.53	0.62	0.51	0.60	0.031	0.001	0.000	0.090	0.0010			
rs1024694	X	145.85	ACGAAGTCAGTCAGGTAAGAAGGATGGTCTAGGGATCGGCAGGAAAGGGTATACC[A/C]GCATGTGCAACAATGTGGACATGTGACACCATGTGGTCTAGTCAAATAAGGGGCT	A	C	0.62	0.48	0.51	0.56	0.54	0.18	0.27	0.44	0.68	0.52	0.49	0.010	0.034	0.005	0.058	0.0005			
rs1990383	X	145.91	CAAGTCAGTGGGTGGGAAGAGGAGGAGGGGGGTGATGAGGGATAGACGCTAACACC[A/C]CAGAAAACCTAAAAATGGTGCTGGCACTTTTGTTAGCACTAATACTAACAATGT	A	C	0.10	0.28	0.29	0.33	0.23	0.14	0.15	0.36	0.32	0.22	0.24	0.017	0.006	0.006	0.010	0.0013			
rs6540401	X	146.02	AXTTCTTACCTTAGCTTTTTCAGCCAAACCAATTCCTAACCTCATACCTAAAGGTA[A/C]TGGTTGAGACGATGGCTGCTGCTGTCTTGAAAGGTAATGAGGGAGGCTGGGCTGC	A	C	0.31	0.05	0.09	0.07	0.04	0.00	0.03	0.06	0.04	0.08	0.09	0.018	0.027	0.014	0.065	0.0005			
rs1547932	X	146.31	TTTGATGTTCTAGAAGCTTTTGTGAATTTTTGAAAATAAACTTTTGCAAACCTTC[A/C]TCACCACTCTCACTGCCGCTTTCCATGAAAAAGTCATATTTAGCTGAGGGTAAACAAA	A	C	0.14	[illegible]	[illegible]	[illegible]	[illegible]	[illegible]	[illegible]	[illegible]	[illegible]	[illegible]	[illegible]	[illegible]	[illegible]	[illegible]	[illegible]	[illegible]			
rs985595	X	146.43	GAAGGTGGGTAATTGATGTGTAAATCAGAGCTCAATCACTTGGTAATAGAAATGGT[C/G]CCACTGAAAATATGCTTGTTGAACACAATCACACAGAAGCCAGAGGGAGGGAGG	C	G	0.07	[illegible]	[illegible]	[illegible]	[illegible]	[illegible]	[illegible]	[illegible]	[illegible]	[illegible]	[illegible]	[illegible]	[illegible]	[illegible]	[illegible]	[illegible]			
rs1860929	X	146.59	GGAGGCTGGAGATGGGACTTAAGTGGATATGACAACAGAGATAAGGGAGAGTGGG[A/G]ATGAGAAGGGTGGAGGAGGGGTGGGTGACATGCTTCATTATGAGGACATCTCTT	A	G	0.17	[illegible]	[illegible]	[illegible]	[illegible]	[illegible]	[illegible]	[illegible]	[illegible]	[illegible]	[illegible]	[illegible]	[illegible]	[illegible]	[illegible]	[illegible]			
rs758439	X	146.77	GACCTGCTATTAGATCCTGGCTTTTTGGCTCTCTGGGCTCTGTGGCCCTCAGGAC[A/G]GAGCTGAACTTCTCTGAGGCCAGAAACAGTGGCAGCAGAACTTTCCCTGCAGAG	A	G	0.26	[illegible]	[illegible]	[illegible]	[illegible]	[illegible]	[illegible]	[illegible]	[illegible]	[illegible]	[illegible]	[illegible]	[illegible]	[illegible]	[illegible]	[illegible]			
rs764908	X	147.08	AGTGGCTTTGTGTGCACCAGTTAACTTTCCACCAAGATTTTAGGAGTGTGACATT[A/G]GAGCCTATGAATTTAGATAGCTCATTGGTTGTGGGACACTAACCACGCACCAGTAT	A	G	0.29	[illegible]	[illegible]	[illegible]	[illegible]	[illegible]	[illegible]	[illegible]	[illegible]	[illegible]	[illegible]	[illegible]	[illegible]	[illegible]	[illegible]	[illegible]			
rs530501	X	147.27	CTTGTACTGTATATTGATTCCTGTTTAAATTTGTCTTGGTTCTGGACCATCATTC[A/G]GGCCTTTCTGGATCTTTCTGAGCTTGCTTTATACTACCCAATTTTTATTTTTTGCACTTT	A	G	0.36	[illegible]	[illegible]	[illegible]	[illegible]	[illegible]	[illegible]	[illegible]	[illegible]	[illegible]	[illegible]	[illegible]	[illegible]	[illegible]	[illegible]	[illegible]			
rs7064676	X	147.80	TTGGGTCTTTTGTCTCCTTTTTAAAAATTATTTGCTTACTTGTTTTGCTGGAGCAC[A/G]TGCTTTAATAGCTTCCTAAATAAAGGTTTATGACAAATTATATTAGTCAGCTTTTTAGT	A	G	0.23	[illegible]	[illegible]	[illegible]	[illegible]	[illegible]	[illegible]	[illegible]	[illegible]	[illegible]	[illegible]	[illegible]	[illegible]	[illegible]	[illegible]	[illegible]			
rs611711	X	148.26	CCACTCTCCCTCATCCTGAAAACATCGCTAACAGAGATTTGCTTCAGGCATTCAGCTT[A/G]ACTGGTTCTCTTGTTCTTTCCTAGAAAACAAACAAACAAACAAAAAACATGTTTC	A	G	0.57	[illegible]	[illegible]	[illegible]	[illegible]	[illegible]	[illegible]	[illegible]	[illegible]	[illegible]	[illegible]	[illegible]	[illegible]	[illegible]	[illegible]	[illegible]			
rs480199	X	148.31	GATGTCGCTGTTTCCACCCTGGTCTGCCTTGCCTTTCCCTGTCTTTGGAAGCCACA[A/G]ATGAAAGGATATCAGCTAGCTTGCCTTGAGAATTATTGTCCCTCACCTATTCTCTC	A	G	0.55	[illegible]	[illegible]	[illegible]	[illegible]	[illegible]	[illegible]	[illegible]	[illegible]	[illegible]	[illegible]	[illegible]	[illegible]	[illegible]	[illegible]	[illegible]			
rs222398	X	148.52	TTAGGATTCACAGGCCTCAGGCACAACAAGAATATTGTTGCTAAAATCAGTGT[C/G]CCTGCTGAATAAAGCTGGGCTTCCCTCCTCTCCGGGAACAGCCACTTACCTCTCCC	C	G	0.86	[illegible]	[illegible]	[illegible]	[illegible]	[illegible]	[illegible]	[illegible]	[illegible]	[illegible]	[illegible]	[illegible]	[illegible]	[illegible]	[illegible]	[illegible]			
rs644345	X	148.98	CATCAGCTGCTCCTCAGATCAGAGCCTTGCAGGTCAGGCCAGAGGGGAGACTCAG[A/G]AAGTGTTTTTGGTGTTTGTTCCAAATGGGGGCTCATTGTGGGCCCTGTGTCACTG	A	G	0.79	[illegible]	[illegible]	[illegible]	[illegible]	[illegible]	[illegible]	[illegible]	[illegible]	[illegible]	[illegible]	[illegible]	[illegible]	[illegible]	[illegible]	[illegible]			
rs1202872	X	148.99	CGCCCTCTCACTACTTCTTTGGTCTCAGACTAAATTTTACAGGGCTTCCCTGCTTC[C/G]CCTCAGTTTTCACTCTTAGGTTACCTCGGAACGGTAAGTATTTGACTTGGCAAGGTT	C	G	0.60	[illegible]	[illegible]	[illegible]	[illegible]	[illegible]	[illegible]	[illegible]	[illegible]	[illegible]	[illegible]	[illegible]	[illegible]	[illegible]	[illegible]	[illegible]			
rs760109	X	149.19	ATATGAACAGAGGTCATACGTGGTTGTCAGACTCTCATTCGCTCACTCGCTCCTCA[A/G]TGTGTGGTCCTGGAAAAGACACTTTATCTCTTTTGACCTTAATTCCTACATGAGTG	A	G	0.79	[illegible]	[illegible]	[illegible]	[illegible]	[illegible]	[illegible]	[illegible]	[illegible]	[illegible]	[illegible]	[illegible]	[illegible]	[illegible]	[illegible]	[illegible]			
rs1882719	X	149.24	TTGAAGAGGTAGGTCAGGCAGCAATCAAGGGCTGTGGGCACCTAAGACCAGCAGGA[A/G]GGTGCTATGTGCCTAGGGTACTGCAAAAGACAATAAAGAAAGGAAGGTGGGAC	A	G	0.38	[illegible]	[illegible]	[illegible]	[illegible]	[illegible]	[illegible]	[illegible]	[illegible]	[illegible]	[illegible]	[illegible]	[illegible]	[illegible]	[illegible]	[illegible]			
rs941400	X	149.62	TGACAAGCTGAACTGACTGCTCTTTTCCATTTCCTGTGCTCCAAGAAGAGGTTTTCA[A/G]CCAGGTTTGGGGGCAGAAAGAAGCTATGAAATAAGACTTATGTCAGGGAGTAGT	A	G	0.64	[illegible]	[illegible]	[illegible]	[illegible]	[illegible]	[illegible]	[illegible]	[illegible]	[illegible]	[illegible]	[illegible]	[illegible]	[illegible]	[illegible]	[illegible]			
rs2032438	X	149.80	TCTGCCAATTCTGTAGCAGCTGTAGCACCAAGGGAGGCAGACATCCTAACACTCACATAATGCTAGCCACTCA[A/G]CAACACCTTCTGAGTGGGTGCTAAGTTACCTACAGGA	A	G	0.31	[illegible]	[illegible]	[illegible]	[illegible]	[illegible]	[illegible]	[illegible]	[illegible]	[illegible]	[illegible]	[illegible]	[illegible]	[illegible]	[illegible]	[illegible]			
rs980934	XY	0.54	CTCATCCAGATGCCCAACGTGCTCCTGCGCTCCCCACAGCCGGAGGGAGGAG[A/G]GGGTGGTTTGTAGCAGAGGACAGGGCTGGAGAGGGGAGGAGGGAGATAGAAACGC	A	G	0.12	[illegible]	[illegible]	[illegible]	[illegible]	[illegible]	[illegible]	[illegible]	[illegible]	[illegible]	[illegible]	[illegible]	[illegible]	[illegible]	[illegible]	[illegible]			
rs2027987	XY	0.64	ATGTGGCAGCAAAGAGATTTGAGCTGTACTTTTCAAAACAGCTGGCTGGCATACTG[A/C]AAGAGAAGGCCGGTGTTGTGTGGGACTGATTGCAAAGTGCATTTTCTGATAATTA	A	C	0.45	[illegible]	[illegible]	[illegible]	[illegible]	[illegible]	[illegible]	[illegible]	[illegible]	[illegible]	[illegible]	[illegible]	[illegible]	[illegible]	[illegible]	[illegible]			
rs2045059	XY	1.55	TGTCTTAATCCATTTGCTGTTGCTACAACAGAGTAACACCGTCTGGGTAATTCACA[A/G]AAGAAGTTTATTTTGTAAAGTCCTGGAGGCTGGCAAGTCCAAAAGGCATGGTGCCT	A	G	0.33	[illegible]	[illegible]	[illegible]	[illegible]	[illegible]	[illegible]	[illegible]	[illegible]	[illegible]	[illegible]	[illegible]	[illegible]	[illegible]	[illegible]	[illegible]			
rs1044307	XY	2.00	ATGAAGACAGAGCCGGGACAGTTCACATTTCCACGTGAGTGAGGAAAAGC[A/G]AAGGATTGTTAGAGACGTGAAGAGAACGCTGGCAATGCTACAGAATGGAG	A	G	0.86	[illegible]	[illegible]	[illegible]	[illegible]	[illegible]	[illegible]	[illegible]	[illegible]	[illegible]	[illegible]	[illegible]	[illegible]	[illegible]	[illegible]	[illegible]			
rs311094	XY	2.25	CTTCCCTTTGTGTTGAAAACGTGGGTGTTCCTTCACACCAACACTGGAAATAAAT[A/G]AATACCTATTGATCTGTGCATCTCTTACGCGTTTCCAATTCTGTTGGCTTATGCTGT	A	G	0.60	[illegible]	[illegible]	[illegible]	[illegible]	[illegible]	[illegible]	[illegible]	[illegible]	[illegible]	[illegible]	[illegible]	[illegible]	[illegible]	[illegible]	[illegible]			
rs311093	XY	2.25	TGGGAGTCATTTAGTGGTTAGGGAGAACAGTTGAAACTGTGGAGTTCACGTAGC[A/G]TAAACTGGCGAGGCTGTCTCTGCTGCTGTCATTAAAAGTTACGCTGCTGGCTTGGC	A	G	0.50	[illegible]	[illegible]	[illegible]	[illegible]	[illegible]	[illegible]	[illegible]	[illegible]	[illegible]	[illegible]	[illegible]	[illegible]	[illegible]	[illegible]	[illegible]			
rs311097	XY	2.26	GTATTGAAGCTCAGTAAACACTTGCAGAATAAATCACAGAAAAGCCTCTCTCCTTAA[C/G]AAGTTAAGAGATTGGCTCTGATACCAACAACTACCATACTATTCCAAATACTATTA	C	G	0.38	[illegible]	[illegible]	[illegible]	[illegible]	[illegible]	[illegible]	[illegible]	[illegible]	[illegible]	[illegible]	[illegible]	[illegible]	[illegible]	[illegible]	[illegible]			
rs311100	XY	2.26	TTGTTTTTATACGGGTGTTGGGTCCTTTTCTCATTGATCTATAGGGACTGTTTAC[A/G]CCTCAGTGAAGTAGGTGGCAGATATTCTTTTTCACTTAATCCTTAGTCTGGTGAATCT	A	G	0.33	[illegible]	[illegible]	[illegible]	[illegible]	[illegible]	[illegible]	[illegible]	[illegible]	[illegible]	[illegible]	[illegible]	[illegible]	[illegible]	[illegible]	[illegible]			
rs311103	XY	2.26	TTGAGGCAAGAGTAGACAAGCTTTTCTGCAGAAGGTCAGATAGTAAAGGCATTTAT[C/G]TTTGCAAACCATATGGATTCTGCTGCAATATGTAACCAATGTCTGTGCGCCAAGTG	C	G	0.58	[illegible]	[illegible]	[illegible]	[illegible]	[illegible]	[illegible]	[illegible]	[illegible]	[illegible]	[illegible]	[illegible]	[illegible]	[illegible]	[illegible]	[illegible]			
rs312233	XY	2.27	TGCACACACATTTTAAGTCATGTTGATACGGACAGGAGGCCTGAAGATACTAGATA[C/G]AAGAGGGCAGTTCCCCAGCAAAGGCCCCGCCCCTCAAGCCTGGAAACCTGTAGTC	C	G	0.43	[illegible]	[illegible]	[illegible]	[illegible]	[illegible]	[illegible]	[illegible]	[illegible]	[illegible]	[illegible]	[illegible]	[illegible]	[illegible]	[illegible]	[illegible]			
rs312231	XY	2.27	AATTTTGGAGACGCACAATTCAACCCTTACAGAGGTAACTCATTCATTGGAATACA[A/G]ATGTGTTTGTCCACTGTGGCTGCTGCCATAGCAAAGTCCCACAGACCAGGCTGTTT	A	G	0.62	[illegible]	[illegible]	[illegible]	[illegible]	[illegible]	[illegible]	[illegible]	[illegible]	[illegible]	[illegible]	[illegible]	[illegible]	[illegible]	[illegible]	[illegible]			
rs2535444	XY	2.28	GGGTGGGCAGATGAGACTCTTGGGTGCCTGGCATTACTGCAACAGAACTCAATGC[A/G]TAGTAAGTGCTGACTCAATGTGCTCCTGGCTGCATCCTCTACTTCCTGATAAATTC	A	G	0.60	[illegible]	[illegible]	[illegible]	[illegible]	[illegible]	[illegible]	[illegible]	[illegible]	[illegible]	[illegible]	[illegible]	[illegible]	[illegible]	[illegible]	[illegible]			
rs2857323	XY	2.28	CCTCCTGCAAGCCTAGGGTCGCCCAGAGCACTAAAACTAACCTGACCATGAGCTAC[A/G]TGGGAGATGCAACCACCACCTCCACCTAGTCCCAGAGCATACTCATCACCCAAA	A	G	0.52	[illegible]	[illegible]	[illegible]	[illegible]	[illegible]	[illegible]	[illegible]	[illegible]	[illegible]	[illegible]	[illegible]	[illegible]	[illegible]	[illegible]	[illegible]			
rs311150	XY	2.28	GCTGAAAATATGTGACCTGGGCCCTGCAGAAAGTCCTGCTTGAGGCAGATAGTCT[A/G]ATTGAGATTCAGGTGTATTCAGACCGCGGTGGGTGGGAGAAAGGGAAAGATTTGC	A	G	0.55	[illegible]	[illegible]	[illegible]	[illegible]	[illegible]	[illegible]	[illegible]	[illegible]	[illegible]	[illegible]	[illegible]	[illegible]	[illegible]	[illegible]	[illegible]			
rs311121	XY	2.28	GGATCTCACTTTGCAGGACAGAGACTTGGTAATGGCTTCCCAGAGTTGTTACAATG[A/C]AATCCCAAAGACTGGGCAGCTTAAACAACAACCTTGATTCTCCCACAGTCCTGGA	A	C	0.36	[illegible]	[illegible]	[illegible]	[illegible]	[illegible]	[illegible]	[illegible]	[illegible]	[illegible]	[illegible]	[illegible]	[illegible]	[illegible]	[illegible]	[illegible]			
rs311122	XY	2.28	TGTCCTGAGGGATCCGTGTGCATGCCACTCTGGGAAGACGGGTTTATAACACACC[A/G]TCAACCAGTGCCTCATTGCATGGTCTGCGGTATTTTCCTCGAGTTTTCCTTGCACC	A	G	0.33	[illegible]	[illegible]	[illegible]	[illegible]	[illegible]	[illegible]	[illegible]	[illegible]	[illegible]	[illegible]	[illegible]	[illegible]	[illegible]	[illegible]	[illegible]			
rs311123	XY	2.28	CTCTATTAGAGCAGACATTGGTAATGAGAATTCAGGCCTCAGTGTCTGTCTGTAAC[A/C]CAACAGACGGTGTCTGCAGAGATCGAAGTATTTTGTCGTCGAAGAGGAAGGAATG	A	C	0.36	[illegible]	[illegible]	[illegible]	[illegible]	[illegible]	[illegible]	[illegible]	[illegible]	[illegible]	[illegible]	[illegible]	[illegible]	[illegible]	[illegible]	[illegible]			
rs2535446	XY	2.28	GAGGAGAGAGGACAGGGGGCCCTGCCTGGGAGATAGATTTGCTGCCTGGCATGC[A/G]GGACTGGAGACTGTCTCAGTGCCCAGGCCGTCTGATGCTGGCCGGAGTCCGCCT	A	G	0.26	[illegible]	[illegible]	[illegible]	[illegible]	[illegible]	[illegible]	[illegible]	[illegible]	[illegible]	[illegible]	[illegible]	[illegible]	[illegible]	[illegible]	[illegible]			
rs2630498	XY	2.28	TTACCAGCGATCACTACAGGCTCGCCTTTAAGAACAACCCAGCAAATCTTTGCTCC[A/C]TCCAGCTCTCTTCTTATTTTTTCTTTATTGTTGTTTTCTTTTTGTTTGTTTTGTTTTTA	A	C	0.62	[illegible]	[illegible]	[illegible]	[illegible]	[illegible]	[illegible]	[illegible]	[illegible]	[illegible]	[illegible]	[illegible]	[illegible]	[illegible]	[illegible]	[illegible]			
rs306892	XY	153.42	GAAAGATTTAATATTGTTAAGATAGAAATACTCTCCAAAGTGATCTACAGAAAACAT[A/T]ATCCCTATCAAAGTCCCAGCCAGATTTTTTGCAAAAATTCATGAATGAACCCTAA	A	T	0.76	[illegible]	[illegible]	[illegible]	[illegible]	[illegible]	[illegible]	[illegible]	[illegible]	[illegible]	[illegible]	[illegible]	[illegible]	[illegible]	[illegible]	[illegible]			
rs700449	XY	153.44	TCTGTCTTTTACCATCAGAGGGATGACTATTACCTGGTATGGTCAAAAGGATTGCC[A/G]GGTTAGTTTGGGAATGACAATGTGTGAGTTAGAATTACCTTCTTCTTTTTCAGGAG	A	G	0.60	[illegible]	[illegible]	[illegible]	[illegible]	[illegible]	[illegible]	[illegible]	[illegible]	[illegible]	[illegible]	[illegible]	[illegible]	[illegible]	[illegible]	[illegible]			
rs700447	XY	153.44	CTAGTTTAGGCCCATATCCTTATTTTCACTCTCAAAGATAAGATCATGACACTACC[A/C]GTGTTGCTGCTGTTAAAGGCCAGGATGGACAAGATAAGGAAAGGCATTTTGGGAAT	A	C	0.21	[illegible]	[illegible]	[illegible]	[illegible]	[illegible]	[illegible]	[illegible]	[illegible]	[illegible]	[illegible]	[illegible]	[illegible]	[illegible]	[illegible]	[illegible]			
rs306881	XY	153.46	ATTTTGGAAACACATATGATAGAAAATGCAGTTCATTTTTTCCTTAATTTACTTTCA[A/G]TCAGCACTCCTGGTAATCCCTAGGGAAAATTTTCTTGTATCTACTTATTCTTCCACCC	A	G	0.81	[illegible]	[illegible]	[illegible]	[illegible]	[illegible]	[illegible]	[illegible]	[illegible]	[illegible]	[illegible]	[illegible]	[illegible]	[illegible]	[illegible]	[illegible]			
rs781717	XY	153.46	CAGCAAAAGAGTAAGTGTTATCCTGAGTGCTGTGAACCACTCCAGCAAATTACTCA[A/C]ACCCAGAGAGGGTGTCATGAGAACTCCTGATTTATAGCTGATCAGTCATGGGTCA	A	C	0.79	[illegible]	[illegible]	[illegible]	[illegible]	[illegible]	[illegible]	[illegible]	[illegible]	[illegible]	[illegible]	[illegible]	[illegible]	[illegible]	[illegible]	[illegible]			
rs3093457	XY	153.66	TTAGTTTGTAATCCTTGAGGTCAGGGCTGCAAATTTTGCCATCTCTAAGAGATAC[A/C]CAAGGCCAGGACACCAGAGGAGGTAAAGCTAGGGTGGCGCTCTCAAATGCCACAC	A	C	0.57	[illegible]	[illegible]	[illegible]	[illegible]	[illegible]	[illegible]	[illegible]	[illegible]	[illegible]	[illegible]	[illegible]	[illegible]	[illegible]	[illegible]	[illegible]			
rs3093505	XY	153.67	CACAGAAGGCACACTCACACTCACAGACACAGCTGCGTTTGCCCACAAATACACAT[A/G]TGCACACACAGGCAAACATGTCTACAGGAACACTTTCACACTCATCACACTTGC	A	G	0.67	[illegible]	[illegible]	[illegible]	[illegible]	[illegible]	[illegible]	[illegible]	[illegible]	[illegible]	[illegible]	[illegible]	[illegible]	[illegible]	[illegible]	[illegible]			

a. Physical position in Megabases (Mb) based on HG Build 34.
b. Allele frequencies determined in 22 African American (AFA), 92 Swedish (SWD), 162 Western European American (WEURA), 41 Central European American (CEURA), 27 Eastern European American (EEURA), 13 East Asian (EAS), 48 Mayan Amerindian (AMI), 48 South Asian (SAS), 13 Finnish (FIN), 71 Italian (ITN) and 82 Spanish (SPN) subjects.
c. The informativeness for assignment (In) for distinction between two different populations or subpopulations are provided.
d. The 2657 SNPs used for initial STRUCTURE studies are indicated.
e. The 400 selected SNPs for European population structure information (used in Figure 1C and Figure 4) are indicated. These SNPs were chosen based on maximizing In using subsets WEURA and SPN subjects.
f. The 768 selected SNPs for European population structure information (used for Table 3 analyses) are indicated. These SNPs were chosen based on maximizing In using subsets WEURA and SPN subjects.
g. Indicates the 19 SNPs from the 768 set that were excluded based on the quality of typing results.
